# Patient-derived brain organoids reveal divergent neuronal activity across subpopulations of autism spectrum disorder

**DOI:** 10.1038/s41398-026-03890-1

**Published:** 2026-02-25

**Authors:** Nisim Perets, Liya Kerem, Nir Waiskopf, Noa Horesh, Itay Goldman, Jasmine Avichzer, Doron Bril, William Tobelaim, Milcah Barashi, Liat David, Ariel Tenenbaum

**Affiliations:** 1 Itay&Beyond LTD., Jerusalem, Israel; 2 Department of Pediatrics, Faculty of Medicine, Hadassah Medical Center, and The Hebrew University of Jerusalem, Jerusalem, Israel

**Keywords:** Autism spectrum disorders, Neuroscience

## Abstract

Patient-derived brain organoids have emerged as a powerful model for investigating the mechanisms underlying neurological and psychiatric disorders. They provide novel insights into autism spectrum disorder (ASD), a heterogeneous neurodevelopmental condition whose underlying mechanisms remain poorly understood. Recent advancements in generating electrophysiological functional 3D brain organoids enable the study of molecular and network-level neuronal activity. Here, we aimed to characterize the neurophysiological underpinnings of ASD by comparing electrophysiological properties of brain organoids derived from eleven individuals diagnosed with autism spectrum disorder, 10 with monogenic syndromic ASD across five genetic subtypes, and 1 with idiopathic ASD, to organoids derived from 4 neurotypical control individuals. We identified distinct differences in baseline activity (resting state) and evoked responses (synaptic plasticity and network dynamics) across ASD subgroups. To comprehensively assess these differences, we applied dimensionality reduction (principal component analysis, PCA) to integrate multiple electrophysiological features into a unified framework. Our findings reveal subtype-specific neurophysiological alterations in ASD brain organoids, offering mechanistic insights into ASD heterogeneity and potential applications for early diagnostics, drug screening, and therapeutic development.

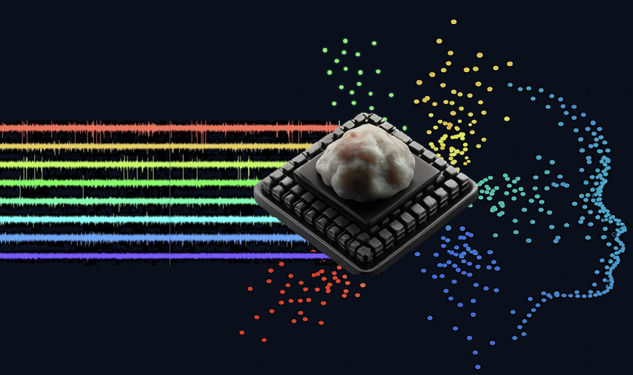

## Introduction

The prevalence of neurological and neuropsychiatric disorders worldwide is striking. According to epidemiological studies from 2021, more than 3 billion people worldwide live with one or more neurological or neuropsychiatric conditions, affecting approximately 43.1% (40.5-45.9%) of the global population [1]. Autism Spectrum Disorders (ASD) are neurodevelopmental conditions characterized by persistent deficits in social communication and interaction, along with restricted repetitive behaviors, with symptoms varying widely in severity [2]. Worldwide, ASD affects approximately 75 million individuals, ranking 12th among all neurological and psychiatric conditions and 5th among neuropsychiatric disorders in terms of prevalence [3].

ASD is driven by a complex interplay of genetic, biological, and environmental factors that disrupt early brain development [4]. Factors such as prenatal exposure to infections, maternal immune activation, and metabolic or environmental insults may also contribute, usually leading to idiopathic ASD. [5, 6] Yet, in recent years, hundreds of genes have been implicated in ASD risk, classified as syndromic ASD.

Syndromic ASD, stemming from single-gene mutations, offers valuable insights into the disorder’s biological basis. Notable examples include SHANK3 haploinsufficiency syndrome, PPP2R5D-related disorder (Jordan’s syndrome), SCN2A-related disorder, GRIN2B-related neurodevelopmental syndrome, and STXBP1-associated disorders, all of which impact synaptic function, neural excitability, and circuit development [7, 8]. The genetic mutations driving these syndromes disrupt essential molecular pathways responsible for neuronal network development and function. **SHANK3 haploinsufficiency**, seen in Phelan-McDermid syndrome, leads to impaired synaptic scaffolding at excitatory synapses, disrupting glutamatergic signaling and synaptic plasticity [9, 10]. **PPP2R5D mutations** (Jordan’s syndrome) alter the regulatory subunit of protein phosphatase 2 A, impacting neuronal development and signal transduction, particularly pathways involved in brain growth and cytoskeletal regulation [11]. **GRIN2B-related syndrome**, caused by mutations in the gene encoding the GluN2B subunit of the NMDA receptor, results in defective excitatory neurotransmission and altered calcium signaling, impairing learning-related plasticity [12]. Mutations in **SCN2A**, which encodes a voltage-gated sodium channel, affect neuronal excitability and are strongly associated with both ASD and epilepsy due to disrupted action potential generation [13]. Lastly, **STXBP1 mutations** impair presynaptic vesicle release by disrupting the SNARE complex, leading to widespread deficits in neurotransmitter release and cortical network function [14, 15]. Together, these mutations converge on different pathways affecting synaptic transmission, plasticity, and network connectivity, providing critical insights into the molecular underpinnings of ASD. Though monogenic autism captures about 10% of the overall ASD population, it can hold an important key to unlock impaired mechanisms of idiopathic populations [16].

However, despite the growing ability to identify new genetic causes of syndromic autism, the extrapolation from genes to neuronal activity patterns is still challenging, especially in human patients. This is compounded by the limited availability of viable human patient-derived brain tissue with preserved functional integrity. Furthermore, traditional animal models often fail to fully capture human-specific features of ASD, limiting the translational relevance of their findings [17].

Brain organoids - three-dimensional, self-organizing structures derived from induced pluripotent stem cells (iPSCs) [18], recapitulate key features of early brain development, including the emergence of distinct brain regions and cell types. Crucially, they retain the patient’s specific genetic background and molecular fingerprint [19–21], enabling the modelling of disease-relevant phenotypes in a human context. As such, patient-derived brain organoids have emerged as a cutting-edge in vitro system, offering valuable molecular insights into the mechanisms underlying ASD and other neurological and neuropsychiatric disorders [22–24].

Notably, they can also replicate key aspects of neuronal diversity and network connectivity, providing a valuable platform to study synaptic plasticity and disrupted neural circuitry that can unlock new mechanisms underlying neuropsychiatric disorders [25].

Here, we analyzed 18 electrophysiological features of patient-derived brain organoids and used principal component analysis (PCA) to reduce them into a 3D space for comparison. Organoids from the same individual showed minimal intra-subject variability, and control patients clustered tightly, reflecting low inter-subject variability. In contrast, syndromic and idiopathic ASD samples were widely dispersed, highlighting significant heterogeneity.

## Materials and methods

### Participant recruitment and sample collection

The conditions studied and the numbers of patients and organoids are summarized in Table 1. The differences in the number of brain organoids among the various groups result primarily from challenges in forming them across different lines while aiming to maintain minimal protocol interventions. A clinical assessment of the patients and their phenotype is summarized in Table S1.

**Table 1:** Study conditions and sample numbers.

Condition	Number of Patients	Number of Organoids
Control	4	184
Idiopathic ASD	1	87
SHANK3	1	4
SCN2A	2	51
STXBP1	1	25
PPP2R5D	4	24
GRIN2B	2	37

Summary of the conditions included in the study and the numbers of patients and derived organoids analyzed for each condition. The idiopathic ASD and one of the control lines are a father and son. The rest of the patients have no familial relationship.

### Urine collection and iPSC generation

iPSC lines were reprogrammed from urine-derived epithelial cells or acquired from the Simons Foundation Autism Research Initiative (SFARI). Urine samples were collected from healthy volunteers and patients with ASD following approval by the Institutional Review Board (IRB) and the ethics committee of Hadassah Medical Organization (Research application: HMO-0021-22). Informed consents were obtained from all participants, and the study was conducted according to institutional ethics committee guidelines. Epithelial cells were precipitated from urine by centrifugation at 400 g for 10 min and later selected and expanded on plates coated with iMatrix-511 (T304, Takara-Clontech), and media containing DMEM/Ham’s F12 1:1, Lonza™ REGM™ Renal Epithelial Cell Growth Medium SingleQuots™ Kit (CC-4127), human serum (AC-001-1B, Access Cell Culture), and PSA as antibiotics [26, 27]. Subsequently, the urine-derived epithelial cells were reprogrammed using Epi5™ Episomal iPSC Reprogramming Kit (A15960, Thermo Fisher Scientific [28]), and kept in liquid nitrogen till organoid generation.

iPSC lines acquired from SFARI: 15653-x1, 16020-x1, 15655-x1, 16051-x1, 15413-x1, 15265-x1, 15473-x1, 15936-x1, 15912-x1.

### iPSC immunostaining

Immunostaining was performed to analyze the expression of key pluripotency markers in induced pluripotent stem cells (iPSCs). The cells were cultured under standard conditions until they reached 40-50% confluency. Following this, iPSCs were fixed with 4% paraformaldehyde for 20 min at room temperature (R.T.). Permeabilization was achieved using Triton X-100 for 45 min at R.T. After blocking with bovine serum albumin for 1 h, the cells were incubated overnight at 4 °C with primary antibodies: Mouse anti-Sox2 (SC-365823) and Anti-Oct-3/4 Antibody (SC-5279) from Santa Cruz Biotechnology, and NANOG Monoclonal Antibody (eBioMLC-51, cat. 14-5761-80, Invitrogen) at concentrations recommended by the manufacturer. The expression of SOX2 and OCT4 was analyzed as markers of pluripotency (data not shown), as both are critical for maintaining stem cell self-renewal and undifferentiated states. NANOG staining was included to further confirm pluripotency, as it is essential for the maintenance of iPSC self-renewal and inhibiting differentiation. Following incubation with primary antibodies, the cells were washed with PBS and incubated with secondary antibodies conjugated to fluorophores from Invitrogen: Goat anti-Mouse IgG (H + L) Alexa Fluor 488 (Cat. A-11001), and Goat anti-Rat IgG (H + L) Alexa Fluor 568 (Cat. A-11077) at a 1:700 dilution for 1 h at R.T. Finally, nuclei were counterstained with Hoechst 33342 (J62134.100, Thermo Fisher Scientific) for 10 min. Images were captured using a fluorescence microscope, and the expression of the markers was analyzed to assess the pluripotency of the iPSCs.

### Brain organoids protocol and characterization

For the generation of brain organoids, we started with an 80%-90% confluency of human pluripotent stem cells (hPSCs) cultured in feeder-free conditions.

Cells were washed once with Dulbecco’s phosphate-buffered saline, and dissociation was conducted using 1 mL of Accutase solution (A6964, Merck). Following detachment, the cells were centrifuged and resuspended using mTeSR Plus media (100-0276, STEMCELL Technologies) containing Y-27632 dihydrochloride (1254/10, Tocris) and FGF2 (233-FB-025/CF, R&D Systems). Cells were counted using Trypan Blue and were seeded in a non-treated U-shape 96-well plate. Cells were allowed to rest and form cell-to-cell connections for 24 h before replacing the media. The day after the seeding, the media was replaced and the cells were treated with Neurobasal Media (Gibco) containing GlutaMAX (Gibco), Penicillin-Streptomycin, N-2 (Gibco), B-27 (Gibco), and supplemented with dual SMAD inhibitors and vitamin A. After the formation of stable 3D spheres, each sphere is transferred into an ultra-low attachment flat 24-well plate for the continuation of neuronal differentiation and maturation with the same Neurobasal media supplemented with fibroblast growth factor 2 (basic), transforming growth factor beta 1, and gamma-secretase inhibitor.

Cryosectioning and immunostaining were performed to analyze the expression of specific markers in organoid samples. Organoids were harvested and fixed with 4% paraformaldehyde for 1 h at room temperature (R.T.). Following fixation, the organoids were embedded in an optimal cutting temperature (OCT) compound and frozen at -80 °C. Sections of 10 µm thickness were obtained using a cryostat and mounted on glass slides. The sections were permeabilized with 0.2% Triton X-100 for 30 min at R.T. and blocked with 5% bovine serum albumin (BSA) for 1 h at R.T. After blocking, the sections were incubated overnight at 4 °C with primary antibodies: Mouse anti-Sox2 (SC-365823, Santa Cruz Biotechnology), Rabbit anti-Tuj1 (GTX129913-25, GeneTex), Guinea pig anti-DCX (AB2253, Sigma-Aldrich), and Mouse anti-SATB2 (ab51502, Abcam) at concentrations recommended by the manufacturer. SOX2 staining was used as a marker for iPSCs, as it is a critical transcription factor essential for maintaining pluripotency and self-renewal in stem cells. Tuj1, a marker for beta-III tubulin, was used to identify differentiated neurons within the organoids. DCX, a microtubule-associated protein expressed in migrating and immature neurons, marked early neurogenic populations, whereas SATB2, a nuclear transcription factor expressed in upper-layer cortical neurons, identified the development of cortical projection neuron identity.

Following primary antibody incubation, the sections were washed with PBS and incubated with secondary antibodies conjugated to fluorophores, such as Goat anti-Mouse IgG (H + L) Alexa Fluor 488, Goat anti-Mouse IgG (H + L) Alexa Fluor 568, and Goat anti-Guinea Pig IgG (H + L) Alexa Fluor™ 647 for 2 h at R.T. Finally, the sections were counterstained with Hoechst for 10 min to visualize the nuclei. Images were captured using a fluorescence microscope, and the expression of the markers was analyzed to assess the pluripotency of the iPSC-derived organoids and the extent of neuronal differentiation.

### Singel cell analysis

Single-cell RNA-seq libraries were prepared at the Crown Genomics Institute of the Nancy and Stephen Grand Israel National Center for Personalized Medicine (G-INCPM), Weizmann Institute of Science, using the 10x Genomics Chromium platform with on-chip multiplexing. Brain organoids were dissociated into a single-cell suspension using Worthington Papain Dissociation System and the cells were diluted in PBS + 0.04% BSA to ~1000 cells/µL and processed immediately.

The 10x Genomics On-Chip Multiplexing technology (Chromium GEM-X Universal 3’ Gene Expression v4 4-plex kit) was used to pool 3 samples and generate single-cell sequencing libraries. 4000 cells were targeted per sample, loaded into the Chromium 3’ OCM chip and processed using the GEM-X Universal 3’ Reagent v4 4-plex according to the manufacturer’s protocol. cDNA and gene expression libraries, along with sample index libraries, were prepared according to the manufacturer’s protocol.

Libraries were quantified by qPCR (NEBNext, New England Biolabs) and Qubit, and size distribution was verified using an Agilent TapeStation. Sequencing was performed on an Illumina NovaSeq X 1.5B 100 cycles.

CellRanger pipeline (v9, 10x genomics), cellranger multi, with default parameters was used for demultiplexing, alignment (GRCh38 reference genome, 2024-A version, downloaded from 10X website), filtering, barcode counting, and UMI counting. scDblFinder with default parameters was used to detect and remove potential doublets. The Seurat R package (v5.1.0) was used for downstream analysis and visualization. Gene-cell matrices after doublets removal were filtered to remove cells with more than 4MAD above the median of (1) percentage of reads mapped to mitochondrial genes, (2) number of genes and (3) number of UMIs. In addition, genes detected in fewer than 10 cells were excluded from the analysis. After implementing these quality control measures for the three samples, a total of 5501, 6497, and 7141 cells were retained for further analysis.

The expression data was normalized using Seurat’s NormalizeData function, which normalizes the feature expression measurements for each cell by the total expression, multiplies this by a scale factor (10,000), and then log-transforms the results. The top 2000 highly variable genes were identified using Seurat’s FindVariableFeatures function with the ‘vst’ method. Potential sources of unspecific variation in the data were removed by regressing out the UMI count and percentage mitochondria using linear models and scaling and centering the residuals as implemented in the function “ScaleData” of the Seurat package. Principal component analysis (PCA) was performed. 25 PCs were used for clustering and data reduction. Cell clusters were generated using Seurat’s unsupervised graph-based clustering functions “FindNeighbors” and “FindClusters” (resolution = 0.5). UMAP was generated using the RunUMAP on the projected principal component (PC) space. Using manual inspection, several clusters representing less than 5% of the data were removed. Cluster marker genes were calculated using Seurat’s “FindAllMarkers” with only.pos = TRUE, min.pct = 0.5 and logfc.threshold = 0.5. For differentially expressed genes, “FindAllMarkers” was run with min.pct = 0.5 and logfc.threshold = 0.5.

Seurat’s functions FeaturePlot and DimPlot were used for visualization. Seurat’s DotPlot and VlnPlot functions were used to visualize gene expression for each cluster. Heatmaps were produced with Seurat’s function DoHeatmap or the R package pheatmap. Marker genes for each cluster were identified by performing differential expression between a distinct cell cluster and the cells of the other clusters with the non-parametric Wilcoxon rank sum test (Seurat’s FindAllMarkers function). Cell types were assigned manually based on the expression of classic marker genes.

### Electrophysiological recording and stimulation system

Brain organoids at approximately 60 days in vitro were transferred onto multielectrode arrays (MEAs; 60MEA200/30iR-Ti, Multichannel Systems) coated with 0.1% polyethyleneimine (PEI) approximately 96 h before recording to ensure stable adherence to the electrode surface while minimizing potential confounding effects of prolonged MEA culture.

The RHS Stim/Recording Controller, connected to the RHS2116 stim/amplifier chips (Intan Technologies, Santa Monica, CA), was used for electrophysiological measurements. Broadband electrophysiological signals were acquired at a rate of 30 kHz, with a 50 Hz notch filter enabled and Intan amplifier bandwidth set to 1.17 – 7.60 kHz. Spontaneous activity was recorded for 5 min to determine baseline activity. Then, three cycles of electrical stimulation were administered, each lasting 20 seconds and comprising 20 biphasic pulses (100 µA, phase width 66.7 µs, duration 10 ms) at a frequency of 100 Hz, delivered simultaneously via four pre-specified bipolar electrode pairs positioned symmetrically around the center of the MEA. Electrode selection was geometric and identical across recordings, and was not based on firing rate, burst frequency, or response latency. Subsequently, data were collected for an additional five minutes to measure the stimulation effect.

Each organoid underwent two recording sessions, separated by 48 h, using the same acquisition settings and stimulation protocol. For analysis, we used the 5-minute pre-stimulation baseline and the 5-minute post-stimulation segment, selected as the minimal windows that yielded stable estimates of firing rate and bursting in preliminary recordings while limiting physiological drift in longer acquisitions.

### MEA data analysis

Raw electrophysiological data were extracted from Intan files using the Intan RHX software and processed in Python. The signals were organized into a structured dictionary for downstream analysis. Each detected event underwent spike sorting. Electrodes with fewer than or equal to 2 spikes per minute were considered inactive. For active electrodes, we quantified several metrics, including mean firing rate, mean spike amplitude, burst activity (single-channel bursts [SCB], network bursts [NB]), as well as network connectivity.

The mean firing rate was calculated in a two-step normalization process: first, we computed the firing rate for each active electrode and then averaged these values across all active electrodes on the MEA. This ensured that differences in firing rate were not driven by varying numbers of active electrodes across organoids.

Spike amplitude was defined as the minimum voltage of the spike waveform after baseline correction. While spike amplitude in MEA systems can be affected by electrode proximity, organoid positioning, and contact variability [29, 30], previous studies have used it as a population-level proxy for neuronal recruitment or network excitability under standardized conditions [31]. In our study, we applied consistent conditions across all organoids (e.g., media, recording duration, and MEA configuration) to minimize confounds. Accordingly, amplitude was used as a secondary, descriptive measure to complement core parameters such as firing rate, bursting behavior, and network connectivity.

A burst was defined as at least five spikes occurring within a 30-ms window. A network burst (NB) was defined as single-channel bursts co-occurring within a 500 ms window on the larger of (i) ≥5 electrodes or (ii) ≥30% of the active electrodes. This hybrid criterion was chosen to reduce false positives in low-coverage recordings and scaling with variable electrode activation. For stimulation experiments, electrodes were classified as showing short-term depression (STD), potentiation (STP), or no change based on mean firing rate. Specifically, the mean spike rate during the 5-minute window prior to stimulation was compared with the 5-minute window following stimulation. A significant decrease was categorized as STD, an increase as STP, and no significant difference as no change. Spike amplitude was analyzed descriptively but not used for classification, consistent with the all-or-none nature of extracellular action potentials.

Functional connectivity networks were then analyzed using graph-theoretical metrics implemented in the NetworkX Python package. Metrics included network size (number of active nodes), density, and clustering coefficient. Connectivity matrices were constructed using spike-time cross-correlation analysis: for each electrode pair, the distribution of time delays between spikes was computed within a 0–500 ms window, normalized by spike counts. Thresholded matrices derived from these cross-correlations were used to generate functional connectivity graphs for quantitative analysis, and statistical comparisons of these metrics were performed across groups and time points.

Dimensionality reduction was performed using Principal Component Analysis (PCA) implemented via the scikit-learn package. High-dimensional electrophysiological features were projected into a 3D space, enabling both visual and quantitative comparisons across experimental groups. Group differences in PCA space (PC1–PC3) were tested using permutational multivariate analysis of variance (PERMANOVA), with Holm-adjusted pairwise post-hoc tests, as normality assumptions were violated. In addition, radar plots were used to visualize group-level averages of standardized biological features, including firing rate, connectivity, and bursting dynamics, across all conditions.

### Data analysis

Data visualization and statistical analysis were performed using GraphPad Prism (GraphPad Software, San Diego, CA). We applied non-parametric approaches, including the Kruskal–Wallis H test, PERMANOVA, and appropriate post-hoc analyses and corrections (e.g., Dunn, Bonferroni). These methods were chosen because the data were not normally distributed, and group sizes were unequal. Non-parametric tests are robust to variability in sample size and variance and are widely used in organoid and iPSC studies under similar constraints [32, 33].

## Results

### From urine to patient-derived brain organoids on MEA (Fig. 1)

**Fig. 1:**
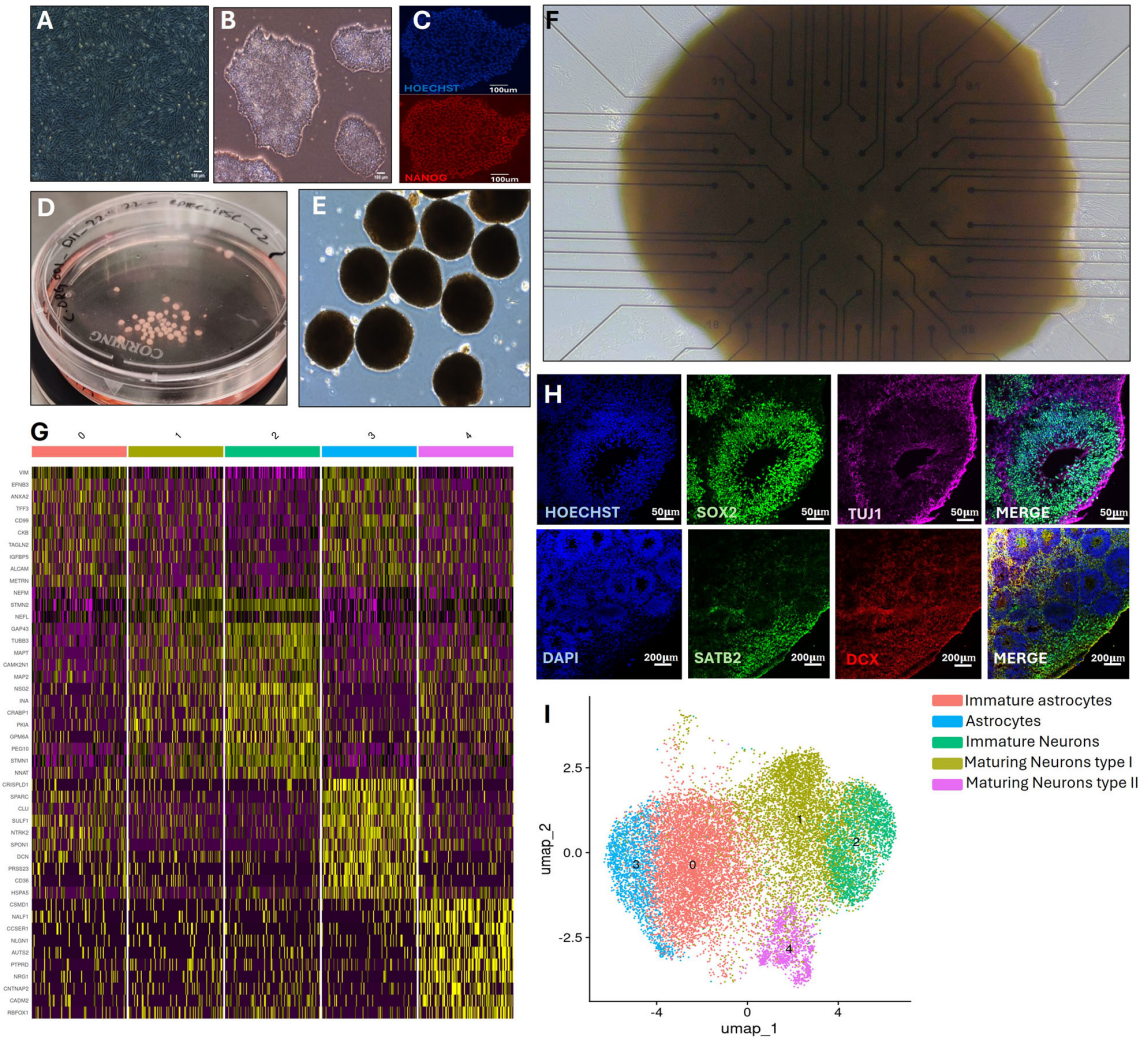
Urine epithelial Patient-derived brain organoids formation and characterization. **A** Patient-derived epithelial progenitor cells. **B** iPSCs colonies generation. **C** iPSC characterization for viability (Hoechst) and pluripotency (NANOG). **D, E**. Visualization of multiple brain organoids formed from iPSC colonies. **F** Multielectrode array recordings of patient-derived brain organoid set-up. **G-I** Characterization of Brain Organoids via scRNA-seq and Immunostaining. **G** Heatmap displaying the top 10 differentially expressed genes within each cluster identified by scRNA-seq. **H** Representative immunostaining images showing Hoechst or DAPI staining for cell nuclei, SOX2 for neural progenitor cells, Tuj1 and DCX for neuronal differentiation and migration, respectively, and SATB2 for mature cortical-like projection neurons, highlighting enlarged neural vesicles. **I** UMAP visualization of the scRNA-seq data, revealing five distinct clusters annotated as immature and mature astrocytes, as well as immature and maturing neuronal subpopulations.

Urine samples were collected and centrifuged to isolate the epithelial cell fraction (Fig. 1A). These cells were cultured and reprogrammed into induced pluripotent stem cell (iPSC) colonies (Fig. 1B), which were validated using standard markers for cell viability (Hoechst) and pluripotency (NANOG) (Fig. 1C). iPSCs were then differentiated into multiple patient-derived brain organoids (Fig. 1D, E) and characterized by single-cell RNA sequencing (scRNA-seq) and immunostaining (Fig. 1G–I).

ScRNA-seq performed on control sample at day 60 revealed five abundant clusters (>5%) in Uniform Manifold Approximation and Projection (UMAP). Clusters were annotated as immature and mature astrocytes, immature neurons and two sub populations of maturing neurons, one (type I) showing strong neurofilament/cytoskeletal maturation signatures (NEFM, NEFL, STMN2, GAP43, MAPT, MAP2) and the second (type II) enriched for synaptic adhesion/axon-guidance genes (CSMD1, NRXN1, NLGN1, CNTNAP2, CADM2, LSAMP) based on canonical markers and the top differentially expressed genes per cluster (Fig. 1G, I). Additional minor subpopulations (e.g., neural progenitor cells) were detected (<5%; not shown). The organoids displayed forebrain/cortical markers, with projection-neuron signatures (CUX1/2, FOXP1) co-expressed with pan-neuronal/synaptic markers (e.g., STMN2, MAP2, RBFOX3, SNAP25, DLG4, CAMK2A). Within the neuron-gated population, we also confirmed the presence of both excitatory-program and inhibitory-program neurons: excitatory identity was supported by DLG4 with GRIA1-3 and CAMK2A together with cortical projection signatures (CUX1/2, FOXP1), whereas inhibitory identity was supported by interneuron regulators (SOX6, ARX), subtype markers (SST, VIP), and inhibitory postsynaptic components (GPHN with GABA_A receptor subunits GABRA2/3, GABRB2/3, GABRG2). We also detected activity/excitability transcripts (EGR1, FOS, NTRK2, SCN1A, CACNA1C), consistent with synaptic maturation.

Figure 1H shows immunostaining for cell nuclei (Hoechst or DAPI), neural progenitor cells (SOX2), early neuronal differentiation and migration (Tuj1 and DCX, respectively), and mature cortical-like projection neurons (SATB2). Morphological organization of the organoids is shown at high magnification, highlighting neural vesicle structures. The functional platform, comprising patient-derived brain organoids cultured on a 64-electrode multi-electrode array (MEA), is illustrated in Fig. 1F.

### Characterization and comparison of basic (resting state) electrophysiological properties of patient-derived brain organoids from syndromic and idiopathic ASD patients, compared with controls (Fig. 2)

**Fig. 2:**
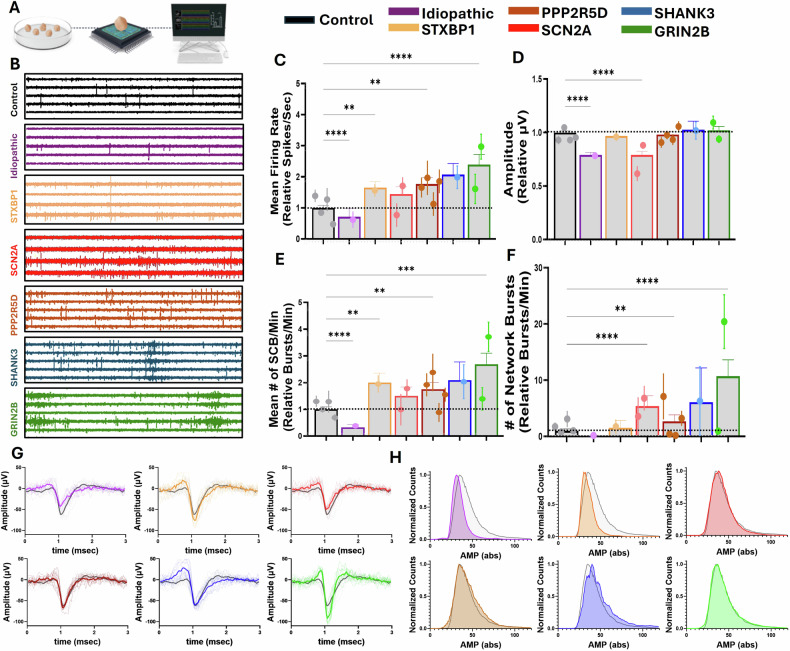
Characterization, comparison, and clustering of patient-derived brain organoids’ basic electrophysiological parameters (resting-state). **A** Experimental setup graphic scheme. **B** Representative recordings of electrophysiological activity of patient-derived brain organoids from different ASD subpopulations with their color coding. **C** Mean firing rate (MFR) of different sub-populations represented relative to control. **D** Amplitude of different sub-populations represented relative to control. **E** Single channel burst (SCB) of different sub-populations represented relative to control. **F** Number of network bursts (NB) of different sub-populations represented relative to control. **G** Spike shape of each ASD subpopulation compared to the representative spike of the control. **H** Normalized amplitude histograms of each ASD sub-population compared to the control. C-F, Dots are the average of all the brain organoids of each patient, and lines are the SEM of all the brain organoids of each patient. Kruskal-Wallis test followed by Dunn’s multiple comparisons (C-F), *>0.05, **>0.01, ****>0.0001.

We characterized the spontaneous, resting-state electrophysiological activity of brain organoids derived from syndromic and idiopathic autism spectrum disorder (ASD) patients and compared them to healthy controls (Fig. 2). Representative four-second recordings illustrate apparent differences in neuronal dynamics across groups, including patterns of hyperactivity, hypoactivity, altered burst synchrony, and amplitude variability. Each ASD subpopulation is consistently color-coded throughout the study for clarity (Fig. 2B).

We quantified several key electrophysiological features: mean firing rate, signal amplitude, and mean number of single-channel bursts. All ASD-derived organoids displayed significant deviations from the control group in at least one of these baseline parameters. The idiopathic ASD organoids showed a consistent hypoactive profile, with significantly reduced mean firing rate, amplitudes, and single-channel bursts compared to controls (all p < 0.0001).

In contrast, most syndromic ASD subtypes exhibited significantly increased mean firing rates, including STXBP1 (p < 0.01), PPP2R5D (Jordan’s syndrome) (p < 0.02), and GRIN2B (p < 0.0001). The SCN2A-derived organoids revealed heterogeneity in firing rate across patient lines, with a significant difference between them. Notably, both SCN2A lines showed significantly reduced amplitudes compared to controls (p < 0.0001), independent of their differing firing rates.

Amplitude dynamics are visualized in Fig. 2G, where 20 randomly selected signals from each group are overlaid on the mean signal and the control average for comparison. The normalized amplitude distribution is presented in Fig. 2H.

### Characterization of short-term synaptic plasticity in patient-derived brain organoids from syndromic and idiopathic ASD patients and neurotypical controls (Fig. 3)

**Fig. 3:**
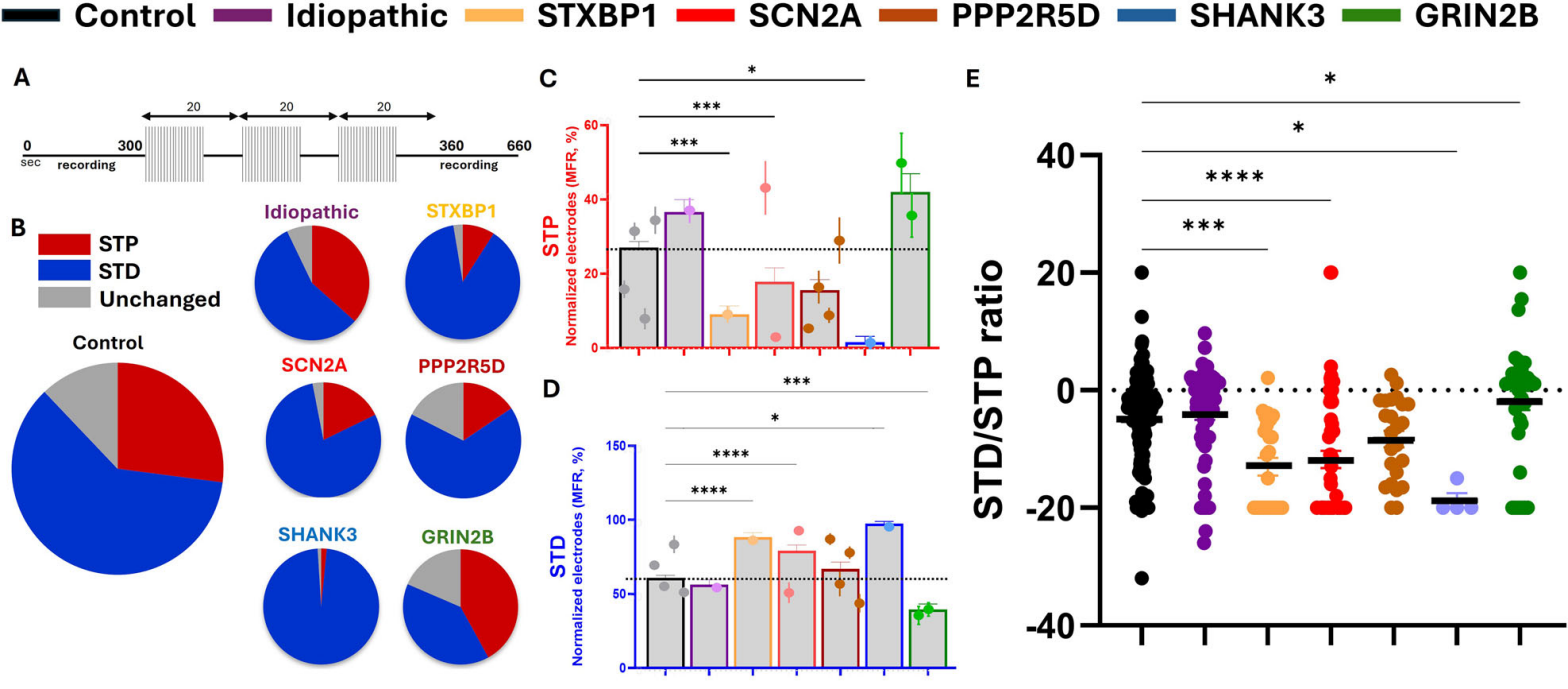
Characterization of short-term plasticity and comparison of various patient-derived brain organoid lines in ASD. **A** Stimulation protocol scheme. **B** Averages of STD, STP, and unchanged electrodes are presented in pie charts. **C** MFR-based STP in each sub-population of patient-derived brain organoids. **D** MFR-based STD in each sub-population of patient-derived brain organoids. **E** MFR-based STP/STD ratio in each sub-population of patient-derived brain organoids, with positive values representing a higher STP relative to STD (STP/STD), and negative values indicating a higher STD relative to STP (STD/STP). The MFR-based STP/STD ratio in each sub-population represents varying depressive tendencies of brain organoids. Kruskal-Wallis test followed by Dunn’s multiple comparisons, *>0.05, **>0.01, ***>0.001.

To investigate short-term synaptic plasticity across brain organoids, we applied a three-cycle electrical stimulation paradigm using a multi-electrode array (MEA) to evoke short-term depression (STD) and short-term potentiation (STP) responses. In this context, we use the terms STP and STD to describe network-level, activity-dependent modulation of evoked responses over short timescales, as detected at the population level by MEA recordings, rather than classical presynaptic–postsynaptic mechanisms. Comparable plasticity-like dynamics have been reported in prior organoid studies using MEA stimulation paradigms [34]. The distribution of STD- and STP-dominant responses, as well as the proportion of unchanged recordings, is shown in the pie charts (Fig. 3B). Across all sub-populations, responses were predominantly STD-biased (blue), with STP (red) representing only a minority. This STD predominance can be explained by the high-frequency stimulation protocol, which is known to favor depression. In addition, the scRNA-seq data indicates a maturing neuronal state together with robust presynaptic GABA_B machinery (GABBR1/2 with KCNJ6), a pathway that depresses transmitter release on seconds-to-minutes timescales, both of which likely contribute to the pronounced STD observed.

Quantitative analysis of STP responses revealed significantly reduced STP response in several syndromic lines compared to controls (Fig. 3C), including STXBP1 (p < 0.001) and SHANK3 (p < 0.05) lines, as well as one of the SCN2A lines (p < 0.001). Complementary data from STD analysis revealed a significant increase in STD for these lines (Fig. 3D). This mirrored response suggests that the increase of the STD portion, to some extent, occurred at the expense of the STP portion.

Notably, GRIN2B-derived organoids exhibited a slightly elevated STP response compared to the control lines (Fig. 3C), while demonstrating a significant reduction in STD (p < 0.01, Fig. 3D). This suggests that the reduction in STD was accompanied by a larger proportion of unchanged responses in the mean firing rate. In contrast to the aforementioned lines, no significant differences were observed in STP or STD amplitudes for the idiopathic and PPP2R5D-derived organoids relative to controls. These findings highlight the variations in STD and STP responses observed among ASD subpopulations, suggesting group-specific plasticity dynamics.

Lastly, individual data points of the STD/STP ratio for each sub-population are presented in Fig. 3E, with positive values reflecting a higher STP relative to STD (STP/STD) and negative values indicating a higher STD relative to STP (STD/STP). The data demonstrates variability across patient-derived organoids, with a similar average ratio observed for the control group and the idiopathic ASD line. The STXBP1, SCN2A, PPP2R5D, and SHANK3 groups exhibited a significant negative shift in the STD/STP ratio, whereas GRIN2B organoids presented a slight positive shift and displayed the most balanced STD/STP distribution, suggesting a deviation from typical short-term depression mechanisms.

Together, these results suggest that specific genetic mutations associated with syndromic ASD can significantly disrupt short-term synaptic plasticity. The observed differences in STD and STP across ASD subtypes support the hypothesis that synaptic dysfunction in ASD is highly heterogeneous and genotype-dependent.

### Network connectivity analysis and comparison of Pre- and post-electrophysiological stimulation in patient-derived brain organoids (Fig. 4)

**Fig. 4:**
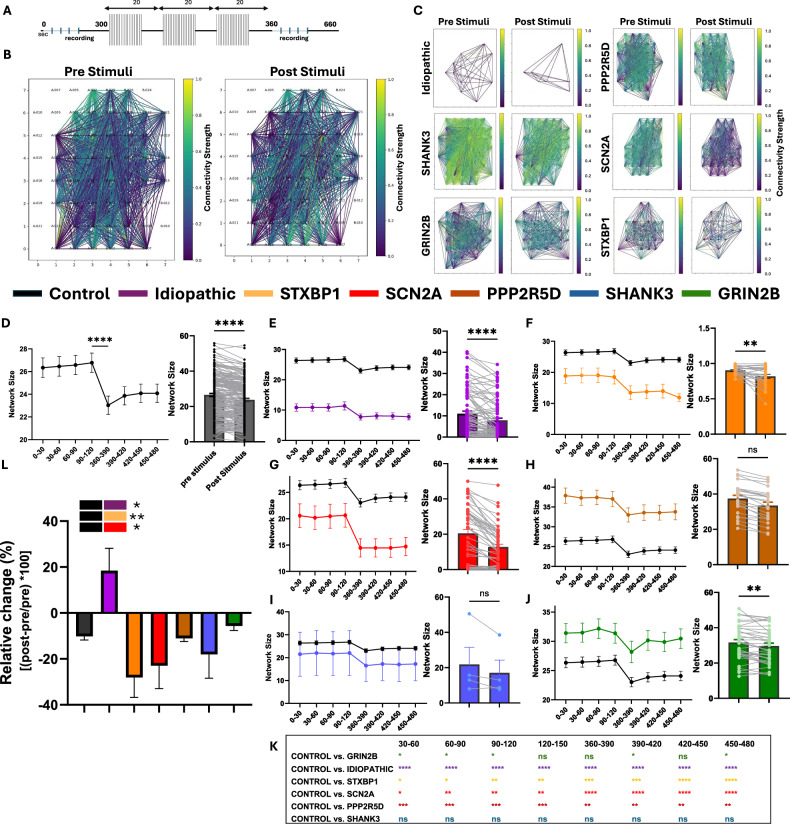
Network size and connectivity dynamic analysis and comparison between different ASD lines of patient-derived brain organoids. **A** Stimulation protocol scheme, the data were divided into 30-second bins to follow the dynamics. **B** Representative analysis of connectivity pre- and post-stimuli in the control group. **C** Representative analysis of connectivity pre- and post-stimuli in ASD groups. **D** Dynamic analysis of network size of the control group (left: time dynamics, right: pre- and post-stimuli comparison in paired analysis). **E-J** Dynamic analysis of the network size of the ASD groups, compared to the control (black). **K** Summary table of the statistical differences for each group compared to the control at each time point. (Fisher’s exact test, *<0.05, **<0.001, ****<0.0001). **L** The relative influence of the stimuli in percentages on the “network size” parameter. Statistics represent the comparison to the control group. Kruskal-Wallis test, *<0.05, **<0.001, ****<0.0001.

To evaluate network-level functional connectivity and its responsiveness to external stimulation across patient-derived brain organoids, we applied a spike-time cross-correlation analysis on spontaneous neuronal activity recorded in 30-second bins before and after electrical stimulation (Fig. 4A). This method enabled quantification of dynamic changes in network structure, including network size (number of functionally connected nodes) and average connectivity strength, across both baseline and evoked states.

Quantitative graph-theoretical analysis of network structure (Fig. 4) revealed differences between neurotypical controls and ASD-derived organoids, including variations in network size, density, and clustering. The control organoids exhibited relatively dense and stable connectivity patterns pre- and post-stimulation, while ASD-derived organoids showed greater variability, with some exhibiting fragmentation or hyperconnectivity, suggesting altered functional organization at the network level. Representative connectivity maps are shown for visualization (Fig. 4B, C), while statistical comparisons of these metrics are presented in Fig. 4D-K.

Temporal dynamics of network size were plotted over a 120-second window (Fig. 4D-J). In the control group (Fig. 4D), connectivity remained relatively stable pre-stimulation, followed by a modest and reproducible decline in network size post-stimulation. This pattern likely reflects a stimulus-induced modulation of functional connectivity following input. Paired t-test analysis confirmed a significant post-stimulation decrease in network size in the control group (p < 0.05).

In contrast, ASD-derived organoids displayed heterogeneous and often dysregulated responses to stimulation:

- **Idiopathic ASD organoids** (Fig. 4E) showed reduced adaptability, with minimal post-stimulation change in network size, diverging significantly from the control profile at multiple time points (p < 0.05).
- **STXBP1-derived organoids** (Fig. 4F) displayed a pronounced and early collapse in network size post-stimulation, with a significant paired reduction (p < 0.001), suggesting impaired recovery and network fragility.
- **SCN2A lines** (Fig. 4G) also showed a significant network reduction post-stimulation (p < 0.01), though with a delayed onset compared to STXBP1.
- **PPP2R5D (Jordan’s syndrome) organoids** (Fig. 4H) maintained higher pre-stimulation network size but demonstrated a sharp drop immediately following stimulation (p < 0.0001), indicating potential hyperconnectivity at baseline with impaired dynamic regulation.
- **GRIN2B organoids** (Fig. 4I) showed the most consistent deviation from control, with an attenuated and erratic response across the full time window (p < 0.05 at multiple time points).
- **SHANK3 organoids** (Fig. 4J) showed a moderate reduction in connectivity, statistically distinct from the control profile at late time points (p < 0.05).

Statistical comparisons at each time point between ASD subgroups and the control (Fig. 4K) confirmed significant deviations in connectivity dynamics in multiple ASD lines, particularly STXBP1, GRIN2B, and PPP2R5D.

Finally, Fig. 4L summarizes the relative change in connectivity, calculated as a percentage [(post-pre)/pre × 100], offering a quantitative measure of the stimulus-driven shift in network activity. STXBP1, SCN2A, PPP2R5D, and GRIN2B organoids all demonstrated significantly greater relative reductions in network size compared to controls, while SHANK3 and idiopathic ASD organoids showed more subtle or variable shifts.

Together, these results indicate that patient-derived organoids from different ASD subtypes exhibit distinct network-level phenotypes, with altered functional connectivity dynamics and reduced plasticity in response to stimulation. These patterns appear to be mutation-specific, supporting the use of network-level analysis as a discriminative biomarker of circuit dysfunction in ASD.

### Multidimensional characterization and comparison of electrophysiological features within and between groups (Fig. 5)

**Fig. 5:**
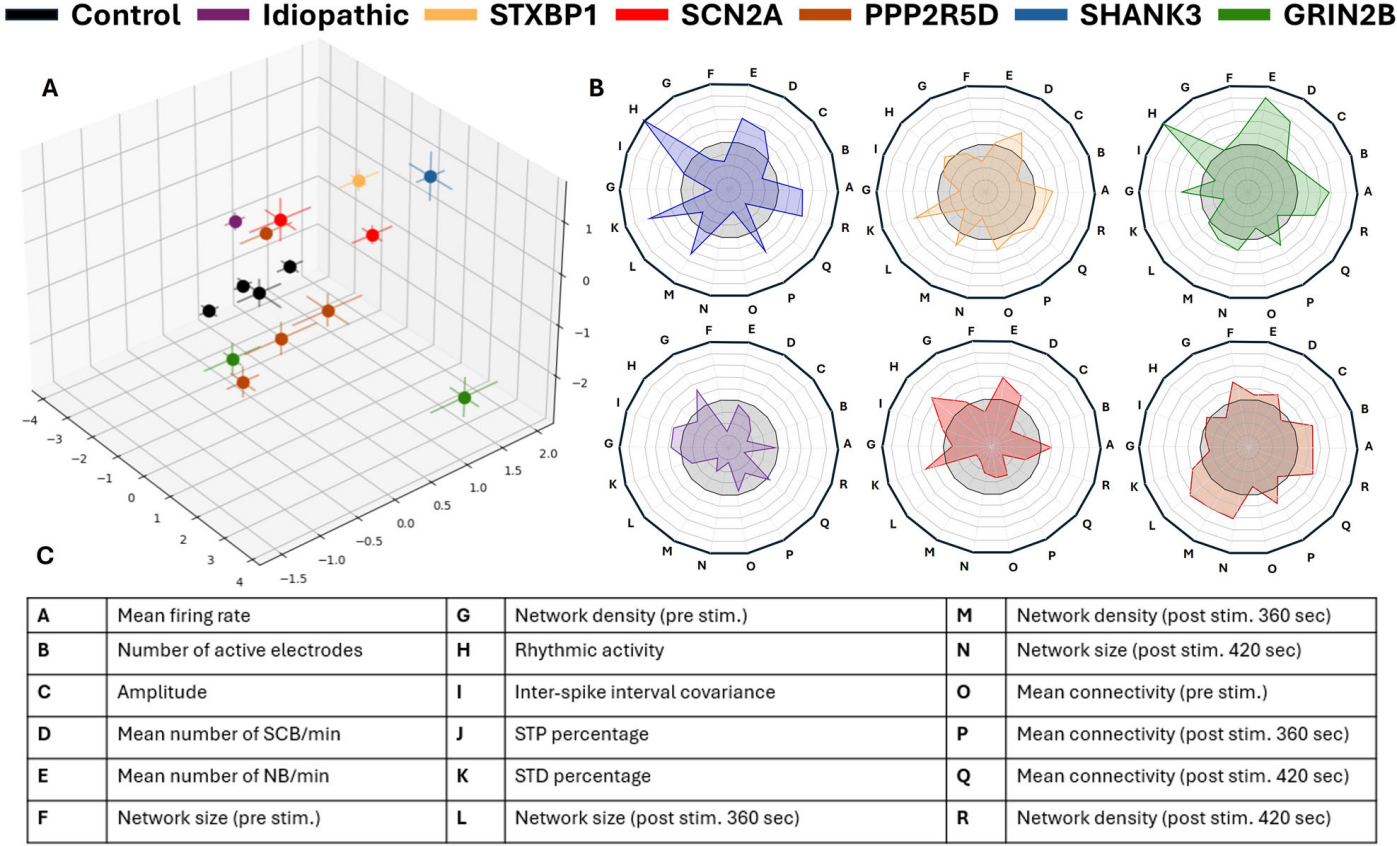
Multidimensional clustering of electrophysiological parameters in brain organoids from ASD subpopulations. **A** Principal component analysis combines 18 parameters into 3D dimensions. Each patient’s data is represented by the mean (dot) and the standard error of the mean (lines) from all the brain organoids of that patient. Group differences in PCA space were statistically evaluated using permutational multivariate analysis of variance (PERMANOVA), which showed a significant effect of group (p < 0.001); Holm-adjusted pairwise comparisons confirmed that controls differed significantly from all ASD groups (p < 0.05). **B** Radar plots show the impact of various parameters, compared to the control (normalized to 1, gray rectangle). **C** List of the features included in the PCA and radar plot analyses.

To summarize and visualize the diversity in electrophysiological features across all samples, we applied principal component analysis (PCA), which enabled us to reduce 18 electrophysiological parameters into a three-dimensional space based on their similarity (Fig. 5A). In this clustering space, each patient is represented by a dot (representing the mean of their brain organoid data) and error bars (representing the standard error of the mean, or SEM), allowing for the assessment of both intra-subject and inter-subject variability. This approach revealed that variation between organoids from the same individual was relatively small, while inter-subject differences were more significant, especially between ASD patients and controls. The control group clustered closely, indicating minimal variability, whereas both syndromic and idiopathic ASD samples showed broader dispersion. These observations were statistically supported by permutational multivariate analysis of variance (PERMANOVA), which revealed a significant group effect (p < 0.001). Holm-adjusted pairwise comparisons further showed that controls differed significantly from all ASD subgroups (p < 0.05).

To better understand which electrophysiological parameters contributed to group differentiation, we used radar plots normalized to the control group (value = 1), highlighting deviations in ASD groups across multiple features (Fig. 5B). A complete list of the 18 parameters included in the PCA is provided in the accompanying table (Fig. 5C).

As with any biological model system, organoids inevitably carry intrinsic variability. However, such variability is expected to influence all lines in a broadly similar fashion. The fact that PCA and radar plots revealed consistent, reproducible clustering of subtype-specific phenotypes, together with converging alterations across multiple electrophysiological domains, strengthens the interpretation that these differences represent genuine genotype-driven signatures rather than artifacts of the model system.

## Discussion

In this study, we aimed to extensively characterize and compare the electrophysiological patterns of brain organoids generated from individuals with syndromic and non-syndromic ASD, including cases with **SHANK3 haploinsufficiency** (Phelan-McDermid syndrome), **PPP2R5D** (Jordan’s syndrome), **GRIN2B syndrome,** **SCN2A syndrome**, and **STXBP1 syndrome**, relative to healthy controls. While previous studies have reported molecular and electrophysiological alterations in ASD brain organoids, including in SHANK3 haploinsufficiency [22, 35], our study provides a novel and systematic comparison across multiple ASD subtypes. We examined organoids derived from individuals with both syndromic and non-syndromic autism and found distinct electrophysiological alterations relative to controls. These abnormalities affect core aspects of neuronal coding, an essential feature of functional neural networks and cognitive processing. Here, we analyzed over 400 brain organoids derived from eleven individuals with diverse autism etiologies and four neurotypical controls. We identified distinct electrophysiological properties both between individuals with autism and controls, and across different monogenic forms of autism. Both resting-state electrophysiological properties and evoked responses (short-term potentiation and short-term depression) were studied, along with network connectivity analysis. Since electrophysiological data contains a high number of parameters, each of which can be influenced differently by the biological basis of the neurons and the organism. We used principal component analysis (PCA), an unsupervised dimensionality reduction method, to standardize and visualize high-dimensional electrophysiological data for coherent comparison. The results, represented by the mean and standard error of the mean (SEM) of all brain organoids from each patient, highlighted the relative resemblance of the brain organoids from the control patients against the high variability among the different ASD patients.

It has been widely reported that syndromic patients can still present with different clinical phenotypes, even when carrying the same genetic mutation [36–38]. This heterogeneity likely reflects multiple biological layers. The functional consequences of a given disruption depend on the specific variant class (e.g., missense, nonsense, truncating, or gain-/loss-of-function mutations) and are further shaped by the individual’s broader genetic background, including polygenic variation and secondary rare variants [39]. In addition, regulatory mechanisms such as non-coding elements and epigenetic or transcriptional programs can bias neuronal lineage specification, subtype composition, and maturation dynamics [40, 41]. Post-translational processes, including protein folding, trafficking, phosphorylation, and ubiquitination, add yet another level of variability, leading to diverse functional outcomes despite ostensibly similar genetic alterations. Together, these multilayered mechanisms provide a compelling framework for understanding inter-individual variability in syndromic ASD.

Consistent with this, we found that patients diagnosed with the same ASD syndrome (e.g., GRIN2B-related syndrome) exhibited distinct patterns of electrical activity and were not always clustered into the same group. This divergence in electrophysiological profiles aligns with the clinical differences observed between these patients (Table S1), reinforcing the notion that shared genetic mutations do not necessarily lead to uniform phenotypic outcomes. For example, only one of the GRIN2B lines had reported seizures, which was reflected in an abnormal rhythmic burst activity (Figure S3). Notably, this phenotype was observed in some but not all seizure-diagnosed patient lines, and was only rarely observed in neurotypical control organoids. This suggests that rhythmic bursting may be associated with seizure history, although larger cohorts will be required to establish its specificity and robustness. This emphasizes the importance of functional electrophysiological data as additional clinical diagnostic information on the subject, in addition to the syndromic and genetic characterizations, for a better understanding of the heterogeneity within the same syndromic ASD and between different ones.

At the same time, our findings also highlight convergent patterns across genetically distinct ASD subgroups. Despite differences in the underlying mutations, multiple lines exhibited qualitatively similar deviations from controls, including altered spontaneous firing, impaired short-term plasticity, and stimulus-induced reductions in functional connectivity (Fig. 4). Such convergence is biologically plausible, as many ASD-associated genes affect central nodes of neuronal communication, whether through ion channel activity (e.g., SCN2A), vesicle release (e.g., STXBP1), or signaling regulation (e.g., PPP2R5D), ultimately constraining the capacity of neural networks to form stable, adaptive circuits. These shared circuit-level disruptions may help explain why diverse genetic etiologies can manifest in overlapping clinical phenotypes, reinforcing the idea that ASD is shaped by both divergent and convergent mechanisms. This convergence at the network level may therefore provide a mechanistic bridge between diverse genetic etiologies and the common behavioral diagnostic criteria of ASD.

A further important dimension relates to idiopathic ASD, which accounts for the vast majority of clinical cases. In our dataset, idiopathic lines exhibited distinct patient-specific electrophysiological phenotypes that were separable using principal component analysis (PCA) based on high-dimensional functional features. These findings suggest that organoid-based functional phenotyping can help identify biologically meaningful sub-populations within idiopathic ASD, reflecting differences in circuit-level properties that are not apparent from genetic data alone. Such stratification may support more refined diagnostic categories and guide future therapeutic targeting.

Furthermore, while animal models have been used for several decades to study neurological and psychiatric disorders, their translational relevance remains limited, mainly in drug testing and development, with less than 8% success in translation from pre-clinical studies to clinical practice [42]. This challenge is further compounded by the human brain’s biological complexity, making it difficult to develop reliable animal models even for monogenic neurodevelopmental disorders. Despite a known single-gene mutation, these models often fail to accurately recapitulate the human phenotype. This limitation is even more pronounced in complex multifactorial conditions such as ASD, where the interplay of genetic and environmental factors cannot be easily replicated in animal systems.

In this context, patient-derived brain organoids provide a complementary human platform for functional phenotyping. Brain organoids do not replicate the full anatomical and structural complexity of the mature human brain. However, many mature brain dysfunctions either originate in, or share a common biological basis with, early neurodevelopmental abnormalities - such as impaired neurogenesis, defective synaptogenesis, or aberrant early network formation [43, 44]. A growing body of evidence suggests that disruptions in early cortical development contribute directly to the pathogenesis of neurological and psychiatric conditions that clinically manifest later in life, including autism spectrum disorder, epilepsy, and schizophrenia [45–47]. Thus, brain organoids can reveal developmental abnormalities that may underlie, precede, or parallel phenotypes observed in mature brain disorders.

Supporting this broader applicability, brain organoids are increasingly being used to model not only early neurodevelopment but also late-onset and progressive conditions such as Alzheimer’s disease, Parkinson’s disease, and schizophrenia - particularly when cultured over extended periods that permit functional and molecular maturation [48–50].

In summary, we provide a comprehensive electrophysiological characterization of brain organoids derived from individuals with both syndromic and non-syndromic ASD, compared to neurotypical controls. By analyzing over 400 organoids from diverse ASD etiologies, we identified distinct patterns of neuronal activity across and within ASD subtypes, including heterogeneity among individuals diagnosed with the same ASD syndrome. These alterations, observed in resting-state activity, evoked responses, and network dynamics, were further resolved using dimensionality reduction. Notably, our study highlights the critical value of functional electrophysiological readouts in revealing ASD-specific signatures and distinguishing subpopulations, even within genetically defined syndromes. This underscores the potential of patient-derived brain organoids to serve not only as a complement to genetic diagnosis but also as a powerful translational platform for uncovering mechanistic insights into ASD heterogeneity, enabling diagnostics in non-syndromic or idiopathic cases, and guiding future personalized therapeutic strategies [51–54].

## Supplementary information

Supplementary Information

Dataset 1

## Data Availability

scRNA sequencing data generated in this study is provided as Supplementary Data files accompanying this manuscript.

## References

[1] Steinmetz JD, Seeher KM, Schiess N, Nichols E, Cao B, Servili C, et al. Global, regional, and national burden of disorders affecting the nervous system, 1990–2021: a systematic analysis for the Global Burden of Disease Study 2021. Lancet Neurol. 2024;23:344–81. 38493795 10.1016/S1474-4422(24)00038-3 PMC10949203

[2] American Psychiatric Association Diagnostic and Statistical Manual of Mental Disorders. Washington DC: American Psychiatric Association; 2022.

[3] Zeidan J, Fombonne E, Scorah J, Ibrahim A, Durkin MS, Saxena S, et al. Global prevalence of autism: A systematic review update. Autism Res. 2022;15:778–90. 35238171 10.1002/aur.2696 PMC9310578

[4] Sandin S, Lichtenstein P, Kuja-Halkola R, Larsson H, Hultman CM, Reichenberg A. The familial risk of autism. JAMA. 2014;311:1770–7. 24794370 10.1001/jama.2014.4144 PMC4381277

[5] Modabbernia A, Velthorst E, Reichenberg A. Environmental risk factors for autism: an evidence-based review of systematic reviews and meta-analyses. *Mol. Autism 2017;*10.1186/s13229-017-0121-4 10.1186/s13229-017-0121-4 PMC5356236 28331572

[6] Chaste P, Leboyer M. Autism risk factors: genes, environment, and gene-environment interactions. Dialogues Clin Neurosci. 2012;14:281–92. 23226953 10.31887/DCNS.2012.14.3/pchaste PMC3513682

[7] Satterstrom FK, Kosmicki JA, Wang J, Breen MS, De Rubeis S, An J-Y, et al. Large-scale exome sequencing study implicates both developmental and functional changes in the neurobiology of autism. Cell. 2020;180:568–84.e23. 31981491 10.1016/j.cell.2019.12.036 PMC7250485

[8] De Rubeis S, He X, Goldberg AP, Poultney CS, Samocha K, Cicek AE, et al. Synaptic, transcriptional and chromatin genes disrupted in autism. Nature. 2014;515:209–15. 25363760 10.1038/nature13772 PMC4402723

[9] Durand CM, Perroy J, Loll F, Perrais D, Fagni L, Bourgeron T, et al. SHANK3 mutations identified in autism lead to modification of dendritic spine morphology via an actin-dependent mechanism. Mol Psychiatry. 2012;17:71–84. 21606927 10.1038/mp.2011.57 PMC3252613

[10] Uchino S, Waga C. SHANK3 as an autism spectrum disorder-associated gene. Brain Dev. 2013;35:106–10. 22749736 10.1016/j.braindev.2012.05.013

[11] Shang L, Henderson LB, Cho MT, Petrey DS, Fong C-T, Haude KM, et al. De novo missense variants in PPP2R5D are associated with intellectual disability, macrocephaly, hypotonia, and autism. Neurogenetics. 2016;17:43–49. 26576547 10.1007/s10048-015-0466-9 PMC4765493

[12] Platzer K, Yuan H, Schütz H, Winschel A, Chen W, Hu C, et al. GRIN2B encephalopathy: Novel findings on phenotype, variant clustering, functional consequences and treatment aspects. J Med Genet. 2017;54:460–70. 28377535 10.1136/jmedgenet-2016-104509 PMC5656050

[13] Weiss LA, Escayg A, Kearney JA, Trudeau M, MacDonald BT, Mori M, et al. Sodium channels SCN1A, SCN2A and SCN3A in familial autism. Mol Psychiatry. 2003;8:186–94. 12610651 10.1038/sj.mp.4001241

[14] Freibauer A, Wohlleben M, Boelman C. STXBP1-related disorders: clinical presentation, molecular function, treatment, and future directions. Genes. 2023;14:2179 10.3390/genes14122179. 38137001 10.3390/genes14122179 PMC10742812

[15] Spoto G, Valentini G, Saia MC, Butera A, Amore G, Salpietro V, et al. Synaptopathies in developmental and epileptic encephalopathies: a focus on pre-synaptic dysfunction. Front Neurol. 2022;13:826211 10.3389/fneur.2022.826211. 35350397 10.3389/fneur.2022.826211 PMC8957959

[16] Sestan N, State MW. Lost in translation: traversing the complex path from genomics to therapeutics in autism spectrum disorder. Neuron. 2018;100:406–23. 30359605 10.1016/j.neuron.2018.10.015 PMC6989093

[17] Avino TA, Barger N, Vargas MV, Carlson EL, Amaral DG, Bauman MD, et al. Neuron numbers increase in the human amygdala from birth to adulthood, but not in autism. Proc Natl Acad Sci USA. 2018;115:3710–5. 29559529 10.1073/pnas.1801912115 PMC5889677

[18] Lancaster MA, Renner M, Martin C-A, Wenzel D, Bicknell LS, Hurles ME, et al. Cerebral organoids model human brain development and microcephaly. Nature. 2013;501:373–9. 23995685 10.1038/nature12517 PMC3817409

[19] Steinberg DJ, Repudi S, Saleem A, Kustanovich I, Viukov S, Abudiab B, et al. Modeling genetic epileptic encephalopathies using brain organoids. EMBO Mol Med. 2021;13:e13610. 34268881 10.15252/emmm.202013610 PMC8350905

[20] Wang L, Li Z, Sievert D, Smith DEC, Mendes MI, Chen DY, et al. Loss of NARS1 impairs progenitor proliferation in cortical brain organoids and leads to microcephaly. Nat Commun. 2020;11:4038. 10.1038/s41467-020-17454-4. 32788587 10.1038/s41467-020-17454-4 PMC7424529

[21] Wulansari N, Darsono WHW, Woo H-J, Chang M-Y, Kim J, Bae E-J, et al. Neurodevelopmental defects and neurodegenerative phenotypes in human brain organoids carrying Parkinson’s disease-linked DNAJC6 mutations. Sci Adv. 2021;7:eabb1540 10.1126/sciadv.abb1540. 33597231 10.1126/sciadv.abb1540 PMC7888924

[22] Wang Y, Chiola S, Yang G, Russell C, Armstrong CJ, Wu Y, et al. Modeling human telencephalic development and autism-associated SHANK3 deficiency using organoids generated from single neural rosettes. Nat Commun. 2022;13:5688. 10.1038/s41467-022-33364-z. 36202854 10.1038/s41467-022-33364-z PMC9537523

[23] Brighi C, Salaris F, Soloperto A, Cordella F, Ghirga S, de Turris V, et al. Novel fragile X syndrome 2D and 3D brain models based on human isogenic FMRP-KO iPSCs. Cell Death Dis. 2021. 10.1038/s41419-021-03776-8. 33993189 10.1038/s41419-021-03776-8 PMC8124071

[24] Chen X, Sun G, Tian E, Zhang M, Davtyan H, Beach TG, et al. Modeling Sporadic Alzheimer’s Disease in Human Brain Organoids under Serum Exposure. Adv Sci. 2021;12:498 10.1002/advs.202101462. 10.1002/advs.202101462 PMC8456220 34337898

[25] Mariani J, Simonini MV, Palejev D, Tomasini L, Coppola G, Szekely AM, et al. Modeling human cortical development in vitro using induced pluripotent stem cells. Proc Natl Acad Sci USA. 2012;109:12770–5. 22761314 10.1073/pnas.1202944109 PMC3411972

[26] Zafarullah M, Jasoliya M, Tassone F. Urine-derived epithelial cell lines: A new tool to model fragile X syndrome (FXS). Cells. 2020;9:2240 10.3390/cells9102240. 33027907 10.3390/cells9102240 PMC7600987

[27] Zhou T, Benda C, Dunzinger S, Huang Y, Ho JC, Yang J, et al. Generation of human induced pluripotent stem cells from urine samples. Nat Protoc. 2012;7:2080–9. 23138349 10.1038/nprot.2012.115

[28] Okita K, Matsumura Y, Sato Y, Okada A, Morizane A, Okamoto S, et al. A more efficient method to generate integration-free human iPS cells. Nat Methods. 2011;8:409–12. 21460823 10.1038/nmeth.1591

[29] Wagenaar DA, Pine J, Potter SM. An extremely rich repertoire of bursting patterns during the development of cortical cultures. BMC Neurosci. 2006;7:11. 10.1186/1471-2202-7-11. 16464257 10.1186/1471-2202-7-11 PMC1420316

[30] Obien MEJ, Deligkaris K, Bullmann T, Bakkum DJ, Frey U. Revealing neuronal function through microelectrode array recordings. Front Neurosci. 2015;8:423 10.3389/fnins.2014.00423. 25610364 10.3389/fnins.2014.00423 PMC4285113

[31] Frega M, Tedesco M, Massobrio P, Pesce M, Martinoia S. Network dynamics of 3D engineered neuronal cultures: A new experimental model for in-vitro electrophysiology. Sci Rep. 2014;4:5489. 10.1038/srep05489. 24976386 10.1038/srep05489 PMC4074835

[32] Bürkner PC, Doebler P, Holling H. Optimal design of the wilcoxon–mann–whitney-test. Biom J. 2017;59:25–40. 27243898 10.1002/bimj.201600022

[33] Fagerland MW. *T-Tests*, non-parametric tests, and large studies-a paradox of statistical practice?. BMC Med Res Methodol. 2012;12:78. 10.1186/1471-2288-12-78. 22697476 10.1186/1471-2288-12-78 PMC3445820

[34] Sharf T, van der Molen T, Glasauer SMK, Guzman E, Buccino AP, Luna G, et al. Functional neuronal circuitry and oscillatory dynamics in human brain organoids. Nat Commun. 2022;13:4403. 10.1038/s41467-022-32115-4. 35906223 10.1038/s41467-022-32115-4 PMC9338020

[35] Mariani J, Coppola G, Zhang P, Abyzov A, Provini L, Tomasini L, et al. FOXG1-dependent dysregulation of GABA/glutamate neuron differentiation in autism spectrum disorders. Cell. 2015;162:375–90. 26186191 10.1016/j.cell.2015.06.034 PMC4519016

[36] Rolland T, Cliquet F, Anney RJL, Moreau C, Traut N, Mathieu A, et al. Phenotypic effects of genetic variants associated with autism. Nat Med. 2023;29:1671–80. 37365347 10.1038/s41591-023-02408-2 PMC10353945

[37] Garotti R, Marino M, Riccio MP, Cappuccio G, Maffettone V, Bravaccio C. Variability in autism spectrum phenotypes linked to heterozygous missense familial ANK2 mutation. Eur J Med Genet. 2025;74:105001 10.1016/j.ejmg.2025.105001. 39978592 10.1016/j.ejmg.2025.105001

[38] Trinh S, Arnett A, Kurtz-Nelson E, Beighley J, Picoto M, Bernier R. Transcriptional subtyping explains phenotypic variability in genetic subtypes of autism spectrum disorder. Dev Psychopathol. 2020;32:1353–61. 32912353 10.1017/S0954579420000784 PMC7709958

[39] Doan RN, Lim ET, De Rubeis S, Betancur C, Cutler DJ, Chiocchetti AG, et al. Recessive gene disruptions in autism spectrum disorder. Nat Genet. 2019;51:1092–8. 31209396 10.1038/s41588-019-0433-8 PMC6629034

[40] Velmeshev D, Schirmer L, Jung D, Haeussler M, Perez Y, Mayer S, et al. Single-Cell Genomics Identifies Cell Type-Specific Molecular Changes in Autism. Science. 2019;364:658–89. 31097668 10.1126/science.aav8130 PMC7678724

[41] Trevino AE, Müller F, Andersen J, Sundaram L, Kathiria A, Shcherbina A, et al. Chromatin and gene-regulatory dynamics of the developing human cerebral cortex at single-cell resolution. Cell. 2021;184:5053–69.e23. 34390642 10.1016/j.cell.2021.07.039

[42] Marshall LJ, Bailey J, Cassotta M, Herrmann K, Pistollato F. Poor Translatability of Biomedical Research Using Animals - A Narrative Review. Altern Lab Anim. 2023;51:102–35. 36883244 10.1177/02611929231157756

[43] Volpe JJ. Brain injury in premature infants: a complex amalgam of destructive and developmental disturbances. Lancet Neurol. 2009;8:110–24. 19081519 10.1016/S1474-4422(08)70294-1 PMC2707149

[44] Di Martino A, Yan C-G, Li Q, Denio E, Castellanos FX, Alaerts K, et al. The autism brain imaging data exchange: Towards a large-scale evaluation of the intrinsic brain architecture in autism. Mol Psychiatry. 2014;19:659–67. 23774715 10.1038/mp.2013.78 PMC4162310

[45] Silbereis JC, Pochareddy S, Zhu Y, Li M, Sestan N. The Cellular and Molecular Landscapes of the Developing Human Central Nervous System. Neuron. 2016;89:248–68. 10.1016/j.neuron.2015.12.008. 26796689 10.1016/j.neuron.2015.12.008 PMC4959909

[46] Stiles J, Jernigan TL. The basics of brain development. Neuropsychology Rev. 2010;20:327–48. 10.1007/s11065-010-9148-4 PMC2989000 21042938

[47] Courchesne E, Pierce K, Schumann CM, Redcay E, Buckwalter JA, Kennedy DP, et al. Mapping early brain development in autism. Neuron. 2007;56:399–413. 17964254 10.1016/j.neuron.2007.10.016

[48] Gonzalez C, Armijo E, Bravo-Alegria J, Becerra-Calixto A, Mays CE, Soto C. Modeling amyloid beta and tau pathology in human cerebral organoids. Mol Psychiatry. 2018;23:2363–74. 30171212 10.1038/s41380-018-0229-8 PMC6594704

[49] Notaras M, Lodhi A, Dündar F, Collier P, Sayles NM, Tilgner H, et al. Schizophrenia is defined by cell-specific neuropathology and multiple neurodevelopmental mechanisms in patient-derived cerebral organoids. Mol Psychiatry. 2022;27:1416–34. 34789849 10.1038/s41380-021-01316-6 PMC9095467

[50] Kim H, Kang S, Cho B, An S, Kim Y, Kim J. Parkinson’s disease modeling using directly converted 3d induced dopaminergic neuron organoids and assembloids. Adv Sci. 2025;12:e2412548 10.1002/advs.202412548. 10.1002/advs.202412548 PMC11984911 39965129

[51] Qian X, Nguyen HN, Song MM, Hadiono C, Ogden SC, Hammack C, et al. Brain-Region-specific organoids using mini-bioreactors for modeling ZIKV Exposure. Cell. 2016;165:1238–54. 27118425 10.1016/j.cell.2016.04.032 PMC4900885

[52] Pas SP. The rise of three-dimensional human brain cultures. Nature. 2018;553:437–45. 29364288 10.1038/nature25032

[53] Chen HI, Song H, Ming GL. Applications of human brain organoids to clinical problems. Dev Dyn. 2019;248:53–64. 30091290 10.1002/dvdy.24662 PMC6312736

[54] Smirnova L, Hartung T. The promise and potential of brain organoids. Adv. Healthc. Mater. 2024;13:e2302745 10.1002/adhm.202302745. 38252094 10.1002/adhm.202302745

