# Supplementary material for: Patient-derived brain organoids reveal divergent neuronal activity across subpopulations of autism spectrum disorder
Source: Transl Psychiatry. 2026 Feb 25;16:164. doi: 10.1038/s41398-026-03890-1 (PMC13022181; doi:10.1038/s41398-026-03890-1)
Supplement: Supplementary file 1 — Supplementary Information [file 41398_2026_3890_MOESM1_ESM.pdf]

Supplementary Information

Sample	Age [years]	Sex	Protein change	Genetic mutation	Co-Morbidity	Psychiatric	Medication
Control	45	M	-	-	-	-	-
Control	35	F	-	-	-	-	-
Control	<1	Unknown	-	-	-	-	-
Control	51	F	-	-	-	-	-
Shank3	11	F	Haploinsufficiency	InsG3680$^{(+/-)}$	Infection, seizures, developmental delay	-	-
Idiopathic	13	M	-	-	-	OCD	Yes
PPP2R5D 15653-x1	9	M	Glu198Lys	592G>A	Allergy, short stature, failure thrive, diarrhea, GERD, infection, end-stage renal disease, crossed eyes, astigmatism, nearsighted, developmental delay, clumsiness, cranial nerve disorder, low and high muscle tone, large head size, spastic cerebral palsy, movement disorder, cerebral palsy	-	-
PPP2R5D 16020-x1	15.5	F	Glu420Lys	1258G>A	Constipation, large head size, movement disorder, low muscle tone, seizures, crossed eyes	-	-
PPP2R5D 15655-x1	2	F	Glu198Lys	592G>A	GERD, large head size, cort blind, low muscle tone, repetitive eye movements, crossed eyes, astigmatism, sensory integration disorder, seizures	-	Yes
PPP2R5D 16051-x1	14.5	F	Glu200Lys	598G>A	Allergy, irregular menses, hemangioma, developmental delay, seizures in sleep, constipation, hypertension, infection, clumsy, large head size, low muscle tone	Attention-deficit hyperactivity disorder	Yes
GRIN2B 15413-x1	9.5	F	Gly611Val	1832G>T	Allergy, hemangioma, short stature, constipation, GERD, infections, clumsy, small head size, low muscle tone, seizures, crosses eyes	-	Yes
GRIN2B 15265-x1	5.5	M	Gly820Ala	2459G>C	Failure thrive, constipation, GERD, infection, low muscle tone, respiratory, osteoporosis, crosses eyes	-	-
SCN2A 15473-x1	4	M	Glu1155Alafs*2	3464_3468 delAACAG	Allergy, constipation, clumsy, movement disorder, low muscle tone, seizures	Attention deficit hyperactivity disorder	Yes
SCN2A 15912-x1	1	M	Val1601Leu	4801G>T	Crossed eyes, nearsighted, seizures, cort blind, movement disorder, low and high muscle	-	Yes

					tone, hydronephrosis, infections, constipation		
STXBP1 15936-x1	4	F	Arg122*	364C>T	Allergy, seizures, constipation, clumsy, movement disorder, low muscle tone	-	Yes

Table S1: Clinical summary of this study's patients and volunteer participants.

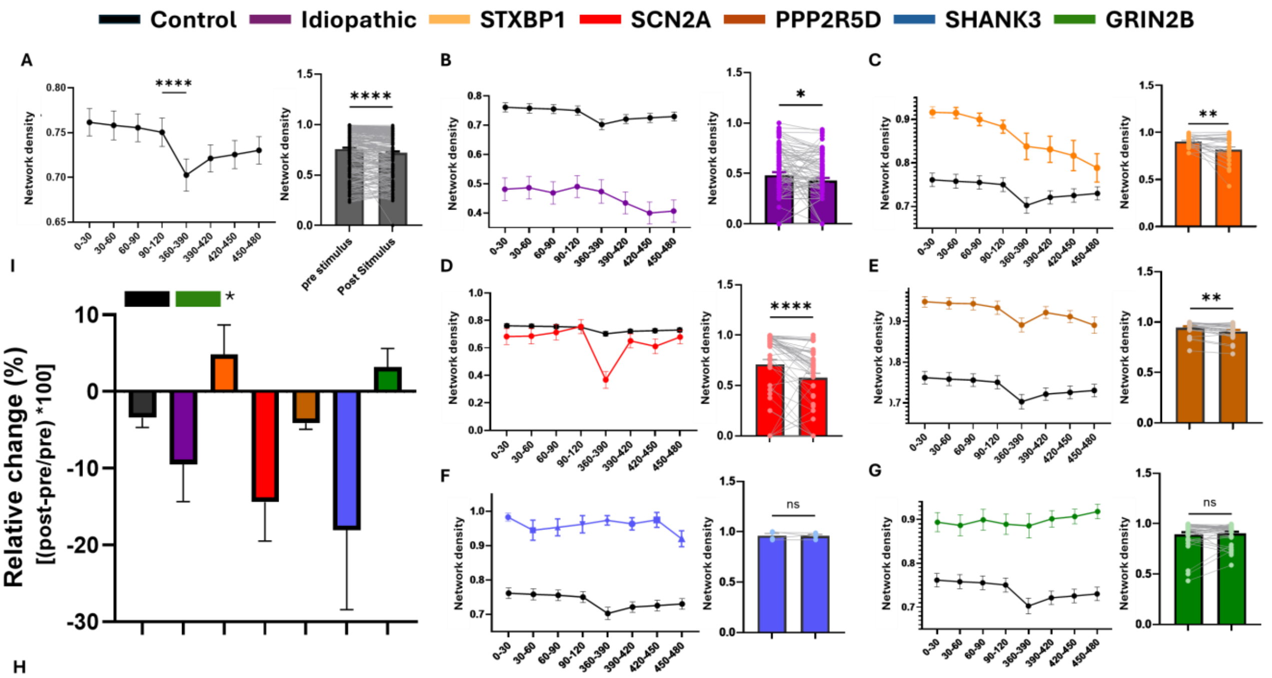

H

	30-60	60-90	90-120	120-150	360-390	390-420	420-450	450-480
Control vs. GRIN2B	**	**	****	***	ns	****	****	****
Control vs. Idiopathic	****	****	****	****	****	****	****	****
Control vs. STXBP1	**	**	*	ns	***	*	ns	ns
Control vs. SCN2A	ns	ns	*	**	****	ns	ns	ns
Control vs. PPP2R5D	****	****	****	****	**	****	***	**
Control vs. SHANK3	**	ns	ns	*	ns	*	**	ns

Figure S1: Network Density and connectivity dynamic analysis and comparison between different ASD lines of patient-derived brain organoids. A. Dynamic analysis of network density of the control group (left: time dynamics, right: pre- and post-stimuli comparison in paired analysis). **B-G**. Dynamic analysis of the network size of the ASD groups, compared to the control (black). **H.** Summary table of the statistical differences for each group compared to the control at each time point. (Fisher's exact test, *<0.05, **<0.001, ****<0.0001). **I.** The relative influence of the stimuli in percentages on the "network density" parameter. Statistics represent the comparison to the control group. (Kruskal-Wallis test, *<0.05, **<0.001, ****<0.0001).

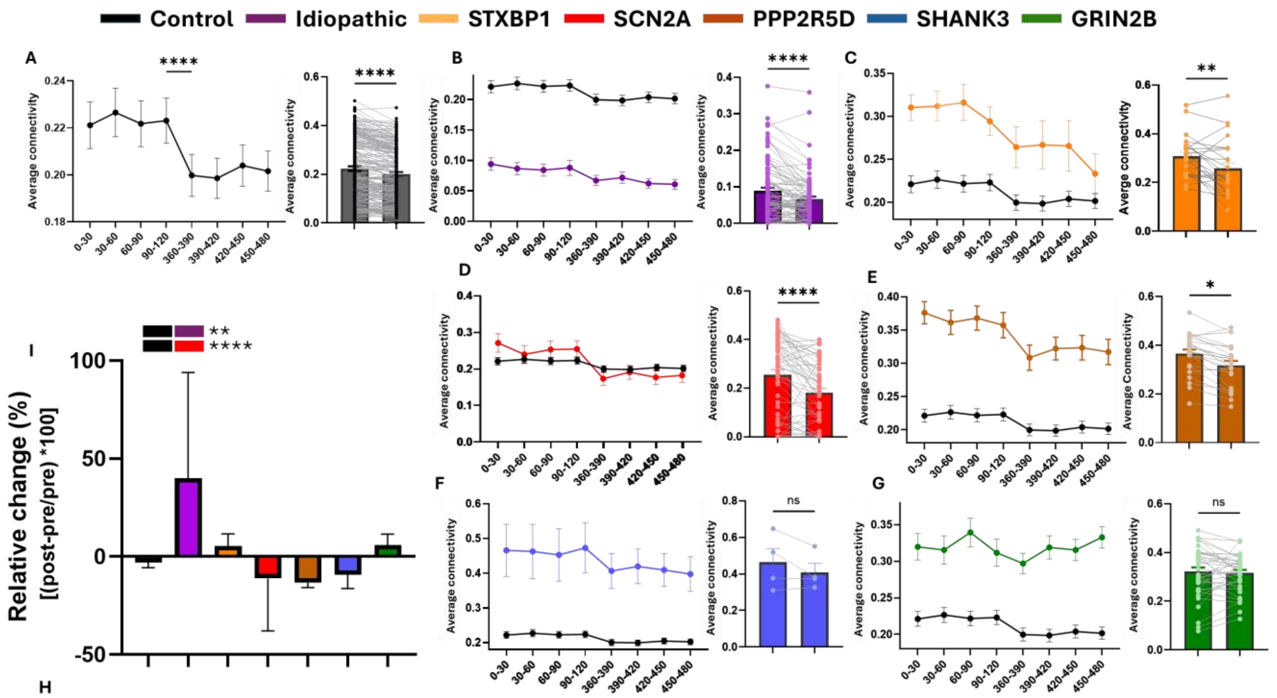

H

	30-60	60-90	90-120	120-150	360-390	390-420	420-450	450-480
Control vs. GRIN2B	***	**	****	***	****	****	****	****
Control vs. Idiopathic	****	****	****	****	****	****	****	****
Control vs. STXBP1	**	*	*	*	*	*	ns	ns
Control vs. SCN2A	ns	ns	ns	ns	ns	ns	ns	ns
Control vs. PPP2R5D	****	****	****	****	***	****	****	***
Control vs. SHANK3	*	*	*	*	**	**	**	*

Figure S2: Network average connectivity and connectivity dynamic analysis and comparison between different ASD lines of patient-derived brain organoids. A. Dynamic analysis of average connectivity of the control group (left: time dynamics, right: pre- and post-stimuli comparison in paired analysis). **B-G**. Dynamic analysis of the network size of the ASD groups, compared to the control (black). **H.** Summary table of the statistical differences for each group compared to the control at each time point. (Fisher's exact test, *<0.05, **<0.001, ****<0.0001). **I.** The relative influence of the stimuli in percentages on the "average connectivity" parameter. Statistics represent the comparison to the control group. (Kruskal-Wallis test, *<0.05, **<0.001, ****<0.0001).

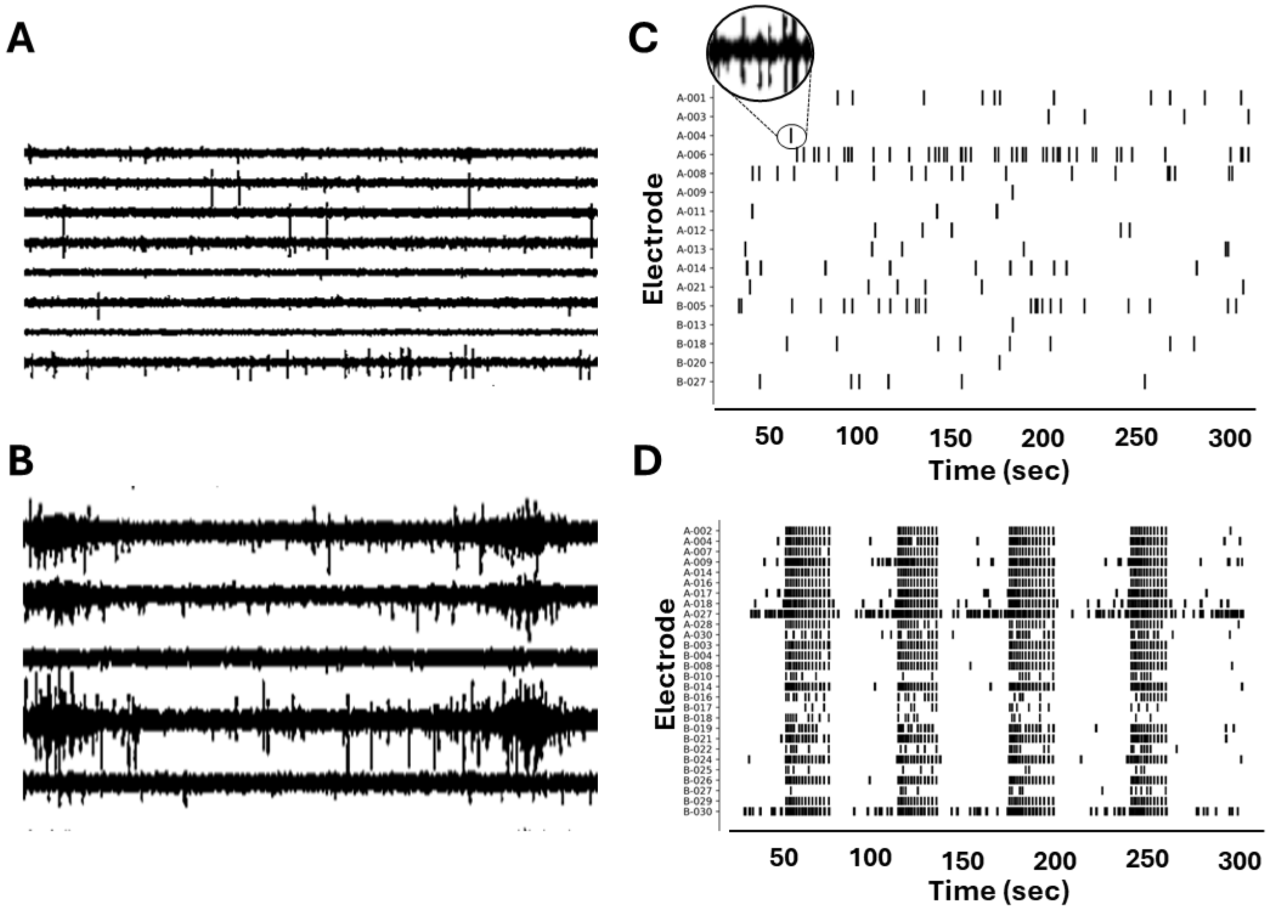

Figure S3: Abnormal rhythmic activity of synchronized bursts, presented by some of the patients with reported clinical seizures. A. Representation of the electrophysiological trace of a control patient. **B.** Representation of the electrophysiological trace of a patient with clinical seizures (GRIN2 B). **C**. Raster plot of the recordings from a control patient, each line is a single channel burst. **D.** Raster plot of the recordings from a patient with clinical seizures (GRIN2B), with each line representing a single-channel burst.
