# Supplementary material for: Patient-derived brain organoids reveal divergent neuronal activity across subpopulations of autism spectrum disorder
Source: Transl Psychiatry. 2026 Feb 25;16:164. doi: 10.1038/s41398-026-03890-1 (PMC13022181; doi:10.1038/s41398-026-03890-1)
Supplement: Supplementary file 2 — Dataset 1 [file 41398_2026_3890_MOESM2_ESM.pdf]

RNA expression in each cluster:

Gene	g0	g1	g2	g3	g4
ENSG00000238009	0.0019075	0.0057604	0.0054558	0.0000000	0.0067351
ENSG00000241860	0.0048007	0.0031739	0.0007378	0.0000000	0.0017986
ENSG00000290385	0.0013308	0.0060416	0.0009351	0.0000000	0.0054023
ENSG00000291215	0.0085606	0.0085411	0.0114114	0.0000000	0.0471034
LINC01409	0.0217304	0.0229829	0.0235124	0.0236240	0.0882061
ENSG00000290784	0.0180514	0.0156343	0.0043854	0.0144912	0.0154566
LINC00115	0.0100223	0.0020504	0.0050173	0.0054177	0.0154722
LINC01128	0.1462551	0.1851440	0.2521116	0.1256970	0.1416102
ENSG00000288531	0.0040766	0.0126859	0.0154651	0.0000000	0.0112339
FAM41C	0.0028741	0.0007587	0.0031944	0.0000000	0.0000000
ENSG00000272438	0.0173567	0.0251237	0.0273750	0.0184885	0.0368481
ENSG00000230699	0.0148786	0.0122529	0.0098456	0.0000000	0.0073584
ENSG00000241180	0.0005975	0.0008654	0.0000000	0.0000000	0.0000000
LINC02593	0.0006928	0.0034092	0.0158446	0.0000000	0.0151875
SAMD11	0.0827299	0.1324285	0.2245037	0.0649874	0.1554119
NOC2L	0.2729301	0.2894700	0.3181909	0.2460576	0.2701049
KLHL17	0.0355079	0.0388006	0.0283393	0.0118426	0.0242826
PLEKHN1	0.0016387	0.0019162	0.0000000	0.0000000	0.0046716
ENSG00000272512	0.0054869	0.0067819	0.0000000	0.0000000	0.0093787
HES4	0.6656916	0.6126319	0.5416559	0.9022208	0.4712218
ISG15	0.0547179	0.0289326	0.0178036	0.0399215	0.0358235
ENSG00000224969	0.0000000	0.0000000	0.0000000	0.0000000	0.0000000
AGRN	0.0777927	0.0898174	0.0864286	0.1393283	0.1680776
ENSG00000291156	0.0411352	0.0752112	0.0774708	0.0289816	0.0312555
C1orf159	0.0442462	0.0499331	0.0541275	0.0428742	0.1514238
ENSG00000285812	0.0023635	0.0047580	0.0049190	0.0000000	0.0217979
TTLL10	0.0017300	0.0000000	0.0063167	0.0031460	0.0026927
SDF4	0.7747645	0.5170883	0.4579274	0.9667125	0.5159973
B3GALT6	0.3157473	0.3400809	0.4573688	0.3823205	0.2311789
C1QTNF12	0.0028699	0.0043735	0.0000000	0.0081345	0.0046362
UBE2J2	0.3480282	0.3266152	0.3086531	0.3350728	0.2810294
LINC01786	0.0009823	0.0010040	0.0057972	0.0009264	0.0112693
SCNN1D	0.0090685	0.0085138	0.0042816	0.0000000	0.0104979
ACAP3	0.0989598	0.1580472	0.1302108	0.0800904	0.3542364
PUSL1	0.1018318	0.1235567	0.1108770	0.1151187	0.0896666
INTS11	0.2829244	0.2558311	0.2543225	0.3222268	0.2380876
CPTP	0.1051344	0.1230172	0.1335652	0.0934341	0.0696004
TAS1R3	0.0019384	0.0000000	0.0011920	0.0000000	0.0107984
DVL1	0.1011326	0.1345436	0.1035624	0.1079808	0.1200799
MXRA8	0.1393205	0.0919823	0.1084287	0.1840855	0.1003023
AURKAIP1	0.7268650	0.7603933	0.7703363	0.7286562	0.5470972
CCNL2	0.3018400	0.3223831	0.2690058	0.3497851	0.5079279
MRPL20-AS1	0.1018670	0.0910617	0.1019430	0.0844616	0.0774075
MRPL20	0.8709322	0.8256093	0.9014145	0.8601109	0.6266646
MRPL20-DT	0.0047064	0.0039818	0.0016575	0.0050301	0.0025298
ANKRD65	0.0038146	0.0068972	0.0099554	0.0057119	0.0131332
VWA1	0.0422415	0.0530023	0.0402170	0.0486229	0.0192082
ATAD3C	0.0010001	0.0054971	0.0050075	0.0000000	0.0088351
ATAD3B	0.0410588	0.0360285	0.0272534	0.0200212	0.0946113
ENSG00000290916	0.0069025	0.0031443	0.0056483	0.0058206	0.0151173
ATAD3A	0.0863153	0.0894909	0.0694320	0.0556220	0.0771632

TMEM240	0.0654969	0.0785526	0.1029139	0.0498057	0.0878944
SSU72	0.8385770	0.8010884	0.8079995	0.7530684	0.5977984
ENSG00000215014	0.0248853	0.0126028	0.0103835	0.0151196	0.0063087
FNDC10	0.1228085	0.1861184	0.1847757	0.0888264	0.1311993
ENSG00000286989	0.0136869	0.0386786	0.0406322	0.0119302	0.0246263
ENSG00000272106	0.1712168	0.1825618	0.1753012	0.2104435	0.1002991
MIB2	0.1778414	0.1387166	0.1258203	0.1281272	0.2107947
MMP23B	0.0049141	0.0032882	0.0009863	0.0062841	0.0084746
CDK11B	0.3340310	0.3306511	0.2854499	0.3531734	0.3538608
ENSG00000272004	0.0032124	0.0006872	0.0000000	0.0079663	0.0000000
SLC35E2B	0.2379256	0.3175303	0.3142855	0.2216129	0.2685120
CDK11A	0.1847378	0.1798262	0.1258807	0.1124964	0.1768635
ENSG00000290854	0.1735053	0.2083680	0.2310454	0.1829343	0.2147296
NADK	0.1365630	0.1148979	0.1067043	0.1088150	0.1551803
GNB1	1.3410427	1.5305884	1.7668429	1.2933486	1.4250300
GNB1-DT	0.0089730	0.0160707	0.0164294	0.0040081	0.0037962
TMEM52	0.0339178	0.0572494	0.0637767	0.0358804	0.0477827
CFAP74	0.0713196	0.0366376	0.0248212	0.1001622	0.0241985
GABRD	0.0040283	0.0030012	0.0017865	0.0130267	0.0134780
PRKCZ	0.2043219	0.3179116	0.3364064	0.2003247	0.3199785
ENSG00000271806	0.0000000	0.0017787	0.0000000	0.0000000	0.0000000
PRKCZ-AS1	0.0037010	0.0055409	0.0110039	0.0037348	0.0013524
FAAP20	0.5610172	0.5707721	0.5655731	0.6190886	0.4562212
ENSG00000234396	0.0000000	0.0013573	0.0010357	0.0000000	0.0000000
SKI	0.1371125	0.1596427	0.1080424	0.1178514	0.3329665
ENSG00000287356	0.0088354	0.0033705	0.0086814	0.0154249	0.0220986
MORN1	0.1200725	0.1010696	0.0840724	0.1407016	0.0943367
ENSG00000272420	0.0098798	0.0072165	0.0033408	0.0058558	0.0030021
RER1	0.4512424	0.3964034	0.3622564	0.5145054	0.3726482
PEX10	0.2553413	0.1696106	0.1422361	0.2918470	0.1537639
PLCH2	0.0239814	0.0398253	0.0309297	0.0229875	0.0798908
PANK4	0.0894220	0.0847728	0.0714453	0.0687499	0.0553172
HES5	0.0111677	0.0071877	0.0051557	0.0158035	0.0111870
ENSG00000272449	0.0184893	0.0125328	0.0083545	0.0230804	0.0032853
TNFRSF14	0.0009658	0.0000000	0.0042203	0.0000000	0.0046972
PRXL2B	0.1344602	0.1371330	0.1677353	0.1322543	0.0727252
MMEL1	0.0078194	0.0097420	0.0023494	0.0011930	0.0212669
MMEL1-AS1	0.0644007	0.0886158	0.1439556	0.0724324	0.0422326
TTC34	0.0032091	0.0000000	0.0000000	0.0051214	0.0000000
ENSG00000285945	0.0051894	0.0000000	0.0014078	0.0000000	0.0000000
PRDM16-DT	0.0009839	0.0000000	0.0008679	0.0000000	0.0033746
PRDM16	0.0003895	0.0000000	0.0021796	0.0000000	0.0055850
ARHGEF16	0.0004470	0.0060590	0.0060920	0.0000000	0.0050636
MEGF6	0.0155517	0.0119684	0.0084179	0.0162179	0.0345674
ENSG00000238260	0.0019178	0.0013789	0.0000000	0.0042516	0.0000000
TPRG1L	0.2802785	0.2649354	0.2782683	0.3162222	0.1821834
WRAP73	0.0903008	0.0828776	0.0711509	0.1080152	0.0849355
TP73	0.0000000	0.0000000	0.0000000	0.0000000	0.0000000
CCDC27	0.0035265	0.0013844	0.0038650	0.0033570	0.0065295
SMIM1	0.0799206	0.0418911	0.0397283	0.0675688	0.0190113
LRRC47	0.3085666	0.3271581	0.4211514	0.2717924	0.2428779
CEP104	0.2661458	0.2746439	0.2823715	0.2589768	0.2674075
DFFB	0.0166247	0.0160463	0.0265703	0.0288824	0.0427993

C1orf174	0.1724622	0.1740661	0.2109604	0.1675067	0.1297324
LINC01134	0.0029239	0.0000000	0.0000000	0.0065998	0.0000000
LINC01346	0.0000000	0.0023801	0.0019220	0.0000000	0.0041632
AJAP1	0.0720581	0.1500862	0.2204594	0.0566197	0.3242357
ENSG00000236948	0.0000000	0.0013374	0.0048122	0.0000000	0.0071930
ENSG00000289893	0.0000000	0.0000000	0.0000000	0.0000000	0.0021357
NPHP4	0.0451117	0.0507580	0.0232278	0.0264449	0.1338958
KCNAB2	0.0521503	0.0645744	0.0812702	0.0314238	0.1122011
CHD5	0.2972569	0.5139715	0.7345531	0.2695011	0.7276448
RPL22	2.6312960	2.6128622	2.4103477	2.5286243	2.3431930
RNF207-AS1	0.0059782	0.0067149	0.0105232	0.0013877	0.0000000
RNF207	0.0069591	0.0081551	0.0130047	0.0045639	0.0250751
ICMT	0.2247022	0.1952035	0.2334187	0.3149251	0.1638276
ICMT-DT	0.0000000	0.0006731	0.0000000	0.0000000	0.0000000
GPR153	0.0418988	0.0865247	0.0958881	0.0262825	0.0880662
ACOT7	0.4924466	0.8080457	0.9664262	0.3907871	0.6076002
ENSG00000271746	0.0054536	0.0032162	0.0036113	0.0000000	0.0021475
HES2	0.0016322	0.0013541	0.0000000	0.0000000	0.0000000
ESPN	0.0075677	0.0081432	0.0000000	0.0065506	0.0116946
TNFRSF25	0.0242262	0.0134008	0.0190814	0.0107964	0.0397352
PLEKHG5	0.0666665	0.0719120	0.0684019	0.0790006	0.1646858
NOL9	0.1376012	0.1589748	0.1755110	0.1683623	0.2268803
TAS1R1	0.0013326	0.0012812	0.0000000	0.0000000	0.0000000
ZBTB48	0.0569954	0.0461419	0.0390228	0.0492104	0.0647003
KLHL21	0.0308532	0.0318829	0.0155261	0.0578620	0.0231359
PHF13	0.1486843	0.1947437	0.2032100	0.1598327	0.1816719
THAP3	0.1781779	0.1459524	0.1853005	0.1722039	0.1585362
DNAJC11	0.1271629	0.1439291	0.1286447	0.1137131	0.1813982
CAMTA1-DT	0.0083039	0.0246323	0.0207803	0.0091650	0.0035834
CAMTA1	1.1020721	1.3155401	1.2133842	1.0884843	1.7720356
CAMTA1-AS3	0.0000000	0.0000000	0.0000000	0.0000000	0.0000000
CAMTA1-IT1	0.0023973	0.0017481	0.0038530	0.0023036	0.0028666
CAMTA1-AS2	0.0000000	0.0000000	0.0000000	0.0000000	0.0071718
ENSG00000270171	0.0009953	0.0043282	0.0128722	0.0000000	0.0000000
ENSG00000270035	0.0008242	0.0025081	0.0000000	0.0000000	0.0036662
ENSG00000269978	0.0090515	0.0083430	0.0107658	0.0012957	0.0087678
VAMP3	0.7282596	0.4420617	0.3553846	0.7579202	0.3339691
ENSG00000269925	0.0015226	0.0026691	0.0000000	0.0026911	0.0000000
PER3	0.1705484	0.1491195	0.0907598	0.1941962	0.2110068
ENSG00000236266	0.0008158	0.0000000	0.0000000	0.0000000	0.0056375
UTS2	0.0140662	0.0106722	0.0127215	0.0028343	0.0391161
PARK7	1.5275533	1.5128403	1.5996377	1.5758516	1.2715846
ENSG00000284747	0.0000000	0.0022120	0.0011288	0.0015747	0.0047023
ENSG00000284716	0.0000000	0.0020891	0.0000000	0.0000000	0.0000000
ERRFI1	0.1582196	0.2563340	0.2427252	0.1199604	0.2006733
ERRFI1-DT	0.0027061	0.0075909	0.0033552	0.0000000	0.0503991
SLC45A1	0.0298627	0.0770662	0.0995885	0.0335073	0.1036900
RERE	0.9582219	1.0217424	1.0971145	0.9250554	1.7495390
RERE-AS1	0.0014198	0.0027648	0.0000000	0.0019740	0.0043423
ENO1	2.6986698	2.4035331	2.2844238	2.5627925	2.1799935
ENO1-AS1	0.0156268	0.0096267	0.0134560	0.0205888	0.0097531
SLC2A5	0.0022044	0.0000000	0.0066130	0.0036343	0.0000000
GPR157	0.0152259	0.0064226	0.0135609	0.0310697	0.0132638

MIR34AHG	0.0078682	0.0060465	0.0031495	0.0146823	0.0209152
LNCTAM34A	0.0638883	0.0368276	0.0271772	0.1275697	0.0379450
H6PD	0.1247939	0.0940050	0.0399769	0.1525046	0.0912015
SPSB1	0.0580581	0.0478064	0.0256598	0.0448929	0.0270772
LINC02606	0.0519279	0.0356362	0.0072401	0.0583496	0.0064878
SLC25A33	0.2687710	0.3168203	0.4164132	0.2530246	0.2665810
TMEM201	0.0490341	0.0575446	0.0407354	0.0617321	0.0804280
PIK3CD	0.0134323	0.0497143	0.0239288	0.0309769	0.0243189
PIK3CD-AS2	0.0314305	0.0280501	0.0243265	0.0275348	0.0135179
CLSTN1	0.6105656	0.7007785	0.9174311	0.8277120	0.7424577
CTNNBIP1	0.3844319	0.5294212	0.5610930	0.3857925	0.4127247
ENSG00000223989	0.0025435	0.0012191	0.0000000	0.0032068	0.0000000
ENSG00000285701	0.0000000	0.0000000	0.0000000	0.0045161	0.0087614
LZIC	0.9048345	0.9147831	0.9289162	0.8498340	0.6809807
NMNAT1	0.1383712	0.1006554	0.0905274	0.1110206	0.1047978
RBP7	0.0058436	0.0015205	0.0030846	0.0016127	0.0046038
UBE4B	0.2522880	0.2428448	0.1965964	0.2475152	0.3872216
KIF1B	1.3876150	1.6302200	1.7420638	1.4290695	1.7278768
ENSG00000284735	0.0027814	0.0024070	0.0032143	0.0000000	0.0274689
ENSG00000284642	0.0243878	0.0243986	0.0176463	0.0045802	0.0454042
PGD	0.5626029	0.5700055	0.5183275	0.5955431	0.4313396
CENPS	0.1587214	0.1655158	0.1641846	0.1900221	0.1848457
CORT	0.0276121	0.0175383	0.0407182	0.0316119	0.0144026
DFFA	0.3596957	0.3657808	0.3732850	0.2916108	0.3514043
ENSG00000203469	0.0003861	0.0000000	0.0015813	0.0000000	0.0027747
PEX14	0.0727913	0.0943832	0.0600709	0.0834606	0.2433869
CASZ1	0.0302543	0.0387953	0.0517427	0.0330153	0.0565232
ENSG00000272078	0.0018825	0.0038405	0.0000000	0.0026207	0.0000000
TARDBP	0.4955335	0.5101612	0.6002781	0.5334991	0.4707681
MASP2	0.0033263	0.0091195	0.0019211	0.0000000	0.0040262
SRM	0.6817599	0.6744715	0.7207660	0.6758593	0.5871454
EXOSC10	0.2262440	0.2347298	0.1712820	0.2316996	0.2474303
ENSG00000226849	0.0031372	0.0004303	0.0000000	0.0037324	0.0016028
EXOSC10-AS1	0.0101412	0.0096510	0.0039401	0.0069567	0.0072417
MTOR	0.1662883	0.1557479	0.1508509	0.1333871	0.2066019
MTOR-AS1	0.0000000	0.0000000	0.0000000	0.0000000	0.0000000
ANGPTL7	0.0031101	0.0000000	0.0000000	0.0075822	0.0000000
UBIAD1	0.0861587	0.1081940	0.0930818	0.0843179	0.0910654
ENSG00000284646	0.0015902	0.0000000	0.0034679	0.0000000	0.0000000
DISP3	0.0111578	0.0147610	0.0212827	0.0093807	0.0525930
ENSG00000284708	0.0012758	0.0015852	0.0045008	0.0000000	0.0000000
FBXO2	0.2511698	0.2776556	0.2792196	0.2641611	0.1485546
FBXO44	0.2140731	0.2563557	0.2568143	0.2040399	0.1774547
MAD2L2	0.5266746	0.4859527	0.4003131	0.5987578	0.4028794
FBXO6	0.1273666	0.0972813	0.0805257	0.1070961	0.0990124
DRAXIN	0.2096256	0.3595612	0.2446170	0.2269116	0.3607639
AGTRAP	0.2357202	0.1228140	0.0651231	0.2890429	0.0809248
C1orf167	0.0014993	0.0026354	0.0056738	0.0000000	0.0019007
MTHFR	0.0953317	0.1170013	0.1213183	0.1149002	0.1831469
CLCN6	0.0650312	0.0846260	0.0750605	0.0804978	0.2213470
NPPA	0.0014667	0.0050455	0.0000000	0.0016564	0.0046563
NPPB	0.0030418	0.0058023	0.0042928	0.0038821	0.0000000
ENSG00000285646	0.0205018	0.0182377	0.0251981	0.0131405	0.0419440

KIAA2013	0.3075109	0.2216636	0.1833069	0.3275300	0.1834823
PLOD1	0.2635327	0.1954561	0.1514192	0.4320072	0.1720427
ENSG00000287384	0.0000000	0.0000000	0.0011671	0.0000000	0.0055850
MFN2	0.2604596	0.3047927	0.2714880	0.2189985	0.2816434
MIIP	0.1405635	0.1708258	0.1771866	0.1526245	0.1699181
TNFRSF8	0.0000000	0.0000000	0.0026961	0.0000000	0.0000000
TNFRSF1B	0.0011529	0.0000000	0.0000000	0.0000000	0.0000000
VPS13D	0.3119706	0.2579142	0.1964571	0.2855274	0.6175353
LINC02766	0.0000000	0.0027789	0.0000000	0.0000000	0.0000000
DHRS3	0.3460888	0.2452259	0.1194387	0.3463225	0.1788529
ENSG00000288927	0.0180547	0.0097493	0.0031640	0.0095992	0.0044508
ENSG00000272482	0.0195793	0.0193512	0.0068650	0.0283323	0.0156762
CFAP107	0.1383793	0.0761558	0.0752464	0.1518958	0.0589716
LRRC38	0.0014020	0.0024214	0.0000000	0.0022040	0.0048432
PDPN	0.1320230	0.0788268	0.0670616	0.2483408	0.0648531
PRDM2	0.4012091	0.4977270	0.4867154	0.3517160	0.6349427
ENSG00000289380	0.0022949	0.0021013	0.0000000	0.0022040	0.0097237
KAZN	0.6698175	0.6877291	0.7019406	0.6978230	1.6542516
ENSG00000231606	0.0000000	0.0053792	0.0040754	0.0057525	0.0113249
KAZN-AS1	0.0000000	0.0014407	0.0018236	0.0000000	0.0153541
ENSG00000287756	0.0000000	0.0000000	0.0000000	0.0000000	0.0015072
TMEM51-AS1	0.0085963	0.0119955	0.0094906	0.0113451	0.0254426
TMEM51	0.0142675	0.0181261	0.0183606	0.0134481	0.0237676
FHAD1	0.1869594	0.1198468	0.0729588	0.1668616	0.0997511
FHAD1-AS1	0.0066375	0.0070790	0.0019906	0.0099894	0.0105263
EFHD2-AS1	0.0019850	0.0018293	0.0052006	0.0106277	0.0000000
EFHD2	0.3282560	0.2572250	0.2352316	0.5081213	0.1760333
CTRC	0.0000000	0.0000000	0.0000000	0.0000000	0.0042172
CELA2A	0.0000000	0.0000000	0.0008981	0.0022963	0.0000000
CELA2B	0.0015226	0.0010918	0.0019603	0.0041312	0.0069462
CASP9	0.0740754	0.0619354	0.0748923	0.0727613	0.0599492
DNAJC16	0.1525960	0.1382634	0.1422814	0.1752613	0.1864511
AGMAT	0.0106819	0.0141724	0.0124605	0.0029645	0.0109200
ENSG00000237301	0.0044009	0.0042787	0.0040533	0.0000000	0.0028046
DDI2	0.0527251	0.0800491	0.0768848	0.0594996	0.1003120
PLEKHM2	0.2980163	0.3116442	0.2860894	0.2749843	0.2283687
ENSG00000237938	0.0011153	0.0015017	0.0000000	0.0000000	0.0070919
SLC25A34	0.0039793	0.0028463	0.0033655	0.0000000	0.0073921
TMEM82	0.0010824	0.0020594	0.0000000	0.0000000	0.0000000
FBLIM1	0.0367949	0.0622551	0.0564975	0.0292098	0.1043181
UQCRHL	0.0193263	0.0262101	0.0121368	0.0289014	0.0400718
SPEN-AS1	0.1068372	0.0829688	0.0820339	0.1125130	0.0604225
SPEN	0.4898077	0.5683637	0.5458205	0.4244893	0.7795929
ZBTB17	0.0859059	0.0915414	0.0714163	0.0578489	0.1315328
HSPB7	0.0111970	0.0077289	0.0000000	0.0000000	0.0145680
CLCNKA	0.0002116	0.0028132	0.0015113	0.0000000	0.0000000
CLCNKB	0.0032197	0.0000000	0.0000000	0.0000000	0.0000000
FAM131C	0.0669000	0.1540343	0.1626792	0.0525746	0.1138850
EPHA2	0.0034555	0.0069718	0.0016702	0.0098223	0.0000000
ARHGEF19	0.0048502	0.0105684	0.0055043	0.0050458	0.0157376
ENSG00000288398	0.0303798	0.0453046	0.0459122	0.0504127	0.0249255
CPLANE2	0.0208561	0.0240242	0.0070574	0.0213564	0.0102477
FBXO42	0.1900046	0.1983253	0.2045901	0.1939542	0.4390943

SZRD1	0.2964877	0.2565070	0.2448254	0.2975563	0.2813026
SPATA21	0.0085921	0.0081472	0.0030550	0.0034975	0.0106374
NECAP2	0.2927336	0.2146321	0.1585739	0.2993718	0.1572333
LINC01772	0.0003340	0.0044751	0.0048392	0.0000000	0.0049684
CROCCP3	0.0499636	0.0488285	0.0524001	0.0479715	0.1860143
ENSG00000261135	0.0409230	0.0882249	0.1302129	0.0499291	0.0758025
ENSG00000224174	0.0008980	0.0012294	0.0048607	0.0000000	0.0000000
LINC01783	0.0000000	0.0006935	0.0046303	0.0042516	0.0104941
NBPF1	0.3455835	0.3409317	0.4059141	0.3030112	0.3277192
ENSG00000271732	0.0018299	0.0077934	0.0019501	0.0024744	0.0000000
CROCCP2	0.1874682	0.1610557	0.1837965	0.1461917	0.2572109
CROCC	0.0662848	0.0878936	0.0909853	0.1200603	0.1971815
ENSG00000290850	0.0020270	0.0014091	0.0000000	0.0000000	0.0031722
ENSG00000290851	0.0034042	0.0019143	0.0000000	0.0000000	0.0000000
ENSG00000228549	0.0008172	0.0000000	0.0022218	0.0059690	0.0000000
ENSG00000238142	0.1216128	0.1238939	0.1295597	0.1529857	0.1418727
ENSG00000272426	0.0033597	0.0019556	0.0012574	0.0012567	0.0085325
ENSG00000290122	0.0315228	0.0382709	0.0208750	0.0264062	0.0689500
MFAP2	0.2430096	0.1209279	0.1470128	0.1947571	0.1750732
ENSG00000226526	0.0049193	0.0000000	0.0000000	0.0000000	0.0159505
ATP13A2	0.1424602	0.2501615	0.3086133	0.1831874	0.2858974
SDHB	0.5075514	0.5785160	0.5168559	0.4814203	0.4281637
PADI2	0.0037155	0.0005680	0.0142432	0.0000000	0.0105466
RCC2	0.1945494	0.1662687	0.1071583	0.1775460	0.1493111
ARHGEF10L	0.0657861	0.0941449	0.0792873	0.0728975	0.2769537
LINC02810	0.0000000	0.0000000	0.0023309	0.0000000	0.0017997
ACTL8	0.0008777	0.0000000	0.0000000	0.0000000	0.0000000
IGSF21	0.2074408	0.3968006	0.5641652	0.1461261	0.3838876
IGSF21-AS1	0.0000000	0.0011317	0.0000000	0.0000000	0.0000000
ENSG00000225387	0.0000000	0.0008116	0.0000000	0.0000000	0.0017223
PAX7	0.0910243	0.0410763	0.0402541	0.0622760	0.0364326
ALDH4A1	0.0561750	0.0318170	0.0311543	0.1067760	0.0490904
IFFO2	0.0853027	0.0687446	0.0769060	0.1035429	0.1124039
ENSG00000272084	0.0000000	0.0030328	0.0000000	0.0000000	0.0000000
UBR4	0.2309039	0.2446212	0.2484662	0.1866653	0.4538955
EMC1-AS1	0.0760603	0.0481903	0.0399881	0.1154194	0.0825102
EMC1	0.1727529	0.1114061	0.1496544	0.3171735	0.1131312
MRTO4	0.2936784	0.3005383	0.3976615	0.3715726	0.2352354
AKR7L	0.0005096	0.0028897	0.0065799	0.0023531	0.0015072
AKR7A3	0.0077939	0.0076620	0.0090940	0.0083946	0.0054999
AKR7A2	0.5363254	0.3832237	0.3319087	0.5498569	0.2988211
SLC66A1	0.0709577	0.0382823	0.0444493	0.0632960	0.0852519
CAPZB	1.2009684	1.1384877	1.1073889	1.2859512	1.0535983
MICOS10	1.1129419	1.1716865	1.1248640	1.1886266	0.9094564
MICOS10-DT	0.0000000	0.0000000	0.0000000	0.0000000	0.0000000
NBL1	0.1625715	0.2827273	0.3510406	0.1992041	0.1851148
HTR6	0.0020503	0.0025565	0.0020297	0.0000000	0.0129234
TMCO4	0.0241457	0.0162755	0.0043241	0.0313206	0.0131864
OTUD3	0.0846304	0.1174491	0.0999228	0.0839532	0.0906562
PLA2G5	0.0000000	0.0010809	0.0000000	0.0016082	0.0000000
PLA2G2C	0.0411605	0.0415019	0.0204024	0.0453725	0.0249430
UBXN10	0.1060908	0.0575277	0.0462836	0.1187796	0.0654564
VWA5B1	0.0201276	0.0173620	0.0221241	0.0168342	0.0262760

ENSG00000226664	0.0000000	0.0000000	0.0000000	0.0000000	0.0000000
LINC01141	0.0000000	0.0000000	0.0023257	0.0000000	0.0060333
CAMK2N1	1.6703352	2.2305285	2.4106074	1.4623244	1.8138129
MUL1	0.1150371	0.1025915	0.0936093	0.1302978	0.0867813
FAM43B	0.0350582	0.0919991	0.1312492	0.0409210	0.1050875
CDA	0.0008295	0.0000000	0.0000000	0.0000000	0.0000000
PINK1	0.4549438	0.5607000	0.6326826	0.4185773	0.4259128
PINK1-AS	0.0311868	0.0223079	0.0272025	0.0616263	0.0342630
DDOST	0.5321781	0.3526244	0.3545297	0.7438037	0.3261801
KIF17	0.0492104	0.0427973	0.0619682	0.0621037	0.0660597
SH2D5	0.0070602	0.0133191	0.0145306	0.0000000	0.0228180
HP1BP3	1.0456026	1.0094218	1.0940962	1.0643643	0.9903589
EIF4G3	0.8203593	0.8871424	0.9305392	0.7816801	1.3993541
ENSG00000287192	0.0018677	0.0008262	0.0015372	0.0000000	0.0000000
ECE1	0.1822071	0.1621403	0.1101125	0.2049986	0.1692825
ENSG00000236936	0.0030178	0.0030831	0.0000000	0.0000000	0.0064609
ECE1-AS1	0.0002143	0.0000000	0.0000000	0.0000000	0.0045117
NBPF3	0.0535130	0.0521595	0.0318170	0.0522038	0.0944578
ALPL	0.0089865	0.0132724	0.0049844	0.0098598	0.0078414
LINC02596	0.0000000	0.0000000	0.0000000	0.0000000	0.0000000
RAP1GAP	0.1467744	0.1391940	0.1856951	0.0675932	0.2067324
USP48	0.5139811	0.5084083	0.5285716	0.4865225	0.5467339
LDLRAD2	0.0013088	0.0004049	0.0015287	0.0082229	0.0000000
HSPG2	0.0232321	0.0187141	0.0118262	0.0580233	0.0127122
LINC01635	0.0020640	0.0000000	0.0009597	0.0000000	0.0000000
LINC00339	0.1466658	0.1606971	0.1596277	0.1477383	0.1208778
CDC42-AS1	0.0060061	0.0040306	0.0074567	0.0000000	0.0110543
CDC42	2.0135749	2.0747992	2.2449149	1.9941301	1.7683788
CDC42-IT1	0.0000000	0.0000000	0.0000000	0.0000000	0.0068352
WNT4	0.2025092	0.2475188	0.3267830	0.2184753	0.2066627
ENSG00000285873	0.0069080	0.0089525	0.0033628	0.0014624	0.0150257
ZBTB40	0.0995494	0.0794481	0.0623181	0.0921055	0.0738245
EPHA8	0.0382292	0.0951741	0.1530684	0.0464325	0.0885349
EPHB2	0.1988484	0.2517914	0.2434550	0.2741524	0.4301479
TEX46	0.0087510	0.0010395	0.0058623	0.0046542	0.0046818
KDM1A	0.8031368	0.8511838	0.9035978	0.8260040	0.8786661
ENSG00000240553	0.0079972	0.0081775	0.0049164	0.0035673	0.0194971
LUZP1	0.1857796	0.1901304	0.2127298	0.1738815	0.3069943
HTR1D	0.0259811	0.0092840	0.0093714	0.0223565	0.0142490
LINC01355	0.0016757	0.0080522	0.0043046	0.0000000	0.0105361
ENSG00000284726	0.0004993	0.0015274	0.0000000	0.0000000	0.0000000
HNRNPR	1.2353523	1.2038595	1.3600821	1.2142047	1.0665722
ZNF436	0.0747566	0.1019561	0.1031153	0.0976793	0.0556843
ZNF436-AS1	0.3821685	0.3184112	0.2946745	0.3265143	0.2525379
ENSG00000271420	0.0032727	0.0014183	0.0000000	0.0041902	0.0000000
TCEA3	0.4229312	0.2619052	0.1984605	0.3586523	0.2325421
ASAP3	0.1154375	0.0658444	0.0618623	0.1004386	0.1025250
E2F2	0.0000000	0.0000000	0.0000000	0.0000000	0.0000000
ENSG00000235052	0.0032984	0.0034625	0.0053122	0.0000000	0.0053413
ID3	1.2093856	0.7535316	0.5593438	1.4857714	0.7240868
MDS2	0.0000000	0.0000000	0.0000000	0.0038925	0.0000000
RPL11	3.0221224	2.9971158	2.8205010	2.9394477	2.7846872
ELOA-AS1	0.1002765	0.0923043	0.1023976	0.0649813	0.1087899

ELOA	0.3060211	0.2735427	0.2954074	0.3302188	0.2714821
PITHD1	0.5799800	0.6369430	0.8780658	0.6046942	0.4759469
ENSG00000289835	0.0708152	0.0432904	0.0204087	0.0574174	0.0226563
LYPLA2	0.3602677	0.3250712	0.3042421	0.4034172	0.2614506
GALE	0.1126387	0.1422432	0.2163939	0.1068684	0.1272241
HMGCL	0.2415953	0.1317975	0.1244981	0.2682926	0.1538099
FUCA1	0.2034237	0.1559304	0.2293675	0.2911650	0.1688610
CNR2	0.0000000	0.0000000	0.0014895	0.0000000	0.0000000
PNRC2	0.4993894	0.4132920	0.4319517	0.5373842	0.3814838
SRSF10	0.6739953	0.5954934	0.5716157	0.6700869	0.6276750
MYOM3	0.0012829	0.0000000	0.0050637	0.0000000	0.0000000
IL22RA1	0.0000000	0.0000000	0.0026175	0.0000000	0.0000000
IFNLR1	0.0053441	0.0096975	0.0171166	0.0023000	0.0389066
GRHL3	0.0406039	0.0152490	0.0135068	0.0517704	0.0302710
STPG1	0.2373707	0.2547042	0.2589316	0.1904340	0.2276104
NIPAL3	0.2387808	0.2528746	0.2476593	0.1941417	0.2398747
ENSG00000288982	0.0208954	0.0104069	0.0102719	0.0012382	0.0046212
RCAN3AS	0.0119759	0.0025645	0.0103000	0.0084519	0.0189048
RCAN3	0.4091692	0.5952778	0.6764319	0.3834769	0.4951238
NCMAP-DT	0.0451196	0.0775557	0.0554527	0.0423676	0.0429026
NCMAP	0.0085168	0.0075356	0.0054789	0.0070192	0.0059619
SRRM1	1.1328238	0.9626482	0.9145901	1.1786499	0.9922040
CLIC4	0.5303129	0.4436655	0.3665555	0.4830859	0.4712377
SYF2	0.5799316	0.5048802	0.4786778	0.5696401	0.3937262
ENSG00000284602	0.0031807	0.0041016	0.0000000	0.0045811	0.0342252
ENSG00000284657	0.0090242	0.0079839	0.0042727	0.0023540	0.0000000
RSRP1	0.3395562	0.3035040	0.2311098	0.3084311	0.3171669
ENSG00000272432	0.0048690	0.0051789	0.0012612	0.0059759	0.0016619
RHD	0.0003054	0.0016718	0.0000000	0.0000000	0.0023834
TMEM50A	0.6717609	0.6412109	0.6752769	0.7915765	0.6280820
RHCE	0.0304918	0.0221004	0.0183426	0.0222098	0.0548476
MACO1	0.2854637	0.3516010	0.3662845	0.2482371	0.5422203
LDLRAP1	0.0245228	0.0137395	0.0072476	0.0140529	0.0147537
MAN1C1	0.0612476	0.0639358	0.0301234	0.0832029	0.1264842
ENSG00000233478	0.0000000	0.0000000	0.0000000	0.0000000	0.0000000
SELENON	0.1315825	0.0751227	0.0605628	0.1507304	0.0768505
ENSG00000228172	0.0088140	0.0008451	0.0043273	0.0133959	0.0000000
MTFR1L	0.3325558	0.3751817	0.3730236	0.3562546	0.3057037
AUNIP	0.0002181	0.0022072	0.0050981	0.0067455	0.0067503
PAQR7	0.0649259	0.0870191	0.0778233	0.0586910	0.1209096
STMN1	2.0240131	2.6851172	3.2731763	1.9629701	2.2707182
PAFAH2	0.0475241	0.0435988	0.0248603	0.0332854	0.0324751
EXTL1	0.0031547	0.0076375	0.0028352	0.0000000	0.0000000
SLC30A2	0.0077311	0.0012793	0.0000000	0.0044097	0.0109376
PDIK1L	0.1988578	0.3199027	0.4686725	0.1760276	0.2638139
FAM110D	0.0038414	0.0096333	0.0247015	0.0000000	0.0101813
C1orf232	0.0011369	0.0020926	0.0046129	0.0000000	0.0119019
ZNF593	0.2135646	0.1626487	0.1626726	0.2215240	0.1105477
CNKSR1	0.0008209	0.0078597	0.0088359	0.0053863	0.0210722
CATSPER4	0.0000000	0.0000000	0.0000000	0.0000000	0.0000000
CEP85	0.0386671	0.0590155	0.0619307	0.0464516	0.0547767
SH3BGRL3	0.8585767	1.0041268	1.0678192	0.9323385	0.7466813
UBXN11	0.2705352	0.1793747	0.1470416	0.2295210	0.2090445

CRYBG2	0.0196898	0.0302368	0.0349851	0.0174614	0.0251329
LIN28A	0.0020278	0.0055365	0.0031944	0.0000000	0.0220163
DHDDS	0.1402607	0.1679290	0.1578469	0.1326092	0.1919312
DHDDS-AS1	0.0005488	0.0000000	0.0000000	0.0000000	0.0000000
HMGN2	1.8395972	1.6683776	1.7917699	1.7688450	1.5046547
RPS6KA1	0.0145619	0.0233636	0.0156463	0.0117678	0.0265618
ENSG00000260063	0.0024428	0.0014183	0.0000000	0.0000000	0.0000000
ARID1A	0.4114074	0.4664646	0.4283732	0.4057459	0.6274853
PIGV	0.0610046	0.0503294	0.0311845	0.1440525	0.0406790
ZDHHC18	0.0563201	0.1205679	0.0944568	0.0821684	0.1155089
SFN	0.0000000	0.0004303	0.0000000	0.0000000	0.0000000
GPN2	0.1042592	0.1036272	0.0926456	0.0831714	0.1249100
ENSG00000289554	0.0018161	0.0005833	0.0022111	0.0000000	0.0000000
GPATCH3	0.0460457	0.0382632	0.0182544	0.0448687	0.0377866
NUDC	1.2588815	1.1396855	1.1122380	1.2317643	1.0036084
KDF1	0.0002570	0.0010426	0.0000000	0.0000000	0.0000000
TRNP1	0.3573811	0.4227427	0.4054705	0.3736110	0.2509145
TENT5B	0.0051166	0.0000000	0.0000000	0.0143388	0.0000000
SLC9A1	0.0497767	0.0356854	0.0577268	0.0214577	0.0330439
WDTC1	0.1943732	0.1871543	0.1380095	0.1993123	0.1956352
TMEM222	0.1737917	0.1715398	0.1677023	0.2037830	0.1275722
SYTL1	0.0139226	0.0079288	0.0025549	0.0103577	0.0116313
MAP3K6	0.0199061	0.0285582	0.0144726	0.0174700	0.0082982
FCN3	0.0010687	0.0014160	0.0000000	0.0000000	0.0000000
CD164L2	0.0049793	0.0009394	0.0016648	0.0000000	0.0066722
GPR3	0.0041988	0.0135360	0.0148135	0.0105123	0.0028456
WASF2	1.1144148	0.7331754	0.5192720	0.9753233	0.6582730
ENSG00000241169	0.0000000	0.0000000	0.0000000	0.0000000	0.0000000
ENSG00000237429	0.0059798	0.0024015	0.0006785	0.0036275	0.0062626
AHDC1	0.2286592	0.2050281	0.1912710	0.2101368	0.2215142
IFI6	0.0865492	0.0664342	0.0427001	0.1643478	0.0554783
ENSG00000287244	0.0011721	0.0000000	0.0000000	0.0055660	0.0000000
FAM76A	0.2654850	0.2244117	0.1200717	0.2692971	0.1899246
STX12	0.6956868	0.7754541	0.8503310	0.6372005	0.5717790
ENSG00000269971	0.0000000	0.0000000	0.0000000	0.0000000	0.0000000
ENSG00000270031	0.0043478	0.0034605	0.0015141	0.0041277	0.0000000
ENSG00000286433	0.0021189	0.0000000	0.0000000	0.0000000	0.0000000
PPP1R8	0.2284951	0.1933915	0.1694279	0.2751706	0.2108668
THEMIS2	0.0119433	0.0193106	0.0140797	0.0090360	0.0053917
RPA2	0.2149376	0.1966634	0.2016220	0.2650701	0.1856732
SMPDL3B	0.0000000	0.0013626	0.0000000	0.0000000	0.0000000
ENSG00000227050	0.0027852	0.0000000	0.0077414	0.0000000	0.0072786
XKR8	0.0424081	0.0178429	0.0066941	0.0642790	0.0295815
EYA3	0.1176657	0.1309751	0.1164291	0.1424984	0.2804346
PTAFR	0.0018612	0.0000000	0.0006660	0.0017819	0.0081851
DNAJC8	1.1812598	1.1835158	1.3613068	1.1664588	0.9463187
ENSG00000290123	0.0008372	0.0000000	0.0000000	0.0047839	0.0111812
ATP5IF1	1.4519522	1.5846632	1.7566116	1.4013728	1.2164561
ENSG00000270605	0.0025966	0.0013552	0.0053324	0.0000000	0.0000000
SESN2	0.3144959	0.2299526	0.1883831	0.2250618	0.2368245
MED18	0.0956185	0.0886318	0.1082249	0.1097066	0.0557362
PHACTR4	0.3971739	0.3890219	0.3487330	0.3613426	0.5307650
RCC1	0.0855617	0.0790104	0.0856279	0.0448481	0.0856775

SNHG3	0.1110929	0.1093922	0.0893079	0.1229291	0.0979131
TRNAU1AP	0.1550083	0.1905499	0.1850716	0.1541416	0.1873848
SNHG12	0.1243686	0.1593681	0.1408134	0.1016374	0.1069014
TAF12	0.2739625	0.2784300	0.2940414	0.2673431	0.2924041
RAB42	0.0191535	0.0050941	0.0011435	0.0079279	0.0383203
TAF12-DT	0.0062118	0.0081877	0.0088444	0.0010830	0.0142080
ENSG00000270103	0.0091686	0.0062724	0.0000000	0.0064298	0.0296223
GMEB1	0.1173127	0.1247306	0.1119527	0.0897596	0.1477341
ENSG00000289291	0.0061744	0.0018824	0.0050946	0.0120435	0.0000000
YTHDF2	0.5126939	0.4841778	0.5767064	0.5910510	0.4505608
OPRD1	0.0065912	0.0048684	0.0019497	0.0039751	0.0159510
ENSG00000233427	0.0012279	0.0024907	0.0000000	0.0055417	0.0000000
EPB41	0.3021305	0.4909914	0.5600775	0.2430502	0.7279158
TMEM200B	0.0000000	0.0035754	0.0009765	0.0046536	0.0000000
ENSG00000225750	0.0000000	0.0000000	0.0000000	0.0000000	0.0000000
SRSF4	0.9165008	0.8300072	0.8851413	0.8422989	0.7902751
MECR	0.1417071	0.1593268	0.1532675	0.1750496	0.1287760
PTPRU	0.0644101	0.0964069	0.1183664	0.0866049	0.1894199
LINC01756	0.0004094	0.0000000	0.0000000	0.0000000	0.0057693
LINC01648	0.0000000	0.0000000	0.0000000	0.0000000	0.0000000
MATN1	0.0068081	0.0048208	0.0072307	0.0000000	0.0097083
MATN1-AS1	0.0093576	0.0070352	0.0072900	0.0050346	0.0054053
LAPTM5	0.0023905	0.0023241	0.0000000	0.0000000	0.0000000
ENSG00000229607	0.0008204	0.0000000	0.0000000	0.0000000	0.0000000
LINC01778	0.0024781	0.0074869	0.0046685	0.0047294	0.0000000
SDC3	0.4117409	0.5195556	0.6396625	0.3649958	0.5602623
PUM1	0.6925793	0.7093483	0.6990012	0.6926081	0.8159249
NKAIN1	0.1762014	0.3762760	0.4003521	0.1660399	0.3553643
SNRNP40	0.3009617	0.2787352	0.3395542	0.2552613	0.3009081
ZCCHC17	0.6875276	0.7448738	0.8795642	0.6411774	0.6014379
ENSG00000229044	0.0014089	0.0000000	0.0014534	0.0000000	0.0167762
FABP3	0.0685981	0.1401065	0.1218137	0.0940620	0.0681794
SERINC2	0.0397777	0.0499824	0.0261429	0.0382989	0.0687393
LINC01226	0.0000000	0.0002825	0.0000000	0.0000000	0.0072896
HCRTR1	0.0027971	0.0004488	0.0000000	0.0029316	0.0083610
PEF1	0.4983344	0.3990604	0.3832052	0.5335709	0.3100302
PEF1-AS1	0.0000000	0.0032966	0.0000000	0.0000000	0.0000000
ENSG00000264078	0.0000000	0.0015421	0.0000000	0.0000000	0.0000000
COL16A1	0.0639323	0.0486353	0.0330300	0.0979315	0.0649445
ADGRB2	0.1262284	0.1297903	0.1196364	0.1850981	0.1543814
SPOCD1	0.0031715	0.0000000	0.0000000	0.0018211	0.0000000
ENSG00000269967	0.0008506	0.0000000	0.0000000	0.0000000	0.0000000
PTP4A2	1.1680585	1.1023367	1.1783908	1.0736807	0.9838558
ENSG00000228634	0.0009932	0.0000000	0.0000000	0.0000000	0.0070110
KHDRBS1	1.2206576	1.2657764	1.3928271	1.2077574	1.1449433
ENSG00000203325	0.0013778	0.0013944	0.0000000	0.0039003	0.0090554
TMEM39B	0.1098744	0.0732216	0.0783366	0.0956166	0.1232363
KPNA6	0.4222505	0.3775032	0.3979481	0.4501934	0.3772971
ENSG00000250135	0.0006597	0.0008290	0.0000000	0.0000000	0.0000000
TXLNA	0.2886210	0.1837088	0.1996823	0.2932584	0.1954384
CCDC28B	0.4543700	0.4984552	0.5736391	0.4419680	0.3389974
ENSG00000224066	0.0019833	0.0008803	0.0000000	0.0000000	0.0058756
IQCC	0.0411613	0.0272247	0.0332227	0.0466777	0.0297928

DCDC2B	0.0034538	0.0044070	0.0000000	0.0031392	0.0039284
TMEM234	0.1097950	0.0744321	0.1233319	0.1227837	0.0798229
EIF3I	0.8265697	0.8096956	0.8345447	0.8328564	0.5585601
ENSG00000291132	0.0317055	0.0238621	0.0150717	0.0262607	0.0142707
ENSG00000290045	0.0011366	0.0013478	0.0000000	0.0030907	0.0034576
FAM167B	0.0024737	0.0045469	0.0048774	0.0000000	0.0000000
HDAC1	0.2495087	0.1554021	0.1480430	0.3345300	0.1865575
MARCKSL1	1.9815557	2.2818200	2.6011583	1.9899089	1.8452281
TSSK3	0.0029668	0.0014241	0.0019096	0.0046988	0.0000000
FAM229A	0.2523922	0.1986853	0.2938623	0.2348051	0.1734971
BSDC1	0.3076902	0.3009289	0.2892927	0.3218468	0.3145630
ZBTB8B	0.0628578	0.1103441	0.1684829	0.0601145	0.1582958
ZBTB8A	0.1498803	0.2105505	0.3050637	0.1319234	0.2376071
ZBTB8OS	0.2985091	0.3301242	0.2622165	0.2807715	0.3277888
RBBP4	1.0034896	0.8791747	0.9147993	0.9630417	0.7704102
SYNC	0.3694948	0.2704732	0.2228704	0.4404039	0.3083463
KIAA1522	0.2144659	0.2085321	0.1855324	0.2190277	0.1659772
YARS1	0.8379648	0.8786582	1.0003541	0.8177594	0.7888764
S100PBP	0.2309072	0.2563543	0.3445103	0.2730229	0.2868271
FNDC5	0.0878834	0.1372658	0.1274464	0.0865849	0.1026559
HPCA	0.0818705	0.1900441	0.2781830	0.0433449	0.0854257
TMEM54	0.1011081	0.1341805	0.1056765	0.0830567	0.0468887
ENSG00000287691	0.0028361	0.0014812	0.0011178	0.0060336	0.0117372
RNF19B	0.0738613	0.1048157	0.1246000	0.0502364	0.1392243
ENSG00000236065	0.0006600	0.0038913	0.0000000	0.0000000	0.0250513
AK2	0.3574849	0.2942797	0.2666987	0.3633007	0.2796149
AZIN2	0.0384765	0.0488703	0.0586439	0.0541158	0.0423097
TRIM62	0.0278490	0.0358345	0.0459221	0.0298052	0.0641406
ENSG00000284721	0.0000000	0.0014953	0.0000000	0.0000000	0.0042064
ZNF362	0.2268277	0.2974432	0.2922424	0.1662581	0.2809283
A3GALT2	0.0000000	0.0000000	0.0000000	0.0019580	0.0046262
ENSG00000225313	0.0019001	0.0032550	0.0032495	0.0018326	0.0166361
PHC2	0.3290195	0.2843488	0.2665791	0.3708470	0.3407705
PHC2-AS1	0.0009241	0.0000000	0.0000000	0.0000000	0.0101850
ZSCAN20	0.0236548	0.0212950	0.0304609	0.0167904	0.0277848
CSMD2	0.1087006	0.1405603	0.1306441	0.1016479	0.7030667
CSMD2-AS1	0.0000000	0.0000000	0.0000000	0.0000000	0.0074370
C1orf94	0.0029272	0.0045362	0.0038382	0.0103514	0.0057461
ENSG00000287703	0.0000000	0.0000000	0.0000000	0.0000000	0.0000000
SMIM12	0.1417869	0.1778357	0.1139465	0.1473305	0.1249900
DLGAP3	0.0265672	0.0750301	0.0918187	0.0248887	0.1219837
TMEM35B	0.1036873	0.0459517	0.0459944	0.2539237	0.0612206
ZMYM6	0.1906512	0.1822330	0.1916150	0.2261526	0.2876303
ZMYM1	0.2346174	0.1701431	0.1989258	0.2365935	0.2154527
ENSG00000284640	0.0012740	0.0008158	0.0000000	0.0000000	0.0039740
SFPQ	0.8454504	0.9662503	0.9945424	0.8952382	1.1956083
ZMYM4	0.8066774	0.7614023	0.8446365	0.8459397	0.8347547
ZMYM4-AS1	0.0018762	0.0022535	0.0017904	0.0000000	0.0195602
KIAA0319L	0.3898924	0.3390748	0.3232291	0.5130835	0.4999015
NCDN	0.1176469	0.1882112	0.2352638	0.1308741	0.0879863
TFAP2E-AS1	0.0035450	0.0155706	0.0230467	0.0000000	0.0043204
TFAP2E	0.0065330	0.0089695	0.0008783	0.0124098	0.0125268
PSMB2	1.0402286	0.9771750	0.9999503	1.0914156	0.7627194

C1orf216	0.1604058	0.3499485	0.4029595	0.1572118	0.1823009
CLSPN	0.0465146	0.0248412	0.0440490	0.0389284	0.0414409
AGO4	0.2053829	0.2476427	0.2668757	0.1919109	0.3834323
AGO1	0.2366084	0.1957079	0.1530639	0.2078889	0.2861628
ENSG00000286899	0.0000000	0.0000000	0.0000000	0.0000000	0.0000000
AGO3	0.3459011	0.3641276	0.3196178	0.3351490	0.5541213
ENSG00000271554	0.0042972	0.0045576	0.0000000	0.0045480	0.0208431
TEKT2	0.1574094	0.1022113	0.0712654	0.1276370	0.0960923
ADPRS	0.2922584	0.2492957	0.2181874	0.3034196	0.1772912
COL8A2	0.0018998	0.0031374	0.0000000	0.0000000	0.0000000
TRAPPC3	0.4286787	0.3838777	0.4174560	0.4526067	0.3011513
MAP7D1	0.2760622	0.3459680	0.3466757	0.2917628	0.3205115
THRAP3	0.8672409	0.8011003	0.8445674	0.8751696	0.7267653
SH3D21	0.0667287	0.0674534	0.0568603	0.0555640	0.1444115
EVA1B	0.2907584	0.1782965	0.0858896	0.3391539	0.1448351
ENSG00000286379	0.0023567	0.0032447	0.0028743	0.0035629	0.0019259
STK40	0.1913194	0.1722986	0.1745252	0.1206325	0.2583594
LSM10	0.4016029	0.3789450	0.3790146	0.3700504	0.2704726
OSCP1	0.1959905	0.1324507	0.0888218	0.2250270	0.1418758
MRPS15	0.3811927	0.4893277	0.4803492	0.3925262	0.3614551
GRIK3	0.0868573	0.1000599	0.1179778	0.0585213	0.5203715
ENSG00000284650	0.0011320	0.0017428	0.0000000	0.0047417	0.0000000
ENSG00000223944	0.0008858	0.0000000	0.0000000	0.0000000	0.0045817
ZC3H12A-DT	0.0247846	0.0070466	0.0127986	0.0264630	0.0351772
ZC3H12A	0.0078976	0.0010611	0.0050138	0.0099534	0.0000000
MEAF6	1.0087759	1.2842370	1.4814653	0.9571763	0.9779599
SNIP1	0.1853630	0.1770638	0.1684970	0.2003228	0.1699737
DNALI1	0.5872476	0.4104161	0.3293083	0.6509608	0.3939052
GNL2	0.4303438	0.4225440	0.3645528	0.4349084	0.3136102
ENSG00000284748	0.0007037	0.0013877	0.0000000	0.0000000	0.0058801
RSPO1	0.0033412	0.0071710	0.0059374	0.0000000	0.0276517
C1orf109	0.1511354	0.0932722	0.0954863	0.0800744	0.0623021
CDCA8	0.0146851	0.0100294	0.0094978	0.0078017	0.0039795
EPHA10	0.0183653	0.0459356	0.0581410	0.0279743	0.1150475
MANEAL	0.0977727	0.1702937	0.1801554	0.1153940	0.1198814
YRDC	0.1222093	0.1237193	0.1192943	0.1674966	0.1122581
C1orf122	0.9674168	0.9430433	0.9771731	1.0019094	0.7715204
MTF1	0.1145750	0.0810376	0.0956175	0.1412370	0.1532901
INPP5B	0.1001848	0.0627038	0.0509659	0.1044339	0.1601433
SF3A3	0.4745279	0.4103037	0.4205934	0.4955537	0.3643332
FHL3	0.0497967	0.0468078	0.0748840	0.0310134	0.0480713
UTP11	0.4060894	0.3845969	0.4321554	0.3352973	0.2678523
POU3F1	0.0277110	0.0688921	0.0353966	0.0338453	0.0563129
MIR3659HG	0.0082686	0.0053285	0.0000000	0.0073563	0.0321698
ENSG00000286552	0.0000000	0.0000000	0.0000000	0.0000000	0.0000000
ENSG00000287987	0.0005919	0.0012471	0.0000000	0.0000000	0.0000000
ENSG00000284632	0.0000000	0.0000000	0.0000000	0.0000000	0.0000000
RRAGC	0.3548761	0.2838980	0.2220968	0.4460682	0.2676726
RRAGC-DT	0.0576243	0.0802395	0.0516211	0.0177498	0.2520480
MYCBP	0.1816953	0.1500036	0.0822872	0.2271965	0.1216932
GJA9	0.0000000	0.0000000	0.0000000	0.0000000	0.0036228
RHBDL2	0.0000000	0.0027892	0.0000000	0.0000000	0.0095958
AKIRIN1	0.8067034	0.8382335	0.9080131	0.8355046	0.6236202

NDUFS5	1.5500090	1.6402807	1.6836637	1.5490637	1.3525228
MACF1	0.9534776	0.9978538	0.9801209	0.8771246	1.6318755
ENSG00000287422	0.0007592	0.0000000	0.0000000	0.0000000	0.0014498
ENSG00000226438	0.0000000	0.0009146	0.0000000	0.0000000	0.0035216
BMP8A	0.0065805	0.0089426	0.0125632	0.0042547	0.0194008
PPIEL	0.0391511	0.0360713	0.0590365	0.0194009	0.1020579
PABPC4	0.7180954	0.7770031	0.8500340	0.6756979	0.6467766
PABPC4-AS1	0.0009247	0.0015472	0.0000000	0.0000000	0.0000000
HEYL	0.0015763	0.0033352	0.0000000	0.0048845	0.0000000
NT5C1A	0.0163903	0.0476551	0.0486106	0.0123976	0.0226040
HPCAL4	0.2723482	0.5120715	0.8123641	0.2459860	0.3964834
PPIE	0.3864698	0.3042756	0.2526399	0.3634520	0.2374674
BMP8B	0.0051952	0.0128147	0.0135699	0.0000000	0.0206099
OXCT2	0.0018976	0.0012598	0.0090369	0.0000000	0.0000000
ENSG00000284719	0.0024423	0.0023946	0.0009802	0.0000000	0.0000000
LINC02811	0.0000000	0.0024387	0.0000000	0.0050767	0.0030476
TRIT1	0.1208262	0.1330143	0.1188198	0.1025888	0.2636933
MYCL	0.0374140	0.0512875	0.0544211	0.0236806	0.0730840
MYCL-AS1	0.0005615	0.0000000	0.0000000	0.0000000	0.0014245
MFSD2A	0.0864827	0.0490301	0.0442844	0.1272319	0.0394687
CAP1	0.8644685	0.8760019	0.8289736	0.9423118	0.6517010
PPT1	0.3315470	0.2803969	0.2732919	0.6029914	0.2085482
RLF	0.4862808	0.4919076	0.5123702	0.3940243	0.7280876
TMCO2	0.0048513	0.0000000	0.0000000	0.0000000	0.0000000
ZMPSTE24-DT	0.1176271	0.1137642	0.1905061	0.0976598	0.0733550
ZMPSTE24	0.1700076	0.1427292	0.1485498	0.2534453	0.1373714
COL9A2	0.0067163	0.0046877	0.0065725	0.0011506	0.0088830
SMAP2	0.5465967	0.7537864	0.9088439	0.5020935	0.6665757
ENSG00000284677	0.0036989	0.0066598	0.0081632	0.0042617	0.0153168
ZFP69B	0.0602063	0.0970490	0.1621434	0.0360380	0.0883518
ENSG00000260920	0.0137434	0.0278139	0.0361624	0.0099682	0.0475867
ZFP69	0.0318774	0.0375419	0.0845994	0.0347727	0.0560211
EXO5-DT	0.0174879	0.0203098	0.0099661	0.0257805	0.0321561
EXO5	0.0288229	0.0327651	0.0295208	0.0278104	0.0108749
ENSG00000238186	0.0009386	0.0022901	0.0000000	0.0019780	0.0000000
ZNF684	0.0587865	0.0407091	0.0374859	0.0510267	0.0357706
ENSG00000286838	0.0029312	0.0011745	0.0000000	0.0000000	0.0097547
RIMS3	0.1235838	0.1985095	0.2596998	0.1162300	0.1927624
ENSG00000287743	0.0005480	0.0008530	0.0021786	0.0000000	0.0000000
ENSG00000237899	0.0076472	0.0091976	0.0100332	0.0071661	0.0143232
NFYC-AS1	0.0036022	0.0032563	0.0044328	0.0017571	0.0108196
NFYC	0.2480350	0.2624346	0.2058366	0.2574305	0.2039614
KCNQ4	0.0124731	0.0010401	0.0035795	0.0179202	0.0069878
CITED4	0.1592098	0.2169353	0.2478733	0.1909953	0.1260360
ENSG00000229528	0.0000000	0.0000000	0.0000000	0.0000000	0.0000000
CTPS1	0.0732135	0.1135689	0.1565330	0.0731840	0.1521466
SLFNL1-AS1	0.0078311	0.0000000	0.0008184	0.0000000	0.0065609
SLFNL1	0.0003083	0.0000000	0.0013273	0.0000000	0.0086405
SCMH1	0.2643023	0.3623219	0.3327373	0.2697452	0.7761529
ENSG00000287400	0.0009615	0.0012634	0.0000000	0.0000000	0.0000000
SCMH1-DT	0.0002926	0.0003577	0.0041206	0.0023342	0.0037728
ENSG00000291157	0.0000000	0.0000000	0.0046432	0.0000000	0.0000000
FOXO6	0.0201924	0.0374475	0.0502095	0.0052973	0.0355278

FOXO6-AS1	0.0000000	0.0000000	0.0000000	0.0000000	0.0000000
EDN2	0.0038237	0.0000000	0.0000000	0.0067484	0.0000000
HIVEP3	0.1878386	0.2213890	0.1731545	0.1159793	0.8379715
ENSG00000287587	0.0033476	0.0035747	0.0075707	0.0000000	0.0000000
FOXJ3	0.4004103	0.4200084	0.3912077	0.2826988	0.5560811
RIMKLA	0.2565196	0.3115217	0.3190131	0.2368987	0.2286644
ZMYND12	0.0534633	0.0348833	0.0240928	0.0208944	0.0208255
PPCS	0.6660330	0.6192864	0.6714982	0.6329341	0.4476580
CCDC30	0.4991283	0.4168310	0.3956551	0.4850397	0.6056656
PPIH	0.2005277	0.2101701	0.3048039	0.2129955	0.1546854
ENSG00000285728	0.0126254	0.0187536	0.0039942	0.0000000	0.0172737
ENSG00000234917	0.0104858	0.0180982	0.0063480	0.0010526	0.0120474
YBX1	3.3476609	3.5516963	3.4096933	3.2544041	3.2061720
CLDN19	0.0029782	0.0000000	0.0000000	0.0000000	0.0000000
P3H1	0.1870491	0.1154719	0.1563285	0.3287200	0.1580482
C1orf50	0.2469134	0.2406612	0.3010092	0.2331352	0.1793415
TMEM269	0.0132777	0.0095025	0.0110075	0.0038033	0.0000000
SVBP	0.8178720	0.8861960	0.9927816	0.7365815	0.7396549
ERMAP	0.0921900	0.0770241	0.0323897	0.1447572	0.0731542
ZNF691-DT	0.0143987	0.0027177	0.0027572	0.0100900	0.0037415
ZNF691	0.0627195	0.0588990	0.0251238	0.0495000	0.0394212
SLC2A1	0.3718715	0.2975951	0.2016252	0.3287338	0.2567029
SLC2A1-DT	0.0333430	0.0360221	0.0214404	0.0278724	0.0250912
CFAP144	0.4314378	0.2769772	0.2201213	0.4214180	0.2447210
EBNA1BP2	0.6090372	0.5349534	0.5988630	0.5903704	0.4240043
CFAP57	0.0755008	0.0399020	0.0211562	0.1026427	0.0209458
TMEM125	0.0039884	0.0088951	0.0038850	0.0127412	0.0034327
TIE1	0.0024639	0.0021018	0.0000000	0.0000000	0.0000000
MPL	0.0180729	0.0078543	0.0070448	0.0075741	0.0129657
CDC20-DT	0.0068798	0.0053194	0.0015338	0.0105681	0.0126187
CDC20	0.0199683	0.0139406	0.0158179	0.0141313	0.0148576
ELOVL1	0.0627597	0.0542541	0.0107992	0.0884476	0.0392040
ENSG00000288772	0.0084583	0.0258715	0.0109876	0.0184178	0.0024068
MED8	0.2681230	0.2481440	0.3248789	0.2373149	0.1881249
SZT2	0.0525595	0.0494327	0.0295723	0.0364904	0.1301665
HYI	0.2078654	0.1856131	0.1740252	0.2008295	0.1223891
HYI-AS1	0.0007803	0.0025684	0.0017132	0.0000000	0.0036537
PTPRF	0.4324265	0.3710019	0.3419560	0.5271233	0.6080070
KDM4A	0.2865435	0.2871517	0.2765747	0.2414790	0.3056295
KDM4A-AS1	0.0092893	0.0113510	0.0089767	0.0288133	0.0160033
ST3GAL3	0.1388098	0.2089393	0.2249624	0.2297112	0.4405153
ST3GAL3-AS1	0.0002980	0.0056155	0.0000000	0.0040956	0.0074199
ARTN	0.0037300	0.0000000	0.0072038	0.0088883	0.0000000
LINC02918	0.0019584	0.0017833	0.0063783	0.0041277	0.0065007
ENSG00000288573	0.0082577	0.0065060	0.0023562	0.0062726	0.0046843
IPO13	0.0682326	0.0467901	0.0609583	0.0769199	0.0671388
ENSG00000285649	0.0019348	0.0023881	0.0031803	0.0012897	0.0000000
DPH2	0.0425036	0.0288992	0.0290273	0.0487551	0.0186705
ATP6V0B	1.0426940	1.1349464	1.3441857	1.1160855	0.9356028
B4GALT2	0.2549942	0.3284883	0.3387055	0.2305761	0.3108069
CCDC24	0.1056050	0.0930762	0.1247803	0.1443374	0.1607302
SLC6A9	0.0575542	0.0540514	0.0695195	0.0324432	0.0817000
ENSG00000230615	0.1018318	0.1054982	0.0993480	0.1575737	0.0686210

DMAP1	0.2488569	0.2038805	0.1842499	0.1994875	0.1536141
ERI3	0.4220635	0.4019555	0.4398280	0.4672528	0.3511806
RNF220	0.3001812	0.2842511	0.3480587	0.2854410	0.5746517
TMEM53	0.1787669	0.1121867	0.1344162	0.1823354	0.0670284
ARMH1	0.0478115	0.0326121	0.0216813	0.0401041	0.1081718
KIF2C	0.0063651	0.0087390	0.0092036	0.0047879	0.0032941
ENSG00000225721	0.0045935	0.0083127	0.0078020	0.0039330	0.0167729
RPS8	3.9721673	3.9850064	3.8165334	3.8728625	3.6930735
BEST4	0.0210811	0.0207806	0.0166210	0.0103997	0.0270550
PLK3	0.1039009	0.0938730	0.0705103	0.0623835	0.0859455
DYNLT4	0.0141029	0.0123333	0.0023834	0.0133494	0.0110471
BTBD19	0.0146906	0.0095584	0.0132912	0.0134019	0.0299569
PTCH2	0.0246867	0.0217789	0.0175308	0.0436674	0.0746720
EIF2B3	0.3015643	0.3471677	0.3360993	0.2584166	0.3689782
HECTD3	0.0425425	0.0341049	0.0351034	0.0476343	0.0513250
UROD	0.2973551	0.2502651	0.1862678	0.2853556	0.1802534
ZSWIM5	0.1379966	0.2088916	0.1841363	0.1332248	0.6102609
LINC01144	0.0067889	0.0049201	0.0015401	0.0086947	0.0054053
HPDL	0.0024212	0.0047490	0.0101150	0.0091010	0.0000000
MUTYH	0.0773358	0.0609014	0.0485932	0.0988788	0.0624834
TOE1	0.0631456	0.0589201	0.0377365	0.0480603	0.0541336
TESK2	0.0239812	0.0099560	0.0083551	0.0212868	0.0060265
CCDC163	0.0121910	0.0065440	0.0035067	0.0159584	0.0023258
MMACHC	0.0369626	0.0302860	0.0367706	0.0458375	0.0541221
PRDX1	1.7459498	1.4551938	1.3613415	1.9129897	1.2101060
AKR1A1	0.4284629	0.4212973	0.4328161	0.4602555	0.2535492
ENSG00000289407	0.0190076	0.0091401	0.0247025	0.0073775	0.0117866
NASP	0.5307180	0.5576416	0.5648322	0.4837257	0.5452644
CCDC17	0.0000000	0.0021783	0.0000000	0.0000000	0.0062535
GPBP1L1	0.3512612	0.2599194	0.2352873	0.2966318	0.3267499
TMEM69	0.1635397	0.1636443	0.1628111	0.1502053	0.1555381
IPP	0.2201615	0.2031048	0.1403948	0.2468893	0.0997409
ENSG00000230896	0.0128198	0.0085827	0.0086866	0.0063076	0.0000000
MAST2	0.1972547	0.2837616	0.2848001	0.1491046	0.6384655
PIK3R3	0.2844220	0.5083117	0.5971663	0.2994840	0.5385818
ENSG00000226957	0.0000000	0.0007508	0.0000000	0.0000000	0.0095653
ENSG00000227857	0.0199880	0.0081823	0.0055707	0.0053774	0.0235370
TSPAN1	0.0025443	0.0070683	0.0008246	0.0000000	0.0000000
POMGNT1	0.1993951	0.1540321	0.1558805	0.3006847	0.1930982
LURAP1	0.0114843	0.0223748	0.0138184	0.0108296	0.0213818
RAD54L	0.0041932	0.0034887	0.0013791	0.0000000	0.0017206
LRRC41	0.3816760	0.3447693	0.2887535	0.3983072	0.2987902
UQCRH	1.6560202	1.8596246	1.8631626	1.5987585	1.5436449
NSUN4	0.0534147	0.0632759	0.0377784	0.0286838	0.0378846
FAAH	0.0262703	0.0332214	0.0426785	0.0132454	0.0552940
ENSG00000291138	0.0013275	0.0014527	0.0067953	0.0000000	0.0000000
TMEM275	0.0003255	0.0038899	0.0021664	0.0000000	0.0000000
MKNK1-AS1	0.0009011	0.0000000	0.0021936	0.0000000	0.0019899
KNCN	0.0059032	0.0011842	0.0017982	0.0000000	0.0065199
MKNK1	0.0445802	0.0551896	0.0432130	0.0387809	0.0531804
MOB3C	0.0039605	0.0024535	0.0088797	0.0146036	0.0022680
ATPAF1	0.5227973	0.5210800	0.4928930	0.5438932	0.4001724
TEX38	0.0011785	0.0000000	0.0000000	0.0000000	0.0000000

EFCAB14-AS1	0.0000000	0.0000000	0.0000000	0.0000000	0.0015531
EFCAB14	0.3809032	0.2898524	0.2414445	0.4243882	0.2852096
CYP4Z2P	0.0000000	0.0000000	0.0041961	0.0027694	0.0067752
CYP4A11	0.0040467	0.0128147	0.0175881	0.0085194	0.0128017
CYP4X1	0.0499645	0.1127583	0.1401651	0.0625453	0.1945505
CYP4Z1	0.0000000	0.0069034	0.0000000	0.0000000	0.0206177
CYP4A22-AS1	0.0000000	0.0000000	0.0000000	0.0000000	0.0014580
CYP4A22	0.0000000	0.0000000	0.0000000	0.0000000	0.0000000
LINC00853	0.0004184	0.0018229	0.0011329	0.0019281	0.0000000
STIL	0.0198751	0.0211195	0.0058092	0.0110771	0.0324165
CMPK1	0.8446289	0.9876223	1.0498522	0.7491122	0.7681202
LINC01389	0.0013939	0.0000000	0.0000000	0.0000000	0.0079335
FOXE3	0.0020062	0.0003461	0.0015009	0.0000000	0.0000000
TRABD2B	0.4694829	0.2467429	0.1837713	0.4455155	0.2525187
ENSG00000225028	0.0019726	0.0038916	0.0029608	0.0000000	0.0000000
LINC02794	0.0014423	0.0014288	0.0000000	0.0017461	0.0000000
ENSG00000290466	0.0012783	0.0049085	0.0000000	0.0000000	0.0000000
ENSG00000291246	0.0000000	0.0000000	0.0000000	0.0000000	0.0034716
ENSG00000223720	0.0019878	0.0000000	0.0000000	0.0000000	0.0000000
SLC5A9	0.0000000	0.0000000	0.0000000	0.0030907	0.0033094
SPATA6	0.6314883	0.4469819	0.2772807	0.7119137	0.6227130
AGBL4	0.1918774	0.1935994	0.2065229	0.1145048	0.9839748
BEND5	0.2963368	0.4766469	0.5751334	0.2932284	0.3592777
ENSG00000286597	0.0000000	0.0012926	0.0000000	0.0000000	0.0000000
ENSG00000229846	0.0010536	0.0056669	0.0000000	0.0000000	0.0247999
AGBL4-AS1	0.0000000	0.0000000	0.0000000	0.0000000	0.0000000
ELAVL4	0.5520048	1.0244781	1.1917648	0.4347387	0.9761805
ELAVL4-AS1	0.0009319	0.0000000	0.0000000	0.0000000	0.0014580
ENSG00000233407	0.0000000	0.0000000	0.0000000	0.0000000	0.0000000
LINC02808	0.0000000	0.0000000	0.0027683	0.0000000	0.0031282
DMRTA2	0.0026227	0.0008606	0.0000000	0.0000000	0.0000000
FAF1	0.4098024	0.4653467	0.4406411	0.3585191	0.8757837
FAF1-AS1	0.0015920	0.0000000	0.0000000	0.0000000	0.0035216
CDKN2C	0.0142096	0.0158506	0.0014595	0.0026302	0.0000000
LINC01562	0.0087105	0.0115639	0.0133936	0.0080491	0.0129393
RNF11	0.9296436	0.9756029	1.1247035	0.8616087	0.7740713
ENSG00000236434	0.0012196	0.0000000	0.0000000	0.0000000	0.0026787
TTC39A	0.0322608	0.0448356	0.0665801	0.0257657	0.0279917
TTC39A-AS1	0.0059753	0.0000000	0.0000000	0.0000000	0.0039758
EPS15	0.9250115	1.1428660	1.2685427	0.7803065	0.9580783
EPS15-AS1	0.0026621	0.0052054	0.0000000	0.0000000	0.0000000
OSBPL9	0.5325008	0.4950695	0.4674354	0.4482519	0.6841960
NRDC	0.9123458	0.8331990	0.8305288	0.8847077	0.8034635
ENSG00000266993	0.0000000	0.0000000	0.0028883	0.0000000	0.0000000
ENSG00000272175	0.0015098	0.0022694	0.0000000	0.0000000	0.0000000
RAB3B	0.4273104	0.8823046	1.0706460	0.4046296	0.5889982
TXNDC12	0.4137300	0.2896917	0.2932806	0.4570706	0.3027005
KTI12	0.0630519	0.0397942	0.0456939	0.0672223	0.0447298
ENSG00000223390	0.0038747	0.0025349	0.0000000	0.0000000	0.0163830
BTF3L4	0.9523936	1.0854175	1.2500820	0.8863095	0.8603822
ZFYVE9	0.3822978	0.4470041	0.4394212	0.3178764	0.5061800
CC2D1B	0.0519908	0.0573844	0.0146262	0.0587037	0.0464875
ENSG00000272100	0.0006545	0.0014218	0.0017990	0.0000000	0.0000000

ENSG00000287078	0.0004480	0.0074563	0.0000000	0.0000000	0.0139896
ORC1	0.0003364	0.0033421	0.0043549	0.0071101	0.0203863
PRPF38A	0.3796969	0.3125010	0.2763952	0.4210976	0.1696675
TUT4	0.7886331	0.8241054	0.7804226	0.7262162	1.1529277
ENSG00000272371	0.0004182	0.0000000	0.0000000	0.0000000	0.0000000
GPX7	0.3273403	0.1935995	0.1556207	0.3455337	0.1781905
SHISAL2A	0.0170596	0.0431772	0.0366179	0.0107616	0.0197772
COA7	0.1270774	0.2409062	0.2229183	0.1020430	0.1126709
ZYG11B	0.4495900	0.6265060	0.8001359	0.4140472	0.6643515
ZYG11A	0.0016776	0.0024756	0.0044053	0.0042672	0.0000000
ECHDC2	0.1071292	0.0763621	0.0706927	0.1311907	0.0682085
SCP2	1.0849569	0.8703531	0.7816761	1.1322697	0.7356288
PODN	0.0007686	0.0043193	0.0000000	0.0000000	0.0099129
CPT2	0.1480364	0.1131319	0.0906641	0.1888601	0.0816345
ENSG00000236723	0.0083064	0.0100361	0.0054753	0.0056997	0.0221960
CZIB	0.4079169	0.3390918	0.2594857	0.4071682	0.2568752
CZIB-DT	0.0010423	0.0000000	0.0050110	0.0000000	0.0000000
MAGOH	0.4551992	0.3552126	0.3363365	0.4551794	0.3333055
MAGOH-DT	0.0212760	0.0170524	0.0168125	0.0102603	0.0195570
LRP8	0.1584463	0.1625430	0.1951240	0.2839169	0.2889050
LRP8-DT	0.0131639	0.0232623	0.0197517	0.0071976	0.0107350
ENSG00000285954	0.0003688	0.0008366	0.0000000	0.0000000	0.0000000
GLIS1	0.0243544	0.0153567	0.0143185	0.0138866	0.0894121
NDC1	0.0644713	0.0518566	0.0467605	0.0499036	0.1077035
YIPF1	0.1859136	0.1414171	0.1539830	0.1163913	0.1656260
DIO1	0.0045431	0.0015489	0.0040367	0.0000000	0.0000000
IFT25	0.4161204	0.4731798	0.5107137	0.5157085	0.3969942
LRRC42	0.2194892	0.1608058	0.1297544	0.2360782	0.1620221
TMEM59	1.1692378	0.9505048	0.9329508	1.3984977	0.9301722
TCEANC2	0.1461118	0.1231538	0.0773222	0.0943368	0.1220008
CDCP2	0.0071263	0.0050045	0.0052445	0.0000000	0.0000000
CYB5RL	0.0403024	0.0377436	0.0232346	0.0368788	0.0373333
MRPL37	0.3617599	0.2955971	0.3089533	0.4114967	0.2627480
SSBP3	0.6957648	0.8066427	0.9155184	0.5672928	1.0208702
SSBP3-AS1	0.0000000	0.0000000	0.0015699	0.0021832	0.0000000
ACOT11	0.0123638	0.0127878	0.0057032	0.0058134	0.0303127
FAM151A	0.0000000	0.0000000	0.0000000	0.0000000	0.0000000
MROH7	0.0099470	0.0069926	0.0061494	0.0101333	0.0484192
TTC4	0.2446085	0.1912393	0.2069511	0.1906549	0.2107826
PARS2	0.0317862	0.0200741	0.0333289	0.0481212	0.0396693
TTC22	0.0000000	0.0000000	0.0000000	0.0012567	0.0045452
LEXM	0.0000000	0.0000000	0.0000000	0.0000000	0.0000000
DHCR24	0.0863495	0.1422665	0.1555966	0.1159637	0.0584998
ENSG00000287724	0.0018250	0.0000000	0.0000000	0.0000000	0.0000000
ENSG00000242396	0.0017975	0.0000000	0.0026545	0.0000000	0.0049667
DHCR24-DT	0.0009129	0.0000000	0.0000000	0.0000000	0.0000000
TMEM61	0.0000000	0.0014625	0.0000000	0.0055470	0.0000000
PCSK9	0.0003724	0.0000000	0.0011718	0.0000000	0.0000000
USP24	0.2560756	0.2579329	0.2579801	0.1954734	0.5011919
MIR4422HG	0.0058786	0.0023791	0.0000000	0.0075074	0.0000000
ENSG00000234810	0.0020129	0.0000000	0.0000000	0.0000000	0.0000000
ENSG00000260971	0.0036657	0.0024687	0.0032218	0.0044963	0.0183778
PLPP3	0.2308833	0.1528284	0.1247025	0.5971990	0.2269817

PRKAA2	0.2493656	0.2963223	0.3230324	0.2605717	0.4190051
FYB2	0.0000000	0.0000000	0.0000000	0.0000000	0.0000000
C8B	0.0024063	0.0019141	0.0000000	0.0000000	0.0000000
ENSG00000236341	0.0000000	0.0008884	0.0000000	0.0000000	0.0000000
DAB1	0.4897786	0.5062693	0.4108683	0.4974625	1.1970995
DAB1-AS1	0.0000000	0.0000000	0.0000000	0.0000000	0.0000000
ENSG00000290536	0.0000000	0.0034004	0.0000000	0.0000000	0.0017997
ENSG00000235038	0.0205152	0.0093529	0.0000000	0.0398811	0.0120615
OMA1	0.1166514	0.0494704	0.0368892	0.1404005	0.0517301
ENSG00000286918	0.0000000	0.0021647	0.0000000	0.0024360	0.0000000
MYSM1	0.1370646	0.1820090	0.2640801	0.1445781	0.3346693
ENSG00000283445	0.0000000	0.0020377	0.0000000	0.0000000	0.0000000
JUN	1.1268425	0.9804736	0.6438052	0.9568559	0.9826006
LINC01135	0.0125705	0.0096089	0.0039450	0.0038709	0.0319933
LINC02777	0.0169449	0.0075730	0.0027481	0.0219482	0.0286906
LINC01358	0.0130068	0.0051969	0.0060395	0.0074627	0.0000000
FGGY-DT	0.0111063	0.0087486	0.0203188	0.0210299	0.0128015
ENSG00000270457	0.0008763	0.0015372	0.0000000	0.0000000	0.0000000
FGGY	0.1320851	0.1307697	0.1035795	0.1620336	0.2429430
ENSG00000226883	0.0003435	0.0000000	0.0000000	0.0044571	0.0000000
HOOK1	0.1250988	0.1369420	0.1971257	0.0927955	0.2466787
CYP2J2	0.0008698	0.0025331	0.0000000	0.0000000	0.0000000
C1orf87	0.0235463	0.0069743	0.0000000	0.0391955	0.0025761
LINC01748	0.0107927	0.0135155	0.0053370	0.0041811	0.0088910
ENSG00000231252	0.0062108	0.0116309	0.0040912	0.0096982	0.0075249
NFIA	1.1071795	0.8442405	0.5131664	1.2741866	1.0003647
NFIA-AS2	0.0842307	0.0596351	0.0350488	0.0683845	0.0577138
NFIA-AS1	0.0025388	0.0010205	0.0000000	0.0000000	0.0000000
ENSG00000284808	0.0011907	0.0000000	0.0000000	0.0000000	0.0034730
ENSG00000287224	0.0009960	0.0018103	0.0026494	0.0000000	0.0046437
TM2D1	0.7231013	0.5886239	0.5618340	0.8382337	0.5775417
PATJ-DT	0.0073749	0.0140887	0.0065163	0.0069173	0.0051767
PATJ	0.2982071	0.2816516	0.2324784	0.3215320	0.5257846
L1TD1	0.0010823	0.0000000	0.0000000	0.0000000	0.0043359
KANK4	0.0072864	0.0128859	0.0372740	0.0098444	0.0187451
USP1	0.4593394	0.3232551	0.3248942	0.4673761	0.2324103
DOCK7	0.7209102	0.6656221	0.5994935	0.7400820	0.9548609
ANGPTL3	0.0000000	0.0000000	0.0000000	0.0000000	0.0000000
DOCK7-DT	0.0161374	0.0229554	0.0175049	0.0128039	0.0193035
ATG4C	0.1630579	0.1627450	0.1667720	0.1780114	0.2179877
LINC00466	0.0011952	0.0000000	0.0000000	0.0032590	0.0000000
FOXD3-AS1	0.0050242	0.0050732	0.0134877	0.0035916	0.0117410
FOXD3	0.0000000	0.0019463	0.0016797	0.0000000	0.0088497
ALG6	0.0673673	0.0869880	0.0748572	0.0612613	0.1263370
ITGB3BP	0.1947186	0.1733359	0.1853675	0.1431593	0.1949387
ENSG00000286429	0.0012124	0.0011635	0.0000000	0.0000000	0.0083117
EFCAB7	0.2363274	0.2404109	0.2343419	0.2248362	0.2308131
DLEU2L	0.0035479	0.0041864	0.0043633	0.0020682	0.0230534
PGM1	0.3080541	0.2170574	0.2092113	0.3351252	0.2738950
ROR1	0.0474599	0.0409779	0.0258636	0.0357714	0.1697789
ROR1-AS1	0.0010499	0.0007236	0.0022052	0.0000000	0.0000000
UBE2U	0.0000000	0.0017343	0.0000000	0.0000000	0.0047864
CACHD1	0.1604631	0.1667112	0.1912364	0.2789695	0.3549873

RAVER2	0.1123078	0.0544458	0.0562881	0.0854987	0.1347448
JAK1	0.8481817	0.8523382	0.8735923	0.8274475	0.8105383
LINC01359	0.0006199	0.0000000	0.0023402	0.0000000	0.0000000
ENSG00000288804	0.0007092	0.0014699	0.0000000	0.0029052	0.0031339
AK4	0.7433253	0.5881552	0.5354257	0.6884495	0.5893116
DNAJC6	0.3272088	0.5709387	0.6996181	0.2865589	0.7758179
ENSG00000229294	0.0051976	0.0035482	0.0013569	0.0000000	0.0361976
ENSG00000290094	0.0011237	0.0000000	0.0040652	0.0000000	0.0174092
LEPROT	0.4884303	0.2884295	0.2730559	0.6134662	0.2665668
LEPR	0.1344915	0.0955423	0.1170758	0.1564838	0.2389218
ENSG00000237852	0.0037748	0.0000000	0.0030686	0.0029843	0.0100009
PDE4B	0.5350831	0.4661949	0.4967091	0.5339368	1.1905148
PDE4B-AS1	0.0000000	0.0000000	0.0000000	0.0000000	0.0000000
SGIP1	0.3844789	0.6430781	0.8363623	0.3286835	1.1713777
ENSG00000248458	0.0007207	0.0017679	0.0000000	0.0000000	0.0042625
DYNLT5	0.7158804	0.4228143	0.2836964	0.8532082	0.4530079
INSL5	0.0009331	0.0000000	0.0000000	0.0000000	0.0000000
DNAI4	0.3555749	0.2695250	0.2656471	0.3473298	0.3386200
MIER1	0.5032955	0.4666204	0.4763724	0.4787399	0.4571765
ENSG00000289394	0.0010682	0.0019683	0.0034305	0.0000000	0.0000000
SLC35D1	0.0924027	0.1090675	0.1097949	0.0847031	0.2093476
C1orf141	0.0027673	0.0000000	0.0017857	0.0000000	0.0000000
ENSG00000275678	0.0000000	0.0000000	0.0000000	0.0000000	0.0000000
IL12RB2	0.0025910	0.0066712	0.0025586	0.0000000	0.0188833
SERBP1	1.7426954	1.6150750	1.6233145	1.7105723	1.3642552
GADD45A	0.4596747	0.3218891	0.2925675	0.3883171	0.3146494
GNG12	0.7221229	0.4207593	0.2933022	0.9320852	0.4303574
GNG12-AS1	0.0448568	0.0207406	0.0166520	0.0555502	0.0461448
DIRAS3	0.1085129	0.1787459	0.4876700	0.0958204	0.1536956
WLS	1.3461441	0.8154590	0.5834303	1.8787263	0.9432819
RPE65	0.0147734	0.0042468	0.0000000	0.0627545	0.0000000
DEPDC1	0.0090152	0.0066443	0.0000000	0.0124598	0.0000000
ENSG00000285407	0.0028435	0.0000000	0.0000000	0.0000000	0.0032778
ENSG00000285473	0.0000000	0.0007729	0.0000000	0.0000000	0.0092940
LINC01707	0.0021507	0.0025125	0.0000000	0.0000000	0.0000000
LRRC7	0.3686650	0.4980388	0.4926547	0.3067822	1.1836099
LRRC7-AS1	0.0010237	0.0000000	0.0000000	0.0000000	0.0200194
LRRC40	0.2361730	0.3181489	0.4055858	0.2126669	0.4132068
SRSF11	1.0678994	1.0088256	1.0653929	1.0768975	1.0579381
ANKRD13C	0.3916300	0.3949866	0.3653043	0.3667067	0.4359984
HHLA3	0.2190507	0.1633147	0.1438032	0.2684150	0.1169573
CTH	0.0626654	0.0504749	0.0538730	0.0671999	0.0661975
ENSG00000271992	0.0046400	0.0050648	0.0049742	0.0045443	0.0000000
PTGER3	0.3502345	0.3449585	0.2402809	0.3971677	0.2281738
ZRANB2-AS1	0.0066210	0.0101207	0.0079631	0.0168496	0.0000000
ZRANB2	1.2485764	1.0868490	1.1426120	1.2352626	1.0120979
ZRANB2-DT	0.0849737	0.0801561	0.0606267	0.0946095	0.2840343
NEGR1	0.4882323	0.6482222	0.6618540	0.4456462	1.5494476
ENSG00000231985	0.0036346	0.0051441	0.0050963	0.0027988	0.0034590
ENSG00000286863	0.0946727	0.0816311	0.0689629	0.0670892	0.3237501
ENSG00000225087	0.0043848	0.0011833	0.0057707	0.0000000	0.0100120
LINC02796	0.0045146	0.0038904	0.0000000	0.0000000	0.0064756
LINC02797	0.0007932	0.0021647	0.0065675	0.0011055	0.0106192

LINC02238	0.0000000	0.0000000	0.0018100	0.0000000	0.0095606
ENSG00000285778	0.0000000	0.0011604	0.0011178	0.0000000	0.0000000
LRRIQ3	0.0195166	0.0186795	0.0137230	0.0065776	0.0162815
FPGT	0.1421768	0.1006876	0.0497675	0.1247678	0.1412897
TNNI3K	0.0655967	0.0663210	0.0290176	0.0611733	0.1633862
ENSG00000237324	0.0000000	0.0000000	0.0000000	0.0000000	0.0000000
ENSG00000233894	0.0000000	0.0000000	0.0026026	0.0000000	0.0000000
LRRC53	0.0008557	0.0025026	0.0000000	0.0000000	0.0000000
ERICH3	0.2330256	0.1819458	0.2122490	0.1901739	0.1994887
ERICH3-AS1	0.0007984	0.0000000	0.0000000	0.0025834	0.0045380
ENSG00000272864	0.0038510	0.0055568	0.0026665	0.0020988	0.0030362
CRYZ	0.4419262	0.2306996	0.1248740	0.4296454	0.1445676
TYW3	0.3158387	0.2043525	0.1292063	0.2238805	0.1925523
SLC44A5	0.1664137	0.2678701	0.1585582	0.1294733	0.9406659
ACADM	0.6331070	0.5391538	0.4497022	0.6215614	0.4316757
RABGGTB	0.5690581	0.5620596	0.4722007	0.5371044	0.3980198
MSH4	0.0182711	0.0094574	0.0099562	0.0046603	0.0462081
ENSG00000225605	0.0000000	0.0000000	0.0000000	0.0000000	0.0059744
ST6GALNAC3	0.5352561	0.3590491	0.2616225	0.7178459	0.6758345
ENSG00000272855	0.0030685	0.0019770	0.0000000	0.0017320	0.0000000
ST6GALNAC5	0.1405185	0.1755467	0.1918307	0.1367540	0.5508704
ENSG00000288543	0.0121311	0.0072571	0.0090085	0.0000000	0.0046038
ENSG00000230498	0.0006714	0.0079837	0.0060115	0.0000000	0.0031863
PIGK	0.1937585	0.1690515	0.1736172	0.2508605	0.1624217
ENSG00000289212	0.0097487	0.0110807	0.0067744	0.0042662	0.0000000
ENSG00000287870	0.0219260	0.0231559	0.0175908	0.0040245	0.0042647
AK5	0.3251143	0.3860747	0.6015395	0.3034098	0.3889000
ZZZ3	0.4315973	0.4046306	0.4112021	0.4122779	0.5079332
USP33	0.5397647	0.6059150	0.6903523	0.5131944	0.6930247
MIGA1	0.3317500	0.3902407	0.3522399	0.4461054	0.3981693
NEXN-AS1	0.0014328	0.0054114	0.0018021	0.0054322	0.0127957
NEXN	0.1222786	0.0500508	0.0152151	0.0904033	0.0752658
FUBP1	0.8513258	0.7880470	0.7791907	0.7811624	0.7909783
DNAJB4	0.7775246	0.5467479	0.4208408	0.7324160	0.4981743
GIPC2	0.1294049	0.1367981	0.1099383	0.1337333	0.1453484
ENSG00000273338	0.0169778	0.0093303	0.0115060	0.0097179	0.0228714
ENSG00000285928	0.0002461	0.0000000	0.0000000	0.0000000	0.0000000
MGC27382	0.0006189	0.0000000	0.0000000	0.0000000	0.0000000
PTGFR	0.0090440	0.0324251	0.0110827	0.0159811	0.0398765
IFI44L	0.0011187	0.0008645	0.0000000	0.0000000	0.0044301
IFI44	0.0164963	0.0180465	0.0116892	0.0290894	0.0495938
ADGRL4	0.0029630	0.0051257	0.0000000	0.0000000	0.0175980
ADGRL2	0.5120248	0.3644257	0.2579333	0.5356215	0.5294324
ENSG00000234953	0.0000000	0.0000000	0.0000000	0.0000000	0.0000000
ENSG00000236676	0.0014430	0.0024170	0.0000000	0.0041811	0.0000000
ENSG00000233290	0.0000000	0.0036396	0.0000000	0.0061785	0.0000000
LINC01362	0.0004139	0.0000000	0.0000000	0.0000000	0.0065864
LINC01361	0.0000000	0.0000000	0.0000000	0.0000000	0.0000000
LINC01725	0.0046991	0.0075602	0.0059341	0.0027381	0.0418913
TTLL7	0.3996494	0.5225168	0.4585643	0.3520183	0.7404921
TTLL7-IT1	0.0047306	0.0014160	0.0000000	0.0000000	0.0072209
PRKACB-DT	0.0188992	0.0262258	0.0168764	0.0200678	0.0021357
PRKACB	0.7818618	1.0966618	1.2109126	0.7016531	1.1991275

SAMD13	0.0378658	0.0522322	0.0154830	0.0505752	0.0355611
DNASE2B	0.0013391	0.0003752	0.0000000	0.0034132	0.0000000
RPF1	0.2438886	0.2164211	0.1785664	0.1961063	0.1763498
GNG5	1.1913021	0.8439966	0.6156106	1.1702033	0.7859566
SPATA1	0.0125423	0.0135475	0.0063909	0.0220178	0.0052477
CTBS	0.2295159	0.1019439	0.0860656	0.2162011	0.1367522
ENSG00000284882	0.0014173	0.0013211	0.0008773	0.0000000	0.0000000
ENSG00000289881	0.0000000	0.0034478	0.0051724	0.0015536	0.0080063
LINC01555	0.0017117	0.0000000	0.0000000	0.0000000	0.0000000
SSX2IP	0.1631666	0.1493084	0.1610273	0.1302380	0.2404637
MCOLN2	0.0000000	0.0000000	0.0000000	0.0000000	0.0000000
DNAI3	0.4298519	0.1822255	0.1040643	0.3962483	0.2045829
MCOLN3	0.0039306	0.0058168	0.0000000	0.0021653	0.0030106
SYDE2	0.1393342	0.1618440	0.1512312	0.1622056	0.1358370
C1orf52	0.4245400	0.4851196	0.6261831	0.3758652	0.4192441
BCL10	0.2724870	0.2829213	0.2070463	0.2704819	0.1805070
BCL10-AS1	0.0229480	0.0340432	0.0214372	0.0310119	0.0396270
DDAH1	0.5728856	0.7660029	0.5219268	0.6540391	0.6960406
ENSG00000282057	0.0242285	0.0066903	0.0084125	0.0199967	0.0193431
CCN1	0.0881093	0.0877569	0.0613319	0.1305366	0.1181485
ZNHIT6	0.3436705	0.3518085	0.2841705	0.3747442	0.3498534
COL24A1	0.0411448	0.0544442	0.0392954	0.0158138	0.1173771
LINC02795	0.0068009	0.0187587	0.0008492	0.0088957	0.0174056
ODF2L	0.6902028	0.6906397	0.6819603	0.6794287	0.7509865
CLCA4-AS1	0.0222316	0.0274364	0.0316996	0.0410614	0.0391491
SH3GLB1	1.2821905	1.3433093	1.3122127	1.3382983	1.1173973
ENSG00000284846	0.0005494	0.0014749	0.0000000	0.0000000	0.0000000
SELENOF	0.6003342	0.5344381	0.5901492	0.8486393	0.4909779
HS2ST1	0.4373950	0.3250264	0.2842213	0.5762510	0.5088639
ENSG00000267734	0.0000000	0.0020412	0.0000000	0.0000000	0.0000000
LINC01140	0.0102430	0.0089579	0.0079816	0.0045753	0.0040567
LMO4	1.0360125	1.2764637	1.2404316	0.8813219	0.9026617
LINC01364	0.0000000	0.0008286	0.0000000	0.0041513	0.0000000
PKN2-AS1	0.0303702	0.0200309	0.0431997	0.0027265	0.0547405
ENSG00000286758	0.0016536	0.0041318	0.0108408	0.0000000	0.0016673
PKN2	0.4506984	0.3380704	0.2753543	0.4540001	0.6333738
GTF2B	0.2932045	0.2748144	0.2192012	0.2907388	0.2825219
KYAT3	0.2051726	0.2403120	0.2617761	0.1922445	0.2009181
RBMXL1	0.1982088	0.2488009	0.2586632	0.1777663	0.1795061
GBP3	0.0038430	0.0021864	0.0000000	0.0000000	0.0000000
ENSG00000286802	0.0000000	0.0000000	0.0000000	0.0000000	0.0071630
GBP4	0.0025767	0.0034190	0.0024807	0.0000000	0.0000000
GBP5	0.0159147	0.0055226	0.0000000	0.0150152	0.0000000
ENSG00000286548	0.0014199	0.0073513	0.0063805	0.0019957	0.0052561
LRRC8B	0.0709741	0.1097450	0.1358190	0.0600730	0.1625939
LRRC8C-DT	0.0788911	0.0540830	0.0773711	0.0446990	0.0720955
LRRC8C	0.0203288	0.0401293	0.0329888	0.0280857	0.0508128
LRRC8D-DT	0.0025504	0.0032315	0.0074449	0.0093786	0.0174771
LRRC8D	0.3712558	0.3246546	0.2480870	0.3612530	0.4942739
ZNF326	0.5241136	0.5010301	0.4741088	0.5263260	0.5308526
ENSG00000287406	0.0055805	0.0048360	0.0046520	0.0051947	0.0034299
ENSG00000287372	0.0000000	0.0000000	0.0000000	0.0000000	0.0000000
LINC02609	0.1285552	0.1706377	0.2047948	0.1410939	0.1991451

LINC02788	0.0000000	0.0025532	0.0000000	0.0000000	0.0000000
LINC01763	0.0023485	0.0023392	0.0085898	0.0000000	0.0065298
ENSG00000272094	0.0000000	0.0000000	0.0000000	0.0000000	0.0034327
ZNF644	0.7067195	0.7425726	0.7495896	0.7078506	0.7661332
HFM1	0.0911220	0.0879344	0.1060425	0.0631377	0.3126835
CDC7	0.0657338	0.0971990	0.0983915	0.0730930	0.0734126
TGFBR3	0.0604653	0.0404622	0.0288179	0.0574511	0.0601466
BRDT	0.0000000	0.0015682	0.0000000	0.0000000	0.0000000
ENSG00000289483	0.0094523	0.0073595	0.0208237	0.0042237	0.0111909
EPHX4	0.0040985	0.0052256	0.0062477	0.0141812	0.0087089
BTBD8	0.3893903	0.6664583	0.7675205	0.2913135	0.6981789
ENSG00000273487	0.0028076	0.0043600	0.0044411	0.0000000	0.0000000
C1orf146	0.0000000	0.0000000	0.0000000	0.0000000	0.0000000
GLMN	0.1577691	0.1838632	0.2105873	0.2248999	0.2788857
RPAP2	0.3384787	0.4084810	0.5044247	0.4045894	0.5205323
GFI1	0.0000000	0.0000000	0.0000000	0.0000000	0.0000000
EVI5	0.6977036	0.5249335	0.4508312	0.6655274	0.6512088
RPL5	3.4270676	3.3807648	3.2019285	3.3579844	3.1308432
DIPK1A	0.2392718	0.2160969	0.2346393	0.2994760	0.2934938
ENSG00000289544	0.0000000	0.0000000	0.0022314	0.0000000	0.0038198
MTF2	0.3275674	0.3895497	0.3386000	0.2782055	0.5654435
TMED5	0.2999676	0.2292686	0.1862095	0.4209508	0.2288363
CCDC18	0.0601370	0.0644646	0.0450406	0.0519195	0.0722299
CCDC18-AS1	0.3009094	0.3447723	0.2825818	0.2848611	0.5629105
DR1	0.4793275	0.5693768	0.6279630	0.4798293	0.4137398
FNBP1L	1.1406092	1.3249152	1.3809882	1.1297070	1.4296965
BCAR3	0.0902740	0.0715593	0.0476652	0.1024180	0.0648131
BCAR3-AS1	0.0000000	0.0012671	0.0000000	0.0000000	0.0000000
ENSG00000260464	0.0134872	0.0208756	0.0272824	0.0039669	0.0263848
DNTTIP2	0.6066205	0.5429310	0.5496948	0.5209200	0.4989860
GCLM	0.5883502	0.3612436	0.2407689	0.5806840	0.3458115
ABCA4	0.0000000	0.0000000	0.0000000	0.0000000	0.0075217
ARHGAP29	0.1349370	0.1060919	0.1150297	0.1588584	0.0999003
ARHGAP29-AS1	0.0013269	0.0011324	0.0045790	0.0000000	0.0099649
ENSG00000286692	0.0006868	0.0000000	0.0000000	0.0000000	0.0000000
ABCD3	0.2289383	0.2531960	0.2381920	0.3129685	0.2954234
F3	0.0065997	0.0067520	0.0108495	0.0600557	0.0336710
ENSG00000288736	0.0024260	0.0000000	0.0000000	0.0039080	0.0000000
SLC44A3	0.0270230	0.0065763	0.0248005	0.0724142	0.0181923
CNN3	1.2344234	0.9363756	0.7516683	1.5806339	0.8604196
CNN3-DT	0.1425132	0.1545795	0.2520657	0.1337989	0.1628882
ALG14	0.1002976	0.1022800	0.0991745	0.1458915	0.1090383
ALG14-AS1	0.0000000	0.0000000	0.0000000	0.0000000	0.0000000
TLCD4	0.1354679	0.1380246	0.1726899	0.1055746	0.1980015
RWDD3-DT	0.0353646	0.0238589	0.0127995	0.0357064	0.0443914
RWDD3	0.1716076	0.1708364	0.1167176	0.2277929	0.1121777
ENSG00000228852	0.0029695	0.0000000	0.0012400	0.0000000	0.0000000
ENSG00000287919	0.0000000	0.0027264	0.0000000	0.0038821	0.0018039
LINC02607	0.0021748	0.0043503	0.0085479	0.0000000	0.0047572
PTBP2	0.4571151	0.6112359	0.7421467	0.3347248	0.9574263
DPYD	0.2727590	0.2468232	0.1548433	0.1992135	0.8690985
DPYD-AS1	0.0014062	0.0026199	0.0000000	0.0000000	0.0251064
DPYD-IT1	0.0000000	0.0000000	0.0000000	0.0000000	0.0000000

MIR137HG	0.1352056	0.1969951	0.2668811	0.0971794	0.8335097
ENSG00000259946	0.0091658	0.0164916	0.0009827	0.0000000	0.0599928
ENSG00000288810	0.0002548	0.0023621	0.0000000	0.0041542	0.0000000
LINC01776	0.0125793	0.0161782	0.0036641	0.0112701	0.0437577
SNX7	0.4654874	0.3420068	0.2902701	0.4519138	0.3149855
PLPPR5	0.1297478	0.3125471	0.4063946	0.1373112	0.3554992
PLPPR5-AS1	0.0044998	0.0119590	0.0194114	0.0105746	0.0456250
PLPPR4	0.0742898	0.1593908	0.2896859	0.0596519	0.2842075
LINC01708	0.0046344	0.0014091	0.0080419	0.0052546	0.0000000
PALMD	0.4935352	0.2866646	0.1563849	0.7777161	0.2643630
FRRS1	0.0068598	0.0012106	0.0033069	0.0030470	0.0113711
AGL	0.2653007	0.3491136	0.3346738	0.3047258	0.3432863
ENSG00000228084	0.0110234	0.0172982	0.0186570	0.0189063	0.0153178
SLC35A3	0.1327881	0.1164401	0.1184808	0.1372673	0.1884014
ENSG00000288826	0.0000000	0.0020936	0.0054693	0.0000000	0.0000000
MFSD14A	0.4037438	0.3223789	0.3326853	0.4119315	0.3845432
ENSG00000241073	0.0018785	0.0028392	0.0008297	0.0038563	0.0112983
SASS6	0.0881735	0.1289628	0.0963292	0.0456810	0.0823249
TRMT13	0.1973783	0.1252332	0.1180178	0.2029440	0.2041663
LRRC39	0.0649919	0.0455216	0.0154107	0.0383410	0.0807959
DBT	0.2615926	0.2265108	0.2079217	0.2511089	0.2140276
ENSG00000285530	0.0000000	0.0016541	0.0008246	0.0000000	0.0079723
RTCA-AS1	0.1017064	0.0855158	0.0606769	0.1211199	0.0817363
RTCA	0.4051878	0.4195623	0.4557449	0.3772923	0.4216270
CDC14A	0.0570692	0.0321905	0.0291137	0.0527150	0.0675288
GPR88	0.0154551	0.0186886	0.0363105	0.0209904	0.0101351
ENSG00000285525	0.0024801	0.0013789	0.0032677	0.0000000	0.0000000
VCAM1	0.0436872	0.0178729	0.0248860	0.2885768	0.0181836
EXTL2	0.2577795	0.2384783	0.2083613	0.3080449	0.1986874
ENSG00000273204	0.0056238	0.0101692	0.0035197	0.0078228	0.0107678
SLC30A7	0.1247068	0.1459400	0.1370111	0.1162595	0.2233408
DPH5	0.2746282	0.2670363	0.2041053	0.2567522	0.2458317
DPH5-DT	0.0894055	0.0811759	0.0660395	0.0477724	0.0883406
ENSG00000289355	0.0007915	0.0000000	0.0025528	0.0033019	0.0000000
S1PR1-DT	0.0077326	0.0021865	0.0000000	0.0096268	0.0000000
S1PR1	0.0237303	0.0082959	0.0062151	0.0821749	0.0356725
LINC01709	0.0000000	0.0000000	0.0052797	0.0000000	0.0162581
OLFM3	0.1222647	0.2485723	0.4356045	0.0984758	0.4623476
ENSG00000289192	0.0000000	0.0084667	0.0026275	0.0000000	0.0182806
COL11A1	0.0041927	0.0011085	0.0035392	0.0000000	0.0243541
RNPC3-DT	0.0015165	0.0020763	0.0029356	0.0179688	0.0177963
RNPC3	0.2358886	0.2081643	0.2297232	0.1570677	0.3580230
AMY2B	0.0112666	0.0172354	0.0212477	0.0302942	0.0860079
LINC01676	0.0005051	0.0013303	0.0000000	0.0000000	0.0049552
PRMT6	0.0869651	0.0950986	0.0965707	0.0883962	0.0781915
NTNG1	0.1649220	0.2537190	0.1593586	0.1065145	0.6209166
ENSG00000289612	0.0645258	0.1108913	0.0968712	0.0249132	0.1330811
VAV3	0.0674114	0.0842173	0.1225158	0.0402904	0.2412552
VAV3-AS1	0.0000000	0.0000000	0.0000000	0.0000000	0.0000000
LINC02785	0.0017819	0.0003960	0.0033772	0.0000000	0.0000000
SLC25A24	0.3179056	0.2632373	0.2252731	0.2004932	0.2079606
ENSG00000260879	0.0165845	0.0147541	0.0167669	0.0197527	0.0057345
EEIG2	0.2010798	0.3240540	0.3398417	0.1795212	0.3387144

HENMT1	0.0305886	0.0529976	0.0944533	0.0233031	0.0336284
ENSG00000285923	0.0075758	0.0056983	0.0061578	0.0000000	0.0000000
ENSG00000290126	0.0027617	0.0000000	0.0027481	0.0036572	0.0055309
PRPF38B	0.4196673	0.3798488	0.3363975	0.4293363	0.4140125
FNDC7	0.0026131	0.0015840	0.0000000	0.0080752	0.0000000
STXBP3	0.4385212	0.2804629	0.2328938	0.5271171	0.3594362
AKNAD1	0.0015884	0.0031488	0.0011538	0.0116078	0.0157041
GPSM2	0.0992553	0.0773292	0.0721811	0.1774418	0.1038863
CLCC1	0.2219728	0.1878226	0.2003478	0.3754192	0.2253271
WDR47	0.3609857	0.5207644	0.7234396	0.3806596	0.5026159
TAF13	0.3131768	0.3109787	0.3538614	0.3051901	0.3016374
TMEM167B-DT	0.0008804	0.0014250	0.0030754	0.0136065	0.0052497
TMEM167B	0.5244174	0.4310307	0.4208578	0.4980253	0.4154532
ENSG00000270066	0.0066315	0.0087951	0.0081195	0.0066988	0.0114801
CFAP276	0.6107544	0.4580261	0.2961833	0.5063867	0.3396647
ELAPOR1	0.2031058	0.1048419	0.1290758	0.3120190	0.2161368
SARS1	1.0974910	1.0458932	1.0959676	0.9903354	0.8830937
CELSR2	0.2678263	0.2115796	0.2617456	0.3674328	0.2938256
PSRC1	0.0233591	0.0221150	0.0318663	0.0710283	0.0319047
MYBPHL	0.0008588	0.0000000	0.0038101	0.0025961	0.0000000
SORT1	0.3408232	0.3002675	0.2948592	0.4412555	0.4215788
PSMA5	0.6292012	0.6129595	0.6827438	0.6653725	0.4856740
SYPL2	0.0000000	0.0000000	0.0000000	0.0017907	0.0000000
ATXN7L2	0.0371437	0.0405012	0.0370373	0.0335787	0.0373317
CYB561D1	0.0229356	0.0416091	0.0305306	0.0217921	0.0342827
AMIGO1	0.0506135	0.0459507	0.0543307	0.0861820	0.0370393
GPR61	0.0077826	0.0175660	0.0057824	0.0039696	0.0089716
ENSG00000290117	0.0015710	0.0024595	0.0069465	0.0000000	0.0032430
GNAI3	0.7381335	0.7156424	0.6374562	0.7599205	0.5855484
GNAT2	0.0008045	0.0000000	0.0000000	0.0000000	0.0075413
AMPD2	0.1154787	0.1681649	0.1980780	0.1420028	0.1921392
GSTM4	0.0875201	0.1001204	0.0896444	0.1049971	0.0801238
GSTM2	0.0321971	0.0412838	0.0283100	0.0593968	0.0480300
GSTM1	0.0004507	0.0000000	0.0000000	0.0000000	0.0000000
GSTM5	0.0011766	0.0012097	0.0000000	0.0000000	0.0000000
GSTM3	0.5777367	0.6229644	0.7478887	0.5470769	0.4024901
CSF1	0.0139302	0.0042330	0.0082622	0.0529077	0.0108388
AHCYL1	0.7228577	0.5685110	0.4889682	0.7510110	0.5803212
STRIP1	0.1468550	0.1797102	0.1903625	0.1500977	0.2502867
ENSG00000258634	0.0196803	0.0311670	0.0259688	0.0308538	0.0399380
ALX3	0.0032715	0.0127279	0.0045958	0.0051947	0.0145571
LINC01397	0.0012155	0.0010445	0.0020787	0.0000000	0.0000000
SLC6A17	0.0140653	0.0292352	0.0778719	0.0240801	0.0905693
SLC6A17-AS1	0.0014488	0.0000000	0.0000000	0.0000000	0.0000000
KCNC4-DT	0.0000000	0.0011200	0.0027353	0.0000000	0.0000000
KCNC4	0.0456363	0.0819617	0.0792922	0.0284503	0.0863642
RBM15-AS1	0.0104911	0.0136981	0.0103715	0.0143661	0.0087572
RBM15	0.0949302	0.1011797	0.0700292	0.0901315	0.1276440
LAMTOR5-AS1	0.0461366	0.0321586	0.0267720	0.0571264	0.1211345
SLC16A4	0.0086269	0.0068487	0.0023294	0.0062080	0.0254439
SLC16A4-AS1	0.0027044	0.0034970	0.0011570	0.0038410	0.0073283
LAMTOR5	1.1011516	0.9918138	0.9899921	1.1070370	0.7710157
KCNA2	0.0496454	0.0870697	0.0816607	0.0350810	0.0888895

KCNA3	0.0407815	0.1144749	0.1962261	0.0404580	0.1118029
ENSG00000288803	0.0015431	0.0006807	0.0134420	0.0000000	0.0040606
ENSG00000261654	0.0031824	0.0011649	0.0030754	0.0000000	0.0050338
ENSG00000232811	0.0113514	0.0139708	0.0110686	0.0154107	0.0183760
LRIF1	0.3215247	0.2972166	0.3806812	0.2827581	0.2573242
ENSG00000273010	0.0063055	0.0055651	0.0013627	0.0044161	0.0000000
DRAM2	0.1308299	0.1510007	0.1106003	0.1886450	0.2027237
CEPT1	0.1349239	0.1434643	0.1567767	0.1636972	0.1788214
ENSG00000273221	0.0003538	0.0016194	0.0000000	0.0076149	0.0089437
CHI3L2	0.0013355	0.0000000	0.0000000	0.0000000	0.0000000
PIFO	1.0299642	0.6458557	0.3991216	1.1766130	0.5572175
OVGP1	0.0037930	0.0126047	0.0102250	0.0000000	0.0282780
ENSG00000260948	0.0065148	0.0063238	0.0091083	0.0000000	0.0210659
ENSG00000243960	0.0057579	0.0077589	0.0066417	0.0053433	0.0208567
WDR77	0.1029424	0.1059185	0.1031973	0.1022135	0.0821524
ATP5PB	1.0402308	1.0733855	1.0801157	1.1516704	0.8226413
C1orf162	0.0030917	0.0085750	0.0029896	0.0060128	0.0289120
TMIGD3	0.0000000	0.0000000	0.0025165	0.0032175	0.0000000
RAP1A	0.5193707	0.4405685	0.4093459	0.5745571	0.5060805
INKA2	0.0398139	0.0223843	0.0111558	0.0515357	0.0438713
INKA2-AS1	0.0173807	0.0373440	0.0308334	0.0196951	0.0170217
ENSG00000284830	0.0000000	0.0043959	0.0053873	0.0000000	0.0000000
DDX20	0.0883592	0.0655178	0.0741154	0.0406449	0.0505420
KCND3	0.2935533	0.2608013	0.2866318	0.3038404	0.5546145
LINC01750	0.0545218	0.0406202	0.0347625	0.0639394	0.0421496
LINC02884	0.0000000	0.0000000	0.0000000	0.0000000	0.0000000
CTTNBP2NL	0.3668862	0.4638029	0.5589577	0.3633542	0.4609645
WNT2B	0.0252971	0.0281434	0.0262413	0.0243574	0.0979953
ENSG00000273483	0.0018846	0.0054367	0.0006833	0.0025371	0.0000000
ST7L	0.1679101	0.1709392	0.1190055	0.1809857	0.2906666
CAPZA1	0.7843157	0.7625805	0.8128631	0.7057449	0.6594909
MOV10	0.0232925	0.0138365	0.0070582	0.0192213	0.0310179
RHOC	0.5719990	0.5485608	0.3532294	0.6022891	0.3129555
PPM1J	0.0241260	0.0505513	0.0646625	0.0372887	0.0168162
TAFA3	0.0008562	0.0000000	0.0000000	0.0000000	0.0000000
LINC01356	0.0107154	0.0163215	0.0251856	0.0064190	0.0236491
LINC01357	0.0362626	0.0526861	0.0953874	0.0319529	0.0703759
SLC16A1	0.3573678	0.2143322	0.1664637	0.6948667	0.1860305
SLC16A1-AS1	0.0994864	0.1139611	0.1119561	0.0859822	0.2028166
ENSG00000287807	0.0052803	0.0051754	0.0022040	0.0067533	0.0014182
LRIG2-DT	0.0535566	0.0407163	0.0369128	0.0376677	0.0807857
LRIG2	0.1443485	0.1476964	0.1650573	0.1531621	0.2838810
MAGI3	0.3370424	0.4664175	0.6000136	0.3013177	0.8388204
PHTF1	0.4272677	0.3427661	0.3115286	0.4565529	0.3845690
RSBN1	0.5702000	0.5860363	0.6273605	0.4629228	0.4884047
ENSG00000231128	0.0017035	0.0000000	0.0000000	0.0000000	0.0132395
PTPN22	0.0021315	0.0010040	0.0013256	0.0000000	0.0141699
AP4B1-AS1	0.0007348	0.0022682	0.0060622	0.0059690	0.0016664
BCL2L15	0.0023616	0.0023718	0.0007696	0.0000000	0.0037366
AP4B1	0.0628701	0.0603233	0.0554683	0.0902903	0.0803730
DCLRE1B	0.0437815	0.0493416	0.0506157	0.0411785	0.0593242
HIPK1-AS1	0.0405136	0.0259231	0.0151683	0.0175650	0.0154351
HIPK1	0.6617010	0.5306871	0.4887985	0.6469765	0.5947492

OLFML3	0.1311473	0.0610169	0.0328995	0.1251665	0.0419596
SYT6	0.2331621	0.4093149	0.9286425	0.1583027	0.3589661
ENSG00000232895	0.0010639	0.0000000	0.0000000	0.0000000	0.0000000
TRIM33	0.4579359	0.4426562	0.4760782	0.4199772	0.6047399
BCAS2	0.6117536	0.6057775	0.6681525	0.5359808	0.5269363
DENND2C	0.0482295	0.0434233	0.0148768	0.0495051	0.0450239
AMPD1	0.0000000	0.0009666	0.0000000	0.0000000	0.0000000
NRAS	0.4441207	0.3718443	0.4228587	0.3991721	0.2845536
CSDE1	1.8234010	1.9157565	1.8936975	1.6849557	1.6501787
SIKE1	0.5383713	0.6421997	0.6887259	0.5597231	0.4957607
SYCP1	0.0000000	0.0009570	0.0000000	0.0000000	0.0000000
TSHB	0.0016565	0.0000000	0.0000000	0.0063543	0.0000000
TSPAN2	0.6193745	0.5512222	0.4983785	0.7199624	0.4032214
LINC01765	0.0612776	0.0455271	0.0138294	0.0542235	0.0151614
NGF-AS1	0.0156210	0.0023993	0.0049190	0.0086631	0.0024821
NGF	0.0014357	0.0046343	0.0021716	0.0037834	0.0015201
VANGL1	0.0908484	0.0496348	0.0488104	0.1780391	0.0571789
CASQ2	0.0012709	0.0025419	0.0000000	0.0000000	0.0000000
NHLH2	0.0221972	0.0677852	0.1255811	0.0206677	0.0822496
LINC01649	0.0023582	0.0038337	0.0035002	0.0000000	0.0048214
SLC22A15	0.0709124	0.1170623	0.1007134	0.0447167	0.2461709
MAB21L3	0.0014241	0.0030343	0.0035132	0.0035629	0.0098251
ATP1A1	0.5169494	0.4428777	0.4695399	0.5133004	0.4887643
ATP1A1-AS1	0.0398922	0.0522292	0.0308437	0.0426878	0.0335627
LINC01762	0.0002281	0.0000000	0.0000000	0.0000000	0.0034327
ENSG00000224950	0.0008107	0.0000000	0.0000000	0.0000000	0.0000000
CD58	0.1595759	0.0787347	0.0358633	0.2473068	0.0581886
IGSF3	0.3005944	0.2153068	0.2359251	0.4071433	0.2593874
CD2	0.0315561	0.0199752	0.0087092	0.0322895	0.0029535
ENSG00000272715	0.0452948	0.0199973	0.0070055	0.0469700	0.0388172
PTGFRN	0.5545518	0.2944211	0.2076407	0.8030434	0.3735552
CD101	0.0196908	0.0063806	0.0160275	0.0167099	0.0126394
CD101-AS1	0.0011144	0.0033235	0.0000000	0.0000000	0.0000000
TTF2	0.0611414	0.0299776	0.0291441	0.0256939	0.0503114
TRIM45	0.0201828	0.0163519	0.0045177	0.0208432	0.0000000
VTCN1	0.0037098	0.0000000	0.0023656	0.0000000	0.0000000
ENSG00000271427	0.0014200	0.0000000	0.0000000	0.0000000	0.0033030
MAN1A2	0.6424716	0.6186932	0.6055678	0.6788334	0.7150575
TENT5C	0.1084954	0.0427154	0.0397129	0.0846610	0.0457999
GDAP2	0.2219487	0.1869819	0.1731359	0.2123674	0.2629813
WDR3	0.2787316	0.2208252	0.2206665	0.2825565	0.2445470
SPAG17	0.0884670	0.0797343	0.0532726	0.1324482	0.1189895
TBX15	0.0000000	0.0030791	0.0000000	0.0000000	0.0000000
WARS2	0.1528088	0.1212397	0.0992062	0.1362461	0.1152444
WARS2-IT1	0.0000000	0.0000000	0.0000000	0.0000000	0.0000000
WARS2-AS1	0.0611630	0.0661967	0.0295836	0.0517186	0.1588772
ENSG00000227712	0.0018735	0.0000000	0.0006102	0.0036549	0.0000000
LINC00622	0.0154633	0.0271510	0.0363856	0.0187680	0.0463840
ZNF697	0.0504018	0.1189570	0.1559131	0.0387762	0.1808117
PHGDH	0.6976085	0.5153230	0.4087425	0.6631045	0.4191844
NOTCH2	0.1625519	0.0950852	0.0686226	0.2964431	0.1582907
ENSG00000273406	0.0045596	0.0005013	0.0000000	0.0000000	0.0067140
SEC22B	0.3216850	0.3111542	0.2892168	0.3663974	0.2751822

ENSG00000290569	0.1149244	0.1074519	0.0783346	0.0944093	0.0809579
NBPF8	0.0500084	0.0449786	0.0318071	0.0454747	0.0961009
ENSG00000290999	0.0305683	0.0377038	0.0297229	0.0229107	0.1026250
NOTCH2NLR	0.0050298	0.0062531	0.0006660	0.0046418	0.0000000
NBPF26	0.0246303	0.0180503	0.0166246	0.0148999	0.0410008
ENSG00000287979	0.0104279	0.0116661	0.0122213	0.0110691	0.0235757
LINC00623	0.0799203	0.1159761	0.1289917	0.0727270	0.1103682
ENSG00000290903	0.0000000	0.0000000	0.0039262	0.0024077	0.0025179
ENSG00000234998	0.0009980	0.0013522	0.0007553	0.0000000	0.0000000
ENSG00000233029	0.0021392	0.0017171	0.0020798	0.0000000	0.0077880
H2BP1	0.0080100	0.0132833	0.0054723	0.0050544	0.0000000
ENSG00000227193	0.0000000	0.0013282	0.0000000	0.0000000	0.0063791
FAM72B	0.0020904	0.0000000	0.0023834	0.0041835	0.0000000
SRGAP2C	0.2045776	0.1938377	0.1696654	0.2240391	0.1876839
SRGAP2-AS1	0.0000000	0.0000000	0.0000000	0.0000000	0.0000000
LINC02798	0.0812847	0.1197667	0.1253908	0.0654580	0.1587516
ENSG00000272583	0.0088081	0.0097838	0.0182468	0.0000000	0.0208567
EMBP1	0.0070133	0.0022592	0.0109178	0.0020988	0.0168650
ENSG00000232721	0.0236692	0.0183839	0.0351615	0.0071542	0.0299469
ENSG00000290735	0.0004654	0.0006151	0.0015540	0.0000000	0.0000000
H3-7	0.0125034	0.0237324	0.0104221	0.0038158	0.0114434
ENSG00000230186	0.0000000	0.0010052	0.0000000	0.0058485	0.0000000
FAM72C	0.0022736	0.0012589	0.0000000	0.0000000	0.0000000
ENSG00000289318	0.0256876	0.0164992	0.0141370	0.0209103	0.0104142
LINC02802	0.0035627	0.0038167	0.0000000	0.0000000	0.0029223
ENSG00000237343	0.0374940	0.0711206	0.0559642	0.0172684	0.0489673
NBPF15	0.1067556	0.1046627	0.0984407	0.0919655	0.1477577
ENSG00000290121	0.0036139	0.0050571	0.0095189	0.0041781	0.0044999
ENSG00000224363	0.0106328	0.0048687	0.0079263	0.0088930	0.0058520
LSP1P5	0.2465893	0.2367399	0.1864370	0.2487415	0.1935482
SRGAP2B	0.1976010	0.2243208	0.2020397	0.2038023	0.1474981
FAM72D	0.0021445	0.0028642	0.0066879	0.0000000	0.0000000
LINC01145	0.0543901	0.0589154	0.0738779	0.0214584	0.1202303
ENSG00000276216	0.0027463	0.0059859	0.0018415	0.0000000	0.0080063
ENSG00000236140	0.0111902	0.0167259	0.0186968	0.0063884	0.0225251
NBPF20	0.0879455	0.0963081	0.1050716	0.0576185	0.1997796
ENSG00000287374	0.0040702	0.0118744	0.0060775	0.0021126	0.0182676
NBPF25P	0.0042016	0.0066622	0.0012400	0.0072508	0.0267684
GPR89A	0.0218626	0.0260402	0.0221445	0.0204473	0.0504479
PDZK1	0.0096175	0.0164875	0.0124378	0.0011920	0.0057154
CD160	0.0000000	0.0000000	0.0026662	0.0021669	0.0014182
RNF115	0.1940090	0.2207688	0.2172241	0.1859845	0.2643125
POLR3C	0.1409566	0.1411581	0.1446368	0.1165646	0.1550454
NUDT17	0.0665820	0.0716564	0.0784069	0.1124813	0.0942657
PIAS3	0.1568464	0.1240321	0.0969217	0.1497933	0.1150470
ANKRD35	0.0248383	0.0355086	0.0372835	0.0197754	0.0105652
ITGA10	0.0049650	0.0044254	0.0022363	0.0041533	0.0033946
PEX11B	0.0951518	0.1030083	0.1199813	0.0969527	0.1040090
RBM8A	0.9392356	0.9153118	0.9726847	0.9122997	0.6459556
LIX1L-AS1	0.0394899	0.0320141	0.0269935	0.0294886	0.0416372
LIX1L	0.2301436	0.1813831	0.1585127	0.2350981	0.1645832
ANKRD34A	0.0318901	0.0547694	0.0870026	0.0159599	0.0321587
ENSG00000278431	0.0005440	0.0000000	0.0029736	0.0000000	0.0000000

POLR3GL	0.5204537	0.5681325	0.5323719	0.4617689	0.4111760
TXNIP	1.2121983	0.9135221	0.7879927	1.2023269	0.9233479
HJV	0.0009830	0.0010617	0.0023388	0.0031857	0.0000000
ENSG00000287190	0.0138545	0.0177446	0.0064764	0.0039267	0.0215468
LINC01719	0.0424818	0.0937731	0.0779163	0.0607287	0.0723670
NBPF10	0.0210001	0.0089314	0.0115618	0.0222059	0.0346592
NOTCH2NLA	0.0456113	0.0279147	0.0119349	0.0365063	0.0247966
ENSG00000276509	0.0242821	0.0169389	0.0098746	0.0127095	0.0034576
ENSG00000290578	0.0175006	0.0224799	0.0202824	0.0126743	0.0622282
ENSG00000287978	0.0327668	0.0205845	0.0343033	0.0277443	0.0232618
ENSG00000291242	0.0089347	0.0072672	0.0075020	0.0027125	0.0297205
ENSG00000289321	0.0032895	0.0015616	0.0036599	0.0000000	0.0123647
ENSG00000290557	0.0014670	0.0050221	0.0007240	0.0000000	0.0085859
ENSG00000291243	0.0000000	0.0000000	0.0000000	0.0000000	0.0000000
NBPF12	0.0628708	0.0437970	0.0614073	0.0685802	0.1533238
ENSG00000234225	0.0000000	0.0005660	0.0000000	0.0000000	0.0000000
PRKAB2	0.3199297	0.3114757	0.2499163	0.2636403	0.2616918
ENSG00000237188	0.1016640	0.1743640	0.1577852	0.1120858	0.1804195
FMO5	0.0068148	0.0158881	0.0068826	0.0113000	0.0116386
CHD1L	0.0299927	0.0198823	0.0123344	0.0295837	0.0209726
LINC00624	0.0028120	0.0050984	0.0000000	0.0029008	0.0348997
BCL9	0.2116078	0.2228587	0.2586607	0.1722596	0.2231436
ENSG00000289419	0.0030357	0.0007763	0.0000000	0.0000000	0.0169814
ACP6	0.0277181	0.0255783	0.0258765	0.0584637	0.0555887
GPR89B	0.0342945	0.0341174	0.0442733	0.0190942	0.0861868
ENSG00000290705	0.0543193	0.0575881	0.0817627	0.0698638	0.0338560
ENSG00000273059	0.0000000	0.0012382	0.0000000	0.0000000	0.0000000
LINC02804	0.0065461	0.0140207	0.0095740	0.0038308	0.0071627
NBPF11	0.0610605	0.0566590	0.0551400	0.0502739	0.0699606
ENSG00000227733	0.0091743	0.0069391	0.0023779	0.0030400	0.0029782
LINC01731	0.0048764	0.0049544	0.0252549	0.0014279	0.0120594
LINC01138	0.2366215	0.3064358	0.3224892	0.1968238	0.4184796
ENSG00000224481	0.0089038	0.0089729	0.0311887	0.0023550	0.0600982
ENSG00000272824	0.0006921	0.0056522	0.0016319	0.0000000	0.0135831
ENSG00000291232	0.0583679	0.0364153	0.0400532	0.0474101	0.0498719
NBPF14	0.0586611	0.0356580	0.0292085	0.0555147	0.0442085
NOTCH2NLB	0.0063021	0.0093409	0.0045469	0.0152480	0.0000000
NUDT4B	0.0217518	0.0118028	0.0026175	0.0403031	0.0155445
PDE4DIP	0.7256614	0.6563741	0.6971288	0.6445428	0.7326325
ENSG00000254539	0.0047889	0.0044277	0.0078224	0.0000000	0.0000000
NBPF9	0.0778442	0.0692329	0.0589738	0.0960858	0.0983709
ENSG00000274265	0.1354192	0.1535058	0.1566354	0.1459476	0.1529387
ENSG00000289614	0.2162554	0.1433209	0.1008338	0.1995289	0.0862389
ENSG00000291233	0.0901534	0.0848895	0.1002148	0.0764195	0.0809665
ENSG00000272755	0.0379808	0.0528782	0.0234808	0.0351575	0.0312343
NOTCH2NLC	0.0145487	0.0113031	0.0081345	0.0270535	0.0258634
NBPF19	0.0348922	0.0291087	0.0416955	0.0613293	0.0281569
LINC00869	0.1866444	0.1893614	0.2320193	0.1986295	0.2039830
ENSG00000290790	0.0000000	0.0000000	0.0000000	0.0000000	0.0000000
H2BC18	0.0102631	0.0049223	0.0049102	0.0059900	0.0396810
ENSG00000233030	0.0000000	0.0009813	0.0029476	0.0000000	0.0000000
ENSG00000290791	0.0064115	0.0311569	0.0370292	0.0043969	0.0367139
ENSG00000290792	0.0076434	0.0136317	0.0201670	0.0171503	0.0455674

ENSG00000264207	0.0250579	0.0215923	0.0346381	0.0242725	0.0414557
H2BC21	0.1554954	0.1725800	0.1954601	0.1705935	0.0966086
H2AC20	0.1721207	0.1361055	0.1220714	0.1702488	0.1565228
H2AC21	0.0143245	0.0074093	0.0068900	0.0106579	0.0044881
BOLA1	0.1098479	0.0913063	0.0646531	0.1367740	0.0562559
SV2A	0.2219955	0.3511955	0.7742222	0.2708613	0.4683985
SF3B4	0.3559731	0.3339154	0.3753608	0.3764150	0.2596314
MTMR11	0.0377813	0.0220979	0.0080310	0.0186729	0.0182882
OTUD7B	0.1036883	0.1022378	0.0593763	0.0782801	0.1782810
ENSG00000285184	0.0498184	0.0351886	0.0541952	0.0659173	0.0739534
VPS45	0.2484573	0.2075612	0.2288471	0.2202684	0.3881034
PLEKHO1	0.3113839	0.4444474	0.5086158	0.3324135	0.3684592
LINC02988	0.0025753	0.0017179	0.0000000	0.0000000	0.0144430
ANP32E	1.0175252	0.7670723	0.6672595	0.8743567	0.7075196
ENSG00000276110	0.0000000	0.0011817	0.0000000	0.0000000	0.0055061
CA14	0.0990519	0.0391725	0.0246190	0.2111006	0.0450756
APH1A	0.8331149	0.6754708	0.5520685	0.9154517	0.5717454
C1orf54	0.4307331	0.3700699	0.2170533	0.5078186	0.3239773
ENSG00000289041	0.0033842	0.0013201	0.0000000	0.0029570	0.0000000
CIART	0.1237605	0.0609546	0.0532279	0.1111851	0.0471264
MRPS21	1.0347854	1.0398653	1.0478539	1.0108944	0.8074354
PRPF3	0.1880114	0.1488493	0.1536247	0.1263176	0.2580200
RPRD2	0.4333743	0.3992829	0.4514478	0.4367765	0.6223459
TARS2	0.0946730	0.1174589	0.0864446	0.1214147	0.1012695
ECM1	0.0120498	0.0020546	0.0000000	0.0122856	0.0070428
FALEC	0.0069511	0.0100567	0.0113003	0.0222418	0.0146507
ADAMTSL4-AS2	0.0000000	0.0000000	0.0000000	0.0000000	0.0000000
ADAMTSL4	0.0213480	0.0160970	0.0036171	0.0232847	0.0077064
ADAMTSL4-AS1	0.0029062	0.0000000	0.0000000	0.0000000	0.0000000
MCL1	0.4301325	0.3166693	0.3345054	0.5032999	0.4250759
ENSG00000290074	0.0020651	0.0025271	0.0000000	0.0000000	0.0000000
ENSA	0.6957791	0.7612167	0.7501262	0.7515053	0.7536839
ENSG00000288880	0.0239494	0.0272962	0.0193115	0.0205187	0.0286443
GOLPH3L	0.1331555	0.1107282	0.0798789	0.1724325	0.0857839
HORMAD1	0.0000000	0.0000000	0.0000000	0.0000000	0.0000000
CTSS	0.0078733	0.0061192	0.0045433	0.0000000	0.0280906
CTSK	0.0126506	0.0080647	0.0050462	0.0023684	0.0302385
ARNT	0.1022564	0.1269213	0.1209440	0.1235037	0.2526614
CTXND2	0.0000000	0.0000000	0.0000000	0.0011582	0.0000000
SETDB1	0.1878194	0.1192605	0.1494307	0.1894333	0.1857172
CERS2	0.4927914	0.3534346	0.2241250	0.5803703	0.3260156
ENSG00000259357	0.0035248	0.0000000	0.0022266	0.0022936	0.0078524
ENSG00000231073	0.0019849	0.0029056	0.0022147	0.0035369	0.0000000
ANXA9	0.0002583	0.0038356	0.0018100	0.0033723	0.0076422
MINDY1	0.0999817	0.0721773	0.0345023	0.1049485	0.0431278
PRUNE1	0.1484724	0.1496060	0.2125815	0.1286288	0.1759504
BNIPL	0.0000000	0.0034294	0.0057495	0.0050679	0.0000000
C1orf56	0.1559138	0.1260374	0.1024975	0.1615282	0.1381696
CDC42SE1	0.2800033	0.2739150	0.2589794	0.2019697	0.2550079
MLLT11	1.4756729	1.9337215	2.2493014	1.4116034	1.4990688
GABPB2	0.0815954	0.0434278	0.0358553	0.0655589	0.1013060
ENSG00000261168	0.0000000	0.0007450	0.0000000	0.0038769	0.0111405
SEMA6C	0.0356321	0.0614369	0.0673665	0.0268884	0.0908537

ENSG00000289288	0.0009380	0.0031989	0.0000000	0.0000000	0.0124213
SCNM1	0.3596532	0.3389348	0.3638777	0.2766548	0.2514167
LYSMD1	0.0965915	0.1137394	0.0919557	0.0922228	0.0848218
TMOD4	0.0044846	0.0077746	0.0047666	0.0000000	0.0013244
VPS72	0.4699792	0.4695776	0.4884108	0.4776238	0.3796109
PIP5K1A	0.1642172	0.1349941	0.1078648	0.1601454	0.2560302
PSMD4	0.8102192	0.7439622	0.7509884	0.8504406	0.6556571
ZNF687-AS1	0.0118499	0.0071290	0.0099946	0.0121249	0.0167408
ZNF687	0.0418299	0.0443151	0.0448633	0.0736391	0.0372149
PI4KB	0.2377926	0.1733356	0.1820947	0.2600956	0.1482505
RFX5	0.0494299	0.0479613	0.0294613	0.0603908	0.0372987
RFX5-AS1	0.0305121	0.0405418	0.0294006	0.0521517	0.0317923
SELENBP1	0.4088953	0.2333042	0.1046358	0.3617288	0.1398333
PSMB4	0.9597741	0.9310829	0.9589048	0.9780868	0.7374717
POGZ	0.4612297	0.4969367	0.5399900	0.3371158	0.7183243
CGN	0.0310972	0.0286351	0.0311472	0.0368511	0.0246010
TUFT1	0.0475518	0.0591951	0.0752370	0.0287030	0.0927695
ENSG00000232536	0.0036381	0.0000000	0.0006116	0.0010951	0.0000000
SNX27	0.4549778	0.5170079	0.6005471	0.3909851	0.4576434
ENSG00000250734	0.0000000	0.0000000	0.0045698	0.0000000	0.0000000
CELF3	0.2200852	0.4955868	0.6033422	0.2386256	0.3959458
ENSG00000227045	0.0000000	0.0009108	0.0000000	0.0000000	0.0067140
RIIAD1	0.1355351	0.1104071	0.0719200	0.0937300	0.0465119
ENSG00000269621	0.0120506	0.0089709	0.0035957	0.0000000	0.0051206
MRPL9	0.2859444	0.3000148	0.3369174	0.3175085	0.2709088
OAZ3	0.0196534	0.0243125	0.0105217	0.0056856	0.0254270
ENSG00000249602	0.0010333	0.0024180	0.0020158	0.0033035	0.0000000
TDRKH	0.0678243	0.0947239	0.1246285	0.1174673	0.1234426
TDRKH-AS1	0.0272965	0.0318690	0.0451003	0.0287311	0.0368986
ENSG00000269489	0.0026870	0.0030050	0.0000000	0.0000000	0.0000000
RORC	0.0009580	0.0076887	0.0000000	0.0000000	0.0000000
C2CD4D	0.0004667	0.0000000	0.0000000	0.0000000	0.0000000
C2CD4D-AS1	0.0000000	0.0023893	0.0015298	0.0000000	0.0100465
THEM4	0.1667102	0.1764146	0.2536981	0.1942972	0.1958380
ENSG00000285651	0.0008671	0.0010451	0.0053576	0.0011730	0.0000000
S100A10	2.3698148	2.1948325	1.6548856	2.4055630	1.8494588
ENSG00000229021	0.0011284	0.0007970	0.0000000	0.0000000	0.0000000
S100A11	0.9307173	0.5608264	0.3684355	0.9435501	0.4951545
TCHH	0.0383457	0.0296984	0.0091059	0.0154911	0.1468072
FLG-AS1	0.0000000	0.0040193	0.0000000	0.0000000	0.0000000
SPRR2G	0.0011566	0.0021928	0.0000000	0.0030106	0.0000000
LORICRIN	0.0064896	0.0140112	0.0157597	0.0024968	0.0014182
S100A6	1.2214570	1.1182412	0.9510414	1.1920750	0.8980628
S100A4	0.0150303	0.0130607	0.0092373	0.0251177	0.0179691
S100A2	0.0046551	0.0000000	0.0000000	0.0024702	0.0000000
ENSG00000285867	0.0028911	0.0000000	0.0000000	0.0057071	0.0027533
S100A16	0.0210805	0.0138063	0.0139842	0.0329086	0.0384948
S100A13	0.2827223	0.2594316	0.2219144	0.3842565	0.1836072
ENSG00000272030	0.0054471	0.0101722	0.0000000	0.0060938	0.0000000
CHTOP	0.5059210	0.5335190	0.5686269	0.5011563	0.3705743
SNAPIN	0.7000481	0.4932174	0.4306522	0.7573002	0.4201291
ILF2	0.8917384	0.7851838	0.7964212	0.9641780	0.6712361
NPR1	0.0000000	0.0000000	0.0036041	0.0000000	0.0061857

INTS3	0.1027893	0.1326078	0.1074761	0.1356670	0.2141085
SLC27A3	0.0784056	0.0673474	0.0559378	0.1118591	0.0561065
GATAD2B	0.3392795	0.3972952	0.3723174	0.3586685	0.6181277
ENSG00000284738	0.0071689	0.0088158	0.0054842	0.0091916	0.0064050
DENND4B	0.0792142	0.1129137	0.1131463	0.0549519	0.1306158
CRTC2	0.0665347	0.0388252	0.0185636	0.0489854	0.0476307
SLC39A1	0.2610393	0.1412550	0.1418706	0.3509119	0.0950352
ENSG00000282386	0.0150266	0.0115368	0.0162691	0.0107824	0.0135830
ENSG00000273026	0.0032478	0.0000000	0.0065532	0.0000000	0.0058809
CREB3L4	0.0543486	0.0465954	0.0371038	0.0600764	0.0528571
JTB	0.8209885	0.7427683	0.8114236	0.7220013	0.5469142
JTB-DT	0.0276034	0.0527417	0.0449049	0.0302132	0.0239240
RAB13	0.8612530	0.6105050	0.4828092	0.6089458	0.5762777
RPS27	2.8197314	2.7134912	2.5417122	2.6642875	2.5663666
NUP210L	0.0096237	0.0039234	0.0070359	0.0000000	0.0412991
TPM3	1.3264187	1.2232080	1.2694243	1.2829250	1.0406346
CFAP141	0.0099410	0.0052652	0.0022648	0.0151792	0.0000000
C1orf43	0.9028793	0.8090079	0.8346671	0.8568147	0.6104433
UBAP2L	0.4828358	0.4702040	0.5182785	0.4526461	0.4730298
HAX1	0.6537404	0.5993325	0.6356310	0.6118364	0.4343030
ATP8B2	0.0762252	0.0896091	0.0610944	0.0565378	0.2562013
IL6R-AS1	0.0037394	0.0000000	0.0000000	0.0000000	0.0035834
IL6R	0.0144459	0.0148684	0.0066369	0.0137898	0.0180962
SHE	0.0052432	0.0055394	0.0068266	0.0000000	0.0030832
TDRD10	0.0012022	0.0000000	0.0000000	0.0000000	0.0063663
UBE2Q1	0.3796379	0.3907368	0.4258750	0.3499819	0.3218721
ENSG00000286391	0.0030433	0.0200743	0.0132738	0.0031460	0.0000000
CHRNB2	0.0100694	0.0326515	0.0401852	0.0049554	0.0245412
ADAR	0.5439465	0.5840668	0.6648114	0.5559992	0.6447936
ENSG00000287064	0.0005278	0.0000000	0.0000000	0.0000000	0.0000000
KCNN3	0.2179373	0.1637857	0.1377847	0.4731134	0.2188130
PMVK	0.3638953	0.3026100	0.2949585	0.4891850	0.1705875
ENSG00000270361	0.0017684	0.0000000	0.0020874	0.0032336	0.0000000
PBXIP1	0.5877309	0.3196907	0.1916617	0.8475000	0.4111296
PYGO2	0.1700733	0.1505384	0.1648171	0.1740288	0.1105637
ENSG00000271380	0.0272144	0.0210268	0.0233425	0.0261664	0.0106313
SHC1	0.1317714	0.1045214	0.0572454	0.1876483	0.0880982
CKS1B	0.2741027	0.2254160	0.1565417	0.2986371	0.1157975
FLAD1	0.1363175	0.1556376	0.1462124	0.1165575	0.0690081
ZBTB7B	0.0301909	0.0070921	0.0068626	0.0167685	0.0113492
DCST2	0.0041073	0.0000000	0.0022326	0.0000000	0.0026749
DCST1	0.0011932	0.0000000	0.0028789	0.0055470	0.0079769
DCST1-AS1	0.0103407	0.0143074	0.0160684	0.0312209	0.0057720
ADAM15	0.0954715	0.0821359	0.0569558	0.1229769	0.0600315
EFNA4	0.0468814	0.0263500	0.0085924	0.0716596	0.0356310
EFNA3	0.1355946	0.2144817	0.2066532	0.1385266	0.1657757
EFNA1	0.0118220	0.0288703	0.0134834	0.0241168	0.0163920
SLC50A1	0.1122142	0.0832045	0.1072695	0.1569291	0.0755095
DPM3	0.4469194	0.3837300	0.3807036	0.4510650	0.3437207
KRTCAP2	0.6887430	0.6674798	0.6451373	0.7539653	0.5323400
TRIM46	0.0256765	0.0375887	0.0349266	0.0217960	0.1038400
MUC1	0.0084790	0.0016014	0.0012912	0.0000000	0.0018081
THBS3-AS1	0.1345889	0.0682282	0.0409195	0.1274186	0.0626396

THBS3	0.0779291	0.0476745	0.0210608	0.1076051	0.0765799
MTX1	0.2451277	0.2173901	0.2041179	0.3227781	0.1183176
GBAP1	0.0087365	0.0060766	0.0008634	0.0142935	0.0145844
GBA1	0.0283502	0.0127415	0.0184154	0.0612121	0.0511532
ENTREP3	0.1328029	0.1740281	0.1617345	0.1233187	0.1522013
SCAMP3	0.2449678	0.2182215	0.2379871	0.2524946	0.2287784
CLK2	0.1012870	0.0779907	0.0653530	0.0990187	0.0689596
HCN3	0.0348608	0.0447357	0.0541384	0.0308685	0.1007852
PKLR	0.0020921	0.0052713	0.0000000	0.0000000	0.0041410
FDPS	0.5320689	0.5002945	0.5695649	0.6951376	0.4040601
RUSC1-AS1	0.0050060	0.0061759	0.0040477	0.0114127	0.0192603
RUSC1	0.2803662	0.4126219	0.4405416	0.2468336	0.3039863
ASH1L	0.7310019	0.7279306	0.7186466	0.6155289	1.0147216
ASH1L-IT1	0.0000000	0.0007880	0.0000000	0.0000000	0.0000000
ASH1L-AS1	0.0215544	0.0284863	0.0292453	0.0317842	0.0265444
MSTO1	0.0878569	0.0932904	0.1224985	0.0920368	0.1385066
ENSG00000232519	0.0007321	0.0000000	0.0000000	0.0012897	0.0000000
ENSG00000287839	0.0016126	0.0026636	0.0093135	0.0000000	0.0031757
YY1AP1	0.1772443	0.1889367	0.2180993	0.1660275	0.3139392
DAP3	0.4941716	0.4136241	0.3984245	0.5203875	0.3711361
GON4L	0.2160397	0.2428573	0.2365693	0.2588272	0.3447070
SYT11	0.8556291	0.9629771	1.0047793	1.1363389	0.9902896
RIT1	0.3818415	0.2687079	0.1876644	0.4172348	0.2595541
KHDC4	0.1178711	0.1224767	0.0733112	0.1168790	0.1747230
ARHGEF2	0.1693308	0.1493134	0.1001503	0.1179122	0.1971962
ARHGEF2-AS2	0.0008511	0.0000000	0.0000000	0.0000000	0.0063922
ARHGEF2-AS1	0.0000000	0.0000000	0.0000000	0.0000000	0.0000000
ENSG00000285677	0.0035984	0.0042664	0.0021912	0.0000000	0.0026235
SSR2	0.6971094	0.7185134	0.6472358	0.6791512	0.5589382
UBQLN4	0.0952464	0.1400288	0.1581318	0.0917749	0.1116901
LAMTOR2	0.6360442	0.6444514	0.6595848	0.7159489	0.5868730
RAB25	0.0017201	0.0006091	0.0020198	0.0000000	0.0000000
MEX3A	0.1967431	0.3299810	0.3263593	0.1758545	0.3340962
LMNA	0.4102565	0.3197233	0.2495723	0.4142285	0.4446276
SEMA4A	0.0330402	0.0344533	0.0824417	0.0813608	0.0575155
SLC25A44	0.0597982	0.0738037	0.0691175	0.0639322	0.0592636
PMF1	0.2805942	0.1977816	0.2155464	0.2661183	0.1635537
BGLAP	0.1134134	0.0904121	0.1243148	0.1483553	0.0554312
PAQR6	0.0387797	0.0285044	0.0234793	0.0426537	0.0527853
SMG5	0.1133961	0.1150341	0.1163139	0.1193370	0.1319424
TMEM79	0.0267456	0.0242099	0.0193366	0.0390503	0.0000000
GLMP	0.2710158	0.1446448	0.1275216	0.2624017	0.1463931
CCT3	1.1833370	1.1477805	1.1496433	1.2646555	0.8856086
TSACC	0.0041660	0.0000000	0.0052103	0.0068003	0.0066722
MIR9-1HG	0.0167343	0.0161216	0.0119924	0.0645230	0.0326385
MEF2D	0.0791514	0.1047977	0.0814173	0.1014833	0.0867023
IQGAP3	0.0009632	0.0009278	0.0018252	0.0000000	0.0038839
NAXE	0.3664984	0.3442468	0.4085957	0.4920484	0.3759177
GPATCH4	0.0933437	0.1111576	0.1089205	0.0988045	0.1051423
ENSG00000272971	0.0028771	0.0023732	0.0048192	0.0044367	0.0000000
HAPLN2	0.0062713	0.0055624	0.0023738	0.0035871	0.0295139
BCAN-AS1	0.0000000	0.0010959	0.0000000	0.0037736	0.0047492
BCAN	0.2210758	0.1468700	0.0699431	0.8984603	0.1676501

ENSG00000272405	0.0008002	0.0033243	0.0000000	0.0000000	0.0000000
BCAN-AS2	0.0015413	0.0000000	0.0000000	0.0025494	0.0000000
NES	1.2741783	0.9511697	0.6008919	1.2581702	0.8846521
ENSG00000285570	0.0008353	0.0012869	0.0000000	0.0031073	0.0000000
CRABP2	0.1002601	0.1112889	0.1330901	0.1357265	0.1178333
ISG20L2	0.2125975	0.2072240	0.1843726	0.2524312	0.1788467
METTL25B	0.0970272	0.0962739	0.0833581	0.1062588	0.0782382
MRPL24	0.3703952	0.3099996	0.2949134	0.3779895	0.2272308
HDGF	0.6480939	0.5158483	0.5312982	0.5969726	0.4552577
PRCC	0.3536098	0.3742173	0.4231943	0.3380602	0.3601819
SH2D2A	0.0067556	0.0010856	0.0096881	0.0019766	0.0000000
NTRK1	0.0134418	0.0127410	0.0009751	0.0093006	0.0041632
INSRR	0.0101561	0.0037554	0.0000000	0.0207409	0.0015171
PEAR1	0.0003529	0.0029139	0.0000000	0.0014092	0.0000000
LRRC71	0.0228278	0.0092484	0.0284428	0.0271746	0.0196496
ARHGEF11	0.2065489	0.3047340	0.3334847	0.2507728	0.5269684
ETV3L	0.0015466	0.0000000	0.0000000	0.0000000	0.0025831
ETV3	0.0882217	0.0782361	0.0650443	0.0748099	0.1106032
KIRREL1	0.0491854	0.0720500	0.0756726	0.0615299	0.2425635
LINC01704	0.0000000	0.0000000	0.0020642	0.0058194	0.0049014
CD1D	0.0050580	0.0054266	0.0050100	0.0000000	0.0000000
ENSG00000176320	0.0030427	0.0000000	0.0011570	0.0000000	0.0000000
PYHIN1	0.0000000	0.0000000	0.0000000	0.0043373	0.0055669
IFI16	0.0279563	0.0442279	0.0168271	0.0343914	0.0499410
AIM2	0.0417925	0.0417013	0.0254920	0.0227553	0.0340579
CADM3	1.7008669	1.3697853	1.2010250	1.7089935	1.2083232
CADM3-AS1	0.0674400	0.0357837	0.0399277	0.0890000	0.0440082
ACKR1	0.0569483	0.0338581	0.0247811	0.0701674	0.0300921
DUSP23	0.2703888	0.2893288	0.2577983	0.2367273	0.1708104
SNHG28	0.0411549	0.0625665	0.0492271	0.0283482	0.0352552
VSIG8	0.0035413	0.0028019	0.0027683	0.0108476	0.0056744
ENSG00000272668	0.0069325	0.0087424	0.0032703	0.0000000	0.0106710
CFAP45	0.5414438	0.3484572	0.2453060	0.5483464	0.4037513
ENSG00000287040	0.0000000	0.0000000	0.0000000	0.0000000	0.0000000
TAGLN2	1.8461830	1.3612169	0.9723227	1.8989582	1.2222087
IGSF9	0.0185108	0.0144750	0.0142875	0.0126331	0.0266983
SLAMF9	0.0054687	0.0032194	0.0043071	0.0020158	0.0033613
LINC01133	0.0379271	0.0178528	0.0086935	0.0595935	0.0420971
KCNJ10	0.0015977	0.0000000	0.0031674	0.0019022	0.0035834
PIGM	0.1061255	0.0943137	0.0958614	0.1226899	0.0940347
ENSG00000225279	0.0006262	0.0043538	0.0000000	0.0000000	0.0188329
KCNJ9	0.0032455	0.0198758	0.0421236	0.0015536	0.0311532
IGSF8	0.2415127	0.2246927	0.2394547	0.4411282	0.2027464
ATP1A2	0.2291609	0.1281910	0.0592084	0.4656521	0.0970630
ATP1A4	0.0000000	0.0029687	0.0000000	0.0000000	0.0000000
CASQ1	0.0057049	0.0030631	0.0000000	0.0064621	0.0000000
ENSG00000227741	0.0023551	0.0055795	0.0064259	0.0000000	0.0037333
PEA15	1.5534006	1.2879083	1.0539889	1.8448605	1.1255153
DCAF8	0.4651795	0.3281697	0.2440078	0.5263589	0.3717630
DCAF8-DT	0.0050783	0.0000000	0.0000000	0.0112775	0.0076833
PEX19	0.3831064	0.2492891	0.2014794	0.4017521	0.2280184
COPA	0.5888009	0.5323204	0.4216808	0.5840021	0.5212603
NCSTN	0.4244594	0.2557590	0.2060879	0.7673035	0.3147519

NHLH1	0.0023830	0.0105373	0.0138731	0.0000000	0.0082850
VANGL2	0.2603485	0.2912045	0.3243333	0.2920503	0.2692587
F11R	0.0825535	0.0600111	0.0640712	0.1218343	0.0945819
TSTD1	0.7607356	0.6254582	0.6052341	0.7959291	0.4783895
USF1	0.1030526	0.1135000	0.1365109	0.1330311	0.0968564
ENSG00000289121	0.0082690	0.0124052	0.0052935	0.0000000	0.0097993
ARHGAP30	0.0014354	0.0000000	0.0027945	0.0000000	0.0035493
NECTIN4	0.0000000	0.0000000	0.0000000	0.0000000	0.0000000
KLHDC9	0.2700260	0.2390766	0.2904204	0.2839684	0.1224523
PFDN2	1.1061206	1.3727722	1.6309255	1.0141298	1.0481939
NIT1	0.2827729	0.2253014	0.2318774	0.2836711	0.2036403
DEDD	0.1756401	0.2037277	0.1626833	0.1687812	0.1227104
UFC1	0.8847601	0.8419321	0.8204681	0.8424229	0.6570229
ENSG00000224985	0.0005783	0.0015056	0.0079877	0.0044064	0.0021357
USP21	0.0991838	0.1036735	0.1108796	0.0863729	0.0643333
ENSG00000290115	0.0000000	0.0000000	0.0016034	0.0000000	0.0000000
PPOX	0.0961382	0.1135768	0.1716018	0.1384878	0.0809969
B4GALT3	0.1974845	0.1794387	0.2488942	0.2152531	0.1875726
ADAMTS4	0.0006173	0.0000000	0.0000000	0.0000000	0.0000000
NDUFS2	0.5339050	0.5396516	0.5373005	0.5568249	0.3910673
FCER1G	0.0000000	0.0010696	0.0000000	0.0000000	0.0000000
TOMM40L	0.0329134	0.0454823	0.0619505	0.0444366	0.0959443
NR1I3	0.0043285	0.0036520	0.0041112	0.0114673	0.0053847
PCP4L1	0.0189015	0.0529058	0.1045364	0.0211509	0.0548671
MPZ	0.0031364	0.0043032	0.0014694	0.0046716	0.0115169
SDHC	0.8486197	0.8150764	0.7991444	0.9079820	0.6583627
ENSG00000289106	0.0257745	0.0507639	0.0288926	0.0301237	0.0369729
CFAP126	0.7548403	0.4565389	0.3289205	0.8075692	0.4578563
ENSG00000288670	0.0471390	0.0794946	0.0563290	0.0313560	0.0744008
ENSG00000289141	0.0012019	0.0000000	0.0000000	0.0000000	0.0037431
ENSG00000283696	0.0090349	0.0090971	0.0029745	0.0000000	0.0150577
ENSG00000288093	0.0260317	0.0141724	0.0117917	0.0129873	0.0043425
ENSG00000283360	0.0085472	0.0064981	0.0084650	0.0036480	0.0135361
FCGR2A	0.0003474	0.0000000	0.0000000	0.0000000	0.0000000
HSPA6	0.0146179	0.0012157	0.0025929	0.0031460	0.0000000
FCRLB	0.0087699	0.0120689	0.0236193	0.0058613	0.0091356
ATF6-DT	0.0052739	0.0175197	0.0064900	0.0331336	0.0127208
DUSP12	0.2383443	0.3033090	0.3248779	0.2176662	0.1745036
ATF6	0.4574861	0.4059237	0.4559152	0.6113211	0.5322654
OLFML2B	0.1607337	0.0821543	0.0408475	0.2664973	0.0942645
NOS1AP	0.3227457	0.2902714	0.2212891	0.3705229	0.6497495
ENSG00000285636	0.0007897	0.0000000	0.0000000	0.0000000	0.0162772
SPATA46	0.0073013	0.0014978	0.0000000	0.0042562	0.0000000
C1orf226	0.0545058	0.0367068	0.0175169	0.0903936	0.0354316
SH2D1B	0.0000000	0.0000000	0.0000000	0.0000000	0.0000000
UHMK1	0.4059916	0.3578757	0.3659200	0.3750506	0.3687787
UAP1	0.2540106	0.2661842	0.3033443	0.2134564	0.1651262
DDR2	0.4432327	0.2603650	0.2248897	0.3482061	0.2890060
HSD17B7	0.0753978	0.0649583	0.0758209	0.0688258	0.1035369
CCDC190	0.0205456	0.0116915	0.0083054	0.0176635	0.0000000
RGS4	0.2233022	0.3340494	0.9878198	0.1916419	0.3856162
RGS5	0.0402569	0.0193981	0.0136232	0.0277531	0.0329148
RGS5-AS1	0.0017429	0.0007567	0.0000000	0.0018378	0.0000000

ENSG00000232995	0.0179159	0.0173542	0.0287897	0.0170249	0.0173913
NUF2	0.0215890	0.0207218	0.0078920	0.0061692	0.0379948
PBX1	1.1301804	1.2788971	1.5388425	1.0686480	1.7644305
ENSG00000269887	0.0028176	0.0000000	0.0000000	0.0050196	0.0201550
PBX1-AS1	0.0000000	0.0000000	0.0000000	0.0021126	0.0069313
ENSG00000271917	0.0038670	0.0000000	0.0016825	0.0026530	0.0076717
RXRG	0.0688901	0.0926790	0.0550389	0.0624543	0.1188653
LRRC52-AS1	0.0000000	0.0000000	0.0000000	0.0042422	0.0000000
LRRC52	0.0000000	0.0000000	0.0000000	0.0030518	0.0000000
ENSG00000236206	0.0010438	0.0040162	0.0022375	0.0069059	0.0167278
MGST3	1.4874336	1.6790232	1.7329113	1.6484781	1.3332755
ALDH9A1	0.5725262	0.3403682	0.2578406	0.6889439	0.3093966
ENSG00000273365	0.0000000	0.0000000	0.0000000	0.0000000	0.0000000
TMCO1	0.7727735	0.6970341	0.6685728	1.0321661	0.6135445
TMCO1-AS1	0.0127618	0.0132103	0.0069598	0.0198703	0.0112580
UCK2	0.0846657	0.1266126	0.1590952	0.0813521	0.1828298
ENSG00000236364	0.0000000	0.0022547	0.0000000	0.0000000	0.0075749
FAM78B	0.0405305	0.1111389	0.1330388	0.0387921	0.1984255
FAM78B-AS1	0.0000000	0.0000000	0.0000000	0.0000000	0.0000000
ENSG00000230898	0.0000000	0.0000000	0.0000000	0.0000000	0.0000000
ENSG00000229588	0.0262329	0.0229937	0.0499568	0.0024483	0.0513086
POGK	0.2249482	0.2148688	0.2386633	0.2298225	0.2308483
TADA1	0.1680094	0.1504649	0.1688193	0.1026361	0.1843272
ILDR2	0.0728182	0.0964518	0.1270256	0.0609741	0.1340490
MAEL	0.0035519	0.0080908	0.0038903	0.0051065	0.0072501
GPA33	0.0000000	0.0011627	0.0013865	0.0000000	0.0066201
STYXL2	0.0048873	0.0000000	0.0000000	0.0000000	0.0057461
POU2F1-DT	0.0019177	0.0000000	0.0011486	0.0000000	0.0000000
POU2F1	0.3475091	0.3724413	0.4312982	0.2798049	0.5782916
ENSG00000272033	0.0000000	0.0022110	0.0025170	0.0020053	0.0035909
CD247	0.0102007	0.0145693	0.0129044	0.0054843	0.0358746
ENSG00000287218	0.0000000	0.0068835	0.0042275	0.0036618	0.0000000
CREG1	0.3076261	0.3181542	0.3206419	0.3575756	0.2477322
RCSD1	0.0000000	0.0010034	0.0000000	0.0000000	0.0000000
MPZL1	0.6741785	0.7052566	0.7786419	0.7776565	0.6599757
ADCY10	0.0078826	0.0110064	0.0104925	0.0111661	0.0234899
MPC2	0.8813929	0.9089109	0.9708079	0.8742489	0.6796074
DCAF6	0.2341518	0.2470747	0.2226664	0.2210676	0.3634108
GPR161	0.0998312	0.1479491	0.2245941	0.1224651	0.2111229
TIPRL	0.8635466	0.8911398	1.0612439	0.8503359	0.7322117
SFT2D2	0.1190028	0.1364582	0.0757549	0.1555982	0.1475102
TBX19	0.0078922	0.0172264	0.0050824	0.0049511	0.0288113
ENSG00000228697	0.0000000	0.0000000	0.0000000	0.0000000	0.0000000
ENSG00000285622	0.0013367	0.0000000	0.0019465	0.0000000	0.0000000
ATP1B1	1.7676694	1.8060164	2.1041731	1.9642931	1.6281647
NME7	0.3894669	0.3295055	0.3742324	0.4232249	0.4922235
ENSG00000235575	0.0006477	0.0000000	0.0000000	0.0000000	0.0142597
BLZF1	0.3366568	0.2530165	0.1910847	0.2436554	0.2709876
CCDC181	0.5299640	0.4551166	0.4275920	0.4530824	0.2995957
SLC19A2	0.0372479	0.0618670	0.0285975	0.0589858	0.0577071
ENSG00000213062	0.0488208	0.0889891	0.0755846	0.0167664	0.1099742
F5	0.4367688	0.2258855	0.1862965	1.3471965	0.3587438
SELP	0.0017821	0.0017657	0.0000000	0.0060682	0.0000000

C1orf112	0.0196965	0.0116033	0.0112517	0.0270468	0.0582584
SELL	0.0682272	0.0641572	0.0184896	0.5178990	0.0691451
METTL18	0.1101600	0.0746415	0.1060749	0.1334170	0.0403701
SCYL3	0.0534874	0.0455219	0.0508086	0.0705870	0.0474860
KIFAP3	0.8824421	1.0556934	1.4144250	0.8484277	1.0642621
NTMT2	0.0000000	0.0020643	0.0039562	0.0029316	0.0000000
LINC01681	0.0000000	0.0000000	0.0000000	0.0000000	0.0000000
GORAB-AS1	0.0093937	0.0039490	0.0007564	0.0000000	0.0041978
GORAB	0.1241516	0.1103393	0.1232256	0.1159001	0.1135851
PRRX1	0.0027206	0.0000000	0.0000000	0.0000000	0.0046792
ENSG00000231424	0.0022322	0.0037679	0.0067556	0.0000000	0.0000000
FMO2	0.0007683	0.0010023	0.0000000	0.0000000	0.0000000
FMO1	0.0011597	0.0009486	0.0000000	0.0000000	0.0000000
FMO4	0.0133055	0.0045502	0.0122050	0.0216954	0.0032430
PRRC2C	1.0961219	1.2080205	1.3152395	1.1462269	1.4714598
MYOCOS	0.0053060	0.0068503	0.0021479	0.0029736	0.0285244
MYOC	0.0000000	0.0025416	0.0000000	0.0000000	0.0033772
VAMP4	0.2254368	0.2236289	0.2607767	0.2309131	0.1623310
METTL13	0.0776413	0.0861960	0.0644001	0.0780353	0.1129347
DNM3	0.3761141	0.6625056	0.8765994	0.3294618	1.1436754
DNM3-IT1	0.0000000	0.0000000	0.0000000	0.0000000	0.0017206
DNM3OS	0.0088932	0.0013013	0.0000000	0.0000000	0.0142786
ENSG00000287336	0.0000000	0.0000000	0.0000000	0.0000000	0.0000000
PIGC	0.2700814	0.2787593	0.2328196	0.2596511	0.2014527
C1orf105	0.0017930	0.0000000	0.0000000	0.0000000	0.0028456
SUCO	0.6888089	0.5716571	0.5331593	0.7281101	0.6797001
ENSG00000224228	0.0000000	0.0000000	0.0000000	0.0000000	0.0090164
TNFSF18	0.0017283	0.0043518	0.0000000	0.0076422	0.0000000
TNFSF4	0.0069589	0.0089848	0.0075518	0.0098729	0.0126006
PRDX6-AS1	0.0402620	0.0486780	0.0350303	0.0543344	0.1751792
PRDX6	1.5229154	1.2336764	1.1335859	1.5980666	1.1100971
SLC9C2	0.0147342	0.0198972	0.0101616	0.0165218	0.0510260
ENSG00000238272	0.0034095	0.0000000	0.0000000	0.0000000	0.0046972
ANKRD45	0.1572575	0.0845916	0.0792851	0.2044478	0.1191958
ENSG00000289426	0.0003183	0.0029242	0.0000000	0.0030907	0.0000000
KLHL20	0.1720948	0.1209062	0.1323359	0.1666959	0.2261602
CENPL	0.0291242	0.0309066	0.0235509	0.0487592	0.0608920
DARS2	0.0430942	0.0534505	0.0419818	0.0631444	0.0500328
GAS5	1.8772987	1.8670208	1.7331711	1.7550111	1.6911530
GAS5-AS1	0.0020311	0.0025218	0.0035812	0.0046836	0.0000000
ZBTB37	0.3270025	0.3663053	0.3669832	0.2549873	0.3182161
SERPINC1	0.0016468	0.0008286	0.0022040	0.0000000	0.0012623
RC3H1	0.3462926	0.4262427	0.4238614	0.3645129	0.5628915
RC3H1-IT1	0.0011032	0.0000000	0.0000000	0.0000000	0.0000000
RC3H1-DT	0.0034446	0.0014527	0.0000000	0.0000000	0.0068646
RABGAP1L-DT	0.0081074	0.0084840	0.0075160	0.0000000	0.0078510
RABGAP1L	0.6866741	0.8060917	0.9027367	0.6110929	1.2044278
GPR52	0.0007744	0.0000000	0.0013034	0.0000000	0.0035346
RABGAP1L-IT1	0.0006360	0.0000000	0.0000000	0.0000000	0.0061020
RABGAP1L-AS1	0.0000000	0.0000000	0.0000000	0.0000000	0.0093297
ENSG00000289425	0.0008233	0.0033962	0.0022682	0.0000000	0.0091859
ENSG00000287697	0.0031287	0.0034406	0.0047663	0.0000000	0.0033946
CACYBP	1.3006887	1.3200612	1.4916723	1.1919717	0.9666715

MRPS14	0.3625804	0.3086695	0.2982658	0.4062334	0.2801651
TNN	0.0031936	0.0023045	0.0000000	0.0043997	0.0000000
KIAA0040	0.0122164	0.0088808	0.0024971	0.0117078	0.0088315
ENSG00000260990	0.0000000	0.0000000	0.0000000	0.0000000	0.0000000
TNR	0.1450453	0.2740152	0.2623142	0.0961847	0.5318358
LINC02803	0.0062431	0.0099694	0.0095366	0.0051980	0.0210932
COP1	0.3856163	0.4446544	0.4686149	0.3728041	0.8582051
COP1-DT	0.0156936	0.0344117	0.0259069	0.0198617	0.0889492
PAPPA2	0.0160097	0.0264093	0.0300519	0.0147322	0.0799068
ENSG00000286754	0.0000000	0.0000000	0.0000000	0.0000000	0.0042206
ASTN1	0.1469230	0.1983435	0.1842884	0.1840542	0.6886496
BRINP2	0.0212352	0.0354853	0.0280752	0.0225635	0.0933152
LINC01645	0.0000000	0.0000000	0.0000000	0.0000000	0.0000000
ENSG00000227579	0.0015269	0.0031087	0.0000000	0.0032517	0.0072839
SEC16B	0.0055562	0.0079089	0.0000000	0.0058498	0.0184637
RASAL2-AS1	0.0030482	0.0093559	0.0187809	0.0046761	0.0000000
RASAL2	0.2669628	0.3614149	0.4067956	0.2679045	0.8679099
CLEC20A	0.0000000	0.0000000	0.0000000	0.0000000	0.0030127
TEX35	0.0000000	0.0032194	0.0017896	0.0000000	0.0000000
C1orf220	0.0140617	0.0200756	0.0206422	0.0357655	0.0309004
ENSG00000273384	0.0266696	0.0166942	0.0146714	0.0131659	0.0070919
RALGPS2-AS1	0.0043611	0.0044044	0.0032121	0.0135038	0.0000000
RALGPS2	1.2416649	0.9099992	0.7600719	1.2687387	1.0205002
ANGPTL1	0.0416432	0.0125034	0.0116265	0.0681254	0.0341707
FAM20B	0.2553820	0.2418741	0.2599235	0.2445469	0.2644488
TOR3A	0.1388199	0.1274082	0.0795199	0.1879095	0.0444018
ABL2	0.1503119	0.2036711	0.2057531	0.1462857	0.3764444
SOAT1	0.0845360	0.0831528	0.0964833	0.1581170	0.1149839
AXDND1	0.0140354	0.0156732	0.0027419	0.0186274	0.0167742
TDRD5	0.0015660	0.0068373	0.0000000	0.0000000	0.0000000
FAM163A	0.0054986	0.0131035	0.0012344	0.0127203	0.0443208
LINC02818	0.0000000	0.0028902	0.0000000	0.0106890	0.0000000
TOR1AIP2	0.5383878	0.4816950	0.4441089	0.5652774	0.3974099
ENSG00000272906	0.0069450	0.0094517	0.0138707	0.0037863	0.0047492
TOR1AIP1	0.4139291	0.2609222	0.1822102	0.4691988	0.2225763
ENSG00000261831	0.0012875	0.0000000	0.0000000	0.0000000	0.0145164
ENSG00000260360	0.0010034	0.0019543	0.0013609	0.0000000	0.0000000
CEP350	0.5618595	0.6081214	0.5649687	0.4128670	0.8219175
QSOX1	0.1047633	0.0951204	0.0675658	0.1875708	0.1466114
LHX4	0.0380592	0.0504582	0.0331187	0.0058620	0.0914566
ACBD6	0.5831024	0.6844252	0.7095416	0.6182082	0.7869733
OVAAL	0.0000000	0.0000000	0.0000000	0.0000000	0.0000000
XPR1	0.7675083	0.9018123	1.2795120	0.8715147	1.1915897
KIAA1614	0.0021099	0.0051696	0.0022183	0.0076666	0.0218931
ENSG00000243155	0.0044830	0.0055216	0.0040637	0.0000000	0.0507264
KIAA1614-AS1	0.0015408	0.0013745	0.0000000	0.0064453	0.0000000
STX6	0.2669371	0.2802959	0.2153294	0.2783632	0.2851668
MR1	0.0021372	0.0000000	0.0020178	0.0000000	0.0000000
ENSG00000289589	0.0005872	0.0006710	0.0010481	0.0000000	0.0000000
IER5	0.1891719	0.0938906	0.0744786	0.2025840	0.1108036
LINC01732	0.0126559	0.0064821	0.0007564	0.0081919	0.0000000
CACNA1E	0.1991906	0.2888760	0.3141244	0.1531108	0.7816966
ZNF648	0.0000000	0.0013426	0.0000000	0.0000000	0.0000000

LINC01344	0.0016555	0.0000000	0.0000000	0.0000000	0.0118968
ENSG00000289573	0.0022573	0.0021486	0.0000000	0.0000000	0.0000000
GLUL	1.1010385	0.7783934	0.5628663	0.9932808	0.7035226
RGSL1	0.0000000	0.0014023	0.0000000	0.0023036	0.0033679
RNASEL	0.1276438	0.0815714	0.0679884	0.1145474	0.0593014
RGS16	0.0347121	0.0418666	0.0382538	0.0465028	0.0331347
LINC01686	0.0021008	0.0023815	0.0000000	0.0010887	0.0000000
RGS8	0.0086319	0.0135808	0.0351550	0.0096031	0.0048241
NPL	0.0129133	0.0131684	0.0186948	0.0215569	0.0468981
DHX9-AS1	0.0970130	0.1217500	0.0691958	0.0439161	0.0776805
DHX9	0.7472962	0.6943346	0.8258433	0.6976262	0.7491889
SHCBP1L	0.0021131	0.0000000	0.0000000	0.0000000	0.0088300
LAMC1	0.1721743	0.1082539	0.1144082	0.3193544	0.2140287
LAMC2	0.0028224	0.0014079	0.0027049	0.0038691	0.0065348
NMNAT2	0.2568255	0.5817757	0.6209591	0.2077617	0.7883548
ENSG00000287929	0.0000000	0.0013955	0.0018766	0.0000000	0.0091052
ENSG00000286372	0.0000000	0.0000000	0.0000000	0.0000000	0.0000000
SMG7-AS1	0.0040198	0.0080515	0.0000000	0.0000000	0.0062037
SMG7	0.3247341	0.3355127	0.3283347	0.3185920	0.4500335
NCF2	0.0020810	0.0000000	0.0000000	0.0000000	0.0016673
ARPC5	1.3324397	1.5449944	1.7048536	1.4053405	1.1754607
RGL1	0.1078576	0.0920881	0.0716692	0.1205189	0.2921537
APOBEC4	0.0000000	0.0000000	0.0000000	0.0000000	0.0046953
ENSG00000289581	0.0000000	0.0013131	0.0000000	0.0000000	0.0000000
COLGALT2	0.1535467	0.1393808	0.1323473	0.2761865	0.1810395
TSEN15	0.3663838	0.3558638	0.3862987	0.3696787	0.2632404
ENSG00000286655	0.0000000	0.0000000	0.0017569	0.0000000	0.0043359
C1orf21-DT	0.0022092	0.0031749	0.0062587	0.0052270	0.0071690
C1orf21	0.3980085	0.5735211	0.6344981	0.3861158	0.7254821
ENSG00000285847	0.0000000	0.0022158	0.0000000	0.0000000	0.0235670
ENSG00000286378	0.0000000	0.0000000	0.0000000	0.0000000	0.0000000
EDEM3	0.2673020	0.3006767	0.3230193	0.3054835	0.3296435
NIBAN1	0.1248256	0.0671995	0.0576643	0.1355829	0.0815145
RNF2	0.3390716	0.4164665	0.5038135	0.2631067	0.3941793
TRMT1L	0.1575230	0.1797410	0.1596765	0.0990033	0.1672479
SWT1	0.1332121	0.1462061	0.1411241	0.1484520	0.1872255
IVNS1ABP	0.2708343	0.3296541	0.4128699	0.2321565	0.3576719
ENSG00000273004	0.0036360	0.0018545	0.0073685	0.0107837	0.0055381
LINC01350	0.0008875	0.0008821	0.0000000	0.0000000	0.0000000
ENSG00000261729	0.0000000	0.0000000	0.0000000	0.0000000	0.0000000
HMCN1	0.0022826	0.0097937	0.0000000	0.0041721	0.0085030
PRG4	0.0000000	0.0005430	0.0000000	0.0000000	0.0000000
TPR	1.0785368	1.0296002	0.9741672	0.9344208	1.0906613
ODR4	0.2042224	0.1670508	0.1810725	0.2153737	0.2715596
PDC-AS1	0.0423398	0.0403515	0.0268311	0.0294863	0.1885426
PDC	0.0000000	0.0000000	0.0000000	0.0000000	0.0121928
ENSG00000273198	0.0000000	0.0000000	0.0000000	0.0000000	0.0000000
PTGS2	0.0000000	0.0036850	0.0023415	0.0020032	0.0077097
PLA2G4A	0.0046394	0.0000000	0.0012574	0.0070440	0.0000000
LINC01036	0.0004962	0.0000000	0.0014006	0.0000000	0.0140933
BRINP3	0.1208368	0.1942181	0.1339647	0.1526654	0.5951495
ENSG00000225811	0.0000000	0.0041478	0.0025528	0.0042453	0.0231675
BRINP3-DT	0.0173896	0.0335930	0.0338069	0.0151490	0.0187195

ENSG00000241505	0.0151633	0.0170126	0.0185421	0.0173722	0.0122457
ENSG00000285638	0.0084459	0.0036098	0.0127980	0.0233368	0.0071757
ENSG00000285280	0.0803429	0.0692036	0.0383521	0.0863352	0.1598751
RGS21	0.0000000	0.0021865	0.0000000	0.0000000	0.0067404
RGS1	0.0499550	0.0282268	0.0051501	0.0328322	0.0111629
RGS2	0.2161335	0.2710219	0.2151535	0.1564615	0.2652013
UCHL5	0.4401807	0.5227969	0.5887806	0.3756696	0.4094942
RO60	1.1125568	0.9502904	0.9296875	1.0773211	0.9657889
GLRX2	0.1977367	0.2378797	0.2319564	0.1867075	0.1902612
CDC73	0.6353195	0.4423553	0.3891132	0.6168641	0.4585694
B3GALT2	0.0715053	0.1263354	0.2178185	0.0504834	0.0930894
LINC01031	0.0000000	0.0000000	0.0013691	0.0000000	0.0000000
ENSG00000286285	0.0000000	0.0000000	0.0000000	0.0000000	0.0052273
ENSG00000227240	0.0029790	0.0025721	0.0000000	0.0064027	0.0000000
KCNT2	0.3335093	0.2983407	0.2839618	0.2963139	0.9137851
CFH	0.0147187	0.0105657	0.0037248	0.0181101	0.0000000
CFHR1	0.0169482	0.0613829	0.0230849	0.0203442	0.0081396
CFHR4	0.0000000	0.0023683	0.0000000	0.0000000	0.0000000
ASPM	0.0039557	0.0191637	0.0000000	0.0147305	0.0251797
ZBTB41	0.5124829	0.5704576	0.6531911	0.4719304	0.5741939
ENSG00000290127	0.0056395	0.0064405	0.0184431	0.0132727	0.0164125
CRB1	0.0708272	0.1319122	0.0485331	0.0700512	0.1408949
DENND1B	0.1861507	0.2183348	0.1749422	0.1367010	0.3669018
ENSG00000224901	0.0000000	0.0000000	0.0000000	0.0000000	0.0088834
C1orf53	0.1471842	0.1126645	0.0803419	0.1326108	0.0915234
NEK7	0.3624866	0.2597121	0.1653694	0.3754778	0.3057255
PTPRC	0.0067709	0.0006921	0.0000000	0.0000000	0.0037529
MIR181A1HG	0.0725878	0.0734161	0.0446638	0.0748662	0.2989659
NR5A2	0.0009612	0.0000000	0.0000000	0.0049937	0.0000000
LINC00862	0.0041543	0.0029548	0.0137785	0.0076293	0.0023981
ENSG00000230623	0.0054644	0.0020534	0.0000000	0.0000000	0.0140580
ZNF281	0.1677638	0.2625437	0.2303539	0.1781251	0.2118673
KIF14	0.0279753	0.0189287	0.0112446	0.0146707	0.0133799
DDX59	0.1615183	0.1451931	0.1600359	0.1705949	0.1718443
DDX59-AS1	0.0566556	0.0405816	0.0234304	0.0410844	0.0351307
CAMSAP2	0.6354211	0.9121292	0.9660001	0.6172242	1.0342469
INAVA	0.0008089	0.0000000	0.0037378	0.0000000	0.0000000
KIF21B	0.1966563	0.3738314	0.6202771	0.1883186	0.4655763
ENSG00000229191	0.0075385	0.0156399	0.0248635	0.0091337	0.0085042
ENSG00000234132	0.0000000	0.0052979	0.0025324	0.0000000	0.0041641
CACNA1S	0.0013602	0.0000000	0.0051757	0.0000000	0.0000000
ASCL5	0.0023434	0.0035843	0.0000000	0.0000000	0.0000000
TMEM9	0.5546316	0.5513136	0.6163450	0.7110522	0.4823727
TNNI1	0.0365187	0.0767315	0.1062122	0.0257167	0.0526433
PHLDA3	0.3012712	0.3033068	0.2950534	0.2711888	0.2223882
CSRP1	0.2576180	0.2593691	0.1932262	0.2251242	0.2019395
CSRP1-AS1	0.0308494	0.0318974	0.0115034	0.0347130	0.0129272
NAV1	0.2790451	0.4871486	0.6358524	0.1787643	0.8030386
IPO9-AS1	0.0321131	0.0125596	0.0231952	0.0163674	0.1029941
ENSG00000235121	0.0041650	0.0000000	0.0040934	0.0000000	0.0000000
IPO9	0.4356012	0.4547946	0.4423274	0.3727829	0.5057143
SHISA4	0.3035003	0.3696283	0.4049430	0.2892953	0.2707795
ENSG00000223774	0.0023140	0.0020619	0.0009751	0.0025239	0.0106644

LMOD1	0.0033545	0.0073445	0.0019337	0.0106113	0.0161411
TIMM17A	0.3998039	0.4803813	0.5107477	0.3632932	0.3344496
RNPEP	0.2198058	0.1501090	0.1113200	0.2334474	0.1268809
ELF3-AS1	0.0000000	0.0019701	0.0000000	0.0000000	0.0095267
ELF3	0.0007669	0.0000000	0.0018539	0.0000000	0.0102678
GPR37L1	0.0745214	0.0261472	0.0258214	0.1860401	0.0398427
ARL8A	0.9617084	1.1544439	1.2586055	1.0070146	0.8491404
PTPN7	0.0000000	0.0000000	0.0000000	0.0035629	0.0000000
LGR6	0.0026010	0.0000000	0.0023088	0.0000000	0.0000000
UBE2T	0.1486357	0.2261672	0.3753654	0.1766166	0.1909666
PPP1R12B	0.3543368	0.3805179	0.2826943	0.3293521	0.5114604
SYT2	0.0213072	0.0235783	0.0445403	0.0000000	0.0587023
ENSG00000226862	0.0007096	0.0024632	0.0025528	0.0000000	0.0017206
KDM5B	0.8377340	0.7785003	0.9187710	0.8131127	0.9152289
PCAT6	0.1858935	0.1702954	0.1732024	0.1429413	0.1115202
ENSG00000291234	0.0119674	0.0187708	0.0211497	0.0017393	0.0374887
RABIF	0.1507794	0.1861247	0.2022467	0.1148887	0.1395200
KLHL12	0.1726652	0.2018226	0.2240059	0.1596234	0.1882870
ADIPOR1	0.6181393	0.4888108	0.4541048	0.5966983	0.4484738
CYB5R1	0.5358386	0.3258045	0.2306885	0.6139971	0.2566598
TMEM183A	0.5672317	0.7617911	0.8137242	0.5315338	0.5660046
PPFIA4	0.1191662	0.1441845	0.1650727	0.0866004	0.2946412
ADORA1	0.0211562	0.0359590	0.0386314	0.0133405	0.0512164
ENSG00000234775	0.0004832	0.0025492	0.0000000	0.0000000	0.0000000
CHIT1	0.0000000	0.0000000	0.0000000	0.0000000	0.0000000
LINC01353	0.0043154	0.0024704	0.0017210	0.0000000	0.0187906
ENSG00000288925	0.0005444	0.0008310	0.0000000	0.0000000	0.0035962
BTG2-DT	0.0000000	0.0000000	0.0031919	0.0000000	0.0000000
BTG2	0.5658765	0.2949837	0.2112597	0.4456978	0.3676952
FMOD	0.0046552	0.0038780	0.0000000	0.0069393	0.0037496
PRELP	0.0018381	0.0000000	0.0000000	0.0048478	0.0000000
ATP2B4	0.2601146	0.2093906	0.2183952	0.2627424	0.4234083
LAX1	0.0000000	0.0000000	0.0000000	0.0000000	0.0000000
ZBED6	0.0035169	0.0031589	0.0006832	0.0000000	0.0067404
ZC3H11A	0.0151706	0.0238947	0.0292546	0.0160820	0.0267373
SNRPE	0.8404051	0.8579868	0.8424841	0.8339043	0.6718547
ENSG00000286383	0.0017535	0.0000000	0.0017146	0.0000000	0.0000000
ENSG00000286572	0.0010841	0.0000000	0.0015850	0.0018234	0.0090880
SOX13	0.0344306	0.0302906	0.0228075	0.0703976	0.0929051
ETNK2	0.2480938	0.4197029	0.4964506	0.2545398	0.2708955
REN	0.0008382	0.0000000	0.0013677	0.0053651	0.0000000
KISS1	0.0006826	0.0000000	0.0117124	0.0000000	0.0000000
GOLT1A	0.0227297	0.0241641	0.0569261	0.0228864	0.0192755
PLEKHA6	0.1779349	0.1971537	0.2004018	0.0969685	0.4308850
ENSG00000231691	0.0027527	0.0011535	0.0000000	0.0000000	0.0119800
PPP1R15B-AS1	0.0016487	0.0061700	0.0058887	0.0029331	0.0144120
PPP1R15B	0.3048740	0.2832492	0.3018041	0.3151376	0.2615118
ENSG00000288934	0.0102507	0.0092327	0.0032804	0.0068144	0.0136996
PIK3C2B	0.0567929	0.0748106	0.1040654	0.0190199	0.1581785
MDM4	0.3587249	0.3681181	0.4105694	0.2527341	0.5192958
ENSG00000240710	0.0023436	0.0000000	0.0031125	0.0000000	0.0015072
LRRN2	0.1904341	0.1378312	0.1391041	0.4284081	0.2128453
ENSG00000287197	0.0007867	0.0000000	0.0027814	0.0000000	0.0056926

NFASC	0.2339513	0.2613328	0.2224103	0.2783301	0.6989135
CNTN2	0.0334969	0.0543315	0.0799147	0.0212402	0.0592960
TMEM81	0.0086737	0.0159073	0.0077674	0.0062739	0.0073347
RBBP5	0.1372928	0.1571261	0.1349461	0.1067418	0.1866676
DSTYK	0.2158173	0.2351641	0.1908574	0.2186286	0.2237505
TMCC2	0.0798142	0.1504688	0.1279972	0.0742634	0.1285214
NUAK2	0.0338465	0.0154254	0.0101171	0.0414452	0.0180870
KLHDC8A	0.0408676	0.0639731	0.0889766	0.0533351	0.0730458
LEMD1-AS1	0.0009158	0.0000000	0.0015819	0.0000000	0.0000000
LEMD1	0.0527351	0.1077628	0.1505740	0.0607380	0.1191251
BLACAT1	0.0069311	0.0303209	0.0140711	0.0000000	0.0350196
LEMD1-DT	0.0099679	0.0204889	0.0469459	0.0026800	0.0056033
CDK18	0.0207634	0.0214106	0.0116610	0.0225405	0.0380031
MFSD4A	0.0228668	0.0542283	0.0632649	0.0147343	0.0606925
ELK4	0.0946142	0.1174767	0.0667999	0.0555083	0.1172918
SLC45A3	0.0000000	0.0000000	0.0000000	0.0067541	0.0000000
NUCKS1	1.9238088	1.8760954	1.8928070	1.8602440	1.6228182
RAB29	0.1007880	0.0615668	0.0372941	0.1509309	0.0894282
SLC41A1	0.2473347	0.1543190	0.1073677	0.1931417	0.1795982
ENSG00000286619	0.0478719	0.0654547	0.0629161	0.0396715	0.1386059
PM20D1	0.0000000	0.0012653	0.0000000	0.0000000	0.0030832
RHEX	0.0069636	0.0076586	0.0206875	0.0055470	0.0030546
FAM72A	0.0011213	0.0018274	0.0000000	0.0115467	0.0031923
SRGAP2	0.2542834	0.2686561	0.2934467	0.2858881	0.3442650
IKBKE	0.0129117	0.0086459	0.0000000	0.0118698	0.0017069
IKBKE-AS1	0.0000000	0.0000000	0.0000000	0.0000000	0.0000000
RASSF5	0.1539240	0.1007935	0.0555670	0.1129513	0.1014437
EIF2D	0.2528208	0.1716316	0.1059550	0.2184165	0.1719148
DYRK3-AS1	0.0207136	0.0052256	0.0222682	0.0346138	0.0031293
DYRK3	0.3411482	0.1968430	0.1873003	0.3341839	0.1612680
MAPKAPK2	0.5268881	0.3225475	0.2027825	0.5122867	0.2797297
IL19	0.0000000	0.0013552	0.0000000	0.0000000	0.0057461
FCMR	0.0270895	0.0069436	0.0118351	0.0183115	0.0400614
PFKFB2	0.0523220	0.0410469	0.0308318	0.0328437	0.0208712
YOD1	0.0998119	0.1389687	0.1535284	0.0782578	0.1602130
C4BPB	0.0000000	0.0000000	0.0000000	0.0000000	0.0000000
C4BPA	0.0015115	0.0014137	0.0000000	0.0000000	0.0034437
ENSG00000275392	0.0003167	0.0014527	0.0006832	0.0000000	0.0000000
LINC02942	0.0000000	0.0022004	0.0000000	0.0000000	0.0000000
CD55	0.1613113	0.1396961	0.1411110	0.1831400	0.1408751
CR2	0.0000000	0.0000000	0.0000000	0.0029331	0.0000000
CR1	0.0000000	0.0031125	0.0000000	0.0000000	0.0000000
CR1L	0.0009918	0.0014601	0.0036888	0.0093762	0.0053380
CD46	0.2879499	0.2710963	0.2267791	0.3580851	0.5515482
MIR29B2CHG	0.0312518	0.0243687	0.0357720	0.0212653	0.1873307
CD34	0.0007392	0.0000000	0.0000000	0.0000000	0.0000000
LINC02767	0.0016696	0.0015829	0.0054788	0.0000000	0.0000000
PLXNA2	0.1692294	0.1423274	0.1368790	0.2017430	0.4410434
ENSG00000287220	0.0000000	0.0000000	0.0000000	0.0000000	0.0000000
ENSG00000286198	0.0011538	0.0005680	0.0000000	0.0000000	0.0113931
LINC01717	0.0000000	0.0000000	0.0000000	0.0016235	0.0000000
CAMK1G	0.0222629	0.0456228	0.0749465	0.0140030	0.0754863
LAMB3	0.0107768	0.0137039	0.0467955	0.0029889	0.0203219

HSD11B1-AS1	0.0072842	0.0073406	0.0179588	0.0000000	0.0104610
G0S2	0.0682490	0.0924185	0.1034457	0.0628524	0.0164548
HSD11B1	0.0008285	0.0039615	0.0021412	0.0000000	0.0000000
TRAF3IP3	0.0000000	0.0000000	0.0000000	0.0000000	0.0000000
C1orf74	0.0196667	0.0184535	0.0164216	0.0145756	0.0405163
IRF6	0.0018063	0.0000000	0.0022436	0.0000000	0.0000000
UTP25	0.1206322	0.1042412	0.1025978	0.1336114	0.1057669
SYT14	0.2973965	0.3495634	0.3852746	0.2691155	0.6539940
ENSG00000287343	0.0000000	0.0000000	0.0000000	0.0000000	0.0048055
SERTAD4-AS1	0.0264955	0.0402070	0.0370100	0.0117043	0.0265407
SERTAD4	0.0224798	0.0337669	0.0355853	0.0377312	0.0344643
HHAT	0.0352027	0.0338752	0.0248174	0.0654149	0.0663419
ENSG00000287354	0.0008598	0.0000000	0.0000000	0.0000000	0.0000000
KCNH1	0.0587404	0.0542163	0.0564300	0.0320174	0.3106701
ENSG00000284376	0.0000000	0.0000000	0.0000000	0.0000000	0.0073662
RCOR3	0.4437289	0.4849355	0.5363012	0.4558116	0.5406806
TRAF5	0.0366067	0.0685693	0.0448536	0.0308570	0.0736810
ENSG00000287033	0.0072922	0.0011193	0.0000000	0.0036722	0.0059372
LINC00467	0.3769057	0.2856817	0.1834292	0.3761431	0.1955668
SLC30A1	0.0809832	0.0434919	0.0335568	0.1268688	0.0315621
NEK2	0.0121348	0.0107207	0.0069246	0.0100391	0.0019545
NEK2-DT	0.0180782	0.0039254	0.0061119	0.0095595	0.0035834
ENSG00000226868	0.0000000	0.0000000	0.0000000	0.0000000	0.0000000
LPGAT1	0.4476856	0.6042937	0.6808459	0.3956142	0.5980931
LPGAT1-AS1	0.0000000	0.0000000	0.0000000	0.0000000	0.0000000
INTS7	0.0786345	0.0597780	0.1044013	0.0713214	0.1466464
DTL	0.0093451	0.0058393	0.0000000	0.0405863	0.0069201
LINC02608	0.0030874	0.0000000	0.0009177	0.0048117	0.0293816
PPP2R5A	0.2227366	0.1323193	0.0892219	0.2152562	0.1308971
ENSG00000234915	0.0052210	0.0037477	0.0018325	0.0020012	0.0065149
PACC1	0.1317414	0.1157383	0.1952227	0.2207339	0.1435584
NENF	0.9358440	0.8663369	0.7437192	0.9190558	0.6922332
ENSG00000286213	0.0009100	0.0000000	0.0000000	0.0000000	0.0158831
ENSG00000260805	0.0642504	0.1180873	0.1518404	0.0659797	0.0513304
ENSG00000288007	0.0014486	0.0009954	0.0000000	0.0000000	0.0060977
ATF3	0.5441780	0.3472625	0.2035159	0.4112269	0.3484648
LINC02773	0.0014039	0.0018178	0.0000000	0.0025305	0.0069651
BATF3	0.0105524	0.0290290	0.0159153	0.0180366	0.0038839
NSL1	0.5383383	0.4566823	0.4901125	0.5077330	0.4780119
TATDN3	0.2281546	0.2067359	0.1979135	0.1657000	0.2510831
SPATA45	0.0025586	0.0041203	0.0000000	0.0100721	0.0105957
FLVCR1-DT	0.0396272	0.0394397	0.0204403	0.0477703	0.0430102
FLVCR1	0.0868631	0.1176285	0.1250139	0.1098905	0.2445146
VASH2	0.0857452	0.2560255	0.2867615	0.0834273	0.2207493
ANGEL2	0.2395101	0.2145486	0.1719665	0.2838806	0.2437497
RPS6KC1	0.2618473	0.3042568	0.3344090	0.2302814	0.5185093
ENSG00000225233	0.0063211	0.0020781	0.0000000	0.0000000	0.0000000
ENSG00000228255	0.0035148	0.0000000	0.0029952	0.0044064	0.0000000
PROX1-AS1	0.0280187	0.0380370	0.0210140	0.0262469	0.0295656
PROX1	0.3728068	0.4932758	0.1303415	0.3224036	0.2740017
LINC02775	0.0008172	0.0000000	0.0000000	0.0000000	0.0000000
SMYD2	0.2448193	0.2839590	0.3100922	0.2694695	0.3083136
ENSG00000228470	0.0000000	0.0000000	0.0000000	0.0000000	0.0000000

PTPN14	0.0906774	0.0459770	0.0350083	0.0846460	0.0744341
CENPF	0.1241991	0.1062038	0.0425571	0.0873272	0.0576095
KCNK2	0.0163422	0.0268007	0.0415235	0.0098641	0.0682739
KCTD3	0.2848353	0.3108290	0.3077267	0.2712258	0.3505201
USH2A	0.0028543	0.0034910	0.0021716	0.0000000	0.0013524
ESRRG	0.1740058	0.1814043	0.1359318	0.1447735	0.4193165
GPATCH2	0.3191194	0.2769010	0.2421212	0.3167634	0.3752696
SPATA17	0.5797983	0.3893747	0.2642771	0.4959071	0.4660614
RRP15	0.3353162	0.3074632	0.3672202	0.2950952	0.2481533
TGFB2-AS1	0.0155294	0.0031088	0.0015315	0.0159839	0.0091716
TGFB2	0.1721237	0.0919673	0.0763167	0.2714613	0.1845604
LINC02869	0.0006008	0.0040134	0.0000000	0.0000000	0.0073662
LYPLAL1-DT	0.0221436	0.0208667	0.0158315	0.0188099	0.0377504
LYPLAL1	0.5474028	0.4344371	0.4653282	0.5532623	0.4713433
ENSG00000277007	0.0017918	0.0000000	0.0000000	0.0000000	0.0048215
ENSG00000287676	0.0040886	0.0057916	0.0033662	0.0000000	0.0000000
LYPLAL1-AS1	0.0017687	0.0037921	0.0000000	0.0000000	0.0033906
ZC3H11B	0.0010230	0.0000000	0.0000000	0.0000000	0.0000000
SLC30A10	0.0839058	0.0849035	0.0885136	0.0953812	0.1016217
EPRS1	1.1712683	1.0880286	1.1745674	1.1227299	0.9334477
BPNT1	0.1167935	0.0938004	0.1063577	0.1206249	0.1250655
IARS2	0.4366515	0.4368978	0.4352230	0.4952680	0.3729204
RAB3GAP2	0.3428895	0.3822064	0.3803864	0.3246789	0.5784357
MARK1	0.3483606	0.5642229	0.6010267	0.3153019	0.9083472
C1orf115	0.0036706	0.0169892	0.0189552	0.0191845	0.0140673
MTARC2	0.0469348	0.0470323	0.0403860	0.0709859	0.0535700
MTARC1	0.0591135	0.0607656	0.0402621	0.0906387	0.0650190
RNU6ATAC35P	0.0000000	0.0019268	0.0007063	0.0021653	0.0000000
LINC01352	0.0000000	0.0000000	0.0000000	0.0000000	0.0000000
HLX	0.0000000	0.0000000	0.0000000	0.0028151	0.0000000
DUSP10	0.2983812	0.1502140	0.0806877	0.3221892	0.1989808
ENSG00000236230	0.0041840	0.0016179	0.0180203	0.0072420	0.0094562
ENSG00000276997	0.0014224	0.0011107	0.0000000	0.0000000	0.0000000
HHIPL2	0.0000000	0.0000000	0.0000000	0.0029435	0.0030106
TAF1A	0.0946388	0.1117268	0.0893827	0.1104073	0.1227182
TAF1A-AS1	0.0390170	0.0614650	0.0675799	0.0599644	0.0386068
MIA3	0.4464648	0.4994324	0.6128985	0.6624299	0.5599908
ENSG00000272750	0.0079907	0.0118765	0.0045980	0.0032282	0.0127597
AIDA	0.5076961	0.4976917	0.4342531	0.5147847	0.3499919
BROX	0.5070677	0.3760624	0.3739538	0.5336075	0.3524265
FAM177B	0.0011075	0.0000000	0.0000000	0.0000000	0.0000000
ENSG00000289880	0.0000000	0.0012859	0.0013879	0.0019740	0.0148995
DISP1	0.1912367	0.1701700	0.2000659	0.1618590	0.4319970
ENSG00000287338	0.0000000	0.0000000	0.0000000	0.0000000	0.0000000
TLR5	0.0049447	0.0121887	0.0145348	0.0062439	0.0131743
SUSD4	0.1059265	0.1602221	0.2079350	0.0899565	0.2926160
CAPN8	0.0022646	0.0000000	0.0000000	0.0000000	0.0000000
CAPN2	0.5262246	0.4020575	0.3319421	0.5271865	0.4354449
TP53BP2	0.3654556	0.2634811	0.2034892	0.3541748	0.2149320
ENSG00000288999	0.0172030	0.0134426	0.0124809	0.0139396	0.0080291
ENSG00000291068	0.0185297	0.0207416	0.0146729	0.0238744	0.0270488
ENSG00000290989	0.0338755	0.0343786	0.0307551	0.0256425	0.0617569
FBXO28	0.1129846	0.1618419	0.0900247	0.1070652	0.1548557

DEGS1	0.3386281	0.2540850	0.2661452	0.4550103	0.2631637
ENSG00000237101	0.0100244	0.0048141	0.0081614	0.0046541	0.0051309
NVL	0.2482896	0.2235088	0.1996524	0.2359687	0.3062267
CNIH4	0.3782045	0.3065599	0.2725641	0.3871168	0.2864150
WDR26	0.3300971	0.3268004	0.2848600	0.3169767	0.4192532
CNIH3	0.2089114	0.2449728	0.2453895	0.2207425	0.3214259
CNIH3-AS2	0.0055641	0.0089103	0.0000000	0.0000000	0.0017501
ENSG00000286174	0.0000000	0.0000000	0.0000000	0.0000000	0.0000000
LINC02813	0.0013027	0.0006303	0.0050207	0.0026290	0.0058600
ENSG00000286719	0.0004918	0.0022814	0.0026996	0.0000000	0.0000000
DNAH14	0.2985678	0.4064841	0.3858473	0.2465585	0.6761833
LBR	0.2171690	0.2044921	0.2099767	0.2166385	0.3634677
LINC02765	0.0000000	0.0013844	0.0000000	0.0000000	0.0000000
ENAH	1.3261791	1.3039693	1.0696826	1.4068619	1.2959124
ENSG00000289602	0.0099286	0.0048209	0.0053316	0.0054668	0.0038318
ENSG00000227496	0.0000000	0.0000000	0.0000000	0.0000000	0.0000000
ENSG00000226349	0.0000000	0.0000000	0.0000000	0.0000000	0.0094307
SRP9	1.5612968	1.4831974	1.4940835	1.5768974	1.2644290
EPHX1	0.4978494	0.3527797	0.2836376	0.7130826	0.2956637
ENSG00000242861	0.0186416	0.0102446	0.0184586	0.0232117	0.0196152
TMEM63A	0.0451804	0.0414435	0.0104596	0.0584012	0.0466180
LEFTY1	0.0021355	0.0052035	0.0032780	0.0000000	0.0000000
PYCR2	0.7606106	0.5253683	0.4342916	0.8216732	0.4584791
ENSG00000248322	0.0000000	0.0000000	0.0000000	0.0000000	0.0000000
LEFTY2	0.0057984	0.0123773	0.0078222	0.0037692	0.0000000
SDE2	0.1275368	0.1206934	0.1158415	0.1828452	0.1141290
ENSG00000289341	0.0055388	0.0036957	0.0069869	0.0000000	0.0188468
H3-3A-DT	0.0312610	0.0188543	0.0241639	0.0368827	0.0305909
H3-3A	2.5183842	2.4262428	2.5232686	2.5253149	2.0784459
LINC01703	0.0123306	0.0152561	0.0157321	0.0090802	0.0360904
ACBD3	0.8383996	0.6833875	0.6424683	0.8202556	0.5965934
ACBD3-AS1	0.0010931	0.0000000	0.0000000	0.0000000	0.0059126
MIXL1	0.0013595	0.0038493	0.0083051	0.0000000	0.0000000
LIN9	0.0309020	0.0243273	0.0280289	0.0371695	0.0591921
PARP1	0.7509234	0.6346001	0.5644989	0.8775880	0.5267017
STUM	0.0178065	0.0154804	0.0054962	0.0097007	0.0181432
ITPKB	0.2341622	0.1066793	0.0935235	0.2794729	0.1257576
ITPKB-AS1	0.0015973	0.0004452	0.0000000	0.0000000	0.0000000
PSEN2	0.0957767	0.1015761	0.1145937	0.0959401	0.1169933
COQ8A	0.0500704	0.0333554	0.0276642	0.0434966	0.0565234
CDC42BPA	0.7801587	0.7904145	0.7491920	0.7050659	1.2856492
ENSG00000287532	0.0021917	0.0017791	0.0024455	0.0000000	0.0156482
ENSG00000228625	0.0000000	0.0000000	0.0000000	0.0000000	0.0038216
LINC01641	0.0015340	0.0000000	0.0000000	0.0018234	0.0000000
ZNF678	0.1599536	0.1854966	0.1945366	0.1385272	0.2462687
SNAP47	0.2170790	0.2545512	0.2680139	0.2050858	0.2225846
JMJD4	0.0897311	0.0678348	0.0656947	0.0918595	0.0946393
ENSG00000286389	0.0008372	0.0099824	0.0100986	0.0036552	0.0000000
PRSS38	0.0000000	0.0000000	0.0000000	0.0000000	0.0000000
WNT9A	0.0492597	0.0448485	0.0305245	0.0424185	0.0926569
WNT3A	0.0000000	0.0012157	0.0017517	0.0000000	0.0000000
ARF1	1.6215506	1.5718317	1.6228954	1.6075957	1.3364875
C1orf35	0.2756986	0.2019517	0.1835789	0.2352839	0.2005017

MRPL55	0.3537236	0.3686875	0.3704692	0.3481761	0.2990575
GUK1	1.5142377	1.5854166	1.5823550	1.4271114	1.3279402
GJC2	0.0128843	0.0083839	0.0121175	0.0069294	0.0218399
IBA57-DT	0.0026421	0.0041642	0.0063032	0.0000000	0.0047917
IBA57	0.0427562	0.0381811	0.0170108	0.0388866	0.0706153
OBSCN-AS1	0.0013122	0.0030452	0.0074520	0.0016235	0.0137285
OBSCN	0.0311066	0.0367966	0.0293569	0.0233886	0.1384734
ENSG00000269934	0.0012258	0.0005777	0.0068469	0.0000000	0.0222781
ENSG00000286773	0.0000000	0.0010918	0.0000000	0.0000000	0.0000000
ENSG00000287315	0.0000000	0.0000000	0.0000000	0.0000000	0.0000000
ENSG00000270104	0.0000000	0.0000000	0.0000000	0.0000000	0.0000000
TRIM11	0.0887179	0.0711247	0.0796646	0.0565744	0.0991958
ENSG00000231563	0.0157011	0.0101238	0.0098384	0.0081938	0.0237248
TRIM17	0.0039519	0.0020775	0.0092292	0.0000000	0.0154849
H2AC25	0.3468625	0.3585082	0.3007517	0.2706864	0.2348192
H2BC26	0.0189222	0.0182434	0.0168548	0.0037378	0.0328565
RNF187	1.1925381	1.1725399	1.2419481	1.0683318	0.9445758
RHOU	0.1268511	0.1485701	0.1659165	0.1530645	0.1538269
LINC02814	0.0009083	0.0000000	0.0032557	0.0000000	0.0000000
ENSG00000233920	0.0036877	0.0000000	0.0022998	0.0027265	0.0030988
RAB4A-AS1	0.0251483	0.0469824	0.0438294	0.0298635	0.0416828
RAB4A	0.9671012	0.9801694	1.1180921	0.9500574	0.8397698
ENSG00000237481	0.0011203	0.0000000	0.0000000	0.0000000	0.0088433
CCSAP	0.1814139	0.2611844	0.2915892	0.1952335	0.1949695
ACTA1	0.0012073	0.0051790	0.0013377	0.0041811	0.0000000
NUP133	0.2937292	0.2427519	0.1702706	0.2484666	0.2457555
NUP133-DT	0.0000000	0.0012926	0.0000000	0.0000000	0.0000000
ABCB10	0.0732058	0.0723806	0.0673871	0.0520666	0.1502626
TAF5L	0.0595650	0.0968907	0.0845118	0.0595247	0.0722284
URB2	0.0328027	0.0212472	0.0198720	0.0400074	0.0242121
GALNT2	0.4339097	0.2946825	0.3238792	0.5955516	0.4563126
ENSG00000227006	0.0012875	0.0010722	0.0000000	0.0000000	0.0120413
ENSG00000224407	0.0007634	0.0000000	0.0007240	0.0053329	0.0017206
PGBD5	0.0828038	0.1223146	0.1549221	0.0732814	0.2856473
ENSG00000282564	0.0043144	0.0133755	0.0180984	0.0081526	0.0152599
COG2	0.2174738	0.1494654	0.0845220	0.2109789	0.1474968
AGT	0.6195816	0.3460764	0.2482769	1.7842643	0.4161532
ENSG00000244137	0.0000000	0.0011324	0.0000000	0.0000000	0.0000000
CAPN9	0.0083486	0.0038111	0.0000000	0.0048763	0.0000000
C1orf198	0.1633482	0.1449112	0.1174276	0.1555782	0.0596342
ENSG00000223393	0.0080493	0.0147329	0.0134775	0.0050055	0.0015171
ENSG00000288037	0.0000000	0.0000000	0.0075196	0.0000000	0.0000000
ENSG00000287395	0.0000000	0.0000000	0.0000000	0.0000000	0.0078956
TTC13	0.1361913	0.1665041	0.2430425	0.1276119	0.3102158
ARV1	0.2173696	0.2543598	0.2366639	0.1693761	0.1894208
FAM89A	0.0061076	0.0115335	0.0079825	0.0000000	0.0314290
TRIM67	0.0949251	0.1873357	0.1918297	0.0679395	0.1834113
C1orf131	0.2975700	0.2368759	0.2022706	0.3223256	0.2583801
GNPAT	0.3034457	0.2911858	0.1970900	0.2820664	0.2232438
EXOC8	0.0895607	0.1096032	0.1137127	0.0790577	0.0632632
SPRTN	0.1065180	0.0632600	0.0666118	0.1528849	0.0420904
EGLN1	0.4605384	0.6143570	0.5936966	0.4393266	0.5464424
ENSG00000233461	0.0173636	0.0295806	0.0048655	0.0151858	0.0209364

TSNAX	0.3802992	0.3355283	0.3508993	0.3929932	0.3351418
LINC00582	0.0000000	0.0000000	0.0000000	0.0000000	0.0000000
DISC1	0.0399675	0.0352633	0.0168557	0.0470932	0.0989518
ENSG00000286071	0.0000000	0.0011339	0.0000000	0.0000000	0.0000000
SIPA1L2	1.5265676	1.1033037	1.0976412	1.6962512	1.2561458
LINC01744	0.0000000	0.0000000	0.0019944	0.0050414	0.0000000
MAP10	0.0515112	0.0626195	0.0627864	0.0803684	0.0606937
ENSG00000286774	0.0000000	0.0000000	0.0008856	0.0000000	0.0000000
NTPCR	0.5557141	0.4844958	0.3709132	0.6093796	0.3291912
PCNX2	0.2087127	0.2558983	0.1865383	0.1860052	0.6831398
MAP3K21	0.0279305	0.0831324	0.0569771	0.0271576	0.1023510
ENSG00000289305	0.0000000	0.0000000	0.0050096	0.0000000	0.0033906
KCNK1	0.0043455	0.0073948	0.0165217	0.0098863	0.0061045
SLC35F3	0.0563800	0.1205439	0.0886034	0.0433984	0.5381659
ENSG00000236358	0.0000000	0.0041893	0.0055401	0.0139967	0.0000000
ENSG00000273367	0.0003904	0.0000000	0.0000000	0.0015620	0.0000000
COA6-AS1	0.0418711	0.0325819	0.0199672	0.0217120	0.0262672
COA6	0.3573526	0.3347727	0.3519201	0.3490839	0.2435354
TARBP1	0.1266190	0.1562815	0.1110696	0.1076321	0.4158633
LINC01354	0.0389946	0.0169644	0.0026891	0.0612514	0.0194895
ENSG00000288760	0.0028074	0.0000000	0.0024897	0.0000000	0.0000000
ENSG00000230404	0.0000000	0.0000000	0.0000000	0.0000000	0.0000000
IRF2BP2	0.9369850	0.7486026	0.7350807	0.8626722	0.6632691
ENSG00000228830	0.0103258	0.0047168	0.0009535	0.0042232	0.0108583
ENSG00000228044	0.0000000	0.0000000	0.0029995	0.0000000	0.0000000
ENSG00000230628	0.0024301	0.0022258	0.0000000	0.0000000	0.0000000
ENSG00000282097	0.0000000	0.0012690	0.0000000	0.0000000	0.0000000
LINC01132	0.0000000	0.0047954	0.0000000	0.0000000	0.0041410
ENSG00000286263	0.0038467	0.0034400	0.0000000	0.0027513	0.0169302
LNCATV	0.0038854	0.0005904	0.0000000	0.0025302	0.0058520
LINC01348	0.0000000	0.0008741	0.0000000	0.0000000	0.0000000
ENSG00000273416	0.0025874	0.0060524	0.0000000	0.0000000	0.0038927
TOMM20	1.5071806	1.4624210	1.3489866	1.5091315	1.2541428
RBM34	0.0597904	0.0967502	0.0995657	0.0397157	0.0868393
ARID4B	0.7007471	0.6320606	0.6074785	0.6564793	0.9003598
ENSG00000289114	0.0000000	0.0000000	0.0015717	0.0000000	0.0000000
GGPS1	0.5255599	0.5717776	0.6484795	0.4905358	0.4643451
TBCE	0.1601443	0.1785344	0.1362325	0.1470313	0.1692805
B3GALNT2	0.0997533	0.0702625	0.0432889	0.1005559	0.1019055
GNG4	0.6978771	1.2411820	1.5739166	0.6562444	0.9788843
LYST	0.2311269	0.3296056	0.3434788	0.2204299	0.6244451
LINC02768	0.0002608	0.0000000	0.0000000	0.0000000	0.0000000
NID1	0.0262687	0.0202560	0.0201147	0.0110167	0.0695560
GPR137B	0.2270927	0.1706280	0.1228895	0.2785264	0.2275091
ERO1B	0.0601979	0.0453466	0.0344478	0.0799091	0.0945529
EDARADD	0.0169359	0.0330743	0.0377511	0.0068081	0.0154677
LGALS8	0.2016307	0.2172901	0.3099908	0.1971938	0.2030599
LGALS8-AS1	0.0007820	0.0009802	0.0049279	0.0000000	0.0022413
ENSG00000273058	0.0037746	0.0000000	0.0051371	0.0000000	0.0000000
ENSG00000230325	0.0144811	0.0143012	0.0050421	0.0062496	0.0100925
HEATR1	0.1716040	0.1543977	0.1468270	0.1417520	0.2221415
ACTN2	0.0122090	0.0123794	0.0105893	0.0071288	0.0585936
MTR	0.2707007	0.2268165	0.1993599	0.1797441	0.3444695

RYR2	0.1771913	0.1817572	0.1099123	0.1593949	0.5801851
ENSG00000289628	0.0000000	0.0000000	0.0000000	0.0000000	0.0000000
ENSG00000237250	0.0009247	0.0000000	0.0000000	0.0028219	0.0000000
ENSG00000234464	0.0061137	0.0000000	0.0000000	0.0000000	0.0014454
LINC01139	0.0088662	0.0011939	0.0018992	0.0019230	0.0059786
CHRM3	0.0724192	0.1038504	0.1643226	0.0789746	0.3979945
CHRM3-AS2	0.0000000	0.0021647	0.0037548	0.0000000	0.0130388
FMN2	0.2504781	0.2866219	0.2112490	0.2250704	0.7063207
ENSG00000233735	0.0006880	0.0011489	0.0000000	0.0000000	0.0052051
GREM2	0.0424332	0.0735563	0.1672258	0.0267906	0.1717919
ENSG00000230015	0.0000000	0.0000000	0.0000000	0.0000000	0.0180088
ENSG00000226919	0.0000000	0.0014288	0.0000000	0.0000000	0.0000000
RGS7	0.3129712	0.4649955	0.5217259	0.2752196	1.2820088
ENSG00000286496	0.0201778	0.0270700	0.0156358	0.0109483	0.0062217
ENSG00000287516	0.0156262	0.0162523	0.0237026	0.0124715	0.0228540
ENSG00000287738	0.0000000	0.0026869	0.0000000	0.0000000	0.0108185
FH	0.3512909	0.3462585	0.4537215	0.3619544	0.2881902
KMO	0.0035639	0.0011722	0.0027758	0.0000000	0.0000000
OPN3	0.1220494	0.0786544	0.0624923	0.1113300	0.0443121
CHML	0.3247808	0.6197795	0.5851736	0.2884808	0.4171000
ENSG00000287513	0.0018587	0.0036996	0.0067979	0.0036572	0.0000000
WDR64	0.0025795	0.0000000	0.0012235	0.0056296	0.0091243
ENSG00000288723	0.0132699	0.0063275	0.0027758	0.0091221	0.0017069
EXO1	0.0039397	0.0036572	0.0000000	0.0056255	0.0000000
MAP1LC3C	0.0085057	0.0023863	0.0034287	0.0072133	0.0101207
PLD5	0.1256044	0.1452807	0.1497269	0.2968881	0.3745486
ENSG00000272865	0.0004383	0.0000000	0.0000000	0.0000000	0.0059236
ENSG00000291216	0.0000000	0.0012534	0.0000000	0.0000000	0.0000000
LINC01347	0.0023652	0.0020573	0.0000000	0.0000000	0.0124423
CEP170	0.8802120	1.2826062	1.4824479	0.8117112	1.3047947
ENSG00000227230	0.0000000	0.0000000	0.0000000	0.0000000	0.0040799
SDCCAG8	0.5266572	0.5002379	0.4294854	0.5495721	0.6646546
AKT3	0.7598379	0.9970400	1.0228427	0.6920267	1.1911657
ENSG00000236031	0.0000000	0.0000000	0.0000000	0.0000000	0.0000000
AKT3-IT1	0.0009946	0.0000000	0.0000000	0.0000000	0.0033732
LINC02774	0.0025393	0.0064016	0.0040138	0.0000000	0.0000000
ENSG00000289055	0.0035571	0.0017184	0.0013773	0.0063954	0.0065199
ZBTB18	0.1706157	0.0918116	0.0674185	0.1901694	0.1358155
ENSG00000289439	0.0003565	0.0000000	0.0000000	0.0000000	0.0000000
C1orf100	0.0067039	0.0061736	0.0000000	0.0033339	0.0036030
ENSG00000240963	0.0261376	0.0337778	0.0075942	0.0031732	0.0547115
ADSS2	1.2644414	0.9467965	0.8961346	1.2040357	0.8349285
CATSPERE	0.0374885	0.0212290	0.0136680	0.0270907	0.0448828
DESI2	0.4635632	0.4030738	0.4005898	0.5213663	0.2563794
ENSG00000284188	0.0029317	0.0000000	0.0000000	0.0000000	0.0000000
ENSG00000287601	0.0026675	0.0013855	0.0011896	0.0000000	0.0000000
COX20	1.2423514	1.3141949	1.4324324	1.2308672	1.0849670
HNRNPU	1.4843236	1.3928558	1.4356933	1.4589955	1.3879570
ENSG00000273175	0.0078213	0.0094794	0.0055987	0.0051878	0.0107260
ENSG00000272195	0.0289480	0.0337621	0.0209288	0.0220559	0.0086890
EFCAB2	0.4440825	0.3341451	0.2480109	0.3331803	0.4546371
KIF26B	0.2741003	0.3038597	0.2977416	0.2392788	0.9461151
KIF26B-AS1	0.0298523	0.0116686	0.0193634	0.0228243	0.0383523

SMYD3	0.3711858	0.4306872	0.4471791	0.3708356	1.3052338
ENSG00000235096	0.0000000	0.0000000	0.0000000	0.0000000	0.0049014
SMYD3-AS1	0.0000000	0.0000000	0.0000000	0.0000000	0.0032553
TFB2M	0.0793487	0.0785681	0.0765218	0.1069259	0.0830797
CNST	0.2490143	0.3688241	0.3221854	0.2396086	0.3311702
ENSG00000225300	0.0000000	0.0000000	0.0012319	0.0000000	0.0000000
ENSG00000235021	0.0035005	0.0027789	0.0000000	0.0035916	0.0000000
SCCPDH	0.6665161	0.6138982	0.6827132	0.6687264	0.5336769
ENSG00000260855	0.0073435	0.0110628	0.0138233	0.0000000	0.0254840
LINC01341	0.0033979	0.0094771	0.0052762	0.0043115	0.0173906
AHCTF1	0.2417571	0.2651611	0.2307578	0.2096018	0.4110574
ZNF695	0.0083702	0.0173770	0.0164877	0.0200255	0.0342405
ZNF670	0.0212267	0.0499977	0.0626447	0.0423709	0.0879931
ZNF669	0.1002969	0.0880070	0.0818501	0.0562525	0.0840706
ENSG00000286747	0.0075843	0.0095123	0.0122748	0.0135353	0.0064286
LINC02897	0.0057168	0.0098969	0.0042748	0.0240422	0.0000000
ZNF124	0.0770678	0.1037408	0.0915860	0.0554115	0.2079149
ENSG00000259865	0.0196582	0.0139511	0.0367081	0.0043253	0.0215839
ENSG00000290683	0.0139062	0.0186830	0.0139557	0.0142678	0.0149199
ENSG00000289566	0.0084720	0.0009785	0.0018156	0.0072693	0.0123548
ZNF496	0.1052942	0.1152745	0.1343332	0.0761215	0.1644907
ZNF496-DT	0.0513530	0.0856281	0.1278459	0.0716956	0.1265893
ENSG00000286216	0.0009625	0.0000000	0.0025278	0.0000000	0.0026235
NLRP3	0.0056576	0.0015854	0.0102648	0.0034589	0.0000000
ENSG00000235749	0.0003655	0.0010445	0.0007835	0.0080221	0.0090449
TRIM58	0.0280830	0.0705669	0.0866493	0.0076251	0.0345342
OR2T8	0.0010193	0.0078217	0.0140147	0.0000000	0.0077544
OR2AJ1	0.0018533	0.0000000	0.0000000	0.0000000	0.0077873
ENSG00000289367	0.0012680	0.0000000	0.0041025	0.0031580	0.0233282
OR2L8	0.0000000	0.0000000	0.0013814	0.0000000	0.0000000
OR2AK2	0.0000000	0.0000000	0.0000000	0.0000000	0.0000000
ENSG00000290612	0.0025662	0.0028347	0.0019465	0.0041872	0.0173659
OR2L5	0.0113664	0.0124660	0.0073230	0.0000000	0.0419913
OR2L2	0.0062766	0.0089648	0.0014452	0.0000000	0.0195171
OR2L3	0.0016830	0.0047647	0.0032705	0.0000000	0.0266092
OR2L13	0.0071013	0.0167544	0.0240958	0.0053003	0.0105234
OR2M3	0.0010600	0.0010034	0.0027227	0.0031909	0.0000000
OR2M4	0.0018956	0.0014148	0.0101179	0.0000000	0.0178943
SH3BP5L	0.0640866	0.0723165	0.0823481	0.0740247	0.0426526
ZNF672	0.0992144	0.0980952	0.1176207	0.0964849	0.0689352
ZNF692	0.0565516	0.0609674	0.0653221	0.0659899	0.0766667
ZNF692-DT	0.0014910	0.0000000	0.0000000	0.0000000	0.0016028
PGBD2	0.0345752	0.0349453	0.0389178	0.0406084	0.0788255
FAM110C	0.0016582	0.0032393	0.0121619	0.0082653	0.0127761
SH3YL1	0.3029174	0.2819804	0.2882742	0.3247821	0.2635919
ACP1	0.7925112	0.8048112	0.8699605	0.7666669	0.6377886
ALKAL2	0.0063295	0.0102870	0.0012960	0.0055429	0.0033094
ENSG00000228643	0.0035416	0.0014664	0.0053989	0.0020012	0.0167753
ENSG00000235779	0.0000000	0.0008286	0.0054273	0.0000000	0.0000000
LINC01865	0.0017408	0.0000000	0.0000000	0.0000000	0.0000000
TMEM18	0.4170230	0.3717977	0.3890812	0.4216907	0.2688053
TMEM18-DT	0.0019570	0.0000000	0.0000000	0.0000000	0.0021475
LINC01115	0.0179426	0.0121624	0.0072263	0.0108555	0.0066258

ENSG00000223751	0.0174950	0.0278487	0.0152586	0.0000000	0.0204059
SNTG2-AS1	0.0049695	0.0052605	0.0035629	0.0071285	0.0145919
SNTG2	0.0454317	0.0303859	0.0334806	0.0313477	0.1872874
ENSG00000231482	0.0030707	0.0000000	0.0030256	0.0000000	0.0000000
TPO	0.0025979	0.0013691	0.0000000	0.0000000	0.0027080
ENSG00000228613	0.0000000	0.0000000	0.0000000	0.0000000	0.0049005
ENSG00000203635	0.0008757	0.0012516	0.0000000	0.0000000	0.0085580
PXDN	0.1930364	0.1788251	0.1476491	0.1773650	0.3938108
MYT1L	0.3949156	0.7619148	1.0196352	0.3547460	1.4461512
ENSG00000284600	0.0000000	0.0000000	0.0000000	0.0000000	0.0077818
ENSG00000232057	0.0000000	0.0000000	0.0000000	0.0000000	0.0000000
MYT1L-AS1	0.0010608	0.0019502	0.0000000	0.0000000	0.0137764
ENSG00000289941	0.0000000	0.0000000	0.0000000	0.0000000	0.0000000
ENSG00000290112	0.0048078	0.0030111	0.0085079	0.0020674	0.0056964
ENSG00000234929	0.0013379	0.0012812	0.0000000	0.0000000	0.0000000
ENSG00000237720	0.0033328	0.0005433	0.0018775	0.0000000	0.0000000
LINC01250	0.0107958	0.0139267	0.0089240	0.0024915	0.0734498
ENSG00000236760	0.0029307	0.0005980	0.0022314	0.0025471	0.0240376
EIPR1	0.1855097	0.2097965	0.1994641	0.1812886	0.3234100
TRAPPC12	0.1856273	0.1568668	0.1523424	0.1877470	0.2767203
TRAPPC12-AS1	0.0040123	0.0126612	0.0047415	0.0042145	0.0000000
ENSG00000271868	0.0000000	0.0000000	0.0000000	0.0000000	0.0000000
ADI1	0.1687547	0.1283222	0.0874361	0.2931549	0.1435788
ENSG00000235078	0.0131324	0.0035852	0.0170509	0.0074344	0.0170251
ENSG00000242282	0.0156557	0.0183838	0.0385355	0.0168882	0.0057461
RNASEH1	0.1991107	0.2799097	0.3256142	0.2046066	0.1948753
RNASEH1-DT	0.2087091	0.1674520	0.1573700	0.1764413	0.1965943
ENSG00000287126	0.0190437	0.0355211	0.0388299	0.0427859	0.0199108
RPS7	2.5929939	2.5771791	2.5400589	2.5456950	2.3355271
COLEC11	0.0044858	0.0151946	0.0053824	0.0038410	0.0145126
ENSG00000289136	0.0017662	0.0027018	0.0000000	0.0054747	0.0060726
ALLC	0.0000000	0.0042856	0.0025449	0.0000000	0.0045428
DCDC2C	0.0019082	0.0029829	0.0000000	0.0000000	0.0014182
ENSG00000291325	0.0000000	0.0000000	0.0000000	0.0000000	0.0000000
LINC01248	0.0003816	0.0000000	0.0000000	0.0000000	0.0027882
ENSG00000230090	0.0018429	0.0013933	0.0000000	0.0011543	0.0000000
SOX11	0.9005988	1.2967630	1.6519782	0.7465510	1.3537643
ENSG00000242540	0.0294393	0.0493516	0.0635627	0.0207681	0.0723632
ENSG00000236106	0.0046167	0.0027054	0.0044009	0.0000000	0.0000000
SILC1	0.0598357	0.1116411	0.1224878	0.0137550	0.0655582
LINC00487	0.0000000	0.0000000	0.0015587	0.0000000	0.0052886
NRIR	0.0000000	0.0051073	0.0029863	0.0000000	0.0051936
CMPK2	0.0300982	0.0260896	0.0555850	0.0115752	0.0230927
RSAD2	0.0037862	0.0054188	0.0000000	0.0000000	0.0088600
ENSG00000271947	0.0000000	0.0000000	0.0009009	0.0000000	0.0000000
GRASLND	0.0020161	0.0011785	0.0104034	0.0000000	0.0040093
RNF144A	0.0889087	0.1488099	0.2053098	0.0535548	0.3047273
ENSG00000223884	0.0009466	0.0013541	0.0000000	0.0000000	0.0030664
LINC02973	0.0000000	0.0000000	0.0000000	0.0000000	0.0000000
ENSG00000229727	0.0000000	0.0008342	0.0007240	0.0000000	0.0064970
LINC00299	0.0012783	0.0012671	0.0000000	0.0000000	0.0097812
LINC01814	0.0015256	0.0000000	0.0006373	0.0000000	0.0000000
ID2-AS1	0.0255084	0.0116480	0.0069310	0.0514668	0.0138688

ID2	0.2195780	0.1788560	0.2123693	0.2192366	0.3009824
KIDINS220	0.7922744	1.1216906	1.5013748	0.7753773	1.2449014
MBOAT2	0.5779818	0.5620925	0.6159782	0.4903400	0.8207211
ASAP2	0.3973495	0.3506849	0.3660843	0.4364971	0.5747861
ITGB1BP1	0.6077675	0.6527220	0.7029726	0.6609290	0.5204115
CPSF3	0.1677307	0.1591082	0.1508890	0.1794859	0.1887969
IAH1	0.5214431	0.5139865	0.5760730	0.5635867	0.4236443
ADAM17	0.3088664	0.2496531	0.2513131	0.5295595	0.4239010
ENSG00000239300	0.0067257	0.0131710	0.0063659	0.0045363	0.0075252
ENSG00000271855	0.0118648	0.0146307	0.0143293	0.0097438	0.0144743
YWHAQ	1.8201202	1.9903097	2.3649161	1.9157599	1.7013245
ENSG00000240687	0.0000000	0.0015912	0.0000000	0.0036343	0.0000000
ENSG00000243491	0.0054170	0.0053028	0.0000000	0.0136196	0.0000000
TAF1B	0.2514843	0.2361035	0.2582233	0.3327409	0.3014197
ENSG00000269973	0.0329591	0.0315165	0.0171737	0.0275071	0.0574140
ENSG00000287305	0.0008619	0.0000000	0.0000000	0.0000000	0.0000000
GRHL1	0.0275007	0.0223567	0.0255920	0.0232093	0.0528858
ENSG00000260077	0.0801294	0.0570476	0.0405168	0.0706692	0.0510154
KLF11	0.1710586	0.1932843	0.1403823	0.1533897	0.1759966
ENSG00000271787	0.0015119	0.0000000	0.0000000	0.0053087	0.0000000
CYS1	0.0593747	0.0343855	0.0340766	0.1019869	0.0349662
RRM2	0.0255429	0.0175138	0.0045732	0.0337663	0.0183788
ENSG00000285872	0.0134618	0.0087739	0.0056435	0.0158381	0.0128251
HPCAL1	0.3282549	0.5554028	0.7837544	0.3463345	0.4944267
ODC1	1.4994099	1.3806896	1.4140937	1.5095673	1.1143716
ODC1-DT	0.0246463	0.0200127	0.0098971	0.0309301	0.0000000
NOL10	0.2519063	0.2289598	0.1842690	0.2348647	0.3672897
RN7SL832P	0.0088380	0.0116898	0.0158037	0.0000000	0.0045939
ATP6V1C2	0.0117876	0.0181451	0.0067817	0.0207060	0.0391755
ENSG00000272275	0.0165409	0.0076551	0.0048750	0.0246859	0.0144411
PDIA6	1.6511068	1.2301055	1.0052816	2.0771520	1.1048229
LINC01954	0.0018377	0.0032477	0.0000000	0.0000000	0.0034081
KCNF1	0.0272883	0.0475697	0.0461134	0.0257259	0.0567658
ENSG00000145063	0.0032604	0.0020234	0.0048727	0.0037983	0.0000000
C2orf50	0.1182780	0.0942375	0.0510676	0.1577383	0.0758300
SLC66A3	0.1382138	0.1148603	0.0829672	0.2153234	0.0517933
ROCK2	0.5982955	0.5529630	0.5542477	0.5532327	0.6933906
LINC03037	0.0000000	0.0000000	0.0000000	0.0000000	0.0000000
LINC00570	0.0000000	0.0000000	0.0000000	0.0000000	0.0021475
ENSG00000285569	0.0005465	0.0034115	0.0000000	0.0000000	0.0000000
E2F6	0.2605020	0.2108795	0.1776738	0.2434021	0.1998313
GREB1	0.0786165	0.1271580	0.1998115	0.0919928	0.3488428
NTSR2	0.0000000	0.0008310	0.0031289	0.0000000	0.0000000
LPIN1	0.1240574	0.0934847	0.1017265	0.0840364	0.2372373
MIR3681HG	0.0041016	0.0007805	0.0006655	0.0020370	0.0046509
ENSG00000286427	0.0010446	0.0007312	0.0000000	0.0036897	0.0041410
TRIB2	0.5131009	0.7249206	1.1391673	0.5197915	0.7141814
ENSG00000225649	0.0108712	0.0155685	0.0078893	0.0000000	0.0604931
ENSG00000285876	0.0044462	0.0026097	0.0000000	0.0016936	0.0000000
LINC00276	0.0182517	0.0187424	0.0085986	0.0044674	0.1278085
LRATD1	0.0612230	0.1691712	0.2134438	0.0394400	0.1459767
ENSG00000287291	0.0054918	0.0102959	0.0141865	0.0085768	0.0399791
NBAS	0.4836150	0.5418193	0.4661985	0.4091515	0.9001605

ENSG00000234022	0.0000000	0.0012994	0.0000000	0.0072238	0.0000000
DDX1	0.6703706	0.6650446	0.6107156	0.6725491	0.6434219
LINC01804	0.0006008	0.0000000	0.0000000	0.0000000	0.0000000
MYCNOS	0.0197691	0.0174027	0.0220484	0.0505807	0.0652991
MYCN	0.1424371	0.1522536	0.1499983	0.1424833	0.0660334
ENSG00000236989	0.0047630	0.0000000	0.0013627	0.0000000	0.0000000
ENSG00000237633	0.0023331	0.0038190	0.0000000	0.0000000	0.0000000
CYRIA	0.2613113	0.3886037	0.4078207	0.2332231	0.3125117
RAD51AP2	0.0028289	0.0011099	0.0075197	0.0000000	0.0022372
VSNL1	0.2367225	0.5513603	0.9512863	0.2167916	0.4502842
SMC6	0.2556964	0.2563785	0.2827601	0.2416032	0.3574139
GEN1	0.0323016	0.0359157	0.0154561	0.0267181	0.0443796
KCNS3	0.0077203	0.0356821	0.0219936	0.0120899	0.0527593
ENSG00000287881	0.0000000	0.0011265	0.0000000	0.0026949	0.0023834
ENSG00000260331	0.0011441	0.0060155	0.0000000	0.0000000	0.0000000
RDH14	0.1898016	0.1795035	0.2201842	0.2141144	0.1301717
ENSG00000287284	0.0000000	0.0019162	0.0000000	0.0000000	0.0000000
LINC01376	0.0437559	0.0299287	0.0287234	0.0268792	0.0708903
LINC00954	0.0000000	0.0000000	0.0000000	0.0000000	0.0000000
TTC32	0.2674207	0.1885417	0.1568841	0.2557849	0.2503506
TTC32-DT	0.0033556	0.0047251	0.0019022	0.0000000	0.0000000
WDR35	0.1794772	0.1423282	0.1008444	0.1847143	0.1912849
WDR35-DT	0.0417429	0.0233339	0.0348194	0.0213692	0.0059953
MATN3	0.0108280	0.0100912	0.0035365	0.0139359	0.0126883
LAPTM4A	0.9652951	0.7410769	0.6575783	1.2191537	0.6740288
LAPTM4A-DT	0.0004899	0.0029231	0.0000000	0.0000000	0.0064270
SDC1	0.0310540	0.0650533	0.0550738	0.0467049	0.0598913
PUM2	0.4744707	0.4667401	0.4563948	0.4830452	0.7706525
RHOB	0.6915925	0.7320960	0.7668825	0.7634666	0.6552483
HS1BP3	0.1082418	0.0872201	0.0436175	0.1597854	0.1140445
ENSG00000269976	0.0016909	0.0016844	0.0020861	0.0000000	0.0000000
GDF7	0.0000000	0.0019049	0.0000000	0.0033262	0.0000000
ENSG00000270100	0.0005219	0.0010762	0.0028107	0.0000000	0.0000000
LDAH	0.1996340	0.1879036	0.1978857	0.2293522	0.2720502
ENSG00000233005	0.0023212	0.0032788	0.0000000	0.0011356	0.0073980
ENSG00000228999	0.0078905	0.0096867	0.0071044	0.0237821	0.0100587
LINC01830	0.0004650	0.0000000	0.0000000	0.0037761	0.0046614
ENSG00000232451	0.0085468	0.0087502	0.0059397	0.0130096	0.0339619
LINC02923	0.0013856	0.0019513	0.0000000	0.0000000	0.0000000
KLHL29	0.1294959	0.1301633	0.1021823	0.1274951	0.7996433
ENSG00000224361	0.0000000	0.0000000	0.0017606	0.0000000	0.0000000
ENSG00000283031	0.0012022	0.0013933	0.0000000	0.0000000	0.0012770
ATAD2B	0.2115616	0.2399805	0.2294332	0.1879738	0.5263380
UBXN2A	0.6069770	0.5240354	0.4704917	0.6173110	0.5205184
MFSD2B	0.0041815	0.0079957	0.0014901	0.0023531	0.0169371
WDCP	0.0771143	0.0951015	0.1133012	0.1071647	0.0567055
FKBP1B	0.5234547	0.7281369	0.9026848	0.5275541	0.4968493
SF3B6	1.0861146	0.9744191	0.9528247	1.1096366	0.7448980
FAM228B	0.6181713	0.6690900	0.5982709	0.5503003	0.6300931
TP53I3	0.0547147	0.0398057	0.0171123	0.0871093	0.0349487
PFN4	0.0387648	0.0264819	0.0060127	0.0361243	0.0481135
ENSG00000232642	0.0000000	0.0010546	0.0000000	0.0000000	0.0037236
FAM228A	0.0223157	0.0281581	0.0181786	0.0175665	0.0719882

ITSN2	0.2065258	0.2244651	0.1739204	0.2299652	0.3979681
ENSG00000242628	0.0138603	0.0152028	0.0096322	0.0037228	0.0061302
NCOA1	0.4404179	0.4956184	0.4272106	0.3941134	0.9808764
PTRHD1	0.3823530	0.3882518	0.3053008	0.3780849	0.3780975
CENPO	0.0410856	0.0452031	0.0315765	0.0321445	0.0726534
ADCY3	0.0442237	0.0554011	0.0664498	0.0436605	0.1381324
DNAJC27	0.1021995	0.1516322	0.1767560	0.0728273	0.1792775
DNAJC27-AS1	0.1496368	0.1442047	0.1280308	0.1338636	0.1784393
ENSG00000286645	0.0012738	0.0012699	0.0000000	0.0000000	0.0038198
EFR3B	0.3060869	0.3632441	0.4398368	0.2720904	0.3938915
POMC	0.0000000	0.0000000	0.0000000	0.0000000	0.0048680
LINC01381	0.0017687	0.0000000	0.0000000	0.0000000	0.0000000
DNMT3A	0.2990446	0.2862523	0.3054917	0.2786652	0.4155864
ARNILA	0.0027959	0.0131073	0.0026108	0.0061093	0.0481812
DTNB	0.2273765	0.3101317	0.2653108	0.1537440	0.7150272
DTNB-AS1	0.0014925	0.0000000	0.0000000	0.0000000	0.0017069
ASXL2	0.2232320	0.1653434	0.1278869	0.2468658	0.2624003
KIF3C	0.2833132	0.4655734	0.5452475	0.2044842	0.5001905
RAB10	1.0626952	1.1243201	1.2709372	1.1187367	0.9734561
GAREM2	0.2355918	0.2604615	0.3315675	0.2352398	0.2414613
HADHA	1.0664548	0.9198404	0.7720777	1.1324252	0.7667834
HADHB	0.6794248	0.4654956	0.3062264	0.7331634	0.4025516
ENSG00000286707	0.0046121	0.0049774	0.0019093	0.0129958	0.0134507
ADGRF3	0.0116656	0.0219157	0.0180784	0.0093800	0.0169524
SELENOI	0.1965753	0.2485418	0.2820606	0.1908423	0.3178316
DRC1	0.6245173	0.3849070	0.2528539	0.5665070	0.3814655
OTOF	0.0051894	0.0046587	0.0014457	0.0050409	0.0066722
FAM166C	0.0167687	0.0132122	0.0098529	0.0096024	0.0174929
CIB4	0.0072751	0.0050359	0.0024008	0.0017424	0.0336002
KCNK3	0.1082536	0.2255805	0.3700319	0.0833470	0.2377117
SLC35F6	0.1798507	0.1398021	0.1101700	0.2228380	0.1062173
CENPA	0.0313047	0.0346434	0.0295003	0.0352896	0.0612369
DPYSL5	0.4193012	0.6915291	0.8871601	0.3364995	0.7345425
ENSG00000230286	0.0048310	0.0170752	0.0141577	0.0027524	0.0121722
MAPRE3	0.7284636	0.8512256	1.0126948	0.6924202	0.6361817
MAPRE3-AS1	0.0000000	0.0000000	0.0000000	0.0000000	0.0039649
TMEM214	0.0714806	0.0593865	0.0527208	0.0934007	0.0962397
AGBL5	0.1824327	0.1933268	0.2169595	0.2246442	0.1777776
AGBL5-AS1	0.0010931	0.0018959	0.0036584	0.0035369	0.0000000
ENSG00000272056	0.0022766	0.0035200	0.0099590	0.0000000	0.0036568
ENSG00000272148	0.0047761	0.0011149	0.0019839	0.0000000	0.0043337
OST4	1.7514503	1.5967438	1.5382216	1.7828352	1.4397657
EMILIN1	0.0124816	0.0068733	0.0050926	0.0394135	0.0046590
KHK	0.0723622	0.1095867	0.1074948	0.0811268	0.0730794
CGREF1	0.0853728	0.1407396	0.2252725	0.0858932	0.1812023
ABHD1	0.0118895	0.0040617	0.0025863	0.0188782	0.0017703
PREB	0.1466690	0.1375430	0.1538958	0.1073107	0.1128197
SLC5A6	0.0383852	0.0524837	0.0757730	0.0285046	0.0514717
ATRAID	1.1642904	0.8464963	0.7097051	1.3021330	0.7454152
CAD	0.0471456	0.0325869	0.0277935	0.0475764	0.0690997
SLC30A3	0.0000000	0.0000000	0.0009118	0.0000000	0.0169977
DNAJC5G	0.0000000	0.0018009	0.0000000	0.0000000	0.0000000
TRIM54	0.0000000	0.0028873	0.0013268	0.0000000	0.0017986

UCN	0.0136965	0.0222019	0.0171145	0.0172950	0.0165647
MPV17	0.3905318	0.3037582	0.2920427	0.4085329	0.2586313
GTF3C2	0.0732981	0.0856663	0.0675328	0.0795586	0.0944250
GTF3C2-AS1	0.0020035	0.0000000	0.0040418	0.0000000	0.0015171
GTF3C2-AS2	0.0150486	0.0140378	0.0100617	0.0032828	0.0260695
EIF2B4	0.1441661	0.1429198	0.1332153	0.1346554	0.0769051
SNX17	0.4529788	0.3575543	0.3529692	0.4875702	0.3148590
ZNF513	0.0951761	0.0927188	0.0715301	0.1007264	0.0429657
PPM1G	0.9500784	1.0208149	1.0521352	1.0539934	0.8203149
NRBP1	0.5708663	0.5544121	0.5580191	0.6105044	0.4934856
KRTCAP3	0.0040379	0.0043934	0.0011764	0.0000000	0.0000000
IFT172	0.0994903	0.1140268	0.0882336	0.0885985	0.1904053
FNDC4	0.2936383	0.4886041	0.4393332	0.3393796	0.3469880
GCKR	0.0012665	0.0045314	0.0055587	0.0023091	0.0042647
C2orf16	0.0084746	0.0000000	0.0013406	0.0067482	0.0275120
ZNF512	0.3973791	0.4196008	0.4566390	0.3480196	0.3391381
CCDC121	0.1232785	0.0802028	0.0474948	0.1491409	0.0629675
GPN1	0.2000754	0.1522108	0.1746145	0.1600640	0.1385799
SUPT7L	0.2174959	0.1969820	0.2057709	0.2294278	0.2757642
SLC4A1AP	0.4061835	0.3897193	0.4379871	0.3816806	0.3533215
LINC01460	0.0000000	0.0041371	0.0000000	0.0000000	0.0080881
ENSG00000289326	0.0046403	0.0096496	0.0157644	0.0038158	0.0034249
MRPL33	0.8087850	0.7707817	0.7949146	0.7660148	0.6784265
RBKS	0.1357925	0.0796141	0.1004699	0.1181212	0.1014769
BABAM2	0.3110267	0.2530183	0.2351264	0.2952550	0.5076872
ENSG00000223522	0.0017542	0.0018537	0.0046641	0.0000000	0.0113384
FOSL2-AS1	0.0093075	0.0064080	0.0022652	0.0041924	0.0035317
FOSL2	0.1027894	0.0665420	0.0324723	0.0763486	0.0833473
ENSG00000270640	0.0010727	0.0000000	0.0000000	0.0031240	0.0021475
ENSG00000270210	0.0012615	0.0044155	0.0000000	0.0023370	0.0000000
PLB1	0.0130686	0.0179124	0.0151956	0.0025494	0.0178575
ENSG00000230730	0.0009223	0.0011906	0.0000000	0.0020682	0.0000000
PPP1CB-DT	0.0432984	0.0117389	0.0187464	0.0290736	0.0393014
PPP1CB	1.4699783	1.3214271	1.2842619	1.3494812	1.1845500
SPDYA	0.0207743	0.0190975	0.0091381	0.0121788	0.0538604
ENSG00000273233	0.0012779	0.0011729	0.0025567	0.0033415	0.0064270
TRMT61B	0.1548128	0.2140136	0.1921158	0.1490581	0.2074809
WDR43	0.3568457	0.3588383	0.3048776	0.3430693	0.4235795
TOGARAM2	0.0543975	0.0304457	0.0175377	0.0429666	0.0594191
PCARE	0.0008382	0.0000000	0.0000000	0.0000000	0.0076072
CLIP4	0.1981339	0.1891448	0.1630020	0.1606167	0.3688841
ALK	0.0999156	0.0917245	0.0719453	0.0579574	0.5489345
ENSG00000286963	0.0000000	0.0000000	0.0000000	0.0000000	0.0058440
ENSG00000288553	0.0033620	0.0005835	0.0000000	0.0081438	0.0000000
ENSG00000197644	0.0014076	0.0000000	0.0000000	0.0000000	0.0000000
ENSG00000233862	0.0111370	0.0047251	0.0074828	0.0052183	0.0264884
YPEL5	0.8402852	0.7240801	0.7441220	0.7724836	0.6413102
LBH	0.5434526	0.8176109	0.9301048	0.4307925	0.6421110
LINC01936	0.0022748	0.0070784	0.0015669	0.0104785	0.0000000
LCLAT1	0.1770914	0.1941778	0.1896295	0.2259585	0.4218633
CAPN13	0.0000000	0.0000000	0.0000000	0.0000000	0.0000000
ENSG00000285984	0.0000000	0.0000000	0.0000000	0.0000000	0.0000000
GALNT14	0.0145490	0.0258411	0.0333942	0.0099416	0.0535865

CAPN14	0.0023776	0.0054876	0.0000000	0.0013136	0.0000000
EHD3	0.0876234	0.0888693	0.0878156	0.0585953	0.0640106
XDH	0.0105676	0.0028215	0.0021716	0.0027037	0.0331186
SRD5A2	0.0036623	0.0000000	0.0000000	0.0000000	0.0000000
ENSG00000273165	0.0000000	0.0000000	0.0000000	0.0000000	0.0161633
MEMO1	0.0406918	0.0484344	0.0515709	0.0242678	0.0814098
DPY30	0.7485062	0.7437282	0.7688371	0.7232396	0.5907253
ENSG00000271228	0.0061631	0.0039645	0.0035363	0.0016163	0.0139261
ENSG00000288937	0.0014449	0.0038973	0.0076346	0.0069265	0.0000000
SPAST	0.5753369	0.5484772	0.5966309	0.5574503	0.5446632
SLC30A6-DT	0.0016976	0.0029359	0.0008906	0.0069402	0.0000000
SLC30A6	0.0784214	0.0762165	0.0833071	0.0923878	0.1650654
NLRC4	0.0008815	0.0050124	0.0000000	0.0000000	0.0076018
ENSG00000289727	0.0145970	0.0171857	0.0141276	0.0038158	0.0390738
YIPF4	0.4022141	0.3215001	0.3068596	0.4186957	0.3382752
ENSG00000272754	0.0003313	0.0000000	0.0000000	0.0000000	0.0000000
BIRC6	0.4663632	0.5173539	0.4934505	0.3514631	1.0070351
BIRC6-AS1	0.0000000	0.0000000	0.0000000	0.0020988	0.0000000
ENSG00000276334	0.0159598	0.0157287	0.0122147	0.0160999	0.0306952
ENSG00000276517	0.0016410	0.0083601	0.0031311	0.0035133	0.0093287
BIRC6-AS2	0.0042113	0.0089639	0.0000000	0.0045316	0.0404652
TTC27	0.1461669	0.1534019	0.1203064	0.1697685	0.2553638
LINC00486	0.7020748	0.7166387	0.6519359	0.6353235	0.9035138
ENSG00000236854	0.0018405	0.0000000	0.0000000	0.0000000	0.0000000
LTBP1	0.1555933	0.1141550	0.0641980	0.2272546	0.3355189
ENSG00000285577	0.0000000	0.0000000	0.0000000	0.0000000	0.0085359
RASGRP3	0.0129617	0.0034407	0.0074300	0.0223694	0.0113258
FAM98A	0.3264676	0.2633636	0.2660781	0.3325586	0.2205677
ENSG00000286415	0.0150096	0.0077465	0.0011369	0.0029435	0.0311600
LINC01320	0.0056571	0.0038936	0.0071164	0.0000000	0.0195746
CRIM1-DT	0.1300974	0.1925455	0.2703797	0.1408861	0.0985988
CRIM1	0.4285276	0.3453536	0.4667806	0.5893186	0.5206340
FEZ2	0.8050232	0.6187589	0.5098612	0.9341036	0.6175561
VIT	0.0011118	0.0000000	0.0000000	0.0048157	0.0000000
STRN	0.2558792	0.2345735	0.2163706	0.1678008	0.2832640
HEATR5B	0.2038794	0.2652062	0.3154258	0.1533082	0.3779132
GPATCH11	0.3352404	0.3784896	0.4257266	0.3418867	0.3259014
EIF2AK2	0.6129773	0.5278971	0.5107107	0.6354833	0.5134088
SULT6B1	0.0000000	0.0000000	0.0000000	0.0000000	0.0000000
CEBPZOS	0.8085707	0.7810153	0.7277422	0.7906319	0.6870675
CEBPZ	0.6773130	0.5914986	0.5663894	0.5920357	0.5167268
ENSG00000272054	0.0070211	0.0067634	0.0081447	0.0036139	0.0174712
NDUFAF7	0.1440677	0.1173228	0.1180342	0.1297619	0.1860504
PRKD3	0.1167137	0.0732719	0.0440009	0.1394697	0.1136767
ENSG00000285925	0.0005938	0.0022754	0.0000000	0.0000000	0.0000000
QPCT	0.0437993	0.0925620	0.1480727	0.0386055	0.1051826
ENSG00000287316	0.0000000	0.0000000	0.0000000	0.0000000	0.0072786
LINC03063	0.0022826	0.0000000	0.0015807	0.0000000	0.0030832
CDC42EP3-AS1	0.0021112	0.0000000	0.0000000	0.0000000	0.0000000
ENSG00000290100	0.0032374	0.0027172	0.0000000	0.0000000	0.0014498
CDC42EP3	0.4381528	0.4387572	0.3245994	0.3671010	0.2902980
RMDN2	0.0932142	0.0866042	0.0460072	0.0988862	0.0692656
RMDN2-AS1	0.0018273	0.0000000	0.0035241	0.0000000	0.0074506

CYP1B1	0.0097663	0.0094732	0.0076816	0.0080861	0.0063839
CYP1B1-AS1	0.0115943	0.0033264	0.0000000	0.0057109	0.0237769
ATL2	0.2013595	0.1663096	0.1959200	0.1539907	0.2115857
ENSG00000288994	0.0017592	0.0017326	0.0000000	0.0072319	0.0000000
LINC02613	0.0034831	0.0065473	0.0146471	0.0116664	0.0014580
HNRNPLL	0.4242320	0.3806352	0.4091274	0.4288007	0.3893625
GALM	0.1026451	0.0626656	0.0444035	0.1119315	0.0404317
SRSF7	0.5801999	0.5578074	0.5318546	0.5850674	0.5934931
GEMIN6	0.1547008	0.1396907	0.1549198	0.1625302	0.0690572
DHX57	0.1780525	0.2044362	0.2187522	0.1597770	0.3090069
MORN2	0.8300544	0.6865287	0.5531877	0.8489029	0.6001377
ARHGEF33	0.0369786	0.0699770	0.0628543	0.0390616	0.0524317
ENSG00000269210	0.0035759	0.0168048	0.0080094	0.0000000	0.0000000
SOS1	0.6429654	0.6431229	0.6111823	0.6548625	0.9626648
CDKL4	0.0165716	0.0089763	0.0184375	0.0104247	0.0431664
MAP4K3	0.3656944	0.4005218	0.3907402	0.3381029	0.7987929
ENSG00000273035	0.0010244	0.0011272	0.0000000	0.0000000	0.0049605
MAP4K3-DT	0.2843385	0.2728548	0.3399594	0.2894328	0.3060932
ENSG00000289003	0.0092344	0.0043699	0.0103228	0.0047877	0.0111578
TMEM178A	0.2313866	0.1715396	0.1548459	0.3509640	0.1872673
THUMPD2	0.2069923	0.1939278	0.1678433	0.2019747	0.2409270
SLC8A1-AS1	0.0575125	0.0919563	0.0873189	0.0708398	0.1999250
SLC8A1	0.8565578	1.1319967	1.2955639	0.7903738	1.8695950
ENSG00000289013	0.0051404	0.0057437	0.0006909	0.0068239	0.0417539
ENSG00000288992	0.0000000	0.0060055	0.0000000	0.0000000	0.0052691
LINC01913	0.0000000	0.0000000	0.0000000	0.0000000	0.0052452
PKDCC	0.0147872	0.0209220	0.0197593	0.0127765	0.0227841
EML4-AS1	0.0044011	0.0022258	0.0014810	0.0000000	0.0000000
EML4	0.2705704	0.2556764	0.2056322	0.2923317	0.3854550
COX7A2L	1.3135683	1.4575357	1.4975537	1.2124207	1.1559895
KCNG3	0.0097344	0.0211097	0.0032332	0.0036117	0.0244333
MTA3	0.3476871	0.3439213	0.2719594	0.3331355	0.3420110
HAAO	0.0010767	0.0000000	0.0028901	0.0000000	0.0195811
ENSG00000288886	0.0008619	0.0007437	0.0012348	0.0050634	0.0110938
ENSG00000289082	0.0008619	0.0000000	0.0000000	0.0039482	0.0128725
LINC01819	0.0007702	0.0000000	0.0000000	0.0000000	0.0015531
LINC02580	0.0490467	0.0163099	0.0096345	0.0313776	0.0282796
ENSG00000287387	0.0000000	0.0026266	0.0000000	0.0000000	0.0047891
ZFP36L2	0.4809939	0.3129648	0.1533755	0.5429503	0.3267560
ENSG00000234936	0.0115507	0.0097321	0.0044248	0.0163665	0.0000000
THADA	0.1707315	0.1306553	0.1043854	0.1297879	0.2969809
PLEKHH2	0.0618141	0.0410566	0.0386439	0.0513747	0.0820054
C1GALT1C1L	0.0075067	0.0075174	0.0063202	0.0073417	0.0093439
DYNC2LI1	0.4363156	0.3839689	0.3700004	0.4525912	0.4563504
ABCG5	0.0032910	0.0000000	0.0000000	0.0020601	0.0035216
ABCG8	0.0028980	0.0043791	0.0170209	0.0022544	0.0120598
LRPPRC	0.5753375	0.7688360	0.8385541	0.5882082	0.7891537
PPM1B-DT	0.0010310	0.0000000	0.0000000	0.0000000	0.0053413
PPM1B	0.2357219	0.2886423	0.3047273	0.2255085	0.3793716
ENSG00000289272	0.0000000	0.0000000	0.0027908	0.0033686	0.0101971
SLC3A1	0.0139340	0.0197282	0.0200227	0.0162623	0.0666475
PREPL	1.0940434	1.2609514	1.5221838	0.9791989	1.2083425
CAMKMT	0.1115344	0.1541704	0.1287539	0.0853048	0.4646414

LINC01121	0.0014711	0.0061720	0.0000000	0.0000000	0.0000000
SRBD1	0.2614413	0.2499379	0.1429563	0.3173431	0.3525795
PRKCE	0.2164020	0.4810959	0.3224921	0.2124905	0.9672104
EPAS1	0.0412513	0.0538498	0.0211911	0.0231526	0.1317454
ENSG00000253515	0.0000000	0.0000000	0.0000000	0.0000000	0.0000000
ATP6V1E2	0.0765581	0.0764518	0.0594808	0.0600742	0.1196427
RHOQ	0.7109233	0.6157265	0.5418048	0.7092541	0.5869158
RHOQ-AS1	0.0045478	0.0047907	0.0036262	0.0016673	0.0260064
PIGF	0.3318642	0.2763657	0.2702937	0.3774810	0.3074984
CRIPT	0.5098720	0.5382746	0.5489199	0.4064945	0.3834624
LINC01118	0.0059257	0.0046940	0.0012042	0.0062526	0.0099215
SOCS5	0.1880448	0.2005103	0.1783491	0.1482179	0.2215219
LINC01119	0.0015212	0.0052255	0.0015058	0.0023869	0.0097677
ENSG00000226548	0.0000000	0.0000000	0.0000000	0.0000000	0.0000000
ENSG00000228925	0.0011084	0.0032799	0.0000000	0.0000000	0.0000000
MCFD2	0.6315487	0.5297400	0.5319324	0.6920472	0.3644534
TTC7A	0.0809449	0.0626916	0.0627589	0.1142452	0.1915554
ENSG00000233845	0.0000000	0.0000000	0.0000000	0.0000000	0.0000000
STPG4	0.0062154	0.0149190	0.0099726	0.0040080	0.0263348
CALM2	3.0751423	3.2092649	3.3845442	3.0187419	2.7491173
EPCAM-DT	0.1138719	0.1257801	0.1095135	0.0881952	0.2909874
ENSG00000226087	0.0096627	0.0046349	0.0120956	0.0048134	0.0000000
EPCAM	0.1226659	0.1953162	0.3568008	0.1352672	0.1534785
MSH2	0.4288637	0.4083920	0.4253203	0.3831514	0.5119432
KCNK12	0.0629849	0.1318898	0.1768391	0.0465419	0.1100047
MSH6	0.4120499	0.3805839	0.3725213	0.3858465	0.3795130
FBXO11	0.5694381	0.5668920	0.5759847	0.4991053	0.8491849
ENSG00000233230	0.0149147	0.0220050	0.0061078	0.0201146	0.0105735
ENSG00000230773	0.0054218	0.0008451	0.0026961	0.0023540	0.0243428
FOXN2	0.1681616	0.1550002	0.1112981	0.1453139	0.2321934
PPP1R21-DT	0.0077618	0.0121226	0.0129503	0.0123684	0.0070172
PPP1R21	0.2787649	0.3212853	0.3485214	0.2713164	0.4713535
STON1	0.6016757	0.3507272	0.2120692	0.5976017	0.3599699
GTF2A1L	0.0238286	0.0160707	0.0125130	0.0159694	0.0230085
LHCGR	0.0019704	0.0005625	0.0024455	0.0000000	0.0046920
ENSG00000282890	0.0237123	0.0159685	0.0064415	0.0060874	0.0360880
FSHR	0.0041302	0.0014687	0.0000000	0.0000000	0.0000000
ENSG00000282998	0.0010663	0.0007774	0.0000000	0.0000000	0.0103619
NRXN1	0.9659538	1.2888705	1.5772184	0.7760027	2.4482364
ENSG00000285548	0.0000000	0.0000000	0.0000000	0.0000000	0.0031721
ENSG00000283058	0.0000000	0.0000000	0.0000000	0.0000000	0.0000000
ENSG00000286412	0.0016875	0.0000000	0.0000000	0.0000000	0.0049381
ENSG00000231918	0.0043805	0.0110165	0.0075152	0.0029828	0.0261456
ENSG00000228033	0.0009054	0.0026915	0.0032358	0.0099861	0.0000000
ASB3	0.3522840	0.3563974	0.3513565	0.4026038	0.5702430
CHAC2	0.0103418	0.0163490	0.0229341	0.0261860	0.0318382
ERLEC1	0.9516761	0.7456351	0.6666060	1.0734263	0.6479495
GPR75	0.0110098	0.0195117	0.0297397	0.0232635	0.0231429
PSME4	0.2387657	0.2343144	0.2118885	0.2614819	0.4879268
ACYP2	0.3921387	0.3569533	0.4299346	0.4541873	0.5139621
ENSG00000272156	0.0069657	0.0045182	0.0022845	0.0000000	0.0000000
ENSG00000289065	0.0228057	0.0185136	0.0236652	0.0173995	0.0156357
C2orf73	0.1301571	0.0642010	0.0542223	0.1254661	0.0677796

SPTBN1	1.3680914	1.5492571	1.5953312	1.2909105	1.5908348
SPTBN1-AS1	0.0026993	0.0008916	0.0025718	0.0000000	0.0164303
ENSG00000289627	0.0009319	0.0014335	0.0000000	0.0030679	0.0078747
SPTBN1-AS2	0.0012132	0.0000000	0.0020129	0.0000000	0.0019716
EML6	0.1867265	0.2342372	0.2089111	0.2541161	0.6030699
ENSG00000285519	0.0015642	0.0007963	0.0000000	0.0000000	0.0119700
RTN4	1.8329916	2.0440368	2.2545454	1.9521747	1.9593644
CLHC1	0.1696867	0.1664483	0.1578488	0.1377398	0.1319216
ENSG00000203327	0.0000000	0.0000000	0.0000000	0.0000000	0.0000000
RPS27A	2.9617145	2.9144225	2.7429504	2.8482597	2.6692139
ENSG00000289606	0.0039642	0.0048772	0.0059741	0.0000000	0.0000000
MTIF2	0.2969731	0.3162702	0.3160540	0.3154397	0.1927189
ENSG00000240401	0.0412855	0.0309298	0.0458143	0.0253813	0.0810039
CCDC88A	0.9012879	1.0762056	1.1108552	0.8845092	1.4654057
CFAP36	0.8663435	0.8287601	0.8379686	0.8866976	0.6954143
PPP4R3B	0.7714260	0.7678920	0.8196802	0.7816309	0.7880255
PPP4R3B-DT	0.0104604	0.0066551	0.0024144	0.0076235	0.0045428
PNPT1	0.2232368	0.2051250	0.2110442	0.2196508	0.2632687
EFEMP1	0.5344737	0.2673282	0.1290613	0.7725553	0.2227757
ENSG00000272180	0.0430776	0.0663088	0.0741732	0.0128989	0.0611274
MIR217HG	0.0400942	0.0980553	0.1087427	0.0598940	0.1645915
ENSG00000233251	0.0022447	0.0132503	0.0144052	0.0038283	0.0042989
CCDC85A	0.1271871	0.2672027	0.3244805	0.1383385	0.5334213
ENSG00000285755	0.0025772	0.0019686	0.0047216	0.0000000	0.0324560
VRK2	0.2653647	0.1560392	0.1107241	0.2783104	0.1610622
ENSG00000287875	0.0568465	0.0269080	0.0220801	0.0578744	0.0378003
FANCL	0.2739578	0.2267740	0.1814057	0.2634394	0.2457920
ENSG00000273063	0.0029920	0.0000000	0.0000000	0.0000000	0.0000000
LINC01122	0.0418116	0.0539965	0.0573922	0.0155137	0.1952689
ENSG00000289529	0.0038618	0.0076988	0.0021171	0.0012699	0.0080666
LINC01793	0.0017863	0.0007634	0.0133979	0.0000000	0.0020430
ENSG00000271955	0.0002480	0.0025843	0.0000000	0.0000000	0.0000000
MIR4432HG	0.0000000	0.0000000	0.0000000	0.0038537	0.0031863
BCL11A	0.0224259	0.0637550	0.0550721	0.0447640	0.0920586
PAPOLG	0.1522289	0.1707455	0.1849474	0.1427861	0.1972137
REL-DT	0.0172130	0.0078934	0.0152652	0.0025261	0.0163143
REL	0.1178250	0.1220824	0.1479955	0.0984349	0.1530536
PUS10	0.1125071	0.0550521	0.0730337	0.0927719	0.0531147
PEX13	0.2546851	0.2671963	0.3005254	0.2479522	0.2552790
SANBR	0.2355806	0.3780922	0.3292570	0.2059391	0.5561412
C2orf74-DT	0.2581974	0.2219903	0.2049080	0.2734245	0.1606878
C2orf74	0.4472434	0.4364322	0.4092241	0.3536660	0.3784442
C2orf74-AS1	0.0000000	0.0000000	0.0000000	0.0000000	0.0000000
USP34	0.7052376	0.8392556	0.8627277	0.7015701	1.0562411
ENSG00000273302	0.0000000	0.0000000	0.0000000	0.0000000	0.0000000
USP34-DT	0.0309250	0.0210509	0.0094198	0.0287742	0.0266932
XPO1	0.9430360	0.9151387	0.8965717	0.8329239	1.0618625
ENSG00000285857	0.0000000	0.0000000	0.0000000	0.0000000	0.0100515
ENSG00000289410	0.0053305	0.0087176	0.0019283	0.0036480	0.0568881
ENSG00000289855	0.0044367	0.0025518	0.0000000	0.0019166	0.0021475
FAM161A	0.4587459	0.4771432	0.4807182	0.4858899	0.3673598
CCT4	1.2352673	1.2281774	1.2485585	1.2009282	0.9730292
ENSG00000236498	0.0003402	0.0040333	0.0114823	0.0000000	0.0120238

ENSG00000286360	0.0040452	0.0012690	0.0000000	0.0010951	0.0000000
COMMD1	0.3401420	0.4146199	0.4346632	0.4010394	0.4594972
ENSG00000229839	0.0039857	0.0039552	0.0103011	0.0063924	0.0217152
B3GNT2	0.0545609	0.0629218	0.0760805	0.0708714	0.0569466
ENSG00000228541	0.0036977	0.0009229	0.0000000	0.0069683	0.0089032
TMEM17	0.1771218	0.1116465	0.0918611	0.2185654	0.1145464
ENSG00000226622	0.0112108	0.0038288	0.0000000	0.0077570	0.0051880
EHBP1	0.5508393	0.6484090	0.6120519	0.5131294	1.0306600
ENSG00000226605	0.0000000	0.0011874	0.0015036	0.0000000	0.0465098
EHBP1-AS1	0.0021023	0.0053922	0.0000000	0.0000000	0.0376750
OTX1	0.0000000	0.0000000	0.0000000	0.0000000	0.0000000
ENSG00000286480	0.0022253	0.0007387	0.0000000	0.0000000	0.0133599
WDPCP	0.2377914	0.2246438	0.1662703	0.2107477	0.5446188
MDH1	1.0074162	1.1493904	1.2982771	1.0230354	0.9203133
ENSG00000289943	0.0035510	0.0000000	0.0000000	0.0019455	0.0015531
UGP2	1.1306671	0.9051051	0.7653899	1.1368381	0.8142390
VPS54	0.2692833	0.2068496	0.1797130	0.2191509	0.4835028
ENSG00000228079	0.0000000	0.0000000	0.0015064	0.0000000	0.0062037
PELI1	0.2572029	0.2539415	0.1538703	0.2145768	0.3135861
ENSG00000225889	0.0633705	0.0725961	0.0248619	0.0493047	0.0847994
ENSG00000288932	0.0018180	0.0000000	0.0009415	0.0000000	0.0000000
LGALSL-DT	0.0111715	0.0202008	0.0320362	0.0217763	0.0627474
LGALSL	0.3896534	0.4664893	0.5238491	0.3605243	0.3123797
AFTPH-DT	0.0144491	0.0076502	0.0212472	0.0020579	0.0054053
AFTPH	0.3497404	0.4097810	0.4042352	0.3713053	0.4200902
SERTAD2	0.1917041	0.1357578	0.1307593	0.1268702	0.1653778
ENSG00000226756	0.0050109	0.0034362	0.0000000	0.0000000	0.0000000
ENSG00000288986	0.0008777	0.0000000	0.0000000	0.0000000	0.0000000
ENSG00000287123	0.0000000	0.0000000	0.0000000	0.0000000	0.0000000
LINC01800	0.0056559	0.0118664	0.0014810	0.0032774	0.0126577
LINC02245	0.1026951	0.0868651	0.0769705	0.1109227	0.1513385
SLC1A4	0.2903873	0.2507964	0.2900379	0.3318520	0.2915306
LINC02576	0.0012473	0.0022218	0.0000000	0.0000000	0.0131834
CEP68	0.2000980	0.1790391	0.1655597	0.1670794	0.2049325
RAB1A	0.9748419	1.0919573	1.2409071	0.9896351	0.9648184
ACTR2	1.1543357	1.1366612	1.1416531	1.1662593	0.9507915
SPRED2	0.1217179	0.1290509	0.1194230	0.1229201	0.2947641
ENSG00000232693	0.0000000	0.0000000	0.0000000	0.0000000	0.0000000
LINC02934	0.0512714	0.0765208	0.0552196	0.0637676	0.1673765
ENSG00000234255	0.0034980	0.0000000	0.0011774	0.0000000	0.0018081
LINC03050	0.0006689	0.0000000	0.0000000	0.0027934	0.0000000
ENSG00000281920	0.0102364	0.0087820	0.0071345	0.0067408	0.0181847
LINC01873	0.0016582	0.0022172	0.0015505	0.0000000	0.0091221
MEIS1-AS3	0.0222270	0.0292366	0.0496798	0.0128463	0.0451740
MEIS1	0.5589607	0.7684820	0.8554391	0.5140795	1.3617236
MEIS1-AS2	0.0069045	0.0036508	0.0018129	0.0098573	0.0088298
LINC01798	0.0447297	0.1144014	0.0891744	0.0765277	0.2445406
LINC01797	0.0159745	0.0362809	0.0735189	0.0122583	0.0232034
LINC01799	0.0042598	0.0113070	0.0082087	0.0193997	0.0229221
LINC01828	0.0148086	0.0489214	0.0232227	0.0028243	0.0974361
LINC01829	0.0304191	0.0925452	0.0653495	0.0227767	0.1531475
ENSG00000289533	0.0000000	0.0000000	0.0000000	0.0000000	0.0000000
ETAA1	0.2422271	0.1492304	0.1146885	0.2509660	0.1232030

ENSG00000235495	0.0023999	0.0000000	0.0000000	0.0000000	0.0072910
C1D	0.7039181	0.7580717	0.7652171	0.7403993	0.5398200
DNAAF10	0.2277978	0.1957825	0.1171990	0.1953332	0.1900754
PNO1	0.2323671	0.1943538	0.2082614	0.3023936	0.2318299
PPP3R1	0.5904856	0.8203766	0.9602927	0.5709524	0.6759163
ENSG00000273275	0.0030612	0.0038002	0.0050122	0.0032517	0.0038839
ENSG00000289156	0.0029968	0.0106868	0.0136081	0.0000000	0.0281033
ENSG00000273064	0.0012386	0.0080486	0.0009255	0.0042547	0.0199700
CNRIP1	0.3197174	0.5053853	0.6619459	0.3330600	0.3299644
FBXO48	0.0491810	0.0487206	0.0569906	0.0385279	0.0416813
APLF	0.0242791	0.0252256	0.0154157	0.0554467	0.0410620
ARHGAP25	0.0034811	0.0006384	0.0090103	0.0025032	0.0022244
ANTXR1	0.6150779	0.4258790	0.3117505	0.6952186	0.5423056
GFPT1	0.6767551	0.6792396	0.8199732	0.5632657	0.7332174
NFU1	0.6656375	0.6658626	0.6582796	0.6425113	0.6112043
AAK1	0.4236416	0.5125718	0.5729630	0.3649435	0.8063071
ANXA4	0.2407135	0.1750521	0.0892727	0.2210113	0.1309264
ENSG00000289250	0.0002987	0.0010756	0.0000000	0.0000000	0.0000000
SMANTIS	0.0452090	0.0168654	0.0182449	0.0471789	0.0150086
GMCL1	0.1825060	0.1458890	0.1616094	0.2009652	0.2280079
SNRNP27	0.6759030	0.7365939	0.7908798	0.6471818	0.5530749
MXD1	0.1742269	0.2426148	0.2609034	0.1829511	0.2576482
PCBP1-AS1	0.1494344	0.1816666	0.1826036	0.1403952	0.3996722
PCBP1	1.2358530	1.2507296	1.3170473	1.1500607	0.9739468
ENSG00000233060	0.0000000	0.0015840	0.0021412	0.0000000	0.0159701
LINC01816	0.0153144	0.0191916	0.0234293	0.0147894	0.0111201
C2orf42	0.0652388	0.0375266	0.0449167	0.0775851	0.1044811
TIA1	0.3836365	0.3691508	0.3601091	0.3725376	0.3797771
PCYOX1	0.3826324	0.3028170	0.3908853	0.6253094	0.3133922
SNRPG	0.9191484	0.8597831	0.8190407	0.9366711	0.6413088
FAM136A	0.5702074	0.5851065	0.6283742	0.5716520	0.4396465
TGFA	0.0041113	0.0056851	0.0044474	0.0000000	0.0098893
ADD2	0.2391795	0.5221155	0.5300179	0.1575973	0.6195414
ENSG00000286979	0.0044390	0.0044147	0.0000000	0.0022098	0.0180931
ENSG00000235035	0.0000000	0.0014699	0.0000000	0.0000000	0.0000000
CLEC4F	0.0000000	0.0014455	0.0000000	0.0021296	0.0000000
VAX2	0.0164347	0.0158421	0.0095113	0.0245482	0.0088146
ATP6V1B1	0.0000000	0.0000000	0.0000000	0.0017963	0.0030122
ATP6V1B1-AS1	0.0007777	0.0009108	0.0016880	0.0000000	0.0000000
TEX261	0.1347024	0.0958364	0.1042340	0.1992115	0.1158740
ANKRD53	0.0053000	0.0012444	0.0036991	0.0159734	0.0038629
ENSG00000228384	0.0010743	0.0013552	0.0000000	0.0000000	0.0044787
ENSG00000236469	0.0000000	0.0000000	0.0000000	0.0000000	0.0065199
NAGK	0.3544313	0.3725941	0.4378558	0.3446121	0.2496291
ENSG00000272735	0.0006308	0.0029813	0.0000000	0.0012382	0.0114454
MCEE	0.1211608	0.0915880	0.1147741	0.0921530	0.1168876
ENSG00000288065	0.0000000	0.0000000	0.0000000	0.0000000	0.0000000
MPHOSPH10	0.3881310	0.3927378	0.4404348	0.4425800	0.3395151
PAIP2B	0.1425651	0.1688874	0.1943649	0.0937216	0.1087897
ZNF638	0.7378135	0.8064904	0.7750867	0.6598784	1.2365379
ENSG00000281195	0.0067243	0.0027094	0.0013511	0.0000000	0.0124703
ENSG00000289463	0.0144152	0.0170286	0.0134721	0.0049681	0.0375025
DYSF	0.0030994	0.0026174	0.0022387	0.0000000	0.0111038

CYP26B1	0.0023426	0.0035829	0.0012267	0.0030059	0.0041094
EXOC6B	0.4218771	0.4655643	0.4493659	0.3897423	1.1082738
SPR	0.0223454	0.0244227	0.0270288	0.0286876	0.0132018
SFXN5	0.1433541	0.1207873	0.0845153	0.2039266	0.1928240
RAB11FIP5	0.0414186	0.0458890	0.0290006	0.0485377	0.0636014
ENSG00000272702	0.0354069	0.0376561	0.0378435	0.0145168	0.0444942
SMYD5	0.0598098	0.0673920	0.0690694	0.0780035	0.0985488
PRADC1	0.1178500	0.1244822	0.0977862	0.1704846	0.0671041
CCT7	0.8242527	0.8265881	0.9004960	0.8193738	0.6326221
FBXO41	0.0975395	0.1831220	0.2709713	0.0746880	0.2349239
EGR4	0.0000000	0.0013303	0.0000000	0.0000000	0.0041652
ENSG00000285068	0.0000000	0.0000000	0.0000000	0.0000000	0.0126609
ALMS1	0.2433960	0.2328764	0.2379362	0.2553777	0.5286816
ALMS1-IT1	0.0027893	0.0022024	0.0039839	0.0152042	0.0056888
ENSG00000284902	0.0012829	0.0008920	0.0000000	0.0000000	0.0208588
ALMS1P1	0.0076671	0.0160607	0.0078860	0.0051260	0.0129936
TPRKB	0.3135693	0.3250080	0.3558881	0.2727508	0.2153833
DUSP11	0.1962676	0.1655245	0.1707006	0.1790003	0.1476036
ENSG00000287250	0.0045914	0.0012821	0.0046821	0.0000000	0.0073347
STAMBP	0.4888497	0.4929103	0.4325877	0.5219887	0.3404274
ENSG00000286244	0.0000000	0.0012462	0.0023178	0.0000000	0.0000000
ACTG2	0.0056073	0.0036686	0.0160471	0.0000000	0.0184834
DGUOK	0.7071135	0.7825867	0.9318668	0.6713300	0.6293552
DGUOK-AS1	0.0000000	0.0028671	0.0025371	0.0000000	0.0000000
TET3	0.1850278	0.2386902	0.2248458	0.1415806	0.4502976
ENSG00000217702	0.0247575	0.0382066	0.0242946	0.0129100	0.0235451
BOLA3	0.6739722	0.7265995	0.7552189	0.6645712	0.5894130
BOLA3-DT	0.3528027	0.4488755	0.5121002	0.3217414	0.2697493
MOB1A	0.6773231	0.4533090	0.4029516	0.6051987	0.4199668
MTHFD2	0.9246079	1.1135630	1.1571421	0.8703145	0.8854352
ENSG00000279070	0.0022970	0.0000000	0.0000000	0.0000000	0.0022313
SLC4A5	0.0242298	0.0260130	0.0237818	0.0560297	0.1213084
DCTN1	0.4534385	0.5759651	0.6717482	0.3929544	0.6141360
DCTN1-AS1	0.0255819	0.0377332	0.0412040	0.0140180	0.0350408
C2orf81	0.0895962	0.0495436	0.0308747	0.0559238	0.0527619
ENSG00000286623	0.0029054	0.0111885	0.0073225	0.0067625	0.0149006
WDR54	0.3069097	0.2949144	0.3769192	0.3424221	0.3139466
RTKN	0.0426910	0.0214844	0.0355578	0.0413645	0.0169071
INO80B	0.1000406	0.1150890	0.0850258	0.0766996	0.1494680
WBP1	0.6503334	0.5260084	0.4977368	0.6584398	0.4575534
MOGS	0.0985850	0.0569289	0.0978559	0.1854083	0.1029009
ENSG00000286883	0.0072897	0.0157057	0.0101980	0.0025747	0.0224412
MRPL53	0.0269127	0.0240265	0.0106416	0.0132068	0.0346017
CCDC142	0.0205645	0.0132701	0.0061989	0.0169662	0.0287448
TTC31	0.0752664	0.0354285	0.0233157	0.0600503	0.0326826
LBX2	0.0023185	0.0021920	0.0000000	0.0000000	0.0057078
LBX2-AS1	0.0085309	0.0043288	0.0030618	0.0000000	0.0094551
PCGF1	0.1079567	0.1231282	0.0779543	0.0980534	0.1134518
DQX1	0.0015736	0.0000000	0.0000000	0.0000000	0.0058001
AUP1	0.2867359	0.2562476	0.2451290	0.3281776	0.2028766
HTRA2	0.1900832	0.2401146	0.2352112	0.2654977	0.2001788
LOXL3	0.1248911	0.0785711	0.0460716	0.1846667	0.0647672
DOK1	0.0767709	0.0572309	0.0484843	0.0691384	0.0524539

M1AP	0.0010957	0.0039437	0.0008921	0.0033377	0.0000000
SEMA4F	0.0739063	0.0915491	0.0691761	0.0473152	0.1038536
ENSG00000286739	0.0057086	0.0036661	0.0021602	0.0014762	0.0132739
ENSG00000287687	0.0045129	0.0042056	0.0031256	0.0028028	0.0000000
HK2-DT	0.0064313	0.0112013	0.0000000	0.0176185	0.0075973
HK2	0.2817762	0.3063317	0.2997487	0.2202558	0.2532164
POLE4	0.5977092	0.6072992	0.6030779	0.6281892	0.4593058
TACR1	0.2040313	0.1169008	0.0696393	0.3903743	0.1154862
ENSG00000270571	0.0083404	0.0081302	0.0098982	0.0065505	0.0000000
EVA1A	0.0090318	0.0048947	0.0035942	0.0035013	0.0044485
EVA1A-AS	0.0000000	0.0014737	0.0023309	0.0000000	0.0000000
MRPL19	0.6703509	0.5749262	0.5678817	0.6310507	0.5266767
GCFC2	0.2391482	0.1477561	0.1001609	0.2132549	0.1866583
ENSG00000271452	0.0037368	0.0039212	0.0021149	0.0029052	0.0000000
ENSG00000270462	0.0000000	0.0000000	0.0028426	0.0000000	0.0000000
ENSG00000286045	0.0413996	0.0296031	0.0189554	0.0062044	0.0190653
ENSG00000270996	0.0053055	0.0068419	0.0023088	0.0019003	0.0077248
ENSG00000287474	0.0000000	0.0000000	0.0000000	0.0041661	0.0054431
ENSG00000287172	0.0006692	0.0000000	0.0000000	0.0000000	0.0065168
ENSG00000287749	0.0000000	0.0000000	0.0000000	0.0000000	0.0000000
ENSG00000287026	0.0008466	0.0000000	0.0000000	0.0000000	0.0012770
LRRTM4	0.3612873	0.5062270	0.5108697	0.3354847	1.5233864
ENSG00000287025	0.0000000	0.0000000	0.0000000	0.0000000	0.0042562
LRRTM4-AS1	0.0005911	0.0005918	0.0000000	0.0000000	0.0134025
ENSG00000286932	0.0000000	0.0000000	0.0000000	0.0000000	0.0000000
ENSG00000227088	0.0016318	0.0046710	0.0025686	0.0012694	0.0169110
CTNNA2	0.5581423	0.8077673	0.8259949	0.5907080	1.9248563
CTNNA2-AS1	0.0000000	0.0003109	0.0013234	0.0000000	0.0182513
ENSG00000224731	0.0010333	0.0058812	0.0000000	0.0000000	0.0275639
LRRTM1	0.0532816	0.1120601	0.1730647	0.0368781	0.0718625
ENSG00000237031	0.0034901	0.0022286	0.0008833	0.0052464	0.0219617
SUCLG1	0.6813652	0.7261357	0.8209677	0.7105971	0.5899678
ENSG00000289076	0.0027068	0.0000000	0.0000000	0.0055470	0.0014245
DNAH6	0.1375817	0.0852919	0.0714887	0.0869717	0.1944790
TRABD2A	0.0339585	0.0253388	0.0184337	0.0620586	0.0263112
TMSB10	3.8319564	4.0294341	4.0993080	3.7423157	3.6335611
ENSG00000287625	0.0067610	0.0219454	0.0036409	0.0023202	0.0282888
ENSG00000290085	0.0111411	0.0141632	0.0148120	0.0049851	0.0214570
KCMF1	0.5769489	0.6356459	0.6640525	0.5307240	0.5653444
TCF7L1	0.1310652	0.0794129	0.0472906	0.1384960	0.0577969
TGOLN2	0.5488028	0.4963863	0.5236123	0.7734591	0.5113786
ENSG00000290110	0.0009875	0.0016094	0.0025093	0.0064552	0.0052561
RETSAT	0.0587981	0.0408495	0.0247668	0.1426382	0.0423160
ELMOD3	0.0930795	0.1071606	0.0667904	0.0683420	0.1055383
CAPG	0.1587382	0.0587147	0.0260744	0.2181962	0.0617993
ENSG00000286011	0.0215352	0.0126648	0.0103024	0.0211417	0.0035405
SH2D6	0.0161658	0.0086722	0.0111698	0.0297870	0.0000000
PARTICL	0.0487913	0.0373827	0.0288710	0.0581544	0.0185388
MAT2A	0.3810673	0.2436111	0.2004495	0.5095436	0.4044008
GGCX	0.2092149	0.1612830	0.1509425	0.3200019	0.2049066
VAMP8	0.0413832	0.0161906	0.0100482	0.0232345	0.0432604
VAMP5	0.7290681	0.4862873	0.3144834	0.7479151	0.4241274
ENSG00000288858	0.0032649	0.0007167	0.0016009	0.0055551	0.0000000

RNF181	0.7008043	0.6693798	0.7298632	0.7027977	0.5408900
TMEM150A	0.2913691	0.1619596	0.1163940	0.2783640	0.1043326
USP39	0.3124230	0.2687509	0.3209862	0.2759618	0.2047306
C2orf68	0.2203886	0.1693219	0.1497465	0.1869474	0.1656884
GNLY	0.0000000	0.0015691	0.0019659	0.0000000	0.0068671
ATOH8	0.0418678	0.0238722	0.0130857	0.0428400	0.0430797
ENSG00000229498	0.0000000	0.0011085	0.0000000	0.0000000	0.0000000
ST3GAL5	0.4373450	0.5434279	0.6068167	0.5479007	0.4221756
ENSG00000272564	0.0022544	0.0037830	0.0061644	0.0032941	0.0000000
POLR1A	0.1188630	0.1011507	0.0862750	0.1000073	0.1586681
PTCD3	0.4118199	0.3559017	0.2321001	0.5183635	0.3190726
IMMT	0.6822463	0.7116531	0.7464219	0.6691899	0.6091226
ENSG00000273080	0.0738464	0.0741838	0.0588299	0.0680680	0.0469765
MRPL35	0.3130214	0.3028691	0.2926480	0.3425120	0.2898319
REEP1	0.5702573	0.9778410	1.3665178	0.5219558	1.0105296
KDM3A	0.4729880	0.4294837	0.3994725	0.4347530	0.5212475
CHMP3	0.6801081	0.6919863	0.6186044	0.6431551	0.4960680
CHMP3-AS1	0.0065800	0.0037327	0.0090213	0.0061941	0.0280339
RNF103	0.1059895	0.0889250	0.0966522	0.1517831	0.1099174
RMND5A	0.3281945	0.3548351	0.2950643	0.2553945	0.3179665
CD8A	0.0406842	0.0849791	0.0962172	0.0530475	0.0929459
CD8B	0.0055303	0.0059997	0.0099522	0.0056883	0.0000000
ENSG00000291013	0.0020079	0.0019046	0.0067575	0.0019468	0.0122406
RGPD1	0.0012933	0.0015682	0.0034549	0.0000000	0.0000000
PLGLB1	0.0037020	0.0052151	0.0024782	0.0000000	0.0150997
ENSG00000287931	0.0028758	0.0025673	0.0072314	0.0000000	0.0105662
ENSG00000287763	0.0240942	0.0401714	0.0737936	0.0234464	0.0456531
LINC01943	0.0000000	0.0000000	0.0000000	0.0000000	0.0000000
CYTOR	0.0389684	0.0272071	0.0258350	0.0127111	0.0399031
ENSG00000287670	0.0092943	0.0211612	0.0362110	0.0000000	0.0108795
KRCC1	0.4179212	0.3202722	0.2239777	0.3981407	0.3008892
FABP1	0.0016244	0.0000000	0.0000000	0.0000000	0.0000000
THNSL2	0.1050768	0.0609641	0.0401325	0.1146059	0.0359379
ENSG00000225420	0.0010393	0.0013478	0.0020798	0.0000000	0.0052447
EIF2AK3	0.0860787	0.1085502	0.0991934	0.1058873	0.2693480
EIF2AK3-DT	0.0166033	0.0064037	0.0128422	0.0169690	0.0099522
RPIA	0.1741268	0.1774859	0.1771723	0.1538580	0.1610995
ANKRD36BP2	0.0893607	0.1789409	0.1545670	0.0821312	0.2597598
ENSG00000290846	0.0125996	0.0112480	0.0034022	0.0015620	0.0074140
ENSG00000281904	0.0011275	0.0000000	0.0059988	0.0000000	0.0000000
ENSG00000261600	0.0006704	0.0012480	0.0018021	0.0000000	0.0000000
LSP1P5.1	0.0310674	0.0245328	0.0235090	0.0352249	0.0572604
ENSG00000291142	0.1189833	0.1545712	0.2107623	0.0894791	0.1378792
ENSG00000290941	0.0000000	0.0000000	0.0000000	0.0000000	0.0000000
AQP7B	0.0011144	0.0012208	0.0016797	0.0000000	0.0256677
ENSG00000291025	0.0018448	0.0000000	0.0000000	0.0020616	0.0000000
TEKT4	0.0171519	0.0103968	0.0011718	0.0349887	0.0074820
MAL	0.0260952	0.0302239	0.0079260	0.0524664	0.0059248
ENSG00000287362	0.0020463	0.0007805	0.0032625	0.0000000	0.0052569
MRPS5	0.6167086	0.6505168	0.6492333	0.6144158	0.6415452
ZNF514	0.1254025	0.1480597	0.1516746	0.0936263	0.1657796
ZNF2	0.0370024	0.0266515	0.0111345	0.0171543	0.0288855
ENSG00000289370	0.0176023	0.0376566	0.0617376	0.0154209	0.0211802

ZNF892	0.1008534	0.1302659	0.1218425	0.1101748	0.3069528
PROM2	0.0027054	0.0072019	0.0058893	0.0018419	0.0234140
KCNIP3	0.0303078	0.0352141	0.0335158	0.0312204	0.0366273
FAHD2A	0.0599404	0.0884685	0.0719396	0.0593667	0.1019524
LINC00342	0.2027845	0.2350866	0.1878358	0.1405929	0.8044306
ANKRD36C	0.4962936	0.5889985	0.4928163	0.4480987	1.3236516
ENSG00000291176	0.1087321	0.0922541	0.0955030	0.1413688	0.0618161
GPAT2	0.0000000	0.0000000	0.0017189	0.0046056	0.0000000
ASTL	0.0000000	0.0000000	0.0011329	0.0000000	0.0000000
DUSP2	0.0323814	0.0861122	0.0956658	0.0219952	0.0414830
STARD7	0.4433037	0.4407066	0.4921308	0.4327360	0.4338387
STARD7-AS1	0.0336724	0.0429527	0.0119201	0.0146064	0.0732967
TMEM127	0.1741214	0.1925039	0.2467970	0.1719716	0.1495159
CIAO1	0.4175242	0.3867621	0.3743900	0.4511178	0.3768787
SNRNP200	0.4914405	0.4471445	0.3962593	0.4996736	0.5069193
NCAPH	0.0032877	0.0087635	0.0000000	0.0069387	0.0000000
NEURL3	0.0009776	0.0020392	0.0019382	0.0089851	0.0000000
ARID5A	0.0135095	0.0175574	0.0150928	0.0092918	0.0218150
KANSL3	0.1419304	0.1000466	0.1255820	0.0562812	0.2502305
FER1L5	0.0000000	0.0004858	0.0000000	0.0071088	0.0076808
LMAN2L	0.1403067	0.1015260	0.0683945	0.2253321	0.1433483
CNNM4	0.0426007	0.0458219	0.0546426	0.0334590	0.1313812
CNNM3-DT	0.0006652	0.0000000	0.0000000	0.0000000	0.0000000
ENSG00000289135	0.0365217	0.0261400	0.0179384	0.0413434	0.0233882
CNNM3	0.0378943	0.0348005	0.0195948	0.0533333	0.0617311
ANKRD23	0.0259555	0.0284339	0.0351559	0.0167970	0.0927814
ANKRD39	0.1789396	0.2241174	0.2910493	0.1759998	0.1897512
SEMA4C	0.0870261	0.0849556	0.1004989	0.1006172	0.1090051
FAM178B	0.0062364	0.0039819	0.0045327	0.0000000	0.0196036
FAHD2B	0.1153043	0.1413746	0.1383962	0.1112701	0.0718659
ANKRD36	0.4347647	0.4446909	0.3743818	0.2924183	1.1521989
ENSG00000277701	0.0123500	0.0210827	0.0009802	0.0173069	0.0767592
APPAT	0.0943791	0.0964150	0.1114473	0.0487764	0.2705122
ENSG00000278766	0.0009386	0.0014206	0.0000000	0.0000000	0.0000000
ANKRD36B	0.1787351	0.2350972	0.1538843	0.1321206	0.6167965
COX5B	1.4287723	1.5652219	1.6209637	1.4074224	1.2526001
ACTR1B	0.2671343	0.2622547	0.1878260	0.2723081	0.2079735
C2orf92	0.0310137	0.0158311	0.0159636	0.0099422	0.0359330
ZAP70	0.0009517	0.0000000	0.0000000	0.0000000	0.0000000
TMEM131	0.3853632	0.3298932	0.3524680	0.4763064	0.5973696
VWA3B	0.1489483	0.0770291	0.0581839	0.1298821	0.0747738
ENSG00000222000	0.0059668	0.0009875	0.0050372	0.0037663	0.0034682
CNGA3	0.0025896	0.0065330	0.0125259	0.0090226	0.0410770
INPP4A	0.2246259	0.3409516	0.3810867	0.2024491	0.5437473
COA5	0.5549566	0.5189641	0.4958956	0.5960150	0.3345547
UNC50	0.4346499	0.3115013	0.2927376	0.5536309	0.2634938
MGAT4A	0.2184728	0.3301958	0.4319398	0.2209000	0.4203285
LINC02611	0.0000000	0.0000000	0.0025847	0.0000000	0.0050220
CRACDL	0.1143985	0.0547401	0.0370753	0.1337501	0.0692683
TSGA10	0.3141118	0.3076454	0.2510000	0.2246726	0.3021657
C2orf15	0.0871701	0.1220202	0.1897238	0.0965119	0.1032099
LIPT1	0.1168263	0.0886369	0.0459485	0.1811618	0.0555207
MITD1	0.2060842	0.1778145	0.1221907	0.2301668	0.1439380

MRPL30	0.4571251	0.5269082	0.5642052	0.3814721	0.4441515
LYG2	0.0020560	0.0056354	0.0023575	0.0052300	0.0000000
LYG1	0.0059694	0.0047879	0.0118036	0.0074239	0.0056155
TXNDC9	0.2671164	0.2835752	0.3082493	0.2869958	0.1926553
EIF5B	1.2607561	1.0724175	1.0463327	1.0888374	0.9087692
REV1	0.2819628	0.3085153	0.3371517	0.2727995	0.3987946
ENSG00000273306	0.0000000	0.0000000	0.0010329	0.0000000	0.0024211
AFF3	0.5981555	0.6708874	0.6348234	0.5981307	1.4465301
ENSG00000230393	0.0167926	0.0115721	0.0077665	0.0160077	0.0000000
LONRF2	0.3833097	0.5863175	0.7949859	0.3742957	0.6605588
CHST10	0.0587764	0.0898314	0.1119759	0.0869152	0.0697952
NMS	0.0041104	0.0106207	0.0014831	0.0201818	0.0409772
PDCL3	0.3104661	0.3414008	0.3615400	0.3082691	0.2867397
LINC01849	0.0000000	0.0022102	0.0000000	0.0000000	0.0000000
ENSG00000289077	0.0065684	0.0065714	0.0041267	0.0072725	0.0061493
NPAS2	0.1097926	0.1101770	0.0958321	0.1146331	0.2623351
ENSG00000232034	0.0015440	0.0000000	0.0000000	0.0000000	0.0000000
NPAS2-AS1	0.0000000	0.0011666	0.0000000	0.0000000	0.0039740
ENSG00000223947	0.0000000	0.0010689	0.0000000	0.0000000	0.0000000
RPL31	2.7717209	2.7527651	2.5537708	2.6912860	2.5379691
TBC1D8	0.0564817	0.0854576	0.0890406	0.0685698	0.1343739
TBC1D8-AS1	0.0094220	0.0115918	0.0039098	0.0151665	0.0141743
CNOT11	0.2498287	0.2463355	0.2783128	0.1919834	0.1597668
RNF149	0.2788834	0.1942794	0.2210926	0.4220510	0.3384941
CREG2	0.2350111	0.5587497	0.7460810	0.2105189	0.4258223
RFX8	0.0049777	0.0098851	0.0067129	0.0000000	0.0322437
MAP4K4	0.8971830	1.0108777	0.9176632	0.8012463	1.1567894
ENSG00000288948	0.0008314	0.0062384	0.0000000	0.0000000	0.0075378
LINC01127	0.0000000	0.0000000	0.0000000	0.0000000	0.0000000
IL1R1	0.0186563	0.0023551	0.0014035	0.0090264	0.0065833
IL1RL2	0.0003542	0.0000000	0.0000000	0.0027125	0.0000000
IL18R1	0.0008992	0.0020001	0.0066934	0.0123844	0.0000000
SLC9A2	0.0247902	0.0187943	0.0132490	0.0110738	0.0182472
MFSD9	0.0489346	0.0513547	0.0621751	0.0320607	0.0749272
TMEM182	0.0591883	0.0676862	0.0790688	0.0534750	0.1010165
LINC01796	0.0000000	0.0000000	0.0000000	0.0000000	0.0000000
ENSG00000286290	0.0000000	0.0017053	0.0000000	0.0000000	0.0015171
ENSG00000227157	0.0038293	0.0014991	0.0000000	0.0136794	0.0000000
LINC01102	0.0235928	0.0806311	0.0404490	0.0237521	0.0404356
ENSG00000288565	0.0099374	0.0154695	0.0202701	0.0188406	0.0122163
ENSG00000228528	0.0113805	0.0018595	0.0014901	0.0283550	0.0000000
LINC01114	0.0146530	0.0168138	0.0215663	0.0288266	0.0218039
PANTR1	0.1630696	0.1925374	0.1473292	0.2369534	0.1871800
ENSG00000269707	0.0325812	0.0378595	0.0313625	0.0223483	0.0232940
POU3F3	0.1993416	0.2246693	0.2033948	0.2745745	0.1884010
ENSG00000289498	0.0042859	0.0128780	0.0019886	0.0046468	0.0030127
LINC01159	0.0072109	0.0054386	0.0047047	0.0143265	0.0000000
ENSG00000287135	0.0022250	0.0044180	0.0053069	0.0011920	0.0000000
MRPS9-AS2	0.0081205	0.0060869	0.0034150	0.0074399	0.0169862
MRPS9	0.3726446	0.3455316	0.3925656	0.3359865	0.3917509
MRPS9-AS1	0.0000000	0.0011092	0.0000000	0.0000000	0.0000000
LINC01918	0.0193815	0.0105341	0.0224399	0.0105800	0.0092410
GPR45	0.0006142	0.0033055	0.0027290	0.0000000	0.0087325

ENSG00000272861	0.0028882	0.0000000	0.0013096	0.0021849	0.0000000
TGFBRAP1	0.0968438	0.1054003	0.1135719	0.1168642	0.1109996
ENSG00000235319	0.0061850	0.0061778	0.0020549	0.0018419	0.0054919
C2orf49-DT	0.0518336	0.0535413	0.0525708	0.0460419	0.0370639
C2orf49	0.4368460	0.3958713	0.4452592	0.5202462	0.2878443
FHL2	0.0221366	0.0202505	0.0091223	0.0217916	0.0099789
ENSG00000238273	0.0000000	0.0000000	0.0000000	0.0000000	0.0066309
NCK2	0.3486205	0.3571084	0.3158478	0.3670043	0.3779202
ENSG00000290119	0.0000000	0.0000000	0.0000000	0.0000000	0.0055238
ENSG00000235522	0.0014048	0.0033927	0.0000000	0.0000000	0.0014498
ENSG00000234162	0.0004739	0.0000000	0.0011774	0.0000000	0.0084416
ECRG4	0.6364090	0.4128708	0.3077428	0.8004360	0.4397615
UXS1	0.3980213	0.3482678	0.2539000	0.5218884	0.3913288
ENSG00000233339	0.0000000	0.0000000	0.0009612	0.0000000	0.0000000
ENSG00000291125	0.0000000	0.0000000	0.0000000	0.0000000	0.0000000
RGPD3	0.0000000	0.0000000	0.0015080	0.0000000	0.0031033
ST6GAL2	0.0979702	0.2368383	0.2378680	0.0761801	0.2353668
ST6GAL2-IT1	0.0000000	0.0009018	0.0000000	0.0000000	0.0000000
ENSG00000227294	0.0000000	0.0000000	0.0000000	0.0000000	0.0069935
ENSG00000286218	0.0000000	0.0000000	0.0000000	0.0000000	0.0000000
LINC01885	0.0018784	0.0000000	0.0000000	0.0000000	0.0000000
RGPD4	0.0018258	0.0026454	0.0057631	0.0035133	0.0163856
SLC5A7	0.1883882	0.3608147	0.1633290	0.1426584	0.5204489
LINC01594	0.0037441	0.0000000	0.0000000	0.0000000	0.0000000
SULT1C2	0.0160033	0.0126154	0.0029653	0.0075637	0.0200900
SULT1C5P	0.0036654	0.0000000	0.0000000	0.0000000	0.0000000
SULT1C4	0.3860642	0.1911212	0.1324924	0.3849438	0.2034168
GCC2	0.9419755	0.7808172	0.6483582	0.7364006	0.7808515
GCC2-AS1	0.0123056	0.0078400	0.0069359	0.0053164	0.0160547
LIMS1	0.5012764	0.3798178	0.2026923	0.4784524	0.4772977
RANBP2	0.4817653	0.4803227	0.4642110	0.4403787	0.6253121
CCDC138	0.2292815	0.2276980	0.1943931	0.2004948	0.3735416
EDAR	0.0004507	0.0007858	0.0000000	0.0000000	0.0034299
SH3RF3-AS1	0.0068145	0.0057928	0.0031078	0.0026458	0.0000000
SH3RF3	0.0984304	0.0771998	0.0748118	0.0710435	0.3035707
SEPTIN10	0.6903420	0.3898325	0.2516606	0.6709291	0.3225105
SOWAHC	0.2221005	0.1383636	0.1239109	0.2686047	0.1021747
ENSG00000282033	0.0038161	0.0019216	0.0112588	0.0023064	0.0116051
RGPD5	0.0046253	0.0083549	0.0012337	0.0040415	0.0148336
LINC01123	0.0141756	0.0161330	0.0064377	0.0181807	0.0153827
MALL	0.0031955	0.0057303	0.0027227	0.0215737	0.0000000
NPHP1	0.2488391	0.1493123	0.1402752	0.2433677	0.1593249
MTLN	0.4431581	0.4962949	0.5781662	0.4043512	0.3583504
LINC01106	0.0355782	0.0300794	0.0416763	0.0372029	0.0168313
ENSG00000290733	0.0000000	0.0000000	0.0000000	0.0000000	0.0000000
RGPD6	0.0112422	0.0155703	0.0289569	0.0000000	0.0225967
BUB1	0.0083506	0.0159366	0.0053219	0.0109084	0.0059869
ENSG00000289202	0.0000000	0.0000000	0.0000000	0.0000000	0.0000000
ACOXL	0.0051149	0.0031700	0.0000000	0.0049051	0.0040799
MIR4435-2HG	0.0781762	0.0510463	0.0315140	0.0614583	0.1285826
ACOXL-AS1	0.0014548	0.0073494	0.0000000	0.0000000	0.0059649
BCL2L11	0.0533736	0.0512931	0.0373881	0.0515347	0.0494575
ENSG00000271590	0.0000000	0.0000000	0.0000000	0.0000000	0.0060718

ENSG00000270190	0.0000000	0.0000000	0.0000000	0.0000000	0.0000000
ENSG00000285016	0.0000000	0.0027159	0.0050184	0.0000000	0.0026470
ENSG00000280878	0.0000000	0.0000000	0.0000000	0.0000000	0.0000000
ANAPC1	0.1094809	0.1121230	0.1120117	0.1333697	0.1600989
MERTK	0.0173552	0.0076831	0.0039425	0.0133432	0.0180275
ENSG00000286904	0.0025921	0.0010863	0.0000000	0.0000000	0.0032430
TMEM87B	0.0869785	0.0970664	0.0821824	0.0556079	0.1488687
FBLN7	0.0773634	0.0699971	0.1856532	0.0891764	0.1339049
ZC3H8	0.2891624	0.3592707	0.4105189	0.3219726	0.4232828
ENSG00000286545	0.0004918	0.0000000	0.0000000	0.0000000	0.0125955
ZC3H6	0.5516664	0.5559551	0.4843533	0.5052621	0.4626168
RGPD8	0.0116786	0.0175661	0.0108562	0.0171228	0.0393615
TTL	0.3571212	0.5831539	0.4794959	0.3001298	0.4627447
POLR1B	0.1075814	0.0940511	0.1233099	0.1110912	0.1382548
CHCHD5	0.4926900	0.3703013	0.3304256	0.5225796	0.3127859
ENSG00000243389	0.0165325	0.0056578	0.0115729	0.0315505	0.0499409
ENSG00000228251	0.0000000	0.0011001	0.0000000	0.0000000	0.0000000
SLC20A1-DT	0.0178323	0.0268138	0.0116414	0.0294834	0.0127892
SLC20A1	0.1829154	0.2281749	0.2795340	0.2607891	0.2408712
CKAP2L	0.0062965	0.0108422	0.0123304	0.0031927	0.0152195
IL1A	0.0002980	0.0000000	0.0054263	0.0000000	0.0000000
ENSG00000287937	0.0000000	0.0000000	0.0000000	0.0000000	0.0034437
PSD4	0.0022108	0.0094825	0.0031800	0.0031511	0.0070279
PAX8-AS1	0.0355016	0.0206775	0.0198187	0.0291821	0.0144483
PAX8	0.0212056	0.0105420	0.0100458	0.0177562	0.0083514
LINC02966	0.0041588	0.0027934	0.0104422	0.0033647	0.0072031
ENSG00000272563	0.0014320	0.0062345	0.0049459	0.0076606	0.0177842
ZNG1B	0.1352516	0.1500928	0.1326830	0.1282870	0.1717611
FOXD4L1	0.0000000	0.0000000	0.0000000	0.0000000	0.0000000
ENSG00000287165	0.0004623	0.0010052	0.0000000	0.0000000	0.0000000
WASH2P	0.1033499	0.0970448	0.0843634	0.0666414	0.1115646
RABL2A	0.2065548	0.1714849	0.2044878	0.2035881	0.1642435
LINC02936	0.0038669	0.0033378	0.0013516	0.0000000	0.0143636
SLC35F5	0.2982132	0.2259778	0.1703080	0.3204769	0.4457978
ENSG00000244063	0.0022124	0.0065944	0.0000000	0.0000000	0.0000000
ACTR3-AS1	0.0306278	0.0326623	0.0306075	0.0262603	0.0357097
ENSG00000270019	0.0041581	0.0032859	0.0000000	0.0000000	0.0125573
ACTR3	0.7722633	0.7934825	0.9454212	0.8140954	0.7518803
LINC01191	0.0010600	0.0000000	0.0027908	0.0000000	0.0016664
DPP10	0.3945718	0.4658659	0.6036987	0.2894736	1.3408614
DPP10-AS3	0.0000000	0.0031801	0.0007884	0.0000000	0.0000000
ENSG00000286776	0.0000000	0.0000000	0.0000000	0.0000000	0.0000000
DPP10-AS1	0.0456605	0.0622228	0.1183273	0.0354033	0.0532823
ENSG00000287451	0.0025157	0.0000000	0.0000000	0.0000000	0.0016619
ENSG00000238207	0.0029935	0.0058818	0.0086618	0.0092275	0.0000000
DDX18	0.6604969	0.6318926	0.7020075	0.6331086	0.5310270
ENSG00000236255	0.0059712	0.0073725	0.0068751	0.0081041	0.0167092
ENSG00000290590	0.0000000	0.0000000	0.0000000	0.0000000	0.0000000
CCDC93	0.2526290	0.2249955	0.2097504	0.2292393	0.3915193
ENSG00000235066	0.0128355	0.0227705	0.0411313	0.0359927	0.0025831
INSIG2	0.3410640	0.3212991	0.2800441	0.3441899	0.2972870
THORLNC	0.0040514	0.0025932	0.0000000	0.0000000	0.0079100
EN1	0.0064018	0.0019477	0.0145554	0.0015674	0.0000000

C1QL2	0.0000000	0.0008330	0.0014971	0.0000000	0.0101520
STEAP3	0.0069302	0.0104197	0.0000000	0.0173150	0.0191276
STEAP3-AS1	0.0076887	0.0101395	0.0052354	0.0000000	0.0127523
C2orf76	0.2312955	0.2140608	0.1961087	0.2320228	0.1898892
DBI	0.6780449	0.6935989	0.6683932	0.7070771	0.5553051
TMEM37	0.0052777	0.0000000	0.0026511	0.0011582	0.0000000
SCTR	0.0027008	0.0040900	0.0071744	0.0031580	0.0035214
CFAP221	0.4370237	0.4021543	0.3944957	0.4130506	0.4392641
TMEM177	0.0489003	0.0623123	0.0551870	0.0484128	0.0465145
PTPN4	0.3037725	0.2881667	0.3072629	0.3026617	0.6014008
EPB41L5	0.3420040	0.2847928	0.2937613	0.3015380	0.4212615
ENSG00000224789	0.0068625	0.0044238	0.0048224	0.0037761	0.0457702
TMEM185B	0.0635697	0.0440855	0.0438463	0.1208973	0.0287440
RALB	0.3583282	0.4228501	0.4261335	0.3111899	0.2685274
INHBB	0.0698435	0.0312498	0.0166923	0.1620700	0.0287577
GLI2	0.0076555	0.0092777	0.0090301	0.0028622	0.1116052
TFCP2L1	0.0024514	0.0046585	0.0000000	0.0036094	0.0071095
CLASP1	0.4612440	0.5466967	0.5146227	0.3743753	0.9295419
CLASP1-AS1	0.0382679	0.0401000	0.0307022	0.0263375	0.0858605
NIFK-AS1	0.1381710	0.1675940	0.0895339	0.1328714	0.1780280
NIFK	0.6212385	0.4978589	0.4501035	0.6559915	0.3603398
TSN	0.4426781	0.3331727	0.3521130	0.4625088	0.3504765
LINC01823	0.0015523	0.0033126	0.0000000	0.0061098	0.0073473
ENSG00000286481	0.0028405	0.0063874	0.0013511	0.0053825	0.0189984
CNTNAP5-DT	0.0296500	0.0927303	0.0635623	0.0297614	0.0733267
CNTNAP5	0.2505684	0.3558148	0.3921688	0.1663376	1.1646842
GYPC	0.3752557	0.2597526	0.1836865	0.4018705	0.2279179
ENSG00000260634	0.0074804	0.0142616	0.0073621	0.0055030	0.0094316
ENSG00000286971	0.0073961	0.0083014	0.0118771	0.0039294	0.0067404
ENSG00000260163	0.0055588	0.0034866	0.0141102	0.0052689	0.0013712
BIN1	0.2953254	0.4310729	0.4350047	0.2663106	0.4962647
CYP27C1	0.0655262	0.0459023	0.0330958	0.0506216	0.0685071
ERCC3	0.1972045	0.1289200	0.1674295	0.1720462	0.1157765
ENSG00000286145	0.0019983	0.0015089	0.0119863	0.0123984	0.0774687
MAP3K2	0.5341250	0.5170950	0.5495124	0.3937116	0.6879714
MAP3K2-DT	0.0911576	0.0733076	0.0464340	0.0789842	0.0664090
ENSG00000287742	0.0004051	0.0033577	0.0011172	0.0000000	0.0000000
PROC	0.0010222	0.0085192	0.0161260	0.0000000	0.0026876
IWS1	0.4511853	0.4111928	0.4953150	0.4040852	0.5732645
ENSG00000231731	0.0033971	0.0012268	0.0000000	0.0015041	0.0094113
MYO7B	0.0000000	0.0000000	0.0000000	0.0000000	0.0000000
LIMS2	0.0194322	0.0116962	0.0082551	0.0197883	0.0020430
WDR33	0.4465463	0.4714555	0.5098153	0.4134223	0.5511660
SFT2D3	0.1206022	0.1042110	0.0719683	0.1565141	0.0857798
POLR2D	0.2452019	0.2414287	0.2532478	0.2995188	0.1902198
AMMECR1L	0.0908862	0.0853467	0.0767832	0.1318448	0.0606695
ENSG00000272667	0.0254879	0.0221656	0.0251306	0.0202400	0.0351731
ENSG00000286873	0.0117635	0.0156395	0.0019511	0.0182124	0.0154891
SAP130	0.1374249	0.1508881	0.1231008	0.1049705	0.2424718
ENSG00000289982	0.0010303	0.0014749	0.0000000	0.0000000	0.0000000
UGGT1	0.1878177	0.1750620	0.2116017	0.3181560	0.2312089
HS6ST1	0.2257613	0.3389770	0.3481662	0.1951627	0.3844478
LINC02572	0.0089742	0.0047680	0.0056463	0.0024198	0.0248141

ENSG00000286957	0.0000000	0.0053059	0.0000000	0.0000000	0.0000000
LINC01856	0.0032149	0.0024296	0.0015202	0.0020594	0.0000000
RAB6C-AS1	0.0232197	0.0546692	0.0505982	0.0175734	0.0676279
RAB6C	0.0048486	0.0059147	0.0066789	0.0032790	0.0000000
ENSG00000290591	0.0018228	0.0126360	0.0071497	0.0035673	0.0062081
POTEF	0.0000000	0.0000000	0.0000000	0.0043997	0.0000000
MED15P9	0.0000000	0.0000000	0.0000000	0.0000000	0.0000000
CCDC74B	0.0749942	0.0659929	0.0599974	0.1007444	0.0375331
SMPD4	0.1237152	0.1463561	0.1484750	0.1821537	0.2216214
MZT2B	1.4533699	1.4633354	1.5052212	1.4787626	1.2394955
CCDC115	0.4281069	0.4636630	0.5207755	0.3342619	0.3595886
IMP4	0.2457897	0.2765438	0.2972226	0.3112995	0.2846616
PTPN18	0.1594065	0.1696102	0.1586052	0.1782489	0.1195784
ENSG00000290596	0.2403428	0.2890253	0.3669232	0.2426901	0.2689931
POTEI	0.0054111	0.0010011	0.0018292	0.0000000	0.0105510
CFC1B	0.0025779	0.0057393	0.0000000	0.0088342	0.0000000
ENSG00000232408	0.0000000	0.0000000	0.0000000	0.0039591	0.0000000
CFC1	0.6820369	0.3718771	0.2721413	0.5943254	0.2840191
POTEJ	0.0067623	0.0025715	0.0026682	0.0000000	0.0000000
ENSG00000290700	0.0806041	0.1223521	0.1135787	0.0771004	0.0944087
ENSG00000229797	0.0063608	0.0113507	0.0107956	0.0045337	0.0339899
AMER3	0.0367233	0.0645084	0.0638164	0.0240019	0.0733096
ARHGEF4	0.0219579	0.0250573	0.0220835	0.0218809	0.1580909
FAM168B	0.5772864	0.6936135	0.6963093	0.5541884	0.6613056
PLEKHB2	0.4738574	0.7100998	0.8168771	0.4413710	0.5111433
POTEE	0.0000000	0.0000000	0.0000000	0.0000000	0.0037844
ENSG00000290654	0.0003059	0.0016989	0.0013013	0.0028042	0.0000000
RAB6D	0.0092594	0.0134288	0.0210034	0.0066834	0.0313888
ENSG00000283303	0.0033323	0.0063530	0.0031723	0.0011543	0.0000000
ENSG00000273073	0.0007790	0.0017554	0.0056908	0.0000000	0.0000000
MZT2A	0.8457307	0.9703356	1.0521103	0.8383845	0.6894947
TUBA3D	0.0000000	0.0011964	0.0000000	0.0000000	0.0041350
CCDC74A	0.1420642	0.1145770	0.1219344	0.1664095	0.0952142
C2orf27A	0.1013062	0.1783453	0.1868218	0.0947369	0.3074341
ENSG00000290616	0.0000000	0.0035582	0.0053309	0.0000000	0.0000000
ENSG00000235615	0.0002291	0.0000000	0.0000000	0.0000000	0.0000000
LINC01945	0.0000000	0.0035985	0.0000000	0.0000000	0.0000000
ENSG00000287414	0.0008980	0.0014335	0.0000000	0.0015061	0.0000000
ANKRD30BL	0.0966025	0.1203567	0.0836284	0.0793438	0.5976017
ENSG00000290902	0.0060343	0.0052342	0.0257222	0.0000000	0.0000000
ENSG00000272769	0.0367509	0.0560711	0.0758073	0.0201635	0.0368455
ENSG00000286833	0.0241412	0.0296967	0.0145923	0.0450483	0.0340723
GPR39	0.0162617	0.0165480	0.0215808	0.0174221	0.0281581
LYPD1	0.5471613	0.4864484	0.4668438	0.8256423	0.3690301
ENSG00000230065	0.0000000	0.0000000	0.0000000	0.0000000	0.0000000
NCKAP5	0.1750914	0.1835825	0.1180582	0.2340920	0.5419557
ENSG00000286068	0.0015884	0.0000000	0.0000000	0.0000000	0.0229245
NCKAP5-AS1	0.0000000	0.0000000	0.0000000	0.0000000	0.0000000
NCKAP5-AS2	0.0108991	0.0091705	0.0076659	0.0244839	0.0148808
MGAT5	0.3183722	0.4308910	0.5209246	0.2730100	0.8201027
ENSG00000289534	0.0000000	0.0020288	0.0015470	0.0000000	0.0050764
TMEM163	0.3871165	0.2105068	0.1687117	0.5122912	0.3341301
ENSG00000287463	0.0742460	0.0495139	0.0298171	0.0730751	0.0554556

CCNT2-AS1	0.0715699	0.0605617	0.0458658	0.0588294	0.1082776
ACMSD	0.0015621	0.0008594	0.0000000	0.0000000	0.0102001
CCNT2	0.2878758	0.2837779	0.2899740	0.2673079	0.4402876
MAP3K19	0.0854323	0.0595832	0.0361716	0.1945952	0.0659621
RAB3GAP1	0.4533008	0.4041441	0.3957036	0.3977803	0.6547464
ZRANB3	0.1302264	0.1709471	0.1272452	0.1184081	0.3332913
R3HDM1	0.4244576	0.6285936	0.5701118	0.4661590	0.8808795
UBXN4	1.2696668	1.0524923	0.9796224	1.3734502	1.0914889
LCT	0.0000000	0.0000000	0.0000000	0.0000000	0.0000000
MCM6	0.0643789	0.0522997	0.1039651	0.0662657	0.1053478
DARS1	0.8548532	0.6306447	0.5154810	0.8074964	0.6455570
DARS1-AS1	0.0114977	0.0088142	0.0062909	0.0202437	0.0068021
ENSG00000237262	0.0038062	0.0059035	0.0000000	0.0000000	0.0000000
CXCR4	0.1183019	0.2016104	0.1717112	0.1225833	0.1239083
ENSG00000289974	0.0000000	0.0000000	0.0000000	0.0000000	0.0000000
THSD7B	0.0718344	0.0836431	0.1008192	0.0457884	0.5345177
HNMT	0.1351440	0.0807055	0.0537070	0.2114827	0.0433228
SPOPL-DT	0.0139360	0.0171536	0.0079520	0.0111430	0.0438350
SPOPL	0.2170436	0.2138554	0.1783639	0.1911794	0.2524617
ENSG00000241772	0.0017071	0.0015431	0.0000000	0.0000000	0.0074820
LINC02631	0.0016544	0.0000000	0.0035910	0.0029023	0.0015201
NXPH2	0.0098426	0.0164017	0.0204006	0.0112406	0.0029373
LRP1B	0.6139138	0.6824075	0.5099201	0.5979694	1.5618562
KYNU	0.0000000	0.0016838	0.0000000	0.0027497	0.0000000
ARHGAP15	0.0006211	0.0012616	0.0000000	0.0050817	0.0067152
ENSG00000228655	0.0000000	0.0011050	0.0000000	0.0000000	0.0060461
ENSG00000257277	0.0021622	0.0025571	0.0016371	0.0000000	0.0000000
ENSG00000232377	0.0045820	0.0050073	0.0120510	0.0000000	0.0080156
GTDC1	0.2655331	0.2543657	0.2400422	0.2416951	0.4275647
ZEB2	0.6849540	0.8517994	0.4453593	0.5616223	0.8997318
ZEB2-AS1	0.0273984	0.0204888	0.0100231	0.0219758	0.0073135
LINC01412	0.0000000	0.0024499	0.0014518	0.0072198	0.0050268
LINC02993	0.0514561	0.0435404	0.0235134	0.0523748	0.0202716
TEX41	0.0042152	0.0036235	0.0012170	0.0036162	0.0094660
ACVR2A	0.2971293	0.4040395	0.4750997	0.2717598	0.6850339
ENSG00000223911	0.0013709	0.0039530	0.0050343	0.0054719	0.0000000
ORC4	0.4672933	0.4763612	0.5263443	0.4403348	0.5186806
MBD5	0.3917874	0.4752193	0.3903304	0.3767486	1.2302228
EPC2	0.4342434	0.4122886	0.4393167	0.4309489	0.4942276
KIF5C-AS1	0.0112396	0.0068452	0.0054009	0.0151523	0.0186567
KIF5C	1.6153621	1.9609972	1.9349069	1.4673748	1.8473644
ENSG00000289474	2.8870684	2.9548206	2.8110328	2.7220941	2.9561718
ENSG00000286167	0.0010015	0.0000000	0.0000000	0.0000000	0.0114712
LYPD6B	0.0146851	0.0381093	0.0342414	0.0231252	0.0386591
ENSG00000286335	0.0000000	0.0000000	0.0000000	0.0000000	0.0000000
LYPD6	0.0256893	0.0564448	0.0188148	0.0212411	0.0876760
MMADHC	0.6111556	0.6681527	0.6959844	0.5336741	0.5474165
MMADHC-DT	0.0165292	0.0277971	0.0218803	0.0227794	0.1093616
LINC01931	0.0033116	0.0033981	0.0113737	0.0025494	0.0169441
LINC01818	0.0013861	0.0011361	0.0000000	0.0000000	0.0000000
RND3	0.9001204	0.7409077	0.7044600	0.7644197	0.7353670
ENSG00000222031	0.0000000	0.0000000	0.0025033	0.0000000	0.0040112
RBM43	0.1261920	0.0424179	0.0268775	0.1616357	0.0653991

NMI	0.0191837	0.0041386	0.0153068	0.0405103	0.0015171
TNFAIP6	0.0023558	0.0020140	0.0000000	0.0017529	0.0000000
RIF1	0.4369296	0.4466871	0.4807051	0.3667893	0.6266259
NEB	0.0292653	0.0388962	0.0355536	0.0188833	0.0600225
ARL5A	0.7062305	0.6376160	0.7062433	0.6349505	0.6348966
ENSG00000288066	0.0392604	0.0622253	0.0467532	0.0511680	0.0447879
CACNB4	0.1768977	0.2801259	0.2902286	0.1789777	0.6481748
ENSG00000225214	0.0006262	0.0032251	0.0000000	0.0000000	0.0012623
STAM2	0.3503671	0.3300356	0.3332217	0.3375333	0.4001861
ENSG00000286234	0.0012666	0.0000000	0.0000000	0.0017263	0.0117334
FMNL2	0.7909108	0.6246993	0.4946881	0.8445326	1.0290742
ENSG00000286207	0.0012073	0.0023848	0.0011764	0.0000000	0.0000000
PRPF40A	0.8601459	0.7399212	0.7728087	0.8286149	0.7236181
ARL6IP6	0.3378052	0.2867049	0.2167596	0.3959479	0.3145141
ENSG00000227400	0.0005505	0.0095900	0.0022769	0.0020158	0.0188332
RPRM	0.3607737	0.8390275	1.2501659	0.3568601	0.5210684
GALNT13	0.3766126	0.3887918	0.4814669	0.2545155	0.9960721
ENSG00000224675	0.0010507	0.0008169	0.0028661	0.0000000	0.0126388
ENSG00000287900	0.0062087	0.0182279	0.0268992	0.0000000	0.0362492
KCNJ3	0.0639188	0.1060462	0.0855687	0.0770495	0.2289495
ENSG00000286679	0.0000000	0.0000000	0.0000000	0.0000000	0.0033732
LINC01876	0.0803215	0.0828525	0.0641743	0.0501518	0.3083406
ENSG00000282440	0.0000000	0.0000000	0.0000000	0.0000000	0.0000000
NR4A2	0.0210038	0.0412329	0.0212373	0.0529680	0.0651986
GPD2	0.1378931	0.1813213	0.2156024	0.1290416	0.2204418
GALNT5	0.0084475	0.0042295	0.0057684	0.0100575	0.0053681
ERMN	0.0653356	0.0490024	0.0276218	0.1925335	0.0246295
ACVR1C	0.0232587	0.0095064	0.0130696	0.0205702	0.0109136
ENSG00000234584	0.0037046	0.0064501	0.0021457	0.0018378	0.0030362
ACVR1	0.1794667	0.1037193	0.0705285	0.1681658	0.2468910
UPP2	0.0478516	0.0441870	0.0603395	0.0197710	0.1635264
ENSG00000270557	0.0028436	0.0008767	0.0031267	0.0000000	0.0000000
ENSG00000271320	0.0170723	0.0182126	0.0223431	0.0125316	0.0166751
CCDC148-AS1	0.0011056	0.0022876	0.0032050	0.0000000	0.0016917
CCDC148	0.1299688	0.0853624	0.1047116	0.1042595	0.1594915
PKP4	0.2024905	0.2226418	0.2599035	0.1509290	0.4541234
ENSG00000289488	0.0003465	0.0000000	0.0000000	0.0000000	0.0000000
PKP4-AS1	0.0009380	0.0000000	0.0041097	0.0000000	0.0022244
ENSG00000228586	0.0000000	0.0000000	0.0000000	0.0000000	0.0000000
DAPL1	0.0222931	0.0086888	0.0281825	0.0199230	0.0315285
TANC1	0.0662621	0.0500919	0.0234239	0.0959160	0.0723458
WDSUB1	0.1696102	0.1567509	0.1378312	0.1195133	0.2399379
BAZ2B	0.8583184	0.9978610	0.9433507	0.7425312	1.3364890
ENSG00000223642	0.0007138	0.0013262	0.0000000	0.0000000	0.0216558
BAZ2B-AS1	0.0554722	0.0374134	0.0379995	0.0990582	0.0190735
ENSG00000226266	0.0205963	0.0218449	0.0447946	0.0381639	0.0621305
MARCHF7	0.6917728	0.5785851	0.5538412	0.6803116	0.6970332
CD302	0.1077362	0.0793704	0.0309258	0.3115591	0.0733948
LY75	0.0042843	0.0011128	0.0027084	0.0073462	0.0021172
PLA2R1	0.0213036	0.0215002	0.0227740	0.0294966	0.0512310
ENSG00000285155	0.0042233	0.0000000	0.0000000	0.0000000	0.0000000
LINC02478	0.0370422	0.0346323	0.0193957	0.0556762	0.0461595
RBMS1	0.5753976	0.5524618	0.3309109	0.5272342	0.6290245

TANK-AS1	0.0006536	0.0062329	0.0000000	0.0000000	0.0167619
TANK	0.3293834	0.3441820	0.2788289	0.4284473	0.4104615
PSMD14-DT	0.0010652	0.0042528	0.0008833	0.0000000	0.0016664
PSMD14	0.6739017	0.6247055	0.6007292	0.7136351	0.5047356
TBR1	0.0010594	0.0000000	0.0000000	0.0000000	0.0000000
SLC4A10	0.1383629	0.2254675	0.1674063	0.1646971	0.5157887
ENSG00000288091	0.0032284	0.0120207	0.0063483	0.0000000	0.0400779
DPP4	0.0011912	0.0000000	0.0000000	0.0000000	0.0000000
GCG	0.0236605	0.0343632	0.0141299	0.0216645	0.0427706
FAP	0.0041202	0.0000000	0.0028329	0.0011506	0.0000000
IFIH1	0.0209218	0.0101520	0.0000000	0.0238472	0.0034190
GCA	0.3466447	0.2390385	0.2490817	0.3756667	0.2273707
KCNH7	0.1966034	0.1883566	0.1710032	0.1696600	0.8667888
KCNH7-AS1	0.0008547	0.0018587	0.0000000	0.0000000	0.0324887
FIGN	0.0890692	0.0930939	0.0856293	0.0654054	0.2012324
ENSG00000237844	0.0028415	0.0000000	0.0000000	0.0022936	0.0040357
GRB14	0.0799707	0.0892864	0.0651444	0.1000555	0.0862751
COBLL1	0.2035107	0.0782284	0.0528595	0.2400549	0.1209451
ENSG00000236283	0.0052961	0.0040209	0.0096265	0.0044165	0.0149929
SCN3A	0.3117957	0.4981049	0.6743962	0.3063309	0.7096880
SCN2A	0.3290525	0.5526507	0.7968349	0.2804828	0.7555690
ENSG00000288089	0.0000000	0.0028246	0.0035031	0.0000000	0.0000000
CSRNP3	0.8261401	1.0373736	1.2055650	0.7995206	1.2401305
GALNT3	0.6698088	0.3752417	0.2122375	0.7358512	0.3666868
ENSG00000235192	0.2564562	0.1473686	0.1050348	0.1353930	0.1279537
ENSG00000229195	0.0130374	0.0149837	0.0000000	0.0193620	0.0143634
TTC21B	0.2689867	0.2428212	0.1801993	0.2083535	0.4280590
TTC21B-AS1	0.0010606	0.0000000	0.0000000	0.0000000	0.0000000
SCN1A-AS1	0.0123713	0.0267636	0.0079273	0.0256519	0.1369767
SCN1A	0.1787219	0.2403117	0.2405152	0.1957060	0.4571870
SCN9A	0.3325999	0.4882944	0.4626553	0.3089080	0.8825717
SCN7A	0.0526322	0.0674356	0.0565397	0.0468625	0.1334959
XIRP2	0.0049029	0.0000000	0.0000000	0.0019410	0.0000000
B3GALT1	0.1087892	0.1456087	0.1041905	0.1540325	0.4769789
B3GALT1-AS1	0.0025438	0.0037222	0.0000000	0.0129735	0.0350044
STK39	0.4632099	0.5561326	0.4961599	0.4565826	0.7337785
CERS6	0.5518389	0.7022344	0.7875612	0.5548197	1.1992765
CERS6-AS1	0.0079429	0.0052296	0.0053106	0.0016011	0.0269704
NOSTRIN	0.0038329	0.0062254	0.0032321	0.0000000	0.0196917
SPC25	0.0084582	0.0098490	0.0128838	0.0178557	0.0071854
ABCB11	0.0014150	0.0000000	0.0000000	0.0039481	0.0000000
LRP2	0.3688119	0.2357918	0.0981473	0.7237234	0.1951372
BBS5	0.2231814	0.1634983	0.1265479	0.3073548	0.1595873
KLHL41	0.0429440	0.0244396	0.0100076	0.0105846	0.0131890
FASTKD1	0.1072844	0.1326464	0.1555907	0.0972255	0.1853476
PPIG	1.1510793	0.9970379	0.9946477	1.0683152	0.8267236
CFAP210	0.4955715	0.3050610	0.2383783	0.5187731	0.2962721
PHOSPHO2	0.0742354	0.0589489	0.0534291	0.0618771	0.0847141
KLHL23	0.5464661	0.7884487	0.9257863	0.5396786	0.7282485
SSB	1.5981638	1.3961208	1.4165364	1.6352840	1.1644469
METTL5	0.6397783	0.6763472	0.7040370	0.6090059	0.5733171
UBR3	0.3388062	0.3362495	0.3494495	0.2847097	0.7855217
MYO3B	0.0036289	0.0014583	0.0000000	0.0000000	0.0088091

ENSG00000213981	0.0047354	0.0013043	0.0054923	0.0000000	0.0043425
ERICH2-DT	0.0246434	0.0245969	0.0214577	0.0121989	0.0213311
SP5	0.0011737	0.0022073	0.0000000	0.0000000	0.0035216
ERICH2	0.2546450	0.2226660	0.1962280	0.2327073	0.1605041
GAD1	0.0099703	0.0127026	0.0233233	0.0024525	0.0122545
ENSG00000235934	0.0019075	0.0000000	0.0019877	0.0000000	0.0000000
ENSG00000286115	0.0006885	0.0024556	0.0023088	0.0000000	0.0000000
GORASP2	0.3817861	0.3227382	0.3136274	0.3982049	0.3091522
TLK1	0.6175352	0.5586786	0.5158589	0.5692063	0.6266050
METTL8	0.1611539	0.1486815	0.1389550	0.0849564	0.1348524
DCAF17	0.1825885	0.2331178	0.2655765	0.1153375	0.2483612
CYBRD1	0.0179864	0.0079603	0.0055018	0.0205566	0.0149752
DYNC1I2	1.4132857	1.3809839	1.4609393	1.4310493	1.2607304
SLC25A12	0.1866336	0.2693494	0.2597328	0.2152325	0.4524430
HAT1	0.3416929	0.2362594	0.1930260	0.3756323	0.2785746
METAP1D	0.0222094	0.0194269	0.0174040	0.0263148	0.0382397
DLX1	0.0005624	0.0019268	0.0058223	0.0000000	0.0000000
DLX2	0.0000000	0.0000000	0.0048936	0.0000000	0.0022158
ENSG00000232555	0.0031625	0.0017918	0.0000000	0.0032553	0.0037043
ENSG00000288048	0.0033876	0.0204197	0.0161997	0.0000000	0.0048079
ITGA6	0.2757685	0.2412136	0.2356512	0.4173766	0.2882942
ITGA6-AS1	0.0092094	0.0077877	0.0088230	0.0011694	0.0048241
PDK1-AS1	0.0196607	0.0107967	0.0287793	0.0075855	0.0039758
PDK1	0.3068467	0.4598856	0.4552969	0.2577059	0.4479873
RAPGEF4-AS1	0.0091215	0.0057219	0.0163362	0.0013375	0.0238130
RAPGEF4	0.1253561	0.1534826	0.1528308	0.0956974	0.4241134
MAP3K20	0.2442932	0.1195659	0.0676191	0.2192917	0.2008079
MAP3K20-AS1	0.0000000	0.0034569	0.0000000	0.0000000	0.0036228
CDCA7	0.0342544	0.0172408	0.0221173	0.0406969	0.0201774
SP3	0.2714484	0.2504480	0.2328626	0.2948867	0.3265691
ENSG00000289296	0.0007287	0.0000000	0.0000000	0.0037517	0.0021475
OLA1	1.0839390	1.2723585	1.3570077	1.0416958	1.0906522
CIR1	0.7511856	0.5006061	0.4996307	0.6515511	0.4827829
SCRN3	0.3295778	0.3247170	0.3088890	0.2696821	0.2866971
GPR155	0.1593086	0.1745267	0.1715426	0.2077378	0.2577832
GPR155-DT	0.0530757	0.0391823	0.0238290	0.0581170	0.0172090
ENSG00000236449	0.0029164	0.0041625	0.0021501	0.0061699	0.0016673
WIPF1	0.0993636	0.0875899	0.0568141	0.1343880	0.1432583
ENSG00000290018	0.0811005	0.0685986	0.0718921	0.0934111	0.0514736
CHRNA1	0.0000000	0.0000000	0.0028329	0.0000000	0.0071623
CHN1	0.4575576	0.5921926	0.6172966	0.4443097	0.7915574
ATF2	0.5522568	0.6224305	0.7880162	0.6043957	0.6680299
ATP5MC3	1.5091602	1.6820500	1.7788050	1.5312631	1.3435148
ENSG00000229066	0.0004291	0.0050089	0.0000000	0.0000000	0.0000000
ENSG00000289349	0.0018484	0.0038598	0.0016185	0.0000000	0.0000000
LNPK	0.7189248	0.7120069	0.7941445	0.7807406	0.7356413
EVX2	0.0020925	0.0043238	0.0106609	0.0000000	0.0116957
HOXD-AS2	0.0159398	0.0153699	0.0224648	0.0245082	0.0123429
HOXD9	0.0000000	0.0000000	0.0000000	0.0000000	0.0000000
HOXD8	0.0178397	0.0156350	0.0284092	0.0100603	0.0135519
HOXD3	0.4148448	0.7051058	1.1754765	0.3490605	0.6792226
HOXD4	0.1805225	0.2949390	0.5785003	0.1196868	0.1807477
HAGLR	0.0367617	0.0565528	0.0546773	0.0145754	0.0538356

ENSG00000272729	0.0007910	0.0082587	0.0184910	0.0042750	0.0107024
HAGLROS	0.0900454	0.1265015	0.2057294	0.0705878	0.0859411
HOXD1	0.0583613	0.0357659	0.0597944	0.0166844	0.0354191
MTX2	0.5536680	0.6538832	0.7915052	0.5162440	0.6479974
LINC01117	0.0191752	0.0146103	0.0305796	0.0018727	0.0500450
LINC01116	0.0166692	0.0396496	0.0312684	0.0109659	0.0212333
ENSG00000230552	0.0054547	0.0032882	0.0027138	0.0024545	0.0231613
ENSG00000227098	0.0000000	0.0000000	0.0000000	0.0000000	0.0000000
ENSG00000236501	0.0076072	0.0086752	0.0130777	0.0000000	0.0336778
ENSG00000229337	0.0365049	0.0295298	0.0102791	0.0321003	0.0398608
HNRNPA3	1.5842596	1.6013351	1.6403234	1.4774053	1.4602218
NFE2L2	0.8769687	0.5048120	0.4156679	0.8513730	0.4748376
ENSG00000222043	0.0184478	0.0082594	0.0058546	0.0086634	0.0152512
ENSG00000213963	0.0516798	0.0409830	0.0398383	0.0340912	0.0817905
ENSG00000271996	0.0000000	0.0016424	0.0000000	0.0000000	0.0000000
AGPS	0.4063004	0.4397668	0.3759352	0.3784401	0.5809127
TTC30B	0.1250268	0.0834420	0.0469048	0.1275965	0.0500830
ENSG00000237655	0.0000000	0.0011466	0.0000000	0.0000000	0.0065249
TTC30A	0.0570449	0.0428571	0.0256093	0.0529088	0.0179911
PDE11A	0.0167970	0.0155205	0.0252289	0.0119603	0.0470020
PDE11A-AS1	0.0019704	0.0000000	0.0032963	0.0000000	0.0144253
RBM45	0.1094760	0.0814261	0.0881009	0.1114636	0.0846197
OSBPL6	0.2374741	0.3243020	0.3035717	0.1579354	0.6007041
CHROMR	0.1485017	0.1379021	0.1443857	0.1419548	0.2251215
PRKRA	0.6373959	0.5296632	0.6801478	0.5647983	0.4635057
PJVK	0.0315679	0.0354762	0.0090182	0.0302714	0.0204959
FKBP7	0.0718361	0.0838362	0.0923076	0.0718879	0.0570611
PLEKHA3	0.7297963	0.6556244	0.6216591	0.7204083	0.5670958
TTN-AS1	0.1291828	0.1283709	0.1080875	0.0931149	0.4300471
TTN	0.0084134	0.0058350	0.0055586	0.0000000	0.0419443
ENSG00000270956	0.0018838	0.0014114	0.0000000	0.0000000	0.0000000
ENSG00000270277	0.0023277	0.0011620	0.0000000	0.0000000	0.0000000
ENSG00000271011	0.0002971	0.0040905	0.0012944	0.0000000	0.0167659
ENSG00000270574	0.0015015	0.0022206	0.0017982	0.0000000	0.0000000
ENSG00000271141	0.0000000	0.0029487	0.0000000	0.0000000	0.0016664
ENSG00000271401	0.0024257	0.0000000	0.0000000	0.0014739	0.0000000
ENSG00000267784	0.0016718	0.0085086	0.0121978	0.0000000	0.0201050
ENSG00000287149	0.0000000	0.0024757	0.0000000	0.0000000	0.0026227
CCDC141	0.0004518	0.0000000	0.0056474	0.0024903	0.0118013
SESTD1	0.7062853	0.9448035	1.0518344	0.6444353	0.9432415
ZNF385B	0.1933241	0.0913535	0.0508802	0.0784979	0.1577378
CWC22	0.3859843	0.3323381	0.3251187	0.3431023	0.2998975
ENSG00000225258	0.0009404	0.0020012	0.0000000	0.0000000	0.0000000
SCHLAP1	0.0014214	0.0040227	0.0000000	0.0000000	0.0000000
UBE2E3	1.0774975	1.2718891	1.4458629	1.0436495	1.1920893
ENSG00000238171	0.0000000	0.0000000	0.0000000	0.0000000	0.0024068
LINC01934	0.0180221	0.0065918	0.0098002	0.0211540	0.0581478
ITGA4	0.0089728	0.0098490	0.0062951	0.0125426	0.0148723
CERKL	0.1019388	0.0547038	0.0258658	0.1073631	0.0329731
NEUROD1	0.0065016	0.0000000	0.0000000	0.0000000	0.0000000
ITPRID2-DT	0.0000000	0.0000000	0.0000000	0.0000000	0.0045452
ITPRID2	1.1306704	0.8010850	0.5643323	1.0486542	0.7595843
PPP1R1C	0.1517375	0.4284956	0.1469246	0.1160019	0.2018613

PDE1A	0.0263554	0.0076218	0.0198445	0.0221360	0.0199170
DNAJC10	0.7700802	0.6163843	0.5029465	0.9104524	0.6394307
FRZB	0.0810699	0.2419453	0.1000308	0.1269515	0.1763096
NCKAP1	1.0342787	0.9721821	1.0229079	1.0154143	1.0180356
DUSP19	0.0812521	0.0944389	0.0821632	0.0874712	0.0569439
ENSG00000224643	0.0022478	0.0000000	0.0006116	0.0000000	0.0061151
NUP35	0.0930021	0.0964626	0.1018797	0.0868800	0.1677215
ENSG00000272800	0.0129504	0.0073861	0.0166523	0.0110329	0.0687053
ENSG00000287621	0.0191245	0.0126275	0.0148383	0.0019429	0.1625064
ENSG00000286152	0.0000000	0.0000000	0.0018147	0.0000000	0.0000000
ENSG00000286980	0.0033489	0.0012598	0.0008289	0.0016217	0.0000000
ENSG00000283839	0.0124823	0.0281066	0.0289165	0.0164765	0.0264750
ZNF804A	0.2908463	0.4122943	0.3887078	0.3072682	1.1041538
ENSG00000286797	0.0936153	0.0853418	0.0820409	0.0763373	0.3355799
FSIP2-AS1	0.0218292	0.0286236	0.0372193	0.0242624	0.0956199
FSIP2-AS2	0.0000000	0.0000000	0.0014218	0.0000000	0.0000000
FSIP2	0.1474742	0.1669774	0.1148585	0.1082266	0.3519457
ENSG00000287129	0.0051837	0.0025377	0.0057414	0.0030518	0.0060503
LINC01473	0.1752897	0.1759295	0.1263406	0.1667267	0.5804193
ENSG00000259915	0.0048664	0.0041198	0.0051467	0.0000000	0.0000000
ZC3H15	1.0915027	1.2363125	1.3618824	1.0187716	0.9210735
ITGAV	0.5100937	0.3568913	0.2490089	0.7662305	0.5238218
ENSG00000227227	0.0029891	0.0026938	0.0026358	0.0033262	0.0158642
FAM171B	0.4738166	0.6220355	0.8439350	0.4634895	0.6707797
ZSWIM2	0.0037089	0.0025510	0.0060884	0.0000000	0.0042903
CALCRL-AS1	0.0024857	0.0011207	0.0062290	0.0070958	0.0143786
CALCRL	0.0988402	0.0541428	0.0200601	0.1250293	0.0530269
TFPI	0.1175162	0.0597182	0.0228012	0.6157371	0.1279731
LINC01090	0.0011952	0.0025016	0.0011907	0.0010066	0.0067724
GULP1	0.0769511	0.0700734	0.0629206	0.0775109	0.1909804
ENSG00000223523	0.0000000	0.0037340	0.0000000	0.0000000	0.0000000
COL3A1	0.0727106	0.0695070	0.1095745	0.0992253	0.2709135
COL5A2	0.1195145	0.1006910	0.0372451	0.2466804	0.0879034
ENSG00000288866	0.0060378	0.0017974	0.0000000	0.0038335	0.0000000
WDR75	0.2405583	0.2362735	0.2335650	0.2244752	0.2299735
SLC40A1	0.2818717	0.1821540	0.1466072	0.2197331	0.1214098
ASNSD1	0.3109746	0.3723311	0.4535666	0.3001755	0.2991171
ASDURF	0.0060253	0.0038936	0.0016462	0.0000000	0.0000000
ANKAR	0.0723853	0.1069086	0.0765129	0.0340468	0.1991365
OSGEPL1	0.1494747	0.1453528	0.0869115	0.1174043	0.0783804
OSGEPL1-AS1	0.0091485	0.0110619	0.0142888	0.0101069	0.0229474
ENSG00000273240	0.0008804	0.0020344	0.0000000	0.0021003	0.0000000
ORMDL1	0.6965102	0.6035475	0.7137134	0.7208069	0.5537291
PMS1	0.2205812	0.2661972	0.2741850	0.2177725	0.3873434
C2orf88	0.0875453	0.0606636	0.0448956	0.0460350	0.0854162
MSTN	0.0000000	0.0010959	0.0000000	0.0000000	0.0000000
HIBCH	0.4983862	0.4045239	0.3498112	0.5525777	0.5484397
INPP1	0.2492955	0.2472022	0.3367546	0.2454900	0.3417275
MFSD6	0.2195315	0.2105666	0.2282269	0.2879011	0.3034075
NEMP2	0.0177873	0.0112477	0.0047211	0.0129912	0.0296143
NEMP2-DT	0.0345795	0.0623523	0.0989748	0.0358537	0.0704851
ENSG00000284052	0.0187025	0.0224019	0.0184387	0.0099017	0.0354639
NAB1	0.2185749	0.1560424	0.1027563	0.1828360	0.1076578

ENSG00000228509	0.0015531	0.0003807	0.0000000	0.0000000	0.0000000
ENSG00000235852	0.0012248	0.0017769	0.0000000	0.0000000	0.0000000
GLS	0.4066915	0.6254690	0.7507422	0.3587719	0.7070072
STAT1	0.3004516	0.2099833	0.2176538	0.3412207	0.2690800
STAT4-AS1	0.0005138	0.0028610	0.0000000	0.0000000	0.0083608
STAT4	0.0245001	0.0311054	0.0373709	0.0140835	0.1432682
ENSG00000288064	0.0000000	0.0000000	0.0000000	0.0000000	0.0000000
MYO1B-AS1	0.0061622	0.0065370	0.0000000	0.0022251	0.0014182
MYO1B	0.1730150	0.2684495	0.3540383	0.1352874	0.3720486
ENSG00000288900	0.0008252	0.0031245	0.0011326	0.0000000	0.0000000
NABP1	0.0166346	0.0331213	0.0152985	0.0195585	0.0211891
CAVIN2	0.0517641	0.0117259	0.0069629	0.0448307	0.0192314
CAVIN2-AS1	0.0031577	0.0077210	0.0000000	0.0000000	0.0193970
TMEFF2	0.1682457	0.2975316	0.2555817	0.1463126	0.4705000
ENSG00000286358	0.0295716	0.0528316	0.0536098	0.0287307	0.0099680
ENSG00000260142	0.0000000	0.0000000	0.0000000	0.0000000	0.0000000
LINC01821	0.0000000	0.0018553	0.0077760	0.0019814	0.0000000
SLC39A10	0.5091376	0.5670926	0.7564014	0.6827700	0.6231657
DNAH7	0.2865188	0.2093664	0.1313312	0.2769316	0.2992930
STK17B	0.0183290	0.0074735	0.0045493	0.0178461	0.0293050
ENSG00000272211	0.0000000	0.0000000	0.0000000	0.0000000	0.0000000
HECW2	0.2430418	0.2651562	0.2166828	0.2198752	0.6126594
CCDC150	0.0087619	0.0099014	0.0121246	0.0099977	0.0507761
GTF3C3	0.2332179	0.2120703	0.1774383	0.2461409	0.1725020
C2orf66	0.0019719	0.0031502	0.0000000	0.0022783	0.0013244
PGAP1	0.3009780	0.3604282	0.5814103	0.3160390	0.6610749
ANKRD44	0.0894745	0.1569474	0.1230666	0.0919551	0.4152863
ANKRD44-IT1	0.0000000	0.0014800	0.0000000	0.0000000	0.0000000
ANKRD44-DT	0.0000000	0.0000000	0.0015395	0.0000000	0.0000000
SF3B1	0.8954461	0.8001855	0.8332232	0.8894805	0.8571989
COQ10B	0.2702018	0.2474589	0.2331529	0.2625846	0.2048871
HSPD1	2.2913468	2.0979300	2.0266146	2.3055955	1.7532837
HSPE1	1.4727337	1.3810101	1.3815372	1.6075046	1.0209815
MOB4	0.5950572	0.6045022	0.6748882	0.5874854	0.6361378
RFTN2	0.1420375	0.0656296	0.0382185	0.1220194	0.0596207
ENSG00000222017	0.0149365	0.0031154	0.0083623	0.0033091	0.0067946
MARS2	0.0168897	0.0345996	0.0467805	0.0365386	0.0229878
PLCL1	0.8381907	0.7555248	0.5885747	0.7316149	1.1225085
ENSG00000286020	0.0008671	0.0042915	0.0000000	0.0000000	0.0063055
ENSG00000225421	0.0000000	0.0000000	0.0000000	0.0000000	0.0112465
ENSG00000231557	0.0000000	0.0000000	0.0021479	0.0066452	0.0000000
SATB2	0.0942438	0.0471159	0.0173115	0.0383635	0.0766860
SATB2-AS1	0.0510788	0.0316652	0.0222474	0.0426546	0.0551307
LINC01877	0.0037342	0.0047795	0.0029183	0.0012236	0.0000000
FTCDNL1	0.0315075	0.0414988	0.0288397	0.0423758	0.0305196
ENSG00000232732	0.0000000	0.0007002	0.0017966	0.0000000	0.0012770
C2orf69	0.1979295	0.2409879	0.3229732	0.1402007	0.2671492
TYW5	0.0901244	0.1006237	0.0774513	0.0700710	0.1022510
MAIP1	0.1749598	0.2050456	0.1816065	0.1854642	0.1713846
ENSG00000287027	0.0000000	0.0020990	0.0000000	0.0000000	0.0032553
SPATS2L	1.0247685	0.8693498	0.9149514	1.0953675	0.8101207
ENSG00000287299	0.0044073	0.0030461	0.0000000	0.0073491	0.0078167
KCTD18	0.1782885	0.1280220	0.0706423	0.1333278	0.1078353

SGO2	0.0718727	0.0733141	0.0769745	0.0577657	0.0750846
AOX1	0.0000000	0.0039625	0.0013959	0.0000000	0.0000000
BZW1-AS1	0.0011329	0.0014091	0.0015418	0.0038158	0.0000000
BZW1	1.2570899	1.3432210	1.4828674	1.2811424	1.0410116
CLK1	0.2276396	0.2059507	0.2282080	0.1904466	0.2835080
PPIL3	0.2439578	0.1909132	0.1877976	0.2971493	0.1846733
NIF3L1	0.1556266	0.1531345	0.1400890	0.1326175	0.1251605
ORC2	0.1134673	0.0943132	0.0920584	0.1069745	0.1598038
ENSG00000183308	0.0099100	0.0102647	0.0041078	0.0192854	0.0319127
HYCC2	0.1650498	0.3223003	0.3234362	0.1562925	0.3674841
NDUFB3	0.8667159	0.8023522	0.8698491	0.8820713	0.6284387
CFLAR	0.2212932	0.1716949	0.1378104	0.1739120	0.1973304
CFLAR-AS1	0.0006958	0.0000000	0.0000000	0.0042283	0.0000000
CASP10	0.0019394	0.0013789	0.0000000	0.0022345	0.0000000
CASP8	0.0509901	0.0274952	0.0112969	0.0587345	0.0389951
FLACC1	0.0188743	0.0220625	0.0286980	0.0201399	0.0108846
TRAK2	0.2990201	0.3362559	0.2794178	0.2025739	0.3195086
STRADB	0.2778891	0.3000296	0.3061814	0.2884252	0.2174376
C2CD6	0.0052755	0.0102167	0.0080312	0.0180097	0.0182444
TMEM237	0.2101291	0.2023662	0.1645200	0.2078781	0.1908620
ENSG00000287133	0.0009205	0.0031473	0.0000000	0.0000000	0.0037415
MPP4	0.0006326	0.0073751	0.0014933	0.0071147	0.0259303
ALS2	0.1773432	0.1718983	0.1620868	0.1729642	0.2497497
CDK15	0.0000000	0.0022897	0.0009415	0.0130417	0.0000000
ENSG00000273209	0.0150976	0.0053650	0.0108140	0.0084599	0.0141239
FZD7	0.1759091	0.0747851	0.0489957	0.3114233	0.0949088
KIAA2012	0.1140628	0.0751400	0.0630380	0.1124071	0.1095822
KIAA2012-AS1	0.0033732	0.0050342	0.0012796	0.0132858	0.0086529
ENSG00000287041	0.0042970	0.0009429	0.0008221	0.0000000	0.0044810
ENSG00000231903	0.0036316	0.0104268	0.0118400	0.0089048	0.0032078
SUMO1	1.0337891	1.0987618	1.1417597	1.0567441	0.7544307
NOP58	0.4903405	0.4800819	0.4901774	0.4886608	0.4811035
ENSG00000286223	0.0011311	0.0000000	0.0050430	0.0000000	0.0050239
ENSG00000272966	0.0000000	0.0000000	0.0000000	0.0000000	0.0000000
ENSG00000273456	0.0557396	0.0649011	0.0734044	0.0420336	0.0468932
BMPR2	0.5593229	0.7047759	0.7638607	0.5839041	1.2304539
FAM117B	0.1682316	0.2793725	0.3273837	0.1630252	0.4075662
ICA1L	0.2775556	0.2842952	0.2961386	0.2606380	0.5680559
WDR12	0.2913327	0.3355130	0.2735068	0.3023606	0.2922546
CARF	0.1461605	0.1221950	0.1154891	0.1229743	0.1584058
NBEAL1	0.3206912	0.3282779	0.2162513	0.2329188	0.5224301
ENSG00000289294	0.0915260	0.0897023	0.0819455	0.0849753	0.1158764
CYP20A1	0.3338797	0.3263552	0.3259081	0.2943935	0.2573173
ABI2	0.8284819	0.9881202	1.1395389	0.8811804	1.0288738
RAPH1	0.2776455	0.4139091	0.4934357	0.2947492	0.5730647
ENSG00000237271	0.0021876	0.0061826	0.0028368	0.0000000	0.0068537
PARD3B	0.4445132	0.3322489	0.2319917	0.3262470	0.7061487
ENSG00000287524	0.0000000	0.0000000	0.0000000	0.0000000	0.0000000
NRP2	0.5106557	0.7154820	0.7251147	0.5585940	0.6313563
ENSG00000225610	0.0000000	0.0000000	0.0000000	0.0000000	0.0000000
INO80D	0.3146531	0.2913532	0.3231906	0.2864724	0.4694958
INO80D-AS1	0.0717558	0.0572917	0.0757271	0.0701825	0.0564598
NDUFS1	0.7136643	0.7526239	0.7870839	0.7384952	0.6883379

ENSG00000231955	0.0082294	0.0070874	0.0067168	0.0000000	0.0100047
EEF1B2	1.7171167	1.8905447	1.8872311	1.6215896	1.5858891
CMKLR2	0.0155792	0.0068239	0.0026333	0.0313778	0.0031362
CMKLR2-AS	0.0000000	0.0015363	0.0000000	0.0000000	0.0040877
ZDBF2	0.3731902	0.3480659	0.3808266	0.3873169	0.5269149
ADAM23	0.2805248	0.4025451	0.3914315	0.3392688	0.6777612
ENSG00000228577	0.0002195	0.0000000	0.0000000	0.0000000	0.0000000
FAM237A	0.0000000	0.0009027	0.0000000	0.0000000	0.0000000
DYTN	0.0010244	0.0018252	0.0000000	0.0000000	0.0022158
MDH1B	0.1021271	0.0434493	0.0240248	0.0891167	0.0697387
FASTKD2	0.2075745	0.2072076	0.2272039	0.1825849	0.1963829
ENSG00000229321	0.0209341	0.0327766	0.0160577	0.0218289	0.0527458
CPO	0.0015955	0.0033207	0.0040934	0.0000000	0.0000000
KLF7	0.3462003	0.6296674	0.6401738	0.2959677	0.6360062
KLF7-IT1	0.0000000	0.0018236	0.0000000	0.0000000	0.0165624
MYOSLID	0.0109322	0.0115411	0.0042979	0.0110390	0.0540635
ENSG00000234902	0.0000000	0.0014914	0.0000000	0.0000000	0.0035158
ENSG00000223725	0.0013853	0.0015987	0.0104495	0.0000000	0.0112709
CREB1	0.4440241	0.4787564	0.5816344	0.4109004	0.4983443
METTL21A	0.1902168	0.1739941	0.1480931	0.2153727	0.1228411
CCNYL1	0.1120521	0.0712087	0.1061785	0.1315327	0.1191877
FZD5	0.0141542	0.0182980	0.0210687	0.0142336	0.0062535
PLEKHM3	0.1041951	0.2011918	0.1589511	0.0910716	0.2203226
CRYGD	0.0086579	0.0165996	0.0151187	0.0000000	0.0031033
CRYGA	0.0009065	0.0000000	0.0000000	0.0000000	0.0000000
C2orf80	0.1749629	0.3015460	0.5828596	0.1424333	0.2378881
IDH1	0.3945251	0.5290655	0.6121953	0.4584220	0.4325408
IDH1-AS1	0.0429213	0.0139823	0.0052871	0.0392154	0.0103561
PIKFYVE	0.2290495	0.2345665	0.2490399	0.2458034	0.3499014
PTH2R	0.0366784	0.0165942	0.0095229	0.0088018	0.0042839
MAP2	1.7283941	2.3818265	2.7142301	1.6427624	2.2998137
UNC80	0.1616062	0.2016015	0.1926985	0.1285076	0.5009911
RPE	0.2081673	0.2010310	0.1763192	0.1819591	0.1749394
KANSL1L	0.2653630	0.2370842	0.2481179	0.2338087	0.4091501
ENSG00000272807	0.0037328	0.0076557	0.0084201	0.0040641	0.0105086
KANSL1L-AS1	0.0100763	0.0228433	0.0108985	0.0149421	0.0651804
ENSG00000279317	0.0116620	0.0132837	0.0073348	0.0091089	0.0242533
ACADL	0.0068642	0.0029860	0.0069683	0.0197267	0.0277425
LANCL1-AS1	0.0002827	0.0041682	0.0000000	0.0000000	0.0073536
LANCL1	0.2507123	0.3602800	0.3160270	0.2687178	0.2929420
CPS1	0.0256083	0.0189545	0.0081146	0.0204040	0.0096035
ERBB4	0.2712419	0.2979115	0.1794622	0.2353758	0.9381504
ENSG00000273118	0.0138030	0.0073055	0.0080990	0.0070677	0.0367228
IKZF2	0.0932528	0.0707218	0.0576047	0.0652696	0.1712240
ENSG00000270659	0.0645791	0.0421361	0.0183886	0.0470521	0.0141321
ENSG00000289002	0.0029161	0.0000000	0.0000000	0.0000000	0.0000000
ENSG00000272519	0.0149295	0.0105333	0.0085830	0.0023493	0.0228920
SPAG16-DT	0.0052288	0.0109655	0.0000000	0.0056371	0.0000000
SPAG16	1.3901291	1.0685325	0.8599599	1.4621681	0.9816442
ENSG00000197585	0.0067705	0.0122102	0.0103633	0.0087286	0.0445896
VWC2L	0.0493477	0.1308317	0.0677924	0.0700533	0.2680397
BARD1	0.1466703	0.1091714	0.0754034	0.1845203	0.0956164
SNHG31	0.0439209	0.0434494	0.0310860	0.0661769	0.0436176

ABCA12	0.0026033	0.0027793	0.0000000	0.0000000	0.0034327
ENSG00000227769	0.0000000	0.0021652	0.0000000	0.0000000	0.0096359
ENSG00000286836	0.0012179	0.0073982	0.0139889	0.0103126	0.0080136
ATIC	0.2777772	0.2572959	0.2394948	0.3429664	0.2625359
FN1	0.0821007	0.0422118	0.0293224	0.0919947	0.0606631
ENSG00000225166	0.0006609	0.0000000	0.0031919	0.0000000	0.0000000
ENSG00000237525	0.0000000	0.0053362	0.0000000	0.0000000	0.0000000
LINC00607	0.0034165	0.0000000	0.0000000	0.0000000	0.0058124
ENSG00000226276	0.0000000	0.0000000	0.0000000	0.0000000	0.0000000
MREG	0.0214449	0.0292646	0.0259865	0.0240225	0.0977835
PECR	0.0652782	0.0451593	0.0494070	0.0747657	0.1103002
TMEM169	0.1279992	0.2740452	0.2713998	0.0963503	0.2032025
XRCC5	1.3842202	1.3573755	1.6394978	1.3486046	1.2292026
LINC01963	0.0258912	0.0260655	0.0511084	0.0202520	0.0203353
MARCHF4	0.0540298	0.1256901	0.1368322	0.0345275	0.2567287
ENSG00000231092	0.0000000	0.0000000	0.0000000	0.0000000	0.0000000
ENSG00000233581	0.0000000	0.0000000	0.0000000	0.0000000	0.0000000
SMARCAL1	0.1213978	0.1036072	0.1143862	0.1481414	0.1988694
RPL37A-DT	0.0229804	0.0142994	0.0136345	0.0277263	0.0109192
ENSG00000241520	0.0030671	0.0041698	0.0029800	0.0033434	0.0000000
RPL37A	3.3378845	3.3287094	3.1129715	3.2255512	3.1019357
LINC01280	0.0106602	0.0021298	0.0132128	0.0000000	0.0117823
ENSG00000225794	0.0015604	0.0025759	0.0010030	0.0000000	0.0182140
IGFBP2	2.8847875	2.3409771	2.2644605	3.2202051	2.2623362
IGFBP5	2.3303389	1.7476049	1.3758159	2.4752944	1.8043686
DIRC3-AS1	0.0902088	0.1037545	0.0842225	0.0659222	0.3414836
ENSG00000287498	0.0010022	0.0000000	0.0000000	0.0000000	0.0015072
DIRC3	0.0344073	0.0429271	0.0625238	0.0361699	0.2448926
TNS1	0.0389442	0.0685797	0.0594548	0.0412003	0.1359978
ARPC2	1.3286862	1.4687719	1.5760956	1.3175248	1.1557940
ENSG00000261338	0.0055450	0.0090567	0.0028096	0.0000000	0.0119029
GPBAR1	0.0023740	0.0006834	0.0000000	0.0000000	0.0000000
AAMP	0.3642887	0.3071689	0.2981848	0.3356695	0.2243080
PNKD	0.4836928	0.4351874	0.4168919	0.5275046	0.3898986
TMBIM1	0.0431247	0.0095928	0.0267400	0.0326038	0.0271967
CATIP-AS2	0.0004815	0.0000000	0.0000000	0.0000000	0.0000000
CATIP	0.0758743	0.0316889	0.0309455	0.0870878	0.0587020
CATIP-AS1	0.0038312	0.0049935	0.0032601	0.0015029	0.0055995
ENSG00000288819	0.0067288	0.0107115	0.0026075	0.0063474	0.0052273
SLC11A1	0.0078130	0.0047289	0.0017217	0.0019236	0.0137291
CTDSP1	0.2198330	0.1331976	0.0862264	0.2237768	0.1354643
VIL1	0.0039102	0.0007963	0.0000000	0.0000000	0.0060185
USP37	0.1749780	0.2005817	0.1649685	0.1234512	0.2544494
CNOT9	0.2339554	0.2687310	0.3375632	0.2456488	0.2591124
PLCD4	0.0176103	0.0282154	0.0099287	0.0096279	0.0734793
ZNF142	0.0627610	0.0852506	0.0783881	0.0651475	0.0805281
BCS1L	0.1117411	0.1544332	0.1744920	0.0932257	0.1221079
RNF25	0.1052203	0.0818281	0.1164308	0.1119039	0.1155429
STK36	0.0549573	0.0287915	0.0212297	0.0423981	0.0522648
TTLL4	0.0688879	0.0387516	0.0214626	0.0524923	0.0511535
CYP27A1	0.0842417	0.0395541	0.0568095	0.0908629	0.0256598
ENSG00000286154	0.0002592	0.0000000	0.0000000	0.0000000	0.0000000
WNT10A	0.0008552	0.0019362	0.0033800	0.0000000	0.0000000

LINC01494	0.0071987	0.0043105	0.0022017	0.0088894	0.0152603
ENSG00000288898	0.0018653	0.0010566	0.0000000	0.0000000	0.0000000
CDK5R2-AS1	0.0016796	0.0012869	0.0000000	0.0000000	0.0033679
CDK5R2	0.1505802	0.2778510	0.4105072	0.0916005	0.2566854
LINC00608	0.0018096	0.0022201	0.0006255	0.0000000	0.0000000
FEV	0.0961311	0.1012914	0.4408210	0.0257231	0.0754628
CRYBA2	0.0278401	0.0171389	0.0514354	0.0015624	0.0177352
CFAP65	0.0581066	0.0655680	0.0452110	0.0730822	0.0297326
IHH	0.0000000	0.0021629	0.0040298	0.0000000	0.0000000
NHEJ1	0.0434094	0.0240622	0.0431410	0.0237461	0.0493798
SLC23A3	0.0000000	0.0000000	0.0000000	0.0000000	0.0000000
CNPPD1	0.2540861	0.1951496	0.2190751	0.2461352	0.1916739
RETREG2	0.4289246	0.4688030	0.5348706	0.4428147	0.2923300
ZFAND2B	0.1579474	0.1470595	0.1060387	0.1327479	0.1452085
ABCB6	0.1969829	0.1289307	0.1064594	0.2992747	0.1540788
ATG9A	0.1145900	0.1192097	0.1410181	0.1787001	0.0875687
ANKZF1	0.1587543	0.1280875	0.0974346	0.0838757	0.1861285
GLB1L	0.1030935	0.0510741	0.0345747	0.1563960	0.0299084
STK16	0.2502027	0.2249618	0.2316603	0.1875613	0.1968560
TUBA4A	0.1447457	0.1265854	0.0869536	0.0998156	0.1330539
TUBA4B	0.0042000	0.0023881	0.0000000	0.0000000	0.0059153
DNAJB2	0.6288860	0.5138370	0.4235004	0.5537175	0.4102035
PTPRN	0.1296736	0.2309339	0.3260106	0.1039385	0.2193463
ENSG00000230432	0.0360369	0.0275396	0.0063366	0.0429110	0.0087488
RESP18	0.0196418	0.0756480	0.0385033	0.0228896	0.0362973
DNPEP	0.1362518	0.1568828	0.1338286	0.1497060	0.1100402
DNPEP-AS1	0.0023574	0.0038589	0.0046739	0.0020179	0.0000000
DES	0.0108218	0.0033421	0.0059636	0.0052311	0.0017986
SPEG	0.0484920	0.0644453	0.0663628	0.0472414	0.0961614
ASIC4-AS1	0.0024724	0.0012589	0.0000000	0.0000000	0.0000000
SPEGNB	0.0004918	0.0000000	0.0000000	0.0000000	0.0000000
GMPPA	0.0648138	0.0606282	0.0630697	0.0579812	0.0334770
ASIC4	0.0172317	0.0302793	0.0686813	0.0187053	0.0670282
CHPF	0.5039034	0.3229563	0.3667058	0.6108809	0.3083184
TMEM198	0.0391567	0.0816609	0.0796355	0.0399154	0.0260621
ENSG00000268896	0.0014592	0.0000000	0.0000000	0.0000000	0.0000000
OBSL1	0.5972257	0.5095605	0.4358664	0.5884585	0.4445940
ENSG00000269068	0.0395248	0.0327102	0.0409941	0.0558810	0.0103427
INHA	0.0076408	0.0124844	0.0084625	0.0035938	0.0245160
STK11IP	0.0306088	0.0289799	0.0201019	0.0453894	0.0384922
ENSG00000228973	0.0015272	0.0011997	0.0014054	0.0029183	0.0000000
SLC4A3	0.1001239	0.1965612	0.2231913	0.1212858	0.1562268
ENSG00000267919	0.0000000	0.0000000	0.0034163	0.0000000	0.0000000
LINC02832	0.0019895	0.0014724	0.0000000	0.0000000	0.0000000
ENSG00000239498	0.0000000	0.0000000	0.0000000	0.0000000	0.0000000
ENSG00000236451	0.0000000	0.0017225	0.0088405	0.0000000	0.0000000
ENSG00000224819	0.0000000	0.0011674	0.0000000	0.0000000	0.0071690
ENSG00000286272	0.0011883	0.0016844	0.0000000	0.0000000	0.0065149
ENSG00000290000	0.0009946	0.0010742	0.0011859	0.0000000	0.0053413
EPHA4	0.1741532	0.1878701	0.1243203	0.1809645	0.3394838
ENSG00000272944	0.0232410	0.0161430	0.0039895	0.0258865	0.0000000
ENSG00000234446	0.2004338	0.1260844	0.0493548	0.1359892	0.1055492
PAX3	0.0000000	0.0014953	0.0000000	0.0000000	0.0033946

ENSG00000288658	0.0231507	0.0523848	0.0108562	0.0207985	0.0352675
CT75	0.0168508	0.0479165	0.0091741	0.0155082	0.0262372
SGPP2	0.0180689	0.0235029	0.0158605	0.0105302	0.0569618
FARSB	0.3246793	0.3812149	0.5388360	0.3509963	0.4187678
MOGAT1	0.0007576	0.0000000	0.0013092	0.0000000	0.0028456
ACSL3	0.7637506	0.8436445	1.0224245	0.8774470	0.9564630
ACSL3-AS1	0.0010792	0.0000000	0.0007371	0.0000000	0.0000000
KCNE4	0.0131163	0.0133027	0.0065257	0.0391991	0.0035834
SCG2	0.8015280	0.9996521	1.9394868	1.3120079	1.0809133
AP1S3	0.0261244	0.0330103	0.0429455	0.0210443	0.0601580
WDFY1	0.2962724	0.2624588	0.2295553	0.2493001	0.2766370
MRPL44	0.3897401	0.4333617	0.4370441	0.3837260	0.3217717
SERPINE2	0.1886954	0.2697364	0.4993294	0.1707284	0.3533746
ENSG00000274629	0.0023711	0.0016714	0.0017369	0.0000000	0.0065609
CUL3	0.7789759	0.7469514	0.8301197	0.6765768	0.7922244
DOCK10	0.0390549	0.0751683	0.0423594	0.0478809	0.2070016
NYAP2	0.2708644	0.5815342	0.4400314	0.1831516	0.7574780
ENSG00000289265	0.0137450	0.0404825	0.0193108	0.0078141	0.0557568
IRS1	0.4520638	0.3171963	0.2455667	0.5358056	0.3004246
ENSG00000272622	0.0203331	0.0054231	0.0041029	0.0417349	0.0088656
ENSG00000290086	0.0030680	0.0020046	0.0000000	0.0053200	0.0097677
RHBDD1	0.1728095	0.1414048	0.1174909	0.1973266	0.2603908
COL4A4	0.0071573	0.0111761	0.0159971	0.0031511	0.0279442
COL4A3	0.0018664	0.0043369	0.0046397	0.0030582	0.0112068
MFF-DT	0.0187639	0.0250204	0.0290696	0.0272316	0.0463030
MFF	0.8213942	0.8369876	0.8900545	0.8119020	0.7262931
TM4SF20	0.0010476	0.0000000	0.0009118	0.0000000	0.0038839
AGFG1	0.8004327	0.6401805	0.6168146	0.8503694	0.7293432
C2orf83	0.0000000	0.0000000	0.0000000	0.0000000	0.0039666
SLC19A3	0.0049629	0.0062103	0.0071059	0.0011876	0.0000000
DAW1	0.2269593	0.1276609	0.0606476	0.2885172	0.1130169
SPHKAP	0.0381013	0.0449288	0.0495494	0.0174700	0.0422866
LINC01807	0.0083125	0.0056489	0.0067749	0.0000000	0.0242899
PID1	0.4733432	0.4504176	0.4855545	0.4222450	0.6914230
DNER	0.3913262	0.6067481	1.0770888	0.4571277	0.9120279
TRIP12	0.6602958	0.5790363	0.5539959	0.7604371	0.7346847
FBXO36	0.2212020	0.1594039	0.1677840	0.2432570	0.1732601
SLC16A14	0.2528010	0.2849528	0.3339371	0.3312599	0.3022481
ENSG00000225963	0.0018650	0.0034334	0.0020207	0.0000000	0.0058806
SP110	0.0559771	0.0264927	0.0235005	0.0575030	0.0397795
SP140	0.0028787	0.0035840	0.0034245	0.0000000	0.0122063
SP140L	0.0067458	0.0036606	0.0037639	0.0030776	0.0110461
SP100	0.0064203	0.0020897	0.0032342	0.0028219	0.0040262
CAB39	0.5273282	0.6144571	0.6251162	0.5028404	0.6063850
ITM2C	1.4625557	1.0484788	0.8956518	1.8922344	1.0499301
GCSIR	0.0011320	0.0024171	0.0103929	0.0000000	0.0172018
GPR55	0.0003598	0.0000000	0.0000000	0.0000000	0.0000000
C2orf72	0.0975128	0.0704217	0.0698293	0.1150823	0.0630296
ENSG00000261829	0.0026485	0.0059571	0.0032805	0.0000000	0.0125547
PSMD1	0.8065864	0.7666140	0.7552226	0.8234916	0.6681390
HTR2B	0.0026819	0.0052013	0.0024140	0.0087557	0.0070236
ARMC9	0.2405616	0.2040909	0.1812327	0.2000754	0.2918826
ENSG00000224376	0.0053504	0.0043348	0.0000000	0.0000000	0.0000000

B3GNT7	0.0130038	0.0076366	0.0126880	0.0097458	0.0063200
NCL	2.4197876	2.2570787	2.1989245	2.3631256	2.0596176
LINC00471	0.0517826	0.0625379	0.0316153	0.0217246	0.0611526
ENSG00000286942	0.0000000	0.0037690	0.0000000	0.0000000	0.0000000
NMUR1	0.0025883	0.0026315	0.0050935	0.0000000	0.0000000
ENSG00000289018	0.0037094	0.0045553	0.0018309	0.0063197	0.0052241
PTMA	3.1160738	3.2938754	3.4123359	2.9435633	2.9149969
PDE6D	0.3897756	0.4512863	0.5305378	0.4073408	0.3651415
COPS7B	0.2425869	0.2611901	0.2553443	0.2472422	0.1734063
ENSG00000261096	0.0057032	0.0010709	0.0000000	0.0042859	0.0000000
NPPC	0.0651915	0.1390540	0.1084055	0.0447828	0.1031712
DIS3L2	0.1610795	0.1840058	0.1529420	0.1304797	0.4088928
ENSG00000227033	0.0000000	0.0000000	0.0000000	0.0000000	0.0070221
ECEL1	0.1381461	0.3659827	0.2585109	0.1270231	0.2982254
PRSS56	0.0249103	0.0595166	0.0329788	0.0157236	0.0496875
CHRND	0.0023927	0.0023072	0.0000000	0.0000000	0.0000000
TIGD1	0.0770613	0.0813099	0.0944696	0.0805729	0.0552748
EIF4E2	0.5374160	0.5502002	0.4893659	0.4894354	0.4290621
ENSG00000237126	0.0020941	0.0044539	0.0082856	0.0000000	0.0059094
EFHD1	0.8334854	0.5028466	0.3204366	0.9281654	0.4030379
GIGYF2	0.5469702	0.5263312	0.5562870	0.4685940	0.7432668
ENSG00000241409	0.0013952	0.0000000	0.0000000	0.0000000	0.0071630
KCNJ13	0.0006968	0.0000000	0.0000000	0.0000000	0.0077406
SNORC	0.0359761	0.0644605	0.0854963	0.0297082	0.0757010
NGEF	0.0576220	0.1066162	0.1131789	0.0338421	0.0877265
INPP5D	0.0215684	0.0156590	0.0077886	0.0261814	0.0139184
ATG16L1	0.0941222	0.1122984	0.1471318	0.1005973	0.1915732
SAG	0.0000000	0.0000000	0.0000000	0.0038461	0.0000000
ENSG00000259793	0.0014201	0.0000000	0.0000000	0.0000000	0.0000000
DGKD	0.4946916	0.5121676	0.4810623	0.3768877	0.5219886
USP40	0.1591497	0.1133106	0.0667915	0.1885183	0.2256689
HJURP	0.0071167	0.0032494	0.0034419	0.0128050	0.0190653
TRPM8	0.0044488	0.0033127	0.0077397	0.0024483	0.0098027
SPP2	0.0000000	0.0011890	0.0000000	0.0000000	0.0000000
ENSG00000288040	0.0616581	0.0213756	0.0157477	0.0405273	0.0127293
ARL4C	0.7371981	1.1306200	1.2967105	0.6508153	0.9572937
LINC01173	0.0000000	0.0000000	0.0000000	0.0000000	0.0073529
ENSG00000235726	0.0037608	0.0024659	0.0082898	0.0035433	0.0076218
SH3BP4	0.0993214	0.0878639	0.0684582	0.1088199	0.1610209
ENSG00000226542	0.0000000	0.0000000	0.0000000	0.0000000	0.0044005
AGAP1	0.5184944	0.6689110	0.7572163	0.4817248	1.3731739
ENSG00000222007	0.0000000	0.0000000	0.0000000	0.0000000	0.0000000
GBX2	0.0006839	0.0186103	0.0399152	0.0086895	0.0079318
GBX2-AS1	0.0005637	0.0051991	0.0089780	0.0000000	0.0032078
ASB18	0.0012565	0.0046951	0.0067845	0.0000000	0.0000000
IQCA1	0.1516947	0.0587518	0.0642737	0.2034740	0.1539180
IQCA1-AS1	0.0011492	0.0024350	0.0000000	0.0000000	0.0000000
ACKR3	0.0014117	0.0058004	0.0065608	0.0074646	0.0099068
COPS8-DT	0.0324737	0.0364727	0.0308211	0.0200897	0.0492152
COPS8	0.9363902	0.8061360	0.8317319	1.0021630	0.6664625
COL6A3	0.0225285	0.0359618	0.0259010	0.0338202	0.0440931
MLPH	0.0048037	0.0023254	0.0023670	0.0000000	0.0000000
RAB17	0.0028248	0.0055417	0.0024103	0.0075945	0.0082850

LRRFIP1	0.9661893	0.7908130	0.6758559	1.0375568	0.7655759
ENSG00000286864	0.0000000	0.0000000	0.0000000	0.0000000	0.0000000
RBM44	0.0084243	0.0032767	0.0021512	0.0056995	0.0111778
RAMP1	0.2905482	0.2092880	0.1512004	0.4087737	0.1585689
ENSG00000288857	0.0008897	0.0000000	0.0011329	0.0000000	0.0062037
UBE2F	0.3147840	0.3976101	0.3731908	0.2947686	0.3881759
SCLY	0.0460644	0.0519919	0.0655450	0.0479349	0.0774194
ESPNL	0.0000000	0.0000000	0.0000000	0.0000000	0.0030476
KLHL30	0.0002567	0.0026804	0.0040934	0.0000000	0.0016281
ERFE	0.0107604	0.0075698	0.0117855	0.0000000	0.0045939
ILKAP	0.2775841	0.2561030	0.2675474	0.2465273	0.2437018
LINC02610	0.0817754	0.1005124	0.1553931	0.0646020	0.0943699
ENSG00000225057	0.0028246	0.0035365	0.0023230	0.0000000	0.0064993
HES6	0.5371541	0.3774705	0.3914379	0.5300511	0.2810875
PER2	0.1828861	0.1306700	0.1286692	0.1624149	0.1691996
ENSG00000283635	0.0048048	0.0015739	0.0071123	0.0091431	0.0000000
TRAF3IP1	0.4909443	0.4318928	0.4015454	0.4070468	0.4701078
ASB1	0.1278306	0.1454648	0.1156722	0.0982813	0.1532783
LINC01937	0.0023010	0.0061605	0.0009140	0.0231511	0.0014580
TWIST2	0.0056644	0.0045327	0.0022064	0.0045021	0.0148267
HDAC4	0.1159402	0.1273835	0.1091455	0.0857260	0.5353012
ENSG00000286307	0.0000000	0.0000000	0.0000000	0.0000000	0.0060477
HDAC4-AS1	0.0822337	0.0644655	0.0434323	0.0748245	0.0897270
ENSG00000286525	0.0003876	0.0028001	0.0000000	0.0000000	0.0000000
ENSG00000220256	0.0000000	0.0000000	0.0012385	0.0000000	0.0060718
NDUFA10	0.7345345	0.7505583	0.8087434	0.7125528	0.7040739
COPS9	1.2373647	1.2083624	1.1570640	1.2601888	0.9900708
OTOS	0.0000000	0.0010884	0.0024426	0.0000000	0.0000000
GPC1	0.1960836	0.1809831	0.1535976	0.2629978	0.3200791
GPC1-AS1	0.0150362	0.0213455	0.0098494	0.0085454	0.0085155
ANKMY1	0.0534605	0.0427271	0.0488450	0.0560908	0.0988505
DUSP28	0.1307287	0.1327005	0.1608701	0.1312229	0.1301327
RNPEPL1	0.0615902	0.0308326	0.0179448	0.0975953	0.0401586
CAPN10-DT	0.0052042	0.0053487	0.0106662	0.0030518	0.0000000
CAPN10	0.0505056	0.0443141	0.0810475	0.0612449	0.0583738
GPR35	0.0025831	0.0013157	0.0028035	0.0049051	0.0067404
KIF1A	0.9815083	1.2476478	1.4399625	0.9769764	1.2630391
CROCC2	0.0139650	0.0092029	0.0069791	0.0122877	0.0221337
SNED1	0.0081354	0.0207154	0.0257647	0.0044334	0.0961660
SNED1-AS1	0.0071345	0.0091247	0.0015113	0.0039751	0.0193611
MTERF4	0.1595145	0.1644324	0.1437244	0.1627868	0.2653575
ENSG00000289253	0.0000000	0.0000000	0.0000000	0.0000000	0.0000000
PASK	0.0395973	0.0340013	0.0287592	0.0379882	0.0674581
PPP1R7	0.4489382	0.4778703	0.5337667	0.4055723	0.4416778
ANO7	0.0107796	0.0122838	0.0091952	0.0074897	0.0074318
HDLBP	1.4843943	1.2173770	1.0301879	1.4274189	1.0177446
SEPTIN2	1.6116070	1.1311053	0.8947608	1.7081653	1.1473142
ENSG00000286588	0.0000000	0.0000000	0.0000000	0.0000000	0.0013481
FARP2	0.1079757	0.1150348	0.1016057	0.0912122	0.2094305
ENSG00000288080	0.0025364	0.0000000	0.0000000	0.0000000	0.0041587
STK25	0.3969092	0.4558828	0.5477393	0.4129108	0.3892206
ENSG00000289555	0.0135188	0.0077385	0.0074327	0.0080427	0.0068867
BOK-AS1	0.0043258	0.0024120	0.0018196	0.0024514	0.0104863

BOK	0.0394952	0.0406009	0.0688689	0.0182152	0.0462269
ENSG00000273113	0.0008547	0.0049152	0.0000000	0.0023617	0.0016619
THAP4	0.1739953	0.2150957	0.2581349	0.1988402	0.2126631
ATG4B	0.1833713	0.2168832	0.1950778	0.1731931	0.2440207
DTYMK	0.2233361	0.2560100	0.2531049	0.1982664	0.2286696
ING5	0.2571399	0.2806072	0.2781831	0.2278167	0.2668250
D2HGDH	0.0615851	0.0496533	0.0571062	0.0604112	0.0867131
GAL3ST2	0.0009392	0.0009612	0.0032859	0.0030842	0.0159497
NEU4	0.0010671	0.0000000	0.0018999	0.0000000	0.0000000
RTP5	0.0127010	0.0267047	0.0430315	0.0026482	0.0412827
LINC01237	0.0174002	0.0463468	0.0173036	0.0035499	0.1193860
FAM240C	0.0032111	0.0118779	0.0015898	0.0012213	0.0000000
LINC01238	0.0033987	0.0032413	0.0013013	0.0000000	0.0039405
LINC01880	0.0000000	0.0000000	0.0000000	0.0000000	0.0000000
LINC01238.1	0.0072448	0.0000000	0.0009597	0.0000000	0.0060801
ENSG00000291147	0.1175183	0.1762643	0.1687767	0.0928149	0.1756655
CHL1-AS2	0.1537895	0.1579423	0.2173283	0.1624847	0.1091933
CHL1	0.8505489	0.9568427	1.4524672	1.2259288	1.2633541
CHL1-AS1	0.0018773	0.0000000	0.0000000	0.0000000	0.0000000
LINC01266	0.0109087	0.0081309	0.0000000	0.0128332	0.0288613
CNTN6	0.0774522	0.0724384	0.0875719	0.0907584	0.2250419
ENSG00000288831	0.0772201	0.0689498	0.0649608	0.0570673	0.0501527
CNTN4	0.9896283	0.7439170	0.5908943	1.1380524	1.3234746
CNTN4-AS2	0.0026238	0.0025042	0.0000000	0.0000000	0.0331976
ENSG00000286363	0.0000000	0.0012644	0.0000000	0.0000000	0.0116883
CNTN4-AS1	0.0022943	0.0005310	0.0014297	0.0000000	0.0000000
IL5RA	0.0134142	0.0025448	0.0010884	0.0121155	0.0060044
TRNT1	0.2859715	0.3116082	0.3031455	0.2875473	0.2723343
CRBN	0.5953845	0.5408333	0.5147719	0.5819809	0.4370156
ENSG00000223727	0.0140529	0.0152848	0.0020714	0.0054154	0.0541715
SUMF1	0.2066810	0.1322712	0.0961894	0.2851137	0.4507437
LRRN1	0.1733457	0.2890275	0.3081687	0.2199623	0.5259983
ENSG00000287720	0.0228491	0.0588173	0.0697796	0.0380394	0.1183716
SETMAR	0.1186295	0.1152316	0.1078155	0.1024131	0.0813018
ITPR1-DT	0.0325924	0.0100222	0.0193840	0.0156055	0.0170207
ITPR1	0.0266213	0.0309807	0.0316931	0.0338621	0.0493577
EGOT	0.0000000	0.0000000	0.0000000	0.0030695	0.0031362
ENSG00000235978	0.0079127	0.0012888	0.0115829	0.0142234	0.0246627
BHLHE40-AS1	0.0360147	0.0082734	0.0033354	0.0149332	0.0103239
BHLHE40	0.3208700	0.2137475	0.1439580	0.3055531	0.2101672
ARL8B	0.6339462	0.6485038	0.6647432	0.6218410	0.5163772
ENSG00000233912	0.0228630	0.0172159	0.0101697	0.0318850	0.0281877
EDEM1	0.0964102	0.0805550	0.0427289	0.0750533	0.1016626
ENSG00000229642	0.0040106	0.0042247	0.0000000	0.0000000	0.0000000
ENSG00000226022	0.0690107	0.1191749	0.0860420	0.0557348	0.0745921
ENSG00000189229	0.0195381	0.0126251	0.0047262	0.0036005	0.0066258
GRM7-AS3	0.0039502	0.0100870	0.0000000	0.0058811	0.0069538
GRM7	0.2449643	0.3161963	0.3825826	0.1644982	1.2030291
ENSG00000288030	0.0000000	0.0019119	0.0000000	0.0000000	0.0091992
GRM7-AS1	0.0009658	0.0022088	0.0000000	0.0000000	0.0044881
ENSG00000270207	0.0128730	0.0135763	0.0077980	0.0099466	0.0968589
LMCD1-AS1	0.1596577	0.1706059	0.1149953	0.1495510	0.4418802
ENSG00000228351	0.0007458	0.0006000	0.0014585	0.0000000	0.0049701

ENSG00000231401	0.0009205	0.0037605	0.0000000	0.0000000	0.0114263
LMCD1	0.5036777	0.3020000	0.2077803	0.5489527	0.2859855
SSUH2	0.0052349	0.0030675	0.0000000	0.0038847	0.0792533
ENSG00000291007	0.0000000	0.0013426	0.0000000	0.0000000	0.0072563
CAV3	0.0004592	0.0000000	0.0030104	0.0000000	0.0102344
OXTR	0.0160825	0.0071935	0.0215776	0.0000000	0.0219224
RAD18	0.0882654	0.0771208	0.0721806	0.0944033	0.1516180
SRGAP3	1.0036309	0.9955470	0.9094772	1.0004211	1.3175558
ENSG00000287623	0.0020730	0.0000000	0.0000000	0.0000000	0.0000000
SRGAP3-AS2	0.0018464	0.0020791	0.0000000	0.0000000	0.0000000
SRGAP3-AS3	0.0000000	0.0000000	0.0000000	0.0000000	0.0000000
SRGAP3-AS4	0.0294141	0.0154707	0.0173902	0.0160776	0.0029782
ENSG00000254485	0.0089264	0.0028087	0.0000000	0.0037761	0.0000000
THUMPD3-AS1	0.2415705	0.2790109	0.2758159	0.2418856	0.2403042
THUMPD3	0.5297264	0.4437955	0.3752451	0.5955741	0.3064857
SETD5	0.6747493	0.6734020	0.6100203	0.6243121	0.9434173
LHFPL4	0.0887515	0.1691431	0.2169316	0.0755948	0.3092533
MTMR14	0.0622670	0.0598041	0.0728361	0.0459833	0.0787718
ENSG00000287878	0.0005892	0.0004495	0.0000000	0.0000000	0.0000000
CPNE9	0.0127123	0.0200107	0.0215142	0.0254720	0.0138566
BRPF1	0.0340324	0.0595169	0.0443515	0.0238078	0.0814886
OGG1	0.1427626	0.1388876	0.1729069	0.1894635	0.2245652
CAMK1	0.1936224	0.2302295	0.3090027	0.1821067	0.1152603
TADA3	0.5290839	0.5202909	0.5220245	0.5760803	0.4143565
ARPC4	0.4438954	0.5355111	0.5592796	0.4793574	0.3519458
TTLL3	0.0865459	0.0834153	0.0578193	0.0648078	0.1838081
ENSG00000269886	0.0083289	0.0139615	0.0151494	0.0000000	0.0072708
RPUSD3	0.2178519	0.2522073	0.3153490	0.1737325	0.1528909
JAGN1	0.2746334	0.2255792	0.2226101	0.3198215	0.1396640
IL17RE	0.0158342	0.0189998	0.0364251	0.0091418	0.0000000
IL17RC	0.0458355	0.0358886	0.0092312	0.0653073	0.0261366
CRELD1	0.2901194	0.2465045	0.2286971	0.4102758	0.2217428
PRRT3	0.0367739	0.0522545	0.0734087	0.0596101	0.0368429
PRRT3-AS1	0.0047995	0.0000000	0.0026409	0.0051684	0.0124387
ENSG00000269982	0.0036175	0.0020047	0.0013551	0.0000000	0.0000000
EMC3	0.5130176	0.4973411	0.4937596	0.5729370	0.4111581
ENSG00000291224	0.0220593	0.0415716	0.0304217	0.0414506	0.0268179
ENSG00000206567	0.0364227	0.0218035	0.0311107	0.0255026	0.0156032
ENSG00000291078	0.1197955	0.1525812	0.1776018	0.1338127	0.1230444
FANCD2	0.0166881	0.0136514	0.0040267	0.0136228	0.0282962
FANCD2OS	0.0006803	0.0000000	0.0000000	0.0000000	0.0035685
BRK1	1.3216438	1.3515247	1.3309914	1.2487446	1.0673781
VHL	0.4903869	0.5708152	0.6505164	0.4887598	0.5315315
ENSG00000287086	0.0035393	0.0006938	0.0028470	0.0000000	0.0017986
IRAK2	0.0194565	0.0291898	0.0285964	0.0135644	0.0511245
TATDN2	0.1479191	0.2121329	0.2532562	0.1085186	0.1549523
LINC00852	0.0000000	0.0000000	0.0029715	0.0020019	0.0000000
GHRL	0.0039605	0.0013354	0.0010537	0.0000000	0.0000000
GHRLOS	0.0044698	0.0011466	0.0000000	0.0000000	0.0012770
SEC13	0.6777444	0.5405460	0.5527283	0.6835491	0.5068204
ATP2B2	0.0993245	0.2505217	0.2391760	0.0775894	0.2943389
ATP2B2-IT2	0.0000000	0.0000000	0.0000000	0.0000000	0.0049854
SLC6A11	0.0444473	0.0644204	0.1096285	0.0451341	0.1533705

ENSG00000286962	0.0026340	0.0026933	0.0000000	0.0000000	0.0000000
SLC6A1	0.0690170	0.0771911	0.1236819	0.0620193	0.1279163
SLC6A1-AS1	0.0008285	0.0000000	0.0000000	0.0000000	0.0038318
ENSG00000287964	0.0000000	0.0000000	0.0000000	0.0000000	0.0000000
HRH1	0.0074446	0.0122977	0.0059833	0.0119693	0.0163944
ENSG00000285906	0.0000000	0.0000000	0.0024972	0.0000000	0.0000000
ATG7	0.0868271	0.0813117	0.0929034	0.1254411	0.3577606
VGLL4	0.6570942	0.6484883	0.5114038	0.7440224	0.6757678
ENSG00000272483	0.0002507	0.0036273	0.0134247	0.0000000	0.0155969
TAMM41	0.1337773	0.1124261	0.1212115	0.0954260	0.1794028
SYN2	0.1638673	0.3078741	0.4370606	0.1678447	0.5625883
TIMP4	0.1032558	0.0478466	0.0265372	0.2542867	0.0655864
ENSG00000288952	0.0023083	0.0037979	0.0000000	0.0000000	0.0183207
PPARG	0.0314177	0.0291989	0.0229523	0.0194693	0.0424892
TSEN2	0.0960831	0.0720424	0.0649527	0.0822789	0.1880484
MKRN2OS	0.0062693	0.0009368	0.0000000	0.0000000	0.0172626
MKRN2	0.3001894	0.2746383	0.2341283	0.2835002	0.2255268
RAF1	0.2967046	0.3104008	0.2857827	0.2981622	0.4895464
ENSG00000290072	0.0038641	0.0010592	0.0104442	0.0063266	0.0093792
TMEM40	0.0015991	0.0000000	0.0000000	0.0000000	0.0000000
CAND2	0.0447941	0.0499720	0.0368114	0.0423873	0.0610094
ENSG00000272263	0.0023032	0.0000000	0.0000000	0.0000000	0.0000000
RPL32	3.1565919	3.2062621	3.0180529	3.0198120	2.9443165
LINC02022	0.0020609	0.0000000	0.0000000	0.0029316	0.0134526
IQSEC1	0.2766322	0.3373069	0.2902348	0.2412934	0.6716818
NUP210	0.0465753	0.0481048	0.0543543	0.0400831	0.1483745
HDAC11-AS1	0.0034489	0.0000000	0.0006749	0.0000000	0.0177692
HDAC11	0.1374983	0.1254762	0.1126677	0.1361627	0.1052460
FBLN2	0.0056110	0.0068204	0.0088402	0.0000000	0.0176175
LINC00620	0.0158162	0.0254501	0.0098221	0.0490951	0.0146927
WNT7A	0.1164256	0.0563532	0.1083840	0.1635901	0.0763646
FGD5P1	0.0000000	0.0000000	0.0000000	0.0000000	0.0000000
CHCHD4	0.1757138	0.1784568	0.2001964	0.1728525	0.1373612
TMEM43	0.3355915	0.2144794	0.1675996	0.5312543	0.3130478
ENSG00000290044	0.0016640	0.0000000	0.0000000	0.0000000	0.0000000
XPC-AS1	0.0029070	0.0025405	0.0073309	0.0000000	0.0236667
XPC	0.4455127	0.3091866	0.2121937	0.4314803	0.3629263
LSM3	0.6938373	0.7407028	0.6946995	0.6525281	0.5004713
SLC6A6	0.0267064	0.0418167	0.0556153	0.0362532	0.1101401
GRIP2	0.0282350	0.0445629	0.0447220	0.0122176	0.1322511
ENSG00000283392	0.0000000	0.0000000	0.0000000	0.0000000	0.0000000
CCDC174	0.3819234	0.2956662	0.2739337	0.3304781	0.2340899
C3orf20	0.0383507	0.0428865	0.0408554	0.0301722	0.0514433
ENSG00000285969	0.0000000	0.0044946	0.0078155	0.0000000	0.0000000
FGD5	0.0132333	0.0380497	0.0571319	0.0023709	0.0441610
FGD5-AS1	1.2043395	1.0954712	1.0329836	1.0885239	0.9474536
NR2C2	0.2303486	0.2576141	0.2911897	0.1968360	0.3690442
MRPS25	0.2784391	0.3558968	0.3215039	0.3199120	0.3698913
RBSN	0.2713898	0.2378634	0.2389533	0.2513613	0.2414600
CAPN7	0.5216323	0.4377338	0.4634564	0.4863989	0.4942504
SH3BP5-AS1	0.0152252	0.0169810	0.0201188	0.0024376	0.0323502
SH3BP5	0.3405369	0.6478182	0.8448202	0.2845488	0.6077996
METTL6	0.2469568	0.3630834	0.3231824	0.2491874	0.4727599

EAF1	0.2098039	0.2172476	0.2755075	0.2384481	0.1919878
EAF1-AS1	0.0104459	0.0062872	0.0094029	0.0062391	0.0500279
COLQ	0.0917096	0.0705737	0.0637843	0.0942252	0.1301810
HACL1	0.1734814	0.1201867	0.1308563	0.2004579	0.2339499
BTD	0.1515778	0.1374077	0.0871903	0.2561807	0.1652784
ANKRD28	0.3916896	0.3585638	0.2443064	0.4224676	0.6859491
ENSG00000287042	0.1572981	0.1809047	0.1308572	0.1198989	0.4390927
ENSG00000286689	0.0010816	0.0061322	0.0036559	0.0052622	0.0000000
GALNT15	0.2647759	0.1365865	0.1188995	0.4770346	0.1384488
DPH3	0.5255520	0.6086220	0.5982328	0.4849404	0.4647946
OXNAD1	0.1157809	0.1746030	0.1246402	0.1051858	0.1802830
RFTN1	0.1395018	0.1368935	0.1700095	0.1195857	0.1716426
ENSG00000271964	0.0032820	0.0014079	0.0085000	0.0000000	0.0000000
ENSG00000272498	0.0000000	0.0034322	0.0000000	0.0000000	0.0000000
ENSG00000272529	0.0000000	0.0000000	0.0000000	0.0000000	0.0000000
ENSG00000287377	0.0027243	0.0023862	0.0000000	0.0000000	0.0000000
PLCL2	0.1261990	0.2240072	0.2464808	0.1604133	0.4056365
ENSG00000285731	0.0014892	0.0007580	0.0013773	0.0011568	0.0095520
TBC1D5	0.5834256	0.5411744	0.4631464	0.5595210	0.8853802
BALR6	0.0002567	0.0007005	0.0000000	0.0000000	0.0133361
SATB1	0.2142238	0.1964813	0.1172426	0.1794232	0.1386159
ENSG00000272477	0.0000000	0.0000000	0.0000000	0.0000000	0.0022383
SATB1-AS1	0.0137630	0.0186312	0.0111794	0.0156346	0.0166825
KCNH8	0.0699894	0.0928652	0.1013244	0.0848253	0.5587825
ENSG00000287069	0.0004576	0.0011149	0.0000000	0.0000000	0.0138554
EFHB	0.0282849	0.0191752	0.0093132	0.0408363	0.0088337
RAB5A	0.8303937	0.7338777	0.7186334	0.9330745	0.6776019
ENSG00000289138	0.0289514	0.0405131	0.0212828	0.0088066	0.0327507
PP2D1	0.0018971	0.0051256	0.0000000	0.0018125	0.0080828
KAT2B	0.2099744	0.1347410	0.0834715	0.2130676	0.1889641
SGO1	0.0073857	0.0081637	0.0058408	0.0205394	0.0191124
SGO1-AS1	0.0353775	0.0224882	0.0087581	0.0195475	0.0449214
ENSG00000282987	0.0011347	0.0000000	0.0000000	0.0040556	0.0000000
ZNF385D	0.2456590	0.4336747	0.4163545	0.2056993	0.9538766
ZNF385D-AS1	0.0000000	0.0000000	0.0000000	0.0000000	0.0000000
ZNF385D-AS2	0.0000000	0.0010918	0.0011169	0.0000000	0.0086659
UBE2E2-DT	0.0017016	0.0014253	0.0000000	0.0039294	0.0000000
UBE2E2	0.4675194	0.5880350	0.5448525	0.4092065	1.1469422
UBE2E1-AS1	0.0099656	0.0099353	0.0070149	0.0061280	0.0042436
UBE2E1	0.7643640	0.6304358	0.5794566	0.7416989	0.6975128
NKIRAS1	0.4012079	0.4093888	0.4895430	0.3679528	0.3650868
RPL15	2.9379780	2.9329843	2.8423054	2.8723213	2.6285978
NR1D2	0.3153600	0.2481874	0.1901140	0.3018824	0.2697986
LINC00691	0.0002173	0.0000000	0.0000000	0.0000000	0.0000000
THRB	0.0217524	0.0325410	0.0096437	0.0151574	0.1424055
ENSG00000289130	0.0036059	0.0023461	0.0000000	0.0025857	0.0186562
THRB-AS1	0.0000000	0.0023094	0.0000000	0.0045161	0.0000000
ENSG00000272554	0.0004942	0.0010952	0.0000000	0.0031257	0.0000000
RARB	0.2014244	0.4053010	0.2010502	0.1291370	0.6727697
TOP2B	0.9331302	0.9731082	1.1615298	0.8901447	1.0300238
NGLY1	0.3174185	0.3163547	0.2978827	0.2286781	0.3683834
OXSM	0.1487718	0.1116106	0.0761034	0.1086665	0.1081247
LRRC3B-AS1	0.0035763	0.0028363	0.0000000	0.0000000	0.0000000

LRRC3B	0.2169397	0.1793012	0.1965555	0.1711156	0.2065935
NEK10	0.1265207	0.0890583	0.0642660	0.1002986	0.3119513
ENSG00000287348	0.0028262	0.0025434	0.0019328	0.0000000	0.0095529
SLC4A7	0.4434152	0.5771793	0.5515266	0.3681046	0.6461264
LINC01980	0.0383342	0.0316197	0.0354924	0.0430160	0.0511505
LINC01967	0.0042431	0.0103613	0.0029930	0.0000000	0.0169579
CMC1	0.3541806	0.3181775	0.3259388	0.3317156	0.3200548
AZI2	0.6729820	0.6673778	0.6792302	0.5906164	0.5530821
ZCWPW2	0.0672871	0.0309618	0.0225611	0.0487767	0.1031369
RBMS3	0.4196995	0.6215360	0.3941868	0.3701311	1.2492588
RBMS3-AS3	0.0082084	0.0129429	0.0019881	0.0132768	0.0298727
RBMS3-AS2	0.0008486	0.0049091	0.0000000	0.0000000	0.0195595
RBMS3-AS1	0.0000000	0.0000000	0.0000000	0.0000000	0.0028743
LINC01985	0.0029331	0.0046210	0.0019631	0.0022544	0.0000000
TGFBR2	0.0775613	0.0510738	0.0199160	0.0866999	0.0439042
GADL1	0.0000000	0.0000000	0.0000000	0.0000000	0.0031362
ENSG00000288926	0.0104672	0.0004904	0.0061374	0.0174768	0.0138219
STT3B	0.4983945	0.4326514	0.4283260	0.6393753	0.5662939
OSBPL10	0.0751719	0.0865649	0.0596681	0.0641473	0.1358039
OSBPL10-AS1	0.0006474	0.0000000	0.0000000	0.0000000	0.0071095
ZNF860	0.0077190	0.0082026	0.0078280	0.0022483	0.0101239
GPD1L	0.0926357	0.0947223	0.0729146	0.0923870	0.0691948
ENSG00000261572	0.0824072	0.0314235	0.0224425	0.0322473	0.0179394
CMTM8	1.5872866	1.0203803	0.7226183	1.3986238	1.0395376
CMTM7	0.2840274	0.1650069	0.1145905	0.2439135	0.1828887
CMTM6	0.4376753	0.2803044	0.2517278	0.5879022	0.2231460
DYNC1LI1	0.7480418	0.9034040	1.0332373	0.7222981	0.7623415
CNOT10	0.2084292	0.1916725	0.1674979	0.1927000	0.3111680
CNOT10-AS1	0.0000000	0.0000000	0.0000000	0.0000000	0.0038113
TRIM71	0.0286136	0.0660606	0.0490953	0.0180062	0.1125286
GLB1	0.3115597	0.2395334	0.2121219	0.5416753	0.2674620
TMPPE	0.0017159	0.0036067	0.0029379	0.0063060	0.0000000
CRTAP	0.3705733	0.2810593	0.2244939	0.3273607	0.2195252
SUSD5	0.0323281	0.0345676	0.0231475	0.0404703	0.0650755
ENSG00000289460	0.0128182	0.0235464	0.0249911	0.0234972	0.0281165
FBXL2	0.3063071	0.4493718	0.5074646	0.3663536	0.6207015
UBP1	0.1555704	0.1879627	0.1560576	0.1412953	0.2788996
CLASP2	1.0028435	1.3449571	1.3744145	0.9974523	1.5736131
PDCD6IP-DT	0.0437164	0.0341273	0.0273452	0.0608407	0.0421753
PDCD6IP	0.4943655	0.4324543	0.2886114	0.4666177	0.4485135
LINC01811	0.0011474	0.0008736	0.0051692	0.0024504	0.0070649
ARPP21	0.1818887	0.2626989	0.3487262	0.1413685	0.4446593
STAC	0.0645158	0.1193742	0.2119652	0.0406706	0.2395093
DCLK3	0.0199117	0.0289502	0.0717289	0.0151438	0.0394149
LINC02033	0.0004182	0.0018748	0.0000000	0.0000000	0.0000000
TRANK1	0.0915787	0.0729715	0.1144843	0.0724624	0.2097341
ENSG00000272334	0.0000000	0.0013374	0.0023415	0.0000000	0.0040955
EPM2AIP1	0.4034761	0.4762670	0.5552165	0.3717211	0.5376160
MLH1	0.1845459	0.1742718	0.1745867	0.1545830	0.1768456
LRRFIP2	0.5628652	0.4717075	0.4129962	0.5294991	0.5746157
ENSG00000290046	0.1162483	0.0613674	0.0488529	0.0687565	0.1351146
ENSG00000271993	0.0000000	0.0000000	0.0000000	0.0000000	0.0043359
ENSG00000271653	0.0000000	0.0000000	0.0000000	0.0000000	0.0033235

GOLGA4-AS1	0.0185543	0.0041951	0.0064788	0.0117443	0.0151512
GOLGA4	0.9722142	0.8651409	0.7852684	0.9216598	0.9501109
APRG1	0.0168519	0.0247629	0.0127428	0.0108423	0.0763115
ITGA9	0.0472517	0.0474981	0.0340460	0.0354768	0.1186315
ITGA9-AS1	0.1125042	0.1691947	0.1817470	0.0969125	0.2265044
ENSG00000288972	0.0105487	0.0038970	0.0250894	0.0084632	0.0077833
CTDSPL	0.0767059	0.1067322	0.0756217	0.0406672	0.2643576
VILL	0.0156047	0.0140031	0.0123364	0.0125680	0.0188702
PLCD1	0.1424857	0.0889935	0.0449728	0.1399522	0.0923094
DLEC1	0.0222073	0.0115210	0.0000000	0.0495710	0.0153415
ACAA1	0.2442406	0.1752865	0.1620358	0.3023779	0.1252877
MYD88	0.0261874	0.0401390	0.0601845	0.0306270	0.0166731
OXSR1	0.3795961	0.3509364	0.3926668	0.3006422	0.4779858
SLC22A13	0.0002570	0.0000000	0.0000000	0.0000000	0.0025607
SLC22A14	0.0056847	0.0058254	0.0000000	0.0000000	0.0117900
XYLB	0.0198046	0.0305012	0.0289381	0.0103119	0.0873779
ACVR2B-AS1	0.0036218	0.0034716	0.0041977	0.0033608	0.0000000
ACVR2B	0.3672900	0.5292779	0.5870770	0.3064957	0.5644351
EXOG	0.0796965	0.1202770	0.1260442	0.1045012	0.1761605
SCN5A	0.0063718	0.0062571	0.0049956	0.0029331	0.0049840
SCN11A	0.0245466	0.0176303	0.0154562	0.0126716	0.0716579
WDR48	0.5478645	0.4808012	0.4540292	0.5471256	0.5070530
GORASP1	0.0873029	0.0852581	0.0912064	0.0824478	0.0868563
TTC21A	0.0234240	0.0396702	0.0351074	0.0117016	0.1151680
CSRNP1	0.0449919	0.0300630	0.0167180	0.0798166	0.0484638
ENSG00000284669	0.0036932	0.0033026	0.0000000	0.0010648	0.0000000
ENSG00000287620	0.0215649	0.0239553	0.0109477	0.0204662	0.0216872
ENSG00000287780	0.0130518	0.0066017	0.0120142	0.0175854	0.0165387
SLC25A38	0.2307386	0.1964468	0.1249301	0.2745905	0.1700905
RPSA	2.8012408	2.7895240	2.6119382	2.6932972	2.4980624
ENSG00000286781	0.0022339	0.0010809	0.0025718	0.0000000	0.0039123
MOBP	0.0045512	0.0036381	0.0030514	0.0000000	0.0000000
MYRIP	0.1789585	0.1911540	0.2556531	0.1437042	0.4990402
EIF1B-AS1	0.0582772	0.0436218	0.0258930	0.0703833	0.1181643
EIF1B	1.5447386	1.5530194	1.6471675	1.4050372	1.2496596
ENTPD3-AS1	0.1000319	0.0985886	0.0754607	0.1002596	0.0987508
ENTPD3	0.0271708	0.0512653	0.0360932	0.0818253	0.0768990
RPL14	2.7369911	2.8512788	2.7236200	2.6410416	2.5053944
ZNF619	0.0159027	0.0197682	0.0100382	0.0197687	0.0000000
ZNF620	0.1134469	0.1566260	0.1713293	0.0514130	0.1155594
ZNF621	0.1309292	0.0919328	0.0590829	0.0903476	0.1629802
ENSG00000231873	0.0005298	0.0011923	0.0000000	0.0000000	0.0000000
CTNNB1	1.0827789	0.8876728	0.7624659	1.0945487	0.9557034
ULK4	0.2027003	0.1604996	0.0604822	0.1853634	0.3383557
TRAK1	0.3309064	0.3040633	0.2789604	0.3008366	0.4045582
CCK	0.0239130	0.0566570	0.1237506	0.0348641	0.0404975
VIPR1	0.0022419	0.0048481	0.0026613	0.0025427	0.0033583
VIPR1-AS1	0.0012537	0.0027703	0.0000000	0.0000000	0.0111868
SEC22C	0.2793921	0.2727424	0.2264487	0.2778579	0.2489555
SS18L2	0.2129188	0.2926966	0.3339805	0.2309994	0.2266961
ENSG00000289558	0.0025309	0.0000000	0.0000000	0.0047682	0.0034730
NKTR	0.4108936	0.4145700	0.3357301	0.3285151	0.8839386
ZBTB47-AS1	0.0590843	0.0526880	0.0202974	0.0528665	0.0580681

ZBTB47	0.1140990	0.1231563	0.0846261	0.0842211	0.1248637
ENSG00000287629	0.0007858	0.0047796	0.0015646	0.0000000	0.0034327
HHATL	0.0006457	0.0000000	0.0015634	0.0000000	0.0000000
CCDC13	0.1064638	0.0566050	0.0604966	0.1236107	0.0725147
CCDC13-AS1	0.0004576	0.0032272	0.0000000	0.0082825	0.0077598
CCDC13-AS2	0.0006301	0.0018080	0.0000000	0.0024545	0.0031362
HIGD1A	0.8784217	0.6950600	0.6693438	0.9558531	0.5822457
ENSG00000235288	0.0021595	0.0082279	0.0000000	0.0052084	0.0105449
ACKR2	0.0044486	0.0120485	0.0105542	0.0056543	0.0095383
ENSG00000273328	0.0000000	0.0009847	0.0000000	0.0059937	0.0000000
ZNF662	0.0082455	0.0124806	0.0181724	0.0103697	0.0220749
GASK1A	0.0150796	0.0086045	0.0099948	0.0182036	0.0105101
POMGNT2	0.3397448	0.2376584	0.2260533	0.3700059	0.1985622
SNRK	0.1261304	0.1831355	0.1888162	0.1167919	0.1832726
SNRK-AS1	0.0028102	0.0104167	0.0000000	0.0000000	0.0299975
ANO10	0.2559993	0.2808650	0.2137617	0.2890830	0.5827231
ABHD5	0.2795317	0.2301061	0.2105381	0.2626433	0.1893977
ENSG00000271192	0.0029298	0.0000000	0.0009816	0.0027921	0.0000000
ENSG00000272121	0.0117081	0.0408658	0.0563807	0.0245596	0.0349987
ENSG00000261786	0.0089275	0.0212591	0.0137340	0.0115987	0.0338262
ENSG00000271937	0.0020748	0.0000000	0.0017253	0.0010066	0.0000000
TCAIM	0.3355218	0.2993234	0.3057573	0.3164964	0.3794154
LINC01988	0.0000000	0.0000000	0.0000000	0.0000000	0.0000000
ZNF445	0.2608111	0.3457493	0.3383644	0.2834652	0.3511945
ZNF852	0.0465273	0.0639879	0.0511168	0.0410903	0.0797552
ZKSCAN7	0.0401619	0.0388910	0.0546675	0.0523983	0.0558118
ZKSCAN7-AS1	0.0547405	0.0702705	0.0650839	0.0557053	0.1519564
ZNF660	0.1018098	0.0977970	0.0888998	0.0804512	0.1018111
ZNF197	0.2687625	0.3234731	0.3308497	0.2128633	0.2930374
ZNF197-AS1	0.0000000	0.0025426	0.0024957	0.0000000	0.0000000
ZNF35	0.0667269	0.0861776	0.1089957	0.0761281	0.1050399
ENSG00000272077	0.0038754	0.0121625	0.0129937	0.0091575	0.0000000
ZNF502	0.0530586	0.0447835	0.0333002	0.0476280	0.0661872
ZNF501	0.0520285	0.0533806	0.0689049	0.0881558	0.0520115
KIAA1143	0.4911506	0.5100804	0.4589975	0.4561943	0.3771681
KIF15	0.0167940	0.0310951	0.0144777	0.0341352	0.0374819
TMEM42	0.3473744	0.3336530	0.3655955	0.3203357	0.2458916
TGM4	0.0012258	0.0000000	0.0000000	0.0000000	0.0000000
ZDHHC3	0.2891981	0.3466141	0.3417470	0.2962027	0.3033897
EXOSC7	0.3642425	0.3155595	0.3585588	0.3361150	0.3425036
CLEC3B	0.0052260	0.0000000	0.0000000	0.0000000	0.0000000
CDCP1	0.0051915	0.0014812	0.0000000	0.0016163	0.0000000
TMEM158	0.6527297	0.4960278	0.4700623	0.6448110	0.5172320
LARS2	0.0860572	0.0888788	0.1142976	0.1226283	0.1779711
LARS2-AS1	0.0000000	0.0000000	0.0000000	0.0000000	0.0111101
LIMD1	0.0614394	0.0340077	0.0396595	0.0670340	0.0864156
LIMD1-AS1	0.0233849	0.0213789	0.0388835	0.0329365	0.0260967
SACM1L	0.2282970	0.2100006	0.2059382	0.2759571	0.3193376
SLC6A20	0.0003288	0.0021380	0.0000000	0.0000000	0.0000000
ENSG00000288720	0.0013904	0.0000000	0.0000000	0.0000000	0.0084149
LZTFL1	0.5542655	0.3572170	0.2537287	0.5359854	0.3256391
CCR9	0.0007657	0.0000000	0.0000000	0.0000000	0.0000000
FYCO1	0.0888781	0.0742052	0.0497610	0.0844159	0.0551763

ENSG00000288717	0.0040216	0.0020692	0.0023362	0.0020601	0.0000000
ENSG00000288703	0.0000000	0.0000000	0.0000000	0.0000000	0.0000000
CCR3	0.0019173	0.0000000	0.0000000	0.0000000	0.0059661
LRRC2	0.3345577	0.1765077	0.0690752	0.2375858	0.1119096
ENSG00000286570	0.0008350	0.0000000	0.0015036	0.0000000	0.0077829
ALS2CL	0.0312095	0.0158668	0.0050942	0.0316976	0.0369270
TMIE	0.3689849	0.2267609	0.1632455	0.3584629	0.1678334
MYL3	0.0192471	0.0157240	0.0000000	0.0068902	0.0133265
PTH1R	0.0104138	0.0211169	0.0034684	0.0059211	0.0149305
CCDC12	0.3629647	0.3311766	0.3581953	0.4069780	0.3442728
NBEAL2	0.0053549	0.0100108	0.0042930	0.0101406	0.0640241
SETD2	0.6821114	0.7570626	0.8253317	0.6622787	0.8511784
KIF9-AS1	0.1272651	0.1144239	0.0893297	0.1030858	0.3005812
KIF9	0.6739332	0.5097114	0.3938216	0.7596936	0.4715434
KLHL18	0.1301378	0.1970206	0.1515852	0.1354169	0.2917496
ENSG00000289507	0.0126731	0.0112733	0.0020452	0.0000000	0.0000000
PTPN23-DT	0.0115680	0.0041316	0.0107296	0.0029257	0.0226648
PTPN23	0.0805800	0.0764607	0.0610715	0.1031108	0.1189111
SCAP	0.1613027	0.1573434	0.1407154	0.2002049	0.2775389
ELP6	0.2066762	0.2151879	0.2038661	0.2198017	0.1932631
CSPG5	0.5471289	0.4457690	0.5424770	0.8266661	0.4829630
SMARCC1	0.8449148	0.7216083	0.5783177	0.7403844	1.0115285
ENSG00000214773	0.0007321	0.0004301	0.0014605	0.0024744	0.0000000
DHX30	0.4150160	0.4251088	0.4077422	0.4187263	0.4162105
MAP4	1.5197379	1.7800252	1.7955190	1.4258633	1.4593863
CDC25A	0.0532692	0.1026129	0.1373456	0.0530794	0.0625345
ZNF589	0.0716743	0.0939497	0.0595124	0.0583286	0.1627729
NME6	0.2282840	0.1809682	0.2010457	0.2997453	0.1921975
ENSG00000289043	0.0003347	0.0131532	0.0008344	0.0000000	0.0031282
SPINK8	0.0004811	0.0000000	0.0000000	0.0000000	0.0051457
FBXW12	0.0038441	0.0054973	0.0020267	0.0049838	0.0139942
PLXNB1	0.0666467	0.0654215	0.0391466	0.1441042	0.1008703
CCDC51	0.0849612	0.1067442	0.1128225	0.0980656	0.0593906
TMA7	1.7392183	1.7501171	1.7004252	1.7165897	1.4414504
ENSG00000244380	0.0018510	0.0015719	0.0030686	0.0000000	0.0015298
ATRIP	0.0537509	0.0420526	0.0409674	0.0559436	0.0831856
SHISA5	0.6663819	0.6230662	0.6832679	0.6842745	0.5455053
PFKFB4	0.1284827	0.1414689	0.1364445	0.1010662	0.3829039
COL7A1	0.0265070	0.0335546	0.0329454	0.0264164	0.1534318
UQCRC1	0.5585136	0.5587236	0.5558059	0.5388753	0.3560524
SLC26A6	0.0067377	0.0116566	0.0013305	0.0036710	0.0026801
CELSR3	0.0200373	0.0391071	0.0499536	0.0090200	0.0697478
LINC02585	0.0087803	0.0000000	0.0090863	0.0000000	0.0101949
ENSG00000271973	0.0000000	0.0013201	0.0000000	0.0000000	0.0000000
NCKIPSD	0.0480478	0.0400660	0.0405741	0.0444310	0.0554896
IP6K2	0.5940317	0.6629406	0.7665048	0.5654553	0.5528997
PRKAR2A	0.4764919	0.6187293	0.7264093	0.4148952	0.6907059
PRKAR2A-AS1	0.0072473	0.0000000	0.0023810	0.0035629	0.0039851
SLC25A20	0.0948321	0.0812903	0.0595287	0.1103143	0.0405118
ARIH2OS	0.0446937	0.0343200	0.0141107	0.0377050	0.0133712
ARIH2	0.3685306	0.3835079	0.3831855	0.3256070	0.3890447
ENSG00000235236	0.0057980	0.0031783	0.0090150	0.0086490	0.0026851
ENSG00000223343	0.0202721	0.0059610	0.0216654	0.0036117	0.0125416

ENSG00000273211	0.0177020	0.0155706	0.0128325	0.0104010	0.0000000
P4HTM	0.5178217	0.3747691	0.4530428	0.7616535	0.3494661
WDR6	0.2033433	0.2059641	0.1685176	0.2360047	0.2277577
DALRD3	0.4597078	0.3541802	0.3374590	0.5015167	0.3020837
NDUFAF3	0.7760680	0.6556295	0.6289408	0.7383667	0.5847038
IMPDH2	1.0444481	0.8422599	0.7886003	0.9137901	0.6832199
ENSG00000272434	0.0000000	0.0006848	0.0000000	0.0073611	0.0000000
QRICH1	0.2256761	0.2062524	0.1893669	0.2001367	0.2600358
QARS1	0.2433190	0.2140429	0.1922211	0.2464691	0.1902112
USP19	0.0652443	0.0744364	0.0727048	0.1007431	0.1118266
LAMB2	0.1024176	0.0652720	0.0488157	0.2113763	0.0478675
CCDC71	0.0620683	0.0449360	0.0604776	0.0685964	0.0727034
KLHDC8B	0.4468311	0.2873257	0.2420009	0.4077171	0.2065005
C3orf84	0.0012073	0.0000000	0.0000000	0.0000000	0.0000000
IHO1	0.0021109	0.0013468	0.0000000	0.0000000	0.0080109
ENSG00000225399	0.0012726	0.0014137	0.0000000	0.0000000	0.0018039
C3orf62	0.0305424	0.0544377	0.0378746	0.0022345	0.0368435
USP4	0.2703108	0.2199461	0.2321434	0.2557455	0.3408275
GPX1	1.1332829	1.1098683	1.1239617	1.2248369	0.8024453
RHOA	1.7108152	1.3623026	1.2155210	1.6985707	1.1677450
TCTA	0.1844433	0.1781338	0.1198219	0.1690919	0.1042925
AMT	0.0426777	0.0405814	0.0241556	0.0545818	0.0306803
NICN1	0.0933526	0.1455227	0.1428921	0.1281754	0.0701129
DAG1	0.2984332	0.1918517	0.1283010	0.5111563	0.2222867
BSN-DT	0.0173985	0.0060337	0.0097551	0.0059284	0.0104584
BSN	0.0709001	0.1365158	0.1483391	0.0397454	0.2454862
APEH	0.1931394	0.1548721	0.1671554	0.2128616	0.1062704
MST1	0.0338178	0.0297564	0.0375205	0.0575939	0.0361230
RNF123	0.0461640	0.0470572	0.0378327	0.0250079	0.0627830
GMPPB	0.0625387	0.0520528	0.0629492	0.0542737	0.0551832
IP6K1	0.1543288	0.1436313	0.1373402	0.0936959	0.2591265
CDHR4	0.0014840	0.0016970	0.0031210	0.0000000	0.0085325
INKA1	0.0119708	0.0265857	0.0259314	0.0130919	0.0173012
UBA7	0.0040553	0.0000000	0.0077950	0.0000000	0.0000000
TRAIP	0.0062015	0.0020821	0.0030991	0.0146271	0.0161892
CAMKV	0.0754583	0.1266088	0.1996031	0.0577113	0.1205883
MST1R	0.0000000	0.0000000	0.0000000	0.0000000	0.0000000
MON1A	0.0545819	0.0513895	0.0642859	0.0610209	0.0262102
RBM6	0.4414146	0.4117119	0.4230814	0.3409034	0.7836910
RBM5	0.3700783	0.3026736	0.3015474	0.3831974	0.4947980
RBM5-AS1	0.0014517	0.0008781	0.0015844	0.0000000	0.0105914
SEMA3F-AS1	0.0000000	0.0020582	0.0000000	0.0027880	0.0069749
SEMA3F	0.0102011	0.0177606	0.0069767	0.0000000	0.0464731
GNAT1	0.0010208	0.0000000	0.0000000	0.0000000	0.0018081
SLC38A3	0.0574094	0.0393412	0.0235204	0.0590875	0.0104335
GNAI2	1.0451174	0.7696257	0.6751203	1.1468215	0.7139300
ENSG00000213600	0.0000000	0.0000000	0.0000000	0.0000000	0.0277717
ENSG00000230454	0.0014064	0.0079537	0.0024253	0.0000000	0.0083349
SEMA3B-AS1	0.0053646	0.0027801	0.0000000	0.0044812	0.0000000
SEMA3B	0.0309610	0.0133819	0.0137664	0.0319839	0.0229398
LSMEM2	0.0018892	0.0007182	0.0000000	0.0017625	0.0120949
IFRD2	0.1234388	0.0867854	0.0848632	0.1552095	0.1173952
HYAL3	0.0218770	0.0394866	0.0409260	0.0178973	0.0259624

NAA80	0.0585848	0.0480963	0.0578198	0.0260822	0.0748759
HYAL1	0.0000000	0.0019751	0.0000000	0.0029052	0.0250462
HYAL2	0.3629704	0.2310657	0.2066379	0.5152704	0.1915427
TUSC2	0.3319002	0.4300397	0.4491552	0.3005570	0.3038555
RASSF1	0.1808737	0.1367149	0.1138718	0.1921929	0.1436208
RASSF1-AS1	0.0103099	0.0103666	0.0059237	0.0143106	0.0257025
ZMYND10	0.4526031	0.2579475	0.1377510	0.5733244	0.1860910
NPRL2	0.1535004	0.1555522	0.1903756	0.1422700	0.0891291
CYB561D2	0.1082148	0.0678574	0.0714178	0.1454496	0.1047640
TMEM115	0.1366421	0.0952305	0.0709235	0.1583466	0.0563688
CACNA2D2	0.1080867	0.1461108	0.1953260	0.0976993	0.2356561
ENSG00000271858	0.0176345	0.0290855	0.0160560	0.0244571	0.0084933
C3orf18	0.1019372	0.1077356	0.0648795	0.0821871	0.0585953
HEMK1	0.0901213	0.0737658	0.0649812	0.0567545	0.0878120
CISH	0.0109781	0.0087239	0.0067146	0.0257422	0.0088133
MAPKAPK3	0.1019115	0.0618126	0.0358248	0.0839407	0.0427611
ENSG00000289504	0.0007636	0.0041164	0.0021739	0.0019820	0.0000000
LINC02019	0.0105486	0.0044001	0.0085942	0.0023684	0.0094505
DOCK3	0.4334547	0.5362776	0.4944799	0.3826806	1.4006009
ENSG00000288988	0.0080633	0.0077258	0.0047463	0.0117651	0.0076491
MANF	0.4573837	0.2993967	0.3109579	0.5162725	0.2813825
RBM15B	0.2583787	0.2809719	0.3178567	0.2484744	0.2064898
DCAF1	0.1146076	0.1338167	0.1481017	0.0867169	0.1752283
RAD54L2	0.1622008	0.2125470	0.2054535	0.1502411	0.3825544
TEX264	0.3061521	0.2566566	0.2470729	0.3799358	0.1875098
GRM2	0.0034833	0.0102811	0.0153626	0.0000000	0.0044787
IQCF3	0.0003597	0.0012989	0.0000000	0.0000000	0.0000000
IQCF1	0.0000000	0.0000000	0.0000000	0.0050900	0.0000000
ENSG00000287707	0.0010882	0.0047885	0.0000000	0.0016253	0.0092529
RRP9	0.0771196	0.0744721	0.0952883	0.0843130	0.0528596
PARP3	0.0555231	0.0283415	0.0233519	0.0320623	0.0187857
GPR62	0.0055866	0.0000000	0.0018698	0.0056222	0.0000000
PCBP4	0.2593754	0.3835732	0.4222957	0.2359011	0.3621794
ABHD14B	0.1417712	0.0548451	0.0316105	0.1146465	0.0526275
ABHD14A	0.5517453	0.4873132	0.4889024	0.6065250	0.3536345
ACY1	0.0597979	0.0608723	0.0512434	0.0625020	0.0655199
RPL29	2.6397306	2.5918784	2.4106570	2.5752513	2.3871196
DUSP7	0.0323451	0.0645734	0.0816189	0.0421010	0.0810584
POC1A	0.0122158	0.0106904	0.0103456	0.0095506	0.0314389
ALAS1	0.3287499	0.3459966	0.4116408	0.3349663	0.2310161
TLR9	0.0040458	0.0000000	0.0040055	0.0054946	0.0054122
TWF2	0.2320835	0.2176369	0.1952516	0.2240982	0.1525003
TWF2-DT	0.0012133	0.0015915	0.0031845	0.0012213	0.0000000
PPM1M	0.0567982	0.0415465	0.0261606	0.0755332	0.0339623
WDR82	0.6220712	0.7677137	0.8943104	0.5204852	0.7058342
GLYCTK	0.0124458	0.0082867	0.0118787	0.0050545	0.0101020
GLYCTK-AS1	0.0176420	0.0432327	0.0238405	0.0195477	0.0156373
DNAH1	0.0178185	0.0174743	0.0174347	0.0157910	0.0445617
BAP1	0.0946364	0.0989771	0.0940213	0.0884399	0.0873578
PHF7	0.1212940	0.1507334	0.1559202	0.1255179	0.0658024
TNNC1	0.0016153	0.0049033	0.0053912	0.0095472	0.0000000
NISCH	0.2517194	0.2981674	0.3631058	0.2146202	0.3898976
STAB1	0.0026418	0.0004619	0.0000000	0.0033167	0.0115802

NT5DC2	0.5067851	0.5851010	0.5395540	0.4994089	0.4539248
UQCC5	0.2502459	0.2140744	0.2215626	0.2436873	0.2391889
PBRM1	0.7767387	0.8995468	0.9685668	0.6739161	0.9877574
GNL3	0.5286568	0.5928093	0.5976163	0.5631185	0.4648278
GLT8D1	0.4433920	0.3075019	0.2077566	0.5775083	0.2645841
SPCS1	1.0548189	0.9588867	1.0788186	1.1536490	0.8440752
NEK4	0.2751319	0.2279188	0.1745522	0.2483478	0.2497178
ITIH1	0.0014182	0.0019204	0.0019419	0.0038595	0.0000000
ITIH4	0.0044483	0.0141055	0.0045003	0.0051929	0.0210351
MUSTN1	0.0304024	0.0200294	0.0102797	0.0212587	0.0184049
STIMATE	0.1779708	0.1131631	0.1381101	0.1242131	0.1673527
ENSG00000289519	0.0043615	0.0099333	0.0134191	0.0146203	0.0067617
SFMBT1	0.1186854	0.1084661	0.1265214	0.0976989	0.2103475
RFT1	0.0725361	0.0511461	0.0578527	0.0990391	0.0541972
PRKCD	0.0375527	0.0497693	0.0757597	0.0164571	0.0478955
TKT	1.1125608	1.0011649	1.1677581	1.1373269	0.8817238
DCP1A	0.2396298	0.2644055	0.2369628	0.1991011	0.3063873
CACNA1D	0.1053537	0.1341530	0.1199326	0.0522940	0.5424517
CHDH	0.0189922	0.0086759	0.0053737	0.0011545	0.0288340
IL17RB	0.0050746	0.0092665	0.0049160	0.0028850	0.0021475
ENSG00000271976	0.0242470	0.0393243	0.0457717	0.0269516	0.0515829
ACTR8	0.2362184	0.2616551	0.2677820	0.1957725	0.2370181
SELENOK	1.1838666	1.0230774	0.9246315	1.1663774	0.8690385
ENSG00000289063	0.0006251	0.0029239	0.0030192	0.0000000	0.0036537
ENSG00000286353	0.0223849	0.0699951	0.0366253	0.0044337	0.0627855
CACNA2D3	0.1200960	0.1738811	0.1411893	0.1675178	0.6522464
CACNA2D3-AS1	0.0013912	0.0000000	0.0012981	0.0000000	0.0000000
LRTM1	0.0530235	0.0227719	0.0156627	0.0442166	0.0398713
LINC02030	0.0000000	0.0000000	0.0000000	0.0000000	0.0000000
WNT5A	0.5728083	0.2851332	0.2161343	0.8734581	0.3330123
WNT5A-AS1	0.0137354	0.0101408	0.0000000	0.0127002	0.0146202
ENSG00000242317	0.0003511	0.0007025	0.0000000	0.0000000	0.0119754
ERC2	0.3977779	0.6536033	0.8297238	0.3266156	1.4113710
ERC2-IT1	0.0022584	0.0018829	0.0068851	0.0000000	0.0149616
CCDC66	0.4083744	0.3249315	0.2379355	0.3541003	0.4349132
TASOR	0.6757146	0.5273665	0.4888705	0.6044552	0.5976612
ARHGEF3	0.3298721	0.2426331	0.1898768	0.4362963	0.2437857
SPATA12	0.0000000	0.0000000	0.0011258	0.0000000	0.0024821
ENSG00000272202	0.0000000	0.0010742	0.0000000	0.0000000	0.0059248
IL17RD	0.1759600	0.1836330	0.2239014	0.2059947	0.2139440
HESX1	0.0170967	0.0180579	0.0103514	0.0213383	0.0162922
APPL1	0.8905739	0.7853101	0.7240667	0.7531900	0.7084167
ASB14	0.0057686	0.0009942	0.0055073	0.0041424	0.0246478
ENSG00000286952	0.0046373	0.0063735	0.0058773	0.0080113	0.0137368
DNAH12	0.2046240	0.0978859	0.0713119	0.1410478	0.1220357
ENSG00000287502	0.0005808	0.0000000	0.0000000	0.0030663	0.0000000
PDE12	0.1519542	0.1463215	0.1489637	0.1408191	0.1321746
ARF4	1.3028300	1.0275077	1.0393092	1.3782073	0.9368343
ARF4-AS1	0.0236219	0.0243514	0.0137236	0.0306379	0.0240085
DENND6A	0.1476919	0.1330090	0.0985966	0.1389042	0.2143410
DENND6A-AS1	0.0114623	0.0066709	0.0107634	0.0084147	0.0471275
DENND6A-DT	0.0000000	0.0000000	0.0000000	0.0000000	0.0000000
SLMAP	0.3040987	0.2911201	0.2516863	0.2471271	0.4485119

FLNB	0.3767964	0.3060164	0.1866860	0.2926119	0.4952234
FLNB-AS1	0.0089621	0.0091707	0.0028002	0.0000000	0.0126225
DNASE1L3	0.0004592	0.0000000	0.0000000	0.0000000	0.0000000
ABHD6	0.0851243	0.0787850	0.0539349	0.0991016	0.1050609
RPP14	0.0324548	0.0425261	0.0485737	0.0319323	0.0601584
HTD2	0.0003417	0.0010236	0.0000000	0.0031443	0.0067035
ENSG00000273493	0.0006941	0.0009224	0.0000000	0.0000000	0.0000000
PXK	0.2629454	0.2558804	0.2866042	0.2387445	0.3267448
PDHB	0.4678139	0.4277506	0.4023334	0.4907314	0.3518454
ENSG00000272182	0.0036438	0.0041879	0.0019247	0.0064468	0.0041014
ENSG00000272360	0.0000000	0.0000000	0.0000000	0.0038795	0.0000000
KCTD6	0.1495939	0.1752470	0.1669269	0.1440935	0.1447813
ACOX2	0.0120665	0.0085690	0.0033649	0.0230292	0.0000000
FAM107A	0.0123530	0.0197961	0.0095349	0.0187161	0.0093698
FAM3D-AS1	0.0018727	0.0027110	0.0000000	0.0000000	0.0000000
CFAP20DC	0.0774341	0.0727061	0.0472358	0.0422556	0.0649555
CFAP20DC-AS1	0.0051303	0.0032203	0.0068274	0.0141589	0.0255163
CFAP20DC-DT	0.0045218	0.0042749	0.0025799	0.0000000	0.0387339
FHIT	0.2488487	0.2112570	0.1539198	0.1725046	0.4881416
PTPRG	0.4373638	0.4197407	0.3733842	0.4826548	1.3731119
PTPRG-AS1	0.0512337	0.0630704	0.0279645	0.0321202	0.1317601
C3orf14	0.4399819	0.7032935	0.7206393	0.4094917	0.4661188
CADPS	0.5741641	0.6987765	0.6700547	0.4329466	1.2434256
LINC00698	0.0045209	0.0024609	0.0019687	0.0000000	0.0035288
SYNPR	0.0069408	0.0106557	0.0246190	0.0162019	0.0016673
SYNPR-AS1	0.0061261	0.0037685	0.0035092	0.0016020	0.0141852
SNTN	0.0051991	0.0022147	0.0000000	0.0000000	0.0031362
C3orf49	0.0004592	0.0005373	0.0033422	0.0000000	0.0000000
THOC7	0.6246195	0.8359227	0.8311498	0.5917321	0.6228783
ATXN7	0.1551887	0.1668823	0.1363245	0.1932819	0.2795974
PSMD6-AS2	0.0089576	0.0073300	0.0050237	0.0022544	0.0371665
ENSG00000271843	0.0036042	0.0017912	0.0012396	0.0046056	0.0143890
PSMD6	0.5148804	0.4072847	0.4109540	0.5173235	0.3244350
PSMD6-AS1	0.0000000	0.0015869	0.0000000	0.0000000	0.0063701
ENSG00000272181	0.0008671	0.0000000	0.0000000	0.0000000	0.0000000
PRICKLE2-AS1	0.0735672	0.0622448	0.0594723	0.0941032	0.0670475
LINC00994	0.0000000	0.0000000	0.0000000	0.0077989	0.0053380
PRICKLE2	0.3651549	0.3600952	0.2909475	0.3229984	0.7436754
PRICKLE2-AS3	0.0000000	0.0010566	0.0000000	0.0000000	0.0108761
ADAMTS9	0.0108003	0.0112290	0.0092955	0.0130993	0.0114782
ADAMTS9-AS1	0.0000000	0.0000000	0.0000000	0.0000000	0.0000000
ADAMTS9-AS2	0.0497203	0.0309420	0.0209026	0.0119427	0.1017266
MAGI1	0.3419798	0.4007197	0.3881385	0.2613722	1.0757911
ENSG00000270059	0.0149703	0.0358630	0.0592469	0.0108294	0.0258835
MAGI1-AS1	0.0003101	0.0000000	0.0000000	0.0000000	0.0014245
SLC25A26	0.2311254	0.2487983	0.2747200	0.1384347	0.3000912
LRIG1	0.3754303	0.1808522	0.1458680	0.4922266	0.2695384
KBTBD8	0.0111138	0.0239291	0.0186799	0.0060815	0.0153046
SUCLG2	0.2315769	0.1558896	0.0809479	0.2790926	0.1434321
SUCLG2-DT	0.0629999	0.0520954	0.0388992	0.0391275	0.0768928
TAFA1	0.1338877	0.1732906	0.2060701	0.0420369	0.8122155
ENSG00000286967	0.0000000	0.0000000	0.0000000	0.0000000	0.0000000
TAFA4	0.0580300	0.0565091	0.0224772	0.0730349	0.0845616

EOGT	0.0218159	0.0325279	0.0187116	0.0514929	0.0678934
EOGT-DT	0.0143908	0.0131944	0.0094056	0.0194129	0.0171035
TMF1	0.7262623	0.6708898	0.6091119	0.6499265	0.6634359
UBA3	0.2830974	0.2690981	0.2808792	0.2773365	0.3234822
ARL6IP5	1.3323114	1.2074327	1.3522561	1.6743245	1.0596893
LMOD3	0.0060205	0.0022941	0.0080694	0.0178112	0.0092750
FRMD4B	0.4480221	0.4027921	0.3778184	0.3627003	0.6334978
MITF	0.0546805	0.0229323	0.0043591	0.0252420	0.0257824
SAMMSON	0.0000000	0.0008763	0.0017606	0.0000000	0.0000000
FOXP1	1.0290427	0.6650874	0.4741144	0.9370308	0.8341080
FOXP1-AS1	0.0000000	0.0000000	0.0000000	0.0000000	0.0000000
FOXP1-IT1	0.0000000	0.0000000	0.0000000	0.0000000	0.0055645
ENSG00000277855	0.0226381	0.0106942	0.0076482	0.0193871	0.0202151
FOXP1-DT	0.0000000	0.0000000	0.0024882	0.0000000	0.0031362
EIF4E3	0.2229611	0.2788841	0.3141005	0.1896094	0.3011047
GPR27	0.2380581	0.2841069	0.3186564	0.2768371	0.2388043
LINC00877	0.0245601	0.0295240	0.0140842	0.0322189	0.0608793
LINC00870	0.0000000	0.0020389	0.0000000	0.0000000	0.0037811
RYBP	0.6908144	0.6900335	0.7843356	0.6017129	0.7025287
ENSG00000244345	0.0000000	0.0000000	0.0000000	0.0000000	0.0000000
SHQ1	0.0755702	0.0520925	0.0412406	0.1198917	0.0802959
GXYLT2	0.2079235	0.1696294	0.0747716	0.2172605	0.1750632
PPP4R2	0.5694354	0.4829225	0.5386078	0.5630608	0.6104162
EBLN2	0.0005385	0.0057241	0.0000000	0.0023988	0.0028494
PDZRN3	0.2873540	0.2454051	0.1726370	0.2685727	0.4152198
PDZRN3-AS1	0.0094107	0.0051414	0.0043619	0.0036595	0.0184272
CNTN3	0.0946901	0.0800833	0.0825903	0.0780856	0.2343681
ENSG00000287398	0.0015243	0.0000000	0.0030415	0.0028407	0.0000000
FAM86DP	0.0418546	0.0271691	0.0264224	0.0489905	0.0358037
LINC02018	0.0216889	0.0171402	0.0199154	0.0075708	0.0434294
ENSG00000290410	0.0025003	0.0000000	0.0021854	0.0000000	0.0019259
LINC00960	0.0172468	0.0239403	0.0139664	0.0324232	0.0347504
ZNF717	0.0429745	0.0675588	0.0415736	0.0823418	0.1985777
ROBO2	0.5235564	0.7390586	0.7245287	0.4814087	1.8709150
ENSG00000288793	0.0012238	0.0000000	0.0000000	0.0000000	0.0000000
ROBO1	0.7032940	0.8011464	0.7635694	0.5930693	2.0315507
ENSG00000288105	0.0045093	0.0027600	0.0021368	0.0039294	0.1092680
ENSG00000241593	0.0010423	0.0016471	0.0000000	0.0049851	0.0000000
GBE1	0.2200254	0.1762309	0.1546237	0.1270840	0.3046932
ENSG00000242009	0.0022278	0.1122558	0.0041056	0.1109987	0.0000000
LINC02008	0.0022855	0.0000000	0.0000000	0.0000000	0.0000000
CADM2	0.8664432	1.1153840	1.3286121	0.7223853	2.4002183
CADM2-AS2	0.0015693	0.0000000	0.0000000	0.0018898	0.0095629
CADM2-AS1	0.0017478	0.0014564	0.0020745	0.0000000	0.0211397
VGLL3	0.1293236	0.0753655	0.0303517	0.1232308	0.0395277
LINC00506	0.0000000	0.0073463	0.0053247	0.0017126	0.0208389
CHMP2B	0.7062073	0.6359420	0.6971317	0.7325462	0.5824646
ENSG00000239572	0.0006948	0.0000000	0.0000000	0.0036873	0.0000000
HTR1F	0.0015711	0.0000000	0.0012498	0.0000000	0.0075813
CGGBP1	0.8166645	0.9395949	1.0226802	0.8208849	0.7211484
ZNF654	0.2517072	0.2671129	0.2764280	0.2517592	0.3793723
C3orf38	0.4359853	0.3461526	0.3298761	0.4095551	0.3438719
CSNKA2IP	0.0051789	0.0123116	0.0020083	0.0107143	0.0291330

EPHA3	0.1951805	0.2320012	0.1986543	0.2166183	0.7279583
ENSG00000285780	0.0085520	0.0064266	0.0049645	0.0000000	0.0089421
PROS1	0.1542388	0.0902784	0.0974345	0.1952320	0.1649413
ARL13B	0.2596835	0.1605762	0.1236742	0.2892191	0.2207665
STX19	0.0000000	0.0014946	0.0000000	0.0000000	0.0000000
DHFR2	0.0581101	0.0510739	0.0574820	0.0679004	0.0868081
NSUN3	0.1011742	0.1012258	0.1383745	0.1229273	0.1293244
ENSG00000286447	0.0027956	0.0086541	0.0062161	0.0043841	0.0083695
EPHA6	0.2022707	0.2897528	0.2760472	0.1293569	0.9026926
ARL6	0.1145801	0.1527846	0.1680731	0.1205022	0.2155946
ENSG00000282527	0.0000000	0.0013833	0.0000000	0.0000000	0.0000000
CRYBG3	0.1264370	0.0838275	0.0364182	0.1471717	0.0843474
ENSG00000279658	0.0000000	0.0000000	0.0000000	0.0000000	0.0028353
RIOX2	0.3319120	0.2191793	0.1652366	0.4004040	0.1604924
ENSG00000251088	0.0007324	0.0026688	0.0000000	0.0000000	0.0042185
CLDND1	0.5085041	0.4310737	0.4579301	0.6687711	0.3474514
ENSG00000248839	0.0009361	0.0000000	0.0000000	0.0000000	0.0000000
ENSG00000286602	0.0021971	0.0089560	0.0037342	0.0064555	0.0228162
CPOX	0.0733485	0.0975480	0.1050696	0.0888414	0.0781122
ST3GAL6-AS1	0.0035069	0.0026191	0.0076489	0.0000000	0.0038438
ST3GAL6	0.0292207	0.0525662	0.0408663	0.0177940	0.1020334
DCBLD2	0.3311290	0.3371735	0.3540651	0.3522532	0.4179423
ENSG00000239462	0.0014894	0.0063202	0.0079197	0.0000000	0.0056296
COL8A1	0.0334680	0.0275747	0.0326158	0.0762476	0.0493618
ENSG00000273374	0.0034620	0.0027097	0.0014362	0.0057107	0.0087236
CMSS1	0.4061991	0.5064157	0.5410955	0.4265031	0.6076062
FILIP1L	0.6794530	0.4446175	0.2475840	0.6685172	0.5122004
ENSG00000287378	0.0058431	0.0039437	0.0050491	0.0060371	0.0065920
TBC1D23	0.3784102	0.3015795	0.2491260	0.3302900	0.2884255
NIT2	0.2807938	0.2164503	0.2220498	0.2367332	0.1968620
TOMM70	0.4673372	0.5795449	0.8216540	0.5054482	0.4695416
LNP1	0.1001317	0.1393302	0.1366179	0.0972039	0.1021816
TMEM45A	0.2313748	0.2170924	0.2410707	0.2366061	0.1931112
ADGRG7	0.0013673	0.0000000	0.0000000	0.0000000	0.0000000
TFG	0.7704325	0.5869782	0.5692604	0.7962067	0.5000326
ABI3BP	0.0089491	0.0109975	0.0086269	0.0047839	0.0518481
IMPG2	0.0226807	0.0174229	0.0217331	0.0286392	0.0918610
SENP7	0.4375739	0.4996884	0.4865607	0.4659696	0.7831280
TRMT10C	0.3597419	0.3744516	0.3736275	0.4066766	0.2946701
PCNP	1.1165935	1.0837354	1.1199266	1.1155647	0.9212074
ENSG00000289629	0.0097664	0.0062828	0.0052721	0.0025427	0.0041632
ZBTB11	0.2502798	0.2133464	0.2125417	0.1605678	0.2508696
ZBTB11-AS1	0.0186266	0.0103548	0.0034306	0.0172422	0.0062764
RPL24	2.8330832	2.8235878	2.6979423	2.7021752	2.5864292
CEP97	0.3145153	0.3004761	0.3447872	0.2443794	0.3626430
NXPE3	0.2459993	0.2592725	0.2433570	0.2240943	0.2307768
ENSG00000249474	0.0026118	0.0009351	0.0000000	0.0028137	0.0057307
NFKBIZ	0.0265167	0.0190230	0.0230401	0.0076266	0.0432789
ENSG00000287682	0.0000000	0.0000000	0.0000000	0.0000000	0.0000000
ZPLD1	0.0009367	0.0011466	0.0000000	0.0000000	0.0000000
ALCAM	2.3992030	1.8453384	1.5582001	2.7224216	1.9477729
CBLB	0.2074570	0.2322479	0.1810094	0.1342922	0.5865675
ENSG00000288848	0.0127584	0.0079379	0.0163793	0.0075822	0.0054292

ENSG00000286854	0.0000000	0.0031237	0.0088111	0.0049681	0.0160123
ENSG00000291293	0.0034060	0.0104799	0.0041902	0.0000000	0.0316281
LINC00882	0.2248439	0.2108565	0.2418200	0.2417977	0.4861642
ENSG00000288557	0.0004455	0.0038723	0.0047720	0.0036005	0.0000000
ENSG00000286956	0.0000000	0.0000000	0.0000000	0.0000000	0.0033946
DUBR	0.3053832	0.3284625	0.3465192	0.2733471	0.3924906
CCDC54-AS1	0.0122302	0.0087749	0.0075389	0.0089427	0.0514917
ENSG00000272597	0.0106055	0.0133862	0.0167004	0.0025984	0.0149953
LINC01990	0.0423868	0.0343135	0.0487373	0.0637784	0.0324459
BBX	0.8302126	0.5598702	0.4606631	0.7893327	0.7149311
LINC00636	0.0017367	0.0035940	0.0000000	0.0061736	0.0000000
LINC00635	0.0000000	0.0000000	0.0023634	0.0000000	0.0000000
ENSG00000279277	0.0044464	0.0023685	0.0033444	0.0000000	0.0168371
CD47	0.9030079	0.7181120	0.7599244	1.1766615	0.7652316
IFT57	0.8330487	0.6667179	0.6778712	0.8955182	0.5515451
HHLA2	0.0019465	0.0025951	0.0000000	0.0013877	0.0000000
MYH15	0.0077014	0.0034872	0.0056320	0.0079355	0.0331004
CIP2A	0.0542900	0.0326434	0.0386099	0.0426422	0.0595841
DZIP3	0.7836990	0.7187748	0.7602834	0.8467974	0.8126708
MORC1	0.0012829	0.0000000	0.0000000	0.0000000	0.0000000
LINC00488	0.0060196	0.0023997	0.0056013	0.0000000	0.0000000
LINC01205	0.0007836	0.0023549	0.0033552	0.0000000	0.0000000
ENSG00000242029	0.0000000	0.0000000	0.0000000	0.0000000	0.0000000
NECTIN3-AS1	0.1186986	0.1042702	0.0804095	0.0857037	0.0923475
NECTIN3	1.6034194	1.0959332	0.8120801	1.4261272	1.1953024
CD96	0.0216401	0.0138001	0.0056032	0.0041661	0.0175826
PLCXD2	0.0365463	0.0915980	0.0742603	0.0306249	0.1055027
PHLDB2	0.1754631	0.1208569	0.0778621	0.1667012	0.1823799
ENSG00000286492	0.0059067	0.0006244	0.0034621	0.0043247	0.0071630
ABHD10	0.3390564	0.3976554	0.3913643	0.3803302	0.2462721
TAGLN3	0.8483458	0.9998830	1.1418456	0.8842312	0.7498303
TMPRSS7	0.0046845	0.0028779	0.0011130	0.0077806	0.0000000
C3orf52	0.0007967	0.0000000	0.0000000	0.0000000	0.0062626
SLC9C1	0.0165757	0.0217819	0.0066521	0.0077453	0.0095214
ENSG00000239482	0.0117789	0.0086762	0.0058280	0.0108173	0.0106335
CD200	0.6344215	0.5754639	0.6865289	0.5809945	0.6370177
BTLA	0.0018226	0.0000000	0.0022352	0.0000000	0.0052568
ATG3	0.4711150	0.4093662	0.4381206	0.4272342	0.4068112
SLC35A5	0.1630994	0.1419471	0.1586263	0.2040895	0.1101711
CCDC80	0.7429080	0.4588927	0.3242549	1.2101800	0.4859089
CD200R1	0.0006781	0.0008382	0.0000000	0.0000000	0.0028494
ENSG00000272844	0.0002410	0.0037515	0.0037846	0.0000000	0.0090284
GTPBP8	0.2188460	0.2002847	0.1663405	0.1544903	0.1732277
NEPRO	0.3362379	0.3205978	0.2567296	0.3591655	0.2018944
NEPRO-AS1	0.0448032	0.0287440	0.0385920	0.0435867	0.0934491
ENSG00000241219	0.0027614	0.0005684	0.0000000	0.0000000	0.0204168
LINC02044	0.0016698	0.0000000	0.0000000	0.0000000	0.0000000
BOC	0.0977635	0.0588689	0.0298735	0.0932288	0.0829655
ENSG00000288079	0.0015019	0.0000000	0.0000000	0.0026652	0.0000000
CFAP44	0.3949379	0.2569272	0.1693807	0.3865352	0.3731560
CFAP44-AS1	0.0089334	0.0029926	0.0044313	0.0051341	0.0000000
SPICE1	0.2122438	0.1441268	0.1098428	0.2107508	0.1867880
SIDT1	0.0113820	0.0084293	0.0217004	0.0043974	0.0237364

SIDT1-AS1	0.0000000	0.0000000	0.0000000	0.0000000	0.0000000
USF3	0.3686415	0.3737312	0.3940895	0.3078532	0.2737426
NAA50	0.4754167	0.4690699	0.4726930	0.4951144	0.3715707
ATP6V1A	0.7665172	0.8743365	1.0830770	0.7022562	0.8507290
GRAMD1C	0.0310403	0.0289931	0.0097738	0.0249196	0.0573598
ENSG00000273394	0.0021918	0.0008112	0.0018644	0.0000000	0.0000000
ZDHHC23	0.0142785	0.0209358	0.0251255	0.0074520	0.0541305
CCDC191	0.3886027	0.2509543	0.1843693	0.3783812	0.3565866
ENSG00000287805	0.0012477	0.0009283	0.0000000	0.0000000	0.0000000
ENSG00000285836	0.0000000	0.0000000	0.0000000	0.0000000	0.0091816
QTRT2	0.1275763	0.0509717	0.0645350	0.1175553	0.1201370
DRD3	0.0045018	0.0014940	0.0000000	0.0000000	0.0012770
ENSG00000241490	0.0000000	0.0000000	0.0000000	0.0000000	0.0000000
TIGIT	0.0000000	0.0000000	0.0000000	0.0000000	0.0015072
ZBTB20	0.7913894	0.5700092	0.5237770	0.6674040	0.9743515
ZBTB20-AS1	0.0010155	0.0000000	0.0038281	0.0000000	0.0193593
ZBTB20-AS5	0.0059856	0.0034435	0.0055712	0.0000000	0.1039258
ZBTB20-AS2	0.0000000	0.0000000	0.0000000	0.0000000	0.0100722
ZBTB20-AS3	0.0000000	0.0000000	0.0000000	0.0000000	0.0068745
ZBTB20-AS4	0.0044887	0.0000000	0.0095213	0.0048652	0.0033339
ENSG00000242880	0.0158586	0.0162735	0.0157303	0.0167553	0.0722895
ENSG00000288896	0.0017958	0.0000000	0.0000000	0.0000000	0.0162876
ENSG00000287795	0.0000000	0.0000000	0.0000000	0.0000000	0.0154241
GAP43	1.7622248	2.6437459	3.1583474	1.6408864	2.2181304
ENSG00000289153	0.0000000	0.0024295	0.0000000	0.0097208	0.0020430
LSAMP	0.8156558	1.1881943	1.3131975	0.8065202	2.1947060
LSAMP-AS1	0.0000000	0.0000000	0.0012924	0.0000000	0.0126481
LINC00903	0.0017724	0.0014712	0.0000000	0.0044812	0.0384307
TUSC7	0.0015328	0.0000000	0.0000000	0.0000000	0.0028791
LINC00901	0.0009736	0.0000000	0.0019886	0.0000000	0.0076771
LINC03051	0.2133590	0.2855722	0.1953742	0.1589812	1.1704274
LINC02024	0.0042450	0.0025818	0.0044640	0.0000000	0.0061676
ENSG00000242816	0.0012186	0.0000000	0.0000000	0.0000000	0.0210226
ENSG00000243276	0.0020249	0.0049336	0.0015753	0.0000000	0.0053071
IGSF11	0.0330901	0.0334951	0.0014744	0.0480691	0.0947943
TEX55	0.0000000	0.0000000	0.0000000	0.0000000	0.0052174
UPK1B	0.0000000	0.0000000	0.0000000	0.0000000	0.0000000
B4GALT4	0.2562362	0.1839892	0.1471599	0.3346156	0.1631558
B4GALT4-AS1	0.0038842	0.0020619	0.0000000	0.0000000	0.0083414
ARHGAP31	0.0259616	0.0130971	0.0026502	0.0580787	0.0248820
ARHGAP31-AS1	0.0019978	0.0029263	0.0000000	0.0000000	0.0037333
TMEM39A	0.0579648	0.0470997	0.0487459	0.0491840	0.1266472
POGLUT1	0.1067585	0.0880181	0.1000677	0.1443932	0.1917285
TIMMDC1-DT	0.0000000	0.0000000	0.0000000	0.0000000	0.0000000
TIMMDC1	0.5510861	0.5038899	0.4344369	0.6207011	0.3598381
CD80	0.0008061	0.0018932	0.0000000	0.0000000	0.0030832
ENSG00000272967	0.0017685	0.0000000	0.0000000	0.0000000	0.0000000
ADPRH	0.0051294	0.0080977	0.0016709	0.0088281	0.0062626
PLA1A	0.0000000	0.0000000	0.0000000	0.0028524	0.0000000
POPDC2	0.0078033	0.0159809	0.0208958	0.0010526	0.0159338
ENSG00000289134	0.0000000	0.0000000	0.0013842	0.0000000	0.0000000
COX17	0.7935118	0.7773700	0.9881202	0.6968563	0.6425554
ENSG00000286584	0.0022161	0.0011193	0.0024822	0.0000000	0.0000000

CFAP91	0.2102018	0.1267569	0.1627897	0.2512037	0.1852552
NR1I2	0.0012526	0.0034461	0.0143906	0.0000000	0.0081241
GSK3B	0.7357001	0.8975713	0.9823953	0.6687417	1.1076847
GSK3B-DT	0.0392571	0.0478599	0.0182223	0.0319762	0.0796400
GPR156	0.0166131	0.0082083	0.0049634	0.0090280	0.0361661
LRRC58	0.3625640	0.2721032	0.1821604	0.3973018	0.2750356
FSTL1	1.4964950	0.9577469	0.6715116	1.9794437	0.9338493
ENSG00000286735	0.0133933	0.0092243	0.0103166	0.0071610	0.0166945
NDUFB4	1.1536399	1.1911462	1.2025897	1.1369024	0.9175418
HGD	0.0035747	0.0039665	0.0000000	0.0000000	0.0042647
RABL3	0.1568511	0.1216117	0.0795572	0.1269460	0.0935187
GTF2E1	0.1042389	0.0814508	0.1214607	0.1066950	0.1023981
ENSG00000286827	0.0025525	0.0000000	0.0000000	0.0040841	0.0031362
STXBP5L	0.0906878	0.1149402	0.1529961	0.0565571	0.4903162
ENSG00000287022	0.0002717	0.0000000	0.0000000	0.0000000	0.0000000
POLQ	0.0013673	0.0019762	0.0025528	0.0033784	0.0065348
FBXO40	0.0000000	0.0000000	0.0062258	0.0000000	0.0000000
HCLS1	0.0019921	0.0031132	0.0000000	0.0000000	0.0025831
GOLGB1	0.9480806	0.8466819	0.7545904	0.8942171	1.0649155
ENSG00000288868	0.0082127	0.0144327	0.0105157	0.0015108	0.0278779
IQCB1	0.2461437	0.2094560	0.1892391	0.2410054	0.3189185
EAF2	0.0603055	0.0389833	0.0301749	0.0602325	0.0836265
SLC15A2	0.1251193	0.0676680	0.0513601	0.2596774	0.0532764
CASR	0.0000000	0.0000000	0.0000000	0.0000000	0.0034190
MIX23	0.2396757	0.2356080	0.2611459	0.2467934	0.1875774
FAM162A	1.0702961	0.9745876	1.0049060	0.9875300	0.9284306
WDR5B	0.0718838	0.0595792	0.0346614	0.0492697	0.0360821
WDR5B-DT	0.1352689	0.0804690	0.0782786	0.0908534	0.1054067
KPNA1	0.4033281	0.3737703	0.4050220	0.3952280	0.4535072
ENSG00000287207	0.0062869	0.0017000	0.0031702	0.0081380	0.0040606
PARP9	0.0696213	0.0437292	0.0097884	0.0976228	0.0247494
DTX3L	0.0657837	0.0148959	0.0179792	0.0607053	0.0340808
PARP14	0.1694388	0.1216209	0.0458695	0.1640131	0.1041751
HSPBAP1	0.0322848	0.0330339	0.0362522	0.0291341	0.0459007
SLC49A4	0.0615554	0.0701442	0.0711704	0.1695527	0.1635363
LINC02035	0.0196532	0.0160203	0.0284537	0.0148546	0.0251801
SEMA5B	0.1198262	0.0833301	0.0479603	0.2035329	0.1741988
PDIA5	0.1133063	0.0569351	0.0332255	0.2088680	0.0799770
SEC22A	0.0968399	0.1301715	0.0838832	0.1287572	0.2203570
ENSG00000273454	0.0036837	0.0000000	0.0000000	0.0000000	0.0073194
ADCY5	0.0517956	0.0902030	0.1123594	0.0467849	0.1898880
ENSG00000272678	0.0008873	0.0081293	0.0000000	0.0000000	0.0000000
HACD2	0.2216551	0.1942823	0.2305870	0.2582432	0.2127127
MYLK-AS1	0.0569542	0.0536801	0.0270129	0.0642153	0.0712323
MYLK	0.1023520	0.0460646	0.0421041	0.0518095	0.1023377
MYLK-AS2	0.0015340	0.0025453	0.0000000	0.0000000	0.0048432
ENSG00000288806	0.0048124	0.0067314	0.0000000	0.0037188	0.0000000
CCDC14	0.2146064	0.2722174	0.1726673	0.1872492	0.4163108
KALRN	0.2854891	0.3715217	0.3119055	0.2544881	1.1118338
ENSG00000288713	0.0009837	0.0013977	0.0018817	0.0000000	0.0114670
UMPS	0.1653948	0.1299398	0.1061495	0.2121120	0.1798946
ITGB5	0.2070086	0.0930236	0.0474873	0.3455050	0.0985311
ITGB5-AS1	0.0000000	0.0000000	0.0035760	0.0000000	0.0031362

MUC13	0.0011329	0.0000000	0.0000000	0.0000000	0.0000000
HEG1	0.1301026	0.0727347	0.0259632	0.1712684	0.0575248
SLC12A8	0.0241021	0.0195777	0.0224537	0.0407102	0.0495510
ZNF148	0.6079168	0.7013659	0.6209475	0.5660131	0.8654943
SNX4	0.6335475	0.6259744	0.6277125	0.6686365	0.5752499
OSBPL11	0.2942863	0.2798553	0.2559224	0.3208284	0.2484515
ENSG00000284624	0.0972504	0.0822741	0.0603352	0.1054427	0.1011914
ENSG00000272840	0.0009193	0.0043424	0.0046477	0.0079578	0.0000000
ENSG00000287617	0.0035305	0.0000000	0.0062647	0.0000000	0.0034872
LINC02614	0.0375637	0.0336334	0.0203062	0.0382536	0.1157240
ENSG00000248787	0.0084619	0.0075273	0.0050846	0.0122500	0.0142546
FAM86JP	0.0215161	0.0109226	0.0074122	0.0083701	0.0047598
ENSG00000291096	0.0246200	0.0200543	0.0220363	0.0212092	0.0276955
ROPN1B	0.0027761	0.0012435	0.0170601	0.0050066	0.0087615
SLC41A3	0.4314510	0.3487206	0.3329443	0.4711304	0.2981698
SLC41A3-AS1	0.0450686	0.0351117	0.0474490	0.0337422	0.0129654
ALDH1L1	0.0040566	0.0019415	0.0000000	0.0054025	0.0035640
ALDH1L1-AS1	0.0006095	0.0000000	0.0000000	0.0029023	0.0000000
ENSG00000287232	0.0102731	0.0084573	0.0067869	0.0013375	0.0059046
ENSG00000250934	0.0182858	0.0125425	0.0117124	0.0226503	0.0054061
KLF15	0.0261402	0.0098319	0.0083980	0.0208497	0.0063339
CFAP100	0.1030850	0.0662064	0.0379179	0.0810292	0.0777063
ZXDC	0.2555993	0.2814468	0.2771266	0.2274073	0.3797507
UROC1	0.0014286	0.0000000	0.0000000	0.0055630	0.0000000
CHST13	0.0000000	0.0018314	0.0011859	0.0000000	0.0019007
C3orf22	0.0000000	0.0000000	0.0000000	0.0000000	0.0091132
TXNRD3	0.1279792	0.1156287	0.0848974	0.1573041	0.2376814
CHCHD6	0.3892521	0.4735062	0.5969490	0.3860163	0.4863224
PLXNA1	0.1563960	0.2094355	0.1965231	0.1954980	0.3438402
LINC02016	0.0031817	0.0066142	0.0021171	0.0020123	0.0127374
LINC01471	0.0016014	0.0015507	0.0000000	0.0000000	0.0000000
TPRA1	0.1843574	0.1583621	0.1116121	0.1836178	0.1449814
MCM2	0.0270305	0.0217881	0.0160295	0.0401169	0.0433760
ENSG00000285600	0.0103627	0.0169023	0.0250585	0.0190565	0.0044999
PODXL2	0.4565595	0.7197393	0.9866242	0.4468894	0.5994009
ABTB1	0.0244937	0.0128203	0.0156223	0.0275003	0.0202592
MGLL	0.1088793	0.1570195	0.1349536	0.0636954	0.1342414
ENSG00000287143	0.0010172	0.0000000	0.0000000	0.0000000	0.0000000
KBTBD12	0.0012717	0.0013944	0.0014327	0.0000000	0.0047701
SEC61A1	0.3980155	0.2878995	0.3081891	0.5810883	0.2640489
RUVBL1	0.7330523	0.5478930	0.5657186	0.7828486	0.4211457
EEFSEC	0.2201076	0.2048174	0.1744962	0.1521243	0.3458581
ENSG00000285619	0.0000000	0.0000000	0.0000000	0.0000000	0.0038113
GATA2	0.0754357	0.1675805	0.4603937	0.0397296	0.2423078
GATA2-AS1	0.0224238	0.0557419	0.1474679	0.0060895	0.0330117
ENSG00000286806	0.0006765	0.0018103	0.0026855	0.0000000	0.0000000
LINC01565	0.0000000	0.0005356	0.0000000	0.0000000	0.0097677
RPN1	0.8502768	0.7114604	0.7727285	1.0783316	0.6495643
RAB7A	1.0630522	1.0199332	1.0551246	1.0800593	0.9098890
ENSG00000290241	0.0017866	0.0020353	0.0016097	0.0025617	0.0110061
ENSG00000261159	0.0144069	0.0102423	0.0119256	0.0140510	0.0101095
IFT122P3	0.0082050	0.0024925	0.0010852	0.0000000	0.0130046
ACAD9-DT	0.0082039	0.0213331	0.0197225	0.0073709	0.0258612

ACAD9	0.1614463	0.1390707	0.1523025	0.1390090	0.1796468
CFAP92	0.2688334	0.1546237	0.1173622	0.2570200	0.1130779
EFCC1	0.4424521	0.2265707	0.1505424	0.4855207	0.2543175
RAB43	0.0339834	0.0217430	0.0148172	0.0363853	0.0538922
ISY1	0.2656901	0.2098016	0.1843613	0.2823417	0.2450023
ENSG00000288996	0.0002394	0.0036104	0.0000000	0.0000000	0.0120408
ENSG00000273437	0.0299914	0.0363340	0.0452479	0.0186493	0.0089444
CNBP	1.3317726	1.1160454	1.0690085	1.2230412	0.9060642
ENSG00000289469	0.0389471	0.0494110	0.0196594	0.0661853	0.0529505
ENSG00000289351	0.0006962	0.0000000	0.0000000	0.0000000	0.0000000
COPG1	0.3200038	0.2819850	0.3051268	0.3103374	0.2308768
ENSG00000286729	0.0040674	0.0000000	0.0000000	0.0000000	0.0038318
HMCES	0.2089936	0.1949270	0.1707398	0.1963090	0.1686365
H1-10	1.0605235	1.0973300	1.2683639	0.8588365	0.8736545
H1-10-AS1	0.0391343	0.0240010	0.0149589	0.0225165	0.0434372
ENSG00000290993	0.1169873	0.1234845	0.0950112	0.0881035	0.1656081
EFCAB12	0.0686647	0.0444310	0.0292139	0.0799166	0.0348258
IFT122	0.1086791	0.0849572	0.0559645	0.1326466	0.1052743
MBD4	0.3031700	0.3413485	0.3614187	0.2653917	0.3266003
H1-8	0.0053634	0.0000000	0.0000000	0.0000000	0.0000000
PLXND1	0.0314608	0.0363373	0.0155089	0.0294288	0.0134646
TMCC1	0.4747039	0.5802036	0.5602895	0.4793181	0.8611831
ENSG00000203644	0.0250263	0.0132517	0.0141832	0.0277881	0.0074688
TMCC1-DT	0.0667493	0.0658489	0.0518575	0.0916636	0.0698560
ENSG00000250643	0.0000000	0.0000000	0.0000000	0.0000000	0.0057154
TRH	0.0137511	0.0410748	0.1056349	0.0248020	0.0031970
ALG1L2	0.0042854	0.0021818	0.0018758	0.0000000	0.0000000
LINC02014	0.0000000	0.0012831	0.0017912	0.0000000	0.0016028
ENSG00000291081	0.0207107	0.0370203	0.0252300	0.0020830	0.0196701
LINC02021	0.0312289	0.0173803	0.0147981	0.0371063	0.0221189
ENSG00000288111	0.0036936	0.0020473	0.0016313	0.0000000	0.0000000
COL6A5	0.0000000	0.0000000	0.0009114	0.0000000	0.0000000
COL6A6	0.0062264	0.0068557	0.0023930	0.0025427	0.0250925
PIK3R4	0.0483317	0.0493594	0.0557044	0.0567603	0.0944777
ATP2C1	0.5441429	0.6228872	0.6333574	0.5575633	0.8913717
ENSG00000250592	0.0010236	0.0000000	0.0000000	0.0000000	0.0044881
ASTE1	0.0430033	0.0583519	0.0502646	0.0369672	0.0410958
NEK11	0.5974016	0.3947836	0.2211006	0.6331144	0.4538123
ENSG00000250129	0.0000000	0.0000000	0.0000000	0.0000000	0.0000000
NUDT16-DT	0.0148732	0.0289830	0.0149120	0.0169108	0.1670888
NUDT16	0.3696379	0.3222352	0.3083146	0.3388761	0.3002700
ENSG00000261167	0.0119992	0.0119188	0.0170271	0.0000000	0.0060461
MRPL3	0.5038472	0.5214339	0.6305578	0.5144691	0.4063061
ENSG00000248468	0.0009918	0.0000000	0.0000000	0.0043615	0.0063744
CPNE4	0.0902735	0.1526699	0.1789062	0.0486831	0.2393854
ENSG00000289118	0.0000000	0.0000000	0.0000000	0.0000000	0.0000000
ACP3	0.0019903	0.0000000	0.0000000	0.0033967	0.0000000
DNAJC13	0.2447555	0.2257753	0.2507686	0.2225168	0.3977865
ACAD11	0.1656569	0.1231932	0.0686359	0.1518502	0.2105072
ACKR4	0.0003266	0.0012534	0.0000000	0.0000000	0.0014498
UBA5	0.3908897	0.3802077	0.3883837	0.4154787	0.3414203
NPHP3	0.2025233	0.1488234	0.0745795	0.1421229	0.1818988
NPHP3-AS1	0.0010064	0.0008628	0.0015898	0.0000000	0.0059167

ENSG00000249725	0.0012022	0.0000000	0.0011329	0.0000000	0.0019007
TMEM108	0.2708831	0.2528101	0.1728056	0.2821482	0.7205894
TMEM108-AS1	0.0000000	0.0000000	0.0000000	0.0000000	0.0000000
BFSP2	0.0010695	0.0017571	0.0007672	0.0030695	0.0000000
BFSP2-AS1	0.0000000	0.0000000	0.0000000	0.0000000	0.0046088
ENSG00000272832	0.0000000	0.0018748	0.0000000	0.0000000	0.0000000
CDV3	0.7576729	0.8010725	0.7739931	0.5878010	0.6146777
TOPBP1	0.2570578	0.2921444	0.3156495	0.2711626	0.2962310
ENSG00000291042	0.0329980	0.0350085	0.0393717	0.0421871	0.0902752
TF	0.1856456	0.0990770	0.0636779	0.4032428	0.1823369
SRPRB	0.2362489	0.1829429	0.2351277	0.3246513	0.1585956
ENSG00000285908	0.0013098	0.0029719	0.0006833	0.0000000	0.0182783
RAB6B	0.8456032	1.3809129	1.5261920	0.7713746	1.0357367
SLCO2A1	0.0000000	0.0017842	0.0032784	0.0000000	0.0000000
RYK	0.3566443	0.2451811	0.2501158	0.3936711	0.3526949
LINC02004	0.0007657	0.0000000	0.0000000	0.0000000	0.0031362
ENSG00000260633	0.0000000	0.0056501	0.0028743	0.0000000	0.0000000
AMOTL2	0.0646035	0.1315599	0.1455510	0.0613421	0.1793188
ANAPC13	0.6606217	0.7447910	0.7507736	0.6092327	0.5620508
CEP63	0.1715372	0.1351964	0.1419841	0.1748522	0.1259712
ENSG00000288700	0.0222534	0.0139340	0.0099699	0.0159223	0.0741149
KY	0.0102347	0.0095393	0.0059146	0.0087809	0.0061768
ENSG00000286982	0.0030639	0.0097314	0.0097863	0.0088796	0.0000000
EPHB1	0.1331904	0.2016953	0.2250337	0.1479853	0.4910908
ENSG00000240086	0.0321634	0.0851325	0.0571450	0.0396769	0.1379200
PPP2R3A	0.2548506	0.2982515	0.3620697	0.2563181	0.4986852
MSL2	0.1436402	0.1363082	0.1621544	0.1150272	0.1773973
PCCB	0.3218997	0.2555913	0.1820967	0.3387044	0.2491131
STAG1	0.3517462	0.3444165	0.3519383	0.2933521	0.7344166
STAG1-DT	0.0029343	0.0000000	0.0016034	0.0000000	0.0000000
ENSG00000286915	0.0006466	0.0000000	0.0000000	0.0000000	0.0031362
SLC35G2	0.0716530	0.1086430	0.1333260	0.0725926	0.1412751
NCK1-DT	0.0577613	0.0374368	0.0440890	0.0202524	0.0336342
ENSG00000273486	0.0006692	0.0078125	0.0108251	0.0094442	0.0125224
NCK1	0.2077421	0.2012850	0.1792116	0.1942440	0.2478717
IL20RB	0.0080453	0.0044149	0.0193123	0.0052203	0.0309208
IL20RB-AS1	0.0013373	0.0018255	0.0000000	0.0000000	0.0000000
SOX14	0.0076598	0.0164406	0.0121120	0.0000000	0.0123742
LINC01210	0.0000000	0.0000000	0.0000000	0.0000000	0.0000000
ENSG00000261146	0.0000000	0.0000000	0.0000000	0.0000000	0.0000000
CLDN18	0.0025321	0.0012208	0.0033800	0.0000000	0.0033772
ENSG00000272609	0.0022585	0.0000000	0.0000000	0.0000000	0.0041430
DZIP1L	0.1119216	0.0658130	0.0622403	0.1189223	0.1554521
A4GNT	0.0000000	0.0025089	0.0000000	0.0000000	0.0000000
DBR1	0.0711134	0.0746532	0.0687397	0.0683347	0.0749203
ARMC8	0.2736469	0.3320848	0.3810858	0.2226066	0.4365946
NME9	0.0998288	0.0642402	0.0295849	0.0774750	0.1465983
MRAS	0.2588136	0.4308861	0.6049413	0.1830854	0.2631880
ESYT3	0.0000000	0.0032561	0.0057866	0.0000000	0.0090829
CEP70	0.3486843	0.3339487	0.3051755	0.3245016	0.3341158
FAIM	0.4435144	0.2664593	0.2667996	0.5116874	0.2483072
PIK3CB	0.3543570	0.3184097	0.2890662	0.3206821	0.4429319
MRPS22	0.3451308	0.3275310	0.3590895	0.3374058	0.2898165

ENSG00000272656	0.0000000	0.0000000	0.0025912	0.0000000	0.0000000
COPB2	0.8402262	0.6306513	0.5972198	0.8689501	0.6215849
COPB2-DT	0.2514390	0.2395288	0.2199693	0.2231657	0.5067766
RBP2	0.0010237	0.0014079	0.0012475	0.0026849	0.0038839
RBP1	1.8754603	1.7219737	1.6317510	1.7411770	1.4895236
NMNAT3	0.0936278	0.1135494	0.0934640	0.0758331	0.1403934
ENSG00000251471	0.0013128	0.0000000	0.0039720	0.0000000	0.0151379
CLSTN2	0.6544554	0.8544457	0.7522780	0.6865641	1.4310744
ENSG00000249290	0.0000000	0.0010592	0.0000000	0.0000000	0.0068983
ENSG00000289632	0.0017024	0.0000000	0.0021591	0.0033167	0.0032553
ENSG00000261826	0.0000000	0.0000000	0.0000000	0.0000000	0.0000000
SLC25A36	0.6777706	0.7786746	0.8324819	0.5867890	0.7988436
SPSB4	0.0438429	0.0614557	0.0534972	0.0648797	0.1135381
ENSG00000251270	0.0000000	0.0012774	0.0000000	0.0000000	0.0000000
PXYLP1	0.1338705	0.1762646	0.2355377	0.1580170	0.2688952
ENSG00000287155	0.0000000	0.0008870	0.0044953	0.0000000	0.0071876
ENSG00000249417	0.0004347	0.0024248	0.0032108	0.0029767	0.0288408
ZBTB38	0.4060935	0.5703338	0.7123998	0.3775672	0.6086810
ENSG00000288585	0.0004419	0.0000000	0.0024239	0.0000000	0.0000000
RASA2	0.2427590	0.2621053	0.2147628	0.1784844	0.5720159
RASA2-IT1	0.0000000	0.0013222	0.0000000	0.0000000	0.0000000
RNF7	1.1732134	1.1575587	1.1090705	1.1518057	0.9282536
GRK7	0.0024777	0.0143604	0.0072643	0.0016517	0.0388698
ATP1B3	0.5619817	0.6577821	0.7977027	0.6720700	0.6263637
ATP1B3-AS1	0.0005998	0.0000000	0.0000000	0.0000000	0.0000000
TFDP2	0.4444676	0.2876608	0.2130158	0.3648414	0.4889611
GK5	0.1276565	0.1399524	0.1134276	0.0901459	0.2447087
XRN1	0.4018488	0.4147312	0.3869955	0.3706480	0.6253563
ATR	0.1666809	0.1331421	0.1294049	0.1514477	0.3546389
ENSG00000244327	0.0000000	0.0000000	0.0000000	0.0000000	0.0013692
PLS1	0.0136050	0.0269922	0.0146981	0.0263031	0.0590317
PLS1-AS1	0.0011585	0.0000000	0.0020662	0.0022544	0.0037943
ENSG00000287045	0.0293883	0.0400982	0.0319206	0.0158025	0.2121553
TRPC1	0.2139677	0.2687500	0.2249660	0.2056502	0.5692854
PCOLCE2	0.0191003	0.0209298	0.0192337	0.0230874	0.0060245
PAQR9	0.0158256	0.0366189	0.0349931	0.0058254	0.0141046
PAQR9-AS1	0.0059775	0.0182459	0.0168530	0.0153023	0.0000000
U2SURP	0.8196893	0.7989262	0.9114265	0.8271507	0.8590020
ENSG00000268129	0.0260805	0.0254518	0.0139440	0.0127288	0.0120236
CHST2	0.0323566	0.0542693	0.0755877	0.0696927	0.0697738
ENSG00000241679	0.0000000	0.0000000	0.0000000	0.0000000	0.0017703
SLC9A9	0.0916952	0.0540919	0.0213455	0.1146955	0.1628184
DIPK2A	0.9238197	0.5635512	0.4605582	1.3746562	0.5905553
ENSG00000289986	0.0076701	0.0010401	0.0000000	0.0000000	0.0335341
PLOD2	1.3053643	0.8483207	0.6058597	1.4981549	0.9054564
ENSG00000261051	0.0095120	0.0039820	0.0027424	0.0010887	0.0141506
ENSG00000243415	0.0031172	0.0014091	0.0000000	0.0000000	0.0047971
LNCSRLR	0.0034671	0.0011398	0.0021935	0.0000000	0.0000000
PLSCR4	0.1323624	0.0520449	0.0198315	0.2296358	0.0726924
PLSCR2	0.0022101	0.0048804	0.0082805	0.0148542	0.0124070
PLSCR1	0.0496861	0.0288185	0.0208726	0.0804459	0.0405584
PLSCR5	0.0046375	0.0104080	0.0064319	0.0039723	0.0093792
ZIC1	0.0007419	0.0000000	0.0017318	0.0000000	0.0093792

ENSG00000239922	0.0000000	0.0000000	0.0000000	0.0000000	0.0000000
ENSG00000285557	0.0000000	0.0000000	0.0000000	0.0000000	0.0015298
AGTR1	0.0010461	0.0000000	0.0000000	0.0000000	0.0084944
CPB1	0.0023403	0.0052158	0.0000000	0.0114040	0.0000000
GYG1	0.3413135	0.2972369	0.2225643	0.3709766	0.2447447
HLTF	0.8040711	0.7984738	0.8055251	0.8823657	0.8011542
HPS3	0.1161303	0.1538333	0.1405914	0.1271305	0.1847297
CP	0.0030068	0.0031439	0.0025975	0.0033686	0.0000000
TM4SF1	0.0076171	0.0065355	0.0035957	0.0098619	0.0000000
WWTR1	0.8241577	0.5661053	0.4020291	0.8924665	0.5134020
WWTR1-AS1	0.0226416	0.0078883	0.0047409	0.0236673	0.0031362
COMMD2	0.4914466	0.5137360	0.5250695	0.5260944	0.3931017
ANKUB1	0.3301994	0.1924851	0.1120301	0.3136322	0.1824200
RNF13	0.6871911	0.4770283	0.3981382	0.9276960	0.5006498
PFN2	1.3757238	1.4313247	1.4945966	1.4552096	1.1721842
PFN2-AS1	0.0000000	0.0017931	0.0056490	0.0000000	0.0000000
ENSG00000243944	0.0015473	0.0020508	0.0014078	0.0010179	0.0052756
TSC22D2	0.3194971	0.3555809	0.3951983	0.2593425	0.4608354
SERP1	1.0474100	0.8272771	0.7311866	0.9930882	0.7684710
EIF2A	0.5247756	0.5292192	0.4792159	0.4703298	0.4201788
SELENOT	0.5551023	0.5551326	0.5627608	0.6937843	0.4930748
ERICH6	0.0070121	0.0007161	0.0015036	0.0021940	0.0223865
ERICH6-AS1	0.0802675	0.0796067	0.0366647	0.0839602	0.0336365
SIAH2	0.2251918	0.2353455	0.2998959	0.2072195	0.1797987
SIAH2-AS1	0.0042963	0.0038431	0.0031981	0.0000000	0.0164341
ENSG00000289005	0.0020817	0.0036448	0.0033772	0.0000000	0.0050816
CLRN1-AS1	0.0000000	0.0000000	0.0033800	0.0000000	0.0000000
MINDY4B	0.0000000	0.0000000	0.0000000	0.0000000	0.0000000
CLRN1	0.0000000	0.0011745	0.0000000	0.0000000	0.0014245
MED12L	0.1163634	0.1428147	0.1554712	0.0912542	0.3939324
GPR171	0.0002751	0.0000000	0.0000000	0.0000000	0.0033946
P2RY14	0.0000000	0.0068873	0.0051270	0.0000000	0.0395046
GPR87	0.0005733	0.0014079	0.0000000	0.0000000	0.0045236
P2RY13	0.0000000	0.0000000	0.0000000	0.0000000	0.0000000
P2RY12	0.0000000	0.0018994	0.0000000	0.0000000	0.0043064
IGSF10	0.0024783	0.0042656	0.0038728	0.0047879	0.0000000
AADACL2-AS1	0.0023199	0.0000000	0.0000000	0.0047879	0.0116132
AADACP1	0.0000000	0.0000000	0.0000000	0.0036572	0.0000000
MBNL1	0.3596459	0.3855216	0.3298564	0.3369966	0.5279815
MBNL1-AS1	0.0143191	0.0113603	0.0045126	0.0105634	0.0371103
ENSG00000243305	0.0014104	0.0057345	0.0000000	0.0000000	0.0163085
ENSG00000287706	0.0004978	0.0000000	0.0000000	0.0000000	0.0000000
P2RY1	0.0746897	0.0568477	0.0328607	0.0945670	0.0827574
ENSG00000244268	0.0013355	0.0008930	0.0000000	0.0000000	0.0000000
RAP2B	0.1893273	0.1585426	0.1743398	0.2070712	0.1006081
LINC02006	0.0054089	0.0025293	0.0020652	0.0000000	0.0000000
LINC02877	0.0011228	0.0009869	0.0000000	0.0000000	0.0000000
ARHGEF26-AS1	0.2067040	0.1253174	0.0562011	0.2378598	0.2083458
ARHGEF26	0.2891059	0.1457751	0.0967803	0.3615897	0.1266029
DHX36	1.0138153	1.0662003	1.1198561	0.9616719	1.0359970
GPR149	0.0293025	0.0513499	0.1748121	0.0156161	0.2501197
MME	0.0010592	0.0006267	0.0000000	0.0000000	0.0066349
PLCH1	0.3315640	0.2498602	0.3564136	0.3445825	0.3832988

PLCH1-AS1	0.0011758	0.0000000	0.0000000	0.0000000	0.0000000
C3orf33	0.1411151	0.1605031	0.1757812	0.1457782	0.0895024
SLC33A1	0.2253965	0.1931013	0.1943546	0.2475536	0.1900160
ENSG00000286585	0.0092865	0.0125687	0.0093306	0.0166344	0.0000000
GMPS	0.4745751	0.4346819	0.4687257	0.4989286	0.3878074
KCNAB1	0.0443879	0.0647662	0.1185521	0.0349739	0.0280597
ENSG00000287916	0.0387456	0.0343363	0.0282509	0.0378310	0.0451398
ENSG00000272990	0.0193467	0.0134001	0.0125169	0.0029905	0.0022244
SSR3	0.9489936	0.8023684	0.8140412	1.0267926	0.6513970
TIPARP-AS1	0.0156937	0.0068966	0.0073457	0.0212353	0.0197922
TIPARP	0.3417829	0.2191247	0.2060075	0.3381207	0.2229885
LINC00886	0.0435997	0.0314008	0.0297112	0.0245856	0.0458226
LEKR1	0.0393146	0.0399238	0.0321999	0.0209406	0.0355200
LINC00880	0.1339190	0.0660287	0.0257727	0.1611159	0.0821004
LINC02029	0.0045624	0.0007788	0.0060964	0.0010066	0.0062283
ENSG00000289409	0.0009967	0.0000000	0.0000000	0.0000000	0.0000000
LINC00881	0.0000000	0.0000000	0.0011166	0.0059263	0.0176773
CCNL1	0.3474552	0.2713573	0.2113500	0.2530600	0.4462010
ENSG00000241770	0.0054758	0.0015472	0.0052280	0.0000000	0.0092212
ENSG00000243176	0.0394920	0.0285282	0.0537960	0.0628236	0.1051931
VEPH1	0.0156068	0.0086257	0.0186578	0.0137553	0.0331335
PTX3	0.0252233	0.0178467	0.0478714	0.0739485	0.0494401
ENSG00000242536	0.0014681	0.0017296	0.0026358	0.0105085	0.0125915
SHOX2	0.2969726	0.5735644	1.4724409	0.2376868	0.7585829
RSRC1	0.5690974	0.5800202	0.6069792	0.5388701	0.6869256
MLF1-DT	0.0423302	0.0457649	0.0490887	0.0177579	0.0821683
MLF1	1.5888248	1.1889903	0.9586198	1.6488596	1.1429532
GFM1	0.2905947	0.3300832	0.3185783	0.3022485	0.3429525
LXN	0.0597888	0.0260450	0.0160555	0.0820161	0.0363084
RARRES1	0.0279707	0.0229627	0.0263398	0.0316202	0.0206734
MFSD1	0.1095744	0.0733808	0.0646575	0.2027841	0.1241873
ENSG00000240207	0.0084080	0.0114271	0.0157789	0.0143558	0.0148140
ENSG00000271778	0.0028963	0.0022187	0.0010787	0.0000000	0.0062535
ENSG00000272247	0.0029054	0.0019162	0.0076876	0.0000000	0.0035640
IQCJ	0.0189002	0.0172144	0.0242779	0.0177684	0.1806367
ENSG00000286913	0.0028752	0.0000000	0.0052586	0.0042900	0.0061020
SCHIP1	0.6706732	0.5759266	0.6667985	0.7243853	0.5873626
IL12A-AS1	0.0068241	0.0062998	0.0070590	0.0029693	0.0169492
IL12A	0.0066126	0.0123783	0.0176795	0.0116237	0.0216540
C3orf80	0.0197555	0.0607186	0.0480040	0.0072320	0.0179379
IFT80	0.3752048	0.2779492	0.2298210	0.3799422	0.3120469
SMC4	0.1748077	0.0956687	0.0784022	0.1705178	0.1565070
TRIM59	0.1575604	0.1267119	0.0519408	0.1522552	0.0743813
KPNA4	0.8209114	0.6804555	0.7217153	0.8279242	0.6797572
PPM1L-DT	0.0073488	0.0082232	0.0111514	0.0019800	0.0378327
PPM1L	0.3561572	0.3995328	0.4864268	0.4049672	0.6606534
B3GALNT1	0.4667491	0.5615981	0.7089872	0.5578436	0.4823522
NMD3	0.4579732	0.3727919	0.2959446	0.4298573	0.3632647
SPTSSB	0.3387850	0.2165427	0.1768262	0.4056703	0.1805108
LINC02067	0.0073376	0.0074700	0.0050553	0.0046958	0.0073722
OTOL1	0.0086475	0.0031345	0.0000000	0.0076927	0.0057809
ENSG00000288087	0.0005556	0.0023398	0.0009007	0.0027827	0.0119729
ENSG00000241168	0.0000000	0.0000000	0.0000000	0.0000000	0.0000000

ENSG00000289884	0.0985605	0.0329272	0.0344749	0.0524470	0.0398489
LINC01324	0.0018870	0.0000000	0.0000000	0.0028042	0.0042839
LINC02023	0.0000000	0.0000000	0.0000000	0.0000000	0.0000000
SLITRK3	0.0467239	0.0953974	0.1155838	0.0316949	0.0965438
LINC01322	0.1731072	0.2975504	0.3969475	0.1284601	0.5825914
BCHE	0.3062911	0.1935795	0.1432559	0.6245743	0.1737845
ZBBX	0.3719567	0.2792355	0.1681389	0.3700057	0.2880320
LINC01327	0.0000000	0.0000000	0.0000000	0.0000000	0.0019716
SERPINI2	0.0381425	0.0204268	0.0161296	0.0481374	0.0499673
WDR49	0.1619192	0.1279799	0.0614157	0.1613155	0.2377790
PDCD10	0.6031096	0.4992697	0.5363623	0.7023802	0.5425358
SERPINI1	0.3193311	0.2545369	0.5235587	0.3616806	0.3615208
ENSG00000287319	0.0000000	0.0000000	0.0000000	0.0000000	0.0000000
ENSG00000244706	0.0020728	0.0000000	0.0015424	0.0017126	0.0052401
GOLIM4	1.3099994	0.8167747	0.6004368	1.6190866	0.8532675
ENSG00000286994	0.0000000	0.0041093	0.0020693	0.0088803	0.0000000
ENSG00000241882	0.0000000	0.0000000	0.0011270	0.0000000	0.0032853
LINC02082	0.0125326	0.0103285	0.0073767	0.0127558	0.0697566
ENSG00000273177	0.0016207	0.0009239	0.0000000	0.0000000	0.0000000
LINC01997	0.0000000	0.0000000	0.0000000	0.0000000	0.0000000
MECOM	0.0276625	0.0393324	0.0267414	0.0106293	0.0293914
ACTRT3	0.0049211	0.0065538	0.0052883	0.0106877	0.0037728
ENSG00000269889	0.0026647	0.0035220	0.0000000	0.0000000	0.0021475
MYNN	0.1468081	0.1858449	0.1284753	0.1217578	0.1928123
ENSG00000269984	0.0041452	0.0006684	0.0000000	0.0000000	0.0000000
LRRC34	0.1762686	0.1103450	0.0743212	0.2491674	0.1026606
ENSG00000270135	0.0049100	0.0025496	0.0006897	0.0092038	0.0094238
ENSG00000270096	0.0090953	0.0044599	0.0049752	0.0039108	0.0101868
LRRIQ4	0.0028958	0.0000000	0.0015418	0.0062957	0.0000000
LRRC31	0.0000000	0.0025247	0.0019529	0.0000000	0.0000000
FHL1P1	0.0031344	0.0011057	0.0062443	0.0059384	0.0083967
SEC62	1.7610110	1.6969656	1.8609942	1.8208351	1.5082690
SEC62-AS1	0.0005026	0.0000000	0.0000000	0.0000000	0.0022413
GPR160	0.0040100	0.0055677	0.0000000	0.0019429	0.0085295
PHC3	0.2661269	0.2673514	0.2621528	0.2020156	0.3789142
PRKCI	0.3678218	0.5151042	0.5060340	0.3484430	0.4256014
ENSG00000239628	0.0000000	0.0000000	0.0000000	0.0000000	0.0000000
SKIL	0.4943531	0.5313838	0.6213058	0.5166519	0.6712914
ENSG00000242578	0.0000000	0.0000000	0.0000000	0.0000000	0.0014182
CLDN11	0.0060270	0.0051516	0.0000000	0.0000000	0.0031970
SLC7A14-AS1	0.0102505	0.0042503	0.0093001	0.0000000	0.0126829
SLC7A14	0.0839878	0.1817643	0.2373110	0.0505247	0.2701174
RPL22L1	0.3486508	0.2860444	0.1943964	0.3491426	0.2363661
EIF5A2	0.1122474	0.0934592	0.0998706	0.1144018	0.0952547
ENSG00000286856	0.0052928	0.0057988	0.0029702	0.0000000	0.0282203
SLC2A2	0.0032292	0.0000000	0.0000000	0.0027681	0.0100073
TNIK	0.9785477	0.8849044	0.8958533	0.9380469	1.3100748
ENSG00000240497	0.0819915	0.0667806	0.0582896	0.1170132	0.0700985
PLD1	0.0504310	0.0303614	0.0459927	0.0447561	0.0613702
TMEM212-AS1	0.0000000	0.0000000	0.0023101	0.0000000	0.0031362
TMEM212	0.0016211	0.0000000	0.0000000	0.0017126	0.0064560
FNDC3B	0.8345430	0.5614540	0.3682808	0.8097899	0.8141518
GHSR	0.0091627	0.0158748	0.0056891	0.0024744	0.0246475

TNFSF10	0.0061115	0.0055398	0.0000000	0.0189687	0.0000000
LINC02068	0.0000000	0.0000000	0.0000000	0.0000000	0.0000000
NCEH1	0.0266985	0.0226362	0.0385937	0.0573931	0.0729916
ENSG00000287012	0.0000000	0.0016378	0.0000000	0.0000000	0.0026726
ECT2	0.0630199	0.0544490	0.0271013	0.0654643	0.0765846
SPATA16	0.0033721	0.0010809	0.0000000	0.0000000	0.0031362
ENSG00000237473	0.0020092	0.0000000	0.0000000	0.0000000	0.0000000
NLGN1	0.7174960	0.8055165	0.6990761	0.6727177	2.1217843
ENSG00000289417	0.0011133	0.0035142	0.0023062	0.0000000	0.0185379
NLGN1-AS1	0.0000000	0.0007627	0.0023230	0.0000000	0.0037236
NAALADL2	0.1672220	0.1158673	0.0815307	0.1203656	0.3340883
NAALADL2-AS2	0.0017551	0.0013468	0.0031505	0.0000000	0.0000000
LINC01208	0.0111748	0.0129629	0.0224340	0.0084688	0.0148465
TBL1XR1	0.7835084	0.8337732	0.9636093	0.6811535	1.0415694
TBL1XR1-AS1	0.0017178	0.0000000	0.0000000	0.0000000	0.0000000
LINC00501	0.0003069	0.0025990	0.0000000	0.0000000	0.0048241
ENSG00000226782	0.0002418	0.0079864	0.0055066	0.0065973	0.0148576
LINC00578	0.0075609	0.0035453	0.0083450	0.0078945	0.0207398
ENSG00000286003	0.0000000	0.0000000	0.0000000	0.0000000	0.0031362
ENSG00000223930	0.0000000	0.0000000	0.0000000	0.0079401	0.0057461
KCNMB2	0.2067974	0.1422991	0.2147727	0.1703248	0.3561387
LINC01014	0.0353570	0.0208231	0.0249538	0.0483922	0.0108554
KCNMB2-AS1	0.0305010	0.0315754	0.0292197	0.0241492	0.0535496
ZMAT3	0.4252150	0.3050680	0.2800626	0.4583347	0.3028998
PIK3CA-DT	0.0007786	0.0011107	0.0000000	0.0050414	0.0151833
PIK3CA	0.3862326	0.4569494	0.4926389	0.3448903	0.5462788
KCNMB3	0.0354655	0.0362098	0.0374096	0.0403912	0.0649338
ZNF639	0.3598738	0.3651165	0.3607255	0.3674013	0.3395596
ENSG00000260743	0.0066944	0.0032283	0.0000000	0.0031443	0.0000000
MFN1	0.3232849	0.2894025	0.2798629	0.2984482	0.3757163
ENSG00000289574	0.0164476	0.0176263	0.0178819	0.0120286	0.0916946
GNB4	0.4779786	0.4515062	0.3526592	0.4899150	0.4368906
ENSG00000242539	0.0109766	0.0093759	0.0040654	0.0133444	0.0078259
ENSG00000272699	0.0000000	0.0000000	0.0000000	0.0000000	0.0000000
ACTL6A	0.2412059	0.1794289	0.1129827	0.2760718	0.1507149
ENSG00000272910	0.0000000	0.0024998	0.0011917	0.0000000	0.0000000
MRPL47	0.5382313	0.5864519	0.5760957	0.5546741	0.4475780
NDUFB5	0.9793290	0.9397155	0.8898861	1.1043791	0.7595095
USP13	0.1068188	0.1456120	0.1106987	0.1033792	0.2026600
PEX5L	0.0842510	0.1250131	0.1389640	0.0883048	0.3893843
PEX5L-AS2	0.0006829	0.0000000	0.0000000	0.0000000	0.0000000
ENSG00000287645	0.0008186	0.0000000	0.0000000	0.0000000	0.0017223
LINC02053	0.0000000	0.0000000	0.0000000	0.0000000	0.0000000
TTC14-DT	0.0041309	0.0050269	0.0000000	0.0000000	0.0074937
TTC14	0.2161907	0.2482732	0.1807664	0.2011913	0.4126675
CCDC39	0.4705930	0.2514584	0.1313619	0.4537314	0.1889074
CCDC39-AS1	0.0056575	0.0023960	0.0000000	0.0062009	0.0000000
FXR1	1.7141771	1.4764391	1.4043313	1.6230911	1.4230058
DNAJC19	0.5655064	0.5388159	0.5411897	0.6053593	0.4194872
SOX2-OT	0.5593436	0.4521710	0.5155424	0.7165364	0.4861056
ENSG00000241231	0.0000000	0.0008866	0.0000000	0.0000000	0.0000000
ENSG00000239381	0.0033658	0.0060196	0.0000000	0.0075460	0.0145790
ENSG00000289435	0.0033194	0.0033925	0.0000000	0.0032175	0.0039795

SOX2	1.1589633	0.6950331	0.5361657	1.3232845	0.5330065
LINC01206	0.0017408	0.0041131	0.0000000	0.0000000	0.0000000
ATP11B-DT	0.0000000	0.0005008	0.0000000	0.0000000	0.0083759
ATP11B	0.2031998	0.2097480	0.1902512	0.1320241	0.4549890
ENSG00000273261	0.0000000	0.0035728	0.0000000	0.0000000	0.0000000
DCUN1D1	0.3479621	0.3258384	0.3493641	0.3576186	0.3886079
MCCC1	0.0936707	0.1042631	0.0936186	0.1328615	0.1792672
LAMP3	0.0327399	0.0280877	0.0182982	0.0187068	0.0226122
MCF2L2	0.1457535	0.1994379	0.1852467	0.1073643	0.5920489
B3GNT5	0.2810289	0.1370905	0.1050960	0.2889158	0.1461518
ENSG00000289859	0.0000000	0.0000000	0.0000000	0.0000000	0.0000000
LINC00888	0.3115321	0.3000612	0.4953223	0.2900058	0.3814571
KLHL6	0.0012488	0.0026649	0.0042275	0.0000000	0.0000000
KLHL24	0.4315271	0.3782759	0.3149717	0.3765153	0.5047740
YEATS2	0.3047806	0.3192688	0.2945801	0.2881563	0.6748798
YEATS2-AS1	0.0000000	0.0000000	0.0053378	0.0000000	0.0000000
MAP6D1	0.0274627	0.0665643	0.0852338	0.0344298	0.0455642
PARL	0.2799267	0.2338257	0.2459667	0.3141625	0.2542434
ABCC5	0.1305030	0.1728847	0.1302596	0.1178461	0.3270272
ABCC5-AS1	0.0000000	0.0000000	0.0000000	0.0000000	0.0000000
HTR3E-AS1	0.0000000	0.0000000	0.0000000	0.0000000	0.0038284
HTR3E	0.0000000	0.0000000	0.0000000	0.0000000	0.0047101
ENSG00000273181	0.0010174	0.0027337	0.0000000	0.0028496	0.0000000
EIF2B5-DT	0.0015386	0.0050764	0.0077680	0.0053070	0.0093747
EIF2B5	0.1740960	0.1818231	0.1714487	0.1015774	0.1592286
DVL3	0.3887198	0.3857431	0.4380455	0.3745121	0.2838045
AP2M1	0.9696284	1.1675365	1.3940413	1.0003925	0.9210634
ABCF3	0.1379163	0.1083486	0.1232715	0.1594750	0.0912701
VWA5B2	0.0113650	0.0125887	0.0186812	0.0066543	0.0160696
ALG3	0.1644629	0.1361902	0.1233237	0.1571145	0.0864204
EEF1AKMT4	0.0113359	0.0197895	0.0340667	0.0133579	0.0156091
CAMK2N2	0.3850112	0.7665476	0.9251882	0.3610828	0.5557549
ECE2	0.0095344	0.0264270	0.0046017	0.0146946	0.0231674
PSMD2	0.7848648	0.7242222	0.6980543	0.8343497	0.6056279
EIF4G1	0.5158781	0.5313193	0.5084707	0.6195643	0.4859984
FAM131A	0.2768455	0.2591857	0.3175149	0.3115784	0.2479421
CLCN2	0.0339850	0.0337489	0.0351304	0.0459957	0.0745073
POLR2H	0.5426259	0.4593681	0.4094514	0.5614413	0.3793017
THPO	0.0003449	0.0000000	0.0000000	0.0000000	0.0000000
CHRD	0.0265594	0.0292970	0.0209790	0.0264322	0.0512931
LINC02054	0.0000000	0.0000000	0.0000000	0.0000000	0.0093214
EPHB3	0.0076527	0.0210563	0.0135027	0.0261665	0.0421565
MAGEF1	0.5554213	0.4839976	0.6022034	0.5969518	0.3872520
ENSG00000272970	0.1253124	0.0932355	0.0774983	0.0852363	0.0964603
LINC02069	0.0018111	0.0020933	0.0000000	0.0000000	0.0000000
ENSG00000273403	0.0034396	0.0000000	0.0000000	0.0021832	0.0000000
ENSG00000272922	0.0022313	0.0028049	0.0122013	0.0103873	0.0095779
VPS8	0.2648674	0.3407077	0.3258215	0.2439723	0.5947371
C3orf70	0.1082546	0.1439659	0.1486429	0.1386560	0.1332645
EHHADH	0.0448718	0.0223421	0.0416356	0.0383322	0.0765008
MAP3K13	0.7275336	0.8380998	0.7606253	0.7438838	0.8651616
TMEM41A	0.1060146	0.1208742	0.1125460	0.1808828	0.1179150
ENSG00000286086	0.0029054	0.0021664	0.0000000	0.0033707	0.0059207

LIPH	0.0000000	0.0000000	0.0025063	0.0022040	0.0040955
SENP2	0.4999792	0.4722287	0.4427652	0.4543739	0.4040635
IGF2BP2	0.6962171	0.5918245	0.4850792	0.5621972	0.9775359
IGF2BP2-AS1	0.0012144	0.0009429	0.0000000	0.0000000	0.0027453
TRA2B	0.6996742	0.7414884	0.7268655	0.6539202	0.7279778
NMRAL2P	0.0000000	0.0000000	0.0000000	0.0000000	0.0000000
ETV5	0.0768350	0.0474583	0.0233372	0.0956898	0.0622162
DGKG	0.1312088	0.0948402	0.1034839	0.1187100	0.2680157
CRYGS	0.0056538	0.0067836	0.0050945	0.0000000	0.0114424
TBCCD1	0.0484528	0.0447702	0.0238801	0.0441324	0.0625258
DNAJB11	0.4445531	0.3449556	0.3216257	0.5394806	0.2771709
HRG-AS1	0.0000000	0.0017155	0.0000000	0.0000000	0.0051058
AHSG	0.0000000	0.0000000	0.0009691	0.0000000	0.0000000
ENSG00000263826	0.0041128	0.0032312	0.0000000	0.0063474	0.0031362
EIF4A2	1.8262782	1.8103035	1.7795262	1.7498966	1.5614661
RFC4	0.1233452	0.1417930	0.1639409	0.1264904	0.1309923
ENSG00000231724	0.0000000	0.0005304	0.0000000	0.0000000	0.0000000
LINC02043	0.0000000	0.0044709	0.0000000	0.0000000	0.0097464
ENSG00000289971	0.0121850	0.0265472	0.0399860	0.0056629	0.0250620
ST6GAL1	0.0763629	0.1273876	0.1107161	0.0421353	0.3434374
RPL39L	0.1636294	0.2542467	0.2399710	0.1517289	0.1769564
RTP1	0.0079622	0.0118599	0.0276701	0.0050046	0.0100399
MASP1	0.0504831	0.0832280	0.1577511	0.0710667	0.0638498
ENSG00000283175	0.0025889	0.0053219	0.0064278	0.0000000	0.0000000
RTP4	0.0018226	0.0000000	0.0000000	0.0000000	0.0000000
SST	0.7604346	1.5248844	0.9107443	0.7772380	1.1983492
BCL6	0.0879507	0.0669907	0.0564399	0.0661923	0.1681497
ENSG00000285938	0.0000000	0.0011481	0.0000000	0.0000000	0.0000000
ENSG00000290021	0.0015700	0.0022060	0.0009691	0.0000000	0.0000000
ENSG00000289331	0.0025955	0.0011997	0.0040931	0.0015981	0.0090548
LPP-AS2	0.0155315	0.0082254	0.0044019	0.0033686	0.0000000
LPP	0.3480227	0.2988816	0.2445821	0.3099086	0.7171692
ENSG00000288794	0.0000000	0.0018923	0.0015130	0.0041781	0.0065039
TPRG1-AS1	0.0014034	0.0008497	0.0000000	0.0000000	0.0000000
TPRG1	0.0039353	0.0096997	0.0024140	0.0021816	0.0058044
TP63	0.0006670	0.0036484	0.0000000	0.0000000	0.0152656
P3H2	0.0602180	0.0383949	0.0170888	0.0948864	0.1031660
P3H2-AS1	0.0257801	0.0351861	0.0225507	0.0118669	0.0251480
CLDN1	0.0248634	0.0119372	0.0278996	0.0218017	0.0313527
CLDN16	0.0031888	0.0062461	0.0062722	0.0000000	0.0323616
IL1RAP	0.0304244	0.0264272	0.0335075	0.0838568	0.1270409
GMNC	0.0203808	0.0061043	0.0052108	0.0041116	0.0039851
OSTN	0.0000000	0.0009079	0.0009140	0.0000000	0.0000000
UTS2B	0.0019788	0.0055048	0.0022052	0.0065024	0.0271322
CCDC50	0.6404123	0.7585080	0.9888667	0.5790740	0.6934786
PYDC2-AS1	0.0112591	0.0085345	0.0076806	0.0013619	0.0236628
ENSG00000287178	0.0000000	0.0000000	0.0000000	0.0000000	0.0000000
FGF12	0.6305581	0.9211950	0.9071495	0.6345675	1.3761619
FGF12-AS1	0.0027398	0.0000000	0.0000000	0.0056441	0.0042773
FGF12-AS3	0.0064456	0.0037466	0.0014297	0.0078577	0.0000000
ENSG00000289165	0.0000000	0.0005488	0.0000000	0.0000000	0.0085602
MB21D2	0.1274156	0.2059429	0.1937013	0.0945803	0.3387157
PLAAT1	0.0656911	0.1557200	0.1167391	0.0676329	0.1387944

ATP13A5	0.0000000	0.0009204	0.0025766	0.0000000	0.0000000
ATP13A5-AS1	0.0000000	0.0038565	0.0000000	0.0000000	0.0061768
ATP13A4	0.0241032	0.0158633	0.0184157	0.0267517	0.0411213
OPA1	0.5237037	0.6835105	0.7643236	0.5581674	0.5548819
OPA1-AS1	0.0007626	0.0000000	0.0000000	0.0043946	0.0044318
LINC02026	0.0059011	0.0030727	0.0105322	0.0040935	0.0031362
ENSG00000238043	0.0206114	0.0070178	0.0084424	0.0188149	0.0000000
LINC02028	0.0100311	0.0091122	0.0115607	0.0048214	0.0247327
ENSG00000289385	0.0010385	0.0017500	0.0000000	0.0000000	0.0073247
HES1	1.0421598	0.6075005	0.3925385	1.0008780	0.5076649
LINC02036	0.0015291	0.0079774	0.0107606	0.0035629	0.0031362
LINC00887	0.0020836	0.0000000	0.0000000	0.0017847	0.0039740
ATP13A3	0.3298036	0.2922444	0.2852284	0.3512988	0.5036097
ATP13A3-DT	0.0217217	0.0454569	0.0617262	0.0252746	0.0777366
TMEM44-AS1	0.0606743	0.0400489	0.0203568	0.0568694	0.0400714
ENSG00000288979	0.0002744	0.0000000	0.0000000	0.0065551	0.0000000
TMEM44	0.0401541	0.0767323	0.0832251	0.0652131	0.1630084
TMEM44-AS2	0.0096557	0.0113234	0.0104442	0.0049686	0.0161925
ENSG00000272707	0.0030934	0.0021358	0.0000000	0.0012960	0.0061195
LSG1	0.2541920	0.2239766	0.2265235	0.2757165	0.2575165
FAM43A	0.0548239	0.0455139	0.0381019	0.0438192	0.0257176
LINC01968	0.0023204	0.0000000	0.0026961	0.0000000	0.0225886
XXYLT1	0.3424104	0.2707127	0.2599205	0.3310628	0.3669064
ENSG00000287005	0.0022814	0.0047026	0.0000000	0.0000000	0.0000000
ACAP2	0.5480710	0.4673771	0.4115286	0.5308310	0.6132466
ACAP2-IT1	0.0037809	0.0090768	0.0040495	0.0000000	0.0094123
PPP1R2	0.7551762	0.7728185	0.8482264	0.7835117	0.6324544
ENSG00000223711	0.0000000	0.0000000	0.0000000	0.0000000	0.0000000
APOD	0.0121032	0.0078991	0.0041539	0.0097000	0.0070107
ENSG00000289929	0.0057869	0.0018352	0.0063308	0.0042547	0.0000000
ENSG00000229178	0.0133411	0.0226266	0.0370364	0.0103665	0.0014252
MUC20	0.0233671	0.0326434	0.0317906	0.0155936	0.1448604
MUC4	0.0003563	0.0019683	0.0000000	0.0000000	0.0061370
LINC01983	0.0005933	0.0028876	0.0000000	0.0083622	0.0146300
TNK2	0.0612380	0.0648300	0.0706627	0.0291991	0.1645259
ENSG00000286004	0.0012311	0.0013171	0.0000000	0.0000000	0.0034957
TNK2-AS1	0.0000000	0.0000000	0.0000000	0.0000000	0.0000000
ENSG00000290762	0.0208037	0.0159198	0.0116977	0.0146927	0.0616646
SDHAP1	0.0925286	0.0853690	0.0817837	0.1012289	0.1882214
TFRC	0.2321849	0.2093318	0.2274505	0.2983743	0.2405744
ZDHHC19	0.0021795	0.0028055	0.0076070	0.0000000	0.0082021
SLC51A	0.0024868	0.0036506	0.0019603	0.0124516	0.0042796
PCYT1A	0.1867323	0.1436763	0.0998857	0.1696717	0.1982936
DYNLT2B	0.9146953	0.8186774	0.8925872	0.8833551	0.6789502
TM4SF19-AS1	0.0034471	0.0118216	0.0105090	0.0099271	0.0031362
TM4SF19	0.0000000	0.0018009	0.0000000	0.0000000	0.0041053
UBXN7	0.5075553	0.5491496	0.6338913	0.4960577	0.5766290
UBXN7-AS1	0.0004851	0.0000000	0.0000000	0.0000000	0.0000000
RNF168	0.3105984	0.3088806	0.3275744	0.3056319	0.3101544
ENSG00000273013	0.0047999	0.0059838	0.0032780	0.0078380	0.0000000
SMCO1	0.0004682	0.0003924	0.0000000	0.0000000	0.0026227
WDR53	0.0724088	0.0530907	0.0490924	0.0684188	0.0602197
FBXO45	0.2670607	0.3647735	0.4297821	0.2677461	0.2438183

LINC01063	0.0275838	0.0164725	0.0225536	0.0385105	0.0240999
PIGX	0.4491627	0.4976984	0.5723197	0.5314191	0.4073148
CEP19	0.2721232	0.3313728	0.4048157	0.3188278	0.2163132
PAK2	1.4965794	1.3329742	1.3827708	1.4586826	1.1980601
ENSG00000289446	0.0061777	0.0031881	0.0025364	0.0000000	0.0000000
SENP5	0.3567721	0.3645362	0.3921376	0.3299867	0.4728900
ENSG00000230732	0.0022564	0.0051545	0.0033550	0.0038410	0.0145067
NCBP2	0.7047224	0.6542126	0.7826079	0.7203371	0.5269240
NCBP2-AS1	0.0017303	0.0000000	0.0000000	0.0000000	0.0000000
NCBP2AS2	0.2550828	0.2685962	0.2411433	0.2527727	0.2064073
PIGZ	0.0540110	0.0748841	0.0734771	0.0746620	0.1209442
ENSG00000286017	0.0023705	0.0011890	0.0000000	0.0000000	0.0000000
MELTF	0.0089011	0.0244944	0.0193048	0.0069876	0.0207811
MELTF-AS1	0.1234904	0.1300742	0.1325568	0.1562908	0.1191451
DLG1	0.4070072	0.3600781	0.3350630	0.3514205	0.5768266
ENSG00000289396	0.0000000	0.0014300	0.0049372	0.0036480	0.0069313
DLG1-AS1	0.0007103	0.0012444	0.0000000	0.0000000	0.0044801
ENSG00000286870	0.0000000	0.0000000	0.0000000	0.0000000	0.0000000
ENSG00000289828	0.0000000	0.0007057	0.0027983	0.0000000	0.0000000
LINC02012	0.0008804	0.0000000	0.0000000	0.0000000	0.0000000
BDH1	0.0373873	0.0683536	0.0786911	0.0314252	0.0941481
SDHAP4	0.1257312	0.2057176	0.2020536	0.1318643	0.3120483
ENSG00000273308	0.0033494	0.0049804	0.0036178	0.0000000	0.0064671
ENSG00000286909	0.0053260	0.0074466	0.0044864	0.0035645	0.0177343
ENSG00000236833	0.0012933	0.0040218	0.0000000	0.0042829	0.0000000
ENSG00000287286	0.0224500	0.0406444	0.0962955	0.0381602	0.0553794
RUBCN	0.1556018	0.2895324	0.3788412	0.1599732	0.3016826
FYTTD1	0.8580347	0.9113283	1.0399801	0.8450493	0.8067637
ENSG00000273375	0.0029419	0.0065443	0.0018068	0.0000000	0.0029373
LRCH3	0.2094600	0.1626916	0.1539214	0.2135139	0.4270568
IQCG	0.7339975	0.4738771	0.3586770	0.7571796	0.4755630
RPL35A	2.9413573	2.9043370	2.7413362	2.8699107	2.6686908
LMLN	0.1118897	0.0711196	0.0821360	0.1297837	0.1125413
ENSG00000286755	0.0000000	0.0014562	0.0000000	0.0000000	0.0000000
ANKRD18DP	0.0034008	0.0030216	0.0137933	0.0000000	0.0099000
ENSG00000290937	0.0011007	0.0010483	0.0025528	0.0000000	0.0046690
ZNF595	0.0933099	0.1104298	0.0745449	0.1243747	0.1134626
ENSG00000289361	0.0073715	0.0226986	0.0082206	0.0125909	0.0158809
ZNF718	0.2393465	0.3416654	0.2934058	0.1942877	0.3698999
ENSG00000275426	0.0046269	0.0039884	0.0033744	0.0066059	0.0000000
ZNF876P	0.0355531	0.0441657	0.0599029	0.0275082	0.0956634
ZNF732	0.0004431	0.0036495	0.0000000	0.0000000	0.0054224
ENSG00000286705	0.0797373	0.0851000	0.0826426	0.0669298	0.0839853
ZNF141	0.1595858	0.1758009	0.2169444	0.1230152	0.2490523
ENSG00000281016	0.0039309	0.0039028	0.0061774	0.0032068	0.0159595
ENSG00000272885	0.0017605	0.0026147	0.0000000	0.0039507	0.0023002
ZNF721	0.4084108	0.3969717	0.3969611	0.3840858	0.4822420
PIGG	0.0566036	0.0494139	0.0596828	0.0785864	0.1393122
TMEM271	0.0436991	0.0982823	0.0905170	0.0388000	0.0801603
ENSG00000283183	0.0747466	0.1555123	0.2535850	0.0503941	0.1661328
PDE6B	0.1104464	0.0730384	0.0412326	0.1205706	0.0579611
PDE6B-AS1	0.0071371	0.0108757	0.0047541	0.0162368	0.0421826
ENSG00000272927	0.0869312	0.0534611	0.0514097	0.1176711	0.0058914

ATP5ME	1.1922060	1.1832816	1.1865724	1.2093398	0.9507662
MYL5	0.1687797	0.1199388	0.0944520	0.1517814	0.1171341
PCGF3	0.2816666	0.3889612	0.4470695	0.2740884	0.3465723
ENSG00000272588	0.0027601	0.0000000	0.0000000	0.0000000	0.0000000
PCGF3-AS1	0.1320607	0.1614214	0.1518097	0.1371201	0.3118381
CPLX1	0.1089675	0.2651696	0.1467014	0.0784447	0.2300833
ENSG00000289983	0.0008382	0.0000000	0.0007553	0.0000000	0.0000000
GAK	0.2094143	0.1832333	0.1651552	0.1654090	0.3686471
TMEM175	0.1849013	0.1602844	0.1720777	0.2383021	0.2172588
DGKQ	0.0179604	0.0173356	0.0343776	0.0332839	0.0341456
SLC26A1	0.0156466	0.0214138	0.0092516	0.0031006	0.0059167
IDUA	0.0291598	0.0092919	0.0089259	0.0441784	0.0474434
FGFRL1	0.1211355	0.0567301	0.0242144	0.0965588	0.0504687
RNF212	0.0361402	0.0495749	0.0677104	0.0315249	0.0651168
ENSG00000251652	0.0121857	0.0208649	0.0553531	0.0425449	0.0763544
ENSG00000227189	0.0000000	0.0000000	0.0000000	0.0000000	0.0031804
SPON2	0.0181933	0.0161163	0.0107570	0.0148551	0.0352241
ENSG00000273179	0.0014243	0.0083613	0.0000000	0.0107571	0.0000000
CTBP1-AS	0.0018248	0.0034575	0.0071600	0.0077981	0.0044624
CTBP1	0.5577384	0.6189673	0.7149187	0.5210572	0.6417415
CTBP1-DT	0.1066150	0.1405391	0.1290109	0.1142980	0.1162318
MAEA	0.2916277	0.2723229	0.3255528	0.3292716	0.3705777
UVSSA	0.0732640	0.0559518	0.0627584	0.0484123	0.1541337
NKX1-1	0.0200155	0.0175229	0.0549267	0.0056477	0.0267925
FAM53A	0.0000000	0.0029329	0.0000000	0.0000000	0.0080520
SLBP	0.3328617	0.4029845	0.5244529	0.4284470	0.2844915
TACC3	0.0216105	0.0332159	0.0260490	0.0180885	0.0412970
TMEM129	0.2262560	0.1391733	0.1155075	0.2861686	0.0959780
FGFR3	0.0848231	0.0448923	0.0234720	0.2136516	0.0370651
LETM1	0.1515503	0.1890291	0.2822188	0.1494598	0.2698127
NSD2	0.6313501	0.8062013	0.9155805	0.5563009	0.9479136
NELFA	0.3463058	0.2976733	0.2773840	0.3619363	0.4305188
C4orf48	1.2596729	1.4954529	1.5395394	1.2857892	1.1768905
NAT8L	0.1246681	0.1393514	0.0731679	0.1543954	0.1318867
POLN	0.0490073	0.0382790	0.0245171	0.0527758	0.1068526
HAUS3	0.1421598	0.1167497	0.1526878	0.1182543	0.1475573
MXD4	0.3997022	0.3117131	0.3133896	0.4238915	0.2328923
ZFYVE28	0.0227367	0.0496904	0.0428303	0.0346811	0.1986840
ENSG00000251148	0.0000000	0.0000000	0.0000000	0.0000000	0.0138820
CFAP99	0.0263217	0.0126117	0.0028098	0.0563310	0.0324045
RNF4	0.2659964	0.2242066	0.1449213	0.2118575	0.1578811
FAM193A	0.2356166	0.2652148	0.2203830	0.2250810	0.6747491
TNIP2	0.1114678	0.1158634	0.1045071	0.1136501	0.0919720
SH3BP2	0.1570652	0.1401009	0.1699649	0.1684896	0.1958576
ADD1	0.8018917	0.7073954	0.7661237	0.7835374	0.8001218
ENSG00000287099	0.0000000	0.0000000	0.0000000	0.0000000	0.0000000
MFSD10	0.4768210	0.3662775	0.3368282	0.5881279	0.3571360
NOP14-AS1	0.0948630	0.0824676	0.0818848	0.0924559	0.0914480
NOP14	0.1949018	0.1901617	0.1576457	0.1794530	0.2042589
GRK4	0.2257795	0.3211531	0.3785802	0.1939992	0.4216848
HTT	0.2567968	0.2922081	0.2270086	0.2160598	0.4996277
HTT-AS	0.0025204	0.0010081	0.0027739	0.0094542	0.0070307
MSANTD1	0.0714304	0.1529270	0.1792084	0.0397988	0.1159820

RGS12	0.3035830	0.2339940	0.1864744	0.3191112	0.3502250
HGFAC	0.0044405	0.0075485	0.0040992	0.0057889	0.0000000
ENSG00000286900	0.0006698	0.0000000	0.0000000	0.0000000	0.0000000
DOK7	0.0051623	0.0023540	0.0000000	0.0000000	0.0000000
LRPAP1	0.7298266	0.4786917	0.4915433	1.0214593	0.4999274
LINC02600	0.0014773	0.0034026	0.0020019	0.0000000	0.0071536
ADRA2C	0.0126491	0.0252037	0.0341849	0.0179091	0.0044810
FAM86EP	0.0395762	0.0267449	0.0301959	0.0332911	0.0190362
ENSG00000290888	0.0311589	0.0390565	0.0139364	0.0322149	0.0533713
TMEM128	0.3609889	0.3121032	0.3499823	0.4298079	0.2616128
ENSG00000289032	0.0050774	0.0000000	0.0017966	0.0018326	0.0000000
LYAR	0.1860534	0.1444753	0.1279650	0.1828834	0.1038807
ZBTB49	0.0199025	0.0301342	0.0229227	0.0385456	0.0627035
ENSG00000248516	0.0012343	0.0000000	0.0000000	0.0000000	0.0022383
NSG1	0.8588964	1.4025180	1.7484839	0.8176846	1.0963807
STX18	0.1878802	0.1582430	0.1061021	0.1587944	0.2668357
STX18-AS1	0.0659135	0.0924744	0.0781023	0.0635797	0.3422237
LINC01396	0.0012114	0.0000000	0.0000000	0.0000000	0.0032430
MSX1	0.0629540	0.0338885	0.0295424	0.0666390	0.0279003
CYTL1	0.1166427	0.0592604	0.0364889	0.3402262	0.0882712
STK32B	0.0739846	0.0571424	0.0752307	0.0514597	0.2171543
LINC01587	0.0000000	0.0000000	0.0000000	0.0000000	0.0000000
EVC2	0.0124427	0.0035932	0.0055059	0.0297765	0.0277083
EVC	0.0907876	0.0525735	0.0397755	0.1165343	0.0678986
CRMP1	0.9063108	1.4453505	1.7856805	0.7895652	1.2606241
ENSG00000289414	0.0152954	0.0218597	0.0372898	0.0054968	0.0028791
C4orf50	0.0096901	0.0117271	0.0075487	0.0000000	0.0945374
JAKMIP1	0.0936692	0.2124991	0.2908549	0.0755013	0.1138781
ENSG00000287786	0.0000000	0.0000000	0.0000000	0.0000000	0.0000000
ENSG00000290803	0.0204909	0.0494590	0.0605337	0.0283435	0.0434775
ENSG00000288588	0.0009498	0.0031472	0.0019687	0.0000000	0.0000000
WFS1	0.1265364	0.0681699	0.0767760	0.1843105	0.0462230
ENSG00000286176	0.0012386	0.0053498	0.0031857	0.0000000	0.0000000
PPP2R2C	0.3040106	0.5719159	0.6676053	0.2835450	0.6486896
MAN2B2	0.0516599	0.0492749	0.0546743	0.1104688	0.0612191
MRFAP1	1.5373328	1.4022847	1.4654659	1.5014377	1.1335190
LINC02482	0.1605633	0.1717807	0.1553621	0.1797258	0.1401405
ENSG00000170846	0.1734567	0.2241326	0.2240690	0.1555262	0.1210880
S100P	0.0096836	0.0063706	0.0000000	0.0000000	0.0000000
MRFAP1L1	0.5700864	0.6493847	0.7672761	0.5611235	0.5098624
BLOC1S4	0.2818748	0.2784861	0.3033386	0.3386795	0.1798607
KIAA0232	0.7573651	0.7390193	0.7724297	0.7184216	0.8274904
TBC1D14	0.3507922	0.3977289	0.3521048	0.3379459	0.3384269
ENSG00000287104	0.0731848	0.0642376	0.0722649	0.0313374	0.0818326
ENSG00000245748	0.0076785	0.0035965	0.0076191	0.0009264	0.0145981
CCDC96	0.1427740	0.0936038	0.0600802	0.1944647	0.0706174
TADA2B	0.0912497	0.0879794	0.0932597	0.0946785	0.0817296
GRPEL1	0.3534982	0.3393576	0.3898816	0.3290934	0.2374366
LINC02447	0.0405816	0.0174649	0.0115805	0.0247793	0.0330421
SORCS2	0.0516526	0.0863111	0.0271233	0.0637996	0.3737580
AFAP1-AS1	0.0129853	0.0153762	0.0226096	0.0021304	0.0150468
AFAP1	0.3733139	0.4164210	0.4626269	0.3947062	0.5180915
ENSG00000287164	0.0000000	0.0000000	0.0000000	0.0000000	0.0045621

ABLIM2	0.1243545	0.1410832	0.1525516	0.0866860	0.4596918
ENSG00000290015	0.0224215	0.0236593	0.0145703	0.0101738	0.0281293
SH3TC1	0.0084040	0.0066309	0.0023468	0.0076590	0.0000000
HTRA3	0.0051258	0.0034560	0.0000000	0.0063605	0.0000000
ENSG00000251615	0.0104900	0.0234655	0.0154102	0.0108835	0.0296734
ACOX3	0.0917290	0.0883407	0.0612354	0.1130304	0.2254974
TRMT44	0.0410649	0.0403812	0.0391075	0.0179039	0.0703112
ENSG00000205959	0.0078370	0.0073281	0.0026803	0.0000000	0.0417782
GPR78	0.0016124	0.0000000	0.0000000	0.0000000	0.0000000
HMX1	0.0023930	0.0041070	0.0000000	0.0012986	0.0070107
ENSG00000288075	0.0478723	0.0600321	0.0401844	0.1082045	0.1764678
ENSG00000287972	0.0032281	0.0014787	0.0048493	0.0000000	0.0035405
FAM90A26	0.0000000	0.0000000	0.0031553	0.0000000	0.0000000
ENSG00000287117	0.0592101	0.0481554	0.0528473	0.0463665	0.1315564
SLC2A9	0.0061234	0.0074887	0.0085960	0.0155539	0.0123003
DRD5	0.0000000	0.0004927	0.0000000	0.0000000	0.0095606
WDR1	0.3364869	0.2494841	0.3533061	0.3410049	0.2432583
ENSG00000289865	0.0013695	0.0000000	0.0000000	0.0000000	0.0080063
ZNF518B	0.2710649	0.2997739	0.3746903	0.2527168	0.2822657
ENSG00000287154	0.0021098	0.0058385	0.0000000	0.0000000	0.0044531
CLNK	0.0099742	0.0007206	0.0040975	0.0093005	0.0116588
HS3ST1	0.0917928	0.0785017	0.0687538	0.0904201	0.1322023
ENSG00000287778	0.0082417	0.0082513	0.0050402	0.0093884	0.0225366
ENSG00000251152	0.0000000	0.0000000	0.0000000	0.0000000	0.0000000
ENSG00000249631	0.0036003	0.0000000	0.0031575	0.0027265	0.0110112
LINC02360	0.0027262	0.0000000	0.0000000	0.0000000	0.0062037
LINC02270	0.0000000	0.0000000	0.0000000	0.0000000	0.0000000
ENSG00000250371	0.0000000	0.0014479	0.0000000	0.0000000	0.0021475
ENSG00000250098	0.0000000	0.0000000	0.0000000	0.0000000	0.0069257
RAB28	0.3028998	0.2303940	0.2013853	0.3014780	0.4532275
ENSG00000286297	0.0018071	0.0000000	0.0000000	0.0000000	0.0000000
BOD1L1	0.8296075	0.9685228	0.9887171	0.7310692	0.9166057
LINC01182	0.0070795	0.0070199	0.0043589	0.0076381	0.0083148
ENSG00000249988	0.0000000	0.0024487	0.0000000	0.0000000	0.0040644
ENSG00000287360	0.0004259	0.0010630	0.0018758	0.0000000	0.0000000
LINC00504	0.0051287	0.0081015	0.0118093	0.0000000	0.0193511
CPEB2-DT	0.0049832	0.0054862	0.0103169	0.0089631	0.0157445
CPEB2	0.3370719	0.4189092	0.3476201	0.2957904	0.5828495
C1QTNF7-AS1	0.0998832	0.0894353	0.0900060	0.0718508	0.4253424
C1QTNF7	0.0023032	0.0049992	0.0000000	0.0033742	0.0217181
ENSG00000251379	0.0000000	0.0000000	0.0000000	0.0000000	0.0043097
CC2D2A	0.5121964	0.4181375	0.2299892	0.5585050	0.4552330
ENSG00000273133	0.0016098	0.0000000	0.0000000	0.0028069	0.0037043
FBXL5	0.5535982	0.5111210	0.4813514	0.5408183	0.5817140
FAM200B	0.7994924	0.7592219	0.7158589	0.7763578	0.6034662
BST1	0.0211669	0.0174523	0.0096357	0.0249497	0.0129192
CD38	0.0022138	0.0048587	0.0016348	0.0097709	0.0073809
PROM1	0.1089479	0.0533711	0.0488568	0.2338323	0.2053053
TAPT1	0.2269345	0.2008942	0.2470689	0.2474992	0.2147218
TAPT1-AS1	0.0585738	0.0874091	0.0608601	0.0555093	0.1830903
ENSG00000286888	0.0031545	0.0000000	0.0000000	0.0000000	0.0000000
ENSG00000248138	0.0027881	0.0030084	0.0044849	0.0066587	0.0019839
LDB2	0.2461940	0.2418043	0.3569029	0.3061166	0.3889537

ENSG00000249998	0.0000000	0.0000000	0.0000000	0.0048478	0.0000000
QDPR	0.6695178	0.6131862	0.6628715	0.6952125	0.4566289
LAP3	0.3763532	0.2528636	0.1749068	0.4855440	0.2218094
MED28-DT	0.0122286	0.0063621	0.0056502	0.0101837	0.0017206
MED28	0.4762146	0.4511636	0.5362731	0.4733830	0.4451586
FAM184B	0.0498389	0.1002589	0.1576366	0.0309340	0.1652109
DCAF16	0.3142448	0.2972248	0.3384564	0.2548469	0.2079529
NCAPG	0.0123262	0.0132075	0.0241391	0.0164425	0.0271919
LCORL	0.5977620	0.5314051	0.6162986	0.6276809	0.7769912
ENSG00000286046	0.0065080	0.0092745	0.0048841	0.0000000	0.0065298
ENSG00000248515	0.0060224	0.0036635	0.0064863	0.0031240	0.0202560
SLIT2	1.1477684	1.0694236	0.7344608	1.4100131	1.8793995
PACRGL	0.1965161	0.2248057	0.1684687	0.2693250	0.2347238
KCNIP4	0.6437250	0.8009553	0.8346812	0.5939704	2.1650028
ENSG00000272995	0.0009193	0.0000000	0.0009400	0.0030891	0.0031339
ENSG00000250243	0.0035968	0.0034333	0.0000000	0.0021110	0.0670811
ENSG00000286318	0.0030614	0.0087691	0.0020829	0.0000000	0.0178121
ENSG00000250092	0.0007135	0.0000000	0.0000000	0.0000000	0.0226853
ENSG00000248343	0.0006013	0.0000000	0.0012756	0.0000000	0.0325056
KCNIP4-IT1	0.0099934	0.0188104	0.0022472	0.0107861	0.1024404
ENSG00000250039	0.0007600	0.0012356	0.0000000	0.0000000	0.0000000
ADGRA3	0.1820467	0.1698640	0.1209928	0.1923729	0.2608331
GBA3	0.0044762	0.0032308	0.0023283	0.0000000	0.0065713
ENSG00000250137	0.0000000	0.0000000	0.0000000	0.0000000	0.0000000
PPARGC1A	0.1119101	0.1726853	0.1654078	0.1124790	0.2359392
ENSG00000289201	0.0192763	0.0367009	0.0323250	0.0253023	0.1183607
DHX15	0.4209706	0.3554873	0.3988333	0.4051397	0.3999428
LINC02473	0.0042329	0.0000000	0.0061766	0.0049031	0.0000000
SOD3	0.0219535	0.0165110	0.0063879	0.0558830	0.0190554
CCDC149	0.1799766	0.2018373	0.2058347	0.1787036	0.1792482
LGI2	0.2819526	0.5892066	0.4538881	0.1842464	0.3451225
SEPSECS	0.0797076	0.0631601	0.0621562	0.0829380	0.0271626
SEPSECS-AS1	0.0335327	0.0282512	0.0288271	0.0161611	0.0276571
PI4K2B	0.0414140	0.0478887	0.0370541	0.0862892	0.0607139
ZCCHC4	0.0837004	0.0446141	0.0417629	0.1038396	0.0885726
ANAPC4	0.1742779	0.1514459	0.1476319	0.1173239	0.2012722
ENSG00000248545	0.0018505	0.0020258	0.0012337	0.0000000	0.0072647
SLC34A2	0.0000000	0.0018103	0.0000000	0.0000000	0.0000000
SEL1L3	0.0179285	0.0394148	0.0203161	0.0282392	0.0573978
SMIM20	0.4167200	0.3062889	0.2866132	0.4082813	0.2681466
RBPJ	0.8731021	0.9531384	0.9261833	0.8103367	0.8945812
CCKAR	0.0033086	0.0041792	0.0000000	0.0000000	0.0126971
TBC1D19	0.2586830	0.2507537	0.2053830	0.2280046	0.4751533
STIM2	0.2231480	0.2463558	0.2199473	0.2593104	0.3636096
STIM2-AS1	0.0076709	0.0029583	0.0072433	0.0086529	0.0000000
ENSG00000286321	0.0000000	0.0000000	0.0000000	0.0000000	0.0000000
ENSG00000250064	0.0000000	0.0000000	0.0036262	0.0000000	0.0000000
ENSG00000288940	0.0027347	0.0010242	0.0027232	0.0000000	0.0000000
ENSG00000286596	0.0055373	0.0081970	0.0139099	0.0115214	0.0027453
PCDH7	0.7143466	0.9494941	0.7484891	0.7351045	1.6468856
ENSG00000249678	0.0009517	0.0013262	0.0019301	0.0000000	0.0034872
LINC02497	0.0033657	0.0042558	0.0000000	0.0084506	0.0033772
LINC02506	0.0013791	0.0055867	0.0000000	0.0000000	0.0163424

ENSG00000286784	0.0070929	0.0078043	0.0043689	0.0216101	0.0000000
ENSG00000288321	0.0000000	0.0000000	0.0011886	0.0000000	0.0000000
ENSG00000250597	0.0008766	0.0000000	0.0000000	0.0055683	0.0046589
ARAP2	0.1934981	0.1394246	0.0696490	0.1319434	0.1342688
ENSG00000247193	0.0018227	0.0000000	0.0000000	0.0099140	0.0000000
DTHD1	0.7563635	0.4277392	0.2672052	0.7237475	0.4358853
ENSG00000251438	0.0049447	0.0054078	0.0000000	0.0000000	0.0248170
LINC02505	0.0003601	0.0010370	0.0000000	0.0000000	0.0121245
NWD2	0.0897809	0.1725884	0.1966761	0.0677322	0.2641531
C4orf19	0.0157322	0.0158384	0.0069210	0.0220758	0.0431059
ENSG00000288073	0.0000000	0.0000000	0.0000000	0.0000000	0.0034716
RELL1	0.3673396	0.2286339	0.1542276	0.3901313	0.2234490
PGM2	0.2103962	0.1165448	0.0794461	0.2179800	0.1292007
TBC1D1	0.6388821	0.4245360	0.2785739	0.6183665	0.4712764
LINC01258	0.0000000	0.0000000	0.0000000	0.0000000	0.0098710
KLF3-AS1	0.0418642	0.0477301	0.0511631	0.0304718	0.1186972
KLF3	0.2990018	0.3388846	0.3444097	0.2747142	0.2380424
TLR10	0.0000000	0.0028425	0.0000000	0.0000000	0.0000000
TLR1	0.0000000	0.0007774	0.0000000	0.0015408	0.0000000
TLR6	0.0006712	0.0022217	0.0000000	0.0000000	0.0000000
FAM114A1	0.4445775	0.3333622	0.1792462	0.5380834	0.2577709
TMEM156	0.0000000	0.0000000	0.0000000	0.0000000	0.0000000
KLHL5	1.1895379	0.8813982	0.6992946	1.2816420	0.7614511
ENSG00000249207	0.0218404	0.0210546	0.0156241	0.0053232	0.0429876
ENSG00000249685	0.0031603	0.0000000	0.0030460	0.0000000	0.0034299
WDR19	0.2338995	0.1712540	0.1105689	0.2234304	0.2843708
RFC1	0.8924515	0.7393962	0.6837874	0.9330032	0.6150012
KLB	0.0021449	0.0010683	0.0037767	0.0000000	0.0025179
RPL9	3.0480565	3.0221426	2.8427096	2.9098698	2.7645227
LIAS	0.1954059	0.1709588	0.1128887	0.1328737	0.1432457
UGDH	0.1847010	0.1281091	0.1491074	0.1844978	0.1682160
UGDH-AS1	0.2194644	0.2278337	0.2478614	0.1873410	0.2034618
ENSG00000287262	0.0034192	0.0005197	0.0025247	0.0024989	0.0000000
SMIM14	0.6473467	0.7290013	0.6828085	0.5595049	0.6417467
ENSG00000286349	0.0051855	0.0037823	0.0000000	0.0055154	0.0243468
SMIM14-DT	0.0287380	0.0521310	0.0340269	0.0315945	0.0617760
UBE2K	0.9100691	0.9585722	1.0708643	0.8804759	1.0698767
PDS5A	0.3932007	0.3854515	0.3170997	0.4400864	0.5821951
N4BP2	0.5425690	0.6476681	0.6040297	0.4411988	0.8277635
ENSG00000260296	0.0234458	0.0343815	0.0372881	0.0165671	0.0840938
ENSG00000286089	0.0011205	0.0000000	0.0000000	0.0000000	0.0000000
RHOH	0.0340714	0.0203031	0.0120053	0.0324525	0.0050073
LINC02265	0.0139946	0.0037381	0.0093544	0.0049554	0.0141544
CHRNA9	0.0006823	0.0000000	0.0051243	0.0000000	0.0000000
RBM47	0.0273324	0.0101932	0.0048860	0.0066153	0.0209110
ENSG00000287182	0.0027346	0.0010236	0.0092023	0.0021257	0.0101255
NSUN7	0.1154394	0.0979782	0.1415091	0.0900529	0.0839805
APBB2	0.5136681	0.5417407	0.6748061	0.4058844	0.9381682
ENSG00000250906	0.0003422	0.0008976	0.0000000	0.0000000	0.0012623
UCHL1-DT	0.0116907	0.0048327	0.0023309	0.0022342	0.0204613
UCHL1	2.4686703	2.5556003	2.8196145	2.4781246	2.1920117
LIMCH1	1.1034921	0.8773167	0.8074200	1.1138627	1.1019569
ENSG00000287762	0.0029018	0.0012534	0.0000000	0.0000000	0.0048508

ENSG00000249216	0.0000000	0.0000000	0.0043385	0.0000000	0.0062328
PHOX2B	0.1055116	0.2426484	0.0806909	0.0944901	0.1781467
PHOX2B-AS1	0.0406293	0.0964594	0.0506422	0.0563258	0.2608285
LINC00682	0.0964045	0.1771192	0.0965854	0.0669861	0.1533145
ENSG00000249771	0.0000000	0.0018274	0.0000000	0.0000000	0.0000000
TMEM33	0.3430544	0.3397460	0.3117607	0.3892343	0.3144980
DCAF4L1	0.0000000	0.0010420	0.0000000	0.0000000	0.0162997
ENSG00000272862	0.0012148	0.0082162	0.0072149	0.0052944	0.0098984
SLC30A9	0.6671746	0.7541396	0.7805973	0.7370937	0.6744284
BEND4	0.0320296	0.0678351	0.0737298	0.0258838	0.0932178
ENSG00000285454	0.0047417	0.0228924	0.0153096	0.0024144	0.0268521
ENSG00000250781	0.0025843	0.0000000	0.0000000	0.0000000	0.0080883
ATP8A1	0.2486957	0.4163715	0.4860569	0.2339399	0.8619672
ATP8A1-DT	0.0000000	0.0000000	0.0000000	0.0017005	0.0000000
ENSG00000286891	0.0019160	0.0034852	0.0000000	0.0000000	0.0093543
ENSG00000250863	0.0000000	0.0000000	0.0000000	0.0000000	0.0064451
KCTD8	0.2659696	0.3873474	0.6862987	0.1732990	1.0476814
YIPF7	0.0164598	0.0175314	0.0186595	0.0116440	0.0658263
GUF1	0.3101611	0.3409964	0.3532859	0.3410075	0.2706277
GNPDA2	0.2463341	0.1930873	0.1732748	0.2636366	0.1858586
ENSG00000273369	0.0040821	0.0000000	0.0000000	0.0023684	0.0000000
ENSG00000272936	0.0049593	0.0033054	0.0047325	0.0085796	0.0215195
GABRG1	0.0130435	0.0144658	0.1150289	0.0173496	0.0854405
GABRA2	0.1998331	0.3442057	0.5322940	0.1831651	0.3675717
ENSG00000249330	0.0020079	0.0000000	0.0012177	0.0000000	0.0000000
COX7B2	0.0000000	0.0013072	0.0000000	0.0075526	0.0000000
GABRA4	0.0147584	0.0362483	0.0320060	0.0223432	0.0337582
GABRB1	0.1033612	0.1432236	0.2301527	0.1010440	0.4392683
ENSG00000260918	0.0235680	0.0513088	0.0616679	0.0311131	0.0579894
COMMD8	0.1977193	0.1436653	0.2076098	0.2309754	0.1138645
ENSG00000282904	0.0021262	0.0000000	0.0013623	0.0000000	0.0031863
ENSG00000286294	0.0002752	0.0012917	0.0019433	0.0000000	0.0000000
ATP10D	0.1418889	0.0997813	0.0463918	0.1545905	0.1467743
CORIN	0.3515114	0.2094214	0.1177079	0.6089580	0.2189945
ENSG00000282917	0.0298763	0.0122501	0.0211327	0.0134784	0.0359563
ENSG00000259959	0.0218555	0.0572252	0.0415099	0.0124567	0.0728078
NFXL1	0.2132881	0.1791683	0.2696793	0.1943757	0.3460108
NIPAL1	0.0154234	0.0348599	0.0269897	0.0172743	0.0253798
CNGA1	0.0087796	0.0091963	0.0045995	0.0030076	0.0281155
TXK	0.0049514	0.0033010	0.0000000	0.0000000	0.0000000
TEC	0.0073318	0.0151499	0.0105476	0.0000000	0.0100266
SLAIN2	0.5096662	0.5209958	0.4740984	0.4253871	0.5732038
SLC10A4	0.1077319	0.3049890	0.1295567	0.0917057	0.1006234
ZAR1	0.0000000	0.0060170	0.0000000	0.0000000	0.0000000
FRYL	0.3108410	0.2817072	0.2588842	0.2809213	0.5991760
OCIAD1	1.2449990	1.2348756	1.3398650	1.3628736	1.0528237
OCIAD1-AS1	0.0044286	0.0038613	0.0015522	0.0037492	0.0167860
OCIAD2	0.4308787	0.6295897	0.8192950	0.4172885	0.4847897
DCUN1D4	0.3294829	0.3872877	0.4007958	0.3769151	0.6010813
ENSG00000272576	0.0012929	0.0019900	0.0022411	0.0000000	0.0039284
LRRC66	0.0028515	0.0213224	0.0070448	0.0066149	0.0156722
SGCB	0.7126093	0.6459599	0.6297510	0.8441696	0.5493667
SPATA18	0.1763785	0.0919856	0.0535983	0.1731797	0.1092915

ENSG00000287999	0.0021124	0.0022172	0.0000000	0.0000000	0.0000000
USP46	0.4455741	0.5036287	0.5083216	0.4460192	0.4385089
USP46-DT	0.0106331	0.0066890	0.0048426	0.0014739	0.0178539
DANCR	0.2795110	0.3137041	0.3707971	0.2474482	0.2049075
LINC01618	0.0037096	0.0044938	0.0115358	0.0000000	0.0283627
ENSG00000286161	0.0000000	0.0000000	0.0000000	0.0000000	0.0000000
ERVMER34-1	0.0000000	0.0000000	0.0000000	0.0000000	0.0016917
RASL11B	0.0000000	0.0014637	0.0000000	0.0000000	0.0087381
SCFD2	0.1056912	0.1113529	0.1420142	0.0786341	0.4074616
ENSG00000248115	0.0000000	0.0000000	0.0000000	0.0000000	0.0000000
FIP1L1	0.5569528	0.5052281	0.4932792	0.4811722	0.5334483
LNX1	0.0195702	0.0111438	0.0181767	0.0113963	0.0126432
LNX1-AS1	0.0000000	0.0000000	0.0000000	0.0000000	0.0000000
ENSG00000249706	0.0000000	0.0000000	0.0016682	0.0000000	0.0000000
ENSG00000272650	0.0002408	0.0007434	0.0006660	0.0076081	0.0000000
CHIC2	0.2631267	0.2428074	0.2874596	0.2654687	0.2990077
PDGFRA	0.0087151	0.0072674	0.0076168	0.0110232	0.0192926
KIT	0.0181249	0.0372270	0.0780323	0.0273355	0.0467147
KDR	0.0035147	0.0008879	0.0000000	0.0168542	0.0000000
SRD5A3	0.0815456	0.0737200	0.0491708	0.1006065	0.0778750
SRD5A3-AS1	0.0036355	0.0051495	0.0041091	0.0125391	0.0100424
LINC02928	0.0071739	0.0060878	0.0068769	0.0000000	0.0000000
TMEM165	0.4567818	0.3344772	0.2867450	0.6262188	0.4801258
CLOCK	0.2561417	0.2938929	0.2657923	0.2688397	0.4777000
ENSG00000272969	0.0008924	0.0016225	0.0015972	0.0000000	0.0000000
PDCL2	0.0000000	0.0017360	0.0011903	0.0000000	0.0000000
NMU	0.0563246	0.1153123	0.0912777	0.0560608	0.1189135
EXOC1L	0.0000000	0.0028778	0.0000000	0.0000000	0.0000000
EXOC1	0.4733492	0.4990807	0.5191192	0.4239065	0.6030544
ENSG00000286599	0.0015785	0.0012840	0.0000000	0.0000000	0.0000000
CEP135	0.1480101	0.1002514	0.1090403	0.1269793	0.1868394
CRACD	0.5106799	0.6376691	0.7636805	0.4896503	0.9210538
ENSG00000286093	0.0000000	0.0000000	0.0000000	0.0000000	0.0016673
AASDH	0.1852339	0.1620922	0.1205206	0.1787489	0.1509906
ENSG00000269921	0.0058819	0.0011745	0.0043928	0.0000000	0.0083025
PPAT	0.2636786	0.2442031	0.3301118	0.2673812	0.2694651
ENSG00000270147	0.0038348	0.0000000	0.0000000	0.0017263	0.0020430
ENSG00000290817	0.0084523	0.0083773	0.0099379	0.0104439	0.0072417
PAICS	0.5849698	0.4707701	0.3850457	0.6844736	0.3933176
ENSG00000289393	0.0132331	0.0086518	0.0261211	0.0174950	0.0033534
SRP72	0.7954212	0.7929484	0.9606764	0.8253450	0.6791041
ARL9	0.0226971	0.0166860	0.0313496	0.0154278	0.0305395
THEGL	0.0047578	0.0000000	0.0023481	0.0000000	0.0037844
HOPX	0.0961594	0.2127172	0.2635437	0.0903918	0.0705168
REST	0.4564197	0.2311973	0.1462544	0.5101220	0.2307338
ENSG00000269949	0.0000000	0.0000000	0.0000000	0.0000000	0.0000000
NOA1	0.2161377	0.2242137	0.2178965	0.1828372	0.1715868
POLR2B	0.5078999	0.5242344	0.6228520	0.5015261	0.5204256
IGFBP7	0.5797257	0.3475637	0.2162562	1.2449949	0.4423662
IGFBP7-AS1	0.4136245	0.2614511	0.2118687	0.3277369	0.2152987
ENSG00000251459	0.0028436	0.0010959	0.0000000	0.0000000	0.0059744
ENSG00000250333	0.0000000	0.0000000	0.0000000	0.0000000	0.0000000
ENSG00000249111	0.0011041	0.0012418	0.0031248	0.0000000	0.0239160

ADGRL3	0.7815751	0.9048542	0.8436871	0.7366605	1.8857596
ENSG00000205682	0.0012829	0.0016789	0.0020099	0.0000000	0.0069873
ENSG00000289308	0.0000000	0.0000000	0.0000000	0.0000000	0.0094476
ADGRL3-AS1	0.0306971	0.0291623	0.0181361	0.0248265	0.0312224
ENSG00000288659	0.0000000	0.0018536	0.0000000	0.0061329	0.0106969
TECRL	0.0008421	0.0000000	0.0028810	0.0000000	0.0000000
LINC02232	0.0020202	0.0033196	0.0053536	0.0053192	0.0038769
LINC02835	0.0000000	0.0000000	0.0000000	0.0000000	0.0000000
EPHA5	0.3879543	0.7102515	0.7653003	0.3567277	1.0789345
EPHA5-AS1	0.1045722	0.1040061	0.1284608	0.0993972	0.1196584
ENSG00000248479	0.0055147	0.0010370	0.0013411	0.0027747	0.0000000
ENSG00000249413	0.0000000	0.0019886	0.0000000	0.0000000	0.0086970
ENSG00000250075	0.0250471	0.0170029	0.0224274	0.0021808	0.0958449
CENPC	0.3278851	0.2595983	0.2434982	0.3031616	0.2917617
STAP1	0.0002116	0.0000000	0.0007943	0.0000000	0.0033772
UBA6	0.4427567	0.4466703	0.4355672	0.4159193	0.6216348
UBA6-DT	0.2658465	0.3067070	0.2728524	0.2829433	0.6654679
GNRHR	0.0016281	0.0006494	0.0014605	0.0047102	0.0075813
TMPRSS11D	0.0018478	0.0000000	0.0000000	0.0000000	0.0130295
TMPRSS11A	0.0011266	0.0000000	0.0019650	0.0049218	0.0083345
TMPRSS11F	0.0000000	0.0000000	0.0000000	0.0000000	0.0054516
YTHDC1	1.0353449	0.9282514	0.8276332	0.8922115	0.9253983
UGT2B7	0.0005709	0.0000000	0.0000000	0.0000000	0.0056926
SULT1B1	0.0014703	0.0011956	0.0000000	0.0034891	0.0000000
ENSG00000286848	0.0002195	0.0008957	0.0000000	0.0000000	0.0000000
JCHAIN	0.0004745	0.0000000	0.0006307	0.0000000	0.0057345
UTP3	0.2193631	0.2090820	0.1805355	0.2209752	0.1930971
ENSG00000272986	0.0066427	0.0000000	0.0080762	0.0093774	0.0059248
RUFY3	1.3574954	1.5540207	1.6850836	1.3071277	1.4628725
GRSF1	1.1035079	0.9106742	0.8107908	1.1031546	0.7340189
ENSG00000289019	0.0541291	0.0509373	0.0304888	0.0399574	0.0380369
MOB1B	0.6363282	0.4969666	0.3641678	0.6116157	0.4902508
DCK	0.4138120	0.4925986	0.5636635	0.4851071	0.3236791
SLC4A4	0.3645749	0.2195575	0.1744712	0.6411412	0.2565788
NPFFR2	0.0178989	0.0118018	0.0054185	0.0140735	0.0193340
ADAMTS3	0.0184629	0.0089657	0.0099561	0.0104938	0.0254000
COX18	0.1872659	0.1957446	0.2610078	0.1798547	0.1943645
ANKRD17	0.6563084	0.7257975	0.6865866	0.6684711	1.0856885
ANKRD17-DT	0.0718662	0.0695826	0.0429412	0.0406628	0.1836896
ALB	0.0074830	0.0055220	0.0023930	0.0032755	0.0000000
AFP	0.0003101	0.0000000	0.0023388	0.0108427	0.0000000
RASSF6	0.0070090	0.0014428	0.0022351	0.0133310	0.0037962
CXCL8	0.0032439	0.0022573	0.0007329	0.0047021	0.0000000
CXCL6	0.0216652	0.0020785	0.0053383	0.0150810	0.0000000
CXCL1	0.0035628	0.0000000	0.0019944	0.0173721	0.0063320
CXCL5	0.0663358	0.0406524	0.0188794	0.0560489	0.0272719
ENSG00000287037	0.0041206	0.0017916	0.0000000	0.0000000	0.0000000
CXCL3	0.0180267	0.0127662	0.0085866	0.0453918	0.0116643
CXCL2	0.0165434	0.0022892	0.0039735	0.0335523	0.0015298
ENSG00000289241	0.0044959	0.0000000	0.0000000	0.0036320	0.0000000
MTHFD2L	0.4265057	0.3686308	0.2933865	0.5801066	0.5033220
ENSG00000269559	0.0062752	0.0023519	0.0092907	0.0055893	0.0000000
EPGN	0.0000000	0.0012869	0.0015948	0.0000000	0.0078956

EREG	0.0016809	0.0000000	0.0000000	0.0000000	0.0000000
BTC	0.0297795	0.0094879	0.0028823	0.0334889	0.0046920
PARM1	0.2098620	0.1609393	0.0983373	0.4345930	0.1948238
ENSG00000248165	0.0000000	0.0000000	0.0000000	0.0000000	0.0000000
LINC02562	0.0017576	0.0031266	0.0026613	0.0043405	0.0038769
ENSG00000248646	0.0009837	0.0019773	0.0000000	0.0000000	0.0000000
RCHY1	0.3003069	0.3187646	0.3968929	0.2994926	0.2706291
THAP6	0.2183654	0.1951504	0.1836588	0.2085550	0.2162581
ODAPH	0.0042496	0.0043572	0.0069982	0.0000000	0.0109134
CDKL2	0.5466554	0.5870950	0.6000721	0.5632197	0.5166143
G3BP2	1.0588053	1.1975127	1.4294847	1.0296603	1.0817443
USO1	0.4985057	0.3611145	0.2940158	0.4934813	0.4305041
ENSG00000229717	0.0008858	0.0022401	0.0000000	0.0000000	0.0091873
PPEF2	0.0000000	0.0000000	0.0000000	0.0023899	0.0072062
NAAA	0.0367873	0.0380306	0.0572478	0.0587504	0.0452318
SDAD1	0.4728740	0.4354580	0.4707583	0.4502591	0.3999912
SDAD1-AS1	0.0000000	0.0024543	0.0000000	0.0000000	0.0021357
ART3	0.0597523	0.0228593	0.0062112	0.0823361	0.0096902
CXCL11	0.0059868	0.0074042	0.0020031	0.0141667	0.0113455
NUP54	0.2608332	0.2312558	0.2126758	0.2198295	0.3069804
ENSG00000286074	0.0034521	0.0019224	0.0000000	0.0000000	0.0092794
SCARB2	1.0840197	0.9722377	0.9550037	1.3716902	1.0402833
FAM47E	0.1434488	0.1441220	0.1016789	0.1295567	0.0883254
ENSG00000287401	0.0143015	0.0116374	0.0161317	0.0071342	0.0150394
ENSG00000289515	0.0487121	0.0316090	0.0393656	0.0524265	0.0184478
STBD1	0.1793750	0.1261745	0.1056218	0.1485868	0.1120281
CCDC158	0.0035240	0.0131344	0.0096410	0.0000000	0.0038927
SHROOM3	0.2403891	0.1471245	0.1230127	0.2608070	0.2993077
SHROOM3-AS1	0.0027311	0.0050571	0.0000000	0.0000000	0.0027453
SOWAHB	0.0034312	0.0000000	0.0014820	0.0028178	0.0000000
ENSG00000289586	0.1557447	0.3624116	0.3600300	0.1935575	0.2485470
SEPTIN11	1.4352073	1.2119795	1.2215705	1.3802320	1.1366169
CCNI	2.3884984	2.4854420	2.5556626	2.2335098	2.1254885
ENSG00000289443	0.0048345	0.0014953	0.0038529	0.0000000	0.0295621
ENSG00000288888	0.0032166	0.0047959	0.0000000	0.0097045	0.0034773
ENSG00000289496	0.0052436	0.0020473	0.0011413	0.0064675	0.0116960
CCNG2	0.4063437	0.4495580	0.3160012	0.3953454	0.2553874
ENSG00000249036	0.0010767	0.0000000	0.0000000	0.0000000	0.0000000
CXCL13	0.0000000	0.0000000	0.0000000	0.0000000	0.0000000
CNOT6L	0.3382441	0.4110462	0.4331040	0.2987146	0.5738367
MRPL1	0.4212595	0.5334671	0.6225483	0.3691124	0.4548583
FRAS1	0.0366966	0.0406317	0.0251535	0.0501061	0.1797260
ANXA3	0.0061736	0.0051099	0.0039534	0.0000000	0.0033732
LINC01094	0.0125028	0.0108299	0.0053021	0.0278211	0.0267479
BMP2K-DT	0.0006530	0.0000000	0.0000000	0.0000000	0.0016673
BMP2K	0.1569118	0.1455200	0.1387497	0.1732661	0.2785394
PAQR3	0.1686493	0.2339379	0.2274716	0.1934041	0.2409224
LINC01088	0.0229599	0.0218359	0.0231252	0.0216376	0.0301948
NAA11	0.0000000	0.0000000	0.0000000	0.0044606	0.0000000
PCAT4	0.0027871	0.0034821	0.0000000	0.0000000	0.0000000
ANTXR2	0.2477627	0.1243651	0.0800052	0.3252714	0.1517289
PRDM8-AS1	0.0000000	0.0041709	0.0000000	0.0000000	0.0202497
PRDM8	0.0042919	0.0104327	0.0056809	0.0033903	0.0000000

FGF5	0.0032209	0.0037034	0.0025686	0.0039928	0.0051674
CFAP299	0.1203158	0.0877605	0.0578338	0.0976924	0.1827401
BMP3	0.0037070	0.0050318	0.0000000	0.0000000	0.0134229
PRKG2	0.0047659	0.0166495	0.0217714	0.0047294	0.0503820
PRKG2-AS1	0.0000000	0.0000000	0.0000000	0.0000000	0.0070049
RASGEF1B	0.2970973	0.3460880	0.4234351	0.2348055	0.7280594
ENSG00000273156	0.0284217	0.0602630	0.0535470	0.0318633	0.0658297
ENSG00000289480	0.0074119	0.0062407	0.0074688	0.0062557	0.0139758
HNRNPD	1.2089989	1.1740373	1.2686111	1.1677372	1.0827327
HNRNPD-DT	0.0256774	0.0287062	0.0388667	0.0193048	0.0291298
HNRNPDL	1.9185580	1.8171984	1.7611322	1.8938387	1.6982757
ENOPH1	0.6890412	0.5730120	0.6483957	0.7560505	0.5228379
TMEM150C	0.1904969	0.1788541	0.1371096	0.2115369	0.1783626
LINC00575	0.0000000	0.0000000	0.0000000	0.0000000	0.0014252
SCD5	0.5656547	0.6091466	0.7369729	0.7090792	0.6599665
SEC31A	1.0289218	0.9429501	0.9067146	0.9555716	1.0458537
THAP9-AS1	0.1759073	0.2078018	0.2603402	0.1770822	0.2075092
THAP9	0.0998567	0.0744860	0.0722014	0.0942286	0.0937117
LIN54	0.1547826	0.1002768	0.1432187	0.1288098	0.1635825
COPS4	0.4822023	0.4567811	0.5734209	0.5100626	0.4586330
ENSG00000286035	0.0054201	0.0039523	0.0000000	0.0000000	0.0184984
PLAC8	0.0077256	0.0031685	0.0019301	0.0000000	0.0108156
ENSG00000250057	0.0000000	0.0022694	0.0026175	0.0000000	0.0000000
COQ2	0.0904953	0.1030358	0.1203728	0.1342008	0.1032672
HPSE	0.0032511	0.0105290	0.0062621	0.0000000	0.0165741
HELQ	0.1255967	0.1284293	0.0953057	0.0979053	0.1176558
MRPS18C	0.5178424	0.5066906	0.5867059	0.4500242	0.4369633
ABRAXAS1	0.2128006	0.1435606	0.0789491	0.2110314	0.0987121
GPAT3	0.0109539	0.0094222	0.0117483	0.0174526	0.0270656
LINC02994	0.0118322	0.0052075	0.0015424	0.0000000	0.0341114
ENSG00000248749	0.0069326	0.0043480	0.0029942	0.0000000	0.0000000
NKX6-1	0.4977581	0.4422059	0.5150819	0.5899783	0.3626097
CDS1	0.3180083	0.2289881	0.2058036	0.3812792	0.2992687
WDFY3	0.4892637	0.4427128	0.3992495	0.3811055	1.0112617
WDFY3-AS1	0.0046997	0.0054157	0.0000000	0.0077821	0.0166638
WDFY3-AS2	0.1685882	0.1319812	0.0635360	0.1471434	0.0932296
ARHGAP24	0.3544136	0.2574955	0.3344176	0.3263486	0.5379776
MAPK10	0.7980265	0.8171207	0.7946782	0.7229395	1.3218525
MAPK10-AS1	0.0078494	0.0055943	0.0040605	0.0122026	0.0560195
PTPN13	0.4084006	0.2642657	0.2153953	0.3994627	0.3949689
SLC10A6	0.0025223	0.0000000	0.0025433	0.0028566	0.0000000
C4orf36	0.0166778	0.0177461	0.0211152	0.0349343	0.0122341
ENSG00000284968	0.0772304	0.0357695	0.0300411	0.0520933	0.0486575
AFF1	0.1979711	0.1569976	0.1325793	0.1522597	0.2280914
KLHL8	0.1684504	0.3233587	0.3243015	0.1280005	0.3048361
HSD17B13	0.0010561	0.0046147	0.0000000	0.0011055	0.0000000
HSD17B11	0.0686054	0.1004939	0.0766777	0.1352192	0.0544983
NUDT9	0.3471203	0.2731379	0.2713721	0.4339970	0.3138962
SPARCL1	0.4085227	0.3306310	0.2065724	1.6909597	0.6204303
ENSG00000249001	0.0022779	0.0000000	0.0000000	0.0000000	0.0000000
IBSP	0.0069395	0.0060688	0.0000000	0.0000000	0.0069764
ENSG00000286618	0.0030683	0.0039359	0.0016003	0.0027537	0.0000000
SPP1	0.0453703	0.0549267	0.0984897	0.1402614	0.0823167

ENSG00000289034	0.0005219	0.0025479	0.0000000	0.0016996	0.0000000
PKD2	0.1830021	0.1745629	0.1235338	0.1581511	0.4903557
ABCG2	0.0045469	0.0025246	0.0041974	0.0022588	0.0224244
PPM1K	0.8837014	0.8647916	1.0345100	0.8116769	0.8192887
PPM1K-DT	0.0140344	0.0131277	0.0107733	0.0234317	0.0207944
HERC6	0.0693041	0.0455823	0.0289685	0.0828249	0.0576821
HERC5	0.0479232	0.0173675	0.0063139	0.0488005	0.0137518
PIGY-DT	0.0162755	0.0043550	0.0176804	0.0093006	0.0055814
HERC3	0.0918389	0.1608224	0.1979275	0.0601392	0.3080422
NAP1L5	0.6181207	0.9781862	1.2357241	0.4672527	0.7913683
FAM13A-AS1	0.0120677	0.0078332	0.0097853	0.0083764	0.0409189
FAM13A	0.2239334	0.1897882	0.1560850	0.2143182	0.2464335
TIGD2	0.0386920	0.0416528	0.0801595	0.0312616	0.0519628
ENSG00000270720	0.0007712	0.0030577	0.0022190	0.0000000	0.0000000
GPRIN3	0.4176957	0.4754743	0.5530996	0.3204341	0.5167999
ENSG00000251095	0.0022077	0.0043278	0.0073208	0.0000000	0.0130729
ENSG00000288563	0.0000000	0.0013877	0.0026244	0.0000000	0.0000000
SNCA	0.5878968	0.9816651	1.0840824	0.5234411	0.7914404
ENSG00000277695	0.0000000	0.0000000	0.0000000	0.0000000	0.0000000
SNCA-AS1	0.0031421	0.0033351	0.0000000	0.0000000	0.0000000
MMRN1	0.2004693	0.1295764	0.0871069	0.4926128	0.1592801
CCSER1	0.6522685	0.7675845	0.7120832	0.5315360	2.1270069
ENSG00000248984	0.0000000	0.0013272	0.0000000	0.0000000	0.0207294
ENSG00000249049	0.0000000	0.0000000	0.0000000	0.0000000	0.0000000
ENSG00000248511	0.0000000	0.0000000	0.0000000	0.0024577	0.0000000
GRID2	0.4735982	0.5709441	0.5292225	0.4348626	1.7140777
ENSG00000248627	0.0000000	0.0000000	0.0000000	0.0000000	0.0023501
SMARCAD1-DT	0.0021154	0.0015753	0.0000000	0.0000000	0.0019259
SMARCAD1	0.3751511	0.4289458	0.5581939	0.4002549	0.5491713
HPGDS	0.0008382	0.0009219	0.0000000	0.0000000	0.0000000
ENSG00000287552	0.0000000	0.0000000	0.0000000	0.0000000	0.0000000
PDLIM5	0.2934337	0.1862841	0.1015866	0.4363770	0.1522342
ENSG00000249951	0.0008875	0.0023272	0.0029736	0.0012694	0.0000000
BMPR1B-DT	0.0110392	0.0078247	0.0064163	0.0320300	0.0088315
BMPR1B	0.2750471	0.1382487	0.0758916	0.3491308	0.2774165
UNC5C	0.1073767	0.2122329	0.0932118	0.1243566	0.6398634
UNC5C-AS1	0.0097683	0.0287577	0.0315867	0.0119453	0.0198666
LINC02267	0.0000000	0.0009263	0.0000000	0.0000000	0.0000000
ENSG00000253170	0.0078443	0.0015363	0.0051371	0.0078360	0.0000000
STPG2	0.0274243	0.0299528	0.0298939	0.0193838	0.1318687
STPG2-AS1	0.0018303	0.0000000	0.0000000	0.0028028	0.0000000
ENSG00000214559	0.0058528	0.0031493	0.0021670	0.0000000	0.0068820
ENSG00000289532	0.0082397	0.0053782	0.0089116	0.0012954	0.0033679
RAP1GDS1	0.6358027	0.8573088	0.9869706	0.5994606	0.9225015
TSPAN5	0.3073508	0.3756831	0.4574231	0.3459376	0.6332271
ENSG00000251523	0.0023222	0.0000000	0.0000000	0.0000000	0.0000000
TSPAN5-DT	0.0072845	0.0158973	0.0051856	0.0152827	0.0058520
EIF4E	0.6459749	0.6845175	0.8315632	0.5788188	0.6254672
ENSG00000263923	0.0052758	0.0089713	0.0016682	0.0040550	0.0017223
METAP1	0.1064112	0.1669415	0.1684577	0.1268893	0.1971673
ENSG00000272777	0.0034532	0.0010644	0.0025543	0.0033091	0.0000000
ADH5	0.8667848	0.7812525	0.6468244	0.8921020	0.5562807
ENSG00000246090	0.0633734	0.0519568	0.0431415	0.0371944	0.1043362

ADH4	0.0000000	0.0000000	0.0000000	0.0000000	0.0000000
TRMT10A	0.1958171	0.1662082	0.1464837	0.1388011	0.1289575
MTTP	0.0090956	0.0065197	0.0044762	0.0049349	0.0083310
C4orf54	0.0000000	0.0000000	0.0000000	0.0000000	0.0000000
LAMTOR3	0.6427242	0.5807996	0.5734793	0.6154897	0.4287905
DNAJB14	0.5319937	0.4391193	0.5052144	0.5284239	0.4987444
H2AZ1	1.1578010	1.1568153	1.2288352	1.0787767	0.9602195
H2AZ1-DT	0.0112211	0.0184886	0.0132859	0.0132233	0.0207119
DDIT4L	0.0034318	0.0000000	0.0000000	0.0059854	0.0000000
EMCN	0.0008588	0.0000000	0.0014218	0.0000000	0.0149365
PPP3CA	0.5372576	0.7214275	0.8105575	0.5489351	1.1225306
FLJ20021	0.0372012	0.0617819	0.0581510	0.0550914	0.0212884
BANK1	0.0018709	0.0000000	0.0000000	0.0018727	0.0000000
SLC39A8	0.0535420	0.0295550	0.0338486	0.0641286	0.0733062
ENSG00000248161	0.0407004	0.0467735	0.0350621	0.0588710	0.0088161
NFKB1	0.1092876	0.0817722	0.0871793	0.1172271	0.1401429
MANBA	0.2080947	0.1361873	0.1398273	0.3340603	0.3135868
UBE2D3	1.1975533	1.2378898	1.2336133	1.0538790	1.1731179
ENSG00000286242	0.0010484	0.0101463	0.0018589	0.0052134	0.0062827
UBE2D3-AS1	0.1005377	0.0706766	0.0588473	0.0735921	0.1009945
CISD2	0.5524077	0.6018766	0.7490265	0.5939690	0.4397785
SLC9B1	0.0472293	0.0303086	0.0388531	0.0324536	0.0952367
SLC9B2	0.1414076	0.2118779	0.3233274	0.1219096	0.1880213
BDH2	0.3667030	0.2025106	0.1882623	0.4287469	0.2361769
CENPE	0.0277819	0.0627130	0.0576765	0.0338989	0.0464326
LINC02428	0.0136979	0.0174585	0.0132585	0.0085293	0.0584797
TACR3-AS1	0.0036178	0.0055214	0.0058660	0.0000000	0.0088433
TACR3	0.0090965	0.0126847	0.0130278	0.0107563	0.0198972
LINC02503	0.0000000	0.0013112	0.0000000	0.0000000	0.0065249
ENSG00000251170	0.0000000	0.0000000	0.0000000	0.0000000	0.0000000
CXXC4	0.6360473	0.5936063	0.7817623	0.6077136	0.6424327
CXXC4-AS1	0.0103471	0.0240937	0.0065545	0.0065540	0.0486498
ENSG00000248242	0.0000000	0.0032212	0.0000000	0.0000000	0.0084888
ENSG00000248373	0.0006935	0.0000000	0.0000000	0.0000000	0.0000000
ENSG00000251259	0.0081079	0.0048124	0.0006419	0.0093089	0.0000000
TET2	0.3135550	0.3591630	0.3611736	0.2764286	0.5984256
TET2-AS1	0.0000000	0.0000000	0.0034914	0.0000000	0.0057461
PPA2	0.6149875	0.6385265	0.5862165	0.7374861	0.6061142
ENSG00000250522	0.0000000	0.0000000	0.0000000	0.0000000	0.0000000
ARHGEF38	0.0023951	0.0000000	0.0020611	0.0000000	0.0000000
INTS12	0.2016864	0.2506852	0.2396475	0.2026480	0.1764993
GSTCD	0.1558061	0.1649886	0.1652963	0.2005602	0.2666630
NPNT	0.0629956	0.0429803	0.0283998	0.0701720	0.0868299
ENSG00000250740	0.0014314	0.0000000	0.0000000	0.0000000	0.0000000
ENSG00000249635	0.0030966	0.0058746	0.0062689	0.0021517	0.0097257
TBCK	0.2219184	0.2509513	0.2698316	0.1651014	0.6434320
AIMP1	0.7214196	0.6048312	0.5755193	0.6651626	0.5125381
ENSG00000286147	0.0000000	0.0070868	0.0011855	0.0000000	0.0117487
DKK2	0.0116708	0.0213305	0.0124082	0.0087701	0.0645552
PAPSS1	0.5303319	0.5465860	0.6331584	0.5089345	0.6351388
SGMS2	0.0564850	0.0433092	0.0184510	0.0388021	0.0515012
CYP2U1-AS1	0.0042835	0.0266961	0.0008287	0.0050110	0.0031033
CYP2U1	0.1276001	0.0757834	0.0619406	0.1228156	0.0843873

ENSG00000249604	0.0000000	0.0000000	0.0000000	0.0000000	0.0073726
HADH	0.2957747	0.2300929	0.2141728	0.2534893	0.2128457
LEF1	0.0061431	0.0141747	0.0000000	0.0120878	0.0038927
LEF1-AS1	0.0059227	0.0016963	0.0021591	0.0124716	0.0000000
RPL34-DT	0.0274749	0.0310145	0.0118966	0.0186935	0.0563562
RPL34	3.2357419	3.2243769	3.0034302	3.1379408	2.9436531
OSTC	0.9084939	0.7595999	0.6678309	1.1166336	0.6663220
ENSG00000286136	0.0006597	0.0010069	0.0000000	0.0000000	0.0000000
COL25A1	0.0399737	0.0415613	0.0562476	0.0037517	0.0545561
SEC24B-AS1	0.0491692	0.0519299	0.0623420	0.0483601	0.0897512
SEC24B	0.2577164	0.2900225	0.2506833	0.2494072	0.4414736
MCUB	0.0470395	0.0587715	0.0333676	0.0348666	0.0523877
CASP6	0.0830860	0.0272608	0.0201737	0.0792881	0.0641166
PLA2G12A	0.4920622	0.4407013	0.5026307	0.4509084	0.3869679
CFI	0.0022535	0.0055288	0.0036697	0.0073674	0.0000000
GAR1-DT	0.0017647	0.0068038	0.0041971	0.0000000	0.0000000
GAR1	0.3490944	0.2366758	0.1743385	0.3783860	0.2616149
RRH	0.0034382	0.0012598	0.0071894	0.0032050	0.0073392
LRIT3	0.0000000	0.0000000	0.0000000	0.0000000	0.0000000
EGF	0.0118164	0.0155499	0.0000000	0.0694351	0.0065592
ELOVL6	0.2324580	0.3754299	0.4256023	0.1784350	0.5551880
ENSG00000288913	0.0039109	0.0103063	0.0267870	0.0179247	0.0156664
ENPEP	0.0068204	0.0075109	0.0061047	0.0119075	0.0159013
ENSG00000288692	0.0036536	0.0000000	0.0032497	0.0000000	0.0000000
ENSG00000248656	0.0009587	0.0020841	0.0000000	0.0000000	0.0000000
LINC02945	0.0000000	0.0014712	0.0000000	0.0036572	0.0000000
FAM241A	0.0110779	0.0187126	0.0354177	0.0339864	0.0067599
AP1AR-DT	0.0089597	0.0062460	0.0071353	0.0052402	0.0021475
AP1AR	0.3742871	0.3891004	0.4350831	0.3252689	0.3052739
TIFA	0.0365103	0.0588853	0.0861889	0.0427313	0.0870175
ALPK1	0.0139659	0.0086394	0.0095633	0.0243742	0.0115393
NEUROG2	0.0000000	0.0000000	0.0020267	0.0000000	0.0000000
NEUROG2-AS1	0.0038714	0.0026625	0.0011080	0.0040841	0.0045285
ZGRF1	0.0255685	0.0475700	0.0247831	0.0177540	0.0606757
LARP7	0.6767072	0.5753718	0.4003444	0.5832447	0.4219517
MIR302CHG	0.0035426	0.0018627	0.0000000	0.0000000	0.0000000
ENSG00000250046	0.0011298	0.0000000	0.0014559	0.0000000	0.0065149
ANK2	1.2241099	1.6171843	1.6572534	1.1798000	2.1886892
ENSG00000251126	0.0000000	0.0000000	0.0000000	0.0020830	0.0000000
ANK2-AS1	0.0038968	0.0054972	0.0131803	0.0031392	0.1322471
CAMK2D	0.8346861	0.7664969	0.6666025	0.7547996	1.0874313
ARSJ	0.1514168	0.0744354	0.0410585	0.2174603	0.0961740
UGT8	0.0307286	0.0498322	0.1320954	0.0253231	0.0597276
NDST4	0.1242087	0.1883457	0.1267333	0.0827162	0.4620958
ENSG00000287520	0.0000000	0.0000000	0.0000000	0.0000000	0.0123181
ENSG00000287290	0.0090699	0.0152811	0.0034878	0.0000000	0.0212449
ENSG00000286637	0.0000000	0.0010211	0.0000000	0.0017075	0.0000000
TRAM1L1	0.0652902	0.0713060	0.1009233	0.0841941	0.0493442
LINC01378	0.0021520	0.0052582	0.0096056	0.0036275	0.0000000
ENSG00000286269	0.0131728	0.0339929	0.0051279	0.0071471	0.0183915
ENSG00000288921	0.0086486	0.0084030	0.0105839	0.0000000	0.0231779
NDST3	0.2348486	0.4119961	0.2275600	0.1868625	0.5695758
SNHG8	0.4597949	0.4307485	0.4016741	0.3512443	0.3649098

PRSS12	0.0090354	0.0215929	0.0028426	0.0219042	0.0244308
ENSG00000291201	0.0081421	0.0083179	0.0186589	0.0125920	0.0236406
METTL14-DT	0.0096038	0.0104491	0.0097248	0.0051359	0.0282158
METTL14	0.2880098	0.2701054	0.2085576	0.2625871	0.2545605
SEC24D	0.1603481	0.1083589	0.0810408	0.1862045	0.1833391
SYNPO2	0.0275641	0.0293366	0.0223367	0.0280380	0.0541720
MYOZ2	0.0014114	0.0007691	0.0011236	0.0031683	0.0000000
USP53	0.3039766	0.1843708	0.1650149	0.3223711	0.1868323
C4orf3	1.3923140	1.2879142	1.1789447	1.3219193	1.0532237
ENSG00000291202	0.0287009	0.0380308	0.0441441	0.0153751	0.0300940
ENSG00000260091	0.0000000	0.0000000	0.0017369	0.0000000	0.0056651
ENSG00000291203	0.2296584	0.2502421	0.2940628	0.2409209	0.4852910
PDE5A	0.1123002	0.0688790	0.0653248	0.0525766	0.1622882
ENSG00000286241	0.0032103	0.0015682	0.0019392	0.0000000	0.0054431
MAD2L1	0.0686528	0.0799031	0.0801215	0.1042100	0.0480837
MAD2L1-DT	0.0349041	0.0467584	0.0242298	0.0231285	0.1148616
PRDM5	0.3331354	0.2140662	0.1470388	0.3171789	0.1953803
NDNF	0.6380127	0.3476701	0.2192667	1.0157677	0.3273575
NDNF-AS1	0.0000000	0.0000000	0.0000000	0.0019160	0.0033508
TNIP3	0.0009523	0.0152340	0.0095992	0.0000000	0.0000000
QRFPR	0.0048039	0.0089670	0.0250879	0.0019780	0.0185174
ANXA5	1.2592536	0.8419435	0.6291120	1.4495267	0.7826431
EXOSC9	0.2260141	0.2325481	0.2454103	0.2075850	0.2179728
CCNA2	0.0240126	0.0179223	0.0151304	0.0164045	0.0255934
BBS7	0.2805975	0.3236274	0.2412346	0.3124004	0.2862703
BBS7-DT	0.0000000	0.0000000	0.0000000	0.0000000	0.0044301
TRPC3	0.0350034	0.0582769	0.0370976	0.0199786	0.1283331
ENSG00000289342	0.0007225	0.0038224	0.0000000	0.0048570	0.0000000
BLTP1	0.6427483	0.8078663	0.9448255	0.5875993	1.1294686
ADAD1	0.0000000	0.0000000	0.0000000	0.0000000	0.0000000
BBS12	0.1003600	0.0751039	0.0555264	0.1231859	0.0794047
FGF2	0.1292360	0.1855986	0.2377978	0.0917692	0.2237800
ENSG00000273007	0.0012354	0.0037417	0.0000000	0.0035629	0.0000000
NUDT6	0.0977331	0.0757891	0.0728048	0.1650921	0.0488696
SPATA5	0.1013220	0.1082829	0.0883712	0.0871578	0.2277833
ENSG00000287951	0.0007089	0.0000000	0.0000000	0.0000000	0.0000000
SPRY1	0.1285864	0.0858253	0.0998599	0.1159469	0.1387765
LINC01091	0.0807587	0.0435640	0.0338313	0.0482430	0.0588398
LINC02516	0.0000000	0.0012294	0.0000000	0.0000000	0.0000000
ANKRD50	0.3517386	0.3874996	0.5215247	0.3159396	0.4312270
FAT4	0.0248958	0.0222875	0.0269439	0.0106063	0.0755570
ENSG00000286251	0.0000000	0.0000000	0.0021192	0.0000000	0.0000000
INTU	0.2764813	0.2849029	0.2809105	0.3252278	0.4997438
SLC25A31	0.0059712	0.0021772	0.0000000	0.0000000	0.0115775
HSPA4L	0.7165186	0.6944745	0.7042291	0.7261374	0.6917574
ENSG00000261668	0.0017365	0.0000000	0.0000000	0.0000000	0.0000000
PLK4	0.0090101	0.0104912	0.0173011	0.0262721	0.0286142
MFSD8	0.0897907	0.1052689	0.0569981	0.1096711	0.1567396
ABHD18	0.1361466	0.1400589	0.0925013	0.1546669	0.2200853
LARP1B	0.3215171	0.3907191	0.3799464	0.2373965	0.4662099
PGRMC2	0.5243747	0.4170754	0.3584530	0.6065805	0.3777459
LINC02615	0.0289204	0.0239280	0.0204265	0.0263565	0.0712730
ENSG00000273077	0.0009973	0.0013405	0.0031125	0.0016475	0.0000000

ENSG00000248802	0.0003629	0.0010884	0.0000000	0.0045021	0.0000000
ENSG00000289243	0.0012739	0.0069579	0.0000000	0.0000000	0.0048241
JADE1	0.3311020	0.2392600	0.2262955	0.2570514	0.2408574
SCLT1	0.2389549	0.2619576	0.2398398	0.2540506	0.5181284
C4orf33	0.0968396	0.1192495	0.1084049	0.0872350	0.0952311
LINC02465	0.0024522	0.0000000	0.0000000	0.0000000	0.0000000
ENSG00000251555	0.0007596	0.0000000	0.0000000	0.0000000	0.0037043
ENSG00000251598	0.0009283	0.0037377	0.0000000	0.0000000	0.0000000
LINC01256	0.0000000	0.0000000	0.0026342	0.0000000	0.0000000
ENSG00000251488	0.0058097	0.0097080	0.0037640	0.0050414	0.0361373
ENSG00000249513	0.0000000	0.0014966	0.0000000	0.0000000	0.0045817
PCDH10-DT	0.0893786	0.1195648	0.1381518	0.0605949	0.1443875
PCDH10	0.2872364	0.4439854	0.5719310	0.2751896	0.4942037
ENSG00000251388	0.0166617	0.0059242	0.0087929	0.0314554	0.0190900
ENSG00000251199	0.0017519	0.0047751	0.0042717	0.0092347	0.0163533
PABPC4L	0.0165227	0.0013272	0.0000000	0.0052710	0.0071244
ENSG00000248434	0.0000000	0.0000000	0.0009415	0.0000000	0.0000000
LINC02485	0.0038374	0.0022029	0.0023985	0.0000000	0.0000000
LINC02511	0.0011511	0.0052545	0.0028763	0.0000000	0.0125537
PCDH18	0.2393704	0.1281385	0.1198715	0.2480675	0.1145966
ENSG00000287144	0.0088311	0.0153552	0.0057407	0.0029272	0.0046972
LINC00616	0.0000000	0.0006347	0.0000000	0.0068008	0.0000000
SLC7A11-AS1	0.0124431	0.0053017	0.0006144	0.0031683	0.0202855
SLC7A11	0.2037801	0.1356825	0.0745605	0.2687482	0.1508213
LINC00498	0.0000000	0.0000000	0.0000000	0.0000000	0.0000000
LINC00499	0.0032218	0.0000000	0.0021247	0.0000000	0.0044642
ENSG00000250501	0.0005985	0.0000000	0.0000000	0.0000000	0.0124029
ENSG00000250195	0.0246943	0.0159068	0.0105506	0.0193907	0.0987507
NOCT	0.0261748	0.0354201	0.0638279	0.0339716	0.0979431
ELF2	0.5614903	0.6003451	0.5643301	0.4731728	0.7027287
ENSG00000288785	0.0021058	0.0084811	0.0079645	0.0026518	0.0000000
MGARP	0.0273713	0.0154593	0.0046472	0.0046065	0.0000000
NDUFC1	1.3052286	1.2122033	1.2749807	1.2119497	0.9805712
NAA15	0.3790333	0.3505046	0.3410758	0.3764825	0.4574392
RAB33B-AS1	0.1696680	0.2214680	0.1824720	0.1600578	0.3406804
RAB33B	0.2080151	0.2198758	0.1818776	0.1946620	0.2260270
SETD7	0.2108068	0.1630919	0.1239408	0.1865463	0.2301454
ENSG00000272717	0.0038890	0.0000000	0.0013222	0.0000000	0.0000000
ENSG00000286896	0.0000000	0.0000000	0.0020287	0.0000000	0.0046437
QKILA	0.0090969	0.0066865	0.0117274	0.0026458	0.0078223
MGST2	0.2435672	0.1453197	0.0929713	0.3214504	0.1452662
MAML3	0.3980070	0.3937622	0.3452550	0.3430698	1.1372862
ENSG00000286320	0.0011945	0.0008658	0.0000000	0.0000000	0.0023501
ENSG00000250698	0.0000000	0.0000000	0.0000000	0.0000000	0.0030106
SCOC	1.5532176	1.5654454	1.8548539	1.5490702	1.3040937
SCOC-AS1	0.0414566	0.0552793	0.0521749	0.0211719	0.1055449
CLGN	0.3096214	0.1864331	0.2418327	0.2741665	0.2981539
MGAT4D	0.0008598	0.0008560	0.0000000	0.0000000	0.0000000
ELMOD2	0.1953635	0.2054392	0.1953474	0.2248560	0.2394540
UCP1	0.0000000	0.0018827	0.0000000	0.0000000	0.0000000
ENSG00000287449	0.0019358	0.0023864	0.0032749	0.0000000	0.0052305
TBC1D9	0.4042020	0.6046722	0.5980567	0.3619793	0.6189367
ENSG00000248335	0.0000000	0.0000000	0.0000000	0.0000000	0.0041632

ENSG00000273472	0.0085477	0.0088766	0.0080862	0.0106758	0.0120952
RNF150	0.4100945	0.4829876	0.4577318	0.3287096	0.8584616
ZNF330	0.5408828	0.4025199	0.3858258	0.6052737	0.3816449
LINC02432	0.0017928	0.0010884	0.0000000	0.0000000	0.0016664
IL15	0.0027310	0.0016449	0.0033576	0.0034162	0.0055238
INPP4B	0.2413256	0.1898556	0.1619691	0.2410402	0.4702358
ENSG00000251248	0.0000000	0.0000000	0.0000000	0.0000000	0.0000000
ENSG00000249806	0.0038825	0.0005474	0.0000000	0.0000000	0.0201185
USP38-DT	0.2022207	0.0982517	0.0886261	0.1253281	0.1802146
USP38	0.2011029	0.1491265	0.1312111	0.1814968	0.1952140
GAB1	0.0566209	0.0766501	0.0618465	0.0723747	0.1442996
ENSG00000286771	0.0000000	0.0000000	0.0000000	0.0000000	0.0071630
SMARCA5-AS1	0.0017412	0.0071459	0.0000000	0.0035261	0.0042711
SMARCA5	1.3917428	1.1952231	1.2573015	1.3811239	1.1412620
ENSG00000251600	0.0656207	0.1057754	0.0599434	0.1077697	0.3179162
FREM3	0.0143857	0.0064919	0.0083111	0.0062284	0.0232250
GYPE	0.0056490	0.0045340	0.0065287	0.0000000	0.0179914
GYPB	0.0000000	0.0000000	0.0000000	0.0000000	0.0000000
ENSG00000285783	0.0452850	0.0387033	0.0300046	0.0352643	0.2326321
HHIP-AS1	0.0214471	0.0290047	0.0161428	0.0075613	0.0146711
HHIP	0.0283392	0.0379498	0.0309371	0.0171732	0.0932191
ENSG00000289314	0.0018370	0.0000000	0.0000000	0.0000000	0.0025607
ANAPC10	0.2329012	0.2634113	0.2595182	0.3176606	0.3707861
ENSG00000250406	0.0002724	0.0000000	0.0000000	0.0000000	0.0042562
ABCE1	0.5462290	0.6225849	0.6381665	0.5424154	0.5048159
OTUD4	0.1710795	0.1920012	0.1379168	0.1353680	0.2667831
ENSG00000286420	0.0022026	0.0007634	0.0013672	0.0000000	0.0000000
SMAD1	0.5479277	0.4900796	0.3624201	0.5572034	0.4073001
SMAD1-AS2	0.0000000	0.0000000	0.0000000	0.0000000	0.0039562
SMAD1-AS1	0.0008405	0.0033804	0.0000000	0.0000000	0.0000000
MMAA	0.0599227	0.0435483	0.0497545	0.0403059	0.0434483
ENSG00000248356	0.0003238	0.0000000	0.0000000	0.0000000	0.0000000
C4orf51	0.0010915	0.0009278	0.0000000	0.0000000	0.0080857
ZNF827	0.3612073	0.2993943	0.2950935	0.3809913	0.4291467
ENSG00000251687	0.0000000	0.0008011	0.0000000	0.0000000	0.0038318
LSM6	0.4162944	0.4402741	0.4881013	0.4699397	0.3549134
REELD1	0.0025325	0.0000000	0.0000000	0.0000000	0.0000000
SLC10A7	0.0884036	0.0781457	0.0628254	0.1080824	0.2274357
ENSG00000288998	0.0033711	0.0062091	0.0023164	0.0000000	0.0141402
POU4F2	0.0005211	0.0000000	0.0000000	0.0000000	0.0000000
TTC29	0.1476059	0.0876015	0.0520844	0.1980584	0.0451580
ENSG00000286371	0.0009671	0.0000000	0.0000000	0.0000000	0.0000000
EDNRA	0.0157778	0.0222956	0.0046463	0.0012986	0.0135359
TMEM184C-DT	0.0028640	0.0038920	0.0013454	0.0071046	0.0169535
TMEM184C	0.2236000	0.2721994	0.2856975	0.3053312	0.2757787
PRMT9	0.0649402	0.0747489	0.0560710	0.0920262	0.1034386
ARHGAP10	0.0328368	0.0142465	0.0221883	0.0126142	0.0643311
NR3C2	0.0525861	0.0523203	0.0397375	0.0171953	0.1720100
ENSG00000250354	0.0000000	0.0000000	0.0000000	0.0000000	0.0054500
ENSG00000287292	0.0070084	0.0199425	0.0110522	0.0021767	0.0561864
LINC02355	0.0000000	0.0057770	0.0029545	0.0050681	0.0000000
IQCM	0.0018452	0.0000000	0.0000000	0.0000000	0.0000000
DCLK2	0.4862181	0.5554243	0.6315951	0.5249384	0.8288636

LRBA	0.3316998	0.3719463	0.2992086	0.2474406	0.9438395
ENSG00000249690	0.0004145	0.0060658	0.0334921	0.0024504	0.0385942
MAB21L2	0.2962045	0.5920603	1.1890040	0.2242385	0.6366333
RPS3A	3.3550898	3.3162185	3.1392618	3.2408655	3.0416992
SH3D19	0.1994851	0.1738411	0.1513156	0.2139860	0.2211620
ENSG00000270681	0.0258445	0.0208314	0.0213362	0.0456427	0.0126248
PRSS48	0.0000000	0.0000000	0.0000000	0.0000000	0.0000000
ENSG00000270265	0.0029709	0.0034948	0.0000000	0.0015747	0.0000000
FHIP1A	0.0321623	0.0337130	0.0353931	0.0698842	0.0651796
GATB	0.0715958	0.0775322	0.0902193	0.0787247	0.0593700
ENSG00000251455	0.0007723	0.0000000	0.0000000	0.0000000	0.0000000
ENSG00000286066	0.0000000	0.0000000	0.0008833	0.0000000	0.0000000
ENSG00000270302	0.0000000	0.0000000	0.0000000	0.0000000	0.0000000
FBXW7	0.4116275	0.4924918	0.5854229	0.2555151	0.7451923
ENSG00000287555	0.0081757	0.0271118	0.0453620	0.0049878	0.0855026
MIR4453HG	0.0543830	0.0988667	0.1942301	0.0247698	0.0448858
TMEM154	0.0056965	0.0028310	0.0007102	0.0000000	0.0000000
TIGD4	0.0874882	0.0552405	0.0348399	0.0669227	0.0683552
ARFIP1	0.3663906	0.2415515	0.2293768	0.3407305	0.2894557
ENSG00000287642	0.0010375	0.0158174	0.0154043	0.0034082	0.0099992
FHDC1	0.0358598	0.0923977	0.1168222	0.0144523	0.1047851
TRIM2	1.0923090	1.3253695	1.3175677	1.0775800	1.3492280
MND1	0.0323944	0.0294852	0.0198112	0.0441875	0.0398618
TMEM131L	0.0575197	0.0770925	0.0607633	0.0428218	0.2246257
ENSG00000287216	0.0000000	0.0021486	0.0060980	0.0018058	0.0031524
ENSG00000290441	0.0010107	0.0038084	0.0000000	0.0000000	0.0000000
TLR2	0.0000000	0.0006384	0.0000000	0.0026652	0.0000000
RNF175	0.2993718	0.5166233	0.5038506	0.3344636	0.4046764
ENSG00000249309	0.0000000	0.0000000	0.0000000	0.0000000	0.0000000
SFRP2	0.0408526	0.0774978	0.0258870	0.0969230	0.0367768
ENSG00000280241	0.0159031	0.0178748	0.0113494	0.0136646	0.0482493
DCHS2	0.1981691	0.1516375	0.1307124	0.3730400	0.2751181
PLRG1	0.5843297	0.4281338	0.4203172	0.6401459	0.4175209
LRAT	0.1048358	0.0593731	0.0411595	0.0614494	0.0269557
RBM46	0.0008377	0.0049438	0.0043444	0.0000000	0.0000000
MAP9-AS1	0.0047715	0.0074886	0.0032972	0.0162072	0.0151233
NPY2R-AS1	0.0000000	0.0020532	0.0079544	0.0040415	0.0000000
NPY2R	0.0021968	0.0015539	0.0216459	0.0000000	0.0285684
MAP9	1.1603214	1.0264543	1.1539719	1.1615862	0.8614327
GUCY1A1	0.1037337	0.2467304	0.2505282	0.0758599	0.2062999
GUCY1B1	0.2108373	0.3345347	0.4889433	0.2502756	0.3557288
TDO2	0.0023851	0.0020473	0.0000000	0.0000000	0.0000000
CTSO	0.0570358	0.0195389	0.0066002	0.0911924	0.0157176
PDGFC	0.6297853	0.3617011	0.2286275	0.7715023	0.4870901
GLRB	0.1999296	0.2260819	0.2209254	0.1738858	0.2110866
GRIA2	0.3595865	0.5095489	0.6705638	0.3151146	1.0281252
ENSG00000286133	0.0031750	0.0000000	0.0000000	0.0000000	0.0034299
ENSG00000287226	0.0018884	0.0048470	0.0018005	0.0082670	0.0000000
GASK1B	0.9819563	0.5642623	0.3666369	1.3131802	0.5121750
GASK1B-AS1	0.1019671	0.0422675	0.0203437	0.0793872	0.0013524
ENSG00000250604	0.0040292	0.0008074	0.0000000	0.0035805	0.0000000
TMEM144	0.0475645	0.0444271	0.0366947	0.0620635	0.0543857
RXFP1	0.0017113	0.0000000	0.0000000	0.0000000	0.0055345

C4orf46	0.0409915	0.0449966	0.0590926	0.0455766	0.0381941
ETFDH	0.1452851	0.1513250	0.1224047	0.1432236	0.1443663
PPID	0.7302496	0.6882866	0.7483398	0.7206507	0.5269989
FNIP2	0.2417520	0.3916945	0.5073028	0.2012197	0.4790668
C4orf45	0.0082140	0.0371065	0.0522771	0.0283098	0.0314076
RAPGEF2	0.4552304	0.4550574	0.5138453	0.3790471	0.9504066
ENSG00000250180	0.0000000	0.0000000	0.0000000	0.0012567	0.0000000
LINC02233	0.0018695	0.0000000	0.0000000	0.0000000	0.0000000
FSTL5	0.1341027	0.1601457	0.4368990	0.1117664	0.6298215
ENSG00000249419	0.0000000	0.0000000	0.0000000	0.0000000	0.0058922
ENSG00000248431	0.0000000	0.0000000	0.0000000	0.0000000	0.0000000
ENSG00000250027	0.0034328	0.0014699	0.0012851	0.0000000	0.0000000
NAF1	0.2515635	0.1748055	0.1983235	0.2945765	0.2588930
NPY1R	0.0231654	0.0502110	0.0840358	0.0276416	0.1348782
NPY5R	0.0073543	0.0164117	0.0221797	0.0072834	0.0294645
TKTL2	0.0000000	0.0027561	0.0000000	0.0000000	0.0000000
TMA16	0.2986507	0.2052151	0.1982327	0.2294494	0.1977880
MARCHF1	0.5104659	0.6542354	0.5956382	0.3775243	1.4575954
ENSG00000273449	0.0015745	0.0019436	0.0018076	0.0000000	0.0000000
SMIM31	0.0204022	0.0029077	0.0023575	0.0325459	0.0000000
TRIM61	0.0411849	0.0481636	0.0313521	0.0262255	0.0555020
FAM218A	0.0407891	0.0661029	0.0773331	0.0636722	0.0459106
TMEM192	0.2118328	0.2415370	0.1854989	0.2478258	0.2269033
KLHL2	0.2235100	0.2328331	0.3160496	0.1779405	0.2858956
MSMO1	0.7683664	0.6818580	0.7513541	1.1566044	0.7245192
CPE	0.6109568	0.9096647	1.3656490	0.5643552	0.9688466
TLL1	0.0000000	0.0059285	0.0020662	0.0000000	0.0229101
ENSG00000249675	0.0000000	0.0012312	0.0013773	0.0000000	0.0093724
SPOCK3	0.4337664	0.7295173	0.5423220	0.3041292	1.0760227
DDX60	0.0072928	0.0071440	0.0013437	0.0126488	0.0000000
DDX60L	0.0118102	0.0045060	0.0020652	0.0165702	0.0090790
PALLD	0.7428656	0.5556889	0.4555399	0.8372898	0.6247244
ENSG00000249609	0.0033180	0.0069263	0.0077700	0.0031392	0.0000000
CBR4	0.2943536	0.2434829	0.2491766	0.2553288	0.4453661
SH3RF1	0.2216721	0.2212741	0.2294136	0.2133534	0.2913056
ENSG00000251171	0.0002736	0.0000000	0.0000000	0.0000000	0.0048079
NEK1	0.4993912	0.4413714	0.3479167	0.4722555	0.6957552
ENSG00000286302	0.0000000	0.0000000	0.0010104	0.0000000	0.0000000
CLCN3	0.7747251	0.8160500	0.8995892	0.8666980	0.9061583
HPF1	0.6053227	0.7678770	0.7924072	0.5975149	0.5728211
LINC02275	0.0000000	0.0013043	0.0000000	0.0000000	0.0000000
ENSG00000289508	0.0015644	0.0009796	0.0010357	0.0000000	0.0000000
MFAP3L	0.0818895	0.1017794	0.0660459	0.0895818	0.1086975
AADAT	0.1106601	0.1337540	0.1607052	0.1395773	0.1373355
GALNTL6	0.3810241	0.5073102	0.4390596	0.3835389	1.5742516
GALNT7-DT	0.0167518	0.0099073	0.0073450	0.0075088	0.0424679
GALNT7	0.2451295	0.2061105	0.1864509	0.3383785	0.2851361
ENSG00000248774	0.0208832	0.0330356	0.0255695	0.0078918	0.0325024
HMGB2	1.0336356	0.8767488	0.7403670	0.9612375	0.6915007
ENSG00000288728	0.0000000	0.0000000	0.0000000	0.0000000	0.0000000
SAP30-DT	0.2617641	0.1874511	0.1736135	0.2053954	0.1731295
SAP30	0.5432655	0.3764123	0.2853263	0.5082659	0.3546507
SCRG1	0.0571074	0.1151116	0.1009040	0.1454792	0.1064634

ENSG00000288025	0.0030535	0.0000000	0.0000000	0.0140298	0.0231046
ENSG00000286226	0.0000000	0.0014195	0.0000000	0.0000000	0.0074625
ENSG00000286039	0.0007171	0.0000000	0.0000000	0.0020019	0.0000000
HAND2-AS1	0.0003365	0.0006824	0.0000000	0.0000000	0.0000000
LINC02269	0.0000000	0.0000000	0.0000000	0.0000000	0.0000000
LINC02268	0.0291728	0.0695289	0.0167556	0.0205323	0.0373590
ENSG00000289210	0.0076406	0.0135376	0.0010813	0.0139628	0.0241590
FBXO8	0.2413194	0.1618998	0.1098436	0.2571946	0.1307571
CEP44	0.2752514	0.2226545	0.2080745	0.2000125	0.2390941
ENSG00000249875	0.0000000	0.0000000	0.0000000	0.0000000	0.0000000
HPGD	0.0095815	0.0152708	0.0170763	0.0095933	0.0396328
ENSG00000251584	0.0000000	0.0018257	0.0000000	0.0016697	0.0000000
GLRA3	0.0246925	0.0603483	0.1182207	0.0181434	0.1086863
ADAM29	0.0093470	0.0153029	0.0105165	0.0050257	0.0460447
ENSG00000289392	0.0009411	0.0011573	0.0039240	0.0000000	0.0000000
GPM6A	0.8311891	1.0416204	1.8928428	1.0811519	1.2313736
GPM6A-DT	0.0019438	0.0011287	0.0006144	0.0000000	0.0036851
WDR17	0.1479173	0.1736047	0.1817314	0.1729243	0.4216223
SPATA4	0.0436099	0.0368488	0.0369742	0.0238710	0.0143380
ASB5	0.0000000	0.0032739	0.0034733	0.0000000	0.0000000
SPCS3-AS1	0.0125906	0.0239485	0.0185059	0.0180593	0.0232006
ENSG00000289149	0.0019064	0.0043278	0.0104934	0.0029052	0.0000000
SPCS3	0.7396075	0.6567019	0.7317821	0.8613675	0.6446367
ENSG00000289520	0.0074073	0.0103328	0.0012348	0.0125110	0.0175453
ENSG00000248388	0.0000000	0.0016089	0.0000000	0.0000000	0.0000000
VEGFC	0.0134600	0.0192521	0.0121896	0.0122547	0.0249839
ENSG00000287544	0.0005919	0.0000000	0.0000000	0.0076089	0.0000000
NEIL3	0.0013092	0.0035874	0.0000000	0.0000000	0.0000000
AGA	0.2355528	0.1073983	0.0641430	0.4121671	0.0699184
AGA-DT	0.0185624	0.0139851	0.0052902	0.0277130	0.0262607
LINC01098	0.0077784	0.0077837	0.0000000	0.0077586	0.0164281
LINC01099	0.0000000	0.0014850	0.0000000	0.0000000	0.0059953
ENSG00000287083	0.0085473	0.0075139	0.0035905	0.0020988	0.0154002
ENSG00000287775	0.0000000	0.0000000	0.0000000	0.0000000	0.0000000
ENSG00000249883	0.0000000	0.0000000	0.0000000	0.0000000	0.0233303
LINC00290	0.0034307	0.0013102	0.0015172	0.0031443	0.0262141
LINC02500	0.0000000	0.0000000	0.0023329	0.0000000	0.0000000
ENSG00000286860	0.0000000	0.0000000	0.0000000	0.0000000	0.0194856
ENSG00000287948	0.0000000	0.0000000	0.0000000	0.0000000	0.0000000
TEMN3-AS1	0.0000000	0.0000000	0.0000000	0.0044778	0.0000000
TENM3-AS1	0.0369948	0.0387601	0.0211075	0.0332080	0.1057186
TENM3	0.2874151	0.3169246	0.2665968	0.3597545	0.9565739
ENSG00000290012	0.0033996	0.0000000	0.0000000	0.0078380	0.0082681
ENSG00000248816	0.0000000	0.0019506	0.0000000	0.0000000	0.0055516
ENSG00000272646	0.0009517	0.0038221	0.0068440	0.0015751	0.0034590
DCTD	0.3907823	0.3211698	0.2923104	0.4354100	0.2573656
WWC2	0.1694780	0.1119644	0.1069404	0.1452056	0.1986527
CDKN2AIP	0.3231600	0.2923084	0.2860836	0.3093330	0.1759223
ING2-DT	0.0109873	0.0109668	0.0022493	0.0208580	0.0420858
ING2	0.4323053	0.4125159	0.4762182	0.4282392	0.3861666
ENSG00000272744	0.0103161	0.0044805	0.0072428	0.0083241	0.0000000
RWDD4	0.3833940	0.3545446	0.3859684	0.3905277	0.2381452
TRAPPC11	0.2454846	0.2362048	0.2595364	0.2319465	0.3716799

STOX2	0.3673854	0.4342320	0.4686975	0.3105311	0.6659570
ENSG00000248206	0.0030231	0.0042795	0.0057783	0.0040559	0.0178804
ENPP6	0.0052717	0.0068213	0.0038849	0.0041811	0.0185033
LINC02363	0.0070075	0.0078136	0.0021203	0.0000000	0.0127832
LINC02362	0.0039360	0.0007858	0.0015119	0.0017550	0.0068681
IRF2	0.2744336	0.1991165	0.1016254	0.2768473	0.1655394
ENSG00000270426	0.0021450	0.0000000	0.0009597	0.0000000	0.0000000
IRF2-DT	0.0039805	0.0023291	0.0000000	0.0038387	0.0044531
ENSG00000286292	0.0058459	0.0088331	0.0046277	0.0048676	0.0044624
LINC02427	0.0410350	0.0333589	0.0292548	0.0379304	0.0513830
LINC02365	0.0000000	0.0000000	0.0000000	0.0000000	0.0000000
CASP3	0.4657442	0.5314376	0.4898140	0.4701827	0.3698786
PRIMPOL	0.1242584	0.1368391	0.0792086	0.1384836	0.1572703
CENPU	0.0448939	0.0346767	0.0219533	0.0724887	0.0358861
ACSL1	0.1099174	0.1160733	0.1523659	0.0609305	0.1495083
ENSG00000251139	0.0004916	0.0000000	0.0014097	0.0000000	0.0000000
ENSG00000286256	0.0000000	0.0000000	0.0000000	0.0000000	0.0097123
MIR3945HG	0.0014894	0.0000000	0.0000000	0.0049218	0.0019007
ENSG00000287183	0.0015551	0.0036110	0.0024670	0.0000000	0.0000000
SLC25A4	0.8467159	0.9471841	1.1703521	0.8088803	0.7714168
CFAP97	0.9746348	0.8446602	0.9594622	0.9626975	0.6457734
SNX25	0.1299209	0.1813860	0.1572902	0.1488979	0.3588590
LRP2BP	0.3305603	0.2143200	0.1452494	0.3196699	0.3045582
LRP2BP-AS1	0.0178451	0.0118820	0.0070277	0.0105047	0.0592694
ANKRD37	0.2448046	0.1874084	0.1845651	0.1847452	0.1733127
UFSP2	0.7006626	0.6383854	0.5208030	0.6394291	0.4893697
C4orf47	0.4220960	0.2484722	0.1773476	0.2931730	0.2551524
CCDC110	0.0830020	0.0562110	0.0293517	0.0873076	0.0243368
ENSG00000249679	0.1388598	0.1027865	0.0884632	0.1408886	0.0827462
PDLIM3	1.4085263	0.8857368	0.5996156	1.6500290	0.8403259
SORBS2	0.6235918	0.5956567	0.4780203	0.6023298	1.4813248
SORBS2-AS1	0.0026369	0.0011989	0.0015119	0.0040927	0.0059797
ENSG00000235902	0.0039001	0.0000000	0.0000000	0.0016697	0.0000000
TLR3	0.0016884	0.0007173	0.0041282	0.0040025	0.0000000
FAM149A	0.1094291	0.1926745	0.1827416	0.1108693	0.1427425
CYP4V2	0.1289799	0.1645128	0.2085635	0.1418003	0.1779341
KLKB1	0.0011330	0.0050694	0.0000000	0.0000000	0.0084239
F11	0.0019182	0.0035328	0.0000000	0.0000000	0.0089724
F11-AS1	0.0016621	0.0097099	0.0050227	0.0123595	0.0195984
MTNR1A	0.0035751	0.0114884	0.0032229	0.0068601	0.0040857
FAT1	0.5915546	0.3790172	0.4027208	1.2168269	0.4999681
LINC01060	0.0077602	0.0000000	0.0000000	0.0000000	0.0043989
FRG1-DT	0.0599532	0.0536889	0.0423174	0.0272586	0.0912911
FRG1	0.5937137	0.6447570	0.6322122	0.5341472	0.4595913
PLEKHG4B	0.0188026	0.0099691	0.0419735	0.0104078	0.0599929
CCDC127	0.2379343	0.2177712	0.2217985	0.2942206	0.2182015
SDHA	0.2553333	0.3363459	0.3864860	0.3063667	0.2803587
PDCD6-DT	0.0200280	0.0081863	0.0039280	0.0124112	0.0201042
PDCD6	0.7544204	0.5520233	0.4973335	0.7970586	0.5541491
AHRR	0.0519165	0.0361506	0.0273368	0.0480406	0.0936044
EXOC3-AS1	0.1662315	0.1480500	0.1320102	0.1359152	0.1237686
EXOC3	0.2348541	0.2100144	0.1807504	0.2195269	0.1937237
PP7080	0.0000000	0.0035950	0.0000000	0.0069041	0.0107194

SLC9A3	0.0109281	0.0038440	0.0031129	0.0071678	0.0014498
SLC9A3-AS1	0.0528075	0.0627795	0.0664701	0.0553402	0.1006697
CEP72-DT	0.0011805	0.0025727	0.0000000	0.0000000	0.0072113
CEP72	0.0219608	0.0205549	0.0378104	0.0084665	0.0448528
TPPP	0.2650152	0.2385813	0.2117615	0.3247450	0.2360545
ENSG00000289088	0.0003481	0.0034219	0.0019906	0.0085914	0.0000000
ZDHHC11B	0.0617699	0.0545257	0.0714460	0.0488807	0.2536474
ENSG00000288930	0.0041423	0.0031598	0.0155596	0.0126169	0.0000000
ZDHHC11	0.0137834	0.0218404	0.0196632	0.0255289	0.0425216
BRD9	0.2978388	0.3558207	0.3802911	0.3271620	0.3539427
TRIP13	0.0869843	0.0587013	0.0618751	0.0636911	0.0866074
ENSG00000287600	0.0048476	0.0056280	0.0065252	0.0029736	0.0062307
LINC02982	0.0004580	0.0025582	0.0000000	0.0000000	0.0000000
NKD2	0.0176956	0.0413655	0.0483920	0.0114303	0.0184364
SLC12A7	0.0410977	0.0248441	0.0438676	0.0457469	0.0308064
CLPTM1L	0.4334466	0.3645147	0.3785265	0.4164395	0.3490470
ENSG00000286388	0.0725928	0.0599361	0.0314587	0.0578818	0.0288789
SLC6A3	0.0000000	0.0000000	0.0000000	0.0000000	0.0000000
LPCAT1	0.1430170	0.1325573	0.2635421	0.1338559	0.1705763
ENSG00000251532	0.0009913	0.0000000	0.0010569	0.0000000	0.0042989
ENSG00000291055	0.0428447	0.0588579	0.0556257	0.0350834	0.1346062
LINC03067	0.0082190	0.0075465	0.0055323	0.0101305	0.0046088
ENSG00000291079	0.2169270	0.2435705	0.2357365	0.2566846	0.2351450
MRPL36	0.4321603	0.4227612	0.4226353	0.4171705	0.2585227
NDUFS6	0.8852916	0.9842444	1.0588836	0.8648077	0.7773421
IRX4	0.0019952	0.0012005	0.0000000	0.0000000	0.0000000
IRX4-AS1	0.0000000	0.0000000	0.0000000	0.0000000	0.0000000
ENSG00000248994	0.0193698	0.0073855	0.0057777	0.0184385	0.0103109
IRX2	0.0405443	0.0426419	0.0526380	0.0068952	0.0489852
IRX2-DT	0.0334920	0.0499125	0.0262289	0.0174606	0.0557291
ENSG00000289075	0.0000000	0.0009601	0.0000000	0.0021653	0.0000000
LINC01019	0.0008793	0.0000000	0.0000000	0.0000000	0.0048707
IRX1	0.0705623	0.0470496	0.0395759	0.0892748	0.0643751
ENSG00000248973	0.0022851	0.0018463	0.0000000	0.0000000	0.0064609
ADAMTS16-DT	0.0285759	0.0104157	0.0166086	0.0424085	0.0257277
ADAMTS16	0.2419978	0.1314197	0.1068094	0.4921817	0.1982454
ENSG00000250866	0.0000000	0.0000000	0.0000000	0.0000000	0.0000000
ENSG00000286753	0.0187752	0.0219009	0.0186995	0.0228992	0.0195806
ICE1	0.4158445	0.4102866	0.4052580	0.4596092	0.5740563
LINC02145	0.0030064	0.0009234	0.0000000	0.0000000	0.0056190
MED10	0.6681225	0.6258154	0.6739126	0.6541320	0.5342121
ENSG00000287031	0.0006437	0.0000000	0.0021831	0.0032682	0.0000000
UBE2QL1	0.1381511	0.2867976	0.3672851	0.0820876	0.2631858
LINC01018	0.0107901	0.0248463	0.0167434	0.0150374	0.0126972
NSUN2	0.1887038	0.1384308	0.1529837	0.1701688	0.1704771
SRD5A1	0.3910301	0.6359819	0.8011153	0.3548156	0.4211006
LINC02102	0.0000000	0.0010146	0.0000000	0.0031040	0.0096389
TENT4A	0.0662910	0.0891127	0.0731449	0.0631540	0.2664251
LINC02236	0.0053029	0.0120330	0.0039264	0.0024989	0.0120168
LINC02196	0.0076592	0.0020628	0.0026409	0.0036229	0.0103597
ENSG00000290510	0.0035209	0.0008508	0.0055356	0.0015115	0.0000000
ADCY2	1.2380491	0.9142974	0.6432439	1.1536140	1.2113024
ENSG00000250761	0.0052963	0.0008641	0.0094616	0.0077440	0.0397396

CFAP90	1.3370992	0.9284548	0.7308084	1.2692779	0.8124695
MTRR	0.1423772	0.1276764	0.0861869	0.1681024	0.1448865
FASTKD3	0.0406901	0.0473730	0.0274684	0.0687816	0.0183754
ENSG00000251168	0.0000000	0.0000000	0.0000000	0.0000000	0.0060675
ENSG00000288002	0.0215438	0.0253688	0.0140020	0.0404842	0.0792773
LINC02226	0.0069273	0.0019463	0.0050995	0.0041153	0.0119160
ENSG00000272049	0.0056063	0.0000000	0.0000000	0.0028329	0.0000000
MIR4458HG	0.1849697	0.2007014	0.1852787	0.1970329	0.1197757
ENSG00000250619	0.0091469	0.0173944	0.0175678	0.0062892	0.0501658
SEMA5A	0.0821261	0.0910634	0.1436259	0.1031349	0.1666324
ENSG00000248537	0.0000000	0.0014137	0.0000000	0.0000000	0.0000000
SNHG18	0.0391706	0.0434604	0.0168495	0.1013958	0.0447381
ENSG00000248525	0.0003422	0.0014276	0.0000000	0.0000000	0.0023834
TAS2R1	0.0007030	0.0013594	0.0000000	0.0000000	0.0000000
LINC02112	0.0109657	0.0063610	0.0044992	0.0096095	0.0455659
LINC02221	0.0000000	0.0000000	0.0000000	0.0000000	0.0045621
ENSG00000249807	0.0000000	0.0000000	0.0000000	0.0000000	0.0000000
ENSG00000272417	0.0000000	0.0000000	0.0000000	0.0000000	0.0024177
ATPSCKMT	0.0823664	0.0639387	0.0744487	0.0990310	0.1184303
ENSG00000248968	0.0004851	0.0000000	0.0010726	0.0000000	0.0000000
CCT5	1.1827036	1.0743646	1.2346281	1.2390432	0.9043254
ENSG00000271980	0.0078648	0.0060073	0.0000000	0.0076657	0.0061992
CMBL	0.8069449	0.4848978	0.3572325	0.9246250	0.3822007
MARCHF6-DT	0.1532114	0.1113893	0.0860681	0.1180659	0.0793371
MARCHF6	0.7800065	0.8191724	0.8745500	0.8083004	1.0340417
ROPN1L-AS1	0.0007085	0.0000000	0.0016147	0.0012028	0.0000000
ROPN1L	0.2005680	0.1357773	0.0843673	0.2283298	0.1441854
LINC01513	0.0192301	0.0012831	0.0019256	0.0248348	0.0103529
LINC02212	0.0000000	0.0000000	0.0000000	0.0000000	0.0000000
LINC02213	0.0025358	0.0000000	0.0018564	0.0000000	0.0084808
ANKRD33B	0.0315198	0.0378461	0.0141030	0.0167612	0.0231741
DAP	0.5552962	0.3853001	0.2597083	0.5636857	0.3198968
DAP-DT	0.0038239	0.0009214	0.0030911	0.0026290	0.0102958
CTNND2	0.6337397	0.8485998	0.9903322	0.6369770	1.6925960
DNAH5	0.1171185	0.0666005	0.0285133	0.1558632	0.1281800
ENSG00000251423	0.0000000	0.0000000	0.0000000	0.0000000	0.0000000
TRIO	0.4038521	0.5748463	0.4882313	0.3297153	1.3068828
OTULINL	0.2332070	0.1580503	0.1180785	0.3152707	0.0988855
OTULIN-DT	0.0059533	0.0067154	0.0195737	0.0000000	0.0268509
OTULIN	0.0883966	0.0784370	0.0792387	0.0807892	0.0668052
ANKH	0.1189482	0.1824692	0.2209604	0.0789222	0.3143941
ENSG00000286970	0.0000000	0.0010598	0.0000000	0.0000000	0.0033679
ANKH-DT	0.0012648	0.0000000	0.0000000	0.0000000	0.0029223
FBXL7	0.3715929	0.2575389	0.1776168	0.3021234	0.3923776
CTD-2350J17.1	0.0000000	0.0014812	0.0000000	0.0000000	0.0047101
MARCHF11	0.0495777	0.0786757	0.1094196	0.0195019	0.1810420
MARCHF11-AS1	0.0000000	0.0000000	0.0000000	0.0000000	0.0000000
MARCHF11-DT	0.0030494	0.0042478	0.0019382	0.0000000	0.0055597
LINC02150	0.0032575	0.0062128	0.0074859	0.0032481	0.0000000
ZNF622	0.2955726	0.3029343	0.3112662	0.2452361	0.2653669
ENSG00000289112	0.0037541	0.0096812	0.0063658	0.0054010	0.0100915
RETREG1	0.3419154	0.3582216	0.4938899	0.4386242	0.4359790
RETREG1-AS1	0.0027567	0.0079480	0.0083017	0.0000000	0.0033534

MYO10	0.2204065	0.1444745	0.1207733	0.2679975	0.1951600
BASP1	2.0398983	2.6230708	2.9561669	2.0692156	2.2998885
BASP1-AS1	0.1777606	0.3663929	0.3468053	0.1325328	0.6084581
ENSG00000271892	0.0053619	0.0180321	0.0140428	0.0038717	0.0159286
ENSG00000248223	0.0039716	0.0049527	0.0088118	0.0000000	0.0000000
LINC02217	0.0022695	0.0023769	0.0000000	0.0000000	0.0169397
LINC02218	0.0012865	0.0016629	0.0045368	0.0000000	0.0074787
LINC02223	0.0495511	0.0770314	0.0821700	0.0551838	0.1356967
LINC02100	0.0000000	0.0011346	0.0000000	0.0000000	0.0016028
CDH18	0.4203293	0.3131158	0.2699761	0.3403339	0.9219347
CDH18-AS1	0.0009750	0.0014229	0.0000000	0.0062584	0.0000000
LINC02241	0.0052815	0.0028061	0.0000000	0.0000000	0.0546241
ENSG00000286751	0.0009980	0.0051965	0.0000000	0.0025371	0.0027595
ENSG00000249359	0.0000000	0.0000000	0.0000000	0.0000000	0.0021475
GUSBP1	0.1113317	0.1216483	0.1155578	0.1155793	0.3454449
ENSG00000272130	0.0006071	0.0000000	0.0000000	0.0000000	0.0062626
ENSG00000253766	0.0125902	0.0196451	0.0048037	0.0067618	0.0318103
CDH12	0.2150100	0.3583251	0.1672592	0.2015044	1.0745729
ENSG00000290530	0.0000000	0.0000000	0.0000000	0.0000000	0.0000000
ENSG00000286961	0.0026578	0.0021250	0.0013013	0.0000000	0.0092640
LINC02899	0.0000000	0.0027164	0.0010176	0.0038283	0.0000000
CDH10	0.1800727	0.2539283	0.1927010	0.2432625	0.5067398
ENSG00000251294	0.0021014	0.0058284	0.0000000	0.0000000	0.0270075
LINC02228	0.0015098	0.0014749	0.0000000	0.0000000	0.0000000
LINC02211	0.0038928	0.0025236	0.0045314	0.0068977	0.0000000
CDH9	0.0092222	0.0050408	0.0173815	0.0000000	0.0385284
PURPL	0.0026885	0.0044181	0.0000000	0.0046648	0.0158313
ENSG00000250453	0.0019678	0.0023468	0.0000000	0.0000000	0.0108325
ENSG00000286432	0.0017220	0.0014863	0.0000000	0.0058843	0.0000000
LINC02109	0.0000000	0.0000000	0.0020099	0.0000000	0.0000000
ENSG00000287176	0.0033776	0.0078731	0.0000000	0.0000000	0.0000000
ENSG00000254138	0.0008018	0.0030753	0.0000000	0.0000000	0.0143501
CDH6	0.3613133	0.3675397	0.2670121	0.4525388	0.4864269
DROSHA	0.1797011	0.2056971	0.2866653	0.1882317	0.3338593
C5orf22	0.2903610	0.3922836	0.4738580	0.2327719	0.3204303
PDZD2	0.0536705	0.0977414	0.0506884	0.0326433	0.3684711
ENSG00000250764	0.0047485	0.0012840	0.0059341	0.0000000	0.0317258
GOLPH3	0.4521272	0.3546132	0.3828311	0.3932242	0.3735718
GOLPH3-DT	0.0424839	0.0558572	0.0315073	0.0232591	0.0300100
MTMR12	0.1794897	0.1571529	0.1478121	0.0955933	0.2698927
ZFR	1.1726199	1.1326336	1.0891704	1.1757087	1.0135931
SUB1	1.6579481	1.5408818	1.6294842	1.6833573	1.2686269
NPR3	0.0534768	0.1257546	0.0704180	0.0561053	0.0634701
ENSG00000250697	0.0016176	0.0000000	0.0000000	0.0000000	0.0015298
ENSG00000249102	0.0018653	0.0000000	0.0000000	0.0000000	0.0000000
TARS1-DT	0.0248477	0.0331378	0.0378837	0.0266322	0.0346119
TARS1	0.9680066	0.8585851	0.8461317	0.9076741	0.7365991
ADAMTS12	0.1278937	0.0736070	0.0594884	0.1484449	0.1379813
RXFP3	0.0054633	0.0011294	0.0013627	0.0000000	0.0000000
SLC45A2	0.0088099	0.0046672	0.0044319	0.0021849	0.0348303
AMACR	0.1921190	0.1512970	0.1356792	0.1999145	0.1126794
C1QTNF3	0.0284036	0.0328343	0.0346047	0.0520259	0.0704600
ENSG00000290548	0.0642523	0.0618197	0.0694525	0.0583356	0.3734351

ENSG00000286543	0.0123898	0.0068852	0.0021138	0.0000000	0.0130645
RAI14-DT	0.0054485	0.0032857	0.0000000	0.0000000	0.0000000
RAI14	0.9233510	0.6661867	0.4263224	0.9251603	0.7404730
TTC23L-AS1	0.0511989	0.0475276	0.0323532	0.0199233	0.0371652
TTC23L	0.1991931	0.1163196	0.1118639	0.1980999	0.1682315
RAD1	0.1816599	0.1592327	0.1881210	0.1821492	0.1676417
BRIX1	0.3294405	0.3017329	0.2854195	0.3029235	0.3140385
DNAJC21	0.6152685	0.6335000	0.7764489	0.6268157	0.5951189
AGXT2	0.0000000	0.0000000	0.0000000	0.0000000	0.0000000
PRLR	0.0021349	0.0050521	0.0131970	0.0082230	0.0397065
SPEF2	0.3118919	0.2561452	0.1582816	0.3313263	0.3078309
ENSG00000248969	0.0017717	0.0060523	0.0000000	0.0000000	0.0158485
CAPSL	0.1660067	0.0646569	0.0486269	0.1086830	0.1010412
UGT3A2	0.0016757	0.0000000	0.0000000	0.0000000	0.0000000
LMBRD2	0.1784839	0.2349603	0.2467420	0.1746312	0.2993003
SKP2	0.1216491	0.0835465	0.0626226	0.0953170	0.1207096
NADK2	0.7551849	0.5009089	0.4113549	0.7527523	0.6046230
RANBP3L	0.1424490	0.0874893	0.0560551	0.2186438	0.0488070
SLC1A3	0.6443540	0.3478125	0.2137987	1.2567678	0.3899756
ENSG00000274441	0.0000000	0.0000000	0.0000000	0.0000000	0.0028353
SLC1A3-AS1	0.0036282	0.0018488	0.0000000	0.0000000	0.0000000
ENSG00000272103	0.0028001	0.0091798	0.0024411	0.0052885	0.0088772
NIPBL-DT	0.1532943	0.2300920	0.3052616	0.1311295	0.2189767
NIPBL	0.5978120	0.5849501	0.6100708	0.5503303	0.8492526
CPLANE1	0.3662525	0.3841167	0.4460611	0.2810768	0.7416201
CPLANE1-AS1	0.0095179	0.0072640	0.0110586	0.0028343	0.0055669
NUP155	0.1312506	0.1417518	0.1306090	0.1059018	0.1317577
WDR70	0.2274295	0.2821471	0.2153044	0.2352732	0.5801103
GDNF-AS1	0.0019223	0.0169603	0.0066038	0.0098610	0.0072426
GDNF	0.0013765	0.0052072	0.0064943	0.0027934	0.0123126
EGFLAM	0.0043289	0.0023782	0.0074466	0.0000000	0.0000000
LIFR	0.4527360	0.3757480	0.2581215	0.6300012	0.4304919
LIFR-AS1	0.0871812	0.0646609	0.0526247	0.0664540	0.0901133
OSMR-DT	0.0016784	0.0000000	0.0000000	0.0000000	0.0000000
OSMR	0.0007628	0.0010181	0.0000000	0.0193341	0.0016028
RICTOR	0.1930155	0.2227021	0.1607804	0.1681304	0.4445130
ENSG00000289196	0.0044315	0.0011114	0.0000000	0.0000000	0.0000000
FYB1	0.0000000	0.0000000	0.0031267	0.0000000	0.0000000
C9	0.0023099	0.0000000	0.0046441	0.0000000	0.0026726
DAB2	0.4984685	0.2100805	0.0988630	0.2843441	0.2478060
LINC02104	0.0046724	0.0045559	0.0081573	0.0000000	0.0064941
TTC33	0.2650862	0.3010281	0.3078055	0.2818993	0.2860965
ENSG00000289540	0.0012354	0.0050023	0.0000000	0.0000000	0.0000000
PTGER4	0.0052935	0.0103516	0.0248542	0.0056222	0.0241233
PRKAA1	0.1290064	0.0984801	0.0688029	0.1080211	0.0975743
ENSG00000288911	0.0229825	0.0101152	0.0084659	0.0152587	0.0223574
RPL37	3.0989582	3.0503956	2.8309089	2.9860456	2.8008523
CARD6	0.0000000	0.0011128	0.0000000	0.0000000	0.0000000
C7	0.0031820	0.0023963	0.0000000	0.0087950	0.0059925
C6	0.0058534	0.0012382	0.0051053	0.0000000	0.0000000
PLCXD3	0.3381580	0.3618819	0.2751996	0.2207917	0.4447459
OXCT1	0.3930053	0.5246327	0.7660471	0.3164484	0.4856781
ENSG00000286164	0.0000000	0.0000000	0.0000000	0.0000000	0.0000000

OXCT1-AS1	0.0244989	0.0137387	0.0206042	0.0420591	0.0071991
RIMOC1	0.3916269	0.3424500	0.3801048	0.3555520	0.2592803
FBXO4	0.0308143	0.0241977	0.0323230	0.0550899	0.0246257
GHR	0.1060996	0.0677495	0.0457488	0.1141952	0.1009496
CCDC152	0.0955581	0.1128332	0.1014672	0.1062419	0.0671419
SELENOP	0.0545000	0.0351444	0.0165164	0.0466084	0.0392959
ENSG00000272234	0.0000000	0.0008476	0.0010706	0.0000000	0.0000000
ENSG00000286271	0.0000000	0.0010205	0.0000000	0.0000000	0.0000000
ENSG00000287263	0.0000000	0.0013989	0.0018236	0.0025617	0.0000000
ENSG00000271788	0.0000000	0.0002840	0.0000000	0.0000000	0.0000000
ANXA2R-OT1	0.0242391	0.0145945	0.0106656	0.0230040	0.0164363
ENSG00000251131	0.0000000	0.0009258	0.0000000	0.0000000	0.0000000
ANXA2R	0.0000000	0.0000000	0.0000000	0.0000000	0.0000000
ANXA2R-AS1	0.0071077	0.0007873	0.0000000	0.0122907	0.0087427
ZNF131	0.2652997	0.3219137	0.3363355	0.2793591	0.4738789
NIM1K	0.1238119	0.1146844	0.0974012	0.1506723	0.1787961
HMGCS1	1.0995643	0.9745878	1.0657914	1.3449533	1.0255855
ENSG00000261604	0.0047209	0.0064411	0.0063979	0.0045057	0.0209510
CCL28	0.0069497	0.0017035	0.0000000	0.0079437	0.0049638
TMEM267	0.1461710	0.1281161	0.1205994	0.1462676	0.1717881
ENSG00000249492	0.0037213	0.0084367	0.0020257	0.0000000	0.0213230
C5orf34	0.0283642	0.0319415	0.0226432	0.0253187	0.0290950
C5orf34-AS1	0.0000000	0.0000000	0.0000000	0.0000000	0.0000000
ENSG00000248240	0.0000000	0.0037180	0.0000000	0.0000000	0.0000000
PAIP1	0.8359863	0.8032894	0.8705121	0.8390154	0.6099616
NNT-AS1	0.4500677	0.4388217	0.4086627	0.4161097	0.3323974
NNT	0.2971653	0.3137348	0.3370259	0.2764151	0.3492383
FGF10	0.0407860	0.1169792	0.0255624	0.0180056	0.0806985
FGF10-AS1	0.0029751	0.0009676	0.0019105	0.0000000	0.0000000
LINC02224	0.0051488	0.0021197	0.0000000	0.0033262	0.0233954
MRPS30-DT	0.1936266	0.1467532	0.1043486	0.1308092	0.1207085
ENSG00000248779	0.0000000	0.0000000	0.0000000	0.0000000	0.0000000
MRPS30	0.3294179	0.3446600	0.3726834	0.2577296	0.2417367
ENSG00000272335	0.0581379	0.0891506	0.0920939	0.0274385	0.1035311
HCN1	0.0768902	0.1235897	0.1362945	0.1111721	0.3567071
EMB	0.0478895	0.1116817	0.1432752	0.0300000	0.1557214
PARP8	0.2231221	0.2460749	0.2927592	0.2262331	0.5365620
ENSG00000289056	0.0153298	0.0103137	0.0125271	0.0103367	0.0155637
ENSG00000250360	0.0010179	0.0032835	0.0000000	0.0031874	0.0000000
LINC02106	0.0038443	0.0177129	0.0042669	0.0080714	0.0100324
ISL1-DT	0.0668488	0.1737472	0.0642284	0.0600370	0.0930775
ISL1	0.4006914	1.0849550	0.4006228	0.4111515	0.6350087
ENSG00000288035	0.0256328	0.0715343	0.0231368	0.0185005	0.0558237
PELO-AS1	0.0151012	0.0087962	0.0000000	0.0072679	0.0035216
PELO	0.2226680	0.2036607	0.1998471	0.1891071	0.1845133
ITGA1	0.0202734	0.0163957	0.0139984	0.0219280	0.0254037
ITGA2-AS1	0.0000000	0.0000000	0.0000000	0.0000000	0.0000000
ITGA2	0.0215383	0.0204933	0.0206628	0.1047657	0.0132514
ENSG00000272123	0.0040197	0.0031334	0.0035214	0.0000000	0.0022244
MOCS2	0.5678562	0.5822769	0.6705520	0.6061300	0.3855582
MOCS2-DT	0.0509885	0.0227520	0.0311377	0.0576091	0.0489813
FST	0.0084349	0.0138197	0.0319372	0.0143711	0.0038147
NDUFS4	0.8386511	1.0424256	1.0787908	0.8272828	0.8009738

LINC02105	0.0000000	0.0000000	0.0000000	0.0000000	0.0000000
ENSG00000272416	0.0023972	0.0026068	0.0010064	0.0000000	0.0000000
ARL15	0.2666480	0.3787873	0.3686241	0.2790930	0.8927883
ENSG00000287087	0.0007354	0.0005638	0.0000000	0.0000000	0.0000000
ENSG00000288957	0.0066267	0.0011635	0.0086218	0.0047604	0.0640623
LINC01033	0.0010832	0.0012936	0.0000000	0.0021445	0.0273112
SNX18	0.2078995	0.1561700	0.1234091	0.1936578	0.2020226
ENSG00000287367	0.0039625	0.0013191	0.0011570	0.0000000	0.0056320
ENSG00000289060	0.0007038	0.0014545	0.0000000	0.0016951	0.0000000
CDC20B	0.0008804	0.0013933	0.0030126	0.0000000	0.0000000
GPX8	0.4669317	0.2240015	0.1501372	0.5959141	0.2503526
CCNO	0.0035318	0.0028249	0.0034419	0.0000000	0.0112413
CCNO-DT	0.0053528	0.0005292	0.0027466	0.0000000	0.0100070
DHX29	0.4439634	0.3441741	0.4067473	0.4258117	0.4637453
MTREX	0.3668963	0.3862919	0.4310935	0.3461550	0.4765187
PLPP1	0.3549163	0.2406719	0.2183042	0.5287632	0.3554006
SLC38A9	0.0436934	0.0588025	0.0449793	0.0414201	0.1708759
DDX4	0.0028770	0.0017554	0.0000000	0.0000000	0.0000000
IL31RA	0.0032072	0.0020618	0.0000000	0.0000000	0.0000000
IL6ST	1.1614488	0.8269946	0.7188190	1.3701374	0.8209804
ENSG00000249236	0.0002408	0.0037016	0.0017124	0.0000000	0.0396459
IL6ST-DT	0.0396021	0.0340707	0.0201217	0.0173306	0.0115935
ANKRD55	0.0318243	0.0328461	0.0434515	0.0211071	0.0562937
LINC01948	0.0003619	0.0009965	0.0034767	0.0000000	0.0000000
MAP3K1	0.0456541	0.0609124	0.0322454	0.0377490	0.1413358
ENSG00000237705	0.0000000	0.0027789	0.0000000	0.0000000	0.0000000
ENSG00000225230	0.0000000	0.0000000	0.0000000	0.0037062	0.0043337
SETD9	0.0779965	0.0818637	0.0623932	0.0471266	0.0652643
MIER3	0.1480029	0.1622213	0.1250680	0.1111265	0.1526594
ENSG00000235635	0.0007963	0.0000000	0.0000000	0.0000000	0.0000000
ENSG00000289152	0.0018273	0.0020093	0.0000000	0.0024755	0.0018550
GPBP1	0.8509946	0.7925523	0.8463273	0.7431897	0.7993050
RMEL3	0.0000000	0.0019484	0.0000000	0.0000000	0.0000000
LINC02101	0.0003764	0.0000000	0.0000000	0.0000000	0.0000000
PLK2	0.1612575	0.2326258	0.3613810	0.1977434	0.2368208
RAB3C	0.7111175	1.1506677	1.5557542	0.5758972	1.2112372
ENSG00000248475	0.0007569	0.0000000	0.0000000	0.0000000	0.0278856
ENSG00000248733	0.0000000	0.0000000	0.0000000	0.0000000	0.0040262
PDE4D	0.5956323	0.5202377	0.5428238	0.5300302	1.5356599
ENSG00000247345	0.0016557	0.0026108	0.0028368	0.0000000	0.0045212
ENSG00000248935	0.0000000	0.0000000	0.0000000	0.0000000	0.0000000
PART1	0.0041018	0.0094814	0.0016729	0.0029331	0.0000000
DEPDC1B	0.0073328	0.0118822	0.0187720	0.0133512	0.0081281
ENSG00000287434	0.0030142	0.0000000	0.0000000	0.0000000	0.0000000
ELOVL7	0.0074615	0.0089438	0.0076624	0.0076961	0.0588874
ERCC8	0.1665410	0.2315553	0.2489911	0.1959400	0.2539417
NDUFAF2	0.6493256	0.7800254	0.8948624	0.4794826	0.7096662
SMIM15	0.5956567	0.5324236	0.5360989	0.6381332	0.4204099
SMIM15-AS1	0.0354540	0.0367373	0.0214480	0.0191435	0.0729674
LINC02057	0.0012063	0.0010742	0.0000000	0.0000000	0.0041632
ENSG00000288936	0.0004145	0.0031321	0.0007329	0.0000000	0.0042562
ZSWIM6	0.2419912	0.3001222	0.3184737	0.1920338	0.8497530
C5orf64	0.0050962	0.0066405	0.0000000	0.0000000	0.0059661

ENSG00000248529	0.0000000	0.0000000	0.0000000	0.0000000	0.0000000
KIF2A	1.3983562	1.5304216	1.7286603	1.3429776	1.2848125
DIMT1	0.1530641	0.1228238	0.1404958	0.1347672	0.1177499
IPO11	0.2614069	0.1984052	0.1429847	0.2541649	0.3742910
LRRC70	0.0028583	0.0053115	0.0066847	0.0069975	0.0101495
HTR1A	0.0010907	0.0067008	0.0127363	0.0000000	0.0097259
ENSG00000248285	0.0000000	0.0000000	0.0023088	0.0016517	0.0058801
RNF180	0.1572060	0.2635102	0.2451203	0.1425398	0.3929434
RGS7BP	0.1636720	0.3959578	0.2603270	0.1300228	0.3561286
SHISAL2B	0.0443013	0.1001211	0.0469402	0.0489002	0.0680576
SREK1IP1	0.5108037	0.5326586	0.5293822	0.4826838	0.4164558
CWC27	0.5857613	0.5414635	0.4911847	0.5108160	0.6497908
ADAMTS6	0.0752768	0.0998310	0.0642595	0.0647225	0.1650870
CENPK	0.0373758	0.0324521	0.0221585	0.0726568	0.0345240
PPWD1	0.2982600	0.2116452	0.1476148	0.3003687	0.2286859
TRIM23	0.4467093	0.4261220	0.4721312	0.4467828	0.4076611
TRAPPC13	0.1278486	0.1612782	0.1596985	0.1228842	0.1781856
SHLD3	0.0287011	0.0332385	0.0340232	0.0471006	0.0143513
SGTB	0.3663501	0.4159241	0.4495278	0.3033402	0.3864744
NLN	0.3878734	0.5723058	0.6711710	0.3015880	0.5930089
ERBIN	0.5652778	0.4258350	0.3919977	0.4721892	0.6813626
ERBIN-DT	0.0056833	0.0021484	0.0032608	0.0042162	0.0000000
ENSG00000286314	0.0011037	0.0000000	0.0022360	0.0000000	0.0037811
ENSG00000285999	0.0072471	0.0021973	0.0000000	0.0000000	0.0105037
SREK1	0.6003151	0.5307503	0.4461411	0.6307661	0.4899211
LINC02065	0.0000000	0.0000000	0.0019963	0.0000000	0.0000000
ENSG00000251391	0.0000000	0.0000000	0.0000000	0.0000000	0.0000000
ENSG00000286062	0.0007548	0.0016417	0.0000000	0.0000000	0.0000000
MAST4	0.1517950	0.1420618	0.1724968	0.1468569	0.3939894
MAST4-IT1	0.0000000	0.0000000	0.0000000	0.0000000	0.0000000
MAST4-AS1	0.0104489	0.0157498	0.0026908	0.0137462	0.0000000
LINC02997	0.0011670	0.0025453	0.0040176	0.0000000	0.0101142
LINC02242	0.0005469	0.0000000	0.0000000	0.0019580	0.0000000
ENSG00000250421	0.0000000	0.0085380	0.0076376	0.0068532	0.0075831
PIK3R1	1.3538436	1.1358311	1.0105351	1.1992528	1.0519683
ENSG00000248359	0.0000000	0.0000000	0.0000000	0.0000000	0.0016917
ENSG00000250237	0.0027881	0.0069602	0.0000000	0.0043341	0.0150266
ENSG00000249335	0.0051269	0.0214254	0.0031137	0.0039870	0.0593937
SLC30A5	0.2324278	0.2389262	0.2434137	0.2333721	0.2964207
ENSG00000248664	0.0375745	0.0443047	0.0314106	0.0233252	0.0163902
CCNB1	0.0915403	0.0706888	0.0969662	0.0516148	0.0314013
CENPH	0.1705424	0.1167863	0.1674088	0.1372460	0.1318855
MRPS36	0.6080998	0.6332070	0.7226722	0.5660784	0.5260063
CDK7	0.2207656	0.1633042	0.1975537	0.2155003	0.2044369
CCDC125	0.3246036	0.1922164	0.1506197	0.2936358	0.2792305
AK6	0.4978890	0.4174957	0.3729110	0.4979775	0.2851524
TAF9	0.4183592	0.5302509	0.6056774	0.4210500	0.2992085
RAD17	0.3143676	0.3247725	0.3127257	0.3553124	0.2352200
MARVELD2	0.0715989	0.0391179	0.0110966	0.0866843	0.0101967
ENSG00000249295	0.0002823	0.0009841	0.0000000	0.0026290	0.0000000
OCLN	0.0713746	0.0500645	0.0352152	0.1221964	0.0669767
GTF2H2C	0.0889483	0.0867037	0.0536609	0.0678700	0.0744831
ENSG00000290560	0.0486289	0.0526622	0.0353211	0.0269420	0.1271953

SERF1B	0.0220829	0.0219132	0.0131717	0.0172560	0.0182355
SMN2	0.1250392	0.0860837	0.0673614	0.1178264	0.0502221
ENSG00000291220	0.0170811	0.0281776	0.0280283	0.0311409	0.0767361
SERF1A	0.0116760	0.0108841	0.0117301	0.0155004	0.0082465
SMN1	0.3100392	0.2148456	0.1737759	0.2822850	0.1562063
NAIP	0.0765705	0.0805808	0.0857861	0.0463810	0.1495026
ENSG00000289810	0.0049831	0.0046658	0.0062014	0.0018855	0.0028551
GTF2H2	0.1107974	0.0792629	0.1062454	0.1395711	0.1509111
LINC02197	0.0077044	0.0024143	0.0018503	0.0000000	0.0083937
BDP1	0.7202078	0.8164133	0.8279181	0.6280167	1.0966138
MCCC2	0.1687559	0.1026721	0.0996120	0.1759355	0.1496093
CARTPT	0.0034927	0.0074164	0.0168058	0.0000000	0.0049840
ENSG00000285804	0.0188102	0.0142196	0.0042893	0.0083625	0.0283212
MAP1B	3.0720618	3.4881827	3.7345643	2.9232197	3.2728574
MRPS27	0.2415969	0.2425451	0.2162516	0.2294517	0.1848822
PTCD2	0.0801734	0.1129895	0.1236541	0.1109630	0.0829080
LINC02056	0.0104543	0.0033211	0.0014647	0.0045541	0.0044904
ENSG00000251613	0.0111115	0.0131630	0.0000000	0.0061683	0.0031305
TNPO1-DT	0.0081421	0.0063255	0.0084801	0.0029465	0.0172326
TNPO1	0.6182845	0.5558106	0.6360200	0.5891813	0.7878349
FCHO2-DT	0.0029702	0.0070668	0.0048534	0.0211207	0.0142300
FCHO2	0.2124481	0.1910851	0.1512512	0.1489201	0.3404440
TMEM171	0.0000000	0.0000000	0.0013709	0.0000000	0.0000000
ENSG00000249743	0.0000000	0.0000000	0.0000000	0.0000000	0.0000000
FOXD1	0.0164598	0.0049681	0.0054420	0.0153651	0.0037728
BTF3-DT	0.0311504	0.0331308	0.0595448	0.0233925	0.0425325
BTF3	1.8134189	1.8018761	1.7416127	1.7279897	1.5432906
ANKRA2	0.2210744	0.2203262	0.2187843	0.2060800	0.1620381
UTP15	0.0943769	0.0740211	0.0633603	0.1023307	0.0654723
ARHGEF28	0.2303303	0.1325686	0.1133602	0.1936464	0.1842282
LINC01331	0.0035280	0.0008358	0.0016430	0.0042829	0.0000000
ENC1	0.8412358	0.9141430	1.4126841	0.9005219	0.8828913
HEXB	0.3567142	0.2394878	0.1818850	0.5541221	0.1866643
GFM2	0.2391314	0.2289146	0.2221631	0.2480929	0.2432707
NSA2	1.0502391	1.1602126	1.1607450	0.9568215	0.8817708
FAM169A	0.3804291	0.4613273	0.5472497	0.3509635	0.5332966
FAM169A-AS1	0.0220890	0.0213207	0.0105002	0.0440542	0.0065348
ENSG00000289639	0.0043673	0.0066057	0.0063373	0.0046745	0.0128505
GCNT4	0.0042303	0.0094768	0.0098321	0.0070972	0.0141924
ANKRD31	0.0081392	0.0142883	0.0164805	0.0099884	0.0473544
ENSG00000247372	0.0009568	0.0044341	0.0000000	0.0000000	0.0356902
HMGCR	0.4397873	0.4572420	0.6657492	0.6170147	0.5653852
CERT1	0.4795905	0.4480132	0.4304522	0.4153194	0.6158929
POLK	0.3002239	0.3323448	0.3226025	0.2290343	0.4319858
ANKDD1B	0.0044265	0.0014539	0.0029439	0.0000000	0.0275225
POC5	0.1736123	0.1978692	0.2004718	0.0973051	0.1477908
SV2C-AS1	0.0000000	0.0027512	0.0000000	0.0043533	0.0000000
SV2C	0.3746598	0.7343550	0.4721620	0.2620810	0.7885649
ENSG00000250348	0.0000000	0.0023985	0.0021831	0.0000000	0.0100072
IQGAP2	0.4948923	0.2835532	0.1451962	0.6119093	0.4230859
F2RL2	0.0032557	0.0012039	0.0000000	0.0024238	0.0043054
ENSG00000225407	0.0000000	0.0000000	0.0000000	0.0000000	0.0030362
F2R	0.1458535	0.1471616	0.1102236	0.3195918	0.1659601

F2RL1	0.0114148	0.0038807	0.0042351	0.0261022	0.0043293
S100Z	0.0016244	0.0000000	0.0000000	0.0000000	0.0000000
CRHBP	0.0169960	0.0502812	0.1475125	0.0126997	0.0273886
AGGF1	0.5923748	0.4972224	0.5034570	0.6184361	0.3570041
ZBED3	0.2219719	0.1499635	0.1034739	0.2468136	0.1450084
ZBED3-AS1	0.0679908	0.0569631	0.0349426	0.0799425	0.1183618
PDE8B	0.3086204	0.1836622	0.1022846	0.3713199	0.2910450
WDR41	0.6351894	0.5894111	0.4647201	0.6665064	0.5084024
OTP	0.0094549	0.0097049	0.0116889	0.0145456	0.0221890
TBCA	1.8993071	1.9315737	2.0123759	1.8705106	1.6740549
AP3B1	0.4283454	0.3514223	0.2716233	0.4412861	0.4106574
SCAMP1-AS1	0.1679280	0.1216752	0.0924484	0.1515926	0.0986973
SCAMP1	0.6545766	0.7409123	0.8929914	0.7735614	0.7364635
LHFPL2	0.0867248	0.0909888	0.0721167	0.1121585	0.1050796
ARSB	0.0648750	0.0787655	0.0375119	0.0975890	0.0883485
DMGDH	0.0822293	0.0812222	0.0317694	0.0344318	0.0730530
BHMT2	0.0012707	0.0000000	0.0000000	0.0000000	0.0000000
JMY	0.2757746	0.2196071	0.2180560	0.2070278	0.2950896
HOMER1	0.1670584	0.2393888	0.2333662	0.1188957	0.2956029
TENT2	0.2149805	0.1966887	0.1620180	0.2153075	0.4334304
CMYA5	0.0312756	0.0287104	0.0256400	0.0139663	0.0400790
ENSG00000250258	0.0011007	0.0000000	0.0000000	0.0000000	0.0000000
MTX3	0.2120592	0.1772300	0.1810546	0.1745776	0.1415254
THBS4	0.1066844	0.0878794	0.0508496	0.1001093	0.0699814
THBS4-AS1	0.0011161	0.0012507	0.0023139	0.0000000	0.0000000
SERINC5	0.1761938	0.1372947	0.1027919	0.2552316	0.1881304
ENSG00000251675	0.0023827	0.0010945	0.0070773	0.0115138	0.0000000
ENSG00000288741	0.0010903	0.0016581	0.0066965	0.0024903	0.0000000
ENSG00000289317	0.0007261	0.0011729	0.0000000	0.0000000	0.0000000
ZFYVE16	0.4379209	0.3053935	0.3173876	0.4126470	0.6028531
FAM151B-DT	0.1923491	0.2218093	0.2450845	0.1734433	0.2232967
FAM151B	0.0735932	0.0789065	0.0746674	0.1111465	0.0887531
ANKRD34B	0.0110919	0.0120286	0.0221182	0.0081443	0.0032840
LINC01337	0.0009146	0.0013612	0.0020099	0.0000000	0.0000000
DHFR	0.2748134	0.2220719	0.1910283	0.3161699	0.1668812
MSH3	0.3160484	0.3146416	0.2998461	0.2544894	0.3739399
RASGRF2-AS1	0.0085217	0.0026400	0.0047458	0.0028665	0.0153417
RASGRF2	0.0725946	0.0487927	0.0504706	0.0880701	0.1075518
ENSG00000249772	0.0010385	0.0013778	0.0000000	0.0015807	0.0143133
CKMT2-AS1	0.1555890	0.1785477	0.1924265	0.1131899	0.2012797
CKMT2	0.0082760	0.0078693	0.0093606	0.0022536	0.0069893
ZCCHC9	0.3120696	0.2402120	0.1988989	0.2673852	0.2079456
ACOT12	0.0091921	0.0117785	0.0092182	0.0021179	0.0385883
ENSG00000286721	0.0044057	0.0070932	0.0072868	0.0000000	0.0244658
SSBP2	0.8399261	0.9378306	1.0219311	0.8276352	1.3945493
ENSG00000249483	0.0011932	0.0036789	0.0000000	0.0028566	0.0000000
ATG10	0.1264854	0.1331603	0.1664262	0.1031411	0.2548205
RPS23	2.9781034	2.9877028	2.7461605	2.8653630	2.7140244
LINC01338	0.1581738	0.1094647	0.0534815	0.1866766	0.0638349
ENSG00000248112	0.0041733	0.0012718	0.0012574	0.0000000	0.0000000
ENSG00000271862	0.0005543	0.0011265	0.0007943	0.0038283	0.0000000
TMEM167A	0.8591325	0.9250033	1.0146462	0.8908097	0.6764767
XRCC4	0.1759419	0.1351095	0.1541497	0.1702239	0.2749666

VCAN	0.8433603	0.7434843	0.7269064	1.2377820	0.7927530
VCAN-AS1	0.0023662	0.0029022	0.0008860	0.0075427	0.0090448
HAPLN1	0.0011293	0.0051065	0.0018698	0.0064965	0.0000000
EDIL3	0.5141336	0.8264901	1.1363837	0.4469965	1.3379181
EDIL3-DT	0.0301532	0.0613682	0.1087486	0.0394263	0.1321050
COX7C	2.2209212	2.3136864	2.3252139	2.1878007	1.9555974
LINC01949	0.0000000	0.0000000	0.0000000	0.0000000	0.0044999
RASA1	0.3964351	0.3592557	0.3868804	0.3200580	0.6227251
CCNH	0.2540573	0.2633772	0.2337967	0.3188462	0.4493726
ENSG00000285190	0.0108941	0.0085440	0.0121114	0.0053759	0.0114925
ENSG00000287862	0.0052784	0.0041967	0.0013222	0.0132220	0.0120917
ENSG00000289462	0.0054262	0.0053553	0.0031894	0.0018314	0.0052497
TMEM161B	0.0923951	0.1404781	0.1092453	0.1158899	0.2014692
TMEM161B-DT	0.3374862	0.3552400	0.3231594	0.3426643	0.5343403
LINC02060	0.0008864	0.0000000	0.0031845	0.0000000	0.0037236
ENSG00000271904	0.0065372	0.0000000	0.0014078	0.0071420	0.0000000
LINC00461	0.1805150	0.0853861	0.0377793	0.2237265	0.0866488
MEF2C-AS2	0.0088026	0.0136668	0.0064447	0.0440356	0.0083079
MEF2C	0.4063220	0.2278439	0.1793477	0.3016801	0.2875084
MEF2C-AS1	0.0708886	0.0753437	0.0316786	0.0822045	0.1058613
LINC02161	0.0000000	0.0000000	0.0000000	0.0000000	0.0000000
ENSG00000214942	0.0000000	0.0000000	0.0000000	0.0000000	0.0062581
LINC01339	0.0015779	0.0006335	0.0000000	0.0000000	0.0060624
ENSG00000260871	0.0013964	0.0061147	0.0000000	0.0000000	0.0068978
CETN3	0.5120995	0.3606310	0.3217960	0.4831763	0.2985770
ENSG00000255647	0.0038319	0.0020399	0.0030802	0.0045265	0.0110244
MBLAC2	0.1608136	0.2025385	0.2733837	0.1168818	0.1856396
POLR3G	0.0686840	0.0366455	0.0157100	0.0492407	0.0313661
LYSMD3	0.2299269	0.1811266	0.1628775	0.2944080	0.1187329
ADGRV1	0.5387067	0.4907521	0.3235586	0.8612572	0.6410633
LUCAT1	0.0132577	0.0156602	0.0025896	0.0040695	0.0475289
ENSG00000286638	0.0005446	0.0011761	0.0013411	0.0015022	0.0000000
ARRDC3	0.6423637	0.6441540	0.5431024	0.6054159	0.5764479
ARRDC3-AS1	0.0198029	0.0222584	0.0198932	0.0241149	0.0432778
ENSG00000286121	0.0088665	0.0301611	0.0023834	0.0203098	0.0531652
ENSG00000250049	0.0910223	0.1259515	0.1040582	0.0703563	0.6160382
ENSG00000249776	0.0096772	0.0112030	0.0053534	0.0131885	0.1027781
ENSG00000249169	0.0000000	0.0000000	0.0000000	0.0000000	0.0000000
ENSG00000248588	0.0000000	0.0000000	0.0000000	0.0000000	0.0000000
NR2F1-AS1	0.2457031	0.3194045	0.2110546	0.1695467	0.4343429
NR2F1	1.1561647	1.1976975	0.7192672	1.3270493	0.8217930
ENSG00000289274	0.0038619	0.0005872	0.0016430	0.0029008	0.0089701
FAM172A	0.6671246	0.6332143	0.6182143	0.6470763	0.8934080
ENSG00000287180	0.0055461	0.0067750	0.0108656	0.0040556	0.0051966
POU5F2	0.0030757	0.0000000	0.0018472	0.0035916	0.0144105
ENSG00000287447	0.0000000	0.0000000	0.0000000	0.0000000	0.0000000
ENSG00000251023	0.0002281	0.0009852	0.0000000	0.0000000	0.0082388
ENSG00000286577	0.0025283	0.0024186	0.0039228	0.0039507	0.0000000
KIAA0825	0.1890466	0.1679161	0.1238650	0.1883923	0.3806024
SLF1	0.2085013	0.1434036	0.1230638	0.1300042	0.2144923
MCTP1	0.0742600	0.1318235	0.1084327	0.1394139	0.4566499
FAM81B	0.5488462	0.3646683	0.2384532	0.4665222	0.3897346
SKIC3	0.7398507	0.6077257	0.5807802	0.7715202	0.8003489

ARSK	0.0976872	0.0710188	0.0676744	0.1526974	0.0653954
GPR150	0.0064321	0.0060733	0.0084989	0.0000000	0.0000000
RFESD	0.0582247	0.0446978	0.0426781	0.0830688	0.0289573
SPATA9	0.0051670	0.0057969	0.0059458	0.0067790	0.0023501
ENSG00000250240	0.0019457	0.0038240	0.0059137	0.0000000	0.0000000
RHOBTB3	1.2718767	0.9236089	0.6837738	1.3060254	0.8086423
GLRX	0.1980479	0.2254086	0.1958627	0.2446640	0.1941007
LINC01554	0.0020879	0.0016378	0.0000000	0.0000000	0.0030416
ELL2	0.2075776	0.1145787	0.0987323	0.2702453	0.1339371
ENSG00000251314	0.0153007	0.0102100	0.0071273	0.0000000	0.0456680
PCSK1	0.0639193	0.0772369	0.4640011	0.0092678	0.1165217
CAST	1.3703222	0.9457785	0.8002185	1.2677940	0.8573037
ERAP1	0.1631424	0.1125023	0.0925887	0.1798445	0.1286826
ENSG00000248734	0.0010898	0.0000000	0.0000000	0.0079182	0.0000000
ENSG00000272109	0.0000000	0.0000000	0.0000000	0.0000000	0.0056829
ENSG00000247121	0.0561075	0.0590277	0.0489564	0.0298591	0.1469593
ERAP2	0.0062064	0.0040551	0.0000000	0.0159860	0.0095340
LNPEP	0.2748049	0.2771666	0.2077948	0.3396678	0.3839666
LIX1-AS1	0.0275496	0.0198355	0.0216881	0.0160678	0.1154654
LIX1	0.1960501	0.1432886	0.0719657	0.2845003	0.1474600
RIOK2	0.3041151	0.2004146	0.1986422	0.2838866	0.1352534
ENSG00000286828	0.0004577	0.0035927	0.0058416	0.0000000	0.0155984
ENSG00000248758	0.0013773	0.0000000	0.0000000	0.0000000	0.0066411
LINC01340	0.0027723	0.0049040	0.0046396	0.0033205	0.0096217
ENSG00000286953	0.0009146	0.0015958	0.0000000	0.0000000	0.0171943
LINC02234	0.0016798	0.0051065	0.0000000	0.0037834	0.0000000
LINC01846	0.0000000	0.0010146	0.0000000	0.0000000	0.0000000
RGMB	0.1916967	0.2019538	0.2413490	0.2430266	0.1953781
RGMB-AS1	0.0134791	0.0125686	0.0023142	0.0117473	0.0055238
ENSG00000279232	0.0105753	0.0153729	0.0025488	0.0035651	0.0000000
CHD1	0.4182003	0.3508020	0.2996897	0.4285588	0.5653214
CHD1-DT	0.0997826	0.0717603	0.0470381	0.1193744	0.1802885
LINC02113	0.0000000	0.0000000	0.0000000	0.0000000	0.0000000
ENSG00000249787	0.0031346	0.0025161	0.0051724	0.0000000	0.0106694
FAM174A-DT	0.0057263	0.0068954	0.0026308	0.0000000	0.0263869
FAM174A	0.2356056	0.1874374	0.1782598	0.2101733	0.1784587
ST8SIA4	0.1629752	0.2321145	0.3073650	0.1437229	0.4189707
ENSG00000289912	0.0050175	0.0028457	0.0052658	0.0000000	0.0030832
SLCO4C1	0.0039726	0.0000000	0.0000000	0.0016517	0.0103095
SLCO6A1	0.0027694	0.0052774	0.0008344	0.0000000	0.0000000
LINC00491	0.0082630	0.0061301	0.0128600	0.0000000	0.0129463
ENSG00000286338	0.0012248	0.0048508	0.0045850	0.0035433	0.0000000
PAM	0.8500221	0.6385829	0.7148202	1.1479143	1.2417047
GIN1	0.1372500	0.1373331	0.1559898	0.1439792	0.0859758
PPIP5K2	0.5528107	0.7791077	0.9593645	0.5183346	0.7822999
MACIR	0.1199789	0.2424646	0.2631113	0.1600011	0.1302517
LINC02115	0.0016835	0.0049950	0.0037061	0.0061006	0.0031524
NUDT12	0.1926792	0.1397168	0.0808075	0.1897117	0.1076780
NIHCOLE	0.0015440	0.0021513	0.0030939	0.0000000	0.0033534
ENSG00000251574	0.0000000	0.0000000	0.0000000	0.0000000	0.0140990
LINC01950	0.0023555	0.0000000	0.0027702	0.0000000	0.0175614
EFNA5	0.1598769	0.2064965	0.2543835	0.1618150	0.7522086
FBXL17	0.4446191	0.4777052	0.4559193	0.3927947	1.1365030

ENSG00000286503	0.0030005	0.0098627	0.0000000	0.0083569	0.0253853
LINC01023	0.0222694	0.0330696	0.0096268	0.0491783	0.0250846
ENSG00000289260	0.0104810	0.0043144	0.0015069	0.0014766	0.0000000
FER	0.4804532	0.5316416	0.5546784	0.4621969	1.0290590
ENSG00000250383	0.0000000	0.0000000	0.0000000	0.0050900	0.0000000
ENSG00000286882	0.0009146	0.0000000	0.0000000	0.0000000	0.0000000
ENSG00000249476	0.0098617	0.0070918	0.0090772	0.0057313	0.0556357
PJA2	1.6326068	1.7964114	1.9509094	1.5357824	1.5086333
MAN2A1-DT	0.0005412	0.0040029	0.0000000	0.0000000	0.0000000
MAN2A1	0.1497847	0.1666856	0.0935702	0.1506119	0.3040835
TMEM232	0.5236281	0.3860562	0.2523747	0.4641504	0.6181405
SLC25A46	0.3756773	0.4338750	0.4615201	0.3616169	0.4362096
TSLP	0.0044465	0.0000000	0.0013641	0.0027814	0.0000000
ENSG00000253613	0.0000000	0.0013131	0.0000000	0.0000000	0.0047049
WDR36	0.2168590	0.2201952	0.2130700	0.1720772	0.2542066
CAMK4	0.3521252	0.6365537	0.8084562	0.3287216	0.7647177
ENSG00000288965	0.0000000	0.0028980	0.0031505	0.0000000	0.0102665
ENSG00000248268	0.0000000	0.0000000	0.0000000	0.0000000	0.0000000
STARD4	0.1271150	0.1337308	0.0974984	0.1731580	0.1370314
STARD4-AS1	0.2804994	0.3797291	0.4127247	0.2667188	0.3859805
NREP	2.0672463	2.3561898	2.5883271	1.8855952	2.0837950
NREP-AS1	0.0000000	0.0026632	0.0000000	0.0000000	0.0000000
EPB41L4A	0.2674527	0.2755910	0.3027317	0.3109301	0.4385185
EPB41L4A-AS1	0.5473893	0.6083365	0.5555395	0.3784007	0.4603894
ENSG00000251187	0.0006971	0.0012812	0.0000000	0.0000000	0.0052857
ENSG00000251076	0.0000000	0.0000000	0.0000000	0.0000000	0.0033235
EPB41L4A-DT	0.0153359	0.0076013	0.0130173	0.0090501	0.0023501
LINC02200	0.0089147	0.0013468	0.0012851	0.0000000	0.0000000
APC	0.8536859	1.3039240	1.3631626	0.8509625	1.2539515
SRP19	0.6882586	0.7206944	0.8157854	0.7224835	0.6863415
REEP5	0.6932022	0.7748199	0.9556355	0.7838432	0.6305329
DCP2	0.4042065	0.3808347	0.3356952	0.4459652	0.3599769
MCC	0.1508993	0.1242627	0.0902619	0.0804224	0.2718444
ENSG00000232633	0.0007450	0.0057921	0.0052550	0.0029843	0.0000000
YTHDC2	0.2150932	0.2187232	0.2175697	0.1741293	0.4094207
KCNN2	0.0389464	0.0240164	0.0282976	0.0251780	0.1083806
ENSG00000246316	0.0000000	0.0000000	0.0006954	0.0000000	0.0000000
TRIM36	0.4372635	0.6331433	0.6727912	0.4016893	0.5189942
TRIM36-IT1	0.0000000	0.0014762	0.0000000	0.0000000	0.0067724
PGGT1B	0.2360724	0.1841758	0.1266251	0.2555001	0.2329592
CCDC112	0.7550739	1.0424605	1.2163262	0.6419415	0.7906901
ENSG00000289497	0.0000000	0.0000000	0.0000000	0.0115360	0.0000000
FEM1C	0.2928684	0.2521920	0.1876170	0.2891694	0.2604917
TICAM2	0.0516951	0.0331945	0.0486876	0.0664199	0.0340715
TICAM2-AS1	0.0378161	0.0294694	0.0301707	0.0735259	0.0207781
TMED7	0.5658322	0.4367916	0.4298335	0.7215383	0.4271445
ENSG00000249021	0.0000000	0.0011882	0.0000000	0.0000000	0.0033746
CDO1	1.3377391	0.9200772	0.7692968	1.4171111	0.8462324
ATG12	0.6555719	0.7576208	0.8210272	0.5589702	0.6000227
AP3S1	0.8382742	0.9329791	1.0199199	0.7742013	0.8191814
LINCADL	0.0000000	0.0000000	0.0000000	0.0000000	0.0000000
LVRN	0.0042114	0.0086402	0.0040993	0.0039397	0.0157352
ARL14EPL	0.0026105	0.0000000	0.0023834	0.0000000	0.0000000

ENSG00000271918	0.0182305	0.0172466	0.0058807	0.0281401	0.0050173
COMMD10	0.2976945	0.2284582	0.2260206	0.2514258	0.3359569
ENSG00000250015	0.0016941	0.0011185	0.0078845	0.0019116	0.0000000
SEMA6A	0.5687459	0.4684668	0.4581927	0.6753834	0.7420317
SEMA6A-AS1	0.0063144	0.0079717	0.0074978	0.0075522	0.0924763
SEMA6A-AS2	0.0094836	0.0046615	0.0234161	0.0039672	0.0048432
LINC00992	0.0021689	0.0027711	0.0047226	0.0028665	0.0025600
LINC02147	0.0033985	0.0008632	0.0034943	0.0000000	0.0452403
LINC02208	0.0053428	0.0059732	0.0021524	0.0050294	0.0043847
ENSG00000286745	0.0000000	0.0000000	0.0000000	0.0000000	0.0000000
ENSG00000249426	0.0000000	0.0005546	0.0000000	0.0000000	0.0000000
DTWD2	0.1100824	0.1070844	0.0743469	0.0868598	0.1196803
DMXL1-DT	0.0149510	0.0140858	0.0055917	0.0094201	0.0106652
DMXL1	0.3935580	0.3997198	0.3636294	0.3729097	0.5812187
TNFAIP8	0.0250772	0.0286771	0.0308681	0.0468771	0.0171243
HSD17B4	0.5863071	0.4154844	0.4314204	0.6724852	0.4255679
PRR16	0.0219801	0.0248355	0.0366174	0.0406299	0.1516144
ENSG00000248927	0.0059583	0.0079918	0.0076799	0.0064615	0.0178832
SRFBP1	0.4055126	0.4570037	0.3857392	0.4327702	0.4265438
LOX	0.0792014	0.0446108	0.0372037	0.0900375	0.0432843
ENSG00000247311	0.0202306	0.0154133	0.0086761	0.0266865	0.0250036
ZNF474	0.1199228	0.0676931	0.0359917	0.1140903	0.0571607
ENSG00000250803	0.0023269	0.0000000	0.0000000	0.0000000	0.0000000
ENSG00000272139	0.0175727	0.0078341	0.0087178	0.0242155	0.0000000
SNCAIP	0.7028310	0.4584136	0.3023866	0.7868912	0.4745195
ENSG00000249621	0.0019368	0.0021257	0.0000000	0.0000000	0.0000000
ENSG00000289054	0.0010990	0.0014914	0.0000000	0.0000000	0.0000000
ENSG00000249916	0.0000000	0.0000000	0.0000000	0.0000000	0.0083632
MGC32805	0.0022722	0.0062776	0.0009721	0.0060372	0.0238775
LINC02201	0.0005349	0.0017155	0.0020357	0.0000000	0.0071630
SNX2	0.6357910	0.5215618	0.4573193	0.6241477	0.4720712
SNX24	0.2482130	0.2820112	0.2016959	0.2890495	0.2533336
PPIC	0.0408579	0.0308560	0.0167838	0.0699179	0.0525363
PPIC-AS1	0.0014257	0.0046988	0.0000000	0.0023684	0.0148481
PRDM6	0.0025332	0.0028727	0.0119597	0.0039108	0.0099874
CEP120	0.1509683	0.2105730	0.2237442	0.1432959	0.2129372
ENSG00000288766	0.0225948	0.0152806	0.0144730	0.0227737	0.0067404
CSNK1G3	0.4399378	0.5104790	0.5124371	0.4466326	0.5036695
ENSG00000288890	0.0017912	0.0074095	0.0050886	0.0000000	0.0125024
LINC01170	0.0086666	0.0085693	0.0047535	0.0012486	0.0341699
ENSG00000248296	0.0003453	0.0000000	0.0020352	0.0000000	0.0014498
ZNF608	0.6677830	0.7387492	0.7777185	0.5559511	0.9499221
ENSG00000249112	0.0000000	0.0022488	0.0000000	0.0024107	0.0105734
ENSG00000251456	0.0008526	0.0017487	0.0000000	0.0000000	0.0000000
LINC02240	0.0044394	0.0110445	0.0026996	0.0015092	0.0249950
ENSG00000248752	0.0043612	0.0077872	0.0037823	0.0112827	0.0311214
GRAMD2B	0.4218654	0.1966552	0.1022621	0.4961135	0.2397614
ENSG00000250602	0.0082019	0.0068158	0.0000000	0.0022509	0.0000000
ALDH7A1	0.5894343	0.4616315	0.5438594	0.6468866	0.4030985
PHAX	0.6505089	0.6920182	0.7125575	0.6658065	0.5259108
TEX43	0.0000000	0.0000000	0.0022332	0.0000000	0.0000000
LMNB1-DT	0.0082360	0.0104046	0.0063247	0.0043931	0.0324044
LMNB1	0.1401922	0.1327516	0.2325102	0.1349772	0.2017921

MARCHF3	0.0939349	0.1402370	0.1728586	0.1135138	0.3192241
C5orf63	0.0406660	0.0397683	0.0500495	0.0110536	0.0358208
ENSG00000283897	0.0022264	0.0040580	0.0092615	0.0030978	0.0120304
ENSG00000286615	0.0152818	0.0044298	0.0047970	0.0301135	0.0038629
MEGF10	0.0956079	0.0452445	0.0365820	0.1952491	0.1054337
PRRC1	0.2842222	0.2382372	0.2480376	0.2470450	0.2746536
CTXN3	0.0405950	0.0467972	0.1156446	0.0215699	0.0691702
CCDC192	0.0009054	0.0014253	0.0079089	0.0062584	0.0185920
ENSG00000250603	0.0000000	0.0000000	0.0000000	0.0000000	0.0000000
SLC12A2-DT	0.3297658	0.2619683	0.2285109	0.2998844	0.3260270
SLC12A2	0.4609277	0.3356846	0.2313459	0.4912436	0.4765680
FBN2	0.0501026	0.0476110	0.0353621	0.1246004	0.1326644
SLC27A6	0.0063995	0.0074409	0.0169187	0.0159866	0.0446674
ENSG00000248634	0.0013505	0.0000000	0.0000000	0.0000000	0.0000000
ISOC1	0.2348002	0.3086652	0.4457501	0.2333659	0.2087218
ADAMTS19-AS1	0.0015494	0.0075293	0.0095300	0.0046657	0.0100405
ADAMTS19	0.0132859	0.0084595	0.0136727	0.0268622	0.0251388
ENSG00000251680	0.0558851	0.0571634	0.0122828	0.0331469	0.2856674
MINAR2	0.0177666	0.0290769	0.0712091	0.0098513	0.0267211
CHSY3	0.0457151	0.1130638	0.0605667	0.0559454	0.4062507
ENSG00000287390	0.0000000	0.0013552	0.0000000	0.0000000	0.0000000
HINT1	2.1538706	2.1242021	2.1541043	2.0521881	1.8089275
LYRM7	0.2502879	0.2332012	0.2483207	0.2195982	0.1374517
ENSG00000290020	0.0016640	0.0018762	0.0096133	0.0000000	0.0175676
CDC42SE2	0.3700442	0.3891065	0.4386234	0.3770084	0.5341774
RAPGEF6	0.4110219	0.4941348	0.3573150	0.3493192	1.0147120
FNIP1	0.3054230	0.2539227	0.2717012	0.2585610	0.5176833
MEIKIN	0.0086860	0.0071644	0.0010569	0.0042766	0.0077958
ENSG00000234758	0.0000000	0.0000000	0.0000000	0.0000000	0.0063815
ACSL6	0.0497056	0.0906448	0.1053671	0.0523565	0.2031759
ACSL6-AS1	0.0007419	0.0011443	0.0000000	0.0000000	0.0000000
ENSG00000231585	0.0000000	0.0049738	0.0044051	0.0000000	0.0000000
P4HA2-AS1	0.0037881	0.0005445	0.0029221	0.0000000	0.0000000
P4HA2	0.4874884	0.3514874	0.3284836	0.6566423	0.3275595
PDLIM4	0.0705596	0.0514310	0.0431627	0.0965290	0.0153750
SLC22A4	0.0351726	0.0560633	0.0551356	0.0272876	0.0871756
MIR3936HG	0.1616281	0.2271076	0.2471975	0.1490200	0.4374738
SLC22A5	0.0596493	0.0593223	0.0642326	0.0758689	0.0747202
IRF1-AS1	0.0963372	0.0702550	0.0456296	0.1111326	0.0444812
IRF1	0.0933641	0.0490089	0.0297759	0.1026034	0.0395312
IL5	0.0004685	0.0000000	0.0000000	0.0000000	0.0036428
RAD50	0.5869584	0.6377493	0.5860145	0.5499551	0.5384751
TH2LCRR	0.0012439	0.0029228	0.0047751	0.0000000	0.0000000
IL13	0.0000000	0.0000000	0.0014736	0.0000000	0.0000000
IL4	0.0000000	0.0000000	0.0000000	0.0000000	0.0013481
ENSG00000230612	0.0015168	0.0017379	0.0018837	0.0018314	0.0076546
KIF3A	0.8197611	1.1653037	1.4038435	0.7650288	1.0789872
CCNI2	0.0000000	0.0000000	0.0009712	0.0014762	0.0097348
SEPTIN8	0.1520140	0.1490326	0.2155143	0.1667925	0.1641576
SOWAHA	0.0088572	0.0053916	0.0360873	0.0058641	0.0270343
SHROOM1	0.0100588	0.0213741	0.0234271	0.0056517	0.0383822
GDF9	0.0103077	0.0172349	0.0090752	0.0067606	0.0076978
UQCRQ	1.4571233	1.4674500	1.5173534	1.4505742	1.1777832

LEAP2	0.0086461	0.0041185	0.0000000	0.0074881	0.0096831
AFF4	0.7630056	0.6347666	0.5809757	0.6690541	0.8890040
ZCCHC10	0.2814863	0.2438128	0.2462552	0.2405535	0.2012472
ENSG00000287054	0.0016830	0.0018687	0.0053500	0.0016697	0.0000000
ENSG00000286408	0.0046074	0.0088392	0.0021046	0.0086018	0.0033679
HSPA4	0.9705165	0.9996054	1.0322672	1.0682201	0.9709582
ENSG00000248245	0.0018525	0.0030817	0.0035091	0.0014739	0.0155054
FSTL4	0.0675560	0.0879858	0.1226885	0.0572804	0.5145733
ENSG00000249478	0.0000000	0.0000000	0.0000000	0.0000000	0.0000000
ENSG00000250244	0.0021573	0.0035393	0.0041391	0.0044092	0.0000000
C5orf15	0.3869280	0.2995206	0.2678776	0.5872732	0.2507171
VDAC1	1.6023511	1.7096578	1.7931725	1.6121123	1.4256802
ENSG00000271737	0.0732685	0.0393511	0.0467112	0.0409808	0.0240012
TCF7	0.0439582	0.0113746	0.0064157	0.0494294	0.0622123
SKP1	2.3494219	2.2871879	2.3734953	2.3806313	2.0463504
PPP2CA	0.9112837	1.0045729	1.1128843	0.9412057	0.8866862
PPP2CA-DT	0.0006832	0.0000000	0.0000000	0.0000000	0.0104252
CDKL3	0.2569584	0.2637136	0.2983579	0.2589971	0.3448896
UBE2B	0.7736359	0.8276044	0.8709888	0.7875996	0.7527938
ENSG00000248559	0.0035871	0.0034305	0.0076294	0.0000000	0.0095568
CDKN2AIPNL	0.2704638	0.4251177	0.4398457	0.2375978	0.2872156
LINC02999	0.0007149	0.0000000	0.0000000	0.0000000	0.0000000
ENSG00000250994	0.0021451	0.0015004	0.0014078	0.0000000	0.0116725
JADE2	0.0492161	0.0629260	0.0513692	0.0326147	0.0848171
SAR1B	0.3937524	0.3407945	0.3769681	0.4552629	0.3712163
SEC24A	0.1924923	0.1507368	0.1488206	0.1984408	0.1666310
CAMLG	1.0305831	1.0503662	1.0602297	1.0155417	0.8945129
DDX46	0.6023769	0.5523960	0.5629580	0.5808791	0.5643971
C5orf24	0.5944161	0.6218550	0.7783140	0.5278841	0.4705709
TXNDC15	0.3864015	0.3586049	0.3254239	0.6069551	0.3693374
PCBD2	0.1859391	0.1909326	0.1425675	0.1893409	0.2017988
CATSPER3	0.0043704	0.0018667	0.0000000	0.0000000	0.0000000
PITX1-AS1	0.0008847	0.0085771	0.0026408	0.0000000	0.0434010
MACROH2A1	0.8986814	0.9174796	0.9981913	0.9344755	0.7101024
ENSG00000270021	0.0218993	0.0203668	0.0239855	0.0288245	0.0141275
SLC25A48	0.0011569	0.0000000	0.0000000	0.0000000	0.0025298
CXCL14	0.0161485	0.0221693	0.0137270	0.0232460	0.0030416
TGFBI	0.0426592	0.0290572	0.0229123	0.0436060	0.0506607
SMAD5-AS1	0.0036205	0.0026165	0.0023244	0.0000000	0.0000000
SMAD5	0.4785619	0.3940549	0.2991582	0.4542759	0.3723863
SMIM32	0.0057697	0.0058959	0.0177911	0.0000000	0.0113071
TRPC7	0.0039637	0.0029783	0.0000000	0.0043212	0.0229514
TRPC7-AS1	0.0008869	0.0028765	0.0023562	0.0000000	0.0000000
SPOCK1	0.1657500	0.2081090	0.3919743	0.2307892	0.5994155
KLHL3	0.0306187	0.0755233	0.0392598	0.0327455	0.1260992
HNRNPA0	0.9076636	0.9056332	0.9302368	0.8696256	0.7208447
PKD2L2-DT	0.0098619	0.0219692	0.0154314	0.0079671	0.0365586
MYOT	0.0000000	0.0000000	0.0000000	0.0000000	0.0000000
PKD2L2	0.0077263	0.0024956	0.0036678	0.0044825	0.0528284
FAM13B	0.2830821	0.2519684	0.2521042	0.2601870	0.5160312
FAM13B-AS1	0.0023374	0.0076095	0.0045258	0.0000000	0.0135263
WNT8A	0.0005499	0.0052590	0.0043659	0.0042115	0.0000000
NME5	0.2994344	0.2284031	0.1978792	0.3683137	0.3020477

BRD8	0.3176539	0.3184280	0.3202088	0.3868908	0.3171653
KIF20A	0.0037176	0.0022237	0.0000000	0.0000000	0.0100072
CDC23	0.2515688	0.1919320	0.1611262	0.2461153	0.2130701
GFRA3	0.0110156	0.0213483	0.0326308	0.0111552	0.0293870
CDC25C	0.0046107	0.0061761	0.0031815	0.0015524	0.0000000
FAM53C	0.1397666	0.1305627	0.0931329	0.1312558	0.1669740
KDM3B	0.3332728	0.2914446	0.2494907	0.3711311	0.4152671
REEP2	0.5415103	0.6500592	0.6339801	0.6321925	0.5698329
ENSG00000289267	0.0007832	0.0000000	0.0000000	0.0000000	0.0087387
EGR1	0.6915578	0.4907383	0.3863641	0.5021337	0.6988039
ETF1	0.5792915	0.5290065	0.5068409	0.5540366	0.5140404
ENSG00000289155	0.0019690	0.0022079	0.0055964	0.0027747	0.0000000
HSPA9	1.3881770	1.2015969	1.0684018	1.3167301	0.9487137
CTNNA1	1.3874258	1.1053996	0.9139463	1.4158524	1.0310784
CTNNA1-AS1	0.0076364	0.0018265	0.0000000	0.0030728	0.0000000
LRRTM2	0.1809338	0.3611413	0.5096403	0.1771219	0.3047687
SIL1	0.4041409	0.2984420	0.2101733	0.6215138	0.4541800
ENSG00000249593	0.0045762	0.0033005	0.0000000	0.0000000	0.0013481
SNHG4	0.0063911	0.0134630	0.0201205	0.0040415	0.0253743
MATR3	1.7477617	1.7982634	2.0366448	1.7185039	1.6428640
PAIP2	1.2912789	1.1229561	1.2458619	1.1937147	1.1009852
ENSG00000272742	0.0045969	0.0000000	0.0014741	0.0036457	0.0000000
SLC23A1	0.0019523	0.0000000	0.0000000	0.0000000	0.0000000
MZB1	0.0000000	0.0015322	0.0000000	0.0032535	0.0000000
PROB1	0.0068669	0.0060267	0.0088217	0.0135937	0.0000000
SPATA24	0.0893733	0.0642509	0.0420402	0.0549726	0.0603237
DNAJC18	0.2659168	0.2648063	0.2470687	0.2517913	0.3707529
STING1	0.0008465	0.0057723	0.0050586	0.0137199	0.0067724
UBE2D2	1.0633936	1.1029007	1.0711050	1.0579575	0.9505674
CXXC5	0.9413824	1.0832498	1.2266474	1.0062424	0.8916769
ENSG00000249526	0.0041514	0.0065167	0.0047019	0.0065299	0.0221473
PSD2-AS1	0.0000000	0.0000000	0.0000000	0.0000000	0.0000000
PSD2	0.0652202	0.1495436	0.2077351	0.0697440	0.1986883
NRG2	0.0512475	0.0817368	0.0758906	0.0415538	0.3874492
ENSG00000250692	0.0000000	0.0000000	0.0014804	0.0000000	0.0073853
MALINC1	0.0124243	0.0148477	0.0169767	0.0043765	0.0156712
PURA	0.4993133	0.3821910	0.3056375	0.3756936	0.4051776
IGIP	0.0504972	0.0457249	0.0476234	0.0277686	0.0468059
ENSG00000254363	0.0000000	0.0000000	0.0022147	0.0000000	0.0027158
CYSTM1	0.6069195	0.4811258	0.4339226	0.5732829	0.4865073
ENSG00000250069	0.0000000	0.0000000	0.0000000	0.0000000	0.0000000
PFDN1	0.7282719	0.7784598	0.8006254	0.7830975	0.5503849
HBEGF	0.0760202	0.1418745	0.1511679	0.1000644	0.1052205
ENSG00000253965	0.0024340	0.0021557	0.0011329	0.0071088	0.0019899
SLC4A9	0.0000000	0.0000000	0.0000000	0.0000000	0.0000000
ANKHD1-DT	0.0603102	0.0481557	0.0691945	0.0488593	0.0269335
ANKHD1	0.4833341	0.3463628	0.2834107	0.4790603	0.6300029
SRA1	0.3606476	0.3191261	0.3519534	0.3791580	0.2291428
EIF4EBP3	0.2186241	0.1181615	0.0475628	0.1982163	0.0980112
APBB3	0.0818641	0.1595805	0.1647763	0.0739015	0.0981724
SLC35A4	0.0890192	0.0819489	0.0404353	0.1302999	0.0530982
CD14	0.0024740	0.0040389	0.0051084	0.0000000	0.0063320
NDUFA2	0.7978444	0.8049336	0.7812868	0.7212395	0.6138980

TMCO6	0.0282092	0.0354371	0.0328330	0.0204639	0.0090499
IK	1.3016060	1.1136469	1.0587303	1.2105154	0.9319360
WDR55	0.0790458	0.0878607	0.0842779	0.0482106	0.0736564
DND1	0.0038349	0.0077264	0.0031610	0.0031909	0.0118127
HARS1	0.2358547	0.3390123	0.3412827	0.2904769	0.2591230
HARS2	0.0743575	0.0659383	0.0717920	0.0800532	0.1107475
ZMAT2	0.6501015	0.6852526	0.6896677	0.6477082	0.4964075
ENSG00000279726	0.0256264	0.0488750	0.0372821	0.0324797	0.0288844
PCDHA1	0.0000000	0.0000000	0.0013828	0.0000000	0.0089327
PCDHA2	0.0247745	0.0460171	0.0653661	0.0095483	0.0479378
PCDHA3	0.0150424	0.0201620	0.0380049	0.0180756	0.0363171
PCDHA4	0.0348994	0.0470186	0.0597347	0.0867275	0.0870686
PCDHA5	0.0035936	0.0106727	0.0045218	0.0176984	0.0338844
PCDHA6	0.0265480	0.0222954	0.0286862	0.0330799	0.0379589
PCDHA7	0.0003993	0.0022730	0.0014642	0.0000000	0.0050044
PCDHA8	0.0022423	0.0067695	0.0043513	0.0000000	0.0088923
PCDHA9	0.0008275	0.0030880	0.0012400	0.0000000	0.0195987
PCDHA10	0.1155543	0.1004736	0.1072840	0.2035215	0.1971861
ENSG00000278946	0.0028577	0.0000000	0.0000000	0.0000000	0.0000000
PCDHA11	0.0177424	0.0252376	0.0206732	0.0516308	0.0211827
PCDHA12	0.0268792	0.0289455	0.0363159	0.0690873	0.0339591
ENSG00000278915	0.0000000	0.0009219	0.0000000	0.0030679	0.0038198
ENSG00000278901	0.0000000	0.0000000	0.0000000	0.0000000	0.0056391
PCDHA13	0.0014574	0.0022934	0.0019140	0.0030924	0.0031293
PCDHAC1	0.0102528	0.0146815	0.0066148	0.0067248	0.0100418
PCDHAC2	0.0086194	0.0086336	0.0217349	0.0109048	0.0045404
ENSG00000288892	0.0029449	0.0013082	0.0000000	0.0000000	0.0192902
PCDHB1-AS1	0.0038264	0.0030091	0.0006071	0.0069649	0.0051831
PCDHB1	0.0007727	0.0000000	0.0000000	0.0000000	0.0000000
PCDHB2	0.3021972	0.3568671	0.4219458	0.5194541	0.3633871
ENSG00000272154	0.0106347	0.0242043	0.0387825	0.0197822	0.0494538
PCDHB3	0.0113355	0.0285686	0.0251729	0.0130892	0.0508030
ENSG00000272108	0.0287960	0.0294230	0.0235102	0.0104705	0.0578623
PCDHB4	0.0152304	0.0191002	0.0091977	0.0314243	0.0423290
PCDHB5	0.0805706	0.0677534	0.0846595	0.1746004	0.0559237
ENSG00000280029	0.0089832	0.0219538	0.0096531	0.0111089	0.0462679
PCDHB6	0.0031770	0.0094679	0.0028763	0.0048519	0.0054919
ENSG00000290893	0.0041993	0.0090382	0.0000000	0.0000000	0.0027158
ENSG00000280336	0.0009796	0.0000000	0.0000000	0.0000000	0.0031675
PCDHB7	0.0020819	0.0012307	0.0007444	0.0040528	0.0106266
PCDHB8	0.0012124	0.0000000	0.0000000	0.0000000	0.0000000
PCDHB16	0.0107591	0.0243312	0.0119525	0.0121288	0.0324296
PCDHB9	0.0380537	0.0660796	0.0772005	0.0329890	0.1079531
PCDHB10	0.0775159	0.0885544	0.1645749	0.1403198	0.1191839
PCDHB11	0.0256879	0.0329514	0.0231144	0.0313936	0.0184065
PCDHB12	0.0280927	0.0388462	0.0399142	0.0518039	0.0123159
PCDHB13	0.0369662	0.0197850	0.0259992	0.0354426	0.0263862
PCDHB14	0.0531953	0.0743687	0.0895641	0.0891703	0.0477610
PCDHB18P	0.0029183	0.0044784	0.0035625	0.0000000	0.0000000
ENSG00000290895	0.0016290	0.0057865	0.0000000	0.0057107	0.0051609
PCDHB15	0.0164950	0.0440158	0.0374174	0.0285709	0.0276730
TAF7	1.0259885	0.9438837	0.9963269	1.0401770	0.7995729
ENSG00000288095	0.0082854	0.0131608	0.0118488	0.0000000	0.0053413

ENSG00000272070	0.0064920	0.0120719	0.0059835	0.0065909	0.0129121
PCDHGA1	0.0053791	0.0055925	0.0148059	0.0120789	0.0242450
PCDHGA2	0.0004258	0.0000000	0.0000000	0.0000000	0.0024211
PCDHGA3	0.0780070	0.0798347	0.1795745	0.1735567	0.0781737
PCDHGB1	0.0000000	0.0000000	0.0026075	0.0000000	0.0000000
PCDHGA4	0.0000000	0.0000000	0.0000000	0.0000000	0.0025298
PCDHGB2	0.0010309	0.0007269	0.0036806	0.0033589	0.0085133
PCDHGA5	0.0312072	0.0326586	0.0695697	0.0444916	0.0169329
PCDHGA6	0.0067476	0.0155773	0.0164125	0.0298240	0.0404976
PCDHGA7	0.0661478	0.0836479	0.1316365	0.1526582	0.0500426
PCDHGB4	0.0217374	0.0103472	0.0345480	0.0321295	0.0102663
PCDHGA8	0.0012160	0.0037958	0.0024807	0.0000000	0.0114220
PCDHGB5	0.0045187	0.0039410	0.0009751	0.0148903	0.0080288
PCDHGA9	0.0040717	0.0034091	0.0027908	0.0132431	0.0168344
PCDHGB6	0.2419677	0.1809160	0.2795788	0.4865398	0.2015146
PCDHGA10	0.1499864	0.1179693	0.1286757	0.3242125	0.1581266
PCDHGB7	0.0277349	0.0335911	0.0182699	0.1061798	0.0567420
PCDHGA11	0.0066920	0.0087067	0.0097869	0.0178334	0.0080779
PCDHGA12	0.0052469	0.0045544	0.0020662	0.0058972	0.0250707
ENSG00000273557	0.0014666	0.0012286	0.0000000	0.0000000	0.0000000
PCDHGC3	0.0452872	0.0217712	0.0383270	0.1750998	0.0603581
PCDHGC4	0.0021254	0.0059668	0.0136454	0.0000000	0.0000000
PCDHGC5	0.0002335	0.0024980	0.0027373	0.0000000	0.0037333
DIAPH1	0.1365890	0.1832813	0.1353989	0.0869747	0.2172766
DIAPH1-AS1	0.0013805	0.0000000	0.0000000	0.0000000	0.0000000
ENSG00000228737	0.0017702	0.0000000	0.0000000	0.0000000	0.0035640
HDAC3	0.2327014	0.1914357	0.1930979	0.2177348	0.2165226
RELL2	0.0346056	0.0982685	0.1144431	0.0442754	0.0578670
FCHSD1	0.0270861	0.0284932	0.0217136	0.0200833	0.0452199
ARAP3	0.0076446	0.0024052	0.0079488	0.0040303	0.0800180
ENSG00000286736	0.0149749	0.0447199	0.0319875	0.0186393	0.1932542
ENSG00000287527	0.0022060	0.0042830	0.0000000	0.0000000	0.0334159
ENSG00000278925	0.0000000	0.0010657	0.0000000	0.0000000	0.0000000
PCDH1	0.0439436	0.0433788	0.0686178	0.0379439	0.1372723
ENSG00000287726	0.0068481	0.0163978	0.0147933	0.0000000	0.0080973
DELE1	0.2083740	0.1661628	0.1015448	0.2113440	0.2764624
PCDH12	0.0000000	0.0000000	0.0000000	0.0000000	0.0033946
RNF14	0.3594821	0.3466891	0.3074485	0.3224002	0.2955695
ENSG00000254099	0.0000000	0.0009190	0.0000000	0.0000000	0.0000000
GNPDA1	0.7306271	0.4584667	0.4311644	0.8535861	0.3674070
NDFIP1	1.3699727	1.3525392	1.4149476	1.5516342	1.2902142
ENSG00000289306	0.0007454	0.0031573	0.0028368	0.0019268	0.0143869
SPRY4	0.0114523	0.0032399	0.0194024	0.0000000	0.0378049
SPRY4-AS1	0.0176918	0.0218918	0.0344078	0.0333350	0.0686574
FGF1	0.4573045	0.2850317	0.1426673	0.5694050	0.2286923
ENSG00000261757	0.0000000	0.0000000	0.0023561	0.0000000	0.0000000
LINC01844	0.0020827	0.0040928	0.0137712	0.0103200	0.0000000
ARHGAP26	0.2468353	0.2849478	0.2657664	0.2153636	0.6110900
ARHGAP26-AS1	0.0000000	0.0012391	0.0000000	0.0000000	0.0034730
NR3C1	0.3610232	0.2853723	0.2617508	0.3576004	0.3345530
ENSG00000249881	0.0000000	0.0009512	0.0052372	0.0000000	0.0067193
YIPF5	0.5030792	0.4598712	0.3885790	0.5401765	0.3540084
KCTD16	0.5461411	0.4168899	0.3854437	0.4267148	0.7728933

ENSG00000285605	0.0000000	0.0000000	0.0000000	0.0000000	0.0179853
ENSG00000251031	0.0072691	0.0139598	0.0123475	0.0081004	0.0573659
ENSG00000250842	0.0130032	0.0127123	0.0047837	0.0070547	0.0069651
ENSG00000248125	0.0000000	0.0000000	0.0000000	0.0000000	0.0046920
PRELID2	0.1913466	0.1550999	0.1174723	0.1758504	0.2298874
SH3RF2	0.0017826	0.0010000	0.0000000	0.0058809	0.0000000
PLAC8L1	0.0028062	0.0130866	0.0018522	0.0100433	0.0350526
ENSG00000251556	0.0051303	0.0000000	0.0000000	0.0000000	0.0000000
LARS1	1.2072916	1.1728091	1.1350681	1.2442084	1.0519191
RBM27	0.5619952	0.5281548	0.5041898	0.4861066	0.5004230
ENSG00000250025	0.0012760	0.0000000	0.0000000	0.0000000	0.0000000
TCERG1	0.4654260	0.4650602	0.5051261	0.4099064	0.6751240
GPR151	0.0000000	0.0000000	0.0000000	0.0000000	0.0000000
ENSG00000250407	0.0009152	0.0035421	0.0000000	0.0073425	0.0028917
PPP2R2B	0.7945440	1.1925513	1.4221763	0.8118469	1.3785209
STK32A-AS1	0.0404920	0.0252606	0.0347020	0.0189164	0.0360837
STK32A	0.4014973	0.3718786	0.3177102	0.3696207	0.4564319
DPYSL3	1.2074432	1.7116168	2.1427615	0.9746991	1.5728543
ENSG00000272239	0.0013340	0.0054967	0.0022497	0.0084175	0.0000000
JAKMIP2-AS1	0.0098971	0.0178847	0.0152945	0.0094103	0.0675166
JAKMIP2	0.7460815	0.8473751	0.8742200	0.6523427	1.1377669
ENSG00000287630	0.0000000	0.0000000	0.0000000	0.0000000	0.0000000
SPINK1	0.0034269	0.0081927	0.0053516	0.0122172	0.0000000
SPINK5	0.0020908	0.0024629	0.0109431	0.0024157	0.0062761
FBXO38-DT	0.0932980	0.1213099	0.0919742	0.0638022	0.4177624
SPINK14	0.0000000	0.0000000	0.0000000	0.0000000	0.0000000
MARCOL	0.0016698	0.0000000	0.0000000	0.0000000	0.0123151
SPINK13	0.0000000	0.0034006	0.0038815	0.0022208	0.0030106
SPINK9	0.0021599	0.0009333	0.0029046	0.0000000	0.0136048
FBXO38	0.1479994	0.1123086	0.1175034	0.0870368	0.2168171
ENSG00000251330	0.0000000	0.0012489	0.0000000	0.0000000	0.0122556
HTR4	0.0037187	0.0027642	0.0034298	0.0070399	0.0089105
ADRB2	0.0049530	0.0041953	0.0026773	0.0025305	0.0000000
ABLIM3	0.1388601	0.2026133	0.2682261	0.1091885	0.3650565
ENSG00000248647	0.0027938	0.0011064	0.0000000	0.0000000	0.0187107
ENSG00000253406	0.0000000	0.0000000	0.0000000	0.0000000	0.0000000
AFAP1L1	0.0071972	0.0089884	0.0029405	0.0095225	0.0148023
ENSG00000285736	0.0000000	0.0000000	0.0017803	0.0000000	0.0000000
GRPEL2	0.1201048	0.1648390	0.1523966	0.1072672	0.1559089
GRPEL2-AS1	0.0037268	0.0048654	0.0010329	0.0000000	0.0000000
PCYOX1L	0.1081293	0.0989046	0.1124724	0.1277589	0.1323081
IL17B	0.0020556	0.0013043	0.0000000	0.0000000	0.0088926
CARMN	0.0000000	0.0000000	0.0000000	0.0000000	0.0000000
CSNK1A1	1.4385210	1.3656432	1.3659424	1.3331032	1.2401460
ARHGEF37	0.0595431	0.0542162	0.0366651	0.0482416	0.0418457
PPARGC1B	0.0130584	0.0599595	0.0455527	0.0090035	0.0999291
PDE6A	0.0000000	0.0000000	0.0024972	0.0000000	0.0000000
SLC26A2	0.1445634	0.1146795	0.0997415	0.1538942	0.1712058
TIGD6	0.0614854	0.0317704	0.0336231	0.0228522	0.0495268
HMGXB3	0.2139451	0.1760037	0.1826721	0.2206073	0.2262943
CSF1R	0.0027853	0.0019978	0.0031553	0.0015041	0.0044554
PDGFRB	0.0587527	0.0257254	0.0086718	0.1025454	0.0339735
SLC6A7	0.0048131	0.0066060	0.0117203	0.0033357	0.0098463

CAMK2A	0.1109567	0.1210969	0.1546935	0.1294701	0.2205228
ARSI	0.0049480	0.0053237	0.0000000	0.0097021	0.0058321
TCOF1	0.1758794	0.2131250	0.2223275	0.1760415	0.3162485
CD74	0.0177601	0.0123004	0.0206436	0.0076766	0.0276938
RPS14	3.0989781	3.0555041	2.8666871	3.0017766	2.7951086
NDST1-AS1	0.0104080	0.0060901	0.0047169	0.0173190	0.0038113
NDST1	0.1243739	0.1320583	0.0957522	0.1080627	0.1817710
SYNPO	0.0167118	0.0201478	0.0193830	0.0454599	0.0255441
MYOZ3	0.0092086	0.0206154	0.0311980	0.0101526	0.0187868
RBM22	0.4902060	0.4444394	0.4869579	0.5068935	0.3865261
DCTN4	0.5786676	0.5376843	0.5331152	0.5618093	0.4929008
SMIM3	0.0652008	0.0580933	0.0500772	0.0501090	0.0230466
IRGM	0.0007085	0.0012260	0.0000000	0.0000000	0.0000000
ZNF300	0.0546277	0.0885989	0.0827147	0.0550463	0.1249592
ZNF300P1	0.0118617	0.0026685	0.0102889	0.0000000	0.0021475
GPX3	0.1025851	0.1007274	0.1170775	0.0657976	0.0711198
TNIP1	0.2769135	0.2298561	0.1746590	0.2763166	0.1795179
ENSG00000289970	0.0010483	0.0015640	0.0034087	0.0103656	0.0031863
ANXA6	0.7135996	0.4210474	0.2653229	0.6953821	0.4462366
CCDC69	0.0117491	0.0161281	0.0038815	0.0106904	0.0066207
GM2A	0.1031980	0.0811956	0.0914036	0.1742240	0.0599347
SLC36A1	0.0315514	0.0476896	0.0750231	0.0258396	0.0394478
FAT2	0.0011455	0.0009229	0.0017636	0.0000000	0.0031628
ENSG00000271795	0.0024648	0.0014125	0.0000000	0.0000000	0.0000000
ENSG00000288081	0.0000000	0.0023848	0.0000000	0.0000000	0.0000000
ENSG00000260581	0.0000000	0.0007390	0.0061229	0.0000000	0.0016917
SPARC	1.4956007	0.9840975	0.6922954	2.2792613	0.9273161
CLMAT3	0.0011594	0.0000000	0.0000000	0.0000000	0.0045212
ENSG00000272112	0.0015973	0.0091204	0.0013879	0.0014497	0.0053985
ATOX1	0.9172393	1.0449934	1.1207121	0.8809703	0.8346234
ATOX1-AS1	0.0017752	0.0000000	0.0000000	0.0000000	0.0030832
ENSG00000275765	0.0447548	0.0350626	0.0325395	0.0279900	0.0246174
G3BP1	0.4627563	0.4210622	0.3443278	0.4845976	0.4877670
GLRA1	0.0119718	0.0221162	0.0154757	0.0032175	0.0873270
LINC01933	0.0140778	0.0287417	0.0149415	0.0158528	0.0779867
ENSG00000286749	0.0208813	0.0293394	0.0259040	0.0302066	0.1100414
NMUR2	0.0000000	0.0000000	0.0000000	0.0000000	0.0000000
LINC01470	0.0017711	0.0034847	0.0000000	0.0000000	0.0042436
GRIA1	0.2556862	0.2079763	0.2808467	0.4540358	0.8330045
FAM114A2	0.2017424	0.1231894	0.1089089	0.1687882	0.1249406
MFAP3	0.1496416	0.1702139	0.2117058	0.2139001	0.2891290
GALNT10	0.0438675	0.0409054	0.0235528	0.0958141	0.0928921
SAP30L-AS1	0.0082089	0.0067994	0.0017758	0.0093598	0.0000000
SAP30L	0.2072379	0.1805225	0.1785354	0.2146019	0.1270737
LARP1	0.9927004	1.0970279	1.0979968	0.9435165	0.9784533
FAXDC2	0.1289195	0.0822554	0.0386743	0.1514855	0.0801434
CNOT8	0.2401847	0.2476924	0.2345007	0.2216812	0.2352417
GEMIN5	0.1481897	0.1137383	0.0982211	0.1558878	0.1949490
MRPL22	0.4177927	0.3993988	0.4833421	0.3224281	0.3059120
KIF4B	0.0032020	0.0067074	0.0072485	0.0000000	0.0017501
ENSG00000287963	0.0000000	0.0000000	0.0000000	0.0017320	0.0149115
SGCD	0.1575402	0.2127571	0.1253208	0.1689511	0.7463531
ENSG00000286690	0.0000000	0.0000000	0.0000000	0.0000000	0.0000000

ENSG00000254163	0.0000000	0.0000000	0.0000000	0.0000000	0.0000000
PPP1R2B	0.0003754	0.0010939	0.0048169	0.0028821	0.0035043
HAVCR1	0.0000000	0.0000000	0.0000000	0.0000000	0.0000000
HAVCR2	0.0596388	0.0994842	0.0986374	0.0656377	0.1407673
MED7	0.2065990	0.2220953	0.2896018	0.2167636	0.1623148
ITK	0.0000000	0.0000000	0.0000000	0.0000000	0.0000000
ENSG00000253653	0.0027439	0.0042023	0.0021379	0.0077540	0.0070801
CYFIP2	0.4673724	0.6556510	0.7335662	0.4797110	0.7482639
FNDC9	0.0000000	0.0127383	0.0298707	0.0044443	0.0025600
NIPAL4-DT	0.0000000	0.0000000	0.0000000	0.0000000	0.0000000
ENSG00000248544	0.0010451	0.0000000	0.0000000	0.0000000	0.0216148
ADAM19	0.0422864	0.0827011	0.0632870	0.0319521	0.1224570
NIPAL4	0.0000000	0.0000000	0.0000000	0.0000000	0.0000000
SOX30	0.0346196	0.0361245	0.0363248	0.0362969	0.0170224
C5orf52	0.0025361	0.0040153	0.0033069	0.0074652	0.0105364
THG1L	0.1173317	0.0954205	0.0508385	0.0930880	0.1164197
LSM11	0.2958624	0.5485589	0.6612769	0.2319939	0.4442649
CLINT1	0.6536944	0.5493376	0.5903797	0.6906048	0.5845311
ENSG00000254135	0.0000000	0.0000000	0.0000000	0.0000000	0.0000000
EBF1	0.1839028	0.3146569	0.2940305	0.1804912	0.9448299
ENSG00000253811	0.0000000	0.0000000	0.0000000	0.0000000	0.0000000
LINC02202	0.0066098	0.0126462	0.0125635	0.0053087	0.0000000
RNF145	0.3640595	0.3768253	0.4164302	0.4229979	0.5195408
ENSG00000253256	0.0000000	0.0018352	0.0000000	0.0000000	0.0000000
ENSG00000288764	0.0100725	0.0079468	0.0084237	0.0091629	0.0172450
UBLCP1	0.4843320	0.4721893	0.4856000	0.4940985	0.4513547
ENSG00000249738	0.0029380	0.0059041	0.0075902	0.0000000	0.0248002
LINC01847	0.0020707	0.0020437	0.0000000	0.0000000	0.0000000
ADRA1B	0.0154783	0.0307476	0.0655086	0.0163435	0.0745906
TTC1	0.8047777	0.7461494	0.6915815	0.7738622	0.6770923
PWWP2A	0.2980447	0.3228940	0.3157087	0.2855355	0.3574952
FABP6	0.0402953	0.0938723	0.1434151	0.0248823	0.0285986
FABP6-AS1	0.0010310	0.0006583	0.0000000	0.0000000	0.0097408
CCNJL	0.0047432	0.0081507	0.0010465	0.0122568	0.0356359
C1QTNF2	0.0033602	0.0067559	0.0063645	0.0049134	0.0034730
FAM200C	0.1403224	0.1142306	0.1352418	0.1047498	0.0853576
SLU7	0.5847555	0.5123534	0.4952480	0.5358525	0.4214229
PTTG1	0.1117580	0.1217125	0.0941414	0.0896446	0.1379019
ATP10B	0.0002861	0.0000000	0.0010221	0.0000000	0.0000000
GABRB2	0.0906524	0.1397201	0.1503819	0.0750023	0.2785020
GABRA6	0.0040313	0.0112593	0.0043343	0.0000000	0.0191630
GABRA1	0.0276425	0.0470727	0.0393240	0.0111231	0.0485035
GABRG2	0.1432045	0.3370090	0.3753706	0.1246508	0.3367935
ENSG00000254186	0.0385270	0.0201181	0.0220622	0.0369276	0.0776152
CCNG1	0.8416062	0.7452560	0.6737245	0.7988946	0.5413298
NUDCD2	0.6146597	0.5185876	0.4641027	0.7159128	0.4347275
HMMR	0.0148581	0.0203215	0.0180525	0.0200816	0.0109932
MAT2B	0.2757135	0.1658604	0.1099187	0.2044867	0.1565765
LINC03000	0.1118877	0.1097957	0.0710786	0.0632392	0.5174520
LINC02143	0.0000000	0.0038945	0.0000000	0.0000000	0.0119064
ENSG00000285549	0.0033823	0.0000000	0.0000000	0.0000000	0.0063483
LINC01938	0.0000000	0.0000000	0.0000000	0.0000000	0.0000000
ENSG00000253693	0.0670059	0.0476238	0.0491885	0.0176584	0.3424285

ENSG00000254171	0.0023148	0.0014395	0.0066296	0.0061340	0.0077374
LINC01947	0.0000000	0.0000000	0.0000000	0.0000000	0.0037529
TENM2	0.6005756	0.6798173	0.7027227	0.5739261	2.2358772
ENSG00000254365	0.0000000	0.0002844	0.0000000	0.0000000	0.0061121
ENSG00000253527	0.0000000	0.0000000	0.0000000	0.0000000	0.0000000
ENSG00000289264	0.0000000	0.0008251	0.0000000	0.0000000	0.0298086
ENSG00000254187	0.0000000	0.0000000	0.0016935	0.0036094	0.0588928
ENSG00000253660	0.0000000	0.0000000	0.0000000	0.0000000	0.0014580
ENSG00000253925	0.0189298	0.0065955	0.0033217	0.0262927	0.0456645
TENM2-AS1	0.0180354	0.0115805	0.0147615	0.0215999	0.0239287
WWC1	0.2084285	0.1295161	0.0896664	0.3035276	0.1894444
RARS1	0.3926672	0.3363288	0.3523227	0.3834772	0.3690032
FBLL1	0.1918432	0.3223787	0.4360589	0.1620046	0.2243505
PANK3	0.5920063	0.8344325	0.9829399	0.5713823	0.8087524
ENSG00000254192	0.0000000	0.0046450	0.0039299	0.0000000	0.0032853
SLIT3	0.3143364	0.5241454	0.2667903	0.3980275	1.0933120
SLIT3-AS2	0.0000000	0.0017483	0.0013494	0.0000000	0.0040472
ENSG00000248965	0.0000000	0.0000000	0.0000000	0.0000000	0.0035216
SLIT3-AS1	0.0000000	0.0000000	0.0000000	0.0000000	0.0000000
SPDL1	0.2285450	0.2400739	0.2402449	0.2250362	0.2059570
DOCK2	0.0000000	0.0051137	0.0057572	0.0000000	0.0185386
INSYN2B	0.0253559	0.0589525	0.0806218	0.0202728	0.1885287
C5orf58	0.0003125	0.0027705	0.0000000	0.0040556	0.0132534
LCP2	0.0064445	0.0013977	0.0028040	0.0000000	0.0000000
LINC01366	0.0000000	0.0000000	0.0000000	0.0000000	0.0000000
KCNIP1	0.1563372	0.2150846	0.2201757	0.1320423	0.6017949
KCNMB1	0.0248995	0.0268725	0.0131645	0.0400816	0.0177905
KCNIP1-AS1	0.0000000	0.0000000	0.0000000	0.0091614	0.0309481
GABRP	0.0000000	0.0000000	0.0000000	0.0000000	0.0000000
RANBP17	0.1603346	0.1919023	0.1882026	0.1483918	0.6457262
ENSG00000275038	0.0000000	0.0000000	0.0000000	0.0000000	0.0000000
TLX3	0.0015898	0.0008577	0.0019410	0.0000000	0.0000000
NPM1	2.3716462	2.1434870	2.0560964	2.2689497	1.9429954
FGF18	0.0189935	0.0147129	0.0213850	0.0065220	0.0198045
FBXW11	0.8175809	0.7493594	0.7990912	0.7271984	0.7298085
STK10	0.0301957	0.0186454	0.0255156	0.0248989	0.1050889
UBTD2	0.4001480	0.4047621	0.4247706	0.4068051	0.4111092
SH3PXD2B	0.2796178	0.2863028	0.2523321	0.3157750	0.3239926
ENSG00000287814	0.0075119	0.0014749	0.0036169	0.0095513	0.0000000
NEURL1B	0.0306556	0.0394372	0.0356721	0.0165939	0.0446483
ENSG00000253295	0.0000000	0.0000000	0.0041787	0.0000000	0.0025607
ENSG00000253736	0.0058409	0.0037513	0.0009351	0.0049314	0.0245499
DUSP1	0.2341726	0.2013718	0.1829345	0.2260377	0.2142977
ERGIC1	0.3389652	0.3358626	0.3245085	0.4344829	0.3558109
RPL26L1-AS1	0.0217498	0.0223392	0.0238869	0.0337803	0.0249860
ENSG00000254295	0.0002427	0.0003686	0.0000000	0.0000000	0.0000000
RPL26L1	0.6087029	0.7018073	0.7507297	0.6574602	0.5157694
ATP6V0E1	0.9670740	0.6558766	0.4611858	1.0683070	0.6221145
CREBRF	0.3214312	0.2408900	0.2220068	0.2752450	0.3670312
ENSG00000253172	0.0000000	0.0000000	0.0000000	0.0000000	0.0092211
BNIP1	0.1214395	0.1494357	0.1318047	0.1186225	0.1743676
STC2	1.3396892	0.9184055	0.6982955	1.3131034	0.7928854
BOD1	0.8795786	0.8371122	0.7917394	0.8795394	0.7332936

LINC01484	0.0000000	0.0025235	0.0000000	0.0000000	0.0021357
CPEB4	0.6693165	0.8630235	1.0152248	0.7005481	0.8812942
ENSG00000289085	0.0041083	0.0037114	0.0000000	0.0047839	0.0592366
C5orf47	0.0038401	0.0090889	0.0044924	0.0130516	0.0079807
NSG2	0.8907700	1.4534114	2.3941259	0.7541770	1.4426466
ENSG00000253447	0.0000000	0.0000000	0.0000000	0.0000000	0.0000000
LINC01411	0.0028394	0.0139645	0.0076929	0.0059384	0.0421020
MSX2	0.0019513	0.0000000	0.0028783	0.0012954	0.0000000
ENSG00000251670	0.0011410	0.0013052	0.0016522	0.0000000	0.0061591
LINC01951	0.0016171	0.0019204	0.0014397	0.0000000	0.0037187
DRD1	0.2509795	0.1123078	0.0677499	0.2147613	0.1267520
SFXN1	0.5345338	0.6018768	0.7524308	0.5316903	0.6177181
HRH2	0.0000000	0.0098045	0.0210513	0.0016217	0.0232837
CPLX2	0.1411709	0.3739096	0.3409596	0.1410341	0.3987467
ENSG00000250820	0.0016407	0.0010278	0.0051615	0.0043581	0.0000000
THOC3	0.0497573	0.0553818	0.0808884	0.0445114	0.0427092
ENSG00000289731	0.0004899	0.0007701	0.0014073	0.0036162	0.0051485
ENSG00000290935	0.0006784	0.0057876	0.0000000	0.0000000	0.0023258
ENSG00000287579	0.0014533	0.0018021	0.0023023	0.0000000	0.0177055
ENSG00000283235	0.0035403	0.0149046	0.0068182	0.0041394	0.0181195
SIMC1	0.1223196	0.1539299	0.1885507	0.1298589	0.2860050
KIAA1191	0.5116960	0.6857284	0.7681015	0.4945090	0.5192400
ENSG00000250909	0.0005736	0.0006593	0.0027102	0.0000000	0.0172099
ENSG00000251414	0.0010423	0.0000000	0.0000000	0.0000000	0.0000000
ARL10	0.1897363	0.2465555	0.2725069	0.1683526	0.2200531
NOP16	0.0849377	0.1089854	0.1181920	0.0905525	0.0625447
HIGD2A	0.7900699	0.8101540	0.8032525	0.7980868	0.6501136
CLTB	0.8139893	1.0905521	1.2762798	0.8053984	0.8058255
FAF2	0.3406785	0.3331123	0.3546764	0.4829268	0.3940055
RNF44	0.1881583	0.2359783	0.2253061	0.1316609	0.2638103
CDHR2	0.0148829	0.0235761	0.0229390	0.0045542	0.0244218
GPRIN1	0.1006095	0.2556124	0.3301742	0.0666767	0.1781762
SNCB	0.2433029	0.5506494	0.8531285	0.1945913	0.3407592
EIF4E1B	0.0050454	0.0229342	0.0271293	0.0083901	0.0173059
TSPAN17	0.1366512	0.2033333	0.2243079	0.1076153	0.1491085
ENSG00000248484	0.0015382	0.0000000	0.0048799	0.0000000	0.0000000
LINC01574	0.0079409	0.0128345	0.0156707	0.0019841	0.0043076
UNC5A	0.0171278	0.0457415	0.0526637	0.0340840	0.1001474
HK3	0.0003270	0.0000000	0.0022290	0.0036595	0.0058742
UIMC1	0.2300339	0.2392383	0.2418458	0.1744055	0.3306333
ZNF346	0.1606421	0.1739042	0.1860609	0.1961014	0.2472888
FGFR4	0.0027842	0.0056658	0.0021106	0.0000000	0.0166310
NSD1	0.5610362	0.5924532	0.6397728	0.5199528	0.6127180
ENSG00000286634	0.0000000	0.0000000	0.0000000	0.0000000	0.0000000
RAB24	0.0424447	0.0526744	0.0524753	0.0480329	0.0366408
MXD3	0.0125584	0.0097441	0.0101324	0.0087151	0.0434067
PRELID1	0.7243170	0.7328814	0.7517469	0.7707211	0.4889322
LMAN2	0.6777552	0.5237354	0.4635705	0.8595101	0.5113746
RGS14	0.1094595	0.1796981	0.1446184	0.0943796	0.1034870
F12	0.1002131	0.1737889	0.3196671	0.0857250	0.1286815
GRK6	0.1603656	0.1856431	0.2394116	0.1149092	0.1816024
PRR7-AS1	0.0049825	0.0039585	0.0000000	0.0044469	0.0112221
PRR7	0.4278634	0.4092312	0.4507936	0.5072536	0.3607281

DBN1	0.5494599	0.5672981	0.5263770	0.4917805	0.5864673
PDLIM7	0.2105623	0.2110771	0.2241035	0.2857775	0.1979500
PDLIM7-AS1	0.0002926	0.0000000	0.0000000	0.0000000	0.0050220
DOK3	0.0004277	0.0089890	0.0074483	0.0025774	0.0270118
DDX41	0.3079065	0.3374923	0.3787444	0.3240101	0.2470460
FAM193B	0.1147855	0.1305463	0.1420893	0.1250564	0.2733527
FAM193B-DT	0.0037238	0.0100631	0.0087390	0.0000000	0.0000000
TMED9	0.8864258	0.6651233	0.6757814	1.2032296	0.7152057
B4GALT7	0.2962406	0.2221061	0.1777580	0.3159487	0.1492724
ENSG00000247679	0.0126174	0.0166078	0.0056584	0.0031752	0.0053416
ENSG00000291283	0.0133047	0.0379453	0.0293358	0.0230589	0.1335045
ENSG00000290968	0.0198569	0.0197034	0.0157375	0.0153956	0.1138322
FAM153A	0.0045253	0.0020329	0.0016417	0.0039481	0.0105586
ENSG00000291113	0.2218387	0.2566850	0.3388397	0.1663865	0.1416605
ENSG00000250101	0.0136235	0.0238744	0.0146161	0.0028893	0.0958230
ENSG00000249684	0.0236070	0.0477174	0.0222753	0.0475662	0.0190688
FAM153CP	0.0171218	0.0138198	0.0112317	0.0113219	0.0535534
N4BP3	0.0511461	0.0595820	0.0865776	0.0465181	0.0810313
RMND5B	0.1493264	0.1477243	0.1310444	0.1364118	0.1470186
NHP2	0.8330421	0.8455025	0.8907663	0.7542979	0.5377833
ENSG00000289726	0.0082279	0.0052852	0.0016423	0.0045266	0.0370186
ENSG00000289390	0.0013750	0.0010952	0.0032424	0.0034922	0.0000000
HNRNPAB	0.6695485	0.8093229	0.9812278	0.7305617	0.5642745
PHYKPL	0.1034074	0.0866194	0.0471267	0.1033337	0.0792252
COL23A1	0.0515861	0.0379048	0.0273010	0.1201133	0.1025490
CLK4	0.1187035	0.1086047	0.1113870	0.0953736	0.1307802
MSANTD5	0.0010008	0.0000000	0.0000000	0.0000000	0.0000000
ZNF354A	0.1439257	0.1870028	0.1996089	0.1173333	0.1232403
AACSP1	0.0000000	0.0000000	0.0000000	0.0000000	0.0000000
ZNF354B	0.0846659	0.1002527	0.0485121	0.0884628	0.1247483
ZFP2	0.0307140	0.0448684	0.0421437	0.0255295	0.1359875
ZNF454-DT	0.0017643	0.0000000	0.0000000	0.0000000	0.0000000
ZNF454	0.0531898	0.0908697	0.0716168	0.0571757	0.1370778
ENSG00000254035	0.0060856	0.0170775	0.0239201	0.0235445	0.0395078
GRM6	0.0005507	0.0081779	0.0048534	0.0034303	0.0063341
ZNF879	0.0600167	0.0589491	0.0436170	0.0706240	0.0631191
ZNF354C	0.0941999	0.1047138	0.1282183	0.0649072	0.1578923
ADAMTS2	0.0522468	0.0408469	0.0126620	0.0711080	0.0818910
ENSG00000290043	0.0022323	0.0000000	0.0063563	0.0000000	0.0012770
RUFY1	0.3845599	0.2586930	0.2342587	0.3705450	0.3654232
RUFY1-AS1	0.0000000	0.0009175	0.0024126	0.0000000	0.0073097
HNRNPH1	0.8009573	0.8352443	0.9484282	0.7666231	0.8437032
ENSG00000250999	0.0055087	0.0116635	0.0117236	0.0058345	0.0266703
CBY3	0.0000000	0.0038528	0.0000000	0.0000000	0.0026227
CANX	2.2035985	1.7710654	1.7223811	2.7077156	1.7204529
MAML1	0.0638629	0.0778667	0.0557841	0.0465934	0.1990508
LTC4S	0.0123407	0.0177640	0.0215468	0.0066892	0.0214645
MGAT4B	0.7949608	0.7034768	0.6375291	0.8985665	0.5224027
SQSTM1	1.3011600	0.9605560	0.7994799	1.2654444	0.8314507
MRNIP	0.3067816	0.2467581	0.2842160	0.2790815	0.3347812
MRNIP-DT	0.0656614	0.0760293	0.0675246	0.0747595	0.0968383
TBC1D9B	0.3178515	0.3151840	0.2479579	0.3167566	0.3875478
RNF130	0.7883499	0.7090431	0.5689165	0.7768113	0.9860938

ENSG00000285865	0.0002931	0.0005202	0.0000000	0.0000000	0.0000000
RASGEF1C	0.0409944	0.1005308	0.1094868	0.0165465	0.1696988
MAPK9	0.3989236	0.6793075	0.7855687	0.3686080	0.5864876
ENSG00000248367	0.0390803	0.0298957	0.0321192	0.0372221	0.0335410
GFPT2	0.0372767	0.0390349	0.0201075	0.0245226	0.0808827
ENSG00000250509	0.0009235	0.0033275	0.0000000	0.0000000	0.0035390
CNOT6	0.2524752	0.2924724	0.3204274	0.2160519	0.3903336
SCGB3A1	0.0041359	0.0117672	0.0013709	0.0033570	0.0085005
FLT4	0.0025930	0.0040979	0.0052974	0.0000000	0.0000000
MGAT1	0.1464118	0.1431630	0.1372113	0.1435080	0.0657634
HEIH	0.1743067	0.1612540	0.1627840	0.1566154	0.1534694
LINC00847	0.2155098	0.1844783	0.1692765	0.1697505	0.1052108
ZFP62	0.1010532	0.1275639	0.1234022	0.0993539	0.1524881
BTNL9	0.0157198	0.0458286	0.0758966	0.0316429	0.0771672
LINC01962	0.0007442	0.0038419	0.0000000	0.0000000	0.0000000
TRIM7-AS2	0.2183173	0.1888056	0.1663639	0.2778616	0.1683682
TRIM7	0.0126219	0.0240158	0.0329516	0.0098299	0.0319551
TRIM7-AS1	0.0003905	0.0008078	0.0000000	0.0000000	0.0000000
ENSG00000248514	0.0024818	0.0086320	0.0126125	0.0039942	0.0201372
ENSG00000286644	0.0020591	0.0000000	0.0037788	0.0000000	0.0035640
TRIM41	0.1298705	0.1160641	0.0708189	0.0808308	0.1245130
RACK1	2.5124996	2.4912197	2.3090996	2.4000852	2.2051389
CTC-338M12.4	0.0833461	0.0694238	0.0439099	0.0499666	0.0794468
TRIM52	0.1330336	0.1673986	0.1896356	0.0838246	0.1861447
TRIM52-AS1	0.2898416	0.2625292	0.2662028	0.2388434	0.1660782
ENSG00000286310	0.0000000	0.0012453	0.0007808	0.0000000	0.0045730
ENSG00000248103	0.0040184	0.0035342	0.0016185	0.0039294	0.0148721
ENSG00000238035	0.0009020	0.0000000	0.0076076	0.0018378	0.0074301
ENSG00000285578	0.0006630	0.0060268	0.0000000	0.0025606	0.0223664
ENSG00000287265	0.0002628	0.0010598	0.0048898	0.0021003	0.0000000
DUSP22	0.2126294	0.2229323	0.2572848	0.2377412	0.2671681
IRF4	0.0000000	0.0000000	0.0000000	0.0000000	0.0000000
ENSG00000286364	0.0000000	0.0000000	0.0000000	0.0000000	0.0000000
EXOC2	0.2392593	0.2459098	0.3055847	0.2089011	0.4110056
HUS1B	0.0034654	0.0043285	0.0020684	0.0054717	0.0000000
ENSG00000271727	0.0129264	0.0098703	0.0144994	0.0120230	0.0312494
ENSG00000287293	0.0000000	0.0000000	0.0000000	0.0000000	0.0027533
LINC01622	0.0036087	0.0007412	0.0077394	0.0043864	0.0000000
FOXQ1	0.0093857	0.0042711	0.0066044	0.0000000	0.0132554
FOXF2	0.0044807	0.0076289	0.0024578	0.0036117	0.0242852
FOXC1	0.0024406	0.0014310	0.0000000	0.0094977	0.0000000
GMDS	0.1398608	0.1601270	0.1406297	0.1060418	0.4999942
ENSG00000285603	0.0000000	0.0000000	0.0000000	0.0000000	0.0054850
GMDS-DT	0.1786952	0.1821854	0.1075261	0.1827576	0.3585248
ENSG00000286512	0.0000000	0.0010897	0.0000000	0.0000000	0.0017069
ENSG00000287903	0.0028274	0.0030757	0.0000000	0.0000000	0.0000000
LINC01600	0.0060975	0.0086163	0.0109260	0.0000000	0.0163380
MYLK4	0.0084368	0.0059940	0.0068612	0.0143269	0.0141334
WRNIP1	0.3118660	0.3597409	0.4158763	0.3187830	0.3413946
SERPINB1	0.1998093	0.0951640	0.0606457	0.2232505	0.0714318
SERPINB9P1	0.0010243	0.0003924	0.0019772	0.0000000	0.0035834
SERPINB9	0.0710996	0.1534479	0.2480437	0.0374741	0.1408899
SERPINB6	0.7733686	0.5540713	0.3771303	0.7732456	0.3651447

LINC01011	0.0477604	0.0345872	0.0542616	0.0363816	0.0456117
NQO2	0.1860726	0.2619379	0.2239350	0.2313990	0.1824814
NQO2-AS1	0.0286385	0.0145208	0.0212778	0.0054485	0.0000000
ENSG00000288612	0.0115668	0.0160590	0.0080903	0.0055898	0.0637545
RIPK1	0.1472466	0.1075736	0.1320206	0.1479581	0.1620068
ENSG00000272277	0.0338409	0.0258926	0.0225538	0.0011694	0.0174720
BPHL	0.1072361	0.1086807	0.1683546	0.1450221	0.1282716
ENSG00000228170	0.0013475	0.0014552	0.0015424	0.0038821	0.0102228
TUBB2A	1.7171017	2.1816583	2.6619005	1.9464920	1.8123268
LINC02525	0.0724975	0.1143079	0.1150359	0.0740912	0.0730198
TUBB2B	2.4859471	2.7562974	3.0690021	2.6130044	2.4023318
ENSG00000288840	0.0143278	0.0248976	0.0111853	0.0159913	0.0269739
PSMG4	0.2494561	0.2817054	0.2947266	0.2452413	0.3493539
SLC22A23	0.1076272	0.1088465	0.1537930	0.0757497	0.2865572
ENSG00000228793	0.0119701	0.0062581	0.0028663	0.0070768	0.0154374
PXDC1	0.4681115	0.2546939	0.1487017	0.5546126	0.2093255
ENSG00000270504	0.0404351	0.0297628	0.0313168	0.0596948	0.0124869
FAM50B	0.1090566	0.0860593	0.1065404	0.1254852	0.0867568
ENSG00000260604	0.0042592	0.0013723	0.0000000	0.0000000	0.0000000
ENSG00000288904	0.0053664	0.0108346	0.0270661	0.0143097	0.0320958
ENSG00000230648	0.0243024	0.0242078	0.0301270	0.0250063	0.0287377
PRPF4B	0.7428405	0.5961983	0.5937850	0.6634988	0.6559402
FAM217A	0.0007710	0.0014195	0.0000000	0.0000000	0.0000000
ECI2	0.5120892	0.3932920	0.2461529	0.5740976	0.3713891
ECI2-DT	0.0002717	0.0000000	0.0000000	0.0024514	0.0000000
ENSG00000285424	0.0032332	0.0017934	0.0000000	0.0000000	0.0056151
ENSG00000285694	0.0017585	0.0000000	0.0000000	0.0000000	0.0000000
CDYL	0.3855865	0.2872561	0.2081367	0.4675599	0.3697512
RPP40	0.0813480	0.0996296	0.1409773	0.0489149	0.0583638
LYRM4-AS1	0.0159622	0.0111826	0.0135349	0.0175261	0.0053413
ENSG00000271978	0.0120027	0.0224381	0.0093077	0.0059656	0.0046792
ENSG00000288548	0.0050730	0.0046102	0.0012145	0.0043584	0.0000000
PPP1R3G	0.0384816	0.0129625	0.0111452	0.0404471	0.0236718
LYRM4	0.4658403	0.4560785	0.4124125	0.4765252	0.4429346
FARS2	0.2010904	0.2088345	0.2117817	0.2362214	0.4876639
FARS2-AS1	0.0014386	0.0000000	0.0000000	0.0000000	0.0060461
ENSG00000270174	0.0000000	0.0000000	0.0000000	0.0000000	0.0030106
ENSG00000233064	0.0026410	0.0063019	0.0098471	0.0053449	0.0323139
NRN1	0.1358948	0.2406555	0.4068081	0.1009699	0.2966249
F13A1	0.0137160	0.0000000	0.0054180	0.0069868	0.0058801
LY86-AS1	0.0008858	0.0026014	0.0000000	0.0040415	0.0000000
ENSG00000261211	0.0000000	0.0057022	0.0062509	0.0000000	0.0056180
ENSG00000226281	0.0000000	0.0017223	0.0046257	0.0000000	0.0000000
ENSG00000287497	0.0000000	0.0014218	0.0000000	0.0000000	0.0058922
ENSG00000288046	0.0016621	0.0019506	0.0011261	0.0000000	0.0016281
RREB1	0.1095204	0.0526814	0.0387114	0.1079947	0.0463281
SSR1	0.6023441	0.5265807	0.5545189	0.7207066	0.5033562
ENSG00000238221	0.0041548	0.0020812	0.0000000	0.0000000	0.0054053
CAGE1	0.0004361	0.0000000	0.0024615	0.0037396	0.0158226
RIOK1	0.1898646	0.3089078	0.3510146	0.2222490	0.1696324
DSP-AS1	0.0107513	0.0091862	0.0056381	0.0125671	0.0085992
DSP	0.2387960	0.1326746	0.1284051	0.2282144	0.1222939
SNRNP48	0.1315774	0.1239745	0.1220187	0.1132465	0.1411490

BMP6	0.0330247	0.0142199	0.0170225	0.0943734	0.0353561
TXNDC5	0.2415033	0.1814251	0.1525282	0.3099935	0.1866804
BLOC1S5	0.2350188	0.2923509	0.2237656	0.3430211	0.1812925
EEF1E1	0.4760863	0.5113697	0.5751234	0.4671480	0.3225024
ENSG00000232234	0.0000000	0.0042546	0.0000000	0.0056883	0.0241721
SLC35B3	0.1270229	0.1704006	0.1443572	0.1474783	0.1537245
HULC	0.0759046	0.1022769	0.0937172	0.0400094	0.3280264
ENSG00000225775	0.0040337	0.0017274	0.0008856	0.0000000	0.0000000
TFAP2A	0.0077529	0.0108910	0.0062756	0.0035133	0.0489897
TFAP2A-AS2	0.0008233	0.0015135	0.0000000	0.0000000	0.0000000
TFAP2A-AS1	0.0016116	0.0000000	0.0000000	0.0000000	0.0103581
GCNT2	0.0240003	0.0413107	0.0434640	0.0301532	0.0945942
ENSG00000285763	0.0006736	0.0000000	0.0007943	0.0000000	0.0000000
C6orf52	0.0624408	0.0613001	0.1055066	0.0626785	0.0610559
PAK1IP1	0.3056809	0.3703873	0.5028242	0.3574973	0.2676811
TMEM14C	1.0877489	1.0543516	1.0220261	1.0780119	0.8452446
TMEM14B-DT	0.0039654	0.0082076	0.0068455	0.0000000	0.0110587
TMEM14B	1.0162953	1.0259755	0.9733522	1.0079501	0.9717286
MAK	0.0492809	0.0234951	0.0132307	0.0538170	0.1387771
GCM2	0.0000000	0.0000000	0.0020417	0.0000000	0.0000000
ENSG00000235051	0.0052538	0.0000000	0.0124574	0.0000000	0.0090410
SYCP2L	0.0476285	0.0516087	0.0663246	0.0498358	0.1952109
ELOVL2	0.0592807	0.0765772	0.1153802	0.0514794	0.0885723
ELOVL2-AS1	0.0026547	0.0156771	0.0250873	0.0000000	0.0129464
SMIM13	0.2999108	0.4316504	0.6055506	0.2108623	0.4307703
ENSG00000247925	0.0109129	0.0119823	0.0056986	0.0062368	0.0525832
NEDD9	0.2127842	0.1223458	0.0786851	0.2273379	0.1252807
ENSG00000287920	0.0005766	0.0000000	0.0000000	0.0000000	0.0000000
ENSG00000242753	0.0000000	0.0008939	0.0000000	0.0000000	0.0000000
TMEM170B	0.2514502	0.2880010	0.3051936	0.1876564	0.3127935
ENSG00000229896	0.0003794	0.0013092	0.0014933	0.0033824	0.0000000
HIVEP1	0.2179355	0.2545228	0.3183185	0.2346824	0.5020505
EDN1	0.0013686	0.0019397	0.0070514	0.0042299	0.0094889
PHACTR1	0.3939751	0.6995396	0.6822581	0.3736165	1.2672498
ENSG00000215022	0.0048549	0.0106260	0.0027665	0.0026518	0.0028917
TBC1D7	0.2176538	0.3374565	0.3831814	0.1943123	0.2157135
ENSG00000272379	0.0030322	0.0062198	0.0035863	0.0000000	0.0152664
ENSG00000289257	0.0076219	0.0061376	0.0026409	0.0068763	0.0076685
GFOD1	0.0935484	0.2239491	0.2713882	0.0930537	0.3100171
SIRT5	0.1225678	0.1343643	0.1149580	0.1017394	0.1292334
ENSG00000261071	0.0078899	0.0246146	0.0161342	0.0051043	0.0186974
NOL7	1.0012945	0.9702725	0.9243898	0.9524147	0.7088925
RANBP9	0.4545390	0.4777261	0.5440526	0.4025380	0.5152685
ENSG00000286316	0.0015991	0.0000000	0.0014317	0.0000000	0.0000000
MCUR1	0.7888166	0.7362997	0.7126559	0.7165823	0.6278929
ENSG00000272209	0.0028999	0.0013262	0.0000000	0.0051652	0.0043425
RNF182	0.0593551	0.0910379	0.1440858	0.0668426	0.0665960
CD83	0.0402667	0.0770990	0.1255408	0.0650167	0.0909720
ENSG00000285639	0.0018273	0.0000000	0.0000000	0.0000000	0.0196442
ENSG00000234540	0.0000000	0.0000000	0.0000000	0.0000000	0.0022680
ENSG00000286277	0.0050253	0.0065545	0.0128985	0.0000000	0.0354298
ENSG00000234261	0.0003724	0.0027777	0.0034031	0.0019606	0.0098125
JARID2-DT	0.0082490	0.0174022	0.0139580	0.0126525	0.0055238

JARID2	0.2894818	0.4041649	0.4753021	0.3218986	0.7230261
JARID2-AS1	0.0045133	0.0020209	0.0010558	0.0029169	0.0000000
DTNBP1	0.3148892	0.3460221	0.3982655	0.3565531	0.4049766
ENSG00000287626	0.0000000	0.0000000	0.0000000	0.0000000	0.0020430
ENSG00000289953	0.0040879	0.0012430	0.0120540	0.0000000	0.0094241
LINC02543	0.0011066	0.0000000	0.0000000	0.0000000	0.0045117
MYLIP	0.0544307	0.0765797	0.0616482	0.0209619	0.0734388
GMPR	0.1194617	0.0891948	0.0394492	0.1231277	0.0910226
ENSG00000282024	0.0000000	0.0000000	0.0000000	0.0000000	0.0075438
ATXN1	0.7534781	0.6179288	0.4600197	0.5620829	1.0126824
ATXN1-AS1	0.1295463	0.0949650	0.0774329	0.1495049	0.0858888
ENSG00000272341	0.0321508	0.0320269	0.0220048	0.0326963	0.0093473
ENSG00000287347	0.0000000	0.0014383	0.0000000	0.0041277	0.0000000
ENSG00000287359	0.0002712	0.0000000	0.0000000	0.0000000	0.0000000
STMND1	0.0042584	0.0018829	0.0024703	0.0116419	0.0000000
ENSG00000287559	0.0025304	0.0018994	0.0051524	0.0000000	0.0029223
RBM24	0.3524570	0.2060175	0.1707911	0.2471331	0.1762833
CAP2	0.3971454	0.3363443	0.3580786	0.3584354	0.3802300
ENSG00000286885	0.0114038	0.0275132	0.0249194	0.0145211	0.0116059
FAM8A1	0.2961440	0.3048080	0.4227734	0.3097410	0.2799491
NUP153	0.3147215	0.3293354	0.2828363	0.2680634	0.4379558
NUP153-AS1	0.0016704	0.0000000	0.0022147	0.0113372	0.0116154
KIF13A	0.2814441	0.3379796	0.2738319	0.3159316	0.4600874
NHLRC1	0.0074081	0.0094882	0.0082567	0.0023202	0.0000000
TPMT	0.1451498	0.1505235	0.1489774	0.2030079	0.1774515
KDM1B	0.1807116	0.2390702	0.3058509	0.2156400	0.2409749
DEK	0.8719176	0.9020052	0.8895362	0.8673009	0.6473260
RNF144B	0.0583014	0.0357463	0.0199608	0.0778959	0.0336083
MIR548A1HG	0.0000000	0.0009268	0.0000000	0.0030630	0.0000000
ENSG00000231662	0.0112487	0.0033943	0.0084880	0.0101111	0.0197226
LNC-LBCS	0.1500238	0.1453877	0.1029078	0.1246832	0.3606349
ID4	1.4411576	1.7053788	1.3671648	1.7147676	1.2979324
MBOAT1	0.0009141	0.0042126	0.0000000	0.0000000	0.0057746
ENSG00000227803	0.0000000	0.0000000	0.0000000	0.0000000	0.0000000
ENSG00000289981	0.0036810	0.0018585	0.0010324	0.0000000	0.0000000
E2F3	0.1388617	0.1776675	0.1537105	0.1224506	0.2196571
E2F3-IT1	0.0000000	0.0000000	0.0072502	0.0038410	0.0000000
CDKAL1	0.2513943	0.2666801	0.2438906	0.2453911	0.6323235
ENSG00000233848	0.0026116	0.0015854	0.0000000	0.0000000	0.0122337
ENSG00000287404	0.0000000	0.0000000	0.0000000	0.0000000	0.0000000
LINC00581	0.0112796	0.0257550	0.0434126	0.0110860	0.0036522
ENSG00000231754	0.0160088	0.0459068	0.1022342	0.0279850	0.0616711
ENSG00000283480	0.0039292	0.0049617	0.0010104	0.0022023	0.0138989
SOX4	1.2875852	1.7545623	1.8880141	1.1660717	1.6389823
CASC15	0.1316049	0.2129981	0.1526394	0.0683929	0.2939120
NBAT1	0.0082374	0.0168260	0.0153411	0.0053074	0.0093856
ENSG00000233358	0.0024305	0.0000000	0.0000000	0.0000000	0.0093560
ENSG00000289368	0.0003104	0.0023823	0.0000000	0.0045301	0.0049709
NRSN1	0.1701698	0.2726089	0.4600141	0.1440161	0.2741174
DCDC2	0.8272860	0.4563706	0.2974648	1.0467990	0.4455775
KAAG1	0.0205705	0.0133679	0.0030892	0.0320212	0.0036522
MRS2	0.2567073	0.2069231	0.2659494	0.2806417	0.2233042
GPLD1	0.0688443	0.0922916	0.1061957	0.0803389	0.1391662

ALDH5A1	0.6854243	0.5508977	0.6062743	0.5881654	0.4812671
KIAA0319	0.1171822	0.1047742	0.0818149	0.2002642	0.2354700
TDP2	0.3421419	0.3266974	0.2602775	0.3669868	0.2739756
ACOT13	0.5181099	0.4101449	0.3793590	0.5075183	0.4096160
ENSG00000272345	0.0025910	0.0051463	0.0055560	0.0025961	0.0000000
C6orf62	0.8254499	0.8781657	0.8658354	0.7713903	0.7629357
ENSG00000272402	0.0011196	0.0028354	0.0000000	0.0000000	0.0042796
ENSG00000288851	0.0024932	0.0011596	0.0000000	0.0000000	0.0045817
LINC02828	0.0015417	0.0012507	0.0026275	0.0061016	0.0053380
GMNN	0.1613131	0.1184038	0.0804352	0.1282391	0.1183382
ARMH2	0.0000000	0.0000000	0.0000000	0.0000000	0.0020430
RIPOR2	0.0786684	0.1249322	0.1587829	0.0445890	0.2159805
ENSG00000285801	0.0012439	0.0000000	0.0000000	0.0000000	0.0000000
CARMIL1	0.1370667	0.1546373	0.1627641	0.1393494	0.5869694
SCGN	0.0374507	0.0188944	0.0052727	0.0029495	0.0081014
TRIM38	0.0013355	0.0005146	0.0000000	0.0062316	0.0000000
ENSG00000272558	0.0011291	0.0000000	0.0013931	0.0000000	0.0112543
LINC02980	0.0032732	0.0092786	0.0129089	0.0083810	0.0000000
H1-1	0.0246480	0.0433287	0.0526478	0.0253639	0.0210793
H3C1	0.0018258	0.0075405	0.0056690	0.0000000	0.0231714
H4C1	0.0026463	0.0013072	0.0019096	0.0000000	0.0052689
H4C2	0.0005644	0.0006563	0.0000000	0.0000000	0.0000000
H3C2	0.0034332	0.0090334	0.0008618	0.0040613	0.0000000
H2AC4	0.0024479	0.0021354	0.0041909	0.0000000	0.0052561
H1-2	0.3666486	0.2801336	0.2238412	0.3653034	0.2176220
H2BC4	0.0435843	0.0178093	0.0065776	0.0443052	0.0504643
HFE	0.0090783	0.0032181	0.0000000	0.0128737	0.0049754
H4C3	0.5234902	0.5461373	0.5673658	0.5077353	0.4098251
H2AC6	0.1788899	0.1343986	0.1316997	0.1628611	0.1046985
H1-4	0.3552882	0.3822859	0.4510779	0.2932168	0.3538385
ENSG00000289117	0.0022942	0.0000000	0.0000000	0.0043405	0.0035346
H2BC5	0.2284304	0.2492588	0.2662909	0.1270609	0.2086639
ENSG00000283064	0.0024653	0.0049277	0.0034914	0.0000000	0.0000000
H2BC6	0.1319151	0.1624794	0.1642524	0.0911538	0.1039288
H4C4	0.0149564	0.0099109	0.0152327	0.0012699	0.0033906
H3C4	0.0259591	0.0197615	0.0101844	0.0319423	0.0163008
H2AC7	0.0059920	0.0065137	0.0020433	0.0051350	0.0067905
H2BC7	0.0091593	0.0222042	0.0129995	0.0000000	0.0137890
H4C5	0.1459569	0.1526301	0.1443403	0.1446610	0.1345135
H2BC8	0.0007815	0.0064242	0.0024339	0.0000000	0.0000000
H2AC8	0.0567302	0.0640123	0.0845097	0.0356780	0.0508510
H3C6	0.0229759	0.0175522	0.0235537	0.0340436	0.0318119
H1-3	0.1079006	0.1008909	0.0570063	0.0598515	0.0736489
H3C7	0.0013805	0.0000000	0.0016907	0.0000000	0.0069764
H2BC9	0.0054027	0.0054281	0.0042275	0.0021141	0.0039666
H3C8	0.0000000	0.0000000	0.0028387	0.0000000	0.0073097
H4C8	0.0221334	0.0100223	0.0180250	0.0107094	0.0187159
ENSG00000289447	0.0012355	0.0000000	0.0010064	0.0000000	0.0000000
BTN3A2	0.0227890	0.0178606	0.0060935	0.0295300	0.0157256
BTN2A2	0.0620619	0.0265905	0.0156732	0.1357665	0.0399122
BTN3A1	0.0467107	0.0556300	0.0623694	0.0731838	0.0910297
BTN2A3P	0.0006514	0.0011287	0.0000000	0.0033745	0.0044904
BTN3A3	0.0089915	0.0048145	0.0082365	0.0305753	0.0000000

BTN2A1	0.2740614	0.2455912	0.2723469	0.3299269	0.2351170
HCG11	0.0913163	0.0695583	0.0563079	0.1008405	0.0802652
HMGN4	0.3541307	0.2921384	0.3276490	0.2826262	0.2434700
ABT1	0.1738425	0.1642047	0.1427266	0.1632395	0.1164433
ENSG00000275846	0.0000000	0.0014229	0.0000000	0.0000000	0.0000000
ZNF322	0.3605873	0.3104516	0.3083274	0.3729063	0.2788390
ENSG00000285571	0.0011118	0.0013756	0.0000000	0.0045301	0.0033094
ENSG00000261584	0.0000000	0.0000000	0.0019364	0.0000000	0.0044415
ENSG00000291342	0.0028885	0.0000000	0.0000000	0.0000000	0.0000000
GUSBP2	0.0364909	0.0400565	0.0330996	0.0412417	0.0847003
LINC00240	0.0247992	0.0389003	0.0153928	0.0529805	0.0543635
LARRPM	0.0442209	0.0530281	0.0422491	0.0549290	0.0817440
ENSG00000272312	0.0017165	0.0019333	0.0057192	0.0000000	0.0000000
H2BC11	0.0134595	0.0133580	0.0107333	0.0057806	0.0000000
H2AC11	0.0164475	0.0095637	0.0010884	0.0044729	0.0479239
ENSG00000290032	0.2333359	0.1358437	0.1393102	0.2281127	0.1629299
H2BC12	0.0034380	0.0030514	0.0028647	0.0027934	0.0064366
H2AC12	0.0128320	0.0124142	0.0084620	0.0000000	0.0287211
ENSG00000290009	0.0000000	0.0007100	0.0000000	0.0000000	0.0021475
PRSS16	0.0223753	0.0110390	0.0136724	0.0199329	0.0178128
ENSG00000291112	0.1543281	0.2159987	0.3503751	0.1533043	0.2775112
ZNF391	0.0542273	0.0722854	0.0765391	0.0664069	0.0742131
ENSG00000271755	0.0059026	0.0025015	0.0179202	0.0088741	0.0111247
ZNF184	0.0887155	0.0937443	0.0793472	0.1224401	0.0781788
ENSG00000285849	0.0000000	0.0000000	0.0000000	0.0000000	0.0000000
ENSG00000286652	0.0095348	0.0087890	0.0105227	0.0058441	0.0150034
ENSG00000285703	0.0007288	0.0000000	0.0000000	0.0000000	0.0000000
LINC01012	0.0308354	0.0478522	0.0492824	0.0087410	0.0545644
ENSG00000287252	0.0035812	0.0034732	0.0059822	0.0039240	0.0000000
H2BC13	0.0009026	0.0000000	0.0000000	0.0000000	0.0000000
H3C10	0.0072040	0.0105515	0.0089776	0.0095951	0.0122222
H2AC14	0.0014187	0.0009971	0.0013424	0.0000000	0.0000000
H4C11	0.0046422	0.0010863	0.0066232	0.0037097	0.0000000
H2AC15	0.0153622	0.0200607	0.0301862	0.0254626	0.0323739
H2BC15	0.0526440	0.0533102	0.0339886	0.0163738	0.0740934
H2AC16	0.0021781	0.0013702	0.0000000	0.0000000	0.0078594
H1-5	0.0178561	0.0214181	0.0177983	0.0108284	0.0085566
H3C11	0.0009417	0.0014503	0.0014559	0.0000000	0.0000000
H3C12	0.0091145	0.0092113	0.0079144	0.0030874	0.0400375
H2AC17	0.0127330	0.0105195	0.0144392	0.0076888	0.0131407
H2BC17	0.0012238	0.0000000	0.0000000	0.0000000	0.0000000
OR2B6	0.0021165	0.0005949	0.0000000	0.0000000	0.0036741
ZSCAN16-AS1	0.4468987	0.3807665	0.3421284	0.4268750	0.3170133
ENSG00000272009	0.0167780	0.0166220	0.0014933	0.0235834	0.0140061
ZNF165	0.0346067	0.0177625	0.0059360	0.0341985	0.0385163
ENSG00000291008	0.0000000	0.0000000	0.0026647	0.0052894	0.0000000
ZSCAN16	0.0341556	0.0362919	0.0451924	0.0206622	0.0366000
ENSG00000261839	0.0000000	0.0017829	0.0018284	0.0000000	0.0083649
ZKSCAN8	0.1223647	0.0955984	0.1060699	0.1132682	0.1972019
ZKSCAN8P1	0.0081091	0.0075466	0.0038449	0.0019173	0.0231552
ZSCAN9	0.1299677	0.1731896	0.2249093	0.1042216	0.1544001
ZKSCAN4	0.0498452	0.0550399	0.0398157	0.0436734	0.0700686
NKAPL	0.0084099	0.0078125	0.0082119	0.0097307	0.0071599

ZSCAN26	0.2027130	0.1859190	0.1817351	0.2322903	0.2050703
PGBD1	0.0887389	0.1246699	0.1701819	0.0713732	0.1926092
ZSCAN31	0.0497084	0.0214354	0.0228091	0.0563162	0.0110734
ENSG00000289467	0.0006437	0.0022260	0.0000000	0.0000000	0.0000000
ENSG00000290051	0.0035751	0.0049461	0.0154613	0.0042672	0.0256591
ZKSCAN3	0.0487626	0.0492683	0.0403348	0.0327600	0.0203944
ZSCAN12	0.1182513	0.1201548	0.1073616	0.1140766	0.1936528
ZSCAN23	0.0149684	0.0317193	0.0278178	0.0242165	0.0652960
ENSG00000287804	0.0248325	0.0123367	0.0019772	0.0214780	0.0052984
ENSG00000286819	0.0014777	0.0014125	0.0051725	0.0000000	0.0000000
SCAND3	0.1139101	0.1314495	0.2107527	0.1177065	0.0877077
ZBED9-AS1	0.0071288	0.0060174	0.0061884	0.0017461	0.0154634
ENSG00000287279	0.0009355	0.0021629	0.0065924	0.0026278	0.0173091
ENSG00000225173	0.0016193	0.0098071	0.0194479	0.0000000	0.0000000
LINC01623	0.0023018	0.0021302	0.0103291	0.0000000	0.0139282
HCG14	0.0047651	0.0060449	0.0087791	0.0055024	0.0069764
TRIM27	0.2431729	0.2250821	0.3018344	0.2041519	0.2168490
LINC01556	0.0022628	0.0010081	0.0000000	0.0019455	0.0000000
ZNF311	0.0590154	0.0515509	0.0453397	0.0607434	0.0393351
LINC03003	0.0056414	0.0010756	0.0029858	0.0212083	0.0168078
UBD	0.0056791	0.0016677	0.0000000	0.0000000	0.0000000
GABBR1	0.2602648	0.3432844	0.4540305	0.2794425	0.4645915
OR2H2	0.0000000	0.0000000	0.0000000	0.0000000	0.0094393
MOG	0.0000000	0.0000000	0.0000000	0.0000000	0.0027063
HLA-F	0.0035917	0.0018508	0.0000000	0.0064045	0.0035493
HLA-F-AS1	0.0094027	0.0130282	0.0100280	0.0011920	0.0045939
ENSG00000285761	0.0000000	0.0000000	0.0000000	0.0000000	0.0031804
HLA-V	0.0005162	0.0036774	0.0079554	0.0043864	0.0101938
HLA-G	0.0002690	0.0021840	0.0037291	0.0000000	0.0000000
ENSG00000290870	0.0035346	0.0019289	0.0046307	0.0000000	0.0031339
HLA-A	0.5875583	0.4109348	0.4221923	0.8793842	0.3428978
HCG9	0.0068328	0.0113004	0.0116828	0.0000000	0.0000000
POLR1H	0.2492561	0.2430591	0.2044891	0.2323128	0.1763720
PPP1R11	0.5781516	0.5829918	0.6065413	0.4556184	0.3829560
RNF39	0.0003299	0.0029654	0.0000000	0.0000000	0.0000000
TRIM31-AS1	0.0000000	0.0000000	0.0000000	0.0000000	0.0028743
TRIM26	0.1782628	0.1672598	0.1676644	0.1516597	0.1993255
HCG17	0.2945235	0.3484966	0.3856673	0.2155244	0.3914902
HLA-L	0.0126118	0.0406248	0.0610387	0.0548744	0.0710283
HCG18	0.2537294	0.3740828	0.3949528	0.2505928	0.3900426
TRIM39	0.0902108	0.0563293	0.0524924	0.1057933	0.0630053
RPP21	0.4853739	0.5327493	0.6244778	0.4501474	0.4682267
ENSG00000288805	0.0000000	0.0000000	0.0032766	0.0000000	0.0045646
HLA-E	0.7673815	0.4258757	0.2817167	1.1127324	0.3813748
GNL1	0.4088517	0.4163904	0.4112762	0.3766681	0.3953326
PRR3	0.1553629	0.1886255	0.2251431	0.1751378	0.2182363
ENSG00000290047	0.0046919	0.0010333	0.0000000	0.0000000	0.0049042
ABCF1	0.7054346	0.6301859	0.6087377	0.6694104	0.5054824
PPP1R10	0.3433527	0.2894411	0.3880051	0.3345919	0.5695696
MRPS18B	0.4865826	0.4731030	0.5544399	0.4421937	0.3245428
ATAT1	0.3909727	0.6382139	0.7667067	0.3940613	0.5153551
C6orf136	0.1208927	0.1557904	0.1570843	0.1383009	0.0801233
DHX16	0.1490258	0.1401230	0.1379574	0.1631222	0.1596501

PPP1R18	0.1131211	0.1829277	0.1916053	0.1307762	0.1286239
NRM	0.0456118	0.0675625	0.0633104	0.0591003	0.0367334
MDC1	0.0771885	0.0791365	0.0863508	0.0763826	0.1258205
MDC1-AS1	0.0000000	0.0000000	0.0000000	0.0000000	0.0000000
TUBB	2.3076038	2.6755287	2.9717631	2.3247605	2.3000779
ENSG00000272540	0.0378624	0.0501102	0.1036032	0.0373785	0.0822206
FLOT1	0.5869965	0.6479169	0.6322546	0.5467717	0.4823717
IER3-AS1	0.0618605	0.0510855	0.0500431	0.0385224	0.0454287
IER3	0.0406702	0.0463691	0.0186897	0.0463521	0.0212184
HCG20	0.0000000	0.0000000	0.0053156	0.0000000	0.0032805
LINC00243	0.0000000	0.0000000	0.0000000	0.0000000	0.0000000
DDR1-DT	0.0000000	0.0014650	0.0000000	0.0000000	0.0000000
DDR1	0.6207320	0.4076851	0.3958165	0.9544208	0.4257186
GTF2H4	0.0942833	0.0847109	0.0721023	0.1174605	0.0412856
VARS2	0.0341290	0.0278819	0.0104637	0.0283909	0.0747987
SFTA2	0.0035671	0.0053141	0.0050226	0.0032941	0.0000000
MUCL3	0.0000000	0.0000000	0.0000000	0.0000000	0.0090317
HCG22	0.0159893	0.0029022	0.0090001	0.0329814	0.0000000
PSORS1C1	0.0036635	0.0000000	0.0000000	0.0000000	0.0000000
CCHCR1	0.0552887	0.0472732	0.0309552	0.0599702	0.0318832
TCF19	0.0070861	0.0101289	0.0119274	0.0073374	0.0000000
POU5F1	0.0031073	0.0022286	0.0000000	0.0000000	0.0028694
ENSG00000272501	0.0445568	0.0591761	0.1264283	0.0232160	0.0352207
HCG27	0.0025467	0.0033336	0.0037788	0.0049186	0.0042436
HLA-C	0.4901100	0.3741233	0.3398724	0.7592117	0.2878828
HLA-B	0.1397945	0.1031821	0.0911117	0.2402625	0.0863642
ENSG00000285647	0.0000000	0.0006253	0.0000000	0.0000000	0.0030832
MICA	0.0741383	0.0455109	0.0222904	0.0792259	0.0607324
HCP5	0.0000000	0.0032620	0.0008086	0.0084175	0.0000000
MICB-DT	0.0000000	0.0000000	0.0000000	0.0000000	0.0000000
MICB	0.0026171	0.0000000	0.0000000	0.0000000	0.0000000
DDX39B	0.9855704	0.8821936	0.8810453	1.0406028	0.7722475
DDX39B-AS1	0.0049337	0.0018122	0.0024542	0.0000000	0.0028666
ATP6V1G2	0.5363215	0.9022407	1.0743431	0.5003842	0.5285134
NFKBIL1	0.2529660	0.2535732	0.1891343	0.1951668	0.2050396
ENSG00000289406	0.0000000	0.0005609	0.0000000	0.0013130	0.0000000
LTA	0.0007616	0.0023417	0.0016528	0.0000000	0.0056738
TNF	0.0000000	0.0000000	0.0000000	0.0000000	0.0000000
LTB	0.0030525	0.0030274	0.0044119	0.0000000	0.0092018
LST1	0.0006658	0.0063654	0.0011917	0.0000000	0.0000000
AIF1	0.0019652	0.0011874	0.0047738	0.0000000	0.0000000
ENSG00000289375	0.0000000	0.0028523	0.0000000	0.0000000	0.0000000
PRRC2A	0.3006251	0.3234692	0.3355244	0.2604290	0.3590486
BAG6	0.3177971	0.3807964	0.4002109	0.3116160	0.2854589
APOM	0.0510615	0.0351260	0.0206608	0.0532972	0.0188839
C6orf47	0.1015786	0.1064412	0.0823713	0.1278704	0.0708016
C6orf47-AS1	0.0014557	0.0000000	0.0029736	0.0000000	0.0000000
GPANK1	0.2275371	0.1919150	0.1361260	0.1730916	0.1383396
CSNK2B	1.1830118	1.2194716	1.1335862	1.1233844	0.9503841
LY6G5B	0.0132232	0.0202594	0.0090994	0.0034082	0.0208470
LY6G5C	0.0168180	0.0217081	0.0100559	0.0509143	0.0037728
ABHD16A	0.2662087	0.2139483	0.2018219	0.2467663	0.2135935
MPIG6B	0.0014679	0.0012840	0.0000000	0.0000000	0.0120987

LY6G6C	0.0000000	0.0000000	0.0000000	0.0000000	0.0000000
DDAH2	1.1034112	1.1648291	1.0749552	1.1393748	0.9115896
CLIC1	1.0226553	0.6675562	0.4327092	1.0274572	0.5729640
MSH5	0.0102728	0.0091969	0.0042012	0.0034144	0.0261612
SAPCD1	0.0341012	0.0167934	0.0116690	0.0359971	0.0164787
SAPCD1-AS1	0.0065395	0.0086827	0.0070904	0.0086696	0.0086699
VWA7	0.0008567	0.0013457	0.0000000	0.0000000	0.0074687
VARS1	0.1007299	0.1047840	0.0772619	0.0720380	0.1481615
LSM2	0.4789047	0.4427432	0.4543261	0.4517354	0.3326148
HSPA1L	0.0332236	0.0383499	0.0356832	0.0408744	0.0315438
HSPA1A	0.4717508	0.4342669	0.5073018	0.3852460	0.3724809
HSPA1B	0.5335776	0.4310346	0.4427396	0.5268773	0.3818148
SNHG32	1.5402672	1.4533872	1.3727144	1.4378199	1.2709084
NEU1	0.2189082	0.1517776	0.1177037	0.2889174	0.1865139
SLC44A4	0.0005818	0.0000000	0.0000000	0.0000000	0.0039795
EHMT2-AS1	0.0000000	0.0013944	0.0000000	0.0000000	0.0000000
EHMT2	0.3210013	0.2957867	0.3017965	0.3509715	0.3419027
C2	0.0077786	0.0164514	0.0150077	0.0070965	0.0557934
ZBTB12	0.0337969	0.0310592	0.0526471	0.0222836	0.0247245
C2-AS1	0.0017930	0.0016179	0.0029046	0.0000000	0.0065528
CFB	0.0000000	0.0012840	0.0000000	0.0000000	0.0000000
NELFE	0.4729034	0.3826164	0.3384622	0.4866340	0.2936077
SKIC2	0.0548735	0.0300509	0.0436674	0.0538598	0.0267166
DXO	0.0590314	0.0453374	0.0397434	0.0753045	0.0302168
STK19	0.3284123	0.2898138	0.2916623	0.3816313	0.2332323
C4B	0.0000000	0.0000000	0.0000000	0.0036094	0.0000000
CYP21A2	0.0010714	0.0074258	0.0064527	0.0000000	0.0039740
TNXB	0.0529030	0.0371003	0.0238007	0.0754890	0.1228602
ENSG00000286974	0.0000000	0.0000000	0.0000000	0.0000000	0.0000000
ATF6B	0.2301193	0.1680653	0.1609352	0.2931647	0.1951753
FKBPL	0.0606403	0.0315223	0.0179297	0.0425703	0.0236196
PRRT1	0.0162119	0.0137638	0.0364607	0.0200972	0.0163888
ENSG00000284954	0.0947179	0.0671644	0.0495831	0.0613611	0.0594076
PPT2	0.0443428	0.0303927	0.0362257	0.0554572	0.0327902
EGFL8	0.0083094	0.0039910	0.0200980	0.0171865	0.0296465
AGPAT1	0.6392338	0.6874473	0.7208900	0.5697595	0.4709752
RNF5	0.7783987	0.7988175	0.9405879	0.6739742	0.5915639
AGER	0.0022684	0.0050482	0.0038651	0.0047565	0.0335768
ENSG00000273333	0.0000000	0.0015017	0.0000000	0.0000000	0.0072786
PBX2	0.3044371	0.3022671	0.3595055	0.2687818	0.2301788
GPSM3	0.0062970	0.0112490	0.0012948	0.0044812	0.0117225
NOTCH4	0.0523370	0.0604411	0.0545464	0.0648783	0.0223880
TSBP1-AS1	0.0205192	0.0656546	0.0607311	0.0395088	0.1599769
TSBP1	0.0000000	0.0000000	0.0000000	0.0000000	0.0125561
BTNL2	0.0000000	0.0000000	0.0000000	0.0000000	0.0000000
HLA-DRA	0.0000000	0.0000000	0.0000000	0.0000000	0.0080666
HLA-DRB5	0.0120448	0.0211710	0.0378444	0.0094971	0.0133209
HLA-DRB6	0.0000000	0.0075424	0.0057174	0.0000000	0.0000000
HLA-DRB1	0.0465739	0.0969539	0.1829431	0.0429936	0.0184733
HLA-DQB1	0.0042327	0.0064305	0.0106289	0.0000000	0.0049524
HLA-DQB2	0.0000000	0.0000000	0.0000000	0.0000000	0.0000000
HLA-DOB	0.0000000	0.0000000	0.0000000	0.0000000	0.0000000
TAP2	0.0292083	0.0360372	0.0304891	0.0194397	0.0241895

PSMB8	0.0414455	0.0145989	0.0046019	0.0326988	0.0178583
PSMB8-AS1	0.0021794	0.0000000	0.0012127	0.0068814	0.0000000
PSMB9	0.0041340	0.0024384	0.0038208	0.0123608	0.0096409
TAP1	0.0368798	0.0190070	0.0138494	0.0659353	0.0127375
ENSG00000289559	0.0073247	0.0104459	0.0168604	0.0011730	0.0108769
ENSG00000289047	0.1986083	0.3254564	0.3876807	0.1463084	0.1806567
HLA-DMB	0.0065791	0.0079636	0.0006307	0.0052846	0.0119145
HLA-DMA	0.0901552	0.0683702	0.0502847	0.0636615	0.0193438
BRD2	0.5942016	0.6293490	0.5562442	0.5602863	0.6101649
HLA-DOA	0.0004809	0.0011915	0.0000000	0.0000000	0.0000000
HLA-DPA1	0.0101927	0.0048465	0.0166341	0.0019173	0.0000000
HLA-DPB1	0.0228645	0.0450271	0.0577671	0.0236317	0.0347873
ENSG00000291111	0.0438921	0.0849773	0.1456272	0.0214913	0.0956961
HCG24	0.0071052	0.0052106	0.0068993	0.0033686	0.0000000
COL11A2	0.0086996	0.0089704	0.0116065	0.0103773	0.0456999
RXRB	0.1474863	0.0972888	0.1113926	0.1445076	0.1221771
SLC39A7	0.5753077	0.4292968	0.4918204	0.7711957	0.4388044
HSD17B8	0.3170428	0.1707252	0.0789418	0.3182140	0.1395221
ENSG00000288751	0.0000000	0.0000000	0.0000000	0.0000000	0.0000000
RING1	0.3120299	0.2318229	0.1948962	0.2951486	0.2299462
ENSG00000272217	0.0008886	0.0000000	0.0000000	0.0000000	0.0000000
HCG25	0.0034206	0.0035111	0.0060466	0.0065832	0.0060850
VPS52	0.1300768	0.1016042	0.1031491	0.1494132	0.0812178
RPS18	2.7830244	2.8198474	2.6345036	2.6973031	2.5112185
B3GALT4	0.0865125	0.0565037	0.0435365	0.0925142	0.0639101
WDR46	0.1373046	0.1212352	0.1306228	0.1658643	0.0766584
PFDN6	0.4076785	0.4377354	0.5020110	0.4033369	0.3126027
RGL2	0.2789036	0.1830116	0.1678953	0.2440729	0.1642239
ENSG00000289100	0.2006323	0.1071745	0.0928349	0.2392566	0.1360260
TAPBP	0.2695078	0.1642885	0.1383079	0.4290785	0.1457642
ZBTB22	0.1462058	0.1603687	0.1611541	0.1392401	0.1199703
DAXX	0.1590205	0.1812120	0.2246646	0.1465421	0.1310379
KIFC1	0.0126678	0.0086000	0.0081836	0.0183907	0.0000000
PHF1	0.2639016	0.3160207	0.3238268	0.2700151	0.2479667
CUTA	0.9810282	0.9567184	1.0041470	1.0659693	0.7509368
SYNGAP1	0.1326011	0.1827755	0.1699179	0.1425869	0.2494759
SYNGAP1-AS1	0.0079966	0.0058672	0.0009848	0.0094415	0.0197948
ZBTB9	0.0328001	0.0227151	0.0249461	0.0441451	0.0266391
BAK1	0.0352409	0.0232386	0.0366733	0.0346956	0.0419428
ITPR3	0.0012989	0.0022677	0.0000000	0.0000000	0.0087871
UQCC2	0.7927783	0.7548565	0.8939029	0.7393066	0.6251190
IP6K3	0.0270881	0.0143457	0.0034830	0.0218679	0.0159645
LEMD2	0.2360705	0.2042614	0.1700625	0.2762301	0.2083687
MLN	0.0000000	0.0000000	0.0012348	0.0019626	0.0000000
GRM4	0.0212598	0.0311832	0.0580379	0.0159577	0.0924848
ENSG00000288879	0.0000000	0.0000000	0.0000000	0.0000000	0.0000000
HMGA1	0.5492434	0.8115899	0.8862877	0.5170086	0.5588620
SMIM29	0.4617806	0.5889793	0.6242719	0.4798718	0.4828243
ENSG00000225339	0.1313589	0.2228886	0.2880571	0.1257821	0.2811004
NUDT3	1.0907149	1.3942801	1.5778132	1.0366904	1.3588327
RPS10	2.8604299	2.8306137	2.6319234	2.8174257	2.6327505
PACSIN1	0.0442949	0.0502328	0.1017064	0.0354249	0.0673899
SPDEF	0.0086545	0.0075880	0.0019052	0.0000000	0.0121579

ILRUN	0.2680361	0.3236858	0.3016426	0.2846517	0.3615549
ILRUN-AS1	0.1323711	0.0927907	0.0508765	0.1091387	0.0887911
SNRPC	0.8162442	0.7311930	0.8165968	0.7982028	0.6133445
BLTP3A	0.1548118	0.1698370	0.1350597	0.1349267	0.2199039
TAF11	0.4218423	0.4659130	0.5072433	0.4347457	0.4222634
ANKS1A	0.3190163	0.4476906	0.3433836	0.2859752	0.6316497
ENSG00000286550	0.0004822	0.0000000	0.0000000	0.0000000	0.0014498
TCP11	0.0055259	0.0066793	0.0000000	0.0023239	0.0016673
SCUBE3	0.0236397	0.0441506	0.0631473	0.0399390	0.0721968
SCUBE3-AS1	0.0003789	0.0031487	0.0000000	0.0000000	0.0113418
ZNF76	0.0627114	0.0641473	0.0366063	0.0905465	0.0943463
DEF6	0.0037439	0.0052046	0.0104498	0.0000000	0.0000000
PPARD	0.1195871	0.0811722	0.0561944	0.1004277	0.0760713
FANCE	0.0316967	0.0378838	0.0632410	0.0353421	0.0242548
RPL10A	2.2741148	2.2612479	2.1207707	2.1326791	2.0605818
TEAD3	0.0356203	0.0193398	0.0257098	0.0091487	0.0113908
ENSG00000289456	0.0000000	0.0000000	0.0000000	0.0000000	0.0000000
FKBP5	0.0271715	0.0387250	0.0412412	0.0177734	0.0879067
ENSG00000232909	0.0000000	0.0000000	0.0000000	0.0000000	0.0000000
ARMC12	0.0044505	0.0057514	0.0139470	0.0037228	0.0089386
CLPSL1	0.0000000	0.0000000	0.0000000	0.0000000	0.0039758
LHFPL5	0.0255230	0.0566450	0.0602532	0.0235433	0.0933191
SRPK1	0.3569183	0.5154785	0.6275030	0.2795137	0.5318952
SLC26A8	0.0081646	0.0104070	0.0071512	0.0010246	0.0205685
MAPK14	0.1989355	0.1824683	0.1407937	0.2279555	0.3752999
MAPK13	0.0082924	0.0110684	0.0127757	0.0043545	0.0306220
BRPF3-AS1	0.0206218	0.0194589	0.0228078	0.0123169	0.0227909
BRPF3	0.0823192	0.0975084	0.1497953	0.0886874	0.1519060
PNPLA1	0.0020537	0.0035016	0.0042196	0.0024514	0.0046972
BNIP5	0.0000000	0.0000000	0.0000000	0.0000000	0.0000000
ETV7	0.0010727	0.0009685	0.0000000	0.0016011	0.0000000
PXT1	0.0014994	0.0015056	0.0032055	0.0000000	0.0092211
KCTD20	0.4517683	0.4585570	0.4922234	0.4453921	0.4375514
STK38	0.2018563	0.1721200	0.1365816	0.1216663	0.4004394
SRSF3	0.8568189	0.7869637	0.7633787	0.8597373	0.7353731
CDKN1A	0.9085121	0.5275548	0.3026350	0.9596155	0.4583641
DINOL	0.0000000	0.0000000	0.0000000	0.0000000	0.0000000
RAB44	0.0019782	0.0000000	0.0034708	0.0000000	0.0000000
CPNE5	0.0870591	0.1274671	0.1163491	0.1024497	0.1330252
ENSG00000285888	0.0000000	0.0000000	0.0000000	0.0000000	0.0000000
ENSG00000287891	0.0014243	0.0011956	0.0000000	0.0000000	0.0012770
PPIL1	0.2995040	0.3035526	0.3587648	0.3767209	0.2643162
C6orf89	0.4473821	0.3273202	0.2813569	0.6136386	0.2962144
PI16	0.3447323	0.1443780	0.1078966	1.0093371	0.1868107
MTCH1	1.0482965	1.0027028	1.0460467	1.0326961	0.8048978
PIM1	0.0610926	0.0880660	0.0923905	0.0587383	0.0503940
TMEM217	0.0217915	0.0123895	0.0070788	0.0491534	0.0125710
TBC1D22B	0.1046750	0.1373143	0.1222598	0.1138856	0.1381408
ENSG00000286672	0.0000000	0.0000000	0.0000000	0.0000000	0.0000000
RNF8	0.3689501	0.3923133	0.3941308	0.3557157	0.3900388
CMTR1	0.1200724	0.1181651	0.1178600	0.1422030	0.1115497
CCDC167	0.5792510	0.6236663	0.6534163	0.5679606	0.4875485
LINC02520	0.0023064	0.0071162	0.0071598	0.0000000	0.0000000

MDGA1	0.0185924	0.0184966	0.0258539	0.0253356	0.0680693
ZFAND3-DT	0.0063074	0.0073794	0.0008837	0.0123230	0.0000000
ZFAND3	0.8692041	0.7566608	0.6415574	0.8945098	1.2244653
BTBD9	0.2325977	0.2743991	0.2540248	0.1925973	0.8373911
GLO1	1.2532273	1.1363122	1.0024697	1.2731119	0.7962241
DNAH8	0.0007454	0.0031667	0.0031481	0.0049723	0.0048432
GLP1R	0.0032540	0.0027358	0.0000000	0.0049554	0.0085822
SAYSD1	0.1527112	0.1162862	0.1252935	0.1820995	0.1281424
KCNK5	0.0000000	0.0014749	0.0000000	0.0000000	0.0000000
KCNK17	0.0011179	0.0030144	0.0044980	0.0013531	0.0000000
KIF6	0.0489166	0.0449602	0.0265771	0.0360874	0.0996616
DAAM2	0.0427508	0.0265094	0.0057035	0.0362466	0.0627972
DAAM2-AS1	0.0014386	0.0009308	0.0000000	0.0000000	0.0036428
MOCS1	0.0179266	0.0112909	0.0101539	0.0118468	0.0206871
TDRG1	0.0023810	0.0000000	0.0000000	0.0000000	0.0036851
LINC00951	0.0108449	0.0141513	0.0080302	0.0022544	0.0285452
LRFN2	0.0242879	0.0397052	0.0298213	0.0130658	0.2646105
ENSG00000236075	0.0000000	0.0000000	0.0000000	0.0000000	0.0000000
ENSG00000226454	0.0000000	0.0000000	0.0010030	0.0045021	0.0033946
UNC5CL	0.0050592	0.0047316	0.0000000	0.0010246	0.0000000
OARD1	0.5444453	0.4329337	0.3664137	0.4513731	0.2927356
APOBEC2	0.0011525	0.0011092	0.0000000	0.0000000	0.0000000
NFYA	0.1374387	0.1077922	0.1165443	0.0869863	0.0867201
ADCY10P1	0.0267246	0.0256549	0.0115610	0.0057107	0.0719749
ENSG00000290034	0.0000000	0.0000000	0.0000000	0.0000000	0.0000000
TREM1	0.0004399	0.0017971	0.0000000	0.0000000	0.0000000
FOXP4-AS1	0.0141652	0.0119147	0.0000000	0.0236386	0.0096467
LINC01276	0.0026834	0.0008572	0.0028883	0.0012986	0.0000000
FOXP4	0.0881174	0.0709351	0.0475526	0.0461715	0.0649801
MDFI	0.1143067	0.0590125	0.0243281	0.1545041	0.0496783
TFEB	0.0181924	0.0155422	0.0012235	0.0088364	0.0090730
PGC	0.0008930	0.0000000	0.0031821	0.0033686	0.0000000
ENSG00000269387	0.0000000	0.0000000	0.0000000	0.0000000	0.0000000
FRS3	0.1437817	0.2381679	0.2826588	0.1090933	0.1727881
PRICKLE4	0.0013015	0.0017117	0.0013551	0.0000000	0.0062037
TOMM6	0.9639699	0.8370718	0.8378542	0.9465728	0.6997117
USP49	0.0834295	0.1124473	0.1567908	0.0647562	0.2186328
ENSG00000227516	0.0000000	0.0000000	0.0000000	0.0000000	0.0000000
MED20	0.0732020	0.0312639	0.0332512	0.0760469	0.0481873
BYSL	0.0545029	0.0564388	0.0546412	0.0637310	0.0740105
CCND3	0.0600059	0.0958453	0.0805999	0.0320082	0.0547805
TAF8	0.0500746	0.0351588	0.0381671	0.0580530	0.0670001
C6orf132	0.0051341	0.0034332	0.0079045	0.0000000	0.0037366
GUCA1A	0.0315415	0.0523622	0.0399543	0.0406002	0.0514251
GUCA1B	0.0099912	0.0090122	0.0133077	0.0086105	0.0411270
ENSG00000289216	0.0141459	0.0227359	0.0373098	0.0140499	0.0293662
MRPS10	0.3190815	0.3363601	0.4216694	0.2969267	0.2539143
TRERF1	0.0571918	0.0720949	0.0578143	0.0657036	0.2734558
UBR2	0.3012981	0.2930420	0.2464839	0.2896975	0.6122478
PRPH2	0.0208845	0.0255402	0.0416118	0.0071899	0.0520861
TBCC	0.1642811	0.1717111	0.1928669	0.1972566	0.1030760
BICRAL	0.1357312	0.1268541	0.1564531	0.1219847	0.2709441
RPL7L1	0.8201530	0.7740893	0.7266310	0.8642309	0.6800378

C6orf226	0.2398010	0.2269158	0.2567612	0.2120260	0.1694859
ENSG00000287825	0.0743607	0.0583110	0.0502604	0.0695401	0.0569217
CNPY3	0.3850650	0.3120675	0.2779052	0.3904146	0.2845091
LINC02976	0.0541055	0.0470692	0.0534050	0.0610623	0.0576351
GNMT	0.0003191	0.0011581	0.0010324	0.0046313	0.0077248
PEX6	0.1166951	0.0922121	0.1228355	0.1562951	0.1007708
ENSG00000290049	0.0117516	0.0117520	0.0117757	0.0163827	0.0138886
PPP2R5D	0.1424198	0.1790475	0.2011819	0.1190687	0.1534933
MEA1	0.6547141	0.5906099	0.6855982	0.6584196	0.5240289
KLHDC3	0.3737274	0.4493420	0.4680244	0.4191491	0.2948279
RRP36	0.3371847	0.3182727	0.3511735	0.3642923	0.2899672
ENSG00000272223	0.0008587	0.0023900	0.0015058	0.0098622	0.0023981
CUL7	0.1151769	0.0624397	0.0831149	0.0864214	0.0995927
KLC4	0.1183951	0.1008367	0.0955639	0.0616921	0.1720160
MRPL2	0.3311117	0.2823574	0.2799014	0.2902328	0.2449601
KLC4-AS1	0.0078310	0.0056998	0.0000000	0.0036726	0.0066722
PTK7	0.1315423	0.0946802	0.0835064	0.2215983	0.1974011
SRF	0.0658583	0.0782127	0.0535125	0.0723308	0.0554371
CUL9	0.0661260	0.0702292	0.0463387	0.0677123	0.1359886
ENSG00000245261	0.0000000	0.0000000	0.0000000	0.0015108	0.0000000
DNPH1	0.7016150	0.5171045	0.4644190	0.8019896	0.3203758
TTBK1	0.0544880	0.1093475	0.0991458	0.0538269	0.1544116
SLC22A7	0.0000000	0.0000000	0.0000000	0.0000000	0.0000000
CRIP3	0.0039122	0.0051488	0.0190566	0.0023342	0.0063697
ZNF318	0.1984839	0.1772402	0.2377302	0.1879716	0.2185883
ENSG00000287055	0.0014775	0.0025716	0.0008634	0.0000000	0.0099146
ABCC10	0.0474802	0.0586092	0.0526466	0.0565629	0.0952494
DLK2	0.0285439	0.0750327	0.1188491	0.0341742	0.0769946
TJAP1	0.0590074	0.0298728	0.0145934	0.0583611	0.0471765
LRRC73	0.1498648	0.1484957	0.2220975	0.0866037	0.1121687
POLR1C	0.1562733	0.1215327	0.0838589	0.1638420	0.1022191
YIPF3	0.5511712	0.4747351	0.5150637	0.6589900	0.3240164
ENSG00000271754	0.0009837	0.0022189	0.0000000	0.0000000	0.0000000
XPO5	0.1395794	0.1579885	0.1803612	0.1627463	0.2401852
POLH	0.1155422	0.0976279	0.0657754	0.1097659	0.1140345
POLH-AS1	0.0021986	0.0059948	0.0029356	0.0000000	0.0000000
GTPBP2	0.1102157	0.1268723	0.0665815	0.0828103	0.0823018
MAD2L1BP	0.1617521	0.1724209	0.1751799	0.1737832	0.0649749
RSPH9	0.5305732	0.4506228	0.5175773	0.4832884	0.4626437
MRPS18A	0.2252867	0.1575815	0.1458546	0.1865009	0.0682067
VEGFA	0.8580486	0.7517893	0.7189333	0.7984252	0.8485586
ENSG00000272114	0.0126299	0.0067947	0.0155516	0.0049433	0.0106942
ENSG00000283573	0.0013952	0.0008560	0.0000000	0.0000000	0.0000000
LINC01512	0.0126428	0.0159468	0.0376407	0.0098714	0.0169727
SCIRT	0.0502073	0.0593246	0.0863421	0.0521209	0.0575406
ENSG00000289609	0.0070118	0.0081053	0.0000000	0.0056441	0.0024001
ENSG00000231881	0.0007915	0.0000000	0.0044858	0.0000000	0.0000000
ENSG00000287562	0.0260313	0.0283904	0.0312855	0.0130533	0.0264628
MRPL14	0.3654474	0.3767456	0.4602836	0.3617398	0.2229308
TMEM63B	0.2182440	0.2933750	0.3049468	0.1929435	0.2742113
CAPN11	0.0018283	0.0000000	0.0000000	0.0000000	0.0149040
MYMX	0.0117377	0.0011573	0.0057078	0.0015624	0.0050487
SLC29A1	0.6236574	0.3984110	0.3318255	0.4654689	0.3311535

HSP90AB1	3.3495514	3.2619798	3.4127252	3.2583085	2.9339518
SLC35B2	0.1279114	0.0747485	0.0709684	0.1811106	0.1302222
NFKBIE	0.0354115	0.0403135	0.0698435	0.0288816	0.0186310
TMEM151B	0.1303686	0.2401180	0.2596402	0.1091716	0.1878070
TCTE1	0.0150900	0.0139781	0.0202127	0.0133949	0.0132414
AARS2	0.0622910	0.0611451	0.0535886	0.0610168	0.0482302
SPATS1	0.0017346	0.0106098	0.0009487	0.0115252	0.0090104
CDC5L	0.7152480	0.6366587	0.6669717	0.7913452	0.5735440
ENSG00000286417	0.0018176	0.0027686	0.0026345	0.0167900	0.0203531
SUPT3H	0.4649165	0.4948663	0.4287058	0.4256278	1.0186208
RUNX2	0.0156698	0.0095169	0.0150060	0.0153665	0.0457426
ENSG00000271857	0.0014644	0.0000000	0.0000000	0.0112674	0.0000000
CLIC5	0.0055275	0.0303519	0.0661682	0.0087301	0.0552067
ENSG00000231769	0.0606749	0.0731597	0.0719756	0.0245775	0.0838433
ENPP4	0.2823232	0.2230290	0.2528505	0.3617602	0.2470235
ENPP5	0.0742577	0.0525917	0.0390103	0.1609409	0.0962317
RCAN2	0.3445662	0.7250125	0.5629977	0.2581553	0.5577550
RCAN2-DT	0.0026021	0.0061292	0.0025766	0.0017550	0.0015171
CYP39A1	0.1223739	0.0851447	0.0642074	0.0934231	0.0660807
SLC25A27	0.1617743	0.2943787	0.3431419	0.1174890	0.2690114
TDRD6-AS1	0.0000000	0.0011997	0.0000000	0.0000000	0.0000000
TDRD6	0.0033446	0.0000000	0.0057535	0.0000000	0.0088300
PLA2G7	0.0039675	0.0214413	0.0156103	0.0162788	0.0166235
ANKRD66	0.0067150	0.0038392	0.0000000	0.0246405	0.0000000
TNFRSF21	0.1184894	0.2028813	0.2780643	0.1611256	0.2271036
CD2AP-DT	0.0093337	0.0072052	0.0062025	0.0111857	0.0107507
CD2AP	0.3183039	0.1899739	0.1499768	0.3139436	0.3781152
OPN5	0.0000000	0.0000000	0.0000000	0.0000000	0.0155487
PTCHD4	0.1043898	0.1980958	0.1344357	0.0954338	0.4344453
MMUT	0.2948836	0.2782953	0.2740849	0.3329438	0.2705112
CENPQ	0.2476971	0.1792030	0.1167504	0.2964859	0.1143120
GLYATL3	0.0000000	0.0009093	0.0000000	0.0000000	0.0087450
C6orf141	0.0010800	0.0000000	0.0000000	0.0000000	0.0000000
TFAP2B	0.0012165	0.0000000	0.0000000	0.0000000	0.0000000
PKHD1	0.0010898	0.0013783	0.0008981	0.0000000	0.0064222
MCM3	0.0951006	0.0468407	0.0592503	0.1016862	0.0815397
PAQR8	0.1172859	0.0940994	0.0809012	0.1008415	0.0885602
EFHC1	0.2929946	0.2247695	0.1623796	0.3034373	0.2983602
ENSG00000289276	0.0000000	0.0000000	0.0019603	0.0000000	0.0077584
TRAM2	0.2088027	0.1358532	0.0564445	0.2413429	0.1323157
TRAM2-AS1	0.1576143	0.1439266	0.1445590	0.1476739	0.0847498
ENSG00000291006	0.1254015	0.1008453	0.1448775	0.1279303	0.0729354
TMEM14A	0.7045606	0.8896891	1.0850870	0.6920784	0.6586758
ENSG00000291036	0.0000000	0.0000000	0.0000000	0.0000000	0.0071630
GSTA4	0.6160643	0.7128345	0.8365604	0.6600457	0.5762800
CILK1	0.1562950	0.1960642	0.2039063	0.0951575	0.1398122
FBXO9	0.4431916	0.5761434	0.5785571	0.4448792	0.4445993
ELOVL5	0.3663823	0.3444221	0.3621179	0.5533582	0.4227827
GCLC	0.2285725	0.1783168	0.1077780	0.2784693	0.2142387
GCLC-AS1	0.0099414	0.0096764	0.0035971	0.0302614	0.0041350
LINC01564	0.0000000	0.0043530	0.0021106	0.0028151	0.0000000
KLHL31	0.0008997	0.0022480	0.0039875	0.0000000	0.0000000
ENSG00000227885	0.0101379	0.0092186	0.0072948	0.0016217	0.0128675

LRRC1	0.0845616	0.0740268	0.0539498	0.0358107	0.1629572
MLIP	0.0118611	0.0069235	0.0086387	0.0247741	0.0284520
HCRTR2	0.0222206	0.0337534	0.0651513	0.0035717	0.0929156
GFRAL	0.0000000	0.0009512	0.0000000	0.0000000	0.0000000
HMGCLL1	0.3957799	0.4627552	0.4252194	0.4258159	0.5257414
BMP5	0.0018123	0.0023968	0.0006621	0.0030204	0.0114189
COL21A1	0.1068133	0.0409936	0.0555161	0.3308505	0.1037029
DST	1.6675843	1.6820965	1.5416281	1.5065743	2.1542106
DST-AS1	0.0107091	0.0194736	0.0206177	0.0102219	0.0138671
BEND6	0.5836840	0.7534782	0.7904657	0.4790494	0.4909961
KIAA1586	0.4632427	0.4836103	0.5308473	0.4987387	0.4500471
ZNF451	0.4585423	0.4648108	0.4959376	0.4055390	0.4920057
ZNF451-AS1	0.0036757	0.0103179	0.0068770	0.0046461	0.0620688
BAG2	0.1756316	0.1435102	0.0869310	0.2119041	0.0738526
RAB23	0.3137284	0.2250838	0.2168237	0.3529916	0.1732935
PRIM2	0.0769473	0.0453990	0.0187837	0.0896589	0.1598821
ENSG00000272541	0.0042013	0.0067140	0.0092805	0.0000000	0.0000000
ENSG00000286723	0.0025097	0.0020668	0.0000000	0.0000000	0.0289996
ENSG00000271761	0.0017863	0.0000000	0.0000000	0.0000000	0.0047680
ENSG00000272316	0.0428167	0.0443393	0.0481548	0.0405198	0.0728010
LINC00680	0.0200899	0.0381841	0.0307168	0.0317705	0.0285987
ENSG00000225096	0.0018294	0.0043332	0.0017796	0.0000000	0.0058005
ENSG00000290597	0.0005241	0.0013562	0.0000000	0.0000000	0.0000000
ENSG00000287598	0.0019860	0.0000000	0.0000000	0.0000000	0.0000000
KHDRBS2-OT1	0.0176864	0.0155214	0.0103291	0.0000000	0.0719697
KHDRBS2	0.1834106	0.2748958	0.3076301	0.1231489	0.8028326
ENSG00000287679	0.0000000	0.0014712	0.0000000	0.0000000	0.0000000
FKBP1C	0.0003446	0.0015255	0.0000000	0.0000000	0.0000000
ENSG00000289911	0.0017324	0.0046018	0.0000000	0.0000000	0.0000000
PTP4A1	0.0000000	0.0012321	0.0000000	0.0000000	0.0159204
ENSG00000266680	0.0025611	0.0003461	0.0006373	0.0000000	0.0109789
PHF3	1.0387450	0.9273592	0.9748980	1.0043024	0.9512207
EYS	0.0086466	0.0052599	0.0095853	0.0083955	0.0443030
ZC3H11C	0.0129836	0.0130536	0.0097672	0.0063284	0.0088324
ENSG00000288088	0.0005766	0.0028485	0.0000000	0.0000000	0.0112104
ADGRB3-DT	0.1342875	0.1869342	0.1833204	0.0996322	0.2056793
ADGRB3	0.6456623	0.7581329	0.6245524	0.6302422	1.8470610
ENSG00000289611	0.0136260	0.0120181	0.0062832	0.0030695	0.0716146
LMBRD1	0.5586658	0.4769065	0.4438789	0.7014735	0.6019037
COL19A1	0.0171750	0.0125034	0.0220553	0.0071597	0.0380038
COL9A1	0.0069508	0.0040294	0.0073082	0.0051901	0.0031305
FAM135A-AS1	0.0052679	0.0038587	0.0000000	0.0064246	0.0000000
FAM135A	0.3170016	0.2711972	0.2695027	0.2874421	0.4058083
SDHAF4	0.3259870	0.3575140	0.4301346	0.3013902	0.3032221
ENSG00000271967	0.0011066	0.0055047	0.0000000	0.0000000	0.0000000
SMAP1	1.0490286	1.2628800	1.4082227	0.9960019	1.1612525
B3GAT2	0.2441877	0.4465217	0.6047861	0.2495526	0.4135792
ENSG00000287939	0.0183512	0.0035845	0.0000000	0.0327149	0.0349299
LINC00472	0.0509037	0.0218886	0.0190540	0.0471973	0.0569145
ENSG00000287380	0.0036241	0.0046095	0.0010545	0.0071607	0.0000000
OGFRL1	0.4157978	0.4039255	0.4011140	0.5813477	0.3270478
RIMS1	0.4400394	0.7014488	0.7215225	0.3241120	1.3612986
KCNQ5-DT	0.0010285	0.0011997	0.0000000	0.0012911	0.0000000

KCNQ5	0.0599911	0.0660877	0.0301043	0.0263229	0.3123535
KHDC1L	0.0008850	0.0023224	0.0009712	0.0048458	0.0000000
KHDC1	0.1346868	0.1176407	0.1181137	0.1498242	0.1538727
KHDC1-AS1	0.1508391	0.1596998	0.1360782	0.1359707	0.1742795
KHDC3L	0.0084992	0.0175432	0.0213476	0.0107701	0.0000000
OOEP	0.1307883	0.1274201	0.1191233	0.1087124	0.1242115
OOEP-AS1	0.0000000	0.0000000	0.0000000	0.0000000	0.0000000
DDX43	0.0000000	0.0000000	0.0000000	0.0000000	0.0000000
CGAS	0.0044789	0.0034915	0.0019301	0.0000000	0.0065448
MTO1	0.0807282	0.0648173	0.0811227	0.0396575	0.0985700
EEF1A1	4.7120697	4.5562920	4.2320799	4.5734954	4.3096322
EEF1A1-AS1	0.0034672	0.0009363	0.0000000	0.0000000	0.0000000
SLC17A5	0.0913942	0.1087408	0.0861507	0.1139979	0.1168902
CD109-AS1	0.0206982	0.0133949	0.0027353	0.0239399	0.0000000
CD109	0.3197974	0.1766728	0.1212144	0.6138377	0.1698757
ENSG00000223786	0.0511774	0.0418702	0.0322807	0.0306376	0.0343468
ENSG00000272243	0.0000000	0.0038770	0.0000000	0.0000000	0.0000000
COL12A1	0.0125123	0.0146965	0.0128114	0.0390935	0.0746536
COX7A2	1.7726647	1.9704276	2.0767296	1.7109311	1.5413737
TMEM30A	0.9665758	0.7739925	0.7885527	1.1677024	0.8093434
TMEM30A-DT	0.0407194	0.0250361	0.0313368	0.0261193	0.0246437
FILIP1	0.0990840	0.1396490	0.0244459	0.1125649	0.1227293
SENP6	0.9325020	0.8645644	0.8191586	0.8381935	0.9230142
MYO6	0.3955975	0.3486188	0.2706926	0.3863733	0.6725702
IMPG1	0.0010865	0.0025246	0.0000000	0.0000000	0.0034730
LINC02540	0.0009289	0.0079433	0.0000000	0.0000000	0.0109120
ENSG00000271945	0.0010157	0.0006315	0.0027084	0.0000000	0.0175101
HTR1B	0.0037086	0.0237553	0.0284648	0.0138051	0.0104428
MEI4	0.0044686	0.0065512	0.0000000	0.0010526	0.0108905
IRAK1BP1	0.2943905	0.2543641	0.2088565	0.2647096	0.3256764
PHIP	0.6756270	0.6604750	0.7384186	0.6157023	1.0187014
ENSG00000286340	0.0162690	0.0265194	0.0213847	0.0163590	0.0492658
HMGN3	1.1189863	0.9007882	0.8176801	1.1283182	0.9145370
HMGN3-AS1	0.0171273	0.0324106	0.0135531	0.0100492	0.0379050
ENSG00000231533	0.0367705	0.0211387	0.0127716	0.0384979	0.0449811
LCA5	0.4398965	0.3000031	0.2660050	0.4783249	0.3191091
ENSG00000287811	0.0316806	0.0168810	0.0256703	0.0359874	0.0692163
ENSG00000272137	0.0036086	0.0017289	0.0000000	0.0000000	0.0022158
SH3BGRL2	0.2450631	0.2744898	0.2834473	0.1730484	0.2434151
ELOVL4	0.1993706	0.3597093	0.5717353	0.1882952	0.3888161
ENSG00000287816	0.0000000	0.0081604	0.0034244	0.0019984	0.0151696
TTK	0.0075143	0.0131355	0.0080329	0.0111125	0.0091890
ENSG00000288071	0.0008189	0.0055250	0.0017533	0.0048478	0.0000000
BCKDHB	0.1502277	0.1148934	0.1206270	0.1365956	0.3142353
ENSG00000272129	0.0033400	0.0000000	0.0029755	0.0000000	0.0021288
ENSG00000233967	0.0039532	0.0094230	0.0063821	0.0038589	0.0273226
ENSG00000260645	0.0000000	0.0021122	0.0009456	0.0066911	0.0000000
TENT5A	0.1221549	0.0884761	0.0731938	0.1071159	0.1308961
LINC02542	0.0067434	0.0034340	0.0114826	0.0000000	0.0137043
IBTK	0.4287715	0.3731897	0.4307879	0.3869944	0.3770130
TPBG	0.1075485	0.0551687	0.0820082	0.1974573	0.0759989
UBE3D	0.1210433	0.1328396	0.0904933	0.0722725	0.3434619
ENSG00000227215	0.0000000	0.0000000	0.0000000	0.0000000	0.0000000

DOP1A	0.2575276	0.2849483	0.2267352	0.2163623	0.6038333
PGM3	0.5932696	0.3745618	0.3162506	0.5671892	0.4486207
RWDD2A	0.1575330	0.1253090	0.1389735	0.1385052	0.1362558
ME1	0.5444948	0.3982701	0.3484223	0.5845300	0.3138082
PRSS35	0.0287827	0.0217304	0.0051745	0.0656593	0.0182690
SNAP91	0.5040531	0.7290586	0.7949560	0.3500147	1.0035168
ENSG00000287705	0.0056070	0.0161265	0.0116448	0.0017571	0.0064756
RIPPLY2	0.0353310	0.0943717	0.1338619	0.0295254	0.0637714
CYB5R4	0.0749954	0.1318502	0.1077711	0.0499108	0.1954382
ENSG00000228679	0.0011474	0.0007218	0.0000000	0.0019606	0.0033772
LINC02857	0.0003347	0.0036772	0.0061404	0.0074386	0.0043425
MRAP2	0.1183182	0.0676297	0.0574496	0.1104481	0.0916534
CEP162	0.1896276	0.1319079	0.1130933	0.2485760	0.1594222
LINC01611	0.0003230	0.0019886	0.0019963	0.0000000	0.0000000
TBX18	0.0059541	0.0032080	0.0055322	0.0000000	0.0071991
NT5E	0.0096975	0.0218484	0.0106602	0.0151171	0.0155224
SNX14	0.3976385	0.4004706	0.3738450	0.4484944	0.6234706
SYNCRIP	0.8584503	0.8258219	0.8649966	0.8775735	0.7860863
SNHG5	1.0800012	0.9582608	0.8359289	1.0015386	0.7625613
ENSG00000288021	0.0016778	0.0031745	0.0029356	0.0028371	0.0157930
HTR1E	0.0015838	0.0116293	0.0109917	0.0000000	0.0405027
ENSG00000272008	0.0124238	0.0155071	0.0158296	0.0107450	0.0300915
ZNF292	0.8033998	0.8724010	0.9379138	0.7200675	1.1686978
GJB7	0.0054651	0.0075913	0.0023644	0.0162714	0.0147353
SMIM8	0.2863628	0.3871261	0.3903042	0.2349259	0.4022337
C6orf163	0.0049223	0.0106401	0.0038056	0.0000000	0.0277057
CFAP206	0.3620002	0.2206126	0.1189705	0.3911242	0.2037471
SLC35A1	0.2147505	0.2082438	0.2326632	0.2285906	0.2858928
RARS2	0.5510602	0.6110571	0.5618050	0.5149308	0.6251831
ORC3	0.4913645	0.4321471	0.4138825	0.4779556	0.4337637
AKIRIN2	0.7958791	0.7845997	0.8455118	0.7231054	0.6343331
CNR1	0.2054810	0.2956140	0.5516521	0.1675654	0.3457974
ENSG00000234426	0.0000000	0.0000000	0.0000000	0.0000000	0.0000000
RNGTT	0.3490499	0.3021625	0.3517867	0.2414846	0.6540915
PNRC1-DT	0.0020712	0.0000000	0.0028078	0.0018154	0.0000000
PNRC1	1.8484186	1.4655775	1.1856319	1.7368217	1.3016187
SRSF12	0.2578970	0.3105925	0.3925411	0.2603417	0.3211244
ENSG00000288009	0.0102311	0.0169480	0.0062095	0.0030842	0.0243773
PM20D2	0.1704197	0.1248663	0.1018720	0.1923345	0.1184319
GABRR1	0.0044432	0.0055501	0.0014097	0.0000000	0.0042775
GABRR2	0.0020425	0.0026553	0.0056655	0.0026231	0.0155848
UBE2J1	0.5465644	0.5714151	0.6009576	0.4611606	0.4783651
RRAGD	1.3306494	0.9071810	0.6410610	1.4768542	0.8075384
ANKRD6	0.3351006	0.5221190	0.3380335	0.3632723	0.5414145
ENSG00000237027	0.0041379	0.0014195	0.0000000	0.0000000	0.0000000
LYRM2	0.8184761	0.6674960	0.6097443	0.7757269	0.6209482
MDN1	0.1712034	0.1791370	0.1631538	0.1232704	0.3502039
MDN1-AS1	0.0013925	0.0024556	0.0066994	0.0055383	0.0000000
CASP8AP2	0.3117800	0.3844616	0.3902525	0.2722174	0.4106209
GJA10	0.0000000	0.0000000	0.0000000	0.0000000	0.0000000
BACH2	0.3491298	0.6320355	0.4309165	0.3598320	1.1925276
ENSG00000287789	0.0000000	0.0000000	0.0000000	0.0000000	0.0000000
ENSG00000260271	0.0084795	0.0046515	0.0015034	0.0074556	0.0226816

MAP3K7	0.3555210	0.3006888	0.3320160	0.3007619	0.3971175
CASC6	0.0006606	0.0022980	0.0000000	0.0037810	0.0000000
ENSG00000285961	0.0125057	0.0000000	0.0095167	0.0060590	0.0000000
EPHA7	0.2059261	0.2646918	0.2921688	0.1652128	0.8686306
ENSG00000287683	0.0184689	0.0123547	0.0164452	0.0242586	0.0902343
ENSG00000288085	0.0017953	0.0000000	0.0000000	0.0037420	0.0207668
MANEA-DT	0.0008135	0.0025433	0.0049784	0.0000000	0.0181112
MANEA	0.2465608	0.1481676	0.1120794	0.2968368	0.1204707
ENSG00000287578	0.0043454	0.0056221	0.0051371	0.0036477	0.0159763
FUT9	0.2538542	0.3736937	0.6306496	0.2365179	0.7474446
UFL1-AS1	0.0162469	0.0192953	0.0365839	0.0199226	0.0729750
UFL1	0.6043778	0.4182573	0.3529304	0.6183022	0.3758005
ENSG00000224384	0.0000000	0.0014613	0.0000000	0.0000000	0.0084213
GPR63	0.0244411	0.0234283	0.0368032	0.0098832	0.0322236
NDUFAF4	0.4106816	0.5634710	0.6534207	0.3433245	0.3543042
KLHL32	0.1059444	0.1203613	0.1535706	0.0975384	0.3487151
MMS22L	0.0357978	0.0370354	0.0130620	0.0602665	0.0861872
ENSG00000271860	0.0634841	0.1498760	0.0994761	0.0911804	0.5248278
ENSG00000283010	0.0016629	0.0000000	0.0000000	0.0030358	0.0127192
POU3F2	0.2117287	0.2468321	0.3302538	0.2991413	0.1849814
FBXL4	0.1897473	0.1862190	0.1771966	0.1664131	0.3482687
FAXC	0.1634991	0.3442312	0.3996221	0.1240836	0.3309197
COQ3	0.0673226	0.0568197	0.0505665	0.0504237	0.0477198
PNISR	0.9301591	0.8086706	0.8541389	0.7968719	0.9519398
PNISR-AS1	0.0778724	0.0446401	0.0400976	0.0266822	0.0495074
USP45	0.1364953	0.1184254	0.1455207	0.0955498	0.1777046
TSTD3	0.3143202	0.2605532	0.2694608	0.2977021	0.2523958
CCNC	0.2825862	0.2629862	0.2602622	0.1960640	0.2402362
SIM1	0.0053721	0.0032687	0.0051970	0.0120631	0.0046589
ASCC3	0.3805372	0.3621135	0.3107408	0.4408871	0.6022510
ENSG00000260000	0.0217920	0.0478134	0.0465900	0.0427400	0.0417167
GRIK2	0.4222297	0.5409326	0.4785172	0.3177708	1.6100098
ENSG00000287616	0.0000000	0.0006472	0.0000000	0.0000000	0.0000000
HACE1	0.1363600	0.1322555	0.1148752	0.1245222	0.2880070
LIN28B-AS1	0.0199112	0.0245626	0.0220561	0.0122495	0.0439280
LIN28B	0.0877920	0.1446661	0.1533624	0.0705811	0.3938933
ENSG00000290011	0.0042025	0.0109229	0.0136414	0.0033429	0.0204892
BVES	0.0179668	0.0114148	0.0103076	0.0196169	0.0176048
BVES-AS1	0.0055336	0.0078307	0.0111900	0.0039977	0.0037333
POPDC3	0.0202132	0.0260372	0.0690711	0.0266573	0.0236775
PREP	0.1508417	0.2172444	0.2324016	0.1796287	0.2696148
ENSG00000286084	0.0100412	0.0307736	0.0332940	0.0109358	0.0421854
ENSG00000284999	0.0007994	0.0000000	0.0014457	0.0063274	0.0000000
PRDM1	0.0059243	0.0000000	0.0000000	0.0040275	0.0044554
ATG5	0.3643508	0.3602958	0.3605386	0.3557054	0.3674329
CRYBG1	0.0000000	0.0111776	0.0023036	0.0000000	0.0153063
RTN4IP1	0.0391978	0.0570985	0.0560294	0.0582582	0.0997135
QRSL1	0.1593222	0.1365977	0.1282639	0.2273730	0.1423754
LINC02526	0.0023287	0.0014253	0.0000000	0.0017625	0.0000000
ENSG00000289020	0.0000000	0.0019683	0.0000000	0.0000000	0.0000000
LINC02532	0.0000000	0.0000000	0.0000000	0.0000000	0.0000000
CD24	1.1787223	1.6449451	2.0090004	1.2231780	1.3413309
MTRES1	0.3812810	0.2957507	0.2840964	0.4286274	0.2502950

BEND3	0.0336185	0.0127257	0.0287102	0.0240865	0.0494847
PDSS2	0.1372137	0.1426107	0.1050808	0.1747564	0.2567979
SOBP	0.5813156	0.7580163	0.8763446	0.5244979	1.1054977
ENSG00000234206	0.0000000	0.0021893	0.0000000	0.0000000	0.0000000
ENSG00000289433	0.0034477	0.0014808	0.0029216	0.0000000	0.0037236
SCML4	0.0280071	0.0325826	0.0357365	0.0090064	0.1815755
SEC63	0.8567250	0.6888196	0.6356803	0.9523169	0.7607346
ENSG00000272476	0.0018236	0.0035630	0.0024128	0.0000000	0.0105915
OSTM1	0.2879374	0.3379818	0.3483518	0.3638979	0.2303944
OSTM1-AS1	0.0000000	0.0000000	0.0000000	0.0000000	0.0238927
SNX3	1.4584969	1.2911775	1.2114271	1.5487687	1.0622129
ENSG00000286562	0.0012467	0.0013888	0.0012851	0.0045802	0.0000000
AFG1L	0.0981948	0.0919095	0.0872267	0.0643276	0.1763400
ENSG00000287044	0.0044375	0.0046970	0.0041536	0.0060372	0.0212720
FOXO3	0.4247920	0.4154053	0.3070631	0.3814693	0.5508613
LINC00222	0.0015146	0.0000000	0.0000000	0.0000000	0.0000000
ENSG00000286511	0.0091676	0.0065307	0.0008163	0.0082477	0.0098563
ARMC2	0.2987639	0.2624336	0.2235060	0.2320834	0.3852157
ARMC2-AS1	0.0000000	0.0000000	0.0000000	0.0000000	0.0081952
SESN1	0.3079333	0.2971926	0.2591807	0.3181460	0.5808360
ENSG00000271730	0.0028699	0.0000000	0.0000000	0.0057363	0.0000000
CEP57L1	0.3768927	0.4401290	0.4519711	0.3384660	0.3989069
CD164	0.8017010	0.5530509	0.3933108	0.9590320	0.4681457
ENSG00000260273	0.0079567	0.0000000	0.0000000	0.0014762	0.0054884
PPIL6	0.7320153	0.5625661	0.4611403	0.7385160	0.5097514
SMPD2	0.0547882	0.0253660	0.0429794	0.0582481	0.0326048
MICAL1	0.1057582	0.1000678	0.0935969	0.0902547	0.2430715
ZBTB24	0.0860489	0.1100852	0.1420137	0.0863523	0.1416974
ZBTB24-DT	0.0020186	0.0111852	0.0082873	0.0108972	0.0040206
AK9	0.2191238	0.2220711	0.2347560	0.1735092	0.2230362
FIG4	0.2428322	0.2136448	0.2559004	0.2292294	0.2666593
GPR6	0.0009479	0.0072004	0.0028372	0.0000000	0.0000000
ENSG00000286691	0.0000000	0.0000000	0.0022302	0.0000000	0.0000000
WASF1	0.5012152	0.7846011	1.0207233	0.4233951	0.6590692
CDC40	0.4102865	0.3818909	0.3390043	0.3525938	0.3163941
METTL24	0.0147914	0.0179536	0.0072015	0.0241872	0.0326468
ENSG00000287268	0.0000000	0.0000000	0.0000000	0.0000000	0.0000000
DDO	0.0047126	0.0005988	0.0000000	0.0107195	0.0000000
ENSG00000289847	0.0000000	0.0000000	0.0000000	0.0000000	0.0043293
CDK19	0.2828262	0.3338810	0.2592070	0.2354162	0.4347041
AMD1	0.4369011	0.4993979	0.5533609	0.3932019	0.5355271
GTF3C6	0.7049474	0.6628830	0.6381760	0.6985287	0.4471918
RPF2	0.4815913	0.4563533	0.3712730	0.4344343	0.3997398
SLC16A10	0.0250092	0.0314984	0.0486203	0.0286666	0.1879650
MFSD4B-DT	0.0013417	0.0012140	0.0000000	0.0000000	0.0000000
MFSD4B	0.0947074	0.1086403	0.0885952	0.0905928	0.2777506
ENSG00000271789	0.0086695	0.0068166	0.0011764	0.0032157	0.0082568
REV3L	0.7767484	0.9965617	1.1778016	0.7223465	1.2273798
ENSG00000272356	0.0048439	0.0108370	0.0100598	0.0043934	0.0137141
REV3L-IT1	0.0000000	0.0000000	0.0000000	0.0000000	0.0031628
TRAF3IP2-AS1	0.3649506	0.3515775	0.3779108	0.3389240	0.4679509
ENSG00000286616	0.0000000	0.0000000	0.0000000	0.0000000	0.0028743
TRAF3IP2	0.0954191	0.0643315	0.0332134	0.1501399	0.0668428

ENSG00000255389	0.0357634	0.0374045	0.0153156	0.0251677	0.0285935
FYN	0.9086399	1.0306182	1.1770586	0.9507805	1.1461614
ENSG00000289256	0.0076251	0.0034789	0.0035494	0.0123780	0.0324069
LINC02527	0.0000000	0.0000000	0.0000000	0.0000000	0.0000000
TUBE1	0.1762090	0.1498199	0.1268449	0.1640655	0.2063958
FAM229B	0.9025128	0.8573235	0.8660776	0.9430245	0.6842574
LAMA4	0.0215387	0.0269836	0.0086818	0.0867248	0.0255553
ENSG00000237234	0.0005993	0.0014359	0.0066761	0.0013687	0.0000000
LAMA4-AS1	0.0000000	0.0000000	0.0000000	0.0000000	0.0044857
ENSG00000288916	0.0007156	0.0007444	0.0000000	0.0000000	0.0000000
LINC02541	0.0052015	0.0023864	0.0049667	0.0000000	0.0121103
ENSG00000289198	0.0355875	0.0239071	0.0166805	0.0568346	0.0145287
MARCKS	2.3896868	2.2236779	2.4727133	2.5971595	2.0729880
LINC02880	0.0000000	0.0013734	0.0000000	0.0000000	0.0000000
HDAC2	1.1678507	1.3255790	1.5356415	1.1075127	1.0525578
HDAC2-AS2	0.0908403	0.1266548	0.0655169	0.0611327	0.2555590
HS3ST5	0.2695800	0.3863325	0.3193959	0.1761630	0.7249981
LNCPOIR	0.0006918	0.0000000	0.0000000	0.0000000	0.0000000
ENSG00000287097	0.0081944	0.0121016	0.0061530	0.0093609	0.0176198
ENSG00000289376	0.0013681	0.0043897	0.0012498	0.0000000	0.0048202
FRK	0.0046205	0.0023588	0.0027610	0.0000000	0.0000000
NT5DC1	0.4060194	0.2616013	0.2154342	0.4033053	0.2880249
COL10A1	0.0000000	0.0000000	0.0028387	0.0000000	0.0109254
TSPYL4	0.5695868	0.7679648	1.0295258	0.4884921	0.6234874
DSE	0.1098622	0.1002008	0.0714197	0.1409218	0.1974995
TSPYL1	0.4156022	0.5452202	0.5523386	0.3925794	0.3952582
CALHM6	0.0367795	0.0935833	0.1325482	0.0346105	0.0206752
TRAPPC3L	0.0010731	0.0033168	0.0029252	0.0000000	0.0080936
ENSG00000289304	0.0029386	0.0023351	0.0040608	0.0051646	0.0042903
RWDD1	1.0455029	1.0603979	1.0116977	0.9158588	0.8652494
RSPH4A	0.2776574	0.1499080	0.0969965	0.3304889	0.1300285
ZUP1	0.1983682	0.2189091	0.2477948	0.2108460	0.1996299
KPNA5	0.4099465	0.4466488	0.4847822	0.3562980	0.5297681
FAM162B	0.0009455	0.0009596	0.0022800	0.0000000	0.0000000
VGLL2	0.0005219	0.0000000	0.0000000	0.0000000	0.0046262
DCBLD1	0.0880284	0.0640913	0.0736811	0.0764130	0.0602394
GOPC	0.7141379	0.6664006	0.6888575	0.6199076	0.6095186
ENSG00000289372	0.0015751	0.0009813	0.0000000	0.0000000	0.0088722
NUS1	0.3170613	0.2757842	0.2927241	0.3188003	0.2986830
SLC35F1	0.4770644	0.6221801	0.5738280	0.4602460	1.0675096
CEP85L	0.3171119	0.2973444	0.2839106	0.2313458	0.5081740
PLN	0.0000000	0.0015188	0.0000000	0.0000000	0.0063420
ENSG00000286339	0.0123834	0.0053303	0.0055451	0.0027524	0.0105234
MCM9	0.0531512	0.0394316	0.0207119	0.0530517	0.1225601
ASF1A	0.3681713	0.2880117	0.2482038	0.4094061	0.2342094
ENSG00000253194	0.0104797	0.0060610	0.0052522	0.0140200	0.0313789
FAM184A	0.5029754	0.5168212	0.5980219	0.5464206	0.5918689
MAN1A1	0.1006081	0.1137517	0.0569453	0.1976082	0.3300539
ENSG00000287100	0.0227745	0.0330293	0.0157108	0.0315076	0.0847752
ENSG00000286540	0.0000000	0.0000000	0.0000000	0.0054802	0.0000000
TBC1D32	0.1501092	0.1369279	0.0942393	0.1310105	0.4092050
GJA1	0.2897505	0.1405913	0.0813773	0.6759679	0.2279879
HSF2	0.4198637	0.3976423	0.4580826	0.3452244	0.3711376

SERINC1	1.2767627	1.2698775	1.5082041	1.4048518	1.2605738
PKIB	0.5492068	0.7011990	0.6821161	0.4440433	0.5244801
ENSG00000287258	0.0000000	0.0000000	0.0000000	0.0000000	0.0000000
ENSG00000272472	0.0008343	0.0000000	0.0000000	0.0000000	0.0000000
FABP7	0.5873612	0.6009485	0.6281662	1.2168187	0.5697629
SMPDL3A	0.0803078	0.0731005	0.0526295	0.1003948	0.0826143
ENSG00000285652	0.0149795	0.0341608	0.0638710	0.0116452	0.0309233
CLVS2	0.1605238	0.3299990	0.4227271	0.1432877	0.4524820
TRDN	0.0043325	0.0044158	0.0000000	0.0000000	0.0000000
TRDN-AS1	0.0000000	0.0000000	0.0000000	0.0000000	0.0000000
NKAIN2	0.2217232	0.3594307	0.3024078	0.2072018	1.2456643
ENSG00000237321	0.0000000	0.0000000	0.0000000	0.0000000	0.0000000
RNF217-AS1	0.0219774	0.0307239	0.0342243	0.0056368	0.0503157
RNF217	0.1893830	0.2557310	0.2400163	0.1390211	0.2969812
TPD52L1	0.0705684	0.1354675	0.2313605	0.0729823	0.1110708
HDDC2	0.6354784	0.7168956	0.7460203	0.6311231	0.6313324
ENSG00000226409	0.0000000	0.0000000	0.0000000	0.0000000	0.0000000
HEY2-AS1	0.0011341	0.0042162	0.0000000	0.0023928	0.0000000
HEY2	0.0365688	0.0086185	0.0123019	0.0595486	0.0275211
NCOA7	0.4397923	0.7482097	0.7941790	0.3828625	0.5601377
NCOA7-AS1	0.0000000	0.0000000	0.0000000	0.0000000	0.0000000
HINT3	0.5419285	0.5541239	0.5650942	0.5116510	0.3511009
TRMT11	0.4866406	0.3601661	0.2958546	0.4731346	0.5023418
CENPW	0.1873689	0.1708666	0.1302353	0.1606410	0.1058975
ENSG00000287731	0.0011170	0.0010352	0.0000000	0.0000000	0.0000000
RSPO3	0.4412151	0.2593850	0.1212402	0.2876468	0.2128699
RNF146	0.5834946	0.5228388	0.4964386	0.5755774	0.4772433
ECHDC1	0.5204663	0.5510693	0.5168765	0.4768421	0.4069094
KIAA0408	0.4264351	0.8099777	0.9203650	0.3593132	0.7624727
SOGA3	0.5466459	0.9651924	1.2373667	0.5306141	0.9056806
C6orf58	0.0148498	0.0125124	0.0048016	0.0044172	0.0357320
THEMIS	0.0010635	0.0000000	0.0000000	0.0000000	0.0015201
PTPRK	0.2064192	0.2033369	0.1684568	0.1850584	0.5249334
PTPRK-AS1	0.0000000	0.0000000	0.0000000	0.0000000	0.0059220
ENSG00000289190	0.0103620	0.0092021	0.0018352	0.0030277	0.0043646
LAMA2	0.0104983	0.0157348	0.0130294	0.0062273	0.0283786
ENSG00000226149	0.0017394	0.0065915	0.0011596	0.0000000	0.0175005
ARHGAP18	0.0616650	0.0382522	0.0523899	0.0796282	0.0645257
L3MBTL3	0.1424551	0.1356900	0.1538004	0.1613565	0.0925129
ENSG00000227678	0.0007719	0.0000000	0.0012400	0.0000000	0.0000000
SAMD3	0.0499780	0.0616654	0.0599233	0.0254646	0.2275133
TMEM200A	0.1820070	0.1612124	0.1481442	0.1599941	0.1576113
EPB41L2	0.6925414	0.4741246	0.3494680	0.7025704	0.6060045
AKAP7	0.2987726	0.2558944	0.3498741	0.2509235	0.3002083
ENSG00000290067	0.0010263	0.0000000	0.0008339	0.0000000	0.0038629
ARG1	0.0000000	0.0019739	0.0026175	0.0000000	0.0060224
MED23	0.1360478	0.1208294	0.1246625	0.1407849	0.2176080
ENPP3	0.0014176	0.0040516	0.0040849	0.0017263	0.0177995
ENPP1	0.0438518	0.0287973	0.0270888	0.0645089	0.0570617
LINC01013	0.1641949	0.1421520	0.1040847	0.1710723	0.2535842
CCN2	0.8142880	0.5043457	0.3578272	1.7446119	0.6573288
MOXD1	0.0481505	0.0435838	0.0595504	0.0540796	0.0411741
STX7	0.7738202	0.9070682	0.9924628	0.7453043	0.7608160

SLC18B1	0.0657368	0.0584949	0.0785345	0.0934435	0.0477653
RPS12	3.3600409	3.3208086	3.0816238	3.2842666	3.0660354
LINC00326	0.0147690	0.0018369	0.0030185	0.0069806	0.0000000
EYA4	0.0521121	0.0228568	0.0227924	0.0760767	0.0330992
TARID	0.0006539	0.0033623	0.0027227	0.0000000	0.0000000
TBPL1	0.3756217	0.4377873	0.5733138	0.3100489	0.3202199
SLC2A12	0.0517725	0.0149804	0.0228022	0.0985933	0.0353739
ENSG00000287413	0.0006604	0.0000000	0.0000000	0.0069472	0.0000000
SGK1	0.0577867	0.0553498	0.0856917	0.0365562	0.2059367
ENSG00000286887	0.0000000	0.0000000	0.0000000	0.0038461	0.0000000
LINC01010	0.0013608	0.0000000	0.0000000	0.0041842	0.0000000
CT69	0.0023138	0.0000000	0.0000000	0.0000000	0.0000000
LINC03002	0.0008007	0.0000000	0.0000000	0.0000000	0.0000000
ALDH8A1	0.0057115	0.0062548	0.0119229	0.0043313	0.0351652
HBS1L	0.4584053	0.4157687	0.4877392	0.3736770	0.4392840
ENSG00000232876	0.0068086	0.0051631	0.0059948	0.0122057	0.0057461
MYB	0.0022716	0.0000000	0.0000000	0.0056005	0.0028632
AHI1	0.7216816	0.8039770	0.8059000	0.6922078	1.3759385
ENSG00000234084	0.0011196	0.0000000	0.0000000	0.0000000	0.0000000
ENSG00000287094	0.0000000	0.0000000	0.0000000	0.0000000	0.0000000
AHI1-DT	0.1103213	0.0748975	0.0368960	0.0687635	0.0992188
PDE7B	0.0252319	0.0164097	0.0080007	0.0227232	0.0725581
ENSG00000286313	0.0000000	0.0000000	0.0000000	0.0000000	0.0000000
PDE7B-AS1	0.0018181	0.0042580	0.0000000	0.0098444	0.0085583
MTFR2	0.0006803	0.0022757	0.0034097	0.0104956	0.0170133
BCLAF1	1.0035108	0.8861718	0.9527682	0.9889179	0.8626371
ENSG00000289312	0.0285581	0.0120302	0.0083663	0.0691130	0.0555140
ENSG00000260418	0.0094425	0.0044044	0.0017765	0.0039931	0.0000000
MAP7	1.0146743	0.7139392	0.4888014	0.9385228	0.9674159
MAP7-AS1	0.0197338	0.0047351	0.0026175	0.0041128	0.0000000
MAP3K5	0.0118380	0.0273597	0.0278836	0.0323427	0.0635246
MAP3K5-AS1	0.0011007	0.0006888	0.0000000	0.0000000	0.0000000
PEX7	0.0640213	0.0644525	0.0433096	0.0669598	0.0768473
IL20RA	0.0121008	0.0045394	0.0000000	0.0074450	0.0032853
IFNGR1	0.2948773	0.2280276	0.2296075	0.4582870	0.2791557
WAKMAR2	0.0057231	0.0056722	0.0108588	0.0072125	0.0154190
TNFAIP3	0.0152932	0.0104697	0.0123209	0.0040056	0.0094975
PERP	0.7685461	0.5007049	0.3560921	0.8318064	0.5085415
ARFGEF3	0.5902876	0.7495448	0.7400397	0.4513461	0.9910404
PBOV1	0.0000000	0.0000000	0.0000000	0.0000000	0.0000000
SMIM28	0.0054319	0.0068567	0.0032494	0.0000000	0.0000000
HEBP2	0.8094600	0.8609624	0.6896654	0.7665995	0.6629525
NHSL1	0.3519232	0.2947795	0.1942832	0.3232790	0.3999215
NHSL1-AS1	0.0095132	0.0187102	0.0193536	0.0147258	0.0083750
CCDC28A-AS1	0.0074576	0.0026031	0.0080554	0.0061376	0.0118665
CCDC28A	0.0868489	0.0576269	0.0469538	0.0483975	0.0891587
ECT2L	0.0074834	0.0088987	0.0079352	0.0096760	0.0261053
REPS1	0.2773630	0.2925256	0.2278457	0.3199795	0.4697913
ABRACL	0.0983692	0.1601490	0.1383304	0.0876616	0.1132952
HECA	0.1330577	0.1756106	0.1817230	0.0719692	0.1741089
ENSG00000231329	0.0064063	0.0056258	0.0037659	0.0042272	0.0224566
TXLNB	0.8194422	0.3930153	0.2043554	0.6629382	0.4217565
ENSG00000226571	0.0082695	0.0103772	0.0044199	0.0033054	0.0180518

CITED2	0.9758128	0.6419796	0.5431632	0.9432203	0.5514337
LINC01625	0.0053551	0.0051454	0.0000000	0.0000000	0.0019007
LINC02941	0.0000000	0.0012391	0.0000000	0.0000000	0.0000000
ENSG00000288714	0.0034867	0.0000000	0.0000000	0.0000000	0.0000000
ENSG00000234147	0.0018596	0.0019683	0.0022807	0.0044097	0.0096143
NMBR	0.0147044	0.0115999	0.0085968	0.0085553	0.0277492
VTA1	0.6260889	0.6379043	0.6775460	0.6323772	0.6080076
ADGRG6	0.0154924	0.0042017	0.0046863	0.0109907	0.0036367
HIVEP2	0.2979809	0.4140163	0.4028961	0.3149448	0.7268650
ENSG00000237851	0.0000000	0.0000000	0.0000000	0.0000000	0.0000000
HIVEP2-DT	0.0019006	0.0092347	0.0096919	0.0148680	0.0138625
LINC01277	0.0264653	0.0274525	0.0162867	0.0127224	0.0546388
ENSG00000227192	0.0042001	0.0036595	0.0000000	0.0174784	0.0028046
AIG1	0.6243398	0.6145631	0.5732440	0.7146163	0.7603913
ENSG00000225752	0.0000000	0.0000000	0.0000000	0.0000000	0.0059786
ADAT2	0.0230573	0.0551441	0.0428129	0.0300666	0.0335427
PEX3	0.1517659	0.1462901	0.1356445	0.1458001	0.2203345
FUCA2	0.3530907	0.2835970	0.2694673	0.5131629	0.1828167
PHACTR2	0.2053310	0.1553950	0.1064329	0.1812963	0.2607205
PHACTR2-AS1	0.0000000	0.0000000	0.0000000	0.0000000	0.0000000
LTV1	0.1963931	0.1449740	0.1838048	0.2179658	0.1811990
ZC2HC1B	0.0028038	0.0020162	0.0025063	0.0000000	0.0279615
PLAGL1	0.0615626	0.0648543	0.0455747	0.0442288	0.1521955
HYMAI	0.0033419	0.0071078	0.0153342	0.0000000	0.0103086
SF3B5	0.8842437	0.9193964	0.8625177	0.9213856	0.6968055
STX11	0.0102981	0.0085385	0.0070334	0.0000000	0.0081743
UTRN	0.2674598	0.2950205	0.1776226	0.2218575	0.4483980
ENSG00000225311	0.0010890	0.0000000	0.0000000	0.0000000	0.0000000
EPM2A	0.1530816	0.1042299	0.0782486	0.1108708	0.2409713
ENSG00000270638	0.0117358	0.0224541	0.0248882	0.0439590	0.0445055
ENSG00000270828	0.0012888	0.0012268	0.0024748	0.0000000	0.0000000
ENSG00000288551	0.0166072	0.0158746	0.0152037	0.0116403	0.0365068
ENSG00000288056	0.0000000	0.0000000	0.0000000	0.0000000	0.0000000
FBXO30	0.2843791	0.3057267	0.3552218	0.2565370	0.3229507
EPM2A-DT	0.3371640	0.2817123	0.2737411	0.3483751	0.2269983
SHPRH	0.2546909	0.2558431	0.2632286	0.3451349	0.5747331
GRM1	0.0269909	0.0346483	0.0285706	0.0530857	0.0684561
RAB32	0.3569130	0.2715430	0.1523952	0.3899309	0.1959108
ADGB	0.0473519	0.0296352	0.0113119	0.0327462	0.0342111
STXBP5-AS1	0.1210383	0.1432606	0.2151987	0.0579017	0.4293640
ENSG00000227748	0.0002597	0.0018488	0.0000000	0.0000000	0.0021475
ENSG00000272397	0.0020143	0.0000000	0.0000000	0.0000000	0.0000000
STXBP5	0.2253603	0.3183812	0.3435144	0.1517080	0.7303739
SAMD5	0.1799884	0.2322722	0.2345642	0.1179369	1.0375974
ENSG00000227681	0.1246312	0.1218520	0.0861000	0.0774439	0.9137052
ENSG00000226249	0.0000000	0.0000000	0.0000000	0.0000000	0.0104275
ENSG00000287976	0.0218673	0.0263011	0.0082028	0.0177512	0.1340727
ENSG00000234675	0.0000000	0.0000000	0.0000000	0.0000000	0.0044438
ENSG00000230205	0.0000000	0.0000000	0.0000000	0.0000000	0.0000000
ENSG00000287473	0.0011922	0.0000000	0.0000000	0.0000000	0.0000000
ENSG00000224658	0.0000000	0.0040489	0.0014741	0.0025217	0.0209488
SASH1	0.2692545	0.2274751	0.1745903	0.2794933	0.6265344
UST	0.0915976	0.1073076	0.0644095	0.0841356	0.2433873

UST-AS1	0.0000000	0.0000000	0.0000000	0.0000000	0.0000000
UST-AS2	0.0000000	0.0000000	0.0000000	0.0014092	0.0000000
ENSG00000289359	0.0000000	0.0000000	0.0000000	0.0000000	0.0000000
TAB2	0.4421196	0.3687005	0.2750417	0.4637618	0.4382273
SUMO4	0.0058606	0.0015070	0.0018514	0.0050458	0.0032497
ZC3H12D	0.0031497	0.0000000	0.0000000	0.0043699	0.0105905
ENSG00000289045	0.0643816	0.0661909	0.0898656	0.0584042	0.0615608
PPIL4	0.8414710	0.7252052	0.6999973	0.8019053	0.6336161
GINM1	0.8496611	0.6253809	0.5315465	0.9895205	0.5920786
ENSG00000281021	0.0028555	0.0007294	0.0000000	0.0000000	0.0025607
ENSG00000233330	0.0035859	0.0045250	0.0007009	0.0042484	0.0111101
KATNA1	0.2425277	0.1757636	0.1416360	0.1736400	0.1860389
LATS1	0.2647747	0.2342613	0.2297625	0.2997603	0.2869766
ENSG00000278899	0.0116128	0.0059476	0.0007307	0.0017529	0.0000000
NUP43	0.1622124	0.1180201	0.1183623	0.1607518	0.1533345
PCMT1	0.8559780	0.8592663	0.9805625	0.9641508	0.7783798
ENSG00000231760	0.0020820	0.0013436	0.0000000	0.0048815	0.0000000
LRP11	0.3690804	0.3318002	0.3435455	0.3741778	0.3901821
RAET1E-AS1	0.0202197	0.0174132	0.0085686	0.0098429	0.0185867
ULBP2	0.0040453	0.0160766	0.0142783	0.0128240	0.0114516
ULBP1	0.0319812	0.0186965	0.0128465	0.0174234	0.0119313
ULBP3	0.2922149	0.1691122	0.1250361	0.2699110	0.2215615
PPP1R14C	1.4199268	1.0527669	0.9443816	1.5116989	0.9928643
IYD	0.0007677	0.0000000	0.0000000	0.0000000	0.0076764
PLEKHG1	0.1737350	0.1312675	0.0971684	0.1520828	0.2524063
MTHFD1L	0.3094492	0.3316766	0.3513256	0.2962707	0.5267289
ENSG00000223598	0.0100987	0.0093232	0.0164804	0.0033620	0.0230114
AKAP12	1.0128683	1.4081178	1.4893611	0.8570517	1.2953738
ZBTB2	0.0768812	0.0506405	0.0542616	0.0530637	0.0560451
RMND1	0.2445166	0.2771653	0.3225063	0.2272782	0.2030775
ARMT1	0.2067464	0.1946385	0.1633646	0.2020066	0.1226543
CCDC170	0.3896229	0.2339464	0.1458035	0.4130359	0.1977650
ESR1	0.0249173	0.0154314	0.0000000	0.0092476	0.0013524
SYNE1	0.7161771	0.6505747	0.6128977	0.6328115	1.1310473
LINC02840	0.0037320	0.0000000	0.0000000	0.0077234	0.0000000
FBXO5	0.0708364	0.0425432	0.0417078	0.0656967	0.0726306
ENSG00000227627	0.0333107	0.0313141	0.0154310	0.0271332	0.0746008
MTRF1L	0.3214506	0.3811019	0.3195550	0.3595169	0.2909285
RGS17	0.2323539	0.4266371	0.5983140	0.1683823	0.5211711
ENSG00000213121	0.0000000	0.0025897	0.0000000	0.0011545	0.0067487
OPRM1	0.0367304	0.0609022	0.1265150	0.0184914	0.0901131
IPCEF1	0.0039722	0.0069102	0.0031144	0.0000000	0.0073759
CNKSR3	0.0584311	0.0504604	0.0263966	0.0523445	0.0692425
ENSG00000287260	0.0000000	0.0000000	0.0000000	0.0019281	0.0000000
SCAF8	0.3137855	0.3161723	0.2797390	0.3172889	0.5731139
TIAM2	0.1623905	0.1693913	0.1501642	0.0966087	0.4643553
ENSG00000235381	0.0063705	0.0132423	0.0084702	0.0066250	0.0053082
TFB1M	0.1404468	0.1193848	0.1353878	0.1313352	0.1227179
CLDN20	0.0000000	0.0000000	0.0000000	0.0000000	0.0000000
ENSG00000232529	0.0012646	0.0000000	0.0045631	0.0000000	0.0062081
ENSG00000287092	0.0134516	0.0190192	0.0037961	0.0042145	0.0690155
ENSG00000271265	0.0028214	0.0012453	0.0000000	0.0036873	0.0027882
ARID1B	0.5149559	0.5955507	0.6256133	0.4803095	1.2485218

ENSG00000271551	0.0309683	0.0253452	0.0200941	0.0234069	0.0109617
ENSG00000288910	0.0003405	0.0000000	0.0024942	0.0095147	0.0228818
ENSG00000233044	0.0019240	0.0011890	0.0000000	0.0000000	0.0000000
TMEM242	0.2805408	0.2894817	0.2467110	0.3534508	0.2220786
TMEM242-DT	0.0029419	0.0017934	0.0030237	0.0000000	0.0000000
ENSG00000286482	0.0010940	0.0000000	0.0000000	0.0000000	0.0028551
ZDHHC14	0.2664973	0.3055665	0.2402716	0.2692163	0.5527018
SNX9	0.2752029	0.2848193	0.2196663	0.2949193	0.4091452
SNX9-AS1	0.0000000	0.0014948	0.0000000	0.0000000	0.0112969
SYNJ2	0.0303901	0.0239420	0.0088078	0.0368229	0.0310271
SERAC1	0.1322558	0.1525097	0.2005653	0.1466460	0.1493864
GTF2H5	0.9193421	0.9471864	0.9382606	0.9877670	0.6641685
TULP4	0.7365398	0.9745141	1.0649328	0.5899411	0.9942328
ENSG00000274023	0.0000000	0.0000000	0.0000000	0.0000000	0.0028551
ENSG00000287591	0.0000000	0.0000000	0.0000000	0.0000000	0.0030362
TMEM181	0.2133525	0.1963060	0.2294026	0.2582181	0.4268339
DYNLT1	1.6837460	1.6182791	1.6026340	1.6661307	1.3299809
SYTL3	0.0376523	0.0151756	0.0099200	0.0349816	0.0257977
EZR	1.1144613	0.7173739	0.4651149	1.1769926	0.6631081
LINC02901	0.0051292	0.0046326	0.0029641	0.0039687	0.0079095
RSPH3	0.2421647	0.2401543	0.1734877	0.3136668	0.2594182
TAGAP-AS1	0.1163322	0.1727151	0.1199566	0.1149311	0.2988659
TAGAP	0.0019300	0.0000000	0.0000000	0.0000000	0.0000000
ENSG00000288845	0.0050151	0.0185722	0.0066356	0.0016564	0.0310869
ENSG00000285492	0.0003197	0.0000000	0.0028843	0.0000000	0.0054053
ENSG00000224478	0.0009479	0.0000000	0.0040572	0.0000000	0.0000000
FNDC1-AS1	0.0219696	0.0097877	0.0120264	0.0420152	0.0000000
FNDC1	0.0297470	0.0231669	0.0056132	0.1446803	0.0362800
ENSG00000286533	0.0165184	0.0074204	0.0202992	0.0095892	0.0273079
ENSG00000237927	0.0000000	0.0024079	0.0011570	0.0000000	0.0000000
SOD2	0.6943153	0.7799425	0.9104354	0.6126105	0.6589846
WTAP	0.5764859	0.5666210	0.6277315	0.5667243	0.5520731
SOD2-OT1	0.0031300	0.0046728	0.0028387	0.0052219	0.0026876
ACAT2	0.3538111	0.4329291	0.5433823	0.4513136	0.3521469
TCP1	1.0960442	1.2036736	1.2943134	1.0003957	0.9611196
MRPL18	0.4812409	0.4811542	0.4580549	0.5470892	0.3406711
PNLDC1	0.0000000	0.0000000	0.0000000	0.0000000	0.0051643
MAS1	0.0006031	0.0000000	0.0000000	0.0000000	0.0070395
IGF2R	0.4209859	0.2840965	0.2319348	0.7020693	0.5323090
AIRN	0.0043955	0.0071777	0.0032599	0.0052040	0.0000000
SLC22A1	0.0028376	0.0027646	0.0000000	0.0000000	0.0015171
SLC22A2	0.0000000	0.0000000	0.0000000	0.0000000	0.0000000
SLC22A3	0.0067639	0.0011064	0.0000000	0.0032682	0.0066151
LPAL2	0.0109952	0.0159459	0.0085830	0.0068197	0.0346033
LPA	0.0204104	0.0261081	0.0228032	0.0056172	0.1084980
PLG	0.0014711	0.0007328	0.0000000	0.0000000	0.0156002
ENSG00000287558	0.0079477	0.0170279	0.0340012	0.0160157	0.0631188
ENSG00000231863	0.0140747	0.0245972	0.0124230	0.0220073	0.0521395
MAP3K4-AS1	0.0707324	0.0749460	0.0499046	0.0832955	0.0287289
MAP3K4	0.2224629	0.2619340	0.2889891	0.1597617	0.4671551
AGPAT4	0.3558018	0.3538706	0.3148112	0.4450883	0.6617012
PRKN	0.1726703	0.2017838	0.1162072	0.1002148	0.7717805
ENSG00000286805	0.0035495	0.0039542	0.0022896	0.0021445	0.0000000

PACRG	0.5258984	0.3879119	0.3655234	0.6452312	0.7117298
ENSG00000285553	0.0000000	0.0000000	0.0000000	0.0000000	0.0000000
PACRG-AS3	0.0017724	0.0000000	0.0022183	0.0000000	0.0120109
PACRG-AS1	0.0000000	0.0016270	0.0000000	0.0000000	0.0000000
ENSG00000285564	0.0230304	0.0119333	0.0066100	0.0184082	0.0150062
CAHM	0.1281265	0.1105584	0.0729644	0.1338162	0.0520756
QKI	1.7691504	1.3873079	1.0019692	1.8139078	1.8070523
ENSG00000228692	0.0000000	0.0008104	0.0000000	0.0000000	0.0000000
ENSG00000235538	0.0000000	0.0000000	0.0000000	0.0000000	0.0000000
ENSG00000288696	0.0054208	0.0019055	0.0000000	0.0000000	0.0091454
ENSG00000260422	0.0007954	0.0007381	0.0021854	0.0000000	0.0000000
ENSG00000287550	0.0149632	0.0030354	0.0049822	0.0356547	0.0000000
ENSG00000287877	0.0032239	0.0048507	0.0007378	0.0037697	0.0120678
C6orf118	0.2330769	0.1275859	0.1013848	0.2461884	0.1663909
PDE10A	0.2878686	0.3505965	0.3486363	0.1873772	1.0883049
ENSG00000236627	0.0011374	0.0000000	0.0000000	0.0000000	0.0237464
ENSG00000223942	0.0043781	0.0011331	0.0000000	0.0025427	0.0062626
LINC00602	0.0000000	0.0000000	0.0000000	0.0044469	0.0022413
ENSG00000280850	0.0049944	0.0041872	0.0022411	0.0045055	0.0028456
PRR18	0.0235946	0.0152326	0.0064611	0.0298843	0.0130666
ENSG00000287189	0.0157672	0.0113492	0.0149423	0.0261704	0.0046716
SFT2D1	0.3880190	0.3038769	0.2687133	0.4296688	0.3328632
ENSG00000286760	0.0549236	0.0396446	0.0328982	0.0407382	0.0348146
MPC1	0.7772251	0.6878636	0.7071592	0.9170724	0.5266726
MPC1-DT	0.0238901	0.0302222	0.0308289	0.0108407	0.0499569
RPS6KA2	0.2895230	0.4146838	0.4842731	0.2321150	0.6853611
RPS6KA2-IT1	0.0000000	0.0000000	0.0000000	0.0000000	0.0065001
RAMACL	0.0023597	0.0090880	0.0059251	0.0035651	0.0124884
RNASET2	0.5138330	0.4208476	0.3804180	0.6868634	0.5244534
ENSG00000227598	0.0091415	0.0084027	0.0127197	0.0099150	0.0158046
ENSG00000285730	0.0000000	0.0000000	0.0000000	0.0000000	0.0000000
CEP43	0.1993105	0.2011177	0.2326640	0.2235896	0.3855084
CCR6	0.0032900	0.0012030	0.0000000	0.0024198	0.0081050
ENSG00000231720	0.0041362	0.0019004	0.0028723	0.0000000	0.0000000
ENSG00000228648	0.0029018	0.0000000	0.0000000	0.0038132	0.0000000
TTLL2	0.0022928	0.0000000	0.0084493	0.0000000	0.0025988
ENSG00000287594	0.0054698	0.0029764	0.0000000	0.0000000	0.0000000
LINC01558	0.0005168	0.0000000	0.0022266	0.0000000	0.0000000
AFDN-DT	0.1383325	0.0999101	0.1134934	0.1243474	0.0496689
AFDN	0.6994260	0.7183172	0.7143236	0.6307297	1.0151050
ENSG00000235994	0.0004988	0.0000000	0.0025093	0.0000000	0.0000000
KIF25-AS1	0.0032002	0.0000000	0.0016462	0.0000000	0.0000000
KIF25	0.0014272	0.0033070	0.0082492	0.0000000	0.0000000
DACT2	0.0319490	0.0254000	0.0266015	0.0300044	0.0265861
ENSG00000224417	0.0011356	0.0014074	0.0000000	0.0000000	0.0000000
SMOC2	0.1278142	0.0814525	0.0467103	0.1583605	0.1331358
ENSG00000235815	0.0000000	0.0007371	0.0000000	0.0000000	0.0000000
ENSG00000289090	0.0000000	0.0012174	0.0000000	0.0000000	0.0000000
THBS2-AS1	0.0126746	0.0101422	0.0127382	0.0037348	0.0209356
THBS2	0.0415463	0.0205730	0.0176341	0.0800327	0.0490317
LINC02519	0.0026731	0.0064274	0.0055457	0.0000000	0.0170382
ENSG00000285887	0.0053969	0.0033962	0.0000000	0.0038887	0.0027882
ENSG00000272848	0.0051239	0.0065441	0.0090340	0.0144501	0.0141709

WDR27	0.1720605	0.1529169	0.1538394	0.1951620	0.3494686
C6orf120	0.2104018	0.1611872	0.1607956	0.1757521	0.1291561
PHF10	0.4492653	0.2901734	0.2340578	0.4327624	0.4209285
ENSG00000227704	0.0971785	0.1083157	0.0851840	0.0794174	0.0790750
ENSG00000232640	0.0029770	0.0044156	0.0010835	0.0075526	0.0000000
DYNLT2	0.0070613	0.0106278	0.0153564	0.0047879	0.0128891
ERMARD	0.0762661	0.0693444	0.0413288	0.0740676	0.0941852
ENSG00000266896	0.0008634	0.0000000	0.0000000	0.0000000	0.0032954
LINC00242	0.0046851	0.0084027	0.0088812	0.0000000	0.0137087
LINC00574	0.0047670	0.0148238	0.0212566	0.0022571	0.0166416
DLL1	0.0354426	0.0643543	0.0593553	0.0224299	0.0761239
FAM120B	0.2053366	0.2305281	0.1792033	0.2488850	0.5050734
ENSG00000261003	0.0008958	0.0016970	0.0011788	0.0000000	0.0053214
ENSG00000288829	0.0316764	0.0310624	0.0175716	0.0775996	0.0469356
PSMB1	1.2865151	1.1425185	1.1421929	1.2645961	0.9483942
TBP	0.1941180	0.1723813	0.2173999	0.1739414	0.1676571
PDCD2	0.4646354	0.4370783	0.4882785	0.4559170	0.3442369
ENSG00000230423	0.0030189	0.0023009	0.0067230	0.0039588	0.0015176
FAM157D	0.0000000	0.0000000	0.0012725	0.0000000	0.0035640
LINC03015	0.0268955	0.0327029	0.0427164	0.0324721	0.0191055
LINC03014	0.0039083	0.0021072	0.0000000	0.0000000	0.0000000
ENSG00000240093	0.0020700	0.0009099	0.0091196	0.0000000	0.0049754
FAM20C	0.1295498	0.1778901	0.1769634	0.1449988	0.2283319
PDGFA	0.2664941	0.3015754	0.2734429	0.2798865	0.2053601
PDGFA-DT	0.0028085	0.0059294	0.0010907	0.0047714	0.0024211
PRKAR1B	0.2673141	0.4099242	0.4961581	0.2625935	0.4925015
DNAAF5	0.1319441	0.0956645	0.0733598	0.2067467	0.1352876
SUN1	0.3153144	0.2601022	0.2419767	0.4046998	0.4494703
GET4	0.0040192	0.0044173	0.0115951	0.0079186	0.0070352
ENSG00000273151	0.0051173	0.0064989	0.0027002	0.0000000	0.0401076
ADAP1	0.0902736	0.1857396	0.1945694	0.0878733	0.1022151
COX19	0.2240681	0.2418435	0.2539184	0.1643417	0.2714027
C7orf50	1.0452522	0.8890838	0.8001595	1.0959511	0.8180013
ENSG00000225146	0.0035887	0.0032077	0.0037614	0.0117836	0.0045621
GPR146	0.0238491	0.0244322	0.0350562	0.0227065	0.0108418
ENSG00000257607	0.0000000	0.0000000	0.0000000	0.0000000	0.0066745
GPER1	0.0261965	0.0104249	0.0009612	0.0450146	0.0159175
ZFAND2A	0.2961921	0.3996517	0.4636613	0.2939992	0.2740344
ZFAND2A-DT	0.1575273	0.1253180	0.0857357	0.1324855	0.1098270
UNCX	0.0023776	0.0007934	0.0079642	0.0000000	0.0089701
MICALL2	0.0228375	0.0284757	0.0191994	0.0178983	0.0542302
MICALL2-DT	0.0023771	0.0000000	0.0000000	0.0000000	0.0000000
ENSG00000273230	0.0092385	0.0151766	0.0200286	0.0115094	0.0275320
INTS1	0.0939724	0.0811891	0.0601413	0.1043912	0.1624186
MAFK	0.0321615	0.0340153	0.0385563	0.0552196	0.0326216
TMEM184A	0.0006318	0.0052719	0.0000000	0.0000000	0.0000000
PSMG3	0.1505839	0.1825330	0.1665259	0.1264501	0.1025052
PSMG3-AS1	0.0338909	0.0295914	0.0094964	0.0330349	0.0244308
ELFN1	0.0428389	0.1019973	0.0945413	0.0349887	0.2685475
MAD1L1	0.1019277	0.1183988	0.1217112	0.0912403	0.2925024
MRM2	0.2238455	0.2404999	0.2278279	0.2471746	0.1670627
NUDT1	0.1743521	0.1596539	0.0948934	0.1853352	0.0745482
SNX8	0.0652351	0.0426444	0.0689087	0.0858665	0.0740317

EIF3B	0.3662487	0.2650210	0.2502279	0.3538855	0.2836790
CHST12	0.1432778	0.1030564	0.0868842	0.2243864	0.1067880
LFNG	0.0375032	0.0219503	0.0112405	0.1513403	0.0098116
BRAT1	0.1664098	0.1209489	0.0628228	0.1410418	0.0908162
IQCE	0.5788549	0.4717644	0.3965127	0.5195712	0.4642907
ENSG00000289352	0.0021712	0.0000000	0.0007625	0.0030728	0.0000000
TTYH3	0.3807480	0.3745725	0.3755407	0.5057783	0.3705936
ENSG00000288792	0.0025659	0.0051618	0.0000000	0.0000000	0.0107047
AMZ1	0.0245805	0.0128288	0.0206584	0.0186118	0.0408831
GNA12	0.4622763	0.3691823	0.2822288	0.4661100	0.4150974
CARD11	0.0027396	0.0029240	0.0046251	0.0000000	0.0083683
ENSG00000228334	0.0000000	0.0012391	0.0000000	0.0000000	0.0000000
ENSG00000217455	0.0080146	0.0043949	0.0009839	0.0068167	0.0107097
SDK1-AS1	0.0026131	0.0000000	0.0024397	0.0041424	0.0000000
SDK1	0.3867300	0.3381681	0.3459958	0.3513940	1.1487667
ENSG00000286874	0.0031459	0.0000000	0.0000000	0.0111591	0.0090643
FOXK1	0.2577912	0.2420874	0.2318014	0.2254835	0.3098357
ENSG00000287665	0.0020943	0.0030921	0.0000000	0.0085543	0.0018550
AP5Z1	0.0581884	0.0458398	0.0408816	0.0861167	0.0179093
RADIL	0.0428708	0.0562233	0.0687901	0.0140291	0.1530649
PAPOLB	0.0000000	0.0012589	0.0000000	0.0023978	0.0000000
MMD2	0.0114545	0.0234437	0.0153835	0.0111221	0.1075553
RNF216P1	0.2786440	0.3262490	0.3541539	0.2895088	0.3783523
RBAK	0.1176973	0.1474979	0.1266053	0.0674004	0.1671719
RBAKDN	0.0247137	0.0061495	0.0154319	0.0201846	0.0100562
ENSG00000291037	0.0043638	0.0010205	0.0011774	0.0000000	0.0000000
WIPI2	0.5235630	0.4463476	0.4828982	0.4883477	0.3720541
SLC29A4	0.0800512	0.1061061	0.0990412	0.0862282	0.1359515
TNRC18	0.3001168	0.3015272	0.2673053	0.2152174	0.6165531
ENSG00000241269	0.0028427	0.0000000	0.0015130	0.0044452	0.0026919
ENSG00000188365	0.0004950	0.0000000	0.0006655	0.0011694	0.0000000
ENSG00000290091	0.0002181	0.0000000	0.0000000	0.0052026	0.0053413
ENSG00000272953	0.0018207	0.0046098	0.0000000	0.0000000	0.0118830
LINC02983	0.0078456	0.0038410	0.0039968	0.0083275	0.0272415
LINC03073	0.0094883	0.0050275	0.0195236	0.0098478	0.0062307
FBXL18	0.0744737	0.0850856	0.0862832	0.0501654	0.0902136
ENSG00000230733	0.0010252	0.0000000	0.0080619	0.0035066	0.0028666
ACTB	3.5947460	3.5621183	3.7483090	3.7571712	3.2391086
FSCN1	0.9039765	0.9914776	1.0767516	1.0589354	0.7787324
RNF216	0.5422696	0.3567686	0.3063275	0.4920737	0.5926811
ENSG00000291099	0.0611036	0.0825327	0.1145272	0.0624664	0.1123199
OCM	0.0068615	0.0108455	0.0161998	0.0000000	0.0137667
CCZ1	0.1769235	0.1747619	0.1902072	0.1839516	0.1312318
RSPH10B	0.0037481	0.0059429	0.0000000	0.0026458	0.0000000
PMS2	0.1824071	0.1917837	0.2016002	0.1836830	0.2216647
AIMP2	0.2723023	0.2800189	0.2441680	0.2785295	0.1852186
EIF2AK1	1.0308542	0.8758352	0.9244763	1.0449645	0.7627584
ANKRD61	0.0000000	0.0011504	0.0019944	0.0000000	0.0042562
USP42	0.1629470	0.1437583	0.1697028	0.1274697	0.2817593
CYTH3	0.0633609	0.0748538	0.0917652	0.0530570	0.1801017
RAC1	1.8130178	1.7618988	1.8495442	1.7836419	1.4279418
DAGLB	0.0311612	0.0164100	0.0321993	0.0560998	0.0611665
KDELR2	0.9107845	0.8226026	0.7735123	0.9816651	0.6702052

GRID2IP	0.0088361	0.0102532	0.0074994	0.0079152	0.0158316
ZDHHC4	0.5003817	0.4347443	0.4147789	0.5179511	0.3288221
ENSG00000232581	0.0008219	0.0060540	0.0056322	0.0000000	0.0030611
INTS15	0.1319803	0.1266616	0.1695698	0.1072775	0.1495361
ZNF853	0.2381343	0.2817384	0.3467763	0.2205500	0.2813628
ZNF316	0.0740949	0.0839883	0.1032127	0.0492479	0.1205419
ENSG00000228010	0.0498099	0.0754989	0.0740771	0.0551474	0.1671615
ZNF12	0.1685888	0.1660434	0.1532377	0.1434893	0.1506195
ENSG00000290835	0.0571186	0.0678074	0.0555349	0.0698450	0.1277594
RSPH10B2	0.0155501	0.0073733	0.0062580	0.0073117	0.0045792
CCZ1B	0.2342348	0.2239393	0.2634133	0.2663209	0.1910648
ENSG00000287860	0.0006139	0.0000000	0.0000000	0.0000000	0.0000000
C1GALT1	0.2961498	0.2418093	0.2245539	0.3269079	0.3190779
LINC03016	0.0100452	0.0051737	0.0020060	0.0067065	0.0183278
COL28A1	0.0146762	0.0119950	0.0042445	0.0000000	0.0744929
ENSG00000272732	0.0036169	0.0034225	0.0037446	0.0041602	0.0153554
MIOS-DT	0.0094460	0.0135787	0.0099613	0.0203000	0.0384686
MIOS	0.1613778	0.1853545	0.1694344	0.1275773	0.2733957
ENSG00000272745	0.0000000	0.0000000	0.0020397	0.0000000	0.0018039
RPA3	0.2673153	0.2147520	0.2453173	0.2560628	0.1598196
UMAD1	0.2227426	0.2079523	0.2229433	0.2365643	0.3691834
ENSG00000234141	0.0040825	0.0132768	0.0049888	0.0014906	0.0120983
GLCCI1-DT	0.0023656	0.0022964	0.0028843	0.0077234	0.0000000
GLCCI1	0.2232031	0.3205710	0.2375150	0.1384097	0.6452630
ICA1	0.2293906	0.3982429	0.4883470	0.2428822	0.4744629
ENSG00000227719	0.0000000	0.0000000	0.0000000	0.0000000	0.0000000
ICA1-AS1	0.0755147	0.0919640	0.0459774	0.0663047	0.0648772
ENSG00000272328	0.0100819	0.0048677	0.0068335	0.0000000	0.0202639
NXPH1	0.2047504	0.2855725	0.3327982	0.1361497	0.9395044
ENSG00000234710	0.0000000	0.0000000	0.0000000	0.0000000	0.0000000
ENSG00000286639	0.0027721	0.0018891	0.0027408	0.0000000	0.0000000
MGC4859	0.0028034	0.0049954	0.0000000	0.0000000	0.0080379
NDUFA4	1.9489837	2.0832063	2.1918261	1.9654448	1.7249999
PHF14	0.8046489	0.9000546	0.9782814	0.7522120	1.1516430
ENSG00000230333	0.0115195	0.0123370	0.0086426	0.0076663	0.0684560
THSD7A	0.4814151	0.3752681	0.3825694	0.5594915	0.7535210
TMEM106B	0.8426338	0.6695639	0.6145490	0.9502008	0.5682518
VWDE	0.0010259	0.0039478	0.0039529	0.0000000	0.0217679
ENSG00000226690	0.0049770	0.0028949	0.0000000	0.0060482	0.0000000
SCIN	0.0049458	0.0085061	0.0020787	0.0000000	0.0021475
ARL4A	0.3111570	0.2740178	0.2709044	0.2162386	0.2502078
ENSG00000229618	0.0085493	0.0068536	0.0072112	0.0098275	0.0382001
ETV1	0.1546497	0.0853860	0.0352753	0.1699361	0.0895094
DGKB	0.1780573	0.2564003	0.1817610	0.2097017	0.7656122
AGMO	0.0004262	0.0038647	0.0014377	0.0063320	0.0000000
MEOX2	0.0012493	0.0009830	0.0000000	0.0000000	0.0088129
ENSG00000286376	0.0008421	0.0035058	0.0012912	0.0000000	0.0017501
CRPPA	0.0618481	0.0875168	0.0403446	0.0380328	0.1695888
CRPPA-AS1	0.0006855	0.0000000	0.0000000	0.0000000	0.0000000
SOSTDC1	0.0008189	0.0049470	0.0041566	0.0000000	0.0131473
LRRC72	0.0000000	0.0016904	0.0000000	0.0000000	0.0041999
ENSG00000272361	0.0017324	0.0045219	0.0022302	0.0000000	0.0113646
ANKMY2	0.3774445	0.4268831	0.4882462	0.3712881	0.3732726

ENSG00000287799	0.0000000	0.0007691	0.0000000	0.0000000	0.0000000
BZW2	0.2640369	0.4191880	0.7141035	0.1893529	0.4329052
ENSG00000235837	0.0013015	0.0015377	0.0028683	0.0000000	0.0000000
TSPAN13	0.4278274	0.6444197	0.8867709	0.4187616	0.5323383
AGR2	0.0000000	0.0014359	0.0000000	0.0000000	0.0025607
AGR3	0.0019246	0.0000000	0.0031493	0.0000000	0.0048241
AHR	0.1485938	0.1042279	0.0580432	0.1622259	0.1483593
ENSG00000237773	0.0371277	0.0277784	0.0271722	0.0363629	0.0653622
LINC02888	0.0009065	0.0011651	0.0013013	0.0000000	0.0000000
LINC02889	0.0000000	0.0013468	0.0000000	0.0000000	0.0113764
SNX13	0.3557162	0.3103413	0.2824647	0.3895610	0.5059893
HDAC9	0.5369784	0.4476039	0.3906123	0.6822628	0.8740425
HDAC9-AS1	0.0000000	0.0000000	0.0000000	0.0000000	0.0000000
TWIST1	0.0312970	0.0114810	0.0147653	0.0176305	0.0398386
ENSG00000236536	0.0015095	0.0026627	0.0000000	0.0000000	0.0050692
ENSG00000229533	0.0114736	0.0044896	0.0000000	0.0016235	0.0000000
FERD3L	0.0316764	0.0178953	0.0068597	0.0250410	0.0204290
ENSG00000223838	0.1369293	0.0933585	0.0667464	0.0923714	0.2366825
POLR1F	0.2820682	0.2717784	0.2873438	0.2939405	0.2306986
TMEM196	0.1958155	0.3456308	0.4875580	0.1176904	0.4603255
ENSG00000237921	0.0009060	0.0044091	0.0043384	0.0106012	0.0000000
ENSG00000243004	0.0467266	0.0671062	0.0463738	0.0518519	0.0914629
ENSG00000267055	0.0019543	0.0017886	0.0006194	0.0000000	0.0054454
MACC1	0.0051842	0.0000000	0.0043226	0.0000000	0.0000000
MACC1-AS1	0.0000000	0.0000000	0.0000000	0.0000000	0.0000000
GIRGL	0.0012131	0.0000000	0.0000000	0.0000000	0.0048106
ENSG00000226097	0.0015390	0.0000000	0.0000000	0.0031358	0.0048584
ITGB8-AS1	0.0310435	0.0222534	0.0298933	0.0445244	0.0298111
ITGB8	0.8777389	0.4778325	0.2940850	1.1546455	0.5853840
ABCB5	0.0012429	0.0000000	0.0000000	0.0037589	0.0101495
SP8	0.0050088	0.0187376	0.0008860	0.0057934	0.0246225
SP4	0.1727982	0.2018648	0.2416640	0.1953044	0.2779045
DNAH11	0.0920528	0.0840701	0.0493369	0.0945826	0.1160077
CDCA7L	0.0530440	0.0392858	0.0284394	0.0921671	0.0318398
RAPGEF5	0.0940233	0.1747942	0.2588316	0.0374800	0.3645351
STEAP1B	0.0481730	0.0466673	0.0433492	0.0413422	0.0511831
STEAP1B-AS1	0.0054872	0.0038526	0.0011203	0.0120360	0.0072173
ENSG00000232949	0.0065334	0.0027304	0.0011203	0.0078065	0.0000000
TOMM7	1.9220420	1.9053643	1.7058722	1.8924577	1.6792296
ENSG00000286703	0.0002885	0.0000000	0.0000000	0.0000000	0.0000000
SNHG26	0.0015633	0.0011972	0.0000000	0.0020756	0.0028353
HYCC1	0.1420945	0.1168574	0.0914245	0.1453227	0.1437543
KLHL7-DT	0.0008476	0.0000000	0.0000000	0.0000000	0.0000000
KLHL7	0.4580670	0.5029938	0.5588577	0.3920794	0.5860765
NUP42	0.2469170	0.2300718	0.2328307	0.2811415	0.2557011
ENSG00000285926	0.0000000	0.0000000	0.0000000	0.0000000	0.0063087
ENSG00000226816	0.0000000	0.0034873	0.0000000	0.0000000	0.0000000
GPNMB	0.0017534	0.0044227	0.0012928	0.0053087	0.0000000
MALSU1	0.3218325	0.2608521	0.2209637	0.2915012	0.1801249
IGF2BP3	0.2889213	0.2577678	0.1897084	0.2975447	0.4865894
ENSG00000289109	0.0014343	0.0000000	0.0000000	0.0000000	0.0000000
TRA2A	0.6097331	0.5256105	0.5787552	0.5816144	0.6862238
CCDC126	0.1748657	0.1692725	0.1395239	0.1980618	0.2029242

ENSG00000234286	0.0069856	0.0050089	0.0079573	0.0146075	0.0046312
FAM221A	0.2515983	0.2612379	0.2864192	0.2608027	0.1945541
STK31	0.0159679	0.0139701	0.0084582	0.0064747	0.0414338
NPY	0.0011966	0.0000000	0.0000000	0.0000000	0.0000000
ENSG00000287523	0.0003908	0.0009847	0.0000000	0.0000000	0.0099609
PALS2	0.3293390	0.4213392	0.4926468	0.2843427	0.5158955
GSDME	0.0638004	0.1041382	0.1260155	0.1035980	0.1183754
ENSG00000287093	0.0000000	0.0017787	0.0000000	0.0000000	0.0000000
OSBPL3	0.0633593	0.0588327	0.0291837	0.0373913	0.1583017
CYCS	1.1049562	1.4699867	1.4843770	1.0840294	1.0278664
C7orf31	0.0557938	0.0458015	0.0370240	0.0385783	0.0824665
ENSG00000260951	0.0010957	0.0029337	0.0019944	0.0129029	0.0044438
ENSG00000285960	0.0021894	0.0013102	0.0020031	0.0021653	0.0000000
LINC03007	0.0003990	0.0000000	0.0047909	0.0051655	0.0040262
NFE2L3	0.0248050	0.0449008	0.0810901	0.0119984	0.1017991
HNRNPA2B1	2.1491532	2.0386679	2.2150025	2.1979090	1.9106069
CBX3	1.3255985	1.4154659	1.4986533	1.2994825	1.1710924
SNX10	0.6843425	0.8817239	1.1081122	0.7256331	0.7424881
SNX10-AS1	0.2958834	0.2273272	0.1474470	0.3238247	0.1490985
LINC02981	0.2599955	0.2206850	0.2558312	0.2044111	0.3007113
KIAA0087	0.0000000	0.0000000	0.0000000	0.0000000	0.0000000
ENSG00000233760	0.0000000	0.0000000	0.0000000	0.0000000	0.0000000
ENSG00000286507	0.0016452	0.0000000	0.0027646	0.0000000	0.0000000
LINC02860	0.0000000	0.0000000	0.0000000	0.0000000	0.0000000
SKAP2	1.1494402	1.0505674	0.9124463	1.1043644	0.9542460
HOXA1	0.0284474	0.0484858	0.0397189	0.0214576	0.0257890
HOTAIRM1	0.7805474	0.7098901	0.5647436	0.6783786	0.6130289
HOXA2	0.2459339	0.1674583	0.1472521	0.2078394	0.1798863
HOXA3	0.4374035	0.4086463	0.3170743	0.3473731	0.4170416
HOXA-AS2	0.1220287	0.0855423	0.0604537	0.1326163	0.0618645
HOXA4	0.6488207	0.6102992	0.5127126	0.6656151	0.4241654
HOXA-AS3	0.0033857	0.0036898	0.0030039	0.0028893	0.0059619
HOXA5	0.2670616	0.2500692	0.1176559	0.2844855	0.2623820
HOXA6	0.0186273	0.0083602	0.0033218	0.0132208	0.0073410
HOXA7	0.0165170	0.0093135	0.0014673	0.0136524	0.0000000
HOXA10-AS	0.0000000	0.0000000	0.0000000	0.0000000	0.0000000
ENSG00000253308	0.0000000	0.0018449	0.0012014	0.0000000	0.0049812
EVX1-AS	0.0000000	0.0000000	0.0050927	0.0000000	0.0029771
EVX1	0.0000000	0.0070072	0.0055395	0.0000000	0.0065513
ENSG00000224322	0.0000000	0.0000000	0.0023388	0.0000000	0.0159896
HIBADH	0.2953663	0.2038068	0.2000876	0.3111709	0.2794193
ENSG00000286478	0.0000000	0.0000000	0.0000000	0.0000000	0.0082451
TAX1BP1-AS1	0.0015487	0.0014927	0.0023362	0.0000000	0.0112744
TAX1BP1	1.5358937	1.4021042	1.4881199	1.4936466	1.2633585
JAZF1	0.5009927	0.6614830	0.8169996	0.4583161	0.8663883
JAZF1-AS1	0.0040076	0.0070751	0.0152452	0.0023082	0.0129541
CREB5	0.7764667	0.6653085	0.3916617	0.7476103	0.9000070
TRIL	0.0169298	0.0156400	0.0119165	0.0190364	0.0152751
CPVL-AS2	0.0056924	0.0037186	0.0010701	0.0000000	0.0076572
CPVL	0.1651835	0.0857114	0.0476885	0.3868512	0.0598618
CHN2	0.2878083	0.2072468	0.1919604	0.2260115	0.2972044
ENSG00000285081	0.0033712	0.0023501	0.0000000	0.0000000	0.0000000
CHN2-AS1	0.0000000	0.0000000	0.0000000	0.0000000	0.0000000

PRR15-DT	0.0024242	0.0035253	0.0006832	0.0000000	0.0155036
PRR15	0.0034230	0.0038847	0.0031267	0.0000000	0.0000000
ENSG00000288973	0.0408535	0.0793800	0.0749010	0.0335260	0.0697929
DPY19L2P3	0.1001172	0.0770885	0.0804574	0.0365598	0.0922722
WIPF3	0.1859127	0.3684965	0.3136869	0.1929421	0.2805569
SCRN1	0.7573158	0.9615470	0.9619504	0.7572493	0.6545005
FKBP14-AS1	0.0121248	0.0281379	0.0214346	0.0086243	0.0321343
FKBP14	0.1193908	0.0857232	0.0470279	0.1694943	0.0705970
PLEKHA8	0.2772817	0.4621908	0.4230071	0.1954027	0.4495336
ENSG00000231519	0.0006530	0.0000000	0.0000000	0.0000000	0.0044415
ENSG00000286847	0.0019980	0.0027494	0.0016034	0.0035155	0.0045040
MTURN	1.0508883	1.1914966	1.3398306	0.8431805	0.9046760
ENSG00000230751	0.0000000	0.0000000	0.0000000	0.0038691	0.0000000
ENSG00000251660	0.0133734	0.0049794	0.0109782	0.0151807	0.0000000
ZNRF2	0.1713086	0.1627498	0.1436574	0.1843999	0.2044769
LINC01176	0.0078008	0.0016653	0.0032118	0.0069256	0.0218556
NOD1	0.0119911	0.0128901	0.0062980	0.0081110	0.0056253
GGCT	0.4337826	0.4503990	0.4569622	0.5483537	0.3844480
GARS1-DT	0.1353853	0.1960842	0.1325122	0.1319687	0.3335668
ENSG00000264520	0.0010393	0.0045350	0.0000000	0.0118825	0.0076526
GARS1	1.0582181	1.0807004	1.0815949	0.9675171	0.9036220
CRHR2	0.0032202	0.0080447	0.0000000	0.0000000	0.0122681
INMT	0.0018608	0.0030120	0.0011236	0.0054687	0.0000000
MINDY4	0.1009549	0.0620065	0.0475065	0.1590349	0.0928982
AQP1	0.1479185	0.0570441	0.0406327	0.2306725	0.0611066
GHRHR	0.0134427	0.0098024	0.0071796	0.0043143	0.0021475
ADCYAP1R1	0.1013877	0.1107183	0.0752035	0.1759927	0.1470113
ENSG00000286487	0.0000000	0.0025588	0.0000000	0.0000000	0.0000000
ITPRID1	0.0012052	0.0040732	0.0024311	0.0000000	0.0000000
PPP1R17	0.1053590	0.3129455	0.0616541	0.1254026	0.1516599
PDE1C	0.4301811	0.3160657	0.1445739	0.3277441	0.4084941
LSM5	0.6437073	0.6074508	0.5922607	0.6209349	0.5009506
AVL9	0.1799592	0.2302234	0.1829429	0.1345003	0.3525448
ENSG00000273014	0.0059444	0.0147808	0.0082603	0.0087643	0.0173640
LINC00997	0.0019145	0.0069166	0.0065857	0.0026398	0.0174323
ENSG00000272905	0.0000000	0.0008310	0.0015598	0.0000000	0.0000000
KBTBD2	0.2781077	0.2668350	0.3013524	0.3041201	0.2693847
RP9P	0.4139496	0.4894388	0.5906145	0.3346066	0.3966184
FKBP9	0.4149182	0.2537971	0.2261730	0.5390010	0.3044076
NT5C3A	0.3845413	0.4186826	0.4474052	0.3968023	0.4148284
RP9	0.3961996	0.3483385	0.3447696	0.3415856	0.3415361
BBS9	0.2446997	0.2364030	0.2597979	0.2147212	0.7021735
ENSG00000236494	0.0000000	0.0000000	0.0000000	0.0000000	0.0000000
BMPER	0.0015425	0.0168841	0.0082061	0.0000000	0.0127706
ENSG00000236212	0.0005211	0.0009512	0.0022017	0.0000000	0.0120439
NPSR1-AS1	0.0039080	0.0082688	0.0029335	0.0000000	0.0000000
NPSR1	0.0000000	0.0009004	0.0000000	0.0000000	0.0000000
DPY19L1	0.0831088	0.0487252	0.0394704	0.1387235	0.0808664
ENSG00000287249	0.0034652	0.0026701	0.0042486	0.0073271	0.0000000
ENSG00000291133	0.0506365	0.0800584	0.0721321	0.0585282	0.2033096
ENSG00000289089	0.0294063	0.0554024	0.0206308	0.0111025	0.0495985
ENSG00000288947	0.0047109	0.0117170	0.0071752	0.0030858	0.0032715
TBX20	0.0081958	0.0258314	0.0170580	0.0108621	0.0286398

ENSG00000287032	0.0018433	0.0022373	0.0000000	0.0000000	0.0104589
HERPUD2	0.2908739	0.2102093	0.1677827	0.2783579	0.2967841
HERPUD2-AS1	0.0590015	0.1056946	0.0952017	0.0471881	0.0654094
LINC03013	0.0021705	0.0026586	0.0041939	0.0286857	0.0000000
SEPTIN7-DT	0.0296584	0.0297659	0.0207660	0.0440899	0.0498844
SEPTIN7	2.1087236	2.1058012	2.1878144	2.1429741	1.8816505
ENSG00000287893	0.0000000	0.0000000	0.0000000	0.0000000	0.0000000
ENSG00000232930	0.0040361	0.0022139	0.0000000	0.0038647	0.0000000
EEPD1	0.1022353	0.0554389	0.0324789	0.1309955	0.0864786
MATCAP2	0.2647270	0.3142607	0.4008199	0.2804028	0.3155342
ANLN	0.0233991	0.0251012	0.0288983	0.0272029	0.0384725
ENSG00000237400	0.0007706	0.0000000	0.0000000	0.0000000	0.0024307
AOAH	0.0224472	0.0231668	0.0178034	0.0123498	0.0874895
ENSG00000229424	0.0000000	0.0000000	0.0000000	0.0000000	0.0000000
ENSG00000230831	0.0019302	0.0035586	0.0014397	0.0000000	0.0000000
ELMO1	1.3885766	1.0005792	0.8349761	0.9588093	1.1211147
ELMO1-AS1	0.0000000	0.0000000	0.0000000	0.0048295	0.0000000
SFRP4	0.0682288	0.0228947	0.0129248	0.0737498	0.0291353
EPDR1	0.2308519	0.1423299	0.0972574	0.2907939	0.1225983
STARD3NL	0.8847250	0.9690751	1.0408239	0.8761683	0.6693703
TRG-AS1	0.0000000	0.0000000	0.0000000	0.0000000	0.0000000
AMPH	0.2152883	0.4569442	0.5785394	0.2323680	0.5374076
VPS41	0.5245854	0.5295429	0.4948706	0.5405539	0.5416425
POU6F2	0.0279476	0.0355042	0.0271822	0.0225466	0.1727529
POU6F2-AS2	0.0042852	0.0000000	0.0000000	0.0000000	0.0000000
YAE1-DT	0.0029218	0.0019639	0.0006307	0.0137157	0.0142882
YAE1	0.2545959	0.2694019	0.2792130	0.2724220	0.1910062
ENSG00000287584	0.0054264	0.0000000	0.0026141	0.0000000	0.0036522
RALA	0.9169475	1.0488847	1.1254776	0.8659846	0.9543353
LINC00265	0.0286253	0.0404978	0.0400765	0.0568708	0.0937388
CDK13-DT	0.0026576	0.0028785	0.0000000	0.0140824	0.0021475
CDK13	0.5038096	0.4732993	0.3533789	0.3446829	0.6613023
ENSG00000289269	0.0016519	0.0031678	0.0016003	0.0000000	0.0033772
MPLKIP	0.5631043	0.5820275	0.5427279	0.5783367	0.4600617
SUGCT	0.0517560	0.0466729	0.0206223	0.0391365	0.0416239
SUGCT-AS1	0.0000000	0.0000000	0.0000000	0.0000000	0.0000000
INHBA	0.0119824	0.0070002	0.0138872	0.0022251	0.0160125
INHBA-AS1	0.0122651	0.0503532	0.0659055	0.0147564	0.0223691
GLI3	0.0613724	0.0261980	0.0268856	0.0165744	0.0428762
ENSG00000290065	0.0082356	0.0134104	0.0021727	0.0033511	0.0159921
ENSG00000261019	0.0032184	0.0000000	0.0000000	0.0000000	0.0000000
C7orf25	0.0715380	0.0755775	0.0964772	0.0742671	0.0574837
PSMA2	1.0211816	1.0020650	1.0461466	1.0530322	0.7541117
MRPL32	0.3147544	0.3094211	0.3110858	0.3057800	0.2553903
ENSG00000232006	0.0114115	0.0089773	0.0049167	0.0100070	0.0256283
HECW1	0.1372205	0.2138005	0.2189222	0.1227020	0.6544114
HECW1-IT1	0.0000000	0.0038797	0.0000000	0.0087770	0.0019545
ENSG00000228680	0.0000000	0.0000000	0.0000000	0.0000000	0.0000000
LUARIS	0.0000000	0.0000000	0.0000000	0.0000000	0.0070078
STK17A	0.1924106	0.1522098	0.0860669	0.3618478	0.1037872
COA1	0.5981028	0.5779030	0.6657570	0.5991859	0.5411951
BLVRA	0.3543381	0.3824532	0.3621575	0.4135507	0.2329523
MRPS24	0.6925840	0.7098802	0.7297886	0.6515807	0.4948187

URGCP	0.1376168	0.1025464	0.1115550	0.1079135	0.2170979
UBE2D4	0.2225759	0.1874443	0.2321228	0.2328003	0.1212919
SPDYE1	0.0004613	0.0000000	0.0011920	0.0000000	0.0076223
ENSG00000228434	0.0043239	0.0070864	0.0006194	0.0043438	0.0623651
ENSG00000290872	0.0052125	0.0072011	0.0000000	0.0172770	0.0242173
ENSG00000290758	0.0428082	0.0394654	0.0403242	0.0411617	0.0733506
LINC00957	0.0167902	0.0285659	0.0343242	0.0110366	0.0119359
ENSG00000288746	0.0025668	0.0040107	0.0000000	0.0071616	0.0056450
DBNL	0.3144424	0.2535150	0.2503790	0.3384151	0.2901005
PGAM2	0.0070476	0.0048518	0.0036268	0.0182027	0.0000000
POLM	0.0215137	0.0179183	0.0142659	0.0233692	0.0357676
AEBP1	0.3886184	0.2026428	0.1174201	1.0164430	0.2660084
POLD2	0.5351369	0.4132039	0.3851247	0.4789962	0.3185645
MYL7	0.0010767	0.0008641	0.0000000	0.0045766	0.0041014
GCK	0.0385239	0.0549983	0.0825160	0.0262028	0.0511389
YKT6	0.3261337	0.3130855	0.3304545	0.2937378	0.2295158
CAMK2B	0.2971948	0.5072646	0.6210143	0.2300260	0.6079380
NUDCD3	0.2423872	0.3556970	0.4344828	0.2062784	0.2359811
NPC1L1	0.0000000	0.0000000	0.0000000	0.0019173	0.0000000
DDX56	0.1491261	0.1349933	0.1548849	0.1957235	0.1300044
TMED4	0.9886196	0.7194296	0.5638358	1.1871640	0.6327982
ENSG00000287574	0.0076603	0.0102922	0.0077095	0.0079763	0.0170539
OGDH	0.1681674	0.1537710	0.1686124	0.1532901	0.2630820
ZMIZ2	0.0863120	0.1030342	0.1509818	0.0906950	0.2016315
PPIA	2.2587652	2.3791207	2.4919851	2.3052252	2.0070364
H2AZ2	0.9628548	0.9259213	0.8725696	0.9710038	0.8237396
H2AZ2-DT	0.0016531	0.0049961	0.0040443	0.0056496	0.0000000
PURB	0.3480995	0.2551526	0.2430569	0.2908272	0.2488090
ENSG00000272768	0.0045540	0.0000000	0.0012612	0.0029767	0.0000000
MYO1G	0.0216580	0.0066746	0.0099310	0.0167178	0.0123819
SNHG15	0.1862134	0.2994198	0.3469437	0.2135216	0.1992007
CCM2	0.2645177	0.2989830	0.3221054	0.2722798	0.2832097
NACAD	0.1203681	0.1334634	0.0955100	0.0581744	0.1727311
TBRG4	0.1088240	0.1349418	0.1467034	0.1465863	0.0753249
RAMP3	0.0023950	0.0056451	0.0019301	0.0278646	0.0000000
ENSG00000286738	0.0000000	0.0000000	0.0000000	0.0000000	0.0000000
ADCY1	0.1903078	0.3587242	0.3884158	0.1795839	0.3969755
ENSG00000291207	0.2820684	0.3306997	0.3553208	0.2049460	0.2358447
ENSG00000291208	0.0064072	0.0135185	0.0029135	0.0028580	0.0163190
IGFBP3	0.0634395	0.0608858	0.1289386	0.0436842	0.0900537
ENSG00000286315	0.0020245	0.0023755	0.0011445	0.0000000	0.0037236
TNS3	0.3021072	0.2136331	0.1368505	0.2844418	0.2990670
LINC01447	0.0053584	0.0032503	0.0025124	0.0028137	0.0000000
PKD1L1	0.0042838	0.0034621	0.0093677	0.0064965	0.0465856
HUS1	0.1846546	0.1800060	0.1884926	0.1735497	0.1983944
SUN3	0.0019731	0.0000000	0.0000000	0.0000000	0.0000000
C7orf57	0.1940286	0.0897580	0.0512384	0.2064627	0.0638845
UPP1	0.1017638	0.1193320	0.1844193	0.1179071	0.2069801
ABCA13	0.0028295	0.0049823	0.0000000	0.0000000	0.0154598
LINC02838	0.2227179	0.1332665	0.0833356	0.1883230	0.1325538
VWC2	0.0421190	0.0821613	0.1301961	0.0525407	0.1715143
ZPBP	0.0023922	0.0016904	0.0000000	0.0000000	0.0070397
FIGNL1	0.0716608	0.0998267	0.1383544	0.1087400	0.1374898

ENSG00000290114	0.0202239	0.0160023	0.0170887	0.0175341	0.0143476
DDC	0.0748954	0.1302499	0.3304202	0.0611423	0.2477770
GRB10	0.2190258	0.2614953	0.2546617	0.2113964	0.5479087
ENSG00000228204	0.0000000	0.0000000	0.0000000	0.0000000	0.0000000
COBL	0.0821441	0.0686878	0.0810693	0.1017130	0.1047878
ENSG00000285741	0.0055562	0.0034775	0.0020317	0.0013687	0.0095849
VSTM2A	0.1700565	0.3344367	0.4585969	0.1697802	0.3850566
VSTM2A-OT1	0.0246524	0.0409814	0.0460002	0.0385099	0.0504370
SEC61G	1.7943466	1.8311304	1.8423602	1.6746109	1.5077946
SEC61G-DT	0.0227375	0.0272199	0.0170652	0.0113614	0.0248788
EGFR	0.0578133	0.0744845	0.0452803	0.0460459	0.1485998
LANCL2	0.3946927	0.4059724	0.4061075	0.3267642	0.4038098
VOPP1	0.7175462	0.6341651	0.7416515	0.8022720	0.6288296
VOPP1-DT	0.0019964	0.0000000	0.0040654	0.0051947	0.0076833
FKBP9P1	0.0025422	0.0055749	0.0000000	0.0030924	0.0000000
SEPTIN14	0.0000000	0.0000000	0.0000000	0.0000000	0.0000000
ZNF713	0.1338037	0.1884685	0.1985345	0.1177981	0.2886236
NIPSNAP2	0.9897868	0.7738765	0.7955473	1.0251010	0.7163964
MRPS17	0.2960573	0.2827825	0.3257996	0.2882516	0.2191460
PSPH	0.3969463	0.2937822	0.2371068	0.4348026	0.2622998
CCT6A	1.2240868	1.2240204	1.3905590	1.2719988	0.9763824
SUMF2	0.3207799	0.2769620	0.2578346	0.4604116	0.3186618
PHKG1	0.0537183	0.0445372	0.0438233	0.0779247	0.0656403
CHCHD2	0.9929205	0.9496152	0.9159029	0.9838121	0.7549993
ENSG00000291184	0.0000000	0.0000000	0.0000000	0.0022023	0.0000000
ZNF736	0.1436653	0.1511165	0.1685410	0.1848861	0.2051308
ENSG00000287985	0.0035235	0.0058036	0.0097831	0.0067644	0.0000000
ZNF680	0.1855666	0.1778724	0.1500130	0.1769685	0.1570134
ENSG00000286456	0.0156911	0.0211809	0.0144098	0.0122489	0.0137523
ZNF107	0.0608878	0.0665934	0.0421711	0.0430657	0.0492148
ZNF138	0.2444365	0.3550059	0.4341688	0.2083724	0.3612663
ENSG00000287580	0.0006612	0.0000000	0.0026191	0.0000000	0.0027747
ZNF273	0.0734958	0.1126005	0.1549195	0.0702134	0.1015433
ENSG00000189316	0.0022537	0.0049376	0.0170793	0.0015624	0.0181872
ENSG00000286342	0.0268580	0.0350647	0.0531996	0.0051755	0.0078260
ZNF117	0.1593732	0.2062332	0.2485307	0.1260389	0.1908641
ERV3-1	0.0596725	0.0546021	0.0674516	0.0452067	0.0926780
CCT6P3	0.0259206	0.0349833	0.0209966	0.0068977	0.0814545
INTS4P1	0.0158928	0.0196315	0.0084193	0.0114078	0.0475687
ENSG00000289108	0.0031731	0.0045921	0.0052366	0.0023493	0.0015298
ENSG00000282381	0.0000000	0.0012338	0.0000000	0.0000000	0.0047864
ZNF92	0.1997942	0.1827655	0.2080568	0.1360240	0.2519229
LINC03006	0.1268237	0.1243262	0.1095711	0.0770731	0.1665748
ENSG00000290553	0.0048239	0.0057331	0.0081022	0.0015747	0.0073352
ENSG00000291124	0.0461544	0.0607486	0.0635482	0.0398630	0.0616786
VKORC1L1	0.3059765	0.3665792	0.3527008	0.2665573	0.3906633
GUSB	0.1590405	0.0953596	0.0776217	0.2120112	0.0899610
ASL	0.0447492	0.0409059	0.0392044	0.0437736	0.0843322
CRCP	0.3512746	0.3800051	0.3579471	0.3180994	0.3047882
ENSG00000234185	0.0125917	0.0169120	0.0221704	0.0182123	0.0249894
TPST1	0.4209079	0.4017158	0.3787266	0.4632202	0.7663906
LINC00174	0.0492019	0.0552715	0.0452987	0.0279706	0.1973585
LINC03011	0.1849448	0.1827669	0.1464147	0.2180572	0.1168872

ENSG00000291136	0.1869928	0.2683194	0.2318332	0.1796835	0.3429421
KCTD7	0.1360614	0.1811897	0.1965311	0.1088686	0.2170207
ENSG00000226824	0.0748116	0.0869171	0.0978044	0.0671006	0.1332910
ENSG00000289015	0.0111798	0.0159876	0.0025417	0.0119195	0.0189791
RABGEF1	0.3427848	0.3305020	0.3245508	0.3359639	0.5743159
ENSG00000272831	0.3348052	0.2307644	0.1861413	0.3635458	0.1896687
GTF2IRD1P1	0.0084841	0.0110342	0.0045068	0.0060500	0.0337035
ENSG00000289177	0.0053467	0.0028452	0.0090382	0.0000000	0.0206243
LINC02604	0.0045213	0.0000000	0.0040256	0.0038537	0.0097168
TMEM248	0.4737499	0.4056120	0.4256527	0.5636163	0.3416582
SBDS	1.3071088	1.0577597	1.0065159	1.3010067	0.8500247
TYW1	0.1149616	0.1299826	0.0823792	0.1029688	0.2440843
PMS2P4	0.2401982	0.2788644	0.3167599	0.2050016	0.4742032
SPDYE21	0.0000000	0.0009639	0.0000000	0.0000000	0.0108293
STAG3L4	0.3591660	0.4094581	0.3984383	0.2711246	0.3105989
ENSG00000273448	0.0013763	0.0085525	0.0031314	0.0000000	0.0227693
ENSG00000226829	0.0014591	0.0000000	0.0021557	0.0000000	0.0000000
ENSG00000225718	0.0029337	0.0022172	0.0011751	0.0000000	0.0052886
CT66	0.0125622	0.0069358	0.0141089	0.0093302	0.0103395
AUTS2	0.6957953	0.7953517	0.6803743	0.7002869	2.1299053
GALNT17	0.0786665	0.1083257	0.1282125	0.1251382	0.4616325
CALN1	0.1155158	0.1547123	0.1742543	0.0731909	0.7252662
TYW1B	0.0336615	0.0406120	0.0166943	0.0121836	0.1158797
SBDSP1	0.6300254	0.5772400	0.6146025	0.5509009	0.4770978
POM121	0.2377065	0.2789372	0.2771872	0.2098823	0.3546240
ENSG00000289042	0.0018608	0.0067900	0.0036809	0.0000000	0.0018039
ENSG00000272843	0.0006615	0.0026086	0.0027481	0.0000000	0.0059744
NSUN5P2	0.0287116	0.0478716	0.0402554	0.0442591	0.0363969
ENSG00000285886	0.0023896	0.0100907	0.0028040	0.0012986	0.0087666
TRIM74	0.0000000	0.0033326	0.0040732	0.0000000	0.0000000
ENSG00000290832	0.0336671	0.0333071	0.0358249	0.0219339	0.0858163
SPDYE10	0.0015759	0.0058576	0.0013623	0.0011268	0.0109443
NCF1B	0.0000000	0.0011436	0.0000000	0.0000000	0.0000000
ENSG00000290839	0.0024615	0.0046908	0.0107668	0.0050967	0.0000000
NSUN5	0.1398494	0.1776500	0.2174827	0.1084371	0.0936993
FKBP6	0.0000000	0.0000000	0.0000000	0.0000000	0.0000000
FZD9	0.0567740	0.0518024	0.0375674	0.0716184	0.0418871
BAZ1B	0.7713515	0.7730912	0.7405514	0.7802359	0.7436284
BCL7B	0.2361667	0.2051116	0.1743588	0.2727221	0.0889848
TBL2	0.1681405	0.1381869	0.1162869	0.2630548	0.1134639
MLXIPL	0.0051347	0.0100769	0.0037383	0.0000000	0.0671899
ENSG00000274080	0.0011632	0.0000000	0.0000000	0.0000000	0.0138942
VPS37D	0.2192149	0.2169136	0.2180093	0.1843563	0.2190433
DNAJC30	0.1505949	0.1302666	0.1275649	0.1366400	0.0954146
BUD23	0.4305450	0.3887791	0.3875296	0.5364373	0.3658844
STX1A	0.1385933	0.2202353	0.3360740	0.1180791	0.1839764
ABHD11	0.0940466	0.0885112	0.0892326	0.1077101	0.0474868
CLDN3	0.0189226	0.0174817	0.0155840	0.0143196	0.0074956
CLDN4	0.0013863	0.0050147	0.0041370	0.0058178	0.0075008
METTL27	0.0035737	0.0036425	0.0015287	0.0046664	0.0000000
ELN	0.0400192	0.0271944	0.0122038	0.0500031	0.0183263
ELN-AS1	0.0346081	0.0157930	0.0099326	0.0395234	0.0291652
LIMK1	0.1185401	0.1851892	0.2208123	0.1307559	0.1639456

EIF4H	0.8860889	0.7935262	0.8972008	0.8589877	0.7225846
LAT2	0.0009054	0.0085054	0.0029930	0.0000000	0.0218023
RFC2	0.1267955	0.1111933	0.1279229	0.1560530	0.1419539
ENSG00000287815	0.0017558	0.0026990	0.0000000	0.0031240	0.0092794
CLIP2	0.1952406	0.2180899	0.2503603	0.1838247	0.5227708
GTF2IRD1	0.1273524	0.1331005	0.1134909	0.0848745	0.4295961
ENSG00000273069	0.0000000	0.0000000	0.0020099	0.0000000	0.0000000
GTF2I	0.6987851	0.6337917	0.7302287	0.6918387	0.8674179
GTF2I-AS1	0.0079976	0.0096127	0.0028492	0.0000000	0.0512955
NCF1	0.0004456	0.0008641	0.0023834	0.0000000	0.0000000
GTF2IRD2	0.0885536	0.0768364	0.0583388	0.1344120	0.0982566
ENSG00000290833	0.0605572	0.0782396	0.0941437	0.0599950	0.1886111
SPDYE12	0.0000000	0.0000000	0.0015360	0.0000000	0.0020430
CASTOR2	0.2283603	0.3142541	0.3396854	0.2082726	0.3771569
RCC1L	0.2297697	0.1551199	0.1358492	0.2400368	0.1455629
GTF2IRD2B	0.0798315	0.0427045	0.0427017	0.0831735	0.0774259
GTF2IP1	0.2927488	0.4194812	0.4817646	0.2286425	0.3083097
ENSG00000277675	0.0000000	0.0000000	0.0000000	0.0037133	0.0041350
ENSG00000290951	0.0068675	0.0105582	0.0233588	0.0140973	0.0349263
ENSG00000290834	0.0000000	0.0000000	0.0000000	0.0000000	0.0000000
TRIM73	0.0023297	0.0082056	0.0064640	0.0053455	0.0155911
NSUN5P1	0.0354408	0.0499645	0.0431255	0.0490635	0.0832844
POM121C	0.2046137	0.2272589	0.2750934	0.2310395	0.3065273
SPDYE5	0.0012566	0.0000000	0.0019283	0.0000000	0.0098364
PMS2P3	0.0127021	0.0298901	0.0212349	0.0151887	0.0382067
HIP1	0.2797343	0.5014023	0.5149506	0.2470098	0.5776031
RHBDD2	0.5950142	0.4791765	0.5075669	0.6438148	0.4669617
POR	0.2417614	0.2270451	0.2871689	0.3070887	0.2838663
TMEM120A	0.2587676	0.2936845	0.3243615	0.3445010	0.2249555
STYXL1	0.3132373	0.2509420	0.2639468	0.2715693	0.1924382
MDH2	1.0391830	1.1911867	1.2941350	1.0755411	0.8913473
ENSG00000290729	0.0000000	0.0000000	0.0000000	0.0000000	0.0068978
SRRM3	0.1407104	0.2342402	0.2405695	0.0637876	0.6419918
HSPB1	1.8030267	1.6194020	1.4191808	1.8105404	1.4071048
ENSG00000289059	0.0000000	0.0010959	0.0000000	0.0078380	0.0080806
YWHAG	1.2582551	1.7493833	2.0031697	1.1344702	1.5092509
SSC4D	0.0196874	0.0248428	0.0128042	0.0302572	0.0109511
ZP3	0.0145795	0.0221375	0.0181617	0.0178671	0.0161402
DTX2	0.0392249	0.0343473	0.0274142	0.0124953	0.0287730
UPK3B	0.0000000	0.0000000	0.0000000	0.0000000	0.0041830
SPDYE16	0.0012772	0.0000000	0.0000000	0.0000000	0.0000000
LINC03009	0.1050860	0.0849844	0.1035300	0.0862484	0.4559473
POMZP3	0.0969725	0.1207598	0.1157314	0.0993560	0.0988260
SPDYE18	0.0000000	0.0042139	0.0000000	0.0044916	0.0000000
ENSG00000291121	0.0113793	0.0169322	0.0243679	0.0157372	0.0078597
CCDC146	0.7468963	0.4080542	0.2736708	0.7386963	0.4272154
FGL2	0.1024680	0.0436139	0.0173344	0.1679121	0.0245474
ENSG00000250990	0.0000000	0.0014613	0.0023834	0.0000000	0.0000000
GSAP	0.0301521	0.0418619	0.0379106	0.0216295	0.0706800
ENSG00000287519	0.0014314	0.0000000	0.0000000	0.0069384	0.0063227
PTPN12	0.3596266	0.3973656	0.3036165	0.3337293	0.5623292
APTR	0.1661918	0.1594301	0.2306693	0.1348061	0.1854861
RSBN1L	0.4065995	0.4525720	0.5464555	0.3748389	0.4841495

TMEM60	0.2208660	0.1977924	0.1757716	0.2997755	0.1607399
PHTF2	0.1904013	0.2094851	0.2176572	0.1668857	0.3413277
DDX3ILA1	0.0000000	0.0010546	0.0000000	0.0000000	0.0035909
MAGI2	0.7326164	0.7404078	0.5985231	0.6603560	1.7432362
ENSG00000285892	0.0000000	0.0011627	0.0000000	0.0000000	0.0084519
ENSG00000281120	0.0000000	0.0061447	0.0000000	0.0030534	0.0262970
ENSG00000286855	0.0030629	0.0011001	0.0000000	0.0000000	0.0044554
MAGI2-AS3	0.4982292	0.4142682	0.3607169	0.5199539	0.4042740
GNAI1	0.9920105	0.9423339	0.9912062	1.0496971	0.8482037
ENSG00000234223	0.0000000	0.0000000	0.0000000	0.0000000	0.0000000
ENSG00000232667	0.0008624	0.0005159	0.0024688	0.0000000	0.0000000
CD36	1.2386359	0.8253559	0.5722690	1.8914943	0.8811543
SEMA3C	0.2330892	0.3356821	0.1853462	0.3063084	0.4480612
HGF	0.0023687	0.0000000	0.0032188	0.0000000	0.0058801
CACNA2D1	0.3965816	0.4624530	0.5700891	0.4128493	1.0518679
CACNA2D1-AS1	0.0000000	0.0000000	0.0000000	0.0000000	0.0042647
PCLO	0.8607737	1.1610763	1.3320719	0.6683755	1.6904661
SEMA3E	0.0220389	0.0097733	0.0100929	0.0235690	0.0421901
SEMA3A	0.1444746	0.2546259	0.2024454	0.1395685	0.4284845
ENSG00000232019	0.0069580	0.0119742	0.0071357	0.0073933	0.0166438
SEMA3D	0.2803404	0.1965608	0.1314730	0.3010119	0.2950062
GRM3	0.0661023	0.1442161	0.0843484	0.0755581	0.2268713
GRM3-AS1	0.0146217	0.0084329	0.0077155	0.0032553	0.0447251
ELAPOR2	0.4209185	0.3487112	0.3685552	0.3924332	0.6290962
ENSG00000261462	0.0034033	0.0014206	0.0031524	0.0000000	0.0141654
DMTF1-AS1	0.0461535	0.0431400	0.0309151	0.0525227	0.0307177
DMTF1	0.3613101	0.3874800	0.3788295	0.3196818	0.5314778
TMEM243	0.1980243	0.1085890	0.0977046	0.1511344	0.1296607
TP53TG1	0.3416673	0.3066434	0.2232471	0.3512890	0.2603178
CROT	0.1889312	0.1305935	0.1089496	0.1928182	0.1667433
ABCB4	0.0082387	0.0059291	0.0000000	0.0000000	0.0065632
ABCB1	0.0244871	0.0286827	0.0233240	0.0230748	0.0717504
RUNDC3B	0.3711446	0.6398233	0.7733004	0.2264591	0.6892448
SLC25A40	0.1363969	0.1616841	0.2234656	0.1524797	0.1703911
DBF4	0.1442928	0.1295213	0.0913315	0.2007110	0.1381684
ADAM22	0.2352386	0.2656372	0.2907870	0.3929904	0.5523115
SRI	1.3112233	1.1786556	1.0858498	1.4053530	0.8306006
SRI-AS1	0.0000000	0.0006684	0.0000000	0.0000000	0.0037366
ENSG00000228113	0.0031577	0.0136662	0.0029736	0.0000000	0.0456384
STEAP4	0.0000000	0.0000000	0.0000000	0.0000000	0.0000000
ENSG00000233420	0.0010718	0.0093401	0.0017318	0.0029457	0.0089067
ZNF804B	0.0511264	0.0775447	0.0715196	0.0341295	0.3230835
ENSG00000227863	0.0022868	0.0000000	0.0008221	0.0015981	0.0186468
STEAP2-AS1	0.0015501	0.0041500	0.0010396	0.0098371	0.0080533
ENSG00000238358	0.0023268	0.0000000	0.0000000	0.0000000	0.0000000
DPY19L2P4	0.0033881	0.0168582	0.0222283	0.0090318	0.0056913
STEAP1	0.0004731	0.0065132	0.0025512	0.0117351	0.0106963
STEAP2	0.0266074	0.0352443	0.0406778	0.0467886	0.0885969
CFAP69	0.1286783	0.1193228	0.0843417	0.0953444	0.2209967
ENSG00000225498	0.0016189	0.0037543	0.0000000	0.0028343	0.0058941
FAM237B	0.0009723	0.0000000	0.0000000	0.0029465	0.0000000
GTPBP10	0.1656360	0.1679311	0.1933828	0.1731140	0.2236334
CLDN12	0.1661974	0.1552639	0.1275516	0.2300135	0.2011886

CDK14	0.4862684	0.4185713	0.4179443	0.4483437	0.6874397
ENSG00000223969	0.0491246	0.0210092	0.0125577	0.0455696	0.0295785
FZD1	0.1111023	0.0530409	0.0509621	0.1725587	0.0372788
LINC02932	0.0009717	0.0038556	0.0012267	0.0000000	0.0000000
ENSG00000235450	0.0108664	0.0289128	0.0144725	0.0000000	0.0634497
ENSG00000223665	0.0000000	0.0000000	0.0000000	0.0000000	0.0033235
MTERF1	0.1307483	0.1455627	0.1478663	0.1462783	0.3356925
ENSG00000287672	0.0027621	0.0000000	0.0031074	0.0000000	0.0070510
AKAP9	1.0179338	1.0818427	1.1779414	0.9567126	1.4758307
CYP51A1	1.1849891	1.1543811	1.3000012	1.5532267	1.1314286
CYP51A1-AS1	0.0140066	0.0145824	0.0078708	0.0193974	0.0402999
LRRD1	0.0098859	0.0144241	0.0099834	0.0069157	0.0634773
KRIT1	0.3755896	0.3085617	0.3105402	0.3772588	0.3357857
ANKIB1	0.9697750	0.8397847	0.8143447	0.8847446	1.0856080
GATAD1	0.5253853	0.3718628	0.2990457	0.5088447	0.3652875
ENSG00000244055	0.0563821	0.0739665	0.0932205	0.0261937	0.2151494
ERVW-1	0.0003465	0.0008192	0.0000000	0.0000000	0.0000000
PEX1	0.2012427	0.1856778	0.1607686	0.1911074	0.2602057
RBM48	0.1573625	0.1246592	0.1078951	0.1659791	0.1376040
FAM133B	0.5030516	0.4430790	0.3819988	0.4372213	0.4538742
CDK6	0.1273035	0.1297089	0.0691289	0.2073261	0.1949300
ENSG00000286742	0.0015324	0.0000000	0.0000000	0.0000000	0.0000000
CDK6-AS1	0.0076605	0.0059109	0.0037432	0.0030326	0.0066309
SAMD9	0.0032273	0.0000000	0.0000000	0.0024401	0.0061020
SAMD9L	0.0438632	0.0247734	0.0063967	0.0509114	0.0418738
HEPACAM2	0.0048873	0.0056772	0.0000000	0.0000000	0.0139009
VPS50	0.1918173	0.1974743	0.2172510	0.1630362	0.3513263
CALCR	0.0042721	0.0055556	0.0114009	0.0030122	0.0179791
GNGT1	0.0113082	0.0097708	0.0035392	0.0026482	0.0028046
TFPI2	0.0061251	0.0166496	0.0118116	0.0232325	0.0000000
GNG11	0.0930185	0.0782753	0.0455028	0.1278252	0.0649488
BET1	0.2724259	0.2417371	0.2120070	0.2749703	0.1622224
BET1-AS1	0.0006545	0.0018521	0.0023350	0.0000000	0.0000000
ENSG00000285090	0.0000000	0.0025875	0.0000000	0.0000000	0.0000000
ENSG00000285964	0.0000000	0.0000000	0.0000000	0.0000000	0.0000000
COL1A2	0.0652904	0.0664575	0.0864600	0.1026430	0.1542004
CASD1	0.5195519	0.6018324	0.7387104	0.4967248	0.6553066
SGCE	0.3722905	0.2599721	0.2552719	0.4515502	0.3825995
PEG10	1.9518065	2.4072074	3.2469186	1.8883505	2.2418888
PPP1R9A	0.6694144	0.7741434	0.8343082	0.6500184	1.0479873
PPP1R9A-AS1	0.0424837	0.0236118	0.0095267	0.0635999	0.0641072
PON1	0.0000000	0.0007191	0.0000000	0.0000000	0.0186601
PON3	0.0000000	0.0000000	0.0000000	0.0028151	0.0000000
PON2	1.1681392	0.7593370	0.5143094	1.6228973	0.7230040
ASB4	0.0000000	0.0035014	0.0000000	0.0000000	0.0033235
PDK4-AS1	0.0140465	0.0142556	0.0038819	0.0108312	0.0120095
PDK4	0.0426201	0.0365801	0.0251342	0.0379545	0.0190408
DYNC1I1	0.1367118	0.2408407	0.3749749	0.1359990	0.4532918
SLC25A13	0.2043856	0.3056833	0.3433882	0.2115930	0.2512923
SEM1	1.2109980	1.1207903	1.0568083	1.2062203	0.9487610
DLX6-AS1	0.0020056	0.0034654	0.0162843	0.0000000	0.0122995
DLX5	0.0005096	0.0014079	0.0058686	0.0000000	0.0000000
SDHAF3	0.2014911	0.2073942	0.2010214	0.2393075	0.2179016

TAC1	0.3547848	0.5480915	0.7922327	0.3097334	0.5717123
ASNS	0.0224763	0.0186325	0.0213260	0.0087680	0.0603438
ENSG00000284707	0.1374280	0.1901258	0.1175112	0.1398961	0.4709184
LMTK2	0.1495897	0.2214376	0.2360946	0.1630029	0.3252820
BHLHA15	0.0023549	0.0020356	0.0000000	0.0000000	0.0000000
TECPR1	0.0635834	0.1080938	0.1009009	0.0585090	0.1067505
BRI3	1.2638364	1.2258798	1.2048110	1.1717150	1.1016951
BAIAP2L1	0.0146100	0.0326336	0.0322313	0.0254852	0.0447341
ENSG00000272950	0.0009498	0.0000000	0.0000000	0.0000000	0.0059248
ENSG00000288889	0.0010695	0.0080689	0.0048140	0.0019740	0.0055061
NPTX2	0.3905764	0.2219823	0.2377027	0.7844619	0.1741713
TMEM130	0.3438948	0.3014415	0.4623381	0.5116508	0.3552965
TRRAP	0.2109412	0.2385591	0.1946198	0.2665246	0.4672776
ENSG00000286305	0.0010616	0.0000000	0.0000000	0.0000000	0.0000000
SMURF1	0.1252646	0.1827605	0.1751003	0.1065863	0.3421184
KPNA7	0.0042833	0.0000000	0.0000000	0.0000000	0.0206075
ENSG00000284523	0.0087744	0.0126335	0.0076374	0.0134550	0.0139841
ARPC1A	0.8361431	0.9054413	0.9855204	0.7942519	0.7109219
ARPC1B	0.0842432	0.0848076	0.0981434	0.0724477	0.1094302
PDAP1	1.0980160	1.1401107	1.2512999	1.0948596	0.9109267
BUD31	0.8206425	0.8336165	0.8558802	0.8537260	0.6396593
PTCD1	0.0434444	0.0398820	0.0368583	0.0421444	0.0594693
CPSF4	0.1877109	0.1307864	0.0943839	0.1657643	0.1642442
ATP5MF	1.2102294	1.3370134	1.4026189	1.3321691	0.9816539
ZNF789	0.0838088	0.1080480	0.0867068	0.0687040	0.1371286
ZNF394	0.0974151	0.0927936	0.0531420	0.0759586	0.0437643
ZKSCAN5	0.0932083	0.0965836	0.0734257	0.0943181	0.0747537
FAM200A	0.1959847	0.1811058	0.1732111	0.2616925	0.1494800
ZNF655	0.2426771	0.2569380	0.2581015	0.2382850	0.3041446
TMEM225B	0.0136720	0.0161136	0.0207854	0.0167570	0.0103998
ZSCAN25	0.0358138	0.0295712	0.0432344	0.0359993	0.0270129
CYP3A5	0.0040668	0.0131227	0.0116860	0.0057854	0.0413895
CYP3A7	0.0008782	0.0000000	0.0000000	0.0000000	0.0000000
CYP3A4	0.0000000	0.0000000	0.0000000	0.0000000	0.0000000
ENSG00000273407	0.0000000	0.0000000	0.0000000	0.0000000	0.0000000
CYP3A43	0.0000000	0.0024361	0.0008115	0.0000000	0.0106438
TRIM4	0.1696443	0.1080385	0.0890434	0.1980836	0.1652449
GJC3	0.0000000	0.0027772	0.0032420	0.0000000	0.0000000
ENSG00000237640	0.0031262	0.0052723	0.0023029	0.0014244	0.0000000
AZGP1	0.0016521	0.0017833	0.0000000	0.0119147	0.0000000
ENSG00000286923	0.0089703	0.0050366	0.0039958	0.0029301	0.0092766
ZKSCAN1	0.8224205	0.6959616	0.6941160	0.7678812	0.7141363
ZSCAN21	0.1530758	0.0990069	0.1157439	0.1400004	0.1536147
ZNF3	0.0963633	0.0795613	0.0873114	0.0775216	0.0896400
COPS6	0.7626427	0.7617638	0.8342153	0.7282511	0.6117201
MCM7	0.1104183	0.1039994	0.0719973	0.1174305	0.0674734
AP4M1	0.0959028	0.0965465	0.0781597	0.1042215	0.0888093
TAF6	0.2984106	0.2759322	0.2903490	0.3040028	0.2213537
ENSG00000242798	0.0042260	0.0052128	0.0028694	0.0039539	0.0032941
CNPY4	0.2332349	0.1507096	0.0986719	0.3947668	0.1553539
MBLAC1	0.0544047	0.0445178	0.0552244	0.0815695	0.0168003
ENSG00000235077	0.0021501	0.0022784	0.0012693	0.0000000	0.0069786
ENSG00000288976	0.0018876	0.0000000	0.0015819	0.0000000	0.0100999

LAMTOR4	0.7776752	0.7275009	0.7553786	0.7457982	0.5404248
TRAPPC14	0.0530465	0.0901602	0.1481462	0.0605842	0.0843829
GAL3ST4	0.0094976	0.0051888	0.0178290	0.0104412	0.0000000
GPC2	0.1038327	0.2085112	0.3391036	0.1134565	0.2388166
STAG3	0.0221095	0.0288755	0.0409303	0.0297981	0.0420172
CASTOR3P	0.2645553	0.3281802	0.4025387	0.2562748	0.5390588
PVRIG	0.0004739	0.0000000	0.0000000	0.0000000	0.0016673
ENSG00000291178	0.2427859	0.2715789	0.2849576	0.3003510	0.3180106
SPDYE3	0.0055706	0.0059154	0.0102128	0.0016731	0.0235059
STAG3L5P	0.0661350	0.0852480	0.0682949	0.0576548	0.2918274
PILRB	0.0597929	0.0792148	0.0757673	0.0653811	0.2013264
PILRA	0.0271497	0.0169226	0.0111661	0.0135101	0.1059452
ENSG00000287631	0.0008713	0.0000000	0.0000000	0.0000000	0.0000000
ENSG00000289691	0.0070757	0.0028948	0.0014528	0.0000000	0.0052465
ZCWPW1	0.1151023	0.0960449	0.0746058	0.1241967	0.1413753
MEPCE	0.0578252	0.0572974	0.0496448	0.0517078	0.0834882
PPP1R35	0.2176035	0.1737777	0.1550054	0.1699658	0.1253180
PPP1R35-AS1	0.0000000	0.0027877	0.0000000	0.0000000	0.0000000
SPACDR	0.0073729	0.0046616	0.0055902	0.0023389	0.0041632
TSC22D4	0.1350980	0.0624187	0.0503904	0.1649718	0.0705911
ENSG00000286938	0.0031168	0.0000000	0.0000000	0.0073018	0.0000000
NYAP1	0.1092542	0.2410047	0.3036469	0.1208900	0.1621710
ENSG00000225807	0.0008419	0.0009785	0.0025124	0.0079385	0.0075606
AGFG2	0.0385010	0.0262303	0.0328456	0.0184649	0.0687660
SAP25	0.0030764	0.0010959	0.0000000	0.0027511	0.0102793
LRCH4	0.0206431	0.0233475	0.0287922	0.0249683	0.0662229
FBXO24	0.0101685	0.0038429	0.0030678	0.0065358	0.0000000
PCOLCE-AS1	0.0048249	0.0071694	0.0187559	0.0000000	0.0000000
PCOLCE	0.0469496	0.0919482	0.0754006	0.0304094	0.0656210
MOSPD3	0.2264006	0.2523246	0.2495988	0.2575265	0.1585131
TFR2	0.0109654	0.0326230	0.0764154	0.0031006	0.0859325
ACTL6B	0.1418281	0.3186061	0.4920475	0.1166197	0.2249305
GNB2	1.5292504	1.4726599	1.5093577	1.5524049	1.1314789
GIGYF1	0.0770912	0.0802389	0.1049923	0.0716977	0.1776806
POP7	0.2697134	0.3226994	0.3697395	0.2443398	0.2194012
EPO	0.0015059	0.0008085	0.0000000	0.0000000	0.0000000
ZAN	0.0000000	0.0000000	0.0000000	0.0000000	0.0034730
EPHB4	0.0180889	0.0074464	0.0102758	0.0155072	0.0202005
SLC12A9	0.0615070	0.0431557	0.0461806	0.0959169	0.0602522
SLC12A9-AS1	0.0002737	0.0038329	0.0015905	0.0065330	0.0000000
TRIP6	0.2056751	0.1069096	0.0512326	0.2627076	0.1278091
SRRT	0.2308666	0.2173189	0.2123374	0.2292411	0.2326243
UFSP1	0.0101022	0.0113043	0.0108523	0.0150737	0.0160847
ACHE	0.1200821	0.2018410	0.2605371	0.1062319	0.1314783
MUC3A	0.0123734	0.0365909	0.0420874	0.0194853	0.0428600
ENSG00000274993	0.0030137	0.0011164	0.0044527	0.0000000	0.0000000
MUC12	0.0107317	0.0199373	0.0231992	0.0087308	0.0159817
MUC12-AS1	0.0012754	0.0102216	0.0155041	0.0000000	0.0000000
TRIM56	0.2089156	0.1228077	0.0733248	0.1166420	0.1055705
SERPINE1	0.0122551	0.0174823	0.0112912	0.0050294	0.0186713
AP1S1	0.5482511	0.6974607	0.8641884	0.6129274	0.5612631
VGF	0.0966813	0.1720992	0.4711494	0.0971147	0.2159009
NAT16	0.0076057	0.0193766	0.0177483	0.0075817	0.0224753

MOGAT3	0.0000000	0.0000000	0.0023036	0.0000000	0.0000000
PLOD3	0.1130341	0.1005236	0.0508174	0.1745933	0.1258763
ZNHIT1	0.9090053	0.8943347	0.8678632	0.9445006	0.6861677
CLDN15	0.0091475	0.0125446	0.0053826	0.0066736	0.0143515
FIS1	0.8679692	0.8435576	0.8600713	0.8332436	0.6402367
EMSLR	0.0152579	0.0130745	0.0141579	0.0052586	0.0147128
IFT22	0.8026280	0.6616380	0.5874192	0.8178582	0.5269902
COL26A1	0.0504397	0.0783752	0.1398615	0.0300676	0.2552473
CUX1	0.6654816	0.5532027	0.5359853	0.6575603	0.8043673
ENSG00000272219	0.0002567	0.0009190	0.0000000	0.0000000	0.0055409
SH2B2	0.0331120	0.0489197	0.0706606	0.0367123	0.0867732
PRKRIP1	0.3802912	0.3571007	0.3679984	0.4008493	0.5167073
ENSG00000290830	0.0520637	0.0285196	0.0268626	0.0235921	0.0242335
ENSG00000239480	0.0066610	0.0031079	0.0008679	0.0083653	0.0000000
ORAI2	0.4681624	0.4255817	0.4144498	0.4500295	0.4528912
ALKBH4	0.1457662	0.1509408	0.1622288	0.1384600	0.1277252
LRWD1	0.1211960	0.1399315	0.1419107	0.1125660	0.0755636
POLR2J	0.7130848	0.6051831	0.5950444	0.7133633	0.4968729
RASA4B	0.0136495	0.0254777	0.0097029	0.0218464	0.0177878
UPK3BL2	0.0111607	0.0129036	0.0041757	0.0093393	0.0130658
SPDYE2	0.0000000	0.0000000	0.0000000	0.0000000	0.0000000
POLR2J3	0.0268789	0.0320379	0.0205878	0.0178580	0.1715467
ENSG00000279168	0.0435049	0.0496117	0.0361854	0.0293112	0.0456894
RASA4	0.0217100	0.0125673	0.0224135	0.0178328	0.0349750
UPK3BL1	0.2548116	0.2576202	0.2638633	0.2741440	0.1772502
POLR2J2	0.0000000	0.0009013	0.0025063	0.0000000	0.0047917
ENSG00000286830	0.0442522	0.0286659	0.0209607	0.0414826	0.0243944
FAM185A	0.0680334	0.0800320	0.1095184	0.0509512	0.1197996
FBXL13	0.1666541	0.1337287	0.1440617	0.0843168	0.2331772
LRRC17	0.0143480	0.0111908	0.0161526	0.0259945	0.0089891
NFE4	0.0000000	0.0022004	0.0000000	0.0000000	0.0000000
ARMC10	0.4703554	0.4396765	0.4890452	0.4546888	0.3455328
NAPEPLD	0.0927595	0.0588012	0.0511214	0.0996522	0.0798431
ENSG00000289956	0.0046527	0.0095019	0.0090370	0.0142519	0.0358265
DPY19L2P2	0.1127670	0.1242799	0.1554921	0.0878840	0.1655718
PMPCB	0.4284887	0.4049174	0.4034048	0.4670544	0.3449369
DNAJC2	0.4676400	0.4077263	0.3932416	0.4051419	0.4378094
PSMC2	0.5381216	0.4253081	0.4201586	0.5557295	0.3673987
SLC26A5	0.0000000	0.0016748	0.0000000	0.0087209	0.0089366
SLC26A5-AS1	0.0000000	0.0073697	0.0000000	0.0000000	0.0000000
RELN	0.0355168	0.0517539	0.0356661	0.0132885	0.1646721
ORC5	0.1222663	0.1250532	0.1192188	0.1217256	0.2186134
LHFPL3	0.1125212	0.1744499	0.1620695	0.0865559	0.5258083
LHFPL3-AS1	0.0000000	0.0023335	0.0000000	0.0000000	0.0146894
LHFPL3-AS2	0.0000000	0.0022891	0.0000000	0.0000000	0.0000000
KMT2E	0.9965810	1.0181038	1.1660332	0.9137609	1.1871157
LINC01004	0.0553194	0.0625284	0.0402663	0.0497066	0.1042194
KMT2E-AS1	0.0211211	0.0238970	0.0071469	0.0306427	0.0263439
ENSG00000272918	0.0089267	0.0159602	0.0069920	0.0031240	0.0076663
SRPK2	0.8483996	0.9488867	0.9492104	0.8725836	0.9050720
PUS7	0.0485089	0.0809926	0.0615212	0.0710719	0.1483279
RINT1	0.1121218	0.1267808	0.1114500	0.1181665	0.1042768
EFCAB10	0.3649769	0.2499130	0.1711611	0.4299979	0.2043062

EFCAB10-AS1	0.0023859	0.0012260	0.0107384	0.0074313	0.0039851
ATXN7L1	0.1323280	0.1522748	0.1186383	0.1248439	0.4374601
CDHR3	1.1466873	0.7728850	0.5176219	1.1053452	0.9563530
SYPL1	0.3966197	0.2353975	0.1420737	0.5800970	0.2431388
NAMPT	0.4539628	0.4100706	0.4547654	0.4009344	0.5133495
ENSG00000273320	0.0178852	0.0126159	0.0216751	0.0000000	0.0082248
ENSG00000243797	0.0029374	0.0026991	0.0000000	0.0000000	0.0041350
ENSG00000286076	0.0006806	0.0000000	0.0000000	0.0032264	0.0000000
CCDC71L	0.0812956	0.1137563	0.1596856	0.0630742	0.0919612
PRKAR2B	0.7450600	1.1995998	1.6471024	0.7305257	0.9488876
PRKAR2B-AS1	0.0019358	0.0029390	0.0015813	0.0039214	0.0322117
HBP1	0.2908602	0.1876814	0.1217823	0.3032650	0.1921519
ENSG00000272072	0.0000000	0.0040174	0.0031038	0.0000000	0.0013712
COG5	0.3004891	0.3120005	0.2419916	0.2930690	0.5504806
GPR22	0.0523334	0.1065527	0.2937569	0.0311367	0.0692829
DUS4L	0.2574773	0.2037147	0.2212326	0.1960880	0.2073514
ENSG00000272854	0.0000000	0.0004931	0.0022998	0.0000000	0.0052273
BCAP29	0.4794156	0.4548906	0.3020418	0.6468782	0.3757213
SLC26A4-AS1	0.0008687	0.0031988	0.0069151	0.0000000	0.0019259
SLC26A4	0.0020890	0.0000000	0.0022147	0.0000000	0.0056253
CBLL1-AS1	0.0427995	0.0395936	0.0439723	0.0379076	0.0116991
CBLL1	0.1959936	0.2010393	0.1856255	0.2229058	0.2010307
SLC26A3	0.2640404	0.2566489	0.2026730	0.2404372	0.2895996
DLD	0.4492774	0.4480687	0.4606076	0.4467637	0.3700491
LAMB1	0.0428802	0.0505527	0.0480230	0.0367940	0.0967334
ENSG00000273055	0.0024864	0.0000000	0.0012547	0.0000000	0.0063791
LAMB4	0.0066074	0.0060789	0.0037058	0.0034548	0.0000000
NRCAM	0.8269913	0.7794586	0.8651579	0.9535189	1.1152098
PNPLA8	0.5978331	0.6970970	0.8177345	0.6676151	0.6555136
THAP5	0.5565634	0.4104250	0.4157370	0.5364971	0.4010685
DNAJB9	0.6110734	0.5022276	0.4099418	0.7057640	0.4502802
ENSG00000225647	0.0000000	0.0071907	0.0009839	0.0066300	0.0000000
ENSG00000229603	0.0000000	0.0000000	0.0000000	0.0000000	0.0000000
ENSG00000234273	0.0000000	0.0000000	0.0000000	0.0000000	0.0122366
ENSG00000226965	0.0081075	0.0275674	0.0129269	0.0047140	0.0118517
IMMP2L	0.2108081	0.2210225	0.2176642	0.2425386	0.6338871
LRRN3	0.1564833	0.1801731	0.2887577	0.1943016	0.2553566
DOCK4	0.2998950	0.3275929	0.3019973	0.2129421	0.9618859
ENSG00000287011	0.0000000	0.0000000	0.0000000	0.0000000	0.0000000
DOCK4-AS1	0.0013014	0.0000000	0.0000000	0.0000000	0.0033319
ZNF277	0.3064035	0.3119135	0.3120034	0.2629483	0.2961853
ZNF277-AS1	0.0110272	0.0131470	0.0035394	0.0012960	0.0135734
IFRD1	0.7376859	0.5672301	0.6183592	0.5727903	0.6406108
LSMEM1	0.0088458	0.0084032	0.0070246	0.0023733	0.0053035
LINC03076	0.0189001	0.0183323	0.0203550	0.0176551	0.0537640
TMEM168	0.0777427	0.0803593	0.1097774	0.0937450	0.1578885
BMT2	0.2561239	0.2093128	0.2860572	0.1957573	0.3466927
GPR85	0.0827783	0.2342397	0.2838557	0.0797605	0.1746421
ENSG00000225457	0.0000000	0.0000000	0.0046816	0.0000000	0.0032929
SMIM30	0.8817851	0.6429679	0.4914243	0.8357147	0.5823696
PPP1R3A	0.0005373	0.0016409	0.0000000	0.0000000	0.0000000
FOXP2	0.8751600	0.5650679	0.4295303	0.7567429	0.5949101
ENSG00000224595	0.0063924	0.0000000	0.0023388	0.0041811	0.0000000

ENSG00000282859	0.0000000	0.0011142	0.0012308	0.0000000	0.0000000
MDFIC	0.2405268	0.1335093	0.0910374	0.3299406	0.1405392
LINC01393	0.0000000	0.0000000	0.0000000	0.0000000	0.0000000
TES	0.0977944	0.1444591	0.1785733	0.0599172	0.1440630
ENSG00000237813	0.0013634	0.0000000	0.0000000	0.0033511	0.0117844
CAV2	0.0484783	0.0510383	0.0431327	0.0693005	0.0202888
CAV1	0.0638078	0.0603988	0.0089856	0.1099524	0.0436361
MET	0.1012960	0.1837307	0.1204671	0.0768270	0.2604379
CAPZA2	0.8132595	0.8200884	0.9274867	0.8832998	0.7864263
ST7-AS1	0.0245703	0.0147249	0.0207282	0.0180700	0.0189808
ST7	0.2288048	0.2471501	0.2463001	0.1830906	0.5128189
ST7-OT4	0.0150499	0.0078986	0.0026631	0.0055328	0.0045046
ENSG00000228368	0.0000000	0.0000000	0.0000000	0.0000000	0.0037844
ST7-AS2	0.0150325	0.0072662	0.0214385	0.0112881	0.0695992
ENSG00000287829	0.0000000	0.0000000	0.0000000	0.0000000	0.0000000
WNT2	0.0005032	0.0025035	0.0015036	0.0000000	0.0044531
CFTR	0.0224666	0.0163802	0.0149005	0.0516940	0.0303255
ENSG00000083622	0.0000000	0.0000000	0.0000000	0.0000000	0.0000000
CTTNBP2	0.2240161	0.2044709	0.1593028	0.1551452	0.4733214
ENSG00000234826	0.0009905	0.0033239	0.0000000	0.0000000	0.0036568
LSM8	0.4704160	0.5003066	0.4974011	0.4610918	0.3913353
ANKRD7	0.0496897	0.0390486	0.0348376	0.0436369	0.2123423
LINC02476	0.0000000	0.0020047	0.0000000	0.0000000	0.0048106
ENSG00000233417	0.0000000	0.0000000	0.0000000	0.0000000	0.0000000
KCND2	0.1448854	0.2052823	0.2668232	0.1194668	0.7787592
TSPAN12	0.0263488	0.0115508	0.0085049	0.0216337	0.0155162
ING3	0.1878954	0.1596988	0.1909896	0.1940241	0.1784032
CPED1	0.0189292	0.0170447	0.0115268	0.0060758	0.0511242
ENSG00000287554	0.0005567	0.0000000	0.0000000	0.0000000	0.0000000
WNT16	0.0104043	0.0030064	0.0066952	0.0248829	0.0000000
FAM3C	0.5509683	0.6418844	0.8067791	0.6666762	0.6067646
PTPRZ1	0.4054234	0.2696634	0.2275899	1.2331947	0.4294423
AASS	0.0662419	0.0274913	0.0327197	0.0711377	0.0796858
ENSG00000234418	0.0003957	0.0067343	0.0021591	0.0050021	0.0000000
FEZF1-AS1	0.0030472	0.0042512	0.0000000	0.0033007	0.0000000
CADPS2	0.2062531	0.1197732	0.1484903	0.2725524	0.2178105
ENSG00000240499	0.0025666	0.0015382	0.0000000	0.0042622	0.0000000
IQUB	0.1365757	0.0780635	0.0436437	0.1131624	0.1708900
ENSG00000232524	0.0099238	0.0130676	0.0036011	0.0203926	0.0000000
NDUFA5	1.1115332	1.2846805	1.4687177	1.1000678	1.0145328
ASB15	0.0000000	0.0013658	0.0000000	0.0000000	0.0000000
ASB15-AS1	0.0003133	0.0000000	0.0000000	0.0000000	0.0000000
LMOD2	0.0000000	0.0010321	0.0000000	0.0000000	0.0205399
WASL	0.8059286	0.7089064	0.7455285	0.8058931	0.6352895
WASL-DT	0.1174653	0.1092256	0.0682351	0.1019096	0.0660386
TMEM229A	0.0072308	0.0083580	0.0102292	0.0000000	0.0000000
ENSG00000242593	0.0583025	0.0815115	0.0534345	0.0564676	0.3116424
ENSG00000243574	0.0000000	0.0000000	0.0006535	0.0000000	0.0000000
GPR37	0.0754663	0.0375049	0.0226602	0.1047552	0.0163035
LINC03043	0.0000000	0.0000000	0.0000000	0.0000000	0.0000000
POT1	0.1639050	0.1115468	0.0973766	0.1417777	0.2309820
POT1-AS1	0.1526397	0.1650895	0.0929612	0.1322580	0.4142034
ENSG00000219445	0.0000000	0.0000000	0.0000000	0.0000000	0.0000000

ENSG00000241921	0.0027594	0.0000000	0.0000000	0.0000000	0.0000000
GRM8	0.0690526	0.1219242	0.2063201	0.0884925	0.4576659
ENSG00000287702	0.0000000	0.0000000	0.0000000	0.0000000	0.0103636
GRM8-AS1	0.0000000	0.0000000	0.0000000	0.0000000	0.0000000
ZNF800	0.2763654	0.3012479	0.2856036	0.2809387	0.3416251
GCC1	0.0575897	0.0515210	0.0457985	0.1085478	0.0404617
ARF5	0.4393917	0.4755203	0.5806062	0.4417090	0.3192057
FSCN3	0.0025399	0.0032259	0.0024533	0.0038132	0.0000000
SND1-DT	0.0003481	0.0000000	0.0000000	0.0039778	0.0000000
SND1	0.5035261	0.4971561	0.3872531	0.4432157	0.6428384
SND1-IT1	0.0000000	0.0013222	0.0000000	0.0000000	0.0110480
LRRC4	0.1353489	0.1084975	0.1065796	0.1180190	0.1248737
ENSG00000292309	0.0049092	0.0153959	0.0087695	0.0128927	0.0987726
ENSG00000289434	0.0000000	0.0000000	0.0000000	0.0000000	0.0071630
LEP	0.0000000	0.0000000	0.0000000	0.0000000	0.0037415
ENSG00000272915	0.0005856	0.0000000	0.0000000	0.0000000	0.0101255
RBM28	0.3030599	0.2924708	0.3090965	0.2316714	0.3627874
PRRT4	0.0308039	0.0591101	0.0732981	0.0134051	0.0917565
IMPDH1	0.1289169	0.1799172	0.2413364	0.1111957	0.1322298
HILPDA	0.3883695	0.3238761	0.2694496	0.3114069	0.3224605
METTL2B	0.2682109	0.3488160	0.4135231	0.2503950	0.2631213
LINC03072	0.1168154	0.1639345	0.1682655	0.0908812	0.1983056
ENSG00000271553	0.0013390	0.0052377	0.0000000	0.0017393	0.0143209
GARIN1A	0.0045789	0.0060877	0.0068079	0.0139500	0.0302047
ENSG00000271344	0.0067396	0.0041200	0.0000000	0.0000000	0.0024821
GARIN1B	0.0026457	0.0022725	0.0018005	0.0280707	0.0180503
CALU	1.1321169	0.7683893	0.5863951	1.6051382	0.7390677
OPN1SW	0.0009164	0.0100414	0.0034784	0.0000000	0.0059207
CCDC136	0.3884763	0.4684029	0.6254680	0.3475375	0.5510470
FLNC	0.4767635	0.2497666	0.1609543	0.4561448	0.2495907
FLNC-AS1	0.0000000	0.0000000	0.0000000	0.0000000	0.0000000
KCP	0.0274591	0.0222377	0.0200245	0.0490443	0.0380689
ATP6V1F	1.2029605	1.3184034	1.4770114	1.0814962	1.0515840
ATP6V1FNB	0.0047603	0.0276424	0.0225681	0.0196957	0.0155074
IRF5	0.0071392	0.0055630	0.0082912	0.0023598	0.0133098
TNPO3	0.3168138	0.3364278	0.3240808	0.2857489	0.3971893
TSPAN33	0.0829064	0.0807345	0.0661731	0.1250195	0.0958721
SMO	0.0902333	0.0531798	0.0426342	0.1118006	0.0440777
AHCYL2	0.1498812	0.1939068	0.1785552	0.1551609	0.4199867
ENSG00000286722	0.0000000	0.0011581	0.0013196	0.0000000	0.0101016
STRIP2	0.0125263	0.0102635	0.0322449	0.0207885	0.0129590
SMKR1	0.0853090	0.0924526	0.1480258	0.1334632	0.0842788
ENSG00000273329	0.0812874	0.0630735	0.0654486	0.0700174	0.0529553
NRF1	0.1094034	0.1343313	0.1028932	0.1041085	0.3363223
ENSG00000288881	0.0011066	0.0020332	0.0000000	0.0000000	0.0048052
ENSG00000242078	0.0000000	0.0000000	0.0000000	0.0000000	0.0000000
UBE2H	1.0289916	1.1118753	1.0863422	1.0109505	1.0862237
UBE2H-DT	0.0022839	0.0007701	0.0036041	0.0000000	0.0181687
ZC3HC1	0.0987810	0.1081044	0.0656207	0.1362248	0.1296315
KLHDC10	0.4430122	0.5712050	0.6514331	0.3541981	0.6183234
LINC03008	0.0087708	0.0038171	0.0101721	0.0083318	0.0057154
TMEM209	0.1692273	0.1053718	0.1021606	0.1954039	0.1365428
ENSG00000240571	0.0052981	0.0007182	0.0008854	0.0000000	0.0092712

SSMEM1	0.0000000	0.0000000	0.0000000	0.0043470	0.0052853
CPA2	0.0016090	0.0049788	0.0042909	0.0000000	0.0039740
CPA4	0.0008382	0.0034421	0.0054604	0.0000000	0.0000000
CPA5	0.0037911	0.0053905	0.0015825	0.0000000	0.0070524
CEP41	0.2705498	0.2436036	0.2635488	0.2848689	0.1766843
ENSG00000259920	0.0015388	0.0043121	0.0000000	0.0023540	0.0077010
MESTIT1	0.0132802	0.0101974	0.0110585	0.0231602	0.0120077
MEST	1.0738794	0.8669905	1.0365535	1.2177203	0.8381959
ENSG00000270823	0.0031840	0.0016444	0.0015898	0.0025316	0.0024177
COPG2	0.3226273	0.4386049	0.6799109	0.2880653	0.6462929
ENSG00000270953	0.1263112	0.2379679	0.4300661	0.0904970	0.3409636
COPG2IT1	0.7153527	1.1429026	1.5956948	0.5245408	1.3231203
TSGA13	0.0015324	0.0000000	0.0000000	0.0000000	0.0000000
LINC-PINT	0.1678220	0.1451643	0.0784926	0.1432069	0.3719867
LINC00513	0.0300165	0.0138035	0.0050220	0.0332876	0.0670586
ENSG00000271204	0.0010832	0.0014276	0.0000000	0.0000000	0.0078889
ENSG00000273319	0.0224584	0.0105877	0.0126080	0.0174348	0.0106916
MKLN1	0.4846805	0.4243069	0.4073000	0.4252427	0.7224067
MKLN1-AS	0.0297976	0.0274285	0.0204531	0.0754635	0.0072450
ENSG00000273489	0.0011330	0.0013242	0.0134230	0.0056315	0.0030021
PODXL	0.1110964	0.0676136	0.0447452	0.1830206	0.1037891
ENSG00000224865	0.0018458	0.0010980	0.0018152	0.0030030	0.0000000
PLXNA4	0.1682951	0.3281842	0.2645484	0.1584062	0.7867455
ENSG00000225144	0.0000000	0.0000000	0.0000000	0.0000000	0.0000000
ENSG00000223436	0.0000000	0.0000000	0.0000000	0.0000000	0.0032853
CHCHD3	0.5738050	0.5583280	0.5458002	0.5503428	0.6675149
ENSG00000227197	0.0024656	0.0014825	0.0000000	0.0000000	0.0035493
EXOC4	0.4836327	0.5002968	0.4158193	0.4426479	0.8904942
LRGUK	0.0459373	0.0583106	0.0215848	0.0518110	0.1254155
SLC35B4	0.2724361	0.2683630	0.2650115	0.3404595	0.2402149
LINC03060	0.0057268	0.0108579	0.0149828	0.0074569	0.0072417
AKR1B1	1.3615449	0.9886510	0.8436101	1.2051316	0.8033769
AKR1B10	0.0126867	0.0041123	0.0080656	0.0094273	0.0000000
BPGM	0.3925886	0.3668674	0.3877139	0.3921431	0.2668723
ENSG00000224375	0.0000000	0.0000000	0.0000000	0.0000000	0.0000000
CALD1	1.3809721	0.9604459	0.7465556	1.3232171	1.0085598
ENSG00000286458	0.0103310	0.0162746	0.0299053	0.0107607	0.0818724
AGBL3	0.0632212	0.0554435	0.0509358	0.0523314	0.1143690
CYREN	0.1879100	0.1155902	0.0858220	0.2072772	0.1259443
TMEM140	0.0155394	0.0029371	0.0100281	0.0250268	0.0123575
ENSG00000287733	0.0297419	0.0516087	0.0547871	0.0515561	0.0183886
WDR91	0.0382169	0.0393337	0.0380628	0.0229805	0.0437415
CNOT4	0.5526507	0.5039552	0.5402459	0.4912080	0.6139719
NUP205	0.0875257	0.1181590	0.1193021	0.1074584	0.1899034
STMP1	1.0943534	1.0531398	1.0284726	1.1180970	0.9003940
SLC13A4	0.0019673	0.0029515	0.0036779	0.0000000	0.0093138
ENSG00000273219	0.0006337	0.0000000	0.0000000	0.0000000	0.0000000
FAM180A	0.0008153	0.0020449	0.0000000	0.0000000	0.0000000
ENSG00000224746	0.0034125	0.0038831	0.0069564	0.0000000	0.0168079
MTPN	1.0818328	1.1948552	1.2627474	1.0553716	0.9840432
ENSG00000232053	0.0058733	0.0056213	0.0000000	0.0000000	0.0047127
ENSG00000234352	0.0111027	0.0118022	0.0270567	0.0085425	0.1294823
CHRM2	0.1183893	0.2149012	0.2332719	0.0885600	0.4751764

PTN	0.6650993	0.5680218	0.5723001	1.4025408	0.6044579
ENSG00000231114	0.0000000	0.0000000	0.0000000	0.0000000	0.0000000
ENSG00000228031	0.0000000	0.0000000	0.0000000	0.0000000	0.0013524
DGKI	0.2215040	0.3165497	0.3728291	0.1352180	0.8259486
ENSG00000289438	0.0026628	0.0239140	0.0282254	0.0000000	0.0227762
CREB3L2	0.1548470	0.0667653	0.0488507	0.1252040	0.1429036
AKR1D1	0.0024538	0.0000000	0.0000000	0.0000000	0.0000000
ENSG00000225559	0.0005494	0.0015096	0.0008981	0.0000000	0.0000000
TRIM24	0.5796992	0.5926229	0.6918706	0.5046924	0.7362178
SVOPL	0.0000000	0.0034406	0.0047222	0.0000000	0.0067404
ATP6V0A4	0.0066247	0.0079071	0.0017966	0.0000000	0.0198895
TMEM213	0.0019341	0.0028559	0.0000000	0.0049554	0.0265675
KIAA1549	0.4425491	0.3818331	0.4260404	0.5349144	0.5650146
ZC3HAV1L	0.0457264	0.0567142	0.0453413	0.0462554	0.0405819
ZC3HAV1	0.1619633	0.1210547	0.0901210	0.1959377	0.0912214
TTC26	0.1560521	0.1225343	0.0617776	0.1515805	0.1437407
UBN2	0.3789218	0.4415946	0.4501807	0.4330603	0.6039749
FMC1	0.5002049	0.4766491	0.4408649	0.4495740	0.3333210
LUC7L2	0.8537233	0.7579320	0.7425481	0.7205447	0.8559714
ENSG00000273391	0.0179473	0.0133602	0.0102320	0.0182699	0.0318389
KLRG2	0.0011613	0.0000000	0.0014111	0.0000000	0.0000000
CLEC2L	0.0957407	0.2011808	0.2707244	0.0997430	0.1763382
HIPK2	0.9929930	0.9505539	1.0151422	0.8187161	1.0649757
TBXAS1	0.0116448	0.0135073	0.0214025	0.0085890	0.0343018
PARP12	0.0053917	0.0033393	0.0041156	0.0052976	0.0000000
KDM7A	0.1230035	0.1703176	0.1531144	0.0961509	0.3596602
KDM7A-DT	0.0627646	0.0585053	0.0422038	0.0281541	0.0101028
SLC37A3	0.0945263	0.0861550	0.0814631	0.0891351	0.1627126
RAB19	0.0000000	0.0000000	0.0058759	0.0000000	0.0000000
MKRN1	0.6006205	0.6260898	0.7223753	0.5298658	0.4822589
DENND2A	0.3653583	0.3778795	0.2492328	0.3967889	0.4703872
ENSG00000285904	0.0156375	0.0102451	0.0134984	0.0178383	0.0087140
ADCK2	0.1411245	0.1899382	0.2384091	0.1168763	0.1529606
NDUFB2	1.2691925	1.4204321	1.4018530	1.2762210	1.0644933
NDUFB2-AS1	0.0170037	0.0167865	0.0195858	0.0065403	0.0248758
BRAF	0.3877706	0.4910923	0.4852058	0.3017749	0.7696292
ENSG00000289788	0.0055586	0.0000000	0.0029005	0.0000000	0.0297533
MRPS33	0.7991446	0.7169352	0.7839439	0.7125267	0.5244194
TMEM178B	0.3688040	0.6076321	0.6071273	0.3514088	1.2450488
ENSG00000285841	0.0000000	0.0000000	0.0015575	0.0000000	0.0136659
AGK-DT	0.0048529	0.0047027	0.0030806	0.0111419	0.0115979
ENSG00000261797	0.0020549	0.0006514	0.0026409	0.0000000	0.0028551
AGK	0.1614256	0.1981789	0.2544364	0.1677353	0.2110812
ENSG00000244701	0.0015624	0.0076497	0.0052926	0.0048157	0.0171919
DENND11	0.1277829	0.0763283	0.0489510	0.1275893	0.1567412
ENSG00000270157	0.0030124	0.0032514	0.0014826	0.0049836	0.0037043
WEE2-AS1	0.0322178	0.0212643	0.0141947	0.0444486	0.0293516
WEE2	0.0042666	0.0022732	0.0063518	0.0000000	0.0089901
SSBP1	0.8611321	0.8567360	0.9474588	0.8404319	0.7423300
TAS2R4	0.0037420	0.0102792	0.0024677	0.0011268	0.0066633
TAS2R5	0.0007640	0.0012926	0.0009177	0.0000000	0.0000000
MGAM	0.0072490	0.0085380	0.0080766	0.0143260	0.0210380
OR9A4	0.0008897	0.0000000	0.0000000	0.0000000	0.0050816

CLEC5A	0.0000000	0.0000000	0.0000000	0.0000000	0.0000000
MGAM2	0.0000000	0.0014479	0.0000000	0.0000000	0.0000000
ENSG00000289938	0.0000000	0.0000000	0.0000000	0.0000000	0.0000000
EPHB6	0.1330422	0.1998582	0.2092505	0.0844373	0.0992504
TRPV6	0.0000000	0.0000000	0.0000000	0.0000000	0.0000000
TRPV5	0.0000000	0.0000000	0.0000000	0.0000000	0.0000000
KEL	0.0018711	0.0037525	0.0000000	0.0059372	0.0098838
ENSG00000268170	0.0000000	0.0000000	0.0000000	0.0000000	0.0000000
GSTK1	0.3092778	0.2208093	0.2008684	0.3319747	0.2092577
TMEM139-AS1	0.0213599	0.0204491	0.0248362	0.0443726	0.0384834
TMEM139	0.0013004	0.0036594	0.0084724	0.0068539	0.0000000
CASP2	0.0983819	0.0945105	0.0673072	0.0993413	0.1126712
CLCN1	0.0017863	0.0000000	0.0025047	0.0000000	0.0000000
FAM131B	0.0526275	0.1069983	0.0936084	0.0218276	0.0772191
FAM131B-AS1	0.0314125	0.0409919	0.0684696	0.0180315	0.0034957
FAM131B-AS2	0.1629385	0.1670164	0.1605612	0.2107182	0.1198449
ZYX	0.3982298	0.3242226	0.2715559	0.3707026	0.2753329
EPHA1	0.0000000	0.0013292	0.0000000	0.0000000	0.0042185
EPHA1-AS1	0.0080819	0.0105755	0.0114615	0.0019281	0.0205816
TCAF2	0.0180306	0.0141747	0.0087898	0.0090634	0.0176962
ENSG00000291149	0.0265872	0.0315303	0.0249673	0.0145229	0.0536707
TCAF1	0.6716691	0.8154833	0.8952122	0.5798734	0.7868870
OR2F1	0.0016449	0.0006888	0.0000000	0.0000000	0.0000000
ARHGEF35	0.0013293	0.0023690	0.0000000	0.0000000	0.0000000
ARHGEF35-AS1	0.0055249	0.0028969	0.0000000	0.0050516	0.0059265
TPK1	0.0524520	0.0693538	0.0712615	0.0502221	0.1545323
CNTNAP2	0.8262439	1.0939381	1.1896410	0.6862390	2.3676798
CNTNAP2-AS1	0.0016449	0.0000000	0.0000000	0.0000000	0.0000000
ENSG00000230190	0.0000000	0.0000000	0.0000000	0.0000000	0.0059144
ENSG00000287636	0.0023454	0.0003476	0.0000000	0.0000000	0.0000000
ENSG00000273314	0.0752397	0.0471614	0.0673685	0.0505611	0.0908145
CUL1	0.4310255	0.4566269	0.4882917	0.5101290	0.5205773
EZH2	0.1046862	0.1035812	0.0549417	0.1051429	0.1818887
GHET1	0.0073973	0.0065634	0.0048477	0.0035133	0.0066113
PDIA4	0.8293720	0.5686688	0.4873201	1.3331539	0.5340669
ZNF786	0.0462248	0.0724172	0.0796872	0.0315033	0.0773791
ZNF425	0.0841414	0.0742668	0.0751740	0.0675996	0.0821914
ZNF398	0.0909942	0.1002252	0.1061318	0.0590321	0.1323158
ZNF282	0.0575390	0.0600218	0.0394580	0.0545319	0.0851980
ZNF212	0.0407677	0.0291467	0.0208210	0.0334390	0.0486920
ZNF783	0.0323560	0.0356305	0.0244740	0.0364375	0.0551240
ENSG00000290600	0.0000000	0.0022927	0.0036144	0.0000000	0.0000000
ENSG00000228151	0.0000000	0.0000000	0.0000000	0.0000000	0.0000000
ENSG00000261842	0.0000000	0.0000000	0.0000000	0.0000000	0.0156863
ZNF777	0.0708420	0.0815478	0.0715104	0.0722646	0.0760794
ZNF746	0.0752983	0.1073578	0.0928245	0.0937136	0.0469425
ZNF767P	0.0362524	0.0536919	0.0367937	0.0310146	0.1742884
KRBA1	0.0291682	0.0181575	0.0161497	0.0286912	0.0208911
ZNF467	0.0802749	0.0642965	0.0323685	0.0958420	0.0456811
ZNF862	0.1202697	0.1289604	0.1375561	0.1190958	0.1809552
ENSG00000273419	0.0000000	0.0014335	0.0028723	0.0030232	0.0043097
ATP6V0E2-AS1	0.0345913	0.0281811	0.0350466	0.0210109	0.0333697
ATP6V0E2	0.6530395	0.7941303	0.9605038	0.7726197	0.5971918

ENSG00000273011	0.0129836	0.0068610	0.0008911	0.0111797	0.0204631
ENSG00000241449	0.0000000	0.0011406	0.0000000	0.0000000	0.0138220
ACTR3C	0.0009003	0.0024251	0.0010720	0.0000000	0.0000000
LRRC61	0.0729363	0.0952631	0.1252618	0.0623422	0.0614547
RARRES2	0.0228685	0.0355013	0.0111478	0.0370354	0.0156931
REPIN1-AS1	0.0028377	0.0017383	0.0045563	0.0000000	0.0056738
REPIN1	0.2539859	0.2001103	0.1823768	0.2430821	0.1728860
ZNF775	0.0933487	0.0870115	0.0988446	0.0974277	0.1069574
ENSG00000284691	0.0442435	0.0373876	0.0564934	0.0348642	0.0475178
LINC00996	0.0000000	0.0000000	0.0000000	0.0000000	0.0000000
GIMAP8	0.0016068	0.0000000	0.0000000	0.0000000	0.0000000
GIMAP2	0.0129947	0.0057621	0.0055738	0.0181143	0.0073391
GIMAP1	0.0031511	0.0037061	0.0000000	0.0140624	0.0000000
TMEM176B	0.0536883	0.0701579	0.0793879	0.0461052	0.0537481
TMEM176A	0.0506688	0.0715637	0.0360853	0.0196445	0.0287044
ENSG00000289052	0.0008285	0.0022836	0.0011124	0.0082691	0.0000000
KCNH2	0.2444032	0.2350330	0.1850829	0.2336598	0.1986201
NOS3	0.0000000	0.0008286	0.0000000	0.0018030	0.0000000
ATG9B	0.0353821	0.0511902	0.0836080	0.0514627	0.0320013
ABCB8	0.0683019	0.0659097	0.0828507	0.0764451	0.1242492
ENSG00000243433	0.0014277	0.0000000	0.0000000	0.0000000	0.0000000
ASIC3	0.0055211	0.0046188	0.0042401	0.0021110	0.0080071
CDK5	0.1969247	0.2879001	0.4259689	0.2073363	0.2096759
SLC4A2	0.0959203	0.0671375	0.0500305	0.1184298	0.0538964
FASTK	0.2451845	0.2490614	0.2250045	0.2402792	0.1724807
TMUB1	0.2899170	0.2873067	0.2343729	0.3306722	0.2813124
AGAP3	0.1737698	0.2539499	0.2555858	0.1458174	0.2505481
GBX1	0.0009289	0.0000000	0.0000000	0.0000000	0.0071731
IQCA1L	0.0009884	0.0000000	0.0000000	0.0000000	0.0000000
ABCF2	0.1802009	0.1517254	0.1622922	0.2332311	0.1061505
CHPF2	0.0489346	0.0252366	0.0318952	0.0893751	0.0612755
SMARCD3	0.3075207	0.5076361	0.5443237	0.3129310	0.3452335
ENSG00000243018	0.0000000	0.0000000	0.0022795	0.0000000	0.0000000
NUB1	0.6432638	0.5039700	0.5221925	0.6074215	0.4008654
WDR86	0.0457237	0.1092801	0.0787913	0.0373442	0.1519228
WDR86-AS1	0.0031540	0.0032729	0.0019328	0.0000000	0.0000000
RHEB	1.1557566	1.1720205	1.1713129	1.1390784	0.9565204
PRKAG2	0.1920482	0.2215244	0.1937342	0.1998293	0.2840547
ENSG00000290095	0.0000000	0.0000000	0.0000000	0.0000000	0.0000000
PRKAG2-AS1	0.0976416	0.2103886	0.2083771	0.0822645	0.1314780
GALNTL5	0.0000000	0.0000000	0.0000000	0.0000000	0.0000000
GALNT11	0.5714309	0.6194350	0.6626211	0.6027897	0.6661274
ENSG00000229591	0.0000000	0.0000000	0.0020642	0.0000000	0.0044508
KMT2C	0.5878974	0.5855730	0.5526364	0.5594123	1.1650211
LINC01003	0.1825303	0.1894743	0.2328472	0.2104988	0.1199834
XRCC2	0.0064034	0.0065789	0.0059169	0.0059618	0.0181035
ENSG00000289146	0.0115546	0.0172008	0.0153789	0.0037594	0.0185568
ACTR3B	0.0903440	0.1357696	0.1923931	0.0727556	0.2167099
ENSG00000286565	0.0009918	0.0000000	0.0015113	0.0000000	0.0000000
DPP6	0.7155900	0.6860511	0.7914128	0.8717557	1.6879027
ENSG00000236408	0.0000000	0.0003277	0.0000000	0.0000000	0.0000000
PAXIP1-AS2	0.0045395	0.0000000	0.0000000	0.0039642	0.0023981
PAXIP1	0.0612099	0.0634350	0.0374866	0.0667934	0.1127859

PAXIP1-DT	0.1226427	0.1684084	0.1494223	0.1405380	0.0779049
HTR5A-AS1	0.0245119	0.0808903	0.1070662	0.0307365	0.0736882
HTR5A	0.0321569	0.0725425	0.1678663	0.0300072	0.0441934
INSIG1-DT	0.0271609	0.0571968	0.0602597	0.0174834	0.0301387
INSIG1	0.2659185	0.3016065	0.4269079	0.3503051	0.3021073
LINC03010	0.0038109	0.0043847	0.0000000	0.0056894	0.0097019
EN2	0.0019923	0.0022907	0.0009088	0.0042469	0.0046716
ENSG00000283128	0.0129850	0.0078452	0.0126581	0.0032517	0.0607948
ENSG00000227365	0.0000000	0.0018074	0.0000000	0.0025505	0.0000000
RBM33-DT	0.0164320	0.0192721	0.0122218	0.0206012	0.0155439
RBM33	0.3369268	0.3298597	0.2775439	0.3571338	0.5929373
SHH	0.5414929	0.3516451	0.2352519	0.7225041	0.3332347
RNF32-DT	0.1625844	0.1731498	0.1319700	0.1231381	0.1719990
RNF32	0.1952570	0.1519386	0.1351341	0.1479899	0.1769013
RNF32-AS1	0.0039885	0.0029427	0.0031385	0.0028850	0.0042817
LMBR1	0.2360329	0.3133822	0.2841150	0.2285424	0.4974432
NOM1	0.1321205	0.1358932	0.1447664	0.1052196	0.1981964
MNX1	0.0462731	0.0920755	0.0202189	0.0288269	0.0792396
UBE3C	0.2460662	0.3050774	0.2189921	0.1688484	0.5086651
DNAJB6	1.0307097	1.0823889	1.2567841	0.9226863	0.8534200
ENSG00000234210	0.0011418	0.0003466	0.0011178	0.0082034	0.0014182
PTPRN2	0.4250734	0.4611594	0.4749009	0.3771737	1.5404730
ENSG00000222012	0.0016318	0.0009338	0.0000000	0.0000000	0.0033561
ENSG00000231515	0.0000000	0.0000000	0.0000000	0.0000000	0.0000000
ENSG00000289418	0.0000000	0.0014762	0.0000000	0.0000000	0.0128585
NCAPG2	0.0609660	0.0871951	0.0609578	0.0656796	0.0952970
ESYT2	0.2873108	0.2305499	0.2495894	0.2791872	0.5358792
DYNC2I1	0.6331440	0.5466495	0.3842221	0.6276644	1.0213461
VIPR2	0.0037066	0.0007328	0.0000000	0.0037810	0.0043932
ZNF596	0.0674156	0.0604610	0.0469928	0.0531460	0.1002881
ENSG00000272812	0.0028334	0.0045654	0.0049197	0.0000000	0.0035158
ENSG00000273402	0.0012898	0.0022753	0.0000000	0.0000000	0.0000000
ENSG00000249868	0.0000000	0.0019528	0.0000000	0.0000000	0.0000000
FBXO25	0.2574619	0.3231669	0.3897723	0.2440442	0.2922114
ENSG00000272293	0.0015360	0.0000000	0.0042522	0.0000000	0.0124338
TDRP	0.1685889	0.2467042	0.2032525	0.1807327	0.2182088
ERICH1	0.2207877	0.3019200	0.2638603	0.1800248	0.3945542
DLGAP2	0.2824096	0.3011121	0.2962641	0.2650183	1.3583104
ENSG00000282375	0.0010438	0.0137641	0.0000000	0.0000000	0.0044810
ENSG00000282692	0.0000000	0.0000000	0.0000000	0.0000000	0.0027595
DLGAP2-AS1	0.0018519	0.0000000	0.0000000	0.0000000	0.0018550
CLN8	0.1243956	0.1251481	0.1183530	0.1809223	0.1283290
ENSG00000282021	0.0000000	0.0020692	0.0000000	0.0049851	0.0053985
CLN8-AS1	0.4803095	0.3459717	0.2791971	0.4830666	0.3242021
ARHGEF10	0.2504789	0.1509595	0.1155342	0.2405326	0.2365904
ENSG00000285957	0.0000000	0.0000000	0.0000000	0.0000000	0.0000000
KBTBD11-AS1	0.0551400	0.0347427	0.0169364	0.0470748	0.0264565
KBTBD11-OT1	0.0088751	0.0038670	0.0027414	0.0035952	0.0308141
KBTBD11	0.2335465	0.2828189	0.3329555	0.1395465	0.2435584
ENSG00000270988	0.0015694	0.0000000	0.0024311	0.0000000	0.0000000
ENSG00000289473	0.0000000	0.0019818	0.0000000	0.0000000	0.0067614
MYOM2	0.0444657	0.0515220	0.0846366	0.0305467	0.0619936
ENSG00000287970	0.0000000	0.0000000	0.0000000	0.0000000	0.0000000

ENSG00000253444	0.0000000	0.0024232	0.0000000	0.0000000	0.0000000
ENSG00000282142	0.0036204	0.0181835	0.0115481	0.0076811	0.0265792
LINC03021	0.0035599	0.0033423	0.0102390	0.0047159	0.0148470
LINC03021.1	0.0086089	0.0095761	0.0096902	0.0000000	0.0601511
ENSG00000253853	0.0035141	0.0088846	0.0037758	0.0038847	0.0123290
CSMD1	0.7796662	0.9098863	0.6712509	0.6950365	2.3569357
ENSG00000285861	0.0006287	0.0000000	0.0000000	0.0000000	0.0014252
ENSG00000286934	0.0012783	0.0013272	0.0000000	0.0016222	0.0132566
ENSG00000288110	0.0000000	0.0008916	0.0000000	0.0000000	0.0000000
ENSG00000285977	0.0000000	0.0000000	0.0000000	0.0000000	0.0022413
ENSG00000253880	0.0042284	0.0038463	0.0023700	0.0000000	0.0202934
MCPH1-DT	0.0984944	0.0816151	0.1043229	0.1027544	0.0590162
MCPH1	0.3309564	0.3332976	0.3385551	0.2912549	0.4279892
ANGPT2	0.0194875	0.0105865	0.0048994	0.0159479	0.0631555
ENSG00000271743	0.0045334	0.0075943	0.0046786	0.0047341	0.0014245
MCPH1-AS1	0.1220865	0.1531230	0.1610785	0.0896390	0.1977992
AGPAT5	0.7068442	0.6139052	0.6033538	0.8684494	0.6701363
XKR5	0.0033517	0.0033989	0.0103677	0.0000000	0.0051924
GS1-24F4.2	0.0259762	0.0253385	0.0085533	0.0280959	0.0166850
DEFB1	0.0045275	0.0027557	0.0027135	0.0032979	0.0000000
DEFA5	0.0000000	0.0015824	0.0013959	0.0000000	0.0067193
ENSG00000290357	0.0344671	0.0181285	0.0326820	0.0140667	0.0676658
FAM66B	0.0232327	0.0329243	0.0386802	0.0394663	0.0246943
DEFB109B	0.0000000	0.0000000	0.0000000	0.0000000	0.0077388
FAM66E	0.0039360	0.0016346	0.0046594	0.0000000	0.0000000
FAM85B	0.0193149	0.0067548	0.0121278	0.0023675	0.0596528
FAM86B3P	0.0073006	0.0170232	0.0088670	0.0376740	0.0265726
ENSG00000291048	0.0000000	0.0000000	0.0000000	0.0000000	0.0000000
PRAG1	0.1634391	0.1006920	0.0980800	0.2296286	0.1510278
CLDN23	0.0026085	0.0022883	0.0022845	0.0000000	0.0070919
ENSG00000254367	0.0020144	0.0000000	0.0000000	0.0000000	0.0000000
MFHAS1	0.1268433	0.1654621	0.1945551	0.1173451	0.2280895
ERI1	0.1249094	0.1100862	0.0541634	0.1042919	0.0869962
PPP1R3B	0.0958351	0.0898225	0.0516446	0.0800254	0.0605158
ENSG00000254340	0.0000000	0.0010000	0.0007884	0.0000000	0.0000000
PPP1R3B-DT	0.0046917	0.0031277	0.0020861	0.0000000	0.0014580
ENSG00000253735	0.0000000	0.0000000	0.0000000	0.0000000	0.0000000
ENSG00000254237	0.0000000	0.0000000	0.0000000	0.0000000	0.0000000
TNKS	0.6461457	0.7810856	0.7586043	0.6169846	1.0413803
MIR124-1HG	0.0172631	0.0181939	0.0204785	0.0050417	0.0708780
ENSG00000286622	0.0000000	0.0017571	0.0000000	0.0012236	0.0072246
MSRA-DT	0.0009392	0.0010333	0.0021479	0.0000000	0.0116253
MSRA	0.0905368	0.0762958	0.0638349	0.1078385	0.1179394
ENSG00000285675	0.0000000	0.0000000	0.0000000	0.0000000	0.0000000
ENSG00000261451	0.0000000	0.0016518	0.0000000	0.0000000	0.0000000
LINC03022	0.0000000	0.0009623	0.0000000	0.0025788	0.0099607
PRSS51	0.0000000	0.0000000	0.0000000	0.0000000	0.0148142
ENSG00000272505	0.0000000	0.0000000	0.0000000	0.0000000	0.0000000
PRSS55	0.0000000	0.0000000	0.0000000	0.0000000	0.0036428
RP1L1	0.0000000	0.0034866	0.0000000	0.0000000	0.0000000
SOX7	0.0010377	0.0049160	0.0023139	0.0000000	0.0000000
ENSG00000248896	0.0000000	0.0000000	0.0000000	0.0000000	0.0044415
PINX1	0.1219483	0.1263586	0.1059376	0.1147700	0.1722665

PINX1-DT	0.0148052	0.0000000	0.0090675	0.0067323	0.0123599
XKR6	0.3202009	0.4136169	0.4095894	0.3257317	1.1433132
ENSG00000254839	0.0011118	0.0011346	0.0000000	0.0035761	0.0000000
ENSG00000269918	0.0207053	0.0310391	0.0400424	0.0286174	0.0483807
ENSG00000255310	0.0100411	0.0113276	0.0115703	0.0025926	0.0043708
ENSG00000254936	0.0039949	0.0082501	0.0000000	0.0043997	0.0248489
ENSG00000254556	0.0027418	0.0036921	0.0039714	0.0000000	0.0168316
LINC00529	0.0000000	0.0000000	0.0000000	0.0000000	0.0000000
ENSG00000280273	0.0124427	0.0104279	0.0120198	0.0029154	0.0114695
MTMR9	0.2673549	0.4014400	0.4230618	0.2735560	0.3440623
ENSG00000246477	0.0000000	0.0000000	0.0014407	0.0000000	0.0000000
SLC35G5	0.0029253	0.0037169	0.0031340	0.0000000	0.0052756
TDH-AS1	0.0284366	0.0163254	0.0055328	0.0133548	0.0000000
FAM167A-AS1	0.0028537	0.0000000	0.0000000	0.0070487	0.0092339
FAM167A	0.0431876	0.0501650	0.0455307	0.0508642	0.0794120
ENSG00000269954	0.0010553	0.0038471	0.0007799	0.0000000	0.0099551
ENSG00000286985	0.0014637	0.0043864	0.0000000	0.0069427	0.0000000
NEIL2	0.2305553	0.2825180	0.2627036	0.2726282	0.1725399
FDFT1	0.6276813	0.6318721	0.6867794	0.7390879	0.5486510
ENSG00000255046	0.0081094	0.0074152	0.0021512	0.0169810	0.0327292
CTSB	0.7680260	0.6059464	0.4420705	1.0012131	0.5035995
ENSG00000287798	0.0000000	0.0000000	0.0000000	0.0000000	0.0000000
DEFB134	0.0007786	0.0000000	0.0000000	0.0000000	0.0000000
ENSG00000290829	0.0285326	0.0780943	0.0882366	0.0311367	0.0771371
FAM66D	0.0690770	0.0803861	0.1138849	0.0674226	0.1296711
FAM86B1	0.0093492	0.0064824	0.0015338	0.0138334	0.0169796
ENSG00000255495	0.1261193	0.1517445	0.1530187	0.1462281	0.1288592
FAM66A	0.0269385	0.0227397	0.0171736	0.0166315	0.0564778
ENSG00000270074	0.0026975	0.0018551	0.0024665	0.0036397	0.0000000
FAM86B2	0.0048471	0.0017752	0.0036637	0.0040746	0.0071273
ENSG00000283674	0.1077450	0.1193855	0.1640836	0.1170135	0.4043406
ENSG00000270154	0.0031499	0.0029837	0.0027588	0.0030940	0.0015201
LONRF1	0.3056766	0.3292312	0.3138941	0.2922184	0.3560208
TRMT9B	0.1206067	0.1402303	0.0947955	0.0852557	0.1885852
DLC1	0.3823818	0.4128427	0.2368043	0.4239230	1.2115077
ENSG00000287134	0.0073861	0.0013841	0.0043054	0.0025926	0.0304320
C8orf48	0.0353196	0.0298051	0.0236162	0.0378091	0.0248864
SGCZ	0.0891289	0.1129319	0.0840489	0.0478038	0.6030689
TUSC3	1.1188011	1.0572411	1.1855750	1.2870634	1.1354400
MSR1	0.0230518	0.0261397	0.0213930	0.0074272	0.0269428
ENSG00000253496	0.0143762	0.0074269	0.0035869	0.0023601	0.0346052
FGF20	0.0024826	0.0066764	0.0014073	0.0000000	0.0000000
ENSG00000289225	0.0090152	0.0033787	0.0000000	0.0023684	0.0190411
MICU3	0.3220583	0.5150886	0.6254891	0.3019911	0.5752192
ENSG00000249258	0.0074297	0.0039365	0.0058474	0.0031883	0.0508952
ZDHHC2	0.3638641	0.3261973	0.3291168	0.3330993	0.3223733
CNOT7	0.7851914	0.7825421	0.7937071	0.6905480	0.6132695
ENSG00000289145	0.0626127	0.1203016	0.1199285	0.0567441	0.1130395
VPS37A	0.3542564	0.3761215	0.4049805	0.3453013	0.3357444
MTMR7	0.1278449	0.2095558	0.2178147	0.0848910	0.5719301
SLC7A2	0.1352287	0.0795120	0.0194110	0.0977475	0.0934142
PDGFRL	0.0046230	0.0042162	0.0038806	0.0104754	0.0031970
MTUS1	0.4621582	0.5998691	0.4018678	0.3826093	0.4659086

MTUS1-DT	0.0066380	0.0020967	0.0057960	0.0018125	0.0095156
ENSG00000253671	0.0059458	0.0014407	0.0000000	0.0000000	0.0053715
PCM1	1.2268021	1.1330294	1.2111724	1.1481146	1.2522055
ASAH1	0.8227542	0.6787796	0.8270618	1.0787957	0.6353776
ASAH1-AS1	0.0139709	0.0159687	0.0345414	0.0127670	0.0131164
ENSG00000286542	0.0057115	0.0006253	0.0109266	0.0030013	0.0065649
NAT1	0.0013591	0.0000000	0.0000000	0.0031874	0.0000000
NAT2	0.0061158	0.0000000	0.0000000	0.0302209	0.0000000
PSD3	0.5859620	0.7897945	0.8665357	0.5550702	0.9440136
ENSG00000187229	0.0000000	0.0000000	0.0000000	0.0000000	0.0073992
ENSG00000253335	0.0000000	0.0000000	0.0000000	0.0000000	0.0025260
ENSG00000253557	0.0014145	0.0000000	0.0000000	0.0000000	0.0000000
SH2D4A	0.1585589	0.0763453	0.0603119	0.1453608	0.0747384
CSGALNACT1	0.0404577	0.0428000	0.0339995	0.0909621	0.0821699
INTS10	0.3161035	0.3082923	0.2934587	0.3356333	0.4049205
LPL	0.1581654	0.0822706	0.0861611	0.7261139	0.0734398
ENSG00000253775	0.0011932	0.0000000	0.0000000	0.0000000	0.0000000
SLC18A1	0.0008892	0.0019528	0.0025154	0.0000000	0.0065199
ATP6V1B2	0.3819175	0.4577762	0.6100050	0.3898427	0.3515300
LZTS1	0.0454371	0.0687489	0.0446866	0.0334061	0.1093886
LZTS1-AS1	0.0000000	0.0000000	0.0000000	0.0000000	0.0000000
ENSG00000253300	0.0156242	0.0147468	0.0128431	0.0158394	0.0102978
ENSG00000254092	0.0016719	0.0053879	0.0000000	0.0042516	0.0231454
LINC02153	0.0000000	0.0000000	0.0000000	0.0000000	0.0000000
GFRA2	0.0519223	0.0793455	0.1746312	0.0637392	0.1791620
XPO7	0.3781393	0.2846593	0.3280802	0.3266903	0.4097589
NPM2	0.0866145	0.1616375	0.2756982	0.0673569	0.1689732
FGF17	0.0048149	0.0182669	0.0148637	0.0000000	0.0194417
DMTN	0.1751795	0.2790120	0.3953741	0.1576845	0.2523051
FHIP2B	0.0893033	0.0597017	0.0434469	0.0343418	0.0826570
NUDT18	0.0366871	0.0161509	0.0034418	0.0364528	0.0182792
HR	0.0000000	0.0062265	0.0000000	0.0000000	0.0000000
REEP4	0.0872281	0.0479611	0.0232961	0.0542041	0.0413582
LGI3	0.3266963	0.1631914	0.1179342	0.3501666	0.1135805
SFTPC	0.0897800	0.0560821	0.0369369	0.0238027	0.0217924
BMP1	0.1901767	0.1084191	0.0617949	0.2595939	0.0997324
PHYHIP	0.0796726	0.0882220	0.1289843	0.1265050	0.0720196
POLR3D	0.4367324	0.3849376	0.3672471	0.3643649	0.2863493
PIWIL2-DT	0.0120932	0.0049394	0.0036747	0.0115654	0.0295108
PIWIL2	0.0050315	0.0086550	0.0050267	0.0100222	0.0373054
SLC39A14	0.1657404	0.1192597	0.0818359	0.2662222	0.1621935
PPP3CC	0.1595624	0.2036446	0.1959991	0.1220137	0.2742495
ENSG00000251034	0.0046395	0.0034429	0.0041647	0.0000000	0.0280665
SORBS3	0.1258615	0.1209039	0.0720189	0.1321404	0.1163688
PDLIM2	0.2352252	0.1672952	0.0833404	0.2604917	0.1655377
C8orf58	0.0419864	0.0404181	0.0438455	0.0631177	0.0328942
CCAR2	0.1466582	0.1624094	0.1513215	0.1352191	0.2397436
ENSG00000253200	0.0081713	0.0101144	0.0080793	0.0010320	0.0160188
BIN3	0.0974895	0.0732607	0.0837616	0.1438145	0.1086669
ENSG00000261026	0.0000000	0.0000000	0.0000000	0.0000000	0.0000000
EGR3	0.0084160	0.0015295	0.0000000	0.0083988	0.0141706
ENSG00000253125	0.0016099	0.0012382	0.0000000	0.0038691	0.0130533
ENSG00000289521	0.0037132	0.0026749	0.0021819	0.0030550	0.0000000

PEBP4	0.0439237	0.0606256	0.0466996	0.0639505	0.0326549
ENSG00000245025	0.0053747	0.0013933	0.0032093	0.0141862	0.0105105
RHOBTB2	0.1466909	0.1881140	0.2400250	0.1467694	0.1926569
TNFRSF10B	0.1102472	0.0741336	0.0472573	0.1120831	0.1004143
ENSG00000246130	0.0013878	0.0023873	0.0019886	0.0000000	0.0000000
TNFRSF10C	0.0105729	0.0126966	0.0010275	0.0374431	0.0089032
TNFRSF10D	0.0072067	0.0057013	0.0041713	0.0088764	0.0163080
TNFRSF10A	0.0042288	0.0008447	0.0011445	0.0035558	0.0000000
CHMP7	0.1217763	0.1534981	0.2035474	0.1230301	0.1302746
ENSG00000289584	0.0021078	0.0048411	0.0022278	0.0036618	0.0039051
R3HCC1	0.3305080	0.3995477	0.4232965	0.3005298	0.3099436
LOXL2	0.0125005	0.0099161	0.0099568	0.0182712	0.0727155
LOXL2-AS1	0.0046425	0.0013339	0.0034177	0.0000000	0.0021151
ENTPD4	0.2175513	0.3323221	0.3367888	0.1618529	0.3942318
ENTPD4-DT	0.0009857	0.0074910	0.0000000	0.0000000	0.0000000
ENSG00000287166	0.0010114	0.0004488	0.0000000	0.0000000	0.0000000
SLC25A37	0.1780130	0.1359872	0.1451540	0.1224973	0.2252903
NKX3-1	0.0104206	0.0054753	0.0114428	0.0125548	0.0000000
ENSG00000253471	0.0019754	0.0081503	0.0017896	0.0000000	0.0000000
STC1	0.1138536	0.1260908	0.0826528	0.0913065	0.1511046
ENSG00000253891	0.0074148	0.0070194	0.0093734	0.0033820	0.0076218
ADAM28	0.0079947	0.0100768	0.0076052	0.0112333	0.0477760
ADAM7-AS1	0.0822830	0.1107995	0.0927401	0.0962422	0.4802584
ADAM7	0.0006832	0.0000000	0.0000000	0.0000000	0.0000000
ENSG00000272163	0.0592277	0.1391170	0.1912959	0.0415299	0.0716884
NEFM	2.4214015	3.4487808	2.7124526	2.3065543	2.7796973
NEFL	3.4795000	4.3602899	3.7601160	3.3682846	3.7032750
ENSG00000272157	0.0540345	0.0884614	0.0614286	0.0573500	0.0764939
ENSG00000253832	0.0045134	0.0127693	0.0039199	0.0055683	0.0273519
ENSG00000288055	0.0000000	0.0000000	0.0000000	0.0000000	0.0000000
ENSG00000287185	0.0000000	0.0059780	0.0032651	0.0021678	0.0000000
DOCK5	0.1465656	0.1585552	0.0842488	0.0969850	0.2218390
GNRH1	0.0006645	0.0000000	0.0000000	0.0026219	0.0080554
KCTD9	0.1014832	0.0804226	0.0436250	0.0769708	0.0557758
CDCA2	0.0123320	0.0118413	0.0000000	0.0053527	0.0030832
ENSG00000289357	0.0000000	0.0000000	0.0000000	0.0036967	0.0000000
ENSG00000253100	0.0000000	0.0000000	0.0000000	0.0057617	0.0000000
EBF2	0.0021105	0.0000000	0.0000000	0.0055143	0.0052385
PPP2R2A	0.8023587	0.6582397	0.6873117	0.7658534	0.6436644
BNIP3L	1.7953678	1.5707977	1.6092905	1.6372294	1.4061815
ENSG00000253430	0.0000000	0.0000000	0.0000000	0.0000000	0.0000000
PNMA2	0.9558779	0.9806888	1.2184510	0.9536895	0.7520412
DPYSL2	2.1269304	2.2436658	2.4818941	2.2201566	2.0924299
ADRA1A	0.1587278	0.3235069	0.2841797	0.0717728	0.2853974
ENSG00000253888	0.0000000	0.0038772	0.0000000	0.0000000	0.0000000
STMN4	0.8037010	1.3411047	1.3477845	0.7720487	1.0877411
TRIM35	0.0320831	0.0473746	0.0497126	0.0261357	0.0303489
PTK2B	0.0240592	0.0216297	0.0124863	0.0202950	0.0324349
CHRNA2	0.0000000	0.0013004	0.0042441	0.0000000	0.0000000
EPHX2	0.1130715	0.0771005	0.0429142	0.1174263	0.0685897
CLU	2.0237282	1.3853052	1.1572186	2.9010111	1.4781386
SCARA3	0.0228554	0.0253177	0.0187447	0.0251925	0.0202328
ENSG00000253875	0.0019596	0.0000000	0.0000000	0.0000000	0.0138595

CCDC25	0.5599033	0.5130425	0.6213955	0.5477854	0.3622945
ESCO2	0.0124820	0.0202843	0.0132547	0.0108964	0.0157714
PBK	0.0249820	0.0330871	0.0164413	0.0224408	0.0359210
ELP3	0.2322605	0.2482472	0.2364951	0.2256348	0.2804711
ENSG00000253690	0.0013711	0.0054988	0.0141558	0.0000000	0.0000000
PNOC	0.0347120	0.0422739	0.2131939	0.0454278	0.0530895
ZNF395	0.2389299	0.1926421	0.1347812	0.1896338	0.2133814
FBXO16	0.1453749	0.1298684	0.1673066	0.1351627	0.2820960
ENSG00000286804	0.0000000	0.0009476	0.0000000	0.0000000	0.0000000
ENSG00000254370	0.0010658	0.0032573	0.0031915	0.0000000	0.0101359
ENSG00000253567	0.0000000	0.0004410	0.0012547	0.0000000	0.0000000
FZD3	0.6225679	0.6074853	0.5858112	0.7836353	0.6611919
ENSG00000290005	0.0000000	0.0009093	0.0033938	0.0000000	0.0000000
EXTL3	0.1838392	0.1618056	0.1429919	0.2365858	0.2037270
EXTL3-AS1	0.0017843	0.0000000	0.0014327	0.0103072	0.0157066
INTS9	0.0572861	0.0496160	0.0366680	0.0511998	0.0507887
INTS9-AS1	0.0000000	0.0000000	0.0000000	0.0000000	0.0053447
HMBOX1	0.3134429	0.4134200	0.3582843	0.2604799	0.5410109
ENSG00000259366	0.0070442	0.0152442	0.0200870	0.0047737	0.0137432
KIF13B	0.1921369	0.0940324	0.0614713	0.1556298	0.1838216
ENSG00000254129	0.0012642	0.0000000	0.0034477	0.0000000	0.0014580
ENSG00000288735	0.0000000	0.0005843	0.0014209	0.0000000	0.0024068
DUSP4	0.1595854	0.3231908	0.4751201	0.0926094	0.3339529
ENSG00000285601	0.0008777	0.0017727	0.0016602	0.0000000	0.0000000
ENSG00000260253	0.0053347	0.0089460	0.0095456	0.0084533	0.0000000
LINC00589	0.0013885	0.0000000	0.0000000	0.0000000	0.0000000
LINC02099	0.0000000	0.0000000	0.0018464	0.0000000	0.0000000
ENSG00000248964	0.0009247	0.0000000	0.0000000	0.0000000	0.0000000
SARAF	1.9427303	1.8981437	2.1321036	2.2633287	1.7576850
ENSG00000272256	0.0230051	0.0076125	0.0155137	0.0176633	0.0126779
ENSG00000289334	0.0031404	0.0000000	0.0008783	0.0000000	0.0000000
LEPROTL1	0.2460554	0.2710043	0.2965415	0.3013583	0.2354274
MBOAT4	0.0000000	0.0021701	0.0017758	0.0000000	0.0000000
DCTN6-DT	0.0690236	0.0864802	0.0794046	0.0634515	0.0552662
DCTN6	0.6038162	0.6219537	0.5749938	0.6471383	0.4987577
ENSG00000253708	0.0000000	0.0000000	0.0000000	0.0000000	0.0000000
ENSG00000285669	0.0232523	0.0549278	0.0317994	0.0150466	0.0516281
ENSG00000272375	0.0048909	0.0096989	0.0022375	0.0122819	0.0044810
RBPMS	0.0283764	0.0270239	0.0160008	0.0168081	0.0286740
GTF2E2	0.3559806	0.3235112	0.3199602	0.3470420	0.3330598
SMIM18	0.1493045	0.3021762	0.4675593	0.1118694	0.1888715
GSR	0.3882583	0.3757438	0.4576768	0.4230630	0.3873622
UBXN8	0.1159425	0.1315400	0.0739125	0.1696525	0.1361354
PPP2CB	1.2506973	0.9720032	0.8696279	1.2771116	0.8918817
TEX15	0.1391840	0.0789342	0.0570748	0.1871644	0.0597657
PURG	0.2962636	0.4877820	0.5570422	0.2674289	0.7411140
WRN	0.2015014	0.1965383	0.1620081	0.1878181	0.3901485
ENSG00000253377	0.0000000	0.0000000	0.0022460	0.0000000	0.0000000
NRG1	0.7822766	1.0040080	0.7442086	0.6829463	2.3520870
ENSG00000286648	0.0002885	0.0036681	0.0019641	0.0000000	0.0826250
NRG1-IT1	0.0048012	0.0019950	0.0000000	0.0000000	0.0027407
NRG1-IT3	0.0000000	0.0000000	0.0000000	0.0000000	0.0025997
ENSG00000247134	0.3062709	0.3393716	0.3063087	0.2700895	0.2944275

ENSG00000272338	0.0054710	0.0000000	0.0014111	0.0000000	0.0115718
FUT10	0.1464266	0.0860387	0.0576015	0.1791217	0.1323686
TTI2	0.0726008	0.0718629	0.0477081	0.0575204	0.0983854
MAK16	0.1138784	0.1640271	0.2385727	0.1335414	0.1835504
RNF122	0.0275175	0.0351396	0.0321281	0.0527874	0.0559584
DUSP26	0.1247157	0.2645272	0.3356033	0.0997687	0.1757872
ENSG00000253642	0.0026262	0.0029437	0.0066943	0.0000000	0.0229168
ENSG00000254194	0.0011275	0.0000000	0.0000000	0.0000000	0.0014454
ENSG00000254302	0.0011109	0.0006278	0.0000000	0.0000000	0.0000000
ENSG00000253108	0.0000000	0.0000000	0.0000000	0.0000000	0.0000000
UNC5D	0.2509282	0.3288800	0.3602512	0.2115218	1.3029726
ENSG00000286693	0.0018751	0.0000000	0.0000000	0.0000000	0.0208495
ENSG00000253452	0.0062356	0.0115378	0.0000000	0.0021565	0.0120791
ENSG00000253363	0.0000000	0.0018845	0.0000000	0.0000000	0.0024177
KCNU1	0.0000000	0.0000000	0.0000000	0.0037348	0.0052853
ENSG00000253123	0.0006425	0.0033603	0.0000000	0.0000000	0.0021475
LINC01605	0.0035126	0.0029067	0.0000000	0.0000000	0.0108156
ENSG00000253344	0.0026972	0.0010081	0.0022512	0.0000000	0.0000000
ZNF703	0.3120447	0.3612594	0.3677603	0.3149024	0.2508931
ENSG00000253181	0.0344153	0.0191426	0.0178496	0.0390916	0.0196175
ENSG00000183154	0.0000000	0.0000000	0.0000000	0.0000000	0.0000000
ERLIN2	0.4182445	0.2711564	0.2086884	0.6682237	0.2850388
PLPBP	0.3984418	0.2997340	0.2619288	0.3522725	0.2520651
ADGRA2	0.0113633	0.0164221	0.0120022	0.0119022	0.0987912
BRF2	0.1114985	0.0915186	0.0714174	0.1287582	0.0901334
RAB11FIP1	0.3445647	0.2230970	0.1797194	0.3409213	0.2090085
EIF4EBP1	0.4769626	0.4410207	0.3556912	0.3870887	0.2781404
ENSG00000260949	0.0078788	0.0056205	0.0053331	0.0062490	0.0161522
ENSG00000286677	0.0112243	0.0192468	0.0076128	0.0033377	0.0505479
ENSG00000272128	0.0055685	0.0037134	0.0050430	0.0124227	0.0036947
ASH2L	0.2873522	0.2432813	0.2575882	0.2987858	0.2697997
STAR	0.0184960	0.0118822	0.0163454	0.0190900	0.0116953
ENSG00000253356	0.0009417	0.0063316	0.0051910	0.0064709	0.0116902
LSM1	0.3951931	0.4165477	0.4223813	0.4217184	0.3734829
BAG4	0.1499108	0.2127314	0.2505452	0.1232572	0.1585286
ENSG00000285632	0.0000000	0.0020781	0.0000000	0.0073010	0.0021475
DDHD2	0.3750313	0.5076874	0.5805107	0.3080261	0.4841912
PLPP5	0.2249122	0.1500678	0.1477578	0.3152791	0.1758827
NSD3	1.1207268	1.4108628	1.5904231	1.0138663	1.4005456
ENSG00000254898	0.0000000	0.0000000	0.0000000	0.0000000	0.0000000
ENSG00000272092	0.0024737	0.0079023	0.0039957	0.0074260	0.0000000
LETM2	0.0469554	0.0987916	0.1012469	0.0423990	0.1107162
FGFR1	0.3535888	0.2512132	0.2318300	0.4706664	0.3082339
ENSG00000272159	0.0006445	0.0034748	0.0000000	0.0027628	0.0000000
LINC03042	0.0035759	0.0037095	0.0018758	0.0137419	0.0000000
ENSG00000253361	0.0073123	0.0031463	0.0018758	0.0133331	0.0000000
TACC1	0.3575471	0.3216097	0.2857402	0.3319875	0.3938638
ENSG00000253586	0.0000000	0.0000000	0.0021501	0.0000000	0.0000000
ENSG00000253829	0.0000000	0.0000000	0.0016084	0.0000000	0.0000000
PLEKHA2	0.0654148	0.0815238	0.0716284	0.0672851	0.1084989
ENSG00000253645	0.0134866	0.0063516	0.0108868	0.0089904	0.0176734
HTRA4	0.0015440	0.0011121	0.0000000	0.0000000	0.0000000
TM2D2	0.1938387	0.1722709	0.1850940	0.3184177	0.1464463

ADAM9	0.5598349	0.3712014	0.1999769	0.8595889	0.4046097
ADAM32	0.0407781	0.0377579	0.0276089	0.0121841	0.1094293
ADAM18	0.0012625	0.0011135	0.0000000	0.0021832	0.0000000
IDO1	0.0000000	0.0000000	0.0000000	0.0000000	0.0000000
ENSG00000253939	0.0000000	0.0000000	0.0000000	0.0043864	0.0000000
IDO2	0.0000000	0.0000000	0.0000000	0.0000000	0.0000000
TCIM	0.8483139	0.4859914	0.2871981	0.8058826	0.3622606
SIRLNT	0.0107938	0.0079044	0.0090278	0.0178646	0.0096467
ENSG00000289853	0.0000000	0.0000000	0.0058240	0.0000000	0.0000000
ENSG00000272479	0.0014091	0.0023300	0.0000000	0.0015611	0.0122608
ZMAT4	0.3569534	0.2571808	0.2490537	0.2615377	0.4284759
SFRP1	0.4205781	0.5481680	0.6995335	0.3361496	0.3984479
ENSG00000253509	0.0000000	0.0000000	0.0000000	0.0000000	0.0083659
GOLGA7	0.9485503	0.8778465	0.9289125	0.9573024	0.6761851
GPAT4-AS1	0.0236477	0.0149059	0.0322685	0.0192484	0.0152408
GINS4	0.0268929	0.0176944	0.0037539	0.0354269	0.0152676
GPAT4	0.1345630	0.1624254	0.1892327	0.1299611	0.1866367
ENSG00000264578	0.0004680	0.0000000	0.0025402	0.0000000	0.0097401
NKX6-3	0.0239102	0.0430173	0.1278152	0.0187762	0.0570477
ENSG00000289921	0.0013663	0.0000000	0.0021291	0.0000000	0.0045939
ANK1	0.0601575	0.1078747	0.1269157	0.0629582	0.4302290
ENSG00000253389	0.0000000	0.0000000	0.0026426	0.0000000	0.0149356
ENSG00000260588	0.0004624	0.0011997	0.0013463	0.0000000	0.0202310
KAT6A	0.4903263	0.4934122	0.5124300	0.4884822	0.6931835
ENSG00000271938	0.0089272	0.0037144	0.0018164	0.0000000	0.0095508
ENSG00000289603	0.0016138	0.0023653	0.0000000	0.0000000	0.0000000
AP3M2	0.5022690	0.3605258	0.3950016	0.4891030	0.3359257
PLAT	0.0240631	0.0084482	0.0042720	0.0285173	0.0460990
IKBKB-DT	0.0000000	0.0010352	0.0000000	0.0037492	0.0067697
IKBKB	0.0282358	0.0210722	0.0339405	0.0294093	0.0874168
POLB	0.4021760	0.5854104	0.7040567	0.3594845	0.4646082
DKK4	0.0004918	0.0000000	0.0000000	0.0000000	0.0000000
VDAC3	0.9329044	1.0771734	1.2825725	0.9557804	0.8219832
SLC20A2	0.4706603	0.4279826	0.4055722	0.6192855	0.3912639
ENSG00000254165	0.0000000	0.0016270	0.0000000	0.0000000	0.0000000
SMIM19	0.4687077	0.4117808	0.3552118	0.4500428	0.2624160
CHRNB3	0.0000000	0.0019550	0.0009860	0.0017792	0.0000000
CHRNA6	0.0015991	0.0000000	0.0000000	0.0000000	0.0000000
THAP1	0.2382590	0.2201245	0.2692192	0.2627181	0.2304373
ENSG00000286837	0.0023479	0.0067350	0.0011886	0.0074567	0.0059085
RNF170	0.2349685	0.2212486	0.1716419	0.2610542	0.2652044
HOOK3	0.8142277	0.9367229	0.8350910	0.7355011	1.0430388
FNTA	0.5103128	0.4417683	0.4169848	0.5155539	0.3681921
POMK	0.2193729	0.3036802	0.3762378	0.1718350	0.2989246
HGSNAT	0.1425098	0.1763016	0.1867041	0.1625925	0.3433752
ASNSP1	0.0394978	0.0617240	0.0436760	0.0356263	0.0666727
ENSG00000255366	0.0182956	0.0167194	0.0314702	0.0229465	0.0231080
SPIDR	0.2749361	0.3059519	0.3967032	0.2233426	0.6585457
ENSG00000269924	0.0012870	0.0014241	0.0033175	0.0000000	0.0000000
CEBPD	0.2064733	0.1343893	0.0651600	0.2388119	0.1037257
PRKDC	0.7722420	0.6950037	0.7525323	0.8822859	0.8261748
MCM4	0.1475988	0.1119513	0.1434457	0.1587143	0.1523299
UBE2V2	0.9939710	1.1395069	1.3641924	1.0163799	0.8681826

ENSG00000253608	0.0000000	0.0000000	0.0000000	0.0000000	0.0083226
CLXN	0.3385205	0.2168834	0.1043763	0.3532736	0.2076966
SNAI2	0.0044187	0.0029327	0.0036255	0.0050900	0.0030106
ENSG00000288761	0.0006941	0.0013303	0.0000000	0.0000000	0.0035493
ENSG00000289095	0.0004369	0.0070067	0.0013494	0.0000000	0.0071570
SNTG1	0.2108096	0.2817585	0.1946271	0.1669017	0.8191302
PXDNL	0.0205301	0.0331849	0.0310621	0.0048633	0.0870853
ENSG00000272076	0.0000000	0.0018687	0.0000000	0.0063060	0.0063227
PCMTD1	0.5421417	0.5494362	0.6171925	0.4809602	0.5585589
ENSG00000253475	0.0000000	0.0025419	0.0000000	0.0000000	0.0000000
PCMTD1-DT	0.0605730	0.0603332	0.0550151	0.0613296	0.0618555
ENSG00000272024	0.0013224	0.0028949	0.0044867	0.0000000	0.0000000
ENSG00000253844	0.0020315	0.0033218	0.0045722	0.0000000	0.0040606
ST18	0.0076857	0.0193614	0.0318723	0.0034697	0.0244988
ALKAL1	0.0018975	0.0089631	0.0129418	0.0022023	0.0000000
RB1CC1	0.7261387	0.9271275	0.9704814	0.6234593	0.9277484
ENSG00000288818	0.0022119	0.0033148	0.0075898	0.0000000	0.0095716
NPBWR1	0.0000000	0.0000000	0.0000000	0.0000000	0.0000000
ENSG00000254687	0.0000000	0.0000000	0.0000000	0.0000000	0.0000000
OPRK1	0.0151559	0.0239192	0.0425757	0.0159348	0.0258618
ENSG00000253369	0.0117867	0.0057761	0.0070804	0.0127512	0.0106728
LINC02984	0.0340889	0.0151301	0.0018196	0.0330235	0.0203223
ATP6V1H	0.4676527	0.4574038	0.5330699	0.4555273	0.5448224
RGS20	0.0267929	0.0200208	0.0087259	0.0320934	0.0792077
TCEA1	2.1182720	1.7655893	1.6537905	1.9442412	1.6196185
ENSG00000260955	0.0010064	0.0019036	0.0000000	0.0000000	0.0117641
LYPLA1	0.5416753	0.6105578	0.6534209	0.5274939	0.4821152
MRPL15	0.3193759	0.3679422	0.3327053	0.3501381	0.2360635
RP1	0.0039063	0.0046008	0.0000000	0.0071880	0.0000000
XKR4	0.4266402	0.6895960	0.6704553	0.3795669	1.5167382
ENSG00000253976	0.0025707	0.0014383	0.0000000	0.0031700	0.0301788
ENSG00000253857	0.0010553	0.0000000	0.0024411	0.0000000	0.0063341
XKR4-AS1	0.0000000	0.0011324	0.0000000	0.0000000	0.0000000
TMEM68	0.2255884	0.1703177	0.1591186	0.2810684	0.1550734
TGS1	0.2768704	0.2375004	0.2228373	0.2766506	0.2815304
LYN	0.1389686	0.0903371	0.0457506	0.1435561	0.0548585
RPS20	2.6846891	2.5900277	2.3782052	2.5472634	2.3984452
PLAG1	0.1739126	0.1031703	0.0968917	0.1174033	0.1303344
CHCHD7	0.1949158	0.1922519	0.2006057	0.2196469	0.1747907
ENSG00000272343	0.0105530	0.0081177	0.0119311	0.0115704	0.0146342
SDR16C5	0.0040909	0.0020745	0.0024928	0.0000000	0.0000000
PENK	0.0174526	0.0115259	0.0090966	0.0127101	0.0453066
PENK-AS1	0.0020538	0.0018497	0.0000000	0.0014762	0.0000000
BPNT2	0.4197593	0.3319089	0.3896843	0.5144347	0.3466183
LINC01606	0.0000000	0.0035779	0.0000000	0.0000000	0.0024821
ENSG00000253322	0.0008772	0.0000000	0.0090221	0.0000000	0.0026927
LINC01602	0.0006502	0.0000000	0.0000000	0.0000000	0.0000000
FAM110B	0.2517104	0.3174259	0.3790217	0.1977473	0.5057778
ENSG00000253116	0.0000000	0.0014171	0.0026978	0.0000000	0.0091460
ENSG00000288041	0.0000000	0.0209462	0.0123612	0.0082934	0.0144064
UBXN2B	0.2671805	0.3215666	0.3842017	0.2169400	0.2845192
SDCBP	1.0906572	0.7948213	0.6060953	1.1802063	0.7405239
NSMAF	0.1053682	0.0960267	0.0644299	0.1151498	0.1502176

TOX	0.2888536	0.2399807	0.3598992	0.1880104	0.5945356
TOX-DT	0.0855424	0.1050956	0.1100905	0.0828966	0.0899806
CA8	0.0268206	0.0433954	0.1559974	0.0076610	0.0629707
LINC01301	0.0634116	0.0726003	0.0373739	0.0639286	0.0637568
RAB2A	1.4493488	1.5667293	1.8425233	1.4303627	1.3654785
ENSG00000255321	0.0000000	0.0000000	0.0000000	0.0000000	0.0000000
ENSG00000228862	0.0042718	0.0103418	0.0040960	0.0055999	0.0062036
ENSG00000255289	0.0000000	0.0000000	0.0000000	0.0000000	0.0000000
CHD7	0.4611619	0.3872088	0.2717135	0.4538829	0.6633815
ENSG00000287975	0.0052552	0.0027459	0.0016561	0.0000000	0.0075268
ENSG00000254777	0.0000000	0.0009903	0.0000000	0.0000000	0.0071690
ENSG00000254802	0.0026787	0.0016179	0.0032143	0.0000000	0.0052305
CLVS1	0.1111023	0.1782481	0.2178906	0.0808220	0.2928560
ENSG00000254222	0.0000000	0.0000000	0.0000000	0.0000000	0.0000000
ASPH	0.5534614	0.5532182	0.6218874	0.7037508	0.6663440
LINC02842	0.0000000	0.0011906	0.0000000	0.0000000	0.0057345
ENSG00000285971	0.0099669	0.0152702	0.0147812	0.0035761	0.0211731
NKAIN3	0.3982242	0.3643298	0.3951508	0.3481315	0.7451301
ENSG00000287105	0.0000000	0.0000000	0.0000000	0.0000000	0.0070802
ENSG00000240915	0.0000000	0.0000000	0.0000000	0.0000000	0.0013481
GGH	0.2333850	0.2290364	0.2194118	0.3802299	0.2075846
TTPA	0.0180531	0.0109249	0.0061881	0.0314237	0.0019716
YTHDF3-DT	0.0392135	0.0270440	0.0116065	0.0384347	0.0252188
YTHDF3	0.2782883	0.3253779	0.3202124	0.2548431	0.4134391
ENSG00000261542	0.0045318	0.0157228	0.0153377	0.0000000	0.0061902
ENSG00000253205	0.0006031	0.0000000	0.0014107	0.0000000	0.0000000
ENSG00000253894	0.0048485	0.0012303	0.0044611	0.0000000	0.0062816
ENSG00000253583	0.0024463	0.0027994	0.0104597	0.0046313	0.0045046
ENSG00000287844	0.0010303	0.0049835	0.0118347	0.0000000	0.0225707
LINC01414	0.0725348	0.1321265	0.0719108	0.0460441	0.2586045
ENSG00000253762	0.0007950	0.0000000	0.0006934	0.0000000	0.0266896
LINC01289	0.0000000	0.0014000	0.0000000	0.0000000	0.0059786
MIR124-2HG	0.0706378	0.1463933	0.1158725	0.0557768	0.3812457
ENSG00000287615	0.0000000	0.0000000	0.0000000	0.0000000	0.0000000
BHLHE22	0.0000000	0.0014467	0.0000000	0.0000000	0.0000000
CYP7B1	0.0477328	0.0571569	0.0526443	0.0882756	0.1043963
ENSG00000287998	0.0019592	0.0064838	0.0077582	0.0000000	0.0063273
ENSG00000272010	0.0098897	0.0186470	0.0105205	0.0048628	0.0191128
ARMC1	0.3921172	0.3889532	0.5121531	0.3762328	0.3168712
MTFR1	0.1606686	0.1389608	0.1245629	0.1759776	0.1674605
PDE7A	0.1311616	0.1533687	0.1259143	0.1096050	0.4618016
PDE7A-DT	0.0007657	0.0000000	0.0000000	0.0000000	0.0000000
DNAJC5B	0.0016737	0.0003142	0.0000000	0.0000000	0.0038646
ENSG00000253190	0.0025169	0.0000000	0.0000000	0.0000000	0.0000000
ENSG00000287127	0.0077201	0.0051175	0.0000000	0.0026302	0.0185677
RRS1	0.0959751	0.0511549	0.1015490	0.0976129	0.0951040
ADHFE1	0.0335914	0.0179905	0.0151983	0.0467579	0.0396534
VXN	0.0092897	0.0053565	0.0069612	0.0180837	0.0059578
MYBL1	0.0178250	0.0131665	0.0152876	0.0256365	0.0093478
VCPIP1	0.1872674	0.2070173	0.2511317	0.1347032	0.1952025
C8orf44	0.1372118	0.1486454	0.1177303	0.1207990	0.1172512
SGK3	0.0527432	0.0366069	0.0264400	0.0418486	0.1229182
MCMDC2	0.0076515	0.0164353	0.0012756	0.0081349	0.0046972

SNHG6	1.6498831	1.7682330	1.7054718	1.5472421	1.5256364
TCF24	0.0016475	0.0091561	0.0078587	0.0085105	0.0106402
PPP1R42	0.2308691	0.1511545	0.0926058	0.2551341	0.1078625
COPS5	0.5451117	0.5356306	0.4382309	0.5478862	0.4102686
CSPP1	0.5839720	0.4604643	0.4206530	0.5757554	0.6709054
ARFGEF1	0.4367710	0.5069909	0.4848141	0.4694650	0.7350422
ENSG00000287214	0.0000000	0.0000000	0.0000000	0.0020756	0.0065398
ARFGEF1-DT	0.0232445	0.0217609	0.0197541	0.0391027	0.0094593
CPA6	0.0063256	0.0088220	0.0101879	0.0030760	0.0122912
PREX2	0.3028023	0.1822323	0.1236719	0.3898949	0.2620924
C8orf34-AS1	0.1333543	0.0825492	0.0487521	0.1347898	0.0743051
C8orf34	1.1468461	0.7933248	0.5846585	1.0286865	1.2038236
ENSG00000254337	0.0050382	0.0000000	0.0023036	0.0000000	0.0029782
LINC01592	0.0115044	0.0076040	0.0057363	0.0123268	0.0129359
SULF1	1.8191132	1.2682176	0.9717591	2.6572266	1.3633314
SLCO5A1	0.0926898	0.1399202	0.1399473	0.1180817	0.3551729
ENSG00000254557	0.0000000	0.0000000	0.0000000	0.0000000	0.0000000
SLCO5A1-AS1	0.0903318	0.1667812	0.1691077	0.0378549	0.1055184
PRDM14	0.0009037	0.0000000	0.0000000	0.0000000	0.0070395
NCOA2	0.3594416	0.3990046	0.3915178	0.3129471	0.7625603
ENSG00000288966	0.0119173	0.0140537	0.0254590	0.0152187	0.0134173
TRAM1	0.8916359	0.6134693	0.4873912	1.0027433	0.5455043
LACTB2-AS1	0.0020510	0.0020631	0.0027326	0.0087673	0.0000000
LACTB2	0.2244472	0.1439604	0.0962047	0.2624546	0.0874844
XKR9	0.0243424	0.0295829	0.0610294	0.0265355	0.0986230
ENSG00000254031	0.0036524	0.0029044	0.0000000	0.0000000	0.0079988
EYA1	0.0083704	0.0062581	0.0016469	0.0098114	0.0052331
MSC-AS1	0.0032901	0.0045085	0.0000000	0.0039003	0.0044857
TRPA1	0.0026379	0.0000000	0.0000000	0.0037288	0.0036367
KCNB2	0.3082368	0.3870405	0.3071193	0.2992208	1.3215872
ENSG00000253726	0.0000000	0.0021513	0.0000000	0.0000000	0.0016673
ENSG00000260838	0.0352864	0.0507293	0.0517460	0.0456367	0.0541290
TERF1	0.4682743	0.4384277	0.5046832	0.5201504	0.4607587
ENSG00000253636	0.0230325	0.0409738	0.0403028	0.0203090	0.0588879
SBSPON	0.0397564	0.0224592	0.0087318	0.0437324	0.0288259
C8orf89	0.0042577	0.0076746	0.0059368	0.0025316	0.0108998
RPL7	2.6123827	2.5167275	2.3437786	2.5023945	2.2368231
RDH10	0.0323871	0.0443927	0.0288864	0.0330000	0.0282034
RDH10-AS1	0.0000000	0.0028939	0.0000000	0.0000000	0.0000000
ENSG00000253235	0.0000000	0.0000000	0.0000000	0.0000000	0.0055025
STAU2-AS1	0.0000000	0.0002840	0.0000000	0.0000000	0.0000000
STAU2	0.7099236	0.8532409	0.9331013	0.6316327	1.0338735
ENSG00000254538	0.0060800	0.0059108	0.0041758	0.0090386	0.1013455
UBE2W	0.4927382	0.5005775	0.4886807	0.4464324	0.4915397
ENSG00000286113	0.0000000	0.0000000	0.0000000	0.0000000	0.0094442
ELOC	0.9423158	1.0482426	1.0764758	0.9299978	0.8682522
TMEM70	0.1681221	0.1846820	0.2620738	0.1877546	0.1664230
LY96	0.0014215	0.0020342	0.0015219	0.0000000	0.0000000
ENSG00000253983	0.0006391	0.0000000	0.0000000	0.0000000	0.0037043
JPH1	0.0181831	0.0331939	0.0408196	0.0233976	0.0665631
GDAP1	0.4214052	0.6802562	0.8577359	0.4278835	0.6189041
ENSG00000253596	0.0050337	0.0220020	0.0337315	0.0016127	0.0037962
MIR2052HG	0.0064951	0.0045854	0.0048515	0.0000000	0.0123137

LINC03071	0.0000000	0.0000000	0.0000000	0.0000000	0.0000000
ENSG00000253706	0.0002255	0.0008298	0.0000000	0.0013512	0.0000000
PI15	0.0121271	0.0091964	0.0047520	0.0220904	0.0154245
ENSG00000254043	0.0000000	0.0000000	0.0000000	0.0000000	0.0000000
CRISPLD1	1.0117016	0.6533249	0.4447639	2.1468466	0.7544797
ENSG00000254238	0.0000000	0.0000000	0.0000000	0.0000000	0.0000000
CASC9	0.1138788	0.0864665	0.0489644	0.1281557	0.1115495
HNF4G	0.0114453	0.0119865	0.0166213	0.0243667	0.0121854
ENSG00000287352	0.0000000	0.0032937	0.0000000	0.0000000	0.0000000
ENSG00000270866	0.0127474	0.0367872	0.0549685	0.0143598	0.0206894
LINC01111	0.0000000	0.0029456	0.0000000	0.0000000	0.0000000
ZFHX4-AS1	0.0675964	0.1145502	0.1978655	0.0463370	0.1954684
ZFHX4	0.4289878	0.5736455	1.0510672	0.4415666	0.8149860
ENSG00000253416	0.0000000	0.0000000	0.0000000	0.0000000	0.0000000
PEX2	0.3751577	0.2884757	0.2627994	0.3973582	0.2425786
ENSG00000288756	0.0148836	0.0082663	0.0093616	0.0113948	0.0194962
ENSG00000254366	0.0000000	0.0000000	0.0000000	0.0000000	0.0000000
PKIA-AS1	0.0061466	0.0031149	0.0036930	0.0000000	0.0438089
PKIA	1.0399007	1.7983923	2.1946755	0.9472358	1.5048452
ENSG00000260398	0.0081904	0.0118438	0.0091258	0.0050945	0.0091828
ENSG00000286675	0.0084358	0.0151942	0.0177504	0.0023938	0.0072139
ZC2HC1A	0.8131586	1.1073827	1.2327846	0.6850283	1.0264666
IL7	0.0183974	0.0224841	0.0145416	0.0136263	0.1171097
ENSG00000285744	0.0009546	0.0046872	0.0024138	0.0068168	0.0222738
STMN2	2.7376056	3.6481999	4.0274621	2.5912744	3.1221671
HEY1	0.4151791	0.2813091	0.1974286	0.6988381	0.2668791
LINC01607	0.0465958	0.0391364	0.0320077	0.0468462	0.0220997
ENSG00000249328	0.0114884	0.0018598	0.0041049	0.0202372	0.0108041
ENSG00000285758	0.0025907	0.0000000	0.0010558	0.0057382	0.0000000
MRPS28	0.4635586	0.3662085	0.3249113	0.5243021	0.3647327
ENSG00000272518	0.0016776	0.0014229	0.0000000	0.0000000	0.0012623
TPD52	0.4241685	0.4850990	0.6553077	0.2930755	0.4976829
ENSG00000254205	0.0000000	0.0000000	0.0000000	0.0000000	0.0014252
ENSG00000253238	0.0021950	0.0034347	0.0050472	0.0000000	0.0080408
ENSG00000251867	0.0389840	0.0655274	0.0682883	0.0258668	0.0728720
ZBTB10	0.4354904	0.3948240	0.4218469	0.4479962	0.4587891
ENSG00000254162	0.0026725	0.0000000	0.0000000	0.0000000	0.0029896
LINC02986	0.0007192	0.0055851	0.0039945	0.0018855	0.0029896
ZNF704	0.4522151	0.6554672	0.5699256	0.4103495	1.0029189
ENSG00000286763	0.0000000	0.0004167	0.0000000	0.0000000	0.0055701
PAG1	0.1823786	0.1990371	0.1704572	0.1870116	0.2782961
ENSG00000254177	0.0004532	0.0000000	0.0000000	0.0000000	0.0000000
ENSG00000272425	0.0006609	0.0033095	0.0011015	0.0000000	0.0058720
LNMICC	0.0057286	0.0021276	0.0000000	0.0015171	0.0000000
FABP5	1.0986283	1.3029933	1.5757416	1.4608019	1.0429184
ENSG00000253859	0.0000000	0.0000000	0.0000000	0.0000000	0.0000000
PMP2	0.0047918	0.0011627	0.0000000	0.0068168	0.0117972
FABP12	0.0042088	0.0078135	0.0000000	0.0000000	0.0167733
ENSG00000290914	0.0021034	0.0008219	0.0037355	0.0000000	0.0000000
IMPA1	0.4088642	0.4200955	0.4064610	0.5031695	0.3340119
SLC10A5	0.0053322	0.0083607	0.0094919	0.0026542	0.0000000
ZFAND1	0.3273062	0.4652890	0.4849111	0.3193477	0.3065573
CHMP4C	0.0121223	0.0051763	0.0048552	0.0113030	0.0195965

ENSG00000253334	0.0000000	0.0000000	0.0000000	0.0068248	0.0000000
SNX16	0.1486032	0.2293433	0.2175751	0.1546890	0.1686764
LINC02235	0.0020654	0.0000000	0.0116686	0.0010138	0.0104264
ENSG00000254394	0.0053794	0.0082502	0.0072175	0.0047294	0.0356312
ENSG00000253503	0.0000000	0.0014091	0.0017124	0.0000000	0.0000000
ENSG00000254202	0.0000000	0.0022874	0.0000000	0.0000000	0.0000000
RALYL	0.4775472	0.7101780	0.6804166	0.3891264	1.5223022
ENSG00000288897	0.0000000	0.0044373	0.0000000	0.0000000	0.0360499
LRRCC1	0.3430447	0.2957891	0.2946841	0.4080366	0.2441648
E2F5-DT	0.0046985	0.0032324	0.0014422	0.0000000	0.0000000
E2F5	0.5250848	0.3495820	0.2312306	0.5345001	0.2694945
RBIS	0.8374866	0.8067062	0.7331686	0.7836823	0.6767898
CA13	0.0456532	0.0246980	0.0296619	0.0403016	0.0630839
ENSG00000258256	0.0000000	0.0000000	0.0032395	0.0000000	0.0000000
CA1	0.0007306	0.0012400	0.0014647	0.0000000	0.0134192
CA3	0.0091543	0.0082048	0.0038486	0.0073348	0.0333110
CA3-AS1	0.0017555	0.0040527	0.0012847	0.0033686	0.0055636
CA2	0.0111298	0.0084753	0.0008433	0.0122985	0.0067122
ATP6V0D2	0.0992525	0.0478511	0.0312445	0.1196302	0.0271487
ENSG00000253675	0.0000000	0.0000000	0.0000000	0.0000000	0.0000000
WWP1-AS1	0.0016313	0.0000000	0.0000000	0.0000000	0.0000000
WWP1	0.5259358	0.4429686	0.4384863	0.6101210	0.4434465
RMDN1	0.4691756	0.4690627	0.3517857	0.4727953	0.3743467
CPNE3	0.6729022	0.4981506	0.3445849	0.6520090	0.4420632
CNGB3	0.0015357	0.0000000	0.0000000	0.0016755	0.0057171
ENSG00000254115	0.0010264	0.0045786	0.0000000	0.0012109	0.0000000
CNBD1	0.0000000	0.0042525	0.0000000	0.0031358	0.0000000
ENSG00000253500	0.0000000	0.0012945	0.0000000	0.0000000	0.0000000
ENSG00000253171	0.0009283	0.0000000	0.0000000	0.0000000	0.0037236
MMP16	0.1797012	0.2591059	0.3625118	0.1458633	0.7269820
ENSG00000253553	0.0478880	0.0833353	0.0476914	0.0371468	0.2208802
RIPK2-DT	0.1310797	0.1268339	0.0670044	0.1031794	0.3535405
RIPK2	0.1266686	0.1585282	0.1316047	0.1424006	0.1287202
OSGIN2	0.1989179	0.2060637	0.1775825	0.1744035	0.1309319
NBN	0.3279371	0.2851485	0.3223905	0.3344756	0.2175794
DECR1	0.3382434	0.2468384	0.1706887	0.3566633	0.2420457
CALB1	0.0465429	0.0428721	0.0718424	0.0211874	0.0363214
ENSG00000254180	0.0213912	0.0319559	0.0231036	0.0126363	0.0812558
LINC00534	0.0087203	0.0059641	0.0056250	0.0031190	0.0217359
LINC01030	0.0013518	0.0000000	0.0000000	0.0000000	0.0000000
TMEM64	0.1447580	0.1176113	0.1167600	0.1544767	0.1449288
ENSG00000246792	0.0005446	0.0000000	0.0025670	0.0000000	0.0041632
NECAB1	0.0167914	0.0223878	0.0567160	0.0255345	0.0356223
ENSG00000254251	0.0000000	0.0000000	0.0000000	0.0000000	0.0000000
ENSG00000289502	0.0057324	0.0031819	0.0034763	0.0077658	0.0000000
C8orf88	0.0219201	0.0146632	0.0151530	0.0109961	0.0247498
PIP4P2	0.5636285	0.6413559	0.7398823	0.5606920	0.4533208
OTUD6B-AS1	0.6273356	0.6305657	0.6776018	0.6185116	0.5023802
OTUD6B	0.1476403	0.1346882	0.1593449	0.1237879	0.1262068
LRRC69	0.0096587	0.0023470	0.0062502	0.0124035	0.0268866
SLC26A7	0.0000000	0.0009942	0.0017765	0.0052741	0.0000000
ENSG00000253901	0.0000000	0.0000000	0.0000000	0.0065998	0.0085580
RUNX1T1	0.4812511	1.0153582	0.9367220	0.4138332	1.0764945

ENSG00000253634	0.0000000	0.0015284	0.0014111	0.0000000	0.0035405
FLJ46284	0.0082221	0.0168414	0.0045196	0.0102159	0.0535167
ENSG00000253577	0.0009967	0.0000000	0.0000000	0.0000000	0.0035216
ENSG00000253197	0.0018092	0.0021328	0.0000000	0.0000000	0.0139972
TRIQK	0.4795440	0.4439618	0.4584358	0.5340096	0.3899660
LINC02906	0.0042872	0.0011833	0.0025438	0.0000000	0.0000000
CIBAR1-DT	0.0698007	0.0456637	0.0300255	0.0470906	0.0513128
ENSG00000254089	0.0000000	0.0000000	0.0000000	0.0000000	0.0000000
CIBAR1	0.5662770	0.6127623	0.6562128	0.5196883	0.5414936
ENSG00000271971	0.0231135	0.0275486	0.0291683	0.0155340	0.0330457
ENSG00000253854	0.0011830	0.0000000	0.0009114	0.0026207	0.0000000
RBM12B	0.2578997	0.2737465	0.3633775	0.2333513	0.2739435
RBM12B-DT	0.0004091	0.0000000	0.0000000	0.0031392	0.0095713
TMEM67	0.1691434	0.1306007	0.0851160	0.2348109	0.2900039
ENSG00000254057	0.0000000	0.0000000	0.0000000	0.0000000	0.0000000
PDP1	0.1382054	0.3099299	0.3093459	0.1088283	0.2232904
CDH17	0.0011632	0.0020619	0.0023362	0.0000000	0.0035158
GEM	0.0210304	0.0226785	0.0129305	0.0202066	0.0254519
RAD54B	0.0135950	0.0115967	0.0116409	0.0226011	0.0016028
FSBP	0.0551303	0.0337630	0.0399278	0.0590144	0.0390476
VIRMA	0.3661613	0.2626518	0.2612247	0.3683653	0.2918797
VIRMA-DT	0.0127464	0.0095378	0.0132281	0.0116526	0.0085358
ESRP1	0.0071333	0.0000000	0.0076294	0.0024238	0.0000000
DPY19L4	0.2742270	0.1920373	0.1454237	0.2907324	0.1911421
INTS8	0.2613877	0.2036617	0.2323731	0.2374514	0.2722128
CCNE2	0.0222527	0.0115256	0.0104079	0.0261261	0.0124552
ENSG00000272509	0.0014920	0.0009830	0.0000000	0.0000000	0.0000000
NDUFAF6	0.2577156	0.2510352	0.2975093	0.2003738	0.3160847
TP53INP1	0.1406827	0.1077216	0.0614179	0.2243740	0.1089227
ENSG00000254248	0.0008739	0.0000000	0.0000000	0.0000000	0.0072503
PLEKHF2	0.1417986	0.0894901	0.0585364	0.1631988	0.1008620
CFAP418-AS1	0.0198554	0.0106322	0.0126702	0.0145161	0.0130591
CFAP418	0.2621027	0.1395603	0.1239412	0.2332869	0.1381840
GDF6	0.0008666	0.0020998	0.0000000	0.0032590	0.0047414
UQCRB	1.9760125	2.1255524	2.1122538	1.8461178	1.7696874
UQCRB-AS1	0.0059217	0.0157044	0.0086106	0.0081235	0.0098291
MTERF3	0.1268750	0.0890892	0.0978294	0.1092320	0.1007885
PTDSS1	0.1782955	0.2306851	0.2196168	0.1870457	0.2381987
SDC2	0.9659550	0.6250015	0.5209824	1.2993385	0.6414840
CPQ	0.5672015	0.3570043	0.2725619	0.7109662	0.3539969
TSPYL5	0.1202274	0.2007159	0.2525475	0.1102724	0.1743200
MTDH	0.9867317	0.9254679	0.8622424	1.0198273	0.9134094
LAPTM4B	0.6007110	0.6067499	0.6306776	0.7280410	0.6519135
MATN2	0.0540351	0.0408071	0.0322949	0.0508812	0.1030274
RPL30	2.8992867	2.8801008	2.7380298	2.7809307	2.6073948
RPL30-AS1	0.0112371	0.0059775	0.0123085	0.0058659	0.0311938
ENSG00000288752	0.0448587	0.0481736	0.0756693	0.0278407	0.0217797
ERICH5	0.0314226	0.0340992	0.0586017	0.0034396	0.0603122
RIDA	0.3122990	0.1993960	0.1861517	0.3632628	0.1781922
POP1	0.0641267	0.0792453	0.0748642	0.0592733	0.0546596
NIPAL2	0.0725850	0.0588026	0.0387988	0.0868203	0.0522972
STK3	0.2127760	0.1688120	0.0889546	0.1804898	0.2271085
KCNS2	0.0241753	0.0335644	0.0549702	0.0156942	0.0118396

ENSG00000272321	0.0013809	0.0025438	0.0022360	0.0000000	0.0065448
OSR2	0.0052210	0.0000000	0.0099425	0.0029510	0.0140120
VPS13B-DT	0.3094458	0.2610929	0.2208508	0.3464462	0.1815617
VPS13B	0.2958535	0.3171171	0.2185335	0.2127705	0.7152990
ENSG00000253539	0.0000000	0.0000000	0.0000000	0.0000000	0.0000000
COX6C	1.7446063	1.8880113	1.9016581	1.7723911	1.5547393
RGS22	0.3953144	0.2234702	0.1728805	0.3319040	0.2215312
FBXO43	0.0089262	0.0040138	0.0045069	0.0000000	0.0210300
POLR2K	0.7943634	0.8993689	0.9278127	0.7864514	0.6216903
SPAG1	0.5785305	0.3073856	0.2124863	0.6118924	0.3263429
RNF19A	0.8655657	0.7275312	0.5976092	0.9251868	0.7459159
ENSG00000253824	0.0022313	0.0000000	0.0000000	0.0080224	0.0000000
ENSG00000253666	0.0010364	0.0049472	0.0045537	0.0023909	0.0000000
ANKRD46	0.5159308	0.7488166	1.0151681	0.4595624	0.5543361
SNX31	0.0061234	0.0066844	0.0086557	0.0120014	0.0210186
PABPC1	2.0729725	2.1528840	2.0488824	1.9541816	1.8888193
ENSG00000289284	0.0000000	0.0031880	0.0022278	0.0000000	0.0000000
ENSG00000260368	0.0062228	0.0011287	0.0033856	0.0052511	0.0062948
YWHAZ	2.1354249	2.3133275	2.6083411	2.0748879	2.0124234
FLJ42969	0.0019566	0.0000000	0.0000000	0.0000000	0.0000000
LINC03044	0.0000000	0.0032821	0.0000000	0.0025261	0.0000000
ZNNT1	0.0439320	0.0683451	0.0804087	0.0171161	0.0483904
ZNF706	0.8716382	0.9084820	0.8948251	0.8608714	0.7205791
ENSG00000287479	0.0000000	0.0016966	0.0010852	0.0000000	0.0000000
GRHL2	0.0011932	0.0010959	0.0019715	0.0000000	0.0247956
NCALD	0.8687217	0.8560204	0.7985712	0.9213732	0.8750716
ENSG00000253629	0.0547594	0.0524512	0.0321177	0.0553556	0.0400636
RRM2B	0.5374317	0.3318387	0.2388219	0.5523961	0.3935731
UBR5-DT	0.0329246	0.0250083	0.0085601	0.0418928	0.0228499
UBR5	0.4662888	0.4246167	0.3731540	0.3796685	0.8752641
LINC03047	0.0043907	0.0026586	0.0028368	0.0000000	0.0000000
KLF10	0.1751284	0.1486978	0.1545668	0.1749647	0.2092675
ENSG00000283959	0.0117380	0.0130758	0.0072495	0.0130285	0.0156404
GASAL1	0.0067868	0.0100185	0.0032824	0.0000000	0.0031339
AZIN1	0.9583193	0.9207645	0.9698867	0.8534186	0.7425892
MAILR	0.1549346	0.1712184	0.1767477	0.1567075	0.2203314
ATP6V1C1	0.4142986	0.4969975	0.6428345	0.4429503	0.4284855
BAALC-AS2	0.0087248	0.0065841	0.0217968	0.0027907	0.0091544
BAALC	0.3520236	0.6003097	0.7359692	0.2648864	0.4760727
BAALC-AS1	0.0164857	0.0486976	0.0132177	0.0121152	0.0427116
FZD6	0.1155064	0.0737178	0.0450145	0.1821870	0.1221179
CTHRC1	0.0180284	0.0395261	0.0493080	0.0110583	0.0195393
LINC02933	0.0003926	0.0000000	0.0009570	0.0000000	0.0000000
SLC25A32	0.1088096	0.1649515	0.1823684	0.0940599	0.1861691
DCAF13	0.3064909	0.2561883	0.2783190	0.3854573	0.2661141
ENSG00000288945	0.0010903	0.0058567	0.0012490	0.0038338	0.0073410
ENSG00000261670	0.0000000	0.0009175	0.0000000	0.0000000	0.0000000
ENSG00000271830	0.0092689	0.0189821	0.0250047	0.0022588	0.0196359
ENSG00000253477	0.0217259	0.0367002	0.0634420	0.0105397	0.0585131
RIMS2	0.5023720	0.6470864	0.6498926	0.4247304	1.7321845
DPYS	0.0010157	0.0013616	0.0045071	0.0000000	0.0122542
DCSTAMP	0.0000000	0.0000000	0.0000000	0.0000000	0.0000000
LRP12	0.1389376	0.1756155	0.2389354	0.1875334	0.3465684

ZFPM2	0.1742925	0.1834982	0.1819542	0.1309126	0.5926473
ZFPM2-AS1	0.0061589	0.0097356	0.0109416	0.0070366	0.1118079
ENSG00000253526	0.0000000	0.0000000	0.0000000	0.0000000	0.0059619
OXR1	0.6032747	0.7872458	0.9454094	0.5771903	0.6313686
OXR1-AS1	0.0039815	0.0011229	0.0031219	0.0027049	0.0241443
ANGPT1	0.0172014	0.0227973	0.0138276	0.0643945	0.0628967
ENSG00000287949	0.0017220	0.0000000	0.0000000	0.0000000	0.0031970
RSPO2	0.0160777	0.0499419	0.0178440	0.0132904	0.0862066
EIF3E	1.3877527	1.3416160	1.0991709	1.2952060	1.1567263
ENSG00000289094	0.0050626	0.0081807	0.0143420	0.0134181	0.0132361
EMC2	0.6226448	0.4669678	0.3366229	0.6561227	0.4579397
TMEM74	0.0640376	0.1357694	0.1437150	0.0616265	0.0725211
ENSG00000253796	0.0020022	0.0029352	0.0000000	0.0053392	0.0148986
TRHR	0.0010222	0.0029930	0.0000000	0.0000000	0.0050702
NUDCD1	0.1844783	0.1586646	0.1887347	0.1619016	0.2094977
ENY2	0.7664964	0.7889765	0.7720941	0.7575159	0.6403476
PKHD1L1	0.0165705	0.0109845	0.0107029	0.0138466	0.0000000
EBAG9	0.4987597	0.4343152	0.4100813	0.5052586	0.4047528
SYBU	0.4147995	0.4975495	0.6578534	0.3432192	0.6428441
KCNV1	0.0074166	0.0159437	0.0243141	0.0243683	0.0116705
LINC02237	0.0008930	0.0007774	0.0000000	0.0000000	0.0000000
CSMD3	0.3168083	0.3868519	0.3589028	0.2000634	1.3453740
ENSG00000286946	0.0000000	0.0013201	0.0000000	0.0000000	0.0058301
ENSG00000286937	0.0000000	0.0033224	0.0011221	0.0000000	0.0000000
ENSG00000253207	0.0008319	0.0000000	0.0012924	0.0000000	0.0000000
TRPS1	0.2980668	0.2723077	0.1623429	0.3366192	0.5586007
LINC00536	0.0019698	0.0000000	0.0069726	0.0022345	0.0000000
EIF3H	1.2475952	1.2770585	1.2441675	1.1910535	1.1281341
UTP23	0.4004448	0.3861174	0.4235607	0.3025111	0.4489567
RAD21	1.2870256	1.3715165	1.6021640	1.1881115	1.1723977
RAD21-AS1	0.0022459	0.0113335	0.0042926	0.0000000	0.0073097
AARD	0.0983424	0.0835198	0.1022923	0.0673382	0.0421317
SLC30A8	0.0011410	0.0052019	0.0000000	0.0000000	0.0232972
MED30	0.3207312	0.3211805	0.2994817	0.2575767	0.1666265
EXT1	0.3162062	0.3092498	0.2366214	0.3679656	0.8547024
SAMD12	0.1434790	0.2450669	0.3364177	0.1431147	0.3687749
ENSG00000225885	0.0000000	0.0026226	0.0010813	0.0000000	0.0230169
SAMD12-AS1	0.0067049	0.0094958	0.0093124	0.0069059	0.0339517
TNFRSF11B	0.0290197	0.0196375	0.0086760	0.0635466	0.0205374
COLEC10	0.0016281	0.0037835	0.0046974	0.0000000	0.0000000
MAL2	0.2662936	0.2916461	0.4957937	0.2200366	0.2288755
MAL2-AS1	0.0000000	0.0000000	0.0000000	0.0000000	0.0000000
ENSG00000286282	0.0044052	0.0055641	0.0020317	0.0091887	0.0000000
CCN3	0.1340348	0.1526380	0.1518040	0.3548547	0.1294441
ENSG00000253398	0.0000000	0.0019676	0.0000000	0.0000000	0.0037333
ENPP2	0.3149974	0.2074944	0.1500061	0.5464314	0.3164491
TAF2	0.1448875	0.2190012	0.2350291	0.1973024	0.3065809
ENSG00000286362	0.0396354	0.0142863	0.0340733	0.0461153	0.0083268
DSCC1	0.0161108	0.0171189	0.0106234	0.0454039	0.0152357
DEPTOR-AS1	0.0000000	0.0000000	0.0000000	0.0000000	0.0089642
DEPTOR	0.0424690	0.0570252	0.0640964	0.0479382	0.0543235
ENSG00000254343	0.0152373	0.0331701	0.0377383	0.0064734	0.0201709
COL14A1	0.0120681	0.0167903	0.0210536	0.0134845	0.0382203

MRPL13	0.5627662	0.4890445	0.5572449	0.6199981	0.4400003
MTBP	0.0244478	0.0318585	0.0200832	0.0407080	0.0434479
SNTB1	0.2526666	0.1396379	0.0821082	0.2412749	0.1890578
ENSG00000272502	0.0192622	0.0068867	0.0083391	0.0187685	0.0000000
ENSG00000253619	0.0007343	0.0000000	0.0000000	0.0000000	0.0000000
HAS2	0.1440976	0.0864269	0.0464319	0.2252719	0.0823427
HAS2-AS1	0.1256692	0.0689260	0.0400199	0.0868537	0.0811406
ENSG00000254303	0.0009253	0.0009037	0.0000000	0.0071933	0.0117629
LINC01151	0.0100982	0.0059835	0.0000000	0.0144416	0.0196485
ENSG00000272384	0.0033947	0.0050050	0.0054732	0.0085432	0.0044975
ZHX2	0.2249035	0.1235755	0.0897381	0.2894224	0.2039568
ENSG00000253372	0.0000000	0.0012174	0.0000000	0.0000000	0.0000000
ENSG00000259631	0.0017752	0.0030804	0.0054596	0.0000000	0.0082614
ENSG00000253607	0.0015208	0.0020315	0.0000000	0.0027814	0.0051973
DERL1	0.2383318	0.2007432	0.2613308	0.2655095	0.1902658
TBC1D31	0.1237694	0.0914268	0.0968844	0.0956840	0.0850867
ENSG00000287657	0.0041928	0.0018337	0.0009118	0.0000000	0.0000000
FAM83A	0.0000000	0.0000000	0.0000000	0.0000000	0.0000000
FAM83A-AS1	0.0006918	0.0000000	0.0000000	0.0000000	0.0014252
C8orf76	0.2256400	0.2004579	0.1331255	0.2174891	0.1575937
ZHX1	0.5287708	0.5099433	0.5079600	0.4713528	0.4382586
ATAD2	0.0558281	0.0722384	0.0301834	0.0974749	0.0365586
NTAQ1	0.1508773	0.2360934	0.2524032	0.1366693	0.1285736
FBXO32	0.0642305	0.0356288	0.0148585	0.0256090	0.0887924
ENSG00000253286	0.0003169	0.0000000	0.0000000	0.0000000	0.0000000
ANXA13	0.0085051	0.0056155	0.0007084	0.0000000	0.0124905
FAM91A1	0.2651484	0.2679047	0.2404433	0.2636553	0.2364862
FER1L6	0.0000000	0.0025163	0.0034022	0.0028110	0.0109210
FER1L6-AS1	0.0011641	0.0000000	0.0000000	0.0000000	0.0000000
FER1L6-AS2	0.0023438	0.0000000	0.0000000	0.0000000	0.0102054
ENSG00000214803	0.0037526	0.0029870	0.0048603	0.0096146	0.0166020
ENSG00000253227	0.0079899	0.0049275	0.0000000	0.0035133	0.0000000
TMEM65	0.7894247	0.7107767	0.7098198	0.7411499	0.7488461
TRMT12	0.1200375	0.0922789	0.0872952	0.1147296	0.0698651
RNF139-DT	0.0128667	0.0121720	0.0078140	0.0068721	0.0261069
RNF139	0.2141928	0.1464472	0.1938712	0.2167575	0.1813283
TATDN1	0.4821459	0.5495025	0.5167552	0.4060596	0.4969505
ENSG00000253106	0.0047430	0.0013594	0.0082147	0.0118365	0.0088932
NDUFB9	0.8091654	0.9501790	1.0384789	0.7709478	0.7049697
MTSS1	0.5938994	0.7746953	0.9141127	0.4679846	0.9577500
ENSG00000255491	0.0011104	0.0000000	0.0000000	0.0000000	0.0068155
LINC00964	0.0070264	0.0000000	0.0042167	0.0022183	0.0024821
ZNF572	0.0107445	0.0233592	0.0042148	0.0232484	0.0368540
SQLE-DT	0.0000000	0.0000000	0.0000000	0.0000000	0.0052853
SQLE	0.7229416	0.6722503	0.8113304	1.0338170	0.6847969
WASHC5	0.1357720	0.0631241	0.0648849	0.1444392	0.1583625
WASHC5-AS1	0.0000000	0.0000000	0.0021106	0.0000000	0.0037333
NSMCE2	0.2730589	0.3483937	0.3213816	0.2976032	0.5566438
TRIB1	0.0301243	0.0254896	0.0328406	0.0173165	0.0024001
LINC02964	0.0012175	0.0021043	0.0000000	0.0041306	0.0049014
LINC00861	0.0018008	0.0014102	0.0000000	0.0039381	0.0053082
LRATD2	0.2652386	0.1530663	0.1006846	0.2570933	0.1444828
PCAT1	0.0167538	0.0257495	0.0247636	0.0215248	0.0505928

ENSG00000253573	0.0000000	0.0011682	0.0000000	0.0000000	0.0026919
CASC19	0.0000000	0.0000000	0.0009265	0.0000000	0.0032430
PRNCR1	0.0014255	0.0055053	0.0000000	0.0053164	0.0056180
CASC8	0.0010253	0.0024015	0.0006749	0.0000000	0.0023981
POU5F1B	0.0011088	0.0000000	0.0000000	0.0000000	0.0000000
CASC11	0.0010252	0.0022665	0.0000000	0.0000000	0.0073924
MYC	0.0768743	0.0759950	0.1092743	0.0538131	0.0506438
PVT1	0.0378874	0.0594804	0.0378425	0.0337575	0.2042127
ENSG00000286034	0.0000000	0.0005808	0.0000000	0.0000000	0.0000000
LINC00824	0.0020724	0.0056925	0.0024239	0.0037958	0.0167140
CCDC26	0.0017589	0.0044134	0.0057147	0.0000000	0.0028343
GSDMC	0.0007128	0.0000000	0.0000000	0.0022183	0.0065249
CYRIB	0.7072313	0.9065070	1.0708105	0.6469158	0.8899246
ENSG00000254263	0.0000000	0.0000000	0.0000000	0.0000000	0.0022413
ENSG00000253720	0.0000000	0.0000000	0.0000000	0.0000000	0.0046750
ENSG00000287195	0.0011576	0.0014407	0.0000000	0.0000000	0.0196076
ASAP1	0.5559893	0.7271014	0.5762948	0.5571721	0.9853176
ASAP1-IT2	0.0030633	0.0028618	0.0020198	0.0000000	0.0034114
ENSG00000286535	0.0141427	0.0070459	0.0131831	0.0000000	0.0086970
ADCY8	0.0518503	0.0837123	0.0871171	0.0337876	0.3125638
ENSG00000253259	0.0012144	0.0000000	0.0000000	0.0000000	0.0055850
ENSG00000253992	0.0000000	0.0000000	0.0000000	0.0000000	0.0048707
EFR3A	0.2571774	0.2083139	0.1971448	0.2167787	0.3770357
OC90	0.0000000	0.0012409	0.0020387	0.0000000	0.0000000
HHLA1	0.0021071	0.0020024	0.0051692	0.0045090	0.0284265
KCNQ3	0.1677739	0.3039104	0.3084696	0.1132051	0.7363919
HPYR1	0.0072707	0.0127341	0.0000000	0.0000000	0.0230213
DNAAF11	0.4607844	0.2991332	0.1728535	0.3929221	0.2908714
TMEM71	0.0115223	0.0085483	0.0037009	0.0057677	0.0231338
PHF20L1	0.5199778	0.4448634	0.3801671	0.5113389	0.4915841
ENSG00000270137	0.0010759	0.0000000	0.0000000	0.0000000	0.0000000
ENSG00000223697	0.0000000	0.0000000	0.0000000	0.0000000	0.0000000
TG	0.0029483	0.0000000	0.0025449	0.0043765	0.0017997
SLA	0.0008338	0.0053598	0.0021947	0.0000000	0.0000000
NDRG1	0.0768099	0.0684440	0.0826737	0.0791319	0.1627945
ST3GAL1	0.0948263	0.2130148	0.1613086	0.0827558	0.2716133
ENSG00000289050	0.0000000	0.0011298	0.0000000	0.0000000	0.0000000
ST3GAL1-DT	0.0017643	0.0035563	0.0033348	0.0000000	0.0057749
ENSG00000288067	0.0000000	0.0000000	0.0000000	0.0000000	0.0000000
ZFAT	0.0168619	0.0337849	0.0292547	0.0149514	0.0967388
ENSG00000289405	0.0261505	0.0183058	0.0207283	0.0099397	0.0586330
NCRNA00250	0.0022597	0.0000000	0.0007062	0.0030663	0.0000000
LINC01591	0.0000000	0.0000000	0.0000000	0.0000000	0.0000000
KHDRBS3	0.9465646	0.9787440	0.9898201	0.8180007	1.0821926
LINC02055	0.0134231	0.0106831	0.0125397	0.0257746	0.0114797
ENSG00000285817	0.0048671	0.0024379	0.0000000	0.0000000	0.0048079
ENSG00000253288	0.0136039	0.0124562	0.0021512	0.0098505	0.0454457
ENSG00000254361	0.0026088	0.0000000	0.0000000	0.0000000	0.0016673
FAM135B	0.1956961	0.2364609	0.1569979	0.1833575	0.7527717
COL22A1	0.0062279	0.0144688	0.0060255	0.0183444	0.0132067
KCNK9	0.0522026	0.0954037	0.1422626	0.0287197	0.1932853
TRAPPC9	0.3000921	0.3289186	0.3681051	0.3003745	0.6621260
PEG13	0.0191528	0.0310766	0.0429334	0.0083280	0.0335431

ENSG00000259891	0.0108612	0.0105812	0.0020718	0.0020875	0.0121112
CHRAC1	0.1897589	0.1486058	0.0986211	0.1666969	0.1361080
AGO2	0.4465060	0.5191279	0.5693638	0.3390596	0.5839891
ERICD	0.0026828	0.0040534	0.0008115	0.0052040	0.0000000
PTK2	0.7117377	0.7108064	0.6080879	0.6522975	1.1972018
DENND3	0.0134783	0.0153019	0.0139749	0.0000000	0.0950549
DENND3-AS1	0.0584615	0.0423467	0.0208894	0.0226547	0.0390136
SLC45A4	0.0388650	0.0443188	0.0462449	0.0262398	0.1255225
ENSG00000244998	0.0002822	0.0000000	0.0000000	0.0000000	0.0000000
PTP4A3	0.1640890	0.1778332	0.1563322	0.1338446	0.1067547
MROH5	0.0000000	0.0000000	0.0000000	0.0000000	0.0000000
ENSG00000271959	0.0000000	0.0000000	0.0021591	0.0000000	0.0000000
C8orf90	0.0000000	0.0022312	0.0013851	0.0000000	0.0000000
ENSG00000287677	0.0000000	0.0000000	0.0032093	0.0017529	0.0101557
ENSG00000287332	0.0000000	0.0000000	0.0027814	0.0058194	0.0016664
TSNARE1	0.0192490	0.0257684	0.0283147	0.0233227	0.1549855
ADGRB1	0.0879152	0.0813065	0.0984758	0.0945596	0.2801624
ARC	0.0156369	0.0215217	0.0124138	0.0108470	0.0377845
JRK	0.0419215	0.0369934	0.0999233	0.0396902	0.0654506
PSCA	0.0000000	0.0000000	0.0000000	0.0000000	0.0000000
LNCOC1	0.0026393	0.0119608	0.0060006	0.0049353	0.0083758
THEM6	0.0883735	0.0906920	0.1067989	0.0530532	0.0641242
ENSG00000253196	0.0000000	0.0000000	0.0000000	0.0000000	0.0000000
SLURP2	0.0075885	0.0108438	0.0021796	0.0084666	0.0114717
LYNX1	0.0517283	0.0568161	0.0541736	0.0746861	0.0944925
LY6E-DT	0.0114128	0.0252686	0.0421279	0.0301410	0.0462831
LY6E	0.1426668	0.2543227	0.2837955	0.1107706	0.1454850
LINC02904	0.0144508	0.0367213	0.0346105	0.0062828	0.0295767
LY6H	1.5326660	1.4222407	1.5668384	1.5842266	1.1627661
ZFP41	0.0427174	0.0443223	0.0529333	0.0338255	0.1373244
GLI4	0.1212129	0.1024389	0.1159314	0.1581834	0.0833392
MINCR	0.1852566	0.1313567	0.0915058	0.1979947	0.1098478
ZNF696	0.0596726	0.0903732	0.0678453	0.0781835	0.0616501
ENSG00000272172	0.0277965	0.0309287	0.0276033	0.0132013	0.0390391
TOP1MT	0.0601993	0.0972063	0.1034348	0.0795270	0.0833497
RHPN1-AS1	0.0060199	0.0037162	0.0031216	0.0175073	0.0154383
RHPN1	0.0605692	0.0340024	0.0328174	0.0977715	0.0479662
LINC02990	0.0052152	0.0077272	0.0017735	0.0067389	0.0032715
MAFA-AS1	0.0180571	0.0344686	0.0204729	0.0239254	0.0142125
MAFA	0.1168617	0.3686647	0.1000096	0.0787729	0.1841815
ZC3H3	0.0584276	0.0856110	0.0407753	0.0290219	0.1602847
ENSG00000287576	0.0049783	0.0036454	0.0091568	0.0026579	0.0085824
GSDMD	0.0077113	0.0009481	0.0008221	0.0000000	0.0000000
MROH6	0.0123423	0.0128975	0.0073707	0.0237737	0.0071423
NAPRT	0.0596172	0.0511555	0.0426729	0.0699913	0.0276774
EEF1D	1.1710217	1.0130056	0.8196012	1.0972991	0.9539030
TIGD5	0.0796218	0.0694389	0.0570597	0.0723058	0.0232528
PYCR3	0.0249248	0.0154965	0.0254818	0.0151635	0.0201399
GFUS	0.1612311	0.1615030	0.1367410	0.1834751	0.1406989
ENSG00000254812	0.0000000	0.0016255	0.0000000	0.0000000	0.0036060
ZNF623	0.0858785	0.0968046	0.0970719	0.0975585	0.1388150
ZNF707	0.0165687	0.0152693	0.0172517	0.0158656	0.0404383
LINC02878	0.0095130	0.0025006	0.0023257	0.0000000	0.0058044

MAPK15	0.0979523	0.0356029	0.0327078	0.1189828	0.0605151
FAM83H	0.0091879	0.0164008	0.0148387	0.0199455	0.0115668
IQANK1	0.0417279	0.0299474	0.0203367	0.0388546	0.0356205
ENSG00000254973	0.0046163	0.0144656	0.0153154	0.0034589	0.0054224
SCRIB	0.0990616	0.1071394	0.0680622	0.1020488	0.0947185
PUF60	0.9134664	0.6646859	0.6913120	0.8731334	0.5655706
ENSG00000287222	0.0694546	0.0753116	0.0966819	0.0994155	0.0584613
NRBP2	0.2592221	0.2080282	0.1683565	0.2446606	0.2819019
EPPK1	0.0495320	0.0688273	0.0564008	0.0545311	0.0824728
ENSG00000288096	0.0172011	0.0111747	0.0063382	0.0082814	0.0212046
PLEC	0.1437281	0.1383193	0.0869108	0.1210434	0.3169040
PARP10	0.0038333	0.0014312	0.0000000	0.0000000	0.0014580
GRINA	0.6110748	0.5736729	0.5799796	0.6769427	0.4480865
SPATC1	0.0000000	0.0014479	0.0000000	0.0000000	0.0038198
OPLAH	0.0013350	0.0015950	0.0008833	0.0000000	0.0035158
ENSG00000255224	0.0063328	0.0054121	0.0102780	0.0118666	0.0000000
EXOSC4	0.1749793	0.1561277	0.1486494	0.1402309	0.1141083
GPAA1	0.5290262	0.4054982	0.4051652	0.5866923	0.3141997
CYC1	0.6204137	0.6697703	0.7946571	0.6055434	0.4466317
SHARPIN	0.2415086	0.1980451	0.2191081	0.3110715	0.2259680
MAF1	0.3577406	0.3056900	0.3137186	0.3493496	0.2323680
WDR97	0.0033603	0.0011280	0.0006194	0.0000000	0.0128735
HGH1	0.0397747	0.0226141	0.0229100	0.0451143	0.0262105
MROH1	0.0562680	0.0587104	0.0521305	0.0618100	0.2357466
BOP1	0.0660727	0.1017862	0.1068487	0.0592346	0.0901954
SCX	0.0119206	0.0152968	0.0301087	0.0183594	0.0216941
HSF1	0.4681107	0.4125522	0.3585593	0.4309498	0.3005144
DGAT1	0.0658959	0.0702952	0.0553219	0.0640141	0.0453693
SCRT1	0.0159177	0.0337166	0.0223376	0.0109029	0.0413463
SLC52A2	0.1300751	0.1381469	0.1329612	0.1445384	0.1178697
TMEM249	0.0035187	0.0003577	0.0000000	0.0000000	0.0036820
FBXL6	0.0260576	0.0255522	0.0250912	0.0246293	0.0275688
ADCK5	0.0223351	0.0270240	0.0220527	0.0185920	0.0462248
CPSF1	0.0885103	0.0632618	0.0564906	0.0895271	0.1064924
ENSG00000272115	0.0057636	0.0009813	0.0072003	0.0069627	0.0114926
SLC39A4	0.0083957	0.0048099	0.0062696	0.0088807	0.0109563
VPS28	0.7997524	0.7925164	0.8695865	0.8039812	0.6149830
TONSL	0.0119625	0.0052133	0.0147467	0.0153040	0.0137651
ZFTRAF1	0.0199253	0.0377651	0.0274526	0.0114665	0.0278773
TMEM276	0.2498248	0.1804018	0.1711455	0.3010281	0.2001395
KIFC2	0.1246699	0.1457958	0.2036966	0.0960498	0.2857305
FOXH1	0.0032283	0.0000000	0.0016797	0.0000000	0.0189432
PPP1R16A	0.2405201	0.1292617	0.1305622	0.2565182	0.1892235
ENSG00000255182	0.0006979	0.0056717	0.0000000	0.0000000	0.0063362
GPT	0.0007811	0.0000000	0.0024615	0.0000000	0.0015201
MFSD3	0.1690548	0.1402657	0.1222911	0.1962016	0.1059911
RECQL4	0.0113599	0.0127133	0.0137101	0.0172537	0.0391412
ENSG00000265393	0.0006686	0.0026917	0.0000000	0.0000000	0.0000000
LRRC14	0.0559081	0.0448469	0.0485897	0.0475650	0.0465860
LRRC24	0.1211623	0.1267232	0.1452047	0.1477455	0.1283818
C8orf82	0.2058040	0.2083650	0.2510219	0.2173958	0.1384734
ARHGAP39	0.1533029	0.1328042	0.1044972	0.1202388	0.4518629
ENSG00000254533	0.0027021	0.0079354	0.0023903	0.0019410	0.0085027

ENSG00000290885	0.0450340	0.0973472	0.0814672	0.0069950	0.0455957
ZNF251	0.1503186	0.1422256	0.1390020	0.1319824	0.2443277
ZNF34	0.0796312	0.0939407	0.0910745	0.0699589	0.0844343
RPL8	2.8723900	2.8775363	2.7274894	2.7852684	2.5822191
ZNF517	0.0459542	0.0369833	0.0394955	0.0407954	0.0309852
ZNF7	0.1113372	0.1126117	0.0956731	0.1023233	0.0969870
COMMD5	0.2630619	0.2245933	0.2361278	0.2827552	0.1684527
ENSG00000286681	0.0035905	0.0071991	0.0049635	0.0014154	0.0324459
ZNF250	0.1342798	0.1522883	0.1275698	0.1165179	0.1998619
ZNF16	0.0522740	0.0576440	0.0412294	0.0579565	0.0809088
ZNF252P	0.1952638	0.2037789	0.1972742	0.2432413	0.2041094
ZNF252P-AS1	0.0000000	0.0000000	0.0000000	0.0000000	0.0000000
C8orf33	0.6223617	0.5389222	0.5794907	0.7034766	0.4339980
WASHC1	0.0505724	0.0708205	0.0590000	0.0496366	0.0615440
MIR1302-9HG	0.0025857	0.0062446	0.0093818	0.0000000	0.0197900
PGM5P3-AS1	0.0000000	0.0000000	0.0000000	0.0000000	0.0000000
FOXD4	0.0015834	0.0000000	0.0053200	0.0000000	0.0034716
ZNG1A	0.1668209	0.1778726	0.1349207	0.1314438	0.1677072
ENSG00000287480	0.0004529	0.0045926	0.0000000	0.0000000	0.0062792
DOCK8	0.0038444	0.0029721	0.0000000	0.0075125	0.0168408
DOCK8-AS2	0.0061361	0.0003466	0.0000000	0.0000000	0.0046818
KANK1	0.1212635	0.1052271	0.0576543	0.1070320	0.2021306
ENSG00000228115	0.0023609	0.0023401	0.0022411	0.0000000	0.0022244
ENSG00000227914	0.0000000	0.0000000	0.0000000	0.0000000	0.0000000
DMRT1	0.0000000	0.0000000	0.0000000	0.0000000	0.0000000
DMRT3	0.0000000	0.0046216	0.0000000	0.0000000	0.0000000
SMARCA2	0.4716228	0.5453564	0.4597913	0.4975024	0.4794497
VLDLR-AS1	0.2588613	0.3481190	0.3167289	0.1860671	0.4405092
VLDLR	0.1475786	0.1946666	0.1442306	0.1284572	0.2062124
ENSG00000286670	0.0006539	0.0024943	0.0000000	0.0000000	0.0000000
KCNV2	0.0051045	0.0044298	0.0097405	0.0031700	0.0049154
PUM3	0.3277565	0.4430640	0.5147515	0.3857787	0.5569826
ENSG00000289552	0.0071675	0.0216024	0.0105588	0.0000000	0.0000000
ENSG00000291284	0.0000000	0.0000000	0.0000000	0.0000000	0.0000000
ENSG00000290482	0.0000000	0.0000000	0.0000000	0.0000000	0.0000000
LINC01231	0.0000000	0.0013282	0.0000000	0.0000000	0.0000000
RFX3	0.9261105	0.7786947	0.6421218	0.8400100	1.1125010
ENSG00000288963	0.0027284	0.0009752	0.0025402	0.0072816	0.0000000
RFX3-DT	0.1772649	0.1600128	0.1030539	0.1263100	0.2453412
ENSG00000237359	0.0022599	0.0008750	0.0023468	0.0000000	0.0000000
ENSG00000226669	0.0000000	0.0000000	0.0000000	0.0000000	0.0000000
GLIS3	0.4014165	0.3037656	0.1733183	0.4345831	0.4651007
GLIS3-AS1	0.0003369	0.0000000	0.0000000	0.0000000	0.0000000
ENSG00000230001	0.0000000	0.0000000	0.0000000	0.0000000	0.0000000
GLIS3-AS2	0.0015884	0.0008112	0.0000000	0.0039942	0.0000000
SLC1A1	0.0848670	0.1447256	0.2497013	0.0424371	0.3046799
SPATA6L	0.0914734	0.0658799	0.0611934	0.0499378	0.1481267
PLPP6	0.0446717	0.0629024	0.0602309	0.0298552	0.0751471
CDC37L1-DT	0.0657662	0.1032337	0.0634990	0.0578827	0.0432196
CDC37L1	0.3515949	0.3663284	0.3754629	0.3379056	0.2931201
AK3	0.3726962	0.3588503	0.3505462	0.3246204	0.5145705
ENSG00000287759	0.0050572	0.0093437	0.0000000	0.0018740	0.0102685
RCL1	0.0431897	0.0576661	0.0502246	0.0538774	0.0675133

JAK2	0.1729411	0.2071388	0.1995294	0.1758414	0.4128622
IGHEP2	0.0000000	0.0000000	0.0000000	0.0000000	0.0000000
INSL6	0.0033718	0.0000000	0.0000000	0.0000000	0.0142297
RLN2	0.0123285	0.0133478	0.0127804	0.0119004	0.0065952
RLN1	0.0047552	0.0055111	0.0057687	0.0000000	0.0037612
ENSG00000272866	0.0000000	0.0000000	0.0000000	0.0000000	0.0000000
PLGRKT	0.2151366	0.2111837	0.2073406	0.2791573	0.1464975
CD274	0.0010719	0.0027166	0.0000000	0.0015611	0.0000000
ENSG00000286162	0.0507152	0.0639885	0.0682058	0.0630513	0.1269320
PDCD1LG2	0.0021557	0.0008930	0.0000000	0.0000000	0.0000000
RIC1	0.0945097	0.1077886	0.0770249	0.0559943	0.2320901
ENSG00000225408	0.0000000	0.0000000	0.0013009	0.0000000	0.0000000
ERMP1	0.0780313	0.0722276	0.0557831	0.1134067	0.1348207
KIAA2026	0.3303319	0.3360424	0.3887196	0.2821444	0.4832958
MLANA	0.0000000	0.0009847	0.0000000	0.0000000	0.0030362
RANBP6	0.3343951	0.4286871	0.4759972	0.2991922	0.3250645
IL33	0.0368901	0.0136575	0.0046588	0.1054768	0.0044787
UHRF2	0.1900168	0.1874323	0.1550811	0.1472980	0.3444961
ENSG00000233367	0.0017825	0.0021081	0.0058513	0.0000000	0.0016619
GLDC	0.1266126	0.1052273	0.1123024	0.1907330	0.1969085
ENSG00000236924	0.0595270	0.0549089	0.0387225	0.0855094	0.0326426
LINC02851	0.0046478	0.0013211	0.0010864	0.0031392	0.0018550
ENSG00000225489	0.0145645	0.0084631	0.0202924	0.0135519	0.0055566
KDM4C	0.1575720	0.1996162	0.1159961	0.1321122	0.5751381
ENSG00000285688	0.0037195	0.0023166	0.0020426	0.0000000	0.0101686
ENSG00000288042	0.0000000	0.0000000	0.0000000	0.0000000	0.0000000
ENSG00000273056	0.0009810	0.0000000	0.0000000	0.0000000	0.0000000
DMAC1	0.8037327	0.7494801	0.7548779	0.8255153	0.5725286
PTPRD	0.9352231	1.1724011	1.0585835	0.8621910	2.4714019
ENSG00000235389	0.0000000	0.0008967	0.0038385	0.0000000	0.0000000
PTPRD-AS1	0.0345058	0.0917168	0.1338945	0.0579198	0.0324422
ENSG00000234021	0.0014572	0.0041175	0.0050064	0.0000000	0.0323177
ENSG00000230920	0.0000000	0.0000000	0.0000000	0.0000000	0.0136066
PTPRD-DT	0.0010727	0.0047434	0.0013419	0.0023370	0.0121606
ENSG00000285784	0.0018342	0.0014825	0.0000000	0.0073109	0.0000000
TYRP1	0.0080569	0.0041526	0.0042806	0.0095451	0.0000000
LURAP1L-AS1	0.0432743	0.0635837	0.0680455	0.0267367	0.1264269
LURAP1L	0.2684098	0.1723206	0.1432136	0.2022278	0.2125606
ENSG00000285637	0.0000000	0.0008825	0.0057656	0.0022936	0.0000000
MPDZ	0.6794741	0.4302143	0.3734463	0.6742738	0.5625719
ENSG00000234740	0.0073104	0.0076482	0.0064201	0.0149968	0.0000000
ENSG00000226197	0.0079007	0.0058685	0.0121171	0.0073611	0.0147901
NFIB	1.0995625	0.6052035	0.3676513	1.1538988	0.7389976
ENSG00000288876	0.0012829	0.0026036	0.0059468	0.0000000	0.0127906
NFIB-AS1	0.0246160	0.0106391	0.0084137	0.0560393	0.0194157
ENSG00000287708	0.0044954	0.0000000	0.0040102	0.0017930	0.0061813
ENSG00000272871	0.0020846	0.0000000	0.0011369	0.0000000	0.0062535
ZDHHC21	0.2506851	0.2844812	0.2806259	0.2881646	0.4563995
FREM1	0.0951593	0.0485599	0.0362629	0.1253462	0.0937742
ENSG00000291185	0.0645100	0.0750797	0.0905052	0.0437814	0.1460315
TTC39B	0.0940990	0.1520472	0.1360891	0.0918434	0.2472691
SNAPC3	0.5242573	0.4659622	0.4777175	0.5428483	0.4409733
PSIP1	1.4063959	1.5606671	1.7010817	1.3386054	1.3829370

CCDC171	0.1931826	0.2133781	0.1388393	0.1599953	0.4329353
LINC03041	0.0057956	0.0000000	0.0037823	0.0050066	0.0061992
BNC2	0.2849083	0.3157970	0.1424463	0.1698480	0.4943494
BNC2-AS1	0.0277496	0.0233890	0.0082705	0.0167342	0.0263782
ENSG00000237153	0.0000000	0.0010939	0.0000000	0.0000000	0.0076315
CNTLN	0.3854476	0.2512527	0.2103675	0.2496763	0.3632812
SH3GL2	0.2360362	0.3034183	0.3883154	0.3177232	0.3555398
ADAMTSL1	0.4253063	0.4310371	0.3050585	0.5126187	1.2851623
SAXO1	0.0129734	0.0121350	0.0000000	0.0272014	0.0369547
RRAGA	0.6943954	0.5853464	0.6953813	0.7350388	0.4038684
HAUS6	0.3640294	0.2437025	0.2171257	0.3217227	0.1939756
PLIN2	0.1068837	0.1012592	0.1124319	0.0855433	0.1091874
DENND4C	0.2076390	0.1845473	0.1140646	0.2028210	0.4646844
ENSG00000232978	0.0000000	0.0000000	0.0014637	0.0000000	0.0000000
ENSG00000272842	0.0013131	0.0024065	0.0000000	0.0021965	0.0041410
ENSG00000273226	0.0047189	0.0014253	0.0000000	0.0045021	0.0031804
RPS6	3.0987390	2.9125483	2.6459949	2.9967050	2.6951693
ENSG00000285911	0.0000000	0.0038975	0.0000000	0.0000000	0.0059207
ACER2	0.0119991	0.0159029	0.0146845	0.0028580	0.0472888
ENSG00000260912	0.0042231	0.0061552	0.0087593	0.0000000	0.0122287
SLC24A2	0.0806705	0.1024139	0.1314522	0.0971222	0.2034332
ENSG00000286685	0.0013171	0.0032587	0.0007062	0.0000000	0.0257877
ENSG00000261402	0.0000000	0.0044399	0.0083041	0.0022440	0.0073599
MLLT3	0.6581609	0.7311051	0.9176694	0.5631833	1.0306641
FOCAD	0.2581030	0.2604623	0.2225972	0.2813439	0.7320777
FOCAD-AS1	0.0035879	0.0000000	0.0021127	0.0000000	0.0000000
HACD4	0.0126257	0.0164761	0.0058450	0.0160417	0.0025761
KLHL9	0.4552914	0.5013925	0.5615890	0.4343046	0.4155982
MIR31HG	0.0069595	0.0027420	0.0025528	0.0022936	0.0094966
MTAP	0.2550892	0.2298151	0.2503377	0.2841685	0.2441103
ENSG00000265194	0.0000000	0.0000000	0.0000000	0.0000000	0.0064270
CDKN2A	0.0039649	0.0000000	0.0025512	0.0013130	0.0000000
CDKN2B-AS1	0.0069379	0.0080609	0.0110758	0.0182900	0.0079043
CDKN2B	0.0226357	0.0123118	0.0086412	0.0064291	0.0000000
DMRTA1	0.1380203	0.0630420	0.0341937	0.1314504	0.0792009
LINC01239	0.0036617	0.0032355	0.0000000	0.0097600	0.0047971
ENSG00000284418	0.0026105	0.0068552	0.0026961	0.0044743	0.0277689
ENSG00000283982	0.0964675	0.1265638	0.0540304	0.1029006	0.5713446
ENSG00000233906	0.0000000	0.0010897	0.0000000	0.0000000	0.0000000
ELAVL2	0.6504513	1.1027888	1.1668934	0.5091692	1.2691169
ENSG00000287843	0.0018198	0.0040793	0.0000000	0.0000000	0.0000000
ENSG00000231460	0.0060054	0.0104196	0.0126769	0.0072508	0.0170122
ENSG00000287139	0.0186617	0.0403685	0.0046258	0.0084352	0.0209933
IZUMO3	0.0049788	0.0026491	0.0051060	0.0020852	0.0000000
ENSG00000287318	0.0006331	0.0000000	0.0000000	0.0000000	0.0000000
TUSC1	0.2008724	0.2084187	0.2349605	0.1552614	0.1679946
LINC01241	0.0000000	0.0000000	0.0000000	0.0000000	0.0000000
ENSG00000276759	0.0000000	0.0000000	0.0000000	0.0000000	0.0101495
CAAP1	0.3766316	0.2270234	0.1877250	0.3889738	0.2979518
PLAA	0.3236534	0.2755446	0.2185128	0.2865913	0.2584275
IFT74	0.5190382	0.3158588	0.2406817	0.4886178	0.2731614
IFT74-AS1	0.0050065	0.0023542	0.0026438	0.0040247	0.0047917
LRRC19	0.0000000	0.0000000	0.0000000	0.0000000	0.0047864

ENSG00000254396	0.0007199	0.0000000	0.0065247	0.0000000	0.0000000
TEK	0.0024801	0.0024254	0.0000000	0.0100633	0.0053413
LINC00032	0.0027881	0.0045091	0.0000000	0.0028893	0.0000000
EQTN	0.0014910	0.0000000	0.0000000	0.0000000	0.0000000
MOB3B	0.0687847	0.0498556	0.0330347	0.0347755	0.1395645
ENSG00000285103	0.0025226	0.0000000	0.0000000	0.0000000	0.0044564
C9orf72	0.2969868	0.3004328	0.3119430	0.2611248	0.3486227
LINGO2	0.4816093	0.5855979	0.4661021	0.3734619	1.6940775
ENSG00000287551	0.0009612	0.0009725	0.0000000	0.0000000	0.0024068
ENSG00000287038	0.0082636	0.0060734	0.0078148	0.0095501	0.0076810
ACO1	0.2760912	0.1905461	0.2031911	0.2506187	0.1736872
RIGI	0.0311349	0.0228647	0.0222951	0.0581231	0.0589927
TOPORS	0.2130413	0.1879759	0.2207835	0.2066128	0.2529784
SMIM27	0.3355022	0.3030476	0.2681853	0.2870700	0.2478454
NDUFB6	0.8787995	0.8751709	1.0071849	0.8845652	0.7347916
ENSG00000231193	0.0000000	0.0000000	0.0000000	0.0000000	0.0000000
APTX	0.2642296	0.2936324	0.2672987	0.2633385	0.2851727
DNAJA1	1.7978554	1.8849876	2.1070029	1.7916759	1.6359596
SMU1	0.6450959	0.5319737	0.5404825	0.6505019	0.5081220
B4GALT1	0.1188941	0.0659462	0.0284653	0.1027529	0.0941502
B4GALT1-AS1	0.0094579	0.0136063	0.0131307	0.0083326	0.0000000
SPINK4	0.0000000	0.0000000	0.0000000	0.0000000	0.0000000
BAG1	0.6007533	0.6182378	0.6551000	0.5344350	0.5529469
CHMP5	0.9638286	0.9816305	1.1503226	0.9507607	0.7537676
NFX1	0.2385334	0.2100384	0.1782247	0.2115967	0.3434280
AQP7	0.0000000	0.0000000	0.0012337	0.0000000	0.0094464
AQP3	0.0076073	0.0020533	0.0028830	0.0000000	0.0000000
NOL6	0.0200947	0.0331601	0.0255767	0.0151996	0.0161573
ANKRD18B	0.0026219	0.0107880	0.0094291	0.0070144	0.0155603
PTENP1-AS	0.0366277	0.0420681	0.0481956	0.0121917	0.0354164
TRBV26OR9-2	0.0081101	0.0139230	0.0100523	0.0082321	0.0089492
ENSG00000260947	0.0178863	0.0302171	0.0342312	0.0187735	0.0336789
ENSG00000287063	0.0047020	0.0039106	0.0018182	0.0000000	0.0015201
ENSG00000227301	0.0000000	0.0000000	0.0000000	0.0000000	0.0000000
UBE2R2-AS1	0.0040477	0.0077483	0.0112041	0.0028622	0.0218624
PRSS3	0.0095846	0.0187519	0.0029714	0.0023493	0.0000000
UBE2R2	0.9703302	0.9195147	0.9273176	0.9231719	0.8598086
UBAP2	0.3102236	0.3137928	0.4185766	0.2859674	0.5330265
ENSG00000288949	0.0000000	0.0000000	0.0036148	0.0000000	0.0000000
ENSG00000228352	0.0000000	0.0000000	0.0009315	0.0000000	0.0000000
DCAF12	0.2830066	0.2376604	0.3261433	0.2909339	0.2348718
UBAP1	0.4110706	0.3524466	0.3297581	0.4314687	0.4790050
KIF24	0.0314745	0.0193408	0.0152131	0.0454367	0.0440074
NUDT2	0.3571080	0.3461173	0.3600079	0.3601347	0.3086609
MYORG	0.0340132	0.0162460	0.0232112	0.0330906	0.0154560
C9orf24	0.8030184	0.5078562	0.3811730	0.8841872	0.5483128
FAM219A	0.3289902	0.5193657	0.5083303	0.2764796	0.5154888
DNAI1	0.0515313	0.0450359	0.0237208	0.0529079	0.0361856
ENHO	0.3635440	0.4493909	0.5182499	0.4557389	0.3373531
CNTFR	0.2262932	0.3967766	0.3851815	0.1794042	0.3781037
CNTFR-AS1	0.0007030	0.0000000	0.0038369	0.0040755	0.0000000
RPP25L	0.1240444	0.0740598	0.0584649	0.1507296	0.0649942
DCTN3	0.9245176	1.0596230	1.1806281	0.9038283	0.7940613

ARID3C	0.0117107	0.0255398	0.0353130	0.0068770	0.0289853
SIGMAR1	0.4111718	0.3617444	0.3487789	0.4884118	0.2511685
GALT	0.1387363	0.1329218	0.1206122	0.1619059	0.0748748
IL11RA	0.0541427	0.0593764	0.0387758	0.0703010	0.0475061
CCL27	0.0166399	0.0168246	0.0372538	0.0011506	0.0101083
ENSG00000187186	0.2194762	0.2224239	0.2741001	0.1934026	0.1361468
ENSG00000230074	0.0724358	0.0871059	0.0445716	0.0951791	0.0670050
ENSG00000288583	0.0045445	0.0103644	0.0000000	0.0055470	0.0161961
ENSG00000287368	0.0010816	0.0021934	0.0000000	0.0023684	0.0088133
PHF24	0.0588763	0.1396901	0.1547408	0.0306475	0.1279611
DNAJB5-DT	0.0024770	0.0013510	0.0045090	0.0000000	0.0069764
DNAJB5	0.1653546	0.1868703	0.2352232	0.1794013	0.1940888
ENSG00000286782	0.0002971	0.0026508	0.0000000	0.0000000	0.0100197
ENSG00000287868	0.0027455	0.0044154	0.0026908	0.0016011	0.0000000
VCP	0.8929936	0.7747812	0.7523036	0.9115586	0.7607897
FANCG	0.0520155	0.0411162	0.0486784	0.0645312	0.0375786
PIGO	0.0822289	0.0465655	0.0462878	0.1517105	0.0607072
PIGO-AS1	0.0050356	0.0021372	0.0000000	0.0023988	0.0000000
STOML2	0.6381333	0.5968434	0.5738341	0.5870318	0.4085500
ATOSB	0.0873887	0.1059872	0.1032458	0.0753610	0.1355090
UNC13B	0.1328279	0.1286135	0.0922571	0.1592612	0.2412702
ATP8B5P	0.0179704	0.0143630	0.0028204	0.0120699	0.0093937
RUSC2	0.1693119	0.2613720	0.2833993	0.1213630	0.3224565
FAM166B	0.0699196	0.0475296	0.0172722	0.0763381	0.0242494
ENSG00000288586	0.0069972	0.0093243	0.0109709	0.0000000	0.0023834
TESK1	0.0944561	0.1156771	0.1512533	0.0798041	0.0980861
CD72	0.0127530	0.0044398	0.0062374	0.0248015	0.0175824
CCDC107	0.3274691	0.3315119	0.4144533	0.3752006	0.1892746
ARHGEF39	0.0031355	0.0088763	0.0000000	0.0076120	0.0159902
CA9	0.0065461	0.0029208	0.0063969	0.0076797	0.0103210
TPM2	0.0638568	0.0564126	0.0521296	0.0523482	0.0848791
TLN1	0.6938316	0.4954071	0.4561506	0.7797137	0.4628711
CREB3	0.4165409	0.3504490	0.3570450	0.3797957	0.2163805
GBA2	0.0879529	0.1657181	0.1707615	0.0816960	0.1776564
RGP1	0.1278575	0.1234692	0.1354979	0.1300574	0.1754708
MSMP	0.0059403	0.0081669	0.0076094	0.0000000	0.0129192
ENSG00000228843	0.0000000	0.0010146	0.0028843	0.0000000	0.0000000
ENSG00000227388	0.0127589	0.0142026	0.0105438	0.0173359	0.0451520
NPR2	0.0193237	0.0150877	0.0124818	0.0201626	0.0282128
SPAG8	0.0999513	0.0552691	0.0481800	0.1113170	0.0390417
HINT2	0.4027593	0.2873749	0.2618432	0.3450494	0.2738232
TMEM8B	0.2851466	0.2597144	0.2510619	0.2554471	0.2337858
FAM221B	0.0016747	0.0000000	0.0000000	0.0000000	0.0102830
HRCT1	0.0018260	0.0017326	0.0000000	0.0000000	0.0000000
SPAAR	0.0021882	0.0034311	0.0000000	0.0000000	0.0000000
RECK	0.1452090	0.1038085	0.0858639	0.1373975	0.2552274
GLIPR2	0.4859508	0.3657093	0.2295802	0.5817093	0.2793012
CLTA	1.4171455	1.4493699	1.5173482	1.3896314	1.1485891
GNE	0.1385742	0.1904100	0.1371089	0.1123093	0.1435426
RNF38	0.3783550	0.4261512	0.3847141	0.2948214	0.5067447
MELK	0.0142540	0.0144355	0.0086313	0.0111272	0.0069003
ENSG00000288571	0.0000000	0.0000000	0.0000000	0.0000000	0.0000000
ENSG00000287514	0.0000000	0.0000000	0.0000000	0.0000000	0.0000000

PAX5	0.0020484	0.0044918	0.0123247	0.0052040	0.0232658
ENSG00000260100	0.0000000	0.0034525	0.0024354	0.0000000	0.0000000
EBLN3P	0.4631992	0.5168992	0.5154922	0.4305218	0.3357584
ENSG00000233242	0.0025938	0.0025713	0.0023309	0.0015404	0.0000000
ZCCHC7	0.5437515	0.5057127	0.4937633	0.4659828	0.7739218
GRHPR	0.6265494	0.6347853	0.5746172	0.6082069	0.4436292
ZBTB5	0.0766570	0.1065300	0.1504601	0.0534937	0.0880160
POLR1E	0.1556965	0.1987815	0.1813267	0.1628533	0.1548509
ENSG00000234160	0.0099614	0.0120499	0.0124268	0.0032089	0.0000000
FBXO10	0.0610385	0.0595411	0.0447790	0.0587050	0.1032818
TOMM5	0.8209445	0.7612269	0.7633677	0.8885482	0.6057772
FRMPD1	0.0052992	0.0068402	0.0152879	0.0026518	0.0047971
TRMT10B	0.1485800	0.1239797	0.0729670	0.1549665	0.1514147
EXOSC3	0.2220927	0.2173576	0.2508489	0.2254463	0.2712027
ENSG00000289295	0.0076717	0.0042886	0.0000000	0.0090921	0.0129796
DCAF10	0.3286865	0.2826612	0.2548390	0.3487943	0.4128535
SLC25A51	0.2076163	0.2796511	0.2502226	0.1980092	0.2448296
SHB	0.0893685	0.1403294	0.1838398	0.0660282	0.2444555
ALDH1B1	0.0716583	0.0940007	0.1514371	0.0801908	0.0831535
IGFBPL1	0.0521513	0.0858857	0.1356843	0.0332528	0.0588222
ENSG00000291061	0.0029299	0.0112409	0.0023415	0.0000000	0.0587116
ANKRD18A	0.0294090	0.0338882	0.0383526	0.0268968	0.0417964
ENSG00000290907	0.0000000	0.0000000	0.0000000	0.0000000	0.0000000
FAM201A	0.0081962	0.0048414	0.0162027	0.0183235	0.0048405
ENSG00000228467	0.0000000	0.0000000	0.0000000	0.0000000	0.0063087
ENSG00000287838	0.0052267	0.0116482	0.0176734	0.0116015	0.0025607
CNTNAP3	0.0512998	0.0616135	0.0409394	0.0835151	0.0816840
ENSG00000290717	0.0000000	0.0009070	0.0000000	0.0028850	0.0036615
GLIDR	0.0470568	0.0367815	0.0460663	0.0435404	0.0346546
ENSG00000291181	0.0420384	0.0516837	0.0469492	0.0501358	0.1595899
ENSG00000283886	0.0350933	0.0319176	0.0366417	0.0426393	0.0082637
ENSG00000240240	0.0045235	0.0035786	0.0066320	0.0057219	0.0044834
ENSG00000290969	0.0000000	0.0019430	0.0000000	0.0000000	0.0000000
BMS1P14	0.0016520	0.0009591	0.0024688	0.0044097	0.0032018
ENSG00000289843	0.0065502	0.0069794	0.0272372	0.0035433	0.0070510
ENSG00000275390	0.0018695	0.0000000	0.0000000	0.0000000	0.0000000
ENSG00000291075	0.4073466	0.5332895	0.6380305	0.4092094	0.4948009
PGM5P2	0.0113709	0.0189273	0.0086118	0.0031392	0.0201265
ZNG1F	0.0189433	0.0233182	0.0389136	0.0225296	0.0179318
PTGER4P2-CDK2AP2P	0.0135448	0.0189154	0.0384608	0.0044571	0.0182054
ENSG00000290848	0.0234493	0.0172206	0.0108238	0.0095020	0.0640202
ENSG00000288891	0.1536415	0.1833159	0.1505539	0.1092976	0.4980028
ENSG00000276412	0.0000000	0.0026095	0.0011774	0.0000000	0.0000000
FAM242F	0.0000000	0.0000000	0.0000000	0.0025371	0.0000000
ENSG00000275297	0.0003758	0.0028392	0.0000000	0.0000000	0.0000000
CNTNAP3B	0.0914263	0.0957698	0.0708573	0.1152865	0.2635672
ENSG00000275649	0.0000000	0.0016527	0.0022436	0.0000000	0.0000000
FAM88E	0.0000000	0.0041639	0.0000000	0.0033942	0.0000000
ENSG00000291170	0.0114281	0.0062823	0.0100394	0.0027800	0.0036741
ENSG00000291166	0.0173655	0.0093780	0.0047098	0.0075295	0.0326242
ENSG00000275676	0.0065487	0.0054980	0.0000000	0.0000000	0.0072786
CNTNAP3C	0.0030154	0.0039551	0.0024897	0.0019693	0.0098556
ENSG00000204814	0.0010015	0.0000000	0.0000000	0.0000000	0.0000000

FAM27C	0.3853985	0.3009127	0.4132368	0.3753891	0.2741270
ENSG00000223379	0.0000000	0.0000000	0.0000000	0.0000000	0.0000000
ENSG00000231212	0.0019168	0.0055178	0.0029296	0.0000000	0.0071570
FAM88C	0.0123302	0.0252195	0.0081355	0.0063657	0.0195201
ENSG00000291011	0.2417826	0.2649887	0.3165335	0.1888585	0.1686006
LINC01189	0.0000000	0.0000000	0.0000000	0.0000000	0.0000000
FAM88F	0.0000000	0.0005601	0.0041949	0.0036873	0.0000000
ENSG00000288838	0.0204466	0.0222280	0.0277017	0.0088334	0.0557886
LINC01410	0.0014217	0.0047616	0.0052222	0.0032068	0.0054244
ENSG00000287168	0.0221677	0.0384996	0.0492778	0.0100573	0.0954700
ENSG00000229422	0.0028532	0.0036303	0.0102050	0.0082795	0.0084181
LERFS	0.0087496	0.0095069	0.0051604	0.0025617	0.0276809
FAM88B	0.0126730	0.0225484	0.0195066	0.0186187	0.0361965
ENSG00000290549	0.0247508	0.0327372	0.0288317	0.0344566	0.0447467
ENSG00000225411	0.0000000	0.0000000	0.0000000	0.0000000	0.0000000
FLJ43315	0.0000000	0.0000000	0.0000000	0.0000000	0.0052918
ANKRD20A5P	0.0026633	0.0078110	0.0059434	0.0058194	0.0107151
FOXD4L5	0.0000000	0.0012208	0.0010558	0.0018058	0.0032430
ENSG00000287329	0.0013994	0.0011443	0.0000000	0.0000000	0.0199745
ZNG1E	0.0714637	0.0602190	0.0862587	0.0825772	0.1403412
ENSG00000274893	0.0110772	0.0084965	0.0110054	0.0000000	0.0129113
ENSG00000290971	0.0000000	0.0023920	0.0009177	0.0000000	0.0046646
ZNF658	0.0506544	0.0433338	0.0507937	0.0563266	0.0199842
ENSG00000277350	0.0033636	0.0029755	0.0000000	0.0000000	0.0000000
FAM27E3	0.0473545	0.0491073	0.0341150	0.0512847	0.0570110
ANKRD20A1	0.0011952	0.0093850	0.0014107	0.0048860	0.0100779
ZNG1C	0.0217933	0.0421188	0.0311973	0.0249945	0.0490791
FOXD4L3	0.0000000	0.0007970	0.0031433	0.0000000	0.0000000
PGM5	0.0011435	0.0000000	0.0053059	0.0038769	0.0212027
ENSG00000233178	0.0085986	0.0226022	0.0095664	0.0061823	0.0172397
TMEM252-DT	0.0000000	0.0012671	0.0041567	0.0000000	0.0048735
PIP5K1B	0.0189086	0.0180418	0.0218094	0.0120182	0.0270112
PABIR1	0.2561722	0.2932345	0.2798596	0.2840917	0.1947432
FXN	0.1486545	0.1746151	0.1688009	0.1535591	0.1152316
TJP2	0.0252176	0.0353559	0.0106411	0.0151445	0.0714295
ENTREP1	0.0233311	0.0410490	0.0205968	0.0269925	0.1210886
APBA1	0.1929140	0.2909791	0.3699105	0.1430256	0.7892682
ENSG00000229312	0.0000000	0.0000000	0.0000000	0.0000000	0.0000000
ENSG00000261447	0.0011371	0.0033416	0.0061114	0.0000000	0.0000000
PTAR1	0.3531341	0.3271913	0.2303310	0.3628368	0.3147769
CFAP95	0.0269594	0.0218263	0.0052176	0.0025449	0.0059085
MAMDC2-AS1	0.0374502	0.0243558	0.0234338	0.0300854	0.0968613
MAMDC2	0.0157334	0.0136611	0.0058781	0.0053246	0.0147237
SMC5-DT	0.0314401	0.0442255	0.0325348	0.0356294	0.0722910
SMC5	0.4531954	0.4113972	0.3866015	0.3572011	0.6222056
ENSG00000274421	0.0012471	0.0014537	0.0032143	0.0000000	0.0175253
KLF9	0.1026996	0.0516083	0.0453297	0.0958912	0.0354924
ENSG00000289159	0.0054004	0.0015486	0.0000000	0.0000000	0.0000000
TRPM3	0.1470849	0.2176467	0.2241347	0.1756499	0.6738611
CEMIP2	0.1366716	0.0947558	0.0879651	0.1854860	0.2114256
ABHD17B	0.1498007	0.1615129	0.1574546	0.1338552	0.2099963
C9orf85	0.1834412	0.1698426	0.2111737	0.1846722	0.1727057
C9orf57	0.0000000	0.0000000	0.0000000	0.0000000	0.0000000

ENSG00000286823	0.0023067	0.0000000	0.0016443	0.0000000	0.0462804
GDA	0.0557938	0.0245980	0.0088201	0.0592961	0.0544456
LINC01504	0.0034386	0.0084087	0.0030460	0.0055154	0.0047828
ZFAND5	0.9495259	0.9721043	0.9939966	0.9030664	0.8273716
ENSG00000288865	0.0008172	0.0000000	0.0000000	0.0000000	0.0061992
TMC1	0.0161030	0.0144419	0.0039371	0.0032073	0.0140739
ENSG00000289993	0.0194026	0.0116960	0.0107717	0.0225935	0.0000000
ALDH1A1	0.0069496	0.0012839	0.0151677	0.0110471	0.0067935
ANXA1	1.4855858	0.8678596	0.5698529	1.6606028	0.8821609
RORB-AS1	0.0065045	0.0046019	0.0006315	0.0000000	0.0000000
RORB	0.0410614	0.0287143	0.0374175	0.0249395	0.0679947
TRPM6	0.0000000	0.0027125	0.0025783	0.0000000	0.0033842
C9orf40	0.1352579	0.1819832	0.2040973	0.1724590	0.1642994
CARNMT1-AS1	0.0127252	0.0251098	0.0194492	0.0109083	0.0404144
CARNMT1	0.1716172	0.1400776	0.1171175	0.1563039	0.1349571
ENSG00000229587	0.0057354	0.0051967	0.0031941	0.0086947	0.0000000
NMRK1	0.2163128	0.2140520	0.1790702	0.1960161	0.1917519
OSTF1	0.2209155	0.2466610	0.2798581	0.2018851	0.1486158
PCSK5	0.0661455	0.0637768	0.0373008	0.1197308	0.1653616
RFK	0.7162024	0.9627580	1.2958158	0.6675639	0.6580998
GCNT1	0.0372736	0.0168023	0.0142275	0.0171933	0.0196474
PRUNE2	0.1777812	0.2310711	0.1796777	0.1603902	0.3833621
PCA3	0.0008557	0.0049675	0.0048750	0.0079275	0.0281943
VPS13A	0.1951254	0.2311970	0.2073028	0.1361068	0.3969156
GNA14	0.0029519	0.0124329	0.0009839	0.0000000	0.0066258
GNA14-AS1	0.0000000	0.0009476	0.0000000	0.0000000	0.0015298
GNAQ	0.8825204	1.0517706	1.0839636	0.7853276	1.3703971
ENSG00000289440	0.0289305	0.0114638	0.0200324	0.0251515	0.0290745
CEP78	0.2032333	0.2406690	0.2405836	0.2550276	0.3610836
PSAT1	1.3102457	0.8660805	0.6250823	1.4943103	0.7313478
ENSG00000260995	0.0025283	0.0000000	0.0018260	0.0000000	0.0018081
TLE4	0.2346781	0.4743839	0.5547872	0.1554047	0.6891178
TLE1	0.4076007	0.3540356	0.3740634	0.4495569	0.3769560
TLE1-DT	0.0100896	0.0105927	0.0096904	0.0089464	0.0361371
ENSG00000267559	0.0021894	0.0005109	0.0020099	0.0000000	0.0031362
ENSG00000290551	0.0025103	0.0053161	0.0117964	0.0034082	0.0163856
RASEF	0.0089574	0.0069653	0.0015113	0.0083462	0.0144825
ENSG00000286451	0.0000000	0.0003531	0.0023656	0.0022431	0.0000000
FRMD3-AS1	0.0035103	0.0141239	0.0160434	0.0251294	0.0184280
FRMD3	0.2219964	0.3046111	0.3247964	0.2154819	0.4581349
ENSG00000237529	0.0113035	0.0057966	0.0050993	0.0048099	0.0036428
IDNK	0.1376858	0.1089354	0.0590200	0.1200583	0.0899032
UBQLN1	0.6215121	0.6072359	0.6856329	0.5741245	0.6189127
UBQLN1-AS1	0.1041279	0.0842209	0.0559460	0.0804076	0.0330997
GKAP1	0.4685420	0.4859727	0.5690410	0.4051836	0.3746966
ENSG00000226877	0.0010303	0.0000000	0.0027173	0.0000000	0.0082988
ENSG00000231616	0.0012269	0.0017589	0.0023575	0.0000000	0.0059357
KIF27	0.4343550	0.3316537	0.2483480	0.4491373	0.3667478
C9orf64	0.0527517	0.0248802	0.0282131	0.0712135	0.0386469
HNRNPK	1.5261207	1.3453424	1.4221156	1.5304231	1.2090300
HNRNPK-AS1	0.0006041	0.0000000	0.0000000	0.0000000	0.0000000
RMI1	0.1998684	0.2201598	0.2546917	0.1662451	0.1943477
ENSG00000227463	0.0004850	0.0056815	0.0008618	0.0000000	0.0060218

SLC28A3-AS1	0.0000000	0.0000000	0.0000000	0.0000000	0.0061992
ENSG00000285987	0.0013027	0.0012561	0.0000000	0.0000000	0.0000000
NTRK2	1.8455759	1.3173183	1.0968476	2.5359898	1.5056064
ENSG00000285556	0.0000000	0.0000000	0.0000000	0.0000000	0.0000000
ENSG00000285634	0.0046554	0.0026009	0.0000000	0.0019606	0.0103238
AGTPBP1	0.7183872	1.0270314	1.0813373	0.5801542	1.0835688
ENSG00000230303	0.0271097	0.0254830	0.0157123	0.0125334	0.0362545
ENSG00000290924	0.1180495	0.0989485	0.1516949	0.0674317	0.1769025
ENSG00000289214	0.0025744	0.0014000	0.0011788	0.0028110	0.0000000
NAA35	0.4035007	0.4579187	0.4033932	0.3938803	0.4921811
GOLM1	0.9532891	0.7783531	0.8198517	1.2205562	0.9320444
C9orf153	0.0076878	0.0153823	0.0018309	0.0000000	0.0545841
ISCA1	0.4455343	0.4722107	0.5517438	0.4628095	0.3854961
TUT7	0.2721663	0.2711928	0.2971508	0.2717963	0.3145463
ENSG00000235819	0.0000000	0.0000000	0.0000000	0.0000000	0.0000000
GAS1	0.0061007	0.0047674	0.0181453	0.0073400	0.0000000
GAS1RR	0.0048870	0.0034244	0.0012851	0.0000000	0.0000000
LINC02893	0.0186170	0.0235757	0.0166762	0.0075579	0.0070168
DAPK1	0.2886101	0.3189856	0.2562266	0.2690706	0.6573797
DAPK1-IT1	0.0009891	0.0000000	0.0016423	0.0000000	0.0100195
CTSL	0.6359160	0.4307700	0.3655570	1.0279049	0.4293505
CDK20	0.0875951	0.0867763	0.0489445	0.1237849	0.0704588
LINC03026	0.0198809	0.0325076	0.0318179	0.0184193	0.0384863
ENSG00000287769	0.0281908	0.0374706	0.0351296	0.0294849	0.0205376
SPIN1	0.8462388	1.0186226	1.0997619	0.7241146	0.9143016
NXNL2	0.0826529	0.0637992	0.0306945	0.1251950	0.0656165
ENSG00000286414	0.0000000	0.0020481	0.0000000	0.0000000	0.0000000
S1PR3	0.4606781	0.2445381	0.1524687	0.6835953	0.2068589
SHC3	0.1001479	0.1209859	0.1809763	0.1175565	0.2305350
ENSG00000224945	0.0023924	0.0044172	0.0019501	0.0011920	0.0000000
CKS2	0.1919718	0.1634962	0.1382457	0.1781686	0.1618532
SECISBP2	0.4238770	0.3722618	0.3174508	0.3989695	0.3856454
SEMA4D	0.1030452	0.1468201	0.1823450	0.0969013	0.3314831
ENSG00000285907	0.0130872	0.0083545	0.0065378	0.0015620	0.0105781
GADD45G	0.2835014	0.1744630	0.1254809	0.2232923	0.1745618
LINC03062	0.0000000	0.0015551	0.0000000	0.0000000	0.0000000
ENSG00000237626	0.0016506	0.0021482	0.0042058	0.0000000	0.0221746
LINC01508	0.0092671	0.0014800	0.0022460	0.0169328	0.0025260
LINC01501	0.0035495	0.0010205	0.0000000	0.0000000	0.0037728
DIRAS2	0.1145861	0.1875803	0.2998345	0.1274363	0.1742673
SYK	0.0000000	0.0008398	0.0000000	0.0000000	0.0000000
ENSG00000237422	0.0000000	0.0000000	0.0000000	0.0000000	0.0000000
LINC02937	0.0013902	0.0079229	0.0021457	0.0030534	0.0176837
LINC00484	0.0024388	0.0000000	0.0046612	0.0000000	0.0000000
ENSG00000289123	0.0007845	0.0000000	0.0000000	0.0000000	0.0000000
ENSG00000273381	0.0000000	0.0000000	0.0000000	0.0020019	0.0059207
AUH	0.1949417	0.2085795	0.2263653	0.1560225	0.4290086
NFIL3	0.2452162	0.3599917	0.4025903	0.1756438	0.3709601
ENSG00000233081	0.0021510	0.0043847	0.0000000	0.0000000	0.0000000
ROR2	0.0216974	0.0110762	0.0072078	0.0014220	0.0122055
SPTLC1	0.3402756	0.2412791	0.2562731	0.3902760	0.3185217
ENSG00000290644	0.0034981	0.0047834	0.0000000	0.0042724	0.0000000
ENSG00000236115	0.0000000	0.0000000	0.0000000	0.0000000	0.0000000

IARS1	0.5660999	0.5752669	0.6387925	0.5100824	0.6337507
NOL8	0.2198967	0.2159172	0.2361972	0.2675829	0.1732128
CENPP	0.0556427	0.0822253	0.0755429	0.0637430	0.2338234
OGN	0.0227337	0.0323432	0.0190690	0.0202839	0.0416911
OMD	0.0000000	0.0016838	0.0000000	0.0000000	0.0128202
ASPN	0.0032183	0.0071474	0.0000000	0.0000000	0.0000000
ECM2	0.0123336	0.0143950	0.0072584	0.0494777	0.0309143
ENSG00000285650	0.0000000	0.0012391	0.0000000	0.0000000	0.0081604
IPPK	0.0412209	0.0459368	0.0393001	0.0375119	0.0747907
BICD2	0.1979056	0.2548219	0.2573938	0.1365606	0.1986254
ENSG00000288062	0.0000000	0.0036588	0.0000000	0.0000000	0.0000000
ENSG00000291168	0.0032712	0.0092432	0.0077129	0.0091984	0.0261619
ZNF484	0.1256307	0.1286723	0.1789322	0.0924615	0.2077442
FGD3	0.0536070	0.0448974	0.0542317	0.0792063	0.0819694
SUSD3	0.0148774	0.0081164	0.0000000	0.0142133	0.0041692
CARD19	0.3122190	0.3699629	0.3528784	0.3500536	0.2686981
NINJ1	0.3511821	0.2748433	0.2123393	0.4489574	0.1647053
WNK2	0.3199968	0.3239413	0.2847232	0.2743570	0.6395377
ENSG00000290685	0.0443270	0.1108313	0.1852463	0.0691881	0.0679237
FAM120AOS	0.5427736	0.4555340	0.3737220	0.5148046	0.3914299
FAM120A	0.2892313	0.2412804	0.1791424	0.2874174	0.2931361
ENSG00000289031	0.0000000	0.0000000	0.0000000	0.0000000	0.0129192
PHF2	0.3166301	0.2858700	0.3696000	0.2956872	0.4347842
PTPDC1	0.1014377	0.1112712	0.1214275	0.0991768	0.1995582
ENSG00000286834	0.0031339	0.0015878	0.0058396	0.0000000	0.0019139
MIRLET7A1HG	0.0222462	0.0211196	0.0119395	0.0211638	0.0772491
LINC02603	0.0012450	0.0000000	0.0000000	0.0043798	0.0087507
ZNF169	0.0238439	0.0459399	0.0675154	0.0108419	0.0777682
ENSG00000232063	0.0008021	0.0074775	0.0157860	0.0047839	0.0039263
MFSD14B	0.1381897	0.0985609	0.0813233	0.1616525	0.1667714
ENSG00000286850	0.0015701	0.0142270	0.0012543	0.0020543	0.0510560
PCAT7	0.0091135	0.0223355	0.0183751	0.0048966	0.0143123
FBP2	0.0011669	0.0006778	0.0000000	0.0000000	0.0060217
FBP1	0.0054867	0.0054493	0.0194140	0.0083665	0.0047101
ENSG00000288023	0.0000000	0.0008074	0.0000000	0.0000000	0.0000000
AOPEP	0.2824191	0.2392257	0.1543797	0.2845122	0.5947341
ENSG00000236095	0.0000000	0.0000000	0.0000000	0.0000000	0.0000000
ENSG00000224764	0.0000000	0.0011753	0.0036278	0.0000000	0.0069201
ENSG00000285807	0.0007415	0.0000000	0.0000000	0.0000000	0.0000000
FANCC	0.1126853	0.1036472	0.0935193	0.1049282	0.3602314
PTCH1	0.1166545	0.1530059	0.1810552	0.0784866	0.2655130
ENSG00000271155	0.0016713	0.0108035	0.0070084	0.0079957	0.0000000
ENSG00000228142	0.0014329	0.0042843	0.0000000	0.0000000	0.0000000
ERCC6L2-AS1	0.0820792	0.1276005	0.1295505	0.0599314	0.2262491
ENSG00000268926	0.0040643	0.0014312	0.0000000	0.0037324	0.0064609
ERCC6L2	0.3675129	0.4524383	0.4761611	0.3398140	0.6515671
LINC00092	0.0075211	0.0083421	0.0054664	0.0239864	0.0045524
ENSG00000237212	0.0000000	0.0000000	0.0009760	0.0000000	0.0000000
ENSG00000275465	0.0000000	0.0000000	0.0000000	0.0046901	0.0030611
HSD17B3	0.0007135	0.0000000	0.0032064	0.0031040	0.0000000
SLC35D2	0.0804565	0.0555516	0.0149868	0.1034776	0.0623770
ZNF367	0.0039128	0.0112761	0.0132240	0.0013877	0.0089959
ENSG00000286835	0.0014879	0.0028242	0.0029419	0.0039615	0.0040606

HABP4	0.3282569	0.4060666	0.4638732	0.3147027	0.4202735
CDC14B	0.1309518	0.1519646	0.1135469	0.1352006	0.3697181
PRXL2C	0.1892751	0.1359163	0.0949555	0.1745936	0.1341860
ENSG00000224848	0.0190678	0.0348903	0.0533653	0.0166232	0.0570612
ZNF510	0.1434278	0.1976360	0.1836667	0.1047048	0.1525403
ZNF782	0.0580921	0.0790017	0.0692088	0.0426866	0.2567629
MFSD14CP	0.1807390	0.2598107	0.2878482	0.1561058	0.3223785
NUTM2G	0.0003510	0.0012802	0.0000000	0.0000000	0.0000000
CTSV	0.0051738	0.0117206	0.0206098	0.0108545	0.0235765
ENSG00000242375	0.0021298	0.0000000	0.0000000	0.0000000	0.0115910
ENSG00000203279	0.0487015	0.0760929	0.1049875	0.0537528	0.0778114
ENSG00000235494	0.0022486	0.0000000	0.0007564	0.0038795	0.0122012
CCDC180	0.0816980	0.0639282	0.0571392	0.0534024	0.1718105
ENSG00000286375	0.0036308	0.0059898	0.0000000	0.0045838	0.0000000
TDRD7	0.1660920	0.1424491	0.1079181	0.1637785	0.1568882
TMOD1	0.3231816	0.2874888	0.0991561	0.5428550	0.2342552
ENSG00000228174	0.0000000	0.0000000	0.0000000	0.0000000	0.0000000
TSTD2	0.2301845	0.2597652	0.2277093	0.2365064	0.2481986
NCBP1	0.2833300	0.2300752	0.2345920	0.2997383	0.2537658
ENSG00000231521	0.0000000	0.0000000	0.0000000	0.0000000	0.0000000
XPA	0.4930498	0.4712814	0.5368402	0.4751660	0.3912280
PTCSC2	0.0028773	0.0018065	0.0000000	0.0027537	0.0000000
TRMO	0.0619703	0.0532734	0.0355662	0.0759395	0.0553305
ENSG00000287070	0.0072163	0.0017742	0.0038044	0.0000000	0.0194560
HEMGN	0.0000000	0.0014613	0.0000000	0.0000000	0.0000000
ANP32B	1.6960393	1.4498705	1.2593780	1.4719530	1.3037876
NANS	0.1808790	0.3038182	0.3376518	0.1746761	0.1818656
TRIM14	0.0146696	0.0243393	0.0060382	0.0221679	0.0299261
CORO2A	0.0291051	0.1023985	0.0670298	0.0376812	0.0571068
TBC1D2	0.0055731	0.0120986	0.0037440	0.0077753	0.0405167
GABBR2	0.2853863	0.4935528	0.6354139	0.3647219	0.7469176
ANKS6	0.0416668	0.0305452	0.0330123	0.0793069	0.0626385
ENSG00000285706	0.0017879	0.0201454	0.0156581	0.0000000	0.0129602
GALNT12	0.0101809	0.0116513	0.0077268	0.0302702	0.0520816
ENSG00000267026	0.0000000	0.0000000	0.0000000	0.0000000	0.0000000
COL15A1	0.0044014	0.0098936	0.0034486	0.0104132	0.0289761
ENSG00000270412	0.0000000	0.0000000	0.0000000	0.0000000	0.0000000
TGFBR1	0.3385616	0.3209013	0.2908887	0.3277311	0.3248990
ALG2	0.1505115	0.1075000	0.1001902	0.2529004	0.1455600
SEC61B	1.4193831	1.3366495	1.3691648	1.4018913	1.1193886
NAMA	0.0015663	0.0014460	0.0015669	0.0000000	0.0029223
ENSG00000237461	0.0073329	0.0057108	0.0059536	0.0036112	0.0059238
STX17-DT	0.0810073	0.0725769	0.0713069	0.0913440	0.0807470
ENSG00000234860	0.0000000	0.0000000	0.0031670	0.0000000	0.0054881
NR4A3	0.0500784	0.0320227	0.0441497	0.0712771	0.0737109
STX17	0.4680562	0.3431024	0.3356112	0.3906797	0.2905135
ENSG00000254571	0.0011049	0.0026720	0.0011671	0.0000000	0.0120325
ERP44	0.5879337	0.4152131	0.4031063	0.7379911	0.4761189
INVS	0.1702154	0.1505550	0.1224981	0.1901709	0.3088525
TEX10	0.1559138	0.1209140	0.1586843	0.1301208	0.1727817
MSANTD3	0.4073133	0.6084643	0.7784402	0.3955527	0.4596601
TMEFF1	0.7443458	1.0817927	1.3197721	0.6920314	1.0210352
CAVIN4	0.0275746	0.0260009	0.0286301	0.0161021	0.1110775

PLPPR1	0.8873318	0.7224230	0.8408219	1.1724165	0.9547472
BAAT	0.0057580	0.0078126	0.0100921	0.0014244	0.0194257
MRPL50	0.3906250	0.3497084	0.2846304	0.4441012	0.2228812
ZNF189	0.1664566	0.1822572	0.1437905	0.1500680	0.1555888
ALDOB	0.0043314	0.0033350	0.0000000	0.0057455	0.0044670
TMEM246-AS1	0.0039183	0.0015950	0.0049052	0.0000000	0.0000000
PGAP4	0.2809486	0.4045672	0.5591975	0.2875584	0.3125034
RNF20	0.5513031	0.4539653	0.4448091	0.5343827	0.3441920
GRIN3A	0.0303204	0.0636640	0.0620356	0.0303192	0.0927471
PPP3R2	0.0000000	0.0000000	0.0000000	0.0000000	0.0000000
LINC01492	0.0007235	0.0036714	0.0045437	0.0000000	0.0000000
SMC2-DT	0.0114835	0.0046079	0.0015202	0.0019593	0.0034716
SMC2	0.2467748	0.2993489	0.2528850	0.2254624	0.2395865
ENSG00000290765	0.0000000	0.0000000	0.0000000	0.0000000	0.0048250
NIPSNAP3A	0.4479893	0.4474848	0.3455749	0.5492927	0.2908239
NIPSNAP3B	0.0259724	0.0331969	0.0152438	0.0187730	0.0345426
ABCA1	0.0519522	0.0409678	0.0406388	0.0928828	0.1099738
ENSG00000226334	0.0027571	0.0033318	0.0000000	0.0016011	0.0000000
CT70	0.0022726	0.0012984	0.0012816	0.0000000	0.0071088
ENSG00000286536	0.0038314	0.0072817	0.0043030	0.0101446	0.0274193
SLC44A1	0.4356558	0.3025340	0.2363743	0.5807811	0.3828324
ENSG00000289227	0.0006797	0.0000000	0.0062721	0.0035717	0.0000000
FSD1L	0.5293988	0.4944066	0.4659926	0.4411765	0.6379560
FKTN-AS1	0.0031449	0.0000000	0.0012177	0.0000000	0.0147763
FKTN	0.1772553	0.1585194	0.1370753	0.2022340	0.3117767
TAL2	0.0203058	0.0111012	0.0122811	0.0067419	0.0076477
TMEM38B	0.2889269	0.2102776	0.1574258	0.3460682	0.1935068
LINC01505	0.0238947	0.0267515	0.0245580	0.0121078	0.0924123
ZNF462	0.4833882	0.5661820	0.6744585	0.5291367	0.7544451
ENSG00000230782	0.0014201	0.0000000	0.0028904	0.0000000	0.0000000
RAD23B	1.1033018	1.0075493	0.9219312	1.0629135	0.9161861
LINC01509	0.0023936	0.0013745	0.0000000	0.0034964	0.0000000
KLF4	0.0969802	0.0482699	0.0434226	0.0670354	0.0546313
ENSG00000289987	0.0046480	0.0098451	0.0025093	0.0063709	0.0029535
ELP1	0.1565829	0.1932534	0.2066135	0.1201419	0.3772201
ABITRAM	0.2772900	0.2261639	0.2237538	0.2984896	0.1499754
CTNNAL1	0.1419244	0.0842741	0.0798594	0.1712038	0.2152625
TMEM245	0.2399432	0.3031541	0.2102297	0.2279853	0.3472065
FRRS1L	0.3848237	0.6738746	0.7703800	0.3383931	0.6520645
EPB41L4B	0.0764121	0.1562306	0.1342557	0.0370758	0.3256744
PTPN3	0.1393960	0.2530134	0.1269173	0.0882353	0.1824334
PALM2AKAP2	0.4419426	0.6752645	0.8419876	0.3833982	0.8788234
ENSG00000232939	0.0000000	0.0000000	0.0000000	0.0000000	0.0000000
C9orf152	0.0012749	0.0000000	0.0000000	0.0000000	0.0058321
TXN	1.7205819	1.8393696	2.0174038	1.7205403	1.4469948
SVEP1	0.0047073	0.0094818	0.0119010	0.0042969	0.0204300
MUSK	0.0000000	0.0000000	0.0000000	0.0000000	0.0000000
LPAR1	0.1535222	0.0895081	0.0842897	0.2108516	0.1925411
ENSG00000227531	0.0027566	0.0016549	0.0000000	0.0000000	0.0068101
ECPAS	0.5152869	0.5979043	0.5758776	0.4627894	0.7264237
ZNF483	0.3119349	0.3453149	0.3262035	0.2567002	0.4610603
PTGR1	0.2384292	0.2402732	0.1519519	0.2500826	0.2064910
LRRC37A5P	0.2196348	0.1928560	0.1318656	0.2860540	0.1558091

DNAJC25	0.2229118	0.2021124	0.2224671	0.2123243	0.2782636
GNG10	0.5362789	0.5325399	0.5006396	0.6558006	0.4488842
SHOC1	0.0053197	0.0069138	0.0029081	0.0027694	0.0449646
UGCG	0.4334857	0.6035837	0.9002766	0.3669969	0.6161510
LINC02977	0.0259336	0.0248258	0.0285092	0.0326019	0.0235928
SUSD1	0.0788788	0.0808255	0.0555915	0.1116557	0.1263538
PTBP3	0.5404195	0.6496290	0.7035135	0.4733161	0.6853642
HSDL2	0.5667470	0.3737439	0.3038252	0.5548056	0.3097188
HSDL2-AS1	0.0709576	0.0436181	0.0383604	0.0816991	0.0715910
KIAA1958	0.5953260	0.5321925	0.4753735	0.6216679	0.8357457
INIP	0.3289929	0.3634735	0.3723064	0.3335954	0.3581591
SNX30-DT	0.0008655	0.0019103	0.0012475	0.0000000	0.0000000
SNX30	0.1687467	0.2703065	0.2892109	0.1427617	0.3663516
SLC46A2	0.0000000	0.0000000	0.0000000	0.0000000	0.0063087
ENSG00000291094	0.0377822	0.0572252	0.0463194	0.0181345	0.1272426
ZNF883	0.0795983	0.1785768	0.1447040	0.0877313	0.1992689
ZFP37	0.1250731	0.1766598	0.1979917	0.0803454	0.2345877
SLC31A2	0.0100658	0.0102282	0.0106815	0.0224178	0.0081554
FKBP15	0.1476500	0.1434458	0.1565094	0.1296334	0.1961628
SLC31A1	0.1336885	0.1087355	0.0982127	0.1832990	0.1453826
CDC26	0.3490038	0.2865544	0.2756738	0.3139134	0.2201264
PRPF4	0.2049178	0.2168594	0.2261682	0.1690805	0.1925357
RNF183	0.0000000	0.0000000	0.0006116	0.0000000	0.0000000
WDR31	0.1398711	0.0946203	0.0636247	0.0878767	0.1125505
BSPRY	0.0022569	0.0010918	0.0000000	0.0000000	0.0000000
HDHD3	0.0646682	0.0717890	0.0763372	0.0712375	0.0217927
ALAD	0.1324704	0.1400725	0.1252600	0.1303077	0.1243338
POLE3	0.3634708	0.2726329	0.2363771	0.3324397	0.2341455
C9orf43	0.0097582	0.0038368	0.0111904	0.0122718	0.0027158
RGS3	0.3010101	0.3830827	0.2301027	0.2862722	0.2901212
ENSG00000227482	0.0089918	0.0022503	0.0051787	0.0052973	0.0000000
ZNF618	0.1802829	0.2712771	0.2363903	0.1754941	0.5135788
AMBP	0.0080309	0.0020473	0.0016515	0.0184776	0.0000000
KIF12	0.0040917	0.0038544	0.0018938	0.0017529	0.0000000
COL27A1	0.0796276	0.0492725	0.0490712	0.0584877	0.1598935
AKNA	0.0861893	0.0680265	0.0833523	0.1112382	0.1237240
ENSG00000235119	0.0065990	0.0030201	0.0000000	0.0040247	0.0000000
WHRN	0.1112194	0.1457849	0.0885011	0.1056800	0.2253601
ATP6V1G1	1.5100949	1.4583986	1.4355190	1.4815356	1.2336288
TMEM268	0.1523378	0.2296021	0.2723598	0.0989438	0.2032642
TEX53	0.0000000	0.0008594	0.0049942	0.0000000	0.0000000
TEX48	0.0041508	0.0005918	0.0043388	0.0000000	0.0108135
TNFSF15	0.0088026	0.0168987	0.0024049	0.0000000	0.0032941
DELEC1	0.0140548	0.0054265	0.0042971	0.0176949	0.0723693
TNC	0.3852299	0.2404573	0.1715973	0.8343124	0.3337690
ENSG00000234692	0.0114518	0.0076081	0.0028446	0.0083373	0.0137325
ENSG00000228714	0.0011651	0.0000000	0.0000000	0.0000000	0.0104418
PAPPA	0.1300975	0.2214913	0.0740004	0.1217499	0.3272569
ENSG00000244757	0.0000000	0.0000000	0.0000000	0.0099186	0.0110375
PAPPA-AS1	0.0011451	0.0041967	0.0012482	0.0000000	0.0000000
ASTN2	0.1684008	0.2059534	0.1515577	0.2360215	0.7228247
ASTN2-AS1	0.0000000	0.0000000	0.0000000	0.0000000	0.0055202
ENSG00000230894	0.0000000	0.0000000	0.0000000	0.0000000	0.0000000

TRIM32	0.0806760	0.1229045	0.0984399	0.0887678	0.0648718
ENSG00000285820	0.0034823	0.0012294	0.0000000	0.0000000	0.0155380
TLR4	0.0000000	0.0000000	0.0021490	0.0000000	0.0049154
ENSG00000284977	0.0002597	0.0000000	0.0000000	0.0028151	0.0041999
BRINP1	0.1873415	0.3081193	0.3677359	0.1769292	0.6244027
ENSG00000260970	0.0000000	0.0024248	0.0000000	0.0000000	0.0000000
CDK5RAP2	0.6256238	0.4459025	0.3062097	0.6879705	0.5423699
MEGF9	0.6198113	0.5349949	0.5110933	0.6895179	0.6051972
FBXW2	0.2662810	0.3677323	0.3639406	0.2729112	0.2933593
B3GALT9	0.0338184	0.0288695	0.0231808	0.0487477	0.0301040
PSMD5	0.2873961	0.2328122	0.2154945	0.2466117	0.2772233
PHF19	0.1321014	0.0981199	0.0862628	0.1400376	0.0930664
TRAF1	0.0109645	0.0125728	0.0191530	0.0122953	0.0343034
C5	0.0943549	0.0637416	0.0414107	0.1057379	0.0647093
CNTRL	0.3787499	0.2796251	0.2490777	0.3309711	0.5092164
RAB14	1.1490190	1.2398207	1.3707401	1.2675062	0.9719802
GSN	0.3279092	0.2029630	0.0865551	0.3396980	0.1689204
STOM	0.0441040	0.0353297	0.0078646	0.0924845	0.0266102
ENSG00000227355	0.0029768	0.0023602	0.0025278	0.0000000	0.0000000
GGTA1	0.0074821	0.0162903	0.0119121	0.0062957	0.0082755
DAB2IP	0.2938173	0.2782271	0.2756901	0.2594445	0.2579607
TTLL11	0.1160801	0.1410963	0.1463976	0.0848278	0.3591544
NDUFA8	0.7272955	0.7524913	0.7906881	0.7491806	0.6085774
MORN5	0.3021961	0.1828822	0.1089515	0.3296452	0.0780934
LHX6	0.0016563	0.0039775	0.0000000	0.0056441	0.0035288
RBM18	0.3385992	0.3637536	0.4054884	0.2576035	0.2917812
MRRF	0.0910663	0.0756574	0.0971455	0.0476145	0.0935389
PTGS1	0.0019376	0.0014173	0.0000000	0.0021653	0.0100897
ENSG00000234156	0.0012364	0.0019798	0.0000000	0.0000000	0.0024068
OR1L8	0.0007243	0.0012400	0.0000000	0.0056237	0.0000000
OR1B1	0.0000000	0.0000000	0.0055658	0.0000000	0.0065952
PDCL	0.2675158	0.2783913	0.2438812	0.3182622	0.1411027
RC3H2	0.3470272	0.4125402	0.4647035	0.3418020	0.4651274
ZBTB6	0.1456572	0.1866834	0.2146811	0.1056470	0.1372967
ZBTB26	0.0697348	0.0835930	0.0728306	0.0550741	0.0800981
ENSG00000261094	0.0019023	0.0025858	0.0010425	0.0045196	0.0052830
ENSG00000286718	0.0087228	0.0040477	0.0000000	0.0106139	0.0000000
RABGAP1	0.5401637	0.5463980	0.5382488	0.5476064	0.5928105
GPR21	0.0000000	0.0070161	0.0031847	0.0000000	0.0000000
MIR600HG	0.0060709	0.0179545	0.0127816	0.0095924	0.0164808
STRBP	0.6421709	0.7793011	0.8287423	0.4945992	0.7993123
CRB2	0.0932919	0.0386580	0.0276203	0.2340337	0.0793413
DENND1A	0.2123348	0.2233291	0.2158167	0.2258282	0.6156210
ENSG00000230826	0.0025097	0.0000000	0.0000000	0.0000000	0.0215554
NEK6	0.2826984	0.1932914	0.1122530	0.4359393	0.2197231
PSMB7	0.8096082	0.8257284	0.8728747	0.7612010	0.6200249
ADGRD2	0.0210389	0.0205531	0.0068206	0.0427657	0.0223949
NR5A1	0.0012332	0.0000000	0.0000000	0.0000000	0.0025831
ENSG00000231149	0.0020424	0.0000000	0.0000000	0.0013887	0.0013244
NR6A1	0.1194730	0.1342279	0.1110979	0.1008499	0.5173347
MIR181A2HG	0.0546374	0.0923532	0.1161456	0.0500959	0.2188189
OLFML2A	0.1069895	0.0776686	0.0357524	0.1959250	0.0770205
WDR38	0.1228866	0.0680685	0.0449122	0.1300058	0.0750872

RPL35	2.5256812	2.4449806	2.3288514	2.4994851	2.2041938
ARPC5L	0.5103110	0.5690503	0.5925721	0.4990433	0.3643603
ENSG00000289285	0.0091470	0.0018534	0.0030685	0.0023899	0.0000000
GOLGA1	0.2656957	0.2278927	0.2219594	0.2992358	0.2604199
SCAI	0.2072133	0.3229796	0.3868011	0.1447772	0.4446188
PPP6C	0.3671721	0.3270588	0.3083412	0.3125786	0.3332105
RABEPK	0.2239667	0.2531169	0.3175359	0.2468193	0.2012024
HSPA5	1.9366009	1.4769345	1.5364822	2.6950995	1.5820547
HSPA5-DT	0.0027236	0.0000000	0.0000000	0.0000000	0.0000000
GAPVD1	0.2993294	0.3140978	0.2867818	0.3572040	0.3825199
ENSG00000287782	0.0037591	0.0014991	0.0000000	0.0026640	0.0000000
MAPKAP1	0.5738972	0.5497047	0.5145677	0.6178529	0.6147488
PBX3	0.8992627	1.3349744	1.3362182	0.8147928	1.5267650
ENSG00000232413	0.0011092	0.0000000	0.0000000	0.0000000	0.0018081
MVB12B	0.2463474	0.2379643	0.3053210	0.2476911	0.4572292
ENSG00000233721	0.0000000	0.0010939	0.0000000	0.0000000	0.0000000
ENSG00000226078	0.0000000	0.0000000	0.0000000	0.0000000	0.0040644
LMX1B-DT	0.0662654	0.0413472	0.0550355	0.0691185	0.0338449
LMX1B	0.1399564	0.1198931	0.2349512	0.1070856	0.1997765
ZBTB43	0.1427074	0.1534585	0.1484264	0.1188694	0.1729032
ENSG00000288995	0.0028306	0.0013966	0.0000000	0.0000000	0.0078844
ZBTB34	0.0806796	0.1078409	0.0960286	0.0714501	0.1244848
RALGPS1	0.2075897	0.3140791	0.3119544	0.2041588	0.6641005
ANGPTL2	0.2288486	0.1106954	0.0626674	0.2541932	0.0807053
ENSG00000228487	0.0005895	0.0000000	0.0022845	0.0000000	0.0000000
GARNL3	0.1409131	0.1875105	0.1968749	0.1777261	0.5202379
ENSG00000271833	0.0046896	0.0090492	0.0084105	0.0000000	0.0294302
SLC2A8	0.0580521	0.0477945	0.0424401	0.0749327	0.0585794
ZNF79	0.0169301	0.0113942	0.0223452	0.0107270	0.0378645
RPL12	2.8961926	2.8622123	2.6638401	2.8158026	2.6048124
LRSAM1	0.1757691	0.2078626	0.1613790	0.1619269	0.3735865
NIBAN2	0.0644518	0.0562044	0.0656984	0.0659528	0.0627395
STXBP1	0.5501309	0.7661728	1.1480190	0.4521344	0.9513290
ENSG00000279571	0.0132008	0.0058533	0.0106901	0.0074966	0.0040567
PTRH1	0.2429901	0.1834194	0.1489331	0.3298420	0.1491718
CFAP157	0.0429379	0.0270188	0.0272428	0.0289869	0.0517872
TTC16	0.0020830	0.0045587	0.0016198	0.0029510	0.0031362
TOR2A	0.0666445	0.0725866	0.0842724	0.1060942	0.0623974
SH2D3C	0.0464365	0.1106522	0.0879895	0.0451590	0.1442659
ENSG00000286196	0.0002459	0.0006444	0.0044925	0.0000000	0.0000000
CDK9	0.2832762	0.2275673	0.2586040	0.2943269	0.2701038
FPGS	0.1329999	0.1130578	0.0898622	0.1246521	0.0988143
ENG	0.0065161	0.0035626	0.0026208	0.0128112	0.0145004
ENSG00000225032	0.0020134	0.0066199	0.0000000	0.0041169	0.0142563
AK1	1.7021898	1.4056658	1.2188623	1.6942336	1.2184676
ST6GALNAC6	0.2436286	0.2207680	0.2158725	0.2092856	0.2056014
ST6GALNAC4	0.1512032	0.1164096	0.0802691	0.1169857	0.0988452
PIP5KL1	0.0480023	0.0695138	0.0675737	0.0290182	0.0748032
ENSG00000227218	0.0000000	0.0003520	0.0000000	0.0000000	0.0025607
DPM2	0.3402604	0.3595926	0.3674341	0.3904777	0.2408257
EEIG1	0.1108853	0.0765376	0.0667607	0.0937980	0.2098589
NAIF1	0.0543866	0.0330348	0.0372598	0.0804953	0.0299977
SLC25A25	0.1124389	0.1850418	0.1440850	0.1371175	0.1728298

ENSG00000230536	0.0016207	0.0000000	0.0013038	0.0032535	0.0000000
SLC25A25-AS1	0.0124414	0.0226017	0.0103574	0.0098386	0.0799897
PTGES2	0.2600244	0.2820147	0.3388886	0.2317681	0.2166700
PTGES2-AS1	0.0010783	0.0000000	0.0000000	0.0000000	0.0000000
BBLN	0.9614826	1.0815080	1.1443622	0.9232381	0.8630172
CIZ1	0.3955658	0.4521766	0.4110892	0.4015627	0.3857065
DNM1	0.1731403	0.3068895	0.4266853	0.1486233	0.5623283
GOLGA2	0.6813243	0.6132155	0.5916870	0.6633207	0.5911439
SWI5	0.4387480	0.3307435	0.3171121	0.4685468	0.3199910
TRUB2	0.1790854	0.2048796	0.1949323	0.2308671	0.1508986
ENSG00000272696	0.0097804	0.0113088	0.0012851	0.0064647	0.0000000
COQ4	0.5157028	0.5188102	0.5321210	0.4868600	0.3727394
SLC27A4	0.1525341	0.1556374	0.1199774	0.1467636	0.1560971
URM1	0.4268749	0.3826164	0.3220956	0.4461488	0.2703061
MIR219A2HG	0.0172114	0.0085318	0.0041326	0.0238139	0.0015171
CERCAM	0.1561735	0.0871097	0.0816860	0.1931179	0.1386267
ENSG00000273186	0.0016538	0.0037032	0.0016319	0.0000000	0.0000000
ODF2	0.1910893	0.1673262	0.1707377	0.2038106	0.2024488
ODF2-AS1	0.0000000	0.0000000	0.0000000	0.0036873	0.0042206
GLE1	0.0990812	0.0934169	0.0680089	0.1183880	0.0812292
ENSG00000228395	0.0226473	0.0100839	0.0074978	0.0120903	0.0114608
SPTAN1	1.2829632	1.3469871	1.3034192	1.2030227	1.5516493
ENSG00000280474	0.0022691	0.0000000	0.0000000	0.0000000	0.0028666
DYNC2I2	0.2544365	0.1715192	0.2057656	0.3184935	0.1406731
HMGA1P4	0.0034091	0.0027249	0.0008688	0.0042775	0.0000000
SET	1.9214191	2.0678682	2.2482530	1.8448663	1.7962570
PKN3	0.0016676	0.0000000	0.0020559	0.0028047	0.0074245
ZDHHC12	0.0956799	0.0849224	0.0718637	0.1042497	0.0571943
ZDHHC12-DT	0.0316805	0.0197638	0.0146399	0.0161788	0.0071633
ZER1	0.1723577	0.1828377	0.1830471	0.1608997	0.2228676
TBC1D13	0.1123017	0.0878752	0.1041753	0.1172363	0.1023454
ENDOG	0.3252497	0.4578415	0.5254569	0.3743046	0.2661097
SPOUT1	0.0573664	0.0422554	0.0631120	0.0584091	0.0717700
KYAT1	0.0488661	0.0404544	0.0587779	0.0423256	0.1184775
LRRC8A	0.1247879	0.1604549	0.1674663	0.0858277	0.1421535
PHYHD1	0.0048511	0.0037161	0.0014836	0.0068194	0.0000000
DOLK	0.0546667	0.0543477	0.0371405	0.0937860	0.0394388
NUP188	0.0609013	0.0524829	0.0530295	0.0590138	0.1332613
ENSG00000267834	0.0000000	0.0000000	0.0000000	0.0000000	0.0000000
SH3GLB2	0.3008378	0.3493712	0.4569138	0.2786380	0.2680232
MIGA2	0.0448252	0.0453850	0.0274003	0.0345231	0.0769608
ENSG00000287234	0.0014605	0.0053121	0.0000000	0.0077308	0.0000000
DOLPP1	0.0634227	0.0665234	0.1086527	0.0967525	0.0508375
CRAT	0.1705841	0.1826160	0.1958626	0.1967271	0.1470172
PTPA	0.1720130	0.1928885	0.2414476	0.1962891	0.2408717
ENSG00000268707	0.0045428	0.0079821	0.0030077	0.0000000	0.0000000
IER5L	0.1367742	0.1704165	0.1710344	0.1077304	0.1717133
IER5L-AS1	0.0188303	0.0132598	0.0086355	0.0093318	0.0371996
LINC02975	0.0011187	0.0046804	0.0027945	0.0019116	0.0119639
LINC01503	0.0159183	0.0266403	0.0388442	0.0102169	0.0174197
ENSG00000230676	0.0031284	0.0032475	0.0000000	0.0000000	0.0092272
ENSG00000226355	0.0042403	0.0000000	0.0000000	0.0000000	0.0024307
LINC00963	0.0560050	0.0578698	0.0257226	0.0860125	0.0485745

NTMT1	0.2283541	0.2112273	0.2583515	0.2262565	0.1991346
C9orf50	0.0040634	0.0046149	0.0159908	0.0191824	0.0000000
ASB6	0.0545318	0.0631875	0.0599728	0.0761974	0.0462990
PRRX2	0.0016623	0.0000000	0.0000000	0.0000000	0.0032853
PTGES	0.0174876	0.0270626	0.0301531	0.0118172	0.0153051
TOR1B	0.1303061	0.1970650	0.1970687	0.2088944	0.2239291
TOR1A	0.1543076	0.1538409	0.1648796	0.2141060	0.1409550
C9orf78	0.6307856	0.6241240	0.6223111	0.5762114	0.4997400
USP20	0.0362522	0.0361443	0.0502982	0.0315532	0.0982753
FNBP1	0.5361038	0.5987390	0.5954079	0.5779103	0.7962304
ENSG00000288924	0.0180122	0.0316953	0.0555166	0.0289741	0.0107707
ENSG00000230684	0.0000000	0.0000000	0.0000000	0.0000000	0.0013244
ENSG00000270755	0.0064824	0.0057152	0.0012102	0.0048317	0.0058242
ENSG00000289226	0.0455079	0.0467704	0.0243528	0.0306683	0.0152449
GPR107	0.2100684	0.2033615	0.1898476	0.2963746	0.3586616
NCS1	0.8818726	1.3645577	1.4607823	0.7693665	1.0226072
HMCN2	0.0071635	0.0111322	0.0070964	0.0060484	0.0526123
ASS1	0.1179354	0.1222149	0.2024818	0.0985856	0.1109871
FUBP3	0.4138664	0.3037682	0.2605573	0.4398406	0.3297156
PRDM12	0.0021786	0.0082827	0.0165457	0.0023550	0.0060804
EXOSC2	0.1238111	0.1255144	0.1270641	0.1105817	0.0845469
ABL1	0.2192653	0.2129905	0.2016067	0.2004791	0.4262375
FIBCD1	0.0064694	0.0072398	0.0118860	0.0076634	0.0106076
LAMC3	0.0011518	0.0000000	0.0009751	0.0026278	0.0000000
AIF1L	0.0078768	0.0053188	0.0194615	0.0119686	0.0058257
NUP214	0.2318481	0.2210535	0.2249235	0.2122881	0.2378121
ENSG00000236986	0.0008299	0.0012268	0.0000000	0.0000000	0.0058189
ENSG00000246851	0.0000000	0.0018437	0.0026108	0.0064764	0.0057461
FAM78A	0.0075695	0.0071369	0.0052566	0.0091624	0.0116452
PLPP7	0.0144157	0.0176956	0.0193567	0.0141445	0.0239145
ENSG00000289000	0.0036851	0.0051399	0.0035791	0.0000000	0.0204123
PRRC2B	0.8819129	1.1106131	1.2162674	0.8744907	1.1411145
ENSG00000176868	0.0311383	0.0328362	0.0502709	0.0305404	0.0466986
POMT1	0.1063400	0.0874038	0.0817837	0.1203068	0.1707612
ENSG00000230289	0.0011075	0.0000000	0.0000000	0.0000000	0.0000000
UCK1	0.1586720	0.0867083	0.0828332	0.1321843	0.1311705
PRRT1B	0.0143830	0.0078967	0.0014006	0.0000000	0.0107175
RAPGEF1	0.3063716	0.3198522	0.3458366	0.2855417	0.3824629
MED27	0.2265526	0.2300189	0.2323845	0.2187675	0.3483618
NTNG2	0.0246490	0.0635312	0.0200872	0.0202383	0.0787955
SETX	0.3792655	0.3227649	0.3369504	0.3573263	0.4514737
TTF1	0.2652101	0.2305820	0.2089403	0.1911897	0.1869614
CFAP77	0.1837942	0.1157969	0.0645310	0.2089120	0.1116940
BARHL1	0.0003510	0.0000000	0.0021912	0.0000000	0.0000000
DDX31	0.0517143	0.0628992	0.0441707	0.0576406	0.1136007
GTF3C4	0.2495147	0.2232234	0.1903590	0.2502481	0.2250215
AK8	0.0593287	0.0334637	0.0334930	0.0492100	0.0621645
SPACA9	0.4894787	0.3773297	0.3226028	0.5181921	0.2762667
TSC1	0.2229347	0.2490346	0.2253999	0.1835610	0.3300032
GFI1B	0.0000000	0.0012329	0.0041732	0.0000000	0.0000000
ENSG00000288989	0.0082833	0.0037573	0.0053097	0.0031460	0.0046088
GTF3C5	0.0963571	0.1186931	0.0842956	0.1076746	0.0973878
CEL	0.0014396	0.0020854	0.0000000	0.0000000	0.0000000

ENSG00000290769	0.0000000	0.0000000	0.0025063	0.0000000	0.0000000
RALGDS	0.2432974	0.3106580	0.3072555	0.2377119	0.4037004
GBGT1	0.0080232	0.0065574	0.0000000	0.0023370	0.0060761
ABO	0.0099724	0.0228565	0.0374636	0.0048877	0.0524055
SURF6	0.2936844	0.2748109	0.3273753	0.2684464	0.2147192
MED22	0.0601217	0.0658986	0.0821393	0.0683179	0.0477289
RPL7A	3.0279288	3.0337328	2.8847852	2.9570261	2.7362587
SURF1	0.3949964	0.3830241	0.3333427	0.4051224	0.2380730
SURF2	0.2522242	0.2846925	0.3274665	0.3016738	0.1622004
SURF4	0.4159663	0.2764937	0.2808955	0.4630600	0.2959393
STKLD1	0.0200992	0.0138276	0.0227100	0.0250612	0.0077837
REXO4	0.4169946	0.3693611	0.4220188	0.3453307	0.3490258
ADAMTS13	0.0286965	0.0300038	0.0322415	0.0379366	0.0311336
CACFD1	0.0975382	0.0926003	0.0826442	0.0560761	0.0463731
SLC2A6	0.0387247	0.0711463	0.0725582	0.0507824	0.0652806
ADAMTSL2	0.0023894	0.0009065	0.0009760	0.0057818	0.0053082
FAM163B	0.0082848	0.0312776	0.0270624	0.0050110	0.0475506
DBH	0.0000000	0.0017623	0.0000000	0.0021126	0.0000000
DBH-AS1	0.0014713	0.0022101	0.0000000	0.0026207	0.0078627
SARDH	0.0045245	0.0103996	0.0028264	0.0072125	0.0501850
VAV2	0.1389150	0.1503672	0.1836579	0.1523475	0.3906203
BRD3OS	0.3426796	0.2752841	0.2868660	0.3790328	0.2151962
BRD3	0.6139230	0.5820509	0.6538315	0.6104277	0.5567344
WDR5-DT	0.0211320	0.0213528	0.0155677	0.0312590	0.0353737
WDR5	0.1725895	0.1972664	0.2154328	0.1918404	0.1776001
ENSG00000273473	0.0025281	0.0009965	0.0020317	0.0039003	0.0024001
RXRA	0.1151203	0.1016839	0.1235056	0.1170843	0.2220639
ENSG00000228877	0.0021905	0.0011565	0.0000000	0.0000000	0.0000000
COL5A1	0.0873470	0.0542552	0.0403258	0.1461340	0.0763908
OLFM1	0.4843834	0.8706199	1.1900726	0.4821086	0.6887602
PPP1R26-AS1	0.0060572	0.0173062	0.0041849	0.0107853	0.0179646
PPP1R26	0.1063178	0.1020471	0.1375729	0.0544914	0.0759764
PIERCE1	0.3973144	0.2587091	0.1863220	0.5090211	0.2167945
MRPS2	0.3445831	0.3423092	0.3201500	0.3554838	0.2685490
KCNT1	0.0093830	0.0166349	0.0144693	0.0000000	0.0183970
CAMSAP1	0.3652859	0.5760139	0.6920709	0.3858367	0.6540477
CAMSAP1-DT	0.0529669	0.0798073	0.0775500	0.0448439	0.0686983
UBAC1	0.4435542	0.5605865	0.5916592	0.4512177	0.4032563
NACC2	0.1146492	0.0848539	0.0551848	0.1247442	0.0851323
LINC02846	0.0018878	0.0010980	0.0047311	0.0000000	0.0063982
TMEM250	0.1190395	0.1152440	0.1251601	0.1500678	0.0841431
ENSG00000275329	0.0015909	0.0018012	0.0025433	0.0040556	0.0070395
LHX3	0.0020287	0.0024213	0.0041274	0.0000000	0.0000000
QSOX2	0.0579300	0.0494297	0.0305541	0.0927387	0.0809750
CCDC187	0.0026521	0.0020878	0.0039829	0.0057415	0.0000000
DKFZP434A062	0.0193389	0.0123024	0.0126056	0.0177789	0.0026235
GPSM1	0.1347918	0.1809476	0.1647125	0.0869850	0.2078147
DNLZ	0.2413109	0.2784643	0.3621757	0.2741826	0.1967294
CARD9	0.0000000	0.0011008	0.0045041	0.0000000	0.0223314
SNAPC4	0.0662897	0.0527348	0.0502497	0.0416499	0.1270060
ENTR1	0.0965631	0.0797810	0.0664786	0.0908359	0.0519091
PMPCA	0.1891318	0.2210915	0.2348654	0.2631395	0.1658233
INPP5E	0.0674941	0.0861132	0.0881136	0.0676815	0.0920986

SEC16A	0.2544041	0.1821944	0.1746188	0.2076551	0.3111713
C9orf163	0.0018013	0.0000000	0.0000000	0.0000000	0.0000000
NOTCH1	0.0673524	0.0352688	0.0289660	0.1406964	0.0215684
NALT1	0.0083592	0.0038464	0.0076510	0.0165460	0.0000000
EGFL7	0.0477976	0.0601406	0.0843993	0.0875237	0.0195111
AGPAT2	0.1330138	0.0891129	0.0876916	0.1377212	0.0806088
DIPK1B	0.3819127	0.3822739	0.4496574	0.4421920	0.2996854
SNHG7	1.0594425	0.9278885	0.8694507	1.0444814	0.8070639
TMEM141	0.5183265	0.4102480	0.3630731	0.5150268	0.2509897
CCDC183	0.0690389	0.0586723	0.0541904	0.0582944	0.0734007
CCDC183-AS1	0.0051345	0.0000000	0.0000000	0.0000000	0.0070510
RABL6	0.4149545	0.4846226	0.4288356	0.4123732	0.4935240
AJM1	0.0790114	0.1018659	0.1179280	0.0516866	0.0826654
PHPT1	1.6805939	1.5554788	1.6264129	1.7042179	1.3109981
ENSG00000288873	0.0104979	0.0149482	0.0211237	0.0046700	0.0038839
MAMDC4	0.0234218	0.0171742	0.0210035	0.0222196	0.0584756
EDF1	1.3146304	1.3107746	1.3096793	1.2248155	0.9781657
TRAF2	0.0500705	0.0380350	0.0326399	0.0363023	0.0438626
ENSG00000260190	0.0000000	0.0000000	0.0000000	0.0000000	0.0000000
FBXW5	0.3236247	0.2983073	0.2777115	0.3257948	0.2242222
C8G	0.0018263	0.0018236	0.0008956	0.0028850	0.0000000
LCN12	0.0287495	0.0178264	0.0082952	0.0275037	0.0239750
PTGDS	0.7701860	0.4859835	0.3394005	1.2795317	0.4356316
LCNL1	0.0023043	0.0041948	0.0055294	0.0126477	0.0031582
PAXX	0.4214679	0.4839136	0.5583778	0.4356546	0.3216682
ABCA2	0.1137727	0.0997292	0.0994961	0.1265741	0.1453173
LINC02908	0.0010606	0.0019354	0.0000000	0.0000000	0.0034773
FUT7	0.0041809	0.0017374	0.0000000	0.0000000	0.0015531
NPDC1	0.6459661	0.6578962	0.7748901	0.6930633	0.5911644
ENTPD2	0.0025083	0.0034245	0.0000000	0.0106077	0.0048241
ENSG00000229257	0.0000000	0.0000000	0.0000000	0.0000000	0.0000000
SAPCD2	0.0055154	0.0117990	0.0051502	0.0040899	0.0000000
UAP1L1	0.0246159	0.0204945	0.0141809	0.0334473	0.0170125
MAN1B1-DT	0.0836743	0.0655869	0.0888261	0.0603224	0.0632603
MAN1B1	0.2136060	0.1633894	0.1297062	0.4020341	0.2028647
DPP7	0.5615827	0.3691801	0.3201896	0.7090582	0.4229793
GRIN1	0.0944891	0.1881647	0.2381703	0.0401953	0.2704346
LRRC26	0.0398187	0.0774454	0.1130552	0.0416852	0.0454677
ANAPC2	0.0351294	0.0250121	0.0311752	0.0507446	0.0414595
SSNA1	0.4017992	0.4539170	0.4124524	0.3401085	0.3107642
TPRN	0.0271760	0.0362090	0.0467564	0.0347086	0.0326445
TMEM203	0.2282987	0.2064767	0.2300262	0.2036694	0.2124672
NDOR1	0.0100460	0.0154951	0.0132979	0.0017393	0.0449643
RNF208	0.3061672	0.4127556	0.5116504	0.2703915	0.3469979
CYSRT1	0.0121456	0.0084957	0.0157114	0.0130110	0.0037728
TUBB4B	1.4982716	1.2404882	1.2051696	1.5961677	1.0061598
FAM166A	0.0027075	0.0013272	0.0000000	0.0000000	0.0028456
NELFB	0.2568163	0.2382187	0.1930999	0.2163772	0.2349727
TOR4A	0.0039064	0.0014079	0.0000000	0.0000000	0.0000000
NRARP	0.0322921	0.0225832	0.0220476	0.0138098	0.0155539
EXD3	0.1427533	0.1903568	0.1408046	0.1566424	0.3238541
NOXA1	0.0392064	0.0473229	0.0384608	0.0645777	0.0658768
NSMF	0.7402793	0.5603727	0.4184361	0.7637835	0.5069366

PNPLA7	0.0152346	0.0192123	0.0138938	0.0317598	0.0678815
MRPL41	0.8128303	0.8713977	0.8358529	0.8443052	0.6203809
DPH7	0.1504527	0.1418101	0.1335319	0.1976188	0.1318066
ZMYND19	0.1578826	0.2334902	0.2915652	0.1362932	0.1305496
ARRDC1	0.0696810	0.0546168	0.0421027	0.0705171	0.0671900
ARRDC1-AS1	0.1507500	0.1553557	0.1749448	0.1338213	0.0788250
EHMT1	0.3660963	0.3399999	0.3632027	0.3220770	0.5638961
ENSG00000203987	0.0007099	0.0016718	0.0000000	0.0000000	0.0061857
CACNA1B	0.1606183	0.2027297	0.1418155	0.1390793	0.8983567
ENSG00000290588	0.0020144	0.0084195	0.0118223	0.0000000	0.0220870
ENSG00000237419	0.0149261	0.0009224	0.0035058	0.0103927	0.0000000
TUBB8	0.0007592	0.0007934	0.0000000	0.0000000	0.0000000
ZMYND11	0.7099441	0.8134404	0.8534598	0.7298661	0.8184471
DIP2C	0.3670195	0.4405207	0.3788974	0.2836646	1.2243477
DIP2C-AS1	0.0000000	0.0000000	0.0023101	0.0000000	0.0041152
LARP4B	0.3303922	0.3608263	0.3651229	0.2753471	0.5180601
LARP4B-DT	0.0002291	0.0000000	0.0052748	0.0000000	0.0000000
ENSG00000205740	0.0062939	0.0058138	0.0053870	0.0000000	0.0186235
GTPBP4	0.4154756	0.3878160	0.3550189	0.3852556	0.3371327
IDI2	0.0003199	0.0000000	0.0000000	0.0033129	0.0056530
IDI2-AS1	0.0000000	0.0054169	0.0000000	0.0077039	0.0305291
IDI1	0.6260884	0.6747582	0.7983785	0.7226720	0.5498954
WDR37	0.1765887	0.2475809	0.2771856	0.1199067	0.3303742
LINC00200	0.0000000	0.0024450	0.0034238	0.0032157	0.0000000
ADARB2	0.1248465	0.1936024	0.1482264	0.0967160	1.1710785
ENSG00000282390	0.0000000	0.0008568	0.0067956	0.0000000	0.0077610
ADARB2-AS1	0.0000000	0.0000000	0.0000000	0.0000000	0.0065562
ENSG00000235281	0.0044316	0.0099780	0.0000000	0.0000000	0.0000000
ENSG00000287560	0.0908251	0.0599046	0.0290699	0.0747040	0.0664152
PFKP-DT	0.0302004	0.0214370	0.0334699	0.0138116	0.0233691
PFKP	0.9332406	0.9465661	1.0174140	0.9129657	1.0877772
PITRM1	0.2063753	0.2676843	0.2680433	0.1739710	0.1810940
PITRM1-AS1	0.0134883	0.0173112	0.0042414	0.0113928	0.0364013
LINC02668	0.0000000	0.0000000	0.0012400	0.0000000	0.0000000
LINC02669	0.0000000	0.0020593	0.0000000	0.0031392	0.0091577
KLF6	0.6111148	0.4710596	0.5045132	0.5593966	0.6794070
ENSG00000288755	0.0175171	0.0074899	0.0038451	0.0176151	0.0492154
ENSG00000287175	0.0000000	0.0000000	0.0000000	0.0000000	0.0045046
LINC02660	0.0030464	0.0000000	0.0000000	0.0000000	0.0000000
MANCR	0.0010363	0.0021460	0.0000000	0.0000000	0.0000000
AKR1E2	0.0213818	0.0527983	0.0620041	0.0229087	0.0306946
AKR1C1	0.1538093	0.2771618	0.4508201	0.0716220	0.2058670
AKR1C2	0.1348313	0.3001634	0.4623596	0.1218348	0.1791165
ENSG00000224251	0.0009199	0.0000000	0.0000000	0.0000000	0.0000000
AKR1C3	0.0026067	0.0000000	0.0036983	0.0000000	0.0085155
NET1	0.1186482	0.1212786	0.0976011	0.0825039	0.0986039
LASTR	0.0004268	0.0043489	0.0045528	0.0000000	0.0000000
ASB13	0.0322126	0.0287492	0.0468353	0.0306132	0.0689470
TASOR2	0.3242540	0.3701565	0.2915086	0.3050847	0.4790329
ENSG00000226647	0.0043120	0.0029262	0.0035884	0.0024208	0.0224909
GDI2	1.1239086	1.1451355	1.1318857	1.0843587	0.9514774
ENSG00000272764	0.0039837	0.0009219	0.0000000	0.0000000	0.0000000
ANKRD16	0.1201080	0.1074554	0.0846698	0.1016954	0.1235193

FBH1	0.1451590	0.1292226	0.1204886	0.1514248	0.1406020
ENSG00000232807	0.0000000	0.0003989	0.0000000	0.0000000	0.0000000
IL15RA	0.0005573	0.0000000	0.0007625	0.0057846	0.0000000
RBM17	0.9946749	0.8707325	0.8336686	0.9917457	0.7991138
PFKFB3	0.2721460	0.2545457	0.2226698	0.2425702	0.3808132
ENSG00000213994	0.0055587	0.0054869	0.0096120	0.0033394	0.0000000
LINC02649	0.0338325	0.0414959	0.0286991	0.0494950	0.1969364
LINC02656	0.0052430	0.0027684	0.0039890	0.0072119	0.0041021
PRKCQ	0.0264959	0.0363320	0.0353459	0.0197071	0.0515851
ENSG00000288915	0.0012450	0.0000000	0.0000000	0.0000000	0.0000000
PRKCQ-AS1	0.0397153	0.0495352	0.0349926	0.0353720	0.0548240
LINP1	0.0000000	0.0050954	0.0000000	0.0000000	0.0000000
LINC00707	0.0000000	0.0000000	0.0000000	0.0028552	0.0106510
ENSG00000287277	0.0045466	0.0021593	0.0000000	0.0018030	0.0173194
SFMBT2	0.1690460	0.1718041	0.1301135	0.1218133	0.4829407
LINC02642	0.0014007	0.0000000	0.0000000	0.0000000	0.0000000
ITIH5	0.8438915	0.4564286	0.3009424	1.1838330	0.4602164
ITIH2	0.0713125	0.0578860	0.0196739	0.1375906	0.0400885
KIN	0.3063165	0.2906838	0.2607955	0.3526438	0.2214760
ATP5F1C	1.3520655	1.4059158	1.4918105	1.3759702	1.1245478
TAF3	0.2264865	0.2151961	0.2599917	0.1551048	0.4365947
GATA3	0.1703914	0.3287202	0.9220767	0.1430596	0.4940504
GATA3-AS1	0.0464113	0.0791583	0.2318663	0.0398022	0.0711377
ENSG00000226990	0.0026535	0.0047283	0.0026368	0.0044555	0.0261371
CELF2-DT	0.0024903	0.0000000	0.0012620	0.0037517	0.0000000
CELF2	0.6597205	0.5728104	0.5291194	0.6393665	0.8350343
CELF2-AS2	0.0005420	0.0000000	0.0000000	0.0000000	0.0156948
USP6NL	0.3369740	0.3278701	0.2592667	0.3488884	0.4199597
ECHDC3	0.1420532	0.0851941	0.0424276	0.1240137	0.0653954
PROSER2	0.0244506	0.0151201	0.0172838	0.0331205	0.0334197
PROSER2-AS1	0.0000000	0.0000000	0.0000000	0.0000000	0.0000000
UPF2	0.4017283	0.3555606	0.3318864	0.3576258	0.3784025
DHTKD1	0.1507596	0.1340438	0.1077300	0.1782001	0.1796783
SEC61A2	0.1516941	0.1835192	0.2442064	0.1295575	0.2684984
NUDT5	0.2878757	0.2160496	0.2053243	0.3425195	0.2260471
CDC123	0.4404570	0.4243560	0.4657420	0.4848263	0.3660074
ENSG00000228302	0.0004332	0.0000000	0.0000000	0.0018419	0.0048432
CAMK1D	0.4308487	0.5876336	0.5288183	0.3216457	0.8869791
ENSG00000285994	0.0000000	0.0000000	0.0000000	0.0000000	0.0000000
ENSG00000285520	0.0013417	0.0013745	0.0018826	0.0000000	0.0159164
CCDC3	0.4897346	0.3137484	0.2119710	0.3597808	0.3813839
OPTN	0.9596468	1.1655963	1.2216560	0.8766953	0.9366968
MCM10	0.0050901	0.0070431	0.0000000	0.0128125	0.0000000
UCMA	0.0009223	0.0000000	0.0010626	0.0031683	0.0000000
PHYH	0.3889181	0.3343521	0.3902015	0.3930553	0.3220690
SEPHS1	0.3597016	0.3235378	0.3362582	0.3827397	0.3236735
ENSG00000289585	0.0029793	0.0098444	0.0026596	0.0000000	0.0000000
BEND7	0.2096310	0.2489825	0.3034654	0.1866670	0.1687345
PRPF18	0.2964716	0.3370922	0.3138000	0.2587910	0.3927869
FRMD4A	0.9730856	0.8627167	0.7939263	0.8922642	1.7171496
FRMD4A-AS1	0.0043100	0.0024896	0.0000000	0.0102675	0.0079109
ENSG00000229751	0.0014803	0.0031494	0.0057133	0.0036850	0.0209420
ENSG00000235410	0.0003926	0.0017643	0.0000000	0.0000000	0.0000000

FAM107B	0.5113973	0.4937673	0.5793015	0.5352022	0.4554577
ENSG00000236495	0.0000000	0.0000000	0.0000000	0.0029316	0.0000000
CDNF	0.0253718	0.0178628	0.0157874	0.0038691	0.0197289
MSANTD7	0.0904591	0.0850289	0.1020877	0.0489855	0.0875774
HSPA14	0.1125915	0.0947686	0.1058483	0.1182116	0.1583166
SUV39H2-DT	0.0115838	0.0164384	0.0135336	0.0072652	0.0198865
SUV39H2	0.0980421	0.1025378	0.1095872	0.1304442	0.1009430
DCLRE1C	0.0492142	0.0573949	0.0765958	0.0367785	0.0577303
MEIG1	0.0956615	0.0593931	0.0668594	0.0876483	0.0266709
OLAH	0.0000000	0.0027311	0.0000000	0.0000000	0.0000000
ACBD7	0.0360095	0.0285971	0.0452448	0.0242254	0.0393275
RPP38-DT	0.0097988	0.0131486	0.0033015	0.0088403	0.0027533
RPP38	0.2675628	0.1589949	0.1394713	0.2812567	0.2327008
NMT2	0.1681644	0.1578948	0.2139056	0.1389077	0.2172599
FAM171A1	0.2710449	0.3180002	0.2671130	0.2993847	0.4750480
ENSG00000232739	0.0000000	0.0000000	0.0000000	0.0000000	0.0044554
ITGA8	0.0194626	0.0215275	0.0163046	0.0215711	0.0811753
MINDY3	0.3915140	0.4178496	0.4709133	0.3979589	0.4389419
PTER	0.0041678	0.0032326	0.0010011	0.0000000	0.0165682
C1QL3	0.0253427	0.0229991	0.0298316	0.0301961	0.0081342
ENSG00000287925	0.0047059	0.0042335	0.0080845	0.0000000	0.0000000
RSU1	0.5069014	0.4254935	0.3998041	0.5644332	0.4945493
CUBN	0.1054831	0.0785523	0.0419900	0.0649575	0.1236704
TRDMT1	0.1241991	0.1095019	0.0929562	0.1434572	0.1488166
VIM-AS1	0.1407450	0.0616309	0.0284535	0.1006723	0.1006451
VIM	4.8529023	4.2315482	3.7069494	4.8997683	4.0552660
ENSG00000234961	0.0452385	0.0277794	0.0246675	0.0539367	0.0473366
ST8SIA6	0.0062623	0.0182860	0.0314759	0.0107368	0.0192870
ST8SIA6-AS1	0.0000000	0.0013584	0.0021490	0.0000000	0.0000000
HACD1	0.0852102	0.2061333	0.2846254	0.0932476	0.1338305
STAM-DT	0.0061705	0.0020274	0.0048965	0.0016838	0.0039740
STAM	0.3598766	0.3907644	0.3886483	0.2883426	0.3349013
ENSG00000229190	0.0006048	0.0000000	0.0000000	0.0000000	0.0000000
TMEM236	0.0006590	0.0000000	0.0000000	0.0000000	0.0063635
MRC1	0.0000000	0.0032028	0.0072443	0.0000000	0.0035346
SLC39A12	0.0025152	0.0008632	0.0000000	0.0134684	0.0000000
CACNB2	0.1455285	0.2117028	0.2572868	0.1496402	0.7334477
ENSG00000235020	0.0002884	0.0000000	0.0103864	0.0033824	0.0230476
ENSG00000240291	0.0192561	0.0262417	0.0264917	0.0032388	0.1194761
NSUN6	0.1949282	0.2333432	0.2282278	0.1729632	0.4144150
ARL5B	0.4338637	0.3973377	0.4323500	0.3228554	0.3813159
MALRD1	0.0093776	0.0104679	0.0086501	0.0000000	0.0706729
ENSG00000233968	0.0064787	0.0000000	0.0099664	0.0000000	0.0064396
ENSG00000285852	0.0056212	0.0104470	0.0403876	0.0105600	0.0076956
PLXDC2	0.7799622	0.6296421	0.6190639	1.0355685	1.1266902
ENSG00000238246	0.0000000	0.0000000	0.0010558	0.0000000	0.0057461
ENSG00000287613	0.0017105	0.0038551	0.0000000	0.0020645	0.0000000
NEBL	1.0732722	0.7807316	0.6331770	0.9304444	1.3496069
NEBL-AS1	0.1968321	0.0919024	0.0681018	0.1312192	0.0836443
LINC02643	0.0000000	0.0000000	0.0000000	0.0000000	0.0073662
MIR1915HG	0.0925645	0.0796392	0.0710582	0.0931471	0.0482780
SKIDA1	0.0785869	0.0651707	0.0654957	0.0795152	0.0601758
MLLT10	0.3191333	0.3107908	0.2845927	0.2811351	0.4966512

ENSG00000286881	0.0041038	0.0011874	0.0031125	0.0042466	0.0000000
ENSG00000289528	0.0000000	0.0021277	0.0000000	0.0097268	0.0152037
DNAJC1	0.4759570	0.3167291	0.2283801	0.4660771	0.4114527
ENSG00000279623	0.0000000	0.0032587	0.0000000	0.0000000	0.0000000
COMMD3	0.4921656	0.4558117	0.4877206	0.4404248	0.3616045
BMI1	0.5669882	0.4980693	0.6060140	0.4913850	0.3930473
SPAG6	0.3259706	0.2134053	0.1360292	0.3877917	0.1699174
ENSG00000233451	0.0017574	0.0000000	0.0000000	0.0000000	0.0000000
ENSG00000286810	0.0023259	0.0087353	0.0087184	0.0000000	0.0097492
PIP4K2A	0.2798336	0.3583394	0.4412224	0.2453828	0.3612942
ARMC3	0.6723810	0.3775023	0.2786507	0.6378005	0.4253100
ENSG00000286924	0.0548460	0.0513654	0.0311786	0.0321002	0.0647015
MSRB2	0.5589823	0.5957493	0.5850628	0.5521089	0.4140524
PTF1A	0.0025408	0.0013955	0.0036231	0.0045055	0.0000000
C10orf67	0.0200083	0.0169916	0.0074919	0.0297524	0.0065159
C10orf67-AS1	0.0019892	0.0016068	0.0009005	0.0104019	0.0000000
ENSG00000287124	0.0067621	0.0041266	0.0075840	0.0000000	0.0344587
OTUD1	0.0794110	0.0837958	0.0573950	0.0812631	0.0767182
KIAA1217	0.0818073	0.1302770	0.0759521	0.0999211	0.3257280
ENSG00000289188	0.0016796	0.0000000	0.0000000	0.0000000	0.0000000
ARHGAP21	0.7264323	0.7068630	0.7183318	0.7841317	0.8900647
PRTFDC1	0.3509425	0.2701928	0.1738112	0.4175419	0.2064805
ENSG00000273107	0.0140113	0.0100588	0.0120063	0.0142282	0.0000000
ENKUR	0.7400518	0.4708377	0.2944684	0.8319342	0.3700461
THNSL1	0.1103837	0.1170644	0.1188435	0.1230151	0.0668111
LINC01516	0.0004411	0.0000000	0.0000000	0.0000000	0.0000000
GPR158-AS1	0.0039766	0.0108552	0.0071199	0.0036873	0.0090768
GPR158	0.0749514	0.1009735	0.0972113	0.0778041	0.4138974
LINC00836	0.0000000	0.0020144	0.0000000	0.0000000	0.0000000
MYO3A	0.1064213	0.0752713	0.0379306	0.0750830	0.1111146
GAD2	0.0595110	0.1195943	0.0815882	0.0384373	0.1417169
APBB1IP	0.0013780	0.0012926	0.0029554	0.0000000	0.0000000
PDSS1	0.0834140	0.0679092	0.0679999	0.1037816	0.1163368
ENSG00000235843	0.0000000	0.0000000	0.0000000	0.0000000	0.0000000
ABI1	0.3830101	0.3147550	0.3281889	0.3777138	0.4312459
FAM238C	0.0015203	0.0016677	0.0024154	0.0000000	0.0115530
ANKRD26	0.3985434	0.3830690	0.3685339	0.3325575	0.5895935
YME1L1	0.8743515	0.7824460	0.7374106	0.8798339	0.8240034
MASTL	0.0304118	0.0528751	0.0231804	0.0598742	0.0063205
ACBD5	0.8279951	0.7285830	0.7531137	0.7960482	0.6663801
ENSG00000262412	0.0041169	0.0086681	0.0073473	0.0041868	0.0199615
ENSG00000290843	0.0075543	0.0096217	0.0129966	0.0012957	0.0031362
RAB18	0.9451175	0.8988143	0.9625108	0.9883302	0.7365538
LINC02680	0.0093690	0.0158770	0.0079605	0.0146991	0.0141447
MKX	0.0095921	0.0000000	0.0050086	0.0119816	0.0000000
ODAD2	0.0796847	0.0481834	0.0283752	0.0931410	0.0406106
ENSG00000233472	0.0008190	0.0014455	0.0000000	0.0000000	0.0000000
MPP7	0.1074171	0.1047356	0.0900460	0.1015186	0.1647697
MPP7-DT	0.0000000	0.0002873	0.0018108	0.0000000	0.0000000
LINC02652	0.0011594	0.0023539	0.0000000	0.0000000	0.0210324
WAC-AS1	0.3532631	0.4203565	0.5131603	0.3223505	0.2835771
WAC	0.8531155	0.7247243	0.7722021	0.7995928	0.8528149
BAMBI	0.0481109	0.0409108	0.0550767	0.0604600	0.0942730

LINC01517	0.0000000	0.0022401	0.0013856	0.0070733	0.0000000
ENSG00000287402	0.0017669	0.0137888	0.0153722	0.0087935	0.0127077
SVIL-AS1	0.4394934	0.5026905	0.4986511	0.4224264	0.5876473
SVIL	0.5653434	0.4502322	0.3608354	0.4479558	0.6488165
JCAD	0.0206688	0.0204359	0.0282738	0.0146264	0.0441759
ENSG00000259994	0.0347054	0.0328792	0.0608352	0.0069468	0.0464479
MTPAP	0.2869778	0.3909597	0.4955517	0.2753531	0.3627910
ENSG00000285824	0.0096458	0.0212923	0.0104198	0.0157777	0.0338582
MAP3K8	0.0466671	0.0349430	0.0201083	0.0794214	0.0786696
ZNF438	0.1150874	0.0920552	0.0768909	0.1237099	0.1909267
ENSG00000272914	0.0017745	0.0000000	0.0000000	0.0024018	0.0014454
ENSG00000287564	0.0035262	0.0074180	0.0081840	0.0043212	0.0159563
LINC02664	0.0000000	0.0062338	0.0000000	0.0000000	0.0000000
ZEB1-AS1	0.1045672	0.0909993	0.1032735	0.0989648	0.1115401
ZEB1	0.6761931	0.6089078	0.6107470	0.5953343	0.9213213
ENSG00000223834	0.0006195	0.0041346	0.0024957	0.0075044	0.0050279
ENSG00000289412	0.0016638	0.0039849	0.0000000	0.0058194	0.0000000
ARHGAP12	0.5518214	0.4848199	0.4882030	0.6610980	0.5318758
KIF5B	1.4893082	1.3013224	1.2429431	1.3492773	1.1590331
ENSG00000227253	0.0011727	0.0015459	0.0000000	0.0000000	0.0000000
EPC1	0.5793979	0.5603255	0.5046955	0.5397744	0.8059896
EPC1-AS1	0.0009043	0.0046765	0.0000000	0.0000000	0.0404114
ENSG00000286409	0.0014201	0.0007627	0.0061742	0.0000000	0.0030021
CCDC7	0.1322245	0.1556558	0.0984524	0.0923336	0.3557684
ITGB1	1.4060175	1.0696439	0.8481507	1.8778719	1.1015984
ITGB1-DT	0.0325130	0.0373111	0.0213285	0.0360320	0.0385576
NRP1	0.3671065	0.3687168	0.2252697	0.4693614	0.5533577
ENSG00000287278	0.0031344	0.0000000	0.0000000	0.0053014	0.0000000
PARD3	0.4362770	0.3452046	0.2440520	0.4320584	0.7728407
PARD3-DT	0.0026645	0.0049708	0.0033911	0.0022792	0.0000000
LINC02635	0.0000000	0.0000000	0.0000000	0.0000000	0.0000000
CUL2	0.4591135	0.5004145	0.5697304	0.4450614	0.5111192
ENSG00000230534	0.0000000	0.0040478	0.0000000	0.0000000	0.0000000
CREM	0.4796736	0.3877252	0.3523667	0.4764857	0.3800800
ENSG00000269952	0.0017543	0.0000000	0.0013516	0.0026398	0.0000000
LINC02634	0.0060921	0.0095671	0.0044338	0.0036897	0.0039501
CCNY	0.6927977	0.6326841	0.5535308	0.6406635	0.8543376
CCNY-AS1	0.0332535	0.0133025	0.0220779	0.0369516	0.0750821
FZD8	0.0101346	0.0025326	0.0179569	0.0000000	0.0000000
ENSG00000287528	0.0023516	0.0088396	0.0137148	0.0000000	0.0067671
ANKRD30A	0.0039759	0.0000000	0.0012308	0.0000000	0.0000000
ENSG00000226578	0.0004064	0.0000000	0.0010313	0.0000000	0.0000000
ZNF248	0.2800151	0.3750875	0.4403797	0.2341787	0.3981600
ENSG00000236514	0.0102388	0.0055495	0.0033698	0.0104391	0.0056180
ZNF25	0.2296824	0.2421766	0.3354251	0.2084086	0.3045536
ZNF25-DT	0.0000000	0.0000000	0.0000000	0.0000000	0.0000000
ZNF33A	0.3758501	0.4383171	0.6340452	0.3005608	0.5005642
ZNF37A	0.1808709	0.1882325	0.2036241	0.1676579	0.3569543
ENSG00000272983	0.0077965	0.0018838	0.0096342	0.0016127	0.0204065
ENSG00000290887	0.0216187	0.0093959	0.0155210	0.0069980	0.0681191
HSD17B7P2	0.0064523	0.0101253	0.0085156	0.0118033	0.0235416
SEPTIN7P9	0.0020941	0.0000000	0.0015418	0.0000000	0.0149662
ENSG00000290535	0.0137749	0.0067744	0.0130159	0.0033810	0.0000000

ENSG00000203496	0.0000000	0.0000000	0.0000000	0.0000000	0.0000000
ENSG00000290458	0.0000000	0.0000000	0.0000000	0.0000000	0.0000000
ENSG00000291065	0.1608293	0.1635734	0.1633239	0.1040447	0.3681745
CCNYL2	0.0000000	0.0000000	0.0000000	0.0000000	0.0000000
LINC00839	0.0000000	0.0033603	0.0000000	0.0000000	0.0000000
ZNF33B	0.1991333	0.3089837	0.3450572	0.1842852	0.2971235
ENSG00000285884	0.0376860	0.0242944	0.0452787	0.0311139	0.0101294
ENSG00000259869	0.0000000	0.0079354	0.0022183	0.0000000	0.0189901
BMS1	0.2901783	0.2765421	0.3569799	0.2629628	0.4112930
RET	0.0966272	0.2202715	0.1348755	0.0759034	0.1683974
CSGALNACT2-DT	0.0040088	0.0075420	0.0167081	0.0057098	0.0056964
CSGALNACT2	0.1840981	0.3016906	0.2384791	0.1817180	0.3275786
RASGEF1A	0.0287802	0.0731304	0.1068091	0.0141273	0.0304736
ENSG00000285712	0.0041819	0.0023378	0.0000000	0.0000000	0.0000000
HNRNPF	0.9052243	0.6514002	0.5688862	1.0289946	0.6554341
LINC02916	0.0222505	0.0225209	0.0331433	0.0381177	0.0093219
ZNF487	0.1653434	0.1168716	0.1103504	0.1620222	0.1591918
ZNF239	0.0252137	0.0390692	0.0381274	0.0312890	0.0250134
ZNF485	0.0234681	0.0279537	0.0250897	0.0408402	0.0391819
ZNF32-AS3	0.0117480	0.0214899	0.0208160	0.0149440	0.0207369
ENSG00000237389	0.0006768	0.0000000	0.0000000	0.0013887	0.0000000
ZNF32	0.3975746	0.4618712	0.4838160	0.3577590	0.3141240
ZNF32-AS2	0.0018344	0.0016506	0.0025702	0.0000000	0.0000000
LINC00840	0.0000000	0.0011573	0.0000000	0.0000000	0.0000000
ENSG00000229116	0.0024084	0.0012869	0.0000000	0.0022405	0.0000000
ENSG00000287901	0.0286248	0.0107643	0.0091724	0.0536949	0.0000000
CXCL12	0.4683069	0.2410598	0.1483794	0.9139786	0.1671242
TMEM72-AS1	0.0241711	0.0438649	0.0520652	0.0287099	0.0697157
TMEM72	0.0007888	0.0017861	0.0000000	0.0000000	0.0000000
ENSG00000223462	0.0000000	0.0024622	0.0000000	0.0000000	0.0000000
ENSG00000273363	0.0010218	0.0000000	0.0000000	0.0000000	0.0000000
RASSF4	0.1715169	0.1382384	0.1392372	0.2071468	0.1388212
DEPP1	0.0796536	0.0660225	0.0325044	0.0511954	0.0734891
ZNF22-AS1	0.0319392	0.0312983	0.0968112	0.0450837	0.0156987
ZNF22	0.7393104	0.6103391	0.7062476	0.7506740	0.4853567
ENSG00000227683	0.0011284	0.0000000	0.0000000	0.0000000	0.0000000
OR13A1	0.0000000	0.0000000	0.0030846	0.0000000	0.0000000
ALOX5	0.0046849	0.0032398	0.0066102	0.0000000	0.0131010
ENSG00000231964	0.0000000	0.0000000	0.0000000	0.0000000	0.0028666
MARCHF8	0.1831444	0.1691773	0.2115375	0.1566107	0.2420624
ZFAND4	0.1115598	0.1040587	0.1455822	0.1149443	0.0767557
ENSG00000290460	0.0083976	0.0075317	0.0178387	0.0094021	0.0309438
WASHC2C	0.1282311	0.1113888	0.1418118	0.1027531	0.1380474
FAM25EP	0.0000000	0.0028840	0.0000000	0.0000000	0.0000000
AGAP4	0.0192339	0.0349794	0.0244621	0.0237270	0.0541854
ENSG00000290921	0.0613667	0.0735699	0.0646542	0.0486395	0.2681207
TIMM23	0.2679141	0.2749170	0.2881273	0.2950155	0.2511076
NCOA4	0.5189198	0.5510795	0.5522515	0.5090090	0.3945405
ENSG00000289092	0.0112182	0.0132339	0.0116427	0.0039347	0.0044999
LINC00842	0.0053643	0.0077797	0.0017502	0.0086197	0.0336083
GPRIN2	0.0480021	0.1077092	0.0887052	0.0225564	0.0887373
SYT15	0.0000000	0.0013899	0.0000000	0.0030924	0.0000000
SYT15-AS1	0.0658369	0.0631206	0.0652964	0.0537156	0.0705916

ENSG00000229227	0.0021015	0.0062170	0.0043036	0.0000000	0.0015072
ENSG00000290913	0.0110204	0.0160568	0.0087384	0.0056195	0.0060934
FAM245B	0.0020587	0.0000000	0.0000000	0.0000000	0.0050816
GLUD1P2	0.0376407	0.0597887	0.0612343	0.0171143	0.0608275
BMS1P1	0.0164606	0.0166361	0.0196816	0.0080780	0.0588295
PTPN20	0.0031533	0.0017206	0.0064705	0.0000000	0.0000000
GDF10	0.0047350	0.0337620	0.0265806	0.0036255	0.0213122
ZNF488	0.0000000	0.0033071	0.0000000	0.0000000	0.0000000
ENSG00000224919	0.0009600	0.0004001	0.0000000	0.0000000	0.0014580
AGAP9	0.0131521	0.0118583	0.0108845	0.0032095	0.0526102
ENSG00000290453	0.0062782	0.0163027	0.0036477	0.0207982	0.0768619
LINC02675	0.0007163	0.0000000	0.0000000	0.0000000	0.0000000
SYT15B	0.0081253	0.0072958	0.0040417	0.0093606	0.0042436
ENSG00000276850	0.0007181	0.0023744	0.0000000	0.0000000	0.0000000
AGAP12P	0.0013367	0.0015309	0.0009802	0.0000000	0.0090365
FRMPD2	0.0017056	0.0010069	0.0000000	0.0093605	0.0000000
MAPK8	0.3705826	0.5586896	0.6767092	0.3512452	0.8095430
ARHGAP22	0.0903573	0.1176583	0.0773342	0.0548449	0.1555179
WDFY4	0.0000000	0.0000000	0.0000000	0.0000000	0.0000000
LRRC18	0.0094111	0.0082312	0.0000000	0.0079642	0.0037728
VSTM4	0.0528473	0.0560800	0.0271587	0.0727724	0.0606048
ENSG00000235939	0.0135608	0.0137906	0.0111196	0.0037014	0.0131377
ERCC6	0.2056721	0.1912084	0.1413854	0.1527542	0.2290105
CHAT	0.0421332	0.0719818	0.0231624	0.0331155	0.0908032
SLC18A3	0.0437627	0.0992009	0.0316607	0.0174612	0.0424330
C10orf53	0.0014914	0.0022430	0.0006194	0.0000000	0.0118833
OGDHL	0.0376636	0.0635984	0.0437847	0.0247812	0.0901407
PARG	0.2461583	0.2891425	0.2870597	0.2370768	0.3766566
TIMM23B	0.0956061	0.1155228	0.0971989	0.1136351	0.3075522
ENSG00000285803	0.0005490	0.0005507	0.0000000	0.0000000	0.0000000
AGAP6	0.0293891	0.0386969	0.0295380	0.0026231	0.0714958
FAM21EP	0.0148359	0.0073812	0.0044897	0.0157782	0.0019839
WASHC2A	0.1263455	0.1522647	0.1453025	0.1381782	0.1223218
ASAH2	0.0142857	0.0253050	0.0152791	0.0099516	0.0024307
SGMS1	0.2878576	0.2942204	0.2284914	0.2998013	0.5436621
ENSG00000286401	0.0036616	0.0010087	0.0087896	0.0047565	0.0034730
SGMS1-AS1	0.0157090	0.0057712	0.0102630	0.0206027	0.0092171
ENSG00000287221	0.0078822	0.0112267	0.0133688	0.0135524	0.0133374
ASAH2B	0.0837392	0.0683470	0.0943398	0.0530628	0.0520703
A1CF	0.0022054	0.0014800	0.0000000	0.0000000	0.0000000
PRKG1	0.4574817	0.3879631	0.2799684	0.3675163	1.0084577
ENSG00000223502	0.0171263	0.0087946	0.0113787	0.0297320	0.0000000
ENSG00000289270	0.0013467	0.0000000	0.0025512	0.0055470	0.0000000
CSTF2T	0.1799161	0.1775750	0.1632305	0.1726166	0.1200341
PRKG1-AS1	0.0026340	0.0047163	0.0016963	0.0000000	0.0114894
DKK1	0.0102945	0.0146706	0.0205251	0.0034725	0.0168566
PCDH15	0.1127835	0.1658821	0.1079118	0.0981654	0.5383286
ENSG00000234173	0.0009176	0.0005411	0.0014971	0.0000000	0.0096498
ENSG00000236958	0.0000000	0.0040384	0.0016900	0.0000000	0.0046437
ENSG00000236744	0.0008655	0.0000000	0.0000000	0.0000000	0.0000000
ENSG00000228048	0.0000000	0.0000000	0.0000000	0.0000000	0.0000000
ZWINT	0.0195645	0.0167933	0.0069839	0.0400973	0.0000000
ENSG00000287016	0.0059783	0.0030021	0.0091419	0.0043147	0.0028791

IPMK	0.1944408	0.2325518	0.2577399	0.1162385	0.3168245
CISD1	0.7185389	0.8554550	1.0530279	0.7318199	0.7061817
ENSG00000228527	0.0041264	0.0000000	0.0024042	0.0000000	0.0000000
UBE2D1	0.6170745	0.6235886	0.7670446	0.6140329	0.5315032
TFAM	0.4908149	0.4781493	0.4664984	0.4564829	0.3115892
BICC1	0.3201534	0.2532327	0.2316021	0.2249196	0.3869729
LINC00844	0.0097275	0.0007002	0.0011103	0.0131081	0.0000000
PHYHIPL	0.4241594	0.7561967	1.1075368	0.4594682	0.7588356
FAM13C	0.2202172	0.3378747	0.3764255	0.2067693	0.3877760
ENSG00000236556	0.0000000	0.0000000	0.0000000	0.0000000	0.0031339
ENSG00000287969	0.0024308	0.0008354	0.0000000	0.0021398	0.0049154
ENSG00000235140	0.0000000	0.0000000	0.0000000	0.0000000	0.0074786
SLC16A9	0.3318186	0.2530828	0.1784475	0.4121491	0.3588602
MRLN	0.0034569	0.0000000	0.0013992	0.0040528	0.0063839
CCDC6	0.6228571	0.6608024	0.7742406	0.5306151	0.6587345
ANK3	1.0219065	1.4344582	1.4819301	0.8587966	2.2326509
ENSG00000232682	0.0086228	0.0078750	0.0046689	0.0154451	0.0072111
CDK1	0.0254247	0.0297085	0.0157455	0.0345538	0.0340152
RHOBTB1	0.1855546	0.1519058	0.0958438	0.1830665	0.1538364
ENSG00000289989	0.0056428	0.0000000	0.0084928	0.0000000	0.0000000
LINC00845	0.0053613	0.0099157	0.0104509	0.0072220	0.0173786
TMEM26	0.0050731	0.0118292	0.0000000	0.0042207	0.0062535
TMEM26-AS1	0.0006658	0.0049732	0.0000000	0.0000000	0.0071991
CABCOCO1	0.2200068	0.1547435	0.0804044	0.2521194	0.1119900
ARID5B	0.3260729	0.5362114	0.2124788	0.3906988	0.4102671
RTKN2	0.0353176	0.0323793	0.0296326	0.0288117	0.0207842
ENSG00000288011	0.0033061	0.0094305	0.0037159	0.0052311	0.0020838
ZNF365	0.0448499	0.0940146	0.1015497	0.0536952	0.0341412
LINC02929	0.0006738	0.0050545	0.0044931	0.0000000	0.0069651
ENSG00000238280	0.0011708	0.0012994	0.0000000	0.0000000	0.0035689
ADO	0.1296973	0.2253223	0.1975006	0.1587148	0.1566193
EGR2	0.0011613	0.0000000	0.0030259	0.0042005	0.0035216
NRBF2	0.3252651	0.3211880	0.3545690	0.3211852	0.2892517
JMJD1C	0.7722065	0.6792252	0.6366643	0.6824679	1.4577457
JMJD1C-AS1	0.0233833	0.0108949	0.0294995	0.0375684	0.0200674
REEP3	0.6318738	0.3950472	0.2465519	0.7987127	0.3624473
ENSG00000286373	0.0047316	0.0053341	0.0000000	0.0226151	0.0070862
ENSG00000228566	0.0072413	0.0066449	0.0070055	0.0133525	0.0325649
LINC02671	0.0000000	0.0011882	0.0000000	0.0000000	0.0000000
LINC01515	0.0655750	0.0446788	0.0421864	0.0456769	0.0795333
CTNNA3	0.0370669	0.0569258	0.0663313	0.0202720	0.1731256
LRRTM3	0.1635091	0.2725266	0.4353971	0.1250074	0.6682393
ENSG00000225299	0.0000000	0.0000000	0.0000000	0.0000000	0.0000000
DNAJC12	0.4182491	0.6000646	1.0291783	0.3243253	0.4698480
ENSG00000272892	0.0021878	0.0038734	0.0000000	0.0018436	0.0019007
SIRT1	0.2693438	0.2858960	0.3137727	0.2022923	0.3265471
HERC4	0.3429484	0.3431861	0.2973044	0.3535363	0.6710174
MYPN	0.0008461	0.0013354	0.0000000	0.0000000	0.0051612
ATOH7	0.0063426	0.0086092	0.0098947	0.0037958	0.0038839
LINC02640	0.0037221	0.0032617	0.0000000	0.0000000	0.0018039
PBLD	0.0704710	0.0678775	0.0311630	0.0584243	0.0597714
HNRNPH3	1.2790246	1.2329328	1.3063178	1.2105767	1.1009409
RUFY2	0.5980123	0.5823287	0.7004826	0.6406418	0.7261257

DNA2	0.0370440	0.0386870	0.0442914	0.0232125	0.0982965
SLC25A16	0.1259896	0.1389208	0.1838391	0.1474245	0.2687995
TET1	0.4459098	0.3885558	0.4066633	0.4462677	0.5248229
ENSG00000260400	0.0138808	0.0274113	0.0225980	0.0188931	0.0398533
CCAR1	0.3824908	0.3630168	0.3996612	0.3547475	0.5386628
STOX1	0.1465049	0.1077308	0.0589757	0.1424543	0.0712296
DDX50	0.7133771	0.5668528	0.4797464	0.5734538	0.6208656
DDX21	0.6140068	0.5321232	0.5147976	0.6231621	0.5183325
KIFBP	0.5052108	0.4677430	0.4591935	0.5625710	0.3919355
VPS26A	0.4120901	0.4583845	0.4467129	0.3741894	0.3751764
SUPV3L1	0.1607547	0.2201135	0.1626176	0.1963724	0.1827050
HKDC1	0.0061107	0.0012329	0.0000000	0.0000000	0.0000000
HK1	0.6426401	0.8402001	1.0015617	0.6302465	0.7504698
TACR2	0.0033363	0.0011956	0.0000000	0.0000000	0.0110132
TSPAN15	0.0267840	0.0237682	0.0194540	0.0375182	0.0422253
ENSG00000287306	0.0068826	0.0135448	0.0148581	0.0047007	0.0457539
ENSG00000236154	0.0281284	0.0112870	0.0060511	0.0166549	0.0000000
FAM241B	0.3325902	0.4827498	0.6530618	0.3511301	0.4082794
COL13A1	0.0039185	0.0028485	0.0101289	0.0000000	0.0041213
LINC02636	0.0015633	0.0030089	0.0028898	0.0023027	0.0205693
MACROH2A2	0.4950132	0.5937588	0.8369796	0.5009420	0.5940875
AIFM2	0.0907573	0.0725810	0.0298179	0.1236449	0.0744746
TYSND1	0.0407790	0.0357493	0.0164904	0.0804272	0.0416206
SAR1A	0.5626829	0.4926712	0.5187101	0.6017649	0.4072508
PPA1	0.7906903	1.0047396	1.0912210	0.8382936	0.7881816
NPFFR1	0.0009906	0.0000000	0.0000000	0.0000000	0.0000000
LRRC20	0.1001970	0.1196640	0.1180545	0.1303839	0.1661826
EIF4EBP2	0.4769550	0.5602465	0.5626643	0.4103053	0.4089797
NODAL	0.0000000	0.0018953	0.0089998	0.0052990	0.0014580
PALD1	0.0107186	0.0076961	0.0037783	0.0124103	0.0079453
ADAMTS14	0.0022189	0.0010709	0.0000000	0.0022536	0.0000000
SGPL1	0.1809731	0.1446061	0.0964716	0.2206672	0.2194748
PCBD1	0.9254482	1.0280295	1.0727947	0.9057656	0.7695938
ENSG00000285300	0.0013738	0.0014288	0.0000000	0.0000000	0.0000000
UNC5B	0.4090287	0.2580506	0.2048256	0.3555027	0.3539468
UNC5B-AS1	0.0564522	0.0714808	0.0557211	0.0632944	0.0285524
SLC29A3	0.0126058	0.0103298	0.0138423	0.0090399	0.0674109
CDH23	0.0133673	0.0092687	0.0097535	0.0053255	0.0649470
C10orf105	0.0749522	0.0428355	0.0186680	0.1288366	0.0336774
VSIR	0.0127551	0.0139789	0.0145650	0.0920737	0.0132218
PSAP	1.7514306	1.3638566	1.4106610	2.4916554	1.4110236
ENSG00000289592	0.0253118	0.0137349	0.0074014	0.0148458	0.0142239
CHST3	0.4268359	0.2150070	0.1565022	0.5596949	0.2153725
SPOCK2	0.3021719	0.4426595	0.7339802	0.4070028	0.5004459
ASCC1	0.3368683	0.3066324	0.3098212	0.3463769	0.3216890
ANAPC16	0.6972025	0.6417606	0.5464979	0.6626590	0.5160751
ENSG00000289506	0.0044293	0.0087426	0.0054659	0.0000000	0.0363856
DDIT4	1.3254788	1.1836414	0.9928228	1.1974701	0.9889908
DNAJB12	0.2042525	0.1648520	0.0931683	0.2839121	0.1800043
MICU1	0.4267976	0.4382168	0.4324143	0.4047484	0.5318827
ENSG00000282915	0.0000000	0.0000000	0.0000000	0.0000000	0.0000000
MCU	0.1773857	0.1721959	0.1546828	0.2337678	0.3945305
OIT3	0.0045049	0.0030550	0.0008650	0.0000000	0.0025831

PLA2G12B	0.0011842	0.0000000	0.0025402	0.0000000	0.0000000
P4HA1	1.8460771	1.3082492	1.0196076	2.1510093	1.3779222
ENSG00000272630	0.0010030	0.0027234	0.0085340	0.0000000	0.0104020
NUDT13	0.0090519	0.0105787	0.0036344	0.0128869	0.0216599
ENSG00000272599	0.0011053	0.0000000	0.0000000	0.0000000	0.0029698
ECD	0.1798924	0.1663369	0.1518695	0.1926516	0.2442525
FAM149B1	0.1742348	0.1940970	0.1278710	0.1205464	0.1792936
DNAJC9	0.2721230	0.2724212	0.3652365	0.3001944	0.3476641
ENSG00000288559	0.0014992	0.0029025	0.0000000	0.0000000	0.0000000
DNAJC9-AS1	0.0098036	0.0093562	0.0126954	0.0062501	0.0068119
MRPS16	0.5529782	0.4910978	0.4738999	0.5357511	0.3895850
DNAJC9-AS1.1	0.0514763	0.0571247	0.0427968	0.0263925	0.0267542
CFAP70	0.1052194	0.0850020	0.0873542	0.1440178	0.1559471
ANXA7	0.5369726	0.3981703	0.2900476	0.5870765	0.3447776
MSS51	0.0019436	0.0056910	0.0109931	0.0082238	0.0228209
PPP3CB	0.5545907	0.7928115	0.9404512	0.4694896	0.7683881
PPP3CB-AS1	0.0149380	0.0030580	0.0021636	0.0245124	0.0242004
USP54	0.1133234	0.0966732	0.1284044	0.1464261	0.2753824
ENSG00000268584	0.0098828	0.0143451	0.0081084	0.0077758	0.0139340
ENSG00000272791	0.0005748	0.0011368	0.0059150	0.0000000	0.0052918
MYOZ1	0.0151394	0.0068268	0.0081802	0.0059064	0.0073980
SYNPO2L	0.0002810	0.0037176	0.0000000	0.0018843	0.0000000
SYNPO2L-AS1	0.0123247	0.0153558	0.0102058	0.0068980	0.0399796
AGAP5	0.0131537	0.0143713	0.0058555	0.0031040	0.0333347
ENSG00000272140	0.0066870	0.0025093	0.0016166	0.0042145	0.0000000
ENSG00000290737	0.0115697	0.0196954	0.0113655	0.0044246	0.0336200
GLUD1P3	0.0445343	0.0489668	0.0515732	0.0285718	0.0277091
SEC24C	0.1703095	0.1179773	0.1025900	0.1679935	0.1421417
FUT11	0.3873352	0.4103562	0.4050562	0.3760992	0.3893707
ENSG00000288823	0.0048359	0.0051198	0.0133000	0.0000000	0.0000000
CHCHD1	0.4476437	0.4337548	0.4329189	0.4868375	0.2269478
ZSWIM8	0.1062384	0.0788610	0.0613799	0.0797414	0.1615164
ZSWIM8-AS1	0.0028260	0.0035475	0.0000000	0.0000000	0.0133115
NDST2	0.0385339	0.0437092	0.0314294	0.0187782	0.0286115
CAMK2G	0.2827390	0.4922381	0.4528462	0.3024304	0.3938864
ENSG00000229990	0.0000000	0.0000000	0.0000000	0.0000000	0.0045792
PLAU	0.0000000	0.0030956	0.0022413	0.0000000	0.0097054
VCL	0.2333003	0.2215132	0.2372671	0.3013376	0.3362026
AP3M1	0.2815130	0.2687820	0.3002684	0.2668114	0.2195351
ADK	0.5620870	0.5394689	0.5526668	0.6084345	0.9195868
ENSG00000232342	0.0000000	0.0000000	0.0000000	0.0000000	0.0000000
KAT6B	0.4331616	0.5664223	0.6642616	0.4133586	0.7876794
ENSG00000234149	0.0016092	0.0000000	0.0000000	0.0000000	0.0139849
ENSG00000285810	0.0015891	0.0000000	0.0000000	0.0000000	0.0000000
DUSP13	0.0000000	0.0000000	0.0000000	0.0000000	0.0000000
SAMD8	0.2518912	0.2675679	0.3539529	0.2604158	0.3205649
VDAC2	1.2317443	1.3191077	1.3715126	1.1822578	1.0632712
COMTD1	0.1182853	0.1812836	0.3027485	0.0721202	0.1282221
ZNF503-AS1	0.0007117	0.0048837	0.0019501	0.0000000	0.0098493
ENSG00000270087	0.0039528	0.0044409	0.0087289	0.0188852	0.0115697
ZNF503	0.5884717	0.4685684	0.5993013	0.4581662	0.4234003
ZNF503-AS2	0.3465398	0.2314961	0.2188351	0.3442066	0.2029159
ENSG00000273248	0.0049028	0.0025681	0.0056666	0.0041603	0.0118502

ENSG00000272692	0.0069523	0.0042997	0.0040493	0.0071712	0.0064222
LRMDA	0.3333750	0.2409695	0.1864448	0.2878033	0.3656259
ENSG00000268659	0.0004647	0.0000000	0.0000000	0.0000000	0.0000000
KCNMA1	0.5491504	0.5559885	0.5264177	0.5534444	0.9876180
KCNMA1-AS1	0.0000000	0.0000000	0.0021247	0.0000000	0.0034590
KCNMA1-AS3	0.0000000	0.0000000	0.0000000	0.0000000	0.0000000
ENSG00000228748	0.1657732	0.0985964	0.0738488	0.1257248	0.1764688
DLG5	0.3955607	0.2975250	0.2560464	0.4195642	0.3442932
DLG5-AS1	0.0556987	0.0320693	0.0245969	0.0774713	0.0238836
POLR3A	0.2250386	0.2681763	0.3047889	0.2262814	0.2349330
RPS24	3.5915989	3.5613112	3.3262905	3.4836535	3.3318664
ENSG00000282863	0.0004084	0.0000000	0.0027536	0.0000000	0.0000000
LINC00595	0.0093979	0.0166052	0.0096367	0.0084709	0.0029896
ENSG00000228683	0.0010687	0.0000000	0.0023376	0.0000000	0.0000000
ZMIZ1-AS1	0.0052875	0.0046510	0.0091934	0.0132898	0.0168751
ZMIZ1	0.4403071	0.6404844	0.7612798	0.4192165	0.9535625
PPIF	0.1105116	0.1170401	0.0681971	0.1110020	0.0866442
ZCCHC24	0.0779127	0.0353036	0.0397845	0.0914594	0.0438924
ENSG00000235426	0.0005383	0.0053586	0.0000000	0.0000000	0.0000000
EIF5AL1	0.0157662	0.0181621	0.0110812	0.0298783	0.0205539
SFTPA2	0.0000000	0.0013833	0.0000000	0.0000000	0.0000000
LINC02679	0.0000000	0.0016301	0.0000000	0.0000000	0.0126899
ENSG00000244733	0.0000000	0.0008902	0.0000000	0.0000000	0.0041311
NUTM2B-AS1	0.2672466	0.3540218	0.3821818	0.3178241	0.4205926
ENSG00000272489	0.0000000	0.0000000	0.0000000	0.0012986	0.0000000
ENSG00000272447	0.0651452	0.0689901	0.0547101	0.0362582	0.0678939
NUTM2E	0.0227641	0.0407206	0.0432930	0.0058814	0.0367869
ENSG00000283913	0.0100443	0.0114738	0.0160620	0.0000000	0.0475367
SFTPD	0.0095473	0.0177545	0.0155784	0.0127165	0.0116422
SFTPD-AS1	0.0000000	0.0018728	0.0007063	0.0043276	0.0066309
TMEM254-AS1	0.0310586	0.0479125	0.0174193	0.0303531	0.0351807
TMEM254	0.2085259	0.1612392	0.1432413	0.2732230	0.1562109
PLAC9	0.0605307	0.0503818	0.0257579	0.0413363	0.0216529
ANXA11	0.2768894	0.1565546	0.1190752	0.3325620	0.1630817
MAT1A	0.0014215	0.0000000	0.0000000	0.0000000	0.0000000
DYDC1	0.0011585	0.0000000	0.0000000	0.0021172	0.0000000
DYDC2	0.1328182	0.0853814	0.0839779	0.1629511	0.0731058
PRXL2A	1.1550340	1.1087468	1.0199934	1.3855983	0.9543689
TSPAN14	0.0948628	0.1104414	0.1437949	0.1086877	0.1569834
SH2D4B	0.0008902	0.0013426	0.0000000	0.0000000	0.0141288
ENSG00000287358	0.0185147	0.0126407	0.0187754	0.0205820	0.0020430
NRG3	0.5444570	0.7321467	0.5883012	0.5018938	2.0701001
ENSG00000285739	0.0000000	0.0000000	0.0000000	0.0000000	0.0000000
ENSG00000229458	0.0015194	0.0024208	0.0019906	0.0000000	0.0495498
NRG3-AS1	0.0000000	0.0000000	0.0000000	0.0000000	0.0000000
ENSG00000288918	0.0059297	0.0107008	0.0202547	0.0056878	0.0071092
ENSG00000271933	0.0000000	0.0000000	0.0000000	0.0000000	0.0000000
GHITM	1.1454747	1.0481361	1.1851650	1.0798515	0.9349020
CERNA2	0.0026623	0.0300799	0.0399612	0.0103891	0.0107452
CDHR1	0.0098376	0.0149537	0.0105164	0.0073833	0.0168529
LINC00858	0.0021203	0.0097634	0.0000000	0.0000000	0.0000000
CCSER2	0.6192275	0.7810883	0.8161895	0.6141435	0.9016883
GRID1-AS1	0.0004809	0.0018046	0.0000000	0.0047296	0.0037464

GRID1	0.1080988	0.1487699	0.1235520	0.1126388	0.8359389
ENSG00000287475	0.0013015	0.0050275	0.0113450	0.0017814	0.0014252
WAPL	0.4452515	0.4205796	0.3836594	0.4719633	0.4379557
WAPL-DT	0.0139157	0.0079630	0.0110899	0.0076095	0.0127592
OPN4	0.0000000	0.0000000	0.0000000	0.0000000	0.0000000
LDB3	0.0027231	0.0027638	0.0035944	0.0026732	0.0000000
ENSG00000272631	0.0088897	0.0047711	0.0077104	0.0000000	0.0067862
BMPR1A	0.2888693	0.2366235	0.1911019	0.2845432	0.5286793
MMRN2	0.0047432	0.0017271	0.0103610	0.0049952	0.0250634
SNCG	1.1781354	1.7999226	1.8916310	1.1049975	1.3715577
ADIRF-AS1	0.0066899	0.0068099	0.0044118	0.0000000	0.0150693
ADIRF	0.0194888	0.0443032	0.0268293	0.0500259	0.0385214
ENSG00000229969	0.0000000	0.0000000	0.0000000	0.0000000	0.0000000
GLUD1	0.8586875	0.6624915	0.5539050	1.1543055	0.5981491
SHLD2	0.2080995	0.1830668	0.1362879	0.2481456	0.2613611
NUTM2A-AS1	0.3006377	0.3743234	0.3732226	0.2410442	0.4728380
ENSG00000287077	0.0042260	0.0048653	0.0068809	0.0000000	0.0156254
LINC00863	0.0750883	0.0779299	0.0952710	0.0528419	0.0944113
NUTM2D	0.0027545	0.0041224	0.0000000	0.0000000	0.0000000
ENSG00000286445	0.0109541	0.0113901	0.0075208	0.0159040	0.0113088
MINPP1	0.1671525	0.1435980	0.1239342	0.3264139	0.1602625
ENSG00000225913	0.0050536	0.0022127	0.0000000	0.0008779	0.0000000
ENSG00000196566	0.0150262	0.0080184	0.0049079	0.0154156	0.0048432
PAPSS2	0.4805358	0.2889407	0.1782204	0.2820812	0.2478618
ATAD1	0.5443295	0.6930763	0.8808405	0.5904717	0.7051840
CFL1P1	0.0737263	0.0730391	0.0672353	0.0802839	0.0457801
KLLN	0.0400555	0.0344489	0.0336242	0.0468988	0.0396729
PTEN	1.0122334	0.9779023	0.9810145	0.9765102	1.0388385
RNLS	0.0649438	0.0738792	0.0499919	0.0411361	0.1537866
ENSG00000289952	0.0223482	0.0394877	0.0266688	0.0267439	0.0232938
LIPJ	0.0000000	0.0014171	0.0000000	0.0000000	0.0000000
STAMBPL1	0.2001906	0.1525469	0.1059733	0.2269891	0.1351572
ACTA2-AS1	0.0015937	0.0008493	0.0000000	0.0038435	0.0053181
ACTA2	0.2782795	0.1483585	0.1056265	0.3435797	0.1582442
FAS	0.1119949	0.0728929	0.0556521	0.2606533	0.0529337
ENSG00000286116	0.0015275	0.0000000	0.0017780	0.0106517	0.0000000
CH25H	0.0105148	0.0115826	0.0000000	0.0293582	0.0033613
LIPA	0.2388596	0.1969923	0.2190604	0.3350621	0.3961152
ENSG00000232110	0.0016989	0.0026962	0.0038036	0.0000000	0.0000000
IFIT2	0.1065547	0.0670163	0.0205167	0.0598034	0.0487980
IFIT3	0.0432726	0.0311623	0.0128706	0.0390384	0.0592105
IFIT1B	0.0000000	0.0000000	0.0000000	0.0000000	0.0000000
IFIT1	0.1458927	0.0693015	0.0161298	0.1874808	0.1120092
IFIT5	0.3121976	0.3065253	0.3057866	0.3530632	0.2436946
SLC16A12	0.2238941	0.1086165	0.0605284	0.3057782	0.1279673
PANK1	0.0996523	0.1230867	0.1326201	0.0927975	0.1101381
PANK1-AS1	0.0087863	0.0020170	0.0049294	0.0095839	0.0000000
ENSG00000225836	0.0000000	0.0000000	0.0000000	0.0000000	0.0000000
ENSG00000235100	0.0281519	0.0350039	0.0300412	0.0166716	0.0747739
ENSG00000240996	0.0049505	0.0085073	0.0094366	0.0036873	0.0025298
KIF20B	0.0599674	0.0928743	0.0979128	0.0387837	0.0831726
LINC00865	0.0036466	0.0087081	0.0149320	0.0048144	0.0088772
LINC01374	0.0011850	0.0031510	0.0015516	0.0000000	0.0209232

LINC02653	0.0043448	0.0046231	0.0032084	0.0000000	0.0042647
ENSG00000224750	0.0015797	0.0000000	0.0000000	0.0000000	0.0061020
HTR7	0.0321008	0.0265907	0.0550185	0.0280400	0.0996501
RPP30	0.3036017	0.3189831	0.3482131	0.3182134	0.2230752
LINC00502	0.0000000	0.0000000	0.0000000	0.0000000	0.0016619
PCGF5	0.2460192	0.1907357	0.1776403	0.2305166	0.2343482
ENSG00000289228	0.0252032	0.0232218	0.0134813	0.0119194	0.0637436
HECTD2	0.2866950	0.3293293	0.3697356	0.2835839	0.3466756
PPP1R3C	0.6080553	0.3982341	0.2928795	0.4164794	0.4048983
TNKS2-DT	0.0131073	0.0105746	0.0094403	0.0283614	0.0183631
TNKS2	0.7131902	0.6075895	0.5678709	0.7038579	0.5930695
FGFBP3	0.1267660	0.0929473	0.0841040	0.2624753	0.0436191
ENSG00000272817	0.0053318	0.0014102	0.0013661	0.0000000	0.0000000
BTAF1	0.2826663	0.2354903	0.2292632	0.2549905	0.4701972
CPEB3	0.1550954	0.2511667	0.2402288	0.0900826	0.5394338
MARCHF5	0.3540300	0.3352276	0.3526744	0.3739315	0.3391862
IDE	0.2102340	0.1141876	0.0918926	0.2010910	0.1728953
KIF11	0.0343400	0.0315960	0.0169362	0.0239886	0.0776644
EXOC6	0.2365543	0.2699239	0.3108365	0.2433473	0.5701292
MYOF	0.2705024	0.1309870	0.0633461	0.3844962	0.1110627
CEP55	0.0083557	0.0056770	0.0053957	0.0000000	0.0060847
FFAR4	0.0017520	0.0036195	0.0052470	0.0000000	0.0055417
RBP4	0.0421488	0.0854218	0.0889493	0.0345938	0.0566871
PDE6C	0.0008671	0.0000000	0.0000000	0.0000000	0.0024211
FRA10AC1	0.6769262	0.5863421	0.5273307	0.6452547	0.5197897
LGI1	0.0227240	0.0568715	0.0479124	0.0186336	0.0425360
ENSG00000280660	0.0000000	0.0000000	0.0024882	0.0077032	0.0067831
SLC35G1	0.0983064	0.1450447	0.1868611	0.0595750	0.1242394
PLCE1	0.1009878	0.0672827	0.0568316	0.1554238	0.1729542
NOC3L	0.3742201	0.2532033	0.2185494	0.4024617	0.2068327
TBC1D12	0.1957567	0.2566062	0.2577125	0.1711332	0.4644791
HELLS	0.0625633	0.0890185	0.1059067	0.0667249	0.1418335
CYP2C18	0.0000000	0.0000000	0.0000000	0.0037324	0.0000000
CYP2C19	0.0000000	0.0000000	0.0008344	0.0000000	0.0000000
CYP2C8	0.0149047	0.0134050	0.0204949	0.0130778	0.0209724
ENSG00000231829	0.0118416	0.0069034	0.0119386	0.0067658	0.0216826
ENSG00000288526	0.0143491	0.0146162	0.0152674	0.0253579	0.0809924
ACSM6	0.0038977	0.0016318	0.0034282	0.0037517	0.0321751
ENSG00000234026	0.0027201	0.0018444	0.0121381	0.0018086	0.0050943
PDLIM1	0.1654749	0.1560003	0.0895117	0.1585702	0.1019968
SORBS1	0.1467619	0.1955435	0.2164117	0.1221775	0.6019785
ALDH18A1	0.1691862	0.1432196	0.1024146	0.1666746	0.2135523
TCTN3	0.1927129	0.1077215	0.0973586	0.3475172	0.1645684
ENTPD1	0.0121664	0.0125386	0.0152343	0.0000000	0.0441331
ENTPD1-AS1	0.1561950	0.2306415	0.1818061	0.1265844	0.3696833
CC2D2B	0.0134128	0.0207694	0.0221568	0.0195291	0.0555111
CCNJ	0.1084998	0.1499554	0.1105088	0.0909739	0.0946289
ENSG00000287009	0.0072483	0.0029561	0.0018564	0.0000000	0.0097133
ZNF518A	0.5059734	0.5442973	0.4121243	0.4405270	0.8876044
BLNK	0.0008080	0.0014068	0.0000000	0.0000000	0.0057154
TLL2	0.0082007	0.0076847	0.0053984	0.0114807	0.0061020
TM9SF3	1.0549119	0.8295012	0.7281048	1.2298650	0.8170729
PIK3AP1	0.0072382	0.0047431	0.0112727	0.0048922	0.0139055

LCOR	0.4621057	0.6197381	0.6719973	0.4297180	0.8563339
SLIT1	1.0144381	0.7240278	0.6506487	1.5248657	0.8777995
ARHGAP19	0.1206295	0.1151790	0.1170882	0.1132968	0.1190264
FRAT1	0.0257937	0.0488591	0.0768590	0.0532006	0.0228198
FRAT2	0.0604189	0.0674681	0.0647974	0.0642162	0.0615853
ENSG00000225850	0.0033388	0.0035081	0.0027009	0.0000000	0.0000000
RRP12	0.1165151	0.0999661	0.1049325	0.1340031	0.1167897
ENSG00000231970	0.0057969	0.0048227	0.0026158	0.0021832	0.0015171
PGAM1	1.0663668	1.2533325	1.4493034	1.0096775	0.9825318
EXOSC1	0.2445298	0.2324773	0.2553054	0.2130832	0.2365545
ZDHHC16	0.1429863	0.1492567	0.1534549	0.1742836	0.1356696
MMS19	0.1503689	0.1384225	0.1264997	0.1071770	0.2019560
UBTD1	0.1534777	0.0790084	0.0345989	0.1535835	0.0938229
ANKRD2	0.0000000	0.0014565	0.0009975	0.0000000	0.0000000
HOGA1	0.0179363	0.0108767	0.0047457	0.0240263	0.0000000
MORN4	0.3206130	0.3945325	0.4633970	0.3303123	0.2966982
PI4K2A	0.0498933	0.0851929	0.0938750	0.0659704	0.1083761
AVPI1	0.0897203	0.0944638	0.0995621	0.0846881	0.0558956
MARVELD1	0.0453039	0.0347287	0.0325974	0.0435404	0.0203760
ZFYVE27	0.0839799	0.1165340	0.0922357	0.0777823	0.2096070
SFRP5	0.0065523	0.0084498	0.0073162	0.0000000	0.0021288
GOLGA7B-DT	0.0000000	0.0009628	0.0000000	0.0000000	0.0000000
GOLGA7B	0.0675763	0.1412250	0.1529674	0.0475394	0.1278791
CRTAC1	0.0548441	0.1135807	0.1306995	0.0570985	0.1433569
R3HCC1L	0.1784683	0.2057314	0.1321510	0.2213032	0.2265773
LOXL4	0.0008864	0.0000000	0.0000000	0.0035068	0.0012770
ENSG00000230928	0.0012258	0.0000000	0.0000000	0.0000000	0.0075832
PYROXD2	0.0543542	0.0324074	0.0234677	0.0875070	0.0124314
HPS1	0.1576636	0.1170013	0.0474407	0.1615520	0.0791372
HPS1-AS1	0.0039321	0.0022136	0.0000000	0.0000000	0.0000000
HPSE2	0.0053537	0.0033308	0.0056771	0.0000000	0.0059953
CNNM1	0.0331180	0.0312068	0.0315116	0.0603335	0.1028733
GOT1	0.3733823	0.5144778	0.5183016	0.3317285	0.3692342
GOT1-DT	0.0167636	0.0193936	0.0246169	0.0168515	0.0203855
SLC25A28	0.1195331	0.1182281	0.1493624	0.1364419	0.1482303
SLC25A28-DT	0.0083759	0.0064841	0.0059571	0.0035916	0.0189243
ENSG00000229278	0.0241172	0.0193492	0.0159452	0.0175139	0.0322903
ENTPD7	0.0357891	0.0662278	0.0542401	0.0273093	0.0524356
CUTC	0.2682687	0.2240497	0.2208647	0.2703676	0.1494170
COX15	0.2095966	0.1775142	0.1911240	0.2622447	0.2585490
ABCC2	0.0036237	0.0014966	0.0031772	0.0017571	0.0013692
DNMBP	0.0784637	0.0575315	0.0250824	0.0647374	0.0719821
DNMBP-AS1	0.0000000	0.0006213	0.0000000	0.0039696	0.0000000
CPN1	0.0012012	0.0014383	0.0000000	0.0000000	0.0028456
ERLIN1	0.4200743	0.2482555	0.1695598	0.4686374	0.2456292
CHUK	0.1100866	0.1162656	0.0940263	0.1431755	0.1476212
CHUK-DT	0.0023119	0.0000000	0.0000000	0.0000000	0.0090864
CWF19L1	0.1711083	0.1439716	0.1449478	0.1515174	0.2182343
BLOC1S2	0.6847447	0.7785594	0.8232038	0.6745555	0.5194498
PKD2L1	0.0000000	0.0034883	0.0022583	0.0000000	0.0069935
ENSG00000231188	0.0004139	0.0000000	0.0000000	0.0000000	0.0069651
SCD	0.8307528	0.6890512	0.6633343	1.1195035	0.7606814
ENSG00000289301	0.0000000	0.0000000	0.0000000	0.0000000	0.0034843

OLMALINC	0.2607257	0.1691380	0.0844521	0.2577880	0.1758664
WNT8B	0.0025092	0.0000000	0.0000000	0.0000000	0.0000000
SEC31B	0.0161996	0.0223024	0.0178051	0.0094763	0.1102645
NDUFB8	1.0554341	1.2215686	1.3341069	1.0855100	0.9288924
HIF1AN	0.1272471	0.1300958	0.1585106	0.0862388	0.1620839
PAX2	0.0196505	0.0429972	0.0678426	0.0250139	0.0903235
ENSG00000272572	0.0483579	0.0375291	0.0571557	0.0571609	0.0276353
SLF2	0.4833456	0.3202327	0.3191279	0.4394742	0.4444665
ENSG00000273476	0.0000000	0.0009612	0.0000000	0.0000000	0.0000000
MRPL43	0.6426736	0.5359572	0.5952473	0.7103820	0.4147477
SEMA4G	0.0584765	0.0613488	0.0913435	0.0556118	0.0718456
TWNK	0.0494476	0.0469254	0.0537722	0.0332817	0.0692634
LZTS2	0.2240646	0.1443564	0.1621466	0.2482235	0.0912000
PDZD7	0.0386005	0.0616446	0.0803820	0.0152499	0.1432860
SFXN3	0.0988967	0.1337818	0.1445160	0.0796670	0.1251602
ENSG00000273162	0.0026839	0.0015363	0.0000000	0.0000000	0.0000000
KAZALD1	0.0040087	0.0068870	0.0016921	0.0023128	0.0073980
LBX1	0.0000000	0.0000000	0.0036390	0.0000000	0.0066462
LBX1-AS1	0.0024529	0.0034874	0.0056662	0.0000000	0.0254943
LINC02681	0.0000000	0.0008916	0.0000000	0.0000000	0.0000000
ENSG00000289441	0.0000000	0.0015056	0.0019820	0.0000000	0.0000000
BTRC	0.3800756	0.4289954	0.4624429	0.3994442	0.6337849
DPCD	0.7805173	0.6636375	0.6388695	0.7188137	0.5540165
POLL	0.1027480	0.0831841	0.0565657	0.0835697	0.1033351
FBXW4	0.1880569	0.1644135	0.1402013	0.1543075	0.3215068
ENSG00000286489	0.0038212	0.0023745	0.0025607	0.0020682	0.0027882
LINC03046	0.0000000	0.0000000	0.0000000	0.0000000	0.0022413
FGF8	0.0044423	0.0032545	0.0064084	0.0081122	0.0012783
NPM3	0.2411182	0.1931650	0.2154648	0.2897003	0.1538603
OGA	0.8660140	1.0373483	1.1106682	0.7446378	1.1262731
KCNIP2-AS1	0.0053745	0.0013418	0.0000000	0.0085697	0.0284655
KCNIP2	0.0368000	0.0893115	0.0535275	0.0203695	0.0812739
ARMH3	0.1439986	0.1107841	0.0751907	0.0998232	0.3100462
HPS6	0.0433000	0.0593122	0.0329142	0.0775836	0.0339999
LDB1	0.2291185	0.2623553	0.3004905	0.2429057	0.1933240
PPRC1	0.0453739	0.0440421	0.0350622	0.0210644	0.0665295
NOLC1	0.6282593	0.7397329	0.8745070	0.5791482	0.5857358
ELOVL3	0.0014107	0.0000000	0.0021947	0.0029495	0.0000000
GBF1	0.2474490	0.2344372	0.2509317	0.2352705	0.3908697
NFKB2	0.0482836	0.0151822	0.0143058	0.0189094	0.0260783
PSD	0.2952329	0.3944843	0.4671404	0.2775356	0.3468922
FBXL15	0.2531511	0.2567349	0.2445309	0.2308426	0.1558873
CUEDC2	0.5340069	0.5284522	0.5520271	0.5591457	0.3935847
C10orf95-AS1	0.1743272	0.2314355	0.2393770	0.1854799	0.1479755
C10orf95	0.0265755	0.0414555	0.0330186	0.0200497	0.0421627
MFSD13A	0.0348913	0.0302609	0.0642164	0.0347097	0.0517743
ACTR1A	0.4243769	0.5366013	0.6086283	0.4556478	0.4389050
ENSG00000273262	0.0000000	0.0023148	0.0000000	0.0000000	0.0080288
SUFU	0.0546598	0.0687549	0.0667269	0.0292562	0.1589156
TRIM8	0.4810650	0.5162355	0.6035371	0.5433021	0.3938163
TRIM8-DT	0.0058827	0.0072212	0.0114106	0.0031580	0.0168531
ARL3	1.3241559	1.2610611	1.3807582	1.2839572	1.0896139
SFXN2	0.0094597	0.0072209	0.0036242	0.0057855	0.0215160

WBP1L	0.2613301	0.1949041	0.1993334	0.2859596	0.3162229
ENSG00000282772	0.0108783	0.0013013	0.0101903	0.0158437	0.0107520
BORCS7	0.7310309	0.5523976	0.5684488	0.7703024	0.4689576
AS3MT	0.1341884	0.0912696	0.0833204	0.1506192	0.0918748
ENSG00000286575	0.0018316	0.0023489	0.0045044	0.0115737	0.0107176
ENSG00000272912	0.0019993	0.0000000	0.0000000	0.0000000	0.0000000
CNNM2	0.1322334	0.1331979	0.1276397	0.1064983	0.3103248
NT5C2	0.3054671	0.3346522	0.3740299	0.2871492	0.4478849
RPEL1	0.0000000	0.0000000	0.0000000	0.0000000	0.0000000
INA	0.9584801	1.7901140	2.3706196	0.8575000	1.4077567
PCGF6	0.0784724	0.0537081	0.0718068	0.1108092	0.1293161
TAF5	0.0633828	0.1063079	0.0931830	0.0734427	0.0912904
ATP5MK	1.4469874	1.5927498	1.5900664	1.4148281	1.2721612
PDCD11	0.1624592	0.1558991	0.1522463	0.1396139	0.2172690
CALHM2	0.0024691	0.0019474	0.0000000	0.0000000	0.0051799
ENSG00000273485	0.0025733	0.0027930	0.0000000	0.0078380	0.0077720
ENSG00000234699	0.0000000	0.0000000	0.0000000	0.0000000	0.0000000
NEURL1-AS1	0.0000000	0.0000000	0.0000000	0.0000000	0.0014454
NEURL1	0.0711927	0.1474708	0.1644909	0.0576792	0.1779045
ENSG00000287419	0.0024106	0.0034103	0.0023139	0.0039942	0.0085558
SH3PXD2A	0.2399448	0.2678555	0.2586560	0.2161195	0.5757470
ENSG00000273108	0.0098244	0.0123034	0.0241760	0.0000000	0.0064661
SH3PXD2A-AS1	0.0000000	0.0000000	0.0000000	0.0000000	0.0000000
STN1	0.1583841	0.1450019	0.1304530	0.0902594	0.0745917
ENSG00000289745	0.0113128	0.0221427	0.0084228	0.0043520	0.0169244
SLK	0.4075341	0.3708586	0.3258031	0.3050905	0.3168645
COL17A1	0.0000000	0.0000000	0.0000000	0.0000000	0.0000000
SFR1	0.1161804	0.0841658	0.0510056	0.1010760	0.0548533
CFAP43	0.4270636	0.3108012	0.1902237	0.4161872	0.4341190
GSTO1	0.5157150	0.4310253	0.5446860	0.4905206	0.3135709
GSTO2	0.0337580	0.0501584	0.0329325	0.0280560	0.0596366
ITPRIP	0.0222751	0.0108577	0.0116258	0.0385508	0.0277393
CFAP58-DT	0.0074580	0.0120750	0.0354250	0.0159247	0.0055777
CFAP58	0.0090708	0.0064236	0.0149381	0.0203747	0.0196392
SORCS3	0.1666279	0.2458965	0.3009426	0.1204880	1.0396638
SORCS1	0.1301521	0.1731372	0.0895876	0.2417492	0.3339241
ENSG00000287047	0.0000000	0.0000000	0.0000000	0.0000000	0.0000000
LINC01435	0.0057810	0.0037856	0.0000000	0.0000000	0.0160659
ENSG00000203434	0.0000000	0.0000000	0.0000000	0.0000000	0.0000000
LINC02661	0.0000000	0.0000000	0.0000000	0.0000000	0.0000000
XPNPEP1	0.2723537	0.2865485	0.2768476	0.2822865	0.2340360
ADD3-AS1	0.0242487	0.0235541	0.0149385	0.0394517	0.0167771
ADD3	0.7277943	0.5222330	0.3762111	0.6978393	0.5395736
MXI1	1.3844920	1.0438534	0.8670673	1.2419806	1.0113748
ENSG00000228417	0.0016698	0.0000000	0.0000000	0.0000000	0.0000000
SMNDC1	0.5166059	0.4957450	0.4995261	0.5381389	0.4700773
DUSP5-DT	0.0000000	0.0012869	0.0006832	0.0013262	0.0000000
DUSP5	0.0067306	0.0098844	0.0074748	0.0119572	0.0202005
SMC3	0.8141475	0.7812167	0.7766660	0.7368115	0.6600973
RBM20	0.0344874	0.0399755	0.0288837	0.0345716	0.0949285
PDCD4-AS1	0.0261499	0.0179270	0.0253296	0.0161422	0.0000000
PDCD4	0.7526238	0.5883950	0.4435377	0.6300735	0.6153979
BBIP1	0.3864957	0.4068609	0.3949699	0.4540835	0.3924507

ENSG00000278601	0.0024908	0.0014991	0.0028002	0.0000000	0.0000000
ENSG00000270589	0.0039798	0.0010716	0.0000000	0.0000000	0.0177557
SHOC2	0.8189081	0.9565688	1.0624917	0.6653869	0.8387801
ADRA2A	0.0406030	0.0913531	0.1871453	0.0251733	0.0721543
ENSG00000287231	0.0034585	0.0000000	0.0030415	0.0042330	0.0000000
GPAM	0.1216503	0.1250868	0.1766427	0.1264491	0.1282923
ACSL5	0.0000000	0.0000000	0.0000000	0.0000000	0.0111934
ENSG00000232934	0.0035414	0.0067172	0.0032416	0.0069975	0.0089782
ZDHHC6	0.1828036	0.1750309	0.1618099	0.2179545	0.1636316
VTI1A	0.2248762	0.2570171	0.2277910	0.1787164	0.5897536
ENSG00000233340	0.0000000	0.0000000	0.0000000	0.0000000	0.0000000
ENSG00000285676	0.0000000	0.0016055	0.0000000	0.0000000	0.0000000
ENSG00000260917	0.0312954	0.0306798	0.0260670	0.0311655	0.0222248
LINC02935	0.0052308	0.0041959	0.0055175	0.0000000	0.0390936
TCF7L2	0.4808220	0.2575123	0.1665050	0.5207347	0.3226420
ENSG00000233547	0.0536514	0.0322174	0.0080306	0.0471347	0.0336055
ENSG00000286289	0.0195725	0.0102932	0.0130211	0.0095344	0.0059454
HABP2	0.0000000	0.0010063	0.0000000	0.0000000	0.0000000
CASP7	0.0669368	0.0223165	0.0096695	0.0599739	0.0342328
DCLRE1A	0.1053396	0.0764570	0.0516786	0.1012061	0.0767334
NHLRC2	0.4772908	0.3320903	0.3093153	0.4906037	0.3477042
ADRB1	0.0133807	0.0025254	0.0058429	0.0048600	0.0021475
ENSG00000285540	0.0000000	0.0000000	0.0000000	0.0000000	0.0000000
CCDC186	0.5110669	0.6130441	0.7372014	0.3703003	0.4727218
TDRD1	0.0035795	0.0075602	0.0000000	0.0000000	0.0060804
VWA2	0.0000000	0.0022754	0.0006510	0.0000000	0.0000000
AFAP1L2	0.0355464	0.0655906	0.0803627	0.0217089	0.0740459
ABLIM1	0.4397438	0.6293649	0.6797304	0.3286390	0.8698997
ENSG00000228484	0.0000000	0.0000000	0.0000000	0.0000000	0.0065330
FHIP2A	0.2254816	0.2904000	0.2711833	0.1942801	0.3056332
TRUB1	0.2928479	0.3953668	0.5100824	0.3062139	0.3192012
ATRNL1	0.2623773	0.4034686	0.3437617	0.2149783	1.1191388
ENSG00000285582	0.0068280	0.0090678	0.0015325	0.0000000	0.0375725
GFRA1	0.2592804	0.3777685	0.2579774	0.2864187	0.5198152
ENSG00000285587	0.0160241	0.0200893	0.0135285	0.0037492	0.0128433
C10orf82	0.0399452	0.0778977	0.1662681	0.0405928	0.0469960
HSPA12A-AS1	0.0017471	0.0007374	0.0045174	0.0000000	0.0000000
HSPA12A	0.1647507	0.2718015	0.2278511	0.1792525	0.4638126
ENO4	0.2215590	0.1335875	0.0832970	0.2909711	0.1298033
SHTN1	0.7836565	1.2572706	1.4581648	0.8294522	1.1048727
ENSG00000287655	0.0000000	0.0024282	0.0022509	0.0000000	0.0000000
SLC18A2	0.0111865	0.0375574	0.0583029	0.0107071	0.0164309
ENSG00000277879	0.0049097	0.0013680	0.0088157	0.0059767	0.0014182
PDZD8	0.5847075	0.4784160	0.4546409	0.5271293	0.5463301
EMX2OS	0.0017137	0.0017751	0.0000000	0.0000000	0.0028494
RAB11FIP2	0.4571791	0.6559348	0.7263190	0.4204713	0.6109247
ENSG00000231104	0.0007765	0.0000000	0.0000000	0.0019814	0.0088375
CASC2	0.2078264	0.1727735	0.0908188	0.1497171	0.1917741
LINC02944	0.0000000	0.0000000	0.0000000	0.0000000	0.0040799
FAM204A	0.8049520	0.7735108	0.8713324	0.7753290	0.7500146
LINC00867	0.0010015	0.0049437	0.0000000	0.0000000	0.0099102
PRLHR	0.0217967	0.0237620	0.0250510	0.0127872	0.0234061
CACUL1	0.5868573	0.4719217	0.4430454	0.5679828	0.5357254

ENSG00000277687	0.0022359	0.0005546	0.0072448	0.0039003	0.0036228
LINC03036	0.0417975	0.0300791	0.0104312	0.0367843	0.0638883
NANOS1	0.9023704	0.6217480	0.5805541	0.8171522	0.4995743
EIF3A	1.1557285	1.0348508	0.9102047	1.1061105	0.9937685
ENSG00000289126	0.0016062	0.0000000	0.0000000	0.0039096	0.0043054
DENND10	0.4319490	0.3870508	0.3924020	0.5440865	0.3551647
SFXN4	0.2210359	0.1855489	0.1614717	0.2481399	0.1816848
PRDX3	0.7630313	0.7005936	0.8181324	0.8255360	0.5829699
GRK5	0.2327670	0.2141258	0.2309607	0.2299308	0.3626634
ENSG00000236426	0.0000000	0.0012897	0.0025124	0.0000000	0.0088184
RGS10	0.0121338	0.0086849	0.0149204	0.0039214	0.0031652
TIAL1	0.7654407	0.6533472	0.6734426	0.7178298	0.6182104
BAG3	0.1742276	0.0899227	0.0432755	0.1992003	0.0509657
INPP5F	0.5669771	0.8160754	1.0033314	0.5232777	0.8216135
MCMBP	0.2509321	0.2023080	0.2534486	0.2934419	0.2213503
SEC23IP	0.2515924	0.2104987	0.2053928	0.2867389	0.2320798
PLPP4	0.0404340	0.0759114	0.0767591	0.0415100	0.1202894
LINC01561	0.0015473	0.0068102	0.0036510	0.0000000	0.0056033
LINC02930	0.0041353	0.0086203	0.0218937	0.0036365	0.0451985
WDR11-DT	0.0486070	0.0503125	0.0370777	0.0318405	0.1596312
WDR11	0.2111320	0.1796023	0.1883445	0.2181594	0.2494932
ENSG00000227307	0.0000000	0.0000000	0.0000000	0.0000000	0.0000000
FGFR2	0.1894945	0.1053327	0.1057488	0.2180385	0.2095825
ATE1	0.4625773	0.4760477	0.4922275	0.3595714	0.5692506
ENSG00000276742	0.0049306	0.0059170	0.0023101	0.0000000	0.0114743
NSMCE4A	0.1807870	0.1882807	0.1843252	0.1821858	0.1251410
ENSG00000273891	0.0033099	0.0000000	0.0000000	0.0109791	0.0000000
TACC2	0.3274687	0.3847339	0.4194125	0.3038496	0.4914541
ENSG00000285973	0.0051832	0.0050881	0.0052005	0.0134208	0.0000000
BTBD16	0.0058150	0.0076791	0.0029980	0.0035940	0.0162267
PLEKHA1	0.3982029	0.5416941	0.5026941	0.3352348	0.3803619
ENSG00000285955	0.0006718	0.0000000	0.0055370	0.0042484	0.0030362
ARMS2	0.0007287	0.0000000	0.0013494	0.0000000	0.0000000
HTRA1	0.8977676	0.5458656	0.3437092	1.3307238	0.5040888
CUZD1	0.0000000	0.0048320	0.0016571	0.0000000	0.0000000
FAM24B	0.0134026	0.0458009	0.0443312	0.0146693	0.0267605
C10orf88	0.1734279	0.1891835	0.2139346	0.1405413	0.1918029
PSTK	0.1653528	0.1899991	0.2002993	0.1439228	0.2472514
IKZF5	0.1660990	0.1322313	0.0898148	0.1499308	0.2337813
ACADSB	0.1164405	0.1019649	0.1063626	0.1281443	0.1685868
HMX3	0.0010237	0.0036605	0.0084414	0.0000000	0.0000000
HMX2	0.0000000	0.0008294	0.0010104	0.0000000	0.0000000
BUB3	0.4122712	0.4623815	0.4803802	0.3976984	0.4289515
LINC02641	0.0119144	0.0062002	0.0065517	0.0064698	0.0412379
GPR26	0.0163963	0.0547835	0.0544835	0.0090821	0.0368318
CPXM2	0.0915694	0.0388547	0.0271047	0.2542989	0.0705515
ENSG00000288757	0.0005140	0.0000000	0.0000000	0.0000000	0.0000000
CHST15	0.0714392	0.0913239	0.0643468	0.1586305	0.1287962
OAT	0.5407269	0.5425733	0.5874072	0.5583513	0.4921346
LHPP	0.2002092	0.1568956	0.1614330	0.1868359	0.2144685
FAM53B	0.0687309	0.0607297	0.0495909	0.0566868	0.1655982
FAM53B-AS1	0.0010948	0.0000000	0.0000000	0.0000000	0.0107729
EEF1AKMT2	0.2682578	0.2629208	0.2697100	0.2252029	0.3151264

ABRAXAS2	0.2766446	0.2670773	0.2360263	0.2532038	0.2215680
ENSG00000289280	0.0000000	0.0009190	0.0027870	0.0000000	0.0017986
ENSG00000249456	0.0310573	0.0465946	0.0350687	0.0214340	0.0998957
ZRANB1	0.4486676	0.5029833	0.5671427	0.4328435	0.4821320
ENSG00000226899	0.0000000	0.0016015	0.0000000	0.0000000	0.0000000
CTBP2	0.5651419	0.5117976	0.5018473	0.5471080	0.8706502
ENSG00000273599	0.0021570	0.0000000	0.0000000	0.0051692	0.0066191
ENSG00000282787	0.0033033	0.0025367	0.0000000	0.0051171	0.0000000
ENSG00000228021	0.0004592	0.0011193	0.0028098	0.0046354	0.0022383
EDRF1-DT	0.0524505	0.0411747	0.0269247	0.0421144	0.0288151
ENSG00000234134	0.0010553	0.0018343	0.0024028	0.0000000	0.0000000
EDRF1	0.1846930	0.1826296	0.1709034	0.1948748	0.3364881
EDRF1-AS1	0.0000000	0.0000000	0.0000000	0.0000000	0.0140449
MMP21	0.0016244	0.0026567	0.0026358	0.0000000	0.0000000
UROS	0.4948634	0.4698560	0.4936389	0.4813741	0.3368763
BCCIP	0.8011126	0.7310479	0.7175751	0.8281949	0.5986861
DHX32	0.2954308	0.1953643	0.1951521	0.3558149	0.2966318
FANK1	0.5639064	0.3621771	0.2687595	0.5230180	0.4140719
ADAM12	0.0343512	0.0345385	0.0187244	0.0039916	0.1309314
LINC00601	0.0005361	0.0008577	0.0000000	0.0071728	0.0073473
C10orf90	0.0024231	0.0000000	0.0000000	0.0058194	0.0050105
DOCK1	0.3432674	0.2061672	0.1220354	0.3021865	0.2327987
ENSG00000223528	0.0017303	0.0014737	0.0042477	0.0000000	0.0000000
INSYN2A	0.2163713	0.2859032	0.3132669	0.2048991	0.3705025
PTPRE	0.0616346	0.1162308	0.1858592	0.0597842	0.2810866
ENSG00000232259	0.0005856	0.0000000	0.0000000	0.0016936	0.0000000
MKI67	0.0207487	0.0208545	0.0089390	0.0143571	0.0269598
LINC01163	0.0000000	0.0000000	0.0000000	0.0000000	0.0000000
MGMT	0.3114921	0.3027175	0.2443639	0.2963521	0.2445336
LINC02666	0.0086976	0.0073091	0.0068842	0.0104317	0.0498813
EBF3	0.0317078	0.0859072	0.0449412	0.0228223	0.2024632
ENSG00000275327	0.0011566	0.0000000	0.0000000	0.0000000	0.0000000
ENSG00000275005	0.0011465	0.0000000	0.0000000	0.0043765	0.0013244
EBF3-AS1	0.0000000	0.0000000	0.0000000	0.0000000	0.0000000
C10orf143	0.0231959	0.0202329	0.0104529	0.0099785	0.0043646
GLRX3	0.5005818	0.5247484	0.5677291	0.4714016	0.4771707
LINC02646	0.0087212	0.0038248	0.0097718	0.0065753	0.0475496
ENSG00000285529	0.0000000	0.0000000	0.0000000	0.0000000	0.0000000
TCERG1L	0.0474137	0.1058261	0.0459350	0.0447560	0.1298181
PPP2R2D	0.4799132	0.5362674	0.5796370	0.4225835	0.4998807
BNIP3	2.3681736	2.0753365	1.9432716	2.2287669	1.8513884
ENSG00000277959	0.0021604	0.0040848	0.0023861	0.0000000	0.0000000
ENSG00000273521	0.0024141	0.0016027	0.0000000	0.0000000	0.0000000
JAKMIP3	0.0496757	0.0798197	0.0481914	0.0350051	0.2862971
JAKMIP3-AS1	0.0000000	0.0031280	0.0056784	0.0000000	0.0083659
DPYSL4	0.1291592	0.1954016	0.2077789	0.1664646	0.1968345
STK32C	0.3222805	0.3730679	0.3737539	0.3241353	0.4001382
LRRC27	0.3845299	0.2881842	0.2308628	0.4309977	0.2954415
PWWP2B	0.1020906	0.1020931	0.0840909	0.1102168	0.0775222
ENSG00000231705	0.0038108	0.0000000	0.0000000	0.0041277	0.0114153
ENSG00000226900	0.0002628	0.0009268	0.0000000	0.0091496	0.0061813
INPP5A	0.2475629	0.2674838	0.2527124	0.2099871	0.4436365
NKX6-2	0.2191909	0.3010362	0.1774525	0.2280600	0.2780646

CFAP46	0.3017206	0.2057709	0.1673919	0.2757881	0.2965489
LINC01166	0.0023865	0.0132232	0.0083640	0.0045235	0.0000000
ADGRA1-AS1	0.0136644	0.0427555	0.0507423	0.0014048	0.0332422
ADGRA1	0.0334317	0.0545376	0.0761898	0.0249858	0.0707427
KNDC1	0.1148235	0.1387098	0.1435895	0.1251219	0.2910693
UTF1	0.0006121	0.0010736	0.0035575	0.0033589	0.0000000
ENSG00000273980	0.0007295	0.0000000	0.0000000	0.0000000	0.0000000
ADAM8	0.0049423	0.0070579	0.0035829	0.0060247	0.0155200
TUBGCP2	0.6464554	0.6138846	0.6756255	0.6457204	0.6352661
ZNF511	0.2288429	0.1814622	0.2216580	0.2719434	0.1423936
CALY	0.6249013	1.0001400	1.5096508	0.4677597	0.8723009
ENSG00000226699	0.0011153	0.0000000	0.0000000	0.0000000	0.0028743
FUOM	0.0473903	0.0327110	0.0204401	0.0496070	0.0365402
ECHS1	0.6620135	0.6475032	0.6606527	0.7417997	0.5310717
ENSG00000274685	0.0016116	0.0025913	0.0000000	0.0000000	0.0000000
PAOX	0.0559092	0.0314485	0.0273425	0.0534780	0.0752856
MTG1	0.2186277	0.1844834	0.2141166	0.2167932	0.2302000
SPRN	0.0428136	0.0827035	0.0895217	0.0590488	0.0542279
SCART1	0.0083952	0.0077311	0.0000000	0.0057490	0.0352132
CYP2E1	0.0034353	0.0031287	0.0052798	0.0062332	0.0131899
SYCE1	0.0033568	0.0000000	0.0000000	0.0000000	0.0000000
ENSG00000288107	0.0025985	0.0094555	0.0012539	0.0061123	0.0233232
ENSG00000254468	0.0013467	0.0000000	0.0000000	0.0000000	0.0000000
LINC01001	0.0014591	0.0030028	0.0009599	0.0000000	0.0000000
ENSG00000255229	0.0016449	0.0000000	0.0000000	0.0000000	0.0000000
BET1L	0.1230288	0.0992680	0.0906850	0.0952286	0.0883189
ENSG00000254559	0.0018185	0.0000000	0.0016476	0.0105790	0.0000000
RIC8A	0.2433673	0.2244982	0.2584609	0.2382901	0.1336064
SIRT3	0.1237230	0.1173374	0.0969922	0.1060937	0.1169965
PSMD13	0.4477828	0.4178442	0.3939761	0.4775516	0.3184639
PGGHG	0.0037627	0.0020856	0.0000000	0.0026482	0.0054431
IFITM2	0.0527953	0.0256941	0.0225519	0.0564957	0.0072044
IFITM1	0.0000000	0.0019719	0.0000000	0.0027049	0.0000000
ENSG00000251661	0.0494840	0.0348912	0.0251484	0.0848111	0.0451439
IFITM3	0.3446664	0.2234429	0.1381558	0.4409264	0.2129832
ENSG00000255328	0.0279692	0.0208620	0.0144310	0.0454714	0.0058498
B4GALNT4	0.1837670	0.2292520	0.1724541	0.1748023	0.3512523
SIGIRR	0.1444578	0.2307420	0.1139658	0.1484640	0.1340553
ANO9	0.0011383	0.0009808	0.0000000	0.0000000	0.0000000
PTDSS2	0.1401730	0.0965155	0.0908735	0.1848888	0.1462276
RNH1	0.5844497	0.5220344	0.5556184	0.6389673	0.4233671
ENSG00000288033	0.0026364	0.0000000	0.0032126	0.0000000	0.0262339
ENSG00000289997	0.0017241	0.0007209	0.0041747	0.0079805	0.0365901
HRAS	0.4983333	0.6462673	0.6522167	0.5049757	0.4607454
LRRC56	0.0136156	0.0107087	0.0081685	0.0209888	0.0148266
LMNTD2	0.0134777	0.0105341	0.0296483	0.0150363	0.0169245
LMNTD2-AS1	0.0045974	0.0113349	0.0000000	0.0059903	0.0094452
RASSF7	0.0947319	0.0555774	0.0619938	0.0753123	0.1019849
MIR210HG	0.2219789	0.1617338	0.1212300	0.1855265	0.1045058
PHRF1	0.0993800	0.0988016	0.1069524	0.0977598	0.2086789
IRF7	0.0273655	0.0103667	0.0114944	0.0279285	0.0171052
SCT	0.0212634	0.0169730	0.0035482	0.0119065	0.0056253
DRD4	0.0109359	0.0221620	0.0112445	0.0100203	0.0389492

DEAF1	0.6410458	0.7981334	0.9366121	0.6490159	0.6577374
ENSG00000255158	0.0066461	0.0059212	0.0000000	0.0000000	0.0146868
EPS8L2	0.0934124	0.1784811	0.3354221	0.0847651	0.1124723
TMEM80	0.1392064	0.0999115	0.0692934	0.2178783	0.0683460
ENSG00000269915	0.0022700	0.0016677	0.0000000	0.0000000	0.0000000
TALDO1	0.8339113	0.7355722	0.7469246	0.9441807	0.5350654
GATD1	0.2276262	0.1983837	0.2189635	0.2168072	0.1937622
GATD1-DT	0.2143457	0.1953199	0.2187455	0.1650278	0.1705080
CEND1	0.2202603	0.5157951	0.4664279	0.2513046	0.2802621
SLC25A22	0.1627881	0.2274712	0.2485145	0.1507263	0.1714572
PANO1	0.0266484	0.0197075	0.0265469	0.0396348	0.0435573
PIDD1	0.0382453	0.0252661	0.0319360	0.0620927	0.0596616
RPLP2	2.9233996	2.9362171	2.7699712	2.8292524	2.7326540
PNPLA2	0.1358234	0.0865136	0.0824248	0.1412860	0.1151864
ENSG00000255108	0.0008237	0.0000000	0.0033343	0.0000000	0.0129153
CRACR2B	0.0819258	0.0998157	0.1065658	0.0616622	0.0574751
CD151	0.6573645	0.4153453	0.3525003	0.8588138	0.3886902
POLR2L	1.0682858	0.9567115	0.9814634	1.1194834	0.7755995
TSPAN4	0.3867435	0.3430095	0.3126335	0.4752758	0.3165827
ENSG00000250397	0.0011795	0.0060291	0.0050256	0.0021965	0.0136130
CHID1	0.6708034	0.5621151	0.5283850	0.8284092	0.4882437
AP2A2	0.4163678	0.3619063	0.3633455	0.4082923	0.5381469
MUC6	0.0000000	0.0000000	0.0000000	0.0000000	0.0040206
LINC02688	0.0000000	0.0000000	0.0000000	0.0000000	0.0000000
MUC5AC	0.0013686	0.0019484	0.0032143	0.0016359	0.0000000
TOLLIP	0.2653139	0.2989193	0.3647428	0.2407112	0.2144250
TOLLIP-DT	0.0387074	0.0342120	0.0362646	0.0148524	0.0276294
ENSG00000287935	0.0043135	0.0009517	0.0016166	0.0000000	0.0048247
LINC02689	0.0041046	0.0043986	0.0021677	0.0000000	0.0000000
BRSK2	0.1709795	0.3373481	0.4250574	0.1776448	0.3208198
MOB2	0.1585429	0.1369101	0.1278113	0.1221453	0.1184860
DUSP8	0.2734239	0.5262413	0.4971857	0.2514956	0.3747793
KRTAP5-AS1	0.0434174	0.0799509	0.0715944	0.0279599	0.1753679
KRTAP5-2	0.0004250	0.0006229	0.0012131	0.0000000	0.0000000
IFITM10	0.0401712	0.0525672	0.0756954	0.0470541	0.0605442
CTSD	0.6234643	0.4563918	0.3549899	0.9219398	0.3491750
LSP1	0.0018703	0.0051733	0.0023036	0.0000000	0.0000000
TNNT3	0.0019017	0.0018192	0.0063093	0.0029555	0.0000000
MRPL23	0.1591694	0.1486991	0.1404669	0.1922661	0.1119666
MRPL23-AS1	0.0003297	0.0014397	0.0000000	0.0092731	0.0157773
H19	0.1558703	0.1197901	0.0773793	0.0979796	0.1450365
IGF2	0.1808945	0.0991355	0.0732120	0.1153308	0.1273876
IGF2-AS	0.0080702	0.0032286	0.0082271	0.0021816	0.0000000
INS	0.0033893	0.0035931	0.0000000	0.0000000	0.0157425
TH	0.0307062	0.1105836	0.0358381	0.0257278	0.0556399
ASCL2	0.0018448	0.0035555	0.0089895	0.0000000	0.0049696
TSPAN32	0.0007020	0.0015697	0.0036856	0.0000000	0.0000000
CD81-AS1	0.0332040	0.0225525	0.0272515	0.0190456	0.0330147
ENSG00000290075	0.0005428	0.0015136	0.0016818	0.0000000	0.0044485
ENSG00000290098	0.0015651	0.0019442	0.0000000	0.0000000	0.0043097
CD81	1.3578556	1.1629201	1.0428897	1.8306442	0.9686899
TSSC4	0.2536754	0.2105215	0.2136911	0.2287896	0.1253795
KCNQ1	0.0112571	0.0055200	0.0099302	0.0000000	0.0581627

KCNQ1OT1	0.3295723	0.3779385	0.3316434	0.2677073	1.1371815
KCNQ1-AS1	0.0015757	0.0065039	0.0130367	0.0000000	0.0000000
CDKN1C	0.7042767	0.8933500	0.8841894	0.5050986	0.6160891
SLC22A18AS	0.0058676	0.0021622	0.0014699	0.0000000	0.0119075
SLC22A18	0.0746091	0.0548386	0.0476380	0.1343463	0.0389835
PHLDA2	0.0166236	0.0451111	0.0573860	0.0143483	0.0370281
NAP1L4	1.2091967	1.2039039	1.3442728	1.2628529	1.1955096
CARS1	0.4484361	0.4873115	0.4939466	0.4653952	0.4619686
CARS1-AS1	0.0011162	0.0000000	0.0000000	0.0000000	0.0000000
ENSG00000285644	0.0202832	0.0139734	0.0219342	0.0153132	0.0132461
OSBPL5	0.0495838	0.0741289	0.0676056	0.0547617	0.2712030
ZNF195	0.2467793	0.2566295	0.2217767	0.2385889	0.2811014
ENSG00000290960	0.0284301	0.0705316	0.0318761	0.0135506	0.0931929
ENSG00000254757	0.0058334	0.0013594	0.0000000	0.0000000	0.0436856
ENSG00000255367	0.0156126	0.0024582	0.0119793	0.0053723	0.0289520
ART5	0.0025145	0.0014443	0.0038496	0.0043652	0.0000000
ART1	0.0000000	0.0000000	0.0000000	0.0000000	0.0000000
CHRNA10	0.0000000	0.0000000	0.0066002	0.0000000	0.0014454
NUP98	0.1940822	0.1623784	0.1204990	0.1679356	0.2737302
ENSG00000289468	0.0000000	0.0000000	0.0027572	0.0011568	0.0026919
PGAP2	0.2175035	0.1455655	0.1772365	0.2091794	0.1498408
RHOG	0.1112599	0.0681111	0.0380788	0.0816780	0.0365905
STIM1	0.1292926	0.0989181	0.1070885	0.1659730	0.2424732
RRM1	0.3601281	0.3334342	0.3326590	0.3967521	0.2828309
LINC02749	0.0148346	0.0049341	0.0055641	0.0154948	0.0000000
TRIM21	0.0327507	0.0367728	0.0202211	0.0624772	0.0272122
ENSG00000291144	0.0034746	0.0010339	0.0013013	0.0000000	0.0028666
TRIM68	0.0487305	0.0612685	0.0566360	0.0571137	0.0338094
OR51D1	0.0000000	0.0000000	0.0000000	0.0000000	0.0012623
MMP26	0.0112247	0.0107419	0.0053563	0.0049031	0.0149859
HBB	0.0000000	0.0000000	0.0111277	0.0000000	0.0000000
HBD	0.1742500	0.3674188	1.1974815	0.1632161	0.2803473
ENSG00000290652	0.0126560	0.0250497	0.0810005	0.0027655	0.0120304
UBQLNL	0.0013699	0.0000000	0.0021412	0.0000000	0.0000000
OR52H1	0.0013818	0.0000000	0.0000000	0.0000000	0.0000000
TRIM6	0.0125287	0.0015570	0.0072687	0.0018770	0.0000000
TRIM34	0.0152441	0.0090227	0.0000000	0.0037663	0.0058721
TRIM5	0.0995684	0.0423917	0.0349045	0.1230099	0.0338751
TRIM22	0.0419938	0.0200400	0.0048272	0.0919068	0.0529643
ENSG00000254444	0.0004239	0.0034350	0.0000000	0.0000000	0.0112003
ENSG00000267940	0.0046050	0.0038599	0.0049207	0.0027537	0.0099117
C11orf42	0.0009649	0.0004943	0.0031223	0.0000000	0.0068701
FHIP1B	0.0636967	0.0616210	0.0844817	0.0474994	0.0802950
CNGA4	0.0069541	0.0052214	0.0065145	0.0036229	0.0065870
CCKBR	0.0138481	0.0153718	0.0196098	0.0063831	0.0200632
CAVIN3	0.0555893	0.0285768	0.0227707	0.0330954	0.0417887
ENSG00000282556	0.0004410	0.0007712	0.0000000	0.0000000	0.0000000
SMPD1	0.2274675	0.1392699	0.1007209	0.4010579	0.0821902
APBB1	0.3296606	0.4028300	0.4681181	0.3144863	0.3740343
HPX	0.0057681	0.0063900	0.0081794	0.0161289	0.0087221
TRIM3	0.0594088	0.1080666	0.1313559	0.0734852	0.1023080
ARFIP2	0.1867834	0.1439328	0.1696705	0.1952909	0.1533209
TIMM10B	0.1440721	0.1377120	0.1372796	0.1430814	0.1479255

DNHD1	0.0354683	0.0458228	0.0243699	0.0372291	0.1001489
RRP8	0.1915064	0.1896345	0.2036338	0.1756312	0.1695285
ILK	0.3456591	0.2896861	0.2118866	0.3720338	0.2935923
TAF10	0.9131765	0.8032000	0.7706599	0.8999516	0.6909372
TPP1	0.1717690	0.0698647	0.0618714	0.3362017	0.0827019
DCHS1	0.0752532	0.0716756	0.0850080	0.1174464	0.1127130
DCHS1-AS1	0.0010767	0.0010823	0.0021614	0.0000000	0.0000000
ENSG00000255410	0.0055369	0.0074290	0.0076887	0.0031366	0.0203542
MRPL17	0.5834387	0.4908907	0.4701401	0.4956702	0.3700182
ENSG00000291143	0.0012022	0.0000000	0.0010545	0.0000000	0.0084373
OR2AG2	0.0007897	0.0008314	0.0000000	0.0000000	0.0000000
OR6A2	0.0000000	0.0008294	0.0047748	0.0000000	0.0000000
ENSG00000283415	0.0054558	0.0074498	0.0122396	0.0037038	0.1145586
ZNF215	0.0096140	0.0219434	0.0254862	0.0035633	0.0303702
ZNF214	0.0935499	0.0707378	0.0623231	0.0913987	0.0544573
NLRP14	0.0084736	0.0061555	0.0014327	0.0000000	0.0000000
SYT9	0.0957858	0.0953150	0.1104833	0.0877131	0.2609823
SYT9-AS1	0.0227563	0.0132647	0.0151062	0.0154957	0.0189421
OLFML1	0.0023038	0.0000000	0.0000000	0.0000000	0.0108780
PPFIBP2	0.0425887	0.0352796	0.0134586	0.0279825	0.0365175
CYB5R2	0.0535328	0.0128592	0.0203195	0.0258340	0.0348808
ENSG00000254951	0.0000000	0.0011690	0.0000000	0.0000000	0.0000000
NLRP10	0.0000000	0.0000000	0.0000000	0.0000000	0.0000000
EIF3F	1.4342640	1.4396360	1.3755785	1.3922180	1.1778745
TUB	0.2320692	0.2663047	0.3793577	0.2477523	0.2920198
ENSG00000254921	0.0000000	0.0020332	0.0029513	0.0019003	0.0035405
RIC3	0.1466313	0.1905510	0.2346135	0.1779622	0.3585974
RIC3-DT	0.0021962	0.0030694	0.0012333	0.0000000	0.0039740
LMO1	0.2494908	0.4217014	0.2477713	0.2344232	0.3020876
STK33	0.9164936	0.6621312	0.5391889	0.7956700	0.7175836
TRIM66	0.0728207	0.1187988	0.1288269	0.0387994	0.2512680
RPL27A	2.7304391	2.6145809	2.4484022	2.6051411	2.3773621
DENND2B	0.1861100	0.1024687	0.0767245	0.1590664	0.2165374
ENSG00000254665	0.0029522	0.0008902	0.0000000	0.0080806	0.0000000
DENND2B-AS1	0.0027716	0.0012444	0.0000000	0.0061851	0.0042185
AKIP1	0.2664944	0.2746594	0.3404728	0.2439405	0.1976689
C11orf16	0.0011761	0.0000000	0.0000000	0.0000000	0.0013712
ASCL3	0.0000000	0.0000000	0.0000000	0.0000000	0.0000000
TMEM9B	0.5871632	0.4312650	0.4569475	0.7204582	0.4235740
TMEM9B-AS1	0.0356294	0.0426478	0.0421082	0.0320135	0.0859144
NRIP3	0.0698564	0.1163876	0.1567113	0.0639306	0.1276067
NRIP3-DT	0.0137799	0.0111328	0.0413553	0.0159147	0.0308272
SCUBE2	0.0209512	0.0281501	0.0338247	0.0218110	0.0229185
DENND5A	0.3026922	0.2425799	0.1648201	0.3242811	0.4791985
ENSG00000255097	0.0003318	0.0000000	0.0009597	0.0000000	0.0000000
TMEM41B	0.3348166	0.2662329	0.2965959	0.3901887	0.3903908
IPO7	0.8371266	0.8243218	0.7475586	0.7435455	0.7548876
ENSG00000254397	0.0000000	0.0000000	0.0000000	0.0000000	0.0000000
ENSG00000268403	0.0082014	0.0092108	0.0108980	0.0022040	0.0059658
ZNF143	0.1616790	0.1668929	0.1446138	0.1634305	0.1915597
WEE1	0.1749515	0.1274399	0.0793212	0.1598902	0.0627115
SWAP70	0.1200351	0.1223210	0.0604165	0.1082078	0.1176574
LINC02709	0.0013558	0.0000000	0.0025575	0.0000000	0.0076551

SBF2-AS1	0.0645987	0.0687344	0.0690103	0.0763020	0.0666875
SBF2	0.5148798	0.5021805	0.4506400	0.4075984	1.1265875
ENSG00000255476	0.0070951	0.0038322	0.0065713	0.0027497	0.0297090
ENSG00000286561	0.0006639	0.0012869	0.0000000	0.0000000	0.0024068
ENSG00000254865	0.0000000	0.0012471	0.0000000	0.0000000	0.0095052
ADM	0.0932275	0.0683107	0.0443396	0.1254220	0.0387308
AMPD3	0.0780128	0.0526713	0.0322689	0.0673722	0.0769431
RNF141	0.3783863	0.3003613	0.2562450	0.4160769	0.2524669
IRAG1-AS1	0.0108401	0.0160514	0.0194557	0.0065211	0.0074671
LYVE1	0.0000000	0.0012616	0.0000000	0.0000000	0.0000000
IRAG1	0.0068492	0.0069258	0.0262512	0.0106744	0.0353740
CTR9	0.4066561	0.3561306	0.3269281	0.3835956	0.2580603
EIF4G2	2.2552490	2.4138184	2.6817302	2.1677135	2.0561213
ENSG00000246308	0.0080479	0.0041476	0.0089647	0.0000000	0.0119069
ZBED5	0.4304808	0.4078727	0.3770300	0.4458635	0.3952368
ZBED5-AS1	0.0885954	0.0944474	0.0750006	0.0845587	0.0732207
LINC02752	0.0006301	0.0021300	0.0000000	0.0028028	0.0072113
ENSG00000255260	0.0000000	0.0000000	0.0000000	0.0000000	0.0000000
GALNT18	0.2937434	0.1797349	0.1514752	0.3436108	0.3896306
CSNK2A3	0.0087506	0.0082384	0.0102492	0.0049732	0.0000000
ENSG00000255351	0.0000000	0.0016797	0.0018710	0.0000000	0.0125619
ENSG00000286959	0.0019727	0.0043135	0.0000000	0.0000000	0.0000000
USP47	0.6842134	0.6348840	0.6805928	0.6615872	0.8615797
DKK3	1.4670651	1.2513143	1.2595040	1.8472657	1.1493944
LINC02547	0.0000000	0.0000000	0.0000000	0.0000000	0.0000000
MICAL2	0.0546931	0.0475836	0.0389716	0.0306777	0.0565628
PARVA	0.1944780	0.1678251	0.1234770	0.2003234	0.1936874
ENSG00000286521	0.0000000	0.0000000	0.0000000	0.0000000	0.0000000
TEAD1	0.7882000	0.5715273	0.5402866	0.7290924	0.6765052
LINC00958	0.0144171	0.0083852	0.0000000	0.0100514	0.0044278
RASSF10-DT	0.0100559	0.0030679	0.0027426	0.0031190	0.0000000
RASSF10	0.0253082	0.0121108	0.0055651	0.0240532	0.0096421
ENSG00000290083	0.0018617	0.0000000	0.0000000	0.0090399	0.0000000
BMAL1	0.1557080	0.0945972	0.0572125	0.1526353	0.0987480
BTBD10	0.5335987	0.6471407	0.8635155	0.5531515	0.7314589
ENSG00000287548	0.0000000	0.0010476	0.0011221	0.0000000	0.0054919
PTH	0.0041064	0.0000000	0.0000000	0.0000000	0.0000000
FAR1	0.3531879	0.3789686	0.3625409	0.3178171	0.6245957
FAR1-IT1	0.0009026	0.0000000	0.0000000	0.0000000	0.0028456
LINC02683	0.0046941	0.0053930	0.0000000	0.0027407	0.0000000
SPON1	1.8023304	1.1542763	0.8245145	2.5232846	1.2435040
SPON1-AS1	0.0047811	0.0128644	0.0018505	0.0113235	0.0232232
RRAS2	0.3366091	0.3070478	0.3284822	0.3494444	0.3998480
COPB1	0.7653325	0.6482126	0.5974760	0.8363600	0.6770397
PSMA1	1.0492018	1.0128075	0.9292731	1.0911799	0.9061413
PDE3B	0.1003454	0.1191147	0.0893514	0.0813324	0.5095073
CYP2R1	0.0787206	0.1554006	0.1798962	0.1117003	0.1447755
CALCB	0.1080810	0.2559910	0.0868107	0.1182494	0.1999310
CALCA	0.0445916	0.0828269	0.0330172	0.0536918	0.0888654
INSC	0.0006686	0.0027474	0.0000000	0.0000000	0.0035970
ENSG00000254695	0.0000000	0.0000000	0.0000000	0.0027814	0.0000000
SOX6	0.3260222	0.1810219	0.1128604	0.3251140	0.3402363
ENSG00000254878	0.0011240	0.0015056	0.0000000	0.0000000	0.0000000

C11orf58	1.7217746	1.4992512	1.6303917	1.6205544	1.3001176
PLEKHA7	0.1673721	0.1501066	0.1157508	0.1918917	0.2137181
ENSG00000290957	0.0256409	0.0188001	0.0186207	0.0115762	0.0127932
RPS13	2.7032643	2.7100332	2.5836965	2.6003867	2.4701029
PIK3C2A	0.3432362	0.2763290	0.1762441	0.3827827	0.4392197
NUCB2	1.1419032	0.9506061	0.9349699	1.3489077	0.8279813
ENSG00000285545	0.0000000	0.0010722	0.0000000	0.0000000	0.0092211
NCR3LG1	0.0677389	0.1106062	0.1439732	0.0609177	0.0883783
KCNJ11	0.0057662	0.0146150	0.0312211	0.0219278	0.0417170
ENSG00000260196	0.0205580	0.0274744	0.0196216	0.0130060	0.0223368
ABCC8	0.0247012	0.0222635	0.0118255	0.0293128	0.0844262
ENSG00000287898	0.0016467	0.0114375	0.0069451	0.0000000	0.0000000
USH1C	0.0041090	0.0013112	0.0046676	0.0000000	0.0065348
OTOG	0.0000000	0.0000000	0.0000000	0.0000000	0.0000000
KCNC1	0.0684993	0.1001857	0.1620484	0.0438209	0.1992459
SERGEF	0.1946318	0.2621502	0.2701873	0.2139605	0.4655490
TPH1	0.0285140	0.0056326	0.0076635	0.0059678	0.0118648
SAAL1	0.0589325	0.0994897	0.0895632	0.0651647	0.1057125
SAA4	0.0000000	0.0000000	0.0000000	0.0000000	0.0000000
SAA2	0.0000000	0.0000000	0.0021138	0.0000000	0.0000000
SAA1	0.0000000	0.0000000	0.0000000	0.0000000	0.0000000
HPS5	0.1757690	0.1650454	0.1393100	0.1917137	0.1841177
GTF2H1	0.1719370	0.1539081	0.1309964	0.2150702	0.2554310
LDHA	1.9069216	1.9893392	1.9644596	1.7847181	1.5906489
LDHC	0.0081689	0.0020561	0.0000000	0.0000000	0.0108293
LDHAL6A	0.0000000	0.0000000	0.0009874	0.0031460	0.0138652
TSG101	0.6950643	0.6576476	0.7479851	0.6138805	0.5697778
ENSG00000289499	0.0010865	0.0014455	0.0000000	0.0000000	0.0000000
UEVLD	0.1502962	0.1010524	0.0609169	0.1705944	0.1268304
MISFA	0.0292788	0.0281494	0.0229576	0.0161210	0.0201320
SPTY2D1	0.3231412	0.2020886	0.1878362	0.2519248	0.2388302
TMEM86A	0.0041898	0.0036507	0.0043676	0.0000000	0.0205312
IGSF22	0.0092276	0.0422841	0.0361481	0.0041721	0.0338962
IGSF22-AS1	0.0355693	0.0515712	0.0807575	0.0163383	0.0920073
PTPN5	0.1250929	0.1885372	0.3100200	0.0771547	0.2066819
ZDHHC13	0.1065260	0.1338249	0.1750495	0.1332835	0.1915810
CSRP3	0.0005727	0.0000000	0.0000000	0.0000000	0.0057461
CSRP3-AS1	0.0000000	0.0031658	0.0046624	0.0000000	0.0089814
E2F8	0.0011410	0.0000000	0.0044672	0.0065441	0.0039666
NAV2	0.4913799	0.4985723	0.4130312	0.4568212	1.3625031
NAV2-IT1	0.0021660	0.0000000	0.0000000	0.0000000	0.0013712
NAV2-AS5	0.0000000	0.0000000	0.0000000	0.0000000	0.0025988
NAV2-AS4	0.0010461	0.0000000	0.0000000	0.0000000	0.0041410
NAV2-AS6	0.0097734	0.0327745	0.0335327	0.0058681	0.0208827
NAV2-AS3	0.0000000	0.0000000	0.0000000	0.0000000	0.0070777
NAV2-AS2	0.0000000	0.0000000	0.0000000	0.0000000	0.0000000
NAV2-AS1	0.0049131	0.0041742	0.0016198	0.0033923	0.0000000
HTATIP2	0.0394431	0.0484269	0.0179060	0.0187797	0.0238545
PRMT3	0.1801041	0.1280462	0.0979919	0.2156425	0.2997062
ENSG00000286382	0.0000000	0.0008078	0.0000000	0.0000000	0.0000000
SLC6A5	0.0060333	0.0112682	0.0132351	0.0020088	0.0249794
NELL1	0.2928945	0.3731075	0.4562017	0.2157973	1.0087352
ENSG00000254906	0.0021189	0.0028434	0.0035433	0.0000000	0.0000000

ENSG00000255167	0.0000000	0.0000000	0.0000000	0.0000000	0.0038734
ENSG00000289944	0.0000000	0.0007561	0.0000000	0.0000000	0.0000000
ANO5	0.1684539	0.2726695	0.2655572	0.1433829	0.5116758
ENSG00000287962	0.0043993	0.0008967	0.0006919	0.0028850	0.0000000
ENSG00000255372	0.0000000	0.0000000	0.0052295	0.0000000	0.0000000
SLC17A6-DT	0.0000000	0.0008866	0.0058701	0.0000000	0.0071183
SLC17A6	0.0358570	0.0558546	0.1270753	0.0287025	0.1292628
LINC01495	0.0000000	0.0000000	0.0000000	0.0000000	0.0183073
FANCF	0.2268138	0.1612274	0.2158917	0.2221683	0.1892117
GAS2	0.0494217	0.0510030	0.0446475	0.0353648	0.1540892
SVIP	0.5412445	0.4095324	0.3089602	0.5304549	0.3136570
ENSG00000246225	0.0046309	0.0037735	0.0073388	0.0037157	0.0158700
LINC02718	0.0000000	0.0000000	0.0022489	0.0000000	0.0000000
LUZP2	0.2189531	0.3596985	0.3882253	0.1754054	0.8259177
LINC02699	0.0032081	0.0019333	0.0000000	0.0000000	0.0000000
ANO3	0.0094683	0.0139328	0.0086588	0.0080632	0.0100491
ANO3-AS1	0.0000000	0.0000000	0.0000000	0.0000000	0.0000000
MUC15	0.0296264	0.0130204	0.0024822	0.0112010	0.0412880
SLC5A12	0.0061394	0.0122134	0.0019906	0.0000000	0.0032018
FIBIN	0.4528608	0.2564415	0.1174867	0.7691902	0.2280303
BBOX1	0.1520543	0.0936605	0.0572009	0.2223629	0.0893303
BBOX1-AS1	0.0349262	0.0280807	0.0178605	0.0472914	0.0700570
CCDC34	0.7988710	0.5945496	0.5800554	0.7300384	0.6279967
LGR4	0.2657358	0.1945258	0.1346267	0.3540124	0.3490318
LGR4-AS1	0.0064654	0.0011666	0.0021247	0.0044887	0.0023002
LIN7C	0.7003387	0.9784847	1.1630113	0.6626790	0.7867662
BDNF-AS	0.1903237	0.1582745	0.1486727	0.2076583	0.1814846
BDNF	0.0088620	0.0226438	0.0277170	0.0063601	0.0800633
ENSG00000255496	0.0009485	0.0000000	0.0000000	0.0000000	0.0000000
ENSG00000255094	0.0000000	0.0000000	0.0013260	0.0000000	0.0000000
KIF18A	0.0117749	0.0118086	0.0183436	0.0191563	0.0200124
METTL15	0.2436289	0.2176802	0.2152134	0.1828044	0.4315626
LINC02742	0.0000000	0.0013977	0.0000000	0.0000000	0.0000000
ENSG00000254526	0.0024870	0.0000000	0.0015064	0.0039481	0.0000000
LINC02755	0.0155100	0.0290252	0.0226383	0.0128457	0.0696614
ENSG00000254734	0.0000000	0.0000000	0.0032963	0.0000000	0.0133315
LINC01616	0.0000000	0.0007736	0.0000000	0.0000000	0.0000000
KCNA4	0.0156969	0.0323215	0.0285160	0.0250163	0.0266138
ARL14EP-DT	0.0239462	0.0198171	0.0017347	0.0060100	0.1018225
ARL14EP	0.4520987	0.4091529	0.4253370	0.4334281	0.2677220
MPPED2	0.2885003	0.4292523	0.6023454	0.2705767	0.7637772
ENSG00000255480	0.0000000	0.0000000	0.0000000	0.0000000	0.0000000
MPPED2-AS1	0.0069946	0.0044952	0.0048631	0.0029052	0.0289734
ENSG00000287373	0.0000000	0.0009994	0.0000000	0.0000000	0.0000000
DCDC1	0.2092033	0.1294227	0.0680826	0.1349934	0.1974590
DNAJC24	0.2417288	0.2407159	0.1925155	0.2686614	0.2554493
IMMP1L	0.4633321	0.4952356	0.4765121	0.4024834	0.5005438
ELP4	0.2680107	0.2356246	0.2321901	0.2536208	0.4527514
ENSG00000228061	0.0010355	0.0023451	0.0000000	0.0000000	0.0293386
PAX6	0.0155043	0.0351364	0.0206076	0.0022475	0.0166241
PAUPAR	0.0004183	0.0000000	0.0000000	0.0000000	0.0000000
RCN1	0.9966872	0.7606831	0.6032983	1.2914590	0.6240066
ENSG00000255252	0.0000000	0.0024043	0.0000000	0.0013375	0.0052658

WT1-AS	0.0000000	0.0021486	0.0000000	0.0000000	0.0000000
EIF3M	0.9811796	0.9494430	0.9546671	0.9846103	0.8237480
CCDC73	0.0019600	0.0014147	0.0022230	0.0037324	0.0186884
PRRG4	0.0011029	0.0174167	0.0066736	0.0000000	0.0000000
QSER1	0.5375874	0.5138640	0.5547695	0.4429601	0.6214711
DEPDC7	0.0156698	0.0116557	0.0078442	0.0167063	0.0110042
TCP11L1	0.1696940	0.2092746	0.2144089	0.1504597	0.3031904
LINC00294	0.0342942	0.0495608	0.0379137	0.0220373	0.0423170
CSTF3	0.4880645	0.5069908	0.4406789	0.4487881	0.5671823
CSTF3-DT	0.0054817	0.0071035	0.0026613	0.0164905	0.0000000
HIPK3	0.4895899	0.5511176	0.4793236	0.4015924	0.6097674
KIAA1549L	0.1406899	0.2048838	0.2143404	0.0950872	0.5278276
ENSG00000255202	0.0005290	0.0028103	0.0057125	0.0000000	0.0000000
C11orf91	0.0179404	0.0069272	0.0258458	0.0089525	0.0039345
CD59	0.6303887	0.5371628	0.5247981	0.8038977	0.4877087
FBXO3	0.3286077	0.3182792	0.3096227	0.3029554	0.3027756
LMO2	0.0238174	0.0627489	0.0754796	0.0249265	0.0332033
ENSG00000286626	0.0045210	0.0118921	0.0100738	0.0010526	0.0093622
CAPRIN1	0.9564872	1.0035131	1.0381661	0.9460090	0.8585761
NAT10	0.1534455	0.1477959	0.1405559	0.1341064	0.1605164
ABTB2	0.0364208	0.0477883	0.0352280	0.0090595	0.1802034
CAT	0.3717258	0.2185082	0.1591735	0.3619971	0.2427692
ELF5	0.0090473	0.0024799	0.0006535	0.0234782	0.0000000
APIP	0.3971660	0.3907771	0.4053464	0.3969603	0.4378261
PDHX	0.3468380	0.4031591	0.4593642	0.3511779	0.4186941
ENSG00000289526	0.0000000	0.0000000	0.0000000	0.0000000	0.0000000
CD44	0.3661222	0.2173754	0.1431306	0.5533426	0.1629898
ENSG00000251194	0.0000000	0.0000000	0.0000000	0.0000000	0.0000000
SLC1A2	0.1288882	0.2109718	0.3979478	0.0884779	0.4947117
ENSG00000255542	0.0007777	0.0026426	0.0030148	0.0000000	0.0000000
PAMR1	0.0210493	0.0230691	0.0088622	0.0558632	0.0938813
ENSG00000254669	0.0000000	0.0007100	0.0000000	0.0000000	0.0000000
FJX1	0.8343265	0.6261704	0.4089182	1.2185670	0.4744892
TRIM44	0.7878153	0.8693727	0.9036190	0.7215038	0.9641789
ENSG00000285705	0.0000000	0.0010170	0.0000000	0.0000000	0.0000000
LDLRAD3	0.3878523	0.3073779	0.2053480	0.4433871	0.4394209
ENSG00000255256	0.0000000	0.0000000	0.0000000	0.0000000	0.0000000
COMMD9	0.3217440	0.3229366	0.3278436	0.3165089	0.2470082
PRR5L	0.0599224	0.0465382	0.0163224	0.0647904	0.0296600
TRAF6	0.1248054	0.1236368	0.0806507	0.0672498	0.1642715
RAG1	0.0041960	0.0058659	0.0009138	0.0000000	0.0037464
RAG2	0.0000000	0.0000000	0.0000000	0.0000000	0.0000000
IFTAP	0.4008031	0.4056197	0.4022873	0.3972720	0.3142484
LRRC4C	0.2179229	0.3546376	0.3088775	0.2080170	0.9592767
ENSG00000255132	0.0000000	0.0010918	0.0000000	0.0000000	0.0000000
LINC02745	0.0000000	0.0000000	0.0000000	0.0000000	0.0000000
LINC02740	0.0000000	0.0000000	0.0000000	0.0000000	0.0000000
API5	0.3717820	0.3565933	0.3658897	0.3633066	0.3458782
ENSG00000254907	0.0013590	0.0012831	0.0011788	0.0038033	0.0061857
TTC17	0.4340387	0.3943490	0.3540603	0.5033790	0.6820480
ENSG00000255340	0.0000000	0.0010152	0.0000000	0.0000000	0.0000000
ENSG00000254577	0.0000000	0.0005471	0.0000000	0.0000000	0.0049014
ENSG00000283217	0.0446131	0.0202484	0.0246364	0.0316091	0.0566235

MIR670HG	0.0020376	0.0000000	0.0000000	0.0000000	0.0000000
ENSG00000283341	0.0334016	0.0245096	0.0474956	0.0409053	0.0129913
HSD17B12	0.7563349	0.6288947	0.7676054	1.0228030	0.6086276
ENSG00000246250	0.0365948	0.0407170	0.0850861	0.0598652	0.0597341
ENSG00000283375	0.0000000	0.0010046	0.0000000	0.0000000	0.0059744
ALKBH3	0.1363836	0.1121091	0.1448814	0.1074119	0.1191499
ALKBH3-AS1	0.0004219	0.0000000	0.0000000	0.0000000	0.0000000
ENSG00000254409	0.0042634	0.0000000	0.0007146	0.0062070	0.0210607
C11orf96	0.0411427	0.0711880	0.0649301	0.0290626	0.0856855
ENSG00000244953	0.0000000	0.0011558	0.0011015	0.0000000	0.0044624
ACCS	0.0052495	0.0026644	0.0000000	0.0091201	0.0085254
EXT2	0.3779921	0.2852862	0.2927297	0.5503692	0.4141371
ALX4	0.0017514	0.0025789	0.0013677	0.0000000	0.0118277
CD82	0.0318772	0.0211682	0.0104813	0.0372272	0.0050279
TSPAN18-AS1	0.0000000	0.0017155	0.0000000	0.0000000	0.0000000
TSPAN18	0.1067479	0.2058257	0.1814232	0.1162100	0.4149725
TP53I11	0.1803181	0.2572872	0.3013231	0.1896581	0.2606312
PRDM11	0.0615762	0.1167653	0.1009873	0.0566266	0.1054867
ENSG00000254664	0.0000000	0.0000000	0.0032497	0.0000000	0.0000000
SYT13	0.2419785	0.4068101	0.8298922	0.2162660	0.4206949
LINC02696	0.1158310	0.0915009	0.1047477	0.0873191	0.0701865
LINC02687	0.0009466	0.0000000	0.0000000	0.0000000	0.0059207
ENSG00000255041	0.0000000	0.0017842	0.0000000	0.0000000	0.0000000
ENSG00000286418	0.0000000	0.0000000	0.0066869	0.0000000	0.0000000
ENSG00000254746	0.0005628	0.0000000	0.0000000	0.0000000	0.0000000
CHST1	0.0375835	0.0941747	0.1938403	0.0324717	0.1013525
ENSG00000254519	0.0000000	0.0000000	0.0000000	0.0000000	0.0000000
LINC02716	0.0040535	0.0075563	0.0059601	0.0000000	0.0000000
SLC35C1	0.0550689	0.0674967	0.0794438	0.0736293	0.0310867
ENSG00000255447	0.0000000	0.0037746	0.0000000	0.0000000	0.0037844
CRY2	0.1045693	0.1404611	0.1441661	0.0892332	0.1721349
MAPK8IP1	0.2556925	0.3681846	0.4451374	0.3167875	0.3325840
ENSG00000255498	0.0034093	0.0045883	0.0087698	0.0000000	0.0157023
PEX16	0.1345015	0.1200783	0.1232513	0.1042937	0.0596115
LARGE2	0.0011708	0.0000000	0.0000000	0.0000000	0.0000000
PHF21A	0.4668388	0.4745343	0.4099039	0.4703297	0.6135286
ENSG00000254653	0.0000000	0.0000000	0.0000000	0.0000000	0.0044857
ENSG00000255314	0.0024586	0.0007887	0.0000000	0.0000000	0.0000000
CREB3L1	0.0539776	0.0789322	0.0815413	0.0337982	0.1145792
DGKZ	0.2087115	0.2462797	0.2789984	0.2157327	0.2679922
MDK	1.5786961	1.3079472	1.1810905	1.9768870	1.1943516
CHRM4	0.0141243	0.0137234	0.0308995	0.0141343	0.0059869
AMBRA1	0.1655143	0.1697968	0.1284498	0.1494609	0.3958333
ENSG00000285658	0.0238930	0.0147225	0.0273885	0.0176363	0.0312308
HARBI1	0.0307751	0.0286809	0.0213384	0.0314044	0.0271100
ATG13	0.1644694	0.1828184	0.1275245	0.1957351	0.1322209
ARHGAP1	0.1923384	0.1688845	0.1326137	0.2125072	0.1803073
ZNF408	0.0349636	0.0314752	0.0330266	0.0481351	0.0553896
F2	0.0000000	0.0000000	0.0027720	0.0000000	0.0025607
CKAP5	0.6619525	0.6885691	0.8197654	0.6977267	0.7892089
LRP4-AS1	0.0055299	0.0085687	0.0199667	0.0092930	0.0105043
LRP4	0.0250253	0.0110590	0.0066479	0.0689393	0.0044490
CSTPP1	0.4181576	0.4091152	0.3740535	0.4076900	0.4867325

ENSG00000255520	0.0009515	0.0042033	0.0000000	0.0000000	0.0145620
ARFGAP2	0.2109909	0.2037463	0.2173752	0.2160536	0.2614835
PACSIN3	0.1009894	0.0545850	0.0314894	0.0419284	0.0598979
DDB2	0.0906826	0.0650178	0.0582053	0.0877450	0.0619203
ACP2	0.1260433	0.1176570	0.1093811	0.2290555	0.1017189
NR1H3	0.0563505	0.0683800	0.0729094	0.0512743	0.0738385
MADD	0.1183298	0.1710283	0.2246994	0.0906229	0.2258498
MADD-AS1	0.0000000	0.0000000	0.0000000	0.0000000	0.0000000
MYBPC3	0.0000000	0.0000000	0.0013800	0.0000000	0.0000000
SPI1	0.0009386	0.0000000	0.0037587	0.0000000	0.0023002
SLC39A13-AS1	0.0000000	0.0046558	0.0011917	0.0000000	0.0000000
SLC39A13	0.0784372	0.0703211	0.0770050	0.0930668	0.0859402
PSMC3	0.5685428	0.5392514	0.5475771	0.5999221	0.4197773
RAPSN	0.0008061	0.0000000	0.0000000	0.0000000	0.0062307
CELF1	0.5567424	0.5813658	0.5371261	0.4991146	0.9431804
ENSG00000270072	0.0010252	0.0017414	0.0000000	0.0000000	0.0035158
NDUFS3	0.4994924	0.5405471	0.6300785	0.5570646	0.3924646
PTPMT1	0.4272623	0.3962111	0.3563862	0.4805412	0.3461212
KBTBD4	0.1073261	0.1410535	0.0887387	0.0887790	0.0712703
C1QTNF4	0.2043609	0.3426669	0.5460826	0.1155890	0.2188010
MTCH2	0.6520262	0.7666550	0.8516975	0.6989548	0.5732124
AGBL2	0.0229933	0.0050264	0.0084499	0.0227914	0.0391561
FNBP4	0.4185465	0.3843246	0.3916750	0.3996193	0.6247328
NUP160	0.0962326	0.0854704	0.0842131	0.0878003	0.3068082
PTPRJ	0.1278024	0.1527201	0.1268456	0.0860107	0.4580781
FOLH1	0.0163466	0.0017916	0.0016147	0.0317681	0.0000000
GRM5P1	0.0003250	0.0000000	0.0032832	0.0024514	0.0035154
SEPTIN7P11	0.0024002	0.0000000	0.0000000	0.0019820	0.0000000
LINC02735	0.0000000	0.0011443	0.0019782	0.0011520	0.0014580
LRRC55	0.0162628	0.0474418	0.0380381	0.0088799	0.0258467
TNKS1BP1	0.0756763	0.0787609	0.0494253	0.0430711	0.0546226
ENSG00000254662	0.0000000	0.0004067	0.0012145	0.0000000	0.0105105
SSRP1	0.6366794	0.5947974	0.6494650	0.6016758	0.5265527
P2RX3	0.1117786	0.2103956	0.1214920	0.0528102	0.1496934
SLC43A3	0.0063670	0.0045951	0.0047120	0.0081794	0.0023981
RTN4RL2	0.0282197	0.0546408	0.0327256	0.0160468	0.0364640
SLC43A1	0.0045724	0.0010716	0.0000000	0.0000000	0.0000000
TIMM10	0.1777232	0.1923084	0.1840956	0.1625070	0.1454747
SMTNL1	0.0000000	0.0000000	0.0007043	0.0000000	0.0000000
UBE2L6	0.3349322	0.4849215	0.4909936	0.3505799	0.3482822
SERPING1	0.0354607	0.0498960	0.0296793	0.0563690	0.0280426
MIR130AHG	0.0210308	0.0111014	0.0239939	0.0172368	0.0205681
YPEL4	0.0529977	0.0867014	0.0524645	0.0450771	0.0405880
CLP1	0.0758784	0.0656893	0.0375152	0.0717071	0.0278402
ZDHHC5	0.1547352	0.1627544	0.1922919	0.1566342	0.2053240
MED19	0.3946627	0.4330628	0.4539218	0.4067070	0.2832274
TMX2	0.3472635	0.3494948	0.3517032	0.5609964	0.2970665
SELENOH	1.1701467	1.0563584	1.0862563	1.1823531	0.8232630
BTBD18	0.0006630	0.0000000	0.0000000	0.0000000	0.0059803
CTNND1	0.3536995	0.3166218	0.2683108	0.3108113	0.5375410
ENSG00000290827	0.0016855	0.0000000	0.0000000	0.0000000	0.0000000
OR9Q1	0.0013718	0.0000000	0.0000000	0.0000000	0.0000000
ENSG00000255299	0.0000000	0.0000000	0.0000000	0.0045838	0.0051058

LPXN	0.0491539	0.0408390	0.0472718	0.0578059	0.0605018
ZFP91	0.8737474	0.7746615	0.7267181	0.8091311	0.5816599
CNTF	0.0033935	0.0057502	0.0000000	0.0084737	0.0105489
GLYAT	0.0000000	0.0018371	0.0000000	0.0023036	0.0051589
GLYATL2	0.1152149	0.2746006	0.2419432	0.0780331	0.5309573
ENSG00000290884	0.0066611	0.0120458	0.0050260	0.0000000	0.0202726
GLYATL1	0.0022071	0.0051264	0.0129021	0.0000000	0.0190627
ENSG00000255240	0.0184940	0.0356359	0.0469802	0.0111780	0.0547512
FAM111B	0.0150103	0.0029218	0.0063152	0.0125097	0.0036228
FAM111A-DT	0.0210437	0.0131526	0.0122843	0.0399034	0.0091761
FAM111A	0.1006354	0.0466850	0.0193028	0.1762261	0.0519141
DTX4	0.2600303	0.2906183	0.3740494	0.3526145	0.3411043
OR4D9	0.0000000	0.0000000	0.0000000	0.0000000	0.0000000
ENSG00000287264	0.0039394	0.0042131	0.0000000	0.0026331	0.0000000
LINC02739	0.0021147	0.0043786	0.0094718	0.0032979	0.0000000
ENSG00000255139	0.0280788	0.0147644	0.0132963	0.0272169	0.0241102
OSBP	0.3627316	0.3050488	0.2775558	0.3338636	0.3976412
PATL1	0.1642245	0.1395355	0.1585065	0.1378259	0.1603363
PATL1-DT	0.0036964	0.0008398	0.0013234	0.0068024	0.0000000
STX3	0.0948724	0.1261073	0.1612098	0.0616279	0.1530079
ENSG00000254477	0.0000000	0.0000000	0.0075707	0.0014279	0.0127386
MRPL16	0.1762043	0.1692650	0.1380971	0.2290794	0.1364682
CBLIF	0.0000000	0.0000000	0.0000000	0.0000000	0.0062700
OOSP3	0.0000000	0.0014000	0.0000000	0.0000000	0.0000000
OOSP1	0.0012870	0.0000000	0.0011203	0.0032050	0.0045524
MS4A4E	0.0004416	0.0021043	0.0000000	0.0000000	0.0051058
MS4A7	0.0014681	0.0000000	0.0000000	0.0000000	0.0000000
MS4A13	0.0000000	0.0000000	0.0000000	0.0000000	0.0048241
MS4A8	0.1114841	0.0646531	0.0316168	0.0824393	0.0566161
CCDC86	0.1679866	0.0966995	0.0733560	0.1157425	0.0843957
PTGDR2	0.0074953	0.0009027	0.0000000	0.0079077	0.0000000
PRPF19	0.2699418	0.2241342	0.2802171	0.2631415	0.1802379
PRPF19-DT	0.0036339	0.0008934	0.0020277	0.0050634	0.0000000
TMEM109-DT	0.0005727	0.0009612	0.0000000	0.0000000	0.0091935
TMEM109	0.1531784	0.1138664	0.0623367	0.1828102	0.1044069
ENSG00000256196	0.0029307	0.0004001	0.0038795	0.0000000	0.0000000
TMEM132A	0.2657180	0.2281218	0.3283227	0.4577922	0.2056782
SLC15A3	0.0008486	0.0000000	0.0027684	0.0000000	0.0041632
CD6	0.0069167	0.0144823	0.0250619	0.0000000	0.0092627
VPS37C	0.0608443	0.0450243	0.0406441	0.0693202	0.0311147
VWCE	0.0123359	0.0154611	0.0084853	0.0104853	0.0532123
DDB1	0.4661078	0.4034487	0.3704606	0.4439770	0.3486974
TKFC	0.1295899	0.0947961	0.0583478	0.1138907	0.0493860
CYB561A3	0.0744793	0.0806640	0.0588315	0.0968176	0.0597143
TMEM138	0.1461361	0.0997437	0.1162608	0.1759129	0.1425029
TMEM216	0.1215822	0.0708775	0.0900495	0.1138092	0.0808402
CPSF7	0.1972083	0.2134038	0.1662297	0.1799558	0.2015643
SDHAF2	0.3349002	0.2873285	0.3310378	0.2916921	0.1983951
PPP1R32	0.1304054	0.0651319	0.0643589	0.1508884	0.0771567
ENSG00000255931	0.0060142	0.0036245	0.0036933	0.0071438	0.0132694
LRRC10B	0.0426152	0.0278112	0.0107339	0.0777651	0.0193816
SYT7	0.1138563	0.2017607	0.2460194	0.0806894	0.3091301
ENSG00000256443	0.0012772	0.0011635	0.0006071	0.0000000	0.0229169

DAGLA	0.0318740	0.0335572	0.0351858	0.0134094	0.0454857
MYRF-AS1	0.0003655	0.0000000	0.0007881	0.0000000	0.0000000
MYRF	0.0017261	0.0046133	0.0012772	0.0016951	0.0172391
TMEM258	1.2821562	1.2404226	1.2361230	1.2632820	1.1235641
FEN1	0.0579132	0.0548992	0.0606506	0.0653434	0.0362104
FADS2	0.3374806	0.3290233	0.3756436	0.4931739	0.3698267
ENSG00000289268	0.0302111	0.0332644	0.0403830	0.0244064	0.0552963
FADS1	0.7276699	0.7115464	0.7235531	0.8316918	0.5928823
FADS3	0.0828806	0.1378374	0.1413778	0.0898769	0.1526363
RAB3IL1	0.0349564	0.0408291	0.0197773	0.0250626	0.0247362
BEST1	0.0989884	0.0748073	0.0792554	0.0897191	0.1024736
FTH1	4.5906737	4.4739420	4.2772263	4.4173646	4.2443788
LINC02733	0.0000000	0.0052761	0.0000000	0.0000000	0.0000000
ENSG00000285656	0.0017170	0.0000000	0.0000000	0.0015674	0.0000000
INCENP	0.0267817	0.0641968	0.0466587	0.0414955	0.0440283
ENSG00000289194	0.0108755	0.0071224	0.0297589	0.0152947	0.0332509
SCGB2A1	0.0031506	0.0000000	0.0000000	0.0062992	0.0000000
SCGB1D2	0.0046712	0.0011581	0.0049676	0.0113012	0.0056738
ENSG00000255118	0.0013404	0.0000000	0.0000000	0.0000000	0.0000000
ASRGL1	0.1755909	0.3071323	0.3942019	0.1535008	0.2495761
ENSG00000255126	0.0000000	0.0000000	0.0000000	0.0000000	0.0000000
SCGB1A1	0.0378752	0.0268468	0.0219741	0.1926777	0.0378122
AHNAK	0.1531498	0.1215151	0.0607145	0.1904124	0.1266397
ENSG00000257058	0.0116797	0.0115508	0.0015064	0.0188838	0.0074097
EEF1G	2.4614749	2.4806018	2.2942011	2.4211964	2.1632283
ENSG00000289562	0.0105519	0.0150461	0.0087695	0.0075997	0.0038661
TUT1	0.0553495	0.0594990	0.0325055	0.0871469	0.0354625
MTA2	0.1034390	0.0753262	0.0769367	0.0913151	0.0479667
EML3	0.0077614	0.0058512	0.0054039	0.0176929	0.0028046
ROM1	0.0205867	0.0197722	0.0053077	0.0245128	0.0125817
B3GAT3	0.3712924	0.3839377	0.3524093	0.4563278	0.2924205
GANAB	0.3228024	0.2383833	0.2237016	0.5112806	0.2594168
INTS5	0.0660552	0.0762344	0.0696440	0.0797919	0.0462891
C11orf98	0.0481278	0.0467548	0.0402633	0.0422543	0.0217097
LBHD1	0.0063729	0.0032457	0.0000000	0.0000000	0.0026235
CSKMT	0.0699682	0.0685531	0.0482521	0.0856417	0.1019439
UQCC3	0.2801416	0.2680299	0.2836294	0.3527094	0.2215939
UBXN1	0.6981286	0.6700920	0.7026265	0.6990541	0.5789220
LRRN4CL	0.0189463	0.0039574	0.0077994	0.0102515	0.0054431
BSCL2	1.3822064	1.2710273	1.2572532	1.5378947	1.1218872
GNG3	0.5311932	0.9539426	1.3522779	0.4377743	0.6508488
HNRNPUL2	0.5124575	0.4682145	0.4943885	0.4796230	0.4186547
TTC9C	0.2384984	0.2329466	0.2544754	0.2041969	0.1993988
ZBTB3	0.0400624	0.0410342	0.0340102	0.0408087	0.0502738
POLR2G	0.6965245	0.5292882	0.5003815	0.6792816	0.4666100
ENSG00000267811	0.0409171	0.0167666	0.0290461	0.0365131	0.0221399
TAF6L	0.0423631	0.0267508	0.0485709	0.0671139	0.0283892
TMEM223	0.2620647	0.1829798	0.1754213	0.3090940	0.2237585
ENSG00000269176	0.0433137	0.0172713	0.0130310	0.0244506	0.0264573
TMEM179B	0.4441607	0.2921665	0.2624268	0.5717397	0.2792988
NXF1	0.0738932	0.0850566	0.0596334	0.0629537	0.0831394
STX5	0.2326290	0.1984852	0.1272937	0.2067385	0.2069397
ENSG00000269463	0.0039872	0.0000000	0.0006255	0.0000000	0.0000000

STX5-DT	0.0039113	0.0012821	0.0000000	0.0068846	0.0000000
WDR74	0.1341527	0.1344233	0.1526644	0.1203607	0.0863167
SNHG1	0.2350868	0.2133742	0.1829457	0.2328844	0.1784676
SLC3A2	1.0896787	0.8111833	0.7360290	1.3168715	0.7539633
SLC22A6	0.0017264	0.0037479	0.0028430	0.0085049	0.0038839
PLAAT5	0.0074583	0.0043756	0.0054628	0.0139027	0.0000000
PLAAT4	0.0382314	0.0184666	0.0119699	0.0577911	0.0049869
PLAAT2	0.0048123	0.0040762	0.0051962	0.0070570	0.0000000
PLAAT3	0.9351375	0.7033871	0.5639602	1.0265337	0.6081415
ATL3	0.1225734	0.0995318	0.0570371	0.1364677	0.1382223
ENSG00000256789	0.0000000	0.0000000	0.0000000	0.0000000	0.0061238
RTN3	1.7406092	1.8653126	2.1927095	2.0267806	1.8721571
ZFTA	0.0967860	0.1025215	0.0734827	0.1030207	0.0679723
SPINDOC	0.1120933	0.0930287	0.1097670	0.0940414	0.0796990
MARK2	0.2404420	0.2563975	0.2339755	0.2341007	0.2706654
RCOR2	0.0931812	0.1199939	0.1399040	0.0938577	0.1526417
NAA40	0.1131738	0.1111026	0.1176663	0.1035773	0.1217956
COX8A	1.6091099	1.6897373	1.7772968	1.5235385	1.3841100
OTUB1	0.5163722	0.4755739	0.5466286	0.5275211	0.4039160
MACROD1	0.1641442	0.1782431	0.2332310	0.1585385	0.1970741
FLRT1	0.0428816	0.0667910	0.0527487	0.0500843	0.0586861
ENSG00000256481	0.0058725	0.0093323	0.0057890	0.0000000	0.0000000
ENSG00000256341	0.0016249	0.0108510	0.0138753	0.0000000	0.0115187
ENSG00000288852	0.0055771	0.0102529	0.0080197	0.0072066	0.0206356
ENSG00000289486	0.0095915	0.0122085	0.0078840	0.0112770	0.0093931
STIP1	0.7358427	0.7698969	0.8952219	0.7795088	0.5910024
FERMT3	0.0011766	0.0020116	0.0000000	0.0013619	0.0042221
TRPT1	0.2745729	0.3036755	0.2192519	0.2692457	0.1921524
NUDT22	0.1451810	0.1141538	0.0857356	0.1596664	0.1326384
ENSG00000256116	0.0056003	0.0099757	0.0061951	0.0109825	0.0140215
DNAJC4	0.4536596	0.3970450	0.2949389	0.3936088	0.2641808
VEGFB	1.0720216	1.0907837	1.1225440	1.0527125	0.9071138
FKBP2	0.0721436	0.0461842	0.0504109	0.0661686	0.0464223
ENSG00000286264	0.0057416	0.0107413	0.0024630	0.0018752	0.0000000
PPP1R14B	0.9806152	1.0299897	0.9239694	1.0263580	0.8162118
PPP1R14B-AS1	0.0182571	0.0062509	0.0099199	0.0036233	0.0064805
ENSG00000257086	0.0000000	0.0030174	0.0047254	0.0010179	0.0000000
PLCB3	0.0350000	0.0391106	0.0103043	0.0335321	0.0408182
BAD	0.6814389	0.6497255	0.6631395	0.6740106	0.4724429
GPR137	0.1848509	0.2178742	0.2313560	0.2266488	0.1581114
KCNK4	0.0150268	0.0446588	0.0316242	0.0215795	0.0396892
CATSPERZ	0.0049798	0.0199080	0.0282604	0.0058576	0.0076015
ESRRA	0.0251293	0.0418437	0.0255030	0.0262431	0.0587069
TRMT112	1.2725546	1.1189411	1.1083662	1.1870669	0.9272789
PRDX5	1.5000488	1.6005927	1.6397462	1.4757809	1.2799902
ENSG00000236935	0.0017510	0.0000000	0.0000000	0.0000000	0.0077245
CCDC88B	0.0203436	0.0242125	0.0094963	0.0456852	0.0927432
RPS6KA4	0.0375387	0.0473042	0.0292497	0.0490795	0.0640009
LINC02724	0.0062790	0.0038792	0.0060471	0.0000000	0.0045621
NRXN2	0.3169358	0.5655279	0.7330689	0.1939616	0.7277506
NRXN2-AS1	0.0000000	0.0015188	0.0000000	0.0000000	0.0027945
ENSG00000289058	0.0076835	0.0059359	0.0000000	0.0099149	0.0024068
RASGRP2	0.0754085	0.1373790	0.1292370	0.0507566	0.0964696

PYGM	0.0090619	0.0121817	0.0029583	0.0096272	0.0037060
SF1	0.5251171	0.5293905	0.5308478	0.5799231	0.5187588
SF1-DT	0.0131944	0.0119239	0.0106832	0.0114071	0.0300666
MAP4K2	0.0383004	0.0594653	0.0449581	0.0516712	0.0638964
MEN1	0.0625043	0.0572960	0.0589593	0.0729588	0.0460439
CDC42BPG	0.0000000	0.0033087	0.0000000	0.0000000	0.0035158
EHD1	0.1578623	0.1800697	0.1390900	0.1067511	0.1721341
ATG2A	0.0257104	0.0276037	0.0208732	0.0340446	0.0425287
PPP2R5B	0.2024429	0.3723680	0.3288350	0.1881837	0.2178088
MAJIN	0.0000000	0.0000000	0.0000000	0.0000000	0.0000000
BATF2	0.0017578	0.0012123	0.0090905	0.0000000	0.0000000
ARL2	0.7148758	0.7816872	0.8174495	0.6628017	0.5917435
SNX15	0.1688848	0.1939025	0.2186953	0.1386740	0.1484874
SAC3D1	0.1958227	0.2227548	0.2782232	0.2642462	0.1884726
CDCA5	0.0098429	0.0153132	0.0176369	0.0211276	0.0067404
ZFPL1	0.2398667	0.2221946	0.1898198	0.2634383	0.1816012
TMEM262	0.0025943	0.0000000	0.0017404	0.0044354	0.0000000
VPS51	0.2443229	0.2406985	0.1830414	0.2711249	0.1949013
ENSG00000254501	0.0020096	0.0008645	0.0041285	0.0000000	0.0115944
TM7SF2	0.4452316	0.3677954	0.4177316	0.6135625	0.2813325
ZNHIT2	0.0868496	0.0655594	0.0735840	0.0846219	0.0315728
FAU	2.6425225	2.6708697	2.5613905	2.5454611	2.3652710
SYVN1	0.0711057	0.0574377	0.0440271	0.0730039	0.1053690
MRPL49	0.2936638	0.2364246	0.2062251	0.3506488	0.1886243
CAPN1-AS1	0.0051144	0.0101139	0.0020128	0.0118864	0.0000000
CAPN1	0.1796169	0.2039046	0.2004248	0.1750276	0.2216260
POLA2	0.0269477	0.0141898	0.0231011	0.0306125	0.0316617
ENSG00000287917	0.0237534	0.0041328	0.0051262	0.0223625	0.0088594
CDC42EP2	0.1356878	0.0843952	0.0725611	0.1320645	0.0745906
DPF2	0.2977633	0.2557758	0.2441112	0.2902226	0.2141703
ENSG00000289231	0.0025806	0.0029609	0.0124239	0.0000000	0.0045404
TIGD3	0.0109303	0.0118358	0.0272413	0.0177789	0.0101363
ENSG00000255478	0.0000000	0.0000000	0.0031481	0.0000000	0.0000000
SLC25A45	0.0051392	0.0000000	0.0038279	0.0074270	0.0016673
FRMD8	0.0238506	0.0183670	0.0160125	0.0305386	0.0142692
ENSG00000290057	0.0009675	0.0018728	0.0027554	0.0000000	0.0016619
NEAT1	0.4217437	0.4139857	0.3515182	0.2982917	0.9894261
ENSG00000291174	0.1305900	0.2052606	0.2470828	0.1125450	0.2433118
ENSG00000289883	0.0005818	0.0000000	0.0000000	0.0036549	0.0000000
ENSG00000286756	0.0026953	0.0043862	0.0022206	0.0023665	0.0261099
MALAT1	4.0065703	4.0526788	3.6454298	3.7759244	4.8605505
ENSG00000270117	0.0024823	0.0019419	0.0016984	0.0000000	0.0000000
TALAM1	1.0433588	1.0186894	0.7795979	0.8008328	2.5320614
SCYL1	0.1966350	0.2135241	0.2326316	0.1983579	0.1759126
LTBP3	0.0993509	0.0817157	0.0590090	0.1530950	0.0942721
ZNRD2-DT	0.0097979	0.0046964	0.0047254	0.0128210	0.0057307
ZNRD2	0.2388670	0.1983697	0.1704996	0.2539981	0.1776342
FAM89B	0.3285303	0.4700609	0.4560869	0.3723880	0.2945397
EHBP1L1	0.0191039	0.0139737	0.0329233	0.0083323	0.0408682
KCNK7	0.0000000	0.0000000	0.0000000	0.0012937	0.0032929
MAP3K11	0.0856306	0.0973898	0.0533714	0.1001447	0.0747166
PCNX3	0.0563270	0.0517122	0.0365366	0.0998065	0.1087414
SIPA1	0.0101373	0.0055773	0.0137224	0.0101987	0.0104876

RELA	0.1871981	0.1166606	0.0964083	0.1923599	0.1417010
RELA-DT	0.0857921	0.0540843	0.0500924	0.0519207	0.0204128
KAT5	0.2076229	0.2048711	0.1701762	0.1823065	0.1585565
RNASEH2C	0.3274803	0.2804116	0.2598232	0.3181090	0.2238849
ENSG00000255557	0.0006731	0.0073928	0.0014107	0.0000000	0.0081518
AP5B1	0.0396012	0.0418335	0.0572539	0.0406644	0.0544678
OVOL1-AS1	0.0124804	0.0087233	0.0028040	0.0010526	0.0000000
CFL1	2.1417587	2.2619942	2.5642248	2.2533695	1.8785546
SNX32	0.0243533	0.0509089	0.0289297	0.0156709	0.1592384
MUS81	0.1558390	0.1329308	0.1177531	0.2051057	0.1276231
EFEMP2	0.5659688	0.3747059	0.2272839	0.8226940	0.3236887
FIBP	0.5490337	0.6083394	0.6046077	0.5323147	0.4895190
CCDC85B	0.3619205	0.4538694	0.5448906	0.3722990	0.3042767
FOSL1	0.0007854	0.0012560	0.0000000	0.0026362	0.0015298
C11orf68	0.2120439	0.2110483	0.2369520	0.2114970	0.1302610
DRAP1	0.9330580	1.1261571	1.2859844	0.9261523	0.9115999
TSGA10IP	0.0038812	0.0026760	0.0030643	0.0048478	0.0000000
SART1	0.1771622	0.1472379	0.1538749	0.1630451	0.1525132
EIF1AD	0.0825459	0.1202987	0.0915643	0.0646600	0.0921330
BANF1	1.4567118	1.3718254	1.3980250	1.4021574	1.1471698
GAL3ST3	0.3071063	0.2530965	0.2539906	0.3432207	0.2282566
SF3B2	1.3724760	1.2740424	1.3204664	1.3011980	1.0735777
ENSG00000255038	0.0017743	0.0000000	0.0000000	0.0000000	0.0000000
PACS1	0.6781604	0.8083856	0.9076592	0.5847909	0.9064658
ENSG00000255320	0.0299069	0.0236653	0.0083853	0.0194358	0.0425407
KLC2	0.1223613	0.1797147	0.2228753	0.1281810	0.2500036
ENSG00000254461	0.0020473	0.0000000	0.0028368	0.0000000	0.0089128
RAB1B	0.3229201	0.2950530	0.2315074	0.3077947	0.1497921
ENSG00000245156	0.0020112	0.0048368	0.0000000	0.0040485	0.0048707
ENSG00000254452	0.0042640	0.0034710	0.0046214	0.0082614	0.0043847
CNIH2	0.9930834	1.0992429	1.3120676	0.9269294	0.8798143
YIF1A	0.5850343	0.4013467	0.3144682	0.6204097	0.3486247
TMEM151A	0.0454520	0.0824258	0.1537008	0.0439799	0.1383044
CD248	0.0249841	0.0119942	0.0032625	0.0435762	0.0171759
RIN1	0.0138376	0.0086602	0.0040220	0.0115671	0.0126572
BRMS1	0.0928336	0.0769751	0.0244154	0.0848192	0.0849754
B4GAT1	0.6809452	0.5347532	0.6076842	1.1562579	0.5449757
B4GAT1-DT	0.0573532	0.0573077	0.0648960	0.0439566	0.0809515
SLC29A2	0.0336034	0.0445026	0.0505705	0.0674742	0.0465145
ENSG00000254510	0.0035892	0.0172023	0.0232247	0.0000000	0.0035970
NPAS4	0.0206580	0.0223249	0.0314411	0.0242235	0.0743251
MRPL11	0.2202435	0.2171715	0.2315953	0.1717250	0.1699433
PELI3	0.0681984	0.0797762	0.0844251	0.0477653	0.0468951
DPP3-DT	0.0922393	0.0557435	0.0199867	0.0683991	0.0446892
DPP3	0.1186451	0.1102475	0.0975929	0.1251219	0.1043371
BBS1	0.1686124	0.1278496	0.1148424	0.2019496	0.1699105
ENSG00000286688	0.0040497	0.0025999	0.0037885	0.0000000	0.0126082
ZDHHC24	0.1266450	0.1184595	0.0803301	0.1452347	0.1099315
ACTN3	0.0026400	0.0010534	0.0000000	0.0000000	0.0017986
CTSF	0.0288061	0.0240745	0.0268608	0.0651124	0.0214177
CCDC87	0.0006159	0.0017515	0.0000000	0.0057504	0.0054919
CCS	0.4522991	0.2908883	0.3244623	0.4287228	0.2684894
RBM14	0.1003650	0.1192479	0.1491651	0.1077590	0.2097199

RBM4	0.6598997	0.6562013	0.6847725	0.6333684	0.6447074
RBM4B	0.2789527	0.2538357	0.2784246	0.2751182	0.2283626
SPTBN2	0.1576586	0.2509763	0.2407386	0.1318684	0.3003688
C11orf80	0.1940634	0.2589821	0.2088131	0.1436140	0.5231324
RCE1	0.0604472	0.0473802	0.0565266	0.0393296	0.0591278
PC	0.0615356	0.0327353	0.0438449	0.0518715	0.0640600
LRFN4	0.2499417	0.3398175	0.3283328	0.2586145	0.2994064
SYT12	0.0139757	0.0240262	0.0716912	0.0114887	0.0926219
RHOD	0.0108873	0.0035611	0.0000000	0.0051613	0.0137073
KDM2A	0.3558505	0.3437154	0.3018783	0.3252663	0.6077358
ENSG00000287934	0.0000000	0.0000000	0.0000000	0.0000000	0.0000000
GRK2	0.2263418	0.2949614	0.3844058	0.2081256	0.2489290
ANKRD13D	0.1939837	0.2837213	0.3362833	0.1940980	0.2312638
SSH3	0.0528440	0.0183751	0.0278221	0.0487805	0.0396100
ENSG00000287851	0.0000000	0.0000000	0.0000000	0.0000000	0.0000000
RAD9A	0.0940566	0.1013105	0.0612557	0.1124293	0.2140486
POLD4	0.2143328	0.1555556	0.0966443	0.1793748	0.1455282
CLCF1	0.0104916	0.0038575	0.0102111	0.0000000	0.0179371
PPP1CA	0.3352531	0.3515992	0.3381866	0.3503045	0.2040349
CARNS1	0.0049205	0.0030729	0.0000000	0.0000000	0.0071154
RPS6KB2	0.1239923	0.1018585	0.0622836	0.1394593	0.0614127
CORO1B	0.2488131	0.2188137	0.2078093	0.2306418	0.1203086
CABP4	0.0005131	0.0008000	0.0000000	0.0000000	0.0012783
TMEM134	0.4413290	0.3741426	0.3156640	0.4358441	0.2405784
AIP	0.3616285	0.3044911	0.3066989	0.3693836	0.2517208
PITPNM1	0.1531293	0.1684885	0.1569274	0.1414900	0.1674540
CDK2AP2	0.4418862	0.3523477	0.2835784	0.4829993	0.2696624
ENSG00000289343	0.0000000	0.0012671	0.0000000	0.0000000	0.0000000
GSTP1	1.9885368	1.7213412	1.6038597	2.0133651	1.4987479
NDUFV1-DT	0.0049768	0.0000000	0.0016034	0.0000000	0.0000000
NDUFV1	0.4771020	0.5503973	0.5569144	0.4775768	0.3819403
NUDT8	0.0416788	0.0506920	0.0321832	0.0483508	0.0389391
TBX10	0.0000000	0.0013043	0.0000000	0.0000000	0.0000000
FAM86C2P	0.0467955	0.0274835	0.0244872	0.0588939	0.0221347
LINC02754	0.0028254	0.0000000	0.0042071	0.0037958	0.0050073
ENSG00000290995	0.0050427	0.0050871	0.0009493	0.0049966	0.0128550
UNC93B1	0.2354805	0.1168167	0.0593556	0.2840262	0.1108558
ALDH3B1	0.1051850	0.0488971	0.0240481	0.1146999	0.0620930
ENSG00000255306	0.0015516	0.0033010	0.0000000	0.0000000	0.0015298
NDUFS8	0.8620833	0.8720357	0.8993402	0.8337223	0.6215713
TCIRG1	0.0092258	0.0017897	0.0057303	0.0099039	0.0062856
ENSG00000255031	0.0000000	0.0010514	0.0032625	0.0000000	0.0000000
CHKA	0.4660059	0.4053150	0.3889786	0.5424143	0.3774814
CHKA-DT	0.0046444	0.0024472	0.0012577	0.0000000	0.0016664
KMT5B	0.7452608	0.7610827	0.7679134	0.6809760	0.7841692
C11orf24	0.1395087	0.1283255	0.1413227	0.1644139	0.0814298
ENSG00000286369	0.1044150	0.0676113	0.0930890	0.0951000	0.0483740
LRP5	0.1062503	0.0720102	0.0259852	0.0670067	0.0794673
PPP6R3	0.3246453	0.3205232	0.2938677	0.2682500	0.6318359
ENSG00000260808	0.0010072	0.0013303	0.0000000	0.0000000	0.0000000
GAL	0.0268948	0.0300497	0.0533571	0.0139916	0.0265540
TESMIN	0.0243249	0.0494649	0.0356353	0.0157772	0.0320048
CPT1A	0.0478015	0.0386735	0.0201163	0.0781047	0.0359252

MRPL21	0.6361069	0.6798123	0.7310908	0.6387864	0.5371233
IGHMBP2	0.0827862	0.1115956	0.1075021	0.0655856	0.1444135
MRGPRF	0.0023968	0.0024327	0.0034975	0.0000000	0.0000000
TPCN2	0.0167453	0.0164793	0.0137594	0.0242268	0.0811722
ENSG00000261070	0.0000000	0.0014045	0.0000000	0.0000000	0.0022158
SMIM38	0.0058230	0.0065461	0.0051881	0.0092646	0.0092159
ENSG00000260877	0.0010504	0.0009308	0.0032862	0.0032417	0.0077504
CCND1	0.7893282	0.4954101	0.3169411	0.4972477	0.4994869
LTO1	0.0830519	0.0747602	0.1029394	0.0790629	0.1013815
ANO1	0.0024288	0.0021783	0.0000000	0.0011920	0.0000000
FADD	0.1039211	0.0808866	0.0801870	0.0968414	0.0640392
ENSG00000289074	0.0047762	0.0059394	0.0085833	0.0024107	0.0185480
PPFIA1	0.3263013	0.3625723	0.2604908	0.3300129	0.4434540
ENSG00000254604	0.0122736	0.0137118	0.0154683	0.0182318	0.0382504
ENSG00000254484	0.0007454	0.0000000	0.0000000	0.0000000	0.0000000
CTTN-DT	0.0471414	0.0326266	0.0400061	0.0376062	0.0617010
CTTN	0.4461496	0.4250877	0.4013890	0.4628487	0.4699784
SHANK2	0.2832819	0.2746224	0.2200360	0.2979547	0.7331778
SHANK2-AS1	0.0000000	0.0010918	0.0000000	0.0000000	0.0076089
SHANK2-AS2	0.0000000	0.0000000	0.0000000	0.0000000	0.0000000
ENSG00000286708	0.0000000	0.0012516	0.0000000	0.0000000	0.0100952
DHCR7	0.1599004	0.1097681	0.1078506	0.2029106	0.1143140
ENSG00000254682	0.1409643	0.1271417	0.1604700	0.1508102	0.0873869
NADSYN1	0.0734870	0.1028633	0.0832204	0.0918718	0.1460343
ENSG00000286948	0.0061686	0.0214360	0.0165500	0.0115864	0.0439180
KRTAP5-11	0.0009987	0.0000000	0.0000000	0.0000000	0.0000000
ALG1L9P	0.0400203	0.0420819	0.0228126	0.0489935	0.1746956
ENSG00000290775	0.0005501	0.0018587	0.0000000	0.0000000	0.0069257
ENSG00000291247	0.0008247	0.0000000	0.0000000	0.0000000	0.0054362
ENSG00000291186	0.0222985	0.0309088	0.0157852	0.0124719	0.0367213
XNDC1N	0.0359169	0.0448671	0.0278182	0.0380975	0.0700027
RNF121	0.1105671	0.0997603	0.0804022	0.1038671	0.0925841
IL18BP	0.0057207	0.0087575	0.0095859	0.0037010	0.0278481
NUMA1	0.3640932	0.2879974	0.2713699	0.3373395	0.4356616
ENSG00000251143	0.0031756	0.0049920	0.0007799	0.0000000	0.0017223
LRRC51	0.2843938	0.1805144	0.1361521	0.3261442	0.1735591
LAMTOR1	1.0645447	1.0213328	1.0048458	1.1241885	0.7490058
TOMT	0.0021770	0.0012444	0.0021796	0.0000000	0.0068646
ANAPC15	0.2798834	0.3118049	0.3165259	0.2982720	0.2500246
FOLR3	0.0000000	0.0000000	0.0000000	0.0049959	0.0012770
ENSG00000204971	0.0004832	0.0008684	0.0012847	0.0000000	0.0000000
FOLR1	0.0020363	0.0030630	0.0000000	0.0082181	0.0000000
INPPL1	0.0730176	0.0588202	0.0533045	0.0851146	0.0607727
PHOX2A	0.0155177	0.0310273	0.0290120	0.0343599	0.1035871
ENSG00000285864	0.0024476	0.0064965	0.0066733	0.0036117	0.0110749
CLPB	0.1078897	0.1910721	0.1998547	0.1337006	0.1594605
ENSG00000255672	0.0000000	0.0000000	0.0000000	0.0000000	0.0085325
PDE2A	0.0203336	0.0238252	0.0409139	0.0130059	0.0510267
ARAP1	0.0911394	0.0511658	0.0440102	0.0666038	0.0906069
STARD10	0.1260523	0.1468828	0.1664478	0.0753501	0.1260567
ENSG00000285693	0.0125598	0.0075083	0.0069532	0.0022730	0.0213261
ATG16L2	0.0358084	0.0486319	0.0608931	0.0450049	0.0238029
FCHSD2	0.5728495	0.6027072	0.6176724	0.5351473	0.9925550

ENSG00000256568	0.0026093	0.0000000	0.0000000	0.0000000	0.0000000
P2RY6	0.0000000	0.0014737	0.0000000	0.0000000	0.0000000
ARHGEF17	0.0661273	0.1127311	0.1263587	0.1185661	0.1576559
RELT	0.0256291	0.0249869	0.0461154	0.0243711	0.0339837
FAM168A	0.4505105	0.5533820	0.4885845	0.3885708	0.9346983
ENSG00000256448	0.0000000	0.0018203	0.0020407	0.0000000	0.0124048
PLEKHB1	0.2149956	0.1400784	0.1163014	0.2883674	0.1174268
RAB6A	0.7677334	0.9067172	1.0240103	0.6490816	0.9363449
ENSG00000256034	0.0023353	0.0033277	0.0014518	0.0000000	0.0000000
MRPL48	0.3048108	0.3671924	0.4135739	0.3302731	0.2772413
COA4	0.4920042	0.4587809	0.3632521	0.5109384	0.3018229
PAAF1	0.3059643	0.2431564	0.2130058	0.2345168	0.2328132
DNAJB13	0.0284718	0.0169908	0.0044772	0.0198846	0.0262936
ENSG00000255847	0.0000000	0.0000000	0.0000000	0.0000000	0.0000000
UCP2	0.4073441	0.2519363	0.1485781	0.3841984	0.2405993
ENSG00000287425	0.0027601	0.0014023	0.0000000	0.0000000	0.0000000
UCP3	0.0012327	0.0051713	0.0051266	0.0040830	0.0154520
C2CD3	0.1021170	0.1135633	0.0897917	0.0954316	0.2012579
PPME1	0.4384347	0.4600804	0.5795091	0.4152906	0.4277457
P4HA3	0.0094483	0.0032907	0.0000000	0.0038795	0.0169153
PGM2L1	0.7761202	1.3706257	1.6735460	0.6981823	1.2518403
ENSG00000254631	0.0058988	0.0014737	0.0037517	0.0000000	0.0016619
ENSG00000255440	0.0018554	0.0000000	0.0000000	0.0022483	0.0034299
LIPT2	0.0326474	0.0286670	0.0222666	0.0219206	0.0251981
LIPT2-AS1	0.2189605	0.2822665	0.2833505	0.1899209	0.2359198
POLD3	0.3500627	0.2455000	0.2748596	0.3392276	0.2659411
CHRDL2	0.0000000	0.0018084	0.0031125	0.0018058	0.0000000
RNF169	0.2459470	0.2150100	0.1791450	0.2143831	0.2816441
XRRA1	0.1420684	0.1346154	0.1346162	0.0678240	0.2671427
SPCS2	1.2598737	1.1754405	1.2042506	1.5408374	1.0164979
NEU3	0.0259057	0.0485110	0.0673092	0.0327714	0.0544104
OR2AT4	0.0000000	0.0023275	0.0000000	0.0000000	0.0000000
SLCO2B1	0.0010252	0.0000000	0.0000000	0.0024504	0.0000000
TPBGL-AS1	0.0175088	0.0488658	0.0725379	0.0415522	0.1071083
TPBGL	0.0023950	0.0057932	0.0141463	0.0000000	0.0000000
ARRB1	0.1158991	0.2029168	0.2469563	0.1047002	0.2799462
RPS3	2.7467445	2.6742993	2.5248828	2.6271823	2.4603423
KLHL35	0.0541761	0.1059121	0.1172274	0.0628900	0.0594563
GDPD5	0.0509082	0.0504332	0.0679995	0.0799708	0.1054179
SERPINH1	0.9189259	0.5689792	0.3554370	1.2960927	0.5594359
ENSG00000255326	0.0140844	0.0049938	0.0049204	0.0074228	0.0094427
MAP6	1.3318122	1.4098613	1.5724789	1.2611148	1.2202847
ENSG00000255434	0.0000000	0.0000000	0.0000000	0.0000000	0.0000000
DGAT2	0.0035210	0.0126858	0.0193330	0.0072237	0.0147246
UVRAG-DT	0.0180959	0.0110145	0.0049011	0.0038691	0.0146357
UVRAG	0.3410625	0.3847840	0.3682534	0.3420004	0.5648551
ENSG00000255081	0.0008963	0.0000000	0.0000000	0.0000000	0.0000000
WNT11	0.0063594	0.0076251	0.0178895	0.0017461	0.0000000
LINC02761	0.0000000	0.0013800	0.0015202	0.0000000	0.0000000
THAP12	0.3577117	0.3691212	0.4145100	0.3479080	0.3013747
GVQW3	0.1580391	0.2414802	0.2969118	0.0864567	0.2682175
EMSY-DT	0.2631859	0.2002752	0.2141474	0.2860806	0.1038039
EMSY	0.1749353	0.1661887	0.1653558	0.1712513	0.3368483

LRRC32	0.0035581	0.0012225	0.0042079	0.0028566	0.0000000
TSKU	0.0802776	0.0470681	0.0306281	0.0817964	0.0544416
TSKU-AS1	0.0108046	0.0069760	0.0053011	0.0164539	0.0000000
ENSG00000261578	0.0078462	0.0007544	0.0026926	0.0065551	0.0000000
ACER3	0.1582673	0.2014643	0.1970839	0.1594057	0.3164732
ENSG00000254988	0.0000000	0.0000000	0.0000000	0.0000000	0.0000000
B3GNT6	0.0000000	0.0000000	0.0000000	0.0000000	0.0087132
CAPN5	0.0919684	0.1033395	0.1039834	0.0537159	0.1094063
MYO7A	0.0415528	0.0257129	0.0196071	0.0322216	0.0506796
GDPD4	0.0000000	0.0010749	0.0000000	0.0036094	0.0096255
PAK1	0.3003527	0.4835392	0.6414644	0.2416198	0.5829249
ENSG00000268635	0.0020086	0.0013447	0.0009114	0.0000000	0.0035216
CLNS1A	1.0399253	0.7791008	0.6595701	1.0809664	0.7437274
AQP11	0.1597960	0.1678900	0.1774896	0.1624305	0.1286450
RSF1	1.0791447	1.0232533	1.1261441	1.0428426	1.0400133
RSF1-IT2	0.0004676	0.0000000	0.0000000	0.0000000	0.0000000
RSF1-IT1	0.0014145	0.0000000	0.0000000	0.0000000	0.0053447
AAMDC	0.3977947	0.2806093	0.1865901	0.4028650	0.2416807
ENSG00000254459	0.0147243	0.0118753	0.0035633	0.0060868	0.0138148
INTS4	0.0887993	0.0838158	0.0783549	0.0944766	0.2134784
KCTD14	0.0219167	0.0132922	0.0045587	0.0336067	0.0290731
ENSG00000254675	0.0017477	0.0000000	0.0027308	0.0000000	0.0044005
THRSP	0.0007339	0.0013531	0.0025702	0.0049343	0.0000000
NDUFC2	1.2618324	1.3033297	1.4124868	1.3502031	1.0199556
ALG8	0.1277942	0.1253488	0.1269983	0.2178688	0.1830344
KCTD21-AS1	0.0364404	0.0234589	0.0214820	0.0463016	0.0551411
KCTD21	0.0686318	0.0395824	0.0302347	0.0501224	0.0199311
USP35	0.0221451	0.0281092	0.0392214	0.0092683	0.0287864
GAB2	0.1573881	0.1985489	0.2168823	0.0974014	0.5058133
ENSG00000288538	0.0015146	0.0025951	0.0000000	0.0000000	0.0000000
ENSG00000288853	0.0000000	0.0000000	0.0000000	0.0000000	0.0081124
ENSG00000254420	0.0082371	0.0020914	0.0104469	0.0044641	0.1086246
LINC02728	0.0030826	0.0035867	0.0000000	0.0033357	0.0027080
NARS2	0.2016167	0.2091824	0.2426742	0.1495642	0.2904702
ENSG00000255084	0.0009398	0.0000000	0.0000000	0.0000000	0.0000000
TENM4	0.3547922	0.3485365	0.3376373	0.4283573	0.9671246
ENSG00000254563	0.0000000	0.0000000	0.0000000	0.0000000	0.0000000
ENSG00000255209	0.0000000	0.0020353	0.0000000	0.0000000	0.0000000
ENSG00000254434	0.0000000	0.0000000	0.0000000	0.0000000	0.0026927
LINC02720	0.0000000	0.0030174	0.0000000	0.0000000	0.0033746
ENSG00000287912	0.0010592	0.0009308	0.0000000	0.0052990	0.0091470
MIR4300HG	0.0037909	0.0045351	0.0000000	0.0000000	0.0140761
FAM181B	0.1479718	0.0965094	0.0639170	0.3027484	0.0531325
LINC02734	0.0010499	0.0000000	0.0000000	0.0000000	0.0066509
PRCP	0.3421706	0.2628976	0.2755556	0.5926977	0.2811593
DDIAS	0.0019645	0.0070226	0.0066662	0.0000000	0.0050816
RAB30	0.2215623	0.3464869	0.2720654	0.2318534	0.2822631
RAB30-DT	0.2019539	0.1957695	0.1908267	0.1978062	0.3314501
LINC02951	0.0075139	0.0021758	0.0010211	0.0074331	0.0000000
PCF11	0.3264677	0.2943405	0.2947037	0.2927992	0.3178849
ENSG00000254676	0.0000000	0.0000000	0.0000000	0.0000000	0.0015171
ANKRD42-DT	0.0399200	0.0270025	0.0326676	0.0309117	0.0688002
PCF11-AS1	0.0016909	0.0000000	0.0036911	0.0000000	0.0073601

ANKRD42	0.2057783	0.1380315	0.1707121	0.2545240	0.1291197
ENSG00000254551	0.0015309	0.0014612	0.0036287	0.0000000	0.0034576
CCDC90B	0.9634282	0.9446873	1.0474172	0.9734138	0.7573073
CCDC90B-AS1	0.0204917	0.0052375	0.0000000	0.0000000	0.0403574
DLG2	0.4143543	0.4817325	0.4557562	0.3253034	1.5469167
DLG2-AS2	0.0000000	0.0024393	0.0009860	0.0000000	0.0200626
ENSG00000254787	0.0071400	0.0062177	0.0032580	0.0000000	0.0447126
ENSG00000255555	0.0000000	0.0000000	0.0000000	0.0000000	0.0070703
TMEM126B	0.4598215	0.4134104	0.3582016	0.4506758	0.3830317
TMEM126A	0.3304600	0.3594804	0.3770973	0.3394765	0.2373237
CREBZF	0.1560933	0.1692960	0.1935681	0.1203314	0.2299799
CCDC89	0.0011449	0.0056275	0.0000000	0.0000000	0.0034730
SYTL2	0.0729582	0.0908983	0.0537727	0.0707699	0.0950054
CCDC83	0.0010751	0.0000000	0.0000000	0.0000000	0.0073662
ENSG00000255005	0.0000000	0.0000000	0.0000000	0.0000000	0.0019139
PICALM	0.6071434	0.6015967	0.5787432	0.5235117	0.7946557
EED	0.1669584	0.1453563	0.1211763	0.1623686	0.1443233
ENSG00000288809	0.0009083	0.0011164	0.0000000	0.0043051	0.0055208
HIKESHI	0.5135190	0.5159095	0.5510630	0.5229426	0.4550050
CCDC81	0.2688308	0.1343266	0.1192446	0.3205453	0.1137233
ENSG00000254733	0.0362691	0.0541613	0.0442733	0.0667975	0.1365522
ME3	0.1439504	0.1757820	0.2167794	0.1215838	0.2781569
ENSG00000254731	0.0043481	0.0000000	0.0000000	0.0047140	0.0000000
ENSG00000255250	0.0013175	0.0000000	0.0052547	0.0000000	0.0000000
PRSS23	1.1104417	0.6238260	0.4231206	1.8030879	0.6527307
ENSG00000269895	0.0000000	0.0000000	0.0000000	0.0000000	0.0000000
PRSS23-AS1	0.0860225	0.0588061	0.0825680	0.0943339	0.1096525
FZD4	0.0186914	0.0189065	0.0092711	0.0348400	0.0178879
FZD4-DT	0.0159782	0.0163963	0.0073713	0.0252278	0.0122739
TMEM135	0.0992020	0.1383243	0.0881263	0.1149652	0.3570759
ENSG00000285835	0.0000000	0.0014687	0.0000000	0.0025427	0.0031362
ENSG00000255102	0.0000000	0.0000000	0.0008430	0.0000000	0.0000000
RAB38	0.0026482	0.0016486	0.0012257	0.0038452	0.0067803
CTSC	0.0196248	0.0045355	0.0206161	0.0033765	0.0343619
ENSG00000288018	0.0046433	0.0044135	0.0088318	0.0027907	0.0000000
GRM5-AS1	0.0093268	0.0102910	0.0471941	0.0024463	0.0289944
GRM5	0.0881010	0.1403275	0.1998778	0.0486980	0.5146937
TYR	0.0000000	0.0010034	0.0000000	0.0000000	0.0000000
NOX4	0.0061430	0.0074690	0.0023930	0.0096573	0.0054687
NAALAD2	0.0541029	0.0453144	0.0514522	0.0476553	0.1983602
CHORDC1	0.5516911	0.4524964	0.4415811	0.5509548	0.4842143
DISC1FP1	0.0181000	0.0157085	0.0321200	0.0181419	0.1074116
FAT3	0.6493135	0.6510897	0.4963523	0.7697385	1.2460710
ENSG00000254705	0.0000000	0.0000000	0.0000000	0.0000000	0.0049154
ENSG00000255506	0.0000000	0.0022172	0.0000000	0.0000000	0.0108276
ENSG00000290774	0.0000000	0.0000000	0.0000000	0.0000000	0.0000000
MTNR1B	0.0027142	0.0000000	0.0019772	0.0000000	0.0000000
SLC36A4	0.3148015	0.3943730	0.4222162	0.2070969	0.4945102
DEUP1	0.0419967	0.0319665	0.0238245	0.0080846	0.0476719
SMCO4	0.1058602	0.1153277	0.0662044	0.1355652	0.1324850
CEP295	0.1273990	0.2319527	0.1773267	0.1913360	0.3736487
TAF1D	0.7113187	0.6862874	0.6036451	0.6146329	0.5982084
C11orf54	0.4528363	0.2788091	0.2663429	0.4723378	0.3733148

MED17	0.2464348	0.2172440	0.1887823	0.2361646	0.2807763
VSTM5	0.0088972	0.0305961	0.0369949	0.0083750	0.0444101
HEPHL1	0.0020147	0.0000000	0.0000000	0.0000000	0.0000000
PANX1	0.0897926	0.1293926	0.1647202	0.0776099	0.1108053
ENSG00000250519	0.0180307	0.0238780	0.0103245	0.0063453	0.0336288
GPR83	0.0141947	0.0306511	0.0603864	0.0119060	0.0646178
MRE11	0.1507480	0.0982370	0.0707828	0.1706392	0.1312712
ANKRD49	0.1676568	0.1418728	0.0712514	0.1220512	0.0768648
C11orf97	0.1192687	0.0732937	0.0462697	0.1188343	0.0402446
PIWIL4	0.0043669	0.0111242	0.0000000	0.0056435	0.0242416
FUT4	0.0117903	0.0060970	0.0095451	0.0156623	0.0098803
PIWIL4-AS1	0.0120296	0.0239882	0.0131803	0.0118677	0.0530197
AMOTL1	0.2431481	0.2184171	0.2308810	0.2463107	0.2343069
ENSG00000256469	0.0000000	0.0000000	0.0000000	0.0000000	0.0000000
CWC15	0.9466838	0.9056913	0.9008006	0.8515548	0.7377628
KDM4D	0.0624310	0.0351540	0.0808738	0.0410210	0.0701028
SRSF8	0.3790307	0.3971076	0.4760415	0.3440042	0.3504774
ENDOD1	0.1642443	0.2541968	0.2951554	0.2803613	0.2407527
LNCRNA-IUR	0.0323359	0.0122526	0.0119690	0.0378269	0.0484219
SESN3	0.8634164	0.7555266	0.6510068	0.8460489	0.8051327
ENSG00000285842	0.0000000	0.0000000	0.0000000	0.0000000	0.0049754
FAM76B	0.2139075	0.2703395	0.2664667	0.1880269	0.2111842
CEP57	0.6176201	0.6530191	0.7416947	0.5201857	0.6536928
MTMR2	0.4412594	0.4840230	0.5293245	0.3534315	0.4334804
MAML2	0.6264450	0.4711513	0.3641125	0.5432654	0.8562181
ENSG00000285921	0.0015146	0.0011596	0.0007553	0.0000000	0.0088565
CCDC82	0.5777745	0.5950753	0.6549920	0.6089767	0.6520826
JRKL	0.1901234	0.1669160	0.1472102	0.1841760	0.1688017
ENSG00000254587	0.0000000	0.0035945	0.0000000	0.0000000	0.0000000
CNTN5	0.1864856	0.2109302	0.1666958	0.1326867	0.9422081
ARHGAP42-AS1	0.0037469	0.0021720	0.0000000	0.0083077	0.0000000
ARHGAP42	0.1497503	0.0908765	0.0620175	0.2377033	0.0944109
TRPC6	0.0011320	0.0023516	0.0019944	0.0028015	0.0000000
ANGPTL5	0.0131670	0.0161113	0.0128274	0.0193145	0.0000000
CEP126	1.0638197	0.6717901	0.5174095	0.9496878	0.6780735
CFAP300	0.4353405	0.2726409	0.1646662	0.4166656	0.2437012
ENSG00000260008	0.0012063	0.0025876	0.0000000	0.0032553	0.0000000
ENSG00000277459	0.0173193	0.0112612	0.0102917	0.0159297	0.0156331
YAP1	0.7397840	0.4707280	0.2539064	0.7434235	0.4378013
ENSG00000254422	0.0144818	0.0070124	0.0000000	0.0055556	0.0034957
BIRC3	0.0018759	0.0062615	0.0088228	0.0013531	0.0083333
ENSG00000288833	0.0163625	0.0116188	0.0359348	0.0088958	0.0142926
BIRC2	0.5209314	0.4353726	0.4733739	0.4368315	0.3930978
TMEM123	0.6643222	0.4178801	0.2512806	0.6766290	0.4671672
TMEM123-DT	0.0141585	0.0174884	0.0023957	0.0159166	0.0116595
MMP10	0.0008461	0.0041690	0.0000000	0.0000000	0.0000000
DCUN1D5	0.5273267	0.6517233	0.6640714	0.5328127	0.5203235
DYNC2H1	0.5454419	0.4406967	0.3564869	0.4529602	0.6949423
ENSG00000285878	0.0041422	0.0012736	0.0000000	0.0000000	0.0209709
ENSG00000254987	0.0017762	0.0028602	0.0103312	0.0000000	0.0175879
PDGFD	0.1185019	0.0820398	0.0449691	0.1268388	0.1118156
LINC02552	0.0017767	0.0060176	0.0000000	0.0000000	0.0034872
CARD16	0.0070960	0.0053783	0.0000000	0.0044434	0.0063041

CARD18	0.0047215	0.0144796	0.0032579	0.0051762	0.0067125
ENSG00000289383	0.0000000	0.0000000	0.0000000	0.0000000	0.0000000
GRIA4	0.5008834	0.8720384	0.8566496	0.4088465	1.3865418
ENSG00000285813	0.0010832	0.0000000	0.0000000	0.0000000	0.0000000
MSANTD4	0.3678028	0.4552817	0.5350330	0.3646849	0.3259682
KBTBD3	0.1109578	0.1142711	0.0773026	0.1023943	0.1007931
AASDHPPT	1.0386873	1.2849904	1.5516294	1.0560412	0.9531703
ENSG00000254433	0.0028402	0.0019506	0.0067946	0.0000000	0.0030362
GUCY1A2	0.3644533	0.4607969	0.4925610	0.3249881	0.6493876
ENSG00000261098	0.0157381	0.0225329	0.0348700	0.0075263	0.0190936
CWF19L2	0.3764163	0.3794854	0.3621646	0.3040842	0.4529377
ALKBH8	0.0682039	0.0827816	0.0899763	0.0533180	0.1296737
ELMOD1	0.2861240	0.6161593	0.7617429	0.2721516	0.5689669
SLN	0.0013442	0.0044744	0.0000000	0.0036117	0.0000000
SLC35F2	0.0183372	0.0351941	0.0311998	0.0117956	0.0576575
RAB39A	0.0472306	0.1201471	0.0833759	0.0359896	0.0728824
ENSG00000288012	0.0281064	0.0164946	0.0148118	0.0205882	0.0000000
CUL5	0.6489432	0.5500321	0.6492612	0.6685426	0.6660419
ENSG00000255467	0.0671116	0.0600356	0.0978392	0.0806145	0.0631126
ACAT1	0.4703638	0.5636776	0.5507931	0.5068837	0.4206853
ENSG00000285696	0.0004419	0.0053321	0.0000000	0.0000000	0.0122052
NPAT	0.1751582	0.1613375	0.1897581	0.1187964	0.2356848
ATM	0.3080147	0.2534185	0.2040879	0.2677164	0.4802098
C11orf65	0.0577041	0.0417827	0.0205357	0.0453160	0.0614528
POGLUT3	0.0662631	0.0454622	0.0286619	0.1147690	0.0826718
EXPH5	0.0066107	0.0105613	0.0073243	0.0106388	0.0098557
DDX10	0.2164766	0.2049943	0.2013090	0.1861277	0.3765354
ENSG00000255028	0.0010438	0.0057252	0.0036781	0.0000000	0.0094381
C11orf87	0.0937416	0.1727291	0.2355150	0.0473685	0.1392869
LINC02715	0.0000000	0.0000000	0.0014107	0.0000000	0.0000000
RDX	1.7926937	1.5258471	1.4336977	1.9573145	1.4058119
ENSG00000287245	0.0106554	0.0077669	0.0078910	0.0035761	0.0041331
ZC3H12C	0.1873612	0.1703381	0.1208870	0.1679095	0.3256091
LINC02732	0.0027620	0.0000000	0.0000000	0.0018314	0.0028791
FDX1	0.6022253	0.4836623	0.4049268	0.6578519	0.4295814
ARHGAP20	0.1442621	0.2296301	0.2693964	0.1513577	0.2728698
LINC02550	0.0002867	0.0006718	0.0000000	0.0000000	0.0000000
COLCA1	0.0287546	0.0088030	0.0080222	0.0223665	0.0052886
POU2AF3	0.0418457	0.0088452	0.0181211	0.0309454	0.0149862
POU2AF1	0.0038515	0.0010823	0.0000000	0.0000000	0.0000000
BTG4	0.0033257	0.0013222	0.0018260	0.0021759	0.0000000
MIR34BHG	0.0049643	0.0044499	0.0000000	0.0056195	0.0000000
HOATZ	0.3793535	0.1982917	0.1297076	0.4118389	0.1878836
LAYN	0.0467153	0.0471877	0.0392190	0.0416167	0.0529309
SIK2	0.2276053	0.1802305	0.1775729	0.1715273	0.4513086
PPP2R1B	0.0725190	0.0593446	0.0418721	0.0712893	0.0623179
ENSG00000254990	0.0025244	0.0012831	0.0000000	0.0000000	0.0199807
ALG9	0.1249097	0.1259216	0.1091339	0.1300828	0.1595087
ALG9-IT1	0.0000000	0.0000000	0.0000000	0.0000000	0.0000000
FDXACB1	0.0270286	0.0176017	0.0220263	0.0165398	0.0000000
CFAP68	0.2298397	0.2085645	0.1969219	0.1642717	0.1622579
CRYAB	0.7229137	0.4895005	0.3108890	0.7006140	0.5102068
HSPB2	0.0000000	0.0000000	0.0000000	0.0000000	0.0000000

C11orf52	0.0000000	0.0013712	0.0022314	0.0000000	0.0053082
DIXDC1	0.4512476	0.4870625	0.4598451	0.3799147	0.4736410
DLAT	0.2395422	0.2915257	0.2742403	0.2719078	0.2602767
PIH1D2	0.2435135	0.1085861	0.0643484	0.2740796	0.1020820
NKAPD1	0.4669136	0.4128735	0.4054218	0.4920825	0.3181366
TIMM8B	0.8365393	0.8436897	0.8678294	0.8656294	0.6038074
SDHD	0.4244468	0.2919697	0.2529326	0.3993222	0.2458271
IL18	0.1229650	0.0592334	0.0702491	0.1034395	0.0467805
TEX12	0.0014741	0.0000000	0.0027218	0.0000000	0.0000000
BCO2	0.0653600	0.0772805	0.0877291	0.0773319	0.1421476
PTS	0.6858799	0.7849272	0.9488105	0.7124547	0.7282715
PLET1	0.0005482	0.0014479	0.0032471	0.0000000	0.0217622
ENSG00000268472	0.0010438	0.0000000	0.0000000	0.0000000	0.0035216
LINC02762	0.4855755	0.4339148	0.4260150	0.4161558	0.3798750
LINC02763	0.0101473	0.0075877	0.0055301	0.0127190	0.0183667
LINC02764	0.0009689	0.0021895	0.0019437	0.0014220	0.0000000
ENSG00000285769	0.0023730	0.0091092	0.0050379	0.0016235	0.0064393
ENSG00000288070	0.0018030	0.0018332	0.0000000	0.0052990	0.0000000
ENSG00000247416	0.0307019	0.0278226	0.0213440	0.0481640	0.0364123
NCAM1	1.7058181	1.6804467	1.8561961	1.9763670	2.2260257
NCAM1-AS1	0.0037265	0.0049331	0.0103154	0.0041513	0.0275566
TTC12-DT	0.0170653	0.0059292	0.0105952	0.0200388	0.0181101
TTC12	0.1346941	0.0904476	0.0548931	0.1500969	0.0668498
ENSG00000270179	0.0113590	0.0057091	0.0046232	0.0118143	0.0044975
ANKK1	0.0040479	0.0091419	0.0008981	0.0019922	0.0030223
DRD2	0.0326226	0.0723065	0.0431573	0.0164890	0.0859890
TMPRSS5	0.0032966	0.0053716	0.0063146	0.0011493	0.0043847
ZW10	0.0670285	0.0705893	0.0613140	0.0862561	0.0844444
USP28	0.1420171	0.1039159	0.0457018	0.1032209	0.1460869
ENSG00000256452	0.0000000	0.0000000	0.0000000	0.0000000	0.0050876
HTR3B	0.0016207	0.0024832	0.0000000	0.0000000	0.0161889
HTR3A	0.0047783	0.0221376	0.0069025	0.0128213	0.0132182
ZBTB16	0.0321435	0.0854923	0.1239807	0.0412854	0.1851392
ENSG00000256947	0.0222582	0.0090364	0.0052070	0.0069390	0.0087672
NNMT	0.1129260	0.0760801	0.0444149	0.0584446	0.0301006
ENSG00000256195	0.0010800	0.0000000	0.0000000	0.0000000	0.0035288
C11orf71	0.2317787	0.1479670	0.1690239	0.1932475	0.1297572
RBM7	0.5285472	0.3144110	0.2103019	0.5331506	0.3224030
REXO2	0.8754613	0.6498654	0.4439791	0.8873126	0.4934042
NXPE1	0.0011992	0.0000000	0.0014078	0.0000000	0.0085733
NXPE2	0.0083658	0.0082772	0.0000000	0.0011506	0.0038198
CADM1	0.8314806	1.0820521	1.0987493	0.9025067	1.6910069
ENSG00000256972	0.0000000	0.0000000	0.0000000	0.0000000	0.0038113
ENSG00000255580	0.0031790	0.0056165	0.0042023	0.0035260	0.1093136
CADM1-AS1	0.0000000	0.0000000	0.0000000	0.0000000	0.0000000
LINC02698	0.0020422	0.0019728	0.0044555	0.0058316	0.0000000
LINC00900	0.0231889	0.0348445	0.0199462	0.0341230	0.0000000
LINC02151	0.0009887	0.0022004	0.0042726	0.0000000	0.0000000
BUD13	0.1313977	0.1246519	0.1369698	0.1446583	0.1255903
ZPR1	0.2270024	0.2812607	0.3056690	0.2020035	0.2221054
APOA1	0.0197997	0.0107205	0.0304174	0.0154572	0.0042206
APOA1-AS	0.0014799	0.0000000	0.0000000	0.0021565	0.0044857
SIK3	0.3639380	0.4689094	0.4553023	0.2555604	1.0660177

ENSG00000224077	0.0000000	0.0015004	0.0000000	0.0000000	0.0000000
PAFAH1B2	0.6377340	0.7079444	0.7898922	0.5895388	0.7110907
SIDT2	0.0952430	0.0716193	0.0546187	0.1062796	0.1210992
TAGLN	0.0220771	0.0186322	0.0494946	0.0183314	0.0466209
PCSK7	0.0534893	0.0614898	0.0626597	0.0693640	0.1811246
RNF214	0.4188287	0.3701971	0.4255583	0.3723099	0.2830998
BACE1	0.3975869	0.3812801	0.3578946	0.4107760	0.4106179
BACE1-AS	0.0566897	0.0525995	0.0451643	0.0437077	0.0606368
ENSG00000276505	0.0038771	0.0020908	0.0000000	0.0021653	0.0039411
CEP164	0.1531506	0.1607792	0.1458996	0.1424163	0.3404075
ENSG00000250699	0.0072723	0.0012489	0.0009891	0.0034122	0.0258079
DSCAML1	0.0384354	0.0263538	0.0253119	0.0397918	0.3652413
ENSG00000270403	0.0028940	0.0000000	0.0000000	0.0023840	0.0000000
FXYD2	0.0049590	0.0073791	0.0126347	0.0047682	0.0185328
FXYD6-AS1	0.0076347	0.0098683	0.0195439	0.0026542	0.0201451
FXYD6	0.8029971	1.0587069	1.4348809	0.7404341	0.8586492
TMPRSS13	0.0009343	0.0000000	0.0000000	0.0000000	0.0000000
IL10RA	0.0053472	0.0109939	0.0241798	0.0097283	0.0241855
TMPRSS4	0.0000000	0.0000000	0.0000000	0.0000000	0.0000000
SCN4B	0.0046188	0.0200726	0.0103043	0.0041852	0.0114050
SCN2B	0.0147436	0.0472299	0.0474765	0.0188400	0.0415541
MPZL3	0.0081975	0.0012821	0.0067323	0.0000000	0.0148060
UBE4A	0.2831472	0.2694905	0.2549271	0.3123425	0.2672649
ENSG00000254873	0.0009834	0.0032434	0.0042844	0.0000000	0.0102687
ATP5MG	1.7518476	1.8495959	1.8837877	1.7604072	1.5510035
ENSG00000255384	0.0035896	0.0011747	0.0036534	0.0000000	0.0000000
KMT2A	0.7301511	0.8535783	0.8837103	0.6996323	1.1417573
TTC36-AS1	0.0508231	0.0379195	0.0275145	0.0251235	0.0442134
TTC36	0.0043847	0.0117276	0.0088032	0.0064113	0.0050546
TMEM25	0.2164192	0.2171109	0.1737813	0.2729213	0.1916629
IFT46	0.3321231	0.2328406	0.1534276	0.3152275	0.2289381
ARCN1	0.7958794	0.6869292	0.8023757	0.8145557	0.5825883
PHLDB1	0.0566448	0.0635086	0.0679076	0.0865192	0.1385248
ENSG00000255176	0.0000000	0.0000000	0.0000000	0.0000000	0.0000000
TREH	0.0000000	0.0027815	0.0056075	0.0000000	0.0000000
ENSG00000255422	0.0013952	0.0000000	0.0000000	0.0000000	0.0000000
ENSG00000287238	0.0000000	0.0007495	0.0000000	0.0000000	0.0000000
DDX6	1.3647050	1.2237808	1.2810486	1.2286035	1.1461819
ENSG00000278376	0.0556703	0.0361148	0.0412497	0.0396484	0.0596962
CXCR5	0.0033035	0.0010911	0.0039201	0.0088185	0.0000000
BCL9L	0.1567212	0.1585729	0.2039079	0.1750643	0.1493878
UPK2	0.0472094	0.0591488	0.0466714	0.0351671	0.0257730
FOXR1	0.0011983	0.0012917	0.0066829	0.0000000	0.0000000
CENATAC-DT	0.0069146	0.0124767	0.0110809	0.0167314	0.0204977
CENATAC	0.0687441	0.0831032	0.0848062	0.0740065	0.2105225
ENSG00000254428	0.0000000	0.0000000	0.0010540	0.0000000	0.0000000
RPS25	2.5469022	2.4527476	2.3094399	2.3548250	2.1986789
TRAPPC4	0.5981343	0.6974829	0.7027778	0.6225817	0.4742822
SLC37A4	0.0541233	0.0297601	0.0271957	0.0796608	0.0570397
ENSG00000255114	0.0023969	0.0044159	0.0061689	0.0076664	0.0030127
HYOU1	0.4629877	0.3956319	0.4411403	0.6204788	0.4197389
ENSG00000271751	0.0003183	0.0042476	0.0068521	0.0000000	0.0000000
VPS11-DT	0.0000000	0.0010652	0.0051996	0.0000000	0.0000000

VPS11	0.0946410	0.0759909	0.0606580	0.0855053	0.0639194
HMBS	0.0886482	0.0572626	0.0628397	0.0985561	0.0792623
H2AX	0.3727282	0.3039194	0.2312015	0.3927312	0.2334949
DPAGT1	0.1060384	0.0958277	0.0955864	0.1547882	0.0574252
ENSG00000289124	0.0017260	0.0005904	0.0000000	0.0000000	0.0000000
C2CD2L	0.0460002	0.0639221	0.0842201	0.0402489	0.0688434
HINFP	0.0587008	0.0688665	0.0570637	0.0506609	0.0842050
ABCG4	0.0100557	0.0215167	0.0312648	0.0078383	0.0117926
NLRX1	0.0313304	0.0198906	0.0110578	0.0206113	0.0148718
NHERF4	0.0000000	0.0000000	0.0000000	0.0000000	0.0040644
CCDC153	0.0550492	0.0188582	0.0118690	0.0526643	0.0128242
CBL	0.2758070	0.3390225	0.3234911	0.3031507	0.4238240
MCAM	0.1070662	0.1397771	0.0743090	0.1757708	0.0957616
RNF26	0.0851808	0.0707937	0.0924908	0.1176349	0.1066651
USP2	0.1235471	0.0536052	0.0260462	0.1612005	0.0282729
USP2-AS1	0.0777100	0.0530814	0.0432601	0.1284671	0.0466706
THY1	0.2372987	0.5334824	0.5938987	0.2368019	0.3518895
THY1-AS1	0.0017863	0.0060954	0.0000000	0.0000000	0.0085538
NECTIN1	0.0974017	0.1586005	0.2052126	0.0774277	0.2123096
NECTIN1-AS1	0.0000000	0.0000000	0.0000000	0.0000000	0.0016664
NECTIN1-DT	0.0000000	0.0000000	0.0000000	0.0000000	0.0000000
ENSG00000287545	0.0000000	0.0014627	0.0000000	0.0000000	0.0000000
TRIM29	0.0056255	0.0036613	0.0078920	0.0150602	0.0084120
OAF	0.0137516	0.0135503	0.0045218	0.0251496	0.0069799
TLCD5	0.1331013	0.0920754	0.1023777	0.1284326	0.0671583
ARHGEF12	0.5536541	0.6301401	0.7198217	0.4895920	0.8233938
GRIK4	0.2149738	0.1649312	0.1279320	0.2437532	0.2797307
ENSG00000250493	0.0000000	0.0019311	0.0000000	0.0000000	0.0105207
TBCEL	0.1357708	0.1445916	0.1165763	0.1129104	0.1428542
TECTA	0.0138027	0.0250826	0.0080123	0.0124841	0.0545475
SC5D	0.4861415	0.4604832	0.5406648	0.6421135	0.4538841
SORL1	0.0803714	0.1206053	0.0953185	0.1072126	0.2071698
ENSG00000286044	0.0032063	0.0020560	0.0038173	0.0077029	0.0173719
ENSG00000286023	0.0019050	0.0005872	0.0000000	0.0000000	0.0000000
MIR100HG	0.8116993	0.8584124	0.8387816	0.9083721	0.9852917
BLID	0.0000000	0.0000000	0.0000000	0.0000000	0.0000000
ENSG00000255219	0.0015383	0.0017157	0.0029992	0.0035412	0.0155154
UBASH3B	0.3864601	0.3052637	0.2397334	0.3395010	0.4227369
ENSG00000286341	0.0010125	0.0026504	0.0000000	0.0000000	0.0013692
ENSG00000285909	0.0007228	0.0022382	0.0000000	0.0088753	0.0197932
CRTAM	0.0005241	0.0019249	0.0000000	0.0000000	0.0028694
JHY	0.4483637	0.3367566	0.2065743	0.4628839	0.3364316
HSPA8	1.9091524	1.9892793	2.2017225	2.1006660	1.6599702
ENSG00000288061	0.1187954	0.0909726	0.0441704	0.1132672	0.1337882
CLMP	0.0274928	0.0472493	0.0826640	0.0081311	0.0405500
ENSG00000254710	0.0000000	0.0000000	0.0000000	0.0000000	0.0000000
GRAMD1B	0.1180271	0.2033226	0.2121736	0.1303334	0.3203870
SCN3B	0.2449779	0.4740675	0.6783209	0.2166061	0.5932731
ENSG00000254467	0.0000000	0.0021648	0.0094125	0.0047943	0.0000000
ZNF202	0.0246932	0.0267158	0.0394538	0.0489805	0.0342130
VWA5A	0.0960060	0.0945415	0.0979760	0.0702327	0.1311177
TBRG1	0.3549398	0.4781405	0.5686274	0.3133519	0.3627808
SIAE	0.1543806	0.1342198	0.1254405	0.2842898	0.0969698

SPA17	0.5330530	0.3940997	0.2539081	0.6228696	0.3120378
NRGN	0.0071434	0.0092947	0.0021781	0.0000000	0.0182118
ENSG00000255045	0.0000000	0.0000000	0.0000000	0.0000000	0.0000000
ESAM	0.0002969	0.0005695	0.0000000	0.0000000	0.0000000
ESAM-AS1	0.0000000	0.0000000	0.0015905	0.0027655	0.0081794
MSANTD2	0.0905360	0.1267301	0.1330430	0.1088664	0.1713823
ENSG00000279342	0.0024286	0.0061725	0.0103541	0.0000000	0.0022372
MSANTD2-AS1	0.1489058	0.1039097	0.0754467	0.1271932	0.0558317
ROBO3	0.1070442	0.0693295	0.0420740	0.1256370	0.0451760
ROBO4	0.0000000	0.0000000	0.0000000	0.0000000	0.0000000
HEPACAM	0.0685654	0.0448801	0.0067739	0.2108439	0.0571792
HEPN1	0.0353534	0.0248065	0.0160592	0.1271018	0.0559395
CCDC15-DT	0.0361112	0.0175067	0.0221214	0.0357487	0.0176423
CCDC15	0.0574866	0.0396287	0.0383735	0.0461365	0.0550187
SLC37A2	0.0000000	0.0019460	0.0010545	0.0000000	0.0091782
TMEM218	0.2261043	0.1734506	0.1250494	0.2719998	0.1296589
ENSG00000289631	0.0059384	0.0126633	0.0024485	0.0019957	0.0168188
PKNOX2	0.1031842	0.0589599	0.0384654	0.0953492	0.1971398
ENSG00000254932	0.0000000	0.0000000	0.0000000	0.0000000	0.0046690
FEZ1	0.8272136	1.0288387	1.1704070	0.9435727	0.7820555
ENSG00000255537	0.0019164	0.0047643	0.0013213	0.0049320	0.0289298
EI24	0.5797184	0.6320499	0.7896027	0.5886829	0.4941103
STT3A	0.3366658	0.2121819	0.2201954	0.4767201	0.2031643
ENSG00000288907	0.0870191	0.0551761	0.0259208	0.0550666	0.0392219
CHEK1	0.0750533	0.0697258	0.0642780	0.0517575	0.0748134
ACRV1	0.0018677	0.0000000	0.0000000	0.0061122	0.0000000
PATE2	0.0000000	0.0015753	0.0000000	0.0000000	0.0000000
HYLS1	0.0382113	0.0603002	0.0766571	0.0292624	0.0590211
PUS3	0.0800132	0.0881252	0.0801514	0.0802704	0.0954845
ENSG00000255027	0.0028216	0.0079353	0.0000000	0.0000000	0.0468011
DDX25	0.1163079	0.2064939	0.3328690	0.0880453	0.1747638
ENSG00000254790	0.0000000	0.0000000	0.0000000	0.0000000	0.0000000
VSIG10L2	0.0000000	0.0000000	0.0000000	0.0000000	0.0056364
CDON	0.1642142	0.1447745	0.1219415	0.1325074	0.3764198
ENSG00000254833	0.0000000	0.0000000	0.0000000	0.0000000	0.0000000
RPUSD4	0.0804213	0.0842202	0.0450509	0.0651414	0.0745310
ENSG00000254694	0.0000000	0.0000000	0.0000000	0.0000000	0.0000000
FAM118B	0.0977051	0.0932062	0.0444799	0.1668916	0.0853389
SRPRA	0.4708585	0.4263296	0.4296843	0.5932201	0.3343793
FOXRED1	0.0567896	0.0859143	0.0645507	0.0940933	0.0776142
TIRAP	0.0546858	0.0348087	0.0654623	0.0596711	0.0363254
TIRAP-AS1	0.0134500	0.0161851	0.0138557	0.0183801	0.0348871
DCPS	0.0977783	0.0781420	0.0488226	0.0963865	0.0766271
GSEC	0.0224406	0.0265685	0.0235069	0.0285028	0.0356461
ST3GAL4	0.0875408	0.1041464	0.1034943	0.1137652	0.1398142
KIRREL3	0.1462393	0.1870107	0.1047819	0.1029636	0.8378451
KIRREL3-AS1	0.0020261	0.0019078	0.0000000	0.0046498	0.0110556
ENSG00000254607	0.0010800	0.0029675	0.0070932	0.0000000	0.0144731
ENSG00000254938	0.0009083	0.0019533	0.0000000	0.0000000	0.0016028
KIRREL3-AS2	0.0000000	0.0000000	0.0000000	0.0000000	0.0000000
ENSG00000255317	0.0000000	0.0000000	0.0000000	0.0000000	0.0000000
ENSG00000255087	0.0000000	0.0013211	0.0027263	0.0000000	0.0000000
LINC02712	0.0000000	0.0016565	0.0015103	0.0000000	0.0000000

ETS1	0.0426652	0.0174516	0.0184890	0.0350312	0.0234455
FLI1	0.0000000	0.0000000	0.0000000	0.0000000	0.0000000
KCNJ5	0.0036078	0.0061101	0.0014931	0.0050089	0.0104304
TP53AIP1	0.0008048	0.0076085	0.0000000	0.0000000	0.0173859
ARHGAP32	0.2680232	0.3347438	0.3421462	0.1876453	0.7468388
BARX2	0.0046143	0.0034949	0.0012851	0.0033357	0.0000000
ENSG00000287426	0.0000000	0.0000000	0.0015669	0.0000000	0.0000000
TMEM45B	0.0020274	0.0000000	0.0000000	0.0000000	0.0030106
ENSG00000289223	0.0012107	0.0000000	0.0017865	0.0071301	0.0031778
NFRKB	0.1002528	0.0999886	0.0745225	0.0842565	0.0937098
PRDM10	0.0589572	0.0675180	0.0626764	0.0315170	0.0976112
PRDM10-DT	0.0016189	0.0035764	0.0000000	0.0000000	0.0000000
APLP2	1.5414109	1.3103550	1.4824514	2.0847282	1.3787277
ST14	0.0007532	0.0010877	0.0000000	0.0000000	0.0000000
ZBTB44	0.2160163	0.2813151	0.3051855	0.2088797	0.3234763
ZBTB44-DT	0.0071807	0.0032216	0.0043265	0.0046511	0.0066925
ADAMTS8	0.0038909	0.0027992	0.0057322	0.0000000	0.0059953
ADAMTS15	0.0418593	0.0245581	0.0171316	0.0684618	0.0420505
LINC02873	0.0000000	0.0010863	0.0000000	0.0000000	0.0000000
ENSG00000288013	0.0049853	0.0074527	0.0026514	0.0024744	0.0000000
LINC02551	0.0022084	0.0011346	0.0070787	0.0059293	0.0244038
SNX19	0.1609072	0.1638240	0.1700042	0.1485440	0.2248563
ENSG00000231698	0.0005026	0.0004663	0.0000000	0.0030106	0.0000000
NTM	0.5243439	0.7315604	1.2442205	0.4539010	1.6121027
ENSG00000237654	0.0003238	0.0000000	0.0000000	0.0024432	0.0337266
ENSG00000285980	0.0038682	0.0039800	0.0000000	0.0034569	0.0102167
NTM-AS1	0.0019683	0.0007434	0.0000000	0.0040727	0.0115903
ENSG00000238117	0.0046326	0.0013778	0.0052281	0.0000000	0.0246191
ENSG00000224700	0.0000000	0.0024909	0.0000000	0.0000000	0.0110405
OPCML	0.3107053	0.4478400	0.5590461	0.2570890	1.5212919
OPCML-IT2	0.0000000	0.0017291	0.0000000	0.0037420	0.0000000
OPCML-IT1	0.0008718	0.0013105	0.0000000	0.0000000	0.0000000
IGSF9B	0.1165581	0.1702991	0.1595940	0.1130881	0.3343387
LINC02730	0.0118199	0.0054507	0.0122088	0.0117128	0.0113043
LINC02731	0.0248405	0.0287932	0.0462629	0.0282026	0.0329685
JAM3	0.5234410	0.2982430	0.2332895	0.7035329	0.5649256
NCAPD3	0.1019001	0.0873574	0.1226075	0.0748393	0.1525877
ENSG00000255348	0.0000000	0.0000000	0.0000000	0.0000000	0.0000000
VPS26B	0.3557009	0.4966062	0.5370326	0.3807000	0.4163288
THYN1	0.5366558	0.5428706	0.4827401	0.6455419	0.4112834
ACAD8	0.1375445	0.1238774	0.1033316	0.1594700	0.1386693
GLB1L3	0.0047926	0.0022643	0.0083582	0.0069934	0.0035346
ENSG00000289399	0.0021173	0.0153590	0.0129722	0.0028496	0.0080096
GLB1L2	0.0153081	0.0118297	0.0079944	0.0309700	0.0096360
B3GAT1	0.0678873	0.0988248	0.1741033	0.0544364	0.1422138
B3GAT1-DT	0.0236332	0.0332246	0.0453969	0.0240276	0.2081087
ENSG00000254573	0.0008924	0.0000000	0.0000000	0.0000000	0.0000000
LINC02714	0.0000000	0.0000000	0.0000000	0.0000000	0.0000000
LINC02717	0.0000000	0.0019431	0.0030823	0.0000000	0.0000000
IQSEC3	0.0662341	0.1169503	0.1726109	0.0684837	0.2329474
ENSG00000256948	0.0000000	0.0000000	0.0000000	0.0000000	0.0000000
ENSG00000249695	0.0024689	0.0000000	0.0030777	0.0000000	0.0085651
IQSEC3-AS1	0.0010157	0.0000000	0.0000000	0.0000000	0.0000000

SLC6A12	0.0000000	0.0074796	0.0000000	0.0000000	0.0020838
SLC6A12-AS1	0.0008588	0.0000000	0.0000000	0.0000000	0.0000000
SLC6A13	0.0012581	0.0020329	0.0060735	0.0000000	0.0065095
ENSG00000261799	0.0423838	0.0489274	0.0336812	0.0412251	0.0609076
ENSG00000290285	0.0454817	0.0450707	0.0275579	0.0332339	0.0599814
KDM5A	0.5672622	0.5468952	0.4449355	0.5375171	0.7335591
CCDC77	0.0708053	0.0844261	0.0564654	0.1256392	0.0998632
B4GALNT3	0.0093513	0.0098022	0.0096513	0.0385911	0.0867060
NINJ2	0.0000000	0.0012812	0.0021368	0.0014265	0.0000000
NINJ2-AS1	0.1036242	0.0682193	0.0475821	0.0806262	0.0602374
WNK1	0.9464820	0.8576013	0.8311388	0.9826966	0.9498834
ENSG00000285704	0.0000000	0.0012072	0.0000000	0.0000000	0.0038113
RAD52	0.0562975	0.0799007	0.0508748	0.0456233	0.2139966
ENSG00000250132	0.0212464	0.0320439	0.0446464	0.0311643	0.0478937
ERC1	0.7380201	0.8137217	0.9624302	0.6543458	1.3225669
ENSG00000249028	0.0000000	0.0000000	0.0020357	0.0000000	0.0000000
WNT5B	0.1407076	0.1175195	0.0842992	0.1448198	0.2597451
FBXL14	0.0851446	0.0819588	0.0891658	0.0767794	0.0826501
ENSG00000289832	0.0016490	0.0000000	0.0000000	0.0000000	0.0000000
ADIPOR2	0.2658260	0.1886280	0.1959374	0.2249910	0.2852523
ENSG00000285627	0.0000000	0.0000000	0.0000000	0.0000000	0.0023834
CACNA2D4	0.0011892	0.0008196	0.0000000	0.0000000	0.0159910
ENSG00000287698	0.0023333	0.0039258	0.0058140	0.0000000	0.0000000
LRTM2	0.0000000	0.0025507	0.0021607	0.0000000	0.0000000
DCP1B	0.2028627	0.1501266	0.1317938	0.1505103	0.1969784
CACNA1C	0.1962464	0.2524308	0.1693490	0.1875642	1.4433884
ENSG00000203593	0.0000000	0.0057808	0.0000000	0.0063474	0.0000000
ENSG00000285555	0.0005541	0.0000000	0.0000000	0.0000000	0.0013481
ENSG00000285734	0.0000000	0.0000000	0.0000000	0.0000000	0.0079608
CACNA1C-AS2	0.0039485	0.0027227	0.0000000	0.0028137	0.0000000
CACNA1C-AS1	0.0003256	0.0012123	0.0000000	0.0000000	0.0000000
LINC02371	0.0000000	0.0014359	0.0016034	0.0000000	0.0000000
FKBP4	0.5935476	0.6182658	0.6624191	0.5593337	0.4103456
ITFG2-AS1	0.0230633	0.0149581	0.0155767	0.0053797	0.0215969
ITFG2	0.0725714	0.0731637	0.0701059	0.1126315	0.1402861
NRIP2	0.0012998	0.0021728	0.0000000	0.0000000	0.0000000
TEX52	0.0000000	0.0000000	0.0000000	0.0068570	0.0000000
FOXM1	0.0068007	0.0070459	0.0069500	0.0106777	0.0118307
RHNO1	0.3648430	0.3001675	0.3555174	0.3759814	0.2800322
TULP3	0.2145256	0.1270374	0.1008341	0.1813748	0.1559177
ENSG00000278356	0.0029941	0.0057451	0.0009118	0.0000000	0.0000000
TEAD4	0.0000000	0.0025942	0.0000000	0.0000000	0.0039051
TSPAN9	0.0699494	0.0684559	0.0936314	0.0925127	0.1801570
ENSG00000291189	0.0642420	0.0489675	0.1198923	0.0336638	0.1195092
ENSG00000287712	0.0010769	0.0000000	0.0013884	0.0000000	0.0022383
LINC02417	0.0025670	0.0029479	0.0030659	0.0000000	0.0218447
PRMT8	0.1185690	0.1270670	0.1094200	0.0628407	0.3004192
THCAT155	0.0518539	0.0839390	0.1073753	0.0378077	0.0594144
CRACR2A	0.0047833	0.0000000	0.0057018	0.0056748	0.0321680
PARP11	0.1482174	0.1162531	0.1519775	0.1020245	0.2132126
PARP11-AS1	0.0035873	0.0000000	0.0007371	0.0000000	0.0021475
CCND2-AS1	0.1158946	0.1009623	0.0882454	0.1321384	0.0907688
CCND2	1.6932706	1.5189280	1.3006027	1.7629377	1.3718337

ENSG00000290063	0.0000000	0.0014889	0.0000000	0.0000000	0.0071630
TIGAR	0.0947823	0.1031669	0.1233075	0.1562110	0.1656506
FGF23	0.0010029	0.0037851	0.0000000	0.0000000	0.0000000
C12orf4	0.1898391	0.1952097	0.1632392	0.1762295	0.1250179
RAD51AP1	0.0326534	0.0275940	0.0222982	0.0484346	0.0143635
DYRK4	0.5307138	0.4532374	0.4856485	0.4506937	0.4120841
AKAP3	0.0358987	0.0174780	0.0501932	0.0344437	0.0378737
NDUFA9	0.3228140	0.3609694	0.3136190	0.3178922	0.3459816
GAU1	0.0095921	0.0093554	0.0096096	0.0196511	0.0084237
GALNT8	0.0998506	0.0995955	0.0928932	0.1290463	0.2853046
ENSG00000256988	0.0003354	0.0054053	0.0011369	0.0000000	0.0081268
KCNA6	0.0147244	0.0204481	0.0099048	0.0183538	0.0197612
ENSG00000286866	0.0000000	0.0000000	0.0000000	0.0000000	0.0000000
ENSG00000256654	0.0000000	0.0056745	0.0057118	0.0036595	0.0460894
KCNA1	0.0323470	0.0949283	0.0236552	0.0143069	0.0619672
KCNA5	0.0125412	0.0158269	0.0157703	0.0167376	0.0187683
ENSG00000256417	0.0054394	0.0036255	0.0042355	0.0179057	0.0253716
NTF3	0.0075126	0.0149783	0.0018076	0.0098975	0.0234336
ANO2	0.0064815	0.0171368	0.0269624	0.0000000	0.0477492
VWF	0.0003641	0.0018051	0.0031267	0.0000000	0.0000000
CD9	1.0317802	0.6409368	0.4224561	1.6073826	0.5461295
PLEKHG6	0.0004169	0.0000000	0.0000000	0.0000000	0.0000000
TNFRSF1A	0.2719148	0.1579647	0.0799764	0.4581709	0.1297518
SCNN1A	0.0000000	0.0015363	0.0000000	0.0000000	0.0016673
LTBR	0.0076117	0.0029912	0.0000000	0.0081187	0.0068101
CD27-AS1	0.3033280	0.3125800	0.3399013	0.2937475	0.2718148
CD27	0.0000000	0.0005949	0.0017742	0.0000000	0.0000000
TAPBPL	0.0153426	0.0087475	0.0049425	0.0298392	0.0000000
VAMP1	0.1117135	0.2330738	0.2110119	0.1138382	0.1118668
MRPL51	0.8443902	0.8046424	0.7702919	0.8530676	0.6159404
NCAPD2	0.0664354	0.0712168	0.0314067	0.0631594	0.0823134
ENSG00000276232	0.0014488	0.0000000	0.0000000	0.0000000	0.0016281
GAPDH	3.6391673	3.5845127	3.4234708	3.5607330	3.2856718
ENSG00000269968	0.0459947	0.0502007	0.0506537	0.0546962	0.1039259
IFFO1	0.0349046	0.0315883	0.0407606	0.0488025	0.0369796
NOP2	0.0495918	0.0439809	0.0406610	0.0269771	0.0942854
CHD4	0.9974702	1.0864732	1.0217319	1.0478767	0.9705526
ENSG00000247853	0.0026982	0.0011149	0.0045769	0.0086659	0.0000000
LPAR5	0.0010639	0.0013745	0.0032093	0.0047102	0.0000000
ACRBP	0.0067913	0.0139912	0.0019983	0.0083347	0.0046212
ING4	0.3291675	0.2739462	0.2580709	0.3287730	0.2838933
ENSG00000219410	0.0000000	0.0058368	0.0016914	0.0104950	0.0000000
ZNF384	0.1111484	0.1315205	0.1231244	0.1211316	0.1316714
PIANP	0.1308433	0.2643524	0.3772455	0.1423310	0.2329375
ENSG00000288814	0.0020531	0.0063253	0.0091935	0.0160866	0.0041132
COPS7A	0.3270794	0.3149289	0.3145006	0.3214362	0.1802000
ENSG00000269892	0.0026047	0.0045462	0.0000000	0.0000000	0.0000000
MLF2	0.7475836	0.8696249	0.9738485	0.8398184	0.5981133
PTMS	1.3655337	1.6833758	1.9570575	1.4008972	1.4298097
LAG3	0.0028619	0.0065236	0.0051266	0.0000000	0.0038927
CD4	0.0018343	0.0019183	0.0000000	0.0000000	0.0092595
GPR162	0.0809013	0.1489436	0.2059778	0.1120360	0.1114376
P3H3	0.0664301	0.0323374	0.0429350	0.0941680	0.0599105

GNB3	0.0064336	0.0038431	0.0094771	0.0084590	0.0285925
CDCA3	0.0156360	0.0262007	0.0172065	0.0054349	0.0041410
USP5	0.2546906	0.2414629	0.2318643	0.2874580	0.2616973
TPI1	2.1428055	2.0928928	2.1754321	2.1522550	1.7127049
SPSB2	0.0771542	0.0518694	0.0400793	0.0839923	0.0058189
LRRC23	0.5106085	0.3646731	0.3102067	0.5156334	0.3946395
DSTNP2	0.0554094	0.0266929	0.0274228	0.0489067	0.0461590
ENO2	1.0718246	1.4311822	1.5983516	0.9395728	1.1352078
ATN1	0.3647499	0.4204469	0.4691989	0.3617996	0.4679116
C12orf57	0.9519965	1.1699956	1.1905141	0.9452376	0.9627377
ENSG00000272173	0.0082342	0.0219612	0.0219219	0.0037918	0.0069313
PTPN6	0.0082551	0.0159616	0.0036054	0.0283730	0.0258867
PHB2	0.7836529	0.8003387	0.7275919	0.7506733	0.5718242
EMG1	0.2209101	0.1512534	0.1537710	0.2293340	0.1252834
LPCAT3	0.0483854	0.0644745	0.0281221	0.0736474	0.0596576
C1S	0.0405996	0.0249006	0.0104929	0.0417162	0.0656268
C1R	0.0154719	0.0180781	0.0014063	0.0612589	0.0073576
C1RL	0.0213000	0.0171137	0.0116261	0.0289341	0.0160612
C1RL-AS1	0.0014569	0.0009298	0.0089034	0.0110944	0.0055417
RBP5	0.0041837	0.0039728	0.0030014	0.0105706	0.0030832
ENSG00000256967	0.0031188	0.0062461	0.0059535	0.0000000	0.0142296
CLSTN3	0.2489224	0.3185417	0.4551728	0.2694546	0.4305618
ENSG00000285770	0.0005311	0.0000000	0.0000000	0.0000000	0.0000000
ENSG00000255572	0.0002664	0.0000000	0.0000000	0.0000000	0.0000000
PEX5	0.1424012	0.1335601	0.1466762	0.1160276	0.1795624
CD163L1	0.0164332	0.0176214	0.0186334	0.0061753	0.0690027
CLEC4C	0.0017434	0.0054632	0.0022230	0.0000000	0.0000000
NANOG	0.0000000	0.0000000	0.0000000	0.0000000	0.0058922
SLC2A14	0.0022181	0.0000000	0.0010529	0.0000000	0.0018081
ENSG00000287713	0.0038130	0.0017999	0.0044590	0.0095974	0.0046563
SLC2A3	0.4915736	0.5477221	0.5314985	0.5706719	0.6269995
ENSG00000288043	0.0091232	0.0053241	0.0142447	0.0065439	0.0250870
FOXJ2	0.0541918	0.0610644	0.0790717	0.0310133	0.0484264
NECAP1	0.2396421	0.4625240	0.6127829	0.2307867	0.3293458
CLEC4A	0.0050935	0.0032364	0.0037505	0.0115686	0.0186693
FAM66C	0.0810617	0.0987375	0.0988288	0.0883904	0.2199751
ENSG00000290939	0.0018088	0.0032030	0.0121681	0.0037700	0.0015171
ENSG00000276417	0.0000000	0.0055883	0.0049979	0.0056716	0.0132574
ENSG00000275367	0.0089248	0.0318484	0.0115378	0.0239022	0.0941149
FAM90A1	0.0020973	0.0036677	0.0046475	0.0010179	0.0078605
ENSG00000290940	0.0027143	0.0022963	0.0012131	0.0033056	0.0029771
LINC02449	0.0113434	0.0154198	0.0185398	0.0089521	0.0080974
ENSG00000286895	0.0208993	0.0168533	0.0256583	0.0189234	0.0883380
ENSG00000286504	0.0000000	0.0006905	0.0000000	0.0000000	0.0060963
LINC00937	0.0261312	0.0456882	0.0530685	0.0273740	0.1034638
CLEC6A	0.0000000	0.0000000	0.0000000	0.0000000	0.0000000
MFAP5	0.0000000	0.0000000	0.0030777	0.0000000	0.0046563
RIMKLB	0.4042389	0.4634193	0.4472687	0.4066710	0.6618028
A2ML1-AS1	0.0194932	0.0346324	0.0184155	0.0210331	0.1528090
LINC02972	0.0093562	0.0048611	0.0000000	0.0090049	0.0120484
A2ML1	0.0055042	0.0006130	0.0023309	0.0087273	0.0133831
ENSG00000282022	0.0061120	0.0042801	0.0007240	0.0011520	0.0510994
PHC1	0.3037627	0.3112538	0.3421011	0.2367965	0.3357939

M6PR	0.5046130	0.3885013	0.2938491	0.6727149	0.2762025
KLRG1	0.0391511	0.0336661	0.0539805	0.0292062	0.0598880
LINC00612	0.0029571	0.0022784	0.0012131	0.0000000	0.0000000
A2M-AS1	0.0242682	0.0186173	0.0276840	0.0111167	0.0324657
A2M	0.0166842	0.0184303	0.0195445	0.0027381	0.0235719
PZP	0.0004731	0.0013272	0.0044786	0.0000000	0.0000000
A2MP1	0.0006736	0.0000000	0.0000000	0.0000000	0.0114328
LINC00987	0.0090187	0.0117372	0.0080541	0.0021296	0.0299353
ENSG00000291254	0.0000000	0.0000000	0.0000000	0.0000000	0.0022372
LINC02367	0.0018273	0.0017430	0.0019983	0.0000000	0.0048214
ENSG00000284634	0.0086875	0.0150772	0.0137635	0.0141967	0.0677760
ENSG00000290469	0.0033648	0.0033205	0.0038211	0.0019268	0.0139610
KLRB1	0.0000000	0.0000000	0.0000000	0.0000000	0.0057154
ENSG00000291107	0.0128000	0.0087531	0.0052981	0.0198965	0.0103199
ENSG00000257027	0.0040682	0.0062863	0.0063575	0.0149116	0.0106584
CLEC2D	0.0704593	0.0479446	0.0402590	0.0547391	0.0724629
LINC02470	0.0166968	0.0361744	0.0713075	0.0201965	0.0535852
CLEC1B	0.0000000	0.0000000	0.0000000	0.0000000	0.0000000
GABARAPL1	0.7735218	0.8889566	0.9355560	0.7330665	0.6420564
KLRD1	0.0115619	0.0113709	0.0088853	0.0154897	0.0129552
ENSG00000290036	0.0000000	0.0000000	0.0025247	0.0000000	0.0037464
KLRK1-AS1	0.0184720	0.0107611	0.0190890	0.0163484	0.0177446
KLRK1	0.0021023	0.0000000	0.0000000	0.0000000	0.0000000
EIF2S3B	0.0092244	0.0151987	0.0091200	0.0093888	0.0000000
LINC02446	0.0000000	0.0000000	0.0000000	0.0000000	0.0000000
ENSG00000290537	0.0111130	0.0159218	0.0057557	0.0130434	0.0276519
MAGOHB	0.1656237	0.1841070	0.1743050	0.1614603	0.1719426
STYK1	0.0077737	0.0175785	0.0292175	0.0000000	0.0296781
YBX3	1.3684049	1.0439382	0.7758847	1.3546177	0.9780511
TAS2R8	0.0000000	0.0000000	0.0010731	0.0000000	0.0000000
TAS2R9	0.0011775	0.0000000	0.0000000	0.0000000	0.0033094
PRH1	0.0694690	0.0838982	0.0704765	0.0817461	0.1445242
TAS2R10	0.0000000	0.0008736	0.0000000	0.0000000	0.0000000
PRR4	0.0010808	0.0035391	0.0000000	0.0000000	0.0055061
ENSG00000275778	0.0043853	0.0115695	0.0097198	0.0038847	0.0156584
TAS2R13	0.0000000	0.0000000	0.0000000	0.0000000	0.0055669
PRH2	0.0005471	0.0000000	0.0000000	0.0000000	0.0000000
TAS2R14	0.0445967	0.0469306	0.0195756	0.0422473	0.0563419
TAS2R20	0.0414993	0.0550304	0.0513083	0.0600287	0.0126356
TAS2R19	0.0017493	0.0011135	0.0000000	0.0000000	0.0000000
TAS2R31	0.0024822	0.0009313	0.0028465	0.0000000	0.0028666
ENSG00000291182	0.0043701	0.0000000	0.0019184	0.0000000	0.0211910
TAS2R43	0.0000000	0.0000000	0.0000000	0.0000000	0.0000000
TAS2R30	0.0008930	0.0023081	0.0000000	0.0000000	0.0227188
SMIM10L1	0.9301395	0.7891082	0.8638886	0.7979065	0.7049043
ENSG00000275119	0.0042053	0.0050161	0.0006307	0.0063752	0.0071013
PRB3	0.0000000	0.0019397	0.0000000	0.0000000	0.0023981
PRB2	0.0293299	0.1089339	0.1662478	0.0300592	0.0677856
LINC01252	0.0044047	0.0188186	0.0118989	0.0088456	0.0017206
ETV6	0.1383578	0.1130284	0.0611605	0.1559438	0.1700063
BCL2L14	0.0201118	0.0111502	0.0173267	0.0033824	0.0529522
LRP6	0.3768677	0.2765742	0.2644288	0.4510854	0.4869138
MANSC1	0.3747739	0.1791189	0.1366693	0.4832887	0.2190687

LOH12CR2	0.0781104	0.0758967	0.0448449	0.0681956	0.0575279
BORCS5	0.1906266	0.2388637	0.1796611	0.1541376	0.2486894
DUSP16	0.2650494	0.2963417	0.2265874	0.2753567	0.3643478
ENSG00000290048	0.0119327	0.0130013	0.0162110	0.0122461	0.0080778
ENSG00000255670	0.0116792	0.0100348	0.0122984	0.0175735	0.0241703
CREBL2	0.2492931	0.2461142	0.2571334	0.2109172	0.1630270
ENSG00000275963	0.0014794	0.0009982	0.0027908	0.0000000	0.0000000
GPR19	0.1284003	0.1090791	0.1167480	0.1142433	0.1445152
ENSG00000257004	0.0024619	0.0042700	0.0066681	0.0032755	0.0104252
CDKN1B	0.7456086	0.5525538	0.5099651	0.6981692	0.5003301
ENSG00000256658	0.0000000	0.0000000	0.0000000	0.0000000	0.0039740
APOLD1	0.0151450	0.0173323	0.0273999	0.0214037	0.0190774
DDX47	0.0055659	0.0097795	0.0000000	0.0058722	0.0037333
GPRC5A	0.0093920	0.0045179	0.0000000	0.0150835	0.0086425
GPRC5D-AS1	0.0324236	0.0304227	0.0397502	0.0322396	0.0562544
GPRC5D	0.0000000	0.0000000	0.0000000	0.0000000	0.0056738
HEBP1	0.5056304	0.3666943	0.3557943	0.5185882	0.2970618
ENSG00000255621	0.0125879	0.0122721	0.0105879	0.0027921	0.0386874
FAM234B	0.0498571	0.1037326	0.1467364	0.0873322	0.1449465
GSG1	0.0024338	0.0030206	0.0015338	0.0000000	0.0000000
EMP1	0.1308366	0.0615668	0.0360177	0.2856648	0.1181035
ENSG00000286869	0.0016733	0.0000000	0.0012851	0.0000000	0.0000000
GRIN2B	0.3694648	0.5735327	0.8142260	0.2686149	1.0337134
ENSG00000256306	0.0000000	0.0011753	0.0017129	0.0022251	0.0000000
ENSG00000287928	0.0026259	0.0012435	0.0000000	0.0000000	0.0070875
ATF7IP	0.7272665	0.7920866	0.7502714	0.6559916	0.8475843
ENSG00000290040	0.0014089	0.0014491	0.0000000	0.0054946	0.0061992
PLBD1	0.0229361	0.0123890	0.0072328	0.0442288	0.0220880
ENSG00000255649	0.0000000	0.0022294	0.0000000	0.0000000	0.0000000
PLBD1-AS1	0.0127659	0.0084683	0.0020307	0.0073739	0.0088405
GUCY2C	0.0010433	0.0000000	0.0031385	0.0000000	0.0000000
GUCY2C-AS1	0.0007131	0.0000000	0.0000000	0.0038691	0.0000000
ENSG00000261324	0.0081192	0.0170097	0.0092336	0.0019116	0.0174137
H4C16	0.0101358	0.0044173	0.0098380	0.0088821	0.0198496
ENSG00000289909	0.0006621	0.0027017	0.0000000	0.0014265	0.0042903
H2AJ	0.8157572	0.5574189	0.3979751	0.6784482	0.5396444
WBP11	0.8414711	0.8073443	0.8441494	0.8313440	0.6440347
C12orf60	0.0505031	0.0523117	0.0445480	0.0281474	0.0300812
SMCO3	0.0238365	0.0324943	0.0100107	0.0325080	0.0169698
ENSG00000286107	0.0005236	0.0000000	0.0064878	0.0000000	0.0118129
ART4	0.0000000	0.0000000	0.0019301	0.0000000	0.0000000
MGP	0.0236742	0.0315486	0.0264469	0.0491379	0.0604924
ARHGDIB	0.0000000	0.0000000	0.0000000	0.0000000	0.0018081
LINC01489	0.0010507	0.0000000	0.0007063	0.0000000	0.0000000
RERG	0.5855529	0.3572438	0.1914346	0.5216713	0.3549793
RERG-AS1	0.0000000	0.0000000	0.0000000	0.0000000	0.0000000
PTPRO	0.5353487	0.4695078	0.5232265	0.6274786	0.8218540
EPS8	0.2500051	0.2068483	0.2501028	0.3120730	0.3367238
STRAP	1.1993146	1.2009241	1.2426577	1.2113988	1.0404833
DERA	0.2776058	0.1461702	0.1008785	0.2772936	0.1457933
MGST1	0.1168468	0.0593433	0.0438415	0.2047417	0.0750672
LMO3	0.2846843	0.3137573	0.3771580	0.1994741	0.3262236
ENSG00000283240	0.0000000	0.0013102	0.0000000	0.0000000	0.0000000

ENSG00000256389	0.0010136	0.0000000	0.0000000	0.0000000	0.0075174
RERGL	0.0422455	0.0214947	0.0094502	0.0170464	0.0358053
PIK3C2G	0.0008480	0.0036312	0.0054122	0.0000000	0.0062544
PLCZ1	0.0033492	0.0038382	0.0086478	0.0014126	0.0000000
PLEKHA5	0.9512954	0.7451112	0.6789402	0.9299539	1.3045687
ENSG00000253284	0.0009337	0.0005310	0.0000000	0.0000000	0.0108880
AEBP2	0.5062873	0.4111182	0.3628580	0.4340292	0.8383673
ENSG00000255910	0.0469700	0.0460313	0.0362091	0.0344926	0.3021039
LINC02398	0.0196029	0.0234394	0.0146552	0.0245359	0.1536730
PDE3A-AS1	0.0181600	0.0059014	0.0062844	0.0117842	0.0000000
PDE3A	0.2321041	0.1480365	0.1124322	0.2652473	0.2672492
SLCO1C1	0.0098958	0.0064152	0.0024084	0.0000000	0.0147703
SLCO1B3	0.0000000	0.0008794	0.0000000	0.0000000	0.0000000
SLCO1B1	0.0000000	0.0000000	0.0000000	0.0000000	0.0046972
SLCO1A2	0.0032891	0.0051908	0.0000000	0.0000000	0.0146424
IAPP	0.0002117	0.0000000	0.0015355	0.0019429	0.0000000
PYROXD1	0.1818243	0.1631890	0.1585194	0.1815645	0.1430257
RECQL	0.1730943	0.1367408	0.1290631	0.1669969	0.1356075
GOLT1B	0.2114133	0.2538585	0.2729969	0.2404971	0.3051767
SPX	0.0072237	0.0036183	0.0020725	0.0030990	0.0038164
GYS2	0.0014422	0.0012708	0.0000000	0.0000000	0.0120715
LDHB	1.8989129	1.8987287	1.8341257	1.9559871	1.5179577
KCNJ8	0.0114540	0.0036644	0.0100530	0.0282442	0.0000000
ABCC9	0.0113208	0.0172481	0.0016528	0.0098857	0.0038421
CMAS	0.7052342	0.7171678	0.8391356	0.6617569	0.6036946
ST8SIA1	0.1506712	0.2615665	0.2708477	0.2286979	0.3764722
ENSG00000274624	0.0014576	0.0000000	0.0000000	0.0046498	0.0000000
C2CD5-AS1	0.0169759	0.0103152	0.0065913	0.0179795	0.0749901
C2CD5	0.2350621	0.2997326	0.3121153	0.1768002	0.5223956
ENSG00000256973	0.0006249	0.0000000	0.0000000	0.0130748	0.0020362
ETNK1-DT	0.0016186	0.0079585	0.0063995	0.0000000	0.0020430
ETNK1	0.2731729	0.4070395	0.4975187	0.3021168	0.5458691
LINC02955	0.0056563	0.0063808	0.0052473	0.0000000	0.0322298
SOX5	0.1407515	0.1510755	0.1284940	0.0828774	0.6410561
SOX5-AS1	0.0006010	0.0000000	0.0000000	0.0000000	0.0015171
BCAT1	0.5648474	0.7288843	0.8499539	0.4658183	0.7789400
IRAG2	0.0000000	0.0000000	0.0000000	0.0010179	0.0212098
ENSG00000258449	0.0022907	0.0000000	0.0000000	0.0071376	0.0035216
ENSG00000276842	0.0078006	0.0096294	0.0055910	0.0024077	0.0127118
DNAI7	0.2550984	0.1424627	0.1204756	0.2573987	0.1828425
ETFRF1	0.4362690	0.3420290	0.2949928	0.4846916	0.2743633
KRAS	0.5815130	0.7573932	0.9702293	0.6260522	0.5736174
ENSG00000274987	0.0027825	0.0000000	0.0035392	0.0000000	0.0000000
ENSG00000275197	0.0000000	0.0000000	0.0000000	0.0000000	0.0000000
ENSG00000278743	0.0068706	0.0135995	0.0096399	0.0000000	0.0226268
ENSG00000289928	0.0021307	0.0014241	0.0018707	0.0085918	0.0000000
LMNTD1	0.0365124	0.0160539	0.0104225	0.0521179	0.0365282
RASSF8-AS1	0.1568586	0.1594536	0.1023782	0.1767024	0.1605262
RASSF8	0.3087033	0.3270297	0.2525577	0.3158228	0.2928788
BHLHE41	0.3185211	0.1693499	0.0771888	0.3509947	0.2043195
SSPN	0.2204200	0.1212142	0.0786196	0.4304012	0.1030826
ENSG00000256894	0.0054366	0.0025878	0.0000000	0.0080837	0.0000000
ITPR2-AS1	0.0000000	0.0032650	0.0024211	0.0000000	0.0048432

ENSG00000255968	0.0002507	0.0029910	0.0000000	0.0000000	0.0000000
ITPR2	0.0986576	0.0851914	0.0403934	0.3664802	0.1196802
ENSG00000234428	0.0000000	0.0000000	0.0000000	0.0040025	0.0000000
INTS13	0.1465486	0.1499081	0.1317220	0.1634416	0.1408748
FGFR1OP2	0.4492679	0.4233162	0.4100404	0.3879594	0.3678215
TM7SF3	0.5153480	0.4288002	0.3172196	0.7109606	0.3661058
ENSG00000247903	0.0006516	0.0026910	0.0000000	0.0000000	0.0000000
MED21	0.3415883	0.3712939	0.4779001	0.3734871	0.2784892
ENSG00000275764	0.0252436	0.0391335	0.0551505	0.0223988	0.0452017
ENSG00000256226	0.0000000	0.0008158	0.0000000	0.0000000	0.0000000
STK38L	0.2626234	0.2540895	0.1731209	0.2844599	0.2985547
BMAL2	0.1207990	0.2015454	0.2502179	0.1005525	0.1796524
BMAL2-AS1	0.0023845	0.0020895	0.0062413	0.0050280	0.0216759
ENSG00000288971	0.0056367	0.0196321	0.0212225	0.0000000	0.0040262
SMCO2	0.0030874	0.0066452	0.0080275	0.0117808	0.0210798
PPFIBP1	0.2976522	0.3448301	0.2704707	0.2591307	0.4404529
MRPS35-DT	0.0018437	0.0000000	0.0019465	0.0023938	0.0000000
REP15	0.0009071	0.0072172	0.0170317	0.0059598	0.0125142
ENSG00000276261	0.0014863	0.0000000	0.0000000	0.0074556	0.0040262
ENSG00000256504	0.0000000	0.0000000	0.0016313	0.0000000	0.0000000
MRPS35	0.5961460	0.6184889	0.7104723	0.6117920	0.4502499
MANSC4	0.0038704	0.0051359	0.0011169	0.0000000	0.0074897
KLHL42	0.3567979	0.4935220	0.5637864	0.2975890	0.4846272
PTHLH	0.0101085	0.0046651	0.0051606	0.0139618	0.0147398
ENSG00000257042	0.0019139	0.0000000	0.0009265	0.0000000	0.0000000
CCDC91	0.5236685	0.5224043	0.4384882	0.4862280	0.7488751
ENSG00000247934	0.0186190	0.0243781	0.0244594	0.0175294	0.0191734
ENSG00000278733	0.0000000	0.0022904	0.0000000	0.0000000	0.0000000
ENSG00000273989	0.0011778	0.0022797	0.0017297	0.0103067	0.0189594
ENSG00000257258	0.0010043	0.0039564	0.0022732	0.0000000	0.0109626
FAR2	0.0919088	0.1621293	0.2276406	0.0661197	0.2529358
ENSG00000275476	0.0000000	0.0000000	0.0000000	0.0011244	0.0000000
ENSG00000257176	0.0094638	0.0092221	0.0066524	0.0020770	0.0175706
ENSG00000274315	0.0062275	0.0011874	0.0023944	0.0033167	0.0000000
ENSG00000273680	0.0008761	0.0015759	0.0019810	0.0000000	0.0089871
ERGIC2	0.4716094	0.4906440	0.5663764	0.5709805	0.4119530
OVCH1-AS1	0.0006793	0.0008173	0.0012370	0.0000000	0.0000000
OVCH1	0.0038388	0.0040182	0.0000000	0.0000000	0.0000000
TMTC1	0.1901683	0.1623132	0.1164477	0.1454896	0.2471638
ENSG00000257456	0.0000000	0.0000000	0.0000000	0.0000000	0.0000000
IPO8	0.1613666	0.1243088	0.1077616	0.1672911	0.1134208
ENSG00000285870	0.0000000	0.0000000	0.0000000	0.0000000	0.0000000
CAPRIN2	0.2660617	0.2790196	0.2256593	0.2140990	0.4250553
ENSG00000246331	0.0021447	0.0011714	0.0000000	0.0046032	0.0000000
ENSG00000285517	0.0028592	0.0105563	0.0084148	0.0041781	0.0060677
TSPAN11	0.0799352	0.0630966	0.0305686	0.1136123	0.0669018
ENSG00000290531	0.0064912	0.0135770	0.0024820	0.0027734	0.0147667
DDX11-AS1	0.0068601	0.0131430	0.0078835	0.0168388	0.0388757
DDX11	0.0527253	0.0623928	0.0472189	0.0543508	0.0667898
ENSG00000291250	0.1130556	0.0608390	0.0239287	0.1047064	0.0563858
ENSG00000275097	0.0012726	0.0000000	0.0015528	0.0000000	0.0000000
SINHCAF	0.5605206	0.3766003	0.3099269	0.5035807	0.3120205
FLJ13224	0.0000000	0.0000000	0.0000000	0.0000000	0.0000000

LINC02387	0.0007501	0.0017751	0.0000000	0.0000000	0.0000000
DENND5B	0.4832512	0.4286773	0.3947696	0.4083698	0.8086815
ENSG00000275769	0.0000000	0.0099580	0.0088089	0.0083838	0.0152454
DENND5B-AS1	0.0053239	0.0040858	0.0000000	0.0070140	0.0064448
AK4P3	0.0140735	0.0108936	0.0040629	0.0108574	0.0076217
ETFBKMT	0.0399424	0.0224924	0.0142646	0.0137552	0.0192825
AMN1	0.2975560	0.3604604	0.4271724	0.3005628	0.3599465
ENSG00000276900	0.0119594	0.0130107	0.0132111	0.0107257	0.0122527
LINC02422	0.0155315	0.0079397	0.0080041	0.0116221	0.0000000
RESF1	0.2738223	0.2784947	0.3069646	0.2457575	0.3344528
ENSG00000276136	0.0006704	0.0033947	0.0007467	0.0000000	0.0095407
BICD1-AS1	0.0010687	0.0028421	0.0000000	0.0000000	0.0000000
BICD1	0.6921353	0.7930254	0.8390803	0.6303789	1.1309093
ENSG00000277342	0.0031408	0.0000000	0.0043827	0.0000000	0.0022158
ENSG00000274964	0.0156318	0.0045652	0.0067252	0.0038334	0.0278196
ENSG00000276115	0.0098025	0.0067575	0.0017318	0.0088977	0.0382737
FGD4	0.2392798	0.3837836	0.4692242	0.1940899	0.4391445
DNM1L	0.5816659	0.7060245	0.7413286	0.5875526	0.7058917
ENSG00000276148	0.0023391	0.0019178	0.0034810	0.0050327	0.0129600
YARS2	0.1301601	0.1341843	0.1297593	0.1520620	0.1344979
ENSG00000275854	0.0018631	0.0118046	0.0109087	0.0037517	0.0083333
PKP2	0.0374109	0.0273059	0.0638639	0.0255843	0.0198166
SYT10	0.0375595	0.0289405	0.0504203	0.0239093	0.0510973
ENSG00000245482	0.0123052	0.0302724	0.0323685	0.0221541	0.0104688
ALG10	0.0431899	0.0589095	0.0814421	0.0653001	0.0318126
ENSG00000256538	0.0050731	0.0126426	0.0048652	0.0073653	0.0233473
ALG10B	0.1149119	0.0908100	0.1155505	0.1003795	0.1001917
ENSG00000258119	0.0006771	0.0008192	0.0000000	0.0000000	0.0000000
CPNE8	0.6023207	0.4151258	0.3381801	0.5856264	0.4494228
CPNE8-AS1	0.0504018	0.0408471	0.0315613	0.0628796	0.0247109
LINC02406	0.0158392	0.0045722	0.0076734	0.0057474	0.0161640
KIF21A	1.3715439	1.4832625	1.6385681	1.2643571	1.4235132
ABCD2	0.0447230	0.0403818	0.0516922	0.0316410	0.0600431
C12orf40	0.0059325	0.0000000	0.0000000	0.0023493	0.0257224
SLC2A13	0.2154271	0.2445399	0.2103932	0.2249776	0.4630830
LRRK2-DT	0.0120423	0.0083321	0.0062725	0.0030940	0.0000000
LRRK2	0.0947057	0.0601929	0.0297707	0.1425537	0.1004453
MUC19	0.0000000	0.0005933	0.0000000	0.0000000	0.0000000
CNTN1	0.4870695	0.7245618	1.0351317	0.3831354	1.2442013
ENSG00000274682	0.0007051	0.0085248	0.0044747	0.0000000	0.0020838
PDZRN4	0.0473870	0.0704520	0.1073266	0.0438234	0.2707674
ENSG00000257228	0.0000000	0.0019796	0.0000000	0.0000000	0.0228713
ENSG00000257239	0.0000000	0.0000000	0.0000000	0.0000000	0.0000000
ENSG00000286591	0.0055793	0.0000000	0.0030550	0.0090188	0.0108015
GXYLT1	0.1820242	0.1390607	0.1432978	0.1570526	0.1978492
YAF2	0.4511130	0.5067935	0.6057530	0.4165864	0.5822221
PPHLN1	0.4780236	0.5235334	0.5135479	0.4663167	0.5432996
ZCRB1	0.8961652	0.8309457	0.8626135	0.9655683	0.6897375
ENSG00000274943	0.0000000	0.0000000	0.0000000	0.0000000	0.0000000
PRICKLE1	0.2808970	0.2322101	0.2297139	0.1671927	0.4772170
ENSG00000257225	0.0045836	0.0088821	0.0041768	0.0050767	0.0085155
ENSG00000258068	0.0000000	0.0018728	0.0000000	0.0000000	0.0051180
ADAMTS20	0.0193042	0.0269351	0.0106684	0.0200159	0.1397341

PUS7L	0.3284268	0.3171182	0.3299717	0.2867327	0.4808892
ENSG00000291253	0.0000000	0.0000000	0.0000000	0.0000000	0.0000000
IRAK4	0.1113682	0.0785865	0.0517300	0.1249239	0.1199678
TWF1	0.6467228	0.6367378	0.6532171	0.7727111	0.4187921
TMEM117	0.0609713	0.0656694	0.0336655	0.0821407	0.3423870
ENSG00000275286	0.0000000	0.0004474	0.0041218	0.0000000	0.0000000
ENSG00000286671	0.0012073	0.0000000	0.0021512	0.0034487	0.0000000
NELL2	0.2056323	0.2796148	0.4558852	0.1860792	0.6854909
DBX2	0.0007681	0.0004414	0.0032193	0.0000000	0.0083476
DBX2-AS1	0.0000000	0.0000000	0.0017282	0.0000000	0.0033561
ANO6	0.2216885	0.2010105	0.1113568	0.2694865	0.4166876
ENSG00000257657	0.0094854	0.0021787	0.0069671	0.0041661	0.0236678
LINC00938	0.1673202	0.2254227	0.3186685	0.1820682	0.2081451
ARID2	0.3000985	0.2469264	0.2114706	0.1930009	0.4428319
SCAF11	0.8814897	0.6356243	0.5611796	0.8294297	0.6717198
ENSG00000289046	0.0036624	0.0082384	0.0072885	0.0042299	0.0000000
SLC38A1	0.5770071	0.7511982	1.0444113	0.5573090	0.9679387
ENSG00000274591	0.0000000	0.0011022	0.0000000	0.0000000	0.0000000
SLC38A2	0.3438057	0.3343796	0.3618940	0.3886042	0.5275748
SLC38A2-AS1	0.0000000	0.0000000	0.0031693	0.0019300	0.0000000
SLC38A4-AS1	0.0476095	0.0525879	0.0542329	0.0472521	0.1684327
ENSG00000257496	0.0015925	0.0000000	0.0022533	0.0029495	0.0204936
ENSG00000275481	0.0011118	0.0000000	0.0000000	0.0000000	0.0016664
ENSG00000272369	0.0054628	0.0000000	0.0000000	0.0030907	0.0000000
SLC38A4	0.0005207	0.0073048	0.0021138	0.0046461	0.0037844
ENSG00000274723	0.0000000	0.0017020	0.0045613	0.0059598	0.0000000
AMIGO2	0.0907905	0.0566395	0.0436044	0.1122922	0.0323800
PCED1B	0.0223942	0.0188523	0.0160636	0.0156757	0.0173110
ENSG00000257925	0.0019017	0.0000000	0.0000000	0.0000000	0.0000000
ENSG00000258181	0.0023491	0.0009982	0.0000000	0.0011298	0.0000000
LINC02416	0.0000000	0.0000000	0.0011062	0.0000000	0.0000000
LINC02156	0.0000000	0.0000000	0.0000000	0.0000000	0.0000000
RPAP3	0.5511822	0.4663842	0.5712168	0.5300080	0.4544249
ENSG00000276390	0.0098224	0.0076688	0.0042205	0.0062659	0.0000000
RPAP3-DT	0.0104263	0.0082137	0.0140942	0.0039774	0.0144957
RAPGEF3	0.0010444	0.0142297	0.0086204	0.0098282	0.0347249
SLC48A1	0.1428639	0.1056317	0.1607047	0.1538773	0.0938228
HDAC7	0.0699777	0.0549073	0.0366073	0.0651314	0.0810889
ENSG00000268069	0.0012376	0.0027658	0.0045530	0.0000000	0.0000000
VDR	0.0010001	0.0000000	0.0023834	0.0000000	0.0046920
ENSG00000278385	0.0011189	0.0031629	0.0102700	0.0000000	0.0000000
TMEM106C	0.5837932	0.4904343	0.5049600	0.6213564	0.4162506
COL2A1	0.0587344	0.0408938	0.0239161	0.1364821	0.0277395
SENP1	0.1322780	0.1385570	0.1556850	0.1522292	0.1976717
PFKM	0.3857439	0.3791329	0.4249473	0.4925071	0.2945603
ASB8	0.4236444	0.4391747	0.4754363	0.4345673	0.3499663
ENSG00000274124	0.0000000	0.0000000	0.0000000	0.0000000	0.0000000
CCDC184	0.3234686	0.6780410	0.8915997	0.2515222	0.4022198
ENSG00000269514	0.0048328	0.0071469	0.0048345	0.0000000	0.0277599
ENSG00000258234	0.0008620	0.0010722	0.0000000	0.0000000	0.0034957
ENSG00000275228	0.0031311	0.0017696	0.0000000	0.0000000	0.0000000
ZNF641	0.1278313	0.1190101	0.1605160	0.1175356	0.1200423
ENSG00000257735	0.0273557	0.0322410	0.0106300	0.0146916	0.0383944

ENSG00000273765	0.0043441	0.0077846	0.0104773	0.0154120	0.0045792
C12orf54	0.0014122	0.0034509	0.0000000	0.0000000	0.0045892
KANSL2	0.1330700	0.1330228	0.1408844	0.0828221	0.1617642
CCNT1	0.1554157	0.2087542	0.1820447	0.1915931	0.2314611
TEX49	0.0000000	0.0016865	0.0052547	0.0000000	0.0000000
ADCY6	0.1099319	0.0898918	0.0935635	0.1309747	0.1969523
ADCY6-DT	0.0260937	0.0281170	0.0253091	0.0495225	0.0037844
CACNB3	0.1204007	0.2272844	0.2404569	0.0973114	0.1468457
DDX23	0.1834293	0.1839010	0.1581933	0.1793323	0.1840895
RND1	0.0204051	0.0301097	0.0710448	0.0267687	0.0331510
CCDC65	0.2612868	0.1586789	0.1653264	0.3019818	0.1619398
FKBP11	0.0618890	0.0683278	0.0324439	0.1114833	0.0462326
ARF3	0.5250247	0.6869270	0.8507104	0.5425411	0.5285196
WNT10B	0.0077259	0.0137414	0.0116933	0.0223187	0.0112897
DDN	0.0037858	0.0024453	0.0026908	0.0000000	0.0000000
DDN-AS1	0.0035224	0.0098245	0.0016423	0.0132851	0.0207113
PRKAG1	0.3242881	0.2344096	0.1980171	0.3307164	0.1831528
KMT2D	0.0632109	0.1008515	0.1034711	0.0549754	0.1659859
RHEBL1	0.0108182	0.0123733	0.0156153	0.0152896	0.0095939
DHH	0.0013725	0.0016444	0.0000000	0.0039294	0.0164608
ENSG00000257346	0.0006402	0.0000000	0.0014518	0.0000000	0.0000000
LMBR1L	0.1119527	0.1330812	0.0850542	0.0530103	0.1957910
TUBA1B	2.4818253	2.8138119	2.9831999	2.4362435	2.3882005
TUBA1B-AS1	0.0817380	0.1165685	0.1731733	0.0713075	0.1641477
TUBA1A	3.3745782	3.7628884	4.1651564	3.5232081	3.3286355
TUBA1C	0.6087293	0.3948358	0.2522694	0.6454833	0.3655061
ENSG00000258101	0.0057361	0.0064169	0.0090601	0.0000000	0.0186838
ENSG00000258232	0.0014529	0.0000000	0.0000000	0.0000000	0.0049812
TROAP-AS1	0.0052379	0.0009343	0.0009597	0.0023540	0.0000000
PRPH	0.0363720	0.1077981	0.0516697	0.0473942	0.0616127
TROAP	0.0068510	0.0052207	0.0023827	0.0000000	0.0043097
C1QL4	0.0202962	0.0307019	0.0728010	0.0246934	0.0579295
DNAJC22	0.0205425	0.0133724	0.0168186	0.0150493	0.0023981
SPATS2	0.2802584	0.3905903	0.4273907	0.2348023	0.4524043
ENSG00000287537	0.0009343	0.0048137	0.0000000	0.0034364	0.0031524
KCNH3	0.0061689	0.0092210	0.0075606	0.0031700	0.0189476
MCRS1	0.2681007	0.2788408	0.2302824	0.3395861	0.2069440
PRPF40B	0.1818735	0.1791612	0.1502600	0.1653628	0.3094857
FAM186B	0.0022014	0.0017157	0.0030839	0.0033338	0.0051612
FMNL3	0.0358647	0.0313168	0.0191072	0.0167242	0.0799979
TMBIM6	1.5138753	1.2191678	1.1998887	2.0402758	1.1509120
NCKAP5L	0.0620429	0.1100373	0.0846848	0.0397827	0.1477807
BCDIN3D-AS1	0.0366214	0.0306331	0.0394836	0.0692550	0.0145345
BCDIN3D	0.1308343	0.1254616	0.0979774	0.1091194	0.1065860
FAIM2	0.1498414	0.2269221	0.3451101	0.1028682	0.2034660
LINC02395	0.0003484	0.0011769	0.0033181	0.0000000	0.0000000
AQP5-AS1	0.0008777	0.0060258	0.0022436	0.0125364	0.0000000
AQP5	0.0265856	0.0295689	0.0204302	0.0205153	0.0041014
AQP6	0.0047638	0.0008632	0.0000000	0.0093559	0.0119923
RACGAP1	0.0481629	0.0780103	0.0858244	0.0553569	0.0616321
ASIC1	0.2784140	0.2393428	0.3011160	0.3823950	0.2086882
SMARCD1	0.2584727	0.2805969	0.2839839	0.2503773	0.2443234
GPD1	0.0500379	0.0254967	0.0094151	0.0348404	0.0135071

COX14	0.5381851	0.5332337	0.5416399	0.5145353	0.3641259
ENSG00000272368	0.0427493	0.0327257	0.0175457	0.0454887	0.0685018
CERS5	0.1846503	0.1517686	0.1151270	0.1962584	0.3243304
LIMA1	0.2034000	0.1392385	0.0846048	0.2327925	0.2676878
ENSG00000257298	0.0009568	0.0000000	0.0000000	0.0000000	0.0000000
ENSG00000257256	0.0016544	0.0000000	0.0000000	0.0043405	0.0035158
FAM186A	0.0010015	0.0000000	0.0000000	0.0000000	0.0052515
LARP4	0.4550116	0.3566575	0.3540597	0.4289388	0.4017662
DIP2B	0.2534178	0.2461094	0.2635917	0.2445066	0.6129832
ATF1	0.2107163	0.1516269	0.1080751	0.1948244	0.2626848
TMPRSS12	0.0008333	0.0018251	0.0063621	0.0000000	0.0014245
METTL7A	0.0120343	0.0081514	0.0131308	0.0415669	0.0183247
ENSG00000274156	0.0006813	0.0007161	0.0000000	0.0000000	0.0044787
HIGD1C	0.0015920	0.0016811	0.0000000	0.0000000	0.0000000
SLC11A2	0.1038503	0.1096125	0.1058960	0.0937441	0.1896880
LETMD1	0.4502611	0.4804796	0.4387464	0.3837448	0.3489341
CSRNP2	0.1420088	0.2263671	0.2553005	0.1478062	0.1895018
TFCP2	0.2049193	0.2203488	0.2053481	0.1892845	0.3865205
POU6F1	0.0497152	0.0492859	0.0659987	0.0460275	0.0549867
ENSG00000278126	0.0170809	0.0162826	0.0131574	0.0032793	0.0189045
DAZAP2	0.6291718	0.4421604	0.4347221	0.6017795	0.3588835
SMAGP	0.0022518	0.0011200	0.0042925	0.0026054	0.0022372
BIN2	0.0000000	0.0000000	0.0000000	0.0000000	0.0033613
SLC4A8	0.1375349	0.2051730	0.3112591	0.1213999	0.3611529
SLC4A8-AS1	0.0000000	0.0019311	0.0000000	0.0000000	0.0000000
SCN8A	0.1231616	0.2238119	0.2102073	0.0822527	0.5383398
ENSG00000260122	0.0000000	0.0018256	0.0000000	0.0000000	0.0000000
ENSG00000260473	0.0015216	0.0030054	0.0000000	0.0000000	0.0071962
ENSG00000261586	0.0007232	0.0011428	0.0000000	0.0000000	0.0000000
FIGNL2	0.0007458	0.0009714	0.0016743	0.0019659	0.0214207
FIGNL2-DT	0.0000000	0.0031223	0.0000000	0.0000000	0.0000000
ANKRD33	0.0000000	0.0000000	0.0000000	0.0000000	0.0000000
ACVR1B	0.1931043	0.2942798	0.3487246	0.2093166	0.4830457
TAMALIN	0.0101941	0.0023900	0.0031076	0.0090265	0.0141793
NR4A1	0.0514596	0.0484976	0.0314920	0.0469275	0.0931328
NR4A1AS	0.0000000	0.0000000	0.0011015	0.0030582	0.0016028
ATG101	0.1780954	0.2362129	0.2271905	0.1808421	0.1309120
ENSG00000257663	0.0000000	0.0018332	0.0000000	0.0000000	0.0075295
SMIM41	0.0006020	0.0000000	0.0000000	0.0000000	0.0000000
KRT80	0.0077897	0.0077059	0.0057007	0.0000000	0.0074885
KRT86	0.0186463	0.0250697	0.0150564	0.0104525	0.0086588
KRT81	0.0039281	0.0010458	0.0000000	0.0000000	0.0000000
ENSG00000287051	0.0032610	0.0023378	0.0000000	0.0010179	0.0000000
ENSG00000257829	0.0042059	0.0018911	0.0035778	0.0000000	0.0052273
KRT4	0.0074601	0.0039235	0.0000000	0.0000000	0.0142444
KRT8	0.6945576	0.4180039	0.2800007	0.6966958	0.4199866
KRT18	0.3090357	0.1626990	0.1283741	0.2821479	0.1421898
EIF4B	1.5412322	1.5619427	1.3861235	1.4789309	1.2810916
ENSG00000257475	0.0000000	0.0014079	0.0000000	0.0000000	0.0052241
TNS2-AS1	0.1156857	0.0952320	0.0846317	0.1031197	0.0688485
TNS2	0.0220445	0.0199102	0.0131657	0.0280235	0.0250870
SPRYD3	0.2578348	0.2287807	0.2274888	0.1981530	0.1936503
IGFBP6	0.2806347	0.1880099	0.1400175	0.1864140	0.1401659

CSAD	0.0699760	0.0516472	0.0694101	0.0678255	0.1775701
ZNF740	0.1069618	0.1004597	0.1049170	0.0822528	0.0642800
ITGB7	0.0000000	0.0000000	0.0000000	0.0000000	0.0077780
RARG	0.0130294	0.0112502	0.0112168	0.0117272	0.0117769
MFSD5	0.0559011	0.0507744	0.0555223	0.0792443	0.0442673
ESPL1	0.0010552	0.0000000	0.0015646	0.0029570	0.0000000
PFDN5	2.0045324	1.9266537	1.8187511	1.8904868	1.7944765
MYG1-AS1	0.0061614	0.0061393	0.0011130	0.0000000	0.0043337
MYG1	0.3449950	0.3534645	0.3925931	0.3561781	0.3286623
AAAS	0.1819932	0.1812717	0.1298169	0.2208143	0.1890273
SP7	0.0012083	0.0000000	0.0000000	0.0000000	0.0000000
SP1	0.1345386	0.1094500	0.0886716	0.1280574	0.1327240
AMHR2	0.0046936	0.0014977	0.0346211	0.0000000	0.0038629
PRR13	0.5866101	0.4802713	0.3388195	0.5986779	0.3828654
PCBP2	1.2292107	1.2640952	1.1846624	1.0850196	1.1278618
PCBP2-OT1	0.0043345	0.0009720	0.0000000	0.0000000	0.0037811
MAP3K12	0.0996446	0.1214975	0.1200733	0.0660385	0.1359777
ENSG00000270175	0.0186648	0.0108952	0.0154188	0.0147381	0.0000000
TARBP2	0.1089432	0.1279866	0.1039683	0.1395027	0.0847546
NPFF	0.0032006	0.0031151	0.0023616	0.0103343	0.0000000
ATF7	0.2045760	0.1573282	0.1489311	0.2307126	0.2624788
ENSG00000257550	0.0037128	0.0009852	0.0050677	0.0130534	0.0000000
ENSG00000285692	0.0014591	0.0000000	0.0071146	0.0083569	0.0501527
ATP5MC2	1.6402733	1.5831601	1.3908233	1.5491836	1.3878117
CALCOCO1	0.1695818	0.1184139	0.1188900	0.1758279	0.1437609
ENSG00000286069	0.0009205	0.0087981	0.0029335	0.0000000	0.0234277
CISTR	0.0019776	0.0029242	0.0009561	0.0000000	0.0114670
ENSG00000260030	0.0000000	0.0000000	0.0000000	0.0000000	0.0000000
HOXC4	0.4133395	0.7085840	1.0487025	0.3113022	0.5447266
HOXC5	0.0147736	0.0229322	0.0299233	0.0088191	0.0429407
FLJ12825	0.0081726	0.0078048	0.0151675	0.0045090	0.0071904
ENSG00000291180	0.0479264	0.0567767	0.1083575	0.0464694	0.0486388
ENSG00000249388	0.0147215	0.0242602	0.0241213	0.0018419	0.0280984
FAM242C	0.0215496	0.0134856	0.0170605	0.0025857	0.0212780
SMUG1	0.1636396	0.1334955	0.1104167	0.1583584	0.1134599
LINC02381	0.5377743	0.6402083	0.9444982	0.4210222	0.4829526
ENSG00000248576	0.0000000	0.0000000	0.0000000	0.0000000	0.0000000
CBX5	1.3744539	1.5266317	1.7924851	1.4044374	1.3994215
SCAT2	0.0000000	0.0000000	0.0000000	0.0000000	0.0000000
ENSG00000258344	0.0065078	0.0025090	0.0000000	0.0000000	0.0182781
ENSG00000289154	0.0025799	0.0009070	0.0000000	0.0027681	0.0118529
HNRNPA1	1.9769517	2.0345056	2.0106460	1.7997038	1.7267597
NFE2	0.0009750	0.0020424	0.0000000	0.0043967	0.0000000
COPZ1	0.7848388	0.7473417	0.7399378	0.7956592	0.5483400
GPR84-AS1	0.0055761	0.0030816	0.0000000	0.0030247	0.0063744
ENSG00000289854	0.0021635	0.0028393	0.0000000	0.0052894	0.0097234
ZNF385A	0.0734690	0.0464518	0.0326096	0.1227456	0.0470985
ITGA5	0.0006533	0.0022286	0.0000000	0.0000000	0.0038284
GTSF1	0.0020362	0.0000000	0.0000000	0.0000000	0.0000000
NCKAP1L	0.0060151	0.0020052	0.0000000	0.0011055	0.0000000
PDE1B	0.0126687	0.0325774	0.0333458	0.0146896	0.0406741
PPP1R1A	0.7151019	1.1095866	1.0753536	0.6577125	0.8438478
NEUROD4	0.0000000	0.0000000	0.0038484	0.0000000	0.0000000

ENSG00000258763	0.0000000	0.0000000	0.0000000	0.0000000	0.0108293
ITGA7	0.0577522	0.0421372	0.0158325	0.0641845	0.0939455
BLOC1S1	0.7311968	0.6778831	0.5606474	0.7852526	0.4918216
RDH5	0.0211999	0.0249931	0.0113394	0.0193953	0.0096467
CD63	2.0046816	1.6617226	1.4920657	2.3626819	1.5055955
CD63-AS1	0.1472470	0.1125553	0.0711367	0.1461743	0.0539638
GDF11	0.0935278	0.1375470	0.2158208	0.1113965	0.1460213
SARNP	0.8388202	0.8640186	0.8734727	0.8559593	0.9283559
ORMDL2	0.1482296	0.1498126	0.1323559	0.2253018	0.1159169
DNAJC14	0.0900858	0.0966452	0.0997516	0.1142063	0.1024811
MMP19	0.0008713	0.0025198	0.0000000	0.0000000	0.0068895
PYM1	0.2160152	0.2529423	0.2313462	0.1913427	0.2237688
DGKA	0.0623712	0.0513876	0.0275004	0.0304604	0.0894827
PMEL	0.0031165	0.0039859	0.0098604	0.0000000	0.0218615
CDK2	0.0613786	0.0272298	0.0300910	0.0621913	0.0320513
RAB5B	0.4266335	0.4573157	0.4905201	0.3708031	0.4353034
SUOX	0.0641865	0.0577434	0.0563018	0.0489898	0.0343511
IKZF4	0.0864523	0.1062287	0.1129484	0.0934808	0.1029192
ENSG00000273890	0.0184378	0.0045245	0.0010537	0.0000000	0.0071423
ENSG00000257449	0.0005236	0.0024465	0.0000000	0.0025217	0.0069993
RPS26	2.4920884	2.4576923	2.2967263	2.4226722	2.1290437
ERBB3	0.0000000	0.0000000	0.0018156	0.0000000	0.0000000
PA2G4	1.0400094	0.9977631	1.0881298	0.9631014	0.7989815
ENSG00000257553	0.0049602	0.0005680	0.0036658	0.0000000	0.0000000
RPL41	3.8172057	3.6855871	3.4684228	3.7351939	3.4359263
ESYT1	0.0863429	0.0649088	0.0251139	0.1064503	0.0793077
ZC3H10	0.0412689	0.0342093	0.0283467	0.0495100	0.0173272
ENSG00000258317	0.0000000	0.0000000	0.0000000	0.0000000	0.0089890
MYL6B-AS1	0.0011015	0.0000000	0.0011062	0.0032488	0.0198079
MYL6B	1.0271668	1.1542188	1.2202289	0.9780240	0.9352634
MYL6	2.5495141	2.4177192	2.3233688	2.6259455	2.1300493
SMARCC2	0.5822958	0.6657169	0.7541620	0.5817010	0.5882164
ENSG00000258199	0.0000000	0.0000000	0.0071477	0.0000000	0.0044438
RNF41	0.2323776	0.2993851	0.2993788	0.2027426	0.1763137
NABP2	0.2760412	0.3055550	0.2949443	0.2649876	0.2622264
SLC39A5	0.0021134	0.0019463	0.0000000	0.0000000	0.0000000
ANKRD52	0.0670389	0.0806379	0.0764962	0.0912262	0.1640224
COQ10A	0.1297916	0.0865367	0.1329911	0.0835406	0.0386893
CS	0.3532503	0.4192794	0.3811910	0.2725290	0.3770347
CNPY2-AS1	0.1217042	0.0878592	0.0580622	0.1160816	0.0911694
ENSG00000257740	0.0030478	0.0000000	0.0000000	0.0000000	0.0000000
CNPY2	0.7744347	0.6273018	0.7123984	0.7634103	0.6313137
PAN2	0.0664226	0.0398394	0.0258090	0.0452504	0.0736065
IL23A	0.0013986	0.0120286	0.0097685	0.0068861	0.0000000
STAT2	0.1474819	0.1225088	0.0646548	0.1014428	0.1929177
TIMELESS	0.0505649	0.0376371	0.0415308	0.0417427	0.0286949
MIP	0.0006053	0.0000000	0.0000000	0.0039833	0.0000000
SPRYD4	0.0509383	0.0549950	0.0880445	0.0450742	0.0627474
GLS2	0.0296273	0.0465120	0.0787337	0.0285201	0.0404353
RBMS2	0.0983043	0.0951531	0.0538364	0.0805334	0.1690957
BAZ2A	0.2177096	0.2439260	0.2331223	0.1940428	0.3077022
ATP5F1B	1.4613771	1.5951258	1.7285378	1.5231446	1.2598281
PTGES3	2.0722458	2.0666783	2.2345175	2.0024747	1.7773371

NACA	2.7890881	2.7669794	2.6680167	2.7404456	2.5243679
PRIM1	0.0893096	0.1499574	0.1748125	0.0998007	0.0951356
HSD17B6	0.0050330	0.0031071	0.0036385	0.0051485	0.0000000
SDR9C7	0.0000000	0.0000000	0.0000000	0.0000000	0.0068600
RDH16	0.0024123	0.0013364	0.0019641	0.0000000	0.0058123
ENSG00000287715	0.0000000	0.0000000	0.0035151	0.0000000	0.0000000
ZBTB39	0.0521916	0.0510995	0.0733486	0.0395052	0.0951747
TAC3	0.0545045	0.0940460	0.0268482	0.0328861	0.1105289
MYO1A	0.0000000	0.0000000	0.0035853	0.0000000	0.0000000
NEMP1	0.0477284	0.0404238	0.0251310	0.0854509	0.0329872
NAB2	0.0781313	0.1106765	0.0800163	0.0850349	0.0776630
STAT6	0.0367914	0.0363353	0.0028628	0.0239546	0.0186166
LRP1	0.4276268	0.3516199	0.4143692	0.6999111	0.5974724
NXPH4	0.3735907	0.3209796	0.2746552	0.3313634	0.2987537
ENSG00000276727	0.0017759	0.0018528	0.0017026	0.0028524	0.0000000
SHMT2	0.8447454	0.6206446	0.3739516	0.8420551	0.5317880
NDUFA4L2	1.2670731	0.8580584	0.6147005	1.0539007	0.8595708
STAC3	0.0029963	0.0045766	0.0018156	0.0074990	0.0236645
R3HDM2	0.8791446	1.1205990	1.1715376	0.8050326	1.0793561
R3HDM2-DT	0.0016522	0.0000000	0.0024703	0.0020370	0.0000000
INHBC	0.0000000	0.0023461	0.0031290	0.0000000	0.0046972
INHBE	0.0053461	0.0033483	0.0044992	0.0123158	0.0000000
ENSG00000287200	0.0028410	0.0021302	0.0020798	0.0000000	0.0031804
GLI1	0.0005019	0.0000000	0.0024942	0.0000000	0.0000000
ARHGAP9	0.0024107	0.0043167	0.0120681	0.0024618	0.0045212
MARS1	0.3751131	0.3710453	0.4618977	0.3535841	0.4630809
DDIT3	0.7979768	0.6131697	0.5262286	0.6790231	0.4701468
MBD6	0.0719546	0.0337835	0.0180901	0.0370720	0.0803560
DCTN2	0.8572246	0.9199200	0.9402671	0.8296438	0.6721083
KIF5A	0.7604711	1.2317438	1.3803681	0.6091888	1.0311825
PIP4K2C	0.0714816	0.0850228	0.0652871	0.0699950	0.0734103
DTX3	0.2831612	0.2883331	0.3421844	0.2584879	0.2605560
ARHGEF25	0.1160914	0.1548471	0.1597951	0.1329084	0.1107800
ENSG00000224713	0.0000000	0.0035942	0.0028544	0.0000000	0.0000000
B4GALNT1	0.0851056	0.1545426	0.1810488	0.0602539	0.1113345
OS9	0.5515209	0.4812268	0.5519748	0.8347297	0.4210708
ENSG00000257342	0.0027556	0.0019572	0.0033471	0.0017080	0.0082531
AGAP2	0.1005568	0.1996932	0.2425703	0.0793365	0.1777309
TSPAN31	0.3269094	0.2560419	0.2051213	0.3991996	0.2017529
CDK4	0.5751144	0.3585170	0.3228228	0.5953123	0.3857667
MARCHF9	0.1587742	0.2193905	0.2265741	0.1529663	0.1724645
CYP27B1	0.0000000	0.0013789	0.0000000	0.0000000	0.0000000
METTL1	0.0316222	0.0365926	0.0411445	0.0530827	0.0370948
EEF1AKMT3	0.0431651	0.0301607	0.0109452	0.0201733	0.0127971
TSFM	0.1795103	0.1859786	0.2457141	0.1961169	0.1522750
AVIL	0.0084546	0.0059848	0.0066510	0.0057543	0.0231295
ENSG00000270039	0.0150494	0.0219170	0.0189586	0.0118926	0.0087175
ENSG00000269903	0.0007181	0.0000000	0.0000000	0.0000000	0.0000000
CTDSP2	0.1531630	0.0679478	0.0573628	0.1694041	0.0696246
ENSG00000257953	0.0000000	0.0000000	0.0024957	0.0000000	0.0075530
ENSG00000245651	0.0146847	0.0098419	0.0012490	0.0016475	0.0127462
ENSG00000273805	0.0047422	0.0013181	0.0000000	0.0024381	0.0000000
ATP23	0.0915014	0.1239737	0.1150375	0.0928082	0.0802743

GIHCG	0.6530470	0.4995823	0.3621472	0.5805796	0.4329290
ENSG00000258231	0.0081137	0.0026556	0.0000000	0.0000000	0.0103203
LINC02388	0.0042518	0.0000000	0.0037035	0.0021501	0.0077388
LRIG3	0.1361308	0.0693202	0.0475265	0.1509665	0.0777645
LRIG3-DT	0.0023875	0.0000000	0.0000000	0.0084630	0.0000000
ENSG00000286351	0.0021389	0.0000000	0.0000000	0.0000000	0.0000000
SLC16A7	0.1374383	0.1519694	0.1174276	0.1280658	0.2440319
ENSG00000286402	0.0080340	0.0067218	0.0014263	0.0100598	0.0343610
TAFA2	0.3542132	0.4288253	0.4285382	0.2570738	1.1142043
ENSG00000257880	0.0000000	0.0000000	0.0000000	0.0000000	0.0117766
USP15	0.4052247	0.5173388	0.5647004	0.4005391	0.6885949
MON2	0.2744751	0.2772436	0.2356837	0.3141977	0.6124514
LINC01465	0.0016852	0.0033784	0.0000000	0.0054274	0.0000000
MIRLET7IHG	0.0213454	0.0293447	0.0362290	0.0276043	0.0843885
ENSG00000275180	0.0030097	0.0027065	0.0013389	0.0042829	0.0000000
PPM1H	0.1070263	0.1911878	0.1727466	0.0962920	0.1989674
AVPR1A	0.0038823	0.0000000	0.0061418	0.0000000	0.0000000
DPY19L2	0.0688386	0.0814785	0.0598907	0.0651389	0.2160704
ENSG00000249753	0.0026236	0.0029844	0.0013491	0.0000000	0.0017206
RXYLT1	0.3323920	0.2432469	0.1829254	0.3955820	0.1869840
SRGAP1	0.4203992	0.5688841	0.6004378	0.4050217	0.8632470
ENSG00000256571	0.0024944	0.0000000	0.0026308	0.0000000	0.0072113
ENSG00000255886	0.0035286	0.0012294	0.0028703	0.0000000	0.0345258
ENSG00000287618	0.0013342	0.0009004	0.0000000	0.0000000	0.0070107
ENSG00000255629	0.0007744	0.0000000	0.0011032	0.0000000	0.0000000
KICS2	0.0377160	0.0373581	0.0403905	0.0361983	0.0471668
C12orf56	0.0008382	0.0018160	0.0013222	0.0000000	0.0000000
XPOT	0.8819590	0.6608844	0.5803806	0.7996602	0.6246302
TBK1	0.1776731	0.1597125	0.1660406	0.1808218	0.2490545
RASSF3	0.0142186	0.0081308	0.0077592	0.0023027	0.0319517
ENSG00000256199	0.0021411	0.0000000	0.0000000	0.0000000	0.0000000
GNS	0.2731502	0.1684878	0.1589958	0.5896351	0.2412769
TBC1D30	0.0875237	0.1707728	0.2230703	0.0623717	0.1500451
ENSG00000286682	0.0005438	0.0010122	0.0020277	0.0012937	0.0000000
LINC02389	0.0197776	0.0484204	0.0429596	0.0291521	0.0828991
LINC02231	0.0009536	0.0004904	0.0061025	0.0000000	0.0037844
WIF1	0.0435655	0.0456226	0.0707216	0.0311459	0.0869001
ENSG00000289319	0.0060169	0.0047485	0.0025863	0.0041035	0.0000000
LEMD3	0.1342457	0.1446495	0.1917847	0.1754666	0.2368325
ENSG00000276853	0.0027419	0.0014183	0.0029602	0.0048478	0.0022372
MSRB3	0.1170768	0.0781020	0.0569242	0.0883241	0.1116347
ENSG00000250280	0.0016242	0.0000000	0.0010214	0.0000000	0.0000000
MSRB3-AS1	0.0010882	0.0000000	0.0000000	0.0000000	0.0000000
HMGA2	0.0097654	0.0175583	0.0127843	0.0171752	0.0217209
HMGA2-AS1	0.0095981	0.0051665	0.0090527	0.0181351	0.0101478
ENSG00000256083	0.0016673	0.0040807	0.0000000	0.0000000	0.0000000
LLPH	0.3609773	0.3491255	0.2913594	0.3872957	0.3279530
LLPH-DT	0.0031847	0.0000000	0.0000000	0.0000000	0.0000000
TMBIM4	0.5967298	0.4418508	0.4626319	0.7278457	0.4076440
IRAK3	0.0116871	0.0256628	0.0082989	0.0065750	0.0316106
HELB	0.0398730	0.0223919	0.0173196	0.0586754	0.0782416
GRIP1	0.3551940	0.4619417	0.3644192	0.3017435	1.3330853
ENSG00000257083	0.0026584	0.0061376	0.0027720	0.0020988	0.0033030

CAND1	0.8515008	0.7693818	0.8219850	0.8394549	0.7995913
ENSG00000286563	0.0046067	0.0085274	0.0033668	0.0000000	0.0192883
LINC02408	0.0013807	0.0051787	0.0000000	0.0000000	0.0050816
DYRK2	0.3395258	0.2777068	0.3393295	0.3233666	0.2870943
ENSG00000235872	0.0029920	0.0030626	0.0000000	0.0012897	0.0058928
LINC01479	0.0000000	0.0009089	0.0000000	0.0042145	0.0000000
IFNG-AS1	0.0009253	0.0002873	0.0000000	0.0000000	0.0000000
MDM1	0.2414028	0.1160038	0.1025401	0.2566251	0.0918406
LINC02384	0.0007386	0.0008911	0.0000000	0.0000000	0.0000000
RAP1B	0.8822327	0.8374411	0.8663301	0.9202020	0.7657433
NUP107-DT	0.0444490	0.0330662	0.0191925	0.0420001	0.0290463
NUP107	0.1419757	0.1332873	0.0741423	0.1273661	0.1596981
SLC35E3	0.2037058	0.2175804	0.2595065	0.2045064	0.3777072
MDM2	0.6905137	0.4503933	0.3913333	0.6596863	0.4525048
ENSG00000256325	0.0000000	0.0014966	0.0000000	0.0000000	0.0000000
ENSG00000257181	0.0044738	0.0039695	0.0000000	0.0071084	0.0014498
CPM	0.0052916	0.0104385	0.0121292	0.0019410	0.0210150
CPSF6	0.4434832	0.4677608	0.5185082	0.4558559	0.7085960
LYZ	0.0024258	0.0000000	0.0000000	0.0000000	0.0038629
ENSG00000257764	0.0000000	0.0000000	0.0000000	0.0000000	0.0000000
YEATS4	0.3617187	0.3268421	0.3665175	0.4437468	0.2186369
LINC02373	0.0000000	0.0000000	0.0000000	0.0000000	0.0036615
FRS2	0.4280100	0.3624888	0.3350897	0.3748583	0.4274038
CCT2	1.2159746	1.1879266	1.2829545	1.2060350	0.9631598
ENSG00000247131	0.0000000	0.0000000	0.0000000	0.0045301	0.0069963
RAB3IP	0.3276448	0.4705872	0.5528278	0.2900663	0.4034772
MYRFL	0.0208561	0.0148813	0.0086348	0.0105060	0.0382105
PRANCR	0.1459061	0.1693572	0.1335713	0.1548481	0.5186961
LINC02821	0.0000000	0.0000000	0.0000000	0.0022553	0.0061335
CNOT2	0.4730275	0.4121797	0.3774158	0.4798957	0.5755993
ENSG00000289283	0.0000000	0.0032318	0.0000000	0.0047839	0.0188380
KCNMB4	0.9448355	0.6969000	0.5919806	0.8802716	0.6914121
ENSG00000258168	0.0000000	0.0017571	0.0041594	0.0000000	0.0000000
PTPRB	0.0058046	0.0088709	0.0057806	0.0060429	0.0125004
PTPRR	0.0343756	0.0697135	0.1192020	0.0295985	0.1879359
ENSG00000257265	0.0000000	0.0000000	0.0000000	0.0000000	0.0000000
ENSG00000258053	0.0000000	0.0000000	0.0000000	0.0000000	0.0000000
TSPAN8	0.0007078	0.0000000	0.0012267	0.0000000	0.0000000
LGR5	0.0037883	0.0013944	0.0115117	0.0049937	0.0119318
ENSG00000257761	0.0000000	0.0000000	0.0000000	0.0000000	0.0000000
ZFC3H1	0.3959446	0.4050942	0.4496876	0.3426340	0.5227560
THAP2	0.3841514	0.3365196	0.3488400	0.3539728	0.2864034
TMEM19	0.1620288	0.1598086	0.2127884	0.1692394	0.1431553
RAB21	0.5810702	0.5600229	0.5394889	0.5602151	0.5305205
ENSG00000257410	0.0008148	0.0000000	0.0012081	0.0000000	0.0000000
TBC1D15	0.5459895	0.3714863	0.2762239	0.4619119	0.4763529
TPH2	0.0163768	0.0208791	0.1347557	0.0089180	0.0329127
TRHDE	0.0461437	0.0687305	0.1534864	0.0524667	0.2310639
TRHDE-AS1	0.0098468	0.0147394	0.0391989	0.0000000	0.0070641
LINC02882	0.0000000	0.0000000	0.0026961	0.0000000	0.0158606
ATXN7L3B	1.2055120	1.4291397	1.6024859	1.1118967	1.0737108
ENSG00000257386	0.0022852	0.0023827	0.0029468	0.0058298	0.0064463
ENSG00000257434	0.0006962	0.0004619	0.0075180	0.0000000	0.0000000

KCNC2	0.1116350	0.1286880	0.1737470	0.1313925	0.3094679
CAPS2	0.1292074	0.0729526	0.0683024	0.1413720	0.1474117
ENSG00000273987	0.0064613	0.0000000	0.0000000	0.0000000	0.0000000
GLIPR1L1	0.0013342	0.0015486	0.0016528	0.0000000	0.0000000
GLIPR1L2	0.1010212	0.0758205	0.0442982	0.1002288	0.0445770
GLIPR1	0.0961688	0.0802699	0.0816146	0.1555045	0.0547702
GLIPR1-AS1	0.0049241	0.0023364	0.0000000	0.0000000	0.0000000
KRR1	0.5275918	0.4387610	0.4306417	0.4729918	0.3587706
ENSG00000258077	0.0046309	0.0050359	0.0000000	0.0000000	0.0210632
PHLDA1	0.3321533	0.3471241	0.3492761	0.3004865	0.2606646
PHLDA1-DT	0.0112620	0.0077182	0.0010529	0.0179523	0.0073726
NAP1L1	2.3934794	2.2876911	2.2092850	2.2620026	2.0930337
LNCOG	0.0000000	0.0000000	0.0000000	0.0000000	0.0000000
BBS10	0.1821270	0.1397293	0.1451606	0.2310075	0.1326145
OSBPL8	0.6805311	0.7809797	0.8304140	0.7215000	0.9925343
ENSG00000257526	0.0110660	0.0249721	0.0221219	0.0297896	0.0220132
ZDHHC17	0.5286782	0.4499125	0.5412603	0.4950295	0.6200958
CSRP2	0.8534280	0.6327647	0.5769347	0.9056920	0.5640651
ENSG00000257910	0.0007020	0.0000000	0.0000000	0.0000000	0.0000000
E2F7	0.0340049	0.0111588	0.0245779	0.0283502	0.0141884
LINC02464	0.0023394	0.0012453	0.0016489	0.0038334	0.0065051
NAV3	0.5597746	0.7091339	0.7259403	0.4492137	1.8550848
ENSG00000257835	0.0008567	0.0014079	0.0000000	0.0000000	0.0026927
ENSG00000289040	0.0058663	0.0085005	0.0000000	0.0120831	0.0585691
ENSG00000258066	0.0000000	0.0000000	0.0000000	0.0000000	0.0000000
ENSG00000258225	0.0000000	0.0000000	0.0000000	0.0000000	0.0060236
ENSG00000258084	0.0016864	0.0020327	0.0000000	0.0000000	0.0054639
SYT1	1.4090804	1.6218995	2.0469130	1.5497548	1.9232369
ENSG00000257191	0.0046682	0.0047600	0.0000000	0.0015092	0.0638840
ENSG00000257894	0.1147924	0.0668316	0.0894098	0.0837748	0.1327521
PAWR	0.6210627	0.4368737	0.3429622	0.6591029	0.4289253
PPP1R12A-AS2	0.0069056	0.0112880	0.0122503	0.0032832	0.0317916
PPP1R12A	0.7088304	0.7371165	0.6316310	0.7185825	0.8798042
ENSG00000277130	0.0011657	0.0000000	0.0000000	0.0000000	0.0000000
PPP1R12A-AS1	0.0250564	0.0290863	0.0135688	0.0401783	0.0101123
OTOGL	0.0014163	0.0014637	0.0044830	0.0029881	0.0109078
PTPRQ	0.0000000	0.0011947	0.0013814	0.0000000	0.0021151
MYF6	0.0037153	0.0106466	0.0163483	0.0028343	0.0101716
LINC01490	0.0000000	0.0000000	0.0000000	0.0000000	0.0031863
LIN7A	0.5402064	0.4775177	0.5558629	0.4600337	0.6245724
ACSS3	0.2472685	0.1675167	0.0877203	0.2211382	0.2796338
ENSG00000258026	0.0000000	0.0000000	0.0000000	0.0000000	0.0094551
PPFIA2	0.5775392	1.0118971	1.0946142	0.4173371	1.6102474
PPFIA2-AS1	0.0086860	0.0061132	0.0153717	0.0000000	0.0433212
ENSG00000258162	0.0069773	0.0079731	0.0099722	0.0019352	0.1098109
LINC02426	0.0000000	0.0000000	0.0000000	0.0000000	0.0000000
CCDC59	0.5588517	0.3770531	0.3120384	0.5102695	0.3877659
METTL25	0.1077194	0.1042797	0.0632864	0.1080999	0.2146123
ENSG00000258170	0.0000000	0.0000000	0.0000000	0.0000000	0.0000000
ENSG00000286043	0.0036712	0.0027977	0.0015898	0.0039833	0.0060977
TMTC2	0.2839259	0.2911540	0.2120581	0.2616500	0.9807947
ENSG00000289309	0.0000000	0.0000000	0.0000000	0.0000000	0.0061591
SLC6A15	0.1480658	0.2289876	0.2287306	0.1242067	0.3120628

ENSG00000289218	0.0034168	0.0031512	0.0185183	0.0000000	0.0161865
TSPAN19	0.0291411	0.0338293	0.0395108	0.0385752	0.0178783
LRRIQ1	0.8173719	0.4402321	0.3429783	0.7367169	0.6147067
ALX1	0.0000000	0.0000000	0.0000000	0.0000000	0.0000000
LINC02820	0.0046153	0.0011157	0.0000000	0.0024545	0.0035390
RASSF9	0.0383566	0.0203276	0.0145306	0.0944582	0.0336310
NTS	0.0117965	0.0144893	0.0150774	0.0108599	0.0179317
MGAT4C	0.1863387	0.3537045	0.3352462	0.2060202	0.9727543
ENSG00000289369	0.0000000	0.0010370	0.0000000	0.0000000	0.0032840
C12orf50	0.0011410	0.0024838	0.0037245	0.0000000	0.0032878
C12orf29	0.1937351	0.2276764	0.2068081	0.2513911	0.1699496
CEP290	0.6993836	0.6757450	0.6607905	0.7166352	0.9538138
TMTC3	0.1824505	0.1285271	0.1354950	0.3252747	0.1778348
ENSG00000289384	0.0029119	0.0000000	0.0000000	0.0000000	0.0016664
KITLG	0.4548101	0.2613366	0.1719339	0.5826553	0.2290058
ENSG00000281333	0.0257670	0.0207624	0.0092542	0.0401477	0.0000000
LINC02458	0.0028198	0.0045853	0.0017904	0.0000000	0.0080590
DUSP6	0.0766760	0.0863926	0.1396220	0.0961986	0.0772468
ENSG00000274021	0.0000000	0.0010716	0.0070922	0.0026362	0.0000000
ENSG00000286608	0.0550538	0.0395942	0.0767112	0.0500212	0.1516465
ENSG00000271327	0.0008387	0.0011451	0.0000000	0.0000000	0.0000000
ENSG00000271259	0.0003318	0.0000000	0.0000000	0.0028496	0.0017069
POC1B	0.1106661	0.0799957	0.0693265	0.0913233	0.1319852
GALNT4	0.0248692	0.0079911	0.0150963	0.0466406	0.0088181
POC1B-AS1	0.0220216	0.0142942	0.0309102	0.0221216	0.0683375
ENSG00000258302	0.0026079	0.0081682	0.0078347	0.0117854	0.0250378
ATP2B1	0.7723160	1.0309876	1.0672428	0.8593877	0.9970340
ATP2B1-AS1	0.1231239	0.2671325	0.2762670	0.1179635	0.2484968
ENSG00000258216	0.0046739	0.0157132	0.0026409	0.0018880	0.0244570
LINC02822	0.0019411	0.0037147	0.0025293	0.0000000	0.0000000
LUM	0.1929914	0.1101686	0.0924456	0.4680783	0.1230270
DCN	1.4031982	0.8701757	0.6363327	2.1863843	0.9750572
ENSG00000289605	0.0015902	0.0011164	0.0000000	0.0000000	0.0000000
LINC01619	0.0769153	0.0918824	0.0709911	0.1029106	0.4767513
ENSG00000286907	0.0000000	0.0000000	0.0000000	0.0000000	0.0000000
BTG1	1.0465466	0.9933281	1.0788915	0.9112007	0.9710054
BTG1-DT	0.0087083	0.0077476	0.0167624	0.0071499	0.0416943
LINC02391	0.0000000	0.0010451	0.0000000	0.0000000	0.0000000
LINC02397	0.0018951	0.0000000	0.0134910	0.0000000	0.0121234
PLEKHG7	0.0000000	0.0007377	0.0000000	0.0000000	0.0000000
EEA1	0.7463477	0.6940419	0.8033416	0.7673346	0.7506940
ENSG00000257322	0.0594983	0.0529039	0.0440316	0.0563614	0.0447229
ENSG00000257252	0.0169451	0.0052278	0.0015141	0.0078813	0.0000000
NUDT4	0.2181204	0.2495693	0.1642235	0.2100072	0.2299223
UBE2N	0.8175854	0.9836088	1.2301186	0.8008162	0.8764553
MRPL42	0.5879282	0.7154828	0.7925405	0.5180965	0.6034631
SOCS2-AS1	0.0230966	0.0329116	0.0313250	0.0118939	0.0441273
SOCS2	0.0685493	0.1576721	0.1918057	0.0977614	0.0997339
CRADD	0.1140660	0.1077687	0.0746652	0.1008777	0.2074241
CRADD-AS1	0.0004793	0.0000000	0.0000000	0.0000000	0.0083179
ENSG00000258303	0.0003764	0.0000000	0.0000000	0.0000000	0.0000000
ENSG00000257283	0.0011289	0.0000000	0.0000000	0.0000000	0.0073619
PLXNC1	0.1243882	0.2486335	0.4306648	0.0850614	0.3983059

ENSG00000258035	0.0000000	0.0000000	0.0007280	0.0000000	0.0015531
ENSG00000287237	0.0000000	0.0000000	0.0000000	0.0000000	0.0013712
ENSG00000258172	0.0000000	0.0012671	0.0025278	0.0000000	0.0000000
ENSG00000258365	0.0008718	0.0000000	0.0000000	0.0000000	0.0132028
CEP83	0.1492327	0.1419412	0.1377560	0.1483371	0.2177270
CEP83-DT	0.0079927	0.0111292	0.0186929	0.0114667	0.0346153
TMCC3	0.1381140	0.1689308	0.1256277	0.0448106	0.1436563
NDUFA12	0.9937365	1.2130113	1.2863112	1.0094930	0.8765848
NR2C1	0.2525590	0.2277556	0.2103252	0.2410622	0.3244535
FGD6	0.1016050	0.0962501	0.0597854	0.0925922	0.1460296
VEZT	0.4507567	0.3857743	0.3490998	0.4734472	0.6181976
ENSG00000289437	0.0028380	0.0088696	0.0030148	0.0028893	0.0174417
ENSG00000289476	0.0031392	0.0035029	0.0024426	0.0000000	0.0192566
ENSG00000289510	0.0062029	0.0108873	0.0103193	0.0032700	0.0066480
METAP2	1.0425039	1.0035554	0.8815428	1.0403990	0.8353986
USP44	0.0112413	0.0251978	0.0067906	0.0214038	0.0314523
NTN4	0.0189937	0.0374218	0.0561338	0.0182599	0.0470742
SNRPF-DT	0.0114262	0.0034807	0.0060538	0.0128084	0.0158941
LINC02410	0.0000000	0.0000000	0.0000000	0.0055207	0.0000000
SNRPF	0.8690856	0.8869665	0.9744330	0.8619770	0.6915262
CCDC38	0.0000000	0.0021876	0.0007835	0.0000000	0.0017223
AMDHD1	0.0049429	0.0000000	0.0016515	0.0000000	0.0000000
HAL	0.0000000	0.0023210	0.0016462	0.0000000	0.0013692
LTA4H	0.4591154	0.3529790	0.3232449	0.3756684	0.3267482
LINC02452	0.0000000	0.0000000	0.0000000	0.0000000	0.0053214
ELK3	0.0268123	0.0241946	0.0139420	0.0610929	0.0052574
ENSG00000287454	0.0004455	0.0000000	0.0000000	0.0000000	0.0000000
CDK17	0.4078922	0.3263970	0.4245880	0.3840479	0.4824322
ENSG00000258272	0.0007719	0.0014000	0.0021557	0.0000000	0.0000000
CFAP54	0.2618624	0.1755901	0.1367705	0.2302123	0.2900445
NEDD1	0.2370134	0.0948446	0.0999072	0.2424333	0.1124183
ENSG00000258131	0.0165533	0.0067195	0.0210912	0.0146821	0.0182868
ENSG00000257470	0.0052238	0.0021936	0.0008241	0.0037638	0.0000000
RMST	1.3250379	1.0653125	0.8163514	1.1836262	1.4199685
ENSG00000258312	0.0005537	0.0051829	0.0010824	0.0027628	0.0068999
LINC02453	0.0112523	0.0059561	0.0144751	0.0065228	0.0000000
TMPO-AS1	0.0143777	0.0146824	0.0203554	0.0244356	0.0080547
TMPO	0.3313316	0.3168989	0.4688941	0.3081889	0.3257525
SLC25A3	1.4458526	1.4760328	1.4696487	1.5225542	1.1962270
IKBIP	0.2044468	0.1463068	0.1023622	0.3121157	0.1342450
APAF1	0.2227193	0.2777160	0.3123005	0.2118401	0.2788892
ANKS1B	0.3866644	0.4814495	0.5908228	0.3352548	1.2661335
ENSG00000257458	0.0000000	0.0000000	0.0030777	0.0000000	0.0254519
ENSG00000258039	0.0000000	0.0000000	0.0000000	0.0000000	0.0000000
BLTP3B	0.3138203	0.4743471	0.5145176	0.2923171	0.3415355
UHRF1BP1L-DT	0.0019350	0.0037943	0.0000000	0.0000000	0.0000000
GOLGA2P5	0.0047146	0.0121389	0.0048889	0.0048694	0.0106015
ENSG00000290576	0.0071384	0.0028989	0.0065564	0.0061815	0.0042647
ACTR6	0.5871832	0.5092625	0.6761350	0.5236622	0.4105593
DEPDC4	0.0353933	0.0350655	0.0249926	0.0363725	0.0618667
SCYL2	0.4158917	0.4967249	0.4957980	0.4120877	0.5860298
SLC17A8	0.0255324	0.0731540	0.0716774	0.0273986	0.0467628
NR1H4	0.0013305	0.0000000	0.0000000	0.0000000	0.0000000

GAS2L3	0.0128943	0.0292753	0.0242909	0.0252709	0.0312737
ANO4	0.0647878	0.0866850	0.0696451	0.0412715	0.3302856
SLC5A8	0.0015477	0.0022250	0.0015789	0.0029843	0.0036662
UTP20	0.0440866	0.0798639	0.0757281	0.0396041	0.0861243
ARL1	0.6744203	0.4904855	0.4723586	0.7168114	0.4549713
ENSG00000274560	0.0000000	0.0011573	0.0048645	0.0000000	0.0000000
CHPT1	0.3761733	0.4597385	0.4280613	0.3464145	0.3105018
SYCP3	0.0031339	0.0011481	0.0000000	0.0023978	0.0039906
GNPTAB	0.4766050	0.6478531	0.7123187	0.4790788	0.5875212
DRAM1	0.0099034	0.0064489	0.0000000	0.0156041	0.0201247
WASHC3	0.3373997	0.3294933	0.3224352	0.3240733	0.2982148
ENSG00000257222	0.0000000	0.0000000	0.0000000	0.0000000	0.0023834
NUP37	0.1662662	0.0915217	0.0615406	0.1270340	0.1245406
PARPBP	0.0225448	0.0132945	0.0269445	0.0157610	0.0268902
HELLPAR	0.0110276	0.0099026	0.0036568	0.0047307	0.0182983
IGF1	0.0394822	0.0337840	0.0171347	0.0296414	0.0675061
PAH	0.0021018	0.0000000	0.0000000	0.0000000	0.0037415
ASCL1	0.0220578	0.0240444	0.0361176	0.0249230	0.0264606
C12orf42	0.0013704	0.0010918	0.0000000	0.0000000	0.0000000
STAB2	0.0012809	0.0010945	0.0000000	0.0000000	0.0000000
ENSG00000257681	0.0000000	0.0015228	0.0000000	0.0041277	0.0000000
NT5DC3	0.0933311	0.0994836	0.1065846	0.0649676	0.1411641
ENSG00000257327	0.0024597	0.0014347	0.0031723	0.0031966	0.0000000
HSP90B1	2.5386023	1.9868016	1.9743995	3.1436291	1.9556336
UQCC6	0.4233912	0.4747675	0.5930916	0.3973264	0.3953557
TDG	0.2846627	0.3085430	0.2661558	0.2041221	0.2555601
ENSG00000288744	0.0054419	0.0064939	0.0057032	0.0000000	0.0016028
GLT8D2	0.0845167	0.0599669	0.1100696	0.0671595	0.0735144
HCFC2	0.3121353	0.2282917	0.1928422	0.3169445	0.2835494
NFYB	0.4617733	0.4233265	0.5034269	0.4318532	0.3836930
TXNRD1	0.6371986	0.6015397	0.5982056	0.6791023	0.6075208
ENSG00000257732	0.0000000	0.0000000	0.0000000	0.0000000	0.0000000
EID3	0.0145270	0.0160988	0.0164876	0.0286182	0.0347490
CHST11	0.1359345	0.1718376	0.1930206	0.1192190	0.4160710
ENSG00000289557	0.0000000	0.0000000	0.0000000	0.0000000	0.0081210
SLC41A2	0.1609538	0.2119591	0.1883397	0.1631062	0.4109192
ENSG00000286410	0.0000000	0.0000000	0.0023779	0.0000000	0.0000000
NOPCHAP1	0.3738523	0.4477023	0.5324617	0.3820274	0.3402985
ALDH1L2	0.1548514	0.1670578	0.1908195	0.1622521	0.2331652
WASHC4	0.2548915	0.1988442	0.1487172	0.2812017	0.2931724
APPL2	0.8021511	0.4366650	0.3280827	0.8293244	0.5232289
C12orf75	1.3565582	1.2218000	1.0774789	1.4493855	1.0246550
CASC18	0.0012852	0.0000000	0.0000000	0.0000000	0.0022770
ENSG00000257890	0.0000000	0.0000000	0.0000000	0.0000000	0.0000000
NUAK1	0.1739426	0.2785714	0.3302810	0.1502071	0.2252875
ENSG00000257438	0.0000000	0.0000000	0.0000000	0.0000000	0.0000000
CKAP4	0.6669194	0.5729786	0.5300833	0.8687721	0.4891568
ENSG00000258355	0.0000000	0.0000000	0.0000000	0.0000000	0.0038927
ENSG00000277715	0.0352736	0.0187539	0.0237777	0.0361564	0.0277319
TCP11L2	0.2250373	0.1500497	0.1360972	0.1893892	0.1495596
ENSG00000290003	0.0328563	0.0210777	0.0079461	0.0297556	0.0150817
POLR3B	0.1919391	0.1433780	0.1344475	0.1405646	0.1455730
ENSG00000257545	0.0871190	0.0811081	0.0831316	0.0879431	0.3655664

RFX4	1.5155834	0.9716149	0.6741513	1.3655343	1.0586504
ENSG00000257711	0.0007727	0.0046489	0.0066233	0.0020830	0.0234494
ENSG00000257918	0.0098532	0.0093855	0.0000000	0.0016731	0.0039758
RIC8B	0.3277709	0.3117215	0.3181614	0.3531131	0.4840444
ENSG00000287957	0.0303257	0.0172322	0.0103613	0.0157005	0.0212231
TMEM263-DT	0.0358273	0.0384295	0.0360251	0.0366761	0.0245273
TMEM263	0.6870014	0.5291180	0.4611024	0.6354939	0.3557258
MTERF2	0.1155480	0.1345809	0.1198668	0.1376404	0.0672452
CRY1	0.1777553	0.1483540	0.1719842	0.1392122	0.2833870
ENSG00000257548	0.0011959	0.0034566	0.0000000	0.0000000	0.0093012
ABTB3	0.0505919	0.1056184	0.1137247	0.0668874	0.3679163
ENSG00000257579	0.0000000	0.0000000	0.0000000	0.0000000	0.0000000
ENSG00000289344	0.0016116	0.0019540	0.0000000	0.0000000	0.0014252
PWP1	0.4514055	0.4481778	0.5337651	0.4795559	0.3571741
PRDM4	0.1494047	0.2258395	0.1771212	0.1514996	0.1501957
PRDM4-AS1	0.0019323	0.0042604	0.0013609	0.0000000	0.0323235
ASCL4	0.0000000	0.0019747	0.0011896	0.0057389	0.0000000
ENSG00000257398	0.0025990	0.0022601	0.0055225	0.0000000	0.0000000
WSCD2	0.0656467	0.0998992	0.1334410	0.0431466	0.1815408
CMKLR1	0.0000000	0.0000000	0.0000000	0.0000000	0.0000000
LINC01498	0.0005849	0.0000000	0.0000000	0.0036005	0.0016028
FICD	0.0525176	0.0352493	0.0488194	0.0657833	0.0209100
SART3	0.2489803	0.2209916	0.1966536	0.2872074	0.2666234
ENSG00000287202	0.0056389	0.0015682	0.0000000	0.0000000	0.0114450
ISCU	0.6364997	0.5861637	0.5438832	0.6285308	0.4468422
TMEM119	0.0000000	0.0032204	0.0000000	0.0037157	0.0070221
SELPLG	0.0021737	0.0034294	0.0026716	0.0009140	0.0066879
ENSG00000257221	0.0000000	0.0000000	0.0000000	0.0000000	0.0000000
CORO1C	0.2888110	0.3667476	0.4423693	0.3023520	0.4318573
SSH1	0.1223255	0.1242013	0.1078211	0.1320253	0.2284197
ENSG00000274598	0.0000000	0.0002873	0.0000000	0.0021965	0.0000000
DAO	0.0041953	0.0011339	0.0047463	0.0108245	0.0101999
SVOP	0.0815534	0.1618796	0.1597049	0.0849822	0.2828971
USP30	0.1078743	0.1085951	0.0679143	0.1195868	0.1866303
ALKBH2	0.2009673	0.1625423	0.1706996	0.1910291	0.1312766
UNG	0.2016821	0.1864529	0.1898000	0.2851803	0.1629698
ACACB	0.0406459	0.0181131	0.0166096	0.0279795	0.0361535
FOXN4	0.0000000	0.0000000	0.0000000	0.0051214	0.0000000
MYO1H	0.0047160	0.0012859	0.0030709	0.0040831	0.0112036
ENSG00000255655	0.0008158	0.0011451	0.0021053	0.0018326	0.0000000
KCTD10	0.0739626	0.1236875	0.1347134	0.0739949	0.0943433
UBE3B	0.1625727	0.1415894	0.0999076	0.1160898	0.1919869
MMAB	0.2612998	0.2201787	0.2169271	0.2859660	0.2013062
MVK	0.0620980	0.0623573	0.0675048	0.0635884	0.0708476
FAM222A	0.0447005	0.0665109	0.0859765	0.0451319	0.1420275
FAM222A-AS1	0.0000000	0.0037802	0.0013884	0.0077234	0.0146347
TRPV4	0.0000000	0.0000000	0.0000000	0.0000000	0.0036428
GLTP	0.3837963	0.2977155	0.3078501	0.3454536	0.2687187
TCHP	0.2296615	0.1949046	0.1696949	0.1457653	0.2761428
GIT2	0.1845445	0.1960740	0.2217587	0.1366463	0.2914819
ANKRD13A	0.1800557	0.2270078	0.1927736	0.2216921	0.2624695
C12orf76	0.7826939	0.7558449	0.7634364	0.7922746	0.5725679
IFT81	0.7588376	0.5237965	0.4855164	0.7660209	0.5534315

ENSG00000289311	0.0096955	0.0101016	0.0183253	0.0106333	0.0249050
ATP2A2	0.8543312	0.7652951	0.8488550	1.1281530	0.8280789
ANAPC7	0.2148414	0.2467735	0.2671687	0.1867031	0.2441185
ARPC3	1.1264346	1.1413723	1.2057651	1.1974841	0.8637732
GPN3	0.2831153	0.2685256	0.2401492	0.2928725	0.2048581
FAM216A	0.3197076	0.5869546	0.7960770	0.3059129	0.3937383
VPS29	0.6892842	0.7448642	0.8023491	0.7144897	0.5818036
RAD9B	0.0102854	0.0069414	0.0069611	0.0031240	0.0127946
PPTC7	0.0795738	0.1000907	0.1095884	0.0737227	0.1119045
TCTN1	0.6794746	0.4403222	0.3876398	0.8750933	0.4027329
HVCN1	0.0087520	0.0104624	0.0191169	0.0252471	0.0065348
PPP1CC	0.5846614	0.5564599	0.6038263	0.5645918	0.4268138
LINC01405	0.0000000	0.0000000	0.0068775	0.0000000	0.0000000
LINC01404	0.0060894	0.0013043	0.0130941	0.0000000	0.0042989
CUX2	0.0818401	0.1240267	0.1321377	0.0446116	0.6701554
PHETA1	0.0347978	0.0324826	0.0277544	0.0316625	0.0516632
LINC02356	0.0045595	0.0103231	0.0111132	0.0019580	0.0239131
SH2B3	0.0264319	0.0298687	0.0256575	0.0147533	0.0605869
ATXN2	0.7233197	0.6937677	0.7274627	0.7240488	1.0057610
ATXN2-AS	0.0025579	0.0000000	0.0014377	0.0062666	0.0057039
BRAP	0.2526127	0.2037226	0.1874631	0.2548853	0.2182492
ACAD10	0.0540438	0.0558488	0.0386219	0.0614229	0.1399300
ALDH2	0.6522282	0.5556932	0.5998977	0.7775411	0.4859126
MAPKAPK5-AS1	0.2332901	0.2556733	0.2284398	0.1887252	0.2189873
MAPKAPK5	0.2315046	0.2644884	0.2937731	0.2147906	0.2604156
TMEM116	0.1085768	0.1100152	0.0936900	0.0947014	0.2149855
ERP29	1.0494107	0.9416420	0.9774800	1.2821360	0.8412673
ENSG00000274227	0.0026768	0.0000000	0.0008433	0.0000000	0.0000000
NAA25	0.2190834	0.2312040	0.2409569	0.2155864	0.3027907
ENSG00000258323	0.0000000	0.0011317	0.0000000	0.0000000	0.0036574
TRAFD1	0.1880781	0.1705637	0.2275037	0.1733093	0.0881948
HECTD4	0.3855477	0.5169666	0.6197655	0.3189730	0.8991980
ENSG00000257494	0.0000000	0.0000000	0.0000000	0.0042330	0.0000000
RPL6	3.1810104	3.1805894	2.9900118	3.1084253	2.8444837
PTPN11	1.1426340	1.1042636	1.0384390	0.9717590	1.0253032
RPH3A	0.1250435	0.2622715	0.5369827	0.1118045	0.2232952
OAS1	0.0000000	0.0020518	0.0000000	0.0000000	0.0000000
ENSG00000257452	0.0000000	0.0000000	0.0000000	0.0000000	0.0000000
OAS3	0.0014942	0.0000000	0.0016016	0.0041661	0.0000000
DTX1	0.0710105	0.1170091	0.1338243	0.0770071	0.1930060
RASAL1	0.0010824	0.0029264	0.0081352	0.0077165	0.0072113
CFAP73	0.0236061	0.0110921	0.0022394	0.0242211	0.0114399
DDX54	0.1940507	0.1677248	0.1515656	0.1692119	0.1034274
RITA1	0.1988543	0.1882158	0.1937895	0.1721435	0.1468632
ENSG00000257286	0.0000000	0.0018588	0.0000000	0.0000000	0.0049154
IQCD	0.2113963	0.1252159	0.0750877	0.2162068	0.1171854
TPCN1	0.0473538	0.0448924	0.0276235	0.0996726	0.1307493
ENSG00000277566	0.0014451	0.0000000	0.0000000	0.0000000	0.0000000
SLC8B1	0.0072529	0.0137030	0.0043295	0.0080567	0.0051612
PLBD2	0.0752223	0.0860144	0.0788470	0.1014408	0.1189431
ENSG00000288863	0.0000000	0.0015384	0.0056534	0.0000000	0.0015201
SDS	0.0012439	0.0000000	0.0000000	0.0000000	0.0075543
SDSL	0.3354720	0.3145225	0.2645724	0.3654691	0.2192900

LHX5	0.0020425	0.0070483	0.0163213	0.0000000	0.0308851
LHX5-AS1	0.0148600	0.0316123	0.0598529	0.0083728	0.0168277
LINC01234	0.0132629	0.0287070	0.0512265	0.0090781	0.0431599
RBM19	0.1288322	0.1270649	0.1454064	0.1136564	0.1072827
ENSG00000257359	0.0000000	0.0000000	0.0000000	0.0000000	0.0000000
TBX3	0.0294552	0.0575138	0.0111301	0.0147860	0.0852022
TBX3-AS1	0.0037674	0.0047645	0.0006071	0.0000000	0.0134590
ENSG00000257407	0.0000000	0.0000000	0.0000000	0.0022668	0.0000000
ENSG00000257781	0.0000000	0.0000000	0.0000000	0.0000000	0.0000000
LINC02463	0.0000000	0.0000000	0.0000000	0.0000000	0.0000000
MED13L	0.4480137	0.5003964	0.4352707	0.3910134	1.0694449
ENSG00000258034	0.0015507	0.0008684	0.0000000	0.0000000	0.0000000
ENSG00000258337	0.0032158	0.0026888	0.0028763	0.0000000	0.0125590
LINC02457	0.0000000	0.0009808	0.0012199	0.0000000	0.0000000
LINC00173	0.0086269	0.0038951	0.0040837	0.0103245	0.0288001
MAP1LC3B2	0.0543505	0.0852040	0.0763599	0.0652644	0.0588600
ENSG00000275898	0.0007781	0.0000000	0.0009265	0.0000000	0.0000000
ENSG00000257883	0.0000000	0.0015318	0.0000000	0.0000000	0.0054224
SPRING1	0.2609616	0.3939238	0.4449578	0.2159931	0.2294364
RNFT2	0.0945735	0.1438505	0.1314631	0.0853698	0.1772742
ENSG00000290033	0.0054272	0.0040371	0.0051144	0.0023899	0.0115077
HRK	0.1574778	0.2774705	0.1152731	0.1154077	0.2062769
FBXW8	0.1091282	0.0569593	0.0759408	0.0828276	0.2001020
ENSG00000257279	0.0000000	0.0000000	0.0000000	0.0000000	0.0013481
TESC	0.0145758	0.0185812	0.0205289	0.0049835	0.0131918
TESC-AS1	0.0000000	0.0000000	0.0030192	0.0000000	0.0041172
FBXO21	0.7149276	0.7593754	0.8270261	0.6531494	0.6937189
NOS1	0.0031966	0.0070201	0.0126479	0.0015202	0.0164615
KSR2	0.1057036	0.1928340	0.1770818	0.0699655	0.5449248
RFC5	0.0935580	0.0950731	0.0612541	0.1144180	0.0447591
ENSG00000277873	0.0029546	0.0032856	0.0020314	0.0000000	0.0000000
WSB2	0.8316710	1.0451439	1.1172528	0.7417726	0.8401423
VSIG10	0.0774119	0.0493117	0.0536297	0.0986023	0.1305488
ENSG00000274859	0.0094983	0.0060926	0.0046303	0.0000000	0.0053413
PEBP1	2.2726337	2.4490207	2.5849189	2.3508034	1.9902557
TAOK3	0.4618428	0.5041957	0.5096814	0.4991140	0.7402029
ENSG00000270482	0.0011524	0.0011923	0.0021935	0.0000000	0.0000000
SUDS3	0.5428403	0.7023043	0.8416726	0.5332907	0.5721316
ENSG00000275759	0.0027215	0.0009203	0.0015669	0.0043276	0.0000000
ENSG00000275409	0.0006084	0.0000000	0.0014810	0.0024839	0.0000000
LINC02440	0.0150273	0.0133999	0.0157963	0.0068852	0.0076653
SRRM4	0.3156015	0.5120656	0.7189814	0.2627166	0.9645662
ENSG00000257095	0.0000000	0.0000000	0.0000000	0.0000000	0.0106762
HSPB8	0.1581441	0.1090598	0.0722005	0.1908212	0.1003129
CCDC60	0.0562325	0.0302732	0.0202927	0.0288648	0.0505081
ENSG00000248636	0.0218364	0.0120802	0.0135736	0.0263049	0.0187352
TMEM233	0.0781659	0.0831781	0.0604890	0.1672468	0.0525676
PRKAB1	0.2082019	0.1732499	0.1736455	0.2290939	0.1423192
CIT	0.1073548	0.1627628	0.2589068	0.0981033	0.3304614
ENSG00000255692	0.0019126	0.0000000	0.0000000	0.0000000	0.0028743
BICDL1	0.0930918	0.1144438	0.1162604	0.0658681	0.4523365
RAB35	0.2527056	0.2693844	0.3051903	0.2358637	0.2214732
RAB35-AS1	0.0432837	0.0550746	0.0558019	0.0693374	0.0371370

GCN1	0.0793711	0.0777693	0.0564681	0.1052749	0.1676910
RPLP0	3.2265603	3.1646973	2.9456484	3.1299049	2.8827915
PXN-AS1	0.1095773	0.0723908	0.0851534	0.0901257	0.0661928
PXN	0.1609513	0.0993174	0.0450211	0.1454043	0.1705285
ENSG00000286067	0.0000000	0.0000000	0.0000000	0.0000000	0.0056663
SIRT4	0.0139475	0.0328189	0.0217147	0.0063458	0.0083135
PLA2G1B	0.0017658	0.0054602	0.0012170	0.0000000	0.0000000
MSI1	0.4227153	0.3086876	0.2337685	0.4404418	0.2494232
COX6A1	1.8042235	2.0608821	2.1243368	1.9212039	1.7590527
TRIAP1	0.2725271	0.2055026	0.1838993	0.2382214	0.1363118
GATC	0.2166000	0.2729536	0.3219512	0.2091713	0.1761423
SRSF9	1.2670832	1.2228309	1.2686663	1.2867532	1.0696181
DYNLL1	2.3277330	2.2907474	2.3742235	2.3588175	1.9687188
NRAV	0.2236489	0.1308316	0.1411531	0.2527294	0.1066785
COQ5	0.2814004	0.2773408	0.2729603	0.2640054	0.1606293
RNF10	0.6648800	0.7550410	0.7976313	0.7206816	0.6606297
ENSG00000289401	0.0016796	0.0020023	0.0000000	0.0000000	0.0160580
POP5	0.2813286	0.2267299	0.2116220	0.3404729	0.2252052
CABP1-DT	0.0067609	0.0179930	0.0369557	0.0047383	0.0185048
CABP1	0.0383733	0.0648792	0.0680251	0.0441489	0.0775646
MLEC	0.9976166	0.7399191	0.7041733	1.1818724	0.6984070
ENSG00000256364	0.0035627	0.0051705	0.0013879	0.0069878	0.0074108
UNC119B	0.1963724	0.1444712	0.0940389	0.2570354	0.1400207
ACADS	0.0704252	0.0446819	0.0330934	0.0657931	0.0493736
ENSG00000255946	0.0043660	0.0010122	0.0069521	0.0030470	0.0601362
SPPL3	0.5329322	0.5446729	0.5865830	0.5703857	0.6835732
ENSG00000286493	0.0000000	0.0000000	0.0000000	0.0000000	0.0030362
HNF1A-AS1	0.0026660	0.0028780	0.0028944	0.0000000	0.0000000
HNF1A	0.0006742	0.0006303	0.0013672	0.0000000	0.0192796
C12orf43	0.1968050	0.2163915	0.2405329	0.2268890	0.1624869
OASL	0.0000000	0.0018198	0.0012333	0.0000000	0.0000000
P2RX7	0.0092848	0.0068342	0.0000000	0.0033858	0.0084011
ENSG00000286248	0.0000000	0.0000000	0.0071678	0.0000000	0.0063461
P2RX4	0.0781333	0.0597266	0.0447980	0.0970476	0.0584469
CAMKK2	0.1486928	0.1674948	0.1799357	0.1315104	0.1870799
ANAPC5	0.3947560	0.4238839	0.4071926	0.3747653	0.4117520
ENSG00000258435	0.0000000	0.0000000	0.0000000	0.0000000	0.0000000
RNF34	0.2564204	0.2542767	0.2283688	0.2386740	0.2075761
KDM2B	0.0835309	0.2040561	0.2085106	0.1021620	0.2569729
KDM2B-DT	0.0046273	0.0064195	0.0081903	0.0044709	0.0342631
ORAI1	0.0388700	0.0249168	0.0522952	0.0457195	0.0204010
MORN3	0.0360464	0.0291337	0.0247378	0.0414445	0.0402931
ENSG00000286586	0.0032878	0.0034234	0.0000000	0.0000000	0.0000000
TMEM120B	0.0900401	0.0588579	0.0643177	0.0845335	0.1764655
RHOF	0.0532875	0.0667972	0.0916010	0.0445651	0.0701296
LINC01089	0.0446142	0.0559165	0.0265615	0.0503498	0.0459463
ENSG00000274292	0.0032955	0.0049888	0.0202250	0.0077385	0.0262032
SETD1B	0.0819620	0.0852569	0.0825731	0.0699431	0.1516667
HPD	0.0008030	0.0004136	0.0000000	0.0000000	0.0058361
ENSG00000287493	0.0000000	0.0000000	0.0000000	0.0000000	0.0000000
PSMD9	0.5693083	0.4581454	0.3579560	0.6168104	0.3896450
CFAP251	0.1061308	0.0812286	0.0460768	0.1003915	0.1774956
ENSG00000255856	0.0059356	0.0124202	0.0086323	0.0074104	0.0043337

BCL7A	0.3046042	0.4391170	0.5374248	0.2813336	0.3170917
LINC02985	0.0073450	0.0099149	0.0212194	0.0158865	0.0143498
MLXIP	0.1413133	0.1481130	0.1339198	0.1310580	0.3080182
LRRC43	0.0641137	0.0366301	0.0496125	0.1039544	0.0970560
B3GNT4	0.0339780	0.0644496	0.0565693	0.0405277	0.0351257
DIABLO	0.0129304	0.0192175	0.0148606	0.0048084	0.0236064
VPS33A	0.1181187	0.1387479	0.1667098	0.1062477	0.1256115
CLIP1	0.5516564	0.6329134	0.6283539	0.4610313	0.8940547
CLIP1-AS1	0.0000000	0.0018870	0.0000000	0.0058370	0.0000000
ZCCHC8	0.2251685	0.1679386	0.1672962	0.2646207	0.1950047
ENSG00000275265	0.0013732	0.0013844	0.0000000	0.0000000	0.0040567
RSRC2	1.0425596	0.9810679	0.9252123	1.0548940	0.8679201
KNTC1	0.0400605	0.0335192	0.0191598	0.0431372	0.0594646
ENSG00000274191	0.0007798	0.0000000	0.0000000	0.0000000	0.0045817
ENSG00000256249	0.0021592	0.0017360	0.0040139	0.0000000	0.0072130
DENR	0.7236570	0.7744901	0.9440859	0.7275369	0.6177928
CCDC62	0.0248356	0.0354065	0.0236201	0.0115983	0.0772400
HIP1R	0.0727788	0.1068695	0.1162782	0.0717173	0.2137435
VPS37B	0.2053736	0.1456606	0.1112158	0.2269812	0.2513039
ENSG00000256152	0.0000000	0.0025345	0.0000000	0.0024577	0.0040206
ABCB9	0.0369782	0.0693850	0.0602919	0.0407775	0.1440630
OGFOD2	0.0611684	0.0490420	0.0397684	0.0686574	0.0079978
ARL6IP4	0.8662926	0.7824856	0.8373935	0.9061977	0.7729309
PITPNM2	0.0565042	0.0703171	0.0550934	0.0352950	0.2467955
MPHOSPH9	0.2145553	0.1792357	0.1565208	0.1708540	0.3263483
MTRFR	0.5713913	0.4949826	0.4268576	0.5623363	0.4315506
CDK2AP1	1.5904145	1.2472289	0.9752636	1.7172337	1.0484922
ENSG00000269980	0.0011103	0.0025910	0.0026325	0.0000000	0.0000000
SBNO1	0.6083638	0.7135590	0.7791232	0.5632171	0.6027561
SBNO1-AS1	0.2327250	0.1719719	0.1252457	0.2121091	0.1026419
KMT5A	0.3942526	0.3867470	0.4200470	0.4789363	0.3405790
RILPL2	0.1673083	0.1103560	0.0594274	0.1879980	0.0595015
SNRNP35	0.2186935	0.1947553	0.2205872	0.2508409	0.1344254
RILPL1	0.1639110	0.1425656	0.1639390	0.1540581	0.2574527
TMED2-DT	0.0161968	0.0114944	0.0130279	0.0050025	0.0037366
TMED2	0.9518272	0.7975805	0.7709050	0.9888275	0.7167228
DDX55	0.1479610	0.1230441	0.1158246	0.1558308	0.1887023
EIF2B1	0.1802779	0.1416868	0.1502577	0.1964067	0.1297208
GTF2H3	0.3814683	0.2684785	0.2604149	0.3837162	0.3137992
TCTN2	0.1793667	0.0951404	0.0663218	0.2695309	0.1286652
ENSG00000255839	0.0043821	0.0000000	0.0000000	0.0037834	0.0183729
ATP6V0A2	0.0944695	0.0878731	0.0727061	0.0970599	0.1932540
ENSG00000286092	0.0000000	0.0000000	0.0000000	0.0000000	0.0000000
DNAH10	0.0448251	0.0616245	0.0537373	0.0338035	0.0837534
CCDC92	0.5199834	0.7326748	0.8064799	0.4849035	0.6218970
DNAH10OS	0.1028888	0.2167465	0.2100464	0.0828788	0.1215942
ENSG00000270130	0.0028669	0.0018028	0.0060728	0.0000000	0.0000000
ENSG00000270048	0.0014726	0.0000000	0.0000000	0.0030630	0.0019545
ENSG00000269997	0.0000000	0.0000000	0.0000000	0.0000000	0.0013481
ENSG00000269938	0.0047231	0.0027472	0.0012752	0.0000000	0.0080521
ENSG00000270061	0.0000000	0.0000000	0.0000000	0.0000000	0.0059085
ENSG00000270095	0.0000000	0.0000000	0.0007378	0.0000000	0.0000000
ZNF664	0.3816687	0.3585747	0.3159422	0.3306420	0.2847895

RFLNA	0.1270205	0.1723531	0.1459342	0.0825972	0.4640153
ENSG00000275389	0.0039509	0.0089953	0.0070177	0.0030295	0.0134847
NCOR2	0.3390852	0.3108813	0.2217906	0.2730344	0.5833579
ENSG00000214650	0.0000000	0.0000000	0.0025355	0.0000000	0.0000000
SCARB1	0.0314040	0.0368110	0.0399108	0.0217283	0.0399082
ENSG00000287242	0.0118478	0.0130388	0.0000000	0.0092482	0.0283480
UBC	2.1717292	2.0670282	2.0902497	2.1099286	1.8236319
DHX37	0.0440533	0.0738902	0.0204572	0.0354245	0.0414304
BRI3BP	0.1082715	0.1638264	0.2416678	0.1345701	0.1284190
THRIL	0.0094561	0.0116292	0.0163319	0.0000000	0.0171446
AACS	0.0732083	0.0817471	0.0781817	0.0599342	0.1256111
ENSG00000256814	0.0000000	0.0000000	0.0000000	0.0000000	0.0016664
TMEM132B	0.0306870	0.0360616	0.0251004	0.0164165	0.1226851
ENSG00000286922	0.0000000	0.0000000	0.0000000	0.0000000	0.0000000
TMEM132C	0.0000000	0.0000000	0.0000000	0.0000000	0.0000000
SLC15A4	0.0888107	0.0596175	0.0531953	0.1400937	0.1055156
GLT1D1	0.0468902	0.0767157	0.1046163	0.0318368	0.0993051
TMEM132D	0.0701921	0.1022591	0.0757981	0.0561074	0.4312019
FZD10-AS1	0.0423479	0.0402946	0.0161602	0.0285127	0.0236647
FZD10	0.0254911	0.0185037	0.0071970	0.0118544	0.0160579
PIWIL1	0.0000000	0.0000000	0.0013476	0.0000000	0.0000000
RIMBP2	0.1254396	0.1741978	0.2064258	0.1408775	0.5936402
ENSG00000256064	0.0000000	0.0032927	0.0000000	0.0000000	0.0106788
ENSG00000256343	0.0000000	0.0000000	0.0000000	0.0000000	0.0000000
STX2	0.2977612	0.2691703	0.2253658	0.3177750	0.2955509
ENSG00000256250	0.0000000	0.0000000	0.0000000	0.0000000	0.0000000
RAN	1.5637494	1.5208012	1.6171341	1.6026479	1.2407936
ADGRD1	0.0070118	0.0108676	0.0134177	0.0133777	0.0100692
LINC01257	0.0027428	0.0015372	0.0000000	0.0000000	0.0065021
LINC02370	0.0000000	0.0011451	0.0000000	0.0000000	0.0000000
ENSG00000275232	0.0000000	0.0000000	0.0000000	0.0000000	0.0000000
SFSWAP	0.3244724	0.3214202	0.3212631	0.3057339	0.5107661
MMP17	0.0396024	0.0993196	0.1709405	0.0345189	0.1926483
ENSG00000256955	0.0048298	0.0014577	0.0000000	0.0059470	0.0326993
ULK1	0.1081082	0.1167987	0.1342474	0.0781938	0.2424802
PUS1-AS1	0.0020034	0.0011612	0.0013609	0.0000000	0.0035522
PUS1	0.0275264	0.0226119	0.0144784	0.0327306	0.0358109
ENSG00000273568	0.0006466	0.0032763	0.0061789	0.0000000	0.0000000
EP400	0.1889102	0.2249713	0.2342547	0.1928885	0.5549105
ENSG00000291171	0.0564644	0.0739234	0.0544161	0.0571084	0.1230394
DDX51	0.0449669	0.0486211	0.0352078	0.0300603	0.0749727
NOC4L	0.0752981	0.1032273	0.1007451	0.0660319	0.0733634
LINC02361	0.0230087	0.0307829	0.0281494	0.0137076	0.0104754
GALNT9	0.0775220	0.1616080	0.1958330	0.0493911	0.1709004
ENSG00000277011	0.0000000	0.0000000	0.0000000	0.0000000	0.0000000
ENSG00000255916	0.0000000	0.0022518	0.0009261	0.0073633	0.0049696
FBRSL1	0.1555092	0.2388277	0.2741690	0.1316140	0.3396822
ENSG00000277186	0.0063773	0.0201548	0.0229903	0.0023239	0.0043847
LRCOL1	0.0000000	0.0020993	0.0000000	0.0000000	0.0000000
POLE	0.0230898	0.0186822	0.0229169	0.0183351	0.0862364
PXMP2	0.2900820	0.3309132	0.4077502	0.2084393	0.2140505
PGAM5	0.1025963	0.1083783	0.1409911	0.1062771	0.0699052
ANKLE2	0.3017996	0.2973140	0.3438591	0.3246692	0.3291515

GOLGA3	0.4319209	0.4883221	0.5020419	0.4276036	0.5496934
CHFR	0.1064134	0.1390958	0.1003278	0.1001490	0.1909280
CHFR-DT	0.0032078	0.0023762	0.0028489	0.0150751	0.0031305
ENSG00000250790	0.0213060	0.0108304	0.0120569	0.0293942	0.0067583
ZNF605	0.1553798	0.2165764	0.1885458	0.2000365	0.2110379
ENSG00000289516	0.0269382	0.0294365	0.0403344	0.0385886	0.0335158
ZNF26	0.0399133	0.0303609	0.0493514	0.0448133	0.1144196
ZNF84-DT	0.0027893	0.0013921	0.0000000	0.0071895	0.0022383
ZNF84	0.2969833	0.3014669	0.2930638	0.3269205	0.3265322
ZNF140	0.0790659	0.0955710	0.0677370	0.1107505	0.1036826
ZNF891	0.0786814	0.1104679	0.0862843	0.0595495	0.2466061
ZNF10	0.0928211	0.1234975	0.0848149	0.0922015	0.1726555
ZNF268	0.1258020	0.0927603	0.1094558	0.1167579	0.1619425
TPTE2	0.0046964	0.0069463	0.0029356	0.0000000	0.0528107
LINC00350	0.0000000	0.0010716	0.0000000	0.0000000	0.0000000
MPHOSPH8	0.8764468	0.8424215	0.8295851	0.8001776	0.6855727
PSPC1	0.3897107	0.4015235	0.4896873	0.3678726	0.6450795
ZMYM5	0.2216018	0.1590824	0.1308312	0.2215856	0.2569255
ENSG00000223576	0.0000000	0.0000000	0.0023296	0.0000000	0.0000000
ENSG00000275964	0.1161707	0.0660971	0.0923705	0.0846963	0.0669053
ZMYM2	0.5990595	0.6796105	0.6560010	0.5721703	0.9432280
LINC01072	0.0000000	0.0000000	0.0000000	0.0000000	0.0000000
CRYL1	0.3389776	0.3053103	0.2704393	0.3297506	0.2591794
ENSG00000277020	0.0018624	0.0051507	0.0010575	0.0000000	0.0000000
IFT88	0.5458579	0.4099175	0.3470664	0.5175640	0.4692128
ENSG00000278291	0.0625807	0.0438858	0.0702302	0.0736561	0.0451283
IL17D	0.1306249	0.1490162	0.1674153	0.1516893	0.1072477
EEF1AKMT1	0.2218766	0.2116206	0.1948497	0.2190793	0.1710700
XPO4	0.1964369	0.1334924	0.1207682	0.1290280	0.2789929
LATS2	0.0780557	0.0530806	0.0456576	0.0724763	0.0689528
SAP18	1.3887498	1.3062125	1.2530710	1.2795278	1.1211758
SKA3	0.0131433	0.0071690	0.0029107	0.0111889	0.0000000
MRPL57	1.2697872	1.0449639	1.0536305	1.2092721	0.9488650
LINC01046	0.0002224	0.0000000	0.0033715	0.0000000	0.0104720
ENSG00000290911	0.0786927	0.0451152	0.0308787	0.0532100	0.0602018
ENSG00000291046	0.2271641	0.1733318	0.1496024	0.2522790	0.2059567
LINC00539	0.0116593	0.0251617	0.0102706	0.0278108	0.0226264
ZDHHC20	0.2378185	0.1797953	0.2130163	0.2490912	0.4957938
MICU2	0.5160347	0.5008739	0.5487836	0.5288975	0.6902869
ENSG00000289860	0.0017828	0.0099863	0.0120402	0.0021398	0.0192515
FGF9	0.0842933	0.2121726	0.3180643	0.0505486	0.2481789
LINC00540	0.0003758	0.0043158	0.0000000	0.0068008	0.0107008
LINC00621	0.0243098	0.0654588	0.0476852	0.0251807	0.0309611
ENSG00000262198	0.0120697	0.0093454	0.0124992	0.0020158	0.0097155
SGCG	0.0100056	0.0083374	0.0058338	0.0154087	0.0247249
SACS	0.3686643	0.5452766	0.6156555	0.3477226	0.6651789
SACS-AS1	0.0000000	0.0000000	0.0000000	0.0000000	0.0000000
LINC00327	0.0050759	0.0072811	0.0039443	0.0049506	0.0039284
ENSG00000289688	0.0000000	0.0009224	0.0014307	0.0000000	0.0076428
TNFRSF19	0.1102503	0.0741666	0.0617358	0.2096505	0.1114255
ENSG00000289332	0.0000000	0.0020329	0.0000000	0.0000000	0.0000000
MIPEP	0.2189862	0.1336295	0.1102987	0.2953736	0.1587436
PCOTH	0.0182002	0.0152845	0.0027191	0.0105108	0.0058520

ENSG00000290660	0.0010370	0.0000000	0.0022460	0.0000000	0.0000000
SPATA13	0.2306516	0.1546596	0.0986110	0.1872538	0.2558101
SPATA13.1	0.0033779	0.0000000	0.0028387	0.0016755	0.0000000
C1QTNF9	0.0006082	0.0000000	0.0000000	0.0000000	0.0106645
LINC00566	0.0002886	0.0000000	0.0000000	0.0000000	0.0062540
PARP4	0.2201493	0.1342736	0.0851302	0.2177630	0.1263301
ENSG00000288103	0.0045090	0.0026376	0.0000000	0.0044674	0.0069313
ENSG00000290443	0.0020463	0.0066767	0.0020178	0.0000000	0.0000000
ATP12A	0.0450479	0.0438957	0.0295436	0.0562614	0.0532044
ENSG00000287887	0.0051684	0.0185610	0.0251552	0.0094847	0.0061768
RNF17	0.0002350	0.0000000	0.0022159	0.0000000	0.0000000
CENPJ	0.1231837	0.1998801	0.2493359	0.1710159	0.3340893
ENSG00000291041	0.0460626	0.0754899	0.0670819	0.0308487	0.1489498
AMER2	0.1100452	0.2280962	0.3432515	0.1152695	0.2386498
LINC01053	0.0000000	0.0000000	0.0000000	0.0000000	0.0000000
LINC00463	0.0003671	0.0012005	0.0000000	0.0000000	0.0000000
MTMR6	0.3251583	0.3984311	0.4095626	0.3343589	0.4718025
NUP58	0.3380096	0.3762258	0.3521216	0.2540957	0.3899907
ATP8A2	0.2819689	0.4488373	0.5294519	0.2193827	0.8438150
ENSG00000289125	0.0000000	0.0000000	0.0000000	0.0000000	0.0000000
SHISA2	0.0116247	0.0169622	0.0120560	0.0025868	0.0189821
RNF6	0.2677797	0.1972160	0.1115558	0.2252995	0.1656501
ENSG00000277368	0.0085324	0.0057170	0.0009013	0.0017529	0.0000000
CDK8	0.4228199	0.4991045	0.5876957	0.3540238	0.5465315
WASF3	0.9079434	0.7757101	0.7071371	0.8983396	0.6739381
WASF3-AS1	0.0009014	0.0012489	0.0000000	0.0053016	0.0000000
ENSG00000285935	0.0075009	0.0199077	0.0047954	0.0173263	0.0040567
GPR12	0.0894184	0.2059368	0.2716870	0.0756296	0.1764229
ENSG00000283579	0.0000000	0.0000000	0.0000000	0.0000000	0.0000000
USP12	0.3497459	0.3985790	0.4637122	0.2778731	0.3437712
USP12-AS1	0.0000000	0.0000000	0.0000000	0.0000000	0.0085419
USP12-DT	0.0036738	0.0031882	0.0000000	0.0000000	0.0000000
LINC02340	0.0356426	0.0307013	0.0279651	0.0263786	0.1073853
LINC00412	0.0018647	0.0011504	0.0000000	0.0000000	0.0021151
RPL21	3.1294724	3.0412241	2.8939054	2.9775296	2.7783361
RASL11A	0.0069839	0.0073950	0.0027264	0.0024483	0.0190915
GTF3A	0.8748874	1.1195791	1.1416295	0.8587914	0.8296103
MTIF3	0.5927739	0.5770765	0.4913636	0.5795388	0.4082507
ENSG00000289827	0.0015398	0.0034899	0.0073820	0.0076237	0.0000000
LNX2	0.0444220	0.0663831	0.0356299	0.0463476	0.0983556
POLR1D	0.7272093	0.7552441	0.7416961	0.6466157	0.6273068
FLT3	0.0098997	0.0081620	0.0073295	0.0053722	0.0307070
PAN3-AS1	0.0178883	0.0123543	0.0205604	0.0180941	0.0202531
PAN3	0.1900687	0.1708202	0.1356768	0.2051272	0.4905522
FLT1	0.0028192	0.0000000	0.0016522	0.0000000	0.0000000
ENSG00000289569	0.0011892	0.0014137	0.0035392	0.0000000	0.0000000
POMP	1.2803794	1.2677635	1.3259791	1.2488455	0.9638133
SLC46A3	0.0423021	0.0197478	0.0391505	0.0512819	0.0764032
MTUS2	0.1194765	0.1615601	0.1409177	0.0755129	0.5685227
MTUS2-AS2	0.0000000	0.0000000	0.0000000	0.0000000	0.0028353
MTUS2-AS1	0.0008729	0.0007126	0.0000000	0.0000000	0.0131471
SLC7A1	0.1558165	0.2295942	0.2093955	0.1627207	0.2865150
UBL3	1.1252645	0.9569310	1.0006446	1.0338895	0.9225461

LINC00365	0.0000000	0.0014253	0.0026802	0.0000000	0.0086096
LINC00384	0.0000000	0.0000000	0.0038529	0.0000000	0.0000000
KATNAL1	0.5974305	0.6444983	0.5978724	0.5167289	0.6754750
LINC00426	0.0000000	0.0034433	0.0000000	0.0000000	0.0038839
ENSG00000289302	0.0010370	0.0000000	0.0000000	0.0021989	0.0026919
UBE2L5	0.0024808	0.0013242	0.0009158	0.0188373	0.0000000
HMGB1	2.4879331	2.5807247	2.8519173	2.4511549	2.2545664
ENSG00000285840	0.0000000	0.0000000	0.0000000	0.0000000	0.0039851
USPL1	0.1700945	0.2350594	0.2766689	0.1819912	0.2431619
ALOX5AP	0.0029388	0.0093637	0.0032376	0.0000000	0.0033906
TEX26-AS1	0.0058608	0.0049902	0.0053500	0.0117545	0.0057154
MEDAG	0.0065326	0.0038329	0.0024807	0.0032573	0.0000000
TEX26	0.2837814	0.1811610	0.1448203	0.2506818	0.1581625
HSPH1	1.1887325	1.0112136	1.0512213	1.1176577	0.9397795
B3GLCT	0.3692358	0.2399928	0.1575911	0.5322865	0.2756128
ENSG00000287904	0.0000000	0.0000000	0.0000000	0.0000000	0.0030832
FRY	0.3862961	0.5885558	0.5646331	0.3260733	1.1713474
ENSG00000289617	0.0015128	0.0127525	0.0041671	0.0023531	0.0000000
FRY-AS1	0.0155793	0.0219647	0.0295216	0.0147401	0.0191373
ZAR1L	0.0010350	0.0028552	0.0028865	0.0192602	0.0000000
BRCA2	0.0137629	0.0298127	0.0299380	0.0121763	0.0076055
N4BP2L1	0.0474902	0.0482640	0.1148916	0.0474627	0.0697170
N4BP2L2	0.7419093	0.6410423	0.6074666	0.7115034	0.7595887
N4BP2L2-IT2	0.0042114	0.0103867	0.0046303	0.0000000	0.0177679
PDS5B	0.5604235	0.7098256	0.8416363	0.5890011	1.1910297
ENSG00000277151	0.0064628	0.0063754	0.0158017	0.0067256	0.0164879
KL	0.0024775	0.0032050	0.0010181	0.0000000	0.0067852
STARD13	0.0355174	0.0579572	0.0679450	0.0594376	0.1298025
STARD13-AS	0.0010612	0.0010401	0.0000000	0.0000000	0.0000000
ENSG00000287274	0.0000000	0.0014023	0.0000000	0.0000000	0.0072356
ENSG00000285621	0.0000000	0.0000000	0.0000000	0.0000000	0.0000000
ENSG00000230490	0.0077715	0.0084053	0.0068594	0.0167653	0.0163922
RFC3	0.0814233	0.1060753	0.1067054	0.0994506	0.2143765
ENSG00000276672	0.0034057	0.0055474	0.0022216	0.0000000	0.0036382
LINC02343	0.0060906	0.0020069	0.0111374	0.0046125	0.0120494
LINC00457	0.0000000	0.0008078	0.0000000	0.0000000	0.0000000
ENSG00000261728	0.0002511	0.0027533	0.0000000	0.0000000	0.0000000
NBEA	0.7695620	0.9219296	0.9200645	0.6350920	1.7221941
MAB21L1	0.0165261	0.0340572	0.0758559	0.0141742	0.0733100
ENSG00000287650	0.0005509	0.0000000	0.0000000	0.0000000	0.0058600
DCLK1	0.8092258	0.8881152	1.0361964	0.9655848	1.5198356
SOHLH2	0.0729291	0.1207832	0.0884238	0.0667488	0.0619671
CCDC169	0.0541143	0.0654383	0.0806262	0.0771777	0.0474478
SPART	1.2879687	0.8694965	0.5650986	1.3473869	0.7540662
SPART-AS1	0.0250279	0.0168745	0.0079855	0.0228146	0.0356443
CCNA1	0.3357900	0.2021584	0.1625456	0.3812514	0.1564983
SERTM1	0.9348281	0.5182360	0.2832605	1.0880908	0.3607466
RFXAP	0.2757208	0.2185541	0.2409695	0.3189900	0.1906474
SMAD9	0.4431534	0.3386139	0.2911410	0.4485789	0.3271315
SMAD9-IT1	0.0000000	0.0033450	0.0000000	0.0000000	0.0000000
ALG5	0.3221991	0.2589341	0.2621583	0.3183671	0.2601767
EXOSC8	0.3886102	0.3041651	0.2983339	0.3931070	0.3426202
SUPT20H	0.4165202	0.3482066	0.3743989	0.4287639	0.4735258

POSTN	0.0148122	0.0097591	0.0617041	0.0431806	0.1502609
TRPC4	0.0331354	0.0294832	0.0452894	0.0305120	0.1318912
ENSG00000287477	0.0000000	0.0000000	0.0000000	0.0000000	0.0000000
LINC02334	0.0000000	0.0006705	0.0000000	0.0000000	0.0060804
LINC00571	0.0257144	0.0284011	0.0139231	0.0087851	0.0130656
UFM1	1.0254123	1.0844421	1.0353484	0.9705592	0.8252969
LINC00366	0.0000000	0.0013122	0.0000000	0.0000000	0.0000000
FREM2	0.0414539	0.0158625	0.0159245	0.0629325	0.0272558
STOML3	0.0130074	0.0118068	0.0039715	0.0205074	0.0022413
PROSER1	0.0763270	0.0675287	0.0397561	0.0599484	0.0765770
NHLRC3	0.1284388	0.1225946	0.1314796	0.1589153	0.1782150
ENSG00000273507	0.0038237	0.0052161	0.0057606	0.0018904	0.0325043
LHFPL6	0.2556394	0.1695353	0.1866874	0.5556067	0.2692637
COG6	0.3244626	0.2424315	0.2467972	0.2837376	0.4439502
LINC00598	0.0047320	0.0042842	0.0034463	0.0092215	0.0251638
FOXO1	0.0605265	0.0437859	0.0633927	0.0729744	0.0699650
MRPS31	0.4979946	0.3869867	0.3702696	0.4777289	0.3921755
SLC25A15	0.0579811	0.1011375	0.0994379	0.0633901	0.0659516
ENSG00000290476	0.0558859	0.0394590	0.0451851	0.0470116	0.1375226
ENSG00000287837	0.0022570	0.0050128	0.0034191	0.0000000	0.0040206
SUGT1P3	0.0062885	0.0050397	0.0118078	0.0076915	0.0043054
ELF1	0.3165868	0.1787137	0.0883261	0.2446295	0.1753034
WBP4	0.5846408	0.4499007	0.3968428	0.5218808	0.3221296
KBTBD6	0.2052415	0.2927292	0.4545108	0.1912822	0.2885941
ENSG00000278390	0.0255537	0.0323245	0.0195066	0.0273311	0.0763627
KBTBD7	0.1170417	0.1324591	0.1792759	0.1179196	0.0578968
MTRF1	0.0702252	0.0727607	0.0725280	0.0681672	0.0809070
ENSG00000277662	0.0004013	0.0014991	0.0010824	0.0000000	0.0042020
NAA16	0.2368604	0.1995976	0.1872490	0.2124586	0.3063715
RGCC	0.0127529	0.0209503	0.0090788	0.0087565	0.0033679
ENSG00000288598	0.0011161	0.0006778	0.0000000	0.0000000	0.0185525
VWA8	0.1596151	0.1784421	0.1324268	0.1621801	0.4835142
VWA8-AS1	0.0037256	0.0067359	0.0061854	0.0132119	0.0284592
DGKH	0.1286834	0.1887324	0.1555405	0.0968314	0.2767202
AKAP11	0.6846383	0.7356787	0.7870355	0.6166514	0.7113155
LINC02341	0.0000000	0.0032615	0.0010907	0.0012486	0.0000000
TNFSF11	0.0000000	0.0000000	0.0000000	0.0055436	0.0000000
FAM216B	0.0569001	0.0479721	0.0322062	0.0869574	0.0337636
EPSTI1	0.0084321	0.0047860	0.0095789	0.0026054	0.0119382
DNAJC15	0.7939106	0.7061602	0.7960450	0.7527503	0.5431011
LINC00400	0.0052260	0.0028867	0.0115746	0.0027965	0.0000000
ENOX1	0.4810508	0.4617549	0.4207464	0.4308096	1.1062800
ENOX1-AS2	0.0129817	0.0038725	0.0030120	0.0129128	0.0122851
ENSG00000287842	0.0003476	0.0000000	0.0000000	0.0000000	0.0045609
ENSG00000287029	0.0003250	0.0023347	0.0000000	0.0018880	0.0112174
CCDC122	0.3174430	0.2202826	0.2031436	0.3877869	0.1962025
ENSG00000274001	0.0000000	0.0013395	0.0000000	0.0000000	0.0156137
LACC1	0.0603652	0.0680255	0.0702538	0.0408128	0.0259286
NRAD1	0.0000000	0.0006718	0.0034838	0.0000000	0.0068988
LINC00390	0.0098357	0.0042194	0.0018792	0.0000000	0.0081555
SMIM2-AS1	0.0047724	0.0020120	0.0060644	0.0030679	0.0199140
ENSG00000277831	0.0013731	0.0076303	0.0030265	0.0000000	0.0040567
SERP2	0.3212074	0.4367859	0.6555409	0.2818260	0.3046572

TUSC8	0.0007773	0.0000000	0.0000000	0.0000000	0.0000000
TSC22D1	1.5088371	1.4711912	1.5554464	1.3964357	1.4709453
TSC22D1-AS1	0.0037676	0.0069675	0.0160011	0.0087896	0.0247522
LINC00407	0.0055412	0.0016814	0.0021490	0.0032121	0.0161402
ENSG00000276527	0.0000000	0.0000000	0.0000000	0.0000000	0.0041430
NUFIP1	0.2331409	0.2282258	0.2099394	0.2227362	0.1540385
ENSG00000223732	0.0063562	0.0045041	0.0021202	0.0090505	0.0206973
GPALPP1	0.3210583	0.2854915	0.2826959	0.3197723	0.3712639
GTF2F2	0.4269603	0.3900112	0.4259343	0.4583327	0.4050003
KCTD4	0.0051505	0.0201616	0.0511142	0.0174086	0.0321987
TPT1	3.5550609	3.3814193	3.1892950	3.3935085	3.2044958
ENSG00000273149	0.0095355	0.0034387	0.0135103	0.0037550	0.0204169
TPT1-AS1	0.2498518	0.2756643	0.2990413	0.1938823	0.4921415
SLC25A30	0.0808871	0.0564121	0.0628203	0.0836722	0.0428708
SLC25A30-AS1	0.0024365	0.0000000	0.0000000	0.0055470	0.0000000
COG3	0.1526371	0.1828798	0.1364623	0.1862516	0.1927835
ERICH6B	0.0055928	0.0025855	0.0008839	0.0017529	0.0524504
SIAH3	0.1421426	0.2986051	0.1531030	0.1054679	0.1505335
ZC3H13	0.9824808	1.0737480	1.1225132	0.8632051	1.0495955
CPB2-AS1	0.1119488	0.2172557	0.1428113	0.1022020	0.1977475
CPB2	0.0000000	0.0008070	0.0077795	0.0012960	0.0156797
LCP1	0.0090246	0.0121371	0.0140104	0.0144438	0.0028666
LRRC63	0.0147385	0.0461093	0.0155957	0.0144743	0.0357549
RUBCNL	0.0101162	0.0276014	0.0099290	0.0157080	0.0200013
ENSG00000277228	0.0066516	0.0080994	0.0084597	0.0031927	0.0000000
LRCH1	0.1448598	0.1725395	0.1447125	0.1636497	0.4663425
ESD	1.2374105	1.0650636	0.8820532	1.2049400	0.8713190
HTR2A	0.0309634	0.0433388	0.0418753	0.0250682	0.0484040
SUCLA2	0.5150486	0.5592399	0.7005532	0.4491778	0.4834275
LINC00562	0.0012933	0.0041111	0.0047180	0.0000000	0.0035346
ENSG00000287456	0.0000000	0.0000000	0.0000000	0.0000000	0.0022413
SUCLA2-AS1	0.0068075	0.0101092	0.0110024	0.0078237	0.0094282
NUDT15	0.2144406	0.2733109	0.3785659	0.2338508	0.2006765
ENSG00000276968	0.0005455	0.0012878	0.0040342	0.0018917	0.0000000
MED4	0.7097392	0.6958705	0.8051826	0.7125348	0.5994178
MED4-AS1	0.0017578	0.0013252	0.0031267	0.0000000	0.0000000
ITM2B	2.0277298	1.5753800	1.5524590	2.5768191	1.5955240
RB1-DT	0.0161386	0.0195297	0.0088453	0.0120024	0.0000000
RB1	0.7127322	0.5184157	0.3899550	0.6846088	0.6468605
LPAR6	0.1166320	0.0381087	0.0233849	0.2356692	0.1038724
RCBTB2	0.1734359	0.1347543	0.0767642	0.2132445	0.1159018
ENSG00000274929	0.0031754	0.0035690	0.0082507	0.0044709	0.0000000
ENSG00000275202	0.0084357	0.0038427	0.0140552	0.0075574	0.0073473
FNDC3A	0.6877763	0.6326575	0.6930284	0.6542244	0.9001943
ENSG00000288743	0.0297972	0.0127328	0.0172671	0.0272094	0.0066699
CDADC1	0.1905942	0.1914049	0.1649240	0.2227381	0.1624488
CAB39L	0.2964227	0.3528630	0.5112901	0.2532151	0.4282393
SETDB2	0.1225030	0.1346241	0.1407508	0.1091988	0.1790267
PHF11	0.2204826	0.1739630	0.1105195	0.1770251	0.1406756
RCBTB1	0.1711547	0.1503269	0.1472205	0.1638080	0.1114002
EBPL	0.4358403	0.5139653	0.4916266	0.5006257	0.4497316
KPNA3	0.7261711	0.6613149	0.7154963	0.6829964	0.6818519
ENSG00000276436	0.0011061	0.0034493	0.0000000	0.0000000	0.0000000

SPRYD7	0.4855509	0.4506617	0.5023898	0.4593469	0.3540888
DLEU2	0.1032238	0.1057544	0.0436939	0.0640808	0.2190697
TRIM13	0.2572229	0.2296856	0.1945057	0.2491759	0.2941271
KCNRG	0.0145523	0.0012148	0.0057049	0.0112154	0.0000000
DLEU1	0.1471935	0.1032312	0.0878668	0.1527401	0.3511890
ENSG00000286191	0.0000000	0.0000000	0.0000000	0.0016823	0.0000000
DLEU7	0.0626583	0.1344962	0.1093462	0.0428048	0.1394806
RNASEH2B-AS1	0.0091486	0.0128557	0.0098844	0.0015092	0.0034081
RNASEH2B	0.3756162	0.3911255	0.3297732	0.2950218	0.2404955
C13orf42	0.0009651	0.0015842	0.0000000	0.0000000	0.0000000
FAM124A	0.0655042	0.0789601	0.0684360	0.0702260	0.1130274
SERPINE3	0.0027588	0.0030129	0.0017819	0.0052468	0.0198306
INTS6	0.2174178	0.1840214	0.1822048	0.1907246	0.3748618
INTS6-AS1	0.0489224	0.0561878	0.0544652	0.0335957	0.0413495
WDFY2	0.2703330	0.2528918	0.1623884	0.3083936	0.4616222
DHRS12	0.1046018	0.0695378	0.0546386	0.0921445	0.0543549
ENSG00000231856	0.0180295	0.0143792	0.0032631	0.0080934	0.0133524
TMEM272	0.0009658	0.0011527	0.0000000	0.0000000	0.0099762
ATP7B	0.0281228	0.0392434	0.0210766	0.0196041	0.0988794
ALG11	0.1441099	0.1546841	0.1572383	0.1888538	0.1998878
NEK5	0.1207002	0.1115709	0.0653626	0.1702317	0.1175126
ENSG00000273523	0.0009277	0.0018332	0.0000000	0.0041218	0.0000000
NEK3	0.1367975	0.1253233	0.0794502	0.1325931	0.1637255
MRPS31P5	0.1050863	0.0966714	0.0920590	0.0687032	0.1312063
ENSG00000290490	0.0032063	0.0037310	0.0000000	0.0000000	0.0036000
THSD1	0.0047607	0.0109908	0.0056332	0.0043008	0.0257656
VPS36	0.4278693	0.5810826	0.5895202	0.3638013	0.4569082
CKAP2-DT	0.0209147	0.0211542	0.0170985	0.0221982	0.0132043
CKAP2	0.2929198	0.2804746	0.3591976	0.3844512	0.2112498
LINC00345	0.0061988	0.0059401	0.0045673	0.0012937	0.0058520
ENSG00000291255	0.0079658	0.0236967	0.0131294	0.0111131	0.0094206
ENSG00000290856	0.1720203	0.2191097	0.2299051	0.1718355	0.1950035
HNRNPA1L2	0.0473973	0.0669034	0.0414307	0.0315834	0.0713029
SUGT1-DT	0.0077411	0.0022104	0.0047334	0.0053031	0.0046589
SUGT1	1.0964286	1.1024783	1.2152347	1.0019868	0.9079339
CNMD	0.9101024	0.6010715	0.3997650	0.9239237	0.5098727
PCDH8	0.0130337	0.0354264	0.0617534	0.0312182	0.0520109
OLFM4	0.0037642	0.0000000	0.0000000	0.0000000	0.0000000
ENSG00000287722	0.0040617	0.0077314	0.0013609	0.0025857	0.0065921
LINC00458	0.0037905	0.0022032	0.0000000	0.0049681	0.0084081
ENSG00000287460	0.0030537	0.0077063	0.0012756	0.0000000	0.0049773
ENSG00000288765	0.0823798	0.1656196	0.6429628	0.0458860	0.3751133
PCDH17	0.1227675	0.2277233	0.5876982	0.1009657	0.4485242
LINC02338	0.0003469	0.0000000	0.0027102	0.0000000	0.0000000
DIAPH3	0.0475625	0.0263195	0.0295631	0.0439287	0.0428137
TDRD3	0.3555746	0.4044443	0.2970775	0.3519875	0.4274109
LINC00378	0.0000000	0.0000000	0.0007696	0.0037958	0.0119792
PCDH20	0.0028521	0.0044887	0.0097831	0.0064682	0.0130039
LINC00355	0.0011766	0.0000000	0.0023834	0.0000000	0.0000000
PCDH9	0.8586926	1.0029670	1.3838722	0.7890444	2.0906304
PCDH9-AS1	0.0070252	0.0074002	0.0144008	0.0000000	0.0185556
PCDH9-AS2	0.0075466	0.0022394	0.0129092	0.0153652	0.1250128
PCDH9-AS3	0.0016935	0.0000000	0.0021412	0.0000000	0.0083089

PCDH9-AS4	0.0009083	0.0024180	0.0000000	0.0000000	0.0381853
ENSG00000285588	0.0022546	0.0022581	0.0121170	0.0048559	0.0339835
ENSG00000287876	0.0055915	0.0051561	0.0022278	0.0097230	0.0127522
KLHL1	0.0818402	0.1519893	0.2263518	0.0533393	0.4309629
ATXN8OS	0.0133883	0.0124765	0.0355464	0.0000000	0.0249571
LINC00348	0.0067950	0.0050085	0.0070037	0.0106349	0.0000000
DACH1	0.3296113	0.6164160	0.5269349	0.3162000	1.1620585
MZT1	0.3702822	0.4853396	0.5120629	0.3900031	0.3588764
BORA	0.0185884	0.0188315	0.0185228	0.0311193	0.0147497
DIS3	0.4458409	0.3831910	0.4143715	0.3738800	0.3412758
PIBF1	0.4243544	0.4686490	0.3410809	0.3832073	0.6707961
KLF5	0.0777014	0.0655916	0.0555326	0.0581827	0.1034663
LINC00393	0.0018603	0.0000000	0.0095965	0.0099498	0.0194850
KLF12	0.2912475	0.3466532	0.3670741	0.2286706	0.8211923
LINC00402	0.0000000	0.0000000	0.0000000	0.0000000	0.0000000
ENSG00000286330	0.0478482	0.0244538	0.0096495	0.0780041	0.0144730
ENSG00000289644	0.0000000	0.0040855	0.0000000	0.0000000	0.0000000
LINC00381	0.0065961	0.0008350	0.0073082	0.0025371	0.0028907
TBC1D4	0.4162905	0.2497337	0.1355382	0.4331307	0.3423169
COMMD6	1.7722049	1.7806480	1.6700098	1.7605460	1.5774057
UCHL3	0.2529049	0.2899601	0.3728002	0.2729396	0.3604547
LMO7-AS1	0.0021227	0.0017683	0.0000000	0.0025371	0.0142091
LMO7	0.0549718	0.0519648	0.0465500	0.0403495	0.2307293
ENSG00000228444	0.0009723	0.0011527	0.0000000	0.0000000	0.0000000
ENSG00000285572	0.0018563	0.0032094	0.0000000	0.0000000	0.0104516
KCTD12	0.2237366	0.2743265	0.2261910	0.1475332	0.2208023
ENSG00000278727	0.0022924	0.0024796	0.0018800	0.0000000	0.0000000
CLN5	0.3373598	0.2108119	0.1794442	0.5435415	0.2340777
FBXL3	0.3146384	0.2182143	0.2256774	0.3142538	0.2035854
MYCBP2-AS1	0.0186445	0.0302343	0.0224289	0.0150117	0.0982437
ENSG00000274898	0.0011187	0.0000000	0.0000000	0.0000000	0.0000000
MYCBP2	0.6927299	1.0363603	1.1951495	0.5258745	1.3842521
MYCBP2-AS2	0.0009106	0.0000000	0.0000000	0.0000000	0.0000000
SLAIN1	0.2124629	0.2046740	0.1536481	0.1780307	0.3711464
EDNRB-AS1	0.0006378	0.0096816	0.0062592	0.0000000	0.0197931
EDNRB	0.4830568	0.2702635	0.2040390	1.3666891	0.3019725
OBI1-AS1	0.0127840	0.0179897	0.0091484	0.0117910	0.0195838
LINC01069	0.0026652	0.0000000	0.0000000	0.0037736	0.0000000
POU4F1	0.0000000	0.0018697	0.0012308	0.0035651	0.0077598
OBI1	0.3322234	0.4502295	0.5357817	0.3591898	0.3388065
RBM26	0.5744215	0.5054931	0.5420008	0.5504430	0.7938721
RBM26-AS1	0.0814617	0.0717284	0.0548491	0.0534786	0.0851721
NDFIP2	0.4557032	0.3842320	0.3342229	0.4372333	0.3344830
LINC01068	0.0059416	0.0040486	0.0025750	0.0025868	0.0101543
ENSG00000285902	0.0000000	0.0017291	0.0000000	0.0000000	0.0000000
LINC00382	0.0000000	0.0000000	0.0000000	0.0000000	0.0000000
ENSG00000284196	0.0000000	0.0000000	0.0000000	0.0000000	0.0045817
LINC01080	0.0000000	0.0000000	0.0000000	0.0000000	0.0000000
SPRY2	0.1945469	0.1569096	0.1188935	0.3037731	0.1619706
ENSG00000286746	0.0000000	0.0010476	0.0000000	0.0044951	0.0176695
ENSG00000286446	0.0014399	0.0080091	0.0128568	0.0063757	0.0067801
SLITRK1	0.0611487	0.1419502	0.2579183	0.0490723	0.2478459
ENSG00000285680	0.0080372	0.0058278	0.0057787	0.0026219	0.0123922

ENSG00000288016	0.0035288	0.0044410	0.0054440	0.0071049	0.0385699
ENSG00000285834	0.0000000	0.0014800	0.0047477	0.0000000	0.0000000
SLITRK6	0.0142588	0.0306783	0.0466523	0.0154779	0.0162239
ENSG00000285699	0.0006519	0.0007901	0.0070208	0.0000000	0.0037236
MIR4500HG	0.1001286	0.1961679	0.2633125	0.1101765	0.2262402
ENSG00000272515	0.0013171	0.0021865	0.0024931	0.0000000	0.0043076
SLITRK5	0.1568612	0.2343442	0.2831366	0.1715904	0.3977826
LINC02336	0.0024419	0.0000000	0.0000000	0.0000000	0.0022680
MIR17HG	0.0017206	0.0027324	0.0019069	0.0170804	0.0143452
GPC5	0.0602285	0.0755366	0.0735581	0.0517398	0.4572189
ENSG00000282997	0.0000000	0.0000000	0.0000000	0.0000000	0.0037962
GPC5-AS2	0.0002737	0.0000000	0.0000000	0.0000000	0.0152005
ENSG00000287159	0.0000000	0.0005990	0.0024957	0.0000000	0.0061370
GPC5-AS1	0.0011574	0.0000000	0.0000000	0.0000000	0.0000000
ENSG00000278177	0.0022903	0.0022505	0.0000000	0.0111613	0.0000000
GPC6	0.5581057	0.5344395	0.4331006	0.5237578	1.3915051
GPC6-AS2	0.0000000	0.0000000	0.0000000	0.0000000	0.0086215
GPC6-AS1	0.0020249	0.0000000	0.0000000	0.0014497	0.0044834
ENSG00000289204	0.0007385	0.0000000	0.0000000	0.0039970	0.0042436
DCT	0.0187632	0.0064251	0.0056378	0.0130321	0.0065749
ENSG00000289878	0.0074804	0.0028472	0.0000000	0.0038410	0.0053850
TGDS	0.1408517	0.1125149	0.0599571	0.2111075	0.1018560
GPR180	0.1563566	0.1392042	0.1676361	0.1743343	0.1498340
LINC00391	0.0153978	0.0112926	0.0076234	0.0072742	0.0204427
SOX21-AS1	0.1018896	0.0352461	0.0301175	0.0944253	0.0381210
SOX21	0.0932660	0.0509661	0.0444434	0.1267402	0.0564191
ENSG00000287635	0.0000000	0.0000000	0.0044479	0.0000000	0.0000000
ABCC4	0.0455253	0.0604632	0.0575443	0.0139977	0.1415687
CLDN10	0.0000000	0.0006723	0.0060275	0.0031477	0.0197285
DZIP1	0.7844493	0.7948622	0.8236021	0.7104845	0.7134727
DNAJC3-DT	0.2171442	0.1568466	0.1540578	0.1294311	0.1512055
DNAJC3	0.7297156	0.4605164	0.4680028	0.9154617	0.5058912
ENSG00000276809	0.0021798	0.0013018	0.0016034	0.0000000	0.0000000
UGGT2	0.3225458	0.3027585	0.2579484	0.4545896	0.7461957
HS6ST3	0.2882929	0.3669939	0.5446340	0.2125696	1.1168293
OXGR1	0.0003913	0.0038826	0.0000000	0.0030728	0.0068701
MBNL2	0.5074662	0.3569529	0.2065557	0.4591416	0.4878385
ENSG00000286334	0.0000000	0.0010604	0.0000000	0.0000000	0.0000000
ENSG00000286416	0.0058715	0.0159307	0.0052980	0.0178563	0.0350198
RAP2A	0.3658155	0.3558114	0.4102921	0.3983491	0.3209650
ENSG00000276573	0.0000000	0.0000000	0.0006897	0.0000000	0.0000000
ENSG00000276704	0.0014629	0.0000000	0.0000000	0.0000000	0.0000000
IPO5	0.7420282	0.7504771	0.6977660	0.7390439	0.6284074
FARP1	0.8261608	0.9697680	1.1411930	0.8206701	1.0288039
ENSG00000269189	0.0004851	0.0000000	0.0058943	0.0000000	0.0013244
FARP1-AS1	0.0017199	0.0015220	0.0000000	0.0015730	0.0000000
STK24	0.5051660	0.4525701	0.4076567	0.5025737	0.4845516
STK24-AS1	0.0077191	0.0036024	0.0081471	0.0000000	0.0000000
SLC15A1	0.0007906	0.0000000	0.0010824	0.0000000	0.0000000
DOCK9	0.0751578	0.0831641	0.0739871	0.0879844	0.3019020
DOCK9-DT	0.0036390	0.0063081	0.0014595	0.0000000	0.0019545
UBAC2-AS1	0.0805879	0.0367745	0.0524233	0.0670106	0.0509952
UBAC2	0.3360302	0.2301706	0.2215941	0.3916217	0.4080994

GPR18	0.0000000	0.0000000	0.0000000	0.0000000	0.0012623
GPR183	0.0011284	0.0000000	0.0000000	0.0059384	0.0030611
TM9SF2	0.6887962	0.5489491	0.6497654	1.0287946	0.7083106
LINC01232	0.0080182	0.0058203	0.0040474	0.0000000	0.0000000
LINC00449	0.0000000	0.0138375	0.0097520	0.0038782	0.0032715
LINC01039	0.0013268	0.0000000	0.0000000	0.0000000	0.0065891
ENSG00000287746	0.0000000	0.0000000	0.0023230	0.0000000	0.0000000
CLYBL	0.0735286	0.0719211	0.0765665	0.0424338	0.1107812
ENSG00000286757	0.0009085	0.0000000	0.0027927	0.0000000	0.0000000
ZIC5	0.0012178	0.0027982	0.0026961	0.0040025	0.0000000
ZIC2	0.0031987	0.0011635	0.0000000	0.0000000	0.0000000
PCCA-DT	0.1489109	0.1446953	0.0847850	0.1747748	0.0660560
PCCA	0.3809831	0.3650616	0.3047625	0.3503966	0.9859852
ENSG00000287330	0.0000000	0.0038257	0.0000000	0.0000000	0.0069089
PCCA-AS1	0.0000000	0.0055386	0.0000000	0.0000000	0.0096472
GGACT	0.0330984	0.0190267	0.0219710	0.0407152	0.0230215
ENSG00000224356	0.0000000	0.0000000	0.0000000	0.0000000	0.0000000
ENSG00000280169	0.0016482	0.0000000	0.0000000	0.0000000	0.0023002
TMTC4	0.2613992	0.3060292	0.3162587	0.2475745	0.2853632
ENSG00000289594	0.0000000	0.0008925	0.0000000	0.0000000	0.0107816
NALCN-AS1	0.0000000	0.0000000	0.0000000	0.0000000	0.0000000
NALCN	0.1088383	0.1402535	0.1141946	0.0744602	0.4044254
ITGBL1	0.0077444	0.0066587	0.0050488	0.0024483	0.0205871
FGF14	0.4946893	0.6148912	0.4950424	0.4943238	1.5555947
FGF14-IT1	0.0014632	0.0018732	0.0000000	0.0104422	0.0082209
FGF14-AS1	0.0000000	0.0000000	0.0014528	0.0000000	0.0240500
FGF14-AS2	0.1297677	0.0575649	0.0551128	0.1511894	0.0635215
TPP2	0.2312686	0.2042243	0.1947065	0.2303404	0.2175506
CCDC168	0.0032799	0.0000000	0.0020178	0.0025427	0.0207557
TEX30	0.0751749	0.0793859	0.0990489	0.0526967	0.0289797
POGLUT2	0.0669262	0.0464828	0.0319888	0.1122460	0.0476455
BIVM	0.2184510	0.2503263	0.3060394	0.2229089	0.2627555
ERCC5	0.1984899	0.2692363	0.2386213	0.2213455	0.2444813
METTL21EP	0.0056493	0.0060964	0.0031385	0.0011493	0.0044670
LINC00343	0.0000000	0.0000000	0.0000000	0.0000000	0.0000000
ENSG00000287923	0.0000000	0.0000000	0.0008971	0.0000000	0.0081124
ENSG00000286780	0.0000000	0.0000000	0.0000000	0.0000000	0.0000000
EFNB2	0.3862071	0.2496718	0.2355199	0.5245509	0.2154759
ENSG00000284966	0.0056170	0.0036809	0.0021994	0.0012811	0.0000000
ARGLU1	0.8575595	0.8014720	0.6792794	0.8586848	1.0974474
LINC00551	0.0210207	0.0350568	0.0092931	0.0427281	0.0366955
NALF1	0.6919114	0.9285264	0.8146990	0.5732902	2.4072371
ENSG00000276740	0.0044383	0.0004129	0.0022363	0.0000000	0.0000000
NALF1-IT1	0.0033293	0.0040573	0.0019687	0.0013130	0.0380897
ENSG00000286343	0.0037621	0.0062881	0.0000000	0.0091823	0.0208408
ENSG00000274718	0.0008588	0.0015196	0.0055535	0.0109622	0.0064609
LIG4	0.1568318	0.2293510	0.2826713	0.1571034	0.1750424
ABHD13	0.1109015	0.1194004	0.1273029	0.1033996	0.1506663
TNFSF13B	0.0080451	0.0082655	0.0050640	0.0044097	0.0386094
ENSG00000283384	0.0000000	0.0013712	0.0000000	0.0000000	0.0015201
MYO16	0.1926601	0.1920414	0.2656187	0.1598880	0.5189729
MYO16-AS1	0.0011766	0.0000000	0.0000000	0.0000000	0.0024307
ENSG00000289901	3.3384040	3.3899568	3.2471403	3.1316942	3.3957382

ENSG00000285534	0.0040667	0.0097965	0.0000000	0.0000000	0.0203416
IRS2	0.1695255	0.1607959	0.1409780	0.1439346	0.2546314
ENSG00000275741	0.0408399	0.0553154	0.0231845	0.0437408	0.0432155
LINC03082	0.0000000	0.0007209	0.0032369	0.0022149	0.0073662
ENSG00000287575	0.0031768	0.0020782	0.0075323	0.0048943	0.0036367
COL4A1	0.0420522	0.0375320	0.0462124	0.1061063	0.1668221
COL4A2	0.0810708	0.0778431	0.1721646	0.1381410	0.1819192
COL4A2-AS1	0.0000000	0.0000000	0.0000000	0.0000000	0.0000000
RAB20	0.2055211	0.1151405	0.0517529	0.1875997	0.0676990
NAXD-AS1	0.0046788	0.0015711	0.0000000	0.0050023	0.0052853
NAXD	0.2255357	0.1986379	0.1566268	0.2764345	0.1783436
CARS2	0.2810539	0.2802459	0.2807739	0.2585273	0.3072977
ING1	0.2001535	0.1825941	0.1744528	0.1690507	0.1336130
PRECSIT	0.0015394	0.0032311	0.0000000	0.0000000	0.0054309
ANKRD10	0.2581227	0.3085589	0.2618333	0.2860763	0.4691462
ANKRD10-IT1	0.0027463	0.0019105	0.0000000	0.0000000	0.0192531
ENSG00000277767	0.0027209	0.0000000	0.0000000	0.0000000	0.0040093
ENSG00000286824	0.0016816	0.0018636	0.0049279	0.0120500	0.0113907
ARHGEF7-AS2	0.0068283	0.0133903	0.0107375	0.0037638	0.0087777
ARHGEF7	0.3525939	0.5404125	0.5601971	0.2926777	0.7701819
ENSG00000285856	0.0017251	0.0046051	0.0000000	0.0000000	0.0057654
TEX29	0.0009684	0.0033554	0.0012127	0.0000000	0.0204218
ENSG00000204398	0.0000000	0.0000000	0.0012498	0.0000000	0.0128293
LINC00354	0.0297929	0.0197293	0.0070964	0.0323981	0.0481554
SOX1-OT	0.0713346	0.0403314	0.0537295	0.0818752	0.0792171
SOX1	0.0929813	0.0661172	0.1236172	0.1212606	0.0567707
TUBGCP3	0.1675873	0.1860296	0.1670340	0.2177833	0.2932474
ENSG00000277159	0.0017514	0.0010994	0.0036569	0.0027947	0.0069538
ATP11AUN	0.0000000	0.0000000	0.0018505	0.0000000	0.0000000
ENSG00000274922	0.0159162	0.0064878	0.0000000	0.0097007	0.0079501
ATP11A	0.1071891	0.0928588	0.0609166	0.0690889	0.3610921
MCF2L	0.2178511	0.3295918	0.3530928	0.1622862	0.4724091
ENSG00000267868	0.0173619	0.0168165	0.0256133	0.0098250	0.0000000
MCF2L-AS1	0.1105526	0.1067153	0.0995461	0.1269915	0.0516657
ENSG00000289354	0.0009283	0.0030602	0.0015113	0.0000000	0.0000000
F7	0.0464520	0.0479996	0.0262808	0.0327117	0.0424585
F10	0.0405250	0.0157972	0.0225282	0.0287057	0.0584557
ENSG00000283828	0.0012561	0.0018371	0.0027795	0.0000000	0.0054919
PROZ	0.0009205	0.0000000	0.0000000	0.0000000	0.0000000
PCID2	0.2617716	0.2037258	0.2108226	0.2801264	0.2087168
CUL4A	0.3127350	0.2474104	0.2010585	0.2896740	0.3585635
LAMP1	0.8647728	0.5859040	0.6193462	1.3485438	0.5490102
GRTP1	0.0235510	0.0075584	0.0070506	0.0310240	0.0000000
GRTP1-AS1	0.0000000	0.0000000	0.0000000	0.0000000	0.0000000
ADPRHL1	0.0079571	0.0174957	0.0148019	0.0116186	0.0049203
DCUN1D2	0.0595979	0.0705632	0.1010792	0.0293278	0.1618166
DCUN1D2-AS	0.0000000	0.0031645	0.0000000	0.0000000	0.0000000
TMCO3	0.4713198	0.4231846	0.3514947	0.4906380	0.4570081
ENSG00000276248	0.0111997	0.0104986	0.0174961	0.0070149	0.0371358
TFDP1	0.2973805	0.2200958	0.2552651	0.2714523	0.3082221
ATP4B	0.0078067	0.0008551	0.0015069	0.0050638	0.0000000
GRK1	0.0000000	0.0039117	0.0000000	0.0035673	0.0000000
TMEM255B	0.0060629	0.0096935	0.0018021	0.0013512	0.0167770

GAS6-AS1	0.0039871	0.0000000	0.0000000	0.0050554	0.0173679
GAS6	0.3667131	0.2388851	0.2200537	0.5404999	0.2772909
GAS6-DT	0.0144997	0.0167684	0.0202806	0.0080841	0.0047414
SWINGN	0.0000000	0.0000000	0.0000000	0.0000000	0.0031804
C13orf46	0.0015178	0.0000000	0.0013222	0.0018691	0.0000000
RASA3	0.1182301	0.0965497	0.0702052	0.0963509	0.2048874
CFAP97D2	0.0442601	0.0140435	0.0170930	0.0572095	0.0253453
ENSG00000283347	0.0011727	0.0005980	0.0024168	0.0000000	0.0053148
CDC16	0.5434138	0.4620955	0.4339492	0.5257972	0.4693856
ENSG00000289904	0.0000000	0.0000000	0.0000000	0.0000000	0.0049014
ENSG00000285672	0.0000000	0.0000000	0.0000000	0.0000000	0.0065199
UPF3A	0.8299071	0.7190730	0.6992081	0.8713601	0.6555547
CHAMP1	0.3114475	0.3168815	0.3660376	0.2549701	0.2825239
TTC5	0.3511969	0.3251612	0.3722902	0.3736127	0.2886540
ENSG00000258768	0.0103420	0.0096531	0.0097857	0.0054025	0.0189798
CCNB1IP1	0.4709581	0.4899639	0.3096992	0.4767726	0.4473192
ENSG00000259001	0.0166795	0.0180635	0.0066589	0.0127570	0.0322866
PARP2	0.2528229	0.3006336	0.3778207	0.1842082	0.2560002
ENSG00000254846	0.0006761	0.0009758	0.0000000	0.0000000	0.0000000
TEP1	0.0225968	0.0132631	0.0080647	0.0339468	0.0358245
KLHL33	0.0000000	0.0000000	0.0021739	0.0000000	0.0000000
OSGEP	0.1179839	0.0946545	0.0859617	0.1232724	0.1203834
APEX1	0.7283114	0.7347647	0.7556812	0.6924168	0.5264669
PIP4P1	0.2278181	0.2138019	0.2907476	0.1914827	0.1713257
PNP	0.0703586	0.0597737	0.0302891	0.0889850	0.0590678
ANG	0.0826701	0.0525633	0.0452327	0.0670506	0.0418668
RNASE4	0.1261102	0.0989804	0.0739649	0.1608570	0.0811945
EGILA	0.0007963	0.0000000	0.0000000	0.0000000	0.0000000
METTL17	0.1137888	0.0787638	0.0614970	0.1184692	0.0894871
ENSG00000258471	0.0000000	0.0000000	0.0000000	0.0000000	0.0034716
NDRG2	0.2104820	0.1833911	0.1170645	0.2355042	0.1331735
ARHGEF40	0.0924647	0.0775384	0.0495564	0.0975510	0.1704140
ZNF219	0.1283363	0.0841611	0.0868979	0.1418279	0.0766095
TMEM253	0.0100448	0.0097534	0.0019312	0.0038795	0.0150479
ENSG00000178107	0.0000000	0.0000000	0.0000000	0.0000000	0.0015171
LINC00641	0.1529835	0.2296206	0.2208722	0.0958260	0.2557461
HNRNPC	1.9355198	1.8083350	1.8879048	1.9014039	1.6177015
RPGRIP1	0.0012715	0.0012793	0.0000000	0.0000000	0.0101019
SUPT16H	1.0351091	1.0631848	1.1900403	1.0883361	0.8952297
ENSG00000260830	0.0014067	0.0007082	0.0011784	0.0000000	0.0000000
CHD8	0.4622869	0.4683511	0.4975118	0.4255150	0.5092206
RAB2B	0.4168412	0.4502738	0.4280501	0.3740320	0.3107309
TOX4	0.5845375	0.5264423	0.5202432	0.5494404	0.4414249
METTL3	0.1356470	0.1009975	0.0922364	0.1480894	0.1437307
SALL2	0.1635104	0.1523384	0.1231283	0.1782535	0.1491192
ENSG00000257096	0.0010415	0.0013374	0.0000000	0.0043765	0.0055669
TRD-AS1	0.0062242	0.0025263	0.0046770	0.0031257	0.0069538
TRAC	0.0008804	0.0000000	0.0000000	0.0000000	0.0000000
DAD1	0.9932290	0.8549990	0.8426717	1.1719222	0.7862561
ENSG00000275552	0.0029799	0.0000000	0.0000000	0.0031927	0.0000000
ABHD4	0.2159166	0.1188128	0.0930013	0.2522013	0.1177262
OXA1L-DT	0.0516940	0.0396382	0.0431939	0.0781341	0.0866633
OXA1L	0.2550814	0.2731887	0.2502746	0.2727104	0.1994140

SLC7A7	0.0062644	0.0052971	0.0038928	0.0032782	0.0126723
MRPL52	0.6774500	0.7313600	0.8288298	0.7411648	0.5275087
MMP14	0.0984438	0.0650355	0.0465097	0.2603188	0.0664591
LRP10	0.4305441	0.2567926	0.1595023	0.5657115	0.2409558
REM2	0.0375405	0.0734579	0.1050415	0.0361200	0.0493112
RBM23	0.3233103	0.2561905	0.2591174	0.3389354	0.2621655
PRMT5-AS1	0.0033475	0.0038165	0.0100543	0.0000000	0.0129526
PRMT5	0.1980229	0.1886055	0.1455238	0.2305541	0.1121738
PRMT5-DT	0.0073774	0.0163280	0.0121246	0.0045301	0.0290876
HAUS4	0.1152918	0.0709811	0.0482947	0.0897067	0.1029159
AJUBA	0.1151177	0.0419999	0.0184400	0.1043326	0.0352939
AJUBA-DT	0.0325991	0.0182473	0.0088311	0.0484710	0.0218487
C14orf93	0.0524149	0.0560762	0.0514584	0.0553007	0.0501260
PSMB5	1.2019698	1.1168025	1.1846265	1.1989731	0.8585623
CDH24	0.0316552	0.0555254	0.0715113	0.0195844	0.0500677
ENSG00000288795	0.0014961	0.0014991	0.0000000	0.0049220	0.0000000
ACIN1	0.5459484	0.4799850	0.4679382	0.4881606	0.5518413
C14orf119	0.3488710	0.3313423	0.2727647	0.4045345	0.2091888
SLC7A8	0.1524643	0.1104285	0.0682469	0.1852851	0.1035453
RNF212B	0.0023692	0.0011745	0.0018292	0.0000000	0.0195607
ENSG00000289968	0.0000000	0.0022344	0.0000000	0.0056884	0.0000000
HOMEZ	0.0703452	0.0643412	0.0282077	0.0613052	0.0448375
ENSG00000290642	0.0000000	0.0016789	0.0000000	0.0000000	0.0000000
PPP1R3E	0.1722906	0.1578783	0.1421602	0.1081617	0.1813989
BCL2L2	0.0735953	0.0706682	0.0429979	0.0672572	0.0990536
PABPN1	0.8970157	0.8361229	0.9182896	0.9723716	0.8885690
SLC22A17	0.3898101	0.4394473	0.6348077	0.4835534	0.4840701
EFS	0.0072131	0.0072505	0.0021958	0.0163930	0.0000000
MYH7	0.0050682	0.0045056	0.0021306	0.0042806	0.0000000
NGDN	0.2421252	0.2114956	0.2189064	0.2634278	0.1595872
ZFHX2-AS1	0.0061957	0.0042861	0.0069583	0.0071397	0.0290400
ZFHX2	0.0879453	0.1019186	0.0836084	0.0937996	0.1951596
THTPA	0.0045004	0.0015959	0.0013476	0.0000000	0.0000000
AP1G2	0.0352155	0.0216120	0.0164093	0.0259851	0.0422428
AP1G2-AS1	0.0145254	0.0235084	0.0152134	0.0158482	0.0327086
JPH4	0.1455368	0.3739817	0.4704944	0.1140420	0.4101608
ENSG00000274002	0.0028094	0.0017484	0.0000000	0.0088945	0.0056008
DHRS2	0.0037753	0.0035915	0.0086561	0.0000000	0.0000000
LINC00596	0.0000000	0.0000000	0.0000000	0.0000000	0.0037236
DHRS4-AS1	0.3036167	0.2281914	0.1728012	0.2978532	0.1562368
DHRS4	0.0748321	0.0634770	0.0609637	0.0753079	0.0416978
DHRS4L2	0.1642525	0.1368135	0.1296577	0.1398852	0.0923542
ENSG00000286931	0.0021671	0.0094647	0.0067547	0.0055366	0.0287649
ENSG00000291039	0.0000000	0.0010884	0.0000000	0.0000000	0.0000000
CARMIL3	0.0274638	0.0529961	0.0956838	0.0255649	0.1348702
CPNE6	0.0000000	0.0045709	0.0000000	0.0000000	0.0026634
NRL	0.0259224	0.0332378	0.0377668	0.0419418	0.0325013
PCK2	0.1854243	0.0909890	0.0815316	0.1549992	0.0984700
DCAF11	0.1871446	0.2090778	0.1566822	0.2208711	0.2026528
FITM1	0.0000000	0.0000000	0.0000000	0.0000000	0.0000000
PSME1	0.7262891	0.6217600	0.6211181	0.7595438	0.4689337
EMC9	0.1237666	0.1573312	0.1873579	0.1328478	0.1349329
ENSG00000259321	0.0009684	0.0014787	0.0064789	0.0000000	0.0000000

PSME2	0.3792443	0.4248182	0.5455830	0.3353751	0.3117899
RNF31	0.0439339	0.0434327	0.0258990	0.0607828	0.0335814
IRF9	0.1134182	0.0873642	0.0449325	0.1438004	0.0830423
REC8	0.1080324	0.1093092	0.1476299	0.1345383	0.0973025
IPO4	0.0628699	0.0511817	0.0694432	0.0840919	0.0322194
ENSG00000287765	0.0062610	0.0069362	0.0073684	0.0069574	0.0030362
TM9SF1	0.2951513	0.1980008	0.1622597	0.4726459	0.2635473
ENSG00000288820	0.0175755	0.0149626	0.0191600	0.0239209	0.0185716
ENSG00000276698	0.0051324	0.0022809	0.0072805	0.0043135	0.0000000
ENSG00000278784	0.0100162	0.0055935	0.0019963	0.0101887	0.0035158
CHMP4A	0.3957310	0.3117348	0.2804163	0.4212413	0.3037401
MDP1	0.2634917	0.1976072	0.1950485	0.2549579	0.1732901
NEDD8	1.1451076	1.1961123	1.2928744	1.1276716	1.0216925
GMPR2	0.3675222	0.3421729	0.2978383	0.3780388	0.2102316
TINF2	0.1991079	0.1746938	0.1392541	0.2424751	0.1010671
ENSG00000289979	0.0007837	0.0011324	0.0025607	0.0000000	0.0000000
TGM1	0.0017609	0.0025593	0.0000000	0.0013512	0.0083060
RABGGTA	0.0682541	0.0854523	0.0964095	0.0728245	0.0858615
ENSG00000288044	0.1085953	0.0936593	0.0901323	0.1710798	0.0795045
DHRS1	0.1458732	0.1655722	0.1755732	0.1769757	0.0862475
NOP9	0.0374664	0.0373302	0.0461871	0.0116717	0.0349005
LTB4R	0.0061728	0.0072944	0.0008699	0.0011876	0.0207477
ADCY4	0.0000000	0.0007002	0.0000000	0.0000000	0.0018550
NFATC4	0.0150379	0.0178318	0.0100773	0.0125672	0.0333545
NYNRIN	0.0322447	0.0596355	0.0632534	0.0427210	0.0678216
CBLN3	0.0094919	0.0024464	0.0000000	0.0000000	0.0000000
KHNYN	0.0479265	0.0210144	0.0187384	0.0436189	0.0284743
SDR39U1	0.3353906	0.3071664	0.2918322	0.3111462	0.2362665
ENSG00000258744	0.0556392	0.0230665	0.0260292	0.0402326	0.0208878
ENSG00000258657	0.0083121	0.0033301	0.0000000	0.0031857	0.0063779
STXBP6	0.3413859	0.7902969	0.5451929	0.3272137	0.5869831
LINC02306	0.0036569	0.0052140	0.0036427	0.0059567	0.0345721
ENSG00000285633	0.0005155	0.0028462	0.0000000	0.0023258	0.0000000
NOVA1	1.3495533	1.4970323	1.7564775	1.4085604	1.7769469
ENSG00000262119	0.0067109	0.0132129	0.0135470	0.0000000	0.0932199
NOVA1-DT	0.0067613	0.0116291	0.0367876	0.0045256	0.0125163
ENSG00000258081	0.0236583	0.0337179	0.0156995	0.0147015	0.1654626
LINC02294	0.0000000	0.0011466	0.0000000	0.0000000	0.0279735
LINC02293	0.0032782	0.0000000	0.0037557	0.0000000	0.0195931
ENSG00000286479	0.0000000	0.0000000	0.0000000	0.0000000	0.0099007
MIR4307HG	0.0000000	0.0000000	0.0000000	0.0000000	0.0033946
LINC00645	0.0038221	0.0000000	0.0043383	0.0000000	0.0068247
ENSG00000257869	0.0000000	0.0000000	0.0000000	0.0000000	0.0000000
LINC02300	0.0000000	0.0000000	0.0014218	0.0055710	0.0060977
LINC01551	0.0011942	0.0010242	0.0000000	0.0000000	0.0000000
LINC02326	0.0000000	0.0000000	0.0000000	0.0000000	0.0000000
ENSG00000258028	0.0000000	0.0007974	0.0000000	0.0000000	0.0000000
ENSG00000257522	0.0008897	0.0025549	0.0000000	0.0000000	0.0000000
ENSG00000286040	0.0025767	0.0041926	0.0000000	0.0000000	0.0000000
PRKD1	0.1085649	0.0703711	0.0549815	0.0605760	0.1985746
ENSG00000257120	0.0000000	0.0000000	0.0000000	0.0000000	0.0037236
ENSG00000287142	0.0000000	0.0023210	0.0000000	0.0000000	0.0000000
ENSG00000257904	0.0016098	0.0000000	0.0020178	0.0000000	0.0000000

ENSG00000248975	0.0000000	0.0000000	0.0000000	0.0000000	0.0000000
G2E3-AS1	0.0035187	0.0027528	0.0000000	0.0041513	0.0103246
G2E3	0.2988055	0.3119399	0.2906909	0.2707634	0.2961960
SCFD1	0.6092282	0.5897500	0.5795477	0.6051489	0.7516590
ENSG00000258558	0.0004522	0.0038796	0.0000000	0.0028496	0.0019839
COCH	0.0560300	0.1078204	0.1229732	0.0356817	0.0912163
ENSG00000258525	0.0049647	0.0099723	0.0070049	0.0053377	0.0182040
STRN3	0.5843773	0.6386929	0.6207982	0.5373822	0.7216212
AP4S1	0.1188921	0.1528660	0.1799641	0.1153064	0.1184089
HECTD1	0.5924221	0.6412720	0.6556001	0.4735333	0.6839512
ENSG00000257831	0.0060472	0.0072850	0.0000000	0.0170160	0.0000000
HEATR5A	0.3420025	0.2140392	0.1833366	0.3602637	0.2628962
HEATR5A-DT	0.0156028	0.0090279	0.0157844	0.0202324	0.0084071
DTD2	0.2926185	0.2189509	0.1646917	0.2927449	0.2080906
NUBPL	0.1858839	0.1999049	0.1628454	0.2104777	0.4556510
NUBPL-DT	0.0026711	0.0036426	0.0000000	0.0084913	0.0000000
LINC02313	0.0000000	0.0000000	0.0000000	0.0000000	0.0025831
ENSG00000258386	0.0000000	0.0000000	0.0000000	0.0000000	0.0268080
ARHGAP5-AS1	0.1057475	0.0790774	0.0669695	0.1084032	0.0319787
ARHGAP5	1.4510197	1.2151088	1.1409502	1.3601151	1.2388615
ENSG00000285608	0.0128870	0.0108347	0.0054006	0.0252284	0.0155664
ENSG00000286527	0.0158613	0.0088429	0.0109318	0.0071662	0.0124874
AKAP6	0.6051202	0.7564939	0.7073693	0.6052410	1.4896077
ENSG00000258580	0.0000000	0.0039039	0.0000000	0.0000000	0.0410618
ENSG00000289111	0.0086607	0.0063424	0.0072047	0.0159444	0.0114911
NPAS3	0.8665362	0.8200624	0.7756467	0.8191364	1.6174946
ENSG00000287777	0.0000000	0.0000000	0.0000000	0.0000000	0.0000000
EGLN3	1.5322170	0.9067490	0.6168917	1.4400158	0.8377206
SPTSSA	0.5220414	0.3854823	0.3799385	0.5926907	0.3821051
EAPP	0.6209864	0.5252362	0.5455608	0.6261049	0.4396411
SNX6	1.0003027	0.9596318	0.8292309	1.0567881	0.8041034
CFL2	0.7382789	0.7335722	0.7110673	0.7119083	0.5349540
BAZ1A	0.1316947	0.0898680	0.0630787	0.1360156	0.1115349
BAZ1A-AS1	0.0151341	0.0102449	0.0162233	0.0226944	0.0168731
SRP54	0.7308210	0.6326033	0.6005636	0.7749928	0.6061291
FAM177A1	0.8449400	1.0354131	1.0812681	0.9163443	0.8441766
PPP2R3C	0.3538841	0.4045477	0.3976630	0.3541370	0.3569449
PRORP	0.2827448	0.2122543	0.1561416	0.2996223	0.2605174
PSMA6	0.9200907	0.9195633	0.9861275	0.9681591	0.7579236
NFKBIA	0.2881084	0.2520305	0.2525180	0.2258998	0.2148300
RALGAPA1	0.6604702	0.6787205	0.6906368	0.6251195	0.9922860
ENSG00000258938	0.0019159	0.0039207	0.0000000	0.0000000	0.0000000
BRMS1L	0.3129108	0.2704351	0.3467626	0.2811259	0.2248191
ENSG00000257272	0.0009967	0.0000000	0.0000000	0.0000000	0.0000000
ENSG00000258342	0.0033081	0.0051695	0.0000000	0.0000000	0.0147533
LINC00609	0.0023866	0.0018748	0.0000000	0.0000000	0.0117026
PTCSC3	0.0000000	0.0000000	0.0000000	0.0000000	0.0000000
ENSG00000258844	0.0011641	0.0005504	0.0000000	0.0000000	0.0000000
MBIP	0.4053261	0.3581279	0.2907091	0.4237199	0.3193285
ENSG00000283098	0.0085207	0.0107264	0.0121965	0.0101124	0.0257552
SFTA3	0.0000000	0.0000000	0.0000000	0.0000000	0.0000000
SLC25A21	0.0355459	0.0261110	0.0358934	0.0257453	0.0391556
SLC25A21-AS1	0.1651793	0.1029986	0.0960697	0.1475481	0.1216738

MIPOL1	0.2172206	0.1464714	0.1116099	0.1972093	0.4066342
ENSG00000259087	0.0000000	0.0000000	0.0000000	0.0000000	0.0140311
ENSG00000289903	0.0023784	0.0021142	0.0000000	0.0000000	0.0000000
FOXA1	0.7923249	0.4245933	0.2580568	0.8805902	0.3616644
TTC6	0.4778566	0.3982667	0.2402723	0.4405537	0.6428199
LINC00517	0.0000000	0.0000000	0.0000000	0.0000000	0.0110631
ENSG00000258696	0.0000000	0.0000000	0.0000000	0.0000000	0.0000000
ENSG00000259048	0.0019535	0.0020943	0.0022183	0.0000000	0.0039429
ENSG00000258649	0.0000000	0.0000000	0.0000000	0.0000000	0.0000000
SSTR1	0.0110277	0.0471475	0.0206354	0.0101160	0.0406581
CLEC14A	0.0000000	0.0000000	0.0000000	0.0041335	0.0000000
ENSG00000259072	0.0008153	0.0026305	0.0014397	0.0000000	0.0000000
LINC00639	0.0005120	0.0014407	0.0000000	0.0000000	0.0158503
SEC23A	0.3418063	0.4046483	0.3980399	0.3769919	0.3899505
SEC23A-AS1	0.0035708	0.0008701	0.0031626	0.0000000	0.0000000
GEMIN2	0.2132793	0.2125950	0.1826023	0.2223062	0.2171931
TRAPPC6B	0.3118342	0.4437861	0.4607933	0.2766204	0.2986071
ENSG00000285830	0.0386688	0.0699247	0.0786382	0.0231183	0.0548675
PNN	0.9304077	0.9900127	0.9102607	0.8415924	0.9502788
MIA2	0.2460876	0.1804238	0.1791194	0.2217424	0.2684223
MIA2-AS1	0.0163258	0.0128188	0.0127972	0.0060660	0.0039740
FBXO33	0.0954498	0.0918282	0.0982624	0.0855429	0.1114147
ENSG00000258526	0.1214643	0.1516951	0.1312108	0.1133591	0.7247766
ENSG00000286123	0.0006996	0.0000000	0.0021434	0.0038626	0.0000000
ENSG00000286099	0.0000000	0.0000000	0.0000000	0.0000000	0.0000000
LINC02315	0.0923935	0.0798448	0.0308877	0.0442292	0.3210634
LRFN5-DT	0.0226453	0.0316776	0.0441819	0.0169479	0.0412880
LRFN5	0.2646786	0.4365058	0.4828892	0.1865445	1.1608389
ENSG00000258394	0.0022308	0.0034995	0.0000000	0.0046718	0.0326631
ENSG00000258850	0.0000000	0.0000000	0.0000000	0.0000000	0.0022383
LINC02307	0.0066726	0.0074965	0.0015197	0.0097927	0.0225710
LINC02277	0.0000000	0.0000000	0.0000000	0.0000000	0.0000000
LINC02302	0.0006717	0.0000000	0.0000000	0.0000000	0.0000000
RRAGAP1-AS1	0.0076897	0.0046669	0.0044663	0.0088569	0.0000000
C14orf28	0.0590776	0.0491969	0.0433309	0.0664305	0.0456556
ENSG00000258949	0.0016062	0.0006583	0.0000000	0.0000000	0.0000000
KLHL28	0.3429453	0.3244308	0.2919369	0.3201858	0.3135582
TOGARAM1	0.2826461	0.2583691	0.2069715	0.2654381	0.4263525
PRPF39-DT	0.0006437	0.0023718	0.0000000	0.0000000	0.0000000
PRPF39	0.1930078	0.1855114	0.2020124	0.1594429	0.2652344
FKBP3	1.0360257	1.1749337	1.3892932	1.0770245	0.8612054
FANCM	0.0368548	0.0301006	0.0257499	0.0219164	0.0784816
MIS18BP1	0.0262136	0.0541086	0.0461061	0.0296052	0.0427208
LINC02303	0.0008702	0.0000000	0.0030415	0.0000000	0.0000000
LINC00871	0.0063312	0.0042862	0.0048395	0.0000000	0.0083325
MDGA2	0.4140705	0.5466905	0.4741442	0.3119274	1.7200898
LINC00648	0.0046677	0.0164807	0.0190035	0.0134202	0.0169220
ENSG00000289195	0.0000000	0.0000000	0.0000000	0.0000000	0.0000000
ENSG00000258751	0.0000000	0.0000000	0.0015036	0.0000000	0.0030378
ENSG00000258868	0.0008858	0.0000000	0.0000000	0.0000000	0.0000000
RPS29	2.5146364	2.5471761	2.3758337	2.3828239	2.3055631
LRR1	0.0441567	0.0341897	0.0222701	0.0577024	0.0188793
RPL36AL	1.2188941	1.2150362	1.0946595	1.0286337	1.0287447

MGAT2	0.1917173	0.1485437	0.1617834	0.2398791	0.1239368
ENSG00000258377	0.0054932	0.0038136	0.0018514	0.0000000	0.0038113
DNAAF2	0.2386809	0.2174538	0.2739367	0.2690403	0.1867657
ENSG00000258450	0.0000000	0.0000000	0.0000000	0.0000000	0.0000000
POLE2	0.0175849	0.0093277	0.0319418	0.0296786	0.0387476
KLHDC1	0.0882098	0.0568616	0.0848221	0.0546507	0.1099950
ENSG00000258400	0.0005236	0.0000000	0.0000000	0.0000000	0.0000000
KLHDC2	0.5331994	0.5092477	0.5595056	0.5275810	0.4062335
NEMF	0.6674381	0.6123570	0.5704669	0.6367017	0.6494138
ENSG00000282885	0.2943071	0.2479623	0.1629274	0.2573286	0.2419576
ENSG00000278002	0.0017787	0.0053227	0.0000000	0.0000000	0.0117975
ARF6	0.3050490	0.3237280	0.3186671	0.2720930	0.1704282
LINC01588	0.0072977	0.0190027	0.0122058	0.0153927	0.1153952
VCPKMT	0.0943329	0.1515300	0.1511008	0.0913124	0.1241230
SOS2	0.3346158	0.3657771	0.3190450	0.3288955	0.4969555
L2HGDH	0.0651442	0.0826141	0.0917485	0.0475007	0.1546779
DMAC2L	0.5065082	0.3960407	0.3242802	0.5005390	0.3693904
CDKL1	0.1326659	0.1597772	0.1073662	0.1265864	0.3538665
MAP4K5	0.4955709	0.5414185	0.5318477	0.5271049	0.8768421
ENSG00000259113	0.0017908	0.0012047	0.0000000	0.0055207	0.0079395
ATL1	0.4866920	0.6594425	0.8143529	0.5366790	0.6226271
SAV1	0.3950326	0.3144442	0.2587133	0.3294734	0.3122781
ENSG00000269906	0.0014697	0.0039804	0.0050901	0.0000000	0.0119572
ENSG00000289866	0.0084255	0.0063380	0.0038928	0.0023230	0.0120381
NIN	0.2881802	0.2625583	0.1720885	0.2384021	0.5388896
ENSG00000270062	0.0000000	0.0011008	0.0000000	0.0024545	0.0065348
ENSG00000285747	0.0005428	0.0017861	0.0029843	0.0059751	0.0024068
PYGL	0.0508891	0.0304340	0.0161421	0.0626543	0.0075647
ABHD12B	0.0003025	0.0009875	0.0060988	0.0000000	0.0032941
ENSG00000258711	0.0007020	0.0005333	0.0013083	0.0000000	0.0282855
TRIM9	0.4797672	0.3919050	0.3654593	0.4235918	0.5639891
ENSG00000287990	0.0000000	0.0000000	0.0000000	0.0019339	0.0000000
TMX1	0.3191633	0.2383250	0.1692747	0.4933775	0.2337283
LINC00519	0.0000000	0.0000000	0.0000000	0.0000000	0.0059744
LINC00640	0.0000000	0.0009278	0.0022870	0.0000000	0.0000000
FRMD6-AS2	0.0124908	0.0075267	0.0043686	0.0082676	0.0452387
FRMD6	0.0466132	0.0240518	0.0256017	0.0466352	0.0658766
GNG2	1.0057628	1.5043413	1.8693401	0.8551963	1.3132455
ENSG00000259007	0.0056360	0.0165655	0.0142718	0.0078740	0.0145016
ENSG00000258854	0.0000000	0.0000000	0.0000000	0.0000000	0.0000000
RTRAF	0.9120458	0.8630350	0.8922216	0.9067531	0.7468202
NID2	0.0554077	0.0198429	0.0202998	0.1494424	0.0310044
LINC02319	0.0000000	0.0009383	0.0024882	0.0000000	0.0000000
ENSG00000289424	0.0060821	0.0124345	0.0047016	0.0313515	0.0016619
PTGER2	0.1267909	0.0611295	0.0570289	0.2735873	0.0978549
TXNDC16	0.2464311	0.2203347	0.2019444	0.3266393	0.3589133
GPR137C	0.1863287	0.3117576	0.3105510	0.1547286	0.5911063
ERO1A	0.4445484	0.3570917	0.3854638	0.4246486	0.4806319
ENSG00000258757	0.0889497	0.0741421	0.0561686	0.0574410	0.1103875
PSMC6	0.8010752	0.7551918	0.8283940	0.7511005	0.7106565
STYX	0.2166174	0.2144585	0.2501941	0.2025108	0.2090375
GNPNAT1	0.2142407	0.1968899	0.2142328	0.2143584	0.1369628
ENSG00000259049	0.0016264	0.0018567	0.0000000	0.0022801	0.0000000

ENSG00000285664	0.0168310	0.0101117	0.0056352	0.0193325	0.0850816
ENSG00000258698	0.0000000	0.0000000	0.0000000	0.0000000	0.0000000
FERMT2	0.4548169	0.3840175	0.2509057	0.5330711	0.4156523
ENSG00000288045	0.0101738	0.0058131	0.0017443	0.0023588	0.0125551
DDHD1	0.2275875	0.3281554	0.3403153	0.2092022	0.3917002
DDHD1-DT	0.0007188	0.0022194	0.0011570	0.0000000	0.0000000
ENSG00000237356	0.0135419	0.0197972	0.0263185	0.0089531	0.0540731
ENSG00000286730	0.0000000	0.0000000	0.0000000	0.0000000	0.0000000
LINC02331	0.0010237	0.0000000	0.0000000	0.0028371	0.0000000
BMP4	0.0050289	0.0036655	0.0000000	0.0076999	0.0000000
CDKN3	0.0301865	0.0453181	0.0613755	0.0341120	0.0278734
CNIH1	0.7499015	0.6694246	0.7048335	0.7469436	0.5925198
GMFB	0.4659528	0.4714312	0.3852033	0.4568624	0.3027664
CGRRF1	0.2414794	0.2086537	0.2330303	0.2981021	0.1513502
SAMD4A	0.2055393	0.2029896	0.1338453	0.1696898	0.4640237
SAMD4A-AS1	0.0025037	0.0011519	0.0000000	0.0000000	0.0033906
GCH1	0.0539151	0.1107240	0.3187612	0.0405009	0.1598826
WDHD1	0.0578032	0.0367477	0.0577446	0.0647825	0.0496114
SOCS4	0.4630514	0.4218305	0.4577555	0.3659739	0.3238769
MAPK1IP1L	0.7396808	0.6550358	0.6309088	0.7667242	0.6355166
ENSG00000289523	0.0127040	0.0137759	0.0089424	0.0180691	0.0142253
LGALS3	0.0471243	0.0307754	0.0090070	0.0381971	0.0331969
DLGAP5	0.0137114	0.0158058	0.0104265	0.0112058	0.0220768
FBXO34-AS1	0.0056523	0.0020753	0.0013618	0.0000000	0.0238752
FBXO34	0.2669002	0.4004701	0.3938663	0.2470953	0.4304799
ENSG00000258455	0.0028840	0.0010495	0.0000000	0.0057816	0.0048241
ATG14	0.1781155	0.1972602	0.1550945	0.1723039	0.1737970
TBPL2	0.0801257	0.0317661	0.0229039	0.0849030	0.0318779
KTN1-AS1	0.1114463	0.1167383	0.1045117	0.0873056	0.1310886
KTN1	1.5931103	1.3013936	1.3060769	1.7216671	1.3467049
ENSG00000258784	0.0005367	0.0042004	0.0000000	0.0021110	0.0174987
ENSG00000259868	0.0143896	0.0120774	0.0048581	0.0121748	0.0266402
ENSG00000277763	0.0230003	0.0089422	0.0018415	0.0154410	0.0060333
PELI2	0.2923413	0.2235007	0.2209620	0.2207622	0.5061627
ENSG00000259483	0.0009743	0.0000000	0.0026511	0.0000000	0.0086120
ENSG00000275569	0.0031738	0.0049713	0.0035187	0.0000000	0.0021172
LINC02284	0.0152628	0.0107952	0.0010362	0.0069472	0.0362934
TMEM260	0.1015396	0.0980582	0.0821671	0.1353768	0.2328875
ENSG00000258428	0.0026413	0.0023276	0.0011369	0.0000000	0.0045646
ENSG00000259133	0.0016468	0.0027587	0.0000000	0.0000000	0.0000000
OTX2-AS1	0.0037238	0.0021814	0.0039579	0.0000000	0.0000000
ENSG00000258776	0.0000000	0.0000000	0.0027683	0.0000000	0.0000000
ENSG00000286257	0.0021338	0.0035714	0.0000000	0.0000000	0.0029535
LINC03059	0.0089664	0.0036141	0.0014518	0.0028736	0.0000000
EXOC5	0.7358279	0.6998619	0.7512661	0.7147307	0.6293941
AP5M1	0.3542640	0.4142695	0.3932674	0.3487767	0.3564335
ENSG00000286355	0.0000000	0.0011157	0.0024411	0.0000000	0.0122138
NAA30	0.2542586	0.2808674	0.3773769	0.1978657	0.2548272
CCDC198	0.1642441	0.0737047	0.0487430	0.0709161	0.0863346
SLC35F4	0.1220406	0.1778145	0.1540331	0.1295488	0.7320718
ENSG00000259039	0.0046769	0.0078287	0.0024339	0.0044469	0.0105455
ENSG00000259969	0.0483371	0.0501990	0.0244796	0.0183728	0.0196924
ARMH4	0.4065975	0.3533999	0.3478185	0.4708306	0.5180995

ACTR10	0.7766250	0.7857156	0.9414984	0.7185582	0.7404439
PSMA3	0.8382441	0.7147192	0.7053798	0.8869282	0.5491192
ENSG00000258682	0.0000000	0.0000000	0.0000000	0.0000000	0.0030021
PSMA3-AS1	0.1777563	0.2042162	0.1577058	0.2211966	0.2394419
ARID4A	0.5767592	0.6485191	0.8370009	0.5488990	0.7333669
TOMM20L-DT	0.0035042	0.0026318	0.0015089	0.0000000	0.0030343
TOMM20L	0.0015246	0.0031436	0.0000000	0.0000000	0.0037844
ENSG00000258378	0.0062102	0.0055138	0.0000000	0.0000000	0.0184417
TIMM9	0.3861710	0.4022172	0.4054494	0.3581098	0.3530803
KIAA0586	0.2050790	0.1948553	0.1966073	0.2294752	0.2980905
DACT1	0.0359887	0.0797181	0.1064951	0.0187161	0.1025801
LINC01500	0.0075957	0.0070411	0.0054248	0.0069537	0.0124786
DAAM1	0.9085543	1.1821636	1.3917012	0.7745742	1.2491055
GPR135	0.0944724	0.0661138	0.1241515	0.0921619	0.2211088
L3HYPDH	0.1411791	0.0845252	0.0799397	0.1386207	0.1052659
JKAMP	0.4643950	0.3414388	0.3269878	0.6693478	0.3444784
CCDC175	0.0249613	0.0112238	0.0240744	0.0168355	0.0564898
RTN1	1.4548355	2.0214772	2.4191181	1.4985795	1.9360681
ENSG00000261120	0.0044045	0.0029157	0.0026308	0.0019116	0.0017223
LRRC9	0.2797539	0.1811835	0.0710063	0.3127658	0.2301285
PCNX4-DT	0.0099184	0.0091440	0.0199831	0.0108757	0.0198194
PCNX4	0.3967234	0.3936558	0.4595241	0.4030989	0.6237116
DHRS7	0.5743332	0.5519955	0.5524502	0.8119280	0.4249596
ENSG00000254718	0.0011418	0.0012856	0.0000000	0.0000000	0.0000000
PPM1A	0.4220950	0.5405603	0.5433907	0.3740300	0.4677702
C14orf39	0.0664696	0.0715734	0.0457797	0.0495397	0.1371893
SIX4	0.0200293	0.0211798	0.0133805	0.0246734	0.0208312
MNAT1	0.5743689	0.4692325	0.3686862	0.4758437	0.7780988
TRMT5	0.2388251	0.1782734	0.2111589	0.2089601	0.1457231
SLC38A6	0.1759664	0.1967482	0.1437947	0.1589135	0.2888683
PRKCH	0.0097374	0.0033721	0.0044056	0.0090307	0.0645002
TMEM30B	0.0030012	0.0000000	0.0012725	0.0000000	0.0000000
PRKCH-AS1	0.0000000	0.0012598	0.0000000	0.0000000	0.0016028
LINC03033	0.0006048	0.0000000	0.0000000	0.0000000	0.0000000
HIF1A-AS1	0.0012158	0.0017972	0.0000000	0.0000000	0.0000000
HIF1A	0.7783571	0.6166425	0.6721443	0.8150356	0.6086834
HIF1A-AS3	0.0967613	0.0886867	0.0391837	0.0842969	0.2619183
ENSG00000288928	0.0037228	0.0027943	0.0000000	0.0000000	0.0168950
SNAPC1	0.4082808	0.2582074	0.1433729	0.4975155	0.3279188
SYT16	0.2301937	0.4697481	0.4927098	0.1791010	0.7085074
ENSG00000258842	0.0000000	0.0000000	0.0000000	0.0000000	0.0000000
ENSG00000258903	0.0000000	0.0054015	0.0031894	0.0000000	0.0021475
ENSG00000288802	0.0076053	0.0194212	0.0230828	0.0058699	0.0155216
KCNH5	0.1818168	0.1694762	0.1389084	0.2305104	0.2418080
RHOJ	0.4516271	0.2141712	0.1278962	0.6278822	0.2423403
ENSG00000258943	0.0016411	0.0000000	0.0000000	0.0000000	0.0000000
GPHB5	0.0000000	0.0000000	0.0000000	0.0000000	0.0000000
PPP2R5E	0.8213653	0.8885172	0.8576274	0.8235384	0.8690796
ENSG00000261242	0.0131157	0.0108508	0.0184522	0.0020875	0.0105301
WDR89	0.1643761	0.1043944	0.0955966	0.1438838	0.1581451
ENSG00000274015	0.0407922	0.0294279	0.0174060	0.0296312	0.0179782
SGPP1	0.0878098	0.0902202	0.0649668	0.1243172	0.1382477
SYNE2	0.1418026	0.1567117	0.1289371	0.1416513	0.3183943

ESR2	0.0185322	0.0102635	0.0059850	0.0169916	0.0986374
ENSG00000214770	0.0038520	0.0042425	0.0032318	0.0000000	0.0097703
ENSG00000284664	0.0000000	0.0046434	0.0009612	0.0033148	0.0052305
MTHFD1	0.1285119	0.1174248	0.1288016	0.1336413	0.1372544
ENSG00000258824	0.0541737	0.0711947	0.0736078	0.0350898	0.0776523
ENSG00000272909	0.0031187	0.0030350	0.0034328	0.0067070	0.0157293
ZBTB25	0.1392990	0.1707081	0.1969108	0.1206391	0.2062032
AKAP5	0.0476301	0.1103206	0.2079194	0.0192291	0.0629330
ZBTB1	0.3467376	0.2876285	0.2047840	0.3259929	0.3464762
HSPA2-AS1	0.0120942	0.0024348	0.0013814	0.0066849	0.0140674
HSPA2	0.0228451	0.0167069	0.0069693	0.0344891	0.0115976
PPP1R36	0.0149029	0.0035604	0.0025799	0.0043212	0.0029896
ENSG00000259076	0.0000000	0.0000000	0.0015036	0.0044812	0.0000000
PLEKHG3	0.0311111	0.0280326	0.0047493	0.0116115	0.0202511
SPTB	0.0376914	0.0913303	0.0747744	0.0247931	0.1001405
CHURC1	0.9145202	0.8042434	0.7386441	0.9549041	0.5662927
GPX2	0.0010584	0.0013343	0.0000000	0.0000000	0.0042796
RAB15	0.1477135	0.3200838	0.3000334	0.1384436	0.1313980
FNTB	0.1484691	0.1468448	0.1397830	0.1741811	0.2242088
ENSG00000272158	0.0042103	0.0026623	0.0035092	0.0000000	0.0126169
MAX	0.3965885	0.4535095	0.5434945	0.3368415	0.4337442
ENSG00000259118	0.0022056	0.0013333	0.0027481	0.0025868	0.0111679
ENSG00000258760	0.0000000	0.0020473	0.0011938	0.0000000	0.0000000
FUT8	0.3923483	0.4558897	0.4100853	0.3801707	0.6905066
FUT8-AS1	0.0153336	0.0197295	0.0089455	0.0085613	0.0169215
ENSG00000258847	0.0089776	0.0080088	0.0095532	0.0018770	0.0055453
ENSG00000258561	0.0353645	0.0482928	0.0251680	0.0560311	0.1961734
ENSG00000287833	0.0000000	0.0000000	0.0000000	0.0000000	0.0000000
CCDC196	0.0000000	0.0036192	0.0022883	0.0000000	0.0000000
GPHN	0.5649934	0.6462428	0.5577864	0.5186716	1.6872133
ENSG00000289208	0.0000000	0.0033432	0.0000000	0.0029154	0.0109799
ENSG00000258490	0.0000000	0.0000000	0.0000000	0.0000000	0.0039679
GARIN2	0.0025664	0.0085238	0.0000000	0.0034082	0.0158382
PALS1	0.3555201	0.2366891	0.2342740	0.3654302	0.2725700
ENSG00000286319	0.0024395	0.0007788	0.0040533	0.0079735	0.0037447
ATP6V1D	1.0079988	1.0173630	1.0986043	0.9407270	0.7541970
EIF2S1	0.5148215	0.5419977	0.5444673	0.4739925	0.4871011
PLEK2	0.0000000	0.0000000	0.0000000	0.0029465	0.0000000
TMEM229B	0.0702613	0.0963714	0.1285424	0.0790996	0.0748445
PLEKHH1	0.0700351	0.0353785	0.0361243	0.0458613	0.1139371
PIGH	0.2394179	0.2281140	0.1617229	0.2621757	0.1241168
ARG2	0.2091478	0.4135699	0.5698435	0.1737613	0.2916016
ENSG00000286861	0.0133578	0.0149193	0.0164439	0.0056221	0.0358419
VTI1B	0.9397855	1.0214341	1.1085736	0.8847774	0.8447530
RDH11	0.3902797	0.3567791	0.4528966	0.5401301	0.4738448
RDH12	0.0049750	0.0042806	0.0060506	0.0000000	0.0073097
ZFYVE26	0.0306385	0.0312272	0.0337767	0.0373449	0.0668403
RAD51B	0.1522193	0.1568253	0.0822708	0.0876584	0.4731439
ENSG00000258837	0.0000000	0.0000000	0.0000000	0.0000000	0.0000000
ENSG00000259038	0.0035042	0.0024646	0.0000000	0.0016936	0.0000000
ENSG00000287913	0.0014145	0.0000000	0.0010540	0.0000000	0.0019716
ZFP36L1	1.4591992	0.9733139	0.6110180	1.4132509	0.9029831
ACTN1	0.2873997	0.1890118	0.1418539	0.3407175	0.2172453

ACTN1-DT	0.0009071	0.0000000	0.0000000	0.0000000	0.0079085
DCAF5	0.3533601	0.3085816	0.3249065	0.3015140	0.4043072
ENSG00000289180	0.0037194	0.0000000	0.0000000	0.0086558	0.0049425
ENSG00000276063	0.0000000	0.0004377	0.0000000	0.0000000	0.0000000
GALNT16-AS1	0.0139191	0.0286466	0.0114186	0.0070205	0.0013524
EXD2	0.1656881	0.1404161	0.1200459	0.1804228	0.1956116
ENSG00000258520	0.0221077	0.0422264	0.0204070	0.0175781	0.0394158
GALNT16	0.1209147	0.1195801	0.1278541	0.2008721	0.1523712
ERH	1.2996169	1.2692227	1.3606755	1.3235414	1.0057652
SLC39A9	0.1718687	0.1719095	0.1799792	0.1649667	0.3099700
PLEKHD1	0.0136809	0.0255505	0.0417933	0.0227007	0.0582151
CCDC177	0.0163221	0.0155027	0.0159096	0.0109438	0.0196040
ENSG00000287310	0.0117362	0.0046256	0.0084835	0.0000000	0.0036060
SUSD6	0.1287897	0.0986412	0.0730587	0.1475106	0.1798503
ENSG00000273797	0.0002454	0.0023036	0.0000000	0.0000000	0.0000000
SRSF5	1.0619736	0.9044406	0.8759804	0.9265693	0.8612812
SLC10A1	0.0000000	0.0017291	0.0039224	0.0000000	0.0106476
SMOC1	0.4666979	0.2320060	0.1487776	0.4904625	0.3524657
SLC8A3	0.0592972	0.0508206	0.0635452	0.0251785	0.2543243
ENSG00000258422	0.0027933	0.0071389	0.0068473	0.0031877	0.0351943
ENSG00000259033	0.0031756	0.0075223	0.0039418	0.0025617	0.0251723
COX16	0.9719296	0.9341156	1.0212797	0.9792896	0.6870441
SYNJ2BP	0.5110905	0.4327285	0.4625226	0.4178582	0.4315716
ADAM21	0.0019017	0.0026559	0.0011270	0.0000000	0.0063839
ENSG00000257759	0.0350582	0.0474394	0.0483047	0.0230219	0.1116381
ADAM20P1	0.0018600	0.0065671	0.0060357	0.0114246	0.0281740
ADAM20	0.0016510	0.0014323	0.0011907	0.0000000	0.0000000
MED6	0.3132277	0.3154042	0.3872914	0.3206148	0.2954247
TTC9-DT	0.0032691	0.0020001	0.0060686	0.0000000	0.0150700
TTC9	0.1439998	0.2713393	0.3106285	0.1920430	0.2022593
LINC01269	0.0000000	0.0000000	0.0000000	0.0000000	0.0027080
MAP3K9	0.1000512	0.1830055	0.1434795	0.1149785	0.2776771
MAP3K9-DT	0.1366065	0.1967140	0.2937023	0.0896526	0.1796739
ENSG00000275630	0.0010222	0.0030689	0.0035351	0.0019281	0.0044531
ENSG00000274818	0.0135072	0.0079045	0.0056273	0.0054376	0.0000000
PCNX1	0.3107605	0.3394433	0.3409736	0.2497452	0.7343075
ENSG00000287643	0.0011401	0.0000000	0.0000000	0.0000000	0.0075681
ENSG00000269927	0.0000000	0.0011535	0.0000000	0.0000000	0.0000000
SIPA1L1-AS1	0.0261302	0.0207978	0.0052681	0.0326501	0.0077539
SIPA1L1	0.2476872	0.2483475	0.1720402	0.2420630	0.4010166
ENSG00000285612	0.0015884	0.0020398	0.0007063	0.0000000	0.0019545
ENSG00000266869	0.0000000	0.0013478	0.0000000	0.0000000	0.0069257
RGS6	0.4942183	0.3512557	0.2487561	0.4541104	0.6216764
ENSG00000286333	0.0007632	0.0023935	0.0000000	0.0063752	0.0197219
ENSG00000258871	0.0011084	0.0000000	0.0000000	0.0000000	0.0110630
DPF3	0.0729176	0.1223296	0.0970047	0.0533352	0.1788704
DCAF4	0.0438800	0.0551821	0.0480317	0.0548032	0.0420840
ZFYVE1	0.0860105	0.0837074	0.0889916	0.0754768	0.0901156
RBM25-AS1	0.0145233	0.0048305	0.0150028	0.0136857	0.0019688
RBM25	1.0080780	0.9188441	0.8745772	0.8138083	1.2314057
PSEN1	0.1540009	0.1306510	0.1268766	0.1908816	0.2408470
PAPLN	0.0025736	0.0028269	0.0025496	0.0069739	0.0025600
ENSG00000258944	0.0010363	0.0000000	0.0000000	0.0046019	0.0041331

NUMB	0.2691748	0.2079917	0.1449812	0.2705334	0.3904058
ENSG00000284930	0.0000000	0.0000000	0.0000000	0.0000000	0.0000000
ENSG00000251393	0.0032583	0.0000000	0.0000000	0.0000000	0.0000000
HEATR4	0.0090730	0.0087388	0.0035531	0.0095683	0.0145352
RIOX1	0.0736966	0.0790688	0.0936322	0.0973526	0.0462113
ENSG00000258695	0.0067669	0.0124949	0.0062870	0.0072328	0.0149221
ACOT1	0.0005478	0.0036386	0.0000000	0.0000000	0.0130615
ENSG00000258603	0.0363964	0.0439824	0.0526751	0.0322022	0.0523574
ACOT2	0.0624502	0.0521816	0.0620000	0.0713760	0.0288856
ACOT6	0.0018800	0.0012258	0.0014218	0.0000000	0.0000000
DNAL1	0.4722570	0.3735385	0.3250219	0.5076073	0.3349048
PNMA1	0.9047990	0.9323356	1.0498978	0.9296632	0.6492565
MIDEAS	0.1937683	0.1822523	0.1342497	0.1110120	0.2560128
MIDEAS-AS1	0.0925486	0.0516339	0.0169466	0.0868841	0.0472780
PTGR2	0.3217823	0.2289972	0.1252803	0.3099757	0.1926465
ENSG00000273711	0.0203504	0.0163094	0.0070265	0.0128459	0.0142874
ZNF410	0.2055444	0.1746484	0.1366345	0.2503519	0.2063061
ENSG00000258891	0.0235879	0.0230412	0.0246648	0.0106101	0.0554594
ENSG00000270140	0.0003085	0.0010193	0.0000000	0.0000000	0.0000000
FAM161B	0.2694760	0.2578729	0.2666313	0.2642585	0.1841584
COQ6	0.0990303	0.1102539	0.1095094	0.0916818	0.1288124
ENTPD5	0.1192267	0.0809456	0.0733123	0.1544264	0.1041304
BBOF1	0.3511584	0.2971993	0.2315473	0.2991570	0.3092408
ALDH6A1	0.4744706	0.3057940	0.2884172	0.5642174	0.3392084
LIN52	0.1232539	0.1106929	0.1624235	0.1258494	0.1666782
VSX2	0.0000000	0.0050941	0.0057404	0.0000000	0.0000000
ABCD4	0.1796655	0.0985431	0.1040075	0.2512279	0.1684804
ENSG00000258559	0.0011401	0.0000000	0.0000000	0.0023531	0.0015298
VRTN	0.0016098	0.0000000	0.0019437	0.0000000	0.0044438
SYNDIG1L	0.0086328	0.0108573	0.0200169	0.0055335	0.0039758
NPC2	1.3692001	1.0263320	0.8092078	1.6329152	0.9647580
ISCA2	0.4309503	0.3206857	0.2838121	0.4449650	0.2606072
LTBP2	0.0039233	0.0000000	0.0000000	0.0000000	0.0033906
AREL1	0.1094306	0.1761444	0.2427581	0.1225079	0.2357528
FCF1	0.2693250	0.2592546	0.2384132	0.2432102	0.1836932
YLPM1	0.3463485	0.4522507	0.3735156	0.3038128	0.6676534
PROX2	0.0000000	0.0000000	0.0000000	0.0000000	0.0000000
DLST	0.1830517	0.2091867	0.1668943	0.1819469	0.2048787
RPS6KL1	0.0542676	0.0955465	0.0868210	0.0486508	0.1560301
PGF	0.0263428	0.0145152	0.0142998	0.0272123	0.0150046
EIF2B2	0.2455687	0.1980019	0.2362662	0.1879662	0.1307470
ENSG00000258646	0.0046309	0.0007729	0.0008339	0.0022792	0.0069505
MLH3	0.3214619	0.2557226	0.2366145	0.2994526	0.3331332
ACYP1	0.4609542	0.5832682	0.7183539	0.4615603	0.4212433
ZC2HC1C	0.1014081	0.0740974	0.0613442	0.1014620	0.0985775
NEK9	0.1863305	0.1554047	0.1123060	0.1718104	0.2038951
ENSG00000259138	0.0010423	0.0000000	0.0000000	0.0000000	0.0069273
TMED10	1.0197044	0.8240886	0.8159060	1.3487731	0.7925703
ENSG00000273565	0.0015514	0.0014148	0.0034078	0.0010320	0.0000000
ENSG00000258740	0.0010193	0.0019801	0.0020652	0.0000000	0.0186876
ENSG00000289340	0.0187935	0.0145010	0.0154471	0.0159958	0.0312942
ENSG00000258820	0.0032940	0.0011391	0.0025623	0.0000000	0.0021475
FOS	1.1136736	0.9285415	0.8005724	0.9271510	1.1380993

LINC01220	0.0064204	0.0135476	0.0012784	0.0328280	0.0054053
JDP2-AS1	0.0038590	0.0036724	0.0087712	0.0033839	0.0035086
JDP2	0.2273081	0.2504694	0.2872320	0.2419758	0.2387036
FLVCR2	0.0007131	0.0000000	0.0039295	0.0000000	0.0000000
TTLL5	0.1680928	0.1411407	0.1399100	0.1327058	0.3623039
ERG28	0.4169523	0.3880716	0.4724225	0.5109160	0.3082114
ENSG00000259103	0.0000000	0.0031499	0.0000000	0.0000000	0.0117607
IFT43	0.3873171	0.2816332	0.2965700	0.4095917	0.3548429
TGFB3	0.0290712	0.0227228	0.0231510	0.0535623	0.0362870
ENSG00000283629	0.0000000	0.0000000	0.0000000	0.0000000	0.0000000
GPATCH2L	0.4756161	0.5603689	0.5129131	0.4581985	0.6275005
ENSG00000258454	0.0006074	0.0010205	0.0000000	0.0044674	0.0000000
ENSG00000258402	0.0000000	0.0000000	0.0000000	0.0000000	0.0000000
ESRRB	0.0055972	0.0070793	0.0117513	0.0000000	0.0185772
ENSG00000259124	0.0116026	0.0104440	0.0053740	0.0074417	0.0277709
VASH1-DT	0.0090272	0.0028573	0.0134963	0.0068731	0.0261267
VASH1	0.0693840	0.1077519	0.0741025	0.0605367	0.1446747
ENSG00000259081	0.0000000	0.0013252	0.0000000	0.0000000	0.0014498
VASH1-AS1	0.0107456	0.0062817	0.0028560	0.0091735	0.0050893
ANGEL1	0.0334118	0.0471782	0.0311427	0.0203943	0.0835015
ENSG00000286437	0.0007698	0.0018708	0.0000000	0.0041721	0.0132357
ENSG00000259058	0.0020880	0.0000000	0.0000000	0.0000000	0.0000000
ENSG00000288762	0.0017014	0.0060937	0.0059291	0.0000000	0.0000000
ENSG00000285966	0.0054839	0.0145217	0.0049861	0.0019410	0.0025988
IRF2BPL	0.5125186	0.5041367	0.5677980	0.4234335	0.5055841
ENSG00000273729	0.0116680	0.0053943	0.0032579	0.0112072	0.0031722
ENSG00000289347	0.0121111	0.0068211	0.0023740	0.0040108	0.0032078
LINC02288	0.0012483	0.0000000	0.0000000	0.0000000	0.0000000
ENSG00000258473	0.0006736	0.0033745	0.0000000	0.0000000	0.0000000
LINC02289	0.0012992	0.0000000	0.0031505	0.0000000	0.0000000
CIPC	0.1316026	0.1470652	0.1719245	0.1179402	0.1199639
TMEM63C	0.0440568	0.0362818	0.0539224	0.0423380	0.1633292
ZDHHC22	0.0271729	0.0639133	0.1003803	0.0239061	0.0578507
ENSG00000269883	0.0007985	0.0000000	0.0028379	0.0000000	0.0048214
NGB	0.0067819	0.0127286	0.0292126	0.0149908	0.0150707
POMT2	0.1812176	0.0941319	0.1074196	0.1985642	0.1458825
GSTZ1	0.0989231	0.1064667	0.0980479	0.1001419	0.0490794
TMED8	0.2662347	0.3040514	0.3422592	0.1942409	0.4105724
SAMD15	0.2237325	0.1083544	0.0945778	0.3128915	0.1219801
NOXRED1	0.0202552	0.0141732	0.0000000	0.0076638	0.0248145
VIPAS39	0.1637735	0.1493526	0.1229909	0.1799808	0.1455917
AHSA1	0.6260340	0.6603759	0.7424980	0.6788239	0.4998176
ISM2	0.0138029	0.0088855	0.0220107	0.0224420	0.0337955
SPTLC2	0.2067543	0.2467715	0.2539404	0.2140508	0.3145522
ALKBH1	0.0476324	0.0558845	0.0708142	0.0767636	0.0494411
SLIRP	0.9245534	1.0306042	1.0827088	0.8796259	0.7598129
SNW1	0.7283773	0.6533297	0.6302518	0.6820264	0.4776573
C14orf178	0.0111953	0.0356805	0.0352073	0.0275493	0.0046437
ADCK1	0.0186906	0.0283779	0.0386252	0.0316718	0.0611234
NRXN3	0.3838490	0.5207809	0.5263354	0.3781121	1.4814268
ENSG00000258829	0.0000000	0.0000000	0.0000000	0.0000000	0.0000000
ENSG00000258419	0.0004377	0.0009796	0.0000000	0.0000000	0.0052988
ENSG00000258662	0.0000000	0.0000000	0.0018292	0.0000000	0.0000000

ENSG00000259106	0.0000000	0.0000000	0.0000000	0.0000000	0.0000000
ENSG00000258637	0.0013134	0.0032071	0.0072960	0.0059981	0.0629832
ENSG00000258416	0.0006147	0.0014749	0.0000000	0.0000000	0.0146252
DIO2	0.0038750	0.0065865	0.0080070	0.0000000	0.0214440
DIO2-AS1	0.0009906	0.0000000	0.0027069	0.0000000	0.0000000
CEP128	0.1036195	0.0857766	0.0440104	0.0633106	0.1582850
TSHR	0.0105277	0.0033498	0.0035575	0.0183348	0.0337102
ENSG00000284959	0.0296949	0.0236524	0.0232669	0.0379136	0.0352160
ENSG00000286686	0.0158486	0.0128302	0.0089101	0.0186594	0.0021475
GTF2A1	0.4346611	0.4233730	0.4230462	0.4762997	0.4118416
GTF2A1-AS1	0.0074893	0.0153815	0.0049981	0.0091512	0.0233190
STON2	0.5316390	0.3655213	0.2908203	0.5605186	0.4195910
SEL1L	0.8422565	0.6729782	0.6457274	1.1051290	0.6882502
ENSG00000259035	0.0008148	0.0035914	0.0000000	0.0022149	0.0000000
LINC02301	0.0441690	0.0301784	0.0352410	0.0306735	0.0540131
ENSG00000258902	0.0024788	0.0000000	0.0000000	0.0049010	0.0043890
ENSG00000258945	0.0041766	0.0020353	0.0000000	0.0000000	0.0000000
FLRT2-AS1	0.0236764	0.0648142	0.0334830	0.0066134	0.0645351
FLRT2	0.0857929	0.1794475	0.1240359	0.0714531	0.3963208
LINC02328	0.0000000	0.0020408	0.0037504	0.0000000	0.0000000
LINC02296	0.0131756	0.0066138	0.0112233	0.0103222	0.0769659
GALC	0.2349768	0.1547634	0.1586425	0.3581777	0.1919982
HISLA	0.0034532	0.0103341	0.0024852	0.0000000	0.0167272
ENSG00000258975	0.0008639	0.0018105	0.0019265	0.0000000	0.0130533
ENSG00000258538	0.0000000	0.0000000	0.0000000	0.0000000	0.0000000
KCNK10	0.0770923	0.1745933	0.2592540	0.0865547	0.3372278
SPATA7	0.4386326	0.4115257	0.3836879	0.3947929	0.5097709
PTPN21	0.1664962	0.1123126	0.0886883	0.2060978	0.1485991
ENSG00000258983	0.0011727	0.0029920	0.0031267	0.0103354	0.0097085
ENSG00000258789	0.0047285	0.0050919	0.0066983	0.0044097	0.0000000
ZC3H14	0.6187184	0.5882253	0.5631259	0.6288428	0.6388269
ENSG00000278576	0.0025009	0.0091001	0.0061788	0.0037228	0.0356935
EML5	0.0922735	0.1030862	0.1150893	0.0623102	0.3063195
ENSG00000274492	0.0020211	0.0024850	0.0017253	0.0000000	0.0000000
TTC8	0.3543313	0.2185948	0.2023270	0.3252694	0.2806055
FOXN3	0.2675516	0.4077301	0.4105798	0.2181669	0.7316821
ENSG00000277801	0.0030890	0.0058705	0.0000000	0.0000000	0.0036030
ENSG00000258752	0.0410585	0.0178228	0.0140327	0.0276335	0.0201740
ENSG00000258380	0.0011306	0.0008572	0.0025201	0.0000000	0.0120730
FOXN3-AS1	0.0150675	0.0122089	0.0158228	0.0085576	0.0376385
FOXN3-AS2	0.0000000	0.0000000	0.0000000	0.0000000	0.0000000
ENSG00000258792	0.0017324	0.0052219	0.0036290	0.0057990	0.0043293
EFCAB11	0.1539467	0.1278781	0.1092405	0.2128134	0.1761127
TDP1	0.0864014	0.1081864	0.0969881	0.0408502	0.1876902
KCNK13	0.0068075	0.0048042	0.0057744	0.0075255	0.0144031
PSMC1	1.1002956	0.9920484	0.9770325	1.0279173	0.7906677
NRDE2	0.1221049	0.1196363	0.1234631	0.1303410	0.1730021
LINC02960	0.0029869	0.0038981	0.0055585	0.0000000	0.0000000
CALM1	3.6192129	3.4658912	3.3562099	3.6533957	3.0873410
ENSG00000258424	0.0017796	0.0000000	0.0000000	0.0000000	0.0000000
LINC00642	0.0027172	0.0000000	0.0000000	0.0000000	0.0030832
TTC7B	0.1921888	0.2083798	0.2038286	0.1633091	0.4837886
TTC7B-AS1	0.0011860	0.0020163	0.0027426	0.0000000	0.0000000

ENSG00000258716	0.0002670	0.0000000	0.0000000	0.0000000	0.0000000
LINC02321	0.0000000	0.0000000	0.0000000	0.0000000	0.0000000
RPS6KA5	0.1394662	0.1610046	0.1558724	0.1067083	0.2396419
DGLUCY	0.2159828	0.1732617	0.1544760	0.2203274	0.1870418
GPR68	0.0077316	0.0179004	0.0263514	0.0165763	0.0137551
ENSG00000258875	0.0008608	0.0000000	0.0000000	0.0000000	0.0017986
ENSG00000286607	0.0008130	0.0011458	0.0035760	0.0000000	0.0064805
CCDC88C	0.0768498	0.0993124	0.0816210	0.1104242	0.1431122
CCDC88C-DT	0.0000000	0.0013933	0.0026786	0.0000000	0.0000000
PPP4R3A	0.4269439	0.3734498	0.3679863	0.3978549	0.5227849
CATSPERB	0.0447267	0.0281324	0.0247026	0.0532755	0.0435737
ENSG00000260711	0.0144804	0.0062556	0.0093558	0.0399056	0.0180403
TC2N	0.2708678	0.1579140	0.0883974	0.3370446	0.1264144
FBLN5	0.3952576	0.2697849	0.1755513	0.8856135	0.1980929
TRIP11	0.5671370	0.4205627	0.3737790	0.5205736	0.5273859
ATXN3	0.2455626	0.3050894	0.2947802	0.2669681	0.3139009
NDUFB1	1.0035655	1.0326627	1.1681415	1.0071163	0.8258127
CPSF2	0.3954731	0.3000230	0.2985829	0.4445432	0.2641584
SLC24A4	0.0041993	0.0007855	0.0033378	0.0027934	0.0033732
RIN3	0.0043045	0.0015309	0.0052436	0.0119424	0.0012623
LGMN	0.3865836	0.2641184	0.2133201	0.7005630	0.3419237
GOLGA5	0.4947271	0.3853253	0.3493442	0.4742510	0.2951464
LINC02833	0.0006318	0.0000000	0.0009005	0.0000000	0.0055669
CHGA	0.3435848	0.5029620	0.6711318	0.1794504	0.4362350
ITPK1	0.1730621	0.1810729	0.2236803	0.1226921	0.2687351
ITPK1-AS1	0.0000000	0.0000000	0.0000000	0.0000000	0.0000000
MOAP1	0.3743770	0.5934070	0.7019993	0.3839514	0.2693579
LYSET	0.1927877	0.2105890	0.2066795	0.2063669	0.1721365
GON7	0.1663768	0.1595969	0.1461973	0.1566646	0.1697437
UBR7	0.1435824	0.0969247	0.1398765	0.1532607	0.1320397
BTBD7	0.3575100	0.3379560	0.3037237	0.3094953	0.4774483
UNC79	0.1632844	0.2676154	0.1780309	0.1609023	0.8535100
ENSG00000278396	0.0011912	0.0013023	0.0017210	0.0000000	0.0000000
COX8C	0.0068594	0.0025712	0.0008672	0.0000000	0.0000000
PRIMA1	0.0259352	0.0238956	0.0145673	0.0129317	0.0441245
FAM181A-AS1	0.0049489	0.0071789	0.0000000	0.0000000	0.0000000
FAM181A	0.0433174	0.0302915	0.0284137	0.0616266	0.0085380
ASB2	0.0042023	0.0024683	0.0054154	0.0056716	0.0111369
OTUB2	0.0376377	0.0462757	0.0363957	0.0685287	0.0223185
DDX24	1.5372332	1.4220779	1.5927801	1.4974880	1.3697640
IFI27L1	0.3267644	0.3222917	0.3110181	0.3491827	0.2567251
IFI27	0.0991160	0.0672307	0.0668263	0.1234787	0.0524094
IFI27L2	1.0653599	0.9181578	0.7990440	1.0439982	0.7856680
PPP4R4	0.2349540	0.1529595	0.1432494	0.2050064	0.2580152
SERPINA10	0.0010008	0.0000000	0.0000000	0.0053726	0.0029782
SERPINA1	0.0521362	0.0212445	0.0133026	0.0462079	0.0255005
SERPINA11	0.0104798	0.0126635	0.0021636	0.0038445	0.0093854
SERPINA9	0.0013537	0.0022408	0.0000000	0.0000000	0.0000000
SERPINA12	0.0035129	0.0110510	0.0000000	0.0000000	0.0036030
ENSG00000287668	0.0161440	0.0065713	0.0026342	0.0140306	0.0072234
SERPINA4	0.0339261	0.0172824	0.0083574	0.0304826	0.0075413
SERPINA5	0.0808776	0.0544660	0.0430824	0.1085582	0.0696610
ENSG00000288028	0.0018346	0.0000000	0.0017239	0.0000000	0.0000000

SERPINA3	0.0082673	0.0011022	0.0026375	0.0203239	0.0000000
DICER1	0.6647669	0.9059605	1.0371437	0.6516432	0.8379556
DICER1-AS1	0.1122327	0.1130664	0.1245410	0.1261390	0.0760707
CLMN	0.0228693	0.0296181	0.0184625	0.0206901	0.1058124
SYNE3	0.0307774	0.0201691	0.0257107	0.0179368	0.0382036
SNHG10	0.0579257	0.0472743	0.0590385	0.0596426	0.0778808
GLRX5	0.7137591	0.6743404	0.7126151	0.6340296	0.5072434
LINC02318	0.0000000	0.0014953	0.0051435	0.0000000	0.0032715
TUNAR	0.1186070	0.2831470	0.3734308	0.1281872	0.1602536
C14orf132	0.5422495	0.6072526	0.4722247	0.4924259	0.4427401
BDKRB2	0.0156138	0.0106340	0.0092044	0.0188331	0.0089465
BDKRB1	0.0051662	0.0017939	0.0051127	0.0088704	0.0040472
ATG2B	0.1477643	0.1540468	0.1909236	0.1408617	0.2137321
GSKIP	0.1569652	0.1636127	0.1830613	0.1315813	0.1213230
AK7	0.3611981	0.1990156	0.1124505	0.4861602	0.2215927
PAPOLA-DT	0.0118113	0.0064146	0.0085095	0.0052964	0.0000000
PAPOLA	1.1463867	1.1450320	1.1765916	1.1303561	1.0384930
ENSG00000258702	0.0086230	0.0185273	0.0356529	0.0132407	0.0057235
ENSG00000287350	0.0006301	0.0000000	0.0000000	0.0000000	0.0000000
LINC02299	0.0014304	0.0048126	0.0036964	0.0000000	0.0000000
VRK1	0.1509985	0.1819860	0.1687200	0.1141849	0.1775112
LINC01550	0.0052659	0.0014253	0.0000000	0.0149555	0.0132590
BCL11B	0.0089001	0.0122060	0.0138575	0.0077875	0.0555762
SETD3	0.4531607	0.4509255	0.4973715	0.4826158	0.4627071
CCNK	0.5043979	0.4386134	0.3153387	0.5277573	0.4086008
CCDC85C	0.1136646	0.1011955	0.0545100	0.1032512	0.1366809
ENSG00000247970	0.0075822	0.0133655	0.0137994	0.0131940	0.0096620
HHIPL1	0.0292390	0.0363799	0.0191033	0.0309384	0.0356667
CYP46A1	0.0848671	0.1269500	0.1883778	0.0661272	0.1663035
EML1	0.2950513	0.3034465	0.2306298	0.3383200	0.3563386
EVL	0.8562053	1.1860403	1.2483824	0.7919045	1.1355746
ENSG00000258560	0.0021022	0.0000000	0.0000000	0.0000000	0.0050638
DEGS2	0.0080072	0.0106416	0.0088766	0.0123255	0.0187294
ENSG00000258982	0.0146193	0.0103559	0.0140645	0.0226086	0.0223598
YY1	0.9746108	0.8789252	0.8551774	0.9112165	0.7647477
ENSG00000259052	0.0000000	0.0015897	0.0034914	0.0060800	0.0117742
SLC25A29	0.1570821	0.1763263	0.1597632	0.1162786	0.3306638
ENSG00000258504	0.0000000	0.0000000	0.0033717	0.0000000	0.0062001
ENSG00000258666	0.0000000	0.0009414	0.0000000	0.0000000	0.0030503
WARS1	0.3674958	0.3347935	0.3113018	0.2765383	0.3173086
WDR25	0.1246699	0.1405432	0.1406392	0.1646648	0.1918852
BEGAIN	0.0351816	0.0684393	0.1078961	0.0305872	0.1882123
DLK1	0.0480863	0.1001561	0.3812501	0.0286200	0.0944189
MEG3	1.0256100	1.4084920	1.1721134	0.9519168	2.3427784
ENSG00000258663	0.0000000	0.0025321	0.0024703	0.0000000	0.0302537
MIR493HG	0.0154381	0.0107309	0.0067701	0.0045802	0.1021690
RTL1	0.0147439	0.0062317	0.0231745	0.0020019	0.0177491
MEG8	0.1773807	0.2349168	0.1469619	0.1860289	0.6131025
MEG9	0.0042047	0.0055078	0.0028485	0.0049792	0.0322181
ENSG00000230805	0.0014353	0.0009363	0.0000000	0.0000000	0.0000000
DIO3OS	0.0221877	0.0219410	0.0219449	0.0339767	0.0237082
DIO3	0.0247084	0.0309989	0.1820795	0.0100333	0.0269513
LINC02320	0.0005648	0.0000000	0.0000000	0.0000000	0.0000000

PPP2R5C	0.5835889	0.6624291	0.6993792	0.5369938	0.7862624
ENSG00000259088	0.0192780	0.0093715	0.0027191	0.0111499	0.0255769
ENSG00000256705	0.0019807	0.0023069	0.0000000	0.0000000	0.0062670
ENSG00000271780	0.0263711	0.0148948	0.0113788	0.0276833	0.0106870
ENSG00000272444	0.0003814	0.0072540	0.0040849	0.0000000	0.0040567
DYNC1H1	1.2390503	1.3828864	1.5094656	1.1589140	1.5082653
HSP90AA1	3.2924405	3.1611699	3.2711969	3.2618685	2.8179832
WDR20	0.1854167	0.1525728	0.1521035	0.1519408	0.2581623
MOK	0.3901830	0.3053838	0.2481815	0.3660149	0.3051921
ZNF839	0.0856859	0.0622589	0.0679782	0.0747510	0.0593837
CINP	0.3313317	0.3202007	0.3605370	0.2484898	0.3266727
TECPR2	0.1827521	0.1546994	0.2002521	0.1840261	0.2760020
ANKRD9	0.3721819	0.3078481	0.2943199	0.4381405	0.2635689
LINC02323	0.0000000	0.0000000	0.0000000	0.0000000	0.0000000
ENSG00000289862	0.0012386	0.0000000	0.0014605	0.0000000	0.0097893
RCOR1	0.1971084	0.1450470	0.1815380	0.1582705	0.2313409
ENSG00000259508	0.0196922	0.0062031	0.0024296	0.0075319	0.0059619
TRAF3	0.4451518	0.4516829	0.4974378	0.4200547	0.6129576
AMN	0.0012808	0.0000000	0.0000000	0.0040163	0.0013692
CDC42BPB	0.5859303	0.6663879	0.7481852	0.6142785	0.7819979
ENSG00000259515	0.0004094	0.0007505	0.0000000	0.0000000	0.0065294
ENSG00000289207	0.0274827	0.0402468	0.0555070	0.0094302	0.0178464
LBHD2	0.0030209	0.0152685	0.0081125	0.0028850	0.0000000
TNFAIP2	0.0072575	0.0031300	0.0074137	0.0150614	0.0128969
ENSG00000278071	0.0012209	0.0000000	0.0014267	0.0000000	0.0067404
EIF5-DT	0.0033850	0.0087299	0.0059944	0.0122767	0.0103109
EIF5	1.3344783	1.1711280	1.1088840	1.2996110	1.0036514
MARK3	0.6012133	0.6140459	0.6014523	0.5879052	0.6979972
CKB	2.4387613	1.9882996	1.7644793	2.5258403	1.7965735
TRMT61A-DT	0.0026382	0.0023773	0.0024028	0.0000000	0.0000000
TRMT61A	0.0498824	0.0368068	0.0544704	0.0424635	0.0312886
ENSG00000258851	0.0000000	0.0005310	0.0000000	0.0000000	0.0000000
BAG5	0.4265507	0.4453950	0.3967706	0.3838490	0.3025658
KLC1	1.1190962	1.5612315	1.8534093	1.0877736	1.3744073
COA8	0.6205568	0.6214986	0.7104316	0.6498923	0.5514292
KLC1-AS1	0.0035467	0.0051098	0.0015844	0.0027655	0.0208193
ENSG00000270108	0.0012834	0.0010829	0.0000000	0.0023550	0.0033613
ENSG00000269910	0.0015953	0.0000000	0.0000000	0.0000000	0.0053380
ENSG00000269940	0.0020359	0.0023848	0.0000000	0.0000000	0.0207844
ENSG00000269958	0.0078163	0.0075162	0.0092547	0.0106909	0.0058361
XRCC3	0.0129522	0.0087461	0.0189084	0.0128332	0.0000000
ZFYVE21	0.5438909	0.3576282	0.3281838	0.5787292	0.3007974
PPP1R13B	0.1575207	0.1962276	0.2247248	0.1249929	0.3958071
PPP1R13B-DT	0.0037365	0.0000000	0.0023861	0.0039833	0.0000000
ENSG00000258534	0.0000000	0.0000000	0.0000000	0.0000000	0.0000000
ATP5MJ	1.2607111	1.4019045	1.4429231	1.2155572	1.1380532
TDRD9	0.0000000	0.0006859	0.0008696	0.0000000	0.0000000
ASPG	0.0000000	0.0024030	0.0027049	0.0000000	0.0000000
KIF26A	0.0285053	0.0583010	0.0495645	0.0126912	0.0774169
LINC02691	0.0866615	0.0071277	0.0048049	0.0082880	0.0313432
ENSG00000258598	0.0011222	0.0054399	0.0035524	0.0028001	0.0000000
TMEM179	0.0660969	0.0882246	0.1255466	0.0542932	0.1674700
INF2	0.0477291	0.0393106	0.0229782	0.0786358	0.0668885

ADSS1	0.1480238	0.1322319	0.1450272	0.0904774	0.1133054
SIVA1	0.4304270	0.3444304	0.3050135	0.4529526	0.2403375
ENSG00000258430	0.0035113	0.0082783	0.0073252	0.0000000	0.0053413
AKT1	0.5051438	0.4943839	0.4657630	0.5165402	0.3527467
ZBTB42	0.0170881	0.0044954	0.0037927	0.0050906	0.0000000
LINC00638	0.0033394	0.0079034	0.0000000	0.0084152	0.0000000
ENSG00000258593	0.0023843	0.0000000	0.0000000	0.0000000	0.0000000
CEP170B	0.0968605	0.1707221	0.1998182	0.0558267	0.1886330
AHNAK2	0.1002670	0.2011844	0.1644794	0.1071394	0.3400571
CLBA1	0.1295171	0.0916252	0.0845532	0.1487131	0.0448832
CDCA4	0.0396885	0.0299886	0.0227084	0.0382492	0.0252256
GPR132	0.0009229	0.0013531	0.0000000	0.0000000	0.0073328
LINC02298	0.0542312	0.0399703	0.0419800	0.0542331	0.0591030
ENSG00000287459	0.0012301	0.0000000	0.0000000	0.0000000	0.0015298
JAG2	0.0319693	0.0505391	0.0693649	0.0281369	0.1137023
NUDT14	0.2878764	0.3457151	0.4210982	0.2693823	0.1942049
BRF1	0.1873091	0.1721002	0.1770099	0.1604327	0.2848596
BTBD6	0.1663628	0.2316698	0.3269136	0.1548597	0.1689414
PACS2	0.3766702	0.4231186	0.4256942	0.3820106	0.6328889
TEX22	0.0216352	0.0187006	0.0120187	0.0096470	0.0509892
MTA1-DT	0.0788593	0.0547241	0.0774392	0.0553878	0.0363884
MTA1	0.4052538	0.4227055	0.4436442	0.3692248	0.3807922
ENSG00000257270	0.0000000	0.0000000	0.0048994	0.0000000	0.0000000
CRIP2	1.1704738	0.9847202	0.9498685	1.1158775	0.8195050
CRIP1	0.4004311	0.2730925	0.1737879	0.4753946	0.1948806
TEDC1	0.0247448	0.0384019	0.0323745	0.0395637	0.0669529
TMEM121	0.0818619	0.1118159	0.1115049	0.0752451	0.0710087
ENSG00000258410	0.0000000	0.0012671	0.0000000	0.0000000	0.0000000
ENSG00000287122	0.0000000	0.0012251	0.0000000	0.0000000	0.0037513
HERC2P3	0.0135821	0.0084965	0.0026342	0.0092902	0.0284186
ENSG00000278626	0.0006414	0.0038291	0.0035580	0.0000000	0.0000000
ENSG00000260409	0.0011214	0.0005601	0.0000000	0.0000000	0.0075108
ENSG00000247765	0.0010608	0.0014102	0.0000000	0.0000000	0.0000000
ENSG00000291003	0.0777410	0.0950380	0.0571354	0.0674255	0.1797797
GOLGA8IP	0.0000000	0.0000000	0.0000000	0.0000000	0.0047971
ENSG00000290399	0.0965218	0.0871936	0.1413746	0.0881756	0.0916159
ENSG00000274253	0.0506475	0.1225836	0.1666796	0.0229564	0.0992016
NIPA1	0.1481384	0.2264024	0.1987166	0.1551385	0.2020687
NIPA2	0.3463062	0.3788315	0.3656631	0.3983748	0.2925970
CYFIP1	0.4081523	0.2025984	0.1255526	0.4218652	0.3060817
TUBGCP5	0.2330764	0.1892659	0.1818558	0.1926766	0.2354945
HERC2P7	0.0294970	0.0357737	0.0222481	0.0236252	0.0386356
GOLGA6L2	0.0110596	0.0118461	0.0037782	0.0079605	0.0200963
ENSG00000260978	0.0025681	0.0063730	0.0054587	0.0000000	0.0281710
MKRN3	0.0610781	0.1191180	0.1019866	0.0671510	0.1840617
MAGEL2	0.0952614	0.1199216	0.1230962	0.0825377	0.1273728
NDN	1.2516062	1.0526442	1.2304781	1.2230209	0.9123314
ENSG00000286973	0.0405903	0.0594560	0.0756663	0.0346197	0.1734670
PWRN4	0.2442367	0.3061069	0.3885675	0.2315154	0.6733990
PWRN1	0.1209943	0.1418850	0.0689373	0.0868728	0.5319152
PWRN2	0.0000000	0.0026147	0.0000000	0.0000000	0.0000000
NPAP1	0.0000000	0.0007294	0.0000000	0.0000000	0.0013244
SNRPN	1.6816914	2.0327600	2.2528077	1.6501098	1.7537893

ENSG00000280118	0.0000000	0.0015349	0.0027632	0.0029139	0.0000000
SNURF	0.0111004	0.0118063	0.0193763	0.0225497	0.0376468
SNHG14	1.6364210	1.9459501	1.9069565	1.4567034	2.8638481
ENSG00000257647	0.0007192	0.0016177	0.0083537	0.0000000	0.0000000
ENSG00000287482	0.0000000	0.0000000	0.0000000	0.0000000	0.0146515
UBE3A	0.6936115	0.7763107	0.8961034	0.6990399	0.9270739
ENSG00000261529	0.0012450	0.0011842	0.0000000	0.0000000	0.0000000
LINC02250	0.0016315	0.0014467	0.0000000	0.0023239	0.0333849
ENSG00000289522	0.0017009	0.0023645	0.0000000	0.0032050	0.0000000
ATP10A	0.0019976	0.0050506	0.0031481	0.0000000	0.0071630
LINC02346	0.0000000	0.0008078	0.0034679	0.0000000	0.0055309
LINC02248	0.0000000	0.0000000	0.0024527	0.0000000	0.0000000
ENSG00000287280	0.0000000	0.0039334	0.0063864	0.0000000	0.0000000
GABRB3	0.3747165	0.6645063	0.7930788	0.3516428	0.9739546
ENSG00000278840	0.0010593	0.0015338	0.0065331	0.0000000	0.0000000
GABRA5	0.0450709	0.0840204	0.1740860	0.0289842	0.0764504
GABRG3	0.0675672	0.0978169	0.1446263	0.0802146	0.4382744
GABRG3-AS1	0.0010727	0.0068604	0.0031577	0.0000000	0.0237599
ENSG00000261426	0.0000000	0.0000000	0.0000000	0.0000000	0.0000000
ENSG00000259168	0.0000000	0.0000000	0.0000000	0.0000000	0.0076534
OCA2	0.0996259	0.0665769	0.0361640	0.1693997	0.0928547
HERC2	0.2638949	0.3140774	0.3724736	0.2132289	0.5641285
ENSG00000291259	0.0340221	0.0506779	0.0266948	0.0441240	0.0992892
HERC2P9	0.0191473	0.0304394	0.0250451	0.0195025	0.0361847
GOLGA8M	0.0056096	0.0030122	0.0000000	0.0054337	0.0156524
ENSG00000290879	0.0873619	0.0824676	0.0645501	0.0562471	0.0446723
ENSG00000291258	0.0638479	0.1158367	0.1703288	0.0441940	0.1134589
GOLGA6L7	0.0000000	0.0012690	0.0000000	0.0000000	0.0000000
ENSG00000289209	0.0126043	0.0182223	0.0369435	0.0019814	0.0183107
ENSG00000287894	0.0000000	0.0000000	0.0007240	0.0000000	0.0000000
APBA2	0.1314997	0.3019731	0.3582351	0.1653241	0.4737239
ENSG00000259277	0.0000000	0.0000000	0.0000000	0.0000000	0.0000000
ENTREP2	0.0792347	0.0736802	0.0623389	0.0491726	0.2180845
NSMCE3	0.2688578	0.2256919	0.2649303	0.3220723	0.1804842
ENSG00000259690	0.0062230	0.0000000	0.0000000	0.0000000	0.0000000
LCIIAR	0.0827953	0.0562703	0.0422453	0.0997587	0.0444419
TJP1	0.6953288	0.4694487	0.3772650	0.7358857	0.5736425
ENSG00000259644	0.0010600	0.0031230	0.0036262	0.0000000	0.0068564
ENSG00000259523	0.0016521	0.0000000	0.0000000	0.0000000	0.0000000
ULK4P3	0.0130921	0.0127337	0.0194269	0.0102524	0.0082451
GOLGA8T	0.0013280	0.0000000	0.0000000	0.0000000	0.0000000
LINC02249	0.0038194	0.0072180	0.0080035	0.0000000	0.0319462
CHRFAM7A	0.0002875	0.0033262	0.0033523	0.0026054	0.0000000
ENSG00000289455	0.0010227	0.0019925	0.0032100	0.0000000	0.0031305
GOLGA8R	0.0037947	0.0028738	0.0054965	0.0020674	0.0038629
ENSG00000289397	0.0031429	0.0022346	0.0013034	0.0000000	0.0000000
LINC02256	0.0569166	0.0740331	0.0947427	0.0565173	0.0770921
ENSG00000290876	0.0711241	0.0876910	0.1460191	0.0566776	0.0852235
ENSG00000270055	0.0287367	0.0313926	0.0512310	0.0126608	0.0228921
ENSG00000260693	0.0020456	0.0010689	0.0059507	0.0027655	0.0000000
ULK4P2	0.0008788	0.0061422	0.0020680	0.0000000	0.0046088
GOLGA8H	0.0012842	0.0008298	0.0013847	0.0032979	0.0000000
ENSG00000270016	0.0008641	0.0007390	0.0009177	0.0130754	0.0051674

ARHGAP11B-DT	0.0026984	0.0084093	0.0021877	0.0062961	0.0161942
ARHGAP11B	0.0043553	0.0062798	0.0000000	0.0131517	0.0000000
ENSG00000290886	0.0017358	0.0012435	0.0019983	0.0000000	0.0130218
FAN1	0.2599461	0.2096780	0.2054958	0.2591723	0.2532286
MTMR10	0.1103370	0.0619089	0.0496046	0.1629000	0.0971659
ENSG00000259720	0.0000000	0.0000000	0.0007240	0.0000000	0.0000000
TRPM1	0.0000000	0.0013272	0.0000000	0.0000000	0.0000000
LINC03034	0.0000000	0.0000000	0.0000000	0.0000000	0.0000000
LINC02352	0.1140727	0.0750978	0.0512211	0.0946627	0.0627607
KLF13	0.2786095	0.2502046	0.1794912	0.3148659	0.2210301
OTUD7A	0.0833952	0.1676122	0.1371047	0.0596805	0.5089499
CHRNA7	0.0341352	0.0516399	0.0400402	0.0266673	0.1231828
GOLGA8K	0.0000000	0.0000000	0.0000000	0.0000000	0.0000000
LINC02256.1	0.0108074	0.0046229	0.0048625	0.0081760	0.0366235
ENSG00000276724	0.0051385	0.0069861	0.0065666	0.0023869	0.0075843
ARHGAP11A-DT	0.0065540	0.0110262	0.0087721	0.0074500	0.0116423
GOLGA8N	0.0000000	0.0073726	0.0022620	0.0068347	0.0000000
ARHGAP11A	0.0205619	0.0301081	0.0160031	0.0142199	0.0288647
ENSG00000285948	0.0039778	0.0093401	0.0007564	0.0000000	0.0000000
SCG5	0.7036141	0.8734692	1.1873108	0.8302947	0.8338293
SCG5-AS1	0.0000000	0.0000000	0.0000000	0.0000000	0.0057137
GREM1	0.0023604	0.0083336	0.0017282	0.0069777	0.0046162
FMN1	0.0872270	0.0617940	0.0348823	0.0637724	0.2002183
RYR3-DT	0.0006948	0.0014564	0.0026175	0.0000000	0.0000000
RYR3	0.1120901	0.1150313	0.0815835	0.0568807	0.6127605
ENSG00000276702	0.0000000	0.0000000	0.0000000	0.0000000	0.0000000
ENSG00000259408	0.0000000	0.0036786	0.0039013	0.0043997	0.0000000
AVEN	0.1292880	0.1622717	0.1569893	0.1115967	0.1762318
CHRM5	0.0131993	0.0138997	0.0193029	0.0088688	0.0678457
EMC7	0.4708056	0.3880138	0.4030628	0.6175614	0.2821173
PGBD4	0.0500442	0.0382789	0.0533537	0.0348218	0.0401368
KATNBL1	0.5262089	0.5258634	0.4718161	0.5160675	0.5721148
EMC4	0.7254387	0.6474602	0.6900569	0.7209049	0.5370897
SLC12A6	0.1016043	0.1115605	0.1525510	0.1068246	0.1237447
ENSG00000259468	0.0000000	0.0000000	0.0000000	0.0000000	0.0023981
NOP10	0.9535182	0.9419662	0.9466801	0.9500721	0.6860853
NUTM1	0.0000000	0.0000000	0.0000000	0.0000000	0.0000000
LPCAT4	0.0996869	0.1687325	0.2220667	0.0924923	0.2058454
GOLGA8A	0.1510974	0.1797284	0.1450023	0.1620463	0.4980032
GOLGA8B	0.1154781	0.1363807	0.1331411	0.0706694	0.3268796
GJD2	0.0129514	0.0077048	0.0283530	0.0000000	0.0141772
GJD2-DT	0.0010215	0.0041908	0.0137626	0.0067495	0.0150241
ACTC1	0.0000000	0.0098154	0.0010318	0.0000000	0.0085155
AQR	0.2320412	0.2457990	0.2207655	0.2344811	0.3238989
ZNF770	0.7598101	0.7878548	0.8891841	0.6872939	0.6905864
ENSG00000259181	0.0000000	0.0020967	0.0000000	0.0000000	0.0035970
ENSG00000289289	0.0000000	0.0004553	0.0000000	0.0000000	0.0000000
ENSG00000259336	0.0185579	0.0121079	0.0042244	0.0122359	0.0706752
DPH6	0.3316634	0.2758124	0.1814895	0.2545440	0.3340666
DPH6-DT	0.0267007	0.0223180	0.0208185	0.0465961	0.1022425
LINC02853	0.0000000	0.0026029	0.0000000	0.0000000	0.0028343
ENSG00000259639	0.0000000	0.0000000	0.0000000	0.0000000	0.0014182
CDIN1	0.1605818	0.2130840	0.2789040	0.1424482	0.4861663

ENSG00000261191	0.0000000	0.0013541	0.0012014	0.0000000	0.0099652
ENSG00000259280	0.1238805	0.2967589	0.2946935	0.1090753	0.2272185
MEIS2	1.4181515	1.6274496	1.9179928	1.3851983	2.0665342
ENSG00000259460	0.0390007	0.0411912	0.0564083	0.0223111	0.0294545
ENSG00000288808	0.0097976	0.0114536	0.0138467	0.0029052	0.0241992
ENSG00000274444	0.0035778	0.0046801	0.0080099	0.0021398	0.0000000
ENSG00000259434	0.0024600	0.0010939	0.0017912	0.0058455	0.0000000
LINC02345	0.0203211	0.0308624	0.0092895	0.0126448	0.0105854
LINC01852	0.0184738	0.0091762	0.0055044	0.0113883	0.0059071
LINC02895	0.0016080	0.0000000	0.0000000	0.0077999	0.0000000
SPRED1	0.5633046	0.4150185	0.2320915	0.6258953	0.4100347
FAM98B	0.3721969	0.3385231	0.3374159	0.3724711	0.3105349
RASGRP1	0.0072168	0.0097722	0.0246305	0.0168697	0.0325932
ENSG00000286786	0.0046155	0.0035925	0.0024852	0.0000000	0.0221071
ENSG00000259326	0.0000000	0.0012435	0.0000000	0.0000000	0.0000000
ENSG00000259598	0.0000000	0.0000000	0.0000000	0.0000000	0.0050816
LINC02694	0.0011611	0.0011931	0.0000000	0.0000000	0.0086654
ENSG00000274281	0.0000000	0.0000000	0.0000000	0.0000000	0.0000000
ENSG00000259345	0.0249809	0.0292833	0.0221289	0.0299079	0.1172301
ENSG00000259447	0.0000000	0.0000000	0.0030261	0.0000000	0.0038734
ENSG00000259269	0.0000000	0.0011489	0.0000000	0.0000000	0.0022413
ENSG00000259390	0.0000000	0.0024945	0.0031505	0.0000000	0.0000000
THBS1	0.0130836	0.0047958	0.0048459	0.0061965	0.0039740
THBS1-IT1	0.0000000	0.0000000	0.0000000	0.0000000	0.0000000
FSIP1	0.1461520	0.1038709	0.0509745	0.1316226	0.1488578
ENSG00000261136	0.0025439	0.0067697	0.0039615	0.0037157	0.0000000
GPR176	0.0548433	0.1068941	0.0520648	0.0423695	0.1933286
ENSG00000259432	0.0000000	0.0000000	0.0000000	0.0030184	0.0000000
GPR176-DT	0.0031052	0.0000000	0.0119704	0.0063752	0.0000000
EIF2AK4	0.5264785	0.4732702	0.4924205	0.4842012	0.5872982
SRP14	2.4095520	2.2933986	2.3854746	2.3900559	1.9643194
SRP14-DT	0.1445291	0.0951474	0.1014735	0.1227807	0.1092115
ENSG00000275636	0.0089385	0.0009824	0.0020840	0.0094700	0.0000000
BMF	0.0576332	0.0508526	0.0239700	0.0450173	0.0278658
BUB1B	0.0169793	0.0157088	0.0235197	0.0224124	0.0153775
PAK6	0.1777629	0.2527219	0.2793301	0.1303500	0.1612687
ENSG00000273786	0.0015456	0.0000000	0.0000000	0.0000000	0.0000000
PLCB2	0.0322152	0.0405317	0.0722711	0.0203759	0.0227762
ANKRD63	0.0138261	0.0036781	0.0082837	0.0012567	0.0157054
ENSG00000273855	0.0000000	0.0034179	0.0080735	0.0000000	0.0000000
INAFM2	0.0774221	0.0668929	0.0653579	0.0731766	0.0699299
CCDC9B	0.0127852	0.0091019	0.0028002	0.0109730	0.0268966
ENSG00000289148	0.0003873	0.0044890	0.0026952	0.0000000	0.0042817
DISP2	0.1107161	0.2060751	0.2632619	0.1533574	0.2062988
KNSTRN	0.1334185	0.1658279	0.1460582	0.1256685	0.1161264
IVD	0.4777743	0.3125993	0.2407745	0.5370798	0.3564943
BAHD1	0.0788407	0.0917828	0.0850977	0.0843596	0.1110506
CHST14	0.1110256	0.0627246	0.0375854	0.2203677	0.0550490
ENSG00000259536	0.0171495	0.0165941	0.0393300	0.0084530	0.0353282
CCDC32	0.2432553	0.2830169	0.3635209	0.2054082	0.2883001
RPUSD2	0.0931805	0.0779631	0.0729925	0.1146417	0.0361756
KNL1	0.0211490	0.0077155	0.0052237	0.0033647	0.0092451
RAD51-AS1	0.0245384	0.0457738	0.0247938	0.0125200	0.0700437

RAD51	0.0037791	0.0021018	0.0000000	0.0104877	0.0000000
RMDN3	0.2771260	0.2101801	0.2295619	0.3086436	0.2082802
GCHFR	0.2849431	0.3697725	0.7656140	0.2253018	0.2437248
DNAJC17	0.1252036	0.1128492	0.1625691	0.1374336	0.1272224
C15orf62	0.0000000	0.0006310	0.0000000	0.0041335	0.0000000
ZFYVE19	0.0783829	0.0915184	0.1126701	0.0705965	0.0629023
PPP1R14D	0.0000000	0.0000000	0.0000000	0.0000000	0.0000000
SPINT1-AS1	0.0089823	0.0182285	0.0231938	0.0000000	0.0097347
SPINT1	0.0077400	0.0123052	0.0268963	0.0093016	0.0114017
RHOV	0.0072512	0.0343407	0.0568768	0.0020601	0.0191503
ENSG00000274798	0.0026350	0.0063034	0.0039229	0.0000000	0.0041978
VPS18	0.0610473	0.0473019	0.0514978	0.0419703	0.0358658
ENSG00000251161	0.0011912	0.0037310	0.0000000	0.0000000	0.0000000
DLL4	0.0029628	0.0016798	0.0170709	0.0000000	0.0158733
CHAC1	0.2564082	0.2526579	0.3071859	0.2257643	0.1870383
INO80	0.1571090	0.1452536	0.1529652	0.1286063	0.4218727
ENSG00000259617	0.0011785	0.0023186	0.0024168	0.0000000	0.0067099
ENSG00000259463	0.0000000	0.0013778	0.0000000	0.0000000	0.0000000
INO80-AS1	0.0005140	0.0000000	0.0000000	0.0000000	0.0033679
ENSG00000280036	0.0000000	0.0007784	0.0000000	0.0000000	0.0045428
EXD1	0.0031644	0.0053768	0.0092202	0.0047605	0.0064756
CHP1	0.2878201	0.3916942	0.4083971	0.3247753	0.3337089
OIP5-AS1	0.7329858	0.7867902	0.6713995	0.7258758	0.6579550
OIP5	0.0077780	0.0039709	0.0042421	0.0055429	0.0199237
NUSAP1	0.0517439	0.0748824	0.0466733	0.1099141	0.0639977
NDUFAF1	0.1843962	0.1721736	0.1582335	0.2169927	0.1271583
RTF1	0.8186764	0.8755147	0.9678362	0.7378908	0.9216648
ITPKA	0.0038342	0.0061843	0.0025093	0.0019780	0.0033534
LTK	0.0000000	0.0000000	0.0000000	0.0000000	0.0030362
RPAP1	0.0804596	0.0824209	0.0887498	0.1117888	0.1386021
TYRO3	0.0617367	0.0982274	0.1001204	0.0721544	0.0834957
ENSG00000260926	0.0003268	0.0017206	0.0000000	0.0000000	0.0000000
MGA	0.2953513	0.2727653	0.2979575	0.2656921	0.4077259
MAPKBP1	0.0682311	0.0608905	0.0599282	0.0602844	0.2115177
JMJD7	0.0479861	0.0565683	0.0626239	0.0634055	0.0610635
PLA2G4B	0.0132330	0.0139839	0.0146186	0.0061776	0.0539343
SPTBN5	0.0000000	0.0024563	0.0011221	0.0000000	0.0000000
ENSG00000174171	0.0024522	0.0023274	0.0000000	0.0018904	0.0027747
EHD4	0.1325755	0.0725891	0.0556317	0.0748422	0.1375957
ENSG00000278493	0.0000000	0.0000000	0.0000000	0.0000000	0.0016619
PLA2G4E-AS1	0.0018874	0.0000000	0.0000000	0.0000000	0.0065249
VPS39	0.1840941	0.1925786	0.1614436	0.1910437	0.2236396
VPS39-DT	0.0000000	0.0012552	0.0000000	0.0000000	0.0000000
TMEM87A	0.4691423	0.3647674	0.4209699	0.5939649	0.5361088
GANC	0.0510073	0.0615340	0.0420949	0.0464372	0.1561734
CAPN3	0.0108183	0.0106873	0.0033883	0.0027265	0.0050649
ZNF106	0.7257924	0.6043386	0.5682750	0.7946152	0.6451404
SNAP23	0.2487195	0.1389324	0.1075406	0.2503312	0.1646883
ENSG00000261684	0.0165282	0.0138247	0.0130282	0.0156869	0.0482527
LRRC57	0.1745256	0.1990062	0.1805745	0.1701546	0.1499093
HAUS2	0.2516211	0.3057698	0.3323107	0.2061092	0.2657313
ENSG00000261822	0.0055071	0.0039343	0.0042193	0.0025471	0.0087139
STARD9	0.0664686	0.0592498	0.0541533	0.0454766	0.2252160

CDAN1	0.0293483	0.0209455	0.0275733	0.0328901	0.0333177
ENSG00000274403	0.0000000	0.0000000	0.0000000	0.0029286	0.0000000
TTBK2	0.3988519	0.6335287	0.6420092	0.3313312	0.6958932
TTBK2-AS1	0.0070368	0.0158172	0.0052337	0.0000000	0.0036979
ENSG00000286877	0.0000000	0.0000000	0.0000000	0.0000000	0.0000000
UBR1	0.2498044	0.2439080	0.2935479	0.2435418	0.5446591
ENSG00000285080	0.2085975	0.1773469	0.2171225	0.1535531	0.2042729
TMEM62	0.1085265	0.0698521	0.0676718	0.1105611	0.0956983
CCNDBP1	0.3019290	0.2848911	0.3713914	0.2693112	0.1713624
LCMT2	0.0297919	0.0380524	0.0222956	0.0274277	0.0157660
ADAL	0.2200761	0.2063884	0.2368976	0.2182534	0.1799935
ZSCAN29	0.0524270	0.0769324	0.0678243	0.0424325	0.0573071
TUBGCP4	0.1227133	0.1292884	0.1456341	0.1137725	0.1120047
TP53BP1	0.6561739	0.7271990	0.8278782	0.6032109	0.9040796
MAP1A	1.0350723	1.4274903	1.4122468	1.0061514	1.1346630
PPIP5K1	0.0637264	0.0416829	0.0493307	0.0392852	0.1337851
CKMT1B	0.0392393	0.1135529	0.1206119	0.0507211	0.1148731
STRC	0.0042055	0.0025990	0.0031397	0.0000000	0.0165653
CATSPER2	0.0274371	0.0441346	0.0186535	0.0091154	0.1063475
ENSG00000275601	0.0092705	0.0070040	0.0125224	0.0044903	0.0166998
CKMT1A	0.0447603	0.1004903	0.1059531	0.0275322	0.0437969
STRCP1	0.0019787	0.0013102	0.0000000	0.0000000	0.0050191
CATSPER2P1	0.0778197	0.0568443	0.0432304	0.0651286	0.0556709
PDIA3	1.8390801	1.4370591	1.4818954	2.4422447	1.5007984
ELL3	0.0130627	0.0097117	0.0000000	0.0082145	0.0000000
SERF2	2.0899842	1.9027969	1.7955066	2.2089507	1.7011599
SERINC4	0.0061232	0.0058318	0.0011329	0.0164393	0.0076017
HYPK	0.0078326	0.0141682	0.0155497	0.0167897	0.0300507
MFAP1	0.6858925	0.5860021	0.4904549	0.6546866	0.4495444
WDR76	0.0342511	0.0215400	0.0571559	0.0454051	0.0306174
FRMD5	0.2326718	0.2662936	0.2825767	0.1883606	0.9806311
ENSG00000289277	0.0133664	0.0274177	0.0190794	0.0099038	0.0454259
GOLM2	1.0566636	0.9638129	0.9653470	1.2278855	1.0605902
ENSG00000259499	0.0000000	0.0000000	0.0000000	0.0000000	0.0000000
CTDSPL2-DT	0.0050623	0.0031055	0.0021529	0.0097616	0.0222693
CTDSPL2	0.3942782	0.4166194	0.3940736	0.4179019	0.4387426
EIF3J-DT	0.2047988	0.1841935	0.2165563	0.1792698	0.1752323
EIF3J	0.9717233	0.9716079	0.9979359	0.9713409	0.7320617
SPG11	0.3114872	0.2734845	0.2201261	0.2734938	0.4111671
ENSG00000259659	0.0000000	0.0003976	0.0018068	0.0000000	0.0000000
PATL2	0.0000000	0.0000000	0.0009653	0.0000000	0.0000000
B2M	0.5592680	0.3651558	0.2986976	0.9342810	0.3884315
TRIM69	0.3290994	0.3488590	0.4178107	0.3589904	0.3144918
ENSG00000276121	0.0000000	0.0000000	0.0000000	0.0000000	0.0000000
SORD2P	0.0000000	0.0000000	0.0000000	0.0000000	0.0000000
SORD	0.0214126	0.0272159	0.0411645	0.0313554	0.0304122
DUOX2	0.0000000	0.0000000	0.0026426	0.0000000	0.0000000
DUOXA1	0.0007854	0.0022144	0.0023834	0.0000000	0.0067724
DUOX1	0.0119077	0.0058579	0.0121315	0.0037517	0.0276654
ENSG00000259539	0.0179562	0.0259218	0.0329803	0.0166923	0.0354405
SHF	0.0731187	0.1430428	0.1905029	0.0580023	0.1837022
ENSG00000259519	0.0025633	0.0011722	0.0011130	0.0000000	0.0118054
ENSG00000259932	0.0006224	0.0009224	0.0000000	0.0000000	0.0000000

SLC28A2-AS1	0.0515949	0.0551616	0.0432403	0.0375615	0.1065080
SLC28A2	0.0000000	0.0000000	0.0000000	0.0000000	0.0115971
GATM	0.4436030	0.2850709	0.2005168	0.7055268	0.1589924
ENSG00000275672	0.0022442	0.0017386	0.0000000	0.0000000	0.0044291
SPATA5L1	0.0732521	0.0820388	0.0715818	0.1017300	0.0560214
C15orf48	0.0023844	0.0013232	0.0000000	0.0000000	0.0066826
SLC30A4-AS1	0.0020256	0.0006997	0.0036294	0.0000000	0.0140511
SLC30A4	0.0579761	0.0907857	0.1008757	0.0364355	0.0944808
ENSG00000275709	0.0011066	0.0010598	0.0000000	0.0000000	0.0025600
BLOC1S6	0.6879030	0.7443272	0.8525171	0.6072327	0.5785747
SQOR	0.0934945	0.0372633	0.0288638	0.0861347	0.0126788
ENSG00000259200	0.0122750	0.0059064	0.0058561	0.0000000	0.0246477
ENSG00000287020	0.0003288	0.0000000	0.0000000	0.0000000	0.0000000
ENSG00000285934	0.0000000	0.0000000	0.0000000	0.0000000	0.0000000
ENSG00000287704	0.4839242	0.4053542	0.3226362	0.3882802	0.6465818
ENSG00000259255	0.3143453	0.1872255	0.1161710	0.2686725	0.1996906
SEMA6D	0.4648683	0.5215565	0.5080086	0.5328860	1.6777125
ENSG00000259554	0.0011708	0.0021658	0.0000000	0.0000000	0.0096228
ENSG00000259221	0.0042649	0.0014978	0.0030572	0.0013531	0.0451543
ENSG00000259360	0.0000000	0.0000000	0.0000000	0.0000000	0.0000000
ENSG00000259588	0.0000000	0.0000000	0.0000000	0.0000000	0.0000000
LINC01491	0.0000000	0.0000000	0.0000000	0.0000000	0.0072478
ENSG00000259754	0.0011041	0.0000000	0.0000000	0.0000000	0.0000000
SLC24A5	0.0077003	0.0069525	0.0087868	0.0165847	0.0069569
MYEF2	0.7399344	0.7863363	0.8602076	0.6365885	0.9110250
CTXN2	0.1484222	0.1799980	0.1819915	0.0973687	0.1410556
SLC12A1	0.2416946	0.1599463	0.1094170	0.1763238	0.1730117
CTXN2-AS1	0.0141008	0.0242319	0.0351713	0.0000000	0.0296754
DUT-AS1	0.0065775	0.0013767	0.0094293	0.0150063	0.0000000
DUT	1.2437995	1.2886796	1.3119465	1.2421849	1.0128538
FBN1	0.0939132	0.0989532	0.0497137	0.1533622	0.2416874
FBN1-DT	0.0249202	0.0139282	0.0101389	0.0344407	0.0231751
CEP152	0.0323173	0.0293732	0.0050291	0.0174618	0.0387645
SHC4	0.0483855	0.0501336	0.0489045	0.0346722	0.0976569
EID1	1.8947629	1.7839880	1.8515144	1.8647767	1.4426569
SECISBP2L	0.7617822	0.6635709	0.6504468	0.7472432	0.6900985
COPS2	0.9042960	0.8443509	0.7416807	0.8910858	0.7063867
GALK2	0.2372037	0.1993252	0.1914636	0.1736541	0.3057698
FAM227B	0.2217033	0.1318364	0.0987144	0.1840277	0.1579995
ENSG00000275580	0.0119527	0.0134544	0.0062394	0.0050126	0.0000000
ENSG00000276593	0.0021930	0.0051123	0.0000000	0.0019659	0.0000000
FGF7	0.0429666	0.0530371	0.0775830	0.0359491	0.0518955
DTWD1	0.5092906	0.4083991	0.3116317	0.4190877	0.4036978
ATP8B4	0.0027457	0.0066765	0.0057882	0.0091807	0.0742153
SLC27A2	0.0353982	0.0303545	0.0362188	0.0345995	0.0607669
GABPB1	0.1585272	0.1711793	0.1725996	0.1122344	0.2206694
GABPB1-IT1	0.2086333	0.2370484	0.2465492	0.1981451	0.1530945
GABPB1-AS1	0.2583588	0.2725050	0.2478957	0.2151153	0.6418219
ENSG00000259715	0.0019365	0.0000000	0.0008163	0.0056496	0.0039758
USP8	0.6749033	0.5306523	0.3854528	0.6847811	0.6190574
USP50	0.0037236	0.0054780	0.0027444	0.0030679	0.0207582
TRPM7	0.2130360	0.2182255	0.1280961	0.2097538	0.2764713
ENSG00000259684	0.0000000	0.0000000	0.0017450	0.0000000	0.0000000

SPPL2A	0.3404734	0.2504413	0.1893190	0.4475710	0.3888255
ENSG00000273674	0.0167670	0.0224006	0.0121887	0.0382242	0.0594522
AP4E1	0.0481656	0.0658168	0.0500039	0.0629676	0.0884278
MIR4713HG	0.0064421	0.0120597	0.0025371	0.0288473	0.0531334
TNFAIP8L3	0.0220785	0.0159891	0.0255137	0.0592722	0.0000000
CYP19A1	0.0031612	0.0000000	0.0000000	0.0027947	0.0000000
GLDN	0.0037919	0.0138540	0.0295526	0.0060193	0.0448829
ENSG00000259678	0.0005430	0.0028453	0.0028815	0.0031580	0.0196062
DMXL2	0.3213341	0.4058528	0.4251642	0.3385612	0.6563979
SCG3	0.6058086	1.0480805	1.4352588	0.6571117	0.7558892
ENSG00000259241	0.0000000	0.0012480	0.0000000	0.0000000	0.0000000
ENSG00000259296	0.0037898	0.0095508	0.0049451	0.0041691	0.0134182
LYSMD2	0.1103979	0.1659421	0.1615565	0.1458406	0.1119574
TMOD2	1.0552913	1.6578558	1.8072394	1.0611025	1.2661523
ENSG00000259377	0.0008066	0.0000000	0.0000000	0.0000000	0.0000000
TMOD3	0.9071578	0.7681207	0.6151354	0.9584010	0.6888096
ENSG00000259201	0.0000000	0.0014183	0.0000000	0.0000000	0.0000000
ENSG00000259185	0.0160806	0.0164403	0.0108418	0.0300174	0.0038927
LEO1	0.3525732	0.3799692	0.4728281	0.3456295	0.3065553
MAPK6	0.7477710	1.0671705	1.1777021	0.7690891	0.8739572
MAPK6-DT	0.0035774	0.0081806	0.0172826	0.0107145	0.0037431
ENSG00000274528	0.0051379	0.0015454	0.0035045	0.0037663	0.0056888
ENSG00000259712	0.0031433	0.0000000	0.0000000	0.0000000	0.0077648
ENSG00000259178	0.0000000	0.0000000	0.0000000	0.0000000	0.0000000
BCL2L10	0.0000000	0.0037922	0.0020002	0.0000000	0.0148637
GNB5	0.3838065	0.5609657	0.6700372	0.3426143	0.5812950
ENSG00000259327	0.0009158	0.0009635	0.0037378	0.0000000	0.0000000
ENSG00000259709	0.0012749	0.0000000	0.0000000	0.0000000	0.0163219
CERNA1	0.0179677	0.0222664	0.0259570	0.0084745	0.0350743
MYO5C	0.0783062	0.0426171	0.0336005	0.0732000	0.0930142
MYO5A	0.7718169	1.1626953	1.2541829	0.6611004	1.2196999
ARPP19	1.3398597	1.2411785	1.3028358	1.2828480	1.0026377
ENSG00000260618	0.0000000	0.0000000	0.0000000	0.0037492	0.0040472
ATOSA	0.1748334	0.1474842	0.1467677	0.1400016	0.2196770
ENSG00000259935	0.0010608	0.0000000	0.0000000	0.0000000	0.0080628
ENSG00000287500	0.0006211	0.0034193	0.0000000	0.0140744	0.0000000
ONECUT1	0.4979052	1.0140468	1.2624607	0.3617249	1.0185051
ENSG00000259203	0.0410468	0.0923353	0.1532333	0.0333922	0.0643328
LINC02490	0.0016679	0.0078144	0.0052230	0.0000000	0.0437187
ENSG00000287596	0.0000000	0.0000000	0.0013371	0.0000000	0.0028932
WDR72	0.0831377	0.1064551	0.0893797	0.0579190	0.0924610
UNC13C	0.0446367	0.0646370	0.0849167	0.0324494	0.1716611
ENSG00000260937	0.0004832	0.0000000	0.0024882	0.0000000	0.0041053
ENSG00000279145	0.0098291	0.0225524	0.0134411	0.0035651	0.0082019
RSL24D1	1.2912096	1.3005029	1.3816553	1.2377310	1.0232546
RAB27A	0.0326436	0.0382586	0.0404282	0.0384357	0.1265982
ENSG00000276533	0.0024941	0.0010884	0.0000000	0.0081475	0.0000000
PIGBOS1	0.2302173	0.1474498	0.1647464	0.1880804	0.1230989
PIGB	0.0290144	0.0320366	0.0137405	0.0523354	0.0334059
CCPG1	0.9982221	0.7404716	0.6214293	1.2766199	0.7835914
ENSG00000274765	0.0000000	0.0000000	0.0000000	0.0000000	0.0031282
ENSG00000277548	0.0013187	0.0000000	0.0000000	0.0000000	0.0000000
PIERCE2	0.1710024	0.1211840	0.0868693	0.1343972	0.0738102

DNAAF4	0.3660982	0.2822797	0.2317535	0.5592407	0.2190519
PYGO1	0.3553400	0.5171558	0.5306846	0.2990554	0.3761536
ENSG00000285805	0.0196097	0.0113544	0.0214235	0.0189646	0.0144476
PRTG	0.2392274	0.2595337	0.2347340	0.2026533	0.4868335
NEDD4	0.1508308	0.1123988	0.0560819	0.1499194	0.1436936
RFX7	0.3314277	0.4389745	0.4664486	0.3506415	0.8879530
TEX9	0.6901708	0.4951391	0.3713876	0.6815744	0.5835244
ENSG00000275417	0.0000000	0.0000000	0.0000000	0.0000000	0.0000000
ENSG00000259941	0.0069663	0.0047729	0.0025278	0.0068814	0.0310543
ENSG00000261823	0.0006829	0.0010395	0.0027702	0.0000000	0.0000000
MNS1	0.6663639	0.4127091	0.2467201	0.7013832	0.3699312
ENSG00000277245	0.0072835	0.0068018	0.0021106	0.0104095	0.0131820
ENSG00000260392	0.0047927	0.0113283	0.0080442	0.0025494	0.0412788
ZNF280D	0.3436149	0.3216127	0.2972569	0.2826619	0.4410278
ENSG00000274667	0.0002791	0.0000000	0.0000000	0.0068489	0.0000000
TCF12-DT	0.0745788	0.1129916	0.1046509	0.0676629	0.1757033
LINC03065	0.0086559	0.0106408	0.0160115	0.0104423	0.0230566
TCF12	0.6265820	0.4793916	0.3653090	0.6474247	0.9995275
LINC00926	0.0128312	0.0146762	0.0124108	0.0094114	0.0000000
LINC01413	0.0000000	0.0010884	0.0015569	0.0000000	0.0000000
CGNL1	0.0690557	0.0541854	0.0206491	0.0550756	0.0558135
MYZAP	0.0014052	0.0045279	0.0031578	0.0000000	0.0000000
GCOM1	0.0060788	0.0075984	0.0103955	0.0104583	0.0124646
POLR2M	0.2747053	0.2663277	0.2418205	0.2124184	0.2005137
ALDH1A2	0.0224247	0.0141050	0.0153694	0.0264323	0.0680713
LIPC	0.0000000	0.0000000	0.0000000	0.0000000	0.0000000
ENSG00000259250	0.0017396	0.0008686	0.0000000	0.0000000	0.0000000
ADAM10	0.2884682	0.2661282	0.3096382	0.3873043	0.4311486
ENSG00000286897	0.0013676	0.0015739	0.0075711	0.0000000	0.0000000
MINDY2-DT	0.0061825	0.0048353	0.0118189	0.0042547	0.0113539
MINDY2	0.4734726	0.4508698	0.5280658	0.4560580	0.5727452
ENSG00000259353	0.0007392	0.0000000	0.0000000	0.0000000	0.0000000
RNF111	0.2935626	0.3048365	0.2974832	0.2890277	0.4086282
SLTM	0.7100541	0.7429544	0.7943453	0.5635848	0.7377091
ENSG00000288745	0.0000000	0.0000000	0.0049396	0.0000000	0.0019139
CCNB2	0.0142529	0.0261687	0.0098216	0.0207850	0.0264452
ENSG00000277144	0.0006422	0.0010676	0.0000000	0.0030776	0.0000000
ENSG00000259732	0.0022721	0.0053530	0.0000000	0.0012109	0.0000000
MYO1E	0.1338045	0.0755035	0.0588627	0.1272020	0.1264789
ENSG00000259735	0.0000000	0.0000000	0.0029046	0.0000000	0.0023834
FAM81A	0.1481397	0.1971020	0.2128259	0.1305238	0.1916708
GCNT3	0.0046422	0.0032622	0.0072931	0.0037638	0.0162319
GTF2A2	0.7815232	0.7929276	0.8393522	0.8491055	0.6393707
BNIP2	0.3810445	0.2351990	0.1845816	0.4285368	0.2442590
ENSG00000289162	0.0000000	0.0000000	0.0000000	0.0000000	0.0000000
FOXB1	0.0075954	0.0027033	0.0081702	0.0000000	0.0000000
ENSG00000259223	0.0029929	0.0023093	0.0049206	0.0035245	0.0000000
ENSG00000287915	0.0026738	0.0000000	0.0000000	0.0010179	0.0000000
ANXA2	1.9986370	1.4252663	1.0514679	2.0422930	1.2398817
ICE2	0.3283663	0.2358260	0.1864575	0.3232375	0.2969094
RORA-AS1	0.0306953	0.0206065	0.0199793	0.0225353	0.0654361
RORA	0.5239158	0.4721715	0.3041824	0.4387148	1.1006041
ENSG00000273958	0.0054319	0.0060292	0.0022669	0.0058252	0.0000000

ENSG00000286457	0.0000000	0.0010540	0.0000000	0.0034755	0.0000000
ENSG00000287508	0.0000000	0.0000000	0.0000000	0.0000000	0.0000000
ENSG00000259481	0.0008634	0.0036546	0.0020882	0.0000000	0.0429481
ENSG00000259575	0.0015259	0.0000000	0.0000000	0.0000000	0.0000000
ENSG00000259616	0.0123084	0.0075625	0.0048220	0.0031511	0.0205250
ENSG00000259675	0.0011165	0.0000000	0.0000000	0.0017263	0.0000000
ENSG00000259564	0.0023782	0.0019338	0.0019274	0.0000000	0.0173090
VPS13C	0.5387891	0.5422502	0.5338727	0.5291719	0.9888113
VPS13C-DT	0.0127315	0.0084276	0.0070726	0.0090349	0.0000000
C2CD4A	0.0000000	0.0000000	0.0000000	0.0000000	0.0000000
C2CD4B	0.0029413	0.0000000	0.0015765	0.0000000	0.0000000
ENSG00000274340	0.0000000	0.0040052	0.0000000	0.0000000	0.0000000
TLN2	0.2380745	0.4554273	0.5296969	0.2045242	0.7311918
MGC15885	0.0000000	0.0000000	0.0000000	0.0000000	0.0000000
ENSG00000259370	0.0025893	0.0009559	0.0000000	0.0000000	0.0094342
TPM1	1.4155022	1.2864535	1.2605065	1.6099298	1.0620129
TPM1-AS	0.0032626	0.0025459	0.0000000	0.0000000	0.0034000
ENSG00000259627	0.0041451	0.0037621	0.0011261	0.0000000	0.0109605
ENSG00000259672	0.0000000	0.0000000	0.0000000	0.0000000	0.0042310
LACTB	0.2051719	0.2913053	0.3628550	0.2512714	0.1754753
RPS27L	1.4803571	1.1863317	0.9900625	1.5043865	1.1152627
RAB8B	0.3681499	0.3632458	0.4978692	0.4035376	0.4256904
APH1B	0.0611163	0.0520546	0.0549507	0.0897119	0.0758699
CA12	0.0364665	0.0343265	0.0117560	0.1935941	0.0330588
LINC02568	0.0033252	0.0000000	0.0000000	0.0000000	0.0000000
ENSG00000276651	0.0006590	0.0013607	0.0019392	0.0000000	0.0000000
USP3	0.3354377	0.3964820	0.3604426	0.3935329	0.5066452
USP3-AS1	0.0377605	0.0528597	0.0268935	0.0365327	0.1128044
FBXL22	0.0221761	0.0374431	0.0354741	0.0136381	0.0092482
HERC1	0.4740680	0.5964139	0.6805084	0.3781307	1.0244638
ENSG00000259589	0.0019046	0.0019333	0.0064493	0.0000000	0.0402487
DAPK2	0.0807459	0.0376231	0.0224095	0.0664731	0.0698304
CIAO2A	0.3529198	0.2441882	0.2026404	0.3839520	0.2486413
SNX1	0.3513997	0.2357079	0.2100436	0.3643626	0.3092129
SNX22	0.0192325	0.0232614	0.0335911	0.0049853	0.0285369
PPIB	1.7163183	1.4472875	1.4308234	2.0431207	1.3179361
CSNK1G1	0.3417100	0.3355205	0.2624396	0.2600685	0.4967682
PCLAF	0.0665299	0.0480371	0.0384258	0.0912829	0.0440282
TRIP4	0.1699488	0.1614373	0.1717290	0.1719855	0.1533724
ZNF609	0.3224420	0.3050387	0.3138072	0.2913850	0.6483057
OAZ2	1.0084921	0.7980961	0.7217756	0.9469673	0.6499024
ENSG00000265967	0.0000000	0.0000000	0.0000000	0.0000000	0.0000000
ENSG00000264937	0.0000000	0.0000000	0.0000000	0.0000000	0.0000000
ENSG00000259635	0.0045847	0.0028452	0.0029148	0.0087493	0.0356992
RBPMS2	0.0206229	0.0192438	0.0269611	0.0172267	0.0137960
PIF1	0.0013542	0.0008884	0.0020488	0.0035916	0.0102577
PLEKHO2	0.0164220	0.0125036	0.0045881	0.0152380	0.0062659
ENSG00000287494	0.0005748	0.0000000	0.0000000	0.0000000	0.0000000
ANKDD1A	0.0471559	0.0717942	0.0956785	0.0505604	0.1851641
ENSG00000274383	0.0042460	0.0023732	0.0020525	0.0000000	0.0000000
SPG21	0.5519988	0.4814503	0.4869010	0.5244328	0.3916455
MTFMT	0.1446706	0.1150039	0.1316754	0.1215288	0.1703521
SLC51B	0.0082472	0.0063999	0.0026026	0.0170225	0.0000000

RASL12	0.0013455	0.0016393	0.0041536	0.0000000	0.0000000
ENSG00000278737	0.0042174	0.0010040	0.0007240	0.0048559	0.0059911
UBAP1L	0.0318894	0.0477359	0.0291573	0.0082447	0.1538351
PDCD7	0.4045308	0.3414355	0.3074466	0.3518810	0.4381058
CLPX	0.4714526	0.4262380	0.4540509	0.5856415	0.4520676
CILP	0.0048130	0.0044669	0.0022363	0.0106113	0.0082312
PARP16	0.1067318	0.1009412	0.1187654	0.0685946	0.1462449
IGDCC3	0.0928680	0.0799687	0.0932238	0.1333078	0.2195847
IGDCC4	0.0784381	0.0693985	0.0644224	0.1056628	0.1338747
DPP8	0.2780241	0.2712277	0.2853904	0.2650999	0.3143010
HACD3	1.0222519	0.9715485	1.0969992	1.4408987	0.9146899
INTS14	0.1276498	0.0947427	0.1157765	0.1695360	0.1161031
SLC24A1	0.0461787	0.0245459	0.0108546	0.0308065	0.0286019
ENSG00000275638	0.0000000	0.0000000	0.0000000	0.0000000	0.0019139
DENND4A	0.2595415	0.2675287	0.2270492	0.2121846	0.5543659
RAB11A	0.9114258	0.9963481	1.0845218	0.9103797	0.8098558
ENSG00000261544	0.0000000	0.0000000	0.0000000	0.0000000	0.0083237
ENSG00000289992	0.0000000	0.0000000	0.0000000	0.0000000	0.0016673
MEGF11	0.0355976	0.0293951	0.0303456	0.0366762	0.1071615
DIS3L-AS1	0.0045923	0.0014863	0.0000000	0.0000000	0.0000000
DIS3L	0.1814682	0.1571131	0.1486919	0.1818867	0.1070112
ENSG00000260773	0.0000000	0.0045677	0.0000000	0.0000000	0.0000000
TIPIN	0.1450529	0.0992286	0.0930277	0.1387736	0.1170317
MAP2K1	0.2407216	0.2829725	0.3584190	0.2692977	0.2680215
ENSG00000261351	0.0156976	0.0167833	0.0039733	0.0252929	0.0044904
SNAPC5	0.3267229	0.3039934	0.3058106	0.3044609	0.1873492
RPL4	2.5044301	2.5597642	2.4121250	2.4169782	2.2872503
ZWILCH	0.1348375	0.2052889	0.2202336	0.1281301	0.1365484
LCTL	0.0018720	0.0014359	0.0000000	0.0029675	0.0000000
LINC01169	0.0008804	0.0004001	0.0000000	0.0000000	0.0083854
SMAD6	0.0258184	0.0116485	0.0064712	0.0322692	0.0087392
ENSG00000259437	0.0000000	0.0008530	0.0000000	0.0000000	0.0040644
ENSG00000259347	0.0082143	0.0041073	0.0000000	0.0032157	0.0031757
SMAD3	0.2423004	0.1246267	0.0788973	0.2305046	0.1134964
ENSG00000259202	0.0000000	0.0000000	0.0000000	0.0000000	0.0000000
AAGAB	0.3978422	0.3981134	0.4638191	0.4190258	0.2935357
IQCH	0.1795855	0.1510983	0.0867019	0.1249918	0.1689890
IQCH-AS1	0.0702947	0.0613115	0.0541963	0.1011882	0.0847727
C15orf61	0.2754649	0.3608983	0.4046077	0.2641565	0.2753344
MAP2K5-DT	0.0092484	0.0061658	0.0027302	0.0063116	0.0138539
MAP2K5	0.2054721	0.2206573	0.2028323	0.1794807	0.3818703
SKOR1	0.0108962	0.0155749	0.0406671	0.0219263	0.0236354
ENSG00000259410	0.0058515	0.0022807	0.0025433	0.0044583	0.0112177
SKOR1-AS1	0.0000000	0.0011302	0.0008906	0.0000000	0.0000000
PIAS1	0.6813620	0.6440806	0.6767573	0.5788623	0.7579943
CALML4	0.0650442	0.0638699	0.0617625	0.0489293	0.0807584
CLN6	0.0220467	0.0389752	0.0165659	0.0304698	0.0410315
ENSG00000260657	0.0019279	0.0078289	0.0044889	0.0000000	0.0000000
FEM1B	0.5882136	0.6862571	0.8592911	0.5782557	0.7136051
ITGA11	0.0000000	0.0020208	0.0064368	0.0030695	0.0000000
CORO2B	0.1569878	0.1823750	0.1816722	0.2223724	0.2915697
ANP32A	1.1430554	1.0763260	1.0556564	1.1259223	0.9093477
SPESP1	0.0200335	0.0454601	0.0204390	0.0200295	0.0921939

ENSG00000285919	0.0007431	0.0000000	0.0000000	0.0000000	0.0012783
NOX5	0.0022157	0.0000000	0.0000000	0.0000000	0.0013524
EWSAT1	0.0024791	0.0020718	0.0012127	0.0040080	0.0000000
GLCE	0.1004015	0.1268348	0.1368398	0.1019073	0.2162980
PAQR5-DT	0.0000000	0.0000000	0.0000000	0.0000000	0.0000000
ENSG00000259504	0.0000000	0.0000000	0.0000000	0.0000000	0.0000000
PAQR5	0.0021508	0.0000000	0.0007564	0.0000000	0.0042020
KIF23-AS1	0.1211888	0.0861155	0.0463925	0.1086974	0.0560951
KIF23	0.0138370	0.0162763	0.0073163	0.0128971	0.0000000
RPLP1	3.6798897	3.6061820	3.4150123	3.5862119	3.3929200
LINC02896	0.0040724	0.0024835	0.0021739	0.0000000	0.0000000
DRAIC	0.0487200	0.0662822	0.0631225	0.0659477	0.1385207
TLE3	0.1296135	0.1237819	0.1306552	0.1423977	0.2315140
UACA	0.1772242	0.1188843	0.0745286	0.1634930	0.1814076
LARP6	0.3342435	0.4029450	0.3080741	0.2981718	0.2314861
LRRC49	0.5204621	0.5841702	0.5643895	0.4929255	0.7089497
THAP10	0.1047483	0.1044288	0.1315424	0.1442833	0.0863578
THSD4	0.1568012	0.1184986	0.0848988	0.2162731	0.1771391
THSD4-AS1	0.0367228	0.0185802	0.0450131	0.0094916	0.0154116
NR2E3	0.0033404	0.0000000	0.0006194	0.0015202	0.0162631
ENSG00000260037	0.0010210	0.0000000	0.0000000	0.0000000	0.0141731
MYO9A	0.4915920	0.6098211	0.5892968	0.4248281	0.9916141
ENSG00000261632	0.0029776	0.0044319	0.0000000	0.0030679	0.0183786
SENP8	0.0888147	0.0947261	0.0967292	0.0839788	0.0659143
ENSG00000260173	0.0009836	0.0020668	0.0009480	0.0000000	0.0000000
GRAMD2A	0.0063679	0.0031959	0.0000000	0.0000000	0.0068871
PKM	1.5393579	1.4686214	1.5357150	1.5457266	1.2624209
PARP6	0.2538123	0.3718811	0.4075800	0.2407175	0.3072660
ENSG00000261460	0.0034032	0.0023249	0.0020099	0.0154960	0.0330755
CELF6	0.0902428	0.2013305	0.3319715	0.0596575	0.1970377
HEXA	0.3343712	0.1982365	0.1647990	0.5538150	0.2427590
HEXA-AS1	0.0007972	0.0019515	0.0044575	0.0000000	0.0000000
TMEM202-AS1	0.0112269	0.0149935	0.0110262	0.0117400	0.0571912
ENSG00000261187	0.0000000	0.0000000	0.0000000	0.0000000	0.0036228
ARIH1	0.5734530	0.6476297	0.6863776	0.5417448	1.0292560
LINC02259	0.0000000	0.0009103	0.0039600	0.0000000	0.0117719
ENSG00000260672	0.0012548	0.0000000	0.0000000	0.0000000	0.0000000
GOLGA6B	0.0006187	0.0021701	0.0000000	0.0035938	0.0000000
HIGD2B	0.0008319	0.0000000	0.0037099	0.0000000	0.0000000
BBS4	0.2999753	0.2197594	0.2174021	0.3055551	0.2313148
ADPGK	0.0803307	0.1017282	0.0811113	0.0810683	0.1416458
ADPGK-AS1	0.0006466	0.0000000	0.0000000	0.0000000	0.0052561
NEO1	0.4677430	0.6032371	0.7257014	0.5061926	0.8400152
HCN4	0.0351528	0.0438299	0.0637932	0.0070676	0.0563430
REC114	0.0000000	0.0016015	0.0032859	0.0000000	0.0036741
NPTN	0.5727121	0.5863851	0.6822970	0.7085442	0.6691768
NPTN-IT1	0.0008275	0.0025671	0.0024822	0.0015713	0.0247241
CD276	0.4323425	0.3434948	0.2803789	0.5580166	0.3576145
INSYN1	0.0360566	0.0592751	0.0439261	0.0213325	0.0850922
INSYN1-AS1	0.0152296	0.0202397	0.0358353	0.0152961	0.0298784
LOXL1-AS1	0.0421451	0.0353562	0.0347840	0.0459011	0.0091327
LOXL1	0.0867700	0.1034085	0.0795247	0.1461194	0.1247621
STOML1	0.0754765	0.1050328	0.1237748	0.1038104	0.1024786

PML	0.0532505	0.0945650	0.0448376	0.0721883	0.0393211
ISLR2	0.0857931	0.1655969	0.2957697	0.0755179	0.1729038
ENSG00000248540	0.0083725	0.0243721	0.0093414	0.0094838	0.0119123
ENSG00000261543	0.0000000	0.0000000	0.0040321	0.0000000	0.0000000
ISLR	0.0219142	0.0123859	0.0131702	0.0399426	0.0139481
STRA6	0.0098245	0.0037838	0.0000000	0.0053184	0.0049696
CCDC33	0.0047117	0.0045352	0.0053027	0.0024107	0.0012623
CYP11A1	0.0042360	0.0046961	0.0055843	0.0041570	0.0048514
ENSG00000261821	0.0011766	0.0000000	0.0016417	0.0000000	0.0000000
SEMA7A	0.0298222	0.0687211	0.0683602	0.0157837	0.0884919
UBL7	0.1970090	0.1947612	0.1950414	0.2347103	0.0805318
UBL7-DT	0.0522213	0.0497506	0.0347334	0.0440364	0.0669216
ARID3B	0.0329861	0.0410223	0.0623831	0.0212643	0.1212790
CLK3	0.1674350	0.1509892	0.1284473	0.1589156	0.1384852
ENSG00000260919	0.0000000	0.0035452	0.0000000	0.0000000	0.0041350
EDC3	0.1020750	0.0996910	0.1016887	0.0943913	0.1050833
CYP1A1	0.0012360	0.0033260	0.0020267	0.0000000	0.0103519
CYP1A2	0.0000000	0.0028758	0.0026075	0.0000000	0.0000000
CSK	0.1371383	0.1619873	0.2099550	0.1023666	0.1927703
LMAN1L	0.0000000	0.0022373	0.0037823	0.0000000	0.0000000
CPLX3	0.0004116	0.0037377	0.0048114	0.0000000	0.0017986
ULK3	0.0882751	0.0941984	0.0841044	0.1060115	0.1122880
SCAMP2	0.2934379	0.1782485	0.1144487	0.3409557	0.0884572
MPI	0.2039173	0.1406681	0.1769093	0.2093344	0.1245855
FAM219B	0.3179154	0.3048455	0.2985075	0.3545965	0.2762219
COX5A	1.3915438	1.5897182	1.6572916	1.3713246	1.2299729
RPP25	0.0284037	0.0480833	0.0695981	0.0194783	0.0227443
SCAMP5	0.1636058	0.2825343	0.4034085	0.1528879	0.2744006
PPCDC	0.0748348	0.0656848	0.0418131	0.0695588	0.1792028
C15orf39	0.0240166	0.0231844	0.0286652	0.0204652	0.0267318
GOLGA6C	0.0000000	0.0051376	0.0020998	0.0000000	0.0000000
COMMD4	0.1271420	0.1028309	0.0816188	0.1326753	0.0997496
ENSG00000275645	0.0232682	0.0432682	0.0341099	0.0275351	0.0168813
NEIL1	0.0161572	0.0257592	0.0099147	0.0148447	0.0527225
MAN2C1	0.0570130	0.0618139	0.0667436	0.0764966	0.0853945
ENSG00000260274	0.0123791	0.0148870	0.0079621	0.0218646	0.0000000
SIN3A	0.1912361	0.1686780	0.1718786	0.1844596	0.1877787
PTPN9	0.1304257	0.1672766	0.1829626	0.1237529	0.2347494
SNUPN	0.1621969	0.1468038	0.1914008	0.1456226	0.1321821
IMP3	0.4162428	0.3410924	0.4168928	0.4262578	0.2729425
ENSG00000275454	0.0026655	0.0000000	0.0097178	0.0000000	0.0000000
SNX33	0.0312797	0.0078026	0.0000000	0.0113956	0.0266713
CSPG4	0.0000000	0.0011535	0.0066195	0.0011917	0.0044624
ODF3L1	0.0193250	0.0066101	0.0140808	0.0116754	0.0108974
DNM1P35	0.0415244	0.0618965	0.0647662	0.0242334	0.0242915
ENSG00000260288	0.0029445	0.0089652	0.0083641	0.0055630	0.0061018
UBE2Q2	0.4387701	0.3745358	0.4333492	0.4487558	0.4077070
FBXO22	0.3424090	0.3867770	0.3622729	0.3700771	0.3851028
NRG4	0.0800490	0.0697028	0.0432540	0.0519936	0.1001078
TMEM266	0.0655879	0.0744621	0.0943110	0.0701016	0.0727108
ENSG00000259422	0.0000000	0.0000000	0.0000000	0.0000000	0.0000000
ETFA	0.5922530	0.5954396	0.5372452	0.6161632	0.3627241
ENSG00000287503	0.0000000	0.0000000	0.0000000	0.0000000	0.0042562

ISL2	0.0096707	0.0357514	0.0075489	0.0030760	0.0030611
SCAPER	0.6727439	0.8058097	0.8737183	0.6002113	1.1146950
RCN2	1.3572420	1.1574887	1.2763827	1.6243400	1.1030673
PSTPIP1	0.0075222	0.0041423	0.0000000	0.0212020	0.0121029
TSPAN3	1.5147964	1.1420908	1.0776821	1.8652258	1.0623037
ENSG00000269951	0.0010754	0.0050216	0.0092400	0.0000000	0.0000000
PEAK1	0.2418339	0.2273246	0.2170361	0.1812834	0.4566585
HMG20A	0.2365191	0.2935934	0.2693504	0.2053997	0.3272420
ENSG00000259362	0.0018485	0.0000000	0.0013494	0.0000000	0.0000000
ENSG00000259420	0.0044853	0.0153812	0.0013636	0.0181992	0.0211363
LINGO1	0.3871003	0.4922300	0.6397081	0.4457195	0.5844291
LINGO1-AS1	0.0009146	0.0000000	0.0000000	0.0000000	0.0048296
LINGO1-AS2	0.0000000	0.0005504	0.0021524	0.0000000	0.0000000
ENSG00000290665	0.0074153	0.0079318	0.0020559	0.0149782	0.0037075
ENSG00000290666	0.0016137	0.0041283	0.0011924	0.0059843	0.0000000
ENSG00000261403	0.0000000	0.0000000	0.0000000	0.0000000	0.0123246
TBC1D2B	0.0586991	0.0674126	0.0770610	0.0891866	0.0921110
SH2D7	0.0052774	0.0034473	0.0000000	0.0000000	0.0075813
CIB2	0.4438736	0.6885300	0.7071696	0.4240289	0.4306152
IDH3A	0.2422067	0.2772559	0.3990530	0.1807759	0.2367961
ACSBG1	0.0061711	0.0028989	0.0061354	0.0106900	0.0215803
DNAJA4-DT	0.0000000	0.0000000	0.0000000	0.0000000	0.0000000
DNAJA4	0.0794404	0.1410298	0.2048942	0.0912921	0.1059777
SKIC8	0.2761759	0.2917499	0.3013891	0.3025044	0.2533748
ENSG00000259322	0.0000000	0.0000000	0.0000000	0.0000000	0.0012623
CRABP1	0.9547869	1.6347738	2.3296998	0.8176840	1.2705650
IREB2	0.4316951	0.5130575	0.5099428	0.4123956	0.5559342
ENSG00000259474	0.0006087	0.0000000	0.0000000	0.0000000	0.0000000
HYKK	0.0261308	0.0157322	0.0082692	0.0269224	0.0069878
PSMA4	1.1257279	1.0691280	1.1743253	1.1392814	0.8952517
CHRNA5	0.0057937	0.0200584	0.0337657	0.0106161	0.0284816
ENSG00000261762	0.0013224	0.0014908	0.0024882	0.0000000	0.0000000
CHRNA3	0.0627260	0.0734581	0.0551946	0.1132923	0.0689130
CHRNB4	0.0074399	0.0164248	0.0096651	0.0036365	0.0319284
ENSG00000259555	0.0018371	0.0020400	0.0030757	0.0000000	0.0034872
ENSG00000290426	0.0000000	0.0000000	0.0026158	0.0000000	0.0080500
ADAMTS7	0.0104938	0.0083088	0.0081797	0.0142352	0.0155317
MORF4L1	2.4198720	2.4258698	2.6799857	2.3650281	2.1354288
CTSH	0.1090399	0.0520082	0.0376277	0.2524340	0.0636783
RASGRF1	0.0829504	0.1700323	0.2093429	0.0548556	0.3106587
ENSG00000177699	0.0000000	0.0000000	0.0031196	0.0000000	0.0000000
ANKRD34C-AS1	0.0031280	0.0126291	0.0265261	0.0000000	0.0056513
ANKRD34C	0.0000000	0.0040617	0.0000000	0.0000000	0.0044810
TMED3	0.3561534	0.3837126	0.4125080	0.4341919	0.3328612
MINAR1	0.1649552	0.3911548	0.4605609	0.1651024	0.3999407
MTHFS	0.1723944	0.2154573	0.2270193	0.1278723	0.1821458
ENSG00000286813	0.0021167	0.0000000	0.0000000	0.0047879	0.0000000
ST20	0.1559482	0.1807657	0.1646347	0.1339215	0.1690971
ENSG00000278600	0.0041345	0.0128627	0.0036870	0.0019936	0.0199899
ST20-AS1	0.0217664	0.0299403	0.0225783	0.0510245	0.0228958
ZFAND6	0.9151347	0.9354270	0.8882983	0.8911820	0.7907555
FAH	0.0954751	0.0712854	0.0529447	0.1030898	0.0573877
CTXND1	0.0050342	0.0032300	0.0055795	0.0038289	0.0000000

LINC00927	0.0026505	0.0040191	0.0044845	0.0000000	0.0048241
ARNT2-DT	0.0723887	0.0865021	0.0761573	0.0465098	0.0479415
ARNT2	0.2136584	0.2922814	0.3676222	0.2073349	0.3781585
ENSG00000259175	0.0000000	0.0000000	0.0000000	0.0000000	0.0000000
ABHD17C	0.2481198	0.2734235	0.3680180	0.3353138	0.2271670
ENSG00000271983	0.0000000	0.0000000	0.0000000	0.0000000	0.0000000
CEMIP	0.0199214	0.0404842	0.0537561	0.0439097	0.1600048
ENSG00000259649	0.0026433	0.0020158	0.0000000	0.0000000	0.0154896
MESD	0.5818795	0.3921242	0.4132426	0.7567205	0.4019448
ENSG00000277782	0.0032947	0.0083129	0.0044674	0.0023089	0.0081743
TLNRD1	0.1046013	0.2071520	0.2893219	0.1010150	0.1966563
CFAP161	0.0748176	0.0614539	0.0399407	0.0919179	0.0681629
IL16	0.0113332	0.0107807	0.0000000	0.0049137	0.0000000
ENSG00000271725	0.0000000	0.0000000	0.0000000	0.0000000	0.0000000
STARD5	0.0031239	0.0076722	0.0012784	0.0027871	0.0073536
ENSG00000286488	0.0003128	0.0014000	0.0000000	0.0000000	0.0000000
TMC3-AS1	0.0242257	0.0563197	0.0428541	0.0332534	0.0984113
TMC3	0.0000000	0.0030633	0.0019328	0.0000000	0.0014580
ENSG00000259543	0.0131531	0.0086917	0.0259741	0.0098976	0.0515579
ENSG00000259594	0.0009730	0.0015181	0.0037719	0.0017492	0.0041350
ENSG00000259692	0.1307535	0.1627958	0.1378149	0.1209015	0.4102857
MEX3B	0.0859783	0.1442748	0.1255493	0.0908124	0.1036193
EFL1	0.1782274	0.1765058	0.1466965	0.1920809	0.1697406
ENSG00000285974	0.0000000	0.0000000	0.0010324	0.0000000	0.0000000
SAXO2	0.1166203	0.0771333	0.0602312	0.1132708	0.0887878
ENSG00000290944	0.0000000	0.0012671	0.0000000	0.0000000	0.0058440
GOLGA6L10	0.0335456	0.0273819	0.0247653	0.0394372	0.0628951
UBE2Q2P2	0.1487315	0.1692244	0.1261861	0.1431001	0.3601476
GOLGA6L9	0.0336558	0.0404063	0.0381119	0.0225277	0.0420758
ENSG00000290946	0.0000000	0.0000000	0.0027209	0.0000000	0.0061370
ENSG00000290947	0.0017043	0.0031955	0.0028174	0.0000000	0.0000000
ENSG00000290948	0.1084644	0.1184952	0.1010185	0.1115161	0.1233504
ENSG00000290949	0.0000000	0.0000000	0.0000000	0.0000000	0.0000000
RPS17	2.6514698	2.6276271	2.4739765	2.5810218	2.3662144
ENSG00000278013	0.0000000	0.0000000	0.0000000	0.0056277	0.0100627
CPEB1	0.1537412	0.2032133	0.1471728	0.1040452	0.2114057
CPEB1-AS1	0.0067844	0.0079111	0.0068047	0.0122162	0.0202415
AP3B2	0.0719576	0.1440950	0.2147390	0.0374941	0.2307491
ENSG00000259442	0.0022384	0.0000000	0.0000000	0.0000000	0.0045070
ENSG00000286817	0.0006491	0.0007528	0.0021636	0.0025316	0.0107101
SNHG21	0.0867726	0.0746054	0.0831703	0.0940013	0.0604372
FSD2	0.0013530	0.0000000	0.0020198	0.0018752	0.0058330
WHAMM	0.1713367	0.1259731	0.0918636	0.1720249	0.1144596
HOMER2	0.2119969	0.4071293	0.3392011	0.1922313	0.3377932
ENSG00000259805	0.0106881	0.0089855	0.0143346	0.0000000	0.0473529
RAMAC	0.1072521	0.1239080	0.1305506	0.0835535	0.0673654
C15orf40	0.3523715	0.2768188	0.2877930	0.3865152	0.2838895
ENSG00000288850	0.0019230	0.0052937	0.0075032	0.0021832	0.0120772
BTBD1	0.6226278	0.5187705	0.6030491	0.6687462	0.4714555
ENSG00000259767	0.0000000	0.0018508	0.0000000	0.0000000	0.0084988
ENSG00000286872	0.0277176	0.0394580	0.0397383	0.0288750	0.1421354
TM6SF1	0.0128595	0.0187726	0.0151133	0.0179433	0.0104156
HDGFL3	1.1236551	1.4921733	1.8218640	1.0401662	1.3217095

ENSG00000259986	0.0236832	0.0290679	0.0202674	0.0129581	0.1045413
BNC1	0.0271683	0.0136111	0.0054774	0.0201905	0.0052465
SH3GL3	0.4776840	0.5146247	0.5053482	0.4655793	0.5997823
ADAMTSL3	0.0082068	0.0080345	0.0082428	0.0109244	0.0703403
ENSG00000291062	0.0105154	0.0264902	0.0226314	0.0050286	0.0776601
ENSG00000290688	0.0043862	0.0039566	0.0054375	0.0024360	0.0306925
ENSG00000290689	0.1360314	0.1495655	0.1406038	0.1223053	0.0958654
GOLGA6L4	0.0054104	0.0097256	0.0124104	0.0000000	0.0325365
ENSG00000290690	0.0022717	0.0041236	0.0091435	0.0018058	0.0358911
ENSG00000291026	0.0010288	0.0021186	0.0036192	0.0031580	0.0013712
UBE2Q2P1	0.0753024	0.1091389	0.0741895	0.0628255	0.3051943
LINC00933	0.0037828	0.0032833	0.0071374	0.0000000	0.0000000
ZSCAN2-AS1	0.0142659	0.0169130	0.0135640	0.0178626	0.0554116
ZSCAN2	0.0441020	0.0565972	0.0548762	0.0369075	0.0843638
ENSG00000276278	0.0065166	0.0069432	0.0066760	0.0051578	0.0000000
ENSG00000291159	0.0414108	0.0659553	0.0317732	0.0377408	0.0770896
WDR73	0.1781349	0.1660218	0.1305389	0.1518328	0.1667117
NMB	0.3625426	0.3083943	0.2745870	0.4566326	0.2557149
SEC11A	1.0592018	0.9638400	0.9152250	1.2577810	0.8533891
ENSG00000285667	0.0294763	0.0270128	0.0261363	0.0534127	0.0965126
ZNF592	0.1363118	0.1248400	0.1236205	0.1078769	0.1653363
ALPK3	0.0888267	0.0688233	0.0548140	0.0688200	0.0982447
SLC28A1	0.0031722	0.0058372	0.0015777	0.0025371	0.0092834
ENSG00000286505	0.0000000	0.0000000	0.0000000	0.0000000	0.0000000
PDE8A	0.1075487	0.1236084	0.0848086	0.0728893	0.4829982
ENSG00000291210	0.0039291	0.0089161	0.0046407	0.0031073	0.0180357
ENSG00000291211	0.0075153	0.0171111	0.0040109	0.0089719	0.0350710
AKAP13	0.5390297	0.4159718	0.3230476	0.4467523	0.4907180
AKAP13-AS1	0.0063132	0.0039782	0.0023508	0.0046461	0.0168908
ENSG00000259544	0.0000000	0.0000000	0.0000000	0.0000000	0.0000000
ENSG00000259407	0.0219954	0.0066264	0.0118933	0.0284585	0.0092727
LINC02883	0.0021572	0.0000000	0.0000000	0.0027641	0.0030127
KLHL25	0.0479933	0.0304277	0.0309562	0.0757749	0.0665128
LINC01584	0.0000000	0.0000000	0.0000000	0.0000000	0.0000000
AGBL1	0.0040982	0.0035711	0.0032754	0.0000000	0.0152829
ENSG00000259560	0.0037957	0.0032852	0.0007884	0.0000000	0.0188566
NTRK3	0.2341907	0.3571085	0.4419031	0.2345389	0.8550900
NTRK3-AS1	0.0025248	0.0059283	0.0093840	0.0067148	0.0042865
MRPL46	0.2263835	0.2347918	0.2696213	0.2634831	0.1545787
MRPS11	0.2407303	0.2434756	0.2973450	0.3069826	0.2259266
DET1	0.0742055	0.0793103	0.0554356	0.0543854	0.0972307
LINC01586	0.0053244	0.0094195	0.0105939	0.0038281	0.0209665
AEN	0.0977839	0.0425377	0.0396230	0.0754928	0.0561453
ISG20	0.0256811	0.0310973	0.0114461	0.0356562	0.0080942
ACAN	0.0000000	0.0000000	0.0012131	0.0000000	0.0028551
HAPLN3	0.0002291	0.0000000	0.0025433	0.0000000	0.0000000
MFGE8	0.0339922	0.0252248	0.0130843	0.0931427	0.0213175
CARMAL	0.0042331	0.0010689	0.0022183	0.0000000	0.0000000
ENSG00000261407	0.0024966	0.0000000	0.0000000	0.0000000	0.0000000
ABHD2	0.3179742	0.3083138	0.2480383	0.3470203	0.2866101
RLBP1	0.0000000	0.0000000	0.0000000	0.0000000	0.0000000
FANCI	0.0244957	0.0226466	0.0058942	0.0357383	0.0361110
POLG	0.0514733	0.0501189	0.0420155	0.0504557	0.1002443

POLG-DT	0.0029104	0.0030112	0.0000000	0.0000000	0.0000000
MIR9-3HG	0.0382996	0.0144761	0.0070571	0.0567020	0.0173173
ENSG00000289128	0.0016207	0.0000000	0.0000000	0.0040303	0.0034000
RHCG	0.0143868	0.0103751	0.0081947	0.0287210	0.0339192
LINC00928	0.0000000	0.0000000	0.0000000	0.0000000	0.0000000
ENSG00000259713	0.0019478	0.0018350	0.0000000	0.0114130	0.0037236
TICRR	0.0000000	0.0034956	0.0000000	0.0000000	0.0000000
KIF7	0.0265401	0.0294767	0.0206768	0.0391748	0.0737270
PLIN1	0.0005537	0.0026749	0.0020936	0.0033167	0.0000000
PEX11A	0.1990701	0.1713392	0.2008268	0.2206627	0.0982601
WDR93	0.0996808	0.0502668	0.0371919	0.0535615	0.0528057
MESP1	0.1716956	0.2735667	0.3732608	0.1436812	0.1530523
MESP2	0.0000000	0.0059355	0.0044055	0.0000000	0.0040093
ANPEP	0.0000000	0.0000000	0.0055131	0.0000000	0.0000000
AP3S2	0.3035747	0.2930309	0.2906392	0.2583239	0.3115690
ARPIN	0.3612031	0.1967926	0.2068551	0.2956962	0.1743651
ZNF710	0.0448216	0.0685819	0.0556881	0.0300193	0.1099595
ENSG00000273691	0.0019786	0.0009628	0.0000000	0.0000000	0.0000000
ZNF710-AS1	0.0841472	0.0816008	0.0713418	0.0666633	0.0612384
IDH2	0.4295700	0.3832492	0.4280094	0.5556561	0.3332768
IDH2-DT	0.0013689	0.0040635	0.0040858	0.0088470	0.0317854
SEMA4B	0.0739997	0.0632749	0.0548924	0.0938463	0.1151619
CIB1	0.6066949	0.4096433	0.2769313	0.7048025	0.3212345
GDPGP1	0.0305652	0.0162420	0.0179173	0.0172831	0.0164115
TTLL13	0.0099773	0.0114275	0.0131976	0.0095959	0.0324583
NGRN	1.4052189	1.5893854	1.7966903	1.3481247	1.2481106
ENSG00000275345	0.0021650	0.0022964	0.0000000	0.0000000	0.0025298
ZNF774	0.0282572	0.0514539	0.0290696	0.0217695	0.0287262
IQGAP1	0.2654088	0.1779449	0.1128992	0.3398424	0.2742680
ENSG00000259177	0.0000000	0.0010122	0.0000000	0.0000000	0.0000000
CRTC3	0.1243119	0.0799766	0.0798052	0.1140096	0.2331586
ENSG00000259314	0.0009164	0.0011542	0.0048144	0.0000000	0.0047937
CRTC3-AS1	0.0149231	0.0169297	0.0215065	0.0262724	0.0774792
ENSG00000278370	0.0000000	0.0041985	0.0032346	0.0000000	0.0000000
BLM	0.0179316	0.0360901	0.0447711	0.0350887	0.0149290
FURIN	0.0530645	0.0420070	0.0366739	0.0535289	0.0312386
FES	0.0063299	0.0069814	0.0052388	0.0060087	0.0119516
MAN2A2	0.0775285	0.0802169	0.0692013	0.0823983	0.0899641
ENSG00000259661	0.0022105	0.0005320	0.0009118	0.0000000	0.0000000
HDDC3	0.1679604	0.1477344	0.1634523	0.1629379	0.0945454
UNC45A	0.1135476	0.0652738	0.0490673	0.1318134	0.1183323
RCCD1-AS1	0.0000000	0.0000000	0.0000000	0.0000000	0.0000000
RCCD1	0.1007548	0.0974276	0.1143391	0.1379478	0.0870341
PRC1	0.0964103	0.1182624	0.1082125	0.1382535	0.0765727
PRC1-AS1	0.0061968	0.0133265	0.0085858	0.0035717	0.0314183
VPS33B	0.0724060	0.0778144	0.0783151	0.0745728	0.0728794
VPS33B-DT	0.0169109	0.0143178	0.0166229	0.0288748	0.0154697
ENSG00000278514	0.0004507	0.0016953	0.0031149	0.0000000	0.0020430
SV2B	0.0443535	0.0662732	0.1513449	0.0127039	0.1986681
ENSG00000285883	0.0000000	0.0000000	0.0000000	0.0000000	0.0000000
CRAT37	0.0000000	0.0033002	0.0026141	0.0000000	0.0022413
ENSG00000258765	0.0000000	0.0024816	0.0000000	0.0000000	0.0000000
SLCO3A1	0.1931600	0.1585873	0.1405026	0.2145199	0.4290861

ENSG00000260661	0.0018475	0.0017338	0.0026613	0.0048092	0.0094410
ST8SIA2	0.1579724	0.1984959	0.3159885	0.1199085	0.2837103
C15orf32	0.0021466	0.0031128	0.0026872	0.0000000	0.0068388
LINC00930	0.0011999	0.0010248	0.0000000	0.0081632	0.0050073
ENSG00000260337	0.0081410	0.0039509	0.0013632	0.0050812	0.0048680
ENSG00000271763	0.0016347	0.0000000	0.0000000	0.0000000	0.0040877
FAM174B	0.2674848	0.1811636	0.1324346	0.1979981	0.1710287
CHASERR	1.6436433	1.4960262	1.5147262	1.5729676	1.3557942
ENSG00000289200	0.0039765	0.0019648	0.0015844	0.0000000	0.0013244
ENSG00000288872	0.0017893	0.0024339	0.0023468	0.0000000	0.0183935
CHD2	0.8089349	0.8765754	0.8921388	0.7696888	1.1093679
ENSG00000289017	0.0034554	0.0023440	0.0013709	0.0000000	0.0136233
RGMA	0.7278801	0.5354400	0.5227090	1.0200417	0.4906024
ENSG00000288991	0.0047564	0.0014171	0.0010944	0.0000000	0.0000000
ENSG00000257060	0.1492345	0.1091976	0.0806984	0.0943897	0.2100382
ENSG00000258631	0.1084053	0.1027968	0.0612067	0.1080157	0.3348196
LINC01579	0.0132355	0.0109679	0.0121060	0.0050803	0.0556284
LINC02207	0.0035564	0.0060746	0.0024615	0.0060237	0.0106175
ENSG00000258831	0.0032099	0.0017070	0.0000000	0.0000000	0.0064395
LINC01580	0.0040453	0.0075362	0.0012964	0.0037958	0.0225192
MCTP2	0.0042075	0.0017554	0.0032611	0.0000000	0.0037529
LETR1	0.0253584	0.0678563	0.1038155	0.0305152	0.0715674
LINC00924	0.0006855	0.0000000	0.0000000	0.0000000	0.0039740
ENSG00000275016	0.0000000	0.0000000	0.0000000	0.0000000	0.0000000
ENSG00000275443	0.0042187	0.0012225	0.0000000	0.0000000	0.0089209
ENSG00000273771	0.0010224	0.0042247	0.0024426	0.0000000	0.0000000
NR2F2-AS1	0.0357800	0.0469820	0.0353845	0.0357042	0.1444972
ENSG00000277135	0.0008836	0.0000000	0.0000000	0.0000000	0.0000000
NR2F2	0.3896487	0.4978921	0.2038197	0.3856979	0.3520553
ENSG00000259275	0.0023342	0.0016347	0.0000000	0.0000000	0.0000000
LINC02253	0.0079353	0.0031111	0.0070243	0.0000000	0.0000000
ENSG00000259403	0.0000000	0.0000000	0.0000000	0.0022801	0.0112046
LINC02254	0.0000000	0.0000000	0.0000000	0.0000000	0.0057257
LINC00923	0.0070185	0.0130762	0.0039145	0.0143116	0.0363579
ARRDC4	0.5554109	0.5159738	0.3466450	0.5259328	0.4159043
ENSG00000259199	0.0108665	0.0127904	0.0048867	0.0099592	0.0335246
IRAIN	0.0098268	0.0081246	0.0062163	0.0169574	0.0045792
IGF1R	0.4056693	0.3952757	0.3572075	0.4827619	0.8058977
ENSG00000278022	0.0000000	0.0000000	0.0000000	0.0040841	0.0000000
ENSG00000287191	0.0019650	0.0012552	0.0000000	0.0000000	0.0000000
ENSG00000259621	0.0000000	0.0000000	0.0000000	0.0000000	0.0000000
SYNM-AS1	0.0109633	0.0047504	0.0076094	0.0051260	0.0383942
PGPEP1L	0.0023271	0.0010850	0.0024615	0.0000000	0.0000000
LUNAR1	0.0000000	0.0027244	0.0010211	0.0000000	0.0000000
SYNM	0.2465302	0.2482332	0.1966250	0.2980242	0.2617199
SYNM-AS2	0.0046189	0.0032105	0.0017569	0.0134742	0.0139987
TTC23	0.1796391	0.1056156	0.0636718	0.1413495	0.1447447
TTC23-AS1	0.0013070	0.0038862	0.0000000	0.0000000	0.0000000
ENSG00000259921	0.0000000	0.0000000	0.0000000	0.0000000	0.0000000
LRRC28	0.2480387	0.2429487	0.2667399	0.2582059	0.3142964
ENSG00000259760	0.0000000	0.0000000	0.0000000	0.0000000	0.0034716
MEF2A	0.4830672	0.4755578	0.4395896	0.3358589	0.6200918
LYSMD4	0.0476121	0.0468152	0.0441922	0.0314684	0.0324693

ENSG00000290767	0.0039758	0.0026237	0.0000000	0.0247696	0.0000000
ENSG00000259363	0.0301868	0.0308447	0.0285128	0.0203975	0.0799727
ADAMTS17	0.0139420	0.0064348	0.0066820	0.0086737	0.0070574
SPATA41	0.0007911	0.0000000	0.0000000	0.0059615	0.0056738
CERS3-AS1	0.0012744	0.0039811	0.0000000	0.0065919	0.0000000
CERS3	0.0027755	0.0026224	0.0000000	0.0074578	0.0193104
ENSG00000270127	0.0021449	0.0000000	0.0036294	0.0000000	0.0000000
LINS1	0.0740487	0.1097773	0.0722895	0.0670839	0.0834254
ASB7	0.0840366	0.0844685	0.0755548	0.0402642	0.0629889
ENSG00000259579	0.0000000	0.0009703	0.0000000	0.0022166	0.0000000
ENSG00000232386	0.0034516	0.0036412	0.0029356	0.0014695	0.0000000
ALDH1A3	0.0014787	0.0000000	0.0000000	0.0000000	0.0000000
LRRK1	0.0071970	0.0037401	0.0000000	0.0018326	0.0031451
ENSG00000259376	0.0034985	0.0086938	0.0067471	0.0045408	0.0078184
ENSG00000259755	0.0009392	0.0014729	0.0000000	0.0000000	0.0214176
CHSY1	0.0680701	0.0703281	0.0659167	0.1046851	0.1685526
SELENOS	0.7146563	0.5254412	0.5091035	0.8676756	0.5223003
SNRPA1	0.3779628	0.4089631	0.4244922	0.3515781	0.2770186
PCSK6	0.0118524	0.0203468	0.0059604	0.0156273	0.0649316
SNRPA1-DT	0.0009500	0.0013757	0.0078474	0.0107263	0.0048799
LINC02348	0.0010550	0.0000000	0.0000000	0.0000000	0.0000000
TM2D3	0.3450022	0.4192750	0.4929747	0.3568420	0.3577130
ENSG00000289028	0.0003239	0.0080331	0.0058109	0.0054136	0.0000000
TARS3	0.4116732	0.3634068	0.3532662	0.3371820	0.4420549
UBE2Q2P13	0.0083100	0.0139952	0.0040624	0.0080967	0.0178680
ENSG00000291225	0.0015106	0.0027318	0.0055249	0.0000000	0.0081156
ENSG00000259553	0.0015233	0.0024233	0.0047351	0.0049892	0.0014245
ENSG00000290823	0.1618329	0.1617189	0.1478869	0.1400197	0.2208389
WASIR2	0.0075608	0.0147302	0.0163118	0.0177660	0.0071984
POLR3K	0.2116018	0.2289791	0.2694244	0.1899505	0.1867620
SNRNP25	0.3882767	0.4716497	0.3905366	0.4638718	0.3622533
RHBDF1	0.0444617	0.0174515	0.0253290	0.0608178	0.0081469
MPG	0.3691848	0.3716687	0.4049380	0.3951763	0.2874044
NPRL3	0.2143579	0.2197288	0.1676458	0.2064044	0.1520523
ENSG00000269482	0.0000000	0.0000000	0.0000000	0.0000000	0.0000000
ENSG00000228779	0.0010600	0.0000000	0.0000000	0.0000000	0.0000000
HBZ	0.0000000	0.0034274	0.0000000	0.0000000	0.0000000
HBQ1	0.0583303	0.1352470	0.1680347	0.0596919	0.0599157
ENSG00000268836	0.0024824	0.0000000	0.0000000	0.0000000	0.0000000
LUC7L	0.4083526	0.4744973	0.5286638	0.4020362	0.6293436
FAM234A	0.4129989	0.2975081	0.2194068	0.5033288	0.3012004
RGS11	0.0630893	0.0629713	0.0678774	0.0514511	0.1428053
ARHGDIG	0.4313658	0.6802434	0.7611938	0.4750707	0.5146613
PDIA2	0.0219027	0.0279036	0.0263474	0.0224666	0.1017260
AXIN1	0.1202749	0.1085928	0.0880481	0.1499204	0.1245097
MRPL28	0.3897525	0.4018835	0.5000306	0.4423627	0.3682888
PGAP6	0.2561538	0.2195547	0.1939266	0.2075882	0.1492310
NME4	0.6991869	0.5397658	0.3971237	0.7787991	0.4379127
DECR2	0.0247940	0.0214993	0.0146024	0.0272922	0.0419165
RAB11FIP3	0.1432411	0.1801778	0.1590425	0.1162763	0.5162229
LINC00235	0.0005933	0.0040380	0.0011903	0.0017907	0.0000000
CAPN15	0.0252689	0.0226197	0.0124675	0.0197308	0.1217945
PIGQ	0.1407382	0.0895232	0.1068158	0.1491237	0.1658480

NHLRC4	0.0367279	0.0197331	0.0075187	0.0538807	0.0168989
RAB40C	0.0913030	0.1145399	0.1178680	0.0574991	0.2088395
WFIKKN1	0.0021271	0.0021679	0.0000000	0.0000000	0.0022244
METTL26	0.6866210	0.7459030	0.6972853	0.6168099	0.5730629
MCRIP2	0.4622094	0.4952640	0.5937005	0.4725012	0.3946754
ENSG00000228201	0.0328863	0.0293303	0.0083698	0.0196819	0.0065973
WDR90	0.0199518	0.0169150	0.0078856	0.0029435	0.0315625
ENSG00000262528	0.0000000	0.0000000	0.0000000	0.0000000	0.0000000
RHOT2	0.0942364	0.0715396	0.0618032	0.0725013	0.0676111
RHBDL1	0.0731062	0.0825659	0.0985390	0.1006308	0.0582836
STUB1-DT	0.0134710	0.0084145	0.0124769	0.0230621	0.0044881
STUB1	0.7324671	0.7012303	0.7321112	0.7174264	0.5280230
JMJD8	0.1688525	0.1349363	0.1083130	0.2103798	0.0769818
WDR24	0.0399551	0.0289904	0.0266579	0.0328972	0.0384876
ENSG00000261659	0.0010179	0.0057148	0.0023101	0.0000000	0.0022372
FBXL16	0.1089369	0.1892051	0.2172443	0.0751193	0.1954174
METRN	2.1445563	1.8016267	1.4211416	2.1371966	1.6901635
ANTKMT	0.3256096	0.2891742	0.2952974	0.3899607	0.2313554
CCDC78	0.0212294	0.0073491	0.0097353	0.0063548	0.0024177
HAGHL	0.1815574	0.1821961	0.2451541	0.2186657	0.0968847
CIAO3	0.0824148	0.0707636	0.0665169	0.0592491	0.1049609
MSLNL	0.0000000	0.0011589	0.0000000	0.0000000	0.0000000
RPUSD1	0.0541968	0.0612467	0.0605337	0.0427462	0.0460405
CHTF18	0.0112667	0.0058437	0.0105173	0.0119916	0.0140197
GNG13	0.0102879	0.0234718	0.0544298	0.0067330	0.0190849
ENSG00000287855	0.0000000	0.0044649	0.0000000	0.0000000	0.0196006
LMF1	0.1026449	0.0781164	0.0734037	0.1462770	0.1642935
ENSG00000260316	0.0000000	0.0008290	0.0000000	0.0000000	0.0000000
LMF1-AS1	0.0000000	0.0000000	0.0000000	0.0000000	0.0000000
ENSG00000276931	0.0000000	0.0000000	0.0000000	0.0000000	0.0037236
CEROX1	0.0304988	0.0875373	0.1236954	0.0343786	0.0743929
SOX8	0.0073139	0.0095711	0.0052860	0.0108814	0.0206126
SSTR5	0.0010076	0.0011135	0.0009456	0.0000000	0.0000000
C1QTNF8	0.0018911	0.0014650	0.0000000	0.0000000	0.0000000
ENSG00000260532	0.0011388	0.0014243	0.0021423	0.0000000	0.0096271
ENSG00000273551	0.0003948	0.0000000	0.0000000	0.0018691	0.0071871
CACNA1H	0.0898564	0.0852973	0.0682510	0.0720478	0.2107710
ENSG00000277010	0.0043586	0.0040390	0.0000000	0.0035938	0.0000000
ENSG00000260710	0.0000000	0.0000000	0.0000000	0.0000000	0.0000000
ENSG00000274751	0.0000000	0.0005680	0.0000000	0.0000000	0.0049552
UBE2I	0.8468155	0.8273424	0.9426726	0.8217707	0.7500085
ENSG00000261505	0.0000000	0.0019941	0.0000000	0.0000000	0.0042562
BAIAP3	0.2595824	0.1530963	0.1561656	0.2668677	0.1784495
TSR3	0.5599117	0.5724099	0.6420441	0.5600427	0.4505226
GNPTG	0.3462613	0.2756163	0.2200637	0.5064966	0.2077990
ENSG00000260425	0.0000000	0.0000000	0.0000000	0.0000000	0.0000000
UNKL	0.0878881	0.0860511	0.0662522	0.1106672	0.1461372
ENSG00000260132	0.0071729	0.0077197	0.0250046	0.0019429	0.0131603
UQCC4	0.1414482	0.1407259	0.1771757	0.1372573	0.1293550
CCDC154	0.0044432	0.0069443	0.0000000	0.0034364	0.0182591
CLCN7	0.0917906	0.0767683	0.0511879	0.0931172	0.1013113
ENSG00000261641	0.0006791	0.0014195	0.0000000	0.0031666	0.0000000
TELO2	0.0547530	0.0719944	0.0494124	0.0560675	0.0506851

IFT140	0.1605341	0.1262222	0.1179174	0.1611182	0.2090818
ENSG00000260646	0.0000000	0.0000000	0.0000000	0.0000000	0.0016028
TMEM204	0.0004550	0.0010199	0.0000000	0.0000000	0.0000000
ENSG00000260989	0.0006593	0.0006253	0.0000000	0.0000000	0.0000000
CRAMP1	0.0791388	0.0785058	0.0891061	0.0413335	0.1887791
JPT2	0.4567543	0.3587314	0.3548362	0.4483719	0.2934665
MAPK8IP3	0.1467468	0.1861760	0.2196879	0.1306653	0.3738174
ENSG00000275092	0.0029776	0.0000000	0.0000000	0.0000000	0.0000000
ENSG00000261207	0.0000000	0.0000000	0.0000000	0.0000000	0.0000000
NME3	0.7503415	0.5627202	0.5163901	0.8666613	0.3759539
MRPS34	0.7186455	0.6461258	0.6688147	0.6883447	0.4791512
EME2	0.0164911	0.0153588	0.0111938	0.0139007	0.0138964
SPSB3	0.2354958	0.2002286	0.2248504	0.2030179	0.1667610
NUBP2	0.2887639	0.2827372	0.2579265	0.2716030	0.2457513
IGFALS	0.0032859	0.0029771	0.0000000	0.0000000	0.0032941
HAGH	0.4628578	0.5087729	0.6687395	0.4651603	0.4855797
FAHD1	0.2858288	0.3062895	0.2719982	0.2537216	0.1790796
HS3ST6	0.0011727	0.0000000	0.0011103	0.0030518	0.0026634
MSRB1	0.0670429	0.0406287	0.0607395	0.0969356	0.0347545
RPL3L	0.0000000	0.0000000	0.0000000	0.0000000	0.0074757
NDUFB10	1.0955258	1.0697009	1.1020695	0.9880783	0.8378392
RPS2	3.9629160	3.9332907	3.7830954	3.9071592	3.6542107
SNHG9	0.3173565	0.2540038	0.2029462	0.3042301	0.1757565
ENSG00000277602	0.0000000	0.0012022	0.0010537	0.0000000	0.0054224
TBL3	0.0880618	0.0726885	0.0865336	0.0911696	0.0769848
NOXO1	0.0020562	0.0022634	0.0093556	0.0000000	0.0034327
GFER	0.1690543	0.1622608	0.1653405	0.1651110	0.1089411
ENSG00000261790	0.0042826	0.0141321	0.0202410	0.0045071	0.0109053
SYNGR3	0.2583087	0.4887333	0.6127125	0.2591107	0.3739306
ZNF598	0.0966488	0.0776368	0.0885269	0.0916860	0.0557957
NPW	0.0207392	0.0112879	0.0150180	0.0239622	0.0312934
NHERF2	0.3066190	0.2738147	0.3164588	0.2841260	0.1927323
NTHL1	0.2244551	0.2352785	0.2977787	0.2860215	0.1381358
TSC2	0.1720294	0.1603165	0.1273303	0.1671414	0.2701053
PKD1	0.1181703	0.1086339	0.0614180	0.0964688	0.3825845
PKD1-AS1	0.0008902	0.0000000	0.0000000	0.0000000	0.0000000
ENSG00000261123	0.0000000	0.0000000	0.0000000	0.0000000	0.0000000
RAB26	0.0199367	0.0293648	0.0449352	0.0337967	0.0364924
SNHG19	0.1914816	0.1710871	0.1549281	0.1776668	0.1484130
TRAF7	0.2584332	0.2831663	0.2044922	0.2749818	0.1974158
CASKIN1	0.1405339	0.1892059	0.2342456	0.1123807	0.2633489
MLST8	0.3456478	0.3086184	0.2562909	0.3339852	0.2569576
BRICD5	0.0043147	0.0051564	0.0046637	0.0018489	0.0118170
PGP	0.2173067	0.2843145	0.3306125	0.2459458	0.2712366
E4F1	0.0554824	0.0481249	0.0539969	0.0627963	0.0623269
DNASE1L2	0.0177459	0.0010081	0.0000000	0.0301089	0.0000000
ECI1	0.6156138	0.5272247	0.4980827	0.5921550	0.3711665
ECI1-AS1	0.0000000	0.0000000	0.0000000	0.0000000	0.0115019
RNPS1	0.8676549	1.0119018	1.1488211	0.8605885	0.7922941
MIR3677HG	0.0072800	0.0013102	0.0000000	0.0092053	0.0000000
ABCA3	0.2042376	0.2470311	0.2407019	0.1798909	0.2984414
CCNF	0.0383803	0.0429246	0.0600794	0.0288312	0.0474953
ENSG00000260095	0.0039227	0.0009835	0.0020917	0.0048831	0.0000000

TEDC2	0.0200381	0.0294657	0.0382486	0.0176305	0.0204643
TEDC2-AS1	0.0015291	0.0000000	0.0000000	0.0000000	0.0030611
TBC1D24	0.1315975	0.1807837	0.2111690	0.1123022	0.2129478
ENSG00000260293	0.0332854	0.0306277	0.0242103	0.0154868	0.0488697
ATP6V0C	1.3555600	1.5597609	1.8693976	1.4091225	1.2973579
AMDHD2	0.0449423	0.0344711	0.0162894	0.0467936	0.0165015
CEMP1	0.0003481	0.0000000	0.0000000	0.0000000	0.0000000
PDPK1	0.2420419	0.3291637	0.3710618	0.2141551	0.2754496
ENSG00000261613	0.0168657	0.0140752	0.0153824	0.0092176	0.0223596
ENSG00000269937	0.0095260	0.0141304	0.0204495	0.0158929	0.0320921
ENSG00000261140	0.0137431	0.0088462	0.0230242	0.0034812	0.0313991
ENSG00000261093	0.0000000	0.0007544	0.0025721	0.0034589	0.0022372
ENSG00000291228	0.1433076	0.1538831	0.2166800	0.1150192	0.2286933
ENSG00000290728	0.0047237	0.0042348	0.0043645	0.0000000	0.0013244
ERVK13-1	0.0463534	0.0560879	0.0574452	0.0254553	0.1029503
KCTD5	0.1951006	0.1523418	0.1479509	0.2152098	0.1307596
PRSS27	0.0154110	0.0313631	0.0448341	0.0122437	0.0279968
SRRM2-AS1	0.0159974	0.0194710	0.0106901	0.0189175	0.0214993
SRRM2	1.1502998	1.1176378	1.1357919	1.1969521	1.2290849
ELOB	1.8542113	1.8906933	1.8735571	1.7826810	1.6473664
ENSG00000276791	0.0150283	0.0160679	0.0146392	0.0072173	0.0248883
ZG16B	0.0026746	0.0030016	0.0000000	0.0024291	0.0000000
ENSG00000263280	0.0195343	0.0199459	0.0081726	0.0203595	0.0053618
FLYWCH2	0.4155725	0.4320356	0.5845423	0.3995524	0.3726359
ENSG00000289222	0.0005340	0.0043561	0.0033209	0.0000000	0.0125991
FLYWCH1	0.0705998	0.0588691	0.0571238	0.0399140	0.0488654
ENSG00000262482	0.0000000	0.0008976	0.0000000	0.0000000	0.0070085
KREMEN2	0.0083285	0.0203278	0.0135929	0.0048478	0.0050250
PKMYT1	0.0026549	0.0061814	0.0073241	0.0194847	0.0030865
PAQR4	0.0864483	0.1476550	0.1710599	0.0622963	0.1144970
ENSG00000274367	0.0000000	0.0018689	0.0039182	0.0000000	0.0000000
ENSG00000262362	0.0011775	0.0025472	0.0021291	0.0000000	0.0052853
ENSG00000289281	0.0014576	0.0017777	0.0009007	0.0000000	0.0000000
ENSG00000272079	0.0125855	0.0156242	0.0210548	0.0159103	0.0161661
CLDN9	0.0007006	0.0000000	0.0018647	0.0000000	0.0000000
CLDN6	0.0384312	0.0785179	0.0199347	0.0087125	0.0493922
TNFRSF12A	0.2098233	0.1319782	0.0890901	0.2791582	0.1422227
HCFC1R1	0.7200026	0.9196783	0.9350214	0.6829678	0.6162536
THOC6	0.0638508	0.0695539	0.0425646	0.0554184	0.0329770
BICDL2	0.0014509	0.0000000	0.0000000	0.0000000	0.0054053
MMP25-AS1	0.0424950	0.0425296	0.0576383	0.0268315	0.1040705
MMP25	0.0000000	0.0019683	0.0013516	0.0000000	0.0000000
IL32	0.0026700	0.0061349	0.0136269	0.0058963	0.0092544
ZSCAN10	0.0000000	0.0000000	0.0000000	0.0000000	0.0000000
ZNF213-AS1	0.0992470	0.0850294	0.0712892	0.0850037	0.0908065
ENSG00000263011	0.1095325	0.0833885	0.0553131	0.1005938	0.0681120
ZNF205	0.1144989	0.1479342	0.1289426	0.1184032	0.0831544
ZNF213	0.0364782	0.0410887	0.0475234	0.0499414	0.0686237
ENSG00000261889	0.0030387	0.0054034	0.0000000	0.0043718	0.0000000
OR1F1	0.0109504	0.0327923	0.0263751	0.0188455	0.0518371
ZNF200	0.0905715	0.0762840	0.0601722	0.0838906	0.0615773
LINC00921	0.0038883	0.0055370	0.0000000	0.0059500	0.0000000
ENSG00000290183	0.0009223	0.0011339	0.0000000	0.0000000	0.0015072

ZNF263	0.0507924	0.0629945	0.0776153	0.0201787	0.0459436
TIGD7	0.1524888	0.1660179	0.1622173	0.1308317	0.0951042
ZNF75A	0.1398829	0.1454456	0.1240789	0.1478711	0.1952120
ZSCAN32	0.0814846	0.0538069	0.0397955	0.0642395	0.0655947
ZNF174	0.1349833	0.1087298	0.1065751	0.0913539	0.0440070
ZNF597	0.0314072	0.0336539	0.0382878	0.0201725	0.0125081
NAA60	0.2691484	0.2374259	0.2724736	0.2934170	0.2071119
CLUAP1	0.5828395	0.4110546	0.3901380	0.5043205	0.3978538
NLRC3	0.0046324	0.0051028	0.0029128	0.0063543	0.0000000
ENSG00000262312	0.0000000	0.0047537	0.0000000	0.0000000	0.0044670
SLX4	0.0490277	0.0897331	0.1058207	0.0247769	0.1730681
DNASE1	0.1120271	0.1310489	0.1146385	0.1085106	0.2142699
ENSG00000263235	0.0000000	0.0000000	0.0000000	0.0000000	0.0000000
TRAP1	0.4123866	0.4175522	0.5045445	0.3632536	0.4251944
CREBBP	0.3095838	0.3307693	0.3961100	0.2670655	0.6182540
LINC02861	0.0414209	0.0174545	0.0102169	0.0170771	0.0086605
ADCY9	0.0834183	0.0904098	0.0629217	0.0541202	0.3730274
ENSG00000263105	0.0008666	0.0000000	0.0000000	0.0000000	0.0000000
SRL	0.0014380	0.0000000	0.0000000	0.0000000	0.0000000
LINC01569	0.0146779	0.0048028	0.0051544	0.0123018	0.0216659
TFAP4	0.0944921	0.0956706	0.0817966	0.0727924	0.0499202
ENSG00000288935	0.0078320	0.0123662	0.0138084	0.0095924	0.0092168
GLIS2	0.1405451	0.0808845	0.0766361	0.1612502	0.1419774
GLIS2-AS1	0.0715007	0.0312148	0.0401142	0.0618911	0.0423988
PAM16	0.3967890	0.5152224	0.5600220	0.3963483	0.4171319
ENSG00000262712	0.0007502	0.0019491	0.0018766	0.0051034	0.0000000
CORO7	0.0450395	0.0904794	0.0731942	0.0674875	0.1071816
VASN	0.1686705	0.0950888	0.0516540	0.3557211	0.0818785
DNAJA3	0.2144059	0.1422333	0.1452699	0.1644389	0.1769053
ENSG00000277170	0.0013608	0.0000000	0.0009975	0.0026750	0.0000000
NMRAL1	0.1560053	0.1796526	0.1718999	0.1263138	0.1772965
HMOX2	0.2884302	0.3865635	0.4946052	0.2822034	0.2210813
CDIP1	0.2382961	0.2713456	0.3060833	0.2358758	0.2595746
C16orf96	0.0011479	0.0000000	0.0017682	0.0029721	0.0044485
ENSG00000261789	0.0000000	0.0000000	0.0000000	0.0000000	0.0000000
UBALD1	0.1036158	0.1157077	0.1348382	0.1151774	0.1147792
MGRN1	0.2012834	0.1831990	0.1823883	0.1829938	0.2393612
ENSG00000261442	0.0004098	0.0000000	0.0000000	0.0000000	0.0000000
NUDT16L1	0.4726018	0.3611242	0.3768222	0.5285032	0.2876935
ANKS3	0.1125987	0.0918266	0.0820038	0.1058437	0.2287471
DNAAF8	0.0419063	0.0380112	0.0136974	0.0634971	0.0191978
ZNF500	0.0308471	0.0314981	0.0391870	0.0469575	0.0716268
SEPTIN12	0.0018272	0.0052015	0.0007884	0.0075502	0.0031628
SMIM22	0.0028895	0.0000000	0.0000000	0.0000000	0.0140594
ROGDI	0.2209064	0.2889964	0.3415286	0.2061899	0.2010198
GLYR1	0.2937214	0.3245720	0.3398891	0.2707361	0.3542127
ENSG00000275056	0.0000000	0.0000000	0.0000000	0.0000000	0.0013481
UBN1	0.3440426	0.2560076	0.2057625	0.2395559	0.3041349
PPL	0.0261120	0.0285599	0.0392377	0.0330832	0.0456295
SEC14L5	0.0021624	0.0091110	0.0072234	0.0084175	0.0271885
NAGPA	0.0260145	0.0253828	0.0227651	0.0290736	0.0218963
ALG1	0.0416526	0.0276901	0.0244006	0.0539081	0.0283226
C16orf89	0.0637978	0.0392939	0.0060716	0.1538401	0.0407907

EEF2KMT	0.0696584	0.0545673	0.0848686	0.0582180	0.0621391
ENSG00000267070	0.0002343	0.0000000	0.0000000	0.0016359	0.0000000
ENSG00000285567	0.0047264	0.0043806	0.0038052	0.0000000	0.0320848
RBFOX1	1.0097710	1.4033945	1.2648835	0.8587647	2.5598585
LINC01570	0.0000000	0.0000000	0.0000000	0.0000000	0.0000000
ENSG00000257180	0.0030358	0.0000000	0.0000000	0.0000000	0.0022244
ENSG00000286635	0.0016112	0.0060884	0.0014307	0.0000000	0.0076527
ENSG00000289107	0.0014459	0.0022039	0.0000000	0.0000000	0.0121848
ENSG00000287340	0.0313198	0.0342148	0.0358437	0.0164806	0.0377686
ENSG00000260289	0.0000000	0.0000000	0.0000000	0.0000000	0.0054053
ENSG00000260003	0.0000000	0.0000000	0.0017289	0.0000000	0.0098500
ENSG00000260058	0.0000000	0.0000000	0.0000000	0.0000000	0.0043348
TMEM114	0.0006334	0.0034466	0.0046186	0.0000000	0.0000000
METTL22	0.0619717	0.0727716	0.0677629	0.0373476	0.0947745
ABAT	0.4114404	0.4694584	0.5468296	0.4736631	0.4453434
TMEM186	0.0887632	0.0807450	0.0854818	0.0839327	0.0296434
PMM2	0.1008121	0.0946428	0.0741222	0.0890868	0.2375848
ENSG00000260350	0.0000000	0.0000000	0.0000000	0.0000000	0.0073222
CARHSP1	0.4207434	0.4657706	0.5259459	0.3652097	0.3197178
CARHSP1-DT	0.0000000	0.0000000	0.0025355	0.0000000	0.0000000
USP7	0.3706670	0.3661715	0.3253737	0.3262566	0.4971598
USP7-AS1	0.0000000	0.0005011	0.0000000	0.0000000	0.0000000
HAPSTR1	0.2440193	0.2648828	0.2250060	0.2709561	0.3281271
ENSG00000260349	0.0173532	0.0214429	0.0344893	0.0124908	0.0222618
LINC02177	0.0020640	0.0013426	0.0000000	0.0085870	0.0150924
ENSG00000260071	0.0119393	0.0143365	0.0086617	0.0055930	0.0223265
ENSG00000283003	0.0007731	0.0071504	0.0070333	0.0000000	0.0094905
ENSG00000260362	0.0013110	0.0012251	0.0000000	0.0000000	0.0000000
GRIN2A	0.4719829	0.3951815	0.2940995	0.5350733	0.7497298
ENSG00000283221	0.0000000	0.0000000	0.0000000	0.0000000	0.0018081
ENSG00000261810	0.0000000	0.0006055	0.0000000	0.0000000	0.0074820
ATF7IP2	0.0670394	0.1010233	0.0622072	0.0767198	0.2070566
ENSG00000289023	0.0000000	0.0000000	0.0000000	0.0000000	0.0023834
EMP2	0.0406078	0.0271916	0.0116423	0.0632927	0.0186470
TEKT5	0.0008275	0.0000000	0.0023402	0.0000000	0.0052964
ENSG00000289073	0.0012810	0.0009752	0.0051361	0.0009588	0.0000000
NUBP1	0.1339975	0.1404270	0.1797817	0.1506056	0.1072100
TVP23A	0.1035272	0.1327100	0.1955500	0.0988114	0.2560270
ENSG00000262151	0.0004832	0.0000000	0.0000000	0.0000000	0.0118066
CIITA	0.0040199	0.0041574	0.0065809	0.0098892	0.0191640
DEXI	0.3083456	0.2902736	0.2732244	0.3313124	0.1690018
CLEC16A	0.1344195	0.1359919	0.1141822	0.1051089	0.3602792
ENSG00000274038	0.0012450	0.0000000	0.0000000	0.0000000	0.0000000
ENSG00000262020	0.0013417	0.0010439	0.0000000	0.0000000	0.0046716
ENSG00000287121	0.0000000	0.0000000	0.0000000	0.0000000	0.0000000
ENSG00000263033	0.0065029	0.0048298	0.0030148	0.0058334	0.0064427
RMI2	0.0433448	0.0476769	0.0219381	0.0602406	0.0183427
SOCS1	0.0830440	0.0527056	0.0355623	0.0381440	0.0320835
ENSG00000263080	0.0012124	0.0012303	0.0010898	0.0000000	0.0036741
ENSG00000188897	0.0183707	0.0036544	0.0000000	0.0171353	0.0265942
LITAF	0.4792832	0.2834631	0.1768782	0.6144583	0.2766749
SNN	0.4638618	0.4783422	0.5124231	0.5585759	0.3125639
TXNDC11	0.0844551	0.0860831	0.0676400	0.1032198	0.1701076

TXNDC11-AS1	0.0064287	0.0030187	0.0029373	0.0054345	0.0000000
ZC3H7A	0.4230685	0.3198517	0.3410504	0.4574230	0.4241055
ENSG00000277369	0.0168625	0.0110226	0.0099330	0.0036752	0.0187535
BCAR4	0.0000000	0.0019120	0.0000000	0.0000000	0.0000000
RSL1D1	1.1470983	1.0181373	1.0374382	1.0122576	0.8528349
RSL1D1-DT	0.0157128	0.0219266	0.0055084	0.0000000	0.0519380
GSPT1	0.9505634	0.8558860	0.8702684	0.9296528	0.8123859
ENSG00000261560	0.0010200	0.0000000	0.0035423	0.0000000	0.0000000
ENSG00000261216	0.0000000	0.0000000	0.0000000	0.0000000	0.0000000
NPIPB2	0.0760655	0.0535384	0.0286236	0.0559334	0.0614550
SNX29	0.1326379	0.1369728	0.1613642	0.1142450	0.3745828
ENSG00000259899	0.0000000	0.0000000	0.0000000	0.0000000	0.0030451
CPPED1	0.0956001	0.0912168	0.0687938	0.0750222	0.0994515
ENSG00000260378	0.0000000	0.0000000	0.0000000	0.0000000	0.0120534
SHISA9	0.0762909	0.0888647	0.1099383	0.1018823	0.2835763
ENSG00000262801	0.0168285	0.0250974	0.0144837	0.0227259	0.1957564
ENSG00000262267	0.0000000	0.0000000	0.0000000	0.0065257	0.0091795
ERCC4	0.3014465	0.2799600	0.2408768	0.2788369	0.1924230
ENSG00000262732	0.0000000	0.0000000	0.0000000	0.0011055	0.0057345
MRTFB	0.2742702	0.3424287	0.4569566	0.2595372	0.5882975
ENSG00000276564	0.0159524	0.0399798	0.0541651	0.0279736	0.0372283
ENSG00000262529	0.0000000	0.0000000	0.0000000	0.0000000	0.0000000
MIR193BHG	0.0400989	0.0733243	0.0515413	0.0467213	0.0844814
ENSG00000263257	0.0011912	0.0023494	0.0029908	0.0000000	0.0150094
PARN	0.2417065	0.2349725	0.2685176	0.2514462	0.3100423
BFAR	0.2809893	0.2385514	0.2351496	0.2520728	0.2489897
PLA2G10	0.0017470	0.0067818	0.0022682	0.0030630	0.0000000
NOMO1	0.0764911	0.0679171	0.1074770	0.0824821	0.0854596
NPIPA1	0.0198384	0.0233091	0.0199786	0.0175268	0.0775321
PDXDC1	0.4449713	0.4614883	0.4657614	0.4210419	0.4842260
ENSG00000275910	0.0014415	0.0000000	0.0000000	0.0000000	0.0000000
NTAN1	0.4316665	0.4697049	0.3618634	0.2937548	0.3471679
RRN3	0.1821569	0.2241913	0.2358285	0.1706625	0.1768576
ENSG00000290396	0.0447823	0.0575263	0.0354648	0.0323983	0.0912419
ENSG00000291272	0.0412380	0.0401474	0.0248377	0.0422308	0.1306642
ENSG00000290394	0.0033427	0.0006434	0.0000000	0.0019116	0.0075063
ENSG00000291273	0.0004558	0.0000000	0.0000000	0.0000000	0.0133286
ENSG00000257391	0.0000000	0.0008262	0.0000000	0.0000000	0.0000000
NPIPA5	0.0030263	0.0042297	0.0014179	0.0000000	0.0018039
MPV17L	0.1155537	0.1480357	0.1579672	0.0928291	0.1029019
BMERB1	0.6669246	0.9196129	1.1150257	0.6375381	0.9438204
MARF1	0.2480228	0.2241264	0.1979107	0.2356707	0.3221056
ENSG00000257769	0.0000000	0.0005197	0.0000000	0.0000000	0.0000000
NDE1	0.1898533	0.1145752	0.0706795	0.1923841	0.1123195
ENSG00000280206	0.0252290	0.0149703	0.0034134	0.0397432	0.0206300
MYH11	0.0142479	0.0090339	0.0012574	0.0011545	0.0110742
CEP20	0.3914087	0.2403835	0.2503690	0.3539743	0.1707449
ABCC1	0.0811135	0.0722983	0.0840849	0.1067307	0.3034407
ABCC6	0.0027106	0.0026653	0.0028783	0.0057490	0.0000000
NOMO3	0.0164605	0.0199377	0.0256078	0.0188191	0.0584287
NPIPA9	0.0016148	0.0037951	0.0017811	0.0060256	0.0351852
NPIPA7	0.0000000	0.0000000	0.0000000	0.0026077	0.0000000
ENSG00000260126	0.0015712	0.0033904	0.0049602	0.0000000	0.0041311

XYLT1	0.1019561	0.1276002	0.1475863	0.0775850	0.5428409
ENSG00000261448	0.0000000	0.0000000	0.0000000	0.0000000	0.0000000
ENSG00000259929	0.0000000	0.0000000	0.0000000	0.0000000	0.0000000
NPIPA8	0.0147208	0.0334836	0.0275600	0.0177926	0.0755150
NPIPA9.1	0.0000000	0.0011001	0.0000000	0.0000000	0.0000000
ENSG00000290383	0.0402245	0.0339789	0.0261873	0.0309531	0.0593446
NOMO2	0.0137650	0.0094300	0.0251532	0.0288853	0.0085422
ABCC6P1	0.0000000	0.0012869	0.0000000	0.0000000	0.0000000
RPS15A	2.9493754	2.8811952	2.7313064	2.8555506	2.6561609
ARL6IP1	1.4190354	1.4317831	1.6164584	1.6744709	1.2406565
ENSG00000260017	0.0028191	0.0000000	0.0000000	0.0000000	0.0152049
SMG1	0.2839522	0.3343533	0.2929407	0.2172148	0.5711477
SMG1-DT	0.0007945	0.0000000	0.0000000	0.0000000	0.0042903
TMC7	0.0351064	0.0246163	0.0393123	0.0234651	0.0730294
COQ7-DT	0.0124842	0.0057575	0.0000000	0.0085840	0.0088007
ENSG00000261357	0.0026014	0.0000000	0.0000000	0.0021808	0.0115910
COQ7	0.3113298	0.2838611	0.2480681	0.2791845	0.2235632
ENSG00000260430	0.0036862	0.0068410	0.0000000	0.0000000	0.0126664
ITPRIPL2	0.1316406	0.0747553	0.0869279	0.1052728	0.0830482
ENSG00000261759	0.0162439	0.0054486	0.0035109	0.0074595	0.0190168
SYT17	0.4857169	0.3599614	0.5062891	0.4378299	0.3988003
CLEC19A	0.0038206	0.0071630	0.0000000	0.0255791	0.0000000
ENSG00000259925	0.0000000	0.0027384	0.0000000	0.0000000	0.0000000
TMC5	0.2267919	0.0835850	0.0686155	0.2541515	0.0782183
ENSG00000260592	0.0000000	0.0010932	0.0027814	0.0000000	0.0152423
GDE1	0.3412006	0.2779438	0.3767252	0.4233492	0.2095668
CCP110	0.3715726	0.4058876	0.4936612	0.3642614	0.4180038
VPS35L	0.2430101	0.2074944	0.2200022	0.2381435	0.2927396
ENSG00000276571	0.0017543	0.0009041	0.0000000	0.0000000	0.0000000
KNOP1	0.4355224	0.4096222	0.4404481	0.4881872	0.3280740
ENSG00000261312	0.0017667	0.0008235	0.0019069	0.0108072	0.0044999
IQCK	0.5082043	0.3029836	0.2859243	0.5956910	0.4355044
ENSG00000261195	0.0007501	0.0011207	0.0000000	0.0000000	0.0034576
GPRC5B	0.1520317	0.1132616	0.0542862	0.2971412	0.1422859
GPR139	0.0364480	0.0623741	0.1488765	0.0122771	0.1524326
ACSM5	0.0068584	0.0088170	0.0000000	0.0077391	0.0000000
ACSM3	0.0049573	0.0068546	0.0000000	0.0000000	0.0060275
ACSM1	0.0213103	0.0086229	0.0157807	0.0379829	0.0235045
THUMPD1	0.7497617	0.5870271	0.4940283	0.7224363	0.6054436
ENSG00000260510	0.0018015	0.0033037	0.0000000	0.0000000	0.0000000
ERI2	0.1258057	0.1183706	0.0827839	0.1067704	0.0855276
REXO5	0.0786138	0.0455326	0.0415046	0.0648853	0.0707944
DCUN1D3	0.0409335	0.0599652	0.0488539	0.0510286	0.0588248
LYRM1	0.2845392	0.2996420	0.3118343	0.2511448	0.2224188
DNAH3	0.0098277	0.0027792	0.0009415	0.0000000	0.0141893
ENSG00000263331	0.0000000	0.0020545	0.0000000	0.0057454	0.0000000
LDAF1	0.2063682	0.3229189	0.3233550	0.1854233	0.2193954
CRYM	0.0107061	0.0094186	0.0241829	0.0082553	0.0180627
ENSG00000290192	0.0035991	0.0000000	0.0034775	0.0000000	0.0000000
NPIPB3	0.0076179	0.0057424	0.0012333	0.0010066	0.0073749
ENSG00000291073	0.0270279	0.0284094	0.0348755	0.0264768	0.1438331
ENSG00000287809	0.0003989	0.0012552	0.0000000	0.0000000	0.0044881
METTL9	1.1048390	1.2473977	1.2780286	1.1162075	0.9195907

ENSG00000261596	0.0011576	0.0034554	0.0030350	0.0026031	0.0074885
IGSF6	0.0000000	0.0000000	0.0021095	0.0000000	0.0119800
OTOA	0.0000000	0.0000000	0.0022934	0.0000000	0.0028666
ENSG00000260306	0.0009141	0.0035915	0.0009769	0.0083572	0.0000000
ENSG00000291066	0.1758693	0.2483890	0.2632727	0.1084423	0.1952218
NPIPB4	0.0114282	0.0146748	0.0155396	0.0090090	0.0710328
ENSG00000291262	0.0000000	0.0000000	0.0026308	0.0042360	0.0140461
ENSG00000290674	0.0006695	0.0000000	0.0000000	0.0000000	0.0076833
ENSG00000274460	0.0000000	0.0020422	0.0019069	0.0000000	0.0013712
UQCRC2	0.8828543	0.9905234	0.9568737	0.8695486	0.7525379
PDZD9	0.0018331	0.0013541	0.0133789	0.0000000	0.0098463
ENSG00000275445	0.0088981	0.0045310	0.0080233	0.0036960	0.0000000
MOSMO	0.3254153	0.4341233	0.6027914	0.2900471	0.4588973
ENSG00000260277	0.0000000	0.0008701	0.0000000	0.0035871	0.0000000
VWA3A	0.1209537	0.0789637	0.0331409	0.0930273	0.1413001
SDR42E2	0.0021497	0.0019099	0.0027245	0.0081326	0.0036979
ENSG00000287739	0.0000000	0.0014737	0.0000000	0.0000000	0.0000000
EEF2K	0.0974155	0.1148322	0.1012677	0.1011450	0.1707510
ENSG00000261113	0.0003980	0.0006296	0.0000000	0.0000000	0.0000000
POLR3E	0.1097722	0.0913197	0.1027902	0.0926651	0.0994393
ENSG00000286808	0.0000000	0.0017070	0.0032195	0.0000000	0.0132591
CDR2	0.1331492	0.1630045	0.2081040	0.1525202	0.2531945
CDR2-DT	0.0000000	0.0009429	0.0026175	0.0060309	0.0021172
RRN3P3	0.0232508	0.0174225	0.0099564	0.0022251	0.0376610
ENSG00000291060	0.0062520	0.0053851	0.0057454	0.0026183	0.0260720
NPIPB5	0.0048578	0.0048790	0.0076693	0.0040303	0.0225232
OTOAP1	0.0000000	0.0000000	0.0000000	0.0000000	0.0000000
ENSG00000283213	0.0060553	0.0123093	0.0319711	0.0060115	0.0269512
HS3ST2	0.0512035	0.1138644	0.2287530	0.0625398	0.3133784
ENSG00000261090	0.0006533	0.0017240	0.0029442	0.0047604	0.0107568
USP31	0.1030068	0.1331676	0.1452202	0.0975469	0.1748109
SCNN1G	0.0014532	0.0000000	0.0000000	0.0000000	0.0076346
COG7	0.1390113	0.1092227	0.0502523	0.1056423	0.1763687
ENSG00000259782	0.0019786	0.0000000	0.0000000	0.0000000	0.0000000
ENSG00000260136	0.0971964	0.0403573	0.0465801	0.1065375	0.0467414
GGA2	0.2210225	0.1621842	0.1446721	0.2235434	0.2873893
EARS2	0.0599390	0.0772224	0.0684256	0.0325125	0.0956809
UBFD1	0.5439570	0.6697818	0.7449242	0.4882605	0.4649801
ENSG00000260751	0.0003364	0.0000000	0.0000000	0.0000000	0.0024068
NDUFAB1	1.0230486	1.1232285	1.2014312	1.0463469	0.8537318
PALB2	0.0989867	0.0816866	0.0915096	0.1087847	0.0833099
ENSG00000261723	0.0000000	0.0000000	0.0000000	0.0000000	0.0000000
DCTN5	0.3114325	0.4269388	0.3894558	0.3195601	0.4281444
PLK1	0.0095728	0.0128994	0.0114452	0.0015624	0.0125420
ENSG00000261266	0.0012124	0.0000000	0.0019328	0.0000000	0.0000000
ERN2	0.0000000	0.0045396	0.0051512	0.0000000	0.0021637
PRKCB	0.7822480	0.7325769	0.7522286	0.7426600	0.8463772
LINC02194	0.0009277	0.0000000	0.0000000	0.0000000	0.0000000
CACNG3	0.0761797	0.1403797	0.1578778	0.0419314	0.1497773
RBBP6	0.4114056	0.4413102	0.3764861	0.4203069	0.5340179
ENSG00000289171	0.0038100	0.0108325	0.0145002	0.0000000	0.0095128
TNRC6A	0.6168452	0.6465842	0.5619268	0.5777026	1.2970094
LINC01567	0.0031785	0.0024226	0.0058625	0.0000000	0.0114016

ENSG00000261669	0.0010128	0.0015815	0.0000000	0.0000000	0.0150151
SLC5A11	0.0029347	0.0015971	0.0000000	0.0000000	0.0000000
ARHGAP17	0.1420614	0.1334006	0.1136247	0.1577856	0.2683764
ENSG00000291179	0.0114599	0.0199892	0.0109075	0.0122550	0.0166985
LINC02175	0.0499813	0.0823942	0.1036036	0.0358938	0.1001329
LCMT1-AS1	0.0249811	0.0296231	0.0394988	0.0361241	0.0561761
ENSG00000275494	0.0507335	0.0541857	0.0665705	0.0636191	0.0689010
LCMT1	0.4126267	0.4747293	0.4914521	0.4033186	0.4217305
LCMT1-AS2	0.0009398	0.0000000	0.0000000	0.0000000	0.0000000
AQP8	0.0000000	0.0011458	0.0006749	0.0000000	0.0000000
ZKSCAN2	0.0906777	0.1235144	0.1605582	0.1072197	0.1200149
ZKSCAN2-DT	0.0088761	0.0124248	0.0159644	0.0025754	0.0576186
LINC02191	0.0000000	0.0000000	0.0000000	0.0000000	0.0000000
HS3ST4	0.4027446	0.3146160	0.1874252	0.3710492	0.4824685
ENSG00000285882	0.0028864	0.0024864	0.0000000	0.0052476	0.0102463
ENSG00000231876	0.0046161	0.0000000	0.0024253	0.0072418	0.0000000
KDM8	0.0180891	0.0242963	0.0168191	0.0074877	0.0310457
ENSG00000259940	0.0000000	0.0008522	0.0000000	0.0000000	0.0016664
NSMCE1	0.4141462	0.3710913	0.3205189	0.4035281	0.3213185
NSMCE1-DT	0.0049588	0.0021301	0.0018021	0.0000000	0.0044999
IL4R	0.0047181	0.0016789	0.0000000	0.0000000	0.0060847
IL21R	0.0000000	0.0000000	0.0000000	0.0000000	0.0124920
IL21R-AS1	0.0000000	0.0000000	0.0000000	0.0000000	0.0000000
GTF3C1	0.2703751	0.2730065	0.3290290	0.2670532	0.3337953
KATNIP	0.0956249	0.0812322	0.0801517	0.0957386	0.2233349
ENSG00000261329	0.0000000	0.0000000	0.0000000	0.0000000	0.0122111
GSG1L	0.0490727	0.1086589	0.2000203	0.0426479	0.1850889
XPO6	0.2439411	0.3081185	0.2717333	0.1963229	0.3998407
ENSG00000283662	0.0000000	0.0007620	0.0000000	0.0000000	0.0000000
SBK1	0.3628585	0.6995304	0.8403735	0.3005564	0.5865645
ENSG00000246465	0.0010055	0.0108935	0.0106644	0.0000000	0.0048434
NPIPB6	0.0087814	0.0072453	0.0067064	0.0000000	0.0095729
EIF3CL	0.0013638	0.0019296	0.0108811	0.0000000	0.0034843
NPIPB7	0.0024476	0.0000000	0.0109852	0.0071062	0.0102767
CLN3	0.0985645	0.0849578	0.0832885	0.1010343	0.0308036
NUPR1	0.4849556	0.3030783	0.1760287	0.5311450	0.1756626
ENSG00000275441	0.0016824	0.0058256	0.0000000	0.0046951	0.0129180
SGF29	0.1051452	0.1042351	0.1011450	0.1196018	0.0801944
SULT1A2	0.0000000	0.0000000	0.0000000	0.0000000	0.0128759
SULT1A1	0.0938623	0.0582066	0.0367894	0.1002215	0.0172306
NPIPB8	0.0000000	0.0000000	0.0007808	0.0000000	0.0050279
EIF3C	0.1012285	0.0999644	0.1295860	0.1001808	0.1272880
NPIPB9	0.0013993	0.0014217	0.0000000	0.0000000	0.0033430
ENSG00000251417	0.0021789	0.0003960	0.0000000	0.0000000	0.0000000
ENSG00000260796	0.0000000	0.0005323	0.0013411	0.0000000	0.0000000
ENSG00000275807	0.0035383	0.0054460	0.0026764	0.0025011	0.0000000
ATXN2L	0.4592466	0.4059280	0.4248133	0.3680831	0.4858162
ENSG00000260570	0.0051431	0.0019099	0.0066684	0.0044097	0.0263386
TUFM	0.5668522	0.5795737	0.5470572	0.7155641	0.4043787
SH2B1	0.1540919	0.1547400	0.1609566	0.1561132	0.2275310
ENSG00000261766	0.0039333	0.0013468	0.0000000	0.0057878	0.0059067
ATP2A1-AS1	0.0434927	0.0495860	0.0360618	0.0257303	0.0366627
ATP2A1	0.0023844	0.0057877	0.0020207	0.0000000	0.0227140

RABEP2	0.0359596	0.0400085	0.0266588	0.0227318	0.0692147
NFATC2IP	0.0785330	0.1176637	0.1004397	0.0657266	0.1528878
NFATC2IP-AS1	0.0032075	0.0008794	0.0000000	0.0000000	0.0019839
ENSG00000260367	0.0021459	0.0027611	0.0000000	0.0057257	0.0000000
SPNS1	0.1283536	0.1195386	0.1314926	0.2287664	0.1013815
LAT	0.0000000	0.0000000	0.0011369	0.0000000	0.0220793
ENSG00000290677	0.0022530	0.0000000	0.0000000	0.0000000	0.0000000
ENSG00000291188	0.0036502	0.0030051	0.0009402	0.0000000	0.0000000
ENSG00000284685	0.0008572	0.0012974	0.0000000	0.0000000	0.0073672
ENSG00000290680	0.0009247	0.0000000	0.0000000	0.0045301	0.0030787
NPIPB11	0.0022246	0.0035127	0.0049642	0.0041218	0.0117996
NPIPB12	0.0003673	0.0000000	0.0000000	0.0033357	0.0071630
SPN	0.0012852	0.0000000	0.0000000	0.0000000	0.0000000
QPRT	0.5150515	0.4220380	0.2641680	0.6065144	0.2236046
KIF22	0.1264104	0.1140635	0.1142284	0.0845602	0.0813505
ENSG00000275857	0.0027391	0.0000000	0.0000000	0.0019281	0.0052561
MAZ	0.6415606	0.6778491	0.7631717	0.5699121	0.5600750
ENSG00000259952	0.0000000	0.0082386	0.0026682	0.0000000	0.0000000
MVP-DT	0.1758408	0.1755689	0.1944628	0.1497775	0.2034748
PRRT2	0.2032054	0.3426058	0.4441089	0.1771436	0.3527286
PAGR1	0.2713938	0.2264077	0.2412777	0.3008893	0.2566225
MVP	0.0707301	0.0344271	0.0374595	0.0734873	0.0477956
CDIPT	0.3215417	0.3495356	0.3683223	0.3780374	0.2921635
ENSG00000278713	0.0007786	0.0011906	0.0042581	0.0040080	0.0000000
SEZ6L2	0.4398795	0.8040144	1.1398537	0.3731197	0.6521543
ASPHD1	0.1419572	0.2529127	0.2709440	0.1066439	0.2723391
KCTD13	0.1778160	0.2707949	0.4085212	0.1789714	0.2635365
KCTD13-DT	0.0138877	0.0189695	0.0236259	0.0156858	0.0256910
TMEM219	0.6446942	0.6017362	0.5637657	0.6727835	0.4631344
TAOK2	0.0945541	0.1057555	0.1410339	0.0706713	0.0938465
HIRIP3	0.2108292	0.2193574	0.2328017	0.1778456	0.2347446
INO80E	0.1287797	0.1613875	0.1758748	0.1058567	0.0999528
DOC2A	0.0738220	0.1528988	0.1650747	0.0756998	0.0855376
C16orf92	0.0000000	0.0025721	0.0021670	0.0000000	0.0000000
TLCD3B	0.2577647	0.5526051	0.6650452	0.2267382	0.3332213
ENSG00000285043	0.0083428	0.0078852	0.0021457	0.0000000	0.0231766
ALDOA	0.2423966	0.2339550	0.1825762	0.2231569	0.1611158
ENSG00000274904	0.0013440	0.0030466	0.0043385	0.0019116	0.0000000
PPP4C	0.7448341	0.6897785	0.7227797	0.7603362	0.4849247
TBX6	0.0051416	0.0011185	0.0049176	0.0000000	0.0222589
YPEL3	0.3282844	0.4407987	0.4630458	0.3513846	0.2430754
YPEL3-DT	0.0355457	0.0462143	0.0640995	0.0284460	0.0409921
GDPD3	0.0034314	0.0055919	0.0023088	0.0000000	0.0031104
MAPK3	0.2361563	0.2677584	0.2592205	0.1897613	0.1907116
CORO1A	0.0702047	0.1364627	0.2209205	0.0756607	0.1213091
BOLA2B	0.0248812	0.0279573	0.0309165	0.0375538	0.0214764
SLX1A	0.0000000	0.0000000	0.0048687	0.0079023	0.0000000
SULT1A3	0.0032769	0.0049559	0.0046398	0.0095556	0.0121939
NPIPB13	0.0184110	0.0278866	0.0126882	0.0065134	0.1113368
ENSG00000291047	0.0194094	0.0111478	0.0184806	0.0081635	0.0807049
SMG1P5	0.1317290	0.2076899	0.2613547	0.1226778	0.1651615
CD2BP2	0.3150427	0.2564428	0.2362199	0.3454709	0.2321213
CD2BP2-DT	0.0080260	0.0127642	0.0053706	0.0133115	0.0046038

TBC1D10B	0.1034190	0.1234668	0.1231889	0.0981267	0.1178696
ENSG00000274653	0.0013763	0.0008943	0.0023402	0.0057962	0.0000000
MYL11	0.0067355	0.0031474	0.0080136	0.0133687	0.0060203
ZNF48	0.0710201	0.0954727	0.1476272	0.0636585	0.0806820
SEPTIN1	0.0577255	0.0621872	0.0706659	0.0483675	0.0672148
ZNF771	0.1633400	0.1821214	0.1886808	0.1939019	0.0775869
DCTPP1	0.2103025	0.2202757	0.2507473	0.1802511	0.1279259
SEPHS2	0.3615417	0.3686809	0.3749910	0.3967705	0.3343688
ITGAL	0.0000000	0.0010809	0.0013591	0.0000000	0.0000000
ZNF768	0.1709856	0.1646560	0.1601691	0.1478428	0.1632382
ZNF747	0.0662720	0.0262152	0.0536026	0.0464570	0.0329576
ZNF747-DT	0.0253906	0.0438198	0.0421249	0.0236357	0.0202854
ZNF764	0.0815532	0.0790582	0.0818286	0.0789571	0.0493502
ZNF688	0.2048275	0.1312392	0.1194946	0.1746457	0.1674953
ENSG00000239791	0.0040688	0.0081997	0.0022147	0.0048888	0.0024307
ZNF785	0.0960014	0.0859217	0.0943417	0.1253594	0.1088824
ENSG00000260167	0.0033470	0.0061048	0.0000000	0.0035998	0.0000000
ZNF689	0.0898257	0.0769398	0.1134934	0.0871973	0.0866798
ENSG00000288983	0.0921641	0.0858730	0.1113529	0.0477397	0.0752916
PRR14	0.1485710	0.1409084	0.1590761	0.1717000	0.1804670
FBRS	0.1644734	0.1494871	0.1583586	0.1614651	0.1793224
ENSG00000261840	0.0549198	0.0395139	0.0415263	0.0514308	0.0231304
SRCAP	0.1145505	0.1353117	0.1300242	0.1312094	0.1380805
TMEM265	0.0533023	0.0401943	0.0228066	0.0258107	0.0316416
PHKG2	0.1264940	0.1247685	0.1392046	0.1326081	0.0788001
CFAP119	0.0683144	0.0581700	0.0660035	0.0880495	0.0566411
RNF40	0.2170321	0.1812500	0.1874012	0.2181580	0.1979525
ZNF629	0.1163913	0.1298685	0.1624948	0.1306292	0.1188878
ENSG00000274678	0.0077442	0.0003543	0.0069795	0.0022431	0.0000000
BCL7C	0.4874788	0.4624082	0.5564171	0.4586444	0.4847271
MIR762HG	0.0571127	0.0623344	0.0578863	0.0391263	0.0698799
CTF1	0.1100247	0.0472839	0.0354935	0.1091418	0.0268936
FBXL19-AS1	0.0073988	0.0145613	0.0127493	0.0090589	0.0143955
FBXL19	0.1633474	0.2350466	0.2704773	0.1285017	0.2390224
ORAI3	0.2403483	0.1337875	0.0903626	0.2772011	0.1258534
ENSG00000275263	0.0016747	0.0023746	0.0013672	0.0000000	0.0000000
SETD1A	0.0476650	0.0504612	0.0409016	0.0251700	0.0762286
HSD3B7	0.0039384	0.0027727	0.0019963	0.0058052	0.0000000
STX1B	0.1652330	0.3511604	0.4894362	0.1209437	0.3947487
STX4	0.2248560	0.1578991	0.2100929	0.1975378	0.1554451
ENSG00000260911	0.0041017	0.0082182	0.0028904	0.0046019	0.0305231
ENSG00000232748	0.0006293	0.0045793	0.0000000	0.0000000	0.0033430
ZNF668	0.0252734	0.0455348	0.0298909	0.0473901	0.0519768
ENSG00000261124	0.0000000	0.0000000	0.0018589	0.0000000	0.0052853
ZNF646	0.0303245	0.0452116	0.0217731	0.0396206	0.0269310
PRSS53	0.0079839	0.0111119	0.0199015	0.0132281	0.0168313
VKORC1	0.6359508	0.5466034	0.4579616	0.6578692	0.4253428
BCKDK	0.2473221	0.2263958	0.2044715	0.2838021	0.1561571
KAT8	0.3359972	0.2534509	0.3243401	0.3348160	0.2822429
ENSG00000262766	0.0012032	0.0003960	0.0000000	0.0000000	0.0000000
ENSG00000278133	0.0240272	0.0207325	0.0184117	0.0272623	0.0338694
PRSS8	0.0010430	0.0000000	0.0000000	0.0067836	0.0000000
PRSS36	0.0099444	0.0154805	0.0102856	0.0022792	0.0052756

FUS	0.9717053	0.9473015	1.0412128	0.9702888	0.9826084
ENSG00000260060	0.0012311	0.0031931	0.0000000	0.0100822	0.0091645
PYCARD	0.0157036	0.0064894	0.0008688	0.0159929	0.0015531
PYCARD-AS1	0.0010114	0.0000000	0.0000000	0.0000000	0.0026010
TRIM72	0.0023024	0.0071282	0.0057555	0.0093530	0.0131954
PYDC1	0.0148583	0.0225649	0.0395597	0.0122206	0.0115291
ITGAM	0.0000000	0.0000000	0.0000000	0.0000000	0.0000000
ZNF843	0.0391882	0.0273970	0.0209327	0.0439591	0.0270805
ENSG00000261474	0.0337749	0.0503422	0.0312026	0.0225279	0.0386676
ENSG00000260267	0.0391342	0.0707132	0.0610158	0.0317390	0.0374680
ARMC5	0.0502608	0.0529925	0.0505126	0.0325814	0.0666904
TGFB1I1	0.0708882	0.0443455	0.0200754	0.0800581	0.0150568
SLC5A2	0.0070363	0.0114703	0.0204487	0.0089951	0.0309844
ENSG00000260740	0.0120566	0.0063743	0.0058084	0.0099924	0.0000000
RUSF1	0.1295495	0.1420602	0.1110230	0.1599660	0.1049252
RUSF1-DT	0.0761401	0.0596480	0.0382278	0.0541366	0.0251279
AHSP	0.0043476	0.0003989	0.0000000	0.0000000	0.0000000
ENSG00000290927	0.0175520	0.0250615	0.0316329	0.0223431	0.0498666
ENSG00000276867	0.0000000	0.0000000	0.0009691	0.0000000	0.0097234
KRBOX5	0.2518800	0.2980609	0.4114401	0.2360733	0.3274817
ENSG00000259810	0.0000000	0.0000000	0.0000000	0.0000000	0.0000000
ZNF267	0.0812889	0.0687105	0.0623670	0.0795768	0.0667051
ENSG00000291271	0.0014564	0.0074776	0.0010529	0.0000000	0.0026227
ENSG00000261200	0.0000000	0.0000000	0.0000000	0.0000000	0.0072113
ENSG00000291264	0.0156300	0.0187567	0.0195743	0.0215902	0.0048241
ENSG00000290429	0.0000000	0.0000000	0.0000000	0.0000000	0.0000000
SHCBP1	0.0060940	0.0045526	0.0000000	0.0044812	0.0044624
VPS35	1.0345130	0.9466318	0.9906715	1.0362327	0.8084713
ORC6	0.0842675	0.0540440	0.0553390	0.0762767	0.0510865
MYLK3	0.1054864	0.0609265	0.0261995	0.1051821	0.0248937
C16orf87	0.6302308	0.5740880	0.7043297	0.6044785	0.4601773
GPT2	0.2675033	0.2184054	0.2111390	0.2530882	0.2915820
DNAJA2	0.9086619	0.8941587	1.0141641	0.9696498	0.8235365
DNAJA2-DT	0.0041071	0.0046081	0.0017682	0.0059077	0.0094744
ENSG00000259821	0.0012611	0.0021801	0.0025465	0.0000000	0.0034000
NETO2	0.2112039	0.3300959	0.4060185	0.1937257	0.3257115
ITFG1-AS1	0.0019288	0.0003752	0.0022064	0.0000000	0.0000000
ITFG1	0.6755778	0.6698910	0.7523724	0.7796275	0.7994474
ENSG00000260744	0.0000000	0.0016978	0.0000000	0.0000000	0.0111932
ENSG00000261369	0.0000000	0.0000000	0.0000000	0.0000000	0.0261515
PHKB	0.4648112	0.3801985	0.3320674	0.5547591	0.5897289
ENSG00000262038	0.0000000	0.0000000	0.0012170	0.0000000	0.0000000
LINC02133	0.0036636	0.0049745	0.0111289	0.0000000	0.0190447
LINC02192	0.0000000	0.0000000	0.0000000	0.0000000	0.0000000
LINC02134	0.0005889	0.0011542	0.0040088	0.0040556	0.0041652
ABCC11	0.0021195	0.0022275	0.0066496	0.0013512	0.0134621
LONP2	0.5866956	0.5595186	0.4720789	0.5534213	0.5956631
SIAH1	0.3762087	0.4022833	0.4432973	0.3620643	0.3276574
N4BP1	0.3859430	0.3508558	0.3746895	0.2947329	0.4166110
ENSG00000261267	0.0005773	0.0005843	0.0000000	0.0000000	0.0044508
ENSG00000289119	0.0054694	0.0075367	0.0120402	0.0033357	0.0058920
ENSG00000260086	0.0085163	0.0034241	0.0000000	0.0145600	0.0064709
ENSG00000287469	0.0068016	0.0000000	0.0000000	0.0065998	0.0041471

CBLN1	0.0252684	0.0783866	0.0869452	0.0218431	0.1114668
ENSG00000279249	0.0207479	0.0285111	0.0367813	0.0236686	0.0504014
ENSG00000262950	0.0012634	0.0000000	0.0000000	0.0000000	0.0080682
ZNF423	0.1092580	0.1043402	0.1285785	0.0895190	0.2906148
ENSG00000275155	0.0048422	0.0042705	0.0021751	0.0021653	0.0023834
CNEP1R1	0.1041728	0.1151143	0.1190401	0.1532141	0.1321265
HEATR3-AS1	0.0031408	0.0039016	0.0015765	0.0072059	0.0054176
HEATR3	0.0726587	0.1078320	0.1167189	0.0576234	0.1063084
ENSG00000260381	0.0000000	0.0000000	0.0028783	0.0000000	0.0000000
ENSG00000287444	0.0010865	0.0012917	0.0000000	0.0017461	0.0050487
TENT4B	0.1608869	0.1823230	0.1889119	0.1716683	0.2163991
ADCY7	0.0360754	0.0391322	0.0773629	0.0513629	0.0799476
BRD7	0.7408050	0.7048145	0.7048438	0.7156248	0.5585649
ENSG00000260573	0.0008414	0.0045865	0.0000000	0.0000000	0.0000000
NKD1	0.1687940	0.1194889	0.0785485	0.1420995	0.1054677
ENSG00000260249	0.0025479	0.0038514	0.0024957	0.0033686	0.0000000
NOD2	0.0015867	0.0011149	0.0000000	0.0000000	0.0000000
CYLD-AS1	0.0000000	0.0000000	0.0000000	0.0000000	0.0039740
CYLD	0.3768391	0.4586487	0.6005325	0.3231360	0.4376549
ENSG00000260616	0.0000000	0.0014206	0.0000000	0.0000000	0.0023834
LINC02128	0.0015410	0.0006684	0.0036470	0.0000000	0.0042817
SALL1	0.3045420	0.2531476	0.1572237	0.3716279	0.2013663
ENSG00000285367	0.0672241	0.0661844	0.0434955	0.0712062	0.1518324
ENSG00000287256	0.0013122	0.0007534	0.0028126	0.0000000	0.0039501
ENSG00000260850	0.0183870	0.0006864	0.0044061	0.0416729	0.0172004
HNRNPA1L3	0.1661984	0.1873624	0.1753814	0.1298674	0.1820455
LINC01571	0.0116224	0.0110713	0.0112845	0.0108337	0.0056180
ENSG00000260975	0.0000000	0.0000000	0.0000000	0.0000000	0.0000000
TOX3	0.3683755	0.4275445	0.5232683	0.2610203	0.4697280
ENSG00000261261	0.0012595	0.0000000	0.0051692	0.0000000	0.0000000
CHD9NB	0.0039447	0.0009937	0.0000000	0.0030518	0.0000000
CHD9	0.7122946	0.8400122	0.8812310	0.6409850	1.1924387
RBL2	0.3050919	0.2626133	0.1850535	0.3261286	0.3290690
ENSG00000259926	0.0000000	0.0000000	0.0000000	0.0000000	0.0000000
AKTIP	0.4398310	0.3578017	0.3204147	0.4252705	0.3610764
RPGRIP1L	0.2897438	0.2790573	0.2905085	0.3011000	0.3173250
ENSG00000275191	0.0000000	0.0000000	0.0000000	0.0000000	0.0000000
FTO	0.4517305	0.5288347	0.5932536	0.4244876	0.7851102
ENSG00000261630	0.0000000	0.0000000	0.0000000	0.0000000	0.0000000
ENSG00000261049	0.0009837	0.0000000	0.0000000	0.0024587	0.0068619
IRX3	0.0542853	0.1396945	0.0616557	0.0214087	0.0291200
CRNDE	0.3625580	0.3946686	0.2678583	0.2963607	0.3853341
IRX5	0.0285821	0.0732222	0.0287980	0.0385317	0.0553430
ENSG00000259711	0.0078685	0.0077590	0.0021117	0.0000000	0.0024068
IRX6	0.0025182	0.0008456	0.0000000	0.0034589	0.0000000
MMP2	0.0842361	0.0679284	0.0532343	0.1284756	0.0819334
LPCAT2	0.0742643	0.0806527	0.0764082	0.0755719	0.1021388
ENSG00000261997	0.0023541	0.0000000	0.0000000	0.0000000	0.0022372
CES5A	0.0007188	0.0055786	0.0000000	0.0000000	0.0000000
GNAO1-DT	0.0483126	0.0604317	0.0709070	0.0249183	0.1545506
ENSG00000260041	0.0000000	0.0025683	0.0029216	0.0000000	0.0050636
GNAO1	0.7135085	1.1878760	1.5142510	0.6474548	1.1230065
GNAO1-AS1	0.0029672	0.0080727	0.0059962	0.0000000	0.0025761

ENSG00000287866	0.0000000	0.0016164	0.0000000	0.0000000	0.0095540
ENSG00000260198	0.0000000	0.0000000	0.0000000	0.0000000	0.0000000
AMFR	0.4673117	0.4101066	0.3440500	0.5340842	0.4188914
ENSG00000260621	0.0011492	0.0000000	0.0000000	0.0000000	0.0000000
NUDT21	0.5224362	0.6866761	0.8433378	0.5252366	0.5716492
OGFOD1	0.2538491	0.2382571	0.2486627	0.2619821	0.1772698
BBS2	0.2590013	0.1946200	0.1682544	0.3162616	0.2321512
MT3	0.3987199	0.5769113	0.5777173	0.5093930	0.4000809
MT2A	0.1824986	0.1151101	0.0455959	0.2005325	0.2039644
MT1E	0.0026053	0.0033322	0.0000000	0.0038847	0.0000000
MT1F	0.0102843	0.0057205	0.0105159	0.0348837	0.0042436
MT1X	0.1039471	0.0907211	0.0255033	0.1028971	0.0296117
NUP93-DT	0.0016320	0.0008184	0.0018940	0.0016235	0.0000000
NUP93	0.2677764	0.3980900	0.4320236	0.2217404	0.3421118
SLC12A3	0.0012886	0.0035784	0.0000000	0.0000000	0.0113996
HERPUD1	1.0527403	0.8252424	0.7507683	1.0277741	0.7295116
ENSG00000261114	0.0030881	0.0071899	0.0012936	0.0024968	0.0000000
NLRC5	0.0012342	0.0027925	0.0000000	0.0000000	0.0046818
CPNE2	0.2454314	0.2359481	0.2378712	0.3585137	0.1572290
PSME3IP1	0.7726777	0.8692107	0.9408314	0.7416684	0.6708867
ENSG00000285979	0.0014137	0.0074437	0.0102178	0.0087525	0.0000000
RSPRY1	0.5030021	0.3965483	0.3848666	0.5161697	0.4053382
ARL2BP	0.7768325	0.6843238	0.6840130	0.8375222	0.5241263
ENSG00000260038	0.0015019	0.0000000	0.0000000	0.0000000	0.0000000
PLLP	0.0128052	0.0130734	0.0088030	0.0271757	0.0168730
CX3CL1	0.0136702	0.0177137	0.0432738	0.0293779	0.0098712
CIAPIN1	0.2929218	0.3236569	0.3631669	0.2663314	0.2340715
COQ9	0.2920876	0.2421927	0.1820318	0.3307437	0.1568952
POLR2C	0.3664819	0.2922906	0.2871784	0.3948871	0.1919960
DOK4	0.1019787	0.1864350	0.1879532	0.0816928	0.1603666
CCDC102A	0.0522183	0.0183306	0.0293798	0.0532415	0.0028917
ADGRG5	0.0018450	0.0039249	0.0000000	0.0000000	0.0114360
ADGRG1	0.1567453	0.1532576	0.2468633	0.2649616	0.2137703
ADGRG3	0.0006334	0.0034710	0.0009975	0.0000000	0.0191402
DRC7	0.0386409	0.0286669	0.0126583	0.0485686	0.0272851
ENSG00000288753	0.0030351	0.0017465	0.0000000	0.0149005	0.0038927
KATNB1	0.1670952	0.1361064	0.1502705	0.2006001	0.0933456
KIFC3	0.1029491	0.1090389	0.1409329	0.1219556	0.1239856
ENSG00000276166	0.0040145	0.0029649	0.0016016	0.0041993	0.0024001
ENSG00000187185	0.0000000	0.0000000	0.0000000	0.0000000	0.0000000
CNGB1	0.0203117	0.0542527	0.0805130	0.0173725	0.1347444
TEPP	0.0061361	0.0139126	0.0092502	0.0211762	0.0000000
ZNF319	0.0224057	0.0618716	0.0556097	0.0169994	0.0212033
USB1	0.2256085	0.3056309	0.3352208	0.2269661	0.2404285
MMP15	0.0628049	0.0748969	0.0815436	0.0655216	0.0632203
CFAP20	0.4692243	0.3713126	0.4065002	0.4435611	0.2880670
ENSG00000260545	0.0000000	0.0000000	0.0000000	0.0000000	0.0000000
ENSG00000260927	0.1253999	0.1043639	0.0494096	0.1158428	0.0890368
CSNK2A2	0.2468525	0.2455004	0.1938750	0.2279788	0.2690842
ENSG00000260867	0.0000000	0.0000000	0.0000000	0.0000000	0.0000000
CCDC113	0.6398258	0.4452927	0.4198350	0.6206215	0.3604024
PRSS54	0.0000000	0.0013112	0.0026996	0.0000000	0.0036741
GINS3	0.0203603	0.0219868	0.0171314	0.0172656	0.0381380

ENSG00000276131	0.0116695	0.0060237	0.0097181	0.0076607	0.0091816
LINC02137	0.0000000	0.0024453	0.0000000	0.0000000	0.0000000
NDRG4	0.3935858	0.5581062	0.7430251	0.3835043	0.5653148
ENSG00000289004	0.0040084	0.0038407	0.0000000	0.0010066	0.0000000
SETD6	0.0899110	0.0979711	0.0984093	0.0869684	0.1282556
CNOT1	0.3638792	0.3177166	0.2963347	0.2898376	0.3768184
SLC38A7	0.0313613	0.0560392	0.0278655	0.0589312	0.0490213
GOT2	0.4052739	0.4995992	0.5651642	0.4005693	0.3619367
ENSG00000245768	0.0093661	0.0009808	0.0054205	0.0026278	0.0098739
ENSG00000286653	0.0000000	0.0021560	0.0025063	0.0000000	0.0069990
CDH8	0.1426457	0.2725259	0.4293845	0.1704592	0.5729275
ENSG00000260600	0.0016099	0.0000000	0.0000000	0.0000000	0.0065249
CDH8-AS1	0.0022327	0.0012525	0.0018309	0.0000000	0.0000000
ENSG00000231589	0.0029097	0.0000000	0.0022363	0.0000000	0.0000000
ENSG00000261502	0.0049571	0.0129052	0.0047294	0.0042538	0.0118669
ENSG00000260658	0.0129381	0.0091733	0.0083292	0.0143188	0.0662155
ENSG00000261028	0.0024319	0.0015472	0.0010313	0.0052666	0.0000000
ENSG00000259846	0.0031793	0.0026282	0.0032120	0.0025857	0.0000000
CDH11	0.3272801	0.2771784	0.2380520	0.5408793	0.3900695
LINC02126	0.0000000	0.0007887	0.0000000	0.0000000	0.0000000
ENSG00000260834	0.0000000	0.0014288	0.0000000	0.0000000	0.0000000
ENSG00000260364	0.0007516	0.0000000	0.0000000	0.0057617	0.0194041
LINC00922	0.0051159	0.0058300	0.0000000	0.0062584	0.0338523
ENSG00000261818	0.0000000	0.0000000	0.0000000	0.0000000	0.0000000
ENSG00000260695	0.0020434	0.0000000	0.0000000	0.0000000	0.0000000
CDH5	0.0000000	0.0000000	0.0000000	0.0000000	0.0014498
LINC00920	0.0048659	0.0025562	0.0111749	0.0022345	0.0000000
BEAN1	0.0307200	0.0734374	0.0489474	0.0122308	0.0489620
BEAN1-AS1	0.0008516	0.0011519	0.0041097	0.0000000	0.0089342
TK2	0.1973936	0.1182749	0.0975860	0.1836400	0.1391247
ENSG00000260755	0.0089015	0.0019679	0.0073995	0.0074439	0.0000000
ENSG00000261519	0.0012342	0.0023475	0.0040493	0.0138350	0.0000000
CKLF	0.3337438	0.2826252	0.2536356	0.3607458	0.3490223
ENSG00000277978	0.0035069	0.0059110	0.0079718	0.0020543	0.0065051
CMTM1	0.0562109	0.0831646	0.1168232	0.0437588	0.0884503
ENSG00000289353	0.1229460	0.1869780	0.2548959	0.1228501	0.1458961
CMTM2	0.0000000	0.0000000	0.0000000	0.0059567	0.0058461
CMTM3	0.2707969	0.1782928	0.1131097	0.3305420	0.1580004
CMTM4	0.2796802	0.2411296	0.1980150	0.2545232	0.2801224
DYNC1LI2	1.5282958	1.4657956	1.4571721	1.3634573	1.3215801
ENSG00000260465	0.0075625	0.0078792	0.0099880	0.0097068	0.0113863
ENSG00000287965	0.0000000	0.0000000	0.0000000	0.0031874	0.0139625
ENSG00000260558	0.0000000	0.0012059	0.0000000	0.0000000	0.0000000
DYNC1LI2-DT	0.0027018	0.0035373	0.0023792	0.0000000	0.0061992
TERB1	0.0042844	0.0056023	0.0000000	0.0000000	0.0138968
NAE1	0.5461082	0.5642543	0.6095588	0.5177975	0.5009139
ENSG00000258122	0.0006885	0.0041404	0.0076300	0.0021110	0.0026235
PDP2	0.1057624	0.1098417	0.1200767	0.1142155	0.1119661
RRAD	0.0147114	0.0072489	0.0075794	0.0326464	0.0109725
CIAO2B	1.1727495	1.1794543	1.2145613	1.1992525	0.9186334
CES2	0.1661227	0.1880615	0.1494063	0.1974581	0.1924779
CES3	0.0012238	0.0042458	0.0031385	0.0000000	0.0147852
CES4A	0.0198705	0.0114974	0.0176086	0.0037280	0.0248272

ENSG00000289415	0.0107285	0.0166110	0.0134052	0.0052761	0.0294781
CBFB	0.2387338	0.2280508	0.1464666	0.1866483	0.3318551
PHAF1	0.0973275	0.0765106	0.1020622	0.0952190	0.1213268
B3GNT9	0.0879503	0.0488486	0.0301048	0.1305087	0.0338059
TRADD	0.0420663	0.0338559	0.0262170	0.0413821	0.0479945
FBXL8	0.0279861	0.0307854	0.0143098	0.0246763	0.0557600
HSF4	0.0332478	0.0480796	0.0216695	0.0261282	0.0284278
NOL3	0.3755744	0.2497837	0.2203671	0.3237390	0.2212745
MATCAP1	0.2327844	0.3779504	0.4023519	0.1957008	0.4054643
EXOC3L1	0.0064771	0.0027844	0.0069485	0.0000000	0.0058842
E2F4	0.1086692	0.1247775	0.1278114	0.1183081	0.0871012
ELMO3	0.0060568	0.0051817	0.0049794	0.0000000	0.0043076
TMEM208	0.3191934	0.2873393	0.3502389	0.4172573	0.2770917
FHOD1	0.0103442	0.0085255	0.0090773	0.0185587	0.0069871
SLC9A5	0.0133142	0.0125739	0.0319124	0.0040613	0.0555508
PLEKHG4	0.0006463	0.0014476	0.0022183	0.0016517	0.0065249
KCTD19	0.0136671	0.0138577	0.0045512	0.0041872	0.0043619
LRRC36	0.0190330	0.0164620	0.0079579	0.0051420	0.0295882
TPPP3	1.7558394	1.2595816	0.9838035	1.5524176	1.1744430
ZDHHC1	0.1669176	0.1396056	0.0734440	0.2008148	0.0961911
HSD11B2	0.0026748	0.0076668	0.0086772	0.0000000	0.0135675
ATP6V0D1	0.3495696	0.4594638	0.5272593	0.3423339	0.3164565
ATP6V0D1-DT	0.0276139	0.0222337	0.0213120	0.0224483	0.0564465
AGRP	0.0037487	0.0010939	0.0019279	0.0000000	0.0013712
ENSG00000276075	0.0642887	0.0789499	0.0732714	0.0621078	0.0418087
RIPOR1	0.1250182	0.1396358	0.1601205	0.1099055	0.2003300
ENSG00000259945	0.0004093	0.0009564	0.0043713	0.0000000	0.0000000
ENSG00000261386	0.0052765	0.0098728	0.0019069	0.0000000	0.0528996
CTCF-DT	0.0056810	0.0042576	0.0042018	0.0000000	0.0000000
CTCF	0.3109479	0.3107001	0.3423268	0.3150601	0.3220503
CARMIL2	0.1037085	0.2026713	0.2809530	0.0567441	0.1350815
ACD	0.1644436	0.1622583	0.1781829	0.1576995	0.1035863
PARD6A	0.1824955	0.2477531	0.3405018	0.1886594	0.0949542
ENKD1	0.1507761	0.0854389	0.0733748	0.1019160	0.0724981
C16orf86	0.0658501	0.0443879	0.0154077	0.0616156	0.0397859
GFOD2	0.1741013	0.2902659	0.3457000	0.1845757	0.3170154
RANBP10	0.1065070	0.0919428	0.0713035	0.1041874	0.1890243
ENSG00000270165	0.0000000	0.0016970	0.0000000	0.0000000	0.0000000
ENSG00000289164	0.0029669	0.0020511	0.0026972	0.0036457	0.0108870
TSNAXIP1	0.0879918	0.0342086	0.0189383	0.0539139	0.0906509
CENPT	0.0970220	0.0797931	0.1050274	0.0969590	0.1152803
THAP11	0.2940239	0.2511151	0.2552863	0.2943455	0.1902536
NUTF2	0.9077540	1.0211936	1.0969682	0.9051102	0.7263533
EDC4	0.0883918	0.1336888	0.1415230	0.1194894	0.1021826
NRN1L	0.0034818	0.0006666	0.0000000	0.0000000	0.0000000
PSKH1	0.0806474	0.0603558	0.0283422	0.0688832	0.0649455
CTRL	0.0040409	0.0047886	0.0093049	0.0067138	0.0072560
PSMB10	0.3595865	0.3520580	0.3017357	0.3637385	0.2181127
LCAT	0.0023143	0.0022322	0.0024852	0.0110090	0.0046262
SLC12A4	0.0912669	0.0300043	0.0346953	0.1119224	0.0732155
DUS2	0.0554953	0.0535379	0.0613822	0.0683308	0.0711767
DDX28	0.0595061	0.0507086	0.0590513	0.0838334	0.0167445
NFATC3	0.1078143	0.0919159	0.0767701	0.1116712	0.2487148

ENSG00000262514	0.0000000	0.0000000	0.0026358	0.0000000	0.0044624
ENSG00000260891	0.0002944	0.0031146	0.0000000	0.0000000	0.0054224
ENSG00000263276	0.0009366	0.0000000	0.0030148	0.0000000	0.0000000
ENSG00000262160	0.0039217	0.0011324	0.0021796	0.0019352	0.0125823
PLA2G15	0.1362237	0.0844852	0.0963912	0.2009528	0.1042771
ENSG00000260441	0.0000000	0.0000000	0.0000000	0.0028496	0.0000000
SLC7A6	0.0432179	0.0722783	0.0691580	0.0277706	0.1659495
SLC7A6OS	0.1992351	0.1481851	0.1320768	0.1586840	0.1539492
PRMT7	0.1039344	0.1017640	0.0705548	0.1032203	0.1295906
SMPD3	0.1118377	0.2037332	0.2490204	0.0741266	0.3189347
ENSG00000287760	0.0139744	0.0274851	0.0317893	0.0035903	0.0083850
ENSG00000274698	0.0023635	0.0114024	0.0105854	0.0013375	0.0067493
ZFP90	0.4596500	0.5551962	0.5785327	0.3972829	0.5975586
ENSG00000260084	0.0007994	0.0000000	0.0000000	0.0000000	0.0044278
ENSG00000275383	0.0056677	0.0010508	0.0116839	0.0000000	0.0064298
CDH3	0.0122231	0.0225455	0.0156618	0.0319036	0.0682116
CDH3-AS1	0.0022689	0.0021380	0.0019328	0.0028421	0.0000000
CDH1	0.1208470	0.0610741	0.0257001	0.3409866	0.0664587
TANGO6	0.0771367	0.0848305	0.0655914	0.0520986	0.1872494
ENSG00000260999	0.0015003	0.0000000	0.0000000	0.0000000	0.0031970
HAS3	0.0118206	0.0347698	0.0538646	0.0125656	0.0074816
CHTF8	0.0174953	0.0278071	0.0112058	0.0082049	0.0142876
DERPC	0.0035981	0.0005957	0.0040728	0.0026911	0.0041311
UTP4	0.1139313	0.1151999	0.1198041	0.1224538	0.1239591
SNTB2	0.0763996	0.0807685	0.0762894	0.0665230	0.1423229
VPS4A	0.5424576	0.6589475	0.6802513	0.4856740	0.5300532
COG8	0.0986144	0.0966850	0.0561684	0.1190041	0.0764315
PDF	0.0047749	0.0037153	0.0089187	0.0048286	0.0023981
NIP7	0.1346368	0.0980313	0.0845874	0.1434245	0.0386418
TMED6	0.0020598	0.0039381	0.0000000	0.0052676	0.0033534
TERF2	0.4570898	0.4607851	0.5829595	0.4425205	0.3951790
CYB5B	0.5426470	0.5755502	0.5731429	0.6079717	0.3844356
NFAT5	0.3621274	0.3115545	0.3371795	0.3556065	0.6966669
NQO1	0.2042627	0.1140241	0.0778820	0.3001731	0.1115490
NQO1-DT	0.0075565	0.0099586	0.0000000	0.0000000	0.0086576
NOB1	0.2377524	0.2134332	0.1313225	0.2054527	0.1933629
WWP2	0.0888716	0.0821664	0.0886559	0.0979050	0.1187708
CLEC18A	0.0000000	0.0000000	0.0000000	0.0000000	0.0000000
ENSG00000290459	0.0112211	0.0114880	0.0057886	0.0081113	0.0445718
ENSG00000290709	0.0336961	0.0337693	0.0448286	0.0169486	0.0653250
PDPR	0.1001062	0.0890616	0.0795524	0.0989440	0.1797996
ENSG00000247228	0.0023279	0.0060685	0.0000000	0.0015981	0.0073858
CLEC18C	0.0000000	0.0000000	0.0000000	0.0000000	0.0000000
SMG1P7	0.0102538	0.0110109	0.0102833	0.0119920	0.0252413
ENSG00000291219	0.0073626	0.0111264	0.0132815	0.0100463	0.0158821
EXOSC6	0.3109772	0.3183962	0.3074325	0.2824221	0.3004977
AARS1	0.4420433	0.3980224	0.3880570	0.4308727	0.3983765
DDX19B	0.1975681	0.1717860	0.1843461	0.1496396	0.2023214
DDX19A-DT	0.0141848	0.0138928	0.0106905	0.0200565	0.0291683
DDX19A	0.2531939	0.2065559	0.1816757	0.2649720	0.2785760
ENSG00000285710	0.0035915	0.0067546	0.0000000	0.0000000	0.0147983
ST3GAL2	0.1042156	0.1173830	0.1242202	0.0867431	0.1458299
ENSG00000260111	0.0023992	0.0013778	0.0000000	0.0000000	0.0077839

FCSK	0.0215349	0.0195413	0.0142426	0.0333241	0.0176053
COG4	0.1722027	0.1712468	0.1482258	0.2195554	0.1858136
SF3B3	0.2771714	0.2770839	0.2848412	0.3146120	0.2566682
IL34	0.0165856	0.0106301	0.0075251	0.0334577	0.0333757
MTSS2	1.3599648	1.0559727	0.7602709	1.1781115	0.9587235
VAC14	0.0982988	0.1017049	0.1335195	0.0725494	0.1274232
ENSG00000260156	0.0050545	0.0011682	0.0031986	0.0000000	0.0000000
VAC14-AS1	0.0265819	0.0175844	0.0058372	0.0377718	0.0344089
HYDIN	0.4259637	0.2707580	0.2051220	0.4085469	0.3822763
ENSG00000259833	0.0000000	0.0000000	0.0000000	0.0000000	0.0000000
CMTR2	0.0745834	0.0814722	0.0675207	0.0636488	0.0806340
ENSG00000261260	0.0007803	0.0042557	0.0053935	0.0000000	0.0040644
CALB2	0.2625542	0.5415792	1.2651000	0.2434238	0.3743922
LINC02136	0.0024628	0.0060509	0.0102937	0.0049899	0.0415204
ZNF23	0.1650380	0.2057194	0.2420596	0.1602767	0.1654185
ENSG00000247324	0.0025360	0.0021412	0.0106289	0.0013531	0.0000000
ZNF19	0.0827240	0.0597937	0.0350856	0.0616529	0.0725795
CHST4	0.0008436	0.0000000	0.0000000	0.0000000	0.0061591
TAT-AS1	0.0053197	0.0051533	0.0068832	0.0000000	0.0049014
MARVELD3	0.0081018	0.0041965	0.0000000	0.0021003	0.0000000
PHLPP2	0.0812456	0.1135965	0.1073801	0.0569979	0.2116164
ENSG00000260185	0.0000000	0.0016036	0.0000000	0.0062584	0.0000000
AP1G1	0.5174242	0.4635216	0.4954643	0.4301161	0.6233902
ATXN1L	0.0899477	0.0817563	0.0509531	0.0918211	0.0950727
IST1	0.4446587	0.3299118	0.3287097	0.4401526	0.3349602
ZNF821	0.2521358	0.2953134	0.4028389	0.2131309	0.2767754
ENSG00000261765	0.0000000	0.0000000	0.0000000	0.0000000	0.0000000
PKD1L3	0.0000000	0.0028599	0.0000000	0.0000000	0.0076020
DHODH	0.0417781	0.0490831	0.0275025	0.0745674	0.0369501
TXNL4B	0.0878444	0.0793147	0.0687230	0.0646969	0.0983810
HP	0.0914403	0.0538910	0.0327593	0.0838268	0.0372046
HPR	0.0000000	0.0020917	0.0000000	0.0000000	0.0000000
DHX38	0.2242058	0.1903789	0.1753074	0.1795067	0.1756824
PMFBP1	0.1378535	0.1924900	0.2969866	0.0944127	0.5011100
ENSG00000261673	0.0012570	0.0030287	0.0044520	0.0000000	0.0108843
LINC01572	0.1348248	0.1545222	0.2223813	0.0765318	0.3812904
ENSG00000260664	0.0065910	0.0000000	0.0000000	0.0000000	0.0220908
ZFHX3-AS1	0.0562454	0.0571459	0.0868003	0.0534665	0.0906808
ENSG00000279206	0.0025255	0.0006528	0.0000000	0.0068088	0.0065001
ZFHX3	0.5604192	0.6139079	0.6640567	0.4705743	1.1538569
ENSG00000259209	0.0003625	0.0000000	0.0119454	0.0015620	0.0000000
ENSG00000272330	0.0000000	0.0000000	0.0000000	0.0000000	0.0055669
ENSG00000271009	0.0015753	0.0000000	0.0000000	0.0019003	0.0000000
HCCAT5	0.0000000	0.0000000	0.0000000	0.0000000	0.0000000
ENSG00000284675	0.0020493	0.0027858	0.0069204	0.0000000	0.0041311
ENSG00000261227	0.0474264	0.0972409	0.1020739	0.0278962	0.1454337
LINC01568	0.0000000	0.0044834	0.0066548	0.0028274	0.0284789
ENSG00000260848	0.0009259	0.0035566	0.0024157	0.0000000	0.0205151
ENSG00000283457	0.0000000	0.0000000	0.0000000	0.0000000	0.0000000
ENSG00000259817	0.0000000	0.0000000	0.0000000	0.0000000	0.0000000
PSMD7-DT	0.0247283	0.0305755	0.0089421	0.0176461	0.1020298
ENSG00000261248	0.0000000	0.0010592	0.0000000	0.0000000	0.0000000
ENSG00000275236	0.0042689	0.0008572	0.0000000	0.0000000	0.0067617

PSMD7	1.0108998	0.9144526	0.9349462	1.0348251	0.7547444
ENSG00000259972	0.0392267	0.0649863	0.0387442	0.0560927	0.2101497
ENSG00000260884	0.0000000	0.0000000	0.0000000	0.0000000	0.0014580
ENSG00000261079	0.0035077	0.0052844	0.0000000	0.0043350	0.0041999
NPIPB15	0.0010453	0.0004908	0.0000000	0.0033511	0.0072786
CLEC18B	0.0042200	0.0056515	0.0022206	0.0072653	0.0000000
ENSG00000261170	0.0000000	0.0000000	0.0000000	0.0000000	0.0078149
GLG1	0.8697488	0.7632136	0.8411469	1.3019107	0.9428718
RFWD3	0.0228133	0.0255684	0.0226005	0.0256175	0.0354039
FA2H	0.0118105	0.0066845	0.0081090	0.0147259	0.0117942
WDR59	0.1604300	0.1495181	0.1873834	0.1358417	0.3071739
ZNRF1	0.4313067	0.6468615	0.8792832	0.4047698	0.6758157
ENSG00000247033	0.0011594	0.0116939	0.0068822	0.0000000	0.0000000
LDHD	0.0133328	0.0037823	0.0107540	0.0142493	0.0000000
ENSG00000261058	0.0041502	0.0033132	0.0000000	0.0085538	0.0243216
ZFP1	0.1790794	0.1951998	0.2389518	0.1879411	0.1804670
CTRB1	0.0014473	0.0000000	0.0000000	0.0000000	0.0000000
BCAR1	0.1103517	0.1341487	0.1205506	0.1230576	0.1949037
CFDP1	1.3610122	1.4460979	1.5510324	1.3551293	1.2860220
ENSG00000261783	0.0042386	0.0027431	0.0000000	0.0000000	0.0000000
ENSG00000274220	0.0338139	0.0325865	0.0224095	0.0142536	0.0501781
TMEM170A	0.4538722	0.3402543	0.2902604	0.5345486	0.3192196
CHST6	0.1118108	0.0638743	0.0167296	0.1322249	0.0587841
ENSG00000203472	0.0033068	0.0037226	0.0000000	0.0023036	0.0027533
ENSG00000273971	0.0009987	0.0025723	0.0013013	0.0000000	0.0036228
ENSG00000291051	0.0320997	0.0177934	0.0344892	0.0238580	0.0286599
CHST5	0.0037202	0.0020204	0.0056580	0.0061991	0.0000000
TMEM231	0.1827400	0.1220900	0.0969432	0.2683290	0.1673418
ENSG00000276408	0.0025208	0.0111623	0.0125510	0.0000000	0.0121500
GABARAPL2	1.4508262	1.5050632	1.5667184	1.4552776	1.1317864
ENSG00000276523	0.0000000	0.0031490	0.0000000	0.0017080	0.0000000
ADAT1	0.0995528	0.1616066	0.1634926	0.0996868	0.2130426
KARS1	0.3707135	0.3620359	0.3784441	0.3673603	0.2990346
TERF2IP	1.5508892	1.6335167	2.0565985	1.4609829	1.4950861
CPHXL2	0.0000000	0.0013468	0.0000000	0.0000000	0.0079149
CPHXL	0.0000000	0.0000000	0.0015424	0.0000000	0.0000000
ENSG00000261313	0.0010546	0.0021698	0.0000000	0.0000000	0.0084152
ENSG00000260223	0.0000000	0.0000000	0.0000000	0.0000000	0.0000000
ENSG00000260983	0.0000000	0.0016440	0.0026803	0.0000000	0.0000000
ENSG00000286841	0.0000000	0.0000000	0.0000000	0.0000000	0.0053413
CNTNAP4	0.0682227	0.0801079	0.0986628	0.0425265	0.3310968
ENSG00000273659	0.0000000	0.0000000	0.0000000	0.0000000	0.0000000
LINC02125	0.0000000	0.0011466	0.0000000	0.0000000	0.0000000
ENSG00000259995	0.0000000	0.0000000	0.0000000	0.0040556	0.0000000
MON1B	0.1266217	0.1204187	0.1146692	0.1037308	0.1306780
SYCE1L	0.0752667	0.0934750	0.1159284	0.0726085	0.0501831
ENSG00000261063	0.0062132	0.0000000	0.0013013	0.0082021	0.0174040
ENSG00000260922	0.0035042	0.0031940	0.0163021	0.0045938	0.0048241
ADAMTS18	0.0337641	0.0808842	0.0284269	0.0248866	0.0846322
ENSG00000260701	0.0000000	0.0009785	0.0100022	0.0000000	0.0096631
NUDT7	0.1001093	0.0541618	0.0522504	0.1241242	0.0507746
VAT1L	0.2384494	0.4598830	0.6542630	0.1749778	0.7168976
CLEC3A	0.0000000	0.0000000	0.0045456	0.0000000	0.0000000

WWOX	0.4958906	0.3877394	0.3028311	0.4531993	0.7362265
ENSG00000276007	0.0218633	0.0242245	0.0112345	0.0086057	0.0094566
ENSG00000260733	0.0000000	0.0000000	0.0000000	0.0000000	0.0032179
ENSG00000260816	0.0029988	0.0022171	0.0000000	0.0000000	0.0070107
ENSG00000260694	0.0000000	0.0000000	0.0000000	0.0000000	0.0000000
ENSG00000277954	0.0244095	0.0153315	0.0422061	0.0098129	0.0274587
ENSG00000285918	0.0034866	0.0046458	0.0114768	0.0075182	0.0058922
ENSG00000261722	0.0069767	0.0100347	0.0055379	0.0024198	0.0022313
ENSG00000261472	0.0000000	0.0000000	0.0000000	0.0000000	0.0000000
MAF	0.6085180	0.3655362	0.2532418	0.4997870	0.3456940
ENSG00000278058	0.0120038	0.0115186	0.0102978	0.0049827	0.0084447
LINC01229	0.0059452	0.0031175	0.0000000	0.0047682	0.0000000
MAFTRR	0.0144524	0.0020832	0.0028783	0.0000000	0.0000000
DYNLRB2-AS1	0.0136674	0.0153796	0.0304899	0.0161282	0.0692379
DYNLRB2	0.2028039	0.1182859	0.1010171	0.1340721	0.0835033
LINC01227	0.0006385	0.0000000	0.0025691	0.0000000	0.0000000
CDYL2	0.0692829	0.0982413	0.1087095	0.0973370	0.2165997
ENSG00000260064	0.0000000	0.0000000	0.0000000	0.0000000	0.0079758
ARLNC1	0.0061050	0.0045322	0.0148998	0.0041774	0.0078893
CMC2	0.4472172	0.5746389	0.6603111	0.5139804	0.5270026
CENPN	0.0302456	0.0422732	0.0270337	0.0370583	0.0246480
CENPN-AS1	0.0110909	0.0073994	0.0069358	0.0086955	0.0087428
ATMIN	0.3926541	0.5394587	0.6232858	0.4658576	0.4471419
C16orf46	0.2098925	0.1688286	0.1852268	0.2250563	0.2103484
ENSG00000261141	0.0072735	0.0022561	0.0113490	0.0102093	0.0057866
ENSG00000261838	0.0000000	0.0011596	0.0000000	0.0000000	0.0074093
ENSG00000245059	0.0019603	0.0007695	0.0035296	0.0000000	0.0091816
GCSH	1.1425451	1.0504615	1.0438675	1.3100488	0.8446458
PKD1L2	0.0060885	0.0053120	0.0033576	0.0018125	0.0179386
BCO1	0.0003654	0.0022458	0.0000000	0.0040755	0.0130799
ENSG00000260495	0.0004442	0.0000000	0.0013389	0.0000000	0.0113417
ENSG00000289067	0.0016755	0.0000000	0.0000000	0.0000000	0.0026801
GAN	0.1875249	0.2296672	0.3056598	0.1663448	0.2602472
CMIP	0.8451072	1.0194748	1.1597029	0.8332003	1.2582223
ENSG00000261218	0.0007128	0.0023098	0.0018826	0.0031240	0.0000000
PLCG2	0.3550977	0.2928598	0.2154566	0.2329851	0.6519678
HSD17B2	0.0000000	0.0000000	0.0000000	0.0000000	0.0033679
HSD17B2-AS1	0.0259356	0.0374034	0.0291311	0.0272463	0.0394242
MPHOSPH6	0.3983936	0.3642312	0.4468884	0.4313264	0.3116027
MPHOSPH6-DT	0.0074185	0.0049744	0.0064955	0.0130017	0.0124655
CDH13	0.0690683	0.1498492	0.1790315	0.0773790	0.4127853
ENSG00000260862	0.0000000	0.0008723	0.0000000	0.0000000	0.0000000
ENSG00000260832	0.0000000	0.0000000	0.0000000	0.0000000	0.0000000
CDH13-AS1	0.0000000	0.0000000	0.0000000	0.0000000	0.0000000
HSBP1	1.9240261	1.8992732	2.0039828	1.9541632	1.5569132
CEDORA	0.0006036	0.0000000	0.0000000	0.0000000	0.0069341
CDH13-AS2	0.0000000	0.0012114	0.0000000	0.0011920	0.0055061
MLYCD	0.1092733	0.0784394	0.0911811	0.0860450	0.0971742
OSGIN1	0.0480650	0.0140299	0.0026725	0.0538189	0.0097812
NECAB2	0.2494941	0.3892291	0.4988189	0.1519015	0.2476182
SLC38A8	0.0021446	0.0058132	0.0000000	0.0124637	0.0033030
MBTPS1	0.5806051	0.4214849	0.3940265	0.5623479	0.5417380
MBTPS1-DT	0.0403137	0.0412207	0.0409842	0.0251216	0.0275259

HSDL1	0.3845106	0.4240870	0.5788784	0.3682736	0.3639383
DNAAF1	0.0514755	0.0350678	0.0288006	0.0706765	0.0171782
TAF1C	0.0415852	0.0426616	0.0189712	0.0720333	0.0296498
ADAD2	0.0004253	0.0010959	0.0023388	0.0000000	0.0000000
ENSG00000250685	0.0000000	0.0000000	0.0042868	0.0000000	0.0000000
WFDC1	0.0202857	0.0364181	0.0200046	0.0400776	0.0103474
ATP2C2	0.0193451	0.0115518	0.0072701	0.0486437	0.0391147
ATP2C2-AS1	0.0073606	0.0127062	0.0071539	0.0049155	0.0306307
MEAK7	0.0861214	0.1158330	0.0908145	0.1185918	0.1368325
COTL1	1.0080241	1.0581307	1.0175471	1.0394013	0.7086058
KLHL36	0.2037618	0.2024678	0.2063805	0.1826995	0.2060005
USP10	0.4423476	0.4867457	0.4648548	0.4437366	0.4057380
ENSG00000285848	0.0000000	0.0020993	0.0000000	0.0000000	0.0000000
CRISPLD2	0.0114208	0.0133135	0.0077465	0.0124695	0.0208616
ZDHHC7	0.1925574	0.1629464	0.1333585	0.2336671	0.1847318
ENSG00000289551	0.0011641	0.0043275	0.0000000	0.0033765	0.0173028
KIAA0513	0.1237439	0.1576585	0.1967046	0.1466704	0.2344982
CIBAR2	0.1703398	0.1061343	0.0586081	0.1614820	0.1052710
GSE1	0.3059741	0.3149834	0.3066020	0.3330605	0.8393188
LINC00311	0.0000000	0.0000000	0.0000000	0.0000000	0.0000000
ENSG00000270124	0.0026985	0.0000000	0.0000000	0.0026862	0.0000000
GINS2	0.0565827	0.0627327	0.0364394	0.1018350	0.0493288
C16orf74	0.0164112	0.0201396	0.0055271	0.0410466	0.0236267
ENSG00000287125	0.0000000	0.0000000	0.0015332	0.0000000	0.0000000
EMC8	0.3490183	0.3302779	0.2473363	0.3871415	0.2124682
COX4I1	1.8638720	2.0326088	2.0131388	1.9093821	1.6223452
IRF8	0.0000000	0.0000000	0.0000000	0.0018691	0.0000000
LINC01081	0.0017090	0.0050900	0.0011326	0.0000000	0.0000000
FENDRR	0.0028880	0.0016301	0.0051252	0.0000000	0.0208469
FOXF1	0.0050069	0.0035920	0.0019265	0.0070997	0.0102016
ENSG00000270020	0.0111256	0.0036711	0.0013432	0.0011545	0.0071269
MTHFSD	0.0987207	0.0899666	0.0677043	0.0709632	0.1069948
FOXC2	0.0016779	0.0013447	0.0000000	0.0000000	0.0000000
FOXL1	0.0018495	0.0000000	0.0011718	0.0000000	0.0000000
LINC02188	0.0000000	0.0000000	0.0038350	0.0000000	0.0000000
C16orf95	0.0374621	0.0436828	0.0371654	0.0280314	0.0872701
C16orf95-DT	0.0521594	0.0352177	0.0312465	0.0394057	0.0433804
FBXO31	0.0778570	0.0772977	0.0672179	0.0871275	0.1576952
ENSG00000269901	0.0000000	0.0013052	0.0000000	0.0000000	0.0074093
MAP1LC3B	1.3705184	1.3981117	1.5311001	1.3864938	1.0442503
ENSG00000261592	0.0000000	0.0000000	0.0000000	0.0000000	0.0061020
ZCCHC14	0.1468880	0.2090891	0.2070748	0.2123856	0.3434705
ENSG00000276337	0.0013063	0.0038166	0.0000000	0.0000000	0.0028494
ZCCHC14-DT	0.0034584	0.0012199	0.0086870	0.0000000	0.0067351
ENSG00000269935	0.0108343	0.0257816	0.0328668	0.0139995	0.0264396
JPH3	0.1907839	0.3190076	0.3385267	0.1853868	0.3848715
ENSG00000277504	0.0000000	0.0025569	0.0000000	0.0000000	0.0000000
ENSG00000226180	0.0012369	0.0000000	0.0000000	0.0000000	0.0000000
KLHDC4	0.0833007	0.0811269	0.0826170	0.0773492	0.1615718
ENSG00000260498	0.0025099	0.0035490	0.0000000	0.0000000	0.0074185
ENSG00000260177	0.0018216	0.0000000	0.0000000	0.0037712	0.0093450
SLC7A5	0.2644671	0.2494631	0.2263238	0.2559821	0.2623483
ENSG00000260466	0.0070429	0.0013562	0.0000000	0.0025449	0.0038927

CA5A	0.0008382	0.0000000	0.0000000	0.0000000	0.0047388
BANP	0.1160441	0.0965469	0.0663268	0.1325047	0.1848857
ENSG00000261193	0.0006468	0.0000000	0.0054273	0.0023342	0.0159104
ENSG00000205037	0.0000000	0.0000000	0.0025623	0.0000000	0.0000000
LINC02182	0.0051918	0.0116035	0.0101923	0.0031165	0.0201983
ENSG00000261273	0.0042387	0.0004908	0.0000000	0.0000000	0.0144922
ZNF469	0.0097213	0.0108759	0.0077912	0.0000000	0.0428391
ZFPM1	0.3336046	0.3109681	0.3029177	0.3118636	0.2461244
ZFPM1-AS1	0.0067480	0.0026033	0.0000000	0.0025449	0.0148263
ENSG00000289151	0.0012227	0.0009863	0.0045745	0.0061329	0.0000000
ZC3H18	0.2644083	0.2808816	0.2898989	0.2097573	0.2971297
CYBA	0.4990585	0.3420596	0.2122152	0.7466702	0.3164062
MVD	0.2680591	0.2623357	0.2592397	0.2949298	0.3119085
SNAI3-AS1	0.0750864	0.1118894	0.1409421	0.0641494	0.1909012
SNAI3	0.0223759	0.0484134	0.0284114	0.0347543	0.0706295
RNF166	0.0845471	0.0819346	0.0888192	0.0723966	0.0672710
CTU2	0.0717662	0.0897168	0.1175648	0.1026254	0.0900585
PIEZO1	0.0053290	0.0083595	0.0065587	0.0028675	0.0123785
ENSG00000260121	0.0017555	0.0019397	0.0007371	0.0000000	0.0000000
ENSG00000224888	0.0042672	0.0018332	0.0000000	0.0000000	0.0034190
ENSG00000182376	0.0012625	0.0012444	0.0000000	0.0000000	0.0000000
CDT1	0.0567333	0.0614408	0.0619308	0.0722786	0.0587922
APRT	0.5500436	0.4470509	0.3737549	0.5765671	0.4006423
GALNS	0.2607621	0.1331205	0.0676635	0.3690324	0.1612029
TRAPPC2L	0.4506295	0.4525360	0.5118269	0.4520330	0.3746982
PABPN1L	0.0000000	0.0006130	0.0000000	0.0000000	0.0067724
CBFA2T3	0.0960520	0.1335447	0.1280764	0.0498720	0.1659836
ENSG00000261226	0.0000000	0.0023470	0.0000000	0.0000000	0.0000000
ENSG00000205018	0.0005931	0.0049645	0.0064118	0.0025239	0.0112560
ACSF3	0.0623782	0.0763307	0.0844916	0.0636977	0.1420843
LINC00304	0.0000000	0.0041310	0.0000000	0.0000000	0.0000000
CDH15	0.0038631	0.0040387	0.0008854	0.0017571	0.0000000
SLC22A31	0.0005505	0.0000000	0.0000000	0.0040080	0.0000000
ZNF778-DT	0.0487572	0.0545455	0.0704559	0.0162702	0.0331873
ZNF778	0.0402531	0.0584618	0.0749271	0.0197526	0.0651562
ANKRD11	0.8695802	0.9460861	0.9892463	0.8994485	1.2203687
ENSG00000268218	0.0013660	0.0000000	0.0000000	0.0000000	0.0098710
ENSG00000260279	0.0072249	0.0067080	0.0058700	0.0070964	0.0086051
ENSG00000261253	0.0000000	0.0000000	0.0000000	0.0000000	0.0000000
ENSG00000287351	0.0013404	0.0000000	0.0000000	0.0000000	0.0157239
SPG7	0.1408078	0.1641116	0.1470643	0.2383568	0.2619535
ENSG00000261118	0.0219641	0.0148023	0.0101008	0.0318077	0.0414218
RPL13	3.5702869	3.4904359	3.2817093	3.4554498	3.2167369
CPNE7	0.0381078	0.0442435	0.0847871	0.0252268	0.1613782
DPEP1	0.0040961	0.0000000	0.0015569	0.0045236	0.0056902
CHMP1A	0.3181974	0.3551651	0.3167950	0.3153811	0.2796862
SPATA33	0.2129827	0.1684603	0.1494065	0.1942852	0.1542286
ENSG00000275734	0.0140316	0.0308374	0.0012257	0.0091207	0.0660340
CDK10	0.1159141	0.1124354	0.1087766	0.1552481	0.2254154
LINC02166	0.0533816	0.0349335	0.0288915	0.0359336	0.0088613
SPATA2L	0.0602624	0.0525196	0.0491303	0.0944805	0.0600463
VPS9D1	0.0776452	0.1057878	0.1084114	0.0828579	0.1084732
VPS9D1-AS1	0.0013779	0.0025499	0.0000000	0.0019455	0.0000000

ZNF276	0.0421076	0.0486885	0.0420921	0.0671981	0.0952337
FANCA	0.0100459	0.0292714	0.0198206	0.0117011	0.0438981
SPIRE2	0.0462612	0.0740127	0.0849145	0.0319058	0.1996825
TCF25	1.1050236	1.0516106	1.1102181	1.1749878	1.1537753
MC1R	0.0174999	0.0166799	0.0124990	0.0168620	0.0877081
ENSG00000259006	0.0012912	0.0043415	0.0017028	0.0029920	0.0065249
TUBB3	1.6910622	2.4570835	2.9018393	1.7189711	1.9860231
DEF8	0.2371939	0.3070841	0.3752504	0.2013038	0.2483829
DBNDD1	0.1618603	0.2158682	0.2892187	0.1958291	0.1516371
GAS8	0.1406185	0.1060753	0.0778943	0.1227720	0.2793604
URAHP	0.0099612	0.0023156	0.0075870	0.0050440	0.0192432
PRDM7	0.0000000	0.0012926	0.0018284	0.0000000	0.0000000
FAM157C	0.0167601	0.0129358	0.0154439	0.0023733	0.0472237
ENSG00000260507	0.0018606	0.0008127	0.0017068	0.0000000	0.0099888
ENSG00000261172	0.0000000	0.0000000	0.0000000	0.0000000	0.0000000
LINC02193	0.0082463	0.0042595	0.0094559	0.0030842	0.0168437
ENSG00000280136	0.0000000	0.0000000	0.0000000	0.0000000	0.0015201
DOC2B	0.2139930	0.3155138	0.4302707	0.2122691	0.3425746
LINC02091	0.0022217	0.0004876	0.0024615	0.0000000	0.0000000
RPH3AL	0.0733034	0.0557837	0.0329605	0.0603940	0.1289477
ENSG00000262920	0.0000000	0.0013092	0.0000000	0.0000000	0.0000000
RPH3AL-AS2	0.0052060	0.0020594	0.0000000	0.0000000	0.0000000
ENSG00000241525	0.0021008	0.0000000	0.0009315	0.0000000	0.0138046
C17orf97	0.3366973	0.2160407	0.1857799	0.3444053	0.2470198
RFLNB	0.2818886	0.2648198	0.2542580	0.1952402	0.1700365
ENSG00000263015	0.0022373	0.0014737	0.0000000	0.0000000	0.0052886
VPS53	0.4191006	0.4042701	0.4590694	0.3099249	0.6495760
ENSG00000263300	0.0011066	0.0033265	0.0000000	0.0019922	0.0136577
TLCD3A	0.1747509	0.1400219	0.1289693	0.2016487	0.1071975
GEMIN4	0.0283099	0.0269984	0.0152782	0.0172281	0.0169029
GLOD4	0.6147175	0.6503420	0.7725923	0.6108378	0.4582393
MRM3	0.1172823	0.1163905	0.1061190	0.0998289	0.0551075
NXN	0.1387887	0.1334413	0.1304640	0.1537342	0.3810270
ENSG00000262133	0.0023964	0.0125114	0.0102908	0.0000000	0.0044078
TIMM22	0.2258306	0.2182893	0.2463933	0.2231433	0.1944628
ABR	0.3246948	0.3392268	0.3232315	0.3085035	0.5214323
ABR-AS1	0.0017754	0.0035834	0.0000000	0.0000000	0.0093689
YWHAE	2.5458742	2.5614391	2.7241878	2.5947297	2.2527815
CRK	0.3906717	0.4136223	0.4394672	0.3356355	0.4244320
MYO1C	0.0651338	0.0875835	0.0458969	0.0403658	0.0568134
INPP5K	0.1049082	0.1069246	0.0707706	0.0660358	0.0927552
PITPNA-AS1	0.2443082	0.2292326	0.2478215	0.2361450	0.1522676
PITPNA	0.4515165	0.5604615	0.6172684	0.4250379	0.4694835
SLC43A2	0.3571330	0.2731809	0.2795435	0.5093343	0.3408073
SCARF1	0.0027458	0.0059670	0.0052226	0.0050626	0.0000000
RILP	0.0249127	0.0299872	0.0665882	0.0442137	0.0357700
PRPF8	0.4459883	0.4712636	0.5387954	0.4273053	0.3229251
ENSG00000277597	0.0110267	0.0019820	0.0015236	0.0088733	0.0186783
TLCD2	0.1163570	0.0813879	0.0483395	0.1111921	0.0741115
MIR22HG	0.0864318	0.0926807	0.0941909	0.0481197	0.0860182
WDR81	0.0291331	0.0223496	0.0140524	0.0255479	0.0217922
SERPINF2	0.0361597	0.0176451	0.0085204	0.0281705	0.0048680
SERPINF1	0.7186419	0.4207732	0.2484875	0.7971822	0.3915314

SMYD4	0.0952991	0.0646860	0.0507673	0.0915599	0.1271170
RPA1	0.2374264	0.1496886	0.1130330	0.2220623	0.1830646
RTN4RL1	0.0169310	0.0240867	0.0104402	0.0036094	0.0551268
ENSG00000262445	0.0006630	0.0000000	0.0000000	0.0000000	0.0000000
DPH1	0.0336714	0.0351219	0.0289413	0.0517093	0.0458099
ENSG00000287553	0.0109516	0.0127654	0.0161725	0.0035391	0.0132363
DPH1-AS1	0.0050790	0.0051253	0.0000000	0.0011876	0.0076298
HIC1	0.0032063	0.0067364	0.0049261	0.0029154	0.0177406
SMG6	0.3105476	0.2645575	0.2491628	0.3598135	0.4786742
ENSG00000262810	0.0000000	0.0000000	0.0022769	0.0000000	0.0000000
SRR	0.2239957	0.2191333	0.2612837	0.2357277	0.2114759
TSR1	0.3094528	0.2483865	0.3106784	0.3007479	0.2447233
SGSM2	0.1454661	0.1702388	0.1892664	0.1607527	0.2806851
SGSM2-AS1	0.0000000	0.0000000	0.0000000	0.0012109	0.0000000
MNT	0.0876431	0.1286745	0.1296327	0.1474629	0.1176956
ENSG00000262456	0.0006110	0.0048607	0.0000000	0.0000000	0.0000000
METTL16	0.3280132	0.2628330	0.2332649	0.3067570	0.3196933
PAFAH1B1	1.3192031	1.5564533	1.7092599	1.3359520	1.4215756
ENSG00000272770	0.0039840	0.0061357	0.0119134	0.0011545	0.0024821
CLUH	0.0401251	0.0538437	0.0349178	0.0496631	0.0826304
ENSG00000262050	0.0233382	0.0283395	0.0333055	0.0313963	0.0353318
CCDC92B	0.0547238	0.0955088	0.0788012	0.0424132	0.0863300
RAP1GAP2	0.0705408	0.0768861	0.0860963	0.0550366	0.3806200
ENSG00000261848	0.0000000	0.0000000	0.0010852	0.0000000	0.0000000
OR3A2	0.0000000	0.0000000	0.0007808	0.0000000	0.0025831
SPATA22	0.0000000	0.0000000	0.0000000	0.0000000	0.0000000
TRPV3	0.0026081	0.0019183	0.0021379	0.0000000	0.0000000
TRPV1	0.0172199	0.0159905	0.0187927	0.0162786	0.0918848
ENSG00000261916	0.0000000	0.0000000	0.0022682	0.0000000	0.0089499
SHPK	0.0201584	0.0192398	0.0126907	0.0384574	0.0582292
CTNS	0.0431973	0.0218732	0.0255800	0.0695965	0.0669140
CTNS-AS1	0.0000000	0.0000000	0.0006144	0.0000000	0.0000000
TAX1BP3	0.1780433	0.1302760	0.0926012	0.1761983	0.1074150
EMC6	0.5042204	0.4786908	0.5692948	0.5001673	0.4167944
P2RX5	0.0177912	0.0292524	0.0389955	0.0030534	0.0177594
ITGAE	0.3539068	0.3234196	0.3527469	0.3441461	0.2906143
ENSG00000262692	0.0003817	0.0000000	0.0000000	0.0000000	0.0012623
HASPIN	0.0005401	0.0000000	0.0000000	0.0000000	0.0059786
NCBP3	0.4410397	0.4529426	0.5968200	0.4682057	0.4619445
CAMKK1	0.0264637	0.0445987	0.0545505	0.0303807	0.1202136
P2RX1	0.0010363	0.0000000	0.0000000	0.0000000	0.0000000
ATP2A3	0.0008615	0.0039425	0.0053762	0.0029052	0.0000000
ZZEF1	0.1334228	0.1494860	0.1198859	0.1085816	0.3035847
CYB5D2	0.3121910	0.2922990	0.2827159	0.3785712	0.3399281
ANKFY1	0.2219459	0.1708810	0.1823499	0.2343468	0.3957195
ENSG00000263165	0.0000000	0.0000000	0.0000000	0.0000000	0.0000000
ENSG00000274363	0.0002905	0.0000000	0.0000000	0.0000000	0.0018039
UBE2G1	0.3522164	0.3510098	0.3334911	0.3741149	0.4451852
SPNS3	0.0006692	0.0010884	0.0000000	0.0000000	0.0000000
ENSG00000262823	0.0012583	0.0052569	0.0000000	0.0023840	0.0035317
SPNS2-AS1	0.0000000	0.0018160	0.0000000	0.0000000	0.0000000
SPNS2	0.0289848	0.0757931	0.0573469	0.0268994	0.0794309
MYBBP1A	0.1050118	0.0840310	0.0967717	0.0711953	0.1263883

SMTNL2	0.1333897	0.0846846	0.0840208	0.1145847	0.0945730
LINC01996	0.0027327	0.0038126	0.0011798	0.0000000	0.0073980
ALOX15	0.0147382	0.0047042	0.0000000	0.0039642	0.0043646
PELP1	0.2224567	0.2559356	0.2457005	0.2045908	0.2175890
ENSG00000235085	0.0008902	0.0018768	0.0000000	0.0000000	0.0049042
PELP1-DT	0.0052102	0.0016059	0.0028781	0.0037014	0.0039740
ARRB2	0.1765927	0.2712505	0.3315900	0.1018871	0.2234980
MED11	0.1920208	0.1420691	0.0862029	0.1963680	0.0884664
CXCL16	0.0507902	0.0278531	0.0223047	0.0562345	0.0375096
ZMYND15	0.0018474	0.0000000	0.0000000	0.0051762	0.0000000
VMO1	0.0034860	0.0081626	0.0419558	0.0114602	0.0134795
GLTPD2	0.0002181	0.0040682	0.0053921	0.0032175	0.0020362
PSMB6	0.8192881	0.8349413	0.8249695	0.8421276	0.5497108
C17orf114	0.0893530	0.0556253	0.0492620	0.0890152	0.0311912
PLD2	0.0305070	0.0250400	0.0207154	0.0389075	0.0206614
MINK1	0.0867093	0.0939424	0.0615623	0.0781470	0.1494469
CHRNE	0.0160658	0.0124105	0.0027634	0.0176662	0.0268213
C17orf107	0.0024626	0.0040538	0.0022896	0.0000000	0.0065298
GP1BA	0.0026304	0.0000000	0.0000000	0.0000000	0.0038147
SLC25A11	0.4633957	0.4871579	0.4709743	0.4441948	0.3075649
RNF167	0.4839938	0.4619088	0.4693160	0.5262880	0.3286270
PFN1	1.7120076	1.6438084	1.6141502	1.7374962	1.3137157
ENO3	0.0148660	0.0156692	0.0415994	0.0075556	0.0329862
SPAG7	0.5625235	0.5424866	0.5119652	0.5493774	0.5027612
CAMTA2	0.0789997	0.1092923	0.0911894	0.0852723	0.1400539
ENSG00000262429	0.0153361	0.0134576	0.0079186	0.0257666	0.0086114
CAMTA2-AS1	0.0015878	0.0040061	0.0026285	0.0012567	0.0000000
ENSG00000262227	0.0203246	0.0298253	0.0274558	0.0397202	0.0160434
INCA1	0.0142695	0.0149071	0.0145720	0.0084833	0.0037431
KIF1C	0.1697945	0.1434558	0.0959041	0.1032520	0.2116688
SLC52A1	0.0010193	0.0011519	0.0000000	0.0012213	0.0079224
ZFP3	0.0229047	0.0373731	0.0382669	0.0321775	0.0197596
ZNF232-AS1	0.2074146	0.1980132	0.2476205	0.1433424	0.1318353
ZNF232	0.0930652	0.0907613	0.0831867	0.0873818	0.1289748
USP6	0.0003480	0.0007173	0.0010790	0.0000000	0.0033094
ZNF594	0.0530214	0.0890243	0.0469343	0.0383396	0.1099906
ZNF594-DT	0.0584882	0.0513658	0.0293640	0.0415423	0.1425948
SCIMP	0.0037834	0.0000000	0.0000000	0.0000000	0.0058520
RABEP1	0.6920758	0.8215271	1.0196326	0.7284027	0.8592356
NUP88	0.1069857	0.1067569	0.1013352	0.1273319	0.1292487
ENSG00000263220	0.0000000	0.0000000	0.0000000	0.0000000	0.0044554
RPAIN	0.8714909	0.9044038	1.0125468	0.8281095	0.7380037
ENSG00000263272	0.1411119	0.1672896	0.2077075	0.1663467	0.1276603
C1QBP	0.6925536	0.7624385	0.9264536	0.7179151	0.6642225
DHX33	0.1775131	0.1055326	0.1064888	0.1344355	0.1347636
DERL2	0.4704795	0.3563240	0.3573154	0.5545834	0.3629780
MIS12	0.1612966	0.1625697	0.1402789	0.1558388	0.0806304
NLRP1	0.0279642	0.0108339	0.0078128	0.0201773	0.0033679
ENSG00000284837	0.0000000	0.0012064	0.0016818	0.0000000	0.0136761
WSCD1	0.1038415	0.2095548	0.2275867	0.1120775	0.2093607
AIPL1	0.0025135	0.0033522	0.0052406	0.0000000	0.0047972
PIMREG	0.0044292	0.0183193	0.0009424	0.0099182	0.0213142
PITPNM3	0.0616122	0.1205174	0.1322163	0.0819231	0.1762268

KIAA0753	0.1377072	0.1368035	0.1372732	0.1998355	0.2591088
ENSG00000282936	0.0011113	0.0100785	0.0073593	0.0052792	0.0156706
TXNDC17	0.5713670	0.6417638	0.6297567	0.5950027	0.4560476
MED31	0.2433471	0.2240392	0.2254909	0.2317872	0.1284461
C17orf100	0.1512958	0.1388450	0.1141492	0.0871534	0.1028955
ENSG00000261996	0.0000000	0.0000000	0.0000000	0.0000000	0.0000000
ENSG00000290366	0.0276425	0.0703117	0.2090883	0.0208336	0.0592801
SLC13A5	0.0020952	0.0043532	0.0000000	0.0059851	0.0040606
XAF1	0.0018843	0.0020377	0.0000000	0.0059587	0.0145623
FBXO39	0.0005048	0.0000000	0.0000000	0.0000000	0.0000000
TEKT1	0.2179539	0.1104800	0.0602892	0.2132507	0.1245944
ALOX12P2	0.0097459	0.0144748	0.0287080	0.0000000	0.0986345
ALOX12-AS1	0.0943688	0.0936823	0.1001867	0.0956674	0.2384917
ENSG00000262089	0.0003211	0.0038235	0.0020559	0.0084700	0.0079248
ALOX12	0.0000000	0.0000000	0.0000000	0.0000000	0.0073733
RNASEK	1.2907032	1.2686064	1.4214406	1.3350335	1.0132429
C17orf49	0.6109777	0.5861072	0.5789587	0.5778684	0.3968855
MIR497HG	0.0012313	0.0045791	0.0053976	0.0000000	0.0037431
SLC16A13	0.0071879	0.0000000	0.0000000	0.0160938	0.0132109
SLC16A11	0.0351213	0.0150529	0.0068610	0.0490816	0.0180231
ASGR2	0.0003526	0.0000000	0.0000000	0.0036827	0.0000000
ENSG00000289977	0.0000000	0.0000000	0.0000000	0.0080914	0.0000000
ASGR1	0.2455225	0.3354431	0.3879964	0.2146557	0.3161731
DLG4	0.2766708	0.4316821	0.5497216	0.2826400	0.4246688
ACADVL	0.4289302	0.2723364	0.2295531	0.4192728	0.2360724
DVL2	0.1064588	0.0695994	0.0480273	0.1349657	0.0603210
PHF23	0.2767653	0.2325859	0.2169444	0.2717133	0.1755157
GABARAP	2.1469664	2.0852968	2.0474816	2.0943268	1.7369472
CTDNEP1	0.7670864	0.8036980	0.8232800	0.6780921	0.6369984
ELP5	0.2735161	0.3157372	0.2605694	0.2365482	0.2045023
CLDN7	0.0034902	0.0028235	0.0000000	0.0000000	0.0058373
SLC2A4	0.0020315	0.0000000	0.0011410	0.0000000	0.0000000
YBX2	0.0183511	0.0523202	0.0605593	0.0111281	0.0169203
EIF5A	0.8178855	0.8450632	0.9290735	0.8486221	0.6624570
GPS2	0.4728862	0.4878184	0.4620825	0.4282242	0.4129893
NEURL4	0.0179892	0.0457983	0.0441766	0.0454530	0.0667096
ACAP1	0.3258221	0.2349372	0.2036315	0.2747375	0.2174900
KCTD11	0.0529446	0.0381749	0.0406761	0.0598671	0.0137175
TNK1	0.0029190	0.0056771	0.0044629	0.0089980	0.0102541
PLSCR3	0.1145593	0.0859826	0.0864842	0.0815415	0.0642008
TMEM256	0.8820846	0.7508287	0.6856969	0.8322443	0.6247374
NLGN2	0.1356735	0.1980935	0.2345411	0.1881148	0.2007435
SPEM2	0.0000000	0.0014443	0.0000000	0.0000000	0.0000000
TMEM102	0.0098627	0.0096051	0.0065364	0.0157089	0.0106862
FGF11	0.2824679	0.3122817	0.2305117	0.2295973	0.2360025
ENSG00000263301	0.0000000	0.0000000	0.0000000	0.0037014	0.0000000
CHRNB1	0.2779928	0.2427052	0.2357052	0.2983908	0.1241723
ZBTB4	0.2999119	0.2688155	0.2483835	0.2505586	0.3028335
POLR2A	0.3255169	0.3322264	0.3368390	0.2877479	0.3886368
TNFSF12	0.0969879	0.0767929	0.0515865	0.1226484	0.0190085
ENSG00000276384	0.0015715	0.0007381	0.0031632	0.0033377	0.0000000
TNFSF13	0.0102510	0.0064559	0.0064074	0.0101887	0.0138036
SENP3	0.0290139	0.0452598	0.0584493	0.0327825	0.0586887

EIF4A1	1.6129817	1.6020958	1.7293959	1.6522600	1.3390856
CD68	0.0140434	0.0067097	0.0044577	0.0077693	0.0113898
ENSG00000233223	0.0380863	0.0360532	0.0383164	0.0271882	0.0456784
MPDU1	0.1024801	0.0958494	0.0981059	0.1458242	0.0630103
SOX15	0.1485356	0.1081776	0.1054069	0.1249555	0.0624704
FXR2	0.1910507	0.1903842	0.1850803	0.2050713	0.1262989
SHBG	0.0101032	0.0053083	0.0043831	0.0124595	0.0049667
SAT2	0.8744138	0.6911617	0.6377544	0.8816862	0.5507236
ATP1B2	0.7173698	0.5413962	0.4737726	0.9169188	0.5195334
TP53	0.1288317	0.1131578	0.0938417	0.1542713	0.0789850
WRAP53	0.1320272	0.0953280	0.0702486	0.1551944	0.0772195
EFNB3	2.3319742	1.7206848	1.3376934	2.4767268	1.5976373
DNAH2	0.0425438	0.0395446	0.0351260	0.0248700	0.1065815
KDM6B	0.2543552	0.2115035	0.2091738	0.1698079	0.3293959
ENSG00000288748	0.0000000	0.0000000	0.0000000	0.0000000	0.0071694
TMEM88	0.0117066	0.0064276	0.0078148	0.0175430	0.0055618
NAA38	0.8532039	0.7916106	0.7251269	0.9254275	0.6592589
CYB5D1	0.1738543	0.1129807	0.1019430	0.1652713	0.0829331
CHD3	0.5043050	0.6221994	0.6182145	0.4785782	0.6290891
RNF227	0.0578839	0.0316620	0.0263454	0.0430186	0.0339871
KCNAB3	0.0026726	0.0036242	0.0041267	0.0000000	0.0108741
TRAPPC1	0.7836305	0.8281043	1.0059115	0.8446950	0.5690067
CNTROB	0.0525554	0.0642364	0.0504689	0.0305968	0.0978929
GUCY2D	0.0143846	0.0263980	0.0269131	0.0045563	0.0115620
ALOX15B	0.0028475	0.0011251	0.0000000	0.0028893	0.0000000
ALOX12B	0.0021390	0.0013745	0.0025702	0.0022792	0.0000000
ENSG00000214999	0.0007884	0.0010802	0.0000000	0.0027011	0.0000000
ALOXE3	0.0009995	0.0010457	0.0036821	0.0024107	0.0037529
HES7	0.0092158	0.0132612	0.0122062	0.0078466	0.0107919
ENSG00000288993	0.0172627	0.0139356	0.0134722	0.0113998	0.0691027
PER1	0.0988690	0.0817120	0.0838798	0.0697243	0.1062443
VAMP2	1.7169779	2.0871287	2.4647370	1.6478819	1.6912390
TMEM107	0.6196183	0.4376841	0.3868774	0.7134403	0.3986799
ENSG00000266824	0.0015107	0.0046424	0.0012693	0.0015611	0.0000000
BORCS6	0.0455128	0.0594938	0.0544339	0.0702321	0.0592992
AURKB	0.0044426	0.0005310	0.0015464	0.0014244	0.0072786
LINC00324	0.0018353	0.0090061	0.0119239	0.0170066	0.0000000
CTC1	0.0291443	0.0181532	0.0175767	0.0185362	0.0305870
PFAS	0.0208597	0.0265063	0.0361703	0.0414381	0.0448763
ENSG00000269947	0.0042670	0.0081936	0.0048440	0.0031591	0.0074730
SLC25A35	0.0242044	0.0135294	0.0030532	0.0151728	0.0195742
RANGRF	0.2629349	0.2914833	0.3444764	0.2833926	0.1703138
ARHGEF15	0.0023629	0.0051570	0.0082853	0.0000000	0.0000000
KRBA2	0.1793639	0.1886583	0.2354276	0.1065166	0.2553631
ENSG00000265749	0.0000000	0.0000000	0.0000000	0.0000000	0.0000000
RPL26	2.8339534	2.8948132	2.7314769	2.7203457	2.6160456
ENSG00000288750	0.0007544	0.0000000	0.0000000	0.0000000	0.0000000
NDEL1	0.2138826	0.2490817	0.2966104	0.1734668	0.3285808
MYH10	0.7609420	0.8009085	0.9003000	0.7445718	1.0902340
PIK3R6	0.0000000	0.0009596	0.0009965	0.0000000	0.0000000
PIK3R5	0.0010719	0.0018708	0.0008911	0.0000000	0.0065348
NTN1	1.6163875	1.0509159	0.8038929	2.1865749	1.2232699
ENSG00000262966	0.0017230	0.0000000	0.0057863	0.0000000	0.0000000

STX8	0.4434017	0.4021770	0.3988988	0.3606259	0.4907639
ENSG00000263708	0.0097318	0.0025200	0.0046997	0.0000000	0.0277770
ENSG00000225751	0.0000000	0.0000000	0.0000000	0.0000000	0.0000000
ENSG00000271851	0.0068055	0.0108890	0.0066817	0.0082726	0.0000000
CFAP52	0.3847050	0.1901918	0.1142243	0.4333518	0.1460306
ENSG00000265349	0.0014371	0.0014195	0.0059832	0.0021678	0.0037366
USP43	0.0130237	0.0111635	0.0145248	0.0157793	0.0111835
GSG1L2	0.0030057	0.0041645	0.0019052	0.0046836	0.0000000
GLP2R	0.0000000	0.0000000	0.0000000	0.0000000	0.0000000
RCVRN	0.0000000	0.0018548	0.0000000	0.0000000	0.0000000
GAS7	0.1788469	0.2075983	0.3223601	0.1988830	0.2797771
ENSG00000214970	0.0024481	0.0023797	0.0039299	0.0000000	0.0000000
MYH3	0.0058475	0.0122797	0.0032340	0.0023827	0.0203252
SCO1	0.2359214	0.2413299	0.2699959	0.2465703	0.2148451
ADPRM	0.0771436	0.0752012	0.0675613	0.0542260	0.0728033
TMEM220	0.0147332	0.0136392	0.0012127	0.0249327	0.0098005
TMEM220-AS1	0.0135227	0.0154396	0.0121778	0.0050473	0.0279563
ENSG00000264016	0.0000000	0.0000000	0.0000000	0.0014739	0.0000000
TMEM238L	0.0036932	0.0044462	0.0023495	0.0050990	0.0050044
PIRT	0.0727550	0.0939917	0.0203917	0.0901996	0.0426384
ENSG00000263508	0.0022196	0.0046559	0.0000000	0.0033035	0.0069764
ENSG00000285541	0.0061225	0.0008274	0.0039256	0.0018030	0.0044975
SHISA6	0.1306951	0.1908395	0.2374874	0.1179044	0.4623666
ENSG00000264727	0.0000000	0.0010058	0.0000000	0.0000000	0.0000000
DNAH9	0.4575699	0.3013182	0.2301292	0.4779075	0.3635576
ENSG00000263684	0.0000000	0.0000000	0.0000000	0.0000000	0.0000000
ENSG00000266368	0.0158851	0.0114499	0.0061746	0.0017299	0.0062995
ZNF18	0.0515714	0.0477871	0.0299633	0.0489062	0.0483671
ENSG00000289910	0.0000000	0.0025243	0.0000000	0.0000000	0.0040567
MAP2K4	0.9674867	0.9555832	0.8845721	0.9092809	0.9565335
ENSG00000265400	0.0000000	0.0000000	0.0000000	0.0000000	0.0000000
MYOCD	0.0037858	0.0064858	0.0015332	0.0000000	0.0027063
ARHGAP44-AS1	0.0000000	0.0008435	0.0000000	0.0000000	0.0000000
ARHGAP44	0.0806489	0.0971099	0.1099584	0.0731552	0.3461202
ELAC2	0.1687454	0.1459056	0.1216527	0.1783827	0.2055644
HS3ST3A1	0.0324029	0.0312433	0.0146727	0.0356661	0.0594846
COX10-DT	0.1095637	0.1397480	0.1249110	0.0797751	0.2830815
ENSG00000266744	0.0000000	0.0004334	0.0021269	0.0000000	0.0000000
COX10	0.1751857	0.1798298	0.1990126	0.1581742	0.2699581
HS3ST3B1	0.1082891	0.0800396	0.0461081	0.0709345	0.0848294
ENSG00000266709	0.0035676	0.0062403	0.0022570	0.0025788	0.0149151
ENSG00000266378	0.0049184	0.0000000	0.0000000	0.0000000	0.0044438
ENSG00000230647	0.0034705	0.0000000	0.0022858	0.0015092	0.0212194
PMP22	0.9286030	0.5713663	0.3445417	1.0377815	0.4785699
TEKT3	0.0108404	0.0053549	0.0000000	0.0011582	0.0233794
ENSG00000266667	0.0070026	0.0082206	0.0126644	0.0016235	0.0410602
CDRT4	0.0833987	0.0732899	0.1114865	0.0704336	0.1770452
TVP23C	0.1364825	0.1389486	0.1573462	0.1247026	0.4010268
ENSG00000266538	0.0018903	0.0030076	0.0000000	0.0034755	0.0123179
FBXW10B	0.0031665	0.0036116	0.0000000	0.0000000	0.0199710
TRIM16	0.3198307	0.3130236	0.2421453	0.4484718	0.2931385
ZNF286A	0.1671862	0.2285746	0.2602799	0.1393389	0.1933658
TBC1D26	0.0043449	0.0028258	0.0000000	0.0000000	0.0042185

ENSG00000265519	0.0024612	0.0019077	0.0009236	0.0000000	0.0000000
ENSG00000276855	0.0329756	0.0498798	0.0254745	0.0275582	0.0220027
LINC02087	0.0021425	0.0000000	0.0000000	0.0000000	0.0014245
ADORA2B	0.0587962	0.0414585	0.0247129	0.0385221	0.0710116
ZSWIM7	0.4676054	0.5065714	0.5127234	0.5024646	0.3538842
TTC19	0.4701423	0.4529470	0.5539899	0.5062719	0.5164419
ENSG00000275413	0.0012886	0.0013354	0.0000000	0.0000000	0.0031427
NCOR1	0.9127227	0.9306250	1.0455820	0.9154473	1.0661405
ENSG00000227782	0.0000000	0.0040421	0.0000000	0.0000000	0.0026927
PIGL	0.1190125	0.1366397	0.1547285	0.1344663	0.2712834
CENPV	0.4420635	0.6125725	0.7922993	0.4073901	0.5297322
UBB	1.8326361	1.8717984	1.9355051	1.8987034	1.5796931
ENSG00000265401	0.0080740	0.0061920	0.0096787	0.0087292	0.0047575
TRPV2	0.0131712	0.0226262	0.0226884	0.0251999	0.0264000
SNHG29	2.0925331	2.2737868	2.1772069	2.0263857	2.0368287
LRRC75A	0.3239005	0.3113578	0.3411058	0.3079759	0.4540451
ENSG00000286743	0.0102561	0.0055029	0.0079255	0.0113273	0.0000000
ZNF287	0.1111753	0.1015392	0.0912837	0.1090825	0.1317262
ZNF624	0.1067775	0.0900667	0.1162872	0.1454716	0.0794829
CCDC144A	0.0088199	0.0255940	0.0138239	0.0170323	0.0692910
ENSG00000290976	0.0007250	0.0008758	0.0000000	0.0000000	0.0000000
ENSG00000287910	0.0109777	0.0211400	0.0250143	0.0168888	0.0078163
MPRIP	0.4367060	0.5721900	0.6668417	0.4911333	0.7862827
MPRIP-AS1	0.0002255	0.0000000	0.0000000	0.0000000	0.0000000
ENSG00000263624	0.0016080	0.0000000	0.0018045	0.0000000	0.0165902
PLD6	0.0194807	0.0231606	0.0106741	0.0146420	0.0122412
FLCN	0.0979636	0.0868404	0.0463197	0.0729899	0.1221540
ENSG00000266498	0.0003288	0.0000000	0.0000000	0.0000000	0.0000000
COPS3	0.4882371	0.5521588	0.6197268	0.4652625	0.4108360
NT5M	0.0945582	0.1340464	0.1438213	0.0823377	0.1139033
MED9	0.1078496	0.0954831	0.1057274	0.1190634	0.1014883
RASD1	0.2191343	0.1105814	0.1020318	0.2145453	0.0692184
PEMT	0.2321461	0.1912786	0.1794038	0.2681408	0.2014450
ENSG00000264666	0.0137597	0.0132564	0.0183646	0.0057548	0.0213474
SMCR2	0.0034762	0.0026326	0.0023283	0.0019475	0.0000000
RAI1	0.1379840	0.1390838	0.1507330	0.1659922	0.4327258
RAI1-AS1	0.0009517	0.0000000	0.0000000	0.0000000	0.0000000
SMCR5	0.0036663	0.0000000	0.0023088	0.0000000	0.0077664
SREBF1	0.0565051	0.0294211	0.0287101	0.0857230	0.0639079
TOM1L2	0.1298317	0.1381994	0.1294219	0.0663034	0.2662089
ENSG00000197815	0.0000000	0.0000000	0.0000000	0.0000000	0.0044909
DRC3	0.0978405	0.0550070	0.0539514	0.1118778	0.0818585
ATPAF2	0.1764780	0.1933632	0.1545726	0.1883767	0.2056077
GID4	0.1609092	0.1517257	0.1586279	0.1444549	0.1397766
DRG2	0.2908143	0.2014067	0.2072415	0.3026201	0.1462624
MYO15A	0.0042647	0.0068387	0.0047320	0.0139786	0.0214753
ENSG00000266677	0.0040515	0.0079977	0.0000000	0.0040191	0.0055061
ALKBH5	0.7252344	0.7049937	0.7360035	0.6860236	0.5044821
LLGL1	0.1018493	0.0557715	0.0486936	0.1301080	0.0567903
FLII	0.2481696	0.2181601	0.1949929	0.2379284	0.2359880
MIEF2	0.0850664	0.0583441	0.0578615	0.0803943	0.0730284
ENSG00000260647	0.0034649	0.0031912	0.0000000	0.0000000	0.0000000
TOP3A	0.0640167	0.0787515	0.0628237	0.0796398	0.1110031

SMCR8	0.0473745	0.0362265	0.0560376	0.0252631	0.0566304
SHMT1	0.0278093	0.0157942	0.0154006	0.0124808	0.0376860
LINC02076	0.0205139	0.0125359	0.0100302	0.0059616	0.0259846
FAM106A	0.0003449	0.0000000	0.0000000	0.0000000	0.0000000
ENSG00000290659	0.0000000	0.0008298	0.0000000	0.0010138	0.0000000
CCDC144BP	0.0093636	0.0123242	0.0072691	0.0045838	0.0368382
ZNF286B	0.0263408	0.0736943	0.0742942	0.0436505	0.0368261
FOXO3B	0.0298106	0.0323950	0.0247254	0.0306608	0.0423665
TRIM16L	0.1744330	0.1187023	0.0934441	0.1885182	0.1367637
FBXW10	0.0017863	0.0000000	0.0000000	0.0000000	0.0000000
TVP23B	0.1188281	0.0718464	0.0906172	0.1149418	0.0794669
ENSG00000265478	0.0019354	0.0000000	0.0024718	0.0000000	0.0097123
PRPSAP2	0.4271023	0.4031027	0.4257782	0.3472247	0.3889479
ENSG00000289213	0.0080695	0.0101357	0.0088292	0.0041980	0.0000000
SLC5A10	0.0000000	0.0000000	0.0000000	0.0000000	0.0000000
FAM83G	0.0067659	0.0078258	0.0034073	0.0119150	0.0075884
GRAP	0.0010222	0.0010426	0.0000000	0.0000000	0.0000000
ENSG00000228157	0.0000000	0.0015927	0.0000000	0.0027576	0.0000000
ENSG00000262202	0.0339418	0.0246469	0.0158304	0.0154908	0.0445935
EPN2	0.6230745	0.6321883	0.7302740	0.6270535	0.6498082
EPN2-AS1	0.0007324	0.0000000	0.0000000	0.0000000	0.0013481
B9D1	0.3107554	0.2298117	0.2848638	0.2759498	0.2160947
ENSG00000286799	0.0000000	0.0000000	0.0000000	0.0000000	0.0000000
MAPK7	0.1228948	0.0955407	0.1040968	0.0977460	0.0774239
MFAP4	0.3674247	0.1958989	0.1293137	0.3734007	0.2043281
RNF112	0.0129277	0.0299027	0.0387247	0.0116909	0.0130152
ENSG00000235979	0.0917682	0.0818012	0.0771233	0.0865621	0.0904237
LINC02094	0.0013569	0.0000000	0.0021479	0.0016217	0.0000000
SLC47A1	0.0032102	0.0000000	0.0019622	0.0000000	0.0098699
ENSG00000262769	0.0000000	0.0007412	0.0000000	0.0000000	0.0049754
ENSG00000290454	0.0000000	0.0000000	0.0000000	0.0009264	0.0000000
ALDH3A2	0.3585368	0.3885209	0.4341722	0.3247159	0.3004807
SLC47A2	0.0045454	0.0007880	0.0011486	0.0135527	0.0246853
ENSG00000262681	0.0000000	0.0000000	0.0000000	0.0000000	0.0000000
ALDH3A1	0.0000000	0.0012869	0.0010558	0.0028315	0.0055705
ULK2	0.3628991	0.4660236	0.5255971	0.3333350	0.5440208
ENSG00000276406	0.0000000	0.0054146	0.0018497	0.0018389	0.0000000
ENSG00000270091	0.0010222	0.0040912	0.0033130	0.0000000	0.0012623
AKAP10	0.1655108	0.1641136	0.1547388	0.1788557	0.2328811
SPECC1-DT	0.0115413	0.0342351	0.0118048	0.0000000	0.0198111
SPECC1	0.4426852	0.4251977	0.4239105	0.4269913	0.5113140
ENSG00000263494	0.0018275	0.0018460	0.0036236	0.0058641	0.0012783
ENSG00000266839	0.0000000	0.0000000	0.0000000	0.0000000	0.0031863
ENSG00000291194	0.0193976	0.0143946	0.0152678	0.0205365	0.0544492
ENSG00000291195	0.0000000	0.0009649	0.0000000	0.0000000	0.0000000
ENSG00000260907	0.0000000	0.0013541	0.0027173	0.0022544	0.0139649
CCDC144NL	0.0000000	0.0010598	0.0000000	0.0000000	0.0072574
CCDC144NL-AS1	0.0272190	0.0174381	0.0228671	0.0071875	0.0808519
USP22	0.9389518	1.1056075	1.2220508	0.8898365	1.0249815
ENSG00000263986	0.0082138	0.0136188	0.0117923	0.0067623	0.0265922
LINC01563	0.0273409	0.0126525	0.0000000	0.0402378	0.0046038
DHRS7B	0.1154464	0.0896171	0.1194626	0.1557093	0.1534351
TMEM11	0.2719553	0.2518558	0.2302456	0.2532167	0.1866430

TMEM11-DT	0.0019205	0.0058061	0.0073820	0.0029052	0.0424087
NATD1	0.0487091	0.0377205	0.0333546	0.0295049	0.0261902
ENSG00000289453	0.0000000	0.0035999	0.0000000	0.0048600	0.0000000
MAP2K3	0.0588152	0.0885182	0.0946300	0.0345753	0.0605473
KCNJ12	0.0243621	0.0487073	0.0427894	0.0036915	0.0642562
LINC02693	0.1213881	0.1536896	0.1620672	0.1119363	0.1198339
ENSG00000287671	0.0000000	0.0013141	0.0000000	0.0000000	0.0000000
UBBP4	0.0067708	0.0044509	0.0035138	0.0098153	0.0061591
ENSG00000264956	0.0008766	0.0015739	0.0071809	0.0000000	0.0086970
WSB1	1.0657140	1.0583903	0.8475361	0.8906105	1.6376886
ENSG00000266313	0.0192899	0.0174534	0.0103887	0.0213723	0.0059760
KSR1	0.0881652	0.1241980	0.0888213	0.1018557	0.2810544
ENSG00000265801	0.0014357	0.0014812	0.0019069	0.0000000	0.0080969
ENSG00000266872	0.0031761	0.0006723	0.0000000	0.0000000	0.0044278
LGALS9	0.0077502	0.0016164	0.0009712	0.0033167	0.0083114
NOS2	0.0037344	0.0064822	0.0012032	0.0000000	0.0000000
ENSG00000266527	0.0000000	0.0000000	0.0000000	0.0000000	0.0000000
LYRM9	0.1008709	0.1016479	0.0964603	0.1160957	0.1291153
NLK	0.2259735	0.3514516	0.3182308	0.1584047	0.5335473
ENSG00000288923	0.0000000	0.0000000	0.0036390	0.0011930	0.0087101
ENSG00000287721	0.0010043	0.0043542	0.0007485	0.0000000	0.0174391
PYY2	0.0027447	0.0019391	0.0067396	0.0000000	0.0000000
ENSG00000260777	0.0024874	0.0019011	0.0013732	0.0000000	0.0048214
TMEM97	0.1579679	0.2006676	0.1772810	0.2276412	0.1861210
IFT20	0.6462278	0.6699099	0.6585035	0.6288189	0.5706650
TNFAIP1	0.3019728	0.2580375	0.2444021	0.3229382	0.2448652
POLDIP2	0.3172768	0.3080118	0.3304053	0.3194530	0.2212431
TMEM199	0.1288313	0.1152402	0.1474261	0.1158737	0.0927209
ENSG00000265618	0.0019707	0.0062616	0.0000000	0.0018419	0.0000000
SARM1	0.0647175	0.0912216	0.0722626	0.0368838	0.1653768
VTN	0.0163246	0.0085126	0.0021159	0.0135356	0.0124349
ENSG00000277450	0.0124287	0.0112568	0.0112831	0.0012911	0.0205344
SLC46A1	0.0078302	0.0131715	0.0278652	0.0068479	0.0184572
ENSG00000265254	0.0081620	0.0066750	0.0016044	0.0036873	0.0000000
RSKR	0.0276725	0.0396661	0.0316145	0.0220480	0.1045029
UNC119	0.3967982	0.4169784	0.5545004	0.4158555	0.3038656
PIGS	0.3602929	0.2807181	0.2796288	0.5461607	0.1623987
ALDOC	0.3193154	0.6489166	0.7588092	0.2845568	0.4493689
ENSG00000265168	0.0015603	0.0000000	0.0019820	0.0000000	0.0000000
SPAG5	0.0061422	0.0067598	0.0032005	0.0009264	0.0146899
SPAG5-AS1	0.0017181	0.0014613	0.0000000	0.0000000	0.0064205
ENSG00000265287	0.0076045	0.0058493	0.0062793	0.0019173	0.0334097
BLTP2	0.2533388	0.1945012	0.1927908	0.2351445	0.3115176
SDF2	0.3066519	0.2540712	0.2100906	0.4686186	0.2234055
SUPT6H	0.2926652	0.3351915	0.2204487	0.2414849	0.3286138
PROCA1	0.0594217	0.0713483	0.0635875	0.0746273	0.0782833
RAB34	0.1613998	0.1116838	0.0670023	0.1666866	0.0586944
RPL23A	2.4979003	2.5084071	2.3816753	2.4104872	2.2759850
ENSG00000264577	0.0086674	0.0094804	0.0105468	0.0044797	0.0000000
TLCD1	0.0481814	0.0244877	0.0152756	0.0474703	0.0242774
NEK8	0.0170965	0.0109254	0.0094738	0.0232645	0.0226366
ENSG00000265073	0.0006781	0.0000000	0.0000000	0.0000000	0.0037844
TRAF4	0.3350560	0.3732957	0.2722045	0.3579067	0.2448668

FAM222B	0.2128515	0.2867432	0.2779692	0.2199866	0.4625279
ERAL1	0.1455294	0.1381398	0.1486327	0.1442334	0.1384366
FLOT2	0.1713269	0.2089745	0.1717791	0.2066869	0.2651685
ENSG00000264304	0.0000000	0.0000000	0.0000000	0.0000000	0.0000000
ENSG00000266642	0.0002224	0.0000000	0.0000000	0.0016996	0.0000000
DHRS13	0.0802392	0.1085051	0.1568237	0.0659561	0.0938945
PHF12	0.2061010	0.1583089	0.1613183	0.1957495	0.1800062
ENSG00000265845	0.0007596	0.0017972	0.0000000	0.0000000	0.0082469
PIPOX	0.0976057	0.0557167	0.0761004	0.1161997	0.0754629
SEZ6	0.0447780	0.1037720	0.1372668	0.0403068	0.1981755
MYO18A	0.1582688	0.2298640	0.2404193	0.1357437	0.3496430
ENSG00000263709	0.0006160	0.0000000	0.0000000	0.0000000	0.0069189
NUFIP2	0.7173474	0.6952905	0.7200754	0.6415297	0.6777421
ENSG00000264808	0.0187617	0.0096761	0.0101399	0.0050143	0.0190721
ENSG00000266111	0.0076259	0.0029925	0.0020494	0.0000000	0.0067193
TAOK1	0.7886626	0.9105306	0.9695175	0.7074064	1.0412482
ABHD15	0.0275806	0.0125272	0.0141976	0.0310060	0.0036662
ABHD15-AS1	0.0075725	0.0040976	0.0029020	0.0040371	0.0156631
TP53I13	0.3818779	0.2048938	0.1552515	0.4738554	0.2003210
ENSG00000264290	0.0000000	0.0000000	0.0023139	0.0000000	0.0025600
GIT1	0.2036861	0.3040891	0.3448864	0.1614681	0.2596531
ANKRD13B	0.0362879	0.0506457	0.0642172	0.0487264	0.0819608
CORO6	0.0034143	0.0100708	0.0097740	0.0060420	0.0218386
ENSG00000264007	0.0000000	0.0000000	0.0000000	0.0000000	0.0000000
SSH2	0.2290278	0.3657863	0.3998484	0.1740177	0.7573400
ENSG00000265625	0.0017324	0.0000000	0.0000000	0.0093325	0.0000000
ENSG00000263657	0.0019652	0.0003548	0.0064142	0.0000000	0.0351846
ENSG00000263477	0.0000000	0.0008723	0.0000000	0.0000000	0.0070255
EFCAB5	0.0261548	0.0365733	0.0255421	0.0100494	0.0462536
ENSG00000265394	0.0022330	0.0022165	0.0014741	0.0000000	0.0176948
NSRP1	0.6633040	0.4992914	0.4431141	0.5499270	0.5366106
ENSG00000265739	0.0010743	0.0000000	0.0000000	0.0000000	0.0027405
ENSG00000266987	0.0000000	0.0000000	0.0000000	0.0000000	0.0037811
SLC6A4	0.0011170	0.0030575	0.0006102	0.0000000	0.0064222
ENSG00000264125	0.0000000	0.0008262	0.0000000	0.0057216	0.0044531
ENSG00000266120	0.0000000	0.0000000	0.0000000	0.0000000	0.0000000
BLMH	0.2563186	0.2522848	0.2337275	0.2675859	0.1813239
TMIGD1	0.0037417	0.0000000	0.0000000	0.0000000	0.0000000
CPD	0.4269695	0.2925298	0.2789596	0.6077935	0.3061574
GOSR1	0.5293993	0.4990265	0.4544025	0.5067845	0.3945124
ENSG00000290404	0.0000000	0.0009937	0.0000000	0.0000000	0.0000000
ENSG00000214719	0.0442699	0.0549618	0.0386799	0.0404553	0.0774490
ENSG00000290395	0.0032910	0.0000000	0.0033800	0.0043864	0.0000000
ENSG00000290450	0.1163619	0.1113564	0.1222325	0.0860391	0.0722313
ENSG00000290928	0.3050532	0.4323938	0.4435460	0.2747426	0.7584471
ENSG00000263603	0.0000000	0.0017341	0.0019419	0.0000000	0.0015201
CRLF3	0.0469487	0.0595532	0.0533082	0.0648702	0.1981702
ENSG00000265791	0.0000000	0.0000000	0.0000000	0.0000000	0.0000000
ENSG00000266490	0.0070031	0.0095838	0.0014782	0.0000000	0.0101434
ENSG00000276250	0.0054146	0.0084234	0.0152829	0.0027087	0.0021288
ATAD5	0.0535343	0.0533286	0.0526557	0.0445923	0.1153231
ENSG00000265334	0.0067768	0.0065436	0.0026613	0.0018314	0.0315413
ENSG00000263531	0.0018735	0.0011769	0.0078848	0.0025926	0.0029771

TEFM	0.0933168	0.1209405	0.0951209	0.1189162	0.1312521
ENSG00000275185	0.0006385	0.0031791	0.0010318	0.0116267	0.0016619
ADAP2	0.0265308	0.0266488	0.0348313	0.0215458	0.0708622
RNF135	0.0147476	0.0127265	0.0011671	0.0189401	0.0077880
ENSG00000264456	0.0000000	0.0025891	0.0032012	0.0000000	0.0000000
ENSG00000266340	0.0057492	0.0039310	0.0000000	0.0029154	0.0056757
ENSG00000291063	0.1015063	0.0838400	0.0753047	0.1130591	0.2272166
ENSG00000290858	0.0007677	0.0000000	0.0000000	0.0000000	0.0000000
MIR4733HG	0.0682451	0.0391745	0.0310188	0.0572856	0.0411648
NF1	0.6544306	0.6955765	0.7128714	0.5775849	1.1247061
ENSG00000266371	0.0050946	0.0000000	0.0000000	0.0000000	0.0014580
OMG	0.0161736	0.0196578	0.0445703	0.0269491	0.0609632
EVI2B	0.0014591	0.0000000	0.0038818	0.0000000	0.0081765
EVI2A	0.0000000	0.0000000	0.0000000	0.0000000	0.0000000
RAB11FIP4	0.2255341	0.4156169	0.4026557	0.2000109	0.4699217
ENSG00000266877	0.0085199	0.0070004	0.0065546	0.0035261	0.0214398
COPRS	0.8763838	0.7717575	0.8743254	0.8095579	0.5818194
UTP6	0.2350835	0.2198445	0.1640518	0.2304114	0.2240845
ENSG00000263990	0.0035321	0.0011842	0.0022744	0.0119498	0.0058440
SUZ12	0.4914276	0.4704548	0.4932761	0.4377324	0.5538646
LRRC37B	0.0783863	0.1120017	0.1612883	0.1039086	0.0980866
ENSG00000290975	0.0051131	0.0025556	0.0070431	0.0000000	0.0070919
ENSG00000277511	0.0026444	0.0140100	0.0053630	0.0117523	0.0028917
ENSG00000214708	0.0062515	0.0055547	0.0011420	0.0000000	0.0000000
RHOT1	0.5348881	0.5067417	0.6233246	0.4955485	0.5289753
ENSG00000287506	0.0000000	0.0000000	0.0000000	0.0015408	0.0000000
RHBDL3	0.0319614	0.0228211	0.0299835	0.0456486	0.0323994
C17orf75	0.3647324	0.4432991	0.5558734	0.3346834	0.3333572
ENSG00000265139	0.0012439	0.0000000	0.0000000	0.0018314	0.0063839
ZNF207	0.4615187	0.3919078	0.5144461	0.4685633	0.5536486
ENSG00000274341	0.0033216	0.0071343	0.0011445	0.0076844	0.0092560
ENSG00000278668	0.0006041	0.0041626	0.0000000	0.0000000	0.0000000
ENSG00000266385	0.0021265	0.0044303	0.0025618	0.0000000	0.0233623
ENSG00000279762	0.0030998	0.0023715	0.0000000	0.0000000	0.0113282
ENSG00000264083	0.0024502	0.0011280	0.0000000	0.0000000	0.0081358
PSMD11	0.7644628	0.6805242	0.7008846	0.8146333	0.6342047
CDK5R1	0.2789851	0.5439199	0.8066626	0.2385824	0.4968319
MYO1D	0.1013673	0.1391444	0.0947337	0.0618736	0.5615506
ENSG00000266718	0.0000000	0.0000000	0.0000000	0.0000000	0.0000000
ENSG00000266599	0.0019653	0.0032583	0.0041056	0.0000000	0.0142133
ENSG00000264458	0.0069161	0.0042948	0.0000000	0.0000000	0.0090597
ENSG00000236377	0.0039264	0.0025058	0.0100803	0.0000000	0.0042647
TMEM98	0.3587660	0.2750702	0.1744319	0.4660955	0.2137960
SPACA3	0.0000000	0.0000000	0.0000000	0.0000000	0.0095540
ASIC2	0.2194856	0.2790537	0.2242554	0.1792324	0.6679978
ENSG00000266535	0.0000000	0.0000000	0.0000000	0.0000000	0.0000000
ENSG00000265125	0.0011041	0.0000000	0.0000000	0.0000000	0.0000000
ENSG00000283417	0.0000000	0.0000000	0.0000000	0.0000000	0.0000000
ENSG00000279668	0.0162624	0.0057815	0.0029481	0.0201498	0.0425242
ENSG00000265775	0.0000000	0.0011050	0.0024168	0.0000000	0.0019899
ENSG00000263571	0.0098504	0.0133629	0.0119339	0.0199298	0.0088315
LINC01989	0.0000000	0.0008116	0.0000000	0.0037958	0.0000000
CCL2	0.0512090	0.0541536	0.0393170	0.2548750	0.0672578

TMEM132E-DT	0.0017996	0.0089283	0.0232366	0.0000000	0.0015072
TMEM132E	0.0412125	0.0668298	0.0695088	0.0446343	0.0961278
CCT6B	0.0354622	0.0438159	0.0142310	0.0244040	0.0477255
ZNF830	0.1949309	0.2174327	0.1869932	0.1826442	0.1681748
LIG3	0.2021311	0.1733659	0.1957425	0.2053569	0.2282532
RFFL	0.1450661	0.1170529	0.1188054	0.1085437	0.2371635
ENSG00000273687	0.0008421	0.0000000	0.0041683	0.0000000	0.0000000
RAD51D	0.0801116	0.0905510	0.1207725	0.0583008	0.1037712
FNDC8	0.0000000	0.0000000	0.0021865	0.0000000	0.0000000
NLE1	0.0349206	0.0482766	0.0503761	0.0358020	0.0339721
ENSG00000266947	0.0409732	0.0345027	0.0325093	0.0734989	0.0396161
SLFN5	0.2543647	0.1948360	0.1817788	0.1726343	0.1474771
SLFN11	0.0011825	0.0008378	0.0000000	0.0000000	0.0000000
ENSG00000267711	0.0000000	0.0000000	0.0000000	0.0064439	0.0000000
SLFN12	0.0366518	0.0190320	0.0111847	0.0616394	0.0150626
SLFN13	0.0000000	0.0000000	0.0000000	0.0000000	0.0062037
SLFN14	0.0000000	0.0003922	0.0000000	0.0000000	0.0000000
SNHG30	0.1694501	0.2029554	0.2311673	0.1708530	0.1263351
PEX12	0.0878763	0.0586637	0.0479407	0.0843334	0.0368092
AP2B1	1.1338510	1.2271594	1.3398541	1.0569157	1.0988656
RASL10B	0.1617389	0.1782360	0.1374051	0.1149436	0.1337630
GAS2L2	0.0037484	0.0009132	0.0000000	0.0112220	0.0117958
MMP28	0.0083934	0.0163550	0.0131783	0.0223101	0.0312184
ENSG00000271392	0.0022227	0.0011481	0.0000000	0.0023258	0.0015176
C17orf50	0.0010469	0.0014708	0.0021926	0.0011622	0.0016664
TAF15	0.8418724	0.7829080	0.7514213	0.7583840	0.8377912
ENSG00000270871	0.0026714	0.0029770	0.0008289	0.0000000	0.0127808
ENSG00000270894	0.0000000	0.0000000	0.0000000	0.0000000	0.0034957
HEATR9	0.0027336	0.0000000	0.0000000	0.0000000	0.0000000
ENSG00000270240	0.0000000	0.0000000	0.0000000	0.0000000	0.0015176
RDM1	0.0212190	0.0186253	0.0158109	0.0097119	0.0118702
CCL14	0.0039576	0.0015753	0.0019820	0.0000000	0.0000000
CCL15	0.0168164	0.0180103	0.0046866	0.0060925	0.0027595
CCL3	0.0008167	0.0000000	0.0000000	0.0000000	0.0000000
ENSG00000274767	0.0081940	0.0126001	0.0038508	0.0054946	0.0032840
CCL3L3	0.0016704	0.0000000	0.0000000	0.0000000	0.0000000
TBC1D3I	0.0008886	0.0024113	0.0000000	0.0041335	0.0000000
ENSG00000289011	0.0000000	0.0000000	0.0000000	0.0000000	0.0000000
ZNHIT3	0.5677971	0.6515618	0.7161282	0.5270788	0.4471365
MYO19	0.0398740	0.0473939	0.0531636	0.0367694	0.1350060
PIGW	0.0130978	0.0221646	0.0163343	0.0278089	0.0273107
GGNBP2	0.5836637	0.5781007	0.5385132	0.5651372	0.5593866
DHRS11	0.0910034	0.0934424	0.1080877	0.0809299	0.0845299
MRM1	0.0125453	0.0139521	0.0098740	0.0158467	0.0093090
ENSG00000275720	0.0000000	0.0000000	0.0016423	0.0010830	0.0000000
ENSG00000275613	0.0014193	0.0029150	0.0000000	0.0108620	0.0051430
LHX1-DT	0.0076097	0.0065797	0.0225838	0.0000000	0.0273580
LHX1	0.0071966	0.0075559	0.0114096	0.0097073	0.0108898
AATF	0.2085087	0.1881096	0.2035044	0.1729013	0.1824465
ENSG00000277589	0.0000000	0.0000000	0.0000000	0.0000000	0.0055309
ENSG00000278638	0.0000000	0.0000000	0.0000000	0.0000000	0.0037529
ACACA	0.2820170	0.3343810	0.3732575	0.2581105	0.6572192
C17orf78	0.0000000	0.0000000	0.0000000	0.0000000	0.0054421

TADA2A	0.1482701	0.1842607	0.1384805	0.1499146	0.2331366
ENSG00000289110	0.0004846	0.0000000	0.0000000	0.0000000	0.0000000
DUSP14	0.4527488	0.2787940	0.1595405	0.3699753	0.2041760
SYNRG	0.3932950	0.4695923	0.5795887	0.4058631	0.4743741
ENSG00000277688	0.0000000	0.0014737	0.0000000	0.0000000	0.0000000
DDX52	0.3683955	0.3983514	0.3532473	0.4195844	0.3139301
ENSG00000277501	0.0019527	0.0000000	0.0035528	0.0000000	0.0042946
HNF1B	0.0103659	0.0115910	0.0056262	0.0027627	0.0054768
YWHAEP7	0.0008034	0.0011596	0.0000000	0.0000000	0.0000000
TBC1D3L	0.0045635	0.0000000	0.0034286	0.0000000	0.0049696
NPEPPSP1	0.0748771	0.0859599	0.0605803	0.0844672	0.1387461
MRPL45	0.3056699	0.2723768	0.2828490	0.2686738	0.2509284
GPR179	0.0013563	0.0060140	0.0008578	0.0000000	0.0060772
SOCS7	0.1926417	0.2540151	0.3314326	0.1805944	0.2691462
ARHGAP23	0.0843998	0.1346406	0.1472189	0.0647767	0.1854009
SRCIN1	0.2011204	0.3445275	0.4367029	0.1934633	0.5532781
ENSG00000274996	0.0000000	0.0000000	0.0000000	0.0000000	0.0000000
EPOP	0.0045734	0.0088093	0.0044594	0.0027265	0.0041291
ENSG00000277969	0.0357036	0.0305152	0.0238609	0.0592519	0.0058721
ENSG00000275532	0.0018331	0.0013966	0.0023986	0.0047392	0.0000000
MLLT6	0.0801105	0.0979900	0.0725420	0.0650480	0.1959725
CISD3	0.2347191	0.2395209	0.2692404	0.2266069	0.1885346
PCGF2	0.3982550	0.3945211	0.5247156	0.3917171	0.4009235
ENSG00000277182	0.0151591	0.0331640	0.0490226	0.0217999	0.0120023
PSMB3	0.8263296	0.8626663	1.0079260	0.7742656	0.6967500
PIP4K2B	0.3539367	0.5606966	0.6584340	0.2701889	0.3825794
CWC25	0.3126041	0.2136039	0.1737438	0.3460443	0.2872244
C17orf98	0.0031240	0.0015295	0.0013476	0.0000000	0.0034576
RPL23	2.8484606	2.7582626	2.5521869	2.7373269	2.4970355
LASP1	0.3552297	0.3372820	0.4802400	0.3277165	0.3260276
ENSG00000265784	0.0006609	0.0043790	0.0012102	0.0000000	0.0047864
LASP1NB	0.1536372	0.2232597	0.2896795	0.1494027	0.1886527
FBXO47	0.0011594	0.0000000	0.0000000	0.0000000	0.0000000
ENSG00000266588	0.0002281	0.0048645	0.0010176	0.0049427	0.0000000
PLXDC1	0.0232952	0.0426375	0.0544032	0.0117409	0.0718901
ENSG00000286443	0.0041585	0.0025909	0.0000000	0.0120615	0.0000000
ARL5C	0.0008176	0.0017020	0.0008618	0.0000000	0.0000000
CACNB1	0.0734823	0.1591754	0.1944875	0.0481093	0.1637650
RPL19	2.7503677	2.7079686	2.5482942	2.6513444	2.5038297
STAC2	0.0096575	0.0245105	0.0098765	0.0096710	0.0131598
FBXL20	0.1957934	0.2451013	0.2434489	0.1747154	0.4661890
ENSG00000266469	0.0184866	0.0120880	0.0064661	0.0023988	0.0315620
MED1	0.2690884	0.2380825	0.2415241	0.2274624	0.2599277
CDK12	0.2369444	0.1808316	0.2328105	0.2235870	0.4300513
NEUROD2	0.0000000	0.0000000	0.0000000	0.0000000	0.0063839
ENSG00000214546	0.0000000	0.0000000	0.0082108	0.0000000	0.0027197
PPP1R1B	0.0373436	0.0254607	0.0109161	0.0177675	0.0280910
STARD3	0.1986943	0.2038249	0.1944252	0.2558475	0.2262416
TCAP	0.0037530	0.0096374	0.0035407	0.0039642	0.0107396
PNMT	0.0080607	0.0123432	0.0085160	0.0156940	0.0071058
PGAP3	0.0601192	0.0371563	0.0362106	0.0704160	0.0609644
ERBB2	0.1715377	0.0794675	0.0574518	0.2136000	0.0819827
MIEN1	0.5555645	0.6382314	0.6125173	0.5934915	0.5171558

GRB7	0.0000000	0.0000000	0.0000000	0.0000000	0.0000000
IKZF3	0.0006777	0.0000000	0.0000000	0.0039347	0.0021172
GSDMB	0.0089033	0.0062425	0.0150683	0.0052573	0.0467957
ORMDL3	0.1688358	0.1333554	0.1072411	0.2094775	0.1150480
GSDMA	0.0000000	0.0011221	0.0000000	0.0021382	0.0049812
PSMD3	0.5400560	0.4658347	0.5199651	0.5348778	0.4150702
MED24	0.1880679	0.2234910	0.2401177	0.1535593	0.2014317
THRA	1.0272726	1.0204575	1.0490598	0.9463000	0.8336759
NR1D1	0.0496214	0.0148344	0.0155825	0.0317396	0.0259491
MSL1	0.4487946	0.5132517	0.5604817	0.3239367	0.4389103
CASC3	0.5255687	0.5526244	0.5693835	0.4248022	0.5993994
RAPGEFL1	0.0288656	0.0453870	0.0593408	0.0103768	0.0702375
WIPF2	0.2176342	0.1785104	0.1752228	0.1741116	0.2820532
CDC6	0.0164630	0.0163149	0.0054426	0.0273283	0.0129677
RARA	0.1699208	0.1453393	0.1135643	0.1817422	0.1316245
ENSG00000287644	0.0000000	0.0000000	0.0014297	0.0000000	0.0000000
RARA-AS1	0.0687232	0.0629806	0.0751534	0.0717038	0.0623532
GJD3	0.0017685	0.0044818	0.0068040	0.0000000	0.0030832
TOP2A	0.0263078	0.0495487	0.0355871	0.0198347	0.0653703
IGFBP4	0.5609362	0.3699434	0.2672059	0.8222910	0.3381758
SMARCE1	1.1414380	1.0498574	1.1814391	1.0617566	0.8785620
ENSG00000278834	0.0517015	0.0305688	0.0469696	0.0509060	0.0217359
KRT222	0.0766088	0.1942106	0.2818521	0.0655368	0.1322355
KRT10	1.1368166	1.0577695	1.0848531	1.1143824	0.8090156
KRT10-AS1	0.1868153	0.1570486	0.1446080	0.1490462	0.1422987
ENSG00000265359	0.0000000	0.0000000	0.0012847	0.0000000	0.0000000
ENSG00000234477	0.0008967	0.0056343	0.0018077	0.0022936	0.0085476
KRT19	0.1984799	0.1554507	0.1147399	0.1244924	0.1343978
KRT17	0.0028222	0.0000000	0.0015905	0.0000000	0.0039411
EIF1	2.9353810	2.8384253	2.7811135	2.7689411	2.5546668
HAP1	0.0611194	0.1945424	0.2137144	0.0374227	0.1200174
JUP	0.1157641	0.1434608	0.1492895	0.0948498	0.1664066
P3H4	0.4219064	0.2320144	0.1971506	0.6046858	0.2221533
FKBP10	0.3711652	0.2014383	0.1261504	0.5030632	0.1717648
NT5C3B	0.5552572	0.6858235	0.8223388	0.4991540	0.5180442
KLHL10	0.0000000	0.0017155	0.0000000	0.0000000	0.0053181
KLHL11	0.0609522	0.0761870	0.0890408	0.0850290	0.1038562
ACLY	0.3042332	0.3510321	0.4038267	0.2809695	0.3005741
ODAD4	0.1221279	0.0901267	0.0689358	0.0994820	0.1393258
CNP	0.2902652	0.2762439	0.3213056	0.3642628	0.2230154
DNAJC7	0.5872454	0.5902146	0.6260545	0.6397081	0.5034281
NKIRAS2	0.4591297	0.4337259	0.4282433	0.4239325	0.3459042
ZNF385C	0.0210681	0.0172451	0.0360425	0.0087648	0.0143569
C17orf113	0.0201656	0.0209462	0.0172771	0.0128043	0.0039411
DHX58	0.0080230	0.0116573	0.0172736	0.0077434	0.0153139
KAT2A	0.0749020	0.0749185	0.1080719	0.0887214	0.1190670
RAB5C	0.7073937	0.7045821	0.7667244	0.7514613	0.5741668
RAB5C-AS1	0.0006636	0.0008907	0.0000000	0.0000000	0.0000000
KCNH4	0.0034148	0.0120606	0.0197897	0.0072915	0.0190513
HCRT	0.0022907	0.0031004	0.0019725	0.0040718	0.0000000
GHDC	0.0149642	0.0116590	0.0048027	0.0316853	0.0030451
STAT5B	0.1625627	0.1533550	0.1604752	0.1342633	0.2801079
ENSG00000267758	0.0000000	0.0000000	0.0000000	0.0000000	0.0048241

ENSG00000236194	0.0016580	0.0009709	0.0000000	0.0000000	0.0000000
ENSG00000278829	0.0000000	0.0019120	0.0000000	0.0000000	0.0059004
STAT5A	0.0171118	0.0202714	0.0249116	0.0105191	0.0220999
STAT3	0.5299486	0.4544165	0.3839311	0.6457313	0.5671286
CAVIN1	0.3693568	0.2650274	0.1960873	0.4299521	0.1720780
ATP6V0A1	0.5083767	0.6372697	0.7516967	0.5410265	0.7538620
ENSG00000267222	0.0018066	0.0012338	0.0000000	0.0000000	0.0087971
ENSG00000267632	0.0055354	0.0092330	0.0017758	0.0155864	0.0317688
NAGLU	0.2070580	0.1326014	0.1047654	0.3891047	0.1243218
HSD17B1-AS1	0.0567434	0.0540770	0.0544387	0.0802238	0.0256606
HSD17B1	0.0484097	0.0258719	0.0205773	0.0458806	0.0326383
COASY	0.2169568	0.1834822	0.1529873	0.2156856	0.1227278
MLX	0.2095461	0.1908065	0.2175753	0.1349791	0.1438609
PSMC3IP	0.1099734	0.1413374	0.1173017	0.1377044	0.0726679
ENSG00000287710	0.0103787	0.0032920	0.0136438	0.0186068	0.0000000
RETREG3	0.1188871	0.1061399	0.1036372	0.1324269	0.1487467
TUBG1	0.1717650	0.2046105	0.2971507	0.2048559	0.1075529
TUBG2	0.1492693	0.1622771	0.1571268	0.1590455	0.1498124
PLEKHH3	0.0919859	0.1122468	0.1137175	0.1108496	0.0939172
CCR10	0.0216273	0.0204165	0.0504484	0.0211581	0.0276773
ENSG00000267042	0.0044166	0.0078873	0.0109001	0.0024545	0.0055597
CNTNAP1	0.0628715	0.0759053	0.0798224	0.0528856	0.1393186
ENSG00000267765	0.0009580	0.0006824	0.0022363	0.0041513	0.0086422
EZH1	0.2681526	0.3130997	0.3194062	0.2764950	0.3089057
ENSG00000273650	0.0015757	0.0024524	0.0044663	0.0041306	0.0121838
RAMP2-AS1	0.0181073	0.0371460	0.0299664	0.0273277	0.0306712
RAMP2	0.1552081	0.2604670	0.3712716	0.1318371	0.1281319
VPS25	0.3647490	0.3082416	0.2982653	0.3537034	0.2692589
WNK4	0.0046132	0.0014775	0.0000000	0.0015029	0.0056738
COA3	0.8604289	0.8217063	0.9137560	0.8769352	0.6914846
CNTD1	0.0075517	0.0118375	0.0124835	0.0160139	0.0023834
BECN1	0.2556548	0.2658804	0.2946491	0.2602661	0.2329244
PSME3	0.2253972	0.2263566	0.1961350	0.2404154	0.1583988
AOC2	0.0024924	0.0166763	0.0154910	0.0000000	0.0170041
AOC3	0.0000000	0.0028621	0.0018817	0.0037736	0.0044787
AOC4P	0.0000000	0.0000000	0.0000000	0.0000000	0.0042206
AARSD1	0.2431872	0.3337360	0.3515317	0.2220389	0.1887964
PTGES3L	0.0490438	0.0805730	0.1239546	0.0458652	0.0685890
RUNDC1	0.1557219	0.1362790	0.1718356	0.1735064	0.1534921
RPL27	2.5256638	2.5269606	2.4111040	2.4494946	2.2734555
IFI35	0.0699593	0.0360090	0.0147825	0.0724096	0.0372283
VAT1	0.8675301	0.8097008	1.0550599	0.8772682	0.6400820
RND2	0.4432624	0.5450313	0.5611559	0.4097006	0.3558579
BRCA1	0.0681894	0.0497333	0.0477733	0.0625177	0.0649788
NBR2	0.1793276	0.1313143	0.1298057	0.1330731	0.1179556
ENSG00000267002	0.1872852	0.2073196	0.2207466	0.1406883	0.2113704
NBR1	0.4422589	0.3930902	0.3249542	0.4083585	0.3417323
TMEM106A	0.0033146	0.0022547	0.0015844	0.0061177	0.0077880
CCDC200	0.0125655	0.0117310	0.0051818	0.0000000	0.0068155
LINC00910	0.0480786	0.0515818	0.0305435	0.0465716	0.1263633
ENSG00000288909	0.0038372	0.0000000	0.0034305	0.0075470	0.0080666
ARL4D	0.2451744	0.1624840	0.1660184	0.2181625	0.1711234
DHX8	0.1650740	0.1719829	0.1629684	0.1899884	0.1883209

ETV4	0.0010781	0.0038197	0.0041190	0.0133501	0.0000000
MEOX1	0.0000000	0.0000000	0.0024513	0.0000000	0.0062580
LINC02594	0.0049206	0.0000000	0.0025324	0.0000000	0.0020838
SOST	0.0000000	0.0025161	0.0000000	0.0000000	0.0000000
DUSP3	0.2196411	0.2250919	0.2068869	0.2641703	0.1997822
CFAP97D1	0.0000000	0.0000000	0.0012120	0.0000000	0.0000000
MPP3	0.1179071	0.2362741	0.2986758	0.1104215	0.1958127
CD300LG	0.0000000	0.0000000	0.0000000	0.0000000	0.0000000
MPP2	0.1629694	0.2628537	0.3286352	0.1154692	0.2483830
LINC01976	0.0000000	0.0115757	0.0046931	0.0052087	0.0068978
PYY	0.0065467	0.0059896	0.0074506	0.0000000	0.0261040
NAGS	0.0128423	0.0086089	0.0069235	0.0121192	0.0048735
TMEM101	0.2164061	0.1919470	0.1587314	0.2592190	0.1294510
LSM12	0.6928636	0.8155150	0.8252959	0.6675781	0.6043937
G6PC3	0.3703409	0.2902089	0.2860040	0.5560387	0.2818232
HDAC5	0.2433165	0.3573955	0.3297501	0.1554521	0.3606339
ENSG00000267638	0.0006077	0.0018877	0.0000000	0.0000000	0.0037447
HROB	0.0036192	0.0064264	0.0062828	0.0123550	0.0000000
ASB16	0.0017149	0.0004281	0.0000000	0.0000000	0.0074555
ASB16-AS1	0.0434946	0.0679742	0.0271707	0.0499391	0.0610882
TMUB2	0.1495981	0.1410873	0.1113764	0.1765613	0.1440619
ATXN7L3	0.2843507	0.3962729	0.4803277	0.2409077	0.2721623
ATXN7L3-AS1	0.0000000	0.0017990	0.0108088	0.0000000	0.0000000
UBTF	0.6685566	0.6173990	0.6268762	0.6831674	0.5909145
ENSG00000260793	0.0326724	0.0159921	0.0079269	0.0204134	0.0397930
ENSG00000288877	0.0231737	0.0266886	0.0416689	0.0064523	0.0420356
SLC4A1	0.0000000	0.0000000	0.0060648	0.0000000	0.0000000
RUNDC3A-AS1	0.0217574	0.0394485	0.0581703	0.0262818	0.0416131
RUNDC3A	0.1669672	0.3487673	0.4536984	0.1490338	0.2634873
SLC25A39	0.6069382	0.5163388	0.4282457	0.6420178	0.3947423
ENSG00000288961	0.0040262	0.0000000	0.0016515	0.0037268	0.0000000
GRN	0.4150880	0.2165254	0.1403361	0.7098690	0.2217115
FAM171A2	0.1894377	0.2762583	0.2899790	0.1540174	0.2472350
ITGA2B	0.0065967	0.0082329	0.0100392	0.0025834	0.0381189
GPATCH8	0.5179827	0.4249056	0.4476617	0.5023664	0.7376831
ENSG00000283045	0.0000000	0.0000000	0.0000000	0.0043864	0.0142874
FZD2	0.3103375	0.1668421	0.1055220	0.3068653	0.1492699
MEIOC	0.0075213	0.0063272	0.0041001	0.0071372	0.0043293
CCDC43	0.4099655	0.3858469	0.4036865	0.4547146	0.2549115
ENSG00000267160	0.0050526	0.0010308	0.0032795	0.0090987	0.0106210
DBF4B	0.0296294	0.0262452	0.0204463	0.0126214	0.0582048
ADAM11	0.0204686	0.0341785	0.0291046	0.0094061	0.0583582
ENSG00000267505	0.0010230	0.0000000	0.0019410	0.0000000	0.0000000
ENSG00000267405	0.0000000	0.0000000	0.0000000	0.0000000	0.0000000
GJC1	0.0304542	0.0553951	0.0395643	0.0611222	0.0973813
HIGD1B	0.0003792	0.0000000	0.0000000	0.0000000	0.0072052
EFTUD2	0.1826040	0.1847430	0.1759309	0.1723730	0.2228613
CCDC103	0.0788649	0.0422635	0.0213533	0.1094776	0.0499623
GFAP	0.1374631	0.0870583	0.0607323	0.2900959	0.2568062
KIF18B	0.0000000	0.0000000	0.0000000	0.0018874	0.0000000
NMT1	0.2774027	0.3196081	0.2771765	0.3209087	0.3027931
C1QL1	0.0436666	0.1037794	0.0842472	0.0652340	0.0760750
DCAKD	0.2671277	0.2551744	0.2759413	0.3009540	0.3333257

PLCD3	0.0768655	0.0336767	0.0237914	0.0581402	0.0261140
ACBD4	0.0454624	0.0433536	0.0390048	0.0486770	0.0394503
ENSG00000276728	0.0161157	0.0114060	0.0184845	0.0148834	0.0017223
HEXIM1	0.2926551	0.2150157	0.1595667	0.2333138	0.2372711
HEXIM2-AS1	0.0173112	0.0093165	0.0137291	0.0070009	0.0330988
HEXIM2	0.0896591	0.0777971	0.0802527	0.0591378	0.0558903
ENSG00000267288	0.0031436	0.0018527	0.0060582	0.0023850	0.0057345
FMNL1-DT	0.0155459	0.0228839	0.0157085	0.0149536	0.0360725
FMNL1	0.0234609	0.0546189	0.0678555	0.0109712	0.0184699
FMNL1-AS1	0.0171603	0.0353019	0.0493077	0.0075971	0.0416188
MAP3K14-AS1	0.0103340	0.0229016	0.0364537	0.0075192	0.0121186
SPATA32	0.0000000	0.0000000	0.0000000	0.0059288	0.0211171
MAP3K14	0.0220248	0.0133733	0.0280955	0.0059569	0.0684388
ENSG00000267446	0.0000000	0.0000000	0.0000000	0.0000000	0.0056180
ARHGAP27	0.0028048	0.0054890	0.0051177	0.0000000	0.0112823
PLEKHM1	0.0438240	0.0464858	0.0191826	0.0675138	0.0716726
ENSG00000236234	0.0000000	0.0000000	0.0000000	0.0000000	0.0107984
ENSG00000291175	0.1193887	0.1597616	0.1711089	0.1007994	0.3394245
ENSG00000267198	0.0005367	0.0000000	0.0000000	0.0000000	0.0000000
CRHR1	0.0141970	0.0503007	0.0475829	0.0259791	0.0433205
MAPT-AS1	0.0226333	0.0515186	0.0689029	0.0253982	0.1571010
MAPT	1.2534476	2.0970894	2.4606021	1.2112195	1.8278461
MAPT-IT1	0.0058664	0.0101390	0.0067163	0.0089504	0.0218323
ENSG00000262881	0.0000000	0.0000000	0.0000000	0.0000000	0.0000000
STH	0.0000000	0.0000000	0.0000000	0.0000000	0.0037962
KANSL1	0.3826863	0.3993960	0.3363266	0.3287453	0.9005456
KANSL1-AS1	0.1652220	0.1433118	0.0849373	0.1190295	0.0713355
ENSG00000291018	0.0051496	0.0039384	0.0070780	0.0067055	0.0000000
ARL17B	0.0420049	0.0720729	0.0621752	0.0386685	0.1129460
LRRC37A	0.0062512	0.0093125	0.0082574	0.0063096	0.0497331
ARL17A	0.0723080	0.0858883	0.0576794	0.0658529	0.1698541
LRRC37A2	0.0362960	0.0721124	0.0659302	0.0310116	0.1401138
FAM215B	0.0054553	0.0023099	0.0153321	0.0082553	0.0376842
NSF	0.4093990	0.6830859	0.9773496	0.3956706	0.6766402
WNT3	0.0462463	0.0473963	0.0770899	0.0489002	0.0378094
WNT9B	0.0006301	0.0000000	0.0000000	0.0010021	0.0000000
LINC01974	0.0018872	0.0030981	0.0073719	0.0000000	0.0000000
GOSR2-DT	0.0042768	0.0030060	0.0044714	0.0031599	0.0100175
GOSR2	0.3496175	0.3893332	0.3862544	0.3941797	0.3298125
RPRML	0.0024775	0.0000000	0.0009588	0.0000000	0.0000000
ENSG00000291209	0.0068466	0.0062634	0.0023428	0.0080634	0.0233392
ENSG00000262879	0.2708839	0.2653726	0.2789638	0.2359949	0.4169715
CDC27	0.4627521	0.3707160	0.4137941	0.3871367	0.4137439
ENSG00000262265	0.0010542	0.0080813	0.0037112	0.0074832	0.0119678
ENSG00000261872	0.0012726	0.0000000	0.0000000	0.0000000	0.0040644
ITGB3	0.0141010	0.0106202	0.0035105	0.0172573	0.0104083
ENSG00000276790	0.0025992	0.0000000	0.0000000	0.0000000	0.0000000
EFCAB13-DT	0.0111258	0.0122955	0.0273684	0.0116849	0.0238038
EFCAB13	0.0513348	0.0693409	0.0493029	0.0290652	0.1649780
ENSG00000253347	0.0112227	0.0177319	0.0073697	0.0033942	0.0628060
MRPL45P2	0.0374124	0.0342299	0.0180183	0.0148707	0.1318704
NPEPPS	0.7368933	0.6711610	0.6673498	0.7218220	0.7021685
KPNB1-DT	0.0258957	0.0217353	0.0108836	0.0103537	0.0910079

KPNB1	0.9635865	0.8373999	0.7434188	0.9252474	0.7853467
ENSG00000264558	0.0043434	0.0008135	0.0028007	0.0045147	0.0000000
TBKBP1	0.0716832	0.1017765	0.0718210	0.0871741	0.1087150
OSBPL7	0.0246269	0.0226848	0.0251839	0.0224289	0.0353754
MRPL10	0.2559836	0.2561098	0.2572226	0.2458933	0.2511500
LRRC46	0.0915122	0.0328905	0.0150271	0.0753739	0.0246641
SCRN2	0.2328318	0.1231681	0.1118370	0.2446020	0.1047455
SP6	0.0006460	0.0021407	0.0000000	0.0065421	0.0000000
SP2-DT	0.1098001	0.1580716	0.1480094	0.1350075	0.0929295
SP2	0.0797063	0.0567536	0.0847353	0.0734228	0.0621045
SP2-AS1	0.0202854	0.0645029	0.0228507	0.0200083	0.0418757
PNPO	0.2147690	0.1619155	0.1356566	0.2506255	0.1291797
ENSG00000264019	0.0000000	0.0009084	0.0009013	0.0000000	0.0000000
CDK5RAP3	0.2729117	0.2092240	0.1854315	0.3307266	0.1901170
ENSG00000264701	0.0022865	0.0009961	0.0000000	0.0000000	0.0000000
ENSG00000289599	0.0002995	0.0000000	0.0000000	0.0000000	0.0000000
COPZ2	0.0926881	0.0683119	0.0304471	0.1428808	0.0250254
NFE2L1-DT	0.0202404	0.0202578	0.0067070	0.0170278	0.0296925
NFE2L1	1.1821417	0.8972885	0.8169859	1.3962615	0.8543685
ENSG00000278765	0.0044188	0.0035789	0.0035211	0.0000000	0.0042647
CBX1	1.2136857	1.2623532	1.5031113	1.2609367	0.9273313
SNX11	0.0849474	0.0946504	0.0814660	0.0692482	0.0893386
SKAP1	0.0768601	0.0383556	0.0228061	0.0228932	0.0614184
SKAP1-AS1	0.0003347	0.0004281	0.0000000	0.0000000	0.0021475
SKAP1-AS2	0.0065890	0.0231630	0.0450939	0.0063770	0.0243115
ENSG00000264451	0.0000000	0.0009394	0.0000000	0.0000000	0.0000000
HOXB1	0.0002948	0.0000000	0.0000000	0.0060498	0.0000000
HOXB2	0.6126201	0.4925040	0.5746923	0.5687807	0.4474003
HOXB-AS1	0.2364985	0.1827915	0.1342030	0.1894382	0.1242141
HOXB3	0.2614205	0.2635144	0.3417927	0.2474768	0.4307041
HOXB-AS3	0.1469191	0.0895959	0.0729719	0.1354792	0.0839980
HOXB-AS2	0.0025658	0.0020932	0.0000000	0.0000000	0.0000000
HOXB4	0.6362599	0.6732293	1.1007555	0.6242879	0.6662684
ENSG00000257178	0.0050338	0.0119276	0.0010907	0.0037166	0.0147991
HOXB5	0.6696831	0.8688713	1.4239757	0.5413054	0.8601010
HOXB6	0.1522439	0.0880456	0.0274018	0.1038834	0.0777952
HOXB7	0.1824709	0.0849150	0.0622343	0.1412319	0.1569021
HOXB8	0.0930540	0.0472696	0.0198192	0.0628335	0.0433510
HOXB9	0.0006667	0.0019752	0.0000000	0.0000000	0.0000000
TTLL6	0.0022268	0.0030692	0.0026996	0.0000000	0.0124680
CALCOCO2	0.6258061	0.3653586	0.2475337	0.5997901	0.3782456
ATP5MC1	0.8091800	1.0247715	1.1229039	0.8215996	0.7173058
UBE2Z	0.4476870	0.5613187	0.6101668	0.5112548	0.4871928
SNF8	0.4167597	0.3550142	0.4512910	0.3501445	0.3341209
ENSG00000230532	0.0000000	0.0014335	0.0022302	0.0026849	0.0085793
ENSG00000289021	0.0187429	0.0076588	0.0020882	0.0254323	0.0113951
GIP	0.0000000	0.0012690	0.0000000	0.0000000	0.0000000
ENSG00000250838	0.0059958	0.0010515	0.0011658	0.0038666	0.0000000
IGF2BP1	0.2265918	0.1644767	0.1341885	0.1847254	0.2324262
ENSG00000251461	0.0009884	0.0000000	0.0016443	0.0000000	0.0013712
B4GALNT2	0.0005103	0.0000000	0.0008856	0.0000000	0.0000000
PHOSPHO1	0.0000000	0.0000000	0.0000000	0.0000000	0.0038284
FLJ40194	0.0039410	0.0014866	0.0054294	0.0035651	0.0190891

ZNF652	0.4060814	0.4859644	0.5195575	0.3922216	0.5934240
ZNF652-AS1	0.0034898	0.0042846	0.0060609	0.0000000	0.0050279
ENSG00000262039	0.0085726	0.0063418	0.0008287	0.0041649	0.0054224
PHB1	0.5864228	0.6258668	0.6089421	0.6498283	0.4993786
NGFR	0.1205313	0.2495412	0.1398300	0.1452698	0.1935098
NGFR-AS1	0.0021519	0.0019591	0.0000000	0.0068943	0.0000000
NXPH3	0.0032576	0.0040539	0.0218336	0.0019255	0.0078652
SPOP	0.5258603	0.5010544	0.5389748	0.5355499	0.4800273
SLC35B1	0.3510942	0.3239406	0.3525056	0.4047928	0.3049777
ENSG00000250751	0.0017312	0.0030436	0.0018758	0.0000000	0.0050876
FAM117A	0.0613614	0.0278602	0.0170597	0.0637318	0.0266498
KAT7	0.3416621	0.3075174	0.3224977	0.3057347	0.2596767
TAC4	0.0000000	0.0033200	0.0000000	0.0000000	0.0018081
ENSG00000248954	0.0008924	0.0000000	0.0000000	0.0000000	0.0095104
ENSG00000254039	0.0021908	0.0000000	0.0000000	0.0000000	0.0012783
ENSG00000248172	0.0000000	0.0000000	0.0023101	0.0000000	0.0000000
PICART1	0.0005827	0.0024823	0.0000000	0.0000000	0.0021475
ITGA3	0.0514213	0.0526053	0.0631134	0.0650194	0.1231820
PDK2	0.1034550	0.1068901	0.0904790	0.1109765	0.0589361
SAMD14	0.0621734	0.1302048	0.1714518	0.0802806	0.1553741
PPP1R9B	0.1214752	0.1556167	0.1858392	0.0800175	0.1063406
ENSG00000236472	0.0000000	0.0000000	0.0019220	0.0000000	0.0000000
ENSG00000253730	0.0102939	0.0199750	0.0287646	0.0142673	0.0297090
COL1A1	0.0671635	0.0757502	0.1114118	0.0858508	0.1343982
TMEM92-AS1	0.0002592	0.0000000	0.0020158	0.0000000	0.0000000
XYLT2	0.0420327	0.0352340	0.0099442	0.0843832	0.0582117
MRPL27	0.4665472	0.4968113	0.5181048	0.4901536	0.3368796
EME1	0.0070124	0.0071562	0.0000000	0.0013877	0.0063555
LRRC59	0.3087102	0.2767940	0.2643787	0.3240558	0.1457406
ACSF2	0.0252502	0.0074269	0.0011369	0.0319874	0.0149990
RSAD1	0.1070871	0.0752898	0.1081156	0.1017355	0.0409753
ENSG00000249451	0.0011718	0.0000000	0.0000000	0.0000000	0.0000000
MYCBPAP	0.0094286	0.0085240	0.0116894	0.0014220	0.0161612
ENSG00000250286	0.0017513	0.0013436	0.0025783	0.0000000	0.0000000
EPN3	0.0010679	0.0022329	0.0007974	0.0000000	0.0000000
SPATA20	0.1808682	0.1282353	0.1577481	0.1295782	0.1226093
CACNA1G-AS1	0.0013699	0.0022088	0.0016084	0.0000000	0.0000000
CACNA1G	0.0171137	0.0303261	0.0441238	0.0027668	0.0963016
ABCC3	0.0010157	0.0000000	0.0000000	0.0000000	0.0000000
ANKRD40	0.2223341	0.1463856	0.1317449	0.2177705	0.1133411
ENSG00000262967	0.0480946	0.0301807	0.0226456	0.0551833	0.0386465
LUC7L3	0.9390451	0.9962432	0.8527329	0.9005518	1.3478557
ANKRD40CL	0.0000000	0.0000000	0.0015759	0.0000000	0.0040472
TOB1	0.5497714	0.3524965	0.2418801	0.6250502	0.3718778
TOB1-AS1	0.0435225	0.0448751	0.0754816	0.0212358	0.0518085
ENSG00000247011	0.0000000	0.0000000	0.0000000	0.0000000	0.0015201
SPAG9	1.0977773	1.1448218	1.0904109	1.0743529	1.1413584
ENSG00000249870	0.0017180	0.0000000	0.0015452	0.0000000	0.0357243
NME1	0.6919833	0.7876190	0.7986812	0.7266912	0.5398718
NME2	1.6367346	1.7111019	1.6834435	1.6677657	1.5359877
MBTD1	0.1859698	0.1272715	0.0912853	0.1780018	0.2343646
ENSG00000264895	0.0071663	0.0092192	0.0023368	0.0035629	0.0368438
UTP18	0.3160030	0.2678236	0.2520540	0.3080492	0.3288487

LINC02071	0.0000000	0.0000000	0.0000000	0.0041842	0.0000000
LINC02073	0.0020482	0.0019913	0.0024527	0.0000000	0.0039411
CA10	0.1558575	0.2883184	0.4054060	0.1725499	0.4930317
LINC01982	0.0312965	0.0288730	0.0354564	0.0271975	0.1484835
ENSG00000285939	0.0088612	0.0074186	0.0031267	0.0000000	0.0000000
ENSG00000288036	0.0000000	0.0020668	0.0000000	0.0049937	0.0118644
TOM1L1	0.2192287	0.1320873	0.1153251	0.1534513	0.2050403
COX11	0.3331887	0.3682225	0.3898857	0.2823599	0.2278789
STXBP4	0.1942372	0.1717194	0.1555440	0.2450753	0.3512739
HLF	0.0179498	0.0213917	0.0109205	0.0101598	0.0353848
MMD	0.5577158	0.8203649	1.0604630	0.5012896	0.5237786
TMEM100	0.0426757	0.0185644	0.0124634	0.0553567	0.0051457
PCTP	0.0729159	0.0708257	0.0461265	0.0676340	0.0687940
ENSG00000262052	0.0018502	0.0000000	0.0048006	0.0000000	0.0054402
ANKFN1	0.1691077	0.1065024	0.0788999	0.1569347	0.1985877
NOG	0.0207314	0.0131932	0.0171885	0.0343829	0.0153548
C17orf67	0.1375874	0.2035861	0.2202731	0.0830664	0.1326696
DGKE	0.1375051	0.1641271	0.3900391	0.1248639	0.2637743
TRIM25	0.0659916	0.0350469	0.0108836	0.0730911	0.0152395
ENSG00000274213	0.0000000	0.0020059	0.0000000	0.0025742	0.0000000
COIL	0.3217596	0.3325951	0.3391422	0.3543066	0.2825902
SCPEP1	0.1508535	0.1032147	0.0860039	0.3263520	0.0969349
AKAP1-DT	0.1091437	0.0646605	0.0576176	0.0773660	0.0317567
AKAP1	0.0927847	0.1019761	0.1390405	0.1291517	0.1325436
ENSG00000263089	0.0000000	0.0000000	0.0000000	0.0033377	0.0031863
ENSG00000286732	0.0015685	0.0019426	0.0000000	0.0125894	0.0068867
MSI2	0.8350242	0.6066917	0.5097158	0.9195054	1.0416213
ENSG00000266100	0.0000000	0.0000000	0.0000000	0.0000000	0.0000000
ENSG00000265542	0.0178168	0.0077283	0.0058622	0.0067014	0.0035158
MRPS23	0.5112588	0.4888584	0.5478104	0.4476679	0.4250879
CUEDC1	0.3557305	0.3069475	0.2689038	0.4235207	0.2995861
ENSG00000264914	0.0000000	0.0017465	0.0000000	0.0000000	0.0054224
VEZF1	0.8656614	0.6104889	0.5408287	0.8642940	0.5564117
ENSG00000264112	0.0318241	0.0394287	0.0210683	0.0456419	0.1069845
SRSF1	0.4123049	0.3824568	0.3760642	0.4046815	0.4194235
ENSG00000285930	0.0020742	0.0010546	0.0000000	0.0016217	0.0039501
DYNLL2	0.6770081	1.0174708	1.2201927	0.5847485	0.7572194
OR4D1	0.0069904	0.0080263	0.0064670	0.0033570	0.0000000
EPX	0.0004774	0.0010952	0.0020237	0.0029008	0.0000000
ENSG00000278627	0.0032185	0.0111459	0.0038467	0.0077545	0.0000000
MKS1	0.1084531	0.0841416	0.0905077	0.1390446	0.0686037
LPO	0.0042485	0.0030506	0.0022147	0.0000000	0.0000000
ENSG00000287337	0.0031722	0.0058216	0.0019140	0.0029645	0.0037431
ENSG00000286788	0.0009736	0.0019015	0.0047402	0.0000000	0.0000000
TSPOAP1	0.0610962	0.0824397	0.0614896	0.0488815	0.2243481
TSPOAP1-AS1	0.0547695	0.0626544	0.0582671	0.0717209	0.0701482
SUPT4H1	0.4797611	0.4286390	0.4486635	0.4844324	0.3691194
RNF43	0.0033011	0.0025732	0.0000000	0.0000000	0.0128645
HSF5	0.0038588	0.0043161	0.0086428	0.0000000	0.0180995
MTMR4	0.1262875	0.2233522	0.2476190	0.1153173	0.3408050
ENSG00000289147	0.0010687	0.0026603	0.0014608	0.0000000	0.0067140
SEPTIN4-AS1	0.0019087	0.0017797	0.0000000	0.0025494	0.0133615
SEPTIN4	0.1217311	0.2162316	0.2143162	0.1453531	0.1875863

TEX14	0.0550605	0.0375349	0.0506715	0.0354032	0.2034311
ENSG00000290039	0.0038474	0.0030370	0.0045194	0.0000000	0.0038969
RAD51C	0.1532661	0.1736996	0.1251266	0.1212199	0.1243315
PPM1E	0.2768401	0.4205695	0.3599999	0.2049563	0.7677054
ENSG00000263938	0.0005567	0.0030561	0.0000000	0.0000000	0.0016673
TRIM37	0.5946616	0.7037387	0.8522089	0.6312464	0.6407301
ENSG00000224738	0.0355639	0.0153821	0.0260583	0.0180394	0.0239420
SKA2	0.6111994	0.6598418	0.6569935	0.6936849	0.4395506
PRR11	0.1189068	0.1121619	0.1392000	0.1468655	0.1055434
SMG8	0.0739260	0.0716762	0.0820494	0.1020430	0.0551844
GDPD1	0.1710045	0.2690703	0.3402208	0.2124372	0.2842827
YPEL2	0.1335033	0.0719552	0.1162812	0.1753508	0.1754690
LINC01476	0.0000000	0.0000000	0.0000000	0.0000000	0.0098291
DHX40	0.7714124	0.5271603	0.3905843	0.7127464	0.4831221
ENSG00000273702	0.1315634	0.1247646	0.1157589	0.0809733	0.1043493
CLTC	1.1066706	1.0288634	1.0886981	1.0837967	0.9769474
PTRH2	0.1380632	0.1140446	0.1241809	0.1970249	0.1958056
VMP1	0.3503216	0.3340356	0.3107428	0.4503479	0.3634906
TUBD1	0.0921659	0.0778992	0.0766769	0.0721873	0.1043685
RPS6KB1	0.2117557	0.2382905	0.1954437	0.1629400	0.2443329
RNFT1	0.0577854	0.0455381	0.0358641	0.0959791	0.0636480
RNFT1-DT	0.0137761	0.0249965	0.0165474	0.0238794	0.0180424
HEATR6	0.2073418	0.1394484	0.1294003	0.2311942	0.1876684
HEATR6-DT	0.0092667	0.0025914	0.0248810	0.0045995	0.0069459
ENSG00000267248	0.0215608	0.0383250	0.0328865	0.0114373	0.0314916
CA4	0.0015308	0.0000000	0.0000000	0.0000000	0.0000000
USP32	0.4402563	0.4610721	0.4553089	0.4417131	0.5930040
CHCT1	0.0005458	0.0029572	0.0000000	0.0048001	0.0000000
APPBP2	0.5889292	0.5181083	0.5012424	0.5632008	0.5995739
APPBP2-DT	0.0084945	0.0013262	0.0018800	0.0027037	0.0016619
PPM1D	0.1435417	0.1129879	0.1139443	0.1363602	0.1345375
BCAS3	0.2197538	0.2489237	0.2083076	0.1892114	0.6296781
BCAS3-AS1	0.0041791	0.0000000	0.0000000	0.0000000	0.0120862
ENSG00000286997	0.0000000	0.0000000	0.0000000	0.0039347	0.0023834
ENSG00000267131	0.0000000	0.0000000	0.0029356	0.0000000	0.0027063
TBX2-AS1	0.0086278	0.0173920	0.0012964	0.0000000	0.0173779
TBX2	0.0226141	0.0547386	0.0228349	0.0092411	0.0519441
NACA2	0.0087669	0.0079856	0.0000000	0.0112664	0.0042647
BRIP1	0.0080292	0.0036948	0.0090543	0.0148906	0.0125233
INTS2	0.0698651	0.0647440	0.0534263	0.0461713	0.1329807
MED13	0.4245139	0.4402150	0.4338764	0.4580699	0.5965517
ENSG00000285879	0.0000000	0.0000000	0.0000000	0.0000000	0.0070707
ENSG00000289293	0.0025447	0.0022995	0.0069217	0.0062143	0.0014182
EFCAB3	0.0000000	0.0041521	0.0044982	0.0037133	0.0142173
METTL2A	0.2902185	0.3255313	0.3929001	0.2879579	0.2769752
TLK2	0.5014708	0.5277034	0.5116611	0.4713214	0.5845963
ENSG00000264546	0.0011683	0.0007551	0.0000000	0.0000000	0.0154913
ENSG00000277463	0.0000000	0.0010502	0.0000000	0.0000000	0.0028533
ENSG00000274565	0.0151859	0.0039628	0.0069456	0.0343828	0.0000000
MRC2	0.2937325	0.1533693	0.0998766	0.4906221	0.1651655
ENSG00000265702	0.0022965	0.0008093	0.0023495	0.0054504	0.0092410
MARCHF10	0.0420700	0.0276113	0.0271379	0.0545944	0.0258840
MARCHF10-DT	0.0000000	0.0020424	0.0000000	0.0068692	0.0000000

ENSG00000283538	0.0345678	0.0788541	0.0725598	0.0332676	0.1213417
TANC2	0.5345829	0.6581601	0.6348362	0.5022062	1.5493929
ENSG00000264513	0.0000000	0.0018009	0.0000000	0.0000000	0.0056293
ENSG00000233635	0.0116200	0.0045552	0.0120821	0.0189929	0.1705664
ENSG00000226797	0.0000000	0.0000000	0.0000000	0.0000000	0.0086229
ENSG00000263644	0.0031493	0.0018065	0.0027536	0.0000000	0.0025607
CYB561	0.2730792	0.2544304	0.2988679	0.3375597	0.1903361
ENSG00000265971	0.0000000	0.0000000	0.0000000	0.0000000	0.0000000
ACE	0.0035292	0.0108677	0.0182868	0.0031735	0.0294749
KCNH6	0.0075614	0.0176263	0.0258853	0.0021257	0.0038734
DCAF7	0.5389323	0.6858469	0.7851408	0.5449415	0.5122316
TACO1	0.2141407	0.1653030	0.1740741	0.1755559	0.1118635
MAP3K3	0.0648733	0.0908332	0.0654009	0.0602334	0.1113537
STRADA	0.1155927	0.0954460	0.0994884	0.1183086	0.1675610
LIMD2	0.1587751	0.1500170	0.1626310	0.1639593	0.1170525
CCDC47	0.5140884	0.4450002	0.3778060	0.8150509	0.3919983
DDX42	0.7483128	0.6637033	0.5515225	0.7439564	0.6062750
FTSJ3	0.2256268	0.1714260	0.1649196	0.2245691	0.1539980
PSMC5	0.5880966	0.5978552	0.6427038	0.6220160	0.4192488
SMARCD2	0.0281332	0.0353968	0.0478989	0.0456555	0.0378662
SCN4A	0.0012083	0.0000000	0.0000000	0.0000000	0.0000000
ENSG00000263489	0.0000000	0.0006799	0.0000000	0.0000000	0.0000000
PRR29-AS1	0.0144336	0.0048986	0.0080718	0.0086808	0.0179873
PRR29	0.1785477	0.0849821	0.0632078	0.1905722	0.0998268
ICAM2	0.0040975	0.0034843	0.0000000	0.0000000	0.0086104
ERN1	0.0668496	0.0671688	0.0532985	0.0298999	0.0784398
SNHG25	0.1381177	0.1319508	0.0808215	0.1278792	0.0870577
TEX2	0.1309074	0.1687699	0.1958415	0.1474694	0.2357085
PECAM1	0.0000000	0.0048873	0.0028562	0.0000000	0.0000000
MILR1	0.0007624	0.0000000	0.0000000	0.0000000	0.0000000
POLG2	0.0705279	0.0554559	0.0348896	0.0600047	0.1283166
DDX5	1.7853475	1.6041553	1.6571089	1.8322820	1.5592614
CEP95	0.2769398	0.2340787	0.1678463	0.2273756	0.4233815
SMURF2	0.3274330	0.2695940	0.2326106	0.2568348	0.3857122
ENSG00000291117	0.0197381	0.0268363	0.0126976	0.0153229	0.0634932
LRRC37A3	0.0747599	0.0815027	0.0679182	0.1472749	0.1939469
ENSG00000265218	0.0024974	0.0039296	0.0000000	0.0000000	0.0036851
ENSG00000290457	0.0000000	0.0000000	0.0012697	0.0000000	0.0000000
AMZ2P1	0.3576603	0.3630841	0.3003936	0.3712846	0.2787285
GNA13	0.3782231	0.3629971	0.2923978	0.3785338	0.3499805
RGS9	0.6270126	0.5871035	0.5656916	0.5448824	0.4724129
AXIN2	0.0472674	0.0579079	0.0453163	0.0665549	0.0742803
CEP112	0.3717120	0.3395444	0.2971794	0.3008760	0.7188181
APOH	0.0000000	0.0014838	0.0000000	0.0000000	0.0000000
PRKCA	0.1613209	0.2142522	0.2266042	0.1488013	0.6319340
PRKCA-AS1	0.0048114	0.0027669	0.0000000	0.0000000	0.0183155
ENSG00000289587	0.0004935	0.0000000	0.0040493	0.0000000	0.0014498
CACNG5	0.0221775	0.0434953	0.0458445	0.0000000	0.0519859
ENSG00000289877	0.0083172	0.0174668	0.0123733	0.0134340	0.0343314
CACNG4	0.5363713	0.5451446	0.5301810	0.5735466	0.5187440
CACNG1	0.0116858	0.0081422	0.0084906	0.0076965	0.0210035
HELZ	0.4327689	0.4458045	0.5022036	0.4352481	0.5460252
ENSG00000285877	0.0413372	0.0362342	0.0154728	0.0460536	0.0913520

HELZ-AS1	0.0132795	0.0203268	0.0013959	0.0180550	0.0013481
PSMD12	0.8289633	0.7519751	0.7669134	0.8324735	0.6995772
PITPNC1	0.4906912	0.4599417	0.4475427	0.5327026	0.6993437
ENSG00000264754	0.0010522	0.0000000	0.0000000	0.0050634	0.0000000
NOL11	0.2187755	0.2109159	0.2188474	0.2369284	0.2219941
BPTF	1.0340965	1.0916019	1.0238112	0.9976732	1.3257493
C17orf58	0.5849561	0.4325620	0.3831788	0.6520803	0.3520464
KPNA2	0.3347816	0.4103750	0.5411888	0.3488138	0.3704641
ENSG00000265055	0.0020157	0.0071943	0.0000000	0.0056606	0.0000000
ENSG00000291214	0.6961187	0.6888417	0.7095524	0.7482412	0.6014380
ENSG00000278730	0.1694644	0.1478545	0.1449608	0.1628887	0.1706086
ENSG00000290646	0.0296214	0.0255675	0.0266603	0.0171206	0.0330618
ENSG00000274561	0.0287143	0.0372921	0.0371057	0.0200397	0.0095684
ENSG00000277476	0.0118575	0.0121041	0.0133760	0.0000000	0.0061951
ENSG00000278740	0.0079992	0.0064099	0.0063462	0.0062158	0.0208924
ENSG00000267731	0.0033512	0.0093784	0.0071204	0.0076113	0.0216680
AMZ2	0.5448820	0.4731029	0.5001084	0.4830989	0.4988036
ENSG00000290793	0.0613538	0.0901969	0.0698012	0.0479026	0.0790591
ENSG00000265100	0.0014608	0.0017861	0.0000000	0.0000000	0.0000000
ARSG	0.1044498	0.0859542	0.0402116	0.1019758	0.1461562
SLC16A6	0.1026401	0.0912101	0.0596050	0.1899115	0.0903697
ENSG00000267009	0.0607136	0.0450498	0.0362941	0.0844993	0.1102440
WIPI1	0.1041956	0.1005432	0.0751408	0.0803049	0.0839898
PRKAR1A	1.0552906	1.0477444	1.1619960	1.0459159	0.8712423
FAM20A	0.0027528	0.0065118	0.0088595	0.0111850	0.0031757
ENSG00000267461	0.0045762	0.0025814	0.0000000	0.0000000	0.0000000
LINC01482	0.0032519	0.0060600	0.0029908	0.0149434	0.0019259
ABCA8	0.0880686	0.0520317	0.0219158	0.2246367	0.0260151
ABCA9-AS1	0.0000000	0.0019886	0.0000000	0.0000000	0.0017703
ABCA9	0.0044427	0.0061895	0.0054393	0.0146009	0.0186927
ABCA6	0.0044696	0.0117515	0.0146064	0.0063087	0.0229625
ABCA10	0.0235409	0.0394977	0.0481574	0.0268641	0.1114728
ENSG00000285931	0.0000000	0.0000000	0.0000000	0.0000000	0.0056888
ABCA5	0.4629096	0.4334973	0.3699525	0.5237538	0.6603696
MAP2K6	0.3063161	0.3599404	0.2949563	0.2927537	0.3791604
ENSG00000267653	0.0008598	0.0000000	0.0000000	0.0000000	0.0000000
LINC01483	0.0011153	0.0014775	0.0000000	0.0083031	0.0083258
ENSG00000230258	0.0000000	0.0000000	0.0000000	0.0000000	0.0000000
KCNJ16	0.0029603	0.0034547	0.0045090	0.0246588	0.0000000
KCNJ2-AS1	0.0184987	0.0097794	0.0046282	0.0150646	0.0078060
KCNJ2	0.0360917	0.0312689	0.0603091	0.0443939	0.0798357
ENSG00000289371	0.0070428	0.0041540	0.0065928	0.0012109	0.0000000
ENSG00000286387	0.0000000	0.0011596	0.0000000	0.0000000	0.0099413
ENSG00000283517	0.0010940	0.0000000	0.0000000	0.0000000	0.0000000
ROCR	0.0038243	0.0010315	0.0064374	0.0000000	0.0000000
LINC01152	0.0201811	0.0061910	0.0159855	0.0039942	0.0126453
SOX9-AS1	0.1211561	0.0808836	0.0523013	0.1151411	0.0960466
LINC02097	0.0000000	0.0000000	0.0000000	0.0000000	0.0000000
SOX9	0.6230382	0.3352528	0.2124793	0.8020917	0.3454976
LINC00511	0.0796313	0.0555774	0.0703918	0.0755030	0.2195392
LINC02003	0.0003754	0.0000000	0.0015954	0.0030679	0.0097677
SLC39A11	0.0483282	0.0377349	0.0462273	0.0566973	0.1565918
SSTR2	0.0655929	0.1499347	0.3182751	0.0560712	0.2331865

COG1	0.1492475	0.1144472	0.1468856	0.2061953	0.1298640
FAM104A	0.2226379	0.1559150	0.1658577	0.1872734	0.1527809
C17orf80	0.2999198	0.2439511	0.2552785	0.3102714	0.2869337
CPSF4L	0.0000000	0.0018141	0.0008956	0.0000000	0.0021357
CDC42EP4	0.2173510	0.1693324	0.1154289	0.3365332	0.1660075
SDK2	0.1451753	0.1512562	0.1269286	0.1720686	0.4436678
ENSG00000264985	0.0000000	0.0000000	0.0000000	0.0000000	0.0067351
LINC00469	0.0013910	0.0000000	0.0011329	0.0000000	0.0000000
LINC02074	0.0068065	0.0044499	0.0029598	0.0198856	0.0038629
RPL38	2.6356718	2.6671029	2.5457348	2.5602125	2.4517000
MGC16275	0.0111268	0.0250469	0.0090791	0.0143504	0.0133089
TTYH2	0.0560996	0.0270739	0.0187394	0.1068678	0.0273058
DNAI2	0.0310039	0.0362138	0.0256151	0.0282268	0.0159852
KIF19	0.0022336	0.0047333	0.0021138	0.0055311	0.0044005
BTBD17	0.0215495	0.0186179	0.0050963	0.0658121	0.0251693
GPRC5C	0.3189137	0.1654590	0.0980887	0.4597004	0.1812497
CD300A	0.0065086	0.0048340	0.0150940	0.0068533	0.0000000
CD300LD-AS1	0.0000000	0.0006553	0.0015765	0.0000000	0.0000000
RAB37	0.0090700	0.0036141	0.0106740	0.0119274	0.0132315
NHERF1	0.2754062	0.1363720	0.0893142	0.2783669	0.0668552
NAT9	0.0873379	0.0736869	0.0464224	0.0826976	0.1091811
TMEM104	0.0283483	0.0183640	0.0073370	0.0576416	0.0532022
GRIN2C	0.0033088	0.0042683	0.0014209	0.0000000	0.0077151
FDXR	0.0972605	0.0645010	0.0413610	0.0715098	0.0316899
FADS6	0.0012267	0.0191762	0.0187534	0.0046187	0.0243157
USH1G	0.0000000	0.0000000	0.0038295	0.0000000	0.0000000
HID1	0.0450137	0.0638504	0.0721614	0.0284783	0.1087096
HID1-AS1	0.0000000	0.0000000	0.0009874	0.0000000	0.0000000
CDR2L	0.0606398	0.0754589	0.1054278	0.0419892	0.0728597
MRPL58	0.2414672	0.1963799	0.2029113	0.1937947	0.1726339
KCTD2	0.1428880	0.2567996	0.3053979	0.1984763	0.1994164
ATP5PD	1.1696187	1.2146533	1.2705699	1.1919948	0.9026015
SLC16A5	0.0058445	0.0016301	0.0000000	0.0000000	0.0000000
ARMC7	0.0357775	0.0364449	0.0515496	0.0230830	0.0570006
NT5C	0.3433385	0.3010939	0.1830508	0.3670192	0.2755014
JPT1	1.2628436	1.7447140	1.9937742	1.2470320	1.4001339
ENSG00000265800	0.0032570	0.0046549	0.0019820	0.0000000	0.0027747
ENSG00000263786	0.0000000	0.0000000	0.0000000	0.0000000	0.0000000
SUMO2	1.8554855	1.8947335	2.0840245	1.8684308	1.6403167
NUP85	0.0989305	0.0710896	0.0695531	0.1185555	0.1014430
GGA3	0.0602628	0.1041796	0.0957146	0.0540506	0.1185953
MRPS7	0.3785624	0.3895890	0.3934747	0.4252004	0.2208086
MIF4GD	0.1743519	0.1333648	0.1385729	0.1935194	0.1199798
MIF4GD-DT	0.0053299	0.0078421	0.0073442	0.0029435	0.0122841
SLC25A19	0.0408439	0.0282479	0.0226534	0.0298744	0.0148414
GRB2	0.8998181	1.1248157	1.3026773	0.9464917	0.9062058
ENSG00000265987	0.0019327	0.0016626	0.0000000	0.0000000	0.0057996
ENSG00000264853	0.0000000	0.0000000	0.0000000	0.0000000	0.0014580
ENSG00000265342	0.0138104	0.0056093	0.0236297	0.0132314	0.0173526
TMEM94	0.0896878	0.0497469	0.0459389	0.1521705	0.1178909
CASKIN2	0.0343042	0.0282029	0.0239037	0.0219480	0.0095135
TSEN54	0.0551690	0.0448661	0.0285319	0.0524627	0.0543032
LLGL2	0.0432517	0.0255399	0.0230268	0.0220833	0.0390865

MYO15B	0.0120868	0.0171159	0.0051580	0.0285906	0.0432786
RECQL5	0.0391929	0.0293855	0.0271676	0.0447312	0.0869326
SMIM5	0.0537411	0.0283916	0.0126755	0.0829501	0.0323673
SMIM6	0.0017519	0.0026940	0.0029720	0.0000000	0.0000000
SAP30BP	0.4051048	0.3493918	0.3705771	0.4413370	0.3321028
ITGB4	0.0423788	0.0240036	0.0203328	0.0732940	0.0252971
GALK1	0.1085922	0.0881277	0.0705556	0.1402569	0.0718859
H3-3B	2.3380746	2.0981289	1.9411157	2.1585985	1.8274189
UNK	0.1300689	0.1470875	0.1202561	0.1695390	0.1745704
UNC13D	0.0012759	0.0008415	0.0035837	0.0000000	0.0134678
WBP2	0.5233946	0.5511123	0.5559958	0.5410482	0.3072721
TRIM47	0.0874864	0.0563821	0.0624632	0.1250629	0.0442662
TRIM65	0.0683440	0.0433490	0.0429796	0.0625714	0.0513413
ENSG00000267342	0.0020539	0.0026571	0.0000000	0.0000000	0.0000000
MRPL38	0.2534158	0.2760342	0.2803354	0.2788762	0.2047673
FBF1	0.0745608	0.0346199	0.0336401	0.0601671	0.0652533
ACOX1	0.3102355	0.2031144	0.1680272	0.2969108	0.2206536
TEN1	0.0516311	0.0533478	0.0409694	0.0313477	0.0478365
CDK3	0.0084794	0.0108371	0.0114137	0.0126550	0.0120739
EVPL	0.0000000	0.0000000	0.0045500	0.0000000	0.0000000
SRP68	0.3654312	0.2704244	0.2368189	0.3237163	0.2794681
ZACN	0.0088850	0.0075015	0.0052778	0.0104599	0.0126876
GALR2	0.0434281	0.0164484	0.0190096	0.0123905	0.0074505
EXOC7	0.3741069	0.2607212	0.1781772	0.3510725	0.3575506
FOXJ1	0.4570075	0.2631529	0.1727770	0.4814383	0.2278713
RNF157-AS1	0.0353516	0.0558784	0.0454167	0.0183772	0.0508957
RNF157	0.3302415	0.5920637	0.5619164	0.2999950	0.5885456
UBALD2	0.1828482	0.1853713	0.2330481	0.2078428	0.1392015
QRICH2	0.0161486	0.0228975	0.0237753	0.0213492	0.1391957
PRPSAP1	0.3079075	0.3132369	0.3206466	0.2838936	0.4025342
SPHK1	0.0056047	0.0038538	0.0028683	0.0050717	0.0000000
UBE2O	0.2020312	0.2551265	0.2435911	0.1999183	0.4032202
AANAT	0.0032119	0.0017333	0.0029952	0.0027655	0.0037728
RHBDF2	0.0074677	0.0058924	0.0000000	0.0123739	0.0196427
CYGB	0.1419359	0.2061657	0.2421988	0.1499109	0.1737542
PRCD	0.0780910	0.1325293	0.2030579	0.0882016	0.1141155
ENSG00000267546	0.0032506	0.0024063	0.0000000	0.0000000	0.0044670
ENSG00000272386	0.0000000	0.0000000	0.0012498	0.0000000	0.0000000
SNHG16	0.9128041	0.8731147	0.7220604	0.8390740	0.6839575
ENSG00000284526	0.0713114	0.0662784	0.0603197	0.0652638	0.0727406
ST6GALNAC2	0.0331433	0.0167555	0.0094437	0.0160898	0.0151630
ST6GALNAC1	0.0000000	0.0008123	0.0007378	0.0000000	0.0000000
ENSG00000261335	0.0281935	0.0209309	0.0155940	0.0167610	0.0265995
MXRA7	1.2702206	1.2737509	1.1648910	1.2864609	1.0345948
ENSG00000277382	0.0000000	0.0000000	0.0000000	0.0000000	0.0000000
JMJD6	0.1674645	0.1760802	0.2103624	0.1268267	0.1485365
METTL23	0.3794053	0.2426467	0.1866652	0.3827857	0.2570569
SRSF2	0.5198765	0.4652711	0.4722106	0.4707821	0.3210407
MFSD11	0.1211816	0.1642012	0.1343797	0.1142584	0.2838678
LINC02080	0.0000000	0.0008033	0.0000000	0.0000000	0.0000000
ENSG00000287836	0.0000000	0.0000000	0.0000000	0.0000000	0.0000000
LINC00868	0.0000000	0.0000000	0.0000000	0.0000000	0.0000000
MGAT5B	0.0608996	0.1068492	0.0701271	0.0279096	0.2143116

ENSG00000267568	0.0118563	0.0277356	0.0195768	0.0087968	0.0203031
SNHG20	0.0585381	0.0455868	0.0544171	0.0688633	0.0498496
SEC14L1	0.7509891	0.5906657	0.4913808	0.8870764	0.5534932
SEPTIN9-DT	0.0000000	0.0000000	0.0000000	0.0000000	0.0000000
SEPTIN9	0.3166196	0.2846475	0.2263282	0.4164203	0.3603028
ENSG00000267665	0.0007431	0.0000000	0.0050457	0.0000000	0.0000000
ENSG00000267506	0.0018134	0.0018721	0.0008115	0.0000000	0.0064756
ENSG00000285535	0.0021355	0.0000000	0.0098473	0.0000000	0.0082720
LINC01973	0.0000000	0.0059774	0.0000000	0.0000000	0.0000000
TNRC6C	0.4023784	0.5584854	0.6823618	0.3248088	1.0502403
TMC6	0.0023288	0.0024917	0.0062575	0.0043373	0.0065198
TMC8	0.0012238	0.0000000	0.0000000	0.0000000	0.0095710
C17orf99	0.0000000	0.0000000	0.0000000	0.0000000	0.0015298
SYNGR2	0.1278988	0.0508010	0.0643407	0.1499095	0.0537822
TK1	0.0031728	0.0047272	0.0000000	0.0018436	0.0000000
AFMID	0.0306070	0.0269654	0.0097554	0.0235597	0.0233318
BIRC5	0.0139333	0.0260000	0.0127293	0.0171461	0.0065822
LINC01993	0.0008144	0.0015089	0.0050972	0.0041602	0.0057345
ENSG00000267737	0.0010569	0.0000000	0.0000000	0.0000000	0.0000000
SOCS3	0.0456782	0.0303043	0.0382592	0.0188317	0.0472177
SOCS3-DT	0.0003303	0.0012014	0.0016016	0.0000000	0.0000000
PGS1	0.0829982	0.1075844	0.1061081	0.0683894	0.0939272
DNAH17	0.0018020	0.0024236	0.0157481	0.0000000	0.0126488
SCAT1	0.0014357	0.0000000	0.0000000	0.0000000	0.0000000
CYTH1	0.1776573	0.1817734	0.1946061	0.1179992	0.4270730
USP36	0.0982139	0.0957160	0.0736893	0.0761422	0.2125848
ENSG00000287539	0.0000000	0.0008976	0.0000000	0.0000000	0.0000000
TIMP2	0.6669478	0.6379696	0.6454728	0.8478158	0.5779527
ENSG00000267601	0.0022656	0.0013181	0.0000000	0.0040576	0.0000000
CEP295NL	0.0008382	0.0017003	0.0016430	0.0000000	0.0025831
LGALS3BP	0.3613155	0.2530890	0.1896837	0.7416366	0.2659977
CANT1	0.1516530	0.1617841	0.1492304	0.2254013	0.1502433
C1QTNF1-AS1	0.0000000	0.0014775	0.0000000	0.0000000	0.0000000
C1QTNF1	0.0053289	0.0063929	0.0117316	0.0000000	0.0000000
ENGASE	0.0252793	0.0294325	0.0406425	0.0617672	0.0381901
RBFOX3	0.1266347	0.3300173	0.3489343	0.1236942	0.7191440
ENSG00000263278	0.0000000	0.0013616	0.0000000	0.0000000	0.0000000
CBX2	0.0270341	0.0203311	0.0374651	0.0253547	0.0236014
CBX8	0.0635856	0.0584042	0.0961643	0.0830333	0.0244372
LINC01977	0.0005200	0.0000000	0.0000000	0.0000000	0.0000000
CBX4	0.2733920	0.2597939	0.2819618	0.2679970	0.2562701
LINC01979	0.0004172	0.0000000	0.0000000	0.0000000	0.0123864
TBC1D16	0.2717641	0.2553612	0.2271203	0.2599372	0.3870468
CCDC40	0.2193211	0.1486668	0.0551314	0.2070650	0.2045697
ENSG00000289689	0.0012444	0.0022543	0.0016313	0.0040956	0.0144697
GAA	0.1194974	0.0778216	0.0605407	0.1971865	0.1179444
EIF4A3	0.5676744	0.4989373	0.4458836	0.5288236	0.3895043
ENSG00000275479	0.0071211	0.0087176	0.0025124	0.0098818	0.0053247
CARD14	0.0000000	0.0085536	0.0040932	0.0100474	0.0253965
ENSG00000262580	0.0244833	0.0084615	0.0148669	0.0184674	0.0209064
SGSH	0.0458794	0.0375399	0.0224362	0.0742453	0.0183913
SLC26A11	0.1634788	0.1582292	0.1990475	0.2215848	0.1936389
RNF213	0.1840837	0.1383482	0.1102320	0.2588676	0.2801789

ENSG00000276863	0.0028869	0.0078916	0.0029036	0.0000000	0.0000000
RNF213-AS1	0.0661689	0.0905129	0.0730783	0.0548588	0.2782849
ENDOV	0.1517539	0.1906103	0.2627217	0.1645664	0.1548501
NPTX1	0.0222291	0.0089652	0.0404625	0.0015115	0.0799419
RPTOR	0.0771674	0.0825178	0.1038698	0.1124753	0.4097232
ENSG00000261924	0.0013823	0.0000000	0.0000000	0.0000000	0.0043646
CHMP6	0.1770147	0.1265399	0.1536007	0.1605978	0.1572188
ENSG00000262873	0.0000000	0.0046281	0.0007835	0.0024060	0.0054098
BAIAP2-DT	0.1270369	0.1099521	0.1223416	0.1403427	0.0943913
BAIAP2	0.3168239	0.2439864	0.2673338	0.3234802	0.2584990
AATK	0.0517577	0.1068708	0.1029340	0.0507660	0.1869681
PVALEF	0.0037967	0.0126169	0.0133736	0.0000000	0.0160133
CEP131	0.0752548	0.0611757	0.0383010	0.0405230	0.0836506
TEPSIN	0.0171885	0.0195703	0.0260663	0.0204928	0.0227821
NDUFAF8	0.8580737	0.7925116	0.6821548	0.9526109	0.6680426
SLC38A10	0.1490137	0.1123123	0.0516900	0.2771782	0.1768024
ENSG00000276101	0.0023305	0.0131579	0.0045951	0.0033765	0.0033679
LINC00482	0.0012429	0.0000000	0.0000000	0.0000000	0.0023981
TMEM105	0.0015208	0.0000000	0.0000000	0.0060085	0.0092013
RENO1	0.0099240	0.0176717	0.0095392	0.0102682	0.0152779
ENSG00000263154	0.0000000	0.0000000	0.0000000	0.0000000	0.0000000
LINC03048	0.0043836	0.0044274	0.0000000	0.0016951	0.0190341
ENSG00000274833	0.0008729	0.0000000	0.0000000	0.0000000	0.0000000
ENSG00000262877	0.0000000	0.0025194	0.0000000	0.0000000	0.0000000
BAHCC1	0.0631185	0.0871415	0.1030812	0.0430163	0.2044811
LINC01971	0.0201985	0.0315617	0.0652535	0.0182742	0.0374923
ACTG1	3.7878770	3.7815271	3.8592756	3.7198655	3.3706336
ENSG00000229848	0.0084412	0.0056086	0.0067124	0.0098576	0.0252274
ENSG00000289182	0.0054319	0.0059567	0.0000000	0.0000000	0.0000000
FSCN2	0.0044042	0.0035096	0.0040374	0.0020019	0.0061370
FAAP100	0.0759567	0.0626479	0.0794801	0.0525173	0.0787407
NPLOC4	0.1431487	0.1305848	0.1310642	0.1356206	0.2401244
TSPAN10	0.0366811	0.0354457	0.0264650	0.0336297	0.0092964
PDE6G	0.0015891	0.0008472	0.0000000	0.0042766	0.0044858
OXLD1	0.1845223	0.1449860	0.1639849	0.1830484	0.1208744
CCDC137	0.2208346	0.1966500	0.2036994	0.2673069	0.1709632
ARL16	0.4189848	0.4700173	0.4865595	0.4353798	0.3678136
HGS	0.1834630	0.2021794	0.1604005	0.1803361	0.1442812
ENSG00000275902	0.0007181	0.0016755	0.0000000	0.0049937	0.0070802
ENSG00000262049	0.0314879	0.0297440	0.0220949	0.0370861	0.0305325
MRPL12	0.4244038	0.4796153	0.5255295	0.4406710	0.3269224
SLC25A10	0.0282361	0.0239878	0.0393137	0.0229574	0.0056180
MCRIP1	0.8445924	0.8980784	0.8899539	0.7502178	0.6992772
PPP1R27	0.0011007	0.0000000	0.0000000	0.0000000	0.0047101
P4HB	1.0303239	0.7399350	0.5514335	1.1530039	0.6759819
ENSG00000262831	0.0089907	0.0026365	0.0000000	0.0144841	0.0036228
ENSG00000262413	0.0088749	0.0070739	0.0092327	0.0102009	0.0000000
ARHGDIA	0.6074058	0.5077934	0.5497788	0.6025463	0.3831037
ENSG00000263731	0.0263347	0.0284472	0.0209197	0.0135582	0.0181801
ENSG00000263859	0.0008144	0.0000000	0.0000000	0.0000000	0.0000000
ALYREF	0.4550012	0.4141025	0.3579371	0.4506070	0.3065130
ANAPC11	1.1026450	1.0847611	1.1077428	1.1135233	0.9098282
NPB	0.1499656	0.2249601	0.1911595	0.1544462	0.1404554

PCYT2	0.1592524	0.1288986	0.1845297	0.2338543	0.1284932
SIRT7	0.0520528	0.0405779	0.0242166	0.0358501	0.0564264
MAFG	0.3367889	0.2774594	0.3037863	0.3007819	0.1916299
MILIP	0.0546266	0.0477081	0.0673309	0.0434105	0.0346707
PYCR1	0.2567407	0.1860863	0.1869326	0.2936293	0.1906579
MYADML2	0.0008070	0.0010211	0.0000000	0.0000000	0.0000000
ENSG00000235296	0.0066866	0.0117704	0.0109937	0.0015713	0.0041191
NOTUM	0.0013979	0.0006253	0.0059341	0.0000000	0.0000000
ASPSCR1	0.1195882	0.1051486	0.1241527	0.1243095	0.1396131
CENPX	0.3765673	0.4106967	0.4371590	0.3269689	0.2843415
LRRC45	0.0425450	0.0340380	0.0353356	0.0255329	0.0426025
RAC3	0.7000229	1.1029815	1.3199106	0.7284237	0.8071748
DCXR	0.6683030	0.7101420	0.7888230	0.7086014	0.4836959
DCXR-DT	0.0021572	0.0011801	0.0000000	0.0034202	0.0000000
RFNG	0.2643536	0.2053270	0.1845155	0.3026115	0.1769139
GPS1	0.3078337	0.2981566	0.2753653	0.3247654	0.2357632
DUS1L	0.1150567	0.1234772	0.1383950	0.1091008	0.1870381
FASN	0.1603259	0.2022931	0.2158987	0.1155596	0.2290404
CCDC57	0.1461422	0.1027624	0.0609884	0.1016254	0.2938083
ENSG00000264548	0.0000000	0.0000000	0.0000000	0.0000000	0.0000000
SLC16A3	0.4378399	0.4626188	0.4585121	0.5367756	0.4064114
CSNK1D	0.5106881	0.4591606	0.4831760	0.5119474	0.4911613
ENSG00000275888	0.0008382	0.0010611	0.0023697	0.0021940	0.0163898
ENSG00000287737	0.0251826	0.0414446	0.0472555	0.0127529	0.0633185
LINC01970	0.0068924	0.0015868	0.0022883	0.0019455	0.0000000
ENSG00000260563	0.0113098	0.0219732	0.0277736	0.0000000	0.0068176
CD7	0.0000000	0.0015882	0.0000000	0.0000000	0.0059744
SECTM1	0.0009337	0.0019617	0.0027049	0.0000000	0.0000000
UTS2R	0.0000000	0.0010973	0.0000000	0.0000000	0.0024211
ENSG00000260011	0.0007131	0.0024987	0.0012177	0.0000000	0.0095224
OGFOD3	0.1959491	0.1355598	0.0797956	0.2470696	0.1403486
ENSG00000264812	0.0000000	0.0014265	0.0019184	0.0000000	0.0000000
HEXD	0.1251714	0.1139303	0.1170302	0.1359917	0.1777299
HEXD-IT1	0.0000000	0.0024698	0.0000000	0.0022536	0.0155978
CYBC1	0.1466870	0.1276468	0.1473536	0.1460366	0.1432890
ENSG00000287193	0.0000000	0.0019856	0.0022858	0.0000000	0.0024001
NARF-AS2	0.0118607	0.0073810	0.0000000	0.0059719	0.0000000
NARF	0.4555086	0.4928571	0.5891236	0.3553311	0.5148363
NARF-IT1	0.0000000	0.0000000	0.0026443	0.0000000	0.0038438
FOXK2	0.1694400	0.2314160	0.2813266	0.1767315	0.4015998
ENSG00000261845	0.0565902	0.0727598	0.0654706	0.0543475	0.0980667
WDR45B	0.7489237	0.6976100	0.7085407	0.6866497	0.6339247
RAB40B	0.1520732	0.2058748	0.2068994	0.1764082	0.1633025
ENSG00000263063	0.0075742	0.0089580	0.0118807	0.0020158	0.0218232
FN3KRP	0.2072074	0.1720422	0.1806005	0.1938288	0.1341049
ENSG00000263321	0.0000000	0.0009434	0.0000000	0.0000000	0.0000000
FN3K	0.1084991	0.1123871	0.1268949	0.1181558	0.1093852
ENSG00000262410	0.0010256	0.0026139	0.0020693	0.0020601	0.0000000
TBCD	0.1953232	0.1920055	0.1849713	0.2522857	0.5072942
ZNF750	0.0000000	0.0000000	0.0000000	0.0000000	0.0058304
ENSG00000262663	0.0000000	0.0000000	0.0000000	0.0000000	0.0086579
B3GNTL1	0.0633077	0.0588193	0.0445927	0.0523559	0.0627814
ENSG00000262339	0.0000000	0.0000000	0.0000000	0.0026362	0.0000000

METRNL	0.7283678	0.5359622	0.4280120	0.7902371	0.4619175
ENSG00000274370	0.0077018	0.0034580	0.0029693	0.0000000	0.0000000
ENSG00000261888	0.0119676	0.0075204	0.0046203	0.0170473	0.0070802
ENSG00000262898	0.0065317	0.0092186	0.0101391	0.0032264	0.0045380
TUBB8B	0.0000000	0.0000000	0.0000000	0.0000000	0.0000000
ROCK1P1	0.0158024	0.0156631	0.0045834	0.0087247	0.0041692
USP14	0.8627113	0.8258809	0.9000733	0.8299174	0.7900868
THOC1	0.1825364	0.1321527	0.0903992	0.1769412	0.3280332
THOC1-DT	0.0044682	0.0029663	0.0049416	0.0000000	0.0000000
ENSG00000264514	0.0041130	0.0000000	0.0000000	0.0154092	0.0055697
COLEC12	0.8655283	0.4952150	0.3474114	1.3210529	0.4906319
ENSG00000265477	0.0000000	0.0000000	0.0000000	0.0000000	0.0000000
CLUL1	0.0312765	0.0673251	0.0441132	0.0392012	0.0750749
TYMSOS	0.0463870	0.0457643	0.0403233	0.0336332	0.0488482
ENSG00000266456	0.0054426	0.0024039	0.0077509	0.0000000	0.0132135
TYMS	0.1404060	0.1050929	0.0631542	0.1675591	0.0756571
ENOSF1	0.1157495	0.0690284	0.0473113	0.1356473	0.1640076
YES1	0.6020469	0.3895454	0.3118283	0.5942138	0.3994595
ENSG00000264635	0.0000000	0.0000000	0.0000000	0.0000000	0.0000000
ENSG00000273355	0.0273691	0.0288246	0.0284664	0.0165677	0.0189522
ENSG00000265671	0.0000000	0.0010081	0.0000000	0.0000000	0.0000000
ENSG00000265179	0.0044941	0.0198353	0.0322028	0.0023531	0.0299569
ADCYAP1	0.0448769	0.0939864	0.2986848	0.0434727	0.1201623
ENSG00000272461	0.0004381	0.0018449	0.0000000	0.0000000	0.0072100
ENSG00000263551	0.0096720	0.0183591	0.0107428	0.0134705	0.0489375
LINC00470	0.0000000	0.0014800	0.0000000	0.0000000	0.0000000
ENSG00000266602	0.0009037	0.0000000	0.0000000	0.0000000	0.0000000
ENSG00000263745	0.0036680	0.0058205	0.0038573	0.0021257	0.0200622
ENSG00000286975	0.0000000	0.0000000	0.0000000	0.0000000	0.0000000
METTL4	0.1116010	0.1083616	0.1180681	0.1011208	0.1250683
ENSG00000266783	0.0005052	0.0011050	0.0020945	0.0000000	0.0000000
NDC80	0.0132517	0.0147618	0.0134377	0.0078970	0.0045817
SMCHD1	0.7524768	0.6766893	0.7253301	0.6428100	0.9090704
ENSG00000266049	0.0116572	0.0099111	0.0057527	0.0031874	0.0616637
EMILIN2	0.0016840	0.0008843	0.0007553	0.0000000	0.0043675
LPIN2	0.4536155	0.4758772	0.7516478	0.4756719	0.5546944
ENSG00000265907	0.0093738	0.0156359	0.0298430	0.0027112	0.0519687
ENSG00000266397	0.0000000	0.0000000	0.0000000	0.0000000	0.0068372
MYOM1	0.0495379	0.0545064	0.1485246	0.0369121	0.0848709
ENSG00000265399	0.0022251	0.0006262	0.0065986	0.0000000	0.0109441
ENSG00000272688	0.0197514	0.0120125	0.0226138	0.0128935	0.0088023
MYL12A	0.7610813	0.5135893	0.4675108	0.8399698	0.4497304
MYL12-AS1	0.0048256	0.0045060	0.0000000	0.0022208	0.0039666
MYL12B	1.2428676	1.3179914	1.4382021	1.2410381	0.9933249
ENSG00000266578	0.0016386	0.0024159	0.0000000	0.0035879	0.0000000
TGIF1	0.3349269	0.1903449	0.1014815	0.3337736	0.1858615
DLGAP1	0.3918995	0.4399253	0.3770585	0.3703265	1.3541985
DLGAP1-AS1	0.1190124	0.0787976	0.0330816	0.1009798	0.0534686
DLGAP1-AS2	0.0382867	0.0293325	0.0209945	0.0372309	0.0030416
ENSG00000266401	0.0009386	0.0000000	0.0000000	0.0000000	0.0049754
DLGAP1-AS3	0.0000000	0.0000000	0.0000000	0.0000000	0.0083645
DLGAP1-AS4	0.0010530	0.0007185	0.0000000	0.0000000	0.0243554
DLGAP1-AS5	0.0037834	0.0021038	0.0026409	0.0044365	0.0149821

ENSG00000266268	0.0000000	0.0051240	0.0011166	0.0000000	0.0238387
AKAIN1	0.0035215	0.0098515	0.0083011	0.0072054	0.0000000
ENSG00000285575	0.0021243	0.0022730	0.0000000	0.0047682	0.0000000
ENSG00000265091	0.0015225	0.0068444	0.0043640	0.0010066	0.0126302
LINC00526	0.0759092	0.0835469	0.0945215	0.0833250	0.0419402
LINC00667	0.3315183	0.3845641	0.3693884	0.2959837	0.2922111
ZBTB14	0.1295851	0.1108456	0.1568492	0.0924127	0.1065169
LINC02974	0.0020234	0.0029595	0.0031524	0.0084207	0.0254325
EPB41L3	0.7466265	0.7310426	0.5753102	0.6965891	0.7675957
ENSG00000265316	0.0000000	0.0000000	0.0000000	0.0000000	0.0000000
ENSG00000264000	0.0000000	0.0000000	0.0000000	0.0000000	0.0014252
TMEM200C	0.1306782	0.0770670	0.0686964	0.1363580	0.0875162
ENSG00000264449	0.0704912	0.0590071	0.0228183	0.0804411	0.0515060
ENSG00000266846	0.0033141	0.0018344	0.0021947	0.0035629	0.0037978
L3MBTL4	0.0202471	0.0221188	0.0246597	0.0251064	0.0802744
LINC01387	0.0004988	0.0018748	0.0000000	0.0000000	0.0097344
ARHGAP28-AS1	0.0175605	0.0111230	0.0088569	0.0237716	0.0102493
ARHGAP28	0.0950411	0.1318438	0.1575384	0.0548975	0.3600090
ENSG00000287834	0.0000000	0.0000000	0.0010352	0.0000000	0.0014182
LINC00668	0.0000000	0.0000000	0.0000000	0.0000000	0.0000000
LAMA1	0.0318731	0.0500651	0.0297573	0.0411208	0.0550920
PTPRM	0.3452417	0.3878993	0.4124179	0.2944740	0.9065072
ENSG00000264596	0.0000000	0.0000000	0.0000000	0.0000000	0.0000000
ENSG00000265413	0.0000000	0.0000000	0.0000000	0.0000000	0.0190146
RAB12	0.3621315	0.3863857	0.3811790	0.3385512	0.4136039
GACAT2	0.0002606	0.0000000	0.0000000	0.0021849	0.0039906
MTCL1	0.0722412	0.0768220	0.1027556	0.0579822	0.2330130
ENSG00000272788	0.0000000	0.0007025	0.0000000	0.0047879	0.0000000
ENSG00000288939	0.0000000	0.0000000	0.0000000	0.0022040	0.0082152
NDUFV2	0.9716738	1.0570997	1.0674434	0.9313107	0.8641387
ENSG00000263847	0.0008963	0.0012480	0.0027191	0.0000000	0.0334677
NDUFV2-AS1	0.0459942	0.0931226	0.0541887	0.0665184	0.0826793
ANKRD12	1.1321463	1.3951312	1.6554251	1.0391861	1.4425313
ENSG00000273284	0.0010015	0.0078575	0.0049598	0.0022963	0.0016281
TWSG1-DT	0.0184316	0.0169325	0.0299035	0.0135892	0.0131561
TWSG1	0.2858233	0.2186599	0.1923961	0.3263414	0.1976865
ENSG00000287509	0.0032609	0.0000000	0.0000000	0.0033205	0.0000000
RALBP1	0.7271769	0.6745711	0.6662403	0.7089340	0.5414261
ENSG00000266805	0.0000000	0.0025444	0.0000000	0.0000000	0.0036382
PPP4R1	0.3070074	0.2819076	0.2094302	0.2694140	0.3590878
ENSG00000266541	0.0000000	0.0000000	0.0000000	0.0000000	0.0000000
PPP4R1-AS1	0.0190616	0.0189842	0.0180147	0.0169503	0.0000000
RAB31	0.3683427	0.2645890	0.2319181	0.4973909	0.1846725
VAPA	1.4796821	1.4971663	1.5825932	1.4060321	1.3241709
ENSG00000265554	0.0447453	0.0338328	0.0118890	0.0504758	0.0345559
APCDD1	0.4075144	0.2322470	0.1130368	0.4493663	0.1528083
NAPG	0.5683737	0.6710930	0.7818400	0.5941455	0.6057194
ENSG00000265728	0.0000000	0.0000000	0.0000000	0.0026542	0.0000000
PIEZO2	0.0144699	0.0487466	0.0158413	0.0195782	0.0786970
GNAL	0.3975581	0.7497212	0.8891437	0.3106620	0.7138964
ENSG00000286812	0.0000000	0.0000000	0.0018021	0.0000000	0.0000000
CHMP1B	0.4379917	0.3427436	0.3377763	0.4089783	0.3530101
CHMP1B-AS1	0.0052234	0.0049711	0.0131536	0.0010047	0.0061857

MPPE1	0.1266806	0.1188116	0.1325220	0.1699454	0.1295952
ENSG00000273141	0.0089993	0.0108314	0.0034795	0.0020830	0.0062126
ENSG00000267079	0.0002840	0.0019398	0.0000000	0.0044469	0.0022313
IMPA2	0.0244359	0.0135198	0.0011751	0.0121934	0.0099910
ENSG00000267069	0.0040439	0.0050628	0.0000000	0.0079167	0.0000000
TUBB6	0.2870377	0.1624421	0.0896098	0.3520828	0.1364420
AFG3L2	0.5116236	0.5242307	0.5487598	0.5280572	0.3656465
PRELID3A	0.0573098	0.0434407	0.0445007	0.0635470	0.0655475
ENSG00000267108	0.0024563	0.0007551	0.0000000	0.0000000	0.0048181
ENSG00000267199	0.0016835	0.0014775	0.0092647	0.0021003	0.0133962
SPIRE1	0.5092243	0.5559631	0.5883683	0.4561959	0.8117871
PSMG2	0.4927108	0.5068927	0.5417082	0.4600270	0.4827264
CEP76	0.0788137	0.0666958	0.1111678	0.0652027	0.0730992
ENSG00000267249	0.0054155	0.0054301	0.0109203	0.0015632	0.0020838
PTPN2	0.2409503	0.1893168	0.1773644	0.2223786	0.2270854
SEH1L	0.1048253	0.1348317	0.1479828	0.1206430	0.1261882
CEP192-DT	0.0076814	0.0125507	0.0134408	0.0051936	0.0141383
CEP192	0.2231216	0.1916309	0.1485188	0.1686412	0.2716338
LDLRAD4	0.2759881	0.2359876	0.2334077	0.2246383	0.7554381
LDLRAD4-AS1	0.0012343	0.0000000	0.0000000	0.0000000	0.0000000
ENSG00000267694	0.0000000	0.0000000	0.0000000	0.0000000	0.0000000
ENSG00000267136	0.0000000	0.0000000	0.0000000	0.0000000	0.0038318
ENSG00000267503	0.0000000	0.0000000	0.0000000	0.0000000	0.0000000
FAM210A	0.3082123	0.2816304	0.2613696	0.3461438	0.2669688
RNMT	0.8556947	0.8649867	1.0128974	0.7795396	0.8433092
ENSG00000288839	0.0041703	0.0000000	0.0044407	0.0044916	0.0111499
ZNF519	0.0598479	0.0916666	0.0982232	0.0283991	0.1116934
ENSG00000290461	0.0033229	0.0078217	0.0053519	0.0000000	0.0110578
ENSG00000283294	0.0017627	0.0029084	0.0007826	0.0000000	0.0000000
ENSG00000286593	0.0073910	0.0275643	0.0377056	0.0199110	0.0569561
ANKRD30B	0.0174801	0.0189321	0.0117236	0.0092033	0.0546719
ENSG00000265737	0.0077326	0.0125078	0.0089810	0.0170812	0.0040780
ENSG00000287723	0.0000000	0.0000000	0.0000000	0.0000000	0.0000000
ENSG00000286293	0.0000000	0.0000000	0.0000000	0.0000000	0.0111274
ROCK1	0.4347433	0.3730726	0.2447482	0.4713200	0.6227135
GREB1L-DT	0.0023663	0.0076336	0.0015287	0.0045388	0.0038629
GREB1L	0.1198829	0.1280603	0.1138971	0.0930732	0.5022990
ENSG00000265751	0.0048716	0.0013921	0.0000000	0.0068008	0.0439789
ESCO1	0.4432065	0.3312225	0.3647568	0.3556230	0.4067586
SNRPD1	0.7787673	0.7306794	0.8677946	0.7697927	0.5882259
ABHD3	0.0248370	0.0352374	0.0216101	0.0852647	0.1111331
ENSG00000265656	0.0000000	0.0000000	0.0000000	0.0000000	0.0000000
MIB1	0.5342554	0.5571511	0.4673848	0.4351228	0.6830086
MIR133A1HG	0.0000000	0.0000000	0.0000000	0.0000000	0.0000000
ENSG00000264149	0.0000000	0.0000000	0.0000000	0.0000000	0.0000000
LINC01900	0.0000000	0.0000000	0.0017210	0.0000000	0.0000000
GATA6-AS1	0.0000000	0.0000000	0.0017584	0.0000000	0.0000000
GATA6	0.0032184	0.0026298	0.0000000	0.0000000	0.0000000
ENSG00000265943	0.0023745	0.0000000	0.0015150	0.0000000	0.0222970
RBBP8	0.2012210	0.2103297	0.2132523	0.2321821	0.2051003
CABLES1	0.0156258	0.0236436	0.0101024	0.0253201	0.0684040
TMEM241	0.0712283	0.1013858	0.1165623	0.0538141	0.1491079
RIOK3	1.0003381	1.0253766	1.0409039	0.8929974	0.8323653

RMC1	0.0991262	0.1129565	0.1452004	0.0726575	0.1025833
NPC1	0.0881654	0.0841049	0.0807642	0.1325158	0.1531981
ANKRD29	0.1572898	0.2300670	0.1965079	0.0515396	0.1844482
LAMA3	0.0136037	0.0235049	0.0155369	0.0057368	0.0683382
LINC02958	0.0089968	0.0051181	0.0028098	0.0134540	0.0044624
TTC39C	0.1682227	0.2540807	0.2466895	0.1426109	0.3477853
TTC39C-AS1	0.0040600	0.0055548	0.0056133	0.0127045	0.0000000
ENSG00000265204	0.0000000	0.0000000	0.0000000	0.0016020	0.0065379
ENSG00000264924	0.0000000	0.0000000	0.0055668	0.0000000	0.0000000
CABYR	0.0674302	0.1057801	0.1259955	0.0386395	0.1289531
ENSG00000265750	0.0022118	0.0000000	0.0000000	0.0000000	0.0000000
OSBPL1A	0.7291120	0.6719217	0.5551247	0.7753086	0.6004671
ENSG00000264365	0.0018782	0.0014576	0.0043158	0.0000000	0.0000000
ENSG00000273321	0.0020900	0.0018870	0.0000000	0.0000000	0.0000000
IMPACT	0.4416805	0.2894139	0.3083517	0.4539537	0.2796716
HRH4	0.0032476	0.0000000	0.0000000	0.0036457	0.0000000
ENSG00000264695	0.0023776	0.0000000	0.0000000	0.0000000	0.0000000
ENSG00000287611	0.0000000	0.0000000	0.0000000	0.0000000	0.0000000
ENSG00000266489	0.0000000	0.0000000	0.0021877	0.0000000	0.0000000
LINC01915	0.0014103	0.0000000	0.0000000	0.0000000	0.0000000
ENSG00000266573	0.0069963	0.0063053	0.0027164	0.0000000	0.0313704
LINC01894	0.0000000	0.0000000	0.0000000	0.0000000	0.0042946
ZNF521	0.1795642	0.2331566	0.1844327	0.1174673	0.4556199
ENSG00000285595	0.0005628	0.0009363	0.0000000	0.0000000	0.0028551
SS18	0.3506529	0.2575151	0.1565312	0.3009073	0.3343654
PSMA8	0.0016830	0.0009847	0.0017904	0.0088665	0.0052465
TAF4B	0.0102183	0.0090276	0.0172143	0.0101764	0.0497842
KCTD1	0.2713545	0.1703786	0.1806557	0.3152166	0.2641439
ENSG00000277534	0.0120463	0.0089295	0.0021716	0.0073619	0.0000000
ENSG00000275805	0.0020640	0.0000000	0.0007539	0.0000000	0.0000000
AQP4-AS1	0.0153605	0.0038062	0.0175337	0.0062732	0.0317885
ENSG00000263677	0.0127204	0.0074305	0.0032421	0.0217895	0.0000000
AQP4	0.1580746	0.1047022	0.0556534	0.8635628	0.2744432
CHST9	0.1529628	0.0551675	0.0574615	0.4715928	0.0720647
ENSG00000264151	0.0000000	0.0000000	0.0000000	0.0000000	0.0079237
ENSG00000227279	0.0047377	0.0094938	0.0094860	0.0034799	0.0202241
CDH2	1.4613415	1.1353739	1.0449430	1.8813255	1.3082331
ENSG00000274578	0.0024880	0.0057836	0.0025371	0.0000000	0.0017501
DSC3	0.0532265	0.0196189	0.0122042	0.1493944	0.0387555
DSC2	0.3040306	0.1283469	0.1093570	0.4727944	0.0916293
DSCAS	0.0084693	0.0144931	0.0023781	0.0097980	0.0000000
DSG2	0.3239487	0.1813032	0.1192070	0.5648311	0.2042330
DSG2-AS1	0.0015597	0.0000000	0.0000000	0.0000000	0.0000000
TTR	1.0322189	0.6932894	0.4601660	0.6402070	0.5227264
B4GALT6	0.2689426	0.2807136	0.2562006	0.3190839	0.3807657
ENSG00000259985	0.0287936	0.0334301	0.0294290	0.0119703	0.0030127
TRAPPC8	0.1894083	0.1867971	0.1874015	0.1825961	0.3505181
ENSG00000263924	0.0000000	0.0000000	0.0000000	0.0000000	0.0034590
ENSG00000276282	0.0017471	0.0000000	0.0000000	0.0000000	0.0000000
ENSG00000263823	0.0046193	0.0086097	0.0079853	0.0090107	0.0048079
RNF125	0.0131717	0.0227196	0.0294411	0.0118839	0.0110987
ENSG00000265008	0.0089148	0.0072751	0.0058033	0.0087008	0.0000000
RNF138	0.2486584	0.2044867	0.2523012	0.1985191	0.2569165

ENSG00000263393	0.0009717	0.0000000	0.0000000	0.0000000	0.0000000
GAREM1	0.2328684	0.2731153	0.2515783	0.1951086	0.5101240
MEP1B	0.0020911	0.0013023	0.0028524	0.0000000	0.0101071
ENSG00000263904	0.0000000	0.0008200	0.0000000	0.0000000	0.0000000
ENSG00000264982	0.0000000	0.0016779	0.0000000	0.0000000	0.0000000
ENSG00000285095	0.0043050	0.0034154	0.0046131	0.0088902	0.0320225
KLHL14	0.0028828	0.0089187	0.0118848	0.0061329	0.0306893
ENSG00000228835	0.0000000	0.0000000	0.0000000	0.0000000	0.0000000
CCDC178	0.0082868	0.0143202	0.0145410	0.0121801	0.0195098
ASXL3-DT	0.0000000	0.0014940	0.0000000	0.0000000	0.0109235
ASXL3	0.3807234	0.6242846	0.6641156	0.3344699	0.9967334
NOL4	0.1286364	0.1930447	0.2680319	0.0713563	0.6127800
ENSG00000278052	0.0000000	0.0016314	0.0000000	0.0000000	0.0000000
DTNA	0.8332407	0.8153590	0.6101126	0.7649222	1.2317514
ENSG00000268873	0.0000000	0.0022774	0.0000000	0.0000000	0.0049154
ENSG00000278464	0.0022564	0.0000000	0.0000000	0.0000000	0.0037464
MAPRE2	0.5923990	0.8186964	1.0033480	0.4962324	0.8566742
ENSG00000274400	0.0061919	0.0113233	0.0026322	0.0126743	0.0187073
ZNF397	0.2057766	0.2453582	0.2712548	0.1833717	0.2508869
ZSCAN30	0.1900232	0.1411094	0.1486493	0.1319481	0.1490238
ENSG00000274184	0.0008070	0.0013004	0.0000000	0.0000000	0.0056926
ENSG00000268573	0.0000000	0.0045518	0.0014337	0.0000000	0.0047492
ENSG00000274918	0.0030071	0.0000000	0.0009415	0.0000000	0.0000000
ZNF24	0.6088702	0.5020778	0.4321571	0.6203288	0.3949694
ZNF396	0.0832453	0.0560439	0.0180221	0.0912938	0.0550474
ENSG00000275512	0.0000000	0.0000000	0.0000000	0.0000000	0.0000000
INO80C	0.1363776	0.1216738	0.1338364	0.1168035	0.1381561
ENSG00000289891	0.0051114	0.0020459	0.0051207	0.0000000	0.0035216
GALNT1	0.6362407	0.5378449	0.5821488	0.7403744	0.6618783
ENSG00000266965	0.0012537	0.0000000	0.0000000	0.0000000	0.0061235
ENSG00000267397	0.0038059	0.0051412	0.0021269	0.0000000	0.0029223
ENSG00000267627	0.0019596	0.0037158	0.0000000	0.0000000	0.0000000
C18orf21	0.2359257	0.2369734	0.2733831	0.2163586	0.1938598
RPRD1A	0.5128619	0.5482148	0.5417637	0.5192608	0.6409241
SLC39A6	0.4743878	0.5177020	0.6898528	0.5811455	0.5398822
ENSG00000288917	0.0057379	0.0105657	0.0205662	0.0050149	0.0073853
ELP2	0.3056455	0.2752642	0.2362120	0.2854565	0.3818199
COSMOC	0.0062752	0.0047966	0.0060946	0.0000000	0.0000000
MOCOS	0.0068810	0.0106348	0.0028524	0.0000000	0.0233240
FHOD3	0.2324971	0.3191577	0.3501056	0.1966782	0.8209710
ENSG00000286890	0.0000000	0.0000000	0.0000000	0.0000000	0.0015072
TPGS2	0.9884492	1.0158050	0.9990638	0.8974273	0.7950547
KIAA1328	0.1805002	0.1826142	0.1367695	0.1178325	0.4814038
ENSG00000267039	0.0019486	0.0000000	0.0000000	0.0000000	0.0132262
ENSG00000267651	0.0033942	0.0070737	0.0011329	0.0031073	0.0000000
CELF4	0.4944411	0.8218623	1.1635023	0.4350365	1.2216071
ENSG00000267707	0.0071731	0.0046147	0.0014518	0.0025239	0.0373660
ENSG00000285940	0.0270177	0.0326899	0.0223595	0.0162898	0.1778322
ENSG00000286716	0.0000000	0.0000000	0.0000000	0.0000000	0.0000000
MIR924HG	0.0399739	0.0195279	0.0153526	0.0407320	0.0579965
LINC01901	0.0003513	0.0000000	0.0032143	0.0000000	0.0000000
LINC01902	0.0254913	0.0142983	0.0209350	0.0467376	0.0366866
ENSG00000286328	0.0024609	0.0033806	0.0025863	0.0018874	0.0171537

KC6	0.0633921	0.0424270	0.0421335	0.0432940	0.2421623
PIK3C3	0.3064579	0.2766201	0.2123778	0.2628111	0.4467955
ENSG00000287007	0.0005301	0.0000000	0.0000000	0.0000000	0.0090480
LINC00907	0.0551636	0.0549158	0.0473307	0.0338982	0.1962537
ENSG00000286976	0.0000000	0.0036561	0.0000000	0.0000000	0.0000000
RIT2	0.0880212	0.1324142	0.3954974	0.0626611	0.2429974
ENSG00000287209	0.0078341	0.0126522	0.0358504	0.0000000	0.0109192
SYT4	0.7740491	1.2588156	1.7061314	0.6655164	1.3179190
LINC01478	0.0016826	0.0000000	0.0000000	0.0037109	0.0000000
SETBP1-DT	0.0019326	0.0045224	0.0026716	0.0000000	0.0108445
SETBP1	0.3132523	0.3621586	0.4180411	0.2803669	0.8115988
ENSG00000267101	0.0040309	0.0018525	0.0000000	0.0000000	0.0044278
SLC14A2	0.0010514	0.0088755	0.0026043	0.0000000	0.0190736
SLC14A2-AS1	0.0025217	0.0000000	0.0000000	0.0019800	0.0061414
ENSG00000285790	0.0000000	0.0006651	0.0000000	0.0000000	0.0000000
ENSG00000287943	0.0040062	0.0019506	0.0028446	0.0021445	0.0115723
ENSG00000288545	0.0005026	0.0019166	0.0000000	0.0052990	0.0094680
SLC14A1	0.0019660	0.0023618	0.0000000	0.0102473	0.0000000
EPG5	0.0609726	0.1306589	0.1078564	0.0859914	0.2545808
SIGLEC15	0.0022003	0.0010023	0.0024688	0.0000000	0.0062764
PSTPIP2	0.0155669	0.0150405	0.0139776	0.0164939	0.0236175
ATP5F1A	1.2003510	1.3090779	1.3334269	1.1972299	1.0071044
HAUS1	0.3456999	0.3373355	0.3620996	0.3117501	0.2351618
C18orf25	0.1041740	0.1270386	0.1072870	0.1101299	0.2541327
RNF165	0.2558298	0.3878767	0.4980099	0.2062614	0.4896197
LOXHD1	0.0000000	0.0015309	0.0000000	0.0000000	0.0000000
ST8SIA5	0.0151417	0.0100265	0.0283545	0.0070893	0.0375274
ST8SIA5-DT	0.0030731	0.0127939	0.0094165	0.0019281	0.0239773
ENSG00000274776	0.0000000	0.0015201	0.0024211	0.0000000	0.0055669
PIAS2	0.3894213	0.3822641	0.3871990	0.3375170	0.4608248
ENSG00000289168	0.0000000	0.0000000	0.0000000	0.0000000	0.0000000
KATNAL2	0.0501608	0.0527557	0.0562536	0.0376815	0.1220466
ENSG00000266957	0.0045396	0.0013224	0.0025449	0.0000000	0.0080063
HDHD2	0.5941990	0.5422038	0.5381989	0.6179759	0.4395573
IER3IP1	0.5401775	0.5621536	0.5880933	0.5444777	0.4154851
SKOR2	0.0020070	0.0116040	0.0000000	0.0049109	0.0457229
ENSG00000287673	0.0000000	0.0000000	0.0000000	0.0000000	0.0000000
MIR4527HG	0.0000000	0.0000000	0.0000000	0.0014766	0.0060503
SMAD2	0.7225819	0.7094812	0.6569337	0.6263303	0.7608019
ENSG00000269365	0.0000000	0.0000000	0.0000000	0.0000000	0.0000000
ZBTB7C	0.0300739	0.0696613	0.0511123	0.0404698	0.2586376
ZBTB7C-AS1	0.0000000	0.0000000	0.0032043	0.0000000	0.0000000
CTIF	0.2003256	0.2690524	0.2621368	0.1438143	0.6102120
ENSG00000267762	0.0000000	0.0000000	0.0000000	0.0000000	0.0000000
ENSG00000267764	0.0000000	0.0015056	0.0000000	0.0000000	0.0051919
SMAD7	0.0416249	0.0325836	0.0360827	0.0245775	0.0308081
DYM-AS1	0.0016355	0.0004313	0.0015923	0.0000000	0.0000000
DYM	0.3760971	0.4727942	0.4405648	0.3751987	0.7266216
ENSG00000265128	0.0000000	0.0024453	0.0000000	0.0000000	0.0000000
ENSG00000266696	0.0000000	0.0006757	0.0000000	0.0000000	0.0049014
C18orf32	1.4889950	1.4490019	1.5158000	1.4941750	1.1791099
ENSG00000263916	0.0002343	0.0000000	0.0000000	0.0000000	0.0033235
ENSG00000265496	0.0000000	0.0025327	0.0013121	0.0000000	0.0048432

RPL17	2.9026107	2.8431196	2.6584706	2.7711327	2.5837220
LIPG	0.0464039	0.0174870	0.0138473	0.1440494	0.0447191
ACAA2	0.5238328	0.3566949	0.2155466	0.5198972	0.3001984
SNHG22	0.0055361	0.0052998	0.0030348	0.0136382	0.0480368
MYO5B	0.0292107	0.0210481	0.0055626	0.0330096	0.0330459
CFAP53	0.5507591	0.3154470	0.2294661	0.4895839	0.2425875
MBD1	0.1681786	0.1462454	0.1333275	0.1609425	0.1472776
CXXC1	0.1342329	0.1191495	0.1257415	0.0993663	0.1184767
SKA1	0.0051220	0.0127145	0.0016660	0.0030499	0.0000000
MAPK4	0.1869682	0.1324233	0.1852823	0.2341975	0.2384211
MRO	0.0170102	0.0146819	0.0040893	0.0286904	0.0000000
ME2	0.5288679	0.6036966	0.7086959	0.5288580	0.5320090
ELAC1	0.0917257	0.1019619	0.1004660	0.1420696	0.0965533
ENSG00000289868	0.0015955	0.0000000	0.0013551	0.0000000	0.0034081
SMAD4	0.3893367	0.3653948	0.3109138	0.4195453	0.4124747
MEX3C	0.2670411	0.2356087	0.1991764	0.3892206	0.2109718
LINC01630	0.0064464	0.0068972	0.0115778	0.0030520	0.0183224
DCC	0.5074453	0.6725162	0.4647152	0.4896406	1.7868306
LINC01917	0.0009536	0.0025628	0.0017682	0.0016564	0.0151079
LINC01919	0.0000000	0.0033243	0.0000000	0.0000000	0.0000000
MBD2	0.1489975	0.1398932	0.1264224	0.1703055	0.2058612
ENSG00000277324	0.0012238	0.0053041	0.0031243	0.0036618	0.0000000
POLI	0.3248019	0.2659677	0.2538822	0.3471612	0.2726601
STARD6	0.0037914	0.0036571	0.0000000	0.0060040	0.0074103
C18orf54	0.0199854	0.0329955	0.0413078	0.0225088	0.0350102
RAB27B	0.1111237	0.2320354	0.3731913	0.0838084	0.2933131
CCDC68	0.0097066	0.0116651	0.0048926	0.0072818	0.0038927
TCF4	0.3423533	0.2914966	0.1925026	0.3681880	0.3717523
TCF4-AS1	0.0002736	0.0000000	0.0000000	0.0030891	0.0000000
TCF4-AS2	0.0000000	0.0000000	0.0000000	0.0000000	0.0000000
ENSG00000267284	0.0095394	0.0011157	0.0020528	0.0023342	0.0197358
LINC01415	0.0011957	0.0000000	0.0040217	0.0000000	0.0000000
LINC01416	0.0009176	0.0000000	0.0000000	0.0000000	0.0000000
ENSG00000267327	0.0046604	0.0091325	0.0036552	0.0000000	0.0058922
LINC03069	0.0020757	0.0027449	0.0000000	0.0000000	0.0000000
LINC01905	0.0028505	0.0038236	0.0011445	0.0086558	0.0062573
TXNL1	0.9600326	0.9714938	1.0452177	0.9506072	0.8297611
WDR7	0.1671349	0.2125538	0.2223784	0.1940774	0.5258080
WDR7-OT1	0.0020502	0.0000000	0.0000000	0.0000000	0.0048106
ST8SIA3	0.1448038	0.3407099	0.4483807	0.1129581	0.3739556
ONECUT2	0.5337890	1.0892843	1.3565263	0.4967355	1.0175973
FECH	0.1325302	0.1448188	0.1263400	0.1384447	0.1337654
ENSG00000278703	0.0016379	0.0007239	0.0038782	0.0000000	0.0065249
NARS1	1.1249444	1.0071761	1.0360666	1.1655739	0.8331290
ATP8B1-AS1	0.0037875	0.0060207	0.0000000	0.0053674	0.0000000
ENSG00000267787	0.0225698	0.0191936	0.0267451	0.0213084	0.0457331
ATP8B1	0.0196673	0.0146910	0.0081641	0.0251157	0.0290588
NEDD4L	0.6578145	0.9421858	0.9525325	0.6064527	1.2190893
ENSG00000267743	0.0000000	0.0000000	0.0000000	0.0000000	0.0031970
ENSG00000267504	0.0000000	0.0000000	0.0020297	0.0000000	0.0098846
ENSG00000267396	0.0019900	0.0024660	0.0000000	0.0000000	0.0000000
MIR122HG	0.0000000	0.0000000	0.0023309	0.0000000	0.0000000
ALPK2	0.0073819	0.0004186	0.0023230	0.0130312	0.0012783

MALT1-AS1	0.0019092	0.0020191	0.0061784	0.0000000	0.0000000
MALT1	0.1230198	0.1336320	0.1389010	0.1564997	0.3210169
ENSG00000267476	0.0010022	0.0000000	0.0038158	0.0000000	0.0139957
ENSG00000267705	0.0000000	0.0000000	0.0017189	0.0000000	0.0000000
ZNF532	0.6077935	0.5646561	0.4945981	0.6133683	0.7353742
SEC11C	1.0149961	1.0977388	1.3344100	0.9648251	0.9325281
GRP	0.0020381	0.0109102	0.0089518	0.0000000	0.0108330
CPLX4	0.0000000	0.0000000	0.0000000	0.0000000	0.0000000
LMAN1	0.8350974	0.6659289	0.6357271	1.0250255	0.6242569
CCBE1	0.0086000	0.0205064	0.0055545	0.0108951	0.1033791
PMAIP1	0.0554457	0.0415827	0.0198919	0.0551191	0.0428073
ENSG00000267686	0.0034856	0.0019203	0.0007533	0.0000000	0.0036963
ENSG00000285681	0.0000000	0.0010414	0.0025033	0.0000000	0.0063087
MC4R	0.0224730	0.0577795	0.0237345	0.0057332	0.0174574
ENSG00000267098	0.0015039	0.0095983	0.0136082	0.0057258	0.0386098
CDH20	0.0790810	0.1163835	0.0728909	0.0805868	0.3355703
ENSG00000288800	0.0012780	0.0000000	0.0000000	0.0021808	0.0091530
ENSG00000267316	0.0000000	0.0000000	0.0000000	0.0023684	0.0063180
ENSG00000267279	0.0000000	0.0000000	0.0000000	0.0000000	0.0000000
ENSG00000267175	0.0096345	0.0058146	0.0102601	0.0010951	0.0378970
RNF152	0.2607825	0.5476492	0.8409044	0.1897282	0.5661067
PIGN	0.1314014	0.1174735	0.0679708	0.1632302	0.2238011
RELCH	0.1680285	0.2604841	0.2457824	0.1814424	0.4116931
ENSG00000266900	0.0020377	0.0000000	0.0000000	0.0000000	0.0053975
TNFRSF11A	0.0000000	0.0003142	0.0000000	0.0000000	0.0242035
ZCCHC2	0.1293435	0.1465096	0.1449719	0.0942233	0.1867650
ENSG00000278017	0.0026365	0.0046536	0.0000000	0.0000000	0.0000000
PHLPP1	0.1317218	0.1065329	0.1273981	0.1634392	0.3506840
BCL2	0.2058279	0.2020241	0.1737168	0.2979556	0.3463653
KDSR	0.3560164	0.3042200	0.3009776	0.5037362	0.2903206
KDSR-DT	0.1166724	0.0989497	0.1252429	0.1142075	0.0873937
VPS4B	0.4419503	0.4298593	0.4209994	0.4430810	0.2959594
HMSD	0.0002281	0.0000000	0.0008618	0.0000000	0.0030832
ENSG00000283667	0.0178811	0.0250407	0.0139390	0.0044003	0.0119251
SERPINB8	0.0247081	0.0190285	0.0254031	0.0349514	0.0016028
LINC01924	0.0014357	0.0000000	0.0056884	0.0000000	0.0000000
CDH7	0.5721238	0.4212894	0.4946868	0.4924025	0.4960197
ENSG00000260578	0.0023525	0.0038080	0.0007847	0.0000000	0.0000000
DSEL	0.0793782	0.1350513	0.2283336	0.0987210	0.2148990
ENSG00000263424	0.0016001	0.0027933	0.0030766	0.0000000	0.0072404
DSEL-AS1	0.0058553	0.0080800	0.0098603	0.0000000	0.0294487
ENSG00000263594	0.0000000	0.0000000	0.0000000	0.0000000	0.0028694
ENSG00000287907	0.0000000	0.0000000	0.0000000	0.0000000	0.0066691
TMX3	0.2283231	0.1838487	0.1667685	0.2881538	0.2730889
CCDC102B	0.1419885	0.1611688	0.1142575	0.1692131	0.2196935
ENSG00000276092	0.0000000	0.0000000	0.0029293	0.0000000	0.0000000
ENSG00000278532	0.0056253	0.0024576	0.0104048	0.0000000	0.0000000
DOK6	0.5078638	0.8162748	0.9278299	0.4304944	1.0436159
ENSG00000265643	0.0004660	0.0010932	0.0021994	0.0000000	0.0391325
ENSG00000266840	0.0000000	0.0000000	0.0000000	0.0000000	0.0000000
CD226	0.0058163	0.0000000	0.0030699	0.0000000	0.0034730
RTTN	0.0453300	0.0433484	0.0379458	0.0314245	0.1447732
ENSG00000287646	0.0017967	0.0030023	0.0044639	0.0000000	0.0000000

SOCS6	0.2819744	0.2058033	0.2006577	0.2085814	0.2043370
LINC01909	0.0023132	0.0007547	0.0015406	0.0000000	0.0000000
LIVAR	0.0011435	0.0000000	0.0000000	0.0066286	0.0000000
ENSG00000288828	0.0038697	0.0148175	0.0057028	0.0044951	0.0294909
ENSG00000286642	0.0010393	0.0000000	0.0000000	0.0000000	0.0024821
CBLN2	0.0451007	0.1282379	0.1130083	0.0493865	0.0577090
NETO1	0.0676967	0.1256249	0.1756739	0.0655361	0.2203614
NETO1-DT	0.0254437	0.0475355	0.0691254	0.0208744	0.0491207
ENSG00000263655	0.0000000	0.0012840	0.0000000	0.0031770	0.0000000
FBXO15	0.0603366	0.0519480	0.0373585	0.0680468	0.0773866
TIMM21	0.2481199	0.2571030	0.1985533	0.3322752	0.2153302
CYB5A	0.3388406	0.3897751	0.4404452	0.3325262	0.3082681
DIPK1C	0.0000000	0.0032491	0.0000000	0.0000000	0.0000000
CNDP2	0.1913334	0.1762215	0.1842970	0.2301134	0.1956921
CNDP1	0.0020157	0.0030975	0.0063417	0.0000000	0.0199394
ZNF407-AS1	0.2305957	0.2296736	0.2303817	0.1711682	0.2209739
ZNF407	0.1416982	0.1289386	0.1193505	0.1062592	0.4805779
PTGR3	0.4135764	0.4112535	0.3363806	0.2876587	0.3296966
TSHZ1	0.3012191	0.4190651	0.2252692	0.3276759	0.4046557
ENSG00000264116	0.0007636	0.0000000	0.0000000	0.0000000	0.0000000
ENSG00000287281	0.0082055	0.0018085	0.0000000	0.0087864	0.0071214
ENSG00000264212	0.0009331	0.0000000	0.0000000	0.0000000	0.0000000
ZNF516	0.1507303	0.1384436	0.0862236	0.1008110	0.3065785
ENSG00000263982	0.0000000	0.0000000	0.0026494	0.0000000	0.0043798
ZNF516-AS1	0.0101637	0.0325795	0.0149147	0.0060805	0.0232000
ZNF516-DT	0.0140628	0.0067025	0.0083972	0.0259425	0.0000000
LINC00683	0.0452764	0.0237741	0.0268386	0.0521921	0.0365334
LINC01927	0.0006357	0.0000000	0.0000000	0.0048362	0.0000000
ZNF236-DT	0.0323313	0.0401655	0.0413225	0.0380948	0.0530586
ENSG00000278107	0.0059604	0.0061943	0.0050812	0.0026518	0.0044810
ZNF236	0.1217335	0.1333132	0.1433800	0.1071622	0.2045509
ENSG00000266844	0.0010086	0.0003142	0.0008856	0.0000000	0.0000000
MBP	0.4560365	0.5039528	0.4584447	0.5047913	0.4695721
ENSG00000275178	0.0125817	0.0023542	0.0000000	0.0172198	0.0131021
ENSG00000265844	0.0059388	0.0010796	0.0011369	0.0000000	0.0145352
GALR1	0.1329273	0.0807938	0.0767889	0.1952106	0.0917255
ENSG00000264015	0.0877794	0.0953077	0.0586802	0.1325841	0.2086144
ENSG00000264693	0.0019911	0.0011135	0.0052295	0.0009264	0.0000000
LINC01896	0.0980229	0.1718516	0.3537022	0.0470458	0.1572776
SALL3	0.0622062	0.1437409	0.3207802	0.0648411	0.2148718
ATP9B	0.2053567	0.2276609	0.1611220	0.1739555	0.5527288
ENSG00000267655	0.0024470	0.0048274	0.0100157	0.0039751	0.0070778
ENSG00000286245	0.0000000	0.0000000	0.0000000	0.0000000	0.0045825
NFATC1	0.0135623	0.0115777	0.0030985	0.0053194	0.0171998
ENSG00000286186	0.0008066	0.0000000	0.0000000	0.0000000	0.0000000
CTDP1-DT	0.0109141	0.0121780	0.0163024	0.0081790	0.0239408
ENSG00000274828	0.0684760	0.0398718	0.0508009	0.0886457	0.0434605
CTDP1	0.0441630	0.0387004	0.0432614	0.0427861	0.0608843
KCNG2	0.0013617	0.0010690	0.0011671	0.0053170	0.0096395
SLC66A2	0.2291835	0.2891644	0.2951711	0.2696993	0.2335553
ENSG00000287879	0.0017274	0.0009032	0.0000000	0.0000000	0.0000000
HSBP1L1	0.0943316	0.0690543	0.0422463	0.1023973	0.0518070
TXNL4A	0.8302060	0.8318152	0.9122924	0.7990231	0.6855450

RBFA	0.1421367	0.1625858	0.2129093	0.1775858	0.1836660
RBFADN	0.0418200	0.0443887	0.0696629	0.0464592	0.0976407
ADNP2	0.1672097	0.1745618	0.1527612	0.1450195	0.2796516
PARD6G-AS1	0.0845486	0.0781807	0.1088899	0.0838870	0.0642635
PARD6G	0.1508296	0.1855408	0.1474484	0.1864534	0.2276212
ENSG00000278000	0.0018068	0.0010823	0.0015874	0.0012028	0.0042989
ENSG00000267251	0.0096047	0.0045518	0.0145822	0.0102166	0.0092722
ENSG00000290384	0.0000000	0.0016565	0.0011221	0.0000000	0.0000000
LINC01002	0.0058978	0.0144866	0.0047537	0.0085705	0.0255943
ENSG00000282393	0.0361258	0.0375101	0.0197344	0.0073176	0.0396715
PLPP2	0.0072109	0.0081482	0.0041737	0.0000000	0.0015176
MIER2	0.0915477	0.0950894	0.1169365	0.0921067	0.0639769
THEG	0.0000000	0.0000000	0.0000000	0.0000000	0.0069594
ENSG00000286667	0.0000000	0.0019978	0.0000000	0.0000000	0.0015531
C2CD4C	0.0421880	0.0922534	0.1116671	0.0227870	0.0545434
SHC2	0.2409003	0.2697646	0.2882029	0.2241684	0.3890409
ODF3L2	0.0010917	0.0000000	0.0000000	0.0016386	0.0000000
MADCAM1	0.0168450	0.0250115	0.0276709	0.0230784	0.0473396
MADCAM1-AS1	0.0045566	0.0056260	0.0019118	0.0011268	0.0074059
TPGS1	0.2851917	0.3909563	0.3152348	0.2637303	0.2660925
CDC34	0.5563591	0.5258436	0.5220406	0.5579654	0.4747533
GZMM	0.0000000	0.0008957	0.0023309	0.0000000	0.0000000
BSG-AS1	0.0045063	0.0061668	0.0090107	0.0106504	0.0000000
BSG	1.1194804	0.9047750	0.8917400	1.2626136	0.8146123
HCN2	0.0793241	0.1123264	0.0851811	0.0494862	0.1636245
POLRMT	0.0548318	0.0637356	0.0541128	0.0495844	0.0808333
FGF22	0.0028662	0.0033627	0.0017803	0.0000000	0.0045070
RNF126	0.2913006	0.3109743	0.3237888	0.2663491	0.2193694
ENSG00000267666	0.0065062	0.0052342	0.0056365	0.0057990	0.0100720
FSTL3	0.0775392	0.0617641	0.0568156	0.0834554	0.0750792
PALM	0.4647756	0.5398721	0.4843296	0.4013708	0.5206345
MISP	0.0024620	0.0008480	0.0025783	0.0000000	0.0222545
LINC01836	0.0000000	0.0000000	0.0000000	0.0000000	0.0000000
PTBP1	0.3213091	0.1707523	0.1246763	0.3235203	0.1783323
PLPPR3	0.1224213	0.1730342	0.2040516	0.1211417	0.1676466
AZU1	0.0117340	0.0307407	0.0260953	0.0083049	0.0277105
PRTN3	0.0067578	0.0229498	0.0192897	0.0101178	0.0201098
CFD	0.0480992	0.0621753	0.0380226	0.0342658	0.0453495
MED16	0.0914626	0.0787209	0.0913303	0.1065394	0.0829333
R3HDM4	0.1750256	0.2299333	0.2930700	0.1774356	0.1884204
KISS1R	0.0348363	0.0486090	0.1133849	0.0267736	0.0500043
ARID3A	0.0623934	0.0597263	0.0401041	0.0259146	0.1519359
WDR18	0.3225207	0.2777213	0.3048483	0.3281248	0.2234102
ENSG00000266990	0.0000000	0.0000000	0.0000000	0.0000000	0.0054620
GRIN3B	0.0111120	0.0183909	0.0116612	0.0304643	0.0309123
TMEM259	0.2793715	0.2555765	0.2492813	0.3597975	0.3244536
ENSG00000274177	0.0002737	0.0000000	0.0010706	0.0000000	0.0000000
CNN2	0.0209045	0.0083071	0.0213027	0.0482815	0.0142385
ABCA7	0.0181563	0.0165985	0.0100402	0.0074598	0.0929110
ARHGAP45	0.0021914	0.0022095	0.0000000	0.0058038	0.0048214
POLR2E	0.7323011	0.7770105	0.8091308	0.7638264	0.5593461
GPX4	1.3689465	1.4371541	1.5330495	1.4169176	1.1576899
SBNO2	0.0137019	0.0105708	0.0085755	0.0121665	0.0356870

STK11	0.2553602	0.2618891	0.2154219	0.2946291	0.2198394
CBARP	0.1057168	0.1087915	0.1481050	0.1378943	0.1329117
CBARP-DT	0.0210689	0.0236251	0.0291990	0.0226762	0.0365382
ATP5F1D	1.1077642	1.2527270	1.2529953	1.1325198	0.9729246
ENSG00000288704	0.0057448	0.0063628	0.0051451	0.0050159	0.0281034
MIDN	0.4505747	0.4613265	0.4677764	0.4293917	0.4797051
CIRBP	1.8320860	1.7475027	1.7867245	1.8142736	1.5242748
CIRBP-AS1	0.0000000	0.0000000	0.0016515	0.0034589	0.0000000
FAM174C	0.1867392	0.1823685	0.1533059	0.2277878	0.0682098
EFNA2	0.1456635	0.2491753	0.2353499	0.1386030	0.1794597
ENSG00000267372	0.0034137	0.0000000	0.0060060	0.0045161	0.0044904
PWWP3A	0.3580744	0.4346082	0.5167875	0.3344559	0.4344077
NDUFS7	0.6066862	0.6451236	0.6707784	0.5537663	0.4641220
ENSG00000248015	0.0061272	0.0014825	0.0071913	0.0000000	0.0111889
GAMT	0.4666231	0.4767038	0.4681258	0.4883154	0.3639092
DAZAP1	0.2528930	0.3104623	0.3178695	0.2351574	0.2904246
RPS15	2.7084703	2.6731656	2.5131399	2.6539265	2.3900833
ENSG00000268798	0.0046747	0.0094673	0.0070067	0.0068416	0.0069145
APC2	0.0698537	0.1308222	0.1724356	0.0739816	0.2645245
ENSG00000267317	0.0245398	0.0065496	0.0018522	0.0300972	0.0215360
C19orf25	0.1875927	0.2201361	0.2164646	0.1813243	0.1583271
PCSK4	0.0483100	0.0366839	0.0180851	0.0326435	0.0456184
REEP6	0.0362139	0.0258582	0.0292338	0.0371473	0.0179968
ADAMTSL5	0.0039243	0.0009214	0.0016729	0.0027602	0.0115454
PLK5	0.0179884	0.0230459	0.0252450	0.0207633	0.0350931
ENSG00000267092	0.0040130	0.0045097	0.0081945	0.0000000	0.0000000
MEX3D	0.0813951	0.1198418	0.0834590	0.0621430	0.1203913
MBD3	0.1678889	0.1702419	0.1513401	0.1942262	0.1844225
UQCR11	1.4632314	1.5012301	1.3941678	1.4394183	1.1833185
TCF3	0.2097024	0.1333154	0.1315067	0.2036780	0.1513899
ONECUT3	0.1473785	0.2302865	0.3673097	0.1127601	0.6666211
ATP8B3	0.0059430	0.0054557	0.0040277	0.0035805	0.0083161
REXO1	0.1156516	0.1365739	0.1421044	0.1031507	0.1736983
ENSG00000267007	0.0000000	0.0000000	0.0000000	0.0021437	0.0000000
KLF16	0.0279552	0.0394110	0.0429788	0.0359022	0.0068877
ENSG00000261526	0.0180201	0.0237815	0.0111921	0.0172687	0.0119321
ABHD17A	0.5744131	0.5271943	0.5491617	0.5445722	0.4960510
SCAMP4	0.1972964	0.1198678	0.1521552	0.3560450	0.1718897
ADAT3	0.0007099	0.0014206	0.0000000	0.0000000	0.0000000
CSNK1G2	0.2947894	0.3503715	0.3891118	0.3274060	0.3549695
BTBD2	0.1751063	0.1878482	0.2086045	0.1430512	0.2252269
ENSG00000267283	0.0006399	0.0034483	0.0000000	0.0056496	0.0000000
MKNK2	0.2760250	0.2886409	0.2208565	0.2186634	0.2451738
MOB3A	0.1056617	0.0950845	0.1073306	0.1144669	0.1161559
IZUMO4	0.0186310	0.0105922	0.0072226	0.0269827	0.0423846
AP3D1	0.5086363	0.5419926	0.5713259	0.4311154	0.6569951
DOT1L	0.0768358	0.0966058	0.0915072	0.0566650	0.2699941
PLEKHJ1	0.3900112	0.4433361	0.4590410	0.3678024	0.3301355
SF3A2	0.2367938	0.2205210	0.2525222	0.1994566	0.1548277
AMH	0.0052596	0.0068170	0.0099932	0.0000000	0.0168972
JSRP1	0.0112490	0.0048496	0.0110287	0.0134989	0.0098948
OAZ1	1.8062915	1.8834748	1.9754608	1.7825945	1.5296379
PEAK3	0.0000000	0.0000000	0.0021947	0.0000000	0.0000000

LINGO3	0.0236271	0.0446652	0.0705683	0.0289185	0.0810396
LSM7	1.1045883	1.2536269	1.3641661	1.0969549	1.0129762
SPPL2B	0.1391261	0.1113556	0.0823293	0.2427675	0.2147996
TMPRSS9	0.0027090	0.0064366	0.0102839	0.0069658	0.0346570
TIMM13	0.7784353	0.7914545	0.7196933	0.7684348	0.5684242
LMNB2	0.1264451	0.1508752	0.1961489	0.1512785	0.1374682
GADD45B	0.1517673	0.0916239	0.0758887	0.1145346	0.1664660
GNG7	0.2160913	0.2372550	0.2020176	0.2709107	0.1983913
ENSG00000267412	0.0008093	0.0007695	0.0000000	0.0000000	0.0000000
ENSG00000267749	0.0000000	0.0000000	0.0000000	0.0000000	0.0000000
DIRAS1	0.1170223	0.2273502	0.2588053	0.1298027	0.1066517
ENSG00000261342	0.0070590	0.0084863	0.0081684	0.0040927	0.0000000
SLC39A3	0.2434296	0.2102850	0.2884067	0.2642264	0.1846260
SGTA	0.2704825	0.2526997	0.2919171	0.2444051	0.2543592
THOP1	0.0753459	0.0667959	0.0568915	0.0917932	0.0728941
ZNF554	0.0567731	0.0875486	0.1038815	0.0372628	0.0306265
ZNF555	0.0871680	0.1156946	0.1492473	0.0981520	0.0662933
ENSG00000287766	0.0050555	0.0090898	0.0019602	0.0000000	0.0115563
ZNF556	0.0053590	0.0049152	0.0111652	0.0053854	0.0246925
ZNF57	0.0366008	0.0295975	0.0131309	0.0272986	0.0454644
ENSG00000253392	0.0128464	0.0139054	0.0056580	0.0080261	0.0159004
ZNF77	0.0544577	0.0331053	0.0440067	0.0477062	0.0301414
ENSG00000288906	0.0025758	0.0007406	0.0024629	0.0000000	0.0000000
TLE6	0.0053131	0.0103671	0.0254434	0.0324365	0.0195422
TLE2	0.0594534	0.0590170	0.0870269	0.0830025	0.0531881
ENSG00000267469	0.0287615	0.0303959	0.0220555	0.0177287	0.0583383
TLE5	1.1491874	1.3095255	1.4801643	1.1563460	1.0820040
GNA11	0.2167936	0.1968466	0.1830837	0.2417289	0.2075782
ENSG00000267688	0.0013850	0.0013436	0.0011903	0.0029052	0.0101857
GNA15	0.0019949	0.0014675	0.0000000	0.0000000	0.0033430
NCLN	0.0541986	0.0749056	0.0585348	0.0908261	0.0841673
CELF5	0.2832742	0.5162728	0.7394243	0.2140203	0.6446204
ENSG00000267448	0.0015591	0.0018711	0.0000000	0.0000000	0.0121017
NFIC	0.4339644	0.3579644	0.2259334	0.4068891	0.3942259
SMIM24	0.0014415	0.0037393	0.0020998	0.0038486	0.0071871
DOHH	0.1280940	0.1391628	0.1783706	0.0979398	0.0896844
FZR1	0.1322296	0.1788364	0.2336552	0.1440691	0.2232442
MFSD12	0.1279341	0.1653511	0.1452433	0.1813100	0.1324268
TEKTIP1	0.0061997	0.0058827	0.0074711	0.0021437	0.0343761
ENSG00000267436	0.0088137	0.0049953	0.0060915	0.0038795	0.0326513
HMG20B	0.3343249	0.1953472	0.1632773	0.4043289	0.1965799
TBXA2R	0.0000000	0.0029412	0.0000000	0.0064603	0.0000000
CACTIN	0.0357555	0.0330186	0.0266472	0.0535442	0.0363968
PIP5K1C	0.1108471	0.1902242	0.1794413	0.1236974	0.2347448
TJP3	0.0017665	0.0026425	0.0000000	0.0000000	0.0000000
APBA3	0.0299971	0.0259247	0.0153511	0.0241149	0.0145470
MRPL54	0.4425491	0.4441908	0.3951823	0.3996236	0.3018118
MATK	0.0276302	0.0797640	0.0843670	0.0401548	0.0780698
ZFR2	0.0952640	0.2176638	0.2408104	0.0743080	0.2388138
ATCAY	0.2885274	0.5865470	0.6386742	0.2234405	0.6368241
ENSG00000289254	0.0011438	0.0030282	0.0033043	0.0118318	0.0139252
NMRK2	0.0000000	0.0000000	0.0000000	0.0000000	0.0000000
DAPK3	0.1575839	0.1466108	0.1293186	0.1739897	0.1114388

EEF2	2.2214487	2.2495746	2.0768974	2.1112043	1.9801056
PIAS4	0.1879704	0.2300486	0.2346844	0.1774409	0.2215916
ZBTB7A	0.1662035	0.1346849	0.1269470	0.1662110	0.1527906
MAP2K2	0.7079024	0.7109270	0.7608313	0.6783594	0.6119939
CREB3L3	0.0000000	0.0000000	0.0029502	0.0000000	0.0000000
SIRT6	0.0440250	0.0615941	0.0565388	0.0236785	0.0698841
ANKRD24	0.0415190	0.0741867	0.0500517	0.0394383	0.1808561
EBI3	0.0003654	0.0000000	0.0000000	0.0000000	0.0000000
YJU2	0.0852924	0.1149751	0.0846137	0.0800493	0.0582961
SHD	0.0523090	0.0927370	0.2188290	0.0342889	0.1043963
TMIGD2	0.0010072	0.0023952	0.0037520	0.0047682	0.0000000
FSD1	0.1680358	0.3061329	0.3506685	0.1787430	0.2253625
STAP2	0.0266330	0.0291396	0.0200700	0.0047857	0.0303656
ENSG00000269425	0.0000000	0.0010296	0.0000000	0.0000000	0.0074884
MPND	0.0729917	0.0998185	0.1234802	0.1000222	0.0738327
ENSG00000269807	0.0015390	0.0021893	0.0000000	0.0091675	0.0140940
ENSG00000269318	0.0000000	0.0023881	0.0000000	0.0000000	0.0000000
SH3GL1	0.3437315	0.2097688	0.2489317	0.3484369	0.2385611
ENSG00000267980	0.0047822	0.0050229	0.0012851	0.0037052	0.0055309
CHAF1A	0.0445995	0.0398785	0.0268114	0.0523341	0.0420128
UBXN6	0.3954492	0.3818806	0.3367546	0.3420696	0.2940218
ENSG00000267011	0.0110478	0.0072309	0.0075593	0.0139406	0.0208468
HDGFL2	0.5192871	0.4501456	0.5006815	0.4328522	0.3865088
PLIN4	0.0006593	0.0000000	0.0000000	0.0000000	0.0000000
PLIN5	0.0096284	0.0087276	0.0127609	0.0070557	0.0167840
LRG1	0.0013171	0.0000000	0.0000000	0.0000000	0.0102055
SEMA6B	0.0382682	0.0530063	0.0987191	0.0500432	0.1720332
TNFAIP8L1	0.0156294	0.0392419	0.0308378	0.0313301	0.0322842
MYDGF	0.6388371	0.5407288	0.5524072	0.8094727	0.4166805
ENSG00000268565	0.0033098	0.0000000	0.0028002	0.0034589	0.0000000
DPP9	0.0554606	0.0488157	0.0345517	0.0760347	0.0571552
DPP9-AS1	0.0000000	0.0024696	0.0000000	0.0000000	0.0012770
MIR7-3HG	0.0336258	0.0437458	0.0424260	0.0228482	0.1032467
ENSG00000268536	0.0012093	0.0003138	0.0000000	0.0031588	0.0000000
FEM1A	0.0865864	0.0524228	0.0539028	0.0592233	0.0717574
ENSG00000269604	0.0667464	0.0588697	0.0661436	0.0784512	0.0630980
TICAM1	0.0205454	0.0284771	0.0136219	0.0154878	0.0267054
ENSG00000267484	0.0051651	0.0000000	0.0059291	0.0000000	0.0094709
PLIN3	0.3281423	0.1891400	0.1276404	0.3771549	0.1764549
UHRF1	0.0083781	0.0046835	0.0045204	0.0217504	0.0046767
KDM4B	0.3101292	0.3407897	0.3177773	0.3044540	0.7561767
PTPRS	0.4853634	0.5664868	0.5536701	0.5024450	0.9697850
PTPRS-AS1	0.0000000	0.0011620	0.0000000	0.0000000	0.0000000
ENSG00000274447	0.0053733	0.0000000	0.0033480	0.0058749	0.0086357
TINCR	0.0417632	0.0384961	0.0337978	0.0324824	0.0347657
SAFB2	0.4078173	0.3673105	0.4069731	0.3342057	0.5091026
SAFB	0.6715481	0.6768121	0.7631708	0.5975033	0.7603143
RPL36	2.6865686	2.6449513	2.4578732	2.6034864	2.3904077
MICOS13	0.8023568	0.7244571	0.7879668	0.7821209	0.5825026
HSD11B1L	0.4174395	0.4834908	0.5915612	0.4784924	0.3798659
LONP1	0.3537925	0.2895272	0.2170187	0.3301947	0.3096725
CATSPERD	0.0098745	0.0040110	0.0030846	0.0033784	0.0055453
PRR22	0.0016153	0.0000000	0.0000000	0.0000000	0.0000000

DUS3L	0.0467191	0.0354914	0.0158721	0.0320046	0.0467601
NRTN	0.0017538	0.0026307	0.0000000	0.0088670	0.0060804
FUT6	0.0005133	0.0000000	0.0000000	0.0000000	0.0000000
FUT3	0.0006224	0.0000000	0.0000000	0.0041218	0.0041311
ENSG00000267709	0.0000000	0.0000000	0.0000000	0.0000000	0.0000000
FUT5	0.0094838	0.0230699	0.0116962	0.0230653	0.0130941
NDUFA11	1.1696692	1.2098818	1.2688545	1.2047670	0.9156173
VMAC	0.0302457	0.0162260	0.0080175	0.0185842	0.0135037
ENSG00000267571	0.0097516	0.0021331	0.0110478	0.0111619	0.0048730
CAPS	0.3493494	0.2132026	0.1338304	0.3384902	0.1617909
RANBP3	0.2043224	0.1755413	0.2081155	0.1794163	0.2337019
RANBP3-DT	0.0094398	0.0082187	0.0158789	0.0050768	0.0224434
RFX2	0.3227766	0.1838783	0.1466694	0.3648108	0.2846408
ENSG00000267262	0.0019387	0.0021061	0.0000000	0.0000000	0.0146567
ENSG00000267299	0.0025812	0.0019932	0.0000000	0.0000000	0.0000000
ACSBG2	0.0018196	0.0023465	0.0015230	0.0039294	0.0071257
MLLT1	0.6198456	0.4344752	0.3279168	0.6472239	0.3424885
ENSG00000267563	0.0000000	0.0000000	0.0000000	0.0000000	0.0000000
ACER1	0.0003289	0.0000000	0.0000000	0.0019403	0.0035216
ENSG00000269802	0.0003289	0.0009338	0.0000000	0.0016755	0.0000000
CLPP	0.6799460	0.7208214	0.7686415	0.5940337	0.5350509
ALKBH7	0.8967584	0.8771628	0.9584508	0.7849055	0.6958951
PSPN	0.0273658	0.0203229	0.0219239	0.0177296	0.0319679
GTF2F1	0.6252517	0.6886477	0.6651542	0.6491455	0.5321098
KHSRP	0.4932884	0.4829693	0.4953842	0.4834094	0.4402449
SLC25A41	0.0104247	0.0124394	0.0119868	0.0069436	0.0127827
SLC25A23	0.2795419	0.3301618	0.3301239	0.2355702	0.2594254
DENND1C	0.0039075	0.0289556	0.0226804	0.0311853	0.0191335
TUBB4A	0.2198919	0.4615056	0.6604549	0.1510543	0.2680542
ENSG00000268203	0.0006539	0.0066381	0.0028904	0.0000000	0.0072234
ENSG00000268191	0.0044217	0.0205303	0.0228374	0.0021713	0.0256463
TNFSF9	0.0144303	0.0164710	0.0290126	0.0048994	0.0160584
CD70	0.0000000	0.0000000	0.0000000	0.0000000	0.0000000
C3	0.0000000	0.0000000	0.0000000	0.0000000	0.0000000
GPR108	0.1145654	0.0864134	0.0586079	0.1095067	0.0709981
TRIP10	0.0357510	0.0329065	0.0263360	0.0511208	0.0147830
SH2D3A	0.0023986	0.0044389	0.0050248	0.0000000	0.0084767
VAV1	0.0000000	0.0000000	0.0046262	0.0000000	0.0031628
MBD3L3	0.0000000	0.0000000	0.0000000	0.0000000	0.0000000
ZNF557	0.0787985	0.0904545	0.0825104	0.0736445	0.0716919
INSR	0.2062034	0.2651022	0.2938269	0.1961528	0.5617406
ARHGEF18	0.1316170	0.0947156	0.1284821	0.1620389	0.1618811
ARHGEF18-AS1	0.0199595	0.0299406	0.0319588	0.0156366	0.0363530
PEX11G	0.0166906	0.0051635	0.0103905	0.0208011	0.0013244
TEX45	0.0090921	0.0039626	0.0000000	0.0108713	0.0201562
ZNF358	0.3366740	0.3662228	0.3769897	0.2537541	0.2902355
MCOLN1	0.0629662	0.0573499	0.0624889	0.0671711	0.0854346
PNPLA6	0.0536770	0.0685608	0.0729130	0.0765114	0.1088745
CAMSAP3	0.0715984	0.1287086	0.1394095	0.0389179	0.1366432
XAB2	0.1310728	0.1182054	0.0973750	0.1573774	0.1231104
PET100	0.4471582	0.5201077	0.5774764	0.4663581	0.3315740
STXBP2	0.0639457	0.0533949	0.0422906	0.0355779	0.0391538
TRAPPC5	0.7310051	0.7515675	0.7807504	0.7291847	0.6324788

CLEC4G	0.0051936	0.0132579	0.0111797	0.0016222	0.0055369
ENSG00000290088	0.0014856	0.0063056	0.0047015	0.0000000	0.0000000
EVI5L	0.1135118	0.1748056	0.2054526	0.1012680	0.1762609
PRR36	0.1052435	0.1549591	0.1515139	0.0928298	0.1449920
ENSG00000268120	0.0069584	0.0062501	0.0202338	0.0046232	0.0065009
LRRC8E	0.0030743	0.0000000	0.0000000	0.0000000	0.0000000
ENSG00000214248	0.0003554	0.0006794	0.0042845	0.0000000	0.0000000
MAP2K7	0.1400003	0.1545006	0.1575531	0.1132211	0.2243141
ENSG00000268149	0.0006630	0.0000000	0.0013463	0.0030842	0.0054224
TGFBR3L	0.0418498	0.0385095	0.0325426	0.0308939	0.0167559
SNAPC2	0.1285237	0.1384018	0.1282914	0.1484833	0.0884222
CTXN1	0.9685550	1.0575522	1.1411934	1.0596563	0.8118450
ENSG00000269139	0.0220599	0.0175070	0.0254813	0.0216560	0.0127409
TIMM44	0.3669412	0.3766109	0.3178402	0.3536329	0.3622219
ENSG00000286138	0.0043843	0.0063520	0.0039244	0.0044916	0.0108264
ELAVL1	0.5494213	0.5117011	0.4815623	0.5365382	0.4947545
ENSG00000269813	0.0034667	0.0009093	0.0000000	0.0031240	0.0033561
ENSG00000289255	0.0030550	0.0051023	0.0052548	0.0061540	0.0109509
ENSG00000267939	0.0532286	0.0465956	0.0362338	0.0636825	0.0274886
CCL25	0.0066531	0.0118934	0.0137442	0.0000000	0.0000000
FBN3	0.0306691	0.0242658	0.0257420	0.0000000	0.1406968
CERS4	0.1715424	0.1378744	0.1382618	0.1494423	0.2258168
ENSG00000271717	0.0042571	0.0017360	0.0000000	0.0000000	0.0181609
CD320	0.2796996	0.2455777	0.2302448	0.3988379	0.2177850
NDUFA7	1.0499853	0.9044122	0.8853493	1.0781922	0.8234949
RPS28	2.7673384	2.7538913	2.6258687	2.6584619	2.5281738
KANK3	0.0173911	0.0344436	0.0175746	0.0078234	0.0531097
ANGPTL4	0.0061929	0.0072452	0.0098181	0.0296601	0.0116094
RAB11B-AS1	0.1718945	0.1223727	0.1024350	0.1849470	0.0884396
RAB11B	0.8373980	0.8874083	0.9459636	0.8094931	0.6626175
MARCHF2	0.2944615	0.3474538	0.3604539	0.3288528	0.2975516
HNRNPM	0.9243331	0.8670748	0.9469034	0.9743776	0.7880796
PRAM1	0.0046248	0.0025056	0.0033800	0.0000000	0.0126648
ZNF414	0.1729090	0.1798459	0.2221824	0.1297725	0.1235364
MYO1F	0.0068301	0.0035981	0.0046130	0.0057758	0.0294625
ADAMTS10	0.0490464	0.0712671	0.0536154	0.0246310	0.1735902
ENSG00000267986	0.0000000	0.0013112	0.0015091	0.0067377	0.0000000
NFILZ	0.0026652	0.0000000	0.0000000	0.0000000	0.0111101
ZNF558	0.0272484	0.0276723	0.0170630	0.0183181	0.0192248
MBD3L1	0.0011746	0.0000000	0.0000000	0.0000000	0.0000000
ZNF317	0.0724338	0.0573292	0.0418428	0.0486428	0.0724351
OR7D2	0.0012419	0.0000000	0.0000000	0.0000000	0.0000000
ZNF699	0.0529202	0.0686987	0.0635441	0.0778135	0.0485329
ENSG00000267510	0.0002715	0.0000000	0.0000000	0.0000000	0.0000000
ZNF559	0.0537559	0.0400305	0.0295208	0.0528406	0.1003681
ZNF177	0.0335634	0.0472883	0.0457354	0.0357065	0.0461486
ENSG00000283108	0.0120047	0.0083541	0.0056625	0.0037300	0.0065009
ZNF266	0.0326156	0.0380114	0.0487692	0.0397609	0.1050085
ENSG00000287275	0.0067300	0.0025364	0.0052968	0.0120318	0.0338325
ZNF560	0.0027635	0.0049330	0.0027964	0.0024360	0.0138090
ENSG00000287960	0.0012901	0.0000000	0.0043088	0.0000000	0.0000000
ZNF426	0.1645391	0.1228584	0.0853404	0.1412710	0.1257102
ZNF426-DT	0.0585376	0.0493534	0.0555022	0.0441118	0.0314926

ZNF121	0.1591396	0.1638789	0.1859078	0.1271444	0.2222300
ZNF561	0.1246412	0.0976600	0.0798315	0.1243516	0.1183530
ZNF561-AS1	0.2744266	0.2236992	0.1508840	0.2340754	0.1880949
ZNF562	0.1455775	0.1736727	0.1990093	0.1436355	0.2281329
ZNF846	0.0790420	0.0598972	0.0381841	0.0958323	0.1074247
ENSG00000266950	0.0022283	0.0029119	0.0000000	0.0075063	0.0000000
FBXL12	0.1187681	0.0860297	0.0880610	0.0994400	0.0814827
UBL5	1.5191327	1.4531485	1.5099468	1.5382998	1.1299189
PIN1-DT	0.0095121	0.0046911	0.0019659	0.0014353	0.0093475
PIN1	0.6242287	0.7952642	0.8612716	0.5756847	0.5298627
OLFM2	0.4880724	0.7382368	0.8944051	0.5386033	0.6851405
COL5A3	0.0074149	0.0018084	0.0000000	0.0166624	0.0106782
SHFL	0.1477249	0.1454119	0.1499990	0.1074373	0.1603763
ANGPTL6	0.0011208	0.0054518	0.0039224	0.0037096	0.0000000
PPAN	0.0936469	0.0850423	0.0716167	0.0590868	0.0624201
P2RY11	0.0204442	0.0210312	0.0153368	0.0155943	0.0150329
EIF3G	0.6262960	0.5659401	0.5450994	0.6305371	0.4754100
DNMT1	0.3065871	0.3756405	0.4997151	0.3356353	0.4359134
S1PR2	0.0310866	0.0399334	0.0345632	0.0447275	0.0227670
MRPL4	0.3556656	0.3413372	0.4142531	0.3842628	0.2640008
LIMASI	0.0000000	0.0000000	0.0000000	0.0024755	0.0000000
ICAM1	0.0013003	0.0020210	0.0025201	0.0069239	0.0098084
ICAM5	0.0247442	0.0378941	0.0339442	0.0148433	0.0328472
ZGLP1	0.0066992	0.0135313	0.0048605	0.0069961	0.0051673
FDX2	0.1739206	0.1899319	0.1952557	0.1774250	0.1505881
RAVER1	0.0660325	0.0482912	0.0503990	0.0663851	0.0771970
ENSG00000274425	0.0091755	0.0108266	0.0140766	0.0114666	0.0000000
ICAM3	0.0277619	0.0058882	0.0052604	0.0098228	0.0049042
TYK2	0.0772699	0.0540221	0.0481634	0.0843523	0.1252695
CDC37	0.6816141	0.6841096	0.7966349	0.7357367	0.6949469
PDE4A	0.0987933	0.1826429	0.1995288	0.0744897	0.1650220
KEAP1	0.2939720	0.2728534	0.2469978	0.3300872	0.1683189
ATG4D	0.0607097	0.0626015	0.0690807	0.0387733	0.0353718
KRI1	0.0845579	0.0525701	0.0782298	0.1163054	0.0753123
CDKN2D	0.8311118	1.3968476	1.6403490	0.7627565	1.0042404
AP1M2	0.0103499	0.0047853	0.0030460	0.0064104	0.0000000
SLC44A2	0.7101893	0.4913842	0.3328112	0.9196014	0.4912936
ILF3-DT	0.4057496	0.5040651	0.5380983	0.3279273	0.3825792
ILF3	0.9537245	0.9228681	1.0111787	0.8868174	0.8948703
QTRT1	0.2116854	0.1930778	0.1995708	0.2376653	0.1849206
DNM2	0.2334747	0.2098290	0.1169484	0.2408478	0.3227533
TMED1	0.3892296	0.2276853	0.1785900	0.5103674	0.1813402
C19orf38	0.0112464	0.0076486	0.0117516	0.0108477	0.0230310
CARM1	0.1067723	0.1082191	0.1022477	0.0876655	0.2408359
YIPF2	0.2881855	0.2224638	0.1961136	0.2716670	0.2593739
TIMM29	0.1627619	0.1570303	0.1208111	0.1574669	0.0868813
SMARCA4	0.6092691	0.7482954	0.8259033	0.5763652	0.9662549
ENSG00000266936	0.0011736	0.0000000	0.0000000	0.0000000	0.0000000
LDLR	0.0793885	0.0515932	0.0356039	0.1258693	0.2050073
SPC24	0.0115171	0.0065633	0.0048578	0.0219455	0.0205233
KANK2	0.1047224	0.0583235	0.0322283	0.1307070	0.0521046
DOCK6	0.0476226	0.0565318	0.0248878	0.0253141	0.0471276
ENSG00000267082	0.0088376	0.0095619	0.0060830	0.0123289	0.0067724

ENSG00000273733	0.0047023	0.0035170	0.0090986	0.0133338	0.0089701
ANGPTL8	0.0054895	0.0093474	0.0096310	0.0045999	0.0032320
ENSG00000267174	0.0044986	0.0060929	0.0059838	0.0064576	0.0112792
TSPAN16	0.0018483	0.0000000	0.0048994	0.0047918	0.0039740
RAB3D	0.0867355	0.0910439	0.0973998	0.0526651	0.1295194
ENSG00000267576	0.0149960	0.0117432	0.0088306	0.0023733	0.0091965
TMEM205	0.4375033	0.3857525	0.3952907	0.5714514	0.2400085
CCDC159	0.0968825	0.0592576	0.0427309	0.0983936	0.0623771
PLPPR2	0.2923287	0.5163164	0.6702044	0.3227616	0.2966213
ENSG00000267277	0.0009159	0.0018482	0.0053399	0.0010951	0.0105695
SWSAP1	0.0188310	0.0250842	0.0233435	0.0147098	0.0182850
EPOR	0.0296339	0.0259777	0.0207301	0.0128723	0.0209906
RGL3	0.0023328	0.0007963	0.0011718	0.0000000	0.0040877
ODAD3	0.0583958	0.0348034	0.0275668	0.0408898	0.0116680
PRKCSH	0.5532872	0.4699449	0.3573631	0.6909585	0.3436526
ELAVL3	0.6411503	1.0492830	1.2329976	0.5447340	1.0366702
ZNF653	0.0432855	0.0421358	0.0533144	0.0304362	0.0539438
ECSIT	0.2077874	0.2709165	0.2842151	0.1898395	0.2363910
CNN1	0.0055600	0.0101460	0.0144430	0.0085406	0.0091644
ELOF1	0.3287059	0.2926473	0.2474014	0.3122840	0.2554501
ZNF627	0.1866572	0.1485399	0.1214588	0.1551358	0.1553009
ACP5	0.0000000	0.0016268	0.0009138	0.0000000	0.0000000
ENSG00000197332	0.0239020	0.0301952	0.0186684	0.0099035	0.0610223
ENSG00000289985	0.0050568	0.0036432	0.0000000	0.0037133	0.0273556
ZNF823	0.0267415	0.0300089	0.0356695	0.0174161	0.0448659
ZNF441	0.1354588	0.1133464	0.1339442	0.0771529	0.1854888
ENSG00000267646	0.0000000	0.0000000	0.0000000	0.0000000	0.0000000
ZNF491	0.0246610	0.0338635	0.0289801	0.0200947	0.0446205
ZNF440	0.0432788	0.0398990	0.0371338	0.0421518	0.0816108
ZNF439	0.0685935	0.0821017	0.0809989	0.0516793	0.2085854
ZNF69	0.0892299	0.1001614	0.1153396	0.0863047	0.2436404
ZNF700	0.0585014	0.0597575	0.0145299	0.0605776	0.1096467
ENSG00000267274	0.0036377	0.0016974	0.0028944	0.0021849	0.0090015
ZNF763	0.0295139	0.0251091	0.0240053	0.0468646	0.0349875
ZNF433-AS1	0.0700155	0.0633785	0.0750671	0.0485694	0.1025188
ZNF433	0.1242676	0.1206272	0.0867315	0.1473709	0.1277314
ZNF878	0.0027250	0.0046949	0.0000000	0.0000000	0.0046554
ZNF844	0.1601561	0.1339259	0.1216113	0.1262169	0.2362906
ENSG00000290812	0.0134289	0.0232136	0.0046297	0.0076356	0.0494861
ZNF20	0.0742892	0.0625620	0.0253531	0.0834167	0.0915150
ZNF625	0.1838046	0.2043869	0.1996795	0.1881839	0.1591015
ZNF136	0.0710685	0.0677445	0.0586824	0.0638083	0.0902629
ENSG00000291101	0.0190624	0.0213772	0.0070507	0.0276633	0.0537361
ZNF44	0.1936619	0.2249995	0.2256533	0.2754269	0.2948419
ZNF563	0.0241177	0.0144387	0.0078447	0.0319309	0.0238137
ZNF442	0.0641429	0.0511577	0.0642990	0.0557651	0.0594842
ZNF799	0.0218760	0.0083492	0.0156500	0.0137166	0.0188344
ZNF443	0.0190044	0.0151443	0.0204963	0.0134630	0.0399208
ENSG00000234848	0.0049243	0.0030794	0.0014826	0.0029052	0.0025607
ZNF709	0.1474090	0.1582011	0.1266246	0.1067104	0.2756903
ZNF564	0.0756663	0.0906980	0.0399571	0.0633585	0.0665150
LINC02926	0.0022511	0.0000000	0.0025418	0.0000000	0.0000000
ZNF490	0.0627770	0.0763052	0.0669238	0.0934605	0.1050797

ZNF791	0.2318914	0.1949848	0.1602243	0.1905200	0.2749179
MAN2B1	0.0515169	0.0346204	0.0422029	0.0713438	0.0631228
WDR83	0.1198108	0.1214935	0.1434281	0.1220174	0.1112194
WDR83OS	1.0747797	1.0408709	1.0338383	1.0500227	0.8312012
DHPS	0.4260318	0.4494106	0.5616523	0.3954269	0.4166909
ENSG00000268945	0.0033308	0.0022782	0.0013226	0.0055630	0.0028551
FBXW9	0.3381612	0.2012538	0.1753770	0.3855250	0.1160993
TNPO2	0.3394397	0.3625543	0.3612934	0.2840750	0.3008711
TRIR	0.9513684	1.0110286	1.0251992	0.9725153	0.8386272
GET3	0.3507473	0.2799824	0.2849130	0.3684240	0.2785261
BEST2	0.0011024	0.0000000	0.0000000	0.0000000	0.0000000
HOOK2	0.1006475	0.0939538	0.0997785	0.1261064	0.2064984
JUNB	0.4320200	0.3145891	0.2418101	0.3554247	0.3803976
PRDX2	1.3618552	1.3709281	1.6238455	1.4355007	1.1175940
ENSG00000267062	0.0028563	0.0030680	0.0020423	0.0053227	0.0000000
THSD8	0.0041852	0.0029433	0.0047981	0.0000000	0.0050279
RNASEH2A	0.0161784	0.0121084	0.0176594	0.0225473	0.0041876
RTBDN	0.0066177	0.0225722	0.0176194	0.0103146	0.0264052
ENSG00000267424	0.0007928	0.0024143	0.0031055	0.0000000	0.0000000
MAST1	0.1369552	0.2407110	0.2993694	0.1059751	0.5638720
DNASE2	0.2677541	0.1119191	0.0902480	0.4281852	0.1375919
ENSG00000267735	0.0000000	0.0000000	0.0006071	0.0048478	0.0000000
KLF1	0.0000000	0.0000000	0.0011420	0.0000000	0.0000000
GCDH	0.1071096	0.0502756	0.0292183	0.1011187	0.0779203
SYCE2	0.0090837	0.0093721	0.0029774	0.0034548	0.0220111
FARSA	0.2399381	0.2351444	0.2219511	0.1972035	0.1899526
CALR	1.5017793	1.1493284	1.1123937	1.9471702	1.1456483
ENSG00000267458	0.0769497	0.0425431	0.0311022	0.1081902	0.1735369
RAD23A	1.1452960	1.0579787	1.0808306	1.0876376	0.8606605
GADD45GIP1	0.8612153	0.9026777	0.9319409	0.8418078	0.6891399
DAND5	0.0029885	0.0000000	0.0000000	0.0037712	0.0041311
NFIX	0.1760458	0.1059467	0.0374951	0.2342753	0.0934020
TRMT1	0.0641773	0.0595274	0.0571386	0.0435091	0.1024012
NACC1	0.0804664	0.0905803	0.0764323	0.0689358	0.0802281
ENSG00000267512	0.0017190	0.0000000	0.0013605	0.0036184	0.0000000
STX10	0.1482943	0.1101431	0.0715403	0.1060847	0.0955845
IER2	0.3738519	0.3062838	0.2234458	0.2664134	0.3914363
CACNA1A	0.1214239	0.1516598	0.1963083	0.1133827	0.7456727
YJU2B	0.3056395	0.2933251	0.2417270	0.3090106	0.2415481
MRI1	0.1254022	0.1655876	0.1096351	0.1870972	0.1661739
ENSG00000267633	0.0045130	0.0092165	0.0061087	0.0094482	0.0000000
C19orf53	1.0598769	0.9700644	0.9425030	1.0322660	0.8285276
ZSWIM4	0.0311103	0.0392481	0.0362611	0.0271762	0.0998874
MIR23AHG	0.0062294	0.0019430	0.0043843	0.0031240	0.0178859
NANOS3	0.1617474	0.2114822	0.2157696	0.1602579	0.1514490
BRME1	0.0337571	0.0553317	0.0660707	0.0265730	0.0590090
CC2D1A	0.0397786	0.0680947	0.0681968	0.0423346	0.1444704
PODNL1	0.0108031	0.0171759	0.0485711	0.0059354	0.0031970
DCAF15	0.1252153	0.1274055	0.1708160	0.1050558	0.0827719
RFX1	0.0806445	0.0730333	0.0561624	0.0625924	0.1089334
ENSG00000275091	0.0009498	0.0090073	0.0029845	0.0073425	0.0000000
IL27RA	0.0109894	0.0404867	0.0451150	0.0076189	0.0499473
PALM3	0.1121924	0.2774387	0.1556269	0.1318681	0.1150932

MISP3	0.2213407	0.1993323	0.2298218	0.2156733	0.1281605
SAMD1	0.1689964	0.2547852	0.2603685	0.1909802	0.2042336
PRKACA	0.3044918	0.4582409	0.5783247	0.3045098	0.3606160
ENSG00000267783	0.0063973	0.0000000	0.0000000	0.0000000	0.0050044
ASF1B	0.0062728	0.0028573	0.0054312	0.0074345	0.0106248
ADGRL1-AS1	0.0384018	0.0179822	0.0131976	0.0273424	0.0186560
ADGRL1	0.2593638	0.4059769	0.4922729	0.2121902	0.5595156
ENSG00000287254	0.0014531	0.0023074	0.0045537	0.0000000	0.0000000
ENSG00000267723	0.0021832	0.0035061	0.0023230	0.0031040	0.0213162
LINC01841	0.0022697	0.0045888	0.0031723	0.0045947	0.0088663
ADGRE5	0.0426844	0.0249289	0.0137963	0.0300970	0.0240826
ENSG00000267379	0.0000000	0.0000000	0.0018092	0.0000000	0.0000000
DDX39A	0.1206962	0.1407555	0.1563711	0.1249102	0.0994938
PKN1	0.4435552	0.3858485	0.3912771	0.3718184	0.3341939
ENSG00000267474	0.0000000	0.0009303	0.0025638	0.0000000	0.0000000
PTGER1	0.0026379	0.0047928	0.0023547	0.0000000	0.0000000
GIPC1	0.3305145	0.2486276	0.2328807	0.3662120	0.2535065
DNAJB1	0.5309825	0.3710088	0.3641773	0.4772044	0.3869849
TECR	0.5018412	0.4779290	0.4081235	0.6328002	0.3353560
NDUFB7	0.8278601	0.8812428	0.9041981	0.8436146	0.7736751
ADGRE3	0.0014531	0.0023433	0.0044101	0.0089257	0.0000000
ZNF333	0.0875271	0.0640889	0.0645247	0.0677461	0.1178157
ADGRE2	0.0000000	0.0000000	0.0000000	0.0000000	0.0041632
SLC1A6	0.1008466	0.2217299	0.3548046	0.1131881	0.1909942
CCDC105	0.0000000	0.0000000	0.0000000	0.0000000	0.0060503
OR1I1	0.0000000	0.0000000	0.0000000	0.0031545	0.0000000
SYDE1	0.0195776	0.0215053	0.0107333	0.0331123	0.0183945
ILVBL	0.1381075	0.1218412	0.1128885	0.1542038	0.1282576
NOTCH3	0.0250225	0.0257280	0.0053038	0.0731291	0.0437284
ENSG00000269635	0.0000000	0.0033981	0.0024126	0.0045742	0.0015201
EPHX3	0.0225280	0.0096941	0.0116909	0.0208892	0.0015298
BRD4	0.7323465	0.7258646	0.7648764	0.6776172	0.8158383
ENSG00000290008	0.0037710	0.0101441	0.0089928	0.0104945	0.0366170
AKAP8	0.0981769	0.0966527	0.0845914	0.1026650	0.1651423
ENSG00000268189	0.0020673	0.0000000	0.0000000	0.0018378	0.0000000
AKAP8L	0.5103295	0.4652758	0.4523749	0.5375751	0.5398100
WIZ	0.1363855	0.1833528	0.2433997	0.1307699	0.1804475
RASAL3	0.0070786	0.0077694	0.0124286	0.0000000	0.0047837
PGLYRP2	0.0000000	0.0014068	0.0000000	0.0000000	0.0046972
CYP4F12	0.0000000	0.0000000	0.0000000	0.0000000	0.0086987
LINC01764	0.0014215	0.0000000	0.0000000	0.0000000	0.0073536
TPM4	0.8535631	0.9641892	1.0391226	0.8103490	0.9102399
RAB8A	0.4532822	0.4207197	0.3799277	0.4908753	0.2796391
ENSG00000269243	0.0000000	0.0019185	0.0055453	0.0000000	0.0052561
HSH2D	0.0000000	0.0014927	0.0000000	0.0038795	0.0012783
FAM32A	0.5188790	0.5704562	0.6056820	0.4665761	0.3925535
ENSG00000267033	0.0000000	0.0000000	0.0000000	0.0000000	0.0059495
AP1M1	0.3483397	0.3900984	0.4841774	0.2902310	0.3376261
KLF2-DT	0.0029618	0.0046759	0.0015569	0.0000000	0.0092851
KLF2	0.0167011	0.0136806	0.0084502	0.0140298	0.0152784
ENSG00000277825	0.0015243	0.0022530	0.0080117	0.0000000	0.0053148
EPS15L1	0.3480167	0.3263989	0.3238103	0.3035861	0.4693599
ENSG00000267703	0.0000000	0.0000000	0.0000000	0.0000000	0.0000000

ENSG00000289330	0.0000000	0.0033223	0.0000000	0.0000000	0.0000000
CALR3	0.0000000	0.0034376	0.0000000	0.0094905	0.0000000
C19orf44	0.0670821	0.0482096	0.0425334	0.0500375	0.0590995
CHERP	0.1253851	0.1153445	0.1236410	0.1164119	0.1779208
ENSG00000269399	0.0012367	0.0035045	0.0073589	0.0041748	0.0000000
SLC35E1	0.2423952	0.2886553	0.3036059	0.2596916	0.2730529
ENSG00000279529	0.0036275	0.0009065	0.0062170	0.0000000	0.0035522
MED26	0.0492845	0.0557057	0.0405388	0.0513501	0.1128621
ENSG00000269578	0.0000000	0.0000000	0.0000000	0.0000000	0.0097765
ENSG00000268087	0.0103279	0.0073290	0.0043665	0.0110135	0.0166413
ENSG00000269427	0.0097134	0.0122559	0.0100232	0.0027005	0.0195925
SMIM7	0.5953684	0.5231013	0.5229875	0.6003249	0.3921082
ENSG00000269044	0.0242643	0.0316063	0.0316610	0.0215070	0.0189648
ENSG00000267904	0.0083648	0.0119391	0.0200313	0.0091442	0.0082991
TMEM38A	0.0677071	0.1048826	0.0774952	0.1175410	0.1261522
NWD1	0.0501994	0.0201695	0.0415982	0.0748051	0.0583587
SIN3B	0.2700677	0.3033693	0.3355777	0.2979854	0.3445437
CPAMD8	0.0687797	0.0814036	0.0744683	0.0480094	0.0525914
HAUS8	0.0498192	0.0390507	0.0213821	0.0224806	0.0088962
MYO9B	0.1311270	0.1064606	0.0828679	0.1120734	0.3870810
ENSG00000268056	0.0012332	0.0000000	0.0000000	0.0000000	0.0000000
ENSG00000269836	0.0000000	0.0000000	0.0000000	0.0000000	0.0047864
USE1	0.2730115	0.2231968	0.1943242	0.2383836	0.2066774
OCEL1	0.1326728	0.1556414	0.1190744	0.1435638	0.0868201
NR2F6	0.3700430	0.3951720	0.4705359	0.3124877	0.3038766
USHBP1	0.0046522	0.0087171	0.0058106	0.0000000	0.0000000
BABAM1	0.3835672	0.4429827	0.4604394	0.3615145	0.3291231
ANKLE1	0.0020358	0.0044095	0.0036939	0.0032068	0.0066802
ABHD8	0.1723985	0.1792083	0.2532564	0.1436665	0.1313411
MRPL34	0.5354163	0.5708304	0.6192996	0.5243572	0.4062519
ENSG00000269815	0.0000000	0.0000000	0.0000000	0.0000000	0.0028791
DDA1	0.4856013	0.5492821	0.6065414	0.5203758	0.3727360
ANO8	0.0400632	0.0377152	0.0535615	0.0607077	0.1116174
GTPBP3	0.0899827	0.1443650	0.1406251	0.0715927	0.1022722
CCDC194	0.0000000	0.0000000	0.0000000	0.0000000	0.0073924
BST2	0.0068033	0.0094650	0.0030039	0.0096723	0.0133308
MVB12A	0.2065884	0.1354973	0.1063156	0.1770347	0.0965402
ENSG00000269481	0.0006692	0.0003640	0.0000000	0.0051903	0.0149214
ENSG00000269053	0.0000000	0.0010333	0.0000000	0.0000000	0.0000000
TMEM221	0.0027553	0.0075184	0.0016807	0.0194987	0.0000000
NXNL1	0.0005192	0.0000000	0.0000000	0.0000000	0.0000000
SLC27A1	0.0555402	0.0580308	0.0263234	0.0949239	0.1610326
PGLS-DT	0.0229081	0.0230929	0.0192783	0.0291624	0.0319186
PGLS	0.7636237	0.6218826	0.5443965	0.7847710	0.5071964
NIBAN3	0.0028208	0.0044207	0.0063463	0.0034364	0.0057761
COLGALT1	0.1019705	0.0884448	0.0885319	0.1639169	0.1123516
UNC13A	0.1458533	0.2656359	0.3567179	0.1229581	0.5008498
MAP1S	0.1364314	0.1779483	0.1869521	0.1676152	0.1786525
ENSG00000268112	0.0000000	0.0007671	0.0000000	0.0000000	0.0047906
FCHO1	0.0399586	0.0639235	0.0632780	0.0607003	0.1245432
B3GNT3	0.0003369	0.0000000	0.0000000	0.0000000	0.0014252
INSL3	0.0047673	0.0148853	0.0064303	0.0092359	0.0219338
JAK3	0.0141727	0.0275351	0.0292555	0.0025505	0.0355062

RPL18A	2.6152685	2.6031750	2.4280430	2.4828017	2.3573577
SLC5A5	0.0005639	0.0000000	0.0008981	0.0000000	0.0000000
CCDC124	0.5061033	0.5902482	0.6535187	0.4641957	0.3739479
KCNN1	0.0588850	0.1041830	0.1290947	0.0501748	0.1118096
ARRDC2	0.0183065	0.0128543	0.0080975	0.0147445	0.0110978
IL12RB1	0.0009606	0.0000000	0.0000000	0.0000000	0.0000000
MAST3	0.0392958	0.0650798	0.0460559	0.0261759	0.0668652
MAST3-AS1	0.0000000	0.0000000	0.0000000	0.0000000	0.0000000
PIK3R2	0.2269299	0.3306121	0.3460592	0.2546449	0.3269522
IFI30	0.0204068	0.0063012	0.0071453	0.0267333	0.0216736
MPV17L2	0.0444613	0.0485895	0.0612736	0.0542461	0.0175744
RAB3A	0.6420058	1.0865856	1.3005174	0.5517140	0.6957265
ENSG00000268650	0.0029746	0.0021810	0.0033911	0.0016996	0.0187967
PDE4C	0.0127022	0.0071523	0.0134186	0.0068349	0.1104957
ENSG00000284797	0.0016757	0.0000000	0.0012011	0.0028412	0.0015171
IQCN	0.0171213	0.0144002	0.0056874	0.0215935	0.0432217
JUND	1.4464978	1.3587418	1.2362298	1.3837620	1.3198249
LSM4	0.8166586	0.8708830	0.8858948	0.8105486	0.6565357
PGPEP1	0.2545201	0.1883390	0.1626991	0.1474827	0.2041157
GDF15	1.0316155	0.5928626	0.4344301	1.0029084	0.5713976
LRRC25	0.0044323	0.0009863	0.0027408	0.0054946	0.0000000
SSBP4	0.6357352	0.6041313	0.6053949	0.6379384	0.4812799
ISYNA1	0.5380866	0.4060668	0.4047636	0.4727009	0.3693573
ENSG00000268199	0.0000000	0.0000000	0.0000000	0.0000000	0.0000000
ELL	0.0391557	0.0330449	0.0318803	0.0484472	0.0927338
FKBP8	0.6928391	0.7643778	0.8479350	0.6939766	0.6253460
ENSG00000269191	0.0018485	0.0000000	0.0000000	0.0000000	0.0000000
KXD1	0.4272866	0.3643294	0.2830581	0.4969123	0.2525889
ENSG00000268030	0.0008902	0.0043666	0.0007799	0.0067691	0.0037333
UBA52	2.0924839	2.0910843	1.9565762	1.9930052	1.8630185
CRLF1	0.1538614	0.0994769	0.0894660	0.2472114	0.0956002
REX1BD	0.5382239	0.4844715	0.5562964	0.5782644	0.3882393
TMEM59L	0.5878253	0.6456582	0.7223066	0.9084583	0.5532788
KLHL26	0.0735195	0.0689816	0.0534571	0.0943694	0.0621991
CRTC1	0.1578643	0.1455406	0.1448227	0.1408852	0.3240855
COMP	0.0002413	0.0000000	0.0000000	0.0000000	0.0062626
UPF1	0.1627175	0.1417439	0.1265388	0.1689655	0.1618638
CERS1	0.4156349	0.3051673	0.2577208	0.6261028	0.2368950
ENSG00000269694	0.0000000	0.0000000	0.0000000	0.0000000	0.0000000
COPE	0.8788255	0.8635726	0.8558432	0.9246130	0.5869564
DDX49	0.2769531	0.3531890	0.3665556	0.2966717	0.2362608
HOMER3	0.2116288	0.1789277	0.1537524	0.2453473	0.1335040
HOMER3-AS1	0.0080746	0.0071374	0.0049073	0.0184752	0.0099210
SUGP2	0.4743602	0.5665597	0.5903588	0.5243699	0.6733236
ARMC6	0.0745459	0.1085113	0.1200563	0.0906250	0.1209576
SLC25A42	0.0628117	0.0686055	0.0593209	0.0780355	0.1067684
TMEM161A	0.0827924	0.0658880	0.0589945	0.1658805	0.0975046
MEF2B	0.0505265	0.0287799	0.0239469	0.0280666	0.0280689
BORCS8	0.1702996	0.2402337	0.2956109	0.1571641	0.1556998
RFXANK	0.1713082	0.1299944	0.0607545	0.1730821	0.0983173
NR2C2AP	0.0740174	0.0778852	0.0724830	0.0755532	0.0617055
NCAN	0.0659794	0.0904169	0.1799376	0.0259183	0.2160131
HAPLN4	0.0021154	0.0083722	0.0024670	0.0090627	0.0243798

TM6SF2	0.0048241	0.0063507	0.0160227	0.0172114	0.0000000
SUGP1	0.1044823	0.0841686	0.0967260	0.0962038	0.1619667
MAU2	0.0922371	0.0977992	0.0893272	0.0785506	0.2338876
GATAD2A	0.3541993	0.3748036	0.3028491	0.3640863	0.3459198
TSSK6	0.0153171	0.0143526	0.0063529	0.0065676	0.0081840
NDUFA13	1.5354424	1.5732687	1.6038292	1.5742003	1.3448734
YJEFN3	0.0467574	0.0663650	0.0581119	0.0253884	0.0918122
CILP2	0.0138442	0.0078122	0.0066353	0.0062326	0.0095296
PBX4	0.0357512	0.0414231	0.0596740	0.0248279	0.1097360
ENSG00000259242	0.0000000	0.0000000	0.0000000	0.0000000	0.0000000
LPAR2	0.1085923	0.1057176	0.0787326	0.1484548	0.1259713
GMIP	0.0190973	0.0205743	0.0249645	0.0210506	0.0336734
ATP13A1	0.0611658	0.0385396	0.0441014	0.1211949	0.0536523
ZNF101	0.0296726	0.0338437	0.0288650	0.0345623	0.0314525
ZNF14	0.0581102	0.0677294	0.0423786	0.0626385	0.1487526
LINC00663	0.0704056	0.0826301	0.0527005	0.0743662	0.0760899
ENSG00000287533	0.0056842	0.0055487	0.0047957	0.0031666	0.0162329
ENSG00000291177	0.0611006	0.0334938	0.0468369	0.0443763	0.0979135
ZNF506	0.2925010	0.2558348	0.3226783	0.2283007	0.3038786
ENSG00000267481	0.0107724	0.0059153	0.0037735	0.0043577	0.0205177
ZNF253	0.1474490	0.1509363	0.1599321	0.1235999	0.2008872
ENSG00000267565	0.0020502	0.0037190	0.0026108	0.0000000	0.0000000
ZNF93	0.1366470	0.1068599	0.1242076	0.0810184	0.1387816
ZNF682	0.1444818	0.1530168	0.1306266	0.1423244	0.1863977
ZNF90	0.0348172	0.0283403	0.0362771	0.0461416	0.0150972
ENSG00000267383	0.1764349	0.1394513	0.1212486	0.1424161	0.1482494
ZNF486	0.0609492	0.0385617	0.0453811	0.0463547	0.0389320
ENSG00000291130	0.2017821	0.1389614	0.1030865	0.2013958	0.1459544
ENSG00000269043	0.0024857	0.0071681	0.0028683	0.0000000	0.0194769
ZNF737	0.2070748	0.1903673	0.1771675	0.1693443	0.3133517
ENSG00000268560	0.0052073	0.0084843	0.0000000	0.0039507	0.0000000
ZNF626	0.3308968	0.3193308	0.3170034	0.3529131	0.3308157
ENSG00000288815	0.0063183	0.0116738	0.0054463	0.0000000	0.0101609
ZNF66	0.0453807	0.0559770	0.0478410	0.0582826	0.0584603
ZNF85	0.1079838	0.1386236	0.1620706	0.1194690	0.1070447
ENSG00000289297	0.0010340	0.0000000	0.0000000	0.0000000	0.0000000
ZNF430	0.1024859	0.1307233	0.0909786	0.0926352	0.2060068
ZNF714	0.1272022	0.1177711	0.1424218	0.0816591	0.1803175
ZNF431	0.1463633	0.1877538	0.2239942	0.0941661	0.1938748
ZNF708	0.2047009	0.2252848	0.2615573	0.1531853	0.3749704
ZNF738	0.2198241	0.3879679	0.5198764	0.2066451	0.2782407
ZNF493	0.1113358	0.1378258	0.1208493	0.0789591	0.2637842
ENSG00000268119	0.0449264	0.0870541	0.1423113	0.0596994	0.0978536
LINC00664	0.0000000	0.0009032	0.0000000	0.0000000	0.0027080
ZNF429	0.2612233	0.2657296	0.2418961	0.2060123	0.2763455
ENSG00000268081	0.0022467	0.0036567	0.0040732	0.0000000	0.0092211
ENSG00000268555	0.0007192	0.0000000	0.0035767	0.0032399	0.0046162
ZNF100	0.0569500	0.0647249	0.0604402	0.0537158	0.0955334
ENSG00000268184	0.0000000	0.0028593	0.0011221	0.0000000	0.0037333
ZNF43	0.2259357	0.2348666	0.2521387	0.2004492	0.2556869
ZNF208	0.0520767	0.0775483	0.0747344	0.0481378	0.0658255
ZNF257	0.0027287	0.0072731	0.0143926	0.0064765	0.0042206
ZNF676	0.0291726	0.0288063	0.0123093	0.0257940	0.0343415

ZNF98	0.0035236	0.0096448	0.0016462	0.0014154	0.0037861
ZNF492	0.0015191	0.0013043	0.0166315	0.0023114	0.0000000
ENSG00000268981	0.0032982	0.0008116	0.0009769	0.0029691	0.0000000
ZNF99	0.0023831	0.0053784	0.0008618	0.0036733	0.0018081
ZNF728	0.0081194	0.0231760	0.0174029	0.0297168	0.0294050
ENSG00000267886	0.0006621	0.0009018	0.0080676	0.0000000	0.0000000
ZNF730	0.1051289	0.1135978	0.1604235	0.1392707	0.1918284
ZNF724	0.0454271	0.0594672	0.0566914	0.0344480	0.0949767
ENSG00000284428	0.0772866	0.0682103	0.1076612	0.0980242	0.1134932
ZNF91	0.7443608	0.9199753	0.7714507	0.6605924	0.9853860
ENSG00000267934	0.0004529	0.0000000	0.0000000	0.0000000	0.0000000
LINC01224	0.0170348	0.0457055	0.0626612	0.0158881	0.0223283
ZNF675	0.2037045	0.2397367	0.2649157	0.1657586	0.2257008
ZNF681	0.0906958	0.1826754	0.1715739	0.0804974	0.1636854
RPSA2	0.3119130	0.3586728	0.3365146	0.2659225	0.4105241
ENSG00000267924	0.0033483	0.0000000	0.0025278	0.0000000	0.0112486
ZNF726	0.0333330	0.0560604	0.0533518	0.0237390	0.0474750
ENSG00000269289	0.0000000	0.0079936	0.0122924	0.0036320	0.0069479
ENSG00000269397	0.0050734	0.0060674	0.0049097	0.0045771	0.0000000
ENSG00000268362	0.0264943	0.0315771	0.0297764	0.0289205	0.0404708
ZNF254	0.2535134	0.2610680	0.3163956	0.2220760	0.4409890
ENSG00000276030	0.0006224	0.0042552	0.0098135	0.0000000	0.0119858
ERVK-28	0.0059447	0.0210990	0.0064975	0.0044486	0.0192002
LINC00662	0.5849016	0.6800846	0.6987519	0.4650165	0.6621494
ENSG00000261770	0.0002930	0.0019200	0.0000000	0.0000000	0.0028666
LINC02987	0.1624697	0.1811973	0.1736748	0.1343949	0.1179528
ENSG00000281468	0.0108505	0.0090087	0.0129533	0.0225029	0.0224305
ENSG00000266977	0.0021937	0.0000000	0.0000000	0.0000000	0.0000000
ENSG00000266893	0.0000000	0.0000000	0.0000000	0.0000000	0.0000000
LINC00906	0.0000000	0.0002815	0.0020580	0.0000000	0.0000000
UQCRFS1	0.8871194	1.0198457	1.0876555	0.8424817	0.7159664
UQCRFS1-DT	0.0231918	0.0116162	0.0048144	0.0132924	0.0136498
ENSG00000276251	0.0027828	0.0009714	0.0000000	0.0029889	0.0025831
ENSG00000276631	0.0030507	0.0009742	0.0000000	0.0000000	0.0000000
VSTM2B-DT	0.0338412	0.0780173	0.0367482	0.0388659	0.0958634
VSTM2B	0.1565377	0.4039289	0.3010948	0.1886325	0.3299035
POP4	0.2266815	0.2269678	0.2579348	0.2963029	0.1676912
PLEKHF1	0.0003405	0.0105254	0.0052214	0.0088408	0.0000000
C19orf12	0.2401467	0.3087063	0.3724055	0.2241453	0.2258249
ENSG00000289030	0.0011785	0.0029965	0.0015874	0.0000000	0.0000000
CCNE1	0.0336804	0.0736628	0.0706140	0.0160899	0.1054176
URI1	1.0359407	0.9603823	0.9705230	1.0645527	0.9522875
ENSG00000287859	0.0000000	0.0019523	0.0000000	0.0000000	0.0000000
ENSG00000267223	0.0009054	0.0042498	0.0000000	0.0000000	0.0175104
ZNF536	0.2754807	0.3783201	0.2011387	0.2054384	0.9616146
ENSG00000289187	0.0011804	0.0008857	0.0020861	0.0000000	0.0177135
ENSG00000267760	0.0000000	0.0000000	0.0000000	0.0000000	0.0036228
ENSG00000284430	0.0009479	0.0000000	0.0000000	0.0000000	0.0000000
TSHZ3	0.2782278	0.3561766	0.4437704	0.2977231	0.6138468
LINC01791	0.0006736	0.0011443	0.0000000	0.0000000	0.0115239
TSHZ3-AS1	0.0567445	0.0376456	0.0331073	0.0583602	0.1461611
ENSG00000267636	0.0016080	0.0022769	0.0000000	0.0000000	0.0000000
ENSG00000261400	0.0015797	0.0098295	0.0032889	0.0000000	0.0000000

LINC01837	0.0012012	0.0000000	0.0023023	0.0000000	0.0000000
ZNF507	0.2232082	0.2439570	0.2456749	0.1835607	0.2421786
DPY19L3-DT	0.0481278	0.0249018	0.0313687	0.0393695	0.0288540
DPY19L3	0.2044128	0.1496845	0.1436101	0.2445220	0.2452824
PDCD5	0.9583440	1.0445903	1.1711024	0.8935424	0.7441938
ANKRD27	0.0904455	0.1142847	0.0839484	0.0939807	0.1797215
ENSG00000267557	0.0000000	0.0000000	0.0000000	0.0000000	0.0045212
RGS9BP	0.0000000	0.0075671	0.0077556	0.0000000	0.0000000
NUDT19-DT	0.0073421	0.0146819	0.0145120	0.0000000	0.0052984
NUDT19	0.0487192	0.0609805	0.0614276	0.0217710	0.1070547
TDRD12	0.0054165	0.0149911	0.0254216	0.0017727	0.0135632
SLC7A9	0.0038416	0.0015070	0.0061757	0.0032517	0.0355840
ENSG00000267555	0.0000000	0.0000000	0.0000000	0.0000000	0.0000000
CEP89	0.3732937	0.3353266	0.3007527	0.3970949	0.4012017
FAAP24	0.0120808	0.0135208	0.0122290	0.0192384	0.0053082
RHPN2	0.2434851	0.1438258	0.0682231	0.2878519	0.1635844
GPATCH1	0.0962282	0.1076404	0.1041584	0.1352335	0.1835841
WDR88	0.0094165	0.0157010	0.0223888	0.0125270	0.0071420
ENSG00000289378	0.0027106	0.0053265	0.0039830	0.0017461	0.0019545
LRP3	0.0796403	0.1631860	0.1307454	0.0519870	0.1470518
ENSG00000267054	0.0012098	0.0000000	0.0029481	0.0027252	0.0000000
SLC7A10	0.0027202	0.0028016	0.0000000	0.0000000	0.0000000
ENSG00000267714	0.0000000	0.0014825	0.0000000	0.0000000	0.0000000
CEBPA	0.0265905	0.0124584	0.0219924	0.0154104	0.0127953
CEBPA-DT	0.0098950	0.0037574	0.0044915	0.0285765	0.0000000
ENSG00000267130	0.0054575	0.0006921	0.0061278	0.0023202	0.0105601
CEBPG	0.9284641	0.9067123	0.8912500	0.7909426	0.7416783
PEPD	0.2435297	0.2114695	0.2796345	0.3309032	0.1830981
CHST8	0.0411733	0.0953460	0.0871580	0.0473299	0.2223314
KCTD15	0.1271029	0.1139527	0.0730757	0.0688989	0.0816507
SUNO1	0.0121771	0.0031536	0.0000000	0.0076710	0.0000000
LSM14A	0.9187630	0.7713506	0.6748252	0.8429916	0.8016138
GARRE1	0.2568069	0.2852619	0.3581427	0.2826493	0.4034000
ENSG00000267219	0.0096674	0.0049285	0.0000000	0.0086229	0.0071004
GPI	1.0805402	1.2523152	1.3578385	1.0104785	1.2164517
PDCD2L	0.1197916	0.1662902	0.2315659	0.0953602	0.1826754
ENSG00000267024	0.0093247	0.0070526	0.0026978	0.0079540	0.0021475
UBA2	0.8766629	0.7226702	0.6665017	0.8464757	0.6582916
WTIP	0.2427282	0.2569983	0.2626951	0.1850463	0.2452943
SCGB1B2P	0.0400592	0.0604632	0.0565945	0.0318764	0.0463091
SCGB2B2	0.0567636	0.0500396	0.0772184	0.0635051	0.2118257
ENSG00000268683	0.0000000	0.0000000	0.0000000	0.0000000	0.0000000
ZNF302	0.2888976	0.3783387	0.2851669	0.3080022	0.3540608
ENSG00000274104	0.0220827	0.0253477	0.0484716	0.0084983	0.0417214
ZNF181	0.1880369	0.1680842	0.1232554	0.1588653	0.1455699
ZNF599	0.1206539	0.0822633	0.0693243	0.1217079	0.0828735
LINC01801	0.0102324	0.0186719	0.0388260	0.0031700	0.0240421
ENSG00000261754	0.0100402	0.0094097	0.0111662	0.0085177	0.0000000
LINC02965	0.0020034	0.0000000	0.0009612	0.0000000	0.0000000
LINC03049	0.0088305	0.0126279	0.0101461	0.0030550	0.0137184
LINC00904	0.0000000	0.0042730	0.0026942	0.0000000	0.0000000
ZNF30-AS1	0.0033572	0.0070860	0.0128808	0.0010246	0.0107724
ZNF30	0.0392738	0.0390664	0.0491138	0.0325672	0.0887571

ZNF792	0.0094188	0.0215474	0.0216852	0.0053125	0.0213218
GRAMD1A	0.3427204	0.4566421	0.4592521	0.2535822	0.3699787
GRAMD1A-AS1	0.0015599	0.0000000	0.0026443	0.0039534	0.0000000
SCN1B	0.0087680	0.0280113	0.0121936	0.0210145	0.0318348
HPN	0.0032785	0.0000000	0.0027572	0.0000000	0.0031937
HPN-AS1	0.0000000	0.0000000	0.0000000	0.0000000	0.0046818
FXYD3	0.0040343	0.0016269	0.0009965	0.0000000	0.0000000
LGI4	0.0165352	0.0428372	0.0210476	0.0115181	0.0234434
FXYD1	0.0770473	0.0903084	0.0837550	0.0875063	0.0725553
FXYD7	0.3648798	0.6832615	0.9219506	0.3100970	0.5179304
FXYD5	0.0064272	0.0038820	0.0000000	0.0000000	0.0059578
LSR	0.0732894	0.0626886	0.0203180	0.0868367	0.0256705
ENSG00000288942	0.0006428	0.0035291	0.0018647	0.0025868	0.0083847
USF2	0.5251018	0.4746455	0.4657146	0.4499746	0.3445242
CD22	0.0000000	0.0000000	0.0000000	0.0000000	0.0000000
FFAR1	0.0000000	0.0000000	0.0000000	0.0000000	0.0000000
KRTDAP	0.0023080	0.0080843	0.0082532	0.0055070	0.0000000
DMKN	0.0662521	0.0686568	0.0984928	0.0411714	0.0644656
SBSN	0.0011240	0.0035667	0.0000000	0.0000000	0.0073270
GAPDHS	0.0000000	0.0000000	0.0006253	0.0000000	0.0031293
TMEM147-AS1	0.0499109	0.0388614	0.0237598	0.0589202	0.0434864
TMEM147	0.7354350	0.6720620	0.6586936	0.7874108	0.5074368
ATP4A	0.0020020	0.0015109	0.0000000	0.0000000	0.0028046
ENSG00000283907	0.0051763	0.0080959	0.0076365	0.0047425	0.0095718
LINC01766	0.0009548	0.0000000	0.0000000	0.0000000	0.0055996
HAUS5-DT	0.0015763	0.0020193	0.0010720	0.0049134	0.0000000
HAUS5	0.0457037	0.0363680	0.0258339	0.0557051	0.0711276
RBM42	0.3677718	0.3358506	0.3131312	0.3401176	0.2039602
ETV2	0.0291878	0.0412546	0.0278627	0.0272779	0.0159807
COX6B1	1.5593372	1.6705069	1.6729846	1.5365064	1.3241660
UPK1A	0.0011922	0.0014966	0.0013551	0.0039066	0.0000000
UPK1A-AS1	0.0005442	0.0000000	0.0000000	0.0000000	0.0040644
ZBTB32	0.0000000	0.0000000	0.0020559	0.0000000	0.0057073
KMT2B	0.1682306	0.1888744	0.1761284	0.1114490	0.2558691
IGFLR1	0.0119508	0.0212313	0.0110766	0.0207959	0.0142071
U2AF1L4	0.1064269	0.1221559	0.1064541	0.0871172	0.0793777
PSENEN	0.7067066	0.5651887	0.6015052	0.8632356	0.4982123
ENSG00000267439	0.0054776	0.0050326	0.0000000	0.0110348	0.0177523
LIN37	0.1309521	0.1811669	0.1702362	0.1255710	0.1330812
ENSG00000267328	0.0048590	0.0020262	0.0061761	0.0157118	0.0000000
HSPB6	0.3817378	0.2098016	0.1529211	0.3010738	0.1994597
PROSER3	0.0600607	0.0665149	0.0682073	0.0889222	0.0902934
ENSG00000267049	0.0013858	0.0000000	0.0000000	0.0000000	0.0054500
ARHGAP33	0.0706279	0.1366291	0.1375779	0.0564720	0.1692594
PRODH2	0.0000000	0.0000000	0.0000000	0.0000000	0.0000000
NPHS1	0.0152047	0.0121333	0.0207324	0.0105731	0.0095681
KIRREL2	0.0158886	0.0063563	0.0061284	0.0197189	0.0000000
APLP1	0.7201998	0.8196508	1.1170440	0.8606048	0.6890379
NFKBID	0.0072996	0.0057574	0.0049174	0.0053911	0.0000000
HCST	0.0143046	0.0157606	0.0209267	0.0022801	0.0161877
LRFN3	0.0902221	0.0997447	0.1327652	0.1059292	0.1075099
ENSG00000267786	0.0403112	0.0536057	0.0374091	0.0085151	0.0580697
ENSG00000267388	0.0098830	0.0134214	0.0164686	0.0076911	0.0054431

SDHAF1	0.1372150	0.1568802	0.1172444	0.1587996	0.1057889
SYNE4	0.0119512	0.0156286	0.0563666	0.0041882	0.0235826
ALKBH6	0.0878922	0.1149229	0.1564418	0.1242093	0.0718918
ENSG00000267698	0.0111765	0.0041720	0.0036522	0.0000000	0.0086074
CLIP3	0.3258856	0.4784678	0.5325887	0.2788164	0.3459201
THAP8	0.1331416	0.1508079	0.1525515	0.0828304	0.1181677
WDR62	0.0030577	0.0021089	0.0000000	0.0031073	0.0075546
POLR2I	0.9136846	0.9209343	0.9182118	0.8359048	0.7097199
TBCB	1.2427132	1.3736242	1.5235429	1.2806384	1.0364264
CAPNS1	0.6087718	0.5729336	0.5396373	0.6644770	0.4127406
ENSG00000270760	0.0000000	0.0015711	0.0000000	0.0000000	0.0000000
COX7A1	0.0493888	0.0380290	0.0367694	0.0505290	0.0503312
ZNF565	0.0641335	0.0777629	0.0571343	0.0591124	0.0836300
ZNF146	0.3340465	0.3232277	0.2946324	0.2856407	0.3902637
ENSG00000267053	0.0224073	0.0283552	0.0210485	0.0212326	0.0065615
LINC00665	0.3160419	0.3348530	0.3428089	0.2938116	0.3082811
ZFP14	0.2027077	0.2572304	0.2739623	0.2261140	0.3359249
ZFP82	0.2061366	0.2587018	0.2643186	0.2345358	0.2550790
ZNF566	0.1527948	0.2047517	0.2312184	0.1795583	0.2595189
ZNF566-AS1	0.0041409	0.0018901	0.0018196	0.0041247	0.0000000
ZNF260	0.2041521	0.2479468	0.2329456	0.1967399	0.1986765
ENSG00000228629	0.0000000	0.0000000	0.0000000	0.0000000	0.0126812
ZNF529	0.1841229	0.2341621	0.3134472	0.1535394	0.2939132
ZNF529-AS1	0.1776760	0.1252254	0.1122687	0.1166212	0.1639995
ZNF382	0.1550766	0.1727777	0.1706317	0.1268009	0.2005715
ZNF461	0.0636429	0.0591796	0.0668273	0.0697721	0.0716780
ENSG00000276071	0.0006630	0.0000000	0.0017803	0.0024545	0.0000000
ZNF567-DT	0.0342087	0.0513297	0.0565111	0.0375462	0.0511459
ZNF567	0.1082746	0.1112460	0.0852041	0.0961348	0.1270221
ZNF850	0.0137696	0.0139348	0.0113613	0.0086044	0.0334253
ENSG00000267260	0.0161508	0.0164096	0.0283335	0.0158407	0.0162147
ZNF790-AS1	0.0576386	0.0992875	0.0700287	0.0640010	0.0873072
ZNF790	0.0607647	0.0841547	0.0873268	0.1035401	0.0921664
ZNF345	0.1035046	0.0994721	0.1066087	0.0494417	0.1989841
ZNF829	0.0421177	0.0399464	0.0289100	0.0356164	0.0185384
ZNF568	0.1716159	0.2558095	0.2213173	0.1861184	0.2586394
ZNF420	0.1027135	0.1006980	0.1077763	0.0956211	0.1838237
ENSG00000267345	0.0063565	0.0129299	0.0171333	0.0100288	0.0120439
ENSG00000267672	0.0000000	0.0023463	0.0026426	0.0000000	0.0027938
ZNF585A	0.1366921	0.1762266	0.2127089	0.1589627	0.1588691
ZNF585B	0.0960934	0.0863863	0.1317337	0.0808633	0.1393825
ZNF383	0.0774930	0.0883014	0.0747892	0.0779136	0.1074883
ENSG00000267605	0.0084040	0.0141174	0.0000000	0.0008779	0.0204493
LINC01535	0.0917986	0.1413088	0.1843222	0.0877346	0.0873149
ENSG00000286449	0.0050680	0.0078577	0.0009751	0.0028199	0.0112790
ZNF875	0.1374537	0.1449314	0.1397916	0.1321477	0.2406547
ENSG00000276846	0.0156584	0.0078641	0.0000000	0.0129758	0.0000000
ZNF527	0.0485273	0.0494194	0.0579035	0.0353952	0.0512916
ZNF569	0.1265822	0.1595547	0.1572950	0.1360222	0.2030789
ZNF570	0.1464730	0.1623255	0.2041102	0.1197253	0.1298846
ZNF793-AS1	0.1040719	0.1313655	0.1250973	0.0819930	0.0946695
ZNF793	0.1762378	0.2485827	0.2998013	0.1244586	0.2475791
ENSG00000180458	0.0056543	0.0243831	0.0071236	0.0000000	0.0071122

ZNF571-AS1	0.0446859	0.0560980	0.0893607	0.0245890	0.0682276
ZNF540	0.1365259	0.2179123	0.2531437	0.0928585	0.2451209
ZNF571	0.0846063	0.1028576	0.1025193	0.0814653	0.1794881
ZFP30	0.2367462	0.2843903	0.3257962	0.2158455	0.3151418
ZNF781	0.0324986	0.0484403	0.0509586	0.0362820	0.0553456
ZNF607	0.0433521	0.0474691	0.0333026	0.0303446	0.0958085
ENSG00000267152	0.0265017	0.0383398	0.0475227	0.0211720	0.0396951
ENSG00000287496	0.0009690	0.0047066	0.0040567	0.0000000	0.0069425
ZNF573	0.0751930	0.0989689	0.0989756	0.0493166	0.1493668
ENSG00000291089	0.1681966	0.1204723	0.1976988	0.1346701	0.1942750
WDR87BP	0.0000000	0.0000000	0.0000000	0.0023550	0.0016673
WDR87	0.0018227	0.0000000	0.0035392	0.0000000	0.0044531
SIPA1L3	0.1044479	0.1202094	0.1094472	0.1093143	0.4919024
DPF1	0.2228849	0.4058165	0.7018666	0.2606721	0.3659295
SPINT2	0.6768021	0.7236865	0.8920652	0.7050702	0.6277824
PPP1R14A	0.0230232	0.0279163	0.0464182	0.0162694	0.0274763
ENSG00000286037	0.0013897	0.0059512	0.0000000	0.0000000	0.0019716
YIF1B	0.2142300	0.2034943	0.2246860	0.1835178	0.1935066
C19orf33	0.0011756	0.0008286	0.0047287	0.0061145	0.0073599
KCNK6	0.0017973	0.0000000	0.0000000	0.0000000	0.0039284
CATSPERG	0.0525250	0.0478485	0.0494898	0.0576455	0.1567077
PSMD8	1.0767258	1.0903843	1.1817911	1.1140146	0.8500175
GGN	0.0058741	0.0055977	0.0037365	0.0065497	0.0000000
SPRED3	0.0331855	0.0370157	0.0657568	0.0386547	0.0639109
FAM98C	0.1844987	0.2141879	0.3114546	0.2244163	0.1354422
RASGRP4	0.0006463	0.0000000	0.0000000	0.0000000	0.0000000
RYR1	0.0590419	0.0531142	0.0294602	0.0454980	0.2436617
MAP4K1	0.0452162	0.0877070	0.1379786	0.0375420	0.0373370
MAP4K1-AS1	0.0050837	0.0077060	0.0093301	0.0044186	0.0062023
EIF3K	0.9772122	1.0127201	0.9575920	0.9719568	0.8719861
ACTN4	0.3069657	0.2151105	0.1879830	0.3689137	0.3532034
ENSG00000267375	0.0000000	0.0000000	0.0000000	0.0000000	0.0016619
CAPN12	0.0029234	0.0032552	0.0031789	0.0018419	0.0000000
ENSG00000267892	0.0004116	0.0051423	0.0024028	0.0000000	0.0088960
LGALS4	0.0031051	0.0060014	0.0000000	0.0000000	0.0097677
ECH1	0.6447976	0.5534324	0.6402762	0.6409158	0.4346683
HNRNPL	0.5579251	0.5519585	0.6193387	0.6152696	0.6083365
ENSG00000269688	0.0046602	0.0008435	0.0035392	0.0029154	0.0217835
RINL	0.0390001	0.0729961	0.0800523	0.0213252	0.0648396
SIRT2	0.3540664	0.3501722	0.3134781	0.3826430	0.3381827
NFKBIB	0.1105323	0.1028009	0.0831828	0.1213203	0.0687315
CCER2	0.0024721	0.0004186	0.0000000	0.0000000	0.0062172
SARS2	0.0295438	0.0320124	0.0095362	0.0093078	0.0396253
MRPS12	0.2148217	0.2760218	0.2957382	0.2719134	0.1443640
ERVK9-11	0.0008367	0.0012802	0.0009471	0.0000000	0.0000000
FBXO17	0.1378422	0.1720130	0.1361571	0.1760971	0.1256515
ENSG00000287271	0.0178340	0.0159986	0.0147006	0.0095368	0.0251975
FBXO27	0.2330928	0.3092579	0.3232051	0.1837678	0.2316472
ACP7	0.0000000	0.0000000	0.0000000	0.0000000	0.0000000
PAK4	0.0929259	0.0804801	0.0695379	0.1132767	0.0917789
LRFN1	0.0700253	0.0917537	0.1439495	0.0417694	0.1222728
GMFG	0.0107605	0.0101656	0.0204296	0.0117156	0.0151777
ENSG00000269246	0.0083608	0.0021437	0.0040848	0.0176371	0.0101959

SAMD4B	0.3624425	0.2819402	0.2532858	0.3225621	0.3211922
PAF1	0.3189837	0.2373925	0.1890244	0.3567331	0.1883164
MED29	0.4582329	0.5021807	0.5480320	0.4385167	0.3906291
ZFP36	0.1368954	0.0695852	0.0593940	0.0784822	0.1077902
PLEKHG2	0.0274662	0.0338086	0.0188804	0.0258387	0.1015185
RPS16	2.4361975	2.4682033	2.3390662	2.3533351	2.2437486
SUPT5H	0.6654652	0.6723564	0.6213169	0.5650176	0.5896679
TIMM50	0.2068834	0.2351820	0.2662018	0.1766623	0.1971520
DLL3	0.3012663	0.4228482	0.6233534	0.2745890	0.3287943
SELENOV	0.0007370	0.0024539	0.0000000	0.0000000	0.0079003
ENSG00000288770	0.0000000	0.0085433	0.0029566	0.0068830	0.0000000
EID2B	0.2375973	0.2728590	0.3763088	0.2045056	0.1958140
EID2	0.3553131	0.3434245	0.4829178	0.3201721	0.2357484
LGALS14	0.0038936	0.0097912	0.0325490	0.0086051	0.0028907
CLC	0.0016435	0.0000000	0.0040935	0.0000000	0.0000000
ENSG00000276445	0.0083564	0.0119666	0.0079355	0.0000000	0.0000000
DYRK1B	0.1208375	0.1264201	0.1467034	0.1197536	0.0791635
FBL	0.5152674	0.4688434	0.4166225	0.4593968	0.4124248
FCGBP	0.0006609	0.0007624	0.0000000	0.0000000	0.0000000
PSMC4	0.5445448	0.4960563	0.5340814	0.5391755	0.3664994
ZNF546	0.0777136	0.0465802	0.0540946	0.0639835	0.1020583
ZNF780B	0.0943746	0.1283453	0.1084982	0.0752848	0.3900437
ZNF780A	0.0659473	0.0640683	0.0799902	0.0701559	0.0738163
ENSG00000269296	0.0005278	0.0000000	0.0000000	0.0000000	0.0000000
MAP3K10	0.0638915	0.1090292	0.1393492	0.0393183	0.1326425
TTC9B	0.4074955	0.7454950	1.0141989	0.3342942	0.4664777
CCNP	0.0075932	0.0063850	0.0123069	0.0000000	0.0026876
AKT2	0.1478234	0.1086309	0.0624189	0.1533372	0.1703255
ENSG00000205041	0.0025588	0.0038832	0.0000000	0.0000000	0.0020838
C19orf47	0.0477374	0.0492413	0.0455768	0.0558109	0.0372938
PLD3	1.2967710	1.1582756	1.2008422	1.7392822	1.1057941
HIPK4	0.0025174	0.0000000	0.0000000	0.0000000	0.0078070
PRX	0.0068085	0.0040065	0.0000000	0.0052723	0.0072008
SERTAD1	0.1034866	0.0469718	0.0387901	0.0573551	0.0509813
ENSG00000277453	0.0025226	0.0000000	0.0051907	0.0000000	0.0052561
SERTAD3	0.1254596	0.0744241	0.0697849	0.0940115	0.0854289
BLVRB	0.5699835	0.4455189	0.3226293	0.6110396	0.3639421
SPTBN4	0.0837351	0.1294217	0.0897169	0.0911096	0.4897070
SHKBP1	0.0516046	0.0484796	0.0550993	0.0508857	0.0574728
LTBP4	0.0493446	0.0411377	0.0310909	0.0733458	0.0970030
NUMBL	0.1661729	0.2179787	0.2548168	0.1811536	0.2447236
COQ8B	0.1178549	0.0822069	0.0742737	0.1076453	0.1449917
ITPKC	0.0454388	0.0423769	0.0275351	0.0301698	0.0456438
ACTMAP	0.0322812	0.0218566	0.0269548	0.0325083	0.0354749
SNRPA	0.2888193	0.2835656	0.2226876	0.3089414	0.1601164
MIA	0.0006806	0.0010939	0.0000000	0.0032774	0.0090649
RAB4B	0.1386279	0.1629498	0.2094039	0.1525898	0.1360791
ENSG00000282951	0.0000000	0.0008848	0.0046562	0.0058868	0.0000000
EGLN2	0.1823995	0.1854956	0.2298801	0.1540376	0.2057936
ENSG00000269843	0.0010446	0.0018070	0.0007485	0.0000000	0.0000000
CYP2A6	0.0000000	0.0000000	0.0012348	0.0000000	0.0000000
CYP2G1P	0.0000000	0.0000000	0.0018758	0.0000000	0.0000000
CYP2B7P	0.0006123	0.0000000	0.0000000	0.0000000	0.0029771

CYP2A13	0.0024055	0.0000000	0.0118231	0.0000000	0.0000000
CYP2F1	0.0000000	0.0002840	0.0000000	0.0000000	0.0000000
CYP2S1	0.0000000	0.0000000	0.0015892	0.0000000	0.0000000
AXL	0.0178581	0.0084528	0.0081902	0.0118713	0.0150987
HNRNPUL1	0.6588796	0.5656739	0.6088121	0.5948301	0.5697951
TGFB1	0.0604761	0.0421852	0.0334566	0.0769409	0.0462124
ENSG00000286177	0.0024827	0.0000000	0.0054124	0.0015511	0.0143095
CCDC97	0.0992378	0.0659217	0.0681983	0.0441975	0.1237669
TMEM91	0.0984573	0.1534399	0.1378498	0.0738484	0.1774714
B9D2	0.0989129	0.0697452	0.0366761	0.0812458	0.0429552
ENSG00000277744	0.0005103	0.0014800	0.0000000	0.0000000	0.0000000
EXOSC5	0.0824903	0.1063306	0.1359940	0.0625439	0.0495774
BCKDHA	0.1881193	0.1666949	0.1406054	0.1773771	0.1318359
B3GNT8	0.0057226	0.0060898	0.0039704	0.0027628	0.0000000
DMAC2	0.1514807	0.1932777	0.1450228	0.1378045	0.1281215
ERICH4	0.0004587	0.0000000	0.0000000	0.0000000	0.0000000
CEACAM21	0.0158338	0.0390326	0.0909675	0.0129456	0.0333665
ENSG00000268833	0.0054350	0.0048270	0.0000000	0.0088459	0.0046690
RPS19	3.0594493	2.9498580	2.7406519	2.9792137	2.7402044
CD79A	0.0042227	0.0014072	0.0000000	0.0000000	0.0000000
ARHGEF1	0.0668180	0.0371250	0.0228187	0.0178916	0.1160564
ERFL	0.0121738	0.0225481	0.0288411	0.0034589	0.0502020
RABAC1	1.0055421	0.9835115	1.0262580	1.0765412	0.8088928
ATP1A3	0.3872480	0.6812236	0.9054254	0.3761744	0.6448962
GRIK5	0.1006515	0.1647121	0.1337143	0.1256886	0.3015525
ZNF574	0.0428626	0.0479477	0.0550459	0.0517404	0.0495098
ENSG00000289115	0.0012921	0.0026077	0.0029749	0.0000000	0.0054224
POU2F2	0.0691990	0.1401420	0.2562532	0.0624394	0.2075483
POU2F2-AS1	0.0008021	0.0010152	0.0041897	0.0000000	0.0000000
POU2F2-AS2	0.0482611	0.1467018	0.2574243	0.0389911	0.0959025
DEDD2	0.1281714	0.1209345	0.0939933	0.0944562	0.0988861
ZNF526	0.0287001	0.0191613	0.0177864	0.0141127	0.0372248
GSK3A	0.3065928	0.3957271	0.5125912	0.2685765	0.2740000
ERF	0.1343742	0.0710215	0.0374929	0.1009358	0.0697663
CIC	0.0762912	0.0810980	0.0790198	0.0679521	0.1516963
PAFAH1B3	0.5594610	0.8774384	1.1112930	0.4966278	0.6247914
PRR19	0.0152093	0.0173598	0.0243378	0.0091581	0.0259913
TMEM145	0.0444489	0.0792259	0.0964626	0.0501657	0.0937317
MEGF8	0.1965094	0.2099941	0.2244361	0.2733676	0.2484878
CNFN	0.0060937	0.0008741	0.0046567	0.0000000	0.0037366
LIPE-AS1	0.1114575	0.1156795	0.0906575	0.0958422	0.3032234
LIPE	0.0086957	0.0156261	0.0310250	0.0136407	0.0346623
CXCL17	0.0010865	0.0000000	0.0000000	0.0000000	0.0000000
CEACAM1	0.0028209	0.0010604	0.0021947	0.0000000	0.0100162
CEACAM8	0.0028956	0.0000000	0.0000000	0.0000000	0.0000000
PSG3	0.0000000	0.0000000	0.0000000	0.0000000	0.0000000
PSG8	0.0000000	0.0000000	0.0000000	0.0000000	0.0000000
PSG8-AS1	0.0008804	0.0000000	0.0000000	0.0000000	0.0046972
PSG11-AS1	0.0056701	0.0012783	0.0054063	0.0047879	0.0190833
PSG11	0.0000000	0.0000000	0.0000000	0.0000000	0.0000000
PSG4	0.0000000	0.0000000	0.0000000	0.0000000	0.0000000
PSG9	0.0000000	0.0000000	0.0000000	0.0000000	0.0000000
TEX101	0.0009612	0.0000000	0.0000000	0.0000000	0.0000000

LYPD3	0.0043777	0.0239332	0.0121288	0.0000000	0.0141901
PHLDB3	0.0214082	0.0076606	0.0135605	0.0116106	0.0334743
ETHE1	0.1754586	0.1832576	0.2108042	0.1418042	0.1697682
ZNF575	0.0343076	0.0522947	0.0697234	0.0534454	0.0512781
XRCC1	0.2249684	0.2043766	0.1991017	0.1802760	0.1791189
PINLYP	0.0454722	0.0305912	0.0230152	0.0429526	0.0379176
IRGQ	0.1823609	0.2549651	0.2602276	0.1545034	0.2510616
ZNF576	0.1468224	0.1520760	0.1223540	0.1698666	0.0862300
SRRM5	0.0043482	0.0067339	0.0009480	0.0031700	0.0171259
ZNF428	1.1453085	1.3668571	1.5100232	1.1217668	1.0219693
CADM4	0.3768810	0.4238660	0.5113498	0.4362913	0.3205296
PLAUR	0.0092439	0.0023927	0.0035921	0.0082209	0.0060693
ENSG00000290609	0.0020592	0.0005680	0.0000000	0.0000000	0.0014498
SMG9	0.0933960	0.0789142	0.0621359	0.1034297	0.1174313
LYPD5	0.0003948	0.0064255	0.0131151	0.0000000	0.0100512
ZNF283	0.0566122	0.0668181	0.0853289	0.0627834	0.0957066
ZNF404	0.0866176	0.0508863	0.0534310	0.0766095	0.0867214
ENSG00000267058	0.0192562	0.0259018	0.0204939	0.0340429	0.0197559
ENSG00000286613	0.0006326	0.0000000	0.0000000	0.0012811	0.0056253
ZNF45-AS1	0.0316628	0.0471396	0.0344323	0.0147593	0.0427381
ZNF45	0.0389275	0.0451004	0.0402397	0.0456166	0.0555132
ZNF221	0.0244628	0.0375200	0.0476623	0.0182501	0.0533542
ZNF155	0.1497116	0.2485859	0.2092835	0.1957446	0.2807770
ENSG00000277806	0.0048835	0.0136679	0.0068083	0.0086947	0.0224922
ENSG00000278492	0.0053546	0.0036222	0.0025623	0.0000000	0.0053148
ZNF230-DT	0.0134627	0.0202362	0.0169064	0.0037615	0.0034081
ZNF230	0.0369014	0.0340609	0.0254008	0.0462379	0.0234494
ZNF222-DT	0.0015920	0.0000000	0.0000000	0.0000000	0.0000000
ZNF222	0.0618790	0.0559795	0.0636313	0.0979353	0.0681888
ZNF223	0.0601626	0.0770197	0.0572203	0.0327365	0.0668565
ZNF284	0.0281391	0.0407366	0.0253359	0.0374442	0.0280232
ZNF224	0.0380472	0.0527266	0.0461430	0.0233504	0.0670719
ZNF225-AS1	0.0321964	0.0547578	0.0575758	0.0293579	0.0678646
ZNF225	0.0209860	0.0356848	0.0076619	0.0276762	0.0340991
ENSG00000289428	0.0220145	0.0279940	0.0346178	0.0078653	0.0144750
ZNF234	0.0617819	0.0552000	0.0530620	0.0805262	0.0597626
ENSG00000287035	0.0000000	0.0000000	0.0000000	0.0000000	0.0076833
ZNF226	0.2672636	0.2472416	0.2826600	0.2421440	0.3060440
ZNF227	0.0770851	0.0874473	0.0904289	0.1029792	0.1013854
ZNF235	0.0761892	0.0896975	0.0640690	0.0547784	0.2621717
ZNF233	0.0228464	0.0277018	0.0284527	0.0259297	0.0350897
ZNF112	0.0522459	0.0568177	0.0690896	0.0603561	0.0730815
ZNF285	0.0473002	0.0547956	0.0316743	0.0224556	0.0525320
ZNF229	0.0567964	0.0787029	0.0730605	0.0523330	0.0950111
ZNF180	0.0647020	0.0648371	0.0491806	0.0369827	0.0559295
CEACAM22P	0.0049540	0.0014749	0.0026080	0.0117068	0.0000000
IGSF23	0.0000000	0.0009065	0.0000000	0.0000000	0.0000000
CEACAM16-AS1	0.0078556	0.0047493	0.0033456	0.0000000	0.0014182
PVR	0.0992135	0.1116041	0.1208733	0.1082306	0.1706799
CEACAM19	0.0110897	0.0199651	0.0185321	0.0150387	0.0652188
BCL3	0.0608659	0.0629517	0.1033910	0.0422622	0.0904060
CBLC	0.0000000	0.0000000	0.0009760	0.0000000	0.0000000
BCAM	0.0706545	0.0452716	0.0378477	0.1207931	0.0230629

NECTIN2	0.2005833	0.1598265	0.1653290	0.3154413	0.2315613
ENSG00000267282	0.0000000	0.0013594	0.0013406	0.0026458	0.0000000
TOMM40	0.2437749	0.2541793	0.3567991	0.1952669	0.2389907
APOE	0.0357526	0.0365554	0.0323843	0.0769205	0.0267841
APOC1	0.0157593	0.0466083	0.0536058	0.0197964	0.0421797
APOC1P1	0.0003474	0.0000000	0.0024440	0.0000000	0.0015176
APOC4	0.0000000	0.0000000	0.0000000	0.0000000	0.0079441
APOC2	0.0000000	0.0000000	0.0000000	0.0000000	0.0000000
ENSG00000267114	0.0002635	0.0051832	0.0007835	0.0000000	0.0176616
CLPTM1	0.4112613	0.3478347	0.3707310	0.4633908	0.3341424
RELB	0.0165339	0.0142337	0.0091524	0.0051120	0.0162119
CLASRP	0.1018673	0.1079209	0.0747277	0.0783841	0.1719292
ZNF296	0.0059503	0.0030269	0.0091235	0.0023202	0.0000000
GEMIN7-AS1	0.0647047	0.0391349	0.0510734	0.0767560	0.0805131
GEMIN7	0.0791426	0.0816396	0.1186458	0.0788114	0.0619215
MARK4	0.2455298	0.3577199	0.3574737	0.2178068	0.3825005
PPP1R37	0.1179135	0.1192340	0.1131950	0.0971489	0.1078271
NKPD1	0.0011932	0.0046622	0.0104599	0.0000000	0.0014182
TRAPPC6A	0.3043220	0.2011003	0.1345950	0.2950975	0.1681895
BLOC1S3	0.0362808	0.0320503	0.0487526	0.0624907	0.0199355
EXOC3L2	0.0026503	0.0000000	0.0021106	0.0000000	0.0046792
CKM	0.0027182	0.0025794	0.0040303	0.0000000	0.0050730
KLC3	0.0134440	0.0232621	0.0166406	0.0103844	0.0370308
ERCC2	0.0686493	0.0692967	0.0733364	0.0679465	0.0645584
PPP1R13L	0.0044374	0.0067625	0.0010211	0.0015751	0.0282918
POLR1G	0.0739572	0.0479562	0.0704262	0.1026907	0.0541743
ERCC1	0.3812884	0.3475818	0.3663180	0.3826157	0.3261223
FOSB	0.1253165	0.0932747	0.0955115	0.0803440	0.1709563
RTN2	0.1040641	0.2410094	0.2852577	0.0792448	0.1770977
PPM1N	0.0035578	0.0139400	0.0054063	0.0000000	0.0202173
VASP	0.1624701	0.1079298	0.0885083	0.1555593	0.1801601
OPA3	0.1033415	0.0973407	0.1040177	0.0865181	0.0983229
EML2	0.2630508	0.2852119	0.2918229	0.2965628	0.2442564
EML2-AS1	0.0012093	0.0009486	0.0000000	0.0000000	0.0000000
GIPR	0.0414898	0.0150768	0.0111262	0.0283603	0.0230173
SNRPD2	1.5110035	1.4896336	1.4901160	1.4543846	1.2501731
QPCTL	0.0262298	0.0166955	0.0098868	0.0242649	0.0497764
FBXO46	0.0821178	0.0767060	0.0629451	0.0705121	0.0600511
MEIOSIN	0.0020752	0.0021354	0.0000000	0.0000000	0.0000000
SIX5	0.0952330	0.0505984	0.0277547	0.1058666	0.0499299
ENSG00000259605	0.0492739	0.0267080	0.0143335	0.0346272	0.0561406
DM1-AS	0.0090324	0.0071078	0.0000000	0.0015022	0.0000000
DMPK	0.0352421	0.0502770	0.0480911	0.0157622	0.1114102
DMWD	0.2228235	0.2257121	0.2401676	0.2175372	0.2240825
RSPH6A	0.0000000	0.0037285	0.0015401	0.0000000	0.0135827
SYMPK	0.1600795	0.1559174	0.1374985	0.1787036	0.3265888
FOXA3	0.0000000	0.0010432	0.0000000	0.0000000	0.0042946
IRF2BP1	0.1428779	0.1058261	0.1548673	0.1468636	0.1067834
MYPOP	0.0483059	0.0432289	0.0431578	0.0438442	0.0481937
NANOS2	0.0000000	0.0013989	0.0000000	0.0000000	0.0000000
NOVA2	0.2471574	0.3511629	0.3597582	0.2251274	0.4045170
CCDC61	0.0265885	0.0271393	0.0256634	0.0324631	0.0430135
PGLYRP1	0.0031317	0.0000000	0.0000000	0.0000000	0.0019007

ENSG00000268401	0.0029566	0.0024909	0.0020393	0.0025788	0.0158178
IGFL4	0.0171187	0.0189070	0.0381281	0.0107379	0.0714081
ENSG00000267922	0.0031698	0.0075493	0.0100216	0.0029828	0.0528262
IGFL2	0.0039090	0.0011956	0.0017326	0.0031073	0.0000000
ENSG00000269729	0.0027478	0.0046195	0.0016977	0.0100152	0.0000000
ENSG00000268460	0.0041356	0.0000000	0.0012547	0.0000000	0.0115551
HIF3A	0.0727374	0.0922148	0.0697738	0.0808827	0.1218198
ENSG00000269124	0.0000000	0.0032031	0.0000000	0.0000000	0.0000000
PPP5C	0.2763488	0.2721030	0.3138735	0.2996911	0.2638222
ENSG00000268810	0.0007250	0.0007434	0.0027720	0.0000000	0.0075252
ENSG00000269151	0.0152960	0.0052003	0.0183107	0.0116437	0.0107934
CCDC8	0.2264931	0.1368949	0.0606696	0.2360618	0.0759049
PNMA8C	0.1772534	0.1823027	0.2741809	0.1653927	0.1885786
PNMA8A	0.3493808	0.4059592	0.6210895	0.2522508	0.3042633
PPP5D1P	0.0748072	0.0723999	0.0799843	0.0806181	0.0751271
PNMA8B	0.1085428	0.1479051	0.2210440	0.0595331	0.1818570
ENSG00000204850	0.0010310	0.0000000	0.0000000	0.0000000	0.0000000
CALM3	1.6351097	2.0826313	2.2978916	1.5440721	1.6696647
ENSG00000269292	0.0033867	0.0088838	0.0050277	0.0062298	0.0225401
PTGIR	0.0005292	0.0000000	0.0000000	0.0000000	0.0000000
GNG8	0.0182403	0.0388641	0.0528525	0.0163056	0.0089974
DACT3	0.1928017	0.3200385	0.3742077	0.1696858	0.2416366
DACT3-AS1	0.0062787	0.0121721	0.0074409	0.0068860	0.0183942
PRKD2	0.0645038	0.0443726	0.0433731	0.0395357	0.0796853
STRN4	0.2086711	0.2509020	0.3365375	0.1502472	0.2533650
FKRP	0.1325151	0.1306085	0.0902806	0.1610714	0.1752776
SLC1A5	0.1417182	0.1162820	0.0626527	0.2065124	0.1551319
ENSG00000275719	0.0070615	0.0047053	0.0007280	0.0000000	0.0000000
AP2S1	0.7644183	0.8550504	0.9605717	0.7717444	0.6804121
ARHGAP35	0.6649715	0.8263397	0.9399727	0.6462103	0.9016890
NPAS1	0.0165756	0.0267871	0.0474583	0.0146053	0.0196784
TMEM160	0.4607127	0.7020362	0.7413089	0.4806450	0.4892899
ZC3H4	0.1493565	0.1337998	0.1097276	0.1676248	0.1830850
SAE1	0.4927233	0.4286219	0.4808684	0.5225337	0.4420157
BBC3	0.3859766	0.3351746	0.2786625	0.3516068	0.2564297
ENSG00000289915	0.0254281	0.0302191	0.0141475	0.0320303	0.0278579
ENSG00000286669	0.0107333	0.0015840	0.0019052	0.0000000	0.0042647
CCDC9	0.0520653	0.0282660	0.0384124	0.0457772	0.0534667
INAFM1	0.2776276	0.3631126	0.5091777	0.3004992	0.2273209
C5AR1	0.0258884	0.0183616	0.0249849	0.0567911	0.0454550
DHX34	0.0252452	0.0262510	0.0178620	0.0239420	0.0396017
ENSG00000288827	0.0261796	0.0177806	0.0244941	0.0270061	0.0439413
MEIS3	0.3664143	0.5810371	0.5575134	0.3712102	0.4758515
SLC8A2	0.0341577	0.0654874	0.1156048	0.0341843	0.1660523
ENSG00000287896	0.0016409	0.0010502	0.0000000	0.0030153	0.0014580
KPTN	0.0637344	0.0610832	0.0706125	0.0409347	0.0443847
NAPA-AS1	0.0536995	0.0265674	0.0380755	0.0515897	0.0263905
NAPA	0.4511178	0.3745464	0.4315087	0.4052596	0.3429891
ZNF541	0.0000000	0.0033918	0.0030475	0.0000000	0.0032018
ENSG00000268746	0.0014879	0.0000000	0.0000000	0.0055630	0.0033430
ENSG00000277383	0.0058059	0.0058812	0.0027113	0.0069550	0.0039666
BICRA	0.0349953	0.0371659	0.0534750	0.0224917	0.0570733
BICRA-AS1	0.0009580	0.0020024	0.0025607	0.0000000	0.0031970

EHD2	0.0804872	0.0370077	0.0187131	0.0793890	0.0479023
NOP53	0.8715948	0.8397811	0.7235168	0.8547027	0.6821964
NOP53-AS1	0.0006692	0.0015654	0.0000000	0.0000000	0.0024001
SELENOW	1.8267202	1.9126249	1.9128658	1.8683666	1.5911796
CRX	0.0000000	0.0000000	0.0000000	0.0000000	0.0000000
ELSPBP1	0.0009864	0.0000000	0.0000000	0.0000000	0.0000000
CABP5	0.0000000	0.0000000	0.0000000	0.0000000	0.0000000
PLA2G4C	0.0681920	0.0568969	0.0540175	0.0277055	0.1325334
PLA2G4C-AS1	0.0000000	0.0000000	0.0000000	0.0000000	0.0000000
LIG1	0.1232699	0.1159477	0.1049897	0.0996462	0.1792806
ENSG00000269534	0.0000000	0.0000000	0.0000000	0.0000000	0.0000000
ZSWIM9	0.1008739	0.1344209	0.1782063	0.1071484	0.1369247
CARD8	0.1226661	0.1209299	0.0869430	0.1337960	0.0772015
ENSG00000268583	0.0016179	0.0026167	0.0000000	0.0000000	0.0103869
CARD8-AS1	0.0103159	0.0140860	0.0084439	0.0082011	0.0080186
ZNF114-AS1	0.0027829	0.0000000	0.0035122	0.0000000	0.0019139
ZNF114	0.0171401	0.0209729	0.0435344	0.0211299	0.0097388
ODAD1	0.0620111	0.0377676	0.0293383	0.0514792	0.0605522
EMP3	0.6061966	0.3992942	0.2921012	0.8117938	0.3231794
TMEM143	0.0499099	0.0572816	0.0571081	0.0474163	0.0424499
SYNGR4	0.0000000	0.0031239	0.0000000	0.0000000	0.0083414
KDELR1	1.5215849	1.3430987	1.2967440	1.6369429	1.1676795
GRIN2D	0.0865532	0.1170957	0.1497525	0.0482828	0.2225643
GRWD1	0.0712223	0.0678378	0.0926972	0.0685967	0.0643149
KCNJ14	0.0224571	0.0283510	0.0289773	0.0217564	0.0430357
ENSG00000268530	0.0020119	0.0000000	0.0000000	0.0000000	0.0000000
CYTH2	0.2669722	0.2268541	0.2446979	0.2805930	0.2719118
LMTK3	0.0527796	0.0887100	0.1095274	0.0441430	0.1581034
ENSG00000287603	0.0011101	0.0012481	0.0000000	0.0000000	0.0053148
SULT2B1	0.0047997	0.0068930	0.0017569	0.0000000	0.0021357
FAM83E	0.0019936	0.0054866	0.0025278	0.0033625	0.0067035
RPL18	2.8208814	2.8183674	2.6687820	2.7323289	2.5144303
ENSG00000268093	0.0022766	0.0033857	0.0044193	0.0000000	0.0162396
SPHK2	0.0447074	0.0375259	0.0326403	0.0513914	0.0471164
DBP	0.1933904	0.0934088	0.0704325	0.1673893	0.1246662
CA11	0.1131638	0.1343313	0.1402711	0.0667867	0.1213292
NTN5	0.0037000	0.0035857	0.0015657	0.0012911	0.0019259
FUT2	0.0000000	0.0029044	0.0016688	0.0000000	0.0042743
MAMSTR	0.0149187	0.0211470	0.0182090	0.0302002	0.0139656
RASIP1	0.0013442	0.0072338	0.0012238	0.0068032	0.0101255
IZUMO1	0.0000000	0.0010260	0.0000000	0.0033434	0.0000000
FUT1	0.0076242	0.0132979	0.0255675	0.0054177	0.0041311
BCAT2	0.2854877	0.2980286	0.2435685	0.1878563	0.2171206
ENSG00000286024	0.0033713	0.0098508	0.0000000	0.0015092	0.0062467
HSD17B14	0.2773184	0.1540539	0.0849322	0.3264567	0.1111885
PLEKHA4	0.0372899	0.0371590	0.0121098	0.0221636	0.0036741
PPP1R15A	0.9689704	0.7675705	0.6705339	0.7868589	0.7454068
TULP2	0.0025505	0.0006091	0.0055814	0.0036850	0.0083570
NUCB1	0.5752101	0.4321029	0.3936862	0.8115379	0.3942021
NUCB1-AS1	0.0000000	0.0017536	0.0000000	0.0038158	0.0015201
ENSG00000260366	0.0011814	0.0000000	0.0000000	0.0000000	0.0000000
DHDH	0.0092090	0.0213027	0.0262088	0.0178309	0.0031652
BAX	0.6220682	0.4610234	0.4093939	0.7333399	0.3394924

FTL	4.0999738	3.7749968	3.4447472	3.9250444	3.6199792
GYS1	0.0835529	0.0595337	0.0593144	0.0910642	0.1358999
RUVBL2	0.3463403	0.3599304	0.3894446	0.3457456	0.2641172
NTF4	0.0000000	0.0000000	0.0000000	0.0023493	0.0000000
SNRNP70	0.5562070	0.4928651	0.4982354	0.5471727	0.5502233
LIN7B	0.3162504	0.4882275	0.6285588	0.2571172	0.3014353
C19orf73	0.0247355	0.0365850	0.0161429	0.0373849	0.0070119
PPFIA3	0.1333112	0.2017476	0.1668356	0.1137110	0.2640584
HRC	0.0167843	0.0131323	0.0189447	0.0124067	0.0250996
TRPM4	0.0406392	0.0623023	0.0435583	0.0677433	0.1691889
SLC6A16	0.0390054	0.0365541	0.0164004	0.0219241	0.0212734
ENSG00000197813	0.0027521	0.0090209	0.0021106	0.0072222	0.0000000
CD37	0.0037560	0.0042619	0.0007329	0.0056803	0.0000000
TEAD2	0.0716727	0.0381552	0.0124029	0.1266570	0.0205222
DKKL1	0.0318256	0.0233059	0.0230567	0.0463608	0.0000000
GFY	0.0342014	0.0629459	0.0150578	0.0442217	0.0129033
SLC17A7	0.0012124	0.0085535	0.0000000	0.0000000	0.0124645
PIH1D1	0.4340444	0.3529232	0.2987298	0.4989411	0.3105882
ALDH16A1	0.0205334	0.0196197	0.0259227	0.0124315	0.0234185
FLT3LG	0.0130292	0.0122732	0.0065777	0.0103230	0.0062104
RPL13A	3.4592880	3.4179519	3.2092814	3.3496109	3.1770841
RPS11	2.7287201	2.6868255	2.5022965	2.5962884	2.4789460
FCGRT	0.2087356	0.1170669	0.0725430	0.2638859	0.1255228
RCN3	0.0135009	0.0077703	0.0162249	0.0051952	0.0116649
NOSIP	0.2698604	0.2945022	0.2874562	0.2994661	0.2391351
PRRG2	0.0036471	0.0023424	0.0055016	0.0000000	0.0051073
PRR12	0.1264121	0.1496792	0.2033328	0.0981202	0.2152117
RRAS	0.2971453	0.1550356	0.1181096	0.3541779	0.1025584
SCAF1	0.1457964	0.1736026	0.1869893	0.1430878	0.1861595
IRF3	0.1728184	0.1187851	0.0950667	0.1884129	0.1134929
BCL2L12	0.0632812	0.0394162	0.0488986	0.0844208	0.0392949
PRMT1	0.9620212	0.9760897	1.1085527	0.9393273	0.7568621
ADM5	0.0036650	0.0010476	0.0006749	0.0000000	0.0118693
CPT1C	0.0633523	0.0691314	0.0905391	0.0516875	0.1345675
AP2A1	0.3336696	0.3908462	0.5161550	0.3649078	0.3259726
FUZ	0.1885068	0.1014242	0.1106503	0.1381500	0.0943800
ENSG00000269194	0.0706921	0.0411014	0.0367983	0.0372424	0.0412644
MED25	0.1416169	0.1261005	0.1363922	0.1397824	0.1357621
PTOV1-AS1	0.0100055	0.0127689	0.0071409	0.0158959	0.0132921
PTOV1	0.9017522	0.9012210	0.9867133	0.9132119	0.7202773
PNKP	0.0673957	0.0660411	0.0643775	0.1028850	0.1193095
AKT1S1	0.1162123	0.1058128	0.0738148	0.1107245	0.0908366
TBC1D17	0.1540257	0.1097418	0.1055470	0.0581100	0.1125514
IL4I1	0.0026802	0.0004197	0.0032395	0.0132477	0.0015201
NUP62	0.1705378	0.1636530	0.1555873	0.1718634	0.0974308
ATF5	1.9181152	1.5508811	1.2416630	1.4921528	1.4062219
SIGLEC11	0.0000000	0.0000000	0.0000000	0.0000000	0.0000000
VRK3	0.2026318	0.1836574	0.1808401	0.2367690	0.1787206
ZNF473	0.0317502	0.0411900	0.0322855	0.0397193	0.0226531
ENSG00000269091	0.0016770	0.0000000	0.0007884	0.0015674	0.0039740
ZNF473CR	0.0155371	0.0289927	0.0304878	0.0109487	0.0174795
ENSG00000267890	0.0102763	0.0171713	0.0117493	0.0193026	0.0054053
ENSG00000287001	0.0008681	0.0005109	0.0055700	0.0071970	0.0115214

MYH14	0.0042306	0.0000000	0.0020060	0.0000000	0.0111101
KCNC3	0.0046856	0.0143458	0.0049207	0.0000000	0.0199387
NR1H2	0.2329965	0.1603158	0.0924258	0.1271572	0.1477687
NAPSB	0.0000000	0.0000000	0.0000000	0.0000000	0.0000000
NAPSA	0.0022571	0.0008861	0.0024542	0.0000000	0.0000000
ENSG00000269392	0.0000000	0.0000000	0.0000000	0.0000000	0.0000000
POLD1	0.0266903	0.0174407	0.0209372	0.0202900	0.0301350
MYBPC2	0.0000000	0.0025345	0.0000000	0.0000000	0.0043250
GARIN5A	0.0940829	0.1239291	0.1748660	0.1291854	0.0967991
EMC10	0.4861635	0.3858963	0.4401221	0.6365787	0.3319319
ENSG00000268854	0.0056760	0.0000000	0.0042506	0.0048605	0.0053181
ENSG00000289950	0.0014776	0.0000000	0.0000000	0.0000000	0.0000000
JOSD2	0.0576462	0.0857395	0.0671353	0.0820578	0.0750750
ASPDH	0.0248601	0.0148486	0.0093871	0.0097060	0.0292156
LRRC4B	0.2489890	0.2837122	0.3438194	0.3066872	0.3647448
SYT3	0.0225572	0.0745186	0.1004704	0.0415465	0.0310704
C19orf81	0.1026003	0.0564154	0.0511012	0.0835380	0.0787606
SHANK1	0.0726296	0.1417272	0.1235828	0.0358033	0.3027495
CLEC11A	0.1432717	0.1411985	0.1827948	0.1594433	0.0802381
SMIM47	0.0051073	0.0136895	0.0117925	0.0030390	0.0268386
ACP4	0.0000000	0.0009136	0.0000000	0.0000000	0.0000000
ENSG00000268375	0.0016066	0.0000000	0.0000000	0.0000000	0.0059207
KLK15	0.0007702	0.0000000	0.0060738	0.0000000	0.0044531
KLKP1	0.0000000	0.0000000	0.0012099	0.0000000	0.0000000
KLK4	0.0016534	0.0032327	0.0012543	0.0000000	0.0168362
KLK10	0.0142519	0.0277562	0.0317637	0.0053117	0.0064560
KLK13	0.0009065	0.0025675	0.0051434	0.0000000	0.0036060
KLK14	0.0000000	0.0015696	0.0000000	0.0000000	0.0019259
CTU1	0.0807965	0.0945372	0.0710667	0.0824036	0.0343529
IGLON5	0.1686401	0.3266495	0.4252244	0.1499805	0.2448928
VSIG10L	0.0417550	0.0683384	0.0785863	0.0458008	0.0346295
ENSG00000267984	0.0007600	0.0012251	0.0000000	0.0000000	0.0000000
ETFB	0.6723432	0.8098644	0.9378490	0.6377037	0.5951482
CLDND2	0.0722391	0.0483868	0.0524275	0.0357646	0.0599745
LIM2-AS1	0.0000000	0.0014467	0.0000000	0.0000000	0.0000000
ZNF175	0.0474007	0.0346044	0.0473293	0.0256353	0.0550975
LINC01530	0.0052485	0.0036682	0.0062529	0.0069806	0.0031652
SPACA6-AS1	0.0000000	0.0012106	0.0000000	0.0000000	0.0000000
SPACA6	0.0516379	0.0412728	0.0354017	0.0421213	0.0791501
HAS1	0.0064419	0.0166339	0.0406269	0.0000000	0.0222062
FPR1	0.0000000	0.0040567	0.0000000	0.0000000	0.0000000
FPR3	0.0000000	0.0000000	0.0000000	0.0000000	0.0000000
ZNF577	0.1674498	0.1570627	0.0938051	0.1636952	0.2277992
ZNF649-AS1	0.0000000	0.0000000	0.0000000	0.0000000	0.0000000
ZNF649	0.1591226	0.1225643	0.1245351	0.1654069	0.1057009
ZNF613	0.0356211	0.0333755	0.0436296	0.0427631	0.0207256
ZNF350-AS1	0.0168016	0.0209141	0.0123398	0.0034029	0.0359440
ZNF350	0.0657683	0.0450700	0.0608876	0.0507977	0.0482903
ZNF615	0.0527348	0.0536133	0.0604455	0.0699600	0.0729330
ZNF614	0.1221779	0.1167517	0.1157595	0.0807786	0.0778258
ZNF432	0.1566952	0.1364271	0.1094105	0.1704192	0.1214614
ENSG00000275055	0.0335706	0.0209167	0.0167416	0.0172512	0.0374426
ENSG00000260160	0.0040661	0.0107424	0.0151566	0.0079971	0.0110045

ZNF841	0.0607813	0.0690523	0.0596708	0.0607023	0.0855875
ZNF616	0.0798610	0.0817288	0.0948220	0.0770306	0.0983655
ENSG00000268458	0.0057901	0.0111381	0.0053631	0.0059145	0.0248045
ZNF836	0.0863687	0.1112144	0.1515361	0.1020279	0.1515530
PPP2R1A	1.2984497	1.3108571	1.4189201	1.3135989	1.1812919
ENSG00000267927	0.0000000	0.0000000	0.0027245	0.0000000	0.0028551
ENSG00000268015	0.0000000	0.0000000	0.0000000	0.0000000	0.0027533
ZNF766	0.1991879	0.2196853	0.1843318	0.2239080	0.1532108
ENSG00000269102	0.0008209	0.0000000	0.0000000	0.0000000	0.0068101
ZNF480	0.1483854	0.1449464	0.1170915	0.1069753	0.0984671
ENSG00000269535	0.0004268	0.0000000	0.0035878	0.0000000	0.0072872
ZNF610	0.1092144	0.1169257	0.1113650	0.1194124	0.0943295
ZNF880	0.1801008	0.2008959	0.1927326	0.2124742	0.1719312
ZNF528-AS1	0.1180151	0.1059119	0.1489132	0.1523812	0.1395082
ZNF528	0.1508598	0.1669037	0.1519913	0.1408168	0.2093228
ZNF534	0.0129371	0.0204562	0.0239541	0.0012897	0.0118995
ZNF578	0.0437410	0.0803215	0.0961046	0.0598601	0.0791051
ENSG00000288253	0.0014736	0.0023272	0.0072482	0.0025471	0.0000000
ZNF808	0.1356471	0.1483127	0.1569755	0.1160966	0.1110577
ZNF701	0.0558937	0.0677279	0.0828533	0.0540537	0.0562793
ENSG00000289102	0.0032305	0.0156888	0.0179662	0.0000000	0.0107916
ENSG00000268886	0.0013569	0.0010432	0.0010835	0.0000000	0.0000000
ENSG00000290721	0.0016292	0.0005156	0.0000000	0.0000000	0.0000000
ZNF83	0.2277659	0.3304157	0.3155601	0.2660088	0.5524929
ENSG00000290073	0.0029760	0.0132158	0.0082132	0.0040308	0.0149651
ENSG00000269825	0.0097818	0.0207471	0.0240056	0.0199565	0.0739969
ENSG00000288953	0.0087470	0.0146719	0.0104135	0.0023869	0.0031863
ZNF611	0.1064791	0.1353183	0.1380572	0.1034823	0.1639990
ZNF600	0.0100837	0.0132473	0.0082422	0.0122349	0.0100331
ZNF28	0.0713173	0.0829793	0.0480490	0.1481218	0.0684607
ZNF468	0.0396435	0.0347541	0.0410714	0.0125340	0.0425473
ZNF320	0.1340856	0.1229002	0.1033836	0.1276273	0.2185451
ZNF888	0.0257258	0.0105243	0.0116541	0.0442994	0.0294513
ZNF888-AS1	0.0060993	0.0036351	0.0022987	0.0000000	0.0012783
ZNF816	0.0874735	0.0655225	0.0315864	0.0770517	0.0435432
ZNF160	0.2867053	0.2493719	0.2235065	0.2897303	0.1884549
ZNF415	0.1874244	0.2530394	0.2809694	0.2082901	0.1349533
ZNF347	0.0757722	0.0798532	0.0726580	0.0599047	0.1159248
ZNF665	0.0870503	0.0594727	0.0628052	0.1174269	0.0769736
ENSG00000291131	0.0296618	0.0404690	0.0253835	0.0425986	0.0211142
ZNF677	0.2020071	0.2182783	0.2449251	0.2173697	0.1640034
ENSG00000269288	0.0014787	0.0028541	0.0000000	0.0035783	0.0000000
ZNF845	0.0401745	0.0537334	0.0283253	0.0281483	0.0293762
ZNF525	0.0362390	0.0601985	0.0418038	0.0402951	0.0913536
ZNF765	0.0754785	0.0851984	0.0866337	0.0585379	0.0960757
ZNF761	0.1396510	0.1266355	0.0923506	0.0729830	0.0812604
ENSG00000287608	0.0026962	0.0034482	0.0020977	0.0075526	0.0088757
ZNF813	0.0737147	0.0628664	0.0374605	0.0764282	0.0759935
ZNF331	0.1882508	0.1904310	0.2555363	0.1863023	0.2489986
DPRX	0.0000000	0.0012089	0.0041465	0.0027524	0.0186059
ENSG00000269842	0.0000000	0.0000000	0.0000000	0.0000000	0.0032941
NLRP12	0.0000000	0.0000000	0.0000000	0.0000000	0.0000000
MYADM-AS1	0.0015308	0.0021732	0.0000000	0.0000000	0.0042436

MYADM	0.3214648	0.2908927	0.3000802	0.3457672	0.2346627
ENSG00000232220	0.0030660	0.0006888	0.0000000	0.0068945	0.0035288
PRKCG	0.0198771	0.0304702	0.0178310	0.0000000	0.0701322
CACNG7	0.1002562	0.1667478	0.2254404	0.0918018	0.1342112
CACNG8	0.0526207	0.1295157	0.1583812	0.0755105	0.1483292
CACNG6	0.0484369	0.0281058	0.0139911	0.0220754	0.0226998
OSCAR	0.0012472	0.0024851	0.0131933	0.0000000	0.0098269
NDUFA3	0.9130250	0.9684069	1.0007745	0.9431045	0.7743874
TFPT	0.1908577	0.1584154	0.1545966	0.1948810	0.0837607
PRPF31	0.3929847	0.2980164	0.3025606	0.3703523	0.2605211
CNOT3	0.2238362	0.1794599	0.1887645	0.2077201	0.1778985
LENG1	0.1424691	0.1336600	0.1441388	0.1218963	0.1605503
TMC4	0.0127270	0.0040335	0.0098344	0.0017792	0.0213202
MBOAT7	0.2345950	0.1985740	0.1756312	0.3294609	0.1850814
TSEN34	0.6047470	0.5427924	0.5514132	0.6101476	0.4343345
RPS9	2.0102460	1.9437804	1.7657150	1.8803184	1.7239904
LILRB3	0.0016601	0.0025229	0.0000000	0.0000000	0.0013244
LAIR1	0.0000000	0.0000000	0.0000000	0.0000000	0.0000000
TTYH1	0.8671167	0.5700976	0.4213626	1.7771565	0.6068000
ENSG00000268496	0.0023906	0.0008736	0.0000000	0.0025427	0.0000000
ENSG00000269873	0.0010438	0.0000000	0.0000000	0.0025261	0.0000000
ENSG00000267838	0.0003108	0.0021976	0.0000000	0.0000000	0.0193796
LENG8-AS1	0.0277357	0.0191300	0.0204784	0.0216857	0.0575306
LENG8	0.0878875	0.0967460	0.0745149	0.0890720	0.2320711
LENG9	0.0193970	0.0208487	0.0214345	0.0241773	0.0308383
CDC42EP5	0.0049012	0.0031976	0.0050519	0.0000000	0.0045380
FCAR	0.0015187	0.0000000	0.0000000	0.0000000	0.0000000
NLRP7	0.0000000	0.0011185	0.0000000	0.0000000	0.0000000
NLRP2	0.0296663	0.0342274	0.0348763	0.0151786	0.0271965
GP6-AS1	0.0218454	0.0252195	0.0189267	0.0029390	0.1036851
GP6	0.0102590	0.0081391	0.0069579	0.0000000	0.0618012
RDH13	0.0770448	0.0665678	0.0859365	0.0706378	0.0958706
EPS8L1	0.0353810	0.0815184	0.1031395	0.0324852	0.0314233
PPP1R12C	0.0779398	0.0711422	0.0766751	0.0617540	0.1563795
ENSG00000287170	0.0023013	0.0058803	0.0085232	0.0000000	0.0037415
TNNT1	0.0175889	0.0409824	0.0595408	0.0320210	0.0203488
TNNI3	0.0234785	0.0362790	0.0244232	0.0187968	0.0219731
DNAAF3	0.0419853	0.0138751	0.0072070	0.0336652	0.0337941
DNAAF3-AS1	0.0010515	0.0010959	0.0000000	0.0000000	0.0049014
SYT5	0.1632266	0.2521506	0.3861272	0.1025195	0.2808999
PTPRH	0.0117913	0.0203565	0.0204236	0.0038597	0.0111321
ENSG00000286230	0.0051637	0.0067853	0.0065601	0.0020370	0.0230447
ENSG00000267649	0.0057453	0.0052095	0.0084173	0.0000000	0.0033723
TMEM86B	0.0073524	0.0114884	0.0125705	0.0039833	0.0175737
PPP6R1	0.1199076	0.1854164	0.1751739	0.1395383	0.1749419
HSPBP1	0.3889912	0.3526478	0.4387405	0.4027867	0.2647794
BRSK1	0.2281613	0.4242587	0.4351593	0.1968169	0.3935047
TMEM150B	0.0000000	0.0000000	0.0000000	0.0000000	0.0049014
ENSG00000276831	0.0009454	0.0003671	0.0014274	0.0020579	0.0000000
KMT5C	0.0480524	0.0455792	0.0377857	0.0407355	0.0637516
COX6B2	0.0044190	0.0043938	0.0300263	0.0000000	0.0023227
IL11	0.0008275	0.0019441	0.0163745	0.0026924	0.0081930
TMEM190	0.1559442	0.0758866	0.0620382	0.1934878	0.0809657

TMEM238	0.0135842	0.0146523	0.0070824	0.0205351	0.0240001
RPL28	2.6936795	2.6433042	2.4475619	2.5907174	2.3325479
UBE2S	0.5808354	0.7979438	0.9220711	0.5673923	0.5985829
SHISA7	0.0199019	0.0425799	0.0466455	0.0259161	0.0737236
ISOC2	0.3184798	0.2717886	0.2640221	0.3693377	0.2200027
ZNF628	0.0101522	0.0169570	0.0172147	0.0033872	0.0215587
NAT14	0.4290607	0.3095806	0.3467301	0.5006893	0.2867993
SSC5D	0.0033601	0.0035892	0.0000000	0.0065034	0.0243041
ZNF579	0.1211575	0.1305750	0.1567544	0.1319212	0.1015079
FIZ1	0.0638502	0.0370998	0.0507816	0.0582145	0.0545758
ZNF524	0.0630848	0.0367705	0.0248171	0.0650287	0.0236231
ZNF865	0.0329896	0.0268385	0.0210312	0.0435975	0.0494666
ZNF784	0.0438808	0.0334478	0.0163486	0.0320794	0.0213093
ZNF580	0.4856174	0.4819772	0.5557231	0.4349475	0.4272203
ZNF581	0.1912112	0.1646230	0.1183304	0.1464305	0.1103602
CCDC106	0.1713081	0.2069426	0.2803698	0.1921492	0.1470170
U2AF2	0.2533706	0.2393839	0.2608500	0.2951803	0.2115856
ENSG00000267523	0.0011311	0.0010000	0.0000000	0.0000000	0.0067015
ENSG00000267096	0.0000000	0.0011512	0.0022620	0.0000000	0.0000000
EPN1	0.3976766	0.4967480	0.5843822	0.3622187	0.4324317
NLRP9	0.0000000	0.0014940	0.0000000	0.0000000	0.0016664
NLRP8	0.0000000	0.0000000	0.0000000	0.0000000	0.0000000
NLRP5	0.0000000	0.0000000	0.0000000	0.0000000	0.0000000
LINC01864	0.0008003	0.0102635	0.0090238	0.0000000	0.0033235
ZNF787	0.1954945	0.1854352	0.1858549	0.1753189	0.1375590
ZNF444	0.1293964	0.1059902	0.1191912	0.1221245	0.1257610
GALP	0.0010378	0.0010029	0.0000000	0.0000000	0.0035086
ZSCAN5A	0.1516764	0.1166925	0.0925111	0.1200069	0.1615354
EDDM13	0.0016563	0.0045854	0.0046293	0.0025261	0.0140994
ENSG00000267298	0.0000000	0.0000000	0.0000000	0.0022972	0.0000000
ZSCAN5A-AS1	0.0118683	0.0058951	0.0034796	0.0091490	0.0229334
ENSG00000267429	0.0006826	0.0006853	0.0000000	0.0000000	0.0069764
ZNF542P	0.2063038	0.2493995	0.2480261	0.2395009	0.2104844
ZNF582	0.0500785	0.0451694	0.0619677	0.0482599	0.0418681
ENSG00000285996	0.0012532	0.0012812	0.0006609	0.0000000	0.0119800
ENSG00000267192	0.0000000	0.0035350	0.0000000	0.0000000	0.0000000
ZNF582-DT	0.0664451	0.0924416	0.1131364	0.0886429	0.0619190
ZNF583	0.1381580	0.2696870	0.2709987	0.1275391	0.2774659
ZNF667	0.1563216	0.1605267	0.1623233	0.1383305	0.1707457
ZNF667-AS1	0.5486451	0.6055883	0.6591475	0.5528547	0.4223409
ZNF471	0.1604279	0.1979258	0.1831405	0.1073054	0.4156047
ENSG00000267224	0.0016402	0.0012039	0.0000000	0.0000000	0.0000000
ZFP28-DT	0.0211352	0.0183156	0.0109393	0.0128690	0.0046843
ZFP28	0.1323248	0.1900196	0.2005108	0.1002764	0.1465518
ZNF470-DT	0.0430738	0.0882182	0.0972443	0.0305944	0.0495557
ZNF470	0.1074917	0.1500551	0.1616449	0.1417289	0.1324371
ZNF71	0.0949945	0.1704869	0.1862966	0.0680367	0.1624810
SMIM17	0.0652708	0.1210356	0.1708667	0.0559906	0.0844453
ZNF835	0.0274716	0.0324779	0.0274431	0.0352933	0.0292067
ENSG00000286125	0.0221018	0.0334622	0.0444473	0.0405173	0.0752764
ZIM2	0.0000000	0.0025667	0.0000000	0.0021445	0.0033508
ZNF264	0.1193850	0.1372877	0.1671287	0.0956290	0.2361297
AURKC	0.0069618	0.0085006	0.0095794	0.0049164	0.0067535

ZNF805	0.0893696	0.0884098	0.0936667	0.0634600	0.0671309
ZNF460-AS1	0.0122694	0.0078218	0.0113145	0.0047102	0.0164611
ZNF460	0.1497646	0.1301364	0.1576636	0.1554658	0.2489962
ENSG00000288899	0.1858314	0.1724512	0.2536028	0.1495908	0.2390377
ENSG00000268205	0.1661355	0.1998324	0.1858885	0.1179232	0.2277292
ZNF543	0.0296293	0.0446555	0.0460296	0.0217218	0.0436266
ENSG00000268678	0.0020679	0.0049019	0.0000000	0.0000000	0.0062902
ZNF304	0.0420970	0.0642377	0.0487043	0.0482017	0.0856209
ZNF547	0.0224751	0.0245896	0.0084334	0.0231901	0.0073281
TRAPPC2B	0.1967838	0.1558617	0.1734443	0.1648568	0.1545715
ZNF548	0.0831461	0.0729493	0.0888630	0.0815336	0.1170302
ZNF17	0.0488166	0.0457939	0.0449766	0.0241856	0.0297489
ZNF749	0.1327788	0.1428040	0.1351779	0.1584416	0.1357361
ENSG00000276449	0.0065230	0.0049222	0.0078782	0.0029649	0.0083798
VN1R1	0.0070934	0.0080637	0.0127683	0.0065253	0.0138837
ZNF772	0.0715007	0.0779107	0.0846578	0.0680971	0.0625660
ENSG00000268266	0.0012311	0.0000000	0.0000000	0.0045090	0.0126350
ZNF419	0.0870096	0.0681748	0.0439318	0.0900130	0.0616229
ZNF773	0.0637781	0.0361150	0.0458918	0.0403617	0.0928950
ZNF549	0.0643142	0.0606781	0.0558592	0.0395187	0.1526344
ZNF550	0.0331478	0.0308202	0.0463868	0.0323085	0.0363914
ZNF416	0.0298652	0.0324105	0.0292785	0.0235850	0.0405429
ZIK1	0.0657505	0.0653042	0.0554712	0.0447810	0.0480848
ZNF530	0.0243416	0.0326825	0.0399566	0.0306593	0.0395967
ZNF134	0.1072758	0.0989497	0.0786977	0.0631466	0.0916580
ENSG00000268392	0.0000000	0.0053368	0.0030192	0.0000000	0.0041152
ZNF211	0.0690391	0.0796594	0.0692149	0.0908555	0.0877656
ZNF551	0.0222882	0.0511194	0.0504885	0.0171459	0.0542888
ZNF154	0.0627181	0.0756298	0.0901293	0.0576777	0.1086247
ZNF671	0.0434202	0.0466728	0.0379685	0.0347219	0.0371184
ZNF776	0.1254520	0.1177088	0.1448367	0.0901945	0.1096473
ZNF586	0.0860898	0.0861770	0.0832461	0.0503141	0.1862286
ZNF552	0.0139690	0.0090944	0.0135032	0.0085984	0.0401347
ZNF587B	0.0120579	0.0131145	0.0112297	0.0119605	0.0937643
ZNF814	0.0772365	0.0814712	0.0806144	0.0843637	0.1281226
ZNF587	0.0823248	0.0793476	0.0588041	0.0631505	0.1016210
ENSG00000269867	0.0014504	0.0000000	0.0000000	0.0000000	0.0089327
ZNF417	0.0662224	0.0701169	0.0632026	0.0380178	0.0614645
ZNF418	0.0421476	0.0490599	0.0462395	0.0292910	0.0784569
ZNF256	0.0449534	0.0331924	0.0344822	0.0483070	0.0205085
C19orf18	0.0047546	0.0051175	0.0000000	0.0024618	0.0215738
ZNF606	0.1298002	0.1143719	0.0781024	0.1223377	0.1411117
ENSG00000176593	0.1356369	0.2669043	0.2594953	0.1547473	0.2118359
ZSCAN1	0.1368483	0.1397161	0.1412939	0.1524705	0.1179070
ENSG00000288830	0.0011211	0.0088672	0.0086641	0.0000000	0.0228326
ZNF135	0.0662295	0.0518612	0.0455264	0.0626791	0.0613734
ZSCAN18	0.4555656	0.3577994	0.3902412	0.4919990	0.3921460
ZNF329	0.1226223	0.1595616	0.1777639	0.1477046	0.1724007
ENSG00000286632	0.0027930	0.0086691	0.0157163	0.0049219	0.0000000
ZNF274	0.1264229	0.1174858	0.0904709	0.1029477	0.1376762
ZNF544	0.3541915	0.3781496	0.3382548	0.3422339	0.4027399
ZNF8-DT	0.0644494	0.0490876	0.0421266	0.0409276	0.1039116
ENSG00000268201	0.0006891	0.0010959	0.0019113	0.0000000	0.0012623

ZNF8	0.1232232	0.1231006	0.1288507	0.0972880	0.1655796
ERVK3-1	0.3240951	0.3586828	0.3563592	0.2996458	0.3466015
ENSG00000283103	0.4082101	0.4275724	0.4123929	0.4376255	0.2794313
ZSCAN22	0.0171298	0.0062557	0.0228466	0.0036596	0.0044314
A1BG	0.2819548	0.2455156	0.1612538	0.2627672	0.1777515
A1BG-AS1	0.0231112	0.0156553	0.0227885	0.0200300	0.0466311
ZNF497	0.0259904	0.0119915	0.0137151	0.0213941	0.0477158
ENSG00000268049	0.0008089	0.0035708	0.0000000	0.0000000	0.0000000
ZNF497-AS1	0.0099857	0.0102425	0.0087784	0.0043972	0.0167837
ZNF837	0.0187875	0.0146504	0.0092106	0.0153168	0.0092493
RPS5	2.0789786	2.1086495	1.9646141	1.9793491	1.7588907
ZNF584	0.1088338	0.1099193	0.1132801	0.1376292	0.1477816
ZNF584-DT	0.0053813	0.0104319	0.0179717	0.0188856	0.0161339
ENSG00000268912	0.0043738	0.0046358	0.0038928	0.0040556	0.0076268
ZNF132	0.0322816	0.0292367	0.0258527	0.0351371	0.0334553
ZNF132-DT	0.0039404	0.0008251	0.0000000	0.0000000	0.0079382
ZNF324B	0.0231135	0.0319568	0.0454686	0.0200376	0.0366586
ZNF324	0.0410922	0.0296129	0.0529363	0.0378514	0.0488722
ZNF446	0.0718538	0.0563516	0.0476117	0.0601873	0.0699240
SLC27A5	0.2252582	0.2740288	0.3259010	0.2083084	0.2329151
ENSG00000273901	0.0000000	0.0000000	0.0000000	0.0027252	0.0000000
ZBTB45	0.0879115	0.1055668	0.1224618	0.1025030	0.0596678
TRIM28	0.4826509	0.4545446	0.4298873	0.3831549	0.3275786
CHMP2A	0.9080229	0.8462516	0.7915126	0.8844212	0.6318640
UBE2M	0.4893685	0.5090632	0.5582529	0.3994852	0.4162026
MZF1-AS1	0.0301680	0.0368706	0.0440788	0.0282050	0.0287527
MZF1	0.0810418	0.0822948	0.1118420	0.0961077	0.0904864
C20orf96	0.3196123	0.2746018	0.2456597	0.2657469	0.1929894
ZCCHC3	0.2321410	0.1989915	0.1831521	0.2059570	0.1590272
ENSG00000286158	0.0022956	0.0000000	0.0000000	0.0058732	0.0000000
NRSN2-AS1	0.0993994	0.1214237	0.1202172	0.0768895	0.1304801
SOX12	0.3356947	0.5111057	0.4742948	0.3219087	0.4326978
NRSN2	0.2030431	0.2319673	0.2739142	0.1947316	0.1113046
TRIB3	0.5293741	0.3298688	0.2287084	0.4085039	0.3162440
RBCK1	0.2488399	0.1986288	0.1390791	0.1990101	0.2443209
TBC1D20	0.2689703	0.2274713	0.2264242	0.2473425	0.1824251
CSNK2A1	0.7669022	0.8359031	1.0043419	0.7139384	0.7223978
TCF15	0.0020640	0.0114192	0.0209354	0.0094031	0.0028917
SRXN1	0.1721487	0.1635747	0.1629529	0.1508797	0.1030363
SCRT2	0.0082965	0.0192487	0.0250556	0.0106831	0.0143712
FAM110A	0.0201810	0.0623627	0.0702355	0.0097022	0.0299946
ANGPT4	0.0010050	0.0042985	0.0034547	0.0000000	0.0144049
RSPO4	0.0151934	0.0173399	0.0326687	0.0000000	0.0321638
ENSG00000286787	0.0550053	0.0461713	0.0325473	0.0304265	0.0497801
PSMF1	0.5541047	0.5894557	0.5700998	0.5465004	0.4538080
TMEM74B	0.0882567	0.1402791	0.1767609	0.0526089	0.1568205
C20orf202	0.0005216	0.0029717	0.0015452	0.0035651	0.0057345
RAD21L1	0.0013770	0.0020643	0.0015452	0.0000000	0.0000000
SNPH	0.0329611	0.0744870	0.0818268	0.0526564	0.0892354
SDCBP2	0.0520385	0.0560676	0.1346882	0.0429971	0.0435762
SDCBP2-AS1	0.0857616	0.0743783	0.0853519	0.0682064	0.1066491
ENSG00000229728	0.0038498	0.0039641	0.0036720	0.0000000	0.0000000
FKBP1A	0.6284746	0.7043935	0.8616312	0.6018833	0.4640017

NSFL1C	0.7236102	0.7091780	0.7652680	0.7059957	0.6001035
SIRPB1	0.0011912	0.0021259	0.0107068	0.0071176	0.0000000
ENSG00000286288	0.0068782	0.0044087	0.0037370	0.0000000	0.0300918
ENSG00000276649	0.0124446	0.0050349	0.0000000	0.0060757	0.0015072
SIRPA	0.0720856	0.0843685	0.1040185	0.0857483	0.0650921
PDYN-AS1	0.0000000	0.0000000	0.0027814	0.0000000	0.0000000
PDYN	0.0035128	0.0036018	0.0279918	0.0000000	0.0039284
STK35	0.0811206	0.0883794	0.0608389	0.0734062	0.0673807
ENSG00000287213	0.0000000	0.0050187	0.0000000	0.0042307	0.0000000
ENSG00000287561	0.0049308	0.0008112	0.0058277	0.0031473	0.0000000
ENSG00000226644	0.1642216	0.1027016	0.0513994	0.1381787	0.0720238
TGM3	0.0030046	0.0022996	0.0000000	0.0000000	0.0000000
SNRPB	0.5039024	0.5121765	0.6060028	0.5043775	0.4059204
ZNF343	0.0343545	0.0402342	0.0400837	0.0237896	0.0388245
TMC2	0.0057783	0.0028869	0.0089651	0.0018211	0.0016028
NOP56	0.6048019	0.5733367	0.7840326	0.6702159	0.5886220
IDH3B	0.2252122	0.2345494	0.2741723	0.2273782	0.1641253
IDH3B-DT	0.0008952	0.0000000	0.0000000	0.0000000	0.0000000
EBF4	0.0459429	0.0650559	0.0603136	0.0758542	0.1320193
CPXM1	0.0312102	0.0257977	0.0242895	0.0313220	0.0424625
PCED1A	0.1222769	0.1047393	0.1188430	0.1227304	0.1368127
VPS16	0.1248313	0.1266591	0.1831543	0.1021988	0.1446184
PTPRA	1.2577746	1.1660009	1.2288462	1.3758457	1.1328800
GNRH2	0.0000000	0.0000000	0.0000000	0.0000000	0.0000000
MRPS26	0.5668724	0.5924110	0.6130498	0.5428654	0.4400399
UBOX5-AS1	0.0022139	0.0010420	0.0000000	0.0057762	0.0069417
UBOX5	0.0414533	0.0298155	0.0208782	0.0234959	0.0619008
FASTKD5	0.0804123	0.0912161	0.0976007	0.1121298	0.0579043
LZTS3	0.1369061	0.2149486	0.2162293	0.1048927	0.2052406
ENSG00000289183	0.0075090	0.0205338	0.0077967	0.0132115	0.0087613
DDRGK1	0.2921083	0.3267040	0.3314204	0.3911144	0.3638616
ENSG00000289494	0.0206688	0.0177154	0.0035311	0.0147047	0.0183516
ITPA	0.2975986	0.3294352	0.3092231	0.2987210	0.2661454
SLC4A11	0.0060092	0.0020859	0.0000000	0.0033804	0.0025831
ENSG00000277287	0.0011952	0.0000000	0.0000000	0.0049712	0.0052853
DNAAF9	0.2370466	0.2937861	0.2971222	0.2108760	0.6957289
ENSG00000288577	0.0027084	0.0057818	0.0071378	0.0043997	0.0030832
ATRN	0.2331150	0.2279992	0.2390195	0.2746478	0.5109258
ADAM33	0.0000000	0.0010856	0.0000000	0.0121633	0.0035405
SIGLEC1	0.0000000	0.0029911	0.0000000	0.0050110	0.0050816
HSPA12B	0.0005856	0.0038607	0.0039739	0.0000000	0.0000000
ADISSP	0.3763519	0.4363442	0.4631812	0.4211499	0.3061197
SPEF1	0.2038901	0.1138009	0.0607030	0.2612012	0.1182050
CENPB	0.1566927	0.1512606	0.1480145	0.1764086	0.1821636
CDC25B	0.1033743	0.0785740	0.0660364	0.0967354	0.0779236
LINC01730	0.0035625	0.0016149	0.0000000	0.0000000	0.0000000
AP5S1	0.1230989	0.0853083	0.0659934	0.1140654	0.0745085
MAVS	0.1678970	0.1467139	0.1007691	0.1807218	0.1847571
PANK2-AS1	0.0206556	0.0147709	0.0231237	0.0163076	0.0177994
PANK2	0.2656648	0.2752512	0.3321593	0.3071424	0.4036528
ENSG00000275582	0.0000000	0.0008879	0.0000000	0.0000000	0.0000000
RNF24	0.2631648	0.3170800	0.4073924	0.2098319	0.6737464
SMOX	0.1017474	0.0685872	0.0497633	0.1048942	0.0522014

ADRA1D	0.0146642	0.0333169	0.0184948	0.0057922	0.0066786
PRNP	0.5469554	0.5527978	0.5862433	0.7519767	0.4728116
PRND	0.0014229	0.0104070	0.0047451	0.0000000	0.0038318
RASSF2	0.1585540	0.1239399	0.0935502	0.2364197	0.1243330
SLC23A2	0.1299285	0.1663106	0.2359968	0.1716163	0.4346889
ENSG00000277087	0.0009770	0.0060273	0.0053683	0.0011268	0.0026927
ENSG00000276768	0.0000000	0.0000000	0.0000000	0.0000000	0.0022770
ENSG00000278816	0.0034585	0.0030861	0.0000000	0.0000000	0.0000000
ENSG00000277425	0.0010499	0.0009954	0.0000000	0.0000000	0.0029896
TMEM230	0.9160089	0.8067433	0.7495606	0.9563009	0.6299575
PCNA	0.2376057	0.1863889	0.1932751	0.3146623	0.1450817
CDS2	0.3141547	0.3193951	0.3290511	0.3631618	0.3283428
PROKR2	0.0000000	0.0035856	0.0034571	0.0000000	0.0000000
ENSG00000266908	0.0060231	0.0050946	0.0000000	0.0045090	0.0291108
LINC00658	0.0000000	0.0014491	0.0026141	0.0000000	0.0058024
LINC00654	0.0126035	0.0317762	0.0386795	0.0131605	0.0365118
ENSG00000235820	0.0000000	0.0000000	0.0000000	0.0000000	0.0069671
GPCPD1	0.6493417	0.5778834	0.4800722	0.6807698	0.6793967
SHLD1	0.0645375	0.0423455	0.0657689	0.0320155	0.1050802
CHGB	0.7174069	0.8775676	1.7109008	0.8035086	0.9715624
TRMT6	0.1355328	0.1583496	0.1762029	0.1971205	0.1468551
MCM8	0.0197887	0.0242065	0.0332245	0.0381114	0.0246331
MCM8-AS1	0.0009973	0.0012671	0.0016900	0.0035433	0.0137662
ENSG00000275632	0.0012829	0.0037093	0.0045234	0.0000000	0.0000000
CRLS1	0.5049403	0.3518678	0.2985248	0.5537222	0.4024440
LRRN4	0.0007131	0.0022667	0.0032500	0.0000000	0.0041053
FERMT1	0.0024291	0.0058758	0.0093056	0.0000000	0.0000000
BMP2	0.0271067	0.0236442	0.0146303	0.0263419	0.0126029
HAO1	0.0000000	0.0000000	0.0042492	0.0000000	0.0000000
TMX4	0.6909561	0.7192961	0.9225971	0.6763795	0.6685400
TMX4-AS1	0.0030140	0.0022240	0.0044098	0.0000000	0.0272895
PLCB1	0.5020566	0.6468217	0.6495627	0.4099524	1.2261554
PLCB4	0.2993542	0.4559941	0.6394869	0.2174579	0.9524351
LAMP5-AS1	0.0024350	0.0091164	0.0042971	0.0000000	0.0110775
LAMP5	0.0762342	0.1316114	0.3247768	0.0292075	0.1173047
PAK5	0.2339258	0.4729881	0.6405740	0.1974390	0.7080480
ENSG00000286740	0.0000000	0.0000000	0.0000000	0.0000000	0.0027453
ENSG00000286470	0.0000000	0.0000000	0.0000000	0.0000000	0.0000000
ANKEF1	0.0858540	0.0891877	0.0681469	0.1059957	0.1275974
SNAP25-AS1	0.0194819	0.0259844	0.0214883	0.0224653	0.1337981
SNAP25	0.8180109	1.5297293	1.6007231	0.7254368	1.2424644
ENSG00000289505	0.0003640	0.0065525	0.0089421	0.0038410	0.0023258
MKKS	0.1464128	0.2038428	0.2285816	0.2063419	0.1860674
SLX4IP	0.1247426	0.1653094	0.2154232	0.1280599	0.3272975
JAG1	0.0373668	0.0367991	0.0464664	0.0768941	0.0336174
ENSG00000270792	0.0000000	0.0041293	0.0000000	0.0085265	0.0183645
ENSG00000261411	0.0027746	0.0014675	0.0057225	0.0000000	0.0016619
BTBD3	0.5305430	0.6121111	0.7453163	0.5022233	0.5883846
BTBD3-AS1	0.0010193	0.0011121	0.0000000	0.0022040	0.0093631
ENSG00000235292	0.0000000	0.0024503	0.0021512	0.0000000	0.0000000
LINC01722	0.0014569	0.0025125	0.0000000	0.0000000	0.0000000
SPTLC3	0.0015734	0.0013899	0.0000000	0.0000000	0.0050816
ISM1	0.0000000	0.0011001	0.0000000	0.0000000	0.0118000

TASP1	0.1592931	0.1184851	0.0862078	0.1443369	0.3630446
ESF1	0.6640803	0.5262710	0.6038269	0.5370102	0.5015805
NDUFAF5	0.1824632	0.2644281	0.3574428	0.1364791	0.2625534
SEL1L2	0.0048406	0.0082066	0.0029397	0.0049851	0.0126328
MACROD2	0.3534426	0.3978723	0.3728393	0.2658426	1.1536460
FLRT3	0.1869323	0.1593318	0.1682406	0.2375847	0.2148729
ENSG00000289572	0.0000000	0.0000000	0.0000000	0.0000000	0.0000000
MACROD2-AS1	0.0003615	0.0000000	0.0000000	0.0000000	0.0216507
ENSG00000286546	0.0000000	0.0000000	0.0000000	0.0000000	0.0000000
ENSG00000287410	0.0000000	0.0000000	0.0000000	0.0000000	0.0000000
KIF16B	0.1272275	0.1201943	0.1047284	0.0816468	0.2023903
ENSG00000273998	0.0002181	0.0000000	0.0000000	0.0030232	0.0069878
SNRPB2	0.9318091	0.9104002	0.9451931	0.9729050	0.6750241
OTOR	0.0013595	0.0022286	0.0000000	0.0022405	0.0000000
ENSG00000286571	0.0000000	0.0000000	0.0047686	0.0000000	0.0000000
ENSG00000287241	0.0011302	0.0026715	0.0029530	0.0033167	0.0079639
PCSK2	0.1004431	0.1767709	0.4327523	0.0746860	0.4789053
BFSP1	0.0654402	0.0508138	0.0232234	0.0402811	0.0556858
DSTN	3.0423208	2.6697787	2.5075131	3.0728013	2.3782399
RRBP1	0.2593431	0.2044056	0.2033695	0.4727935	0.1948524
SNX5	0.7378623	0.5373227	0.3635932	0.7880338	0.4452710
OVOL2	0.0022151	0.0043471	0.0000000	0.0000000	0.0222294
MGME1	0.2036205	0.1151077	0.1414194	0.2348558	0.1108809
KAT14	0.1259675	0.1572353	0.1550160	0.1387235	0.1334495
PET117	0.0955281	0.1361271	0.1161994	0.0996457	0.0649639
ZNF133	0.1032293	0.0938060	0.0826773	0.0646150	0.1470584
ENSG00000230010	0.0036824	0.0020822	0.0000000	0.0000000	0.0151810
DZANK1	0.1114114	0.1394426	0.1176674	0.1478535	0.1649720
POLR3F	0.1900189	0.1904251	0.1638080	0.1894919	0.1551001
RBBP9	0.1912803	0.1602572	0.1436373	0.1979183	0.1175585
SEC23B	0.3272621	0.2414025	0.2237965	0.3320959	0.2438766
SMIM26	0.8461262	0.7189029	0.6621914	0.8062817	0.5542749
DTD1	1.3310290	1.2646062	1.3260064	1.1853322	1.1113180
DTD1-AS1	0.0000000	0.0024767	0.0000000	0.0000000	0.0042946
LINC00652	0.0143848	0.0130240	0.0180493	0.0043650	0.0045939
LCDR	0.0196303	0.0272729	0.0140138	0.0070644	0.0191475
ENSG00000287430	0.0090582	0.0058797	0.0036656	0.0029066	0.0000000
SLC24A3	0.1751586	0.1854498	0.1850524	0.1209548	0.5292055
SLC24A3-AS1	0.0000000	0.0000000	0.0000000	0.0000000	0.0000000
ENSG00000232675	0.0000000	0.0000000	0.0000000	0.0000000	0.0000000
ENSG00000268628	0.0059083	0.0048495	0.0016977	0.0195174	0.0123659
RIN2	0.1375000	0.1099078	0.0669271	0.1269312	0.2746887
NAA20	0.5615500	0.5572199	0.5232877	0.6134476	0.3994527
CRNKL1	0.4169263	0.3576955	0.3548938	0.4358445	0.2048819
CFAP61	0.1018792	0.0555749	0.0428694	0.1052145	0.1433184
CFAP61-AS1	0.0000000	0.0000000	0.0000000	0.0000000	0.0019716
INSM1	0.0191734	0.0261636	0.0639780	0.0132026	0.0208830
RALGAPA2	0.1843201	0.1717412	0.1378780	0.1233426	0.5681252
LINC00237	0.0138991	0.0269249	0.0334592	0.0017393	0.0397019
KIZ	0.2018499	0.1858031	0.1563254	0.1618729	0.2978353
KIZ-AS1	0.0471123	0.0405035	0.0308875	0.0546214	0.0262370
ENSG00000275457	0.0079896	0.0233336	0.0097571	0.0252492	0.0098997
XRN2	0.6857372	0.6391950	0.5602288	0.7877374	0.6545246

ENSG00000225280	0.0000000	0.0000000	0.0000000	0.0068489	0.0000000
ENSG00000278041	0.0026909	0.0006977	0.0000000	0.0000000	0.0000000
NKX2-2	0.0455032	0.0360960	0.0124368	0.1006169	0.0065865
ENSG00000289590	0.0009937	0.0031651	0.0068045	0.0063737	0.0000000
LINC01727	0.0019241	0.0009988	0.0000000	0.0000000	0.0000000
ENSG00000204684	0.0040096	0.0017430	0.0000000	0.0000000	0.0040955
LINC00261	0.9222759	0.5513693	0.3294282	0.6484748	0.4982320
FOXA2	0.7700428	0.4877927	0.3010315	0.6563592	0.3078382
ENSG00000283072	0.0112633	0.0060824	0.0000000	0.0000000	0.0000000
THBD	0.0119945	0.0033919	0.0021390	0.0441591	0.0070919
CD93	0.0059617	0.0066309	0.0000000	0.0025897	0.0000000
ENSG00000230387	0.3266261	0.1694211	0.1225355	0.2404328	0.1732284
NXT1-AS1	0.0000000	0.0013171	0.0008783	0.0000000	0.0042402
NXT1	0.2149917	0.1839811	0.2005656	0.2155476	0.2144513
LINC01431	0.0188426	0.0087284	0.0132197	0.0162114	0.0116611
GZF1	0.2352754	0.1987515	0.2352858	0.2494312	0.1953897
NAPB	0.3760462	0.7884564	1.1045215	0.2855009	0.6303029
CST3	1.1016924	0.8721694	0.6597607	1.2400344	0.6718223
SYNDIG1	0.2265027	0.2233587	0.1777312	0.2068371	0.3295199
ENSG00000228539	0.0000000	0.0000000	0.0000000	0.0000000	0.0052497
APMAP	0.3829294	0.3445940	0.4593779	0.6368112	0.2963683
ACSS1	0.0794192	0.0456562	0.0159508	0.1112696	0.0373319
VSX1	0.0000000	0.0000000	0.0026308	0.0022475	0.0102958
ENSG00000230725	0.0043793	0.0191868	0.0248750	0.0061458	0.0130388
ENSG00000286472	0.0034303	0.0002840	0.0067609	0.0031240	0.0000000
ENTPD6	0.0969222	0.1540280	0.2097275	0.1090154	0.2025617
ENSG00000277938	0.0023014	0.0000000	0.0078812	0.0000000	0.0105549
ENSG00000274414	0.0010907	0.0000000	0.0000000	0.0000000	0.0000000
PYGB	0.2236918	0.3088067	0.2852505	0.2193188	0.2733592
ENSG00000276952	0.0000000	0.0000000	0.0000000	0.0000000	0.0081355
ABHD12	0.3545560	0.3465924	0.3568290	0.3617250	0.4002343
GINS1	0.0336123	0.0527715	0.0669971	0.0429646	0.0355609
NINL	0.1211487	0.1557915	0.1337525	0.0858426	0.4588945
NANP	0.0461358	0.0482092	0.0410208	0.0257728	0.0461941
ZNF337-AS1	0.0698301	0.0357842	0.0581652	0.0404201	0.0611496
ZNF337	0.1558210	0.1275159	0.1111786	0.1305337	0.2184147
ENSG00000278383	0.0000000	0.0020497	0.0000000	0.0000000	0.0000000
ENSG00000226465	0.0657953	0.1117413	0.1089253	0.0445303	0.1058144
FAM182B	0.0711407	0.0407270	0.0856094	0.0584776	0.0695721
FAM182A	0.0672799	0.0688564	0.0715874	0.0865063	0.0490953
ENSG00000231081	0.0000000	0.0000000	0.0028904	0.0026949	0.0128997
ENSG00000287569	0.0009392	0.0022532	0.0000000	0.0000000	0.0000000
MIR663AHG	0.0000000	0.0000000	0.0031196	0.0037062	0.0000000
ENSG00000291100	0.4217774	0.4374912	0.5815942	0.3854046	0.3276424
LINC01597	0.0365630	0.0388654	0.0577153	0.0201763	0.0439960
ENSG00000287288	0.0000000	0.0000000	0.0000000	0.0000000	0.0000000
FAM242A	0.0076704	0.0076777	0.0164419	0.0086489	0.0865844
ENSG00000290901	0.0392105	0.0646447	0.0408159	0.0375752	0.0669802
HM13	0.6257464	0.4844890	0.4253700	0.7470922	0.4583697
HM13-IT1	0.0006636	0.0000000	0.0000000	0.0000000	0.0000000
HM13-AS1	0.0055022	0.0000000	0.0000000	0.0040247	0.0000000
ENSG00000278276	0.0000000	0.0000000	0.0000000	0.0000000	0.0000000
ID1	0.1931071	0.1015788	0.0835942	0.2363030	0.1158393

BCL2L1	0.1842971	0.2523036	0.3356228	0.1420043	0.2143351
BCL2L1-AS1	0.0000000	0.0014838	0.0000000	0.0000000	0.0105129
ABALON	0.0033699	0.0019665	0.0024318	0.0000000	0.0000000
ENSG00000289492	0.0005911	0.0007309	0.0000000	0.0050414	0.0000000
TPX2	0.0781844	0.0702324	0.0325736	0.0976322	0.0423491
MYLK2	0.0007888	0.0000000	0.0000000	0.0000000	0.0000000
FOXS1	0.0018775	0.0048158	0.0016391	0.0051740	0.0275800
DUSP15	0.3080475	0.2683318	0.2371778	0.3371004	0.1898104
TTLL9	0.0846780	0.0747348	0.0679776	0.0636253	0.1457962
PDRG1	0.4460537	0.3592709	0.3006361	0.4312744	0.2877809
XKR7	0.0191080	0.0378960	0.0692152	0.0158249	0.1019446
ENSG00000278012	0.0021070	0.0139963	0.0274183	0.0032416	0.0015072
ENSG00000226239	0.0051239	0.0247570	0.0117216	0.0070535	0.0229078
CCM2L	0.0010624	0.0005109	0.0123467	0.0000000	0.0183703
HCK	0.0047498	0.0014491	0.0032453	0.0000000	0.0000000
TM9SF4	0.2638205	0.2191999	0.1971786	0.4077283	0.3720204
ENSG00000275576	0.0000000	0.0000000	0.0000000	0.0000000	0.0000000
PLAGL2	0.0895209	0.0751806	0.0734449	0.0934383	0.0496119
POFUT1	0.0628382	0.0514960	0.0374577	0.1142194	0.0642792
ENSG00000287850	0.0000000	0.0000000	0.0000000	0.0000000	0.0000000
KIF3B	0.5368214	0.6882036	0.6978629	0.3705850	0.5692687
ENSG00000277692	0.0058536	0.0054871	0.0008833	0.0055256	0.0000000
ASXL1	0.3118435	0.2989426	0.3144984	0.2736507	0.4788698
NOL4L	0.2419201	0.2795236	0.3500236	0.1853247	0.3497192
ENSG00000236772	0.0000000	0.0000000	0.0000000	0.0000000	0.0000000
ENSG00000286311	0.0050456	0.0078218	0.0000000	0.0041781	0.0220368
ENSG00000277301	0.0005545	0.0000000	0.0018800	0.0065074	0.0021637
ENSG00000233293	0.0047198	0.0098431	0.0137886	0.0035629	0.0000000
NOL4L-DT	0.0125343	0.0142640	0.0378928	0.0108964	0.0049230
C20orf203	0.0137192	0.0167093	0.0205652	0.0085879	0.1217871
COMMD7	0.4243078	0.5185466	0.5237731	0.4208317	0.4612900
DNMT3B	0.0097566	0.0164364	0.0147838	0.0112416	0.0792377
MAPRE1	0.7140379	0.8572807	0.9057222	0.6560758	0.6881376
ENSG00000260536	0.0240500	0.0467945	0.0637526	0.0187339	0.0721653
ENSG00000260257	0.0916210	0.1580366	0.1458736	0.0518413	0.1497996
EFCAB8	0.0000000	0.0005546	0.0000000	0.0000000	0.0000000
BPIFB2	0.0076780	0.0069404	0.0068956	0.0119343	0.0000000
BPIFA2	0.0000000	0.0000000	0.0000000	0.0000000	0.0000000
BPIFB1	0.0000000	0.0013272	0.0000000	0.0000000	0.0000000
CDK5RAP1	0.0871756	0.0830467	0.0962627	0.0944267	0.1011605
SNTA1	0.1765413	0.1072046	0.1121331	0.2058864	0.1182926
CBFA2T2	0.2228940	0.2517678	0.2125630	0.1818135	0.5698668
NECAB3	0.1380712	0.1038565	0.1104510	0.1001166	0.0577570
C20orf144	0.0011240	0.0000000	0.0000000	0.0028807	0.0053413
ACTL10	0.0036427	0.0010932	0.0000000	0.0018489	0.0000000
E2F1	0.0293919	0.0147341	0.0058743	0.0159650	0.0119033
PXMP4	0.0656488	0.0474045	0.0261511	0.0791477	0.0539123
ZNF341	0.0028719	0.0099415	0.0101214	0.0063488	0.0000000
ZNF341-AS1	0.0000000	0.0000000	0.0000000	0.0000000	0.0000000
CHMP4B	0.4593175	0.4588488	0.5140601	0.4512605	0.3306642
RALY-AS1	0.0225323	0.0140771	0.0093084	0.0204363	0.0069538
RALY	0.4616802	0.4097590	0.3646774	0.4332956	0.3975345
ENSG00000228386	0.0000000	0.0000000	0.0000000	0.0000000	0.0058321

EIF2S2	1.6146640	1.4993760	1.5263086	1.5361015	1.3155769
ENSG00000287853	0.0051739	0.0082985	0.0087441	0.0211146	0.0000000
ASIP	0.0154284	0.0113930	0.0130113	0.0181434	0.0173666
ENSG00000250917	0.0479141	0.0539886	0.0371699	0.0256158	0.0630552
AHCY	0.3800839	0.3102582	0.2477459	0.3763367	0.2517284
ITCH	0.3063306	0.2961446	0.2517174	0.2948549	0.4452372
ITCH-AS1	0.0000000	0.0000000	0.0000000	0.0000000	0.0000000
DYNLRB1	1.3676340	1.5249703	1.5809882	1.3265385	1.2582331
MAP1LC3A	0.9233464	1.2809448	1.4489158	0.8428552	0.9948489
PIGU	0.1705799	0.1402995	0.1621590	0.2783277	0.2509847
NCOA6	0.2586043	0.2597439	0.2690792	0.2191805	0.4288179
TP53INP2	0.0691371	0.1020105	0.1363010	0.0772178	0.1110205
ENSG00000286803	0.0024071	0.0013292	0.0000000	0.0038691	0.0150764
GGT7	0.1220861	0.1237658	0.1698253	0.1665845	0.2717211
ACSS2	0.0572358	0.0436328	0.0462233	0.0583499	0.0389466
GSS	0.2211519	0.1905739	0.1387864	0.3310427	0.1852961
MYH7B	0.0062035	0.0018558	0.0095094	0.0000000	0.0182158
TRPC4AP	0.2813524	0.2979554	0.2468077	0.3213436	0.4200183
EDEM2	0.0976019	0.0921230	0.1081789	0.2117549	0.0695460
PROCR	0.0558562	0.0364874	0.0196360	0.2851986	0.0217629
MMP24OS	0.4217562	0.4636361	0.4548595	0.4736443	0.3560613
MMP24	0.1274616	0.2215668	0.2523664	0.0923665	0.2101723
EIF6	0.4478977	0.4490544	0.4485201	0.4658091	0.3122217
FAM83C	0.0000000	0.0042874	0.0000000	0.0000000	0.0000000
UQCC1	0.1309131	0.1477642	0.1517563	0.1600018	0.2372716
GDF5	0.0059906	0.0096598	0.0065415	0.0000000	0.0206938
CEP250	0.1895261	0.2104741	0.2383093	0.1496756	0.3405274
CEP250-AS1	0.0007959	0.0017953	0.0029736	0.0000000	0.0018081
ERGIC3	0.9697351	0.8701547	0.7572767	1.0712508	0.7487739
SPAG4	0.0568996	0.0393063	0.0576002	0.0438049	0.0673814
CPNE1	0.4028865	0.3390137	0.3488385	0.3736746	0.3449212
RBM12	0.2321636	0.2613361	0.2567478	0.1831816	0.2746009
NFS1	0.1178089	0.0927206	0.0835167	0.1024599	0.0716259
ROMO1	0.9227767	0.8680262	0.8427065	0.8720500	0.6516604
RBM39	1.1795244	1.0626739	1.0148200	1.1887394	1.0770462
ENSG00000289872	0.0116317	0.0090361	0.0069748	0.0068505	0.0199312
PHF20	0.3654092	0.4477767	0.4387734	0.3831549	0.5038623
SCAND1	0.7436413	0.7049740	0.8042044	0.6899336	0.5255086
CNBD2	0.0386704	0.0291220	0.0331103	0.0473331	0.0164239
NORAD	1.4848397	1.3420902	1.3924713	1.4950186	1.3036591
ENSG00000234139	0.0004290	0.0003109	0.0000000	0.0000000	0.0077809
ENSG00000277235	0.0035261	0.0038607	0.0000000	0.0038740	0.0000000
EPB41L1	0.4396124	0.7591232	0.8916612	0.3823192	0.8256970
EPB41L1-AS1	0.0000000	0.0000000	0.0000000	0.0000000	0.0000000
ENSG00000278035	0.0006752	0.0000000	0.0000000	0.0000000	0.0067831
AAR2	0.1117494	0.1178107	0.1171046	0.1267884	0.1271685
DLGAP4	0.5867203	0.6530594	0.6564319	0.5177835	0.8313246
DLGAP4-AS1	0.0043313	0.0039246	0.0018938	0.0019116	0.0117416
MYL9	0.0327566	0.0256434	0.0159463	0.0464065	0.0361400
TGIF2	0.1923882	0.0949810	0.0550505	0.1863143	0.0863793
RAB5IF	0.5471855	0.5011459	0.5131415	0.5888120	0.3951354
SLA2	0.0011401	0.0026682	0.0000000	0.0022792	0.0061235
NDRG3	0.2859362	0.4232778	0.4504710	0.3833736	0.2645173

ENSG00000289373	0.0000000	0.0000000	0.0000000	0.0000000	0.0000000
DSN1	0.0207271	0.0258508	0.0307608	0.0391758	0.0219307
SOGA1	0.2011611	0.2863476	0.3144593	0.2263666	0.3902836
TLDC2	0.0000000	0.0000000	0.0017912	0.0000000	0.0000000
SAMHD1	0.0751859	0.1109031	0.2037592	0.0866675	0.1299760
RBL1	0.0380806	0.0537805	0.0331195	0.0317426	0.1525441
MROH8	0.0464543	0.0440767	0.0459985	0.0286380	0.0956554
RPN2	0.8916689	0.5911058	0.5766366	1.2672291	0.6161490
GHRH	0.0023289	0.0000000	0.0038150	0.0000000	0.0000000
MANBAL	0.2546108	0.2180411	0.2856634	0.2455989	0.1844403
ENSG00000286240	0.0011193	0.0008957	0.0024513	0.0026231	0.0000000
SRC	0.0648809	0.0959702	0.0903518	0.0545939	0.1131484
BLCAP	0.5885132	0.8009017	1.1982579	0.5705330	0.6937942
NNAT	1.2464857	1.6965509	2.2136368	1.1900704	1.5044447
ENSG00000276603	0.0061279	0.0070639	0.0108435	0.0056018	0.0174672
CTNNBL1	0.2490275	0.2320729	0.2111020	0.2292511	0.2814102
VSTM2L	0.8599267	0.8018476	0.7655306	0.8906615	0.6156263
TTI1	0.1110906	0.1146561	0.1233806	0.0912160	0.1349208
RPRD1B	0.2573212	0.2128724	0.1786360	0.2122798	0.2683118
TGM2	0.0035441	0.0048801	0.0026494	0.0000000	0.0000000
KIAA1755	0.0000000	0.0012286	0.0000000	0.0000000	0.0000000
LBP	0.0000000	0.0031851	0.0000000	0.0000000	0.0000000
ENSG00000224635	0.0020234	0.0018081	0.0019328	0.0000000	0.0015072
SNHG17	0.0935985	0.0938071	0.1197136	0.0929079	0.1116193
SNHG11	0.1147282	0.1414448	0.1887448	0.1120598	0.1160942
RALGAPB	0.1856008	0.2147386	0.2131292	0.1848335	0.3494276
ARHGAP40	0.0014343	0.0000000	0.0000000	0.0000000	0.0000000
SLC32A1	0.0080360	0.0204003	0.0433521	0.0120408	0.0113934
ACTR5	0.0329517	0.0312867	0.0231422	0.0275102	0.0383120
PPP1R16B	0.0317768	0.0714936	0.0773613	0.0319877	0.1108232
FAM83D	0.0065662	0.0034883	0.0006255	0.0030891	0.0348394
DHX35-DT	0.0013433	0.0021893	0.0019906	0.0000000	0.0000000
DHX35	0.0505457	0.0437545	0.0356011	0.0328778	0.0849060
ENSG00000277581	0.0000000	0.0000000	0.0000000	0.0000000	0.0000000
ENSG00000229976	0.0000000	0.0000000	0.0000000	0.0000000	0.0027533
MAFB	0.3888574	1.1544897	0.3789155	0.4180369	0.5939992
ENSG00000286972	0.0008066	0.0070164	0.0041979	0.0035761	0.0019839
TOP1	0.9029205	0.7798914	0.8353584	0.8984507	0.7846225
PLCG1-AS1	0.0104997	0.0129143	0.0279037	0.0077022	0.0408201
PLCG1	0.0821255	0.1029575	0.0831602	0.0874455	0.2064848
ZHX3	0.1999554	0.2102200	0.2189257	0.2561148	0.2991901
LPIN3	0.0076456	0.0038983	0.0031535	0.0042777	0.0077821
EMILIN3	0.0007932	0.0022201	0.0018732	0.0069142	0.0123463
CHD6	0.4941826	0.4868338	0.5187401	0.4641712	0.7470004
ENSG00000273951	0.0000000	0.0000000	0.0021716	0.0000000	0.0000000
ENSG00000288843	0.0000000	0.0000000	0.0000000	0.0000000	0.0053148
ENSG00000225458	0.0012354	0.0000000	0.0000000	0.0041394	0.0000000
PTPRT	0.4969133	0.5027444	0.5356896	0.4603873	1.3313583
PTPRT-DT	0.0706076	0.1434431	0.1965994	0.0805433	0.1206286
SRSF6	0.3443222	0.3156895	0.2072947	0.3496905	0.3285138
L3MBTL1	0.0971198	0.0756103	0.0770093	0.0535190	0.2970473
SGK2	0.0036050	0.0091557	0.0101435	0.0113525	0.0173754
ENSG00000282876	0.0017381	0.0021427	0.0000000	0.0069515	0.0067298

IFT52	0.3931500	0.3662383	0.3879325	0.4094353	0.3556494
MYBL2	0.0080510	0.0115678	0.0062743	0.0309077	0.0174035
TOX2	0.2452989	0.2167699	0.2953134	0.2709829	0.2377347
JPH2	0.0053278	0.0061773	0.0025702	0.0152715	0.0013692
OSER1	0.3471902	0.2791654	0.2528376	0.3082800	0.2292109
OSER1-DT	0.1808953	0.1866541	0.1434638	0.1409732	0.1109789
GDAP1L1	0.4560217	0.8489915	0.8520665	0.4387103	0.5910212
FITM2	0.0588191	0.0674519	0.0962070	0.0691146	0.0693413
R3HDML	0.0023908	0.0000000	0.0000000	0.0000000	0.0000000
HNF4A	0.0004642	0.0014633	0.0000000	0.0025162	0.0000000
LINC01620	0.0000000	0.0000000	0.0000000	0.0000000	0.0015072
TTPAL	0.0888325	0.1210438	0.0843082	0.1147236	0.1004110
SERINC3	0.6859350	0.6122283	0.6141944	0.6707555	0.6422845
PKIG	0.5856644	0.5083453	0.4321908	0.6055156	0.4461256
ADA	0.0162547	0.0202434	0.0039727	0.0129191	0.0400474
LINC01260	0.0385468	0.0637633	0.0857691	0.0308337	0.0391378
KCNK15-AS1	0.0236398	0.0370796	0.0309885	0.0190577	0.0814557
CCN5	0.0013315	0.0011535	0.0026532	0.0000000	0.0071168
KCNK15	0.0011399	0.0023190	0.0027374	0.0000000	0.0000000
RIMS4	0.0696106	0.1531272	0.2289729	0.0386821	0.1379134
ENSG00000286100	0.0024914	0.0009013	0.0050217	0.0000000	0.0258631
YWHAB	1.5622140	1.8323189	2.1380301	1.6497903	1.4510945
PABPC1L	0.0219295	0.0167299	0.0213806	0.0171602	0.0628038
TOMM34	0.2395065	0.2007071	0.1984380	0.2132221	0.2373869
STK4-DT	0.0012318	0.0012926	0.0020756	0.0030907	0.0000000
STK4	0.2528061	0.2239101	0.2165032	0.2103153	0.2000334
KCNS1	0.0014937	0.0031124	0.0065075	0.0000000	0.0000000
MATN4	0.0033398	0.0033506	0.0015972	0.0000000	0.0000000
SDC4	0.0362013	0.0235949	0.0058668	0.0595912	0.0123291
SYS1	0.3114412	0.2151633	0.1680844	0.3531380	0.2216259
TP53TG5	0.0026879	0.0145675	0.0009238	0.0052579	0.0135202
DBNDD2	0.3329459	0.3317754	0.2928167	0.2629327	0.2263405
PIGT	0.4713364	0.2976423	0.2657889	0.5456641	0.3297973
ENSG00000277022	0.0005499	0.0000000	0.0000000	0.0000000	0.0000000
WFDC2	0.1359893	0.2565836	0.5396269	0.1524460	0.1490728
WFDC11	0.0000000	0.0000000	0.0000000	0.0000000	0.0000000
WFDC10B	0.0112324	0.0101070	0.0055761	0.0131317	0.0089058
WFDC3	0.0067410	0.0027975	0.0090233	0.0077368	0.0179324
DNTTIP1	0.2585294	0.1950927	0.1817767	0.2734030	0.2011057
UBE2C	0.0323814	0.0448361	0.0187710	0.0525345	0.0521492
TNNC2	0.0007250	0.0032341	0.0040286	0.0077133	0.0104570
SNX21	0.0997413	0.0918144	0.0783460	0.0721019	0.0504337
ACOT8	0.1900270	0.2130204	0.2490367	0.1864104	0.1598789
ZSWIM3	0.0432243	0.0485943	0.0530227	0.0332708	0.0768544
ZSWIM1	0.0308236	0.0425988	0.0422653	0.0369746	0.0128202
NEURL2	0.0219438	0.0133227	0.0177133	0.0340522	0.0261674
CTSA	0.1071461	0.0661322	0.0742345	0.1592777	0.0715241
PLTP	1.4559292	0.9426759	0.6189830	1.9056475	0.8995730
ENSG00000285796	0.0071094	0.0057261	0.0051821	0.0000000	0.0131276
PCIF1	0.1629573	0.1438168	0.1314275	0.1658287	0.1114301
ZNF335	0.0380988	0.0409813	0.0291398	0.0320685	0.1262501
MMP9	0.0025521	0.0017244	0.0000000	0.0036297	0.0000000
SLC12A5-AS1	0.0038087	0.0108245	0.0076872	0.0048478	0.0205995

SLC12A5	0.1473150	0.2870708	0.3785422	0.1463017	0.2702325
NCOA5	0.1337397	0.1290950	0.1706378	0.1212705	0.2174507
CD40	0.0005556	0.0101292	0.0075636	0.0042330	0.0000000
CDH22	0.0380173	0.0877688	0.0571160	0.0289340	0.2430482
SLC35C2	0.2659748	0.2319332	0.1851345	0.2773946	0.1911984
ENSG00000273828	0.0002752	0.0051151	0.0018292	0.0000000	0.0118069
ELMO2	0.1096592	0.1083561	0.1142918	0.1291824	0.1639526
ZNF334	0.1728186	0.1539492	0.1193121	0.2227077	0.2264100
SLC13A3	0.0328407	0.0331530	0.0392376	0.0412786	0.1258839
ENSG00000273893	0.0019011	0.0000000	0.0000000	0.0034102	0.0000000
TP53RK	0.2107614	0.2113367	0.2514173	0.2225686	0.1468115
TP53RK-DT	0.0045550	0.0028020	0.0006144	0.0000000	0.0000000
SLC2A10	0.0578967	0.0315840	0.0239656	0.0697627	0.0269520
EYA2	0.0279978	0.0218526	0.0078022	0.0288643	0.0346908
ZMYND8	0.4129370	0.4576907	0.5551604	0.3956444	0.6932359
ENSG00000273451	0.0003547	0.0000000	0.0000000	0.0000000	0.0033534
ENSG00000231119	0.0000000	0.0020093	0.0000000	0.0010887	0.0089211
NCOA3	0.3121639	0.3257450	0.3043801	0.2871755	0.5432581
SULF2	0.9381247	0.6658571	0.6165947	1.4064139	0.8409845
ENSG00000255438	0.0050293	0.0000000	0.0031350	0.0121579	0.0000000
LINC01522	0.0049734	0.0120601	0.0047156	0.0034162	0.0037844
ENSG00000287335	0.0000000	0.0000000	0.0000000	0.0000000	0.0068372
ENSG00000286063	0.0000000	0.0000000	0.0055050	0.0000000	0.0031466
PREX1	0.4198404	0.2737584	0.1731435	0.4563135	0.2218882
ENSG00000278231	0.0034468	0.0000000	0.0000000	0.0000000	0.0000000
ARFGEF2	0.1857355	0.2284625	0.2451989	0.1715333	0.3400229
CSE1L-DT	0.0000000	0.0014613	0.0041405	0.0000000	0.0000000
CSE1L	0.3773115	0.3560680	0.4073603	0.3507661	0.3633040
STAU1	1.1116875	1.0678853	1.1830026	1.0448155	1.0262877
DDX27	0.3019961	0.2820416	0.2106734	0.2822056	0.2822300
ZNFX1	0.1047064	0.0743708	0.0943607	0.1077313	0.1278921
ZFAS1	1.6276291	1.6845056	1.5459634	1.4433102	1.5325278
KCNB1	0.0876271	0.1791044	0.2419584	0.0858791	0.3289739
ENSG00000290421	0.0034901	0.0028323	0.0096040	0.0029390	0.0113888
PTGIS	0.0528194	0.1038968	0.1178480	0.0730899	0.0923847
B4GALT5	0.2268123	0.3081547	0.3733216	0.2776177	0.3368437
SLC9A8	0.0774048	0.0554985	0.0643697	0.1085043	0.1189060
SPATA2	0.0504916	0.0538410	0.0550502	0.0361739	0.0662766
RNF114	0.3524910	0.3219314	0.3291667	0.3287603	0.3002457
SNAI1	0.0060661	0.0048548	0.0067293	0.0054382	0.0204202
UBE2V1	1.0989645	1.1349426	1.1603436	1.1359578	0.8765708
PEDS1	0.1982068	0.1973841	0.1710587	0.2657385	0.2308991
LINC01275	0.0021800	0.0014253	0.0000000	0.0025371	0.0000000
LINC01273	0.0125863	0.0081356	0.0060771	0.0119331	0.0000000
CEBPB-AS1	0.0138741	0.0136040	0.0126109	0.0189921	0.0027686
CEBPB	0.5423144	0.3858204	0.3256162	0.4708689	0.2726415
LINC01270	0.0009717	0.0017787	0.0000000	0.0017080	0.0000000
PTPN1	0.2937794	0.3503995	0.3329584	0.2848161	0.3480413
ENSG00000232043	0.0000000	0.0000000	0.0000000	0.0000000	0.0000000
RIPOR3	0.0039755	0.0047296	0.0000000	0.0108160	0.0069624
RIPOR3-AS1	0.0000000	0.0000000	0.0016166	0.0027814	0.0048214
PARD6B	0.3514002	0.2743140	0.1970239	0.4284414	0.2625773
BCAS4	0.3047706	0.3171017	0.3649113	0.2885392	0.2198945

ADNP	0.5809337	0.5458140	0.6279068	0.5190050	0.7717984
ADNP-AS1	0.0235789	0.0102577	0.0206068	0.0091684	0.0209257
DPM1	0.3172490	0.2631859	0.2544509	0.3489943	0.2937981
MOCS3	0.0585062	0.0511002	0.0505407	0.0805712	0.0350268
KCNG1	0.3310986	0.1585308	0.1006797	0.3566201	0.1875145
NFATC2	0.0068993	0.0115950	0.0051608	0.0054153	0.0644479
ATP9A	0.5143521	0.7434552	0.8992087	0.4513718	0.9706215
SALL4	0.0082467	0.0066936	0.0042246	0.0000000	0.0254775
LINC01429	0.0033408	0.0000000	0.0000000	0.0000000	0.0016917
ZFP64	0.1253252	0.1334879	0.1255960	0.1637170	0.1356283
ENSG00000287511	0.0022907	0.0093515	0.0086542	0.0000000	0.0037187
LINC01524	0.0027321	0.0054890	0.0000000	0.0000000	0.0326780
TSHZ2	0.7402614	0.8475704	1.1595706	0.5680148	1.4382148
ENSG00000286865	0.0000000	0.0000000	0.0000000	0.0000000	0.0000000
ENSG00000223492	0.0005853	0.0000000	0.0000000	0.0000000	0.0028353
ENSG00000227705	0.0006999	0.0000000	0.0000000	0.0000000	0.0057548
ENSG00000271774	0.0169445	0.0192103	0.0093635	0.0051450	0.2125236
ENSG00000231703	0.0079620	0.0047234	0.0117633	0.0037983	0.0000000
ENSG00000259723	0.0000000	0.0012174	0.0000000	0.0000000	0.0047271
ENSG00000197670	0.0015779	0.0000000	0.0000000	0.0000000	0.0105600
ZNF217	0.2507035	0.1325100	0.0717825	0.3119689	0.1060487
ENSG00000235415	0.0000000	0.0000000	0.0000000	0.0000000	0.0000000
ENSG00000287886	0.0000000	0.0000000	0.0011369	0.0000000	0.0000000
BCAS1	0.0940358	0.0439066	0.0241951	0.0310487	0.0247882
ENSG00000236352	0.0029283	0.0020377	0.0009265	0.0048428	0.0031863
CYP24A1	0.0000000	0.0000000	0.0019963	0.0000000	0.0000000
PFDN4	0.7999981	0.9356353	1.0326551	0.7286705	0.6784066
ENSG00000286229	0.0016462	0.0019419	0.0006832	0.0000000	0.0000000
DOK5	0.5722423	0.4537480	0.4058445	0.7233612	0.4981674
LINC01440	0.0000000	0.0008556	0.0058383	0.0027827	0.0020838
ENSG00000287478	0.0000000	0.0000000	0.0023930	0.0000000	0.0000000
CBLN4	0.1072428	0.2216568	0.5760822	0.0755979	0.1903182
FAM210B	0.3953640	0.2827035	0.2186591	0.4328137	0.1857399
AURKA	0.0314451	0.0201815	0.0146669	0.0076169	0.0316766
CSTF1	0.1502438	0.1316460	0.1335564	0.1631942	0.1045521
CASS4	0.0025238	0.0037777	0.0037348	0.0000000	0.0082196
RTF2	0.7947432	0.7064109	0.7733541	0.8087374	0.5842748
GCNT7	0.0000000	0.0000000	0.0000000	0.0043147	0.0000000
FAM209A	0.0000000	0.0000000	0.0022595	0.0000000	0.0000000
FAM209B	0.0025532	0.0024845	0.0011178	0.0022040	0.0000000
ENSG00000287079	0.0000000	0.0000000	0.0000000	0.0000000	0.0000000
ENSG00000288946	0.0000000	0.0005333	0.0000000	0.0029390	0.0064463
BMP7	0.4551762	0.3090333	0.1983149	0.6077336	0.2403060
ENSG00000226308	0.0056036	0.0075772	0.0084603	0.0122452	0.0030127
RAE1	0.2726599	0.2499831	0.3068530	0.2885109	0.1683549
RBM38-AS1	0.0000000	0.0000000	0.0012831	0.0000000	0.0000000
RBM38	0.1471089	0.1083197	0.0700363	0.1713666	0.1260959
HMGB1P1	0.0179273	0.0066830	0.0134348	0.0255524	0.0000000
CTCFL	0.0011837	0.0019183	0.0000000	0.0000000	0.0000000
PMEPA1	0.3794684	0.3624046	0.3913099	0.3743410	0.3996909
NKILA	0.0410223	0.0582159	0.0621966	0.0261052	0.0434287
C20orf85	0.0022365	0.0011745	0.0000000	0.0000000	0.0000000
ENSG00000291067	0.0198491	0.0126538	0.0075757	0.0179498	0.0792178

RAB22A	0.5716860	0.7426457	0.7877354	0.6340293	0.5613192
VAPB	0.4447626	0.5720865	0.6136313	0.4137471	0.4056602
APCDD1L	0.0000000	0.0000000	0.0000000	0.0000000	0.0000000
APCDD1L-DT	0.0038169	0.0033828	0.0013009	0.0098499	0.0149449
LINC01711	0.0020453	0.0000000	0.0000000	0.0000000	0.0000000
STX16	0.3178970	0.2861632	0.2724891	0.2955090	0.4287469
NPEPL1	0.0783575	0.0369218	0.0504963	0.0990562	0.1097271
GNAS-AS1	0.0189489	0.0104899	0.0016105	0.0167220	0.0017703
GNAS	2.0639399	2.3048890	2.5151856	2.0033961	2.0924255
ENSG00000225806	0.0123392	0.0060219	0.0075081	0.0198955	0.0122105
ENSG00000285091	0.0028995	0.0023058	0.0024659	0.0000000	0.0125808
NELFCD	0.3685458	0.2637406	0.2643479	0.3722313	0.2904497
CTSZ	0.2801342	0.1649529	0.1101577	0.3343056	0.1705041
TUBB1	0.0000000	0.0000000	0.0020012	0.0000000	0.0000000
ATP5F1E	2.2721037	2.3981702	2.4122014	2.2726445	2.0413434
ENSG00000289837	0.0024759	0.0019921	0.0000000	0.0000000	0.0078339
PRELID3B	0.3569444	0.3381789	0.3846307	0.3802041	0.2796787
ZNF831	0.0000000	0.0000000	0.0000000	0.0000000	0.0000000
EDN3	0.0012152	0.0008999	0.0019622	0.0000000	0.0056180
PHACTR3	0.2569381	0.5289049	0.5993900	0.2413974	0.8174832
SYCP2	0.0100835	0.0097081	0.0041517	0.0045766	0.0450185
FAM217B	0.3288173	0.2627469	0.3024258	0.2864086	0.2969597
PPP1R3D	0.0594356	0.0877800	0.1018169	0.0566623	0.0496003
CDH26	0.0035460	0.0083538	0.0058015	0.0000000	0.0150784
MIR646HG	0.0578517	0.0778810	0.0969247	0.0784580	0.2019119
ENSG00000276627	0.0008634	0.0045202	0.0027173	0.0000000	0.0033679
CDH4	0.1722002	0.2122411	0.2679729	0.1701748	1.3005017
ENSG00000277270	0.0000000	0.0004904	0.0000000	0.0000000	0.0373331
ENSG00000179253	0.0000000	0.0012907	0.0021258	0.0000000	0.0000000
ENSG00000225106	0.0000000	0.0035245	0.0000000	0.0000000	0.0000000
TAF4	0.0612045	0.0498671	0.0455570	0.0163755	0.2187833
ENSG00000283078	0.0056341	0.0072275	0.0102842	0.0018861	0.0066003
ENSG00000289236	0.0020549	0.0046008	0.0055927	0.0000000	0.0090153
LSM14B	0.3007777	0.3814159	0.4506171	0.2959598	0.3014717
PSMA7	1.6716257	1.7008408	1.8573221	1.5975681	1.4476012
SS18L1	0.1392067	0.2342173	0.2718209	0.1047686	0.2722521
ENSG00000289537	0.0209868	0.0128843	0.0110555	0.0283412	0.0021637
MTG2	0.0967509	0.0727487	0.0652969	0.0864277	0.0750007
HRH3	0.0302420	0.0835437	0.0880132	0.0345974	0.0743734
OSBPL2	0.3416932	0.3085281	0.3694061	0.4300601	0.3312465
ADRM1	0.4484437	0.4496544	0.5284067	0.4480346	0.3246185
ENSG00000226332	0.0012472	0.0012005	0.0000000	0.0000000	0.0000000
LAMA5	0.0762810	0.0575938	0.0464483	0.1173769	0.1252576
LAMA5-AS1	0.0165156	0.0132174	0.0117302	0.0075168	0.0000000
ENSG00000273619	0.0248722	0.0335381	0.0146365	0.0240588	0.0207484
RPS21	1.9955059	2.0433181	1.8961749	1.8981157	1.8031768
CABLES2	0.0592686	0.1208617	0.1723767	0.0357260	0.1307444
ENSG00000275437	0.0020670	0.0045837	0.0007533	0.0000000	0.0000000
CRMA	0.0105409	0.0217708	0.0074525	0.0022792	0.0109325
ENSG00000167046	0.0000000	0.0000000	0.0000000	0.0000000	0.0000000
SLCO4A1	0.0098253	0.0140147	0.0196143	0.0025494	0.0467080
NTSR1	0.0041752	0.0038728	0.0031650	0.0000000	0.0145089
MRGBP	0.2421495	0.2302977	0.1778657	0.2124750	0.1601596

OGFR-AS1	0.0082352	0.0089764	0.0016430	0.0025805	0.0036000
OGFR	0.1391813	0.1145628	0.1171061	0.1206764	0.1127755
COL9A3	0.0068379	0.0030538	0.0000000	0.0261681	0.0091224
TCFL5	0.2498593	0.2265697	0.1832084	0.2783630	0.1731621
DIDO1	0.2596372	0.2713642	0.2535941	0.2667371	0.4156621
ENSG00000273759	0.0054870	0.0022160	0.0022370	0.0033765	0.0013244
GID8	0.4593201	0.4317248	0.4701431	0.4377385	0.4221836
SLC17A9	0.0024841	0.0013201	0.0000000	0.0000000	0.0000000
LINC01749	0.0000000	0.0000000	0.0000000	0.0000000	0.0000000
HAR1B	0.0007110	0.0053022	0.0046754	0.0000000	0.0000000
HAR1A	0.0167340	0.0201493	0.0316239	0.0048798	0.0016619
ENSG00000231977	0.0011708	0.0012391	0.0000000	0.0000000	0.0059661
YTHDF1	0.2287445	0.2156324	0.2229489	0.2384080	0.2304296
ENSG00000273821	0.0038367	0.0014045	0.0081958	0.0000000	0.0000000
NKAIN4	0.6075970	0.3161330	0.2201484	1.0629057	0.3181586
FLJ16779	0.0385812	0.0266738	0.0293723	0.0185550	0.0203293
ARFGAP1	0.1208685	0.1067692	0.0998916	0.1359769	0.1675611
COL20A1	0.0009542	0.0027638	0.0064278	0.0000000	0.0183832
CHRNA4	0.0574148	0.1233900	0.1901889	0.0521950	0.1222078
ENSG00000203900	0.0010890	0.0024918	0.0000000	0.0000000	0.0000000
KCNQ2	0.1989758	0.2934911	0.4161185	0.2047777	0.4218391
EEF1A2	0.8864644	1.2536746	1.2252805	0.8552169	0.9639485
ENSG00000230226	0.0005919	0.0000000	0.0000000	0.0000000	0.0000000
PPDPF	2.6938567	2.6042934	2.4239137	2.6084433	2.3794263
PTK6	0.0023153	0.0000000	0.0000000	0.0027265	0.0093927
ENSG00000275812	0.0022425	0.0026140	0.0000000	0.0000000	0.0000000
FNDC11	0.0403175	0.0472901	0.0318563	0.0338278	0.0286758
HELZ2	0.0149746	0.0155431	0.0000000	0.0073285	0.0125845
GMEB2	0.0497622	0.0550077	0.0416560	0.0412273	0.0492354
MHENCR	0.1131041	0.1278758	0.1213966	0.1587227	0.0806440
STMN3	0.3784287	0.6503637	0.8352872	0.3757133	0.5348997
RTEL1	0.0176368	0.0101844	0.0181913	0.0104861	0.0863926
TNFRSF6B	0.0102610	0.0174858	0.0099804	0.0080827	0.0073750
ARFRP1	0.2229204	0.1786406	0.1778258	0.2462963	0.1884219
ZGPAT	0.0509366	0.0379967	0.0300085	0.0307642	0.0278123
LIME1	0.0081357	0.0042467	0.0092903	0.0037517	0.0445143
SLC2A4RG	0.4871981	0.3779772	0.3204840	0.4866656	0.2789427
ZBTB46	0.0984903	0.1584682	0.1436447	0.0938760	0.1960908
ZBTB46-AS2	0.0059092	0.0015056	0.0027238	0.0000000	0.0102372
ZBTB46-AS1	0.0000000	0.0000000	0.0000000	0.0000000	0.0000000
ENSG00000268858	0.0356548	0.0219171	0.0265151	0.0162270	0.0283698
TPD52L2	0.2806392	0.2397198	0.2219374	0.2736299	0.3382203
DNAJC5	0.3140589	0.3587278	0.4600605	0.3053945	0.3600648
UCKL1	0.1012481	0.0976096	0.0962083	0.0977000	0.1019472
UCKL1-AS1	0.0028077	0.0019613	0.0019672	0.0092647	0.0071701
ZNF512B	0.1553736	0.2285453	0.2181731	0.1464861	0.1677506
SAMD10	0.1166615	0.2198986	0.3155936	0.1531244	0.1110326
PRPF6	0.7258471	0.6471182	0.6468152	0.6319635	0.6057897
C20orf204	0.0035466	0.0046118	0.0000000	0.0000000	0.0050314
SOX18	0.0014377	0.0040697	0.0020745	0.0080900	0.0000000
TCEA2	0.6922275	0.6456972	0.6218648	0.7079077	0.4737980
RGS19	0.0465687	0.0790617	0.1150093	0.0487660	0.0524882
OPRL1	0.0216527	0.0396324	0.0793913	0.0388449	0.0318010

LKAAEAR1	0.0207410	0.0397247	0.0593830	0.0206240	0.0185678
ENSG00000286999	0.0000000	0.0018625	0.0000000	0.0073611	0.0000000
MYT1	0.0389220	0.0863555	0.0848509	0.0305038	0.2183936
PCMTD2	0.3399342	0.2920772	0.3391624	0.4059185	0.3580317
ENSG00000274333	0.0000000	0.0000000	0.0000000	0.0020594	0.0000000
ENSG00000279064	0.0010655	0.0032156	0.0034917	0.0115008	0.0000000
ENSG00000288187	0.0087761	0.0229468	0.0089149	0.0201672	0.0000000
ENSG00000278903	0.0354306	0.0655797	0.0466205	0.0221193	0.0667002
ENSG00000280145	0.0762173	0.0838559	0.0666470	0.0809091	0.0286227
ENSG00000280164	0.0253599	0.0320955	0.0685184	0.0296354	0.0477075
ENSG00000279998	0.0000000	0.0000000	0.0018141	0.0028001	0.0000000
ENSG00000280018	0.0029174	0.0052149	0.0136264	0.0000000	0.0000000
ENSG00000278996	0.0097848	0.0047606	0.0000000	0.0069595	0.0058242
ENSG00000280800	0.0042436	0.0069100	0.0000000	0.0000000	0.0000000
ENSG00000281383	0.0096801	0.0157085	0.0105250	0.0047879	0.0000000
ENSG00000280441	0.1870089	0.1560709	0.1037857	0.1227366	0.1555206
ENSG00000280614	0.0076571	0.0070485	0.0107567	0.0031895	0.0062217
ENSG00000281181	0.0062965	0.0091515	0.0059674	0.0072844	0.0051893
ENSG00000286033	0.0000000	0.0000000	0.0000000	0.0000000	0.0115044
TEKT4P2	0.0032496	0.0000000	0.0000000	0.0000000	0.0027158
ENSG00000278932	0.0073733	0.0073837	0.0052452	0.0126756	0.0120577
TPTE	0.0000000	0.0000000	0.0000000	0.0000000	0.0000000
ENSG00000290581	0.0000000	0.0000000	0.0000000	0.0000000	0.0000000
ANKRD20A11P	0.0010735	0.0000000	0.0000000	0.0000000	0.0000000
ENSG00000224905	0.0514730	0.0698712	0.0935116	0.0510671	0.1341141
LIPI	0.0012704	0.0009596	0.0015470	0.0000000	0.0000000
RBM11	0.0591169	0.1172697	0.1492780	0.0517330	0.0573102
ABCC13	0.0006714	0.0021513	0.0014397	0.0000000	0.0049014
HSPA13	0.5568426	0.4952336	0.6766926	0.7336665	0.5530394
SAMSN1	0.0013010	0.0053238	0.0000000	0.0000000	0.0076218
ENSG00000232884	0.0027218	0.0014991	0.0054452	0.0000000	0.0072806
ENSG00000291290	0.0087996	0.0053999	0.0027300	0.0065124	0.0023501
ENSG00000291291	0.0041713	0.0008999	0.0010907	0.0000000	0.0000000
LINC02246	0.0327319	0.0211852	0.0219785	0.0204138	0.0220331
ENSG00000235277	0.0000000	0.0000000	0.0000000	0.0000000	0.0000000
NRIP1	0.4549734	0.6633824	0.7362559	0.3105214	0.8054676
ENSG00000231201	0.0000000	0.0018046	0.0014263	0.0000000	0.0000000
ENSG00000229425	0.0084014	0.0064085	0.0043329	0.0060444	0.0253463
USP25	0.2839467	0.3001345	0.2473006	0.2368402	0.4718932
MIR99AHG	0.6496568	0.6216732	0.4585815	0.5944824	1.3397444
ENSG00000270071	0.0522798	0.0808645	0.0894227	0.0809794	0.1215724
ENSG00000269950	0.0005580	0.0000000	0.0000000	0.0000000	0.0000000
ENSG00000237735	0.0020544	0.0000000	0.0014605	0.0000000	0.0000000
CXADR	0.6985673	0.8578543	1.3701052	0.8101145	0.8597415
BTG3	1.4810437	1.0531329	0.7259119	1.4525428	0.8989177
BTG3-AS1	0.0877331	0.0778366	0.0381452	0.0902986	0.0500096
C21orf91-OT1	0.0018924	0.0007406	0.0000000	0.0000000	0.0090028
C21orf91	0.3073625	0.2381008	0.1544021	0.3380182	0.1681493
ENSG00000244676	0.0008195	0.0000000	0.0000000	0.0000000	0.0071630
CHODL	0.1185391	0.3046601	0.1227019	0.1254308	0.1525846
ENSG00000227330	0.0000000	0.0000000	0.0000000	0.0000000	0.0000000
TMPRSS15	0.0000000	0.0000000	0.0000000	0.0000000	0.0000000
LINC00320	0.0027218	0.0038434	0.0028524	0.0042115	0.0000000

NCAM2	0.4024283	0.5688087	0.5686986	0.3141141	1.2645285
ENSG00000234730	0.0000000	0.0013354	0.0000000	0.0000000	0.0000000
ENSG00000226043	0.0000000	0.0012489	0.0000000	0.0000000	0.0073726
LINC01684	0.0119822	0.0147711	0.0121630	0.0018030	0.0076708
LINC01692	0.0014910	0.0000000	0.0015825	0.0000000	0.0000000
ENSG00000222042	0.0031859	0.0022728	0.0024822	0.0029272	0.0052949
LINC00158	0.0090491	0.0117152	0.0042624	0.0167531	0.0201095
ENSG00000229962	0.0021539	0.0000000	0.0000000	0.0000000	0.0000000
MIR155HG	0.0000000	0.0000000	0.0000000	0.0018489	0.0042775
ENSG00000260583	0.0092277	0.0029505	0.0036207	0.0034141	0.0031305
MRPL39	0.1996884	0.2079641	0.2089283	0.1919997	0.1237905
JAM2	0.2920424	0.1589471	0.1531479	0.5137237	0.2452707
ENSG00000287109	0.0015991	0.0000000	0.0000000	0.0000000	0.0000000
ATP5PF	1.4040396	1.4937581	1.5489317	1.4013101	1.1819037
GABPA	0.2409654	0.1581390	0.1531841	0.2258087	0.1421123
APP	1.8145481	1.8844393	2.2540566	2.3387151	2.0260596
ENSG00000233783	0.0024543	0.0000000	0.0031202	0.0000000	0.0509464
ENSG00000224541	0.0044053	0.0083375	0.0028378	0.0065272	0.0263872
APP-DT	0.0352932	0.0429843	0.0322312	0.0285937	0.0157465
ENSG00000289899	0.0738208	0.0696067	0.0688442	0.0588069	0.0593921
ENSG00000232692	0.0057288	0.0075346	0.0029429	0.0000000	0.0110451
CYYR1-AS1	0.0113544	0.0059692	0.0036237	0.0000000	0.0257317
CYYR1	0.0352373	0.0370530	0.0173108	0.0160764	0.0593622
ADAMTS1	0.1120446	0.0958757	0.0461515	0.1785073	0.0788722
ENSG00000223563	0.0000000	0.0014312	0.0000000	0.0000000	0.0000000
ADAMTS5	0.0296492	0.0506047	0.0190588	0.0195481	0.0409388
ENSG00000231236	0.0002195	0.0020497	0.0000000	0.0044812	0.0090796
LINC01697	0.0000000	0.0019015	0.0000000	0.0023899	0.0000000
LINC01695	0.0087244	0.0074219	0.0026716	0.0051624	0.0544632
ENSG00000232855	0.0417547	0.0618343	0.0319219	0.0327887	0.2487086
N6AMT1	0.1418625	0.1223175	0.1290204	0.1549104	0.1045950
LTN1	0.4075569	0.4212430	0.4901392	0.3752594	0.4252457
RWDD2B	0.0993359	0.1272939	0.1028974	0.1002070	0.0729864
ENSG00000273254	0.0077880	0.0051728	0.0026392	0.0030809	0.0047598
USP16	0.6720082	0.5236993	0.4854166	0.6923411	0.4404839
CCT8	1.1328932	1.0607349	1.1371583	1.0744845	0.9215299
ENSG00000231125	0.0009054	0.0000000	0.0000000	0.0000000	0.0000000
ENSG00000286018	0.0005026	0.0000000	0.0000000	0.0000000	0.0023834
MAP3K7CL	0.0069485	0.0065513	0.0086511	0.0107844	0.0050777
BACH1	0.3948019	0.4307312	0.4564056	0.3141881	0.6966633
BACH1-IT1	0.0000000	0.0000000	0.0070421	0.0000000	0.0000000
ENSG00000273017	0.0033762	0.0051103	0.0056156	0.0011543	0.0265063
BACH1-AS1	0.0000000	0.0012400	0.0000000	0.0000000	0.0000000
BACH1-IT2	0.0006999	0.0010716	0.0006909	0.0000000	0.0000000
GRIK1	0.1486027	0.2765368	0.2748444	0.1110606	0.9064400
GRIK1-AS1	0.0008338	0.0015825	0.0010329	0.0000000	0.0026876
TIAM1	0.1303148	0.1651095	0.1763724	0.1430374	0.4745324
ENSG00000286643	0.0000000	0.0008701	0.0000000	0.0000000	0.0047598
TIAM1-AS1	0.0011771	0.0064046	0.0061830	0.0080086	0.0000000
SOD1-DT	0.0000000	0.0000000	0.0000000	0.0000000	0.0000000
SOD1	1.9137795	1.8102520	1.8178811	1.8769801	1.5223663
ENSG00000273271	0.0056957	0.0117417	0.0053230	0.0000000	0.0121844
SCAF4	0.1944935	0.1885177	0.1920764	0.1850011	0.3077256

ENSG00000273091	0.0020493	0.0000000	0.0000000	0.0000000	0.0000000
HUNK	0.0809262	0.1540825	0.2275155	0.0911382	0.2964967
LINC00159	0.0101130	0.0103484	0.0061751	0.0104901	0.0496995
ENSG00000261610	0.0000000	0.0000000	0.0000000	0.0000000	0.0085057
MIS18A	0.1611365	0.1376782	0.1656482	0.1763065	0.0860465
MIS18A-AS1	0.0116865	0.0144689	0.0121908	0.0194587	0.0052433
MRAP	0.0042188	0.0008135	0.0075504	0.0009588	0.0391251
MRAP-AS1	0.0014606	0.0000000	0.0000000	0.0000000	0.0072540
URB1	0.1377052	0.0857179	0.0693581	0.1299274	0.1550816
URB1-AS1	0.0392468	0.0616853	0.0657187	0.0479185	0.0101036
EVA1C	0.5291341	0.2695479	0.1801077	0.6556549	0.2207287
TCP10L	0.0788059	0.0627838	0.0500085	0.0650007	0.1323278
CFAP298	1.2383380	0.8691272	0.7533638	1.3454454	0.7869550
SYNJ1	0.1434149	0.2120301	0.2892172	0.1155353	0.3289543
PAXBP1-AS1	0.0422008	0.0831614	0.0862581	0.0454784	0.0762830
PAXBP1	0.3200641	0.3098473	0.2639126	0.2773242	0.6082425
C21orf62-AS1	0.0696881	0.0801328	0.0383823	0.0890155	0.1639923
C21orf62	0.1520922	0.1234992	0.1671033	0.3696667	0.1760644
ENSG00000227757	0.0009286	0.0004067	0.0014933	0.0023036	0.0028791
OLIG1	0.0014779	0.0089772	0.0010944	0.0000000	0.0099048
IFNAR2	0.1237977	0.1505841	0.1614335	0.1443186	0.1910821
IL10RB-DT	0.0039412	0.0074935	0.0032497	0.0025371	0.0047101
IL10RB	0.1014850	0.0439975	0.0490835	0.1336842	0.0582675
ENSG00000289238	0.0341514	0.0233361	0.0184681	0.0116286	0.0315459
IFNAR1	0.7992586	0.6275486	0.6860994	0.9821497	0.5859093
IFNGR2	0.3119233	0.3639901	0.3692774	0.3099385	0.2823781
TMEM50B	0.3400662	0.3035022	0.2897125	0.3906377	0.2780840
ENSG00000231355	0.0011365	0.0000000	0.0000000	0.0000000	0.0000000
DNAJC28	0.0350345	0.0443488	0.0421082	0.0915388	0.0597554
GART	0.4028150	0.4058409	0.4052207	0.4624616	0.3989789
SON	1.2462172	1.0899274	1.1343248	1.2737014	1.1394177
DONSON	0.1869321	0.1284085	0.1471504	0.1918136	0.1507728
CRYZL1	0.4073014	0.3408006	0.3020402	0.3545557	0.3354837
ITSN1	1.6608079	1.3488188	1.1829725	1.6192733	1.4410506
ATP5PO	1.3005437	1.4345422	1.4632242	1.3094908	1.0907168
LINC00649	0.0317467	0.0221702	0.0249101	0.0325901	0.0281040
MRPS6	0.6379346	0.7165141	0.8694961	0.6324374	0.5756598
SLC5A3	0.2210568	0.2936428	0.5246511	0.2352193	0.3914775
LINC00310	0.0053996	0.0085280	0.0060378	0.0000000	0.0096709
ENSG00000214955	0.0035659	0.0091439	0.0089030	0.0041101	0.0604319
ENSG00000225555	0.0082718	0.0078017	0.0029005	0.0058099	0.0200045
KCNE2	0.0000000	0.0048127	0.0040314	0.0000000	0.0058922
SMIM11	0.3885718	0.4488490	0.4507025	0.4336438	0.2824631
SMIM34	0.0014070	0.0000000	0.0019867	0.0037492	0.0000000
ENSG00000272958	0.0000000	0.0000000	0.0000000	0.0000000	0.0000000
KCNE1	0.0112250	0.0118626	0.0068995	0.0041928	0.0066362
RCAN1	0.3328581	0.3608244	0.5309241	0.4613509	0.3079639
LINC01426	0.0000000	0.0000000	0.0000000	0.0000000	0.0074108
RUNX1	0.0310998	0.0513286	0.0325620	0.0222747	0.0634708
ENSG00000286153	0.0022880	0.0021948	0.0000000	0.0000000	0.0000000
SETD4	0.1393990	0.1181896	0.1115035	0.1647855	0.1380519
SETD4-AS1	0.0000000	0.0000000	0.0000000	0.0000000	0.0000000
CBR1-AS1	0.0020727	0.0011635	0.0015905	0.0000000	0.0000000

CBR1	0.7426861	0.5340004	0.4009836	0.7986007	0.3378415
ENSG00000289001	0.0164500	0.0249986	0.0172618	0.0327334	0.0392079
CBR3-AS1	0.0100556	0.0085245	0.0122537	0.0106406	0.0229131
CBR3	0.0011346	0.0011706	0.0039348	0.0087582	0.0000000
DOP1B	0.1368213	0.1624823	0.1882217	0.0916242	0.2731614
ENSG00000273199	0.0024195	0.0000000	0.0021636	0.0000000	0.0000000
MORC3	0.2803210	0.3238170	0.2633937	0.2325370	0.3384424
ENSG00000228107	0.0013863	0.0011542	0.0000000	0.0000000	0.0000000
CHAF1B	0.0215596	0.0243183	0.0484391	0.0166163	0.0221460
LNCTSI	0.0000000	0.0034151	0.0000000	0.0000000	0.0000000
CLDN14-AS1	0.0000000	0.0000000	0.0025896	0.0000000	0.0000000
CLDN14	0.0011594	0.0008415	0.0012120	0.0000000	0.0000000
SIM2	0.0152020	0.0051564	0.0043113	0.0021669	0.0190372
HLCS	0.2485242	0.2005036	0.1335841	0.2339136	0.3775732
HLCS-AS1	0.0484355	0.0347425	0.0464069	0.0303024	0.0574949
RIPPLY3	0.1883365	0.1223663	0.0919418	0.0859743	0.0789526
PIGP	0.3603081	0.2997114	0.3079113	0.3192051	0.2683794
TTC3	2.6082409	2.7875219	2.9268338	2.4912047	2.5644044
ENSG00000270116	0.0000000	0.0000000	0.0018999	0.0000000	0.0000000
TTC3-AS1	0.0015877	0.0028835	0.0067724	0.0049511	0.0234367
DSCR9	0.0061595	0.0084875	0.0052024	0.0074028	0.0064533
ENSG00000242553	0.0078209	0.0129741	0.0086809	0.0064455	0.0015298
VPS26C	0.1686803	0.1537848	0.1751892	0.1296373	0.1881061
ENSG00000272948	0.0005648	0.0029835	0.0023088	0.0000000	0.0000000
ENSG00000287637	0.0003555	0.0014000	0.0000000	0.0113113	0.0000000
ENSG00000273210	0.0041820	0.0031562	0.0009009	0.0000000	0.0000000
DYRK1A	0.3899053	0.3867020	0.4158294	0.3377057	0.7033597
ENSG00000286717	0.0003438	0.0012538	0.0000000	0.0000000	0.0039411
KCNJ6	0.1369492	0.2446298	0.3575270	0.0879257	0.4979144
ENSG00000289538	0.0000000	0.0000000	0.0009599	0.0000000	0.0015072
ERG	0.0050538	0.0036041	0.0000000	0.0000000	0.0000000
ETS2	0.0980629	0.2095756	0.3076843	0.0565818	0.1737827
ENSG00000205622	0.0006051	0.0020921	0.0015406	0.0043373	0.0000000
LINC02940	0.0025728	0.0042535	0.0049905	0.0112387	0.0069899
PSMG1	0.2765067	0.2981768	0.2805575	0.3034261	0.2026106
BRWD1	0.7234511	0.8564661	0.9902966	0.6609291	0.9213310
ENSG00000272991	0.0000000	0.0000000	0.0000000	0.0000000	0.0000000
BRWD1-AS2	0.0070209	0.0021746	0.0038257	0.0057560	0.0000000
BRWD1-AS1	0.0007963	0.0000000	0.0000000	0.0000000	0.0060675
HMGN1	1.4394336	1.2385248	1.1798018	1.4505318	1.1601400
GET1	0.9338478	0.8978371	0.9470481	1.0456894	0.7967737
LCA5L	0.1535686	0.0635988	0.0257178	0.1890182	0.0597676
SH3BGR	0.1996524	0.1656849	0.1317815	0.1374611	0.1835447
B3GALT5	0.0236435	0.0155459	0.0266911	0.0099899	0.0595484
B3GALT5-AS1	0.0897647	0.0347856	0.0208534	0.1202984	0.0351334
ENSG00000225330	0.0061105	0.0052018	0.0036607	0.0069333	0.0275421
ENSG00000231713	0.0029709	0.0013112	0.0000000	0.0021989	0.0000000
IGSF5	0.0525624	0.0267150	0.0407873	0.0953293	0.0687622
PCP4	0.3810018	0.8879610	0.5690097	0.3247004	0.4547464
DSCAM	0.1873796	0.2405647	0.1858412	0.1675207	1.0373587
DSCAM-AS1	0.0000000	0.0000000	0.0000000	0.0000000	0.0000000
DSCAM-IT1	0.0000000	0.0000000	0.0000000	0.0000000	0.0000000
ENSG00000288069	0.0000000	0.0000000	0.0000000	0.0000000	0.0100072

ENSG00000286425	0.0020258	0.0030185	0.0037227	0.0086612	0.0054224
BACE2	0.0573522	0.0387639	0.0077946	0.0807388	0.0290013
FAM3B	0.0000000	0.0000000	0.0000000	0.0000000	0.0000000
MX2	0.0014444	0.0034855	0.0006833	0.0000000	0.0000000
MX1	0.0270392	0.0087588	0.0079601	0.0111876	0.0179447
RIPK4	0.0219049	0.0079328	0.0042131	0.0162927	0.0000000
ENSG00000289513	0.0003268	0.0000000	0.0000000	0.0000000	0.0000000
PRDM15	0.0252705	0.0368809	0.0604648	0.0513588	0.0604721
C2CD2	0.0768246	0.0781796	0.0983632	0.0720822	0.1736177
ZBTB21	0.3270858	0.3302166	0.3345986	0.2554177	0.2585052
ZNF295-AS1	0.0144382	0.0133139	0.0135117	0.0071781	0.0034716
UMODL1	0.0000000	0.0010432	0.0000000	0.0000000	0.0000000
ABCG1	0.0595446	0.0687632	0.0571015	0.0843195	0.0972031
TFF3	2.1151719	1.6506892	1.1939503	2.0105576	1.5267336
TFF1	0.0099790	0.0099910	0.0056036	0.0013687	0.0000000
TMPRSS3	0.0157511	0.0032676	0.0046883	0.0025494	0.0015531
RSPH1	1.0147379	0.6616136	0.4828550	0.9700595	0.6258466
SLC37A1	0.1930484	0.1832246	0.2126811	0.1971740	0.2866113
ENSG00000235023	0.0009672	0.0055367	0.0054817	0.0000000	0.0000000
PDE9A	0.1529742	0.1729281	0.1578787	0.1679323	0.2597735
PDE9A-AS1	0.0000000	0.0000000	0.0000000	0.0000000	0.0000000
ENSG00000225218	0.0010022	0.0014027	0.0000000	0.0027087	0.0126974
WDR4	0.0417408	0.0456381	0.0642212	0.0349588	0.0965064
NDUFV3	0.7014765	0.7527607	0.7643312	0.7171544	0.6630528
ERVH48-1	0.0005082	0.0000000	0.0000000	0.0000000	0.0026634
PKNOX1	0.2867778	0.2643857	0.2823839	0.2604272	0.3215594
CBS	0.2918296	0.2874363	0.3500742	0.2988420	0.2544647
U2AF1	0.9220079	0.7796179	0.7818148	0.8514219	0.7184736
ENSG00000286549	0.0026620	0.0064720	0.0017068	0.0037456	0.0033561
CRYAA	0.0019382	0.0012831	0.0006954	0.0040443	0.0000000
SIK1	0.0731079	0.0910472	0.0996274	0.0351011	0.1401869
LINC00313	0.0024592	0.0004108	0.0000000	0.0000000	0.0059248
HSF2BP	0.0060160	0.0159276	0.0112148	0.0208350	0.0161041
RRP1B	0.1997787	0.2451568	0.3748289	0.2195027	0.2978493
PDXK	0.4390915	0.5562191	0.6411311	0.3899131	0.4170234
ENSG00000287507	0.0008148	0.0009084	0.0030686	0.0000000	0.0064078
CSTB	1.1730528	1.0306479	0.9595840	1.1620593	0.8149572
RRP1	0.0860962	0.1104879	0.1034675	0.0767332	0.0921522
AATBC	0.0000000	0.0011029	0.0000000	0.0000000	0.0000000
AGPAT3	0.2584362	0.1952617	0.1859651	0.2864050	0.2632232
ENSG00000289546	0.0000000	0.0000000	0.0000000	0.0038435	0.0000000
TRAPPC10	0.2431725	0.2675342	0.2928219	0.2514207	0.4048650
PWP2	0.0587393	0.0545626	0.0560364	0.0929249	0.0389469
GATD3	0.4666517	0.4556082	0.3915957	0.5280319	0.2626281
ICOSLG	0.0084571	0.0098330	0.0025201	0.0089196	0.0044810
PFKL	0.3313720	0.2383760	0.2114723	0.3509625	0.2380698
ENSG00000241728	0.0007616	0.0000000	0.0011903	0.0042829	0.0039284
CFAP410	0.1833769	0.1436052	0.1616236	0.1488242	0.1283073
ENSG00000184441	0.0144473	0.0082714	0.0065261	0.0090114	0.0255338
ENSG00000232969	0.0000000	0.0000000	0.0000000	0.0000000	0.0075468
TRPM2	0.0121905	0.0344247	0.0277181	0.0131480	0.0513510
LRRC3-DT	0.0071311	0.0120724	0.0073014	0.0040556	0.0068318
LRRC3	0.0666248	0.0662586	0.0680217	0.1012497	0.1033543

HEPFAL	0.0091003	0.0088081	0.0116428	0.0112680	0.0175379
LINC02575	0.0018805	0.0029155	0.0044561	0.0000000	0.0000000
TSPEAR	0.0126599	0.0027718	0.0091004	0.0183482	0.0199590
TSPEAR-AS1	0.0000000	0.0046521	0.0010864	0.0042330	0.0016917
TSPEAR-AS2	0.0012953	0.0025740	0.0000000	0.0000000	0.0016917
UBE2G2	0.4543584	0.3459601	0.3101628	0.3811879	0.4322738
LINC01424	0.0089272	0.0036631	0.0019105	0.0000000	0.0000000
SUMO3	0.6803899	0.7507514	0.8459192	0.6104678	0.4553836
PTTG1IP	1.0338862	0.7461391	0.5864640	1.3956280	0.6964798
ENSG00000289009	0.0163005	0.0206643	0.0169042	0.0142496	0.0053957
ITGB2	0.0010767	0.0021464	0.0026012	0.0085538	0.0035158
ENSG00000273027	0.0004671	0.0054856	0.0000000	0.0000000	0.0000000
LINC01547	0.0117875	0.0205081	0.0089764	0.0260654	0.0100452
SLX9	0.1134855	0.1120982	0.1298187	0.0957506	0.1103101
ENSG00000276529	0.0196698	0.0150610	0.0243083	0.0131295	0.0277514
ADARB1	0.0891946	0.1444142	0.1946551	0.0763179	0.3042439
LINC00334	0.0029284	0.0013944	0.0019229	0.0047371	0.0037415
POFUT2	0.2984969	0.2000087	0.1467118	0.4395295	0.2663962
LINC00205	0.3658871	0.3866635	0.5450369	0.3854802	0.4126647
ENSG00000229382	0.0000000	0.0014300	0.0000000	0.0000000	0.0000000
ENSG00000289448	0.0012313	0.0005227	0.0000000	0.0096980	0.0000000
ENSG00000273796	0.0247148	0.0098095	0.0279327	0.0241037	0.0244605
COL18A1	0.2061856	0.1252850	0.0937652	0.4638691	0.2143264
COL18A1-AS1	0.0000000	0.0000000	0.0000000	0.0000000	0.0000000
SLC19A1	0.0447465	0.0517555	0.0425395	0.0412415	0.0599053
ENSG00000288885	0.0486960	0.0500826	0.0594597	0.0208164	0.0112539
LINC01694	0.0112638	0.0110575	0.0121958	0.0104948	0.0067617
ENSG00000286082	0.0006005	0.0000000	0.0000000	0.0000000	0.0000000
PCBP3	0.0803480	0.1251079	0.0580419	0.0473514	0.2689866
PCBP3-AS1	0.0142972	0.0087455	0.0111541	0.0220586	0.0046920
ENSG00000286886	0.0026421	0.0000000	0.0035679	0.0000000	0.0023227
ENSG00000274248	0.0060978	0.0110993	0.0062088	0.0065967	0.0000000
ENSG00000289224	0.0046983	0.0027955	0.0012396	0.0028412	0.0030223
COL6A1	0.7758537	0.6142559	0.5116583	1.0799416	0.6495533
COL6A2	0.1457583	0.0748606	0.0550443	0.2075708	0.0963046
FTCD	0.0051720	0.0086696	0.0033772	0.0033784	0.0211093
SPATC1L	0.0992135	0.1385695	0.1471657	0.0947768	0.1107011
ENSG00000228404	0.0006460	0.0026042	0.0012344	0.0000000	0.0070049
LSS	0.1379754	0.1059516	0.1097417	0.1294688	0.1955643
ENSG00000223901	0.0000000	0.0000000	0.0000000	0.0000000	0.0000000
MCM3AP-AS1	0.0643498	0.0688953	0.0825756	0.0435719	0.0666777
MCM3AP	0.1594022	0.1362742	0.1404134	0.1669581	0.3100207
ENSG00000228137	0.0000000	0.0000000	0.0000000	0.0000000	0.0000000
ENSG00000239415	0.0046951	0.0041069	0.0080581	0.0000000	0.0148501
YBEY	0.1755093	0.1784636	0.2000888	0.1523360	0.0971664
C21orf58	0.0937783	0.0598166	0.0201846	0.0819067	0.0483541
PCNT	0.1707665	0.2102652	0.2299119	0.1180068	0.5256174
ENSG00000289421	0.0077727	0.0013920	0.0042789	0.0000000	0.0080956
DIP2A	0.1628132	0.1409804	0.1417468	0.1473978	0.4868616
DIP2A-IT1	0.0025614	0.0053861	0.0032950	0.0017625	0.0039284
S100B	0.4047387	0.3005329	0.1886393	0.7049854	0.3104001
PRMT2	1.0954169	1.1020098	1.1357112	1.1475263	0.9623417
ENSG00000289511	0.0024508	0.0039634	0.0017421	0.0074491	0.0000000

ENSG00000290397	0.0390332	0.0474382	0.0385704	0.0173024	0.0838438
ENSG00000280341	0.0013417	0.0000000	0.0000000	0.0000000	0.0000000
TPTEP1	0.0012829	0.0000000	0.0000000	0.0000000	0.0000000
ENSG00000290931	0.0000000	0.0134004	0.0043452	0.0000000	0.0132017
IL17RA	0.0138554	0.0113863	0.0111807	0.0111640	0.0146745
TMEM121B	0.0192866	0.0516048	0.0608592	0.0165971	0.0453808
LINC01664	0.0191063	0.0205388	0.0188439	0.0049875	0.0214748
HDHD5	0.2174220	0.2165875	0.2746070	0.2723059	0.1974995
HDHD5-AS1	0.0572453	0.0417056	0.0369209	0.0379091	0.0501125
ADA2	0.1262806	0.1242311	0.0854539	0.0790929	0.1064756
CECR3	0.0010727	0.0009522	0.0000000	0.0000000	0.0000000
CECR2	0.1623878	0.1323843	0.0814206	0.1100828	0.3418427
ENSG00000286195	0.0026592	0.0042076	0.0000000	0.0021841	0.0100074
SLC25A18	0.0085556	0.0117363	0.0083098	0.0066556	0.0084835
ENSG00000236754	0.0000000	0.0007457	0.0000000	0.0000000	0.0000000
ATP6V1E1	0.4654274	0.6025411	0.5775774	0.4459226	0.3918765
BCL2L13	0.3397442	0.2871311	0.2437498	0.3721754	0.2899824
BID	0.1080123	0.2115227	0.2925812	0.1023110	0.1950244
MICAL3	0.2751519	0.3722512	0.4523004	0.2616446	0.8254077
ENSG00000286990	0.0000000	0.0000000	0.0000000	0.0000000	0.0000000
ENSG00000234913	0.0000000	0.0019459	0.0000000	0.0000000	0.0047971
ENSG00000225335	0.0044938	0.0012321	0.0017803	0.0000000	0.0000000
PEX26	0.1834457	0.2459904	0.2408661	0.1827871	0.2215773
TUBA8	0.0159500	0.0498962	0.0859653	0.0427742	0.0542565
ENSG00000280007	0.0012022	0.0000000	0.0000000	0.0000000	0.0013692
USP18	0.0147441	0.0260011	0.0019944	0.0281033	0.0089784
TMEM191B	0.0164499	0.0194354	0.0236125	0.0137318	0.0046437
PI4KAP1	0.1525268	0.1444546	0.1152703	0.1678303	0.2911386
ENSG00000290711	0.0000000	0.0000000	0.0017297	0.0000000	0.0000000
DGCR6	0.0541175	0.0702558	0.0963101	0.0363154	0.0708634
PRODH	0.0004258	0.0000000	0.0000000	0.0000000	0.0000000
DGCR5	0.1372755	0.2852006	0.3022383	0.1493798	0.4698591
FAM246C	0.0021399	0.0022885	0.0071461	0.0000000	0.0000000
DGCR2	0.1425538	0.1622769	0.1822713	0.1959414	0.3077246
DGCR11	0.0032901	0.0011324	0.0012851	0.0049427	0.0000000
ENSG00000281530	0.0000000	0.0012840	0.0000000	0.0000000	0.0000000
ENSG00000223461	0.0010244	0.0041783	0.0079042	0.0026849	0.0000000
ESS2	0.0735765	0.0806135	0.0591014	0.0565158	0.0511716
LINC01311	0.0015563	0.0000000	0.0000000	0.0000000	0.0019007
SLC25A1	0.4965777	0.4120180	0.4057500	0.4725940	0.3717616
CLTCL1	0.0493549	0.0502056	0.0297605	0.0350322	0.1211072
ENSG00000287146	0.0000000	0.0035014	0.0026200	0.0000000	0.0000000
HIRA	0.0767309	0.0878233	0.1109600	0.0788116	0.1014953
C22orf39	0.5731516	0.5721807	0.6627931	0.4874501	0.4376192
MRPL40	0.5858191	0.5442041	0.5033772	0.6090741	0.3919061
UFD1-AS1	0.0075493	0.0057977	0.0115970	0.0036665	0.0154325
UFD1	0.4432311	0.3537839	0.3589833	0.3647456	0.2811211
ENSG00000273300	0.0010001	0.0000000	0.0017509	0.0000000	0.0000000
ENSG00000273212	0.0000000	0.0000000	0.0018325	0.0000000	0.0000000
CDC45	0.0025943	0.0012480	0.0000000	0.0070419	0.0039666
CLDN5	0.0387852	0.0296786	0.0258043	0.0744651	0.0109471
SEPTIN5	0.3540998	0.5345173	0.6770858	0.3817152	0.4819225
GP1BB	0.0744893	0.1506988	0.1752290	0.0835448	0.1220172

TBX1	0.0000000	0.0000000	0.0000000	0.0000000	0.0000000
GNB1L	0.0414992	0.0594860	0.0460701	0.0539235	0.0743021
RTL10	0.0667194	0.0634082	0.0719489	0.0738551	0.0839870
ENSG00000289960	0.0160325	0.0085844	0.0038911	0.0096303	0.0103096
TXNRD2	0.0928126	0.0743938	0.0567741	0.1063967	0.0648685
COMT	1.1481955	0.8425545	0.6637926	1.3507046	0.7469208
ARVCF	0.1206038	0.0991162	0.0871766	0.1439463	0.2343278
TANGO2	0.0563142	0.0681638	0.0459365	0.0465900	0.0816509
ENSG00000236540	0.0006301	0.0008007	0.0025575	0.0000000	0.0000000
ENSG00000268292	0.0117864	0.0086349	0.0019820	0.0127656	0.0067724
DGCR8	0.0753054	0.0768604	0.0444969	0.0509274	0.1399801
TRMT2A	0.0875768	0.0546688	0.0379766	0.0648010	0.0569280
RANBP1	0.8945768	0.9545106	1.0045341	0.8930991	0.7565270
ZDHHC8	0.0499736	0.0510199	0.0518194	0.0599274	0.0709905
CCDC188	0.0021784	0.0021535	0.0047972	0.0025032	0.0032941
RTN4R	0.2353841	0.1799327	0.2278655	0.3817111	0.1881977
DGCR6L	0.5073466	0.4632225	0.4097320	0.4474516	0.3270643
ENSG00000273343	0.0005543	0.0042081	0.0038452	0.0021832	0.0000000
ENSG00000273139	0.0030047	0.0018404	0.0000000	0.0000000	0.0035834
ENSG00000235578	0.0103665	0.0125146	0.0000000	0.0015092	0.0000000
ENSG00000287446	0.0187217	0.0141448	0.0023449	0.0103150	0.0030611
ENSG00000290950	0.0104135	0.0057323	0.0071877	0.0040702	0.0000000
ZNF74	0.0756921	0.1016593	0.1126949	0.0842131	0.1187049
SCARF2	0.1936753	0.1327103	0.0727238	0.2657740	0.1303001
KLHL22	0.1662628	0.1542937	0.1906406	0.1777367	0.2230391
MED15	0.2142454	0.1917658	0.1627004	0.1842233	0.2671765
ENSG00000290981	0.0008992	0.0008497	0.0000000	0.0000000	0.0000000
TMEM191A	0.1243546	0.1144201	0.1807970	0.1229601	0.1030998
PI4KA	0.1708205	0.1849498	0.1593572	0.1899768	0.5259835
SERPIND1	0.0420737	0.0250844	0.0110194	0.0462654	0.0155160
SNAP29	0.3566561	0.3256405	0.3071086	0.3073321	0.2199762
CRKL	0.2478695	0.2037600	0.2012016	0.2453282	0.2583014
LINC01637	0.0101914	0.0108232	0.0026075	0.0099367	0.0064609
AIFM3	0.0070122	0.0076165	0.0029709	0.0000000	0.0136013
ENSG00000272829	0.0213181	0.0237430	0.0213954	0.0180285	0.0102140
LZTR1	0.0867268	0.0715957	0.0546286	0.0694425	0.0987505
THAP7	0.1742644	0.1470349	0.1266010	0.1638673	0.1019679
THAP7-AS1	0.0712715	0.0498559	0.0441340	0.0775028	0.0549165
TUBA3FP	0.0078231	0.0268037	0.0391877	0.0031190	0.0355243
P2RX6	0.0060131	0.0154792	0.0062733	0.0102180	0.0037236
SLC7A4	0.0072160	0.0105396	0.0103394	0.0027381	0.0165435
ENSG00000226872	0.0000000	0.0000000	0.0000000	0.0000000	0.0076895
ENSG00000290799	0.0402451	0.0235057	0.0135299	0.0355471	0.0228904
LRRC74B	0.0112983	0.0096087	0.0078855	0.0278882	0.0000000
ENSG00000291044	0.0290042	0.0322291	0.0312390	0.0307792	0.0279915
ENSG00000290983	0.0005894	0.0007457	0.0015124	0.0000000	0.0000000
HIC2	0.0807503	0.1079334	0.0997513	0.0827863	0.2557329
TMEM191C	0.1137339	0.1202738	0.1363712	0.1143198	0.0875955
UBE2L3	1.0493513	0.9391688	0.9326733	1.0460552	0.7742761
YDJC	0.1258373	0.1301596	0.1379008	0.1161929	0.0764146
CCDC116	0.0016381	0.0000000	0.0019944	0.0000000	0.0058270
SDF2L1	0.1664950	0.1055473	0.0595398	0.2187914	0.0978460
ENSG00000207751	0.0300564	0.0154970	0.0075258	0.0142358	0.0229491

ENSG00000272954	0.0000000	0.0065315	0.0019382	0.0000000	0.0000000
PPIL2	0.1268628	0.1163730	0.1128774	0.1463315	0.2200289
YPEL1	0.1537649	0.2835787	0.3489481	0.1522239	0.2895547
ENSG00000286127	0.0000000	0.0000000	0.0000000	0.0000000	0.0061791
MAPK1	0.6503059	0.6064334	0.6598667	0.6547028	0.6130355
ENSG00000289564	0.0003347	0.0007544	0.0025702	0.0000000	0.0137601
PPM1F	0.0318999	0.0593159	0.0567784	0.0593635	0.0787662
PPM1F-AS1	0.0096954	0.0149873	0.0188854	0.0168532	0.0105415
TOP3B	0.0155777	0.0206665	0.0218405	0.0168485	0.0414607
ENSG00000279278	0.0000000	0.0000000	0.0000000	0.0000000	0.0000000
ENSG00000274422	0.0626217	0.0669837	0.0497486	0.0431679	0.3313865
ENSG00000234630	0.0000000	0.0000000	0.0000000	0.0000000	0.0038490
IGLV5-52	0.0003241	0.0031655	0.0029609	0.0000000	0.0000000
ZNF280B	0.1263618	0.1181478	0.1485756	0.1289514	0.1273792
RSPH14	0.2047663	0.2257465	0.2483558	0.1239559	0.2030964
GNAZ	0.3432037	0.4835447	0.5992164	0.2535478	0.3918119
RAB36	0.1358325	0.0937107	0.0595093	0.1865680	0.0940291
BCR	0.0998899	0.1992392	0.1979499	0.1085830	0.2259785
LINC02556	0.0000000	0.0000000	0.0000000	0.0000000	0.0000000
ENSG00000290449	0.0117903	0.0350880	0.0546404	0.0145682	0.0645507
ENSG00000290920	0.1032654	0.1914622	0.2602605	0.1160788	0.1738657
LINC01659	0.0017406	0.0026225	0.0031243	0.0050539	0.0000000
PCAT14	0.0007541	0.0000000	0.0007596	0.0023928	0.0000000
IGLL1	0.0011118	0.0000000	0.0000000	0.0000000	0.0000000
ENSG00000272733	0.0016417	0.0010796	0.0026528	0.0061393	0.0134422
DRICH1	0.0025843	0.0017879	0.0014054	0.0000000	0.0015176
ENSG00000290958	0.0272562	0.0300776	0.0137947	0.0226966	0.0243388
ENSG00000291299	0.0108014	0.0089902	0.0072585	0.0103603	0.0605169
RGL4	0.0000000	0.0000000	0.0059341	0.0000000	0.0035086
ENSG00000290440	0.0035678	0.0039700	0.0032864	0.0040191	0.0502216
ZNF70	0.0741501	0.0599924	0.0803879	0.0823219	0.1185396
VPREB3	0.0047155	0.0041626	0.0095897	0.0107319	0.0065001
C22orf15	0.0713661	0.0314858	0.0027423	0.0512953	0.0186434
CHCHD10	0.5930634	0.5734235	0.6712824	0.5458256	0.3689100
MMP11	0.0607283	0.0750834	0.0377083	0.0745346	0.0954604
SMARCB1	0.7427278	0.7416860	0.7614617	0.7935933	0.6415697
DERL3	0.0245658	0.0150179	0.0015036	0.0202875	0.0022313
ENSG00000272973	0.0404354	0.0616622	0.0880152	0.0402744	0.0305686
SLC2A11	0.1253306	0.1352843	0.1304365	0.1152398	0.2043036
ENSG00000279467	0.0000000	0.0000000	0.0000000	0.0000000	0.0000000
MIF	3.1088546	3.0195012	2.9018833	3.0350757	2.7204221
MIF-AS1	0.0384061	0.0428574	0.0105636	0.0391345	0.0483088
ENSG00000273295	0.0049238	0.0214184	0.0106659	0.0019455	0.0016281
GSTT2B	0.0004082	0.0014260	0.0000000	0.0000000	0.0037333
DDTL	0.0046807	0.0021342	0.0034644	0.0068008	0.0034773
DDT	0.9746430	0.9584036	0.9743310	0.9441892	0.7675453
GSTT2	0.0000000	0.0015971	0.0000000	0.0000000	0.0000000
CABIN1	0.2494725	0.2908835	0.2511887	0.2707100	0.4816070
ENSG00000232545	0.0000000	0.0010205	0.0000000	0.0000000	0.0015298
SUSD2	0.0066301	0.0084205	0.0000000	0.0087168	0.0154085
GGT5	0.0053710	0.0045590	0.0000000	0.0426979	0.0000000
POM121L9P	0.0000000	0.0010850	0.0000000	0.0000000	0.0000000
SPECC1L	0.5246787	0.5007687	0.5822152	0.5109935	0.8051774

ADORA2A	0.0375681	0.0493376	0.0177668	0.0325055	0.0732156
ADORA2A-AS1	0.0005434	0.0000000	0.0019437	0.0000000	0.0052694
UPB1	0.0044768	0.0023229	0.0000000	0.0000000	0.0296945
GUCD1	0.0980895	0.0650303	0.0680804	0.1221329	0.0819589
SNRPD3	0.9741039	0.9546645	1.0315356	1.0222683	0.8589536
LRRC75B	0.0845390	0.0872242	0.0857352	0.0973452	0.0960138
GGT1	0.0015456	0.0027928	0.0009222	0.0029974	0.0109788
ENSG00000230947	0.0000000	0.0000000	0.0000000	0.0000000	0.0000000
ENSG00000284070	0.0002772	0.0022832	0.0000000	0.0000000	0.0000000
ENSG00000290697	0.0023134	0.0078444	0.0051573	0.0039751	0.0243662
SGSM1	0.0829130	0.0955932	0.1167358	0.0524530	0.2509046
LHFPL7	0.0000000	0.0000000	0.0000000	0.0000000	0.0000000
KIAA1671	0.1300029	0.0944793	0.0628193	0.1382550	0.1608620
ENSG00000279085	0.0000000	0.0000000	0.0026158	0.0000000	0.0015531
ENSG00000279110	0.0000000	0.0000000	0.0000000	0.0000000	0.0000000
KIAA1671-AS1	0.0026015	0.0025783	0.0049599	0.0053172	0.0000000
CRYBB3	0.0000000	0.0000000	0.0017362	0.0000000	0.0025988
CRYBB2	0.0022972	0.0007908	0.0047477	0.0000000	0.0027747
ENSG00000233577	0.0060180	0.0081201	0.0284165	0.0039942	0.0078731
ENSG00000290796	0.0178603	0.0108905	0.0085902	0.0024198	0.0364249
ENSG00000272942	0.0008299	0.0049527	0.0063250	0.0000000	0.0035970
CRYBB2P1	0.1499430	0.1510309	0.1957621	0.1512394	0.2474005
ENSG00000272977	0.0111087	0.0068620	0.0084599	0.0000000	0.0115718
GRK3-AS1	0.0039301	0.0019572	0.0017966	0.0018326	0.0014182
GRK3	0.2988828	0.4657088	0.4330404	0.2750034	0.6495175
ENSG00000237387	0.0000000	0.0000000	0.0000000	0.0000000	0.0000000
MYO18B	0.0033049	0.0032299	0.0054383	0.0103193	0.0085540
ENSG00000229770	0.0017353	0.0052997	0.0057764	0.0000000	0.0074605
SEZ6L	0.1474180	0.2103942	0.3023938	0.1284330	0.4753327
SEZ6L-AS1	0.0000000	0.0000000	0.0000000	0.0000000	0.0000000
ASPHD2	0.0482406	0.1354177	0.1936179	0.0274745	0.0661550
HPS4	0.1890377	0.1667928	0.2023158	0.1902168	0.2617800
SRRD	0.1606055	0.1727675	0.1966306	0.1569784	0.1542947
TFIP11	0.1280869	0.0992359	0.1141873	0.1382185	0.0805294
TFIP11-DT	0.1202279	0.1610662	0.1688648	0.1164847	0.1241213
TPST2	0.3472169	0.1999216	0.1694011	0.3564254	0.2104982
ENSG00000286326	0.0004775	0.0010432	0.0000000	0.0057219	0.0046972
CRYBB1	0.0030447	0.0032518	0.0000000	0.0000000	0.0000000
CRYBA4	0.0035656	0.0019572	0.0000000	0.0020616	0.0000000
MIAT	0.1380348	0.2476500	0.1433019	0.1349680	0.7350872
ENSG00000206028	0.0059757	0.0066356	0.0062742	0.0000000	0.0103844
MIATNB	0.0290020	0.0263124	0.0148618	0.0300770	0.0645300
LINC01422	0.0161674	0.0138025	0.0041815	0.0075879	0.0353309
ENSG00000279440	0.0003303	0.0000000	0.0000000	0.0000000	0.0081124
ENSG00000231405	0.0028797	0.0006156	0.0101904	0.0000000	0.0000000
LINC01638	0.0000000	0.0020748	0.0000000	0.0016020	0.0000000
LINC02554	0.0013834	0.0011324	0.0000000	0.0000000	0.0052984
MN1	0.0524957	0.1004256	0.0999407	0.0464024	0.1571403
PITPNB	0.5243907	0.5339180	0.5711232	0.5618626	0.4873499
TTC28-AS1	0.1693610	0.2317512	0.2419382	0.1388026	0.3323236
TTC28	0.5104173	0.5444389	0.4821076	0.4813204	1.4612483
CHEK2	0.0434054	0.0248784	0.0119608	0.0449132	0.0239320
HSCB	0.2301099	0.1365929	0.1528228	0.2066827	0.1804313

CCDC117	0.2339112	0.1719209	0.1960443	0.1749595	0.1799892
XBP1	1.0154407	0.7250741	0.5845384	0.9911401	0.6711838
ENSG00000226471	0.0768389	0.0677195	0.0417434	0.0412516	0.0912450
ZNRF3	0.1460072	0.0855547	0.0705774	0.1300552	0.2480335
ZNRF3-AS1	0.0011231	0.0027642	0.0000000	0.0000000	0.0000000
C22orf31	0.0039225	0.0075734	0.0034104	0.0025788	0.0000000
KREMEN1	0.1792769	0.1087664	0.0898630	0.2099970	0.0986362
ENSG00000226772	0.0000000	0.0000000	0.0000000	0.0000000	0.0000000
EMID1	0.0650913	0.0859755	0.1476562	0.0929211	0.1635469
RHBDD3	0.3006462	0.1990474	0.1845325	0.2999324	0.2267057
ENSG00000237015	0.0010438	0.0016082	0.0000000	0.0000000	0.0034716
EWSR1	0.5635381	0.5621582	0.5311734	0.5166716	0.5689863
GAS2L1	0.1254482	0.0746467	0.0346556	0.1678947	0.0684630
RASL10A	0.0523040	0.0440615	0.0203850	0.0605155	0.0430514
AP1B1	0.1652335	0.1519084	0.1604568	0.1853182	0.1295027
ENSG00000290778	0.0000000	0.0013161	0.0000000	0.0000000	0.0030106
RFPL1S	0.2528332	0.5953402	0.6435662	0.1400585	0.4796954
RFPL1	0.0012354	0.0102135	0.0056927	0.0077855	0.0000000
NEFH	0.1684489	0.3533763	0.1642495	0.1368593	0.1968338
THOC5	0.0701350	0.1190319	0.0950699	0.0696101	0.1205772
NIPSNAP1	0.5239549	0.6133277	0.7465238	0.4940252	0.5224407
NF2	0.1878207	0.2100945	0.2856317	0.1972959	0.2603335
CABP7	0.0035837	0.0078670	0.0006315	0.0000000	0.0013481
ZMAT5	0.2045671	0.1930760	0.2230855	0.2057341	0.1369603
UQCR10	1.3104067	1.4287026	1.5086521	1.2438048	1.0868309
ASCC2	0.1235143	0.1139815	0.1035070	0.1435056	0.1423502
MTMR3	0.1458666	0.1769898	0.1583091	0.1621639	0.3661508
ENSG00000279159	0.0142480	0.0154362	0.0158547	0.0150230	0.0339594
HORMAD2-AS1	0.0013952	0.0000000	0.0000000	0.0000000	0.0267000
HORMAD2	0.0033477	0.0008472	0.0000000	0.0000000	0.0000000
ENSG00000225676	0.0023288	0.0000000	0.0000000	0.0039696	0.0000000
LIF	0.0008234	0.0027187	0.0000000	0.0055417	0.0000000
CASTOR1	0.0482373	0.0253970	0.0205575	0.0775034	0.0201691
TBC1D10A	0.0464726	0.0474770	0.0580050	0.0279216	0.0726279
SF3A1	0.2591895	0.2228236	0.2025389	0.2266593	0.1650300
CCDC157	0.0699853	0.0831617	0.0584011	0.0752134	0.0783165
RNF215	0.1223466	0.1200699	0.1273640	0.1582705	0.1630832
SEC14L2	0.0363684	0.0233673	0.0249039	0.0433175	0.0390975
ENSG00000272689	0.0027370	0.0011383	0.0000000	0.0000000	0.0019688
MTFP1	0.0806391	0.0796521	0.0663183	0.0382390	0.0510002
ENSG00000273428	0.0000000	0.0000000	0.0000000	0.0000000	0.0000000
SEC14L3	0.0000000	0.0000000	0.0000000	0.0000000	0.0000000
SEC14L4	0.0010890	0.0000000	0.0000000	0.0000000	0.0000000
SEC14L6	0.0104095	0.0071606	0.0086416	0.0177510	0.0000000
GAL3ST1	0.0254571	0.0246747	0.0437482	0.0098408	0.0443916
PES1	0.2081726	0.1965902	0.1778298	0.1485406	0.1302709
TCN2	0.0270972	0.0286973	0.0490997	0.0313467	0.0618548
SLC35E4	0.1052455	0.1083764	0.1346841	0.1111972	0.1588372
DUSP18	0.2597011	0.2604037	0.2328600	0.3061648	0.3093201
OSBP2	0.0560478	0.0751624	0.1038430	0.0539177	0.3125201
MORC2-AS1	0.0196097	0.0311216	0.0188174	0.0039833	0.0300341
MORC2	0.1490740	0.2267872	0.2671455	0.1449791	0.2915195
TUG1	0.4985393	0.5475415	0.6462463	0.5001272	0.5910117

ENSG00000269987	0.0045194	0.0000000	0.0091940	0.0010179	0.0000000
ENSG00000278920	0.0026311	0.0000000	0.0000000	0.0000000	0.0034730
SMTN	0.0103164	0.0163288	0.0109309	0.0076078	0.0134827
SELENOM	0.4220099	0.3798135	0.3111809	0.4841286	0.2792457
INPP5J	0.0250406	0.0460495	0.0359011	0.0170663	0.0333595
RNF185	0.1782450	0.1271312	0.1124953	0.1345737	0.1425688
LIMK2	0.0985208	0.1159589	0.1433180	0.1251714	0.2376434
PIK3IP1	0.0300443	0.0125232	0.0305803	0.0948141	0.0082896
PIK3IP1-DT	0.0049283	0.0048202	0.0038664	0.0000000	0.0224805
PATZ1	0.2021920	0.1347918	0.1350534	0.1550693	0.1397376
LINC01521	0.0226597	0.0251851	0.0234618	0.0115407	0.0275149
DRG1	0.3469547	0.3176077	0.3140471	0.3705152	0.3229216
EIF4ENIF1	0.2958425	0.3412874	0.2871442	0.2315934	0.2586747
ENSG00000240591	0.0008608	0.0000000	0.0000000	0.0040641	0.0055669
SFI1	0.0634011	0.0683833	0.0391162	0.0595189	0.1576377
ENSG00000287817	0.0081177	0.0066200	0.0036990	0.0100379	0.0034190
PISD	0.0955071	0.0866129	0.0788269	0.0763364	0.1357230
PRR14L	0.3547053	0.4493442	0.4878184	0.3539099	0.4591298
DEPDC5	0.0422183	0.0500133	0.0399045	0.0414460	0.1385961
YWHAH-AS1	0.0008295	0.0024670	0.0113463	0.0076824	0.0070279
YWHAH	1.4847270	1.9523029	2.3348233	1.4022691	1.5151249
ENSG00000227519	0.0000000	0.0000000	0.0000000	0.0000000	0.0000000
LINC02558	0.0016384	0.0000000	0.0000000	0.0000000	0.0000000
SLC5A1	0.0000000	0.0000000	0.0000000	0.0000000	0.0000000
C22orf42	0.0558454	0.1236216	0.3042492	0.0523735	0.1206812
RFPL2	0.0088320	0.0387820	0.0130277	0.0051545	0.0370982
SLC5A4-AS1	0.0269266	0.0510140	0.0604510	0.0261837	0.1560511
SLC5A4	0.0000000	0.0012802	0.0000000	0.0000000	0.0014252
ENSG00000289873	0.0050745	0.0027336	0.0040501	0.0000000	0.0084878
ENSG00000273325	0.0055870	0.0072751	0.0017326	0.0000000	0.0098736
RFPL3S	0.0043985	0.0054786	0.0111602	0.0041661	0.0161531
RTCB	0.3829970	0.3613331	0.3435324	0.4393082	0.2119495
BPIFC	0.0000000	0.0010945	0.0000000	0.0036229	0.0028456
FBXO7	0.4962096	0.4089499	0.3756528	0.4501416	0.3468105
SYN3	0.2242035	0.4153594	0.3605047	0.2492514	1.2636161
ENSG00000229673	0.0006290	0.0000000	0.0000000	0.0000000	0.0097841
TIMP3	0.4603678	0.2351342	0.1442672	1.0369823	0.2546140
LINC01640	0.0010193	0.0000000	0.0000000	0.0011493	0.0033613
LARGE1	0.1086314	0.1406864	0.1423527	0.0747751	0.5544219
LARGE-AS1	0.0006385	0.0000000	0.0000000	0.0000000	0.0036626
LARGE1-AS1	0.0000000	0.0020742	0.0024489	0.0058194	0.0000000
LINC01643	0.0000000	0.0000000	0.0000000	0.0000000	0.0000000
LINC02885	0.0036147	0.0078654	0.0084724	0.0027220	0.0193243
HMGXB4	0.5398112	0.5191473	0.5531563	0.4707575	0.5267177
ENSG00000273176	0.0000000	0.0000000	0.0000000	0.0000000	0.0040567
TOM1	0.1590452	0.1408800	0.0968316	0.1570972	0.1232887
HMOX1	0.0322432	0.0264786	0.0253105	0.0361313	0.0361955
MCM5	0.0176071	0.0186928	0.0118805	0.0073960	0.0000000
RASD2	0.0002996	0.0122299	0.0301912	0.0065169	0.0231954
MB	0.0000000	0.0000000	0.0042624	0.0000000	0.0000000
APOL6	0.0159540	0.0118737	0.0056058	0.0000000	0.0132607
APOL5	0.0000000	0.0000000	0.0000000	0.0000000	0.0000000
RBFOX2	0.7986487	1.2324601	1.5519135	0.7350879	1.4043353

ENSG00000279652	0.0000000	0.0000000	0.0000000	0.0000000	0.0050762
ENSG00000279927	0.0000000	0.0000000	0.0026375	0.0000000	0.0060546
ENSG00000279217	0.0012829	0.0000000	0.0000000	0.0040556	0.0000000
APOL3	0.0013237	0.0000000	0.0032093	0.0031494	0.0000000
APOL4	0.0077241	0.0015514	0.0000000	0.0122275	0.0051058
APOL2	0.1356717	0.0557326	0.0475459	0.1537368	0.0862033
APOL1	0.0087874	0.0036121	0.0016515	0.0023928	0.0025600
MYH9	0.3485965	0.2166812	0.1106567	0.3580841	0.2507554
ENSG00000287269	0.0006530	0.0000000	0.0000000	0.0000000	0.0000000
MYH9-DT	0.0022512	0.0017865	0.0031267	0.0020616	0.0039758
ENSG00000228719	0.0016468	0.0014253	0.0027683	0.0026542	0.0000000
TXN2	0.6893649	0.6866364	0.6786862	0.7118510	0.4558917
FOXRED2	0.0604061	0.0728626	0.0749237	0.1306990	0.0601048
EIF3D	0.6331755	0.5431245	0.4232052	0.6136134	0.4595034
ENSG00000229971	0.0421331	0.0302947	0.0182389	0.0587435	0.0336632
CACNG2	0.1493902	0.2937389	0.3293227	0.1255322	0.3385340
CACNG2-DT	0.0380302	0.0601164	0.1113237	0.0503867	0.0532902
IFT27	0.4422579	0.3866616	0.3795180	0.4505858	0.3613039
ENSG00000286485	0.0008588	0.0055046	0.0151030	0.0000000	0.0074936
PVALB	0.0172654	0.0612188	0.0169215	0.0174078	0.0204946
TST	0.0910985	0.0664277	0.0373649	0.1448172	0.0211714
MPST	0.2662737	0.2402313	0.2111712	0.3069306	0.1753249
KCTD17	0.2767248	0.2722874	0.3198604	0.3038947	0.1668814
TMPRSS6	0.0000000	0.0000000	0.0000000	0.0000000	0.0114363
C1QTNF6	0.1314640	0.1020528	0.0829854	0.1262245	0.0868193
SSTR3	0.0031776	0.0013712	0.0007467	0.0030924	0.0000000
RAC2	0.0005892	0.0052663	0.0084465	0.0018691	0.0000000
ENSG00000237862	0.0000000	0.0000000	0.0020129	0.0000000	0.0057021
ELFN2	0.0038886	0.0000000	0.0000000	0.0000000	0.0017206
ELFN2.1	0.0513801	0.0926432	0.1229990	0.0511305	0.1078503
ENSG00000272694	0.0004455	0.0010069	0.0000000	0.0012109	0.0000000
MFNG	0.0149214	0.0080843	0.0015470	0.0030696	0.0000000
CARD10	0.0067365	0.0119270	0.0127354	0.0056460	0.0172720
CDC42EP1	0.2312659	0.1234590	0.0746704	0.3039505	0.0436379
LGALS2	0.0009083	0.0010592	0.0049550	0.0000000	0.0061768
GGA1	0.0950391	0.1047175	0.0851726	0.1035448	0.1280469
SH3BP1	0.0129805	0.0125641	0.0077445	0.0154572	0.0080469
PDXP-DT	0.0012134	0.0037420	0.0010011	0.0089357	0.0076141
PDXP	0.3771503	0.5715325	0.7790107	0.3378898	0.3246445
LGALS1	0.7716386	0.6333689	0.4143504	0.9638822	0.5425541
NOL12	0.1330699	0.1584453	0.1822929	0.1184695	0.1271925
TRIOBP	0.3502809	0.2686117	0.2017255	0.3144942	0.4529930
H1-0	0.6634911	0.8208825	0.8514738	0.5925284	0.6338973
GCAT	0.2444228	0.1780073	0.1363464	0.2908431	0.1179862
GALR3	0.0000000	0.0000000	0.0042492	0.0043470	0.0040262
ANKRD54	0.3162402	0.2297427	0.2562623	0.3296824	0.2577714
EIF3L	1.0889548	1.1841653	1.0415993	1.0282599	0.9281051
ENSG00000279738	0.0507070	0.0620890	0.0562954	0.0480906	0.0636424
MICALL1	0.0806350	0.0748898	0.0660947	0.0848498	0.0766198
C22orf23	0.1690336	0.1367770	0.0913654	0.1668072	0.1047270
ENSG00000272582	0.0085732	0.0137997	0.0033444	0.0187000	0.0251725
POLR2F	0.5094138	0.5533406	0.5917776	0.5052011	0.4609945
SOX10	0.0000000	0.0000000	0.0000000	0.0000000	0.0000000

PICK1	0.0859811	0.1214629	0.1621852	0.0906251	0.1121435
ENSG00000233739	0.0014089	0.0007901	0.0000000	0.0000000	0.0030416
SLC16A8	0.0029050	0.0199630	0.0207523	0.0143993	0.0114018
BAIAP2L2	0.0040498	0.0062686	0.0105226	0.0062555	0.0044670
ENSG00000272720	0.0031843	0.0000000	0.0060811	0.0063558	0.0063982
PLA2G6	0.0701100	0.0425034	0.0378933	0.0428100	0.0647624
ENSG00000279080	0.0000000	0.0038682	0.0020787	0.0012213	0.0055061
MAFF	0.0804437	0.0815684	0.0589893	0.0570373	0.1150051
TMEM184B	0.1700158	0.1459869	0.1242220	0.1477905	0.2054117
CSNK1E	0.9399255	1.0527581	1.1029587	0.9191361	0.8495376
ENSG00000291015	0.1322134	0.0950616	0.0746575	0.0956825	0.1638628
KCNJ4	0.0577967	0.0353505	0.0269575	0.0820360	0.0227875
KDELR3	0.1346476	0.0862233	0.0546518	0.1578430	0.0768205
DDX17	1.3677765	1.2770275	1.3526064	1.3031724	1.3212744
DMC1	0.0103883	0.0046117	0.0062337	0.0088690	0.0214743
FAM227A	0.1051027	0.0819498	0.0832663	0.1378661	0.2302312
CBY1	0.4948985	0.3870281	0.3205388	0.5200439	0.3395951
ENSG00000230912	0.0000000	0.0000000	0.0000000	0.0000000	0.0079782
ENSG00000228274	0.0378748	0.0328725	0.0269445	0.0208056	0.0726885
ENSG00000287219	0.0019386	0.0041707	0.0034525	0.0000000	0.0098882
TOMM22	0.5124452	0.5461795	0.5648069	0.5274754	0.3600902
JOSD1	0.3103207	0.3558497	0.3296093	0.3086702	0.3049656
GTPBP1	0.1305466	0.1636004	0.2049976	0.0843264	0.2032595
SUN2	0.0982629	0.0880853	0.0931508	0.1054264	0.1906686
ENSG00000244491	0.0000000	0.0000000	0.0000000	0.0000000	0.0000000
ENSG00000225450	0.0024308	0.0049884	0.0000000	0.0000000	0.0148958
ENSG00000272669	0.0000000	0.0011620	0.0000000	0.0000000	0.0000000
ENSG00000273076	0.0000000	0.0000000	0.0000000	0.0000000	0.0070221
DNAL4	0.2973386	0.1973141	0.1727021	0.2757115	0.1603807
NPTXR	0.1462823	0.2055094	0.3189800	0.1579001	0.2130455
CBX6	0.4754181	0.4939652	0.5557959	0.4315509	0.5080000
ENSG00000288106	0.0069781	0.0154915	0.0043630	0.0096583	0.0088772
APOBEC3A	0.0000000	0.0000000	0.0000000	0.0026949	0.0000000
APOBEC3B	0.0000000	0.0000000	0.0000000	0.0000000	0.0000000
APOBEC3C	0.0081219	0.0029608	0.0025569	0.0260007	0.0069481
APOBEC3F	0.0256555	0.0175538	0.0059622	0.0188013	0.0115255
ENSG00000289963	0.0000000	0.0000000	0.0000000	0.0000000	0.0053850
APOBEC3G	0.0133332	0.0034682	0.0000000	0.0152603	0.0075813
CBX7	0.0462830	0.0300970	0.0517719	0.0474462	0.0401691
ENSG00000279833	0.0005301	0.0000000	0.0030126	0.0000000	0.0085749
PDGFB	0.0363718	0.0499084	0.1495173	0.0289669	0.0699547
ENSG00000284633	0.0052113	0.0077688	0.0091545	0.0043845	0.0056762
RPL3	3.0711396	2.9024518	2.6262284	2.8607920	2.6468375
SYNGR1	0.1113780	0.1102434	0.2064863	0.0783895	0.2228730
TAB1	0.0872178	0.0837489	0.0628337	0.0821022	0.1473852
MGAT3	0.1132543	0.2731412	0.2701783	0.1099113	0.2968175
MIEF1	0.1764296	0.2341753	0.2268052	0.1798511	0.1915239
ATF4	1.1894384	1.0941049	1.0658913	1.1567625	0.9478286
RPS19BP1	0.6952037	0.6114451	0.6797801	0.5984087	0.4409896
CACNA1I	0.0201828	0.0237610	0.0377730	0.0112514	0.0622802
ENTHD1	0.0012186	0.0012888	0.0000000	0.0000000	0.0000000
GRAP2	0.0000000	0.0000000	0.0000000	0.0000000	0.0000000
TMA7B	0.0091170	0.0080442	0.0175323	0.0098866	0.0041999

FAM83F	0.0021732	0.0000000	0.0014078	0.0000000	0.0000000
TNRC6B-DT	0.0013355	0.0000000	0.0000000	0.0000000	0.0000000
TNRC6B	0.9924921	1.0204718	1.0566955	0.9404798	1.3562276
ADSL	0.2994412	0.2976126	0.2949570	0.3277003	0.2382527
SGSM3	0.1664332	0.1714546	0.1763672	0.2012106	0.1865792
SGSM3-AS1	0.0041387	0.0034477	0.0068424	0.0000000	0.0079111
MRTFA	0.1215587	0.1470390	0.0966234	0.0859401	0.5700727
MRTFA-AS1	0.0010759	0.0008589	0.0000000	0.0000000	0.0028252
ENSG00000289292	0.0000000	0.0000000	0.0000000	0.0000000	0.0000000
MCHR1	0.0037323	0.0142858	0.0195755	0.0160313	0.0161458
SLC25A17	0.2075910	0.1623545	0.1814565	0.1974570	0.2338512
ST13	1.8884159	1.7702163	1.7093124	1.9007028	1.5229316
XPNPEP3	0.2465023	0.2406335	0.2368256	0.2354242	0.2126053
DNAJB7	0.0000000	0.0000000	0.0000000	0.0000000	0.0121056
RBX1	1.3441962	1.3836103	1.4006995	1.3019637	1.1831808
EP300	0.3865557	0.3694471	0.3619192	0.3815131	0.5511514
ENSG00000232754	0.0035957	0.0020627	0.0058093	0.0000000	0.0082403
EP300-AS1	0.0056265	0.0074806	0.0000000	0.0225792	0.0145915
L3MBTL2	0.1147487	0.1325518	0.1189534	0.1255870	0.1176850
L3MBTL2-AS1	0.0213265	0.0205824	0.0056718	0.0182692	0.0314508
CHADL	0.0337796	0.0383202	0.0529944	0.0268272	0.0454180
RANGAP1	0.1650158	0.2353410	0.3204500	0.1753162	0.2406521
ZC3H7B	0.2698557	0.2913757	0.2571978	0.2668422	0.3177990
TEF	0.0331605	0.0257014	0.0228820	0.0232674	0.0478970
TOB2	0.3581543	0.2690294	0.1975480	0.4219626	0.3517007
ACO2	0.6735315	0.6993706	0.6652858	0.6579160	0.5914362
PHF5A	0.3179188	0.3278251	0.3691939	0.3476464	0.2721341
POLR3H	0.3817056	0.3082960	0.3095675	0.3969097	0.2177123
ENSG00000286491	0.0574179	0.0568500	0.0617515	0.0413960	0.0361604
CSDC2	0.1927772	0.1510636	0.1966540	0.2156502	0.1673839
PMM1	0.5452727	0.5147757	0.5035777	0.5220869	0.2937465
DESI1	0.2991695	0.3435393	0.3259857	0.2934355	0.2815466
XRCC6	0.9762017	0.8709515	0.9714589	0.9270604	0.7571720
SNU13	1.1296718	1.2091993	1.3101022	1.0280000	0.9376099
MEI1	0.0016252	0.0000000	0.0029046	0.0036897	0.0124381
CCDC134	0.0366104	0.0399281	0.0186214	0.0561745	0.0778095
SREBF2-AS1	0.0029451	0.0110268	0.0077564	0.0000000	0.0442774
SREBF2	0.2448513	0.3278948	0.3489742	0.1946075	0.5332459
SHISA8	0.0003917	0.0029393	0.0010030	0.0084111	0.0000000
TNFRSF13C	0.0033063	0.0029701	0.0000000	0.0041781	0.0000000
CENPM	0.0136625	0.0109390	0.0169113	0.0394402	0.0143573
SMIM45	0.0172760	0.0233342	0.0494994	0.0340313	0.0134319
SEPTIN3	0.8003139	1.0966383	1.2964487	0.8154095	0.8224873
WBP2NL	0.0083590	0.0344254	0.0178541	0.0192259	0.0218769
NAGA	0.0858102	0.0452061	0.0542296	0.1519933	0.0621177
ENSG00000286659	0.0012277	0.0052212	0.0014422	0.0000000	0.0000000
PHETA2	0.0008223	0.0028536	0.0000000	0.0014695	0.0000000
SMDT1	0.8810550	0.9164671	0.9872313	0.8709329	0.6985434
NDUFA6	0.7722930	0.7664416	0.8386567	0.7706679	0.5909692
ENSG00000270083	0.0022194	0.0059865	0.0042433	0.0000000	0.0000000
NDUFA6-DT	0.0543069	0.0535220	0.0430497	0.0427234	0.1243946
CYP2D6	0.0021513	0.0012859	0.0000000	0.0000000	0.0116522
ENSG00000232710	0.0007556	0.0000000	0.0000000	0.0000000	0.0038965

ENSG00000281538	0.0097064	0.0176138	0.0118990	0.0050161	0.0085927
CYP2D7	0.0053755	0.0008382	0.0012693	0.0000000	0.0135188
TCF20	0.2830053	0.3222737	0.3331186	0.2365548	0.5304389
ENSG00000288719	0.0000000	0.0026321	0.0000000	0.0000000	0.0027951
OGFRP1	0.0008061	0.0009522	0.0000000	0.0000000	0.0035158
LINC01315	0.3806702	0.2936438	0.2484910	0.3881213	0.2644849
NFAM1	0.0005109	0.0042345	0.0009760	0.0000000	0.0000000
ENSG00000230107	0.0000000	0.0008251	0.0012184	0.0000000	0.0000000
RRP7A	0.2553794	0.2262890	0.2334506	0.2238254	0.1534344
SERHL2	0.0161673	0.0051438	0.0053261	0.0163472	0.0033381
RRP7BP	0.0558630	0.0620815	0.0321141	0.0603112	0.1405007
POLDIP3	0.2581263	0.2799632	0.2954004	0.2190663	0.2015412
ENSG00000270022	0.0052782	0.0031401	0.0014078	0.0024238	0.0225247
CYB5R3	0.8162436	0.6867055	0.6609873	0.9282624	0.5417694
ATP5MGL	0.0006834	0.0000000	0.0000000	0.0027252	0.0000000
A4GALT	0.0064972	0.0181399	0.0107311	0.0027880	0.0044531
ARFGAP3	0.7676892	0.5676494	0.6156057	0.7476056	0.5559143
PACSIN2	0.3838624	0.3443184	0.3652441	0.4377572	0.4263829
TTLL1-AS1	0.0044122	0.0119689	0.0024502	0.0027497	0.0084766
TTLL1	0.3376504	0.3331388	0.3282045	0.3422737	0.2758461
BIK	0.0050925	0.0063004	0.0167326	0.0047918	0.0144496
MCAT	0.1664808	0.1251846	0.1787566	0.1755665	0.1317958
TSPO	0.5806163	0.4053697	0.3039242	0.5941160	0.3434916
TTLL12	0.0697292	0.0710279	0.0607464	0.0798988	0.0687848
SCUBE1	0.0605899	0.0393491	0.0480883	0.0711192	0.0976728
MPPED1	0.0114120	0.0053949	0.0293268	0.0000000	0.0031524
EFCAB6-AS1	0.0005909	0.0021756	0.0000000	0.0000000	0.0000000
EFCAB6	0.1018875	0.1018268	0.0494178	0.1124431	0.2842887
EFCAB6-DT	0.0038602	0.0074467	0.0000000	0.0000000	0.0072093
SULT4A1	0.2039836	0.5429140	0.5440164	0.1756221	0.3883096
PNPLA5	0.0000000	0.0000000	0.0000000	0.0000000	0.0000000
PNPLA3	0.0124988	0.0301588	0.0379199	0.0049457	0.0108139
SAMM50	0.3591508	0.3168290	0.2860472	0.3751917	0.2341849
PARVB	0.1558117	0.1310559	0.1558683	0.1625748	0.1695127
ENSG00000279175	0.0000000	0.0000000	0.0000000	0.0000000	0.0033430
ENSG00000280434	0.0004240	0.0025041	0.0000000	0.0024483	0.0064335
PARVG	0.0398514	0.0117739	0.0108536	0.0286222	0.0197087
ENSG00000280011	0.0032413	0.0131169	0.0020357	0.0000000	0.0142895
SHISAL1	0.0952681	0.0917041	0.1666685	0.0706153	0.1033568
RTL6	0.1634994	0.1486597	0.1707116	0.1300090	0.1155858
LINC00229	0.0000000	0.0000000	0.0000000	0.0000000	0.0000000
PRR5	0.0486253	0.1118642	0.1362056	0.0577281	0.1751113
ARHGAP8	0.0385158	0.0648732	0.0828808	0.0257995	0.1933475
PHF21B	0.0483921	0.0757031	0.0505419	0.0515791	0.2118740
NUP50-DT	0.0832260	0.1026335	0.1061634	0.0750615	0.0866140
NUP50	0.2823568	0.2498247	0.3093165	0.2146710	0.3286425
KIAA0930	0.1906959	0.2697607	0.3000488	0.1393256	0.2826152
ENSG00000273287	0.0004930	0.0032906	0.0022206	0.0000000	0.0065398
FAM118A	0.0864232	0.0904504	0.0927633	0.0882739	0.0771639
SMC1B	0.0000000	0.0000000	0.0020287	0.0033765	0.0044670
RIBC2	0.0358353	0.0206862	0.0094217	0.0509562	0.0241067
FBLN1	0.0492499	0.0790473	0.0453471	0.0444545	0.0780550
LINC01589	0.0000000	0.0008916	0.0000000	0.0000000	0.0000000

ENSG00000280383	0.0106211	0.0096790	0.0076736	0.0052087	0.0442668
ATXN10	0.9495544	1.0040932	1.1525698	0.9246001	0.9760803
ENSG00000235091	0.0000000	0.0014491	0.0000000	0.0000000	0.0000000
WNT7B	0.0037485	0.0034301	0.0019791	0.0000000	0.0012783
ENSG00000273145	0.0568681	0.0266109	0.0149437	0.0353566	0.0088004
LINC00899	0.0219440	0.0050941	0.0019983	0.0097001	0.0019688
PRR34-AS1	0.0766539	0.0611475	0.0340936	0.0629207	0.0312874
MIRLET7BHG	0.0045807	0.0046588	0.0080154	0.0146750	0.0294225
LINC02939	0.0256035	0.0229463	0.0220042	0.0402885	0.0326400
PPARA	0.1249583	0.1199170	0.0604457	0.1148859	0.1933111
CDPF1	0.0933637	0.0881136	0.0736156	0.1063884	0.0424622
ENSG00000289858	0.0359597	0.0268797	0.0440600	0.0120545	0.0483469
TTC38	0.0473736	0.0398920	0.0211887	0.0519673	0.0473729
GTSE1-DT	0.0067527	0.0044133	0.0104723	0.0000000	0.0013481
GTSE1	0.0111826	0.0235819	0.0126903	0.0175325	0.0145401
TRMU	0.1457756	0.0990781	0.1210533	0.1243182	0.1556629
CELSR1	0.1481657	0.0815890	0.0534039	0.1838558	0.0907479
ENSG00000288754	0.0246653	0.0076624	0.0056745	0.0191965	0.0165664
LINC02925	0.0129917	0.0040728	0.0000000	0.0195453	0.0142506
GRAMD4	0.1053527	0.1197437	0.0912340	0.1101314	0.1918202
CERK	0.3665768	0.5056645	0.4848501	0.2867692	0.4146907
TBC1D22A-DT	0.0874033	0.1160217	0.1706387	0.1106630	0.1009907
TBC1D22A	0.1801239	0.1903442	0.1578941	0.1730707	0.4654410
EPIC1	0.0009302	0.0000000	0.0011130	0.0000000	0.0107925
MIR3667HG	0.0027667	0.0030565	0.0137409	0.0000000	0.0059985
BRD1	0.1751872	0.2150546	0.2393798	0.2058419	0.2594221
ENSG00000279345	0.0007532	0.0000000	0.0000000	0.0000000	0.0000000
ENSG00000260613	0.0021383	0.0007337	0.0000000	0.0042299	0.0000000
ZBED4	0.1079659	0.1469848	0.1385525	0.0992952	0.2922599
ALG12	0.0849053	0.0327279	0.0385987	0.0998508	0.0386014
ENSG00000273192	0.0000000	0.0028299	0.0010352	0.0000000	0.0000000
CRELD2	0.3182173	0.2127766	0.2196884	0.3544910	0.1825854
PIM3	0.1566014	0.2099251	0.1799743	0.1542733	0.1790526
IL17REL	0.0016506	0.0091343	0.0000000	0.0000000	0.0067635
TTLL8	0.0000000	0.0000000	0.0000000	0.0055683	0.0000000
MLC1	0.5775821	0.3107579	0.1523005	1.0722986	0.2784010
MOV10L1	0.0157237	0.0165767	0.0041900	0.0264908	0.0115158
PANX2	0.0663913	0.0760544	0.0784010	0.0852738	0.0757428
TRABD	0.0617674	0.0381248	0.0428084	0.0756843	0.0481341
ENSG00000273188	0.0032125	0.0139114	0.0025099	0.0073312	0.0026801
TRABD-AS1	0.1195536	0.0864324	0.0766944	0.0979737	0.0451158
SELENOO	0.0696031	0.0796808	0.0543476	0.0808286	0.1682451
SELENOO-AS1	0.0000000	0.0000000	0.0000000	0.0000000	0.0059619
TUBGCP6	0.0417800	0.0278731	0.0402109	0.0240035	0.0894206
ENSG00000288871	0.0030398	0.0000000	0.0059142	0.0038435	0.0015171
HDAC10	0.0557686	0.0773470	0.1084363	0.0633132	0.1234772
MAPK12	0.0942101	0.1269128	0.1692990	0.0990569	0.1447414
MAPK11	0.0305641	0.0545484	0.0677254	0.0337834	0.0747390
PLXNB2	0.1702203	0.1143208	0.0873688	0.3408527	0.1558962
DENND6B	0.1504315	0.1414576	0.1473030	0.1726078	0.2093290
PPP6R2	0.2235064	0.2850893	0.3155403	0.2813887	0.4198761
SBF1	0.2231286	0.3098544	0.2644278	0.1566838	0.3027354
ADM2	0.0424865	0.0228801	0.0062806	0.0308587	0.0344327

MIOX	0.0018843	0.0000000	0.0000000	0.0026207	0.0000000
LMF2	0.1298635	0.0791914	0.0689264	0.1932939	0.0910470
NCAPH2	0.1229996	0.1432999	0.1447272	0.1055752	0.1217793
SCO2	0.1280197	0.1526893	0.1541198	0.1939244	0.0858884
TYMP	0.0452174	0.0342101	0.0200575	0.0215447	0.0235232
ODF3B	0.3517234	0.2451259	0.1338643	0.3259759	0.1729165
KLHDC7B	0.0057362	0.0053076	0.0012960	0.0000000	0.0140192
SYCE3	0.0231824	0.0175051	0.0141364	0.0352090	0.0374349
CPT1B	0.0339758	0.0281279	0.0225512	0.0145050	0.0434638
CHKB	0.1066718	0.1326697	0.1101901	0.1003277	0.1260909
CHKB-DT	0.0726028	0.0989977	0.1177132	0.0519882	0.0640371
ENSG00000272940	0.0008836	0.0013747	0.0000000	0.0048845	0.0000000
MAPK8IP2	0.1278053	0.2756454	0.2603428	0.1237905	0.1832447
ARSA	0.1025110	0.0813995	0.0600885	0.1905909	0.0852759
ENSG00000289244	0.0052855	0.0068598	0.0136878	0.0036838	0.0109582
SHANK3	0.1009714	0.1716519	0.1463610	0.0427957	0.1710709
ACR	0.0049803	0.0095530	0.0058815	0.0000000	0.0022313
RPL23AP7	0.5042135	0.5539547	0.5533744	0.5262086	0.3757858
RABL2B	0.2946033	0.2453745	0.2634518	0.2857947	0.2485352
PLCXD1	0.0541080	0.0970878	0.1188866	0.0491294	0.1119008
GTPBP6	0.2135127	0.2149825	0.2422617	0.2019872	0.1735000
LINC00685	0.0050088	0.0128237	0.0097450	0.0118369	0.0691792
PPP2R3B	0.1930329	0.1635226	0.1444754	0.1555869	0.1652800
ENSG00000281849	0.0006507	0.0012418	0.0000000	0.0000000	0.0000000
ENSG00000280767	0.0000000	0.0000000	0.0000000	0.0000000	0.0000000
SHOX	0.0050542	0.0040712	0.0020015	0.0000000	0.0263281
ENSG00000237531	0.0000000	0.0000000	0.0000000	0.0000000	0.0124680
CRLF2	0.0000000	0.0000000	0.0000000	0.0000000	0.0000000
CSF2RA	0.0013231	0.0036402	0.0034421	0.0000000	0.0000000
IL3RA	0.0030559	0.0110412	0.0058686	0.0143383	0.0957385
SLC25A6	1.5683284	1.6690906	1.5972258	1.5237713	1.4435398
LINC00106	0.0081754	0.0081311	0.0000000	0.0000000	0.0043646
ASMTL-AS1	0.0088482	0.0040454	0.0057912	0.0000000	0.0157871
ASMTL	0.1729570	0.1589675	0.1153429	0.1567447	0.1580364
P2RY8	0.0005880	0.0000000	0.0000000	0.0066051	0.0000000
AKAP17A	0.2301502	0.2310886	0.1922625	0.2020046	0.1726725
ASMT	0.0000000	0.0000000	0.0000000	0.0000000	0.0000000
LINC02968	0.0000000	0.0029571	0.0040216	0.0000000	0.0014182
DHRSX	0.5165437	0.3640119	0.3084440	0.5081448	0.5803244
DHRSX-IT1	0.0012162	0.0007698	0.0000000	0.0000000	0.0039284
ZBED1	0.1553104	0.1899265	0.2080961	0.1910490	0.1703422
ENSG00000289007	0.0776018	0.1061413	0.1762681	0.0959041	0.0825684
CD99P1	0.0327453	0.0275831	0.0274751	0.0127654	0.0446986
CD99	2.2871244	1.7405900	1.3284635	2.6473195	1.6932299
XG	0.0050141	0.0035861	0.0047574	0.0055830	0.0142338
GYG2	0.1310179	0.0784759	0.0940208	0.1263627	0.0989402
ARSD	0.0711827	0.0521009	0.0621020	0.1132680	0.0723566
ARSL	0.0068426	0.0076759	0.0011329	0.0414160	0.0000000
MXRA5	0.0064829	0.0030620	0.0084931	0.0034713	0.0148182
PRKX	0.0648054	0.1045156	0.0977800	0.0602652	0.1215229
PRKX-AS1	0.0020850	0.0000000	0.0000000	0.0000000	0.0046972
ENSG00000285756	0.1090263	0.1327580	0.1745694	0.0598386	0.1425912
ENSG00000286431	0.0065437	0.0067066	0.0044372	0.0019300	0.0016664

LINC03070	0.0013858	0.0039161	0.0020178	0.0000000	0.0045450
NLGN4X	0.2540660	0.3062606	0.3201762	0.3309666	0.6578764
PUDP	0.1572734	0.1228649	0.1208355	0.1389353	0.1147730
STS	0.0746417	0.1008825	0.0626392	0.0608881	0.1718441
PNPLA4	0.1872146	0.1999014	0.1597305	0.1718766	0.1584088
ENSG00000285679	0.0301469	0.0445159	0.0144646	0.0080554	0.1904169
ENSG00000287171	0.0000000	0.0055372	0.0030674	0.0000000	0.0000000
ANOS1	0.0691209	0.0649793	0.1335718	0.1190458	0.1406864
FAM9A	0.0027458	0.0037365	0.0082671	0.0000000	0.0058801
ENSG00000229012	0.0000000	0.0000000	0.0000000	0.0000000	0.0000000
ENSG00000285896	0.0017699	0.0066118	0.0121740	0.0000000	0.0000000
FAM9B	0.0000000	0.0000000	0.0000000	0.0000000	0.0069935
ENSG00000228543	0.0057070	0.0095108	0.0000000	0.0038692	0.0000000
TBL1X	0.6233502	0.3650437	0.2518927	0.5148484	0.5633474
GPR143	0.0350565	0.0259699	0.0216019	0.0307458	0.0512724
SHROOM2	0.0708166	0.1129393	0.2046909	0.0722340	0.1319837
CLDN34	0.0000000	0.0000000	0.0000000	0.0033377	0.0055777
WWC3	0.1420911	0.1045766	0.0890728	0.1907199	0.1326664
WWC3-AS1	0.0000000	0.0000000	0.0019229	0.0000000	0.0026919
CLCN4	0.2005510	0.2530338	0.3676598	0.2361644	0.2794791
ENSG00000227042	0.0024788	0.0020497	0.0000000	0.0000000	0.0099257
MID1	0.1759460	0.0999495	0.0845201	0.2012425	0.1412188
HCCS-DT	0.0231650	0.0163659	0.0158287	0.0240618	0.0720458
HCCS	0.0968337	0.1103809	0.1001244	0.1345036	0.0280521
ARHGAP6	0.3224545	0.1569861	0.1261268	0.2581717	0.4251784
ENSG00000282840	0.0057442	0.0036426	0.0150110	0.0000000	0.0000000
MSL3	0.3023819	0.2815678	0.3133806	0.3169620	0.1999063
FRMPD4	0.1125268	0.1202146	0.1010372	0.0714293	0.5055753
PRPS2	0.0646343	0.0755087	0.0528826	0.0437898	0.0882670
TMSB4X	3.8192856	3.6889202	3.6742122	3.9201577	3.4170266
FAM9C	0.0011483	0.0000000	0.0000000	0.0000000	0.0026227
EGFL6	0.0277724	0.0385535	0.1248713	0.0220401	0.0250152
TCEANC	0.0572565	0.0381211	0.0485089	0.0505405	0.0479790
RAB9A	0.4239847	0.2629856	0.1657215	0.5067608	0.2066017
TRAPPC2	0.1768198	0.2313479	0.2513474	0.1429269	0.1451895
OFD1	0.1913406	0.1746386	0.1671752	0.1398095	0.3231780
GPM6B	1.7825616	1.3389440	1.0976369	2.3811608	1.3403813
ENSG00000289449	0.0189798	0.0196498	0.0147298	0.0630738	0.0307980
ENSG00000233535	0.0000000	0.0000000	0.0000000	0.0046019	0.0000000
ENSG00000212663	0.0017117	0.0018667	0.0000000	0.0000000	0.0000000
GEMIN8	0.1956475	0.1649378	0.1311070	0.2397165	0.1841330
GLRA2	0.1615449	0.2937754	0.4515321	0.1143226	0.4587764
FANCB	0.0118267	0.0271678	0.0161150	0.0147769	0.0140990
MOSPD2	0.1971382	0.1537451	0.1272284	0.1951154	0.1309980
ASB11	0.0013404	0.0000000	0.0000000	0.0000000	0.0000000
PIGA	0.0347455	0.0399514	0.0444788	0.0343927	0.0268668
VEGFD	0.0065570	0.0034940	0.0000000	0.0057846	0.0172803
PIR	0.5352090	0.3343587	0.2026621	0.6371063	0.3521956
BMX	0.0023712	0.0000000	0.0017517	0.0000000	0.0027453
ACE2	0.0014705	0.0000000	0.0019149	0.0000000	0.0000000
CLTRN	0.0255613	0.0237933	0.0132072	0.0178306	0.0370274
CA5BP1	0.0572984	0.0886166	0.0702906	0.0593897	0.0834958
CA5B	0.0858303	0.0772706	0.0406150	0.0656745	0.1138778

INE2	0.0052499	0.0068229	0.0049305	0.0000000	0.0083169
ZRSR2	0.1870311	0.2168803	0.1705626	0.1355072	0.2437000
AP1S2	0.5716179	0.8436229	1.1245313	0.4562351	0.6118528
GRPR	0.0007858	0.0003809	0.0040797	0.0000000	0.0000000
ENSG00000238178	0.0000000	0.0021407	0.0032841	0.0000000	0.0000000
CTPS2	0.1357028	0.1283557	0.1029450	0.1409222	0.1629490
SYAP1	0.8363835	0.7787433	0.7522265	0.9248490	0.6174662
TXLNG	0.2716898	0.1876780	0.1849567	0.2566691	0.2899368
RBBP7	0.8002679	0.7195506	0.7508976	0.7676579	0.5708528
REPS2	0.1136257	0.0952072	0.0976171	0.0607108	0.1789452
NHS	0.2930502	0.2748155	0.3153184	0.2834655	0.6647432
ENSG00000235834	0.0000000	0.0000000	0.0000000	0.0000000	0.0000000
NHS-AS1	0.0000000	0.0000000	0.0000000	0.0000000	0.0062307
SCML1	0.1182876	0.1692818	0.1365960	0.0836254	0.1429891
RAI2	0.0433560	0.0746279	0.0831037	0.0243845	0.1033027
LINC01456	0.0346539	0.0566451	0.0399277	0.0317621	0.1734226
BEND2	0.0009117	0.0011043	0.0000000	0.0000000	0.0000000
SCML2	0.0788565	0.0819944	0.0900813	0.0718262	0.1359485
CDKL5	0.2657349	0.3215053	0.3349669	0.2399765	0.5276480
RS1	0.0030388	0.0006946	0.0022570	0.0029331	0.0052853
PPEF1	0.0025467	0.0079320	0.0022062	0.0125137	0.0117637
PHKA2-AS1	0.0000000	0.0000000	0.0000000	0.0000000	0.0000000
PHKA2	0.1017713	0.0632900	0.0573763	0.0617549	0.1111505
ENSG00000286724	0.0093996	0.0014386	0.0000000	0.0019653	0.0044683
ADGRG2	0.0291537	0.0533547	0.0255427	0.0390966	0.0160918
PDHA1	0.3842598	0.5462386	0.6125288	0.3939950	0.3562681
MAP3K15	0.0098783	0.0096761	0.0108511	0.0044362	0.0071943
SH3KBP1	0.5923231	0.6329991	0.5527371	0.5477755	0.7167729
BCLAF3	0.1734718	0.1919629	0.1456804	0.1816708	0.2755475
MAP7D2	0.1999882	0.4709474	0.5522327	0.2088209	0.4131823
EIF1AX	1.3144068	1.4258822	1.4273246	1.2961524	1.1802011
RPS6KA3	0.3512099	0.2777563	0.2743729	0.3189232	0.4463511
ENSG00000283380	0.0063993	0.0013447	0.0000000	0.0036275	0.0044278
CNKSR2	0.1870157	0.2453267	0.2613521	0.1798302	0.6049509
KLHL34	0.0000000	0.0000000	0.0000000	0.0014497	0.0000000
MBTPS2	0.2115600	0.1721838	0.1973829	0.1949947	0.1999185
YY2	0.0027662	0.0074118	0.0036515	0.0020616	0.0063180
SMS	1.0451217	1.3900852	1.5310062	1.0403030	1.0578350
PHEX	0.1365231	0.0883489	0.0419828	0.1744970	0.0491796
PHEX-AS1	0.0104641	0.0104987	0.0072033	0.0099258	0.0128704
PTCHD1-AS	0.0126855	0.0229904	0.0081092	0.0147741	0.0542746
ENSG00000289084	0.1239079	0.1790222	0.0821958	0.1214653	0.5886505
ENSG00000237019	0.0008158	0.0030181	0.0034440	0.0071088	0.0107495
PTCHD1	0.1426785	0.1877252	0.1050666	0.0841827	0.2517248
PRDX4	0.6975955	0.5172757	0.4868760	0.8646772	0.5292079
ACOT9	0.1173752	0.1065766	0.0566869	0.1320379	0.0701016
ENSG00000233785	0.0000000	0.0000000	0.0000000	0.0000000	0.0000000
SAT1	0.7539948	0.5611894	0.4240565	0.9170918	0.5660420
APOO	0.2973882	0.3562360	0.3884313	0.2836841	0.2308689
CXorf58	0.0051688	0.0120468	0.0091210	0.0000000	0.0014245
KLHL15	0.0717149	0.0640099	0.0625199	0.0501779	0.0811926
EIF2S3	0.9340257	0.8759581	0.7353348	0.9285088	0.7353485
ZFX-AS1	0.0006394	0.0030443	0.0000000	0.0000000	0.0046818

ZFX	0.2234793	0.1499707	0.1439969	0.2187213	0.2367153
ENSG00000289472	0.0022233	0.0044386	0.0000000	0.0000000	0.0165286
PDK3	0.2025071	0.1832097	0.2101125	0.2259181	0.3265161
PCYT1B	0.1039492	0.1066725	0.0696915	0.1828050	0.0592427
POLA1	0.1522073	0.0814988	0.1453668	0.1570979	0.3067457
ARX	0.6893794	0.3834142	0.2315211	0.6613127	0.3325435
ENSG00000242021	0.0000000	0.0014712	0.0000000	0.0000000	0.0000000
ENSG00000223742	0.0050353	0.0014407	0.0022732	0.0000000	0.0000000
IL1RAPL1	0.3372840	0.3687273	0.2947664	0.2540238	1.3331183
ENSG00000237994	0.0008025	0.0000000	0.0000000	0.0047682	0.0025831
NR0B1	0.0014514	0.0020079	0.0101676	0.0000000	0.0044415
GK	0.0917405	0.0917080	0.0945963	0.1201026	0.1018390
ENSG00000241886	0.0015884	0.0012699	0.0000000	0.0000000	0.0000000
GK-AS1	0.0000000	0.0009971	0.0000000	0.0000000	0.0053380
TAB3	0.1069199	0.1636828	0.1781866	0.0938291	0.1832065
DMD	0.6933364	0.6464034	0.5697772	0.6493971	1.3238010
ENSG00000285900	0.0000000	0.0018647	0.0000000	0.0000000	0.0000000
ENSG00000289215	0.0000000	0.0010802	0.0017927	0.0000000	0.0028494
ENSG00000233928	0.0054451	0.0058002	0.0036063	0.0069920	0.0093992
ENSG00000233571	0.0040602	0.0000000	0.0000000	0.0000000	0.0131062
TMEM47	1.2251456	0.7813188	0.5743197	1.3947336	0.6616129
CFAP47	0.1647348	0.1256098	0.0792903	0.1107797	0.1976909
ENSG00000226484	0.0003365	0.0000000	0.0000000	0.0000000	0.0000000
ENSG00000286475	0.0068635	0.0033084	0.0044428	0.0235313	0.0243943
ENSG00000290955	0.0191937	0.0296924	0.0456015	0.0147075	0.0581094
PRRG1	0.0571635	0.1059561	0.0950176	0.0912399	0.1963132
LANCL3	0.0384227	0.1106036	0.1004914	0.0366435	0.0768868
XK	0.0197689	0.0410095	0.0643153	0.0216031	0.0271252
CYBB	0.0040538	0.0027228	0.0055589	0.0052846	0.0000000
DYNLT3	0.5159081	0.6900848	0.6235780	0.5290549	0.5339411
ENSG00000259977	0.0000000	0.0010426	0.0000000	0.0000000	0.0073347
SYTL5	0.0026887	0.0000000	0.0020549	0.0000000	0.0030832
SRPX	0.0132972	0.0136197	0.0136712	0.0036072	0.0307320
ENSG00000226679	0.0018848	0.0078002	0.0007135	0.0000000	0.0000000
RPGR	0.3357535	0.1940538	0.1587063	0.3374477	0.1884535
OTC	0.0008572	0.0011521	0.0000000	0.0014353	0.0000000
TSPAN7	0.4949813	0.7273252	1.0463666	0.6526992	0.8047580
ENSG00000235806	0.0033197	0.0033491	0.0000000	0.0000000	0.0000000
ENSG00000287729	0.0034113	0.0014991	0.0014111	0.0000000	0.0060675
MID1IP1	0.4770683	0.3114728	0.2246062	0.4915833	0.2288280
ENSG00000289127	0.0000000	0.0028646	0.0057423	0.0000000	0.0029283
ENSG00000224610	0.0045643	0.0000000	0.0000000	0.0000000	0.0000000
LINC03053	0.0039280	0.0004666	0.0000000	0.0000000	0.0030787
BCOR	0.1763095	0.1782934	0.2147045	0.1499081	0.2233987
ENSG00000288856	0.0000000	0.0047396	0.0000000	0.0104508	0.0000000
ENSG00000236393	0.0000000	0.0000000	0.0000000	0.0000000	0.0114670
ATP6AP2	0.7397945	0.7082815	0.9405540	1.1988792	0.6252923
CXorf38	0.1871307	0.1409637	0.1215764	0.1894096	0.1616578
MED14	0.3088968	0.2154720	0.1926515	0.2770508	0.2214848
MED14OS	0.0234341	0.0191445	0.0107372	0.0172800	0.0093468
ENSG00000287549	0.0000000	0.0003706	0.0000000	0.0020815	0.0000000
USP9X	0.5408786	0.5042687	0.5003812	0.4650251	0.6342029
LINC02601	0.0000000	0.0000000	0.0011261	0.0000000	0.0000000

DDX3X	0.8071427	0.7102093	0.8114455	0.8851002	0.7232864
NYX	0.0000000	0.0000000	0.0000000	0.0000000	0.0019716
CASK	0.6327633	0.7125178	0.8867850	0.5788223	1.1148193
GPR34	0.0019384	0.0012480	0.0000000	0.0053775	0.0000000
GPR82	0.0000000	0.0000000	0.0000000	0.0041394	0.0062724
ENSG00000231772	0.0000000	0.0000000	0.0015819	0.0000000	0.0141924
MAOA	0.4460905	0.2932727	0.3651694	0.3997345	0.2734348
MAOB	0.8135942	0.4895461	0.5046283	0.9484907	0.4323946
NDP	0.0038203	0.0018046	0.0097495	0.0047981	0.0120984
NDP-AS1	0.0005648	0.0011324	0.0032795	0.0000000	0.0000000
EFHC2	0.1931327	0.1025120	0.0693852	0.2864198	0.0642203
FUNDC1	0.3444780	0.3518157	0.3382200	0.4060323	0.2326281
KDM6A	0.1389379	0.1374524	0.0850246	0.1464326	0.4185928
MIR222HG	0.0021559	0.0014800	0.0000000	0.0050900	0.0115061
LINC02595	0.0011392	0.0000000	0.0034747	0.0000000	0.0014182
LINC01186	0.0000000	0.0025831	0.0000000	0.0000000	0.0052465
KRBOX4	0.1255917	0.1445456	0.1334373	0.1099784	0.1658444
ZNF674	0.0300333	0.0375473	0.0573350	0.0274017	0.0742202
ZNF674-AS1	0.0133374	0.0222204	0.0193438	0.0173905	0.0184611
CHST7	0.0364619	0.0652707	0.0626312	0.0411240	0.0343290
SLC9A7	0.1226828	0.2143767	0.2861754	0.0524489	0.3456168
RP2	0.0809586	0.0626879	0.0642944	0.1008571	0.0674727
LINC01545	0.0032481	0.0000000	0.0018244	0.0000000	0.0000000
JADE3	0.0393291	0.0421874	0.0648030	0.0270229	0.1445857
RGN	0.0126266	0.0087122	0.0093696	0.0015536	0.0171445
ENSG00000288759	0.0079284	0.0047693	0.0046967	0.0000000	0.0000000
NDUFB11	1.0706587	1.1802756	1.2554651	1.0275545	0.9359574
RBM10	0.2041831	0.1818898	0.1832461	0.1900496	0.2472433
UBA1	0.3585352	0.3111389	0.3874264	0.3075482	0.2471594
INE1	0.0005236	0.0000000	0.0000000	0.0028594	0.0000000
CDK16	0.4514904	0.5214982	0.4992223	0.4135766	0.3530580
USP11	0.8238189	0.8358414	0.8840513	0.8431899	0.7821523
ZNF157	0.0183754	0.0126550	0.0124128	0.0036651	0.0303373
ZNF41	0.0521034	0.0588696	0.0436384	0.0639875	0.0725835
LINC01560	0.0334562	0.0369921	0.0393086	0.0198616	0.0257307
ENSG00000289890	0.0057210	0.0157432	0.0155631	0.0072796	0.0169209
ARAF	0.1381119	0.0966133	0.0787052	0.1141913	0.0867006
SYN1	0.1473142	0.2989546	0.4047341	0.1126361	0.3052815
TIMP1	0.8579265	0.5954813	0.4012161	0.9476291	0.4883444
CFP	0.0025957	0.0118018	0.0145021	0.0040247	0.0000000
ELK1	0.0981417	0.0640380	0.1039276	0.0962617	0.0728718
UXT	0.6070308	0.5913433	0.5503564	0.5905692	0.3647915
UXT-AS1	0.0080353	0.0091346	0.0088366	0.0109679	0.0100178
ZNF81	0.0805452	0.0837679	0.0645088	0.0910432	0.1219834
ZNF182	0.0458030	0.0720835	0.0415348	0.0252528	0.0509684
ZNF630	0.0223103	0.0269231	0.0217530	0.0193482	0.0241129
ZNF630-AS1	0.0007434	0.0000000	0.0000000	0.0000000	0.0000000
ENSG00000287757	0.0125334	0.0049747	0.0000000	0.0000000	0.0049154
SLC38A5	0.0016171	0.0000000	0.0042684	0.0000000	0.0000000
FTSJ1	0.1176088	0.1401218	0.1444811	0.1385082	0.1112448
PORCN-DT	0.0032211	0.0043939	0.0000000	0.0043682	0.0064366
PORCN	0.0727347	0.0817113	0.1090850	0.0708816	0.1039576
EBP	0.1432865	0.1937131	0.2321983	0.1543728	0.1371952

TBC1D25	0.0660575	0.0678796	0.0916477	0.0637711	0.1229133
ENSG00000204620	0.0033538	0.0024730	0.0074754	0.0000000	0.0085167
RBM3	0.4054741	0.5340694	0.4340342	0.4094865	0.4113251
WDR13	0.4529134	0.3726840	0.3631288	0.4450208	0.3631788
WAS	0.0071772	0.0056219	0.0033907	0.0040882	0.0000000
SUV39H1	0.0096418	0.0023148	0.0050175	0.0127769	0.0187284
ENSG00000232828	0.0075114	0.0023740	0.0107891	0.0021110	0.0654822
HDAC6	0.2102303	0.2421281	0.3098180	0.1877209	0.2750677
ERAS	0.0100772	0.0114309	0.0221717	0.0075464	0.0094207
PCSK1N	1.8690525	1.9851677	2.1070839	1.9044534	1.7437159
PQBP1	0.2927082	0.3415551	0.2838607	0.2816288	0.2388033
TIMM17B	0.5030988	0.5489282	0.6490870	0.4981002	0.4028082
SLC35A2	0.1190029	0.1082670	0.0735378	0.1631806	0.0880915
PIM2	0.0440242	0.0397147	0.0354550	0.0296931	0.0342733
OTUD5	0.2825224	0.2709927	0.2679975	0.2568302	0.2714352
ENSG00000288908	0.0063503	0.0043385	0.0034419	0.0023569	0.0000000
KCND1	0.0032802	0.0034260	0.0087293	0.0000000	0.0136701
GRIPAP1	0.3567772	0.4198132	0.4580800	0.3275460	0.5472920
TFE3	0.1370982	0.1132505	0.0939248	0.1157076	0.1245994
CCDC120	0.0350098	0.0243004	0.0481853	0.0178250	0.0460589
PRAF2	0.5042343	0.5603805	0.6311572	0.5298295	0.4749106
WDR45	0.2230111	0.1902996	0.1740033	0.1743822	0.1516871
GPKOW	0.1376191	0.1301354	0.1321570	0.0871799	0.0944072
ENSG00000289245	0.0027396	0.0027512	0.0035142	0.0000000	0.0046088
MAGIX	0.0389397	0.0262305	0.0405656	0.0347800	0.0209183
PLP2	0.2345212	0.1456650	0.1156120	0.2099003	0.1477546
PRICKLE3	0.0150556	0.0110047	0.0045594	0.0068570	0.0070493
SYP	0.2238467	0.4846459	0.7127422	0.2065824	0.5686043
SYP-AS1	0.0000000	0.0000000	0.0000000	0.0000000	0.0000000
CACNA1F	0.0000000	0.0000000	0.0000000	0.0000000	0.0000000
CCDC22	0.0619754	0.0532306	0.0476827	0.0531944	0.0598631
FOXP3	0.0009612	0.0000000	0.0009863	0.0019339	0.0000000
ENSG00000286181	0.0000000	0.0012926	0.0048378	0.0035690	0.0000000
PPP1R3F	0.2034761	0.1843129	0.2031563	0.1919958	0.1806946
ENSG00000270012	0.0000000	0.0000000	0.0000000	0.0000000	0.0048241
ENSG00000286031	0.0000000	0.0009942	0.0000000	0.0030470	0.0000000
GAGE10	0.0027316	0.0000000	0.0000000	0.0031460	0.0044810
USP27X-DT	0.0141115	0.0107246	0.0084582	0.0208293	0.0025260
USP27X	0.1382985	0.1273642	0.1689646	0.1081245	0.0515284
CLCN5	0.1435671	0.1088669	0.1048491	0.1518899	0.2238023
CCNB3	0.0064926	0.0087677	0.0144091	0.0018880	0.0061965
DGKK	0.0458033	0.1095606	0.1536106	0.0464742	0.1131144
SHROOM4	0.0057003	0.0219340	0.0074288	0.0076927	0.0357554
LINC01284	0.0017343	0.0104472	0.0122070	0.0016253	0.0378394
ENSG00000229151	0.0042235	0.0015626	0.0070307	0.0032755	0.0341220
NUDT10	0.0946650	0.1626686	0.2291994	0.0985840	0.0905996
ENSG00000226530	0.0590292	0.0561020	0.1079820	0.0384937	0.0721423
EZHIP	0.0000000	0.0000000	0.0012558	0.0034405	0.0000000
NUDT11	0.2583455	0.3608985	0.5720010	0.2713992	0.2793804
CENPVL3	0.0397309	0.0782984	0.1227886	0.0537032	0.0400981
GSPT2	0.3789698	0.3859380	0.4396003	0.4425484	0.3040443
MAGED1	1.3696342	1.1800546	1.3515094	1.4996353	1.1835544
ENSG00000279750	0.0007299	0.0006848	0.0164006	0.0000000	0.0000000

MAGED4B	0.0000000	0.0000000	0.0022509	0.0000000	0.0000000
MAGED4	0.0039512	0.0000000	0.0013432	0.0070451	0.0035970
ENSG00000287215	0.0160562	0.0269683	0.0550186	0.0202574	0.0437979
ENSG00000286118	0.0022932	0.0000000	0.0038243	0.0000000	0.0025988
FAM156A	0.0088398	0.0233015	0.0165685	0.0111768	0.0486091
GPR173	0.1357530	0.2156214	0.1897783	0.1330408	0.3443645
TSPYL2	0.2370625	0.2857448	0.3713970	0.1969128	0.2639490
KANTR	0.1512781	0.2712009	0.2515947	0.1099023	0.3442828
KDM5C	0.1951995	0.2323208	0.2414204	0.2038043	0.2908877
KDM5C-IT1	0.0010743	0.0010829	0.0000000	0.0000000	0.0000000
IQSEC2	0.0486828	0.0636347	0.0887499	0.0799413	0.0805040
SMC1A	0.5257675	0.4658504	0.4530271	0.4777307	0.5355453
RIBC1	0.1923345	0.1021442	0.0514460	0.1968738	0.0861328
HSD17B10	0.3488856	0.3239873	0.2863974	0.4310819	0.2392971
HUWE1	0.7102047	0.7712691	0.8444667	0.6419222	0.8768980
PHF8	0.0997465	0.0745540	0.0676687	0.0745956	0.2039321
FAM120C	0.1302242	0.0808738	0.0743514	0.1628260	0.1351360
WNK3	0.4323756	0.5858223	0.8218127	0.4323942	0.5719225
TSR2	0.5091949	0.4527649	0.4742464	0.4564499	0.3480312
FGD1	0.0911838	0.1442644	0.1571777	0.0776733	0.1860716
GNL3L	0.2213807	0.1879790	0.1699022	0.1879459	0.1995440
ITIH6	0.0000000	0.0003706	0.0025201	0.0000000	0.0000000
MAGED2	1.1453718	1.1280697	1.2322718	1.2603597	0.8998201
TRO	0.2059938	0.2909474	0.2983322	0.2181320	0.3257557
PFKFB1	0.0053752	0.0018111	0.0094312	0.0000000	0.0298848
APEX2	0.0691014	0.0388304	0.0496455	0.0770516	0.0452184
FAM104B	0.2794638	0.2635333	0.2612555	0.2925178	0.1475924
MAGEH1	0.5769734	0.4525289	0.6272538	0.7370969	0.3869387
USP51	0.1096034	0.1430726	0.1144323	0.1508036	0.0735399
ENSG00000288739	0.0028275	0.0056650	0.0012490	0.0112792	0.0067778
RRAGB	0.3608121	0.4404781	0.5979545	0.3698901	0.3752708
ENSG00000227486	0.1040113	0.1160224	0.0781033	0.0866655	0.4454590
KLF8	0.0301406	0.0617353	0.0582420	0.0383142	0.0727891
UBQLN2	0.3989184	0.4224924	0.5963756	0.3794353	0.3234151
ENSG00000230105	0.0035589	0.0160333	0.0143049	0.0131222	0.0069796
NBDY	0.9114515	0.8803263	1.0532241	0.8749318	0.7209418
SPIN3	0.1112886	0.1578077	0.1713753	0.0755263	0.1556903
SPIN2B	0.1246306	0.1700440	0.1740407	0.1401354	0.1214852
ENSG00000226310	0.0069206	0.0131284	0.0095966	0.0097556	0.0497061
SPIN2A	0.0036477	0.0021838	0.0059913	0.0085021	0.0098812
ENSG00000286977	0.0113150	0.0067006	0.0074187	0.0048838	0.0036522
FAAH2	0.0494978	0.0640029	0.0629662	0.0395067	0.1836091
ZXDB	0.0400679	0.0681134	0.0609702	0.0372593	0.0659609
ZXDA	0.0245667	0.0205688	0.0493589	0.0201211	0.0351377
LINC01278	0.2330895	0.2448945	0.2129358	0.2179381	0.3823455
SPIN4	0.0787753	0.1335311	0.1682431	0.0596290	0.0931028
SPIN4-AS1	0.0027428	0.0000000	0.0015180	0.0000000	0.0000000
ARHGEF9	0.3705824	0.5735271	0.7475930	0.4289704	0.6798918
AMER1	0.0441847	0.0747398	0.1277176	0.0286699	0.0736149
ENSG00000287370	0.0006326	0.0000000	0.0000000	0.0000000	0.0088634
ASB12	0.0004141	0.0022552	0.0000000	0.0000000	0.0000000
MTMR8	0.0180182	0.0312296	0.0166417	0.0063989	0.0980665
ZC4H2	0.1652359	0.2022180	0.2406651	0.1221027	0.3682757

ZC3H12B	0.2481614	0.2624452	0.2031355	0.1694479	0.9479745
LAS1L	0.1356588	0.1073248	0.1102542	0.1140442	0.2163777
MSN	0.6522423	0.4655891	0.3195469	0.7161195	0.3846889
HEPH	0.0433773	0.0398287	0.0286253	0.0451238	0.0728427
EDA2R	0.0447061	0.0423595	0.0313664	0.1057979	0.0322968
AR	0.1897146	0.0891431	0.0633911	0.2219814	0.0853407
OPHN1	0.0965612	0.1887828	0.1711278	0.1296850	0.2942108
ENSG00000260118	0.0009632	0.0022617	0.0000000	0.0000000	0.0000000
ENSG00000289038	0.0087679	0.0021867	0.0110884	0.0008779	0.0000000
YIPF6	0.3240816	0.2699782	0.2669397	0.4141469	0.2902728
STARD8	0.0024842	0.0026017	0.0022732	0.0000000	0.0058440
EFNB1	0.1797745	0.1324786	0.0649441	0.2303318	0.1534114
PJA1	0.3994913	0.5012570	0.6875184	0.3471501	0.3633215
LINC00269	0.0007210	0.0020747	0.0000000	0.0000000	0.0109560
NALF2	0.0377916	0.0796058	0.0816327	0.0293812	0.0479017
EDA	0.0950227	0.0663690	0.0438345	0.0605117	0.1469550
OTUD6A	0.0000000	0.0016149	0.0000000	0.0000000	0.0000000
IGBP1	0.5439517	0.5918602	0.5141696	0.5104463	0.4482768
DGAT2L6	0.0000000	0.0000000	0.0000000	0.0000000	0.0044670
ARR3	0.0000000	0.0000000	0.0000000	0.0000000	0.0045939
PDZD11	0.5199895	0.5269754	0.5595661	0.5776943	0.3540248
KIF4A	0.0288087	0.0277009	0.0329234	0.0878754	0.0223911
GDPD2	0.1196499	0.0812394	0.0403481	0.3807704	0.1097137
ENSG00000284391	0.0049387	0.0032511	0.0020724	0.0000000	0.0035288
DLG3	0.1723371	0.2087172	0.2078030	0.1295219	0.2618757
TEX11	0.0042869	0.0055454	0.0052215	0.0000000	0.0019139
SLC7A3	0.0553974	0.0455769	0.0936750	0.0664693	0.0619031
SNX12	0.1327207	0.1743986	0.1631606	0.1175408	0.0948855
FOXO4	0.0090739	0.0107475	0.0012831	0.0021274	0.0137191
MED12	0.0638798	0.0668697	0.0615025	0.0785656	0.0444528
NLGN3	0.0927362	0.1063408	0.1443744	0.1963966	0.1839004
ENSG00000228427	0.0009517	0.0013822	0.0000000	0.0000000	0.0059869
ZMYM3	0.2195126	0.2059767	0.2070125	0.2369926	0.2030402
NONO	1.1741546	0.9678267	0.9468050	1.1083692	0.8677578
ITGB1BP2	0.0000000	0.0009224	0.0000000	0.0000000	0.0000000
TAF1	0.2727377	0.2923553	0.2815101	0.2314846	0.3859071
OGT	0.2676453	0.2710100	0.2606927	0.2430854	0.4516717
GCNA	0.0176289	0.0167798	0.0179413	0.0135265	0.0861236
NHSL2	0.0983512	0.1279206	0.1489732	0.0700816	0.1828247
RTL5	0.1176208	0.1395860	0.1465843	0.1171256	0.1400457
PIN4	0.5613713	0.5997289	0.6390557	0.5087399	0.4965463
ERCC6L	0.0018339	0.0000000	0.0022858	0.0152923	0.0029782
RPS4X	2.6758119	2.6484501	2.4537566	2.5551066	2.3197705
CITED1	0.0258711	0.0355893	0.0528596	0.0094703	0.0342659
HDAC8	0.2345204	0.2295369	0.2254125	0.1593617	0.4183198
PHKA1	0.0716789	0.0494334	0.0410275	0.0351554	0.0741141
DMRTC1B	0.0039257	0.0050303	0.0000000	0.0043537	0.0000000
FAM226B	0.0384319	0.0849214	0.0908385	0.0321328	0.0621217
DMRTC1	0.0022502	0.0000000	0.0022111	0.0000000	0.0000000
PABPC1L2B	0.0116343	0.0236276	0.0575930	0.0043173	0.0105470
PABPC1L2A	0.0146523	0.0278074	0.0492619	0.0173055	0.0596571
NAP1L2	0.2840684	0.4865484	0.7425545	0.2886106	0.3750095
CHIC1	0.1421765	0.1710018	0.1586743	0.1588717	0.2566483

TSIX	0.0021572	0.0000000	0.0000000	0.0000000	0.0086813
XIST	0.0012103	0.0000000	0.0000000	0.0000000	0.0000000
FTX	0.5255777	0.6517041	0.5160528	0.4160477	1.7309190
JPX	0.4734894	0.4715547	0.4629254	0.4684259	0.5923282
ENSG00000271533	0.0186493	0.0203738	0.0198901	0.0138384	0.1942990
SLC16A2	0.1921748	0.1752610	0.2033979	0.1670032	0.1957170
RLIM	0.4341472	0.5141197	0.5412487	0.4247349	0.5288697
NEXMIF	0.2193918	0.4253488	0.6541480	0.1351955	0.7004809
ABCB7	0.2209172	0.1684733	0.1774243	0.1761014	0.1652666
UPRT	0.0924250	0.1138804	0.1393790	0.1129849	0.0900896
ZDHHC15	0.1374639	0.1246982	0.1352798	0.1649269	0.2688051
MAGEE2	0.0056577	0.0074830	0.0325160	0.0121214	0.0032430
PBDC1	0.3910017	0.3533383	0.4808543	0.3386700	0.3630640
MAGEE1	0.0858281	0.1651547	0.2356489	0.0699200	0.1170203
MIR325HG	0.2119912	0.2542839	0.2587025	0.1420719	0.9450991
FGF16	0.0008655	0.0011956	0.0016489	0.0000000	0.0000000
ATRX	1.2059300	1.2916624	1.4423818	1.1155963	1.3850951
MAGT1	0.4106184	0.2258788	0.1681289	0.7527266	0.2459512
COX7B	1.1893743	1.3418048	1.4618931	1.1940547	1.0348857
ATP7A	0.0784048	0.0934312	0.1053255	0.1263535	0.1252751
PGK1	1.7561583	1.7384151	1.8248608	1.6639564	1.4723092
PGAM4	0.0023587	0.0042678	0.0031097	0.0000000	0.0030865
TAF9B	0.1380767	0.1384204	0.1136954	0.1492540	0.1237792
CYSLTR1	0.0025783	0.0014875	0.0000000	0.0000000	0.0021172
LPAR4	0.0572421	0.0339333	0.0186928	0.1252018	0.0380216
ITM2A	0.0098451	0.0065504	0.0089918	0.0339797	0.0000000
TENT5D	0.0000000	0.0000000	0.0000000	0.0000000	0.0000000
BRWD3	0.1905394	0.2156450	0.2126472	0.1522637	0.3012044
ENSG00000289132	0.0230933	0.0217076	0.0168798	0.0126313	0.0206390
HMGN5	0.3129757	0.2400828	0.2093244	0.1929668	0.2275024
SH3BGRL	1.0175730	1.0534267	0.9795907	1.1830124	0.9035673
ENSG00000288059	0.0000000	0.0020532	0.0099277	0.0000000	0.0214033
POU3F4	0.1068774	0.0456002	0.0286704	0.1808486	0.0730302
CYLC1	0.0017612	0.0000000	0.0000000	0.0000000	0.0000000
RPS6KA6	0.1144660	0.1834209	0.1927444	0.0850857	0.3441576
HDX	0.1293846	0.1407476	0.1662704	0.0936071	0.3123006
APOOL	0.4096205	0.4468378	0.3849335	0.3509908	0.2520106
SATL1	0.0742608	0.1179184	0.1185990	0.0692234	0.1034318
ZNF711	0.3460398	0.5181414	0.5653530	0.3397609	0.5151639
POF1B	0.0020970	0.0000000	0.0000000	0.0000000	0.0000000
CHM	0.2232072	0.2096474	0.1539258	0.1624987	0.3593177
DACH2	0.0835255	0.1338375	0.1788469	0.0587414	0.5466583
KLHL4	0.0089429	0.0046745	0.0083043	0.0056366	0.0320044
PABPC5-AS1	0.0020477	0.0000000	0.0026406	0.0026302	0.0000000
PABPC5	0.0863821	0.1051348	0.1507883	0.1021977	0.0810278
PCDH11X	0.1983660	0.3308082	0.2785536	0.1802814	0.7826948
ENSG00000289479	0.0000000	0.0000000	0.0000000	0.0000000	0.0000000
NAP1L3	0.6098708	0.8996089	1.2448450	0.5542898	0.6886965
FAM133A	0.0386379	0.0828716	0.1197592	0.0128843	0.0706150
ENSG00000286523	0.0013355	0.0016424	0.0000000	0.0000000	0.0000000
DIAPH2	0.1076843	0.0801965	0.0620602	0.0961390	0.2266999
RPA4	0.0006530	0.0000000	0.0000000	0.0000000	0.0000000
DIAPH2-AS1	0.0002817	0.0011064	0.0000000	0.0000000	0.0180980

LINC03077	0.0708288	0.0680850	0.0745752	0.0578023	0.5034086
PCDH19	0.0475776	0.0875266	0.1577767	0.0689498	0.1715184
TSPAN6	0.5369863	0.4114730	0.3614642	0.8457753	0.3516321
SRPX2	0.0091528	0.0013669	0.0013454	0.0134999	0.0046563
SYTL4	0.0418142	0.0241225	0.0148290	0.0538816	0.0024307
CSTF2	0.0802828	0.0740832	0.0680491	0.0830089	0.0548214
NOX1	0.0007138	0.0021368	0.0041974	0.0000000	0.0000000
XKRX	0.0013404	0.0000000	0.0000000	0.0000000	0.0000000
ARL13A	0.0000000	0.0010932	0.0000000	0.0000000	0.0044810
TRMT2B	0.0478826	0.0427453	0.0240890	0.0292013	0.0369561
TRMT2B-AS1	0.0013404	0.0000000	0.0000000	0.0000000	0.0000000
TMEM35A	0.5417130	0.8873135	1.3945919	0.4131827	0.6988797
CENPI	0.0117149	0.0143563	0.0155222	0.0176990	0.0000000
DRP2	0.0235116	0.0783417	0.0801575	0.0265752	0.1182624
TAF7L	0.0017401	0.0020163	0.0000000	0.0000000	0.0000000
TIMM8A	0.1002581	0.0907262	0.0830616	0.1468122	0.0831036
BTK	0.0013404	0.0033972	0.0018681	0.0000000	0.0064448
RPL36A	2.4721902	2.4967332	2.3150755	2.3571219	2.1971443
GLA	0.0669362	0.0535822	0.0260493	0.1008211	0.0361901
HNRNPH2	0.5399022	0.4949658	0.5853035	0.5455640	0.4173940
ARMCX4	0.1059466	0.1732816	0.1346889	0.1303905	0.2859823
ARMCX1	0.4500457	0.4822677	0.5512342	0.5029332	0.3577327
ARMCX6	0.0351991	0.0283093	0.0228044	0.0187544	0.0400484
ARMCX3	0.9107458	0.9488909	0.9167949	0.8675838	0.7543028
ARMCX3-AS1	0.0023094	0.0000000	0.0069306	0.0029375	0.0202067
ENSG00000261101	0.0023939	0.0087247	0.0025528	0.0000000	0.0158174
ENSG00000286000	0.0010608	0.0034847	0.0000000	0.0056678	0.0000000
ARMCX2	0.3196171	0.3645697	0.3815803	0.3740469	0.2208886
ENSG00000290798	0.0024725	0.0010376	0.0000000	0.0000000	0.0015531
ZMAT1	0.1195321	0.1141027	0.0782047	0.0859931	0.2150512
TCEAL2	0.9413118	1.1378505	1.3103480	0.9018488	0.8514767
TCEAL6	0.1988345	0.3703099	0.6655253	0.1939504	0.2341716
ENSG00000286794	0.0245332	0.0520472	0.0882711	0.0306335	0.0612069
BEX5	0.5615234	0.9093479	1.1426437	0.4263535	0.5625577
TMSB15A	0.4857429	0.9216598	1.2598922	0.4556804	0.6805368
ARMCX5	0.0828794	0.0743283	0.1327438	0.0538310	0.1166367
ARMCX5-GPRASP2	0.1492686	0.2292378	0.2875360	0.1035663	0.3153913
ENSG00000270050	0.0054056	0.0071901	0.0013511	0.0000000	0.0107244
GPRASP1	0.2038727	0.2760263	0.3523561	0.2027672	0.3331480
ENSG00000286327	0.0000000	0.0039266	0.0000000	0.0000000	0.0030106
GPRASP2	0.2206402	0.2835635	0.3703411	0.1957320	0.2873014
ARMCX5-GPRASP2.1	0.0045356	0.0052181	0.0083292	0.0023036	0.0070137
GPRASP3	0.0069798	0.0170818	0.0212486	0.0302505	0.0071570
LINC00630	0.0594441	0.0574039	0.0543547	0.0514458	0.1824649
ENSG00000239407	0.0408528	0.0667645	0.0584530	0.0603940	0.0992756
RAB40AL	0.0015779	0.0000000	0.0000000	0.0000000	0.0000000
ENSG00000286846	0.0035138	0.0000000	0.0000000	0.0000000	0.0054128
BEX1	1.2131314	1.7124015	2.0724578	1.2000148	1.2977093
BEX4	1.1516755	1.1983926	1.4226275	1.1505300	1.0073032
TCEAL8	0.8892890	0.7879526	0.6727102	0.8306760	0.5876169
TCEAL5	0.3325734	0.5393745	0.7231335	0.3262026	0.3656381
BEX2	1.1061601	1.3054472	1.6205217	0.9605569	0.9976350
TCEAL7	0.8338770	1.1905650	1.3541336	0.8309902	0.9013873

TCEAL9	0.8831894	0.6441110	0.5160886	0.8586738	0.5840059
BEX3	2.0328054	2.0251592	2.1370693	2.0303536	1.7194695
ENSG00000281091	0.0053535	0.0094119	0.0113826	0.0033299	0.0111713
ENSG00000234405	0.0116916	0.0232594	0.0203440	0.0124118	0.0383394
RAB40A	0.0089899	0.0089045	0.0128680	0.0033311	0.0051512
ENSG00000287619	0.0073215	0.0069676	0.0015528	0.0145043	0.0032941
TCEAL4	1.6019132	1.4920412	1.5427631	1.5245616	1.2490246
TCEAL3	1.0044512	0.9387565	0.9393318	0.9618936	0.7628369
TCEAL1	0.4099465	0.3427607	0.3584465	0.3748221	0.2954453
MORF4L2	1.5363503	1.4531785	1.6091934	1.4627931	1.3624986
MORF4L2-AS1	0.0069142	0.0064415	0.0066242	0.0000000	0.0262997
TMEM31	0.0089865	0.0143527	0.0140599	0.0087021	0.0000000
PLP1	0.0567555	0.0359445	0.0084358	0.2409965	0.0752178
RAB9B	0.1623887	0.2632217	0.3739282	0.1539107	0.2445487
TMSB15B-AS1	0.0573860	0.0769327	0.0678693	0.0365281	0.1940570
TMSB15B	0.0313672	0.0626056	0.0763745	0.0292439	0.0513725
H2BW2	0.0035978	0.0050579	0.0027084	0.0010246	0.0000000
TMSB15C	0.0450596	0.0729671	0.0903702	0.0415554	0.0907239
SLC25A53	0.1925476	0.2202244	0.1973660	0.2282068	0.2174496
ZCCHC18	0.0776715	0.0662145	0.1220065	0.0931095	0.0733278
FAM199X	0.4924043	0.4707697	0.4897567	0.5090957	0.3955005
IL1RAPL2	0.0308931	0.0406851	0.0162822	0.0246527	0.1891471
NRK	0.0053285	0.0030317	0.0011658	0.0000000	0.0000000
PWWP3B	0.0105378	0.0189233	0.0096011	0.0052065	0.0155291
RADX	0.0950202	0.0837798	0.1934651	0.0642654	0.1129533
RNF128	0.0509489	0.0465914	0.0377280	0.0608402	0.0767974
TBC1D8B	0.0152018	0.0124810	0.0101362	0.0192266	0.0028694
MORC4	0.2396265	0.1114796	0.0672240	0.2809490	0.1764076
CLDN2	0.0000000	0.0000000	0.0000000	0.0000000	0.0043076
RBM41	0.1312525	0.1834074	0.1958824	0.1655525	0.3171361
NUP62CL	0.0929963	0.0610122	0.0454347	0.0805505	0.0638981
DNAAF6	0.0454061	0.0187161	0.0207139	0.0447440	0.0152328
FRMPD3	0.0464715	0.0994149	0.1006284	0.0550686	0.2802641
FRMPD3-AS1	0.0000000	0.0000000	0.0067844	0.0000000	0.0080130
PRPS1	0.1960217	0.2328275	0.3053404	0.2129080	0.1992739
TSC22D3	0.5556703	0.6867183	0.7209853	0.5476091	0.5661893
MID2	0.1435234	0.1550167	0.1726764	0.0804949	0.1760136
ENSG00000236064	0.0019437	0.0072762	0.0074468	0.0000000	0.0218533
VSIG1	0.0026910	0.0052967	0.0032093	0.0000000	0.0000000
PSMD10	0.5145383	0.4382184	0.5759528	0.5053536	0.3947574
ATG4A	0.0755378	0.0701877	0.0448935	0.0613164	0.0523515
COL4A6	0.0386276	0.0252318	0.0067745	0.0715603	0.0530461
COL4A5	0.1340540	0.0777559	0.0514197	0.2636485	0.1716116
IRS4	0.0055697	0.0031666	0.0039297	0.0028123	0.0000000
IRS4-AS1	0.0014254	0.0023647	0.0016034	0.0063474	0.0000000
GUCY2F	0.0017830	0.0000000	0.0000000	0.0000000	0.0025260
NXT2	0.1126122	0.0697715	0.0397171	0.1818530	0.0473117
KCNE5	0.0020737	0.0000000	0.0015874	0.0065515	0.0000000
ACSL4	0.4154477	0.5819186	0.7767429	0.3550007	0.6540159
ENSG00000289365	0.0000000	0.0000000	0.0000000	0.0000000	0.0154591
TMEM164	0.0694023	0.0690363	0.0777897	0.0593356	0.1515041
AMMECR1	0.0228218	0.0241590	0.0322545	0.0291822	0.0860451
GNG5B	0.0014860	0.0000000	0.0000000	0.0000000	0.0000000

RTL9	0.0111728	0.0231724	0.0131909	0.0000000	0.0466869
ENSG00000287685	0.0000000	0.0000000	0.0000000	0.0000000	0.0000000
TDGF1P3	0.0000000	0.0000000	0.0000000	0.0000000	0.0000000
CHRDL1	0.1214126	0.0659525	0.0398839	0.1649714	0.0700842
PAK3	0.7366456	1.2084729	1.5794870	0.5997021	1.2705119
CAPN6	0.0049185	0.0000000	0.0000000	0.0000000	0.0048241
DCX	1.1318058	1.9109740	2.0623821	0.9612139	1.6317285
SERTM2	0.0009418	0.0010776	0.0033827	0.0063825	0.0000000
ALG13	0.2585561	0.2158460	0.2053125	0.2572555	0.3335240
ALG13-AS1	0.0049447	0.0037888	0.0025416	0.0029675	0.0145113
TRPC5	0.0147413	0.0172166	0.0163576	0.0172577	0.0805158
TRPC5OS	0.0020621	0.0000000	0.0000000	0.0000000	0.0000000
RTL4	0.0027164	0.0019886	0.0000000	0.0000000	0.0022770
LHFPL1	0.0073421	0.0013749	0.0109472	0.0049403	0.0075290
AMOT	0.1265779	0.0845681	0.0738341	0.1315678	0.0839252
ENSG00000286072	0.0020395	0.0076910	0.0022351	0.0000000	0.0064608
XACT	0.0104796	0.0084775	0.0000000	0.0030679	0.0206422
HTR2C	0.0056463	0.0091802	0.0077496	0.0000000	0.0217900
IL13RA2	0.1081362	0.0662957	0.0665910	0.1512378	0.0429503
LRCH2	0.3350033	0.3069182	0.3755815	0.2797398	0.4338926
PLS3-AS1	0.0000000	0.0038378	0.0019113	0.0000000	0.0044670
PLS3	0.3540156	0.2089489	0.1092063	0.3248402	0.2307008
DANT2	0.1348852	0.1477954	0.1109954	0.0968449	0.6561302
DANT1	0.0000000	0.0004474	0.0000000	0.0000000	0.0000000
KLHL13	0.2301315	0.3639910	0.5462452	0.2436128	0.3509365
WDR44	0.1940569	0.2711333	0.2418583	0.2066710	0.2846581
DOCK11	0.1073153	0.1587030	0.1143482	0.0721405	0.3150669
IL13RA1	0.0644435	0.0357571	0.0341043	0.0680303	0.0469697
ZCCHC12	0.1414310	0.3486762	0.6521845	0.1253431	0.2157502
LINC01285	0.0065253	0.0140959	0.0075673	0.0050414	0.0052949
LONRF3	0.0015494	0.0107261	0.0071384	0.0092237	0.0072583
PGRMC1	0.9147545	0.9941788	1.5231213	1.0375662	0.9900366
ENSG00000226661	0.0010561	0.0004257	0.0008971	0.0043019	0.0060804
SLC25A43	0.0242662	0.0242809	0.0128624	0.0151716	0.0454448
SLC25A5-AS1	0.0099635	0.0135830	0.0281386	0.0168886	0.0000000
SLC25A5	0.8722013	0.9522097	0.8066491	0.8640859	0.7828844
UBE2A	0.5458480	0.5549340	0.5829363	0.5737912	0.4593479
STEEP1	0.0920005	0.1008522	0.1178379	0.0730829	0.0753951
NKRF	0.1358643	0.1532812	0.2094520	0.1101174	0.1791059
ENSG00000289864	0.0000000	0.0000000	0.0000000	0.0000000	0.0000000
SEPTIN6	0.3548068	0.6413126	0.8439008	0.4194407	0.5707636
SOWAHD	0.0005904	0.0042854	0.0011924	0.0000000	0.0000000
RPL39	3.1586035	3.1946473	2.9850461	3.0826499	2.8903113
ENSG00000286366	0.0209622	0.0189737	0.0079564	0.0190744	0.0232069
UPF3B	0.3465629	0.3297296	0.4379507	0.3666551	0.2898713
RNF113A	0.2244800	0.1807208	0.2016149	0.1772766	0.1198819
NDUFA1	1.2250489	1.3070130	1.3149906	1.2589249	1.0436220
AKAP14	0.0236780	0.0249743	0.0092414	0.0598617	0.0210126
NKAP	0.6000462	0.5326557	0.5261885	0.5717507	0.4728322
RHOXF1P3	0.0049042	0.0041857	0.0023834	0.0103562	0.0076232
RHOXF1-AS1	0.0025556	0.0010327	0.0013034	0.0000000	0.0096126
RHOXF1	0.0533548	0.0617610	0.0487827	0.0782785	0.2251606
ZBTB33	0.1795453	0.1477202	0.2120304	0.1926254	0.1599280

TMEM255A	0.0610227	0.1191274	0.1450639	0.0588699	0.1951790
LAMP2	0.7572435	0.5354827	0.4554569	1.2084522	0.5042840
CUL4B	0.4752663	0.4593644	0.5347270	0.4364743	0.4403001
MCTS1	0.4559385	0.5516720	0.6335998	0.4470797	0.4281914
C1GALT1C1	0.1407126	0.1098755	0.1420140	0.3094729	0.0819703
GLUD2	0.0405092	0.0174439	0.0192677	0.0340527	0.0091824
ENSG00000286163	0.0159519	0.0112634	0.0012539	0.0079139	0.0163405
ENSG00000260585	0.0020844	0.0017348	0.0000000	0.0000000	0.0040780
GRIA3	0.1053344	0.1116391	0.1380811	0.1339338	0.2544645
THOC2	0.5644335	0.4777028	0.3960100	0.5717912	0.7107207
XIAP	0.6128113	0.6791724	0.7948262	0.5062485	0.5568867
STAG2-AS1	0.0552554	0.0279752	0.0177969	0.0582792	0.0495953
STAG2	0.8354638	0.5801960	0.5113209	0.7536887	0.5855290
TENM1	0.1756733	0.1925498	0.2721769	0.1200056	0.7163819
DCAF12L2	0.0049305	0.0132125	0.0416850	0.0000000	0.0143946
PRR32	0.0000000	0.0000000	0.0000000	0.0024587	0.0000000
ENSG00000225689	0.0375520	0.0351480	0.0119170	0.0000000	0.0787897
SMARCA1	0.9576571	0.7838723	0.7265011	0.9302648	0.6795677
OCRL	0.2480819	0.2997410	0.3844940	0.2257843	0.2697895
APLN	0.0210404	0.0167550	0.0077302	0.0072601	0.0242572
ENSG00000287024	0.0410702	0.0255181	0.0107456	0.0374246	0.0114785
ZDHHC9	0.1756345	0.1312589	0.1803758	0.1734135	0.1525863
ENSG00000235189	0.0034797	0.0031002	0.0027814	0.0000000	0.0246638
UTP14A	0.1163943	0.1237302	0.1064387	0.1417678	0.1291990
BCORL1	0.0590256	0.0896384	0.1054644	0.0430341	0.1083900
ELF4	0.0180770	0.0132119	0.0042898	0.0150068	0.0030787
AIFM1	0.1785464	0.2116259	0.2559741	0.1935315	0.1625139
RAB33A	0.1306694	0.2634954	0.3552095	0.1419851	0.1736227
ZNF280C	0.0993995	0.0991500	0.0792438	0.0863836	0.1504575
SLC25A14	0.1027133	0.1160504	0.1463207	0.1311596	0.1206397
RBMX2	0.2969429	0.2262084	0.2743075	0.2862381	0.1834665
ENSG00000290740	0.0039898	0.0187796	0.0177740	0.0041277	0.0342421
ENSG00000287160	0.0000000	0.0006960	0.0000000	0.0000000	0.0000000
ENOX2	0.1292462	0.1942747	0.1449790	0.0769065	0.1876126
LINC01201	0.0613899	0.1949629	0.0960196	0.0580206	0.1275737
ARHGAP36	0.0908813	0.2331404	0.0994063	0.0522453	0.1736930
IGSF1	0.0502123	0.0949470	0.0800337	0.0563091	0.2412805
ENSG00000287806	0.0000000	0.0016833	0.0000000	0.0000000	0.0000000
ENSG00000286060	0.0087644	0.0020443	0.0018539	0.0015041	0.0052497
FIRRE	0.0249637	0.0257598	0.0115222	0.0116540	0.1050367
STK26	0.0304986	0.0119536	0.0227424	0.0102367	0.0147177
RAP2C	0.1637118	0.1086083	0.1034282	0.1254941	0.0841138
RAP2C-AS1	0.1487987	0.1069610	0.0819557	0.1244897	0.1152272
MBNL3	0.0096405	0.0100680	0.0015991	0.0063404	0.0037236
HS6ST2	0.2711733	0.2359113	0.2665826	0.3833491	0.3586280
GPC4	0.1151886	0.0525776	0.0374438	0.2679782	0.0469529
GPC3	0.0397725	0.0369099	0.0679167	0.0494006	0.0929206
CCDC160	0.0674304	0.0847855	0.1678390	0.0780972	0.0555511
PHF6	0.6039613	0.6212290	0.7777371	0.7183058	0.5507750
HPRT1	0.3320323	0.5412113	0.6552810	0.2290582	0.5072509
MIR503HG	0.0121658	0.0022176	0.0013632	0.0000000	0.0132371
LINC00629	0.0252785	0.0194349	0.0089539	0.0266919	0.0102841
PLAC1	0.0187979	0.0098215	0.0052731	0.0180054	0.0186709

PABIR2	0.1139524	0.1970211	0.2604858	0.0718486	0.1745893
PABIR3	0.0771253	0.0606291	0.0464822	0.0645195	0.0791442
MOSPD1	0.1012834	0.0876941	0.1280189	0.1016876	0.0679197
SMIM10	0.1778897	0.1148715	0.0797845	0.2127889	0.0820219
RTL8B	0.1659053	0.1345156	0.1150288	0.1988390	0.1038654
RTL8C	1.2651378	1.1966358	1.2125577	1.2992606	0.9474106
ENSG00000286964	0.0059312	0.0133340	0.0053082	0.0029905	0.0018081
RTL8A	0.7831013	0.6988524	0.6312410	0.7588016	0.4938131
SMIM10L2B-AS1	0.0000000	0.0008052	0.0029250	0.0063344	0.0104691
SMIM10L2B	0.0971582	0.1521954	0.1967283	0.0686690	0.1220920
CT55	0.0000000	0.0000000	0.0000000	0.0000000	0.0000000
ZNF75D	0.0449703	0.0803366	0.0645000	0.0562625	0.0739593
ZNF449	0.0563224	0.0531598	0.0338633	0.0629687	0.0692806
SMIM10L2A	0.1524845	0.2184078	0.2900273	0.1570855	0.1069023
INTS6L	0.1058767	0.0777067	0.0567384	0.0790620	0.2371995
MMGT1	0.3723780	0.4371398	0.4183368	0.4037127	0.3083757
SLC9A6	0.1624640	0.2473627	0.3238190	0.2015255	0.2350098
FHL1	1.0575735	1.0025245	1.0175453	1.3822695	0.8272459
MAP7D3	0.2739565	0.1029038	0.0847907	0.3654904	0.1206364
HTATSF1	1.0084054	0.9948554	1.1379240	0.8692072	0.7699420
ARHGEF6	0.0475406	0.0323330	0.0171473	0.0937466	0.0315358
ENSG00000232611	0.0529525	0.0782174	0.0821046	0.0467727	0.1559548
RBMX	0.7616155	0.7946018	0.8102578	0.7192924	0.7413445
ENSG00000291054	0.0431081	0.0594713	0.0513196	0.0783995	0.1550100
GPR101	0.0318092	0.0176655	0.0047914	0.1255282	0.0000000
ZIC3	0.0005780	0.0000000	0.0093739	0.0000000	0.0000000
ENSG00000283692	0.0019570	0.0013042	0.0000000	0.0000000	0.0060051
FGF13	0.4550669	0.7076050	0.9979677	0.4161945	1.4920719
FGF13-AS1	0.0148651	0.0034122	0.0024028	0.0160669	0.0035909
MCF2	0.0332589	0.0595243	0.0676472	0.0360435	0.0766436
ATP11C	0.0963944	0.1382767	0.1520732	0.0581303	0.2987361
ENSG00000230707	0.0105257	0.0190624	0.0236356	0.0047194	0.0077228
SOX3	0.0253484	0.0213318	0.0067561	0.0172011	0.0214722
LINC00632	1.7315133	1.8772354	1.5485512	1.4591375	2.0187238
CDR1	0.1573606	0.1797686	0.1151753	0.1087759	0.1377788
LDOC1	0.4155860	0.5238700	0.6287164	0.4593272	0.4719608
SPANXA2-OT1	0.0229131	0.0269997	0.0109330	0.0000000	0.0806207
MAGEC3	0.0011795	0.0006384	0.0045697	0.0036457	0.0214258
ENSG00000288098	0.0027412	0.0007600	0.0013851	0.0000000	0.0062902
ENSG00000227303	0.0000000	0.0014613	0.0000000	0.0000000	0.0000000
SLITRK4	0.1906983	0.3532194	0.4199635	0.1896212	0.3480567
UBE2NL	0.0009750	0.0060134	0.0023362	0.0000000	0.0000000
ENSG00000286085	0.0015315	0.0000000	0.0000000	0.0014048	0.0000000
SLITRK2	0.0447217	0.1006223	0.0382011	0.0416035	0.1197191
ENSG00000284377	0.0007824	0.0000000	0.0000000	0.0000000	0.0000000
ENSG00000227083	0.0010592	0.0000000	0.0000000	0.0000000	0.0000000
FMR1-AS1	0.0008426	0.0019932	0.0000000	0.0000000	0.0012623
FMR1	0.3339589	0.3129972	0.3422053	0.2909804	0.3646905
FMR1-IT1	0.0000000	0.0000000	0.0000000	0.0000000	0.0000000
FMR1NB	0.0000000	0.0000000	0.0000000	0.0000000	0.0000000
AFF2	0.2255219	0.3859665	0.5986288	0.1540547	0.8248260
AFF2-IT1	0.0000000	0.0000000	0.0000000	0.0000000	0.0016028
IDS	1.3333206	1.5816164	1.7048922	1.2577952	1.2566058

ENSG00000238039	0.0018905	0.0085440	0.0013856	0.0058800	0.0109395
EOLA1-DT	0.0220257	0.0253054	0.0131496	0.0139748	0.0416942
EOLA1	0.0550392	0.0605527	0.0890098	0.0462766	0.0608351
TMEM185A	0.0981369	0.0874267	0.1027711	0.1336028	0.1296495
LINC00850	0.0000000	0.0000000	0.0000000	0.0000000	0.0000000
EOLA2	0.0562554	0.0844424	0.0682369	0.0257978	0.0551961
EOLA2-DT	0.0679248	0.0605578	0.0319562	0.0336886	0.1714695
MAMLD1	0.0685781	0.0796297	0.1035333	0.0455840	0.1675937
MTM1	0.1284870	0.0819471	0.0693193	0.1605120	0.1606060
MTMR1	0.1388388	0.0918875	0.1271737	0.1102453	0.1738600
CD99L2	0.2588919	0.2466461	0.2844007	0.3176000	0.3062988
HMGB3	0.4801615	0.7027015	0.9976205	0.4295683	0.4454346
ENSG00000287918	0.0093466	0.0178102	0.0092098	0.0089007	0.0263207
VMA21	0.6799182	0.6965877	0.8355764	0.7125810	0.5140687
PRRG3	0.0172444	0.0251836	0.0239964	0.0200745	0.0165045
GABRE	0.0206426	0.0133838	0.0255807	0.0153145	0.0025761
ENSG00000231937	0.0023452	0.0000000	0.0000000	0.0024525	0.0103530
GABRA3	0.0971472	0.2136550	0.3311371	0.0786859	0.3821841
MAGEA3-DT	0.0000000	0.0018122	0.0000000	0.0000000	0.0000000
GABRQ	0.0078407	0.0074956	0.0208998	0.0092500	0.0094201
CETN2	1.5260448	1.1435915	1.0051528	1.5767227	1.0794649
NSDHL	0.1133412	0.1014201	0.1077363	0.1589258	0.0759489
ZNF185	0.0997960	0.0624600	0.0467345	0.0800674	0.0767530
PNMA5	0.0163738	0.0236337	0.0781772	0.0103162	0.0807741
PNMA3	0.0689925	0.1034246	0.1584971	0.0731033	0.0827940
PNMA6A	0.1249408	0.1649426	0.2065020	0.1163184	0.1107522
PNMA6F	0.0065402	0.0136915	0.0387167	0.0053617	0.0340540
ZNF275	0.1081587	0.0920475	0.1041253	0.0879442	0.0990844
ZFP92	0.0000000	0.0023354	0.0065335	0.0021445	0.0047864
HAUS7	0.0493812	0.0471891	0.0504908	0.0409018	0.0376161
BGN	0.0222235	0.0237134	0.0140692	0.0341780	0.0346081
ATP2B3	0.0375458	0.0706199	0.0908963	0.0315628	0.1293396
CCNQ	0.1496684	0.1842808	0.2571577	0.1673434	0.1129460
DUSP9	0.0133152	0.0416902	0.0412064	0.0064410	0.0091421
PNCK	0.1746358	0.2486570	0.3518997	0.1622843	0.3039199
SLC6A8	0.1804260	0.1643546	0.1642854	0.2293505	0.1773285
BCAP31	0.7435862	0.6566383	0.6602773	0.9205193	0.5179289
ABCD1	0.0336246	0.0165283	0.0083029	0.0429116	0.0185874
PLXNB3-AS1	0.0204605	0.0188863	0.0090361	0.0307836	0.0159942
PLXNB3	0.0000000	0.0000000	0.0021636	0.0000000	0.0000000
SRPK3	0.0062304	0.0046342	0.0000000	0.0000000	0.0000000
IDH3G	0.3611242	0.3473634	0.3967803	0.3431610	0.2469311
SSR4	1.1173139	0.9603112	0.8995033	1.1227022	0.8615283
PDZD4	0.1946459	0.2846463	0.3417606	0.1594733	0.3588794
L1CAM	0.1932724	0.3639579	0.5023017	0.1444678	0.5042595
L1CAM-AS1	0.0000000	0.0028954	0.0000000	0.0000000	0.0021475
ARHGAP4	0.0084415	0.0053708	0.0067083	0.0120213	0.0233255
NAA10	0.4235891	0.4653628	0.5157292	0.4529684	0.3980951
RENBP	0.0547719	0.0239845	0.0071718	0.0697541	0.0342242
HCFC1	0.1860427	0.2023784	0.1939562	0.2115038	0.1900751
HCFC1-AS1	0.0026008	0.0016836	0.0025433	0.0031805	0.0000000
TMEM187	0.0731730	0.0401577	0.0324384	0.0864565	0.0596211
IRAK1	0.2078468	0.1554024	0.1530749	0.1596637	0.1535616

MECP2	0.4653351	0.4765588	0.5624206	0.4429235	0.5758970
TKTL1	0.0010109	0.0019300	0.0021401	0.0172131	0.0098978
FLNA	0.5519215	0.3199551	0.1666962	0.6183452	0.3937473
ENSG00000285018	0.0014499	0.0000000	0.0000000	0.0025754	0.0000000
EMD	1.1119925	0.9582846	0.8757154	1.0963380	0.8642564
RPL10	3.2115460	3.1480069	2.9619500	3.0755368	2.8845070
ENSG00000280195	0.0076454	0.0113167	0.0045833	0.0045740	0.0036382
DNASE1L1	0.1133790	0.0757816	0.0735552	0.1100336	0.0662957
TAFAZZIN	0.0544123	0.0616152	0.0469720	0.0496169	0.0777410
ATP6AP1-DT	0.1060464	0.1067501	0.0740204	0.1215744	0.0491081
ATP6AP1	0.7043430	0.6247149	0.7219193	1.0775002	0.5364398
GDI1	1.0686049	1.0933755	1.2825159	1.1068428	0.9504691
FAM50A	0.5664336	0.5326829	0.5896283	0.5961774	0.5526114
PLXNA3	0.0870515	0.0708259	0.0758890	0.1029865	0.1442401
LAGE3	0.8052817	0.7521333	0.7640709	0.7416344	0.5072340
UBL4A	0.2011701	0.1759252	0.2323136	0.1967248	0.1477714
SLC10A3	0.0781729	0.0875839	0.0621221	0.0973991	0.0788387
FAM3A	0.2772456	0.2445587	0.2378478	0.3418201	0.1379192
G6PD	0.2159783	0.1841595	0.1786916	0.1971632	0.1778063
IKBKG	0.0577783	0.0751832	0.0710824	0.0699035	0.0625471
GAB3	0.0000000	0.0013605	0.0000000	0.0000000	0.0066963
DKC1	0.3075901	0.2903242	0.2547270	0.3200301	0.2695112
MPP1	0.0687160	0.1436705	0.1981905	0.0531817	0.1057057
F8	0.0364887	0.0386556	0.0290983	0.0407822	0.0387901
F8A1	0.0892934	0.1377169	0.1845395	0.1284574	0.0455342
FUNDC2	0.5866749	0.5367509	0.5293497	0.5197431	0.4513170
CMC4	0.3244904	0.3800945	0.3178516	0.3805940	0.2039457
MTCP1	0.0021075	0.0019478	0.0024265	0.0031040	0.0414902
BRCC3	0.2283741	0.2841478	0.3653954	0.2740928	0.2220863
VBP1	0.8684539	0.9160591	1.1713861	0.9347450	0.6853738
RAB39B	0.2262067	0.2964096	0.3425099	0.2068889	0.1677353
CLIC2	0.0093749	0.0054387	0.0135942	0.0188343	0.0450628
ENSG00000224216	0.0008718	0.0007331	0.0000000	0.0000000	0.0045621
TMLHE-AS1	0.0072643	0.0034325	0.0000000	0.0011622	0.0401700
TMLHE	0.1443077	0.1126199	0.1154092	0.1529795	0.1891120
SPRY3	0.0263474	0.0291559	0.0226467	0.0229375	0.1074366
VAMP7	0.1917041	0.1527934	0.2268658	0.1927492	0.1507285
WASH6P	0.0532300	0.0593397	0.0597801	0.0352065	0.0514883
SRY	0.0106621	0.0022593	0.0047469	0.0177654	0.0078907
ENSG00000286130	0.0000000	0.0009796	0.0000000	0.0000000	0.0028917
RPS4Y1	1.6059779	1.5963005	1.4417238	1.5545685	1.3803994
ENSG00000278847	0.0025225	0.0101182	0.0143973	0.0060272	0.0085442
ZFY	0.1128667	0.0651029	0.0892948	0.0855516	0.1360860
ZFY-AS1	0.0000000	0.0020545	0.0000000	0.0018086	0.0031524
LINC00278	0.0000000	0.0000000	0.0000000	0.0000000	0.0000000
PCDH11Y	0.1183441	0.1726127	0.1521835	0.1052403	0.3869623
AMELY	0.0000000	0.0000000	0.0009943	0.0029286	0.0000000
TBL1Y	0.0760753	0.0520008	0.0525663	0.0666557	0.0461869
ENSG00000273906	0.0136765	0.0054527	0.0085581	0.0054299	0.0064366
ENSG00000289826	0.0013082	0.0036375	0.0000000	0.0014906	0.0057673
ENSG00000291032	0.0000000	0.0000000	0.0000000	0.0000000	0.0000000
ENSG00000291034	0.0558889	0.1125202	0.1341676	0.0612056	0.2671432
ENSG00000290435	0.0000000	0.0000000	0.0000000	0.0000000	0.0000000

USP9Y	0.5212523	0.6632669	0.7721930	0.4144742	0.8498912
ENSG00000286009	0.0875428	0.0781361	0.1423512	0.0618159	0.0677927
DDX3Y	0.6255823	0.4964051	0.4749783	0.5695233	0.5107914
UTY	0.2458361	0.2201769	0.1500772	0.2084399	0.5618511
TMSB4Y	0.0270604	0.0287910	0.0417784	0.0093085	0.0244232
ENSG00000290853	0.0886988	0.0772397	0.0884560	0.0704813	0.3477989
NLGN4Y	0.2530302	0.2938453	0.3472627	0.2949911	0.8952260
NLGN4Y-AS1	0.0000000	0.0000000	0.0000000	0.0000000	0.0027158
ENSG00000286201	0.0003405	0.0012489	0.0000000	0.0041632	0.0039758
FAM224B	0.0000000	0.0000000	0.0000000	0.0000000	0.0000000
FAM224A	0.0005478	0.0011581	0.0000000	0.0020943	0.0063697
TTTY14	0.2507038	0.2780133	0.2239827	0.1653049	0.9226228
BCORP1	0.0490841	0.0550454	0.0458372	0.0161329	0.0774742
TXLNGY	0.1455601	0.1275910	0.0943140	0.1259175	0.1340764
ENSG00000260197	0.0034893	0.0019386	0.0054541	0.0050196	0.0229186
KDM5D	0.1217303	0.0961052	0.0765317	0.0901725	0.1974878
ENSG00000288049	0.0086196	0.0129652	0.0173482	0.0191160	0.0172121
TTTY10	0.0377592	0.0225198	0.0269473	0.0256857	0.0845845
EIF1AY	0.6078936	0.5297449	0.4843662	0.5168377	0.4518127
SEPTIN14P23	0.0000000	0.0009742	0.0000000	0.0000000	0.0000000
MT-ND1	3.2816844	3.3397095	3.2582416	3.2345868	3.4806704
MT-ND2	3.8010456	3.8327499	3.7150251	3.7278108	3.9531624
MT-CO1	4.6639201	4.8024289	4.6720100	4.5743248	4.8732246
MT-CO2	4.4280239	4.5546143	4.4918200	4.3511885	4.6173447
MT-ATP8	1.2214494	1.2406535	1.1996729	1.1191090	1.3285443
MT-ATP6	3.6160648	3.7142814	3.6215580	3.5365297	3.8037856
MT-CO3	4.5995039	4.7455291	4.6598735	4.5039736	4.8085686
MT-ND3	3.4806246	3.5520426	3.4933209	3.4084086	3.6386306
MT-ND4L	2.9318919	3.0259717	3.0338398	2.8484820	3.1692206
MT-ND4	4.6474947	4.7378253	4.6629807	4.5834405	4.8410358
MT-ND5	3.7294078	3.8257038	3.7375502	3.6517590	3.8869721
MT-ND6	0.7768133	0.8281048	0.6968182	0.7043893	0.9529541
MT-CYB	4.3514217	4.4754856	4.3990337	4.2513294	4.5397539
MAFIP	0.0000000	0.0000000	0.0000000	0.0000000	0.0000000
ENSG00000276256	0.0760888	0.0874185	0.0757255	0.0697547	0.0821114
ENSG00000273748	0.1836004	0.2215577	0.2282405	0.1248301	0.1966835
ENSG00000271254	0.0684114	0.0795517	0.0865986	0.0874721	0.0445719
ENSG00000276345	0.0759181	0.0564322	0.0374806	0.0614085	0.0389896
ENSG00000278817	0.0286301	0.0561101	0.0654489	0.0393846	0.0166190
